# The Unique Immune System of Bats: An Evolutionary Analysis and Bibliometric Study

**DOI:** 10.1002/ece3.70614

**Published:** 2024-11-24

**Authors:** Rui Li, Wenliang Zhao, Apeng Chen, Zhiqiang Wu, Gejing De

**Affiliations:** ^1^ National Health Commission Key Laboratory of Systems Biology of Pathogens, State Key Laboratory of Respiratory Health and Multimorbidity, National Institute of Pathogen Biology Chinese Academy of Medical Sciences & Peking Union Medical College Beijing P.R. China; ^2^ Department of Respiratory Medicine, Children's Hospital of Nanjing Medical University Nanjing Naning P.R. China; ^3^ School of Population Medicine and Public Health Chinese Academy of Medical Sciences & Peking Union Medical College Beijing P.R. China; ^4^ Institute of Chinese Materia Medica China Academy of Chinese Medical Sciences Beijing P.R. China

**Keywords:** bats (Chiroptera), immune, scientometric analysis, visualization analysis

## Abstract

Bats exhibit a greater capacity to tolerate diverse viruses than other terrestrial mammals. To address these questions, we utilized evolutionary and bibliometric analyses to explore the immunological characteristics of bats and identify contemporary research hotspots in bat immunity. To investigate the historical interactions between bats and viral infections, we used tBLASTn software to identify the integrated endogenous retroviruses within the genomes of nine bat species and seven other mammals. To elucidate the immune characteristics of bats, we used the OrthoFinder, CAFE, and Gene Ontology analyses to identify the phylogenetic trees and homologous genes, expanded/contracted gene families, and associated signaling pathways of 28 mammalian genomes. We also used a bibliometric analysis of the “immune system of bats” to identify research hotspots and deepen our understanding of the immune mechanisms in bats. Significant integrations of Gammaretroviruses, Spumaretroviruses, and Deltaretroviruses were observed within bat genomes. Notable expansions in gene families included Type III interferon, heat‐shock protein 90 (HSP90), and members of the tumor necrosis factor receptor superfamily (TNFRSF). These expanded gene families are involved in signaling pathways related to “transcription and replication of influenza virus RNA,” “COVID‐19‐related pathways,” and “positive regulation of protein phosphorylation.” Notable contractions were observed in the “type I interferon” and “antibody‐related gene families.” Bibliometric analysis further underscored the several significance of critical immune genes, such as HSP90, Type I interferon, Type III interferon, and TNF. The exploration of research hotspots revealed two predominant themes: “efficient and varied antiviral responses” and “dampened inflammation to prevent excessive inflammatory reactions,” thereby elucidating the mechanisms underlying the immune adaptations of bats. Through the evolutionary and bibliometric analyses, we identified several critical immune genes and signaling pathways related to bat immunity. Currently, research on the immune system of bats primarily focuses on the themes of “efficient antiviral responses” and “inflammation suppression.”

## Graphical abstract

Through the evolutionary and bibliometric analyses, this study identified several critical immune genes and signaling pathways related to bat immunity. Currently, research on the immune system of bats primarily focuses on the themes of “efficient antiviral responses” and “inflammation suppression.”

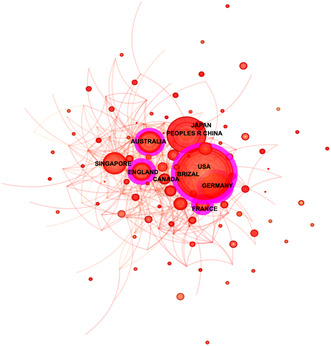

## 1 Introduction

**FIGURE 1:**
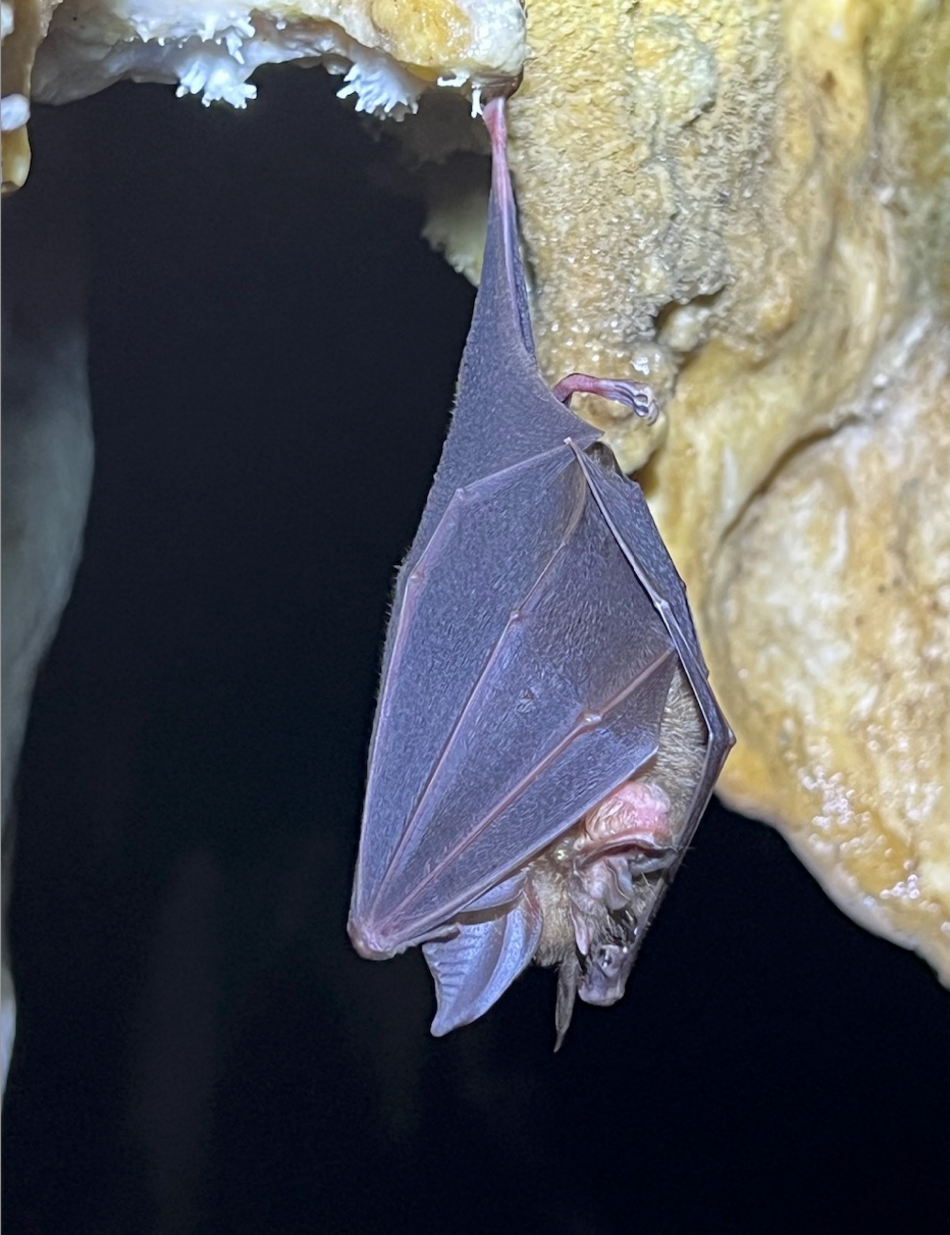
*Rhinolophus pearsonii* hanging upside down in a natural cave habitat.

Bats exhibit a remarkable capacity to harbor more zoonotic viruses without overt pathology when compared to other mammals (Shipley et al. 2019). Most bat species demonstrate carrier several viruses and remain asymptomatic, such as henipaviruses (Nipah and Hendra viruses), coronaviruses (including severe acute respiratory syndromes coronavirus [SARS‐CoV], and SARS‐CoV‐2, Middle East respiratory syndrome coronavirus [MERS‐CoV]), and filoviruses (Ebola (Judson and Munster 2023) and Marburg viruses) (Smith and Wang 2013). Growing studies show that the genetic adaptations and immune responses allow bats to serve as natural carriers for these deadly pathogens (Weinberg and Yovel 2022). However, comprehensive analyses that combine evolutionary and bibliometric approaches in the study of bat immunity remain limited. The integrated endogenous retroviruses (ERVs) within bat genomes offers valuable insights into the historical interactions between bats and viral infections. This study employs evolutionary analyses, including the identifications of ERVs and gene family expansion/contraction, alongside bibliometric analyses to pinpoint current research hotspots in bat immunity. By integrating these methodologies, the study aimed to enhance understanding of the evolutionary mechanisms underlying bat immune adaptations. This study also provides a photograph of a *Rhinolophus pearsonii* shown in Figure 1 that captured in a cave of its natural habitat.

## 2 Materials and Methods

### 2.1 Phylogenetic Analysis and Orthologous Gene Identifications

The Orthofinder software (v2.5.5) was used to infer orthologous genes across 28 mammalian genomes, including 9 bats and 19 other mammals (Table S1). This software integrates multiple methodologies, such as Markov cluster algorithm (MCL), Diamond, MAFFT, FastTree, for identifying the orthogroups and orthologs, as well as inferring rooted species trees (Emms and Kelly 2019). The inferred species tree was rooted with Atlantogenata (Nguyen et al. 2015). The final visualization of the rooted species tree was performed using the iTOL platform (https://itol.embl.de).

### 2.2 Identification of Integrated ERVs in Mammal Genomes

A comprehensive analysis of integrated ERVs was conducted across 16 mammalian genomes, including nine bat genomes and seven additional mammalian genomes. The tBLASTn software (Altschul et al. 1990) was used to identify potential ERVs in the 16 mammalian genomes by using 14 ERV probe sequences as reference (listed in Table S2). An E‐value threshold of ≤ 0.009 and a minimum query length of ≥ 400 amino acids were applied to ensure the specificity and reliability of the identified sequences (Jebb et al. 2020). These 14 ERV probes were viral polyproteins (Pols) from the *Retroviridae* family. MAFFT (v7.310) (Katoh and Standley 2013) was used to align the potentially retroviral (Pol‐like) sequences of the 16 mammalian genomes. CD‐HIT (v4.6) (Fu et al. 2012) was used to cluster the retroviral sequences, and remove redundant sequences. The first representative sequence from each cluster was extracted and were subsequently used to construct the phylogenetic trees by using FastTree (v2.1.11) (Price, Dehal, and Arkin 2009). The 14 probe sequences were also included in the phylogenetic tree construction and were utilized for the classification of ERVs. Based on the phylogenetic relationships of different ERV types, the potential ERVs were classified into the following types: Alpharetroviruses, Betaretroviruses, Gammaretroviruses, Deltaretroviruses, Epsilonretroviruses, Spumaretroviruses, and ungrouped retroviruses. The visualization of the ERVs in the 16 mammalian species was performed using R scripts and Prism 9 software.

### 2.3 Estimation of Divergence Time, Gene Family Expansions, and Contractions

This species tree was used to estimate divergence times using r8s (v1.81) (Sanderson 2003). The estimated divergence time were applied to investigate the contraction/expansion gene families by CAFE (v4.2.1) (Mendes et al. 2021). The identified expansive/contracted gene families within Chiroptera were analyzed for gene set enrichments using the Metascape platform (https://metascape.org) (Zhou et al. 2019) or R packages (clusterProfiler, DOSE, and Enrichplot), with an adjusted *p* value at < 0.05.

### 2.4 Data Collection and Searching Strategies Construction of Bibliometric Analysis

Data collection for the bibliometric analyses was carried out in two primary steps. The first step was to select an ideal bibliometrics database for analyzing the bat immune system. The published papers for this study were ascertained from the Web of Science Core Collection (WoSCC), which covered the SCI‐EXPANDED, SSCI, CPCI‐SSH, BKCI‐S, and IC databases. As a comprehensive and reproducible bibliographic database, the WoSCC provides large‐ranging access to high‐quality refereed journal literature as reliable sources of knowledge, which is widely used for bibliometrics investigations (Xu, Mishra, and Jones 2017; Aggarwal et al. 2018).

The second step was to use appropriate keywords to extract papers from the WoSCC. The representativeness and validity of the keywords should be noted, and multichecks should be performed to evaluate the correlation of each research. Thus, a comprehensive search strategy was conducted by combining the terms “Chiroptera” and “immune” in the topics from the WoSCC. The search was limited to document types classified as “articles” and restricted to publications in “English.” The “articles published in 2024” were excluded due to incomplete publication records. The search strategy can be found in Appendix S1.

### 2.5 Reference Data Analysis and Visualization

Publications were included with full records and cited references in the WoSCC, and the retrieval results were exported to Microsoft Office Excel 2019, Prism 9 V9.5.1, and plain texts for subsequent analysis. The CiteSpace 6.3.R1 software (Chen 2017) was used for bibliometrics investigations and knowledge mapping by analyzing the plain texts.

The country, institution, and reference information were used as the nodes of measurement in CiteSpace. Redundant nodes, such as full names and abbreviations, were merged when deemed appropriate. Betweenness centrality is a measure of the influence of a node within the network and can be used for assessing the strength of collaborative relationships. The bibliometric measurements in this study include the co‐occurrence counts, co‐citation counts, and the automatic cluster labeling by co‐citations. Among these, co‐citation is defined by the number of times two documents are cited by other articles published afterward, while co‐occurrence refers to the frequency at which variables appear together (Zhou et al. 2022). The cluster labeling by co‐citations is automatic clustering and grasping the features of the title, keyword, or abstract in the related references of the field (Chen 2020). Cluster labels were generated using the keywords of cited papers and likelihood ratio statistics (*p* < 0.001) within each co‐citation cluster. Modularity (Q score) is a metric for assessing the strength and quality of how a network has been divided into clusters by using community detection or data clustering algorithm. Q value ranges from 0 to 1, with values closer to 1 signifying a stronger community structure. Sigma (S score) is a metric using the formula (centrality + 1)^burstness^ (Chen 2017) to combine the betweenness centrality and citation burstness. Higher value of the S score indicates research with greater potential for influence.

Citation burstiness reflects the degree to which a particular publication experiences a sudden increase in citations over a certain period (Amrapala et al. 2023). Hotspots refer to research themes or areas that occur with high frequency. The analyses of co‐citation clusters and citation bursts could help researchers swiftly grasp the dynamics of the research field.

## 3 Results

### 3.1 Evolutionary Analysis and Identification of Integrated ERVs in Chiroptera

**FIGURE 2:**
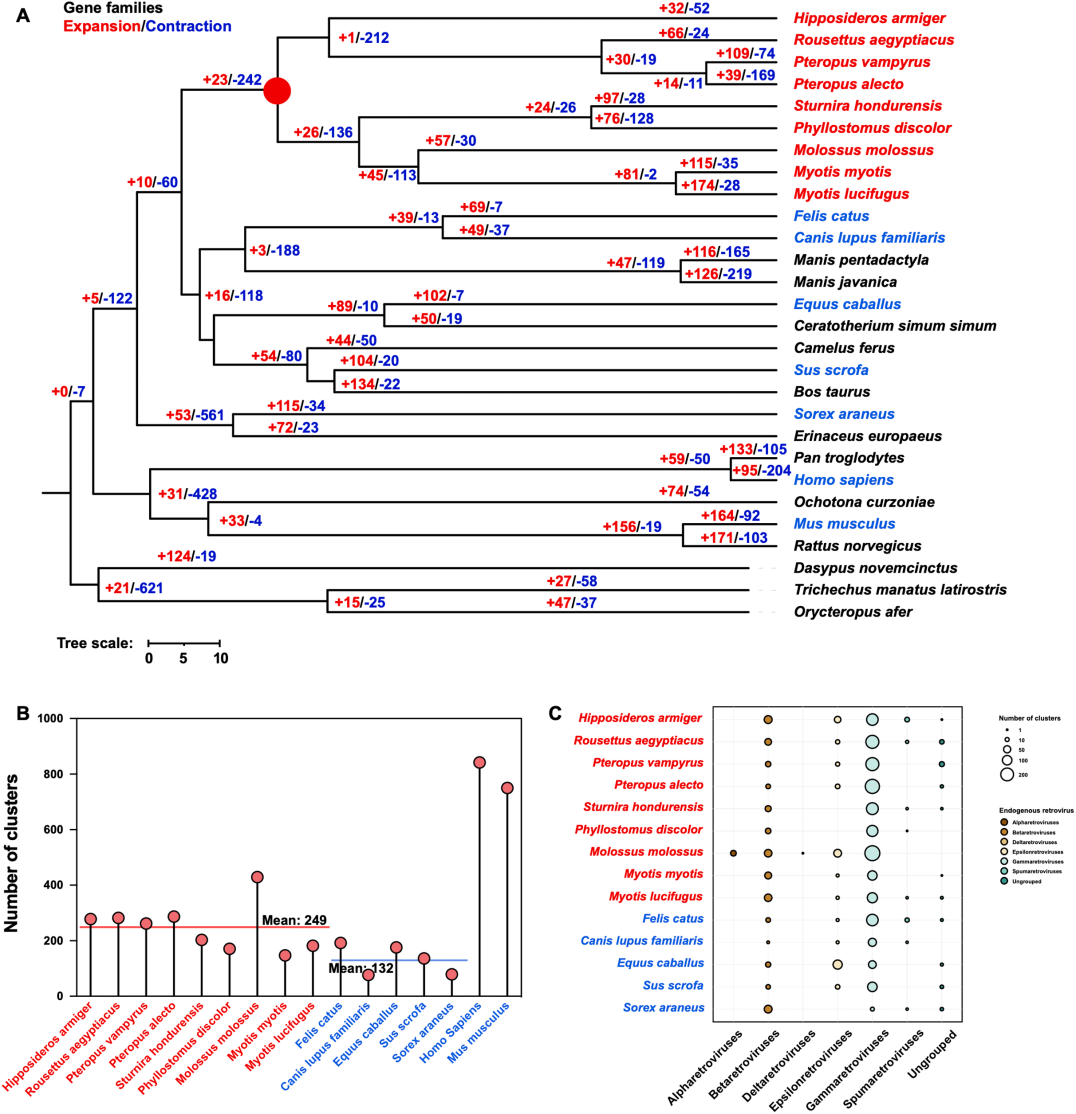
Evolutionary analysis of 28 mammal species and endogenous retrovirus (ERVs) screening. (A) displays the phylogenetic relationships of 9 bat species and 19 other mammals, and roots with Atlantogenata. All nodes achieved 100% bootstrap support across the phylogenetic tree. Bats are highlighted in red fonts, while blue fonts represent outgroup species for subsequent ERVs screening. Chiroptera are indicated by a red circle. Numbers adjacent to each node denote the count of expanded (red) and contracted (blue) gene families. (B) Results of whole‐genome ERVs screening for nine bat species and seven additional mammals. The red line represents the average number of ERVs in the nine bat species, while the blue line indicates the average number of ERVs in the five mammalian species, excluding human and mouse. (C) Detailed classification results of ERVs in nine bat species and five other mammals. Gammaretroviruses, Spumaretroviruses, and Deltaretroviruses are significantly more integrated in bat genomes compared with the other seven mammal genomes.

A comparative genomic analysis was performed using 9 bat genomes and 19 additional mammalian genomes (downloaded from the NCBI database on February 21, 2024, as shown in Table S1). A total of 10,457 orthogroups across all species were extracted and analyzed by Orthofinder. The phylogenetic species tree is presented in Figure 2A. Based on the phylogenetic analysis and divergence time estimation, the divergence between Yinpterochiroptera and Yangochiroptera occurred 69.96 million years ago (Mya) (Figure S1), which is consistent with previous estimates that range from 58 to 71 Mya (Teeling et al. 2005).

As viral infections can leave traces in the host genome in the form of endogenous viral elements, we aimed to determine whether bat genomes contain a higher number and diversity of ERVs compared with other mammals. To assess the presence and diversity of the ERVs, a comparative analysis was conducted using 16 genomes, including 9 bat species and 7 representative nonchiropteran mammalian species, as represented in the phylogenetic trees (Figure 2A; Figures S2–S17). The representative Pol‐like ERV sequences within the 16 mammal genomes were aligned with 14 ERV probes. Figure 2B and Table S3 showed the total number and classification of integrated ERVs identified in the nine bat species, and the seven other mammalian species. The results revealed that the Gammaretroviruses, Spumaretroviruses, and Deltaretroviruses were markedly integrated in bats compared with the other seven mammalian species (Figure 2C). Notably, except for humans and mice, bats exhibit a significantly higher number of integrated EVRs relative to the other five mammalian species. The integrations of Alpharetroviruses, Betaretroviruses, Gammaretroviruses, Spumaretroviruses, and Deltaretroviruses were significantly higher in bats than in the other five mammalian species. Overall, these results demonstrate that bat genomes harbor a diversity of ERVs, providing compelling evidence for past viral infections in the evolution history.

### 3.2 Gene Sets Analyses of Expansive and Contracted Gene Families in Bats

**FIGURE 3:**
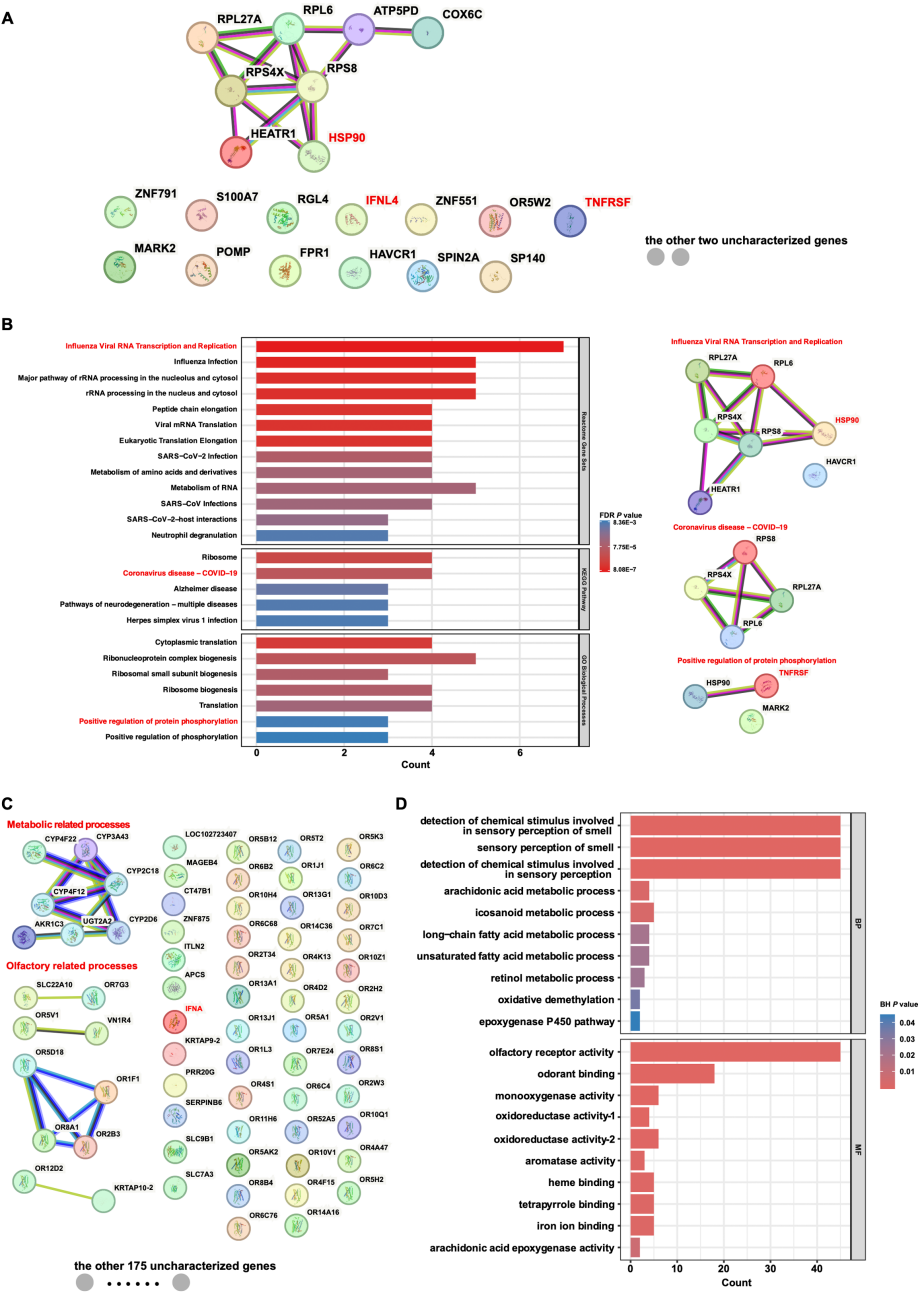
PPI and gene set analyses of expansion and contraction gene families. (A) Protein–protein interaction (PPI) network for 23 expansive gene families. (B) Gene set analysis results for the 23 expansive gene families, primarily involve in signaling pathways related to “influenza viral RNA transcription and replication,” “coronavirus disease,” and “positive regulation of protein phosphorylation.” The corresponding PPI networks for these three signaling pathways are shown in the right section of (B). (C) PPI networks for 242 contracted gene families are mainly associated with metabolic or olfactory pathways. (D) GO analysis results for the 242 contracted gene families mainly focus on metabolic or olfactory pathways.

To understand the mechanisms underlying immune evolution in bats, we conducted an extensive analysis of gene family expansions and contractions within Chiroptera. The gene families of expansion and contraction in Chiroptera are detailed in Figure 3A and Tables S4 and S5, revealing significant expansions in gene families of Type III interferon (such as IFNL4), HSP90, and the tumor necrosis factor receptor superfamily (TNFRSF). These expansions, characterized by increased gene copy numbers, likely enhance the functional capabilities of these gene families. The expansive gene families in Chiroptera are involved in signaling pathways related to “transcription and replication of influenza virus RNA,” “COVID‐19‐related pathways,” and “positive regulation of protein phosphorylation” (Figure 3B; Table S6). The key genes in these pathways include RPL6, RPL27A, RPS8, RPS4X, HEATR1, HAVCR1, HSP90, TNFRSF, and MARK2 (Figure 3B; Table S6). The “type I interferon” and antibody‐related gene families (IGKV1, IGHV3, and IGHV1) were significantly contracted among bats (Table S7). The contractions in gene families associated with metabolic functions and olfactory processes were also observed, as shown in Figure 3C, and detailed in Tables S7 and S8.

### 3.3 Characteristics of Extracted Papers on Bat Immune System Based on the Bibliometric Exploration

**FIGURE 4:**
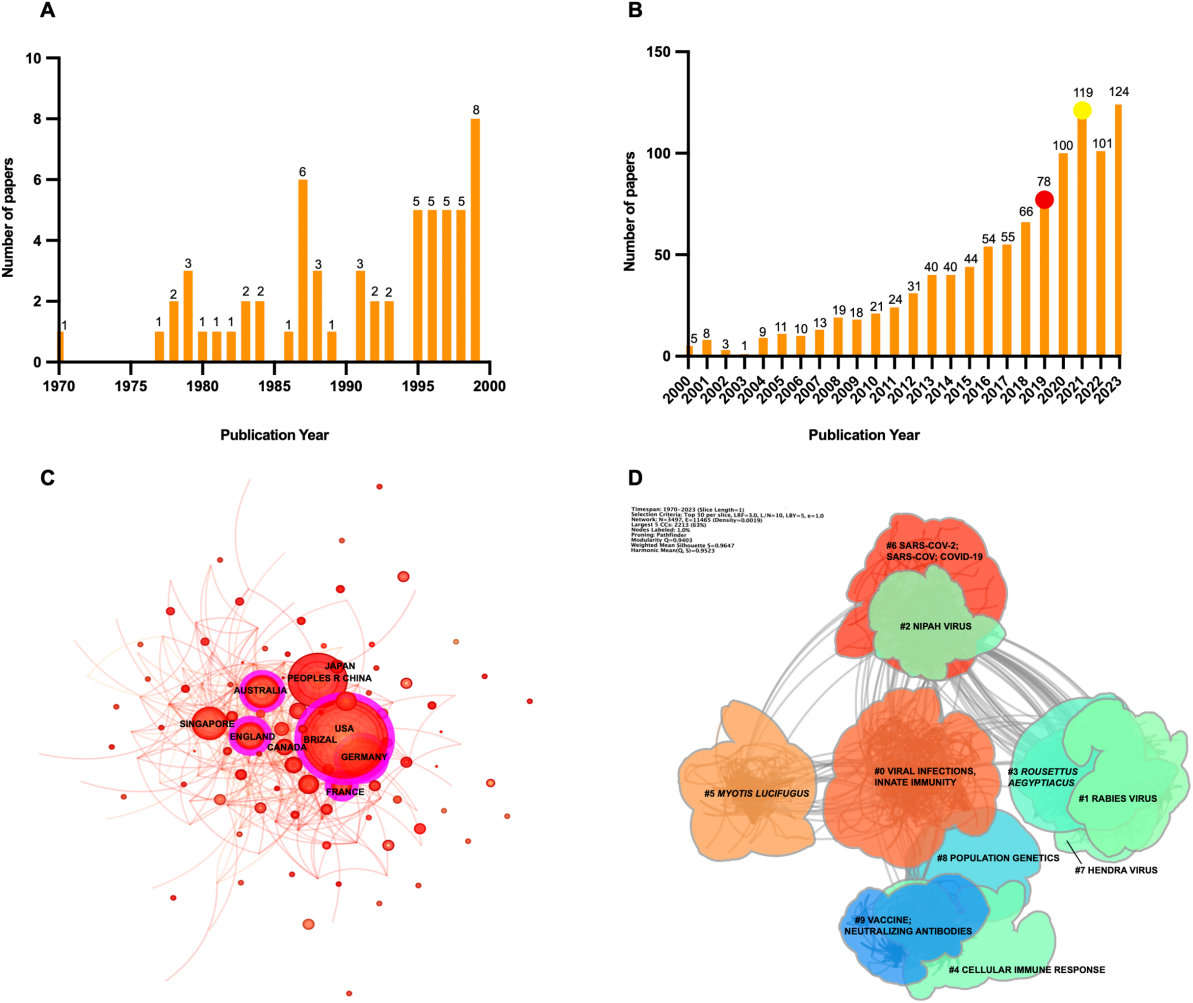
Bibliometric analysis results. (A) Trends in the annual article numbers in the field of bat immunity from 1970 to 1999. From 1970 to 1999, the annual publication volume remained consistently low, with the first paper published in 1970 and the highest number of published articles during this period not exceeding 10 per year. (B) Trends in the annual article numbers in the field of bat immunity from 2000 to 2023. The publication count exhibited a significant increase in the annual publication slope was observed in 2019, marked by a red circle. Following 2019, there was an accelerated upward trend from 2019 to 2021. However, this trend began to decline from 2021 to 2022, with the turning point in 2021 indicated by a yellow circle. (C) Collaborative network map of countries/regions. Links between “nodes” represent the frequency of coauthorship among countries/regions in publications. The size of each node reflects the volume of publications originating from that country or region. A purple annular ring surrounding a node indicates its betweenness centrality, calculated based on total link strength. A thicker purple ring denotes higher betweenness centrality and greater link strength, underscoring the key role of that node in the collaborative network. (D) Cluster view of reference co‐citation analysis. Co‐cited references were categorized into 10 clusters, with the largest cluster centered on “viral infections and innate immunity.”

This bibliometric search strategy yielded 1054 articles (Table S9), with the first paper published in 1970. These articles could be classified into 104 different categories. The three categories with the highest number of literatures were immunology (205 documents, 19.45%), virology (155 documents, 14.71%), and multidisciplinary sciences (131 documents, 12.43%). The annual published papers on bat immunity research, as depicted in Figure 4A,B and Table S10, showed a general upward trend over time. However, a slight deceleration in this growth trend was observed post‐2021. Specifically, during the initial outbreak of COVID‐19 in 2019, the growth trend in annual publications on bat immunity accelerated significantly. Between 2019 and 2021, the volume of literature showed a sharp upward surge. As the COVID‐19 situation gradually eased after 2021, the publication volume began to exhibit a declining trend.

For the bibliometric section, we primarily studied country and institution collaboration networks, as well as hotspot analyses. The research hotspots in this field may further reveal the immune adaptations of bats.

### 3.4 Country and Institution Collaborative Network

From 1970 to 2023, researchers from 90 countries and regions, representing 1753 institutions, published documents on bat immunity (WoSCC). The international collaboration network comprised 90 nodes and 378 links, with the top 10 most productive countries displayed in Figure 4C and Table S11. The USA emerged as the largest contributor with 428 papers published, followed by China (180 publications). Centrality analysis revealed stronger cooperation between Western countries and others, with the USA (centrality: 0.31) and France (0.31) showing particularly robust collaborative relationships. The top 10 most productive institutions accounted for approximately 39.75% of total articles (Table S12). The Centers for Disease Control & Prevention cooperate the closest with other institutes, with a centrality value of 0.12.

### 3.5 Research Hotspots Analysis by the Bibliometric Exploration

#### 3.5.1 Literature Co‐Citation Network

The publication co‐citation analyses were conducted using a 1‐year time slice and a pathfinder network scaling algorithm by CiteSpace. Figure 4D shows the co‐citation network, which yielded 3497 nodes and 11,465 links, summarized into 10 main clusters. The network exhibits high structural integrity and cluster homogeneity, as evidenced by its high Q score of 0.940 and mean S score of 0.965. The color spectrum of the links corresponds to different time slices, with cooler hues indicating earlier years and warmer tones representing more recent publications.

#### 3.5.2 Citation Data With the Top 10 Latest Co‐Citation Clusters

The top 10 latest clusters of keywords (#0 viral infections, #1 rabies virus, #2 nipah virus, #3 *Rousettus aegyptiacus* , #4 cellular immune response, #5 *Myotis lucifugus* , #6 SARS‐CoV‐2, #7 Hendra virus, #8 population genetics, and #9 vaccine) were presented in Table S13. In brief, these clusters are mainly divided into three parts: the immune responses to viral infections (Ahn et al. 2019, 2023; Jebb et al. 2020; Moore et al. 2011; Pavlovich et al. 2018; Tarigan et al. 2020, 2021; Tian et al. 2023; Virtue et al. 2011; Xie et al. 2018; Zhou et al. 2011, 2016); the exploration of the origins of SARS‐CoV (Lau et al. 2005; Poon et al. 2005), SARS‐CoV‐2 (Rastogi et al. 2020; Zhu et al. 2020) and vaccine development; the prevalence and diagnosis of Hendra virus, rabies virus, or Nipah virus (Epstein et al. 2013; Glennon et al. 2015; Hemachudha and Phuapradit 1997; Warrell and Warrell 2004).

The articles in Cluster 0 had the largest size of literature citations and were mainly focused on the topic of “viral infection and immune.” This revealed that bats might have undergone contractions or expansions, mutations under positive selection stress, or nonconserved sequence differences in critical domains of immune genes related to antiviral signaling pathways during their long‐term evolution of resisting viral infections, leading them as generally asymptomatic carriers with detrimental zoonotic viruses to humans (Xie et al. 2018; Pavlovich et al. 2018; Ahn et al. 2019; Mandl et al. 2018). In this cluster, the most research interests extensively studied are about the molecular characterization of genes related to innate immunity, such as the genes of different pathogen‐associated molecular patterns [NLR gene family (Tian et al. 2023), retinoic acid‐inducible gene‐I (RIG‐I) helicases (Tarigan et al. 2020, 2021), and toll‐like receptors (TLRs) (Tarigan et al. 2020, 2021)], and the downstream production of antiviral adaptor or effector molecules [like STING (Xie et al. 2018), Type I or III interferons (IFN) (Zhou et al. 2011, 2016), IFN‐stimulated genes (ISGs) (Virtue et al. 2011), apoptosis‐associated speck‐like protein containing a CARD (Ahn et al. 2023), MyD88 (Tian et al. 2023), APOBEC3 (Jebb et al. 2020)], or the complement protein activity (Moore et al. 2011). Bats may control the pathogen replication and mitigate the immunopathology through changes in distinct pathways. However, they have a final goal of decreasing inflammatory responses and hence, increasing the tolerance of viral diseases. Therefore, research on these critical genes in bats will have the potential to decipher the unique functions of bat immune systems, providing important clues for humans to resist viral infections.

The articles in Cluster 4 mainly focus on adaptive immunity (cellular immune response) in bats (Banerjee et al. 2020a), which explored the antibody‐mediated immune responses (e.g., the IgM, IgE, IgA, and multiple IgGs were detected in several bat species and nearly analogous to corresponding human immunoglobulins (Butler et al. 2011)) and immune cell populations in bats (e.g., the ratio of major immune cells of Indian flying fox [ *Pteropus giganteus*] is similar to that of mice (Sarkar and Chakravarty 1991)) (Banerjee et al. 2020a), and the polymorphism and sequence diversity of major histocompatibility complex (MHC) molecules in bats (Wang et al. 2024). Studies indicated that MHC molecules play a crucial role in the antiviral process, and investigating the MHC polymorphism may in turn, reflect the ability of different populations of bats to respond to infections (Wang et al. 2024). For example, Wang et al. demonstrated that both 3AA and 5AA insertions of MHC Class I (MHC‐I) in bats help strengthen the thermal stability of the MHC‐I complex by enhancing the 3_10_‐helix region, thus improving the binding ability and diversity of peptides (Wang et al. 2024).

#### 3.5.3 Citation Bursts on Bat Immune System

As shown in Table S14, 101 references were marked as citation bursts. Among them, 30 references (Ahn et al. 2019; Andersen et al. 2020; Banerjee et al. 2017, 2020a; Boni et al. 2020; Coronaviridae Study Group of the International Committee on Taxonomy of Viruses 2020; Cui, Li, and Shi 2019; De La Cruz‐Rivera et al. 2018; Goh et al. 2020; Goldstein et al. 2018; Gorbunova, Seluanov, and Kennedy 2020; Hölzer et al. 2019; Irving et al. 2021; Jebb et al. 2020; Kacprzyk et al. 2017; Lam et al. 2020; Letko et al. 2020; Lu et al. 2020; Mandl et al. 2018; Mollentze and Streicker 2020; Olival et al. 2017; Pavlovich et al. 2018; Subudhi, Rapin, and Misra 2019; Wacharapluesadee et al. 2021; Wang and Anderson 2019; Wu et al. 2020; Xie et al. 2018; Zhou et al. 2020a, 2020b; Zhu et al. 2020) became the strongest burst citations lasting until 2023 (Table S15), reflecting the latest trend in this field. There were 16 articles concerned the origin of virus and receptor recognitions, particularly focusing on the proximal origin and structural basis of coronaviruses or filoviruses (Andersen et al. 2020; Boni et al. 2020; Coronaviridae Study Group of the International Committee on Taxonomy of Viruses 2020; Cui, Li, and Shi 2019; Goldstein et al. 2018; Lam et al. 2020; Letko et al. 2020; Lu et al. 2020; Mollentze and Streicker 2020; Olival et al. 2017; Wacharapluesadee et al. 2021; Wang and Anderson 2019; Wu et al. 2020; Zhou et al. 2020a, 2020b; Zhu et al. 2020). While 14 papers investigated the extraordinary viral tolerance mechanism mediated by the unique antiviral and anti‐inflammatory responses in bats (Ahn et al. 2019; Banerjee et al. 2017, 2020a; De La Cruz‐Rivera et al. 2018; Goh et al. 2020; Gorbunova, Seluanov, and Kennedy 2020; Hölzer et al. 2019; Irving et al. 2021; Jebb et al. 2020; Kacprzyk et al. 2017; Mandl et al. 2018; Pavlovich et al. 2018; Subudhi, Rapin, and Misra 2019; Xie et al. 2018) (Table 1), the 14 papers (Ahn et al. 2019; Banerjee et al. 2017, 2020a; De La Cruz‐Rivera et al. 2018; Goh et al. 2020; Gorbunova, Seluanov, and Kennedy 2020; Hölzer et al. 2019; Irving et al. 2021; Jebb et al. 2020; Kacprzyk et al. 2017; Mandl et al. 2018; Pavlovich et al. 2018; Subudhi, Rapin, and Misra 2019; Xie et al. 2018) concerned about “bat immune” were eventually included and analyzed for the exploration of study hotspots in bat immune processes.

**TABLE 1:** Fourteen references with the strongest citation bursts lasting until 2023.

Reference titles	Year	Strength	Begin	End	1970–2023
Dampened STING‐Dependent Interferon Activation in Bats	2018	20.93	2019	2023	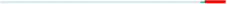
Virus‐ and Interferon Alpha‐Induced Transcriptomes of Cells from the Microbat Myotis daubentonii	2019	4.16	2020	2023	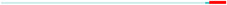
Immune System Modulation and Viral Persistence in Bats: Understanding Viral Spillover	2019	6.54	2021	2023	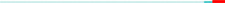
The Egyptian Rousette Genome Reveals Unexpected Features of Bat Antiviral Immunity	2018	24.22	2019	2023	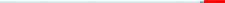
Going to Bat(s) for Studies of Disease Tolerance	2018	10.18	2019	2023	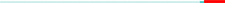
A Potent Anti‐Inflammatory Response in Bat Macrophages May Be Linked to Extended Longevity and Viral Tolerance	2017	6.8	2019	2023	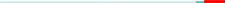
Six reference‐quality genomes reveal evolution of bat adaptations	2020	15.3	2021	2023	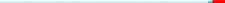
Lessons from the host defenses of bats, a unique viral reservoir	2021	17.5	2021	2023	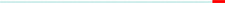
The World Goes Bats: Living Longer and Tolerating Viruses	2020	7.08	2021	2023	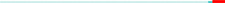
Complementary regulation of caspase‐1 and IL‐1β reveals additional mechanisms of dampened inflammation in bats	2020	5.44	2021	2023	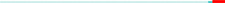
The IFN Response in Bats Displays Distinctive IFN‐Stimulated Gene Expression Kinetics with Atypical RNASEL Induction	2018	7.33	2021	2023	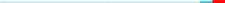
Novel Insights Into Immune Systems of Bats	2020	17.4	2020	2023	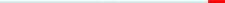
Lack of inflammatory gene expression in bats: a unique role for a transcription repressor	2017	7.51	2020	2023	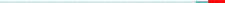
Dampened NLRP3‐mediated inflammation in bats and implications for a special viral reservoir host	2019	20.41	2020	2023	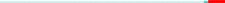

### 3.6 Research Hotspots

#### 3.6.1 The Efficient and Varied Antiviral Response in Bats

Compared with humans, bats appear to have the constitutive IFN activation and stronger combinatorial diversity of immunoglobulin genes without experiencing substantial affinity maturation (Schountz et al. 2017). Due to the constitutive IFN activity and widely naive antibody repertoires, bats could limit virus replications at a lower level in the early phase of infection (Schountz et al. 2017). The previous comparative genomics studies demonstrated that most positively selected genes of bats seemed to be concentrated in the innate immune response and DNA damage checkpoint pathways (Zhang et al. 2013). Pavlovich et al. demonstrated that multiple innate immune response‐associated genes experienced positive selection pressures in the Egyptian rousette bat ( *Rousettus aegyptiacus* . *R. aegyptiacus* ) branch, which included IFNAR1 (a subunit of the Type I IFN receptor), ISG15 (an interferon‐stimulated gene), and SIKE1 (a negative regulator of the interferon response) (Pavlovich et al. 2018). Meanwhile, JAK2 and STAT3, DDX58 (RIG‐I), and TLR8 have experienced purifying selection in *R. aegyptiacus* (Pavlovich et al. 2018). The sensing of cytoplasmic DNA is dampened and led less effective in activating interferons through a regulatory site mutation in bat STING (S358) (Subudhi, Rapin, and Misra 2019). The family members of TLRs (TLR9 (Banerjee et al. 2020a), TLR7, and TLR8) are also found distinctly positive selection, which may contribute to the adaptation evolution of pathogen–host interactions in bats (Jiang et al. 2017). Furthermore, the antiviral effects were also enhanced by the site mutation of IRF3 (S185) (Gorbunova, Seluanov, and Kennedy 2020; Banerjee et al. 2020b), or the expansions of APOBEC3 gene families (Jebb et al. 2020) or MHC‐I genes (Pavlovich et al. 2018) in bats when compared to other mammals (Xie et al. 2018; Irving et al. 2021). These results were consistent with similar analyses in black flying foxes ( *Pteropus alecto* ) and David's myotis ( *Myotis davidii* ) (Zhang et al. 2013).

Apart from the above positive selection or the gene family's contraction or expansion events, bat ISGs exhibited different expression patterns when compared with those of human cells (Hölzer et al. 2019). In the IFN‐stimulated cells, bat ISGs comprise two special temporal subclusters, as well as early induction kinetics and significant late‐phase declines, while human ISGs lack late‐phase declines and always maintain elevated for longer periods (De La Cruz‐Rivera et al. 2018). Furthermore, in unstimulated cells, the expression levels of bat ISGs were also higher than their counterparts in human cells (De La Cruz‐Rivera et al. 2018). Thus, bats could exhibit an outstanding balance between the enhanced immune tolerance and host defense responses against viruses by several mechanisms, for instance, the constitutive expression of IFNs and ISGs, the increased expression levels of heat‐shock proteins (HSPs), and enhanced cell autophagy (Irving et al. 2021). These associated immune genes found obviously positive selection, significant contraction, expansions, or unique expression patterns in the results of comparative genomic and transcriptomic analyses, which would help characterize the immune features of bats (Zhang et al. 2013).

#### 3.6.2 Dampened Inflammatory to Avoid an Excessive Inflammatory Reaction

Besides the efficient and diverse antiviral processes in bats, the dampened inflammatory may also play a critical role in avoiding excessive inflammatory reactions and mediating immune tolerance in bats (Mandl et al. 2018; Irving et al. 2021). Banerjee et al. (2017) demonstrated that a potential repressor (c‐Rel) binding motif of the TNFα promoter in the big brown bat ( *Eptesicus fuscus* ) might significantly suppress basal expression levels of TNFα transcripts. The deletion of this motif in the TNFα promoter or partial knockdown of bat c‐Rel transcripts obviously increased the basal expression of TNFα transcripts in bat cells (Banerjee et al. 2017). Meanwhile, studies found that the proinflammatory response in bat macrophages was balanced by a continuous high‐level transcription of interleukin‐10 (IL‐10, an anti‐inflammatory cytokine), which was not observed in the mouse macrophages (Kacprzyk et al. 2017).

NLR family pyrin domain containing 3 (NLRP3) is also an important sensor for recognizing cellular stresses, as well as bacterial or viral infections (Kuriakose and Kanneganti 2017). Ahn et al. (2019) identified that bat NLRP3 had suppressed transcriptional priming independent of TLR signaling, alternative splicing of exon 7, and/or an altered leucine‐rich repeat domain, which eventually resulting the dampened activity of bat NLRP3 isoforms, further dampening the NLRP3‐mediated inflammation responses. Besides the dampened NLRP3 function, all PYHIN genes (including IFI16 AIM2) were found to be completely lost in bats, including both insect‐ and fruit‐eating bats (Subudhi, Rapin, and Misra 2019). Complementary regulation also reveals additional mechanisms for dampening the inflammation of bats, and the key residues (R371Q, D365N) in the bat caspase‐1 weaken the activity of the enzyme and significantly reduce both IL‐1β and GSDMD substrate processing and maturation in bats (Goh et al. 2020). Meanwhile, Jebb et al. (2020) found several positive selection (INAVA and IL‐1β) and loss of immunity‐related genes (IL36G and LRRC70) were involved in the pro‐inflammatory NF‐κB signaling pathways, which suggested that the altered NF‐κB signaling might take participated in the immune‐related adaptations of bats. To sum up, the dampened inflammation by multiple potential mechanisms might have key implications for cellular stress and viral infection in bats (Jebb et al. 2020; Irving et al. 2021).

## 4 Discussion and Conclusion

This study integrates evolutionary analyses and bibliometric explorations to elucidate the immune evolution mechanisms of bats.

Through comparative genomic evolutionary analysis, several expanded gene families have been identified, including Type III interferon, HSP90, and TNFRSF. These expanded immune‐related genes may play crucial roles in the regulation of the bat immune system. Despite a noted contraction in the “type I interferon” gene family (specifically IFNA) among bats, the constitutive expression of “type I interferons” has also been observed in other research studies (Zhou et al. 2016). The inherently high baseline levels of IFN can effectively suppress viral replication during the early stages of infection (Zhou et al. 2016). This mechanism potentially limits the activation of cellular immunity and prevents potentially harmful excessive immune responses (Schountz et al. 2017). The production of antibodies remains comparatively lower in bats during viral infections (Schountz et al. 2017), which supports the observed contraction in “antibody‐related gene families,” such as IGKV1, IGHV3, and IGHV1. Consistent with these findings, we also observed significant diversity of ERVs integrated in bat genomes.

The bibliometric analysis also reveals a significant increase in publications on bat immunity from 1970 to 2023, particularly following the COVID‐19 pandemic. Researchers have primarily concentrated on several key immune‐related genes that influence bat immunity, including interferons (both Type I and Type III), TLRs, TNFs, HSPs, ISGs, NLRs, “antibody‐related genes involved in cellular immunity” (IgM, IgE, IgA, and various IgG subclasses), and MHC molecules.

Integrating evolutionary analyses with bibliometric approaches enables us to summarize the principal immune characteristics of bats, which can be encapsulated in two themes: “efficient and diverse antiviral responses” and “inflammatory suppression.” These findings provide a substantial theoretical foundation for a deeper understanding of the immune adaptations in bats.

## Author Contributions

**Rui Li:** data curation (equal), formal analysis (lead), investigation (lead), methodology (lead), software (lead), validation (lead), visualization (lead), writing – original draft (lead). **Wenliang Zhao:** visualization (supporting), writing – review and editing (supporting). **Apeng Chen:** data curation (lead), writing – review and editing (equal). **Zhiqiang Wu:** conceptualization (lead), funding acquisition (lead), resources (lead), writing – review and editing (lead). **Gejing De:** conceptualization (equal), writing – review and editing (lead).

## Ethics Statement

This study does not include any research involving humans or animals.

## Consent

The authors have nothing to report.

## Conflicts of Interest

The authors declare no conflicts of interest.

## Supporting information


Data S1.

## Data Availability

The dataset supporting the conclusions of this article is included within the Supporting Information.
