# Supplementary material for: The Unique Immune System of Bats: An Evolutionary Analysis and Bibliometric Study
Source: Ecol Evol. 2024 Nov 24;14(11):e70614. doi: 10.1002/ece3.70614 (PMC11586106; doi:10.1002/ece3.70614)
Supplement: Supplementary file 1 — Data S1. [file ECE3-14-e70614-s001.zip › ece370614-sup-0001-DataS1 /Supplementary_Appendix_1.docx]

**Supplementary Appendix 1**.

**Search strategy (October 21^st^, 2024):**

Select a database: Web of Science Core Collection

Document types: Article

Language: English

Timespan: Excluding the articles published in 2024 due to incomplete publication records.

Citation Indexes: The Science Citation Index Expanded (SCI-EXPANDED), the Social Sciences Citation Index (SSCI), the Conference Proceedings Citation Index – Science (CPCI-S), the Book Citation Index-Science (BKCI-S), the Index Chemicus (IC).

Results: 1,054

Boolean operation rules:

(((((((((((((((((((((((((TS=(bats)) OR TS=(Chiroptera)) OR TS=(Rhinolophus)) OR TS=(Rhinolophidae)) OR TS=(Pteropodidae)) OR TS=(Rhinolophidea)) OR TS=(Yangochiroptera)) OR TS=(Vespertilionidae)) OR TS=(Yingterochiroptera)) OR TS=(Phyllostomidae)) OR TS=(Hipposideridae)) OR TS=(Molossidae))) OR TS=(Craseonycteridae)) OR TS=(Emballonuridae)) OR TS=(Furipteridae)) OR TS=(Megadermatidae)) OR TS=(Mormoopidae)) OR TS=(Mystacinidae)) OR TS=(Myzopodidae)) OR TS=(Natalidae)) OR TS=(Noctilionidae)) OR TS=(Nycteridae)) OR TS=(Rhinopomatidae)) OR TS=(Thyropteridae)) AND ((TS=(immune) OR TS=(immunology)) OR TS=(immunity))
