# Supplementary material for: The Unique Immune System of Bats: An Evolutionary Analysis and Bibliometric Study
Source: Ecol Evol. 2024 Nov 24;14(11):e70614. doi: 10.1002/ece3.70614 (PMC11586106; doi:10.1002/ece3.70614)
Supplement: Supplementary file 1 — Data S1. [file ECE3-14-e70614-s001.zip › ece370614-sup-0001-DataS1 /Figure S12. The phylogenetic tree of the integrated ERVs in the Canis lupus familiaris genome.pdf]

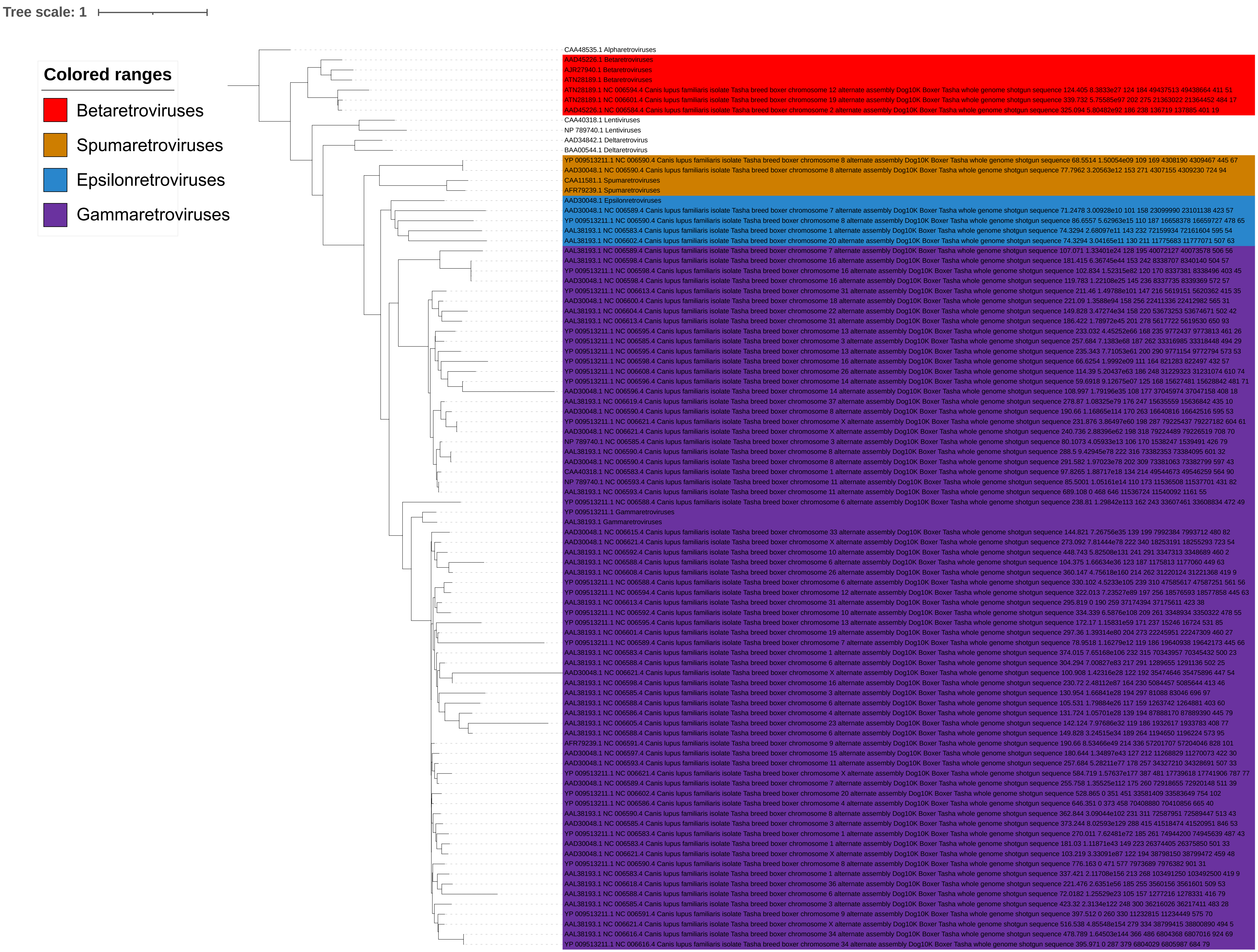
Tree scale: 1
Colored ranges
Betaretroviruses
Spumaretroviruses
Epsilonretroviruses
Gammaretroviruses
CAA48535.1 Alpharetroviruses
AAD45226.1 Betaretroviruses
AJR27940.1 Betaretroviruses
ATN23189.1 Betaretroviruses
ATN23189.1 NC 006594.4 Canis lupus familiaris isolate Tasha breed boxer chromosome 12 alternate assembly Dog10K Boxer Tasha whole genome shotgun sequence 124.405 8.3333e27 124 184 49437513 49438664 411 51
ATN23189.1 NC 006601.4 Canis lupus familiaris isolate Tasha breed boxer chromosome 19 alternate assembly Dog10K Boxer Tasha whole genome shotgun sequence 339.732 5.75585e97 202 275 21363022 21364452 484 17
AAD45226.1 NC 006584.4 Canis lupus familiaris isolate Tasha breed boxer chromosome 2 alternate assembly Dog10K Boxer Tasha whole genome shotgun sequence 325.094 5.80482e92 186 238 136719 137885 401 19
CAA40318.1 Lentiviruses
NP 789740.1 Lentiviruses
AAD34842.1 Deltaretrovirus
BAA00544.1 Deltaretrovirus
YP 009513211.1 NC 006590.4 Canis lupus familiaris isolate Tasha breed boxer chromosome 8 alternate assembly Dog10K Boxer Tasha whole genome shotgun sequence 68.5514 1.50054e09 109 169 4308190 4309467 445 67
AAD30048.1 NC 006590.4 Canis lupus familiaris isolate Tasha breed boxer chromosome 8 alternate assembly Dog10K Boxer Tasha whole genome shotgun sequence 77.7962 3.20563e12 153 271 4307155 4309230 724 94
CAA11581.1 Spumaretroviruses
AFR79239.1 Spumaretroviruses
AAD30048.1 Epsilonretroviruses
AAD30048.1 NC 006589.4 Canis lupus familiaris isolate Tasha breed boxer chromosome 7 alternate assembly Dog10K Boxer Tasha whole genome shotgun sequence 71.2478 3.00928e10 101 158 23099990 23101138 423 57
YP 009513211.1 NC 006590.4 Canis lupus familiaris isolate Tasha breed boxer chromosome 8 alternate assembly Dog10K Boxer Tasha whole genome shotgun sequence 86.6557 5.62963e15 110 187 16658378 16659727 478 65
AAL38193.1 NC 006583.4 Canis lupus familiaris isolate Tasha breed boxer chromosome 1 alternate assembly Dog10K Boxer Tasha whole genome shotgun sequence 74.3294 2.68097e11 143 232 72159934 72161604 595 54
AAL38193.1 NC 006602.4 Canis lupus familiaris isolate Tasha breed boxer chromosome 20 alternate assembly Dog10K Boxer Tasha whole genome shotgun sequence 74.3294 3.04165e11 130 211 11775683 11777071 507 63
AAL38193.1 NC 006589.4 Canis lupus familiaris isolate Tasha breed boxer chromosome 7 alternate assembly Dog10K Boxer Tasha whole genome shotgun sequence 107.071 1.33401e24 128 195 40072127 40073578 506 56
AAL38193.1 NC 006598.4 Canis lupus familiaris isolate Tasha breed boxer chromosome 16 alternate assembly Dog10K Boxer Tasha whole genome shotgun sequence 181.415 6.36745e44 153 242 8338707 8340140 504 57
YP 009513211.1 NC 006598.4 Canis lupus familiaris isolate Tasha breed boxer chromosome 16 alternate assembly Dog10K Boxer Tasha whole genome shotgun sequence 102.834 1.52315e82 120 170 8337381 8338496 403 45
AAD30048.1 NC 006598.4 Canis lupus familiaris isolate Tasha breed boxer chromosome 16 alternate assembly Dog10K Boxer Tasha whole genome shotgun sequence 119.783 1.22108e25 145 236 8337735 8339369 572 57
YP 009513211.1 NC 006613.4 Canis lupus familiaris isolate Tasha breed boxer chromosome 31 alternate assembly Dog10K Boxer Tasha whole genome shotgun sequence 211.46 1.49788e101 147 216 5619151 5620362 415 35
AAD30048.1 NC 006600.4 Canis lupus familiaris isolate Tasha breed boxer chromosome 18 alternate assembly Dog10K Boxer Tasha whole genome shotgun sequence 221.09 1.3588e94 158 256 22411336 22412982 565 31
AAL38193.1 NC 006604.4 Canis lupus familiaris isolate Tasha breed boxer chromosome 22 alternate assembly Dog10K Boxer Tasha whole genome shotgun sequence 149.828 3.47274e34 158 220 53673253 53674671 502 42
AAL38193.1 NC 006613.4 Canis lupus familiaris isolate Tasha breed boxer chromosome 31 alternate assembly Dog10K Boxer Tasha whole genome shotgun sequence 186.422 1.78972e45 201 278 5617722 5619530 650 93
YP 009513211.1 NC 006595.4 Canis lupus familiaris isolate Tasha breed boxer chromosome 13 alternate assembly Dog10K Boxer Tasha whole genome shotgun sequence 233.032 4.45252e66 168 235 9772437 9773813 461 26
YP 009513211.1 NC 006585.4 Canis lupus familiaris isolate Tasha breed boxer chromosome 3 alternate assembly Dog10K Boxer Tasha whole genome shotgun sequence 257.684 7.1383e68 187 262 33316985 33318448 494 29
YP 009513211.1 NC 006595.4 Canis lupus familiaris isolate Tasha breed boxer chromosome 13 alternate assembly Dog10K Boxer Tasha whole genome shotgun sequence 235.343 7.71053e61 200 290 9771154 9772794 573 53
YP 009513211.1 NC 006598.4 Canis lupus familiaris isolate Tasha breed boxer chromosome 16 alternate assembly Dog10K Boxer Tasha whole genome shotgun sequence 66.6254 1.9992e09 111 164 821283 822497 432 57
YP 009513211.1 NC 006608.4 Canis lupus familiaris isolate Tasha breed boxer chromosome 26 alternate assembly Dog10K Boxer Tasha whole genome shotgun sequence 114.39 5.20437e63 186 248 31229323 31231074 610 74
YP 009513211.1 NC 006596.4 Canis lupus familiaris isolate Tasha breed boxer chromosome 14 alternate assembly Dog10K Boxer Tasha whole genome shotgun sequence 59.6918 9.12675e07 125 168 15627481 15628842 481 71
AAD30048.1 NC 006596.4 Canis lupus familiaris isolate Tasha breed boxer chromosome 14 alternate assembly Dog10K Boxer Tasha whole genome shotgun sequence 108.997 1.79196e35 108 177 37045974 37047158 408 18
AAL38193.1 NC 006619.4 Canis lupus familiaris isolate Tasha breed boxer chromosome 37 alternate assembly Dog10K Boxer Tasha whole genome shotgun sequence 278.87 1.08325e79 176 247 15635559 15636842 435 10
AAD30048.1 NC 006590.4 Canis lupus familiaris isolate Tasha breed boxer chromosome 8 alternate assembly Dog10K Boxer Tasha whole genome shotgun sequence 190.66 1.16865e114 170 263 16640816 16642516 595 53
YP 009513211.1 NC 006621.4 Canis lupus familiaris isolate Tasha breed boxer chromosome X alternate assembly Dog10K Boxer Tasha whole genome shotgun sequence 231.876 3.86497e60 198 287 79225437 79227182 604 61
AAD30048.1 NC 006621.4 Canis lupus familiaris isolate Tasha breed boxer chromosome X alternate assembly Dog10K Boxer Tasha whole genome shotgun sequence 240.736 2.88396e62 198 318 79224489 79226519 708 70
NP 789740.1 NC 006585.4 Canis lupus familiaris isolate Tasha breed boxer chromosome 3 alternate assembly Dog10K Boxer Tasha whole genome shotgun sequence 80.1073 4.05933e13 106 170 1538247 1539491 426 79
AAL38193.1 NC 006590.4 Canis lupus familiaris isolate Tasha breed boxer chromosome 8 alternate assembly Dog10K Boxer Tasha whole genome shotgun sequence 288.5 9.42945e78 222 316 73382353 73384095 601 32
AAD30048.1 NC 006590.4 Canis lupus familiaris isolate Tasha breed boxer chromosome 8 alternate assembly Dog10K Boxer Tasha whole genome shotgun sequence 291.582 1.97023e78 202 309 73381063 73382799 597 43
CAA40318.1 NC 006583.4 Canis lupus familiaris isolate Tasha breed boxer chromosome 1 alternate assembly Dog10K Boxer Tasha whole genome shotgun sequence 97.8265 1.88717e18 134 214 49544673 49546259 564 90
NP 789740.1 NC 006593.4 Canis lupus familiaris isolate Tasha breed boxer chromosome 11 alternate assembly Dog10K Boxer Tasha whole genome shotgun sequence 85.5001 1.05161e14 110 173 11536508 11537701 431 82
AAL38193.1 NC 006593.4 Canis lupus familiaris isolate Tasha breed boxer chromosome 11 alternate assembly Dog10K Boxer Tasha whole genome shotgun sequence 689.108 0 468 646 11536724 11540092 1161 55
YP 009513211.1 NC 006588.4 Canis lupus familiaris isolate Tasha breed boxer chromosome 6 alternate assembly Dog10K Boxer Tasha whole genome shotgun sequence 238.81 1.29842e113 162 243 33607461 33608834 472 49
YP 009513211.1 Gammaretroviruses
AAL38193.1 Gammaretroviruses
AAD30048.1 NC 006615.4 Canis lupus familiaris isolate Tasha breed boxer chromosome 33 alternate assembly Dog10K Boxer Tasha whole genome shotgun sequence 144.821 7.26756e35 139 199 7992384 7993712 480 82
AAD30048.1 NC 006621.4 Canis lupus familiaris isolate Tasha breed boxer chromosome X alternate assembly Dog10K Boxer Tasha whole genome shotgun sequence 273.092 7.81444e78 222 340 18253191 18255293 723 54
AAL38193.1 NC 006592.4 Canis lupus familiaris isolate Tasha breed boxer chromosome 10 alternate assembly Dog10K Boxer Tasha whole genome shotgun sequence 448.743 5.82508e131 241 291 3347313 3348689 460 2
AAL38193.1 NC 006588.4 Canis lupus familiaris isolate Tasha breed boxer chromosome 6 alternate assembly Dog10K Boxer Tasha whole genome shotgun sequence 104.375 1.66634e36 123 187 1175813 1177060 449 63
AAL38193.1 NC 006608.4 Canis lupus familiaris isolate Tasha breed boxer chromosome 26 alternate assembly Dog10K Boxer Tasha whole genome shotgun sequence 360.147 4.75618e160 214 262 31220124 31221368 419 9
YP 009513211.1 NC 006588.4 Canis lupus familiaris isolate Tasha breed boxer chromosome 6 alternate assembly Dog10K Boxer Tasha whole genome shotgun sequence 330.102 4.5233e105 239 310 47585617 47587251 561 56
YP 009513211.1 NC 006594.4 Canis lupus familiaris isolate Tasha breed boxer chromosome 12 alternate assembly Dog10K Boxer Tasha whole genome shotgun sequence 322.013 7.23527e89 197 256 18576593 18577858 445 63
AAL38193.1 NC 006613.4 Canis lupus familiaris isolate Tasha breed boxer chromosome 31 alternate assembly Dog10K Boxer Tasha whole genome shotgun sequence 295.819 0 190 259 37174394 37175611 423 38
YP 009513211.1 NC 006592.4 Canis lupus familiaris isolate Tasha breed boxer chromosome 10 alternate assembly Dog10K Boxer Tasha whole genome shotgun sequence 334.339 6.5876e108 209 261 3348934 3350322 478 55
YP 009513211.1 NC 006595.4 Canis lupus familiaris isolate Tasha breed boxer chromosome 13 alternate assembly Dog10K Boxer Tasha whole genome shotgun sequence 172.17 1.15831e59 171 237 15246 16724 531 85
AAL38193.1 NC 006601.4 Canis lupus familiaris isolate Tasha breed boxer chromosome 19 alternate assembly Dog10K Boxer Tasha whole genome shotgun sequence 297.36 1.39314e80 204 273 22245951 22247309 460 27
YP 009513211.1 NC 006589.4 Canis lupus familiaris isolate Tasha breed boxer chromosome 7 alternate assembly Dog10K Boxer Tasha whole genome shotgun sequence 78.9518 1.16279e12 119 186 19640938 19642173 445 66
AAL38193.1 NC 006583.4 Canis lupus familiaris isolate Tasha breed boxer chromosome 1 alternate assembly Dog10K Boxer Tasha whole genome shotgun sequence 374.015 7.65168e106 232 315 70343957 70345432 500 23
AAL38193.1 NC 006588.4 Canis lupus familiaris isolate Tasha breed boxer chromosome 6 alternate assembly Dog10K Boxer Tasha whole genome shotgun sequence 304.294 7.00827e83 217 291 1289655 1291136 502 25
AAD30048.1 NC 006621.4 Canis lupus familiaris isolate Tasha breed boxer chromosome X alternate assembly Dog10K Boxer Tasha whole genome shotgun sequence 100.908 1.42316e28 122 192 35474646 35475896 447 54
AAL38193.1 NC 006598.4 Canis lupus familiaris isolate Tasha breed boxer chromosome 16 alternate assembly Dog10K Boxer Tasha whole genome shotgun sequence 230.72 2.48112e87 164 230 5084457 5085644 413 46
AAL38193.1 NC 006585.4 Canis lupus familiaris isolate Tasha breed boxer chromosome 3 alternate assembly Dog10K Boxer Tasha whole genome shotgun sequence 130.954 1.66841e28 194 297 81088 83046 696 97
AAL38193.1 NC 006588.4 Canis lupus familiaris isolate Tasha breed boxer chromosome 6 alternate assembly Dog10K Boxer Tasha whole genome shotgun sequence 105.531 1.79884e26 117 159 1263742 1264881 403 60
AAL38193.1 NC 006586.4 Canis lupus familiaris isolate Tasha breed boxer chromosome 4 alternate assembly Dog10K Boxer Tasha whole genome shotgun sequence 131.724 1.05701e28 139 194 87888170 87889390 445 79
AAL38193.1 NC 006605.4 Canis lupus familiaris isolate Tasha breed boxer chromosome 23 alternate assembly Dog10K Boxer Tasha whole genome shotgun sequence 142.124 7.97686e32 119 186 1932617 1933783 400 77
AAL38193.1 NC 006588.4 Canis lupus familiaris isolate Tasha breed boxer chromosome 6 alternate assembly Dog10K Boxer Tasha whole genome shotgun sequence 149.828 3.24515e34 189 264 1194650 1196224 573 95
AFR79239.1 NC 006591.4 Canis lupus familiaris isolate Tasha breed boxer chromosome 9 alternate assembly Dog10K Boxer Tasha whole genome shotgun sequence 190.66 8.53466e49 214 336 57201707 57204046 828 101
AAD30048.1 NC 006597.4 Canis lupus familiaris isolate Tasha breed boxer chromosome 15 alternate assembly Dog10K Boxer Tasha whole genome shotgun sequence 180.644 1.34897e43 127 212 11268829 11270073 422 30
AAD30048.1 NC 006593.4 Canis lupus familiaris isolate Tasha breed boxer chromosome 11 alternate assembly Dog10K Boxer Tasha whole genome shotgun sequence 257.684 5.28211e77 178 257 34327210 34328691 507 33
YP 009513211.1 NC 006621.4 Canis lupus familiaris isolate Tasha breed boxer chromosome X alternate assembly Dog10K Boxer Tasha whole genome shotgun sequence 584.719 1.57637e177 387 481 17739618 17741906 787 77
AAD30048.1 NC 006589.4 Canis lupus familiaris isolate Tasha breed boxer chromosome 7 alternate assembly Dog10K Boxer Tasha whole genome shotgun sequence 255.758 1.35525e112 175 260 72918655 72920148 511 39
YP 009513211.1 NC 006602.4 Canis lupus familiaris isolate Tasha breed boxer chromosome 20 alternate assembly Dog10K Boxer Tasha whole genome shotgun sequence 528.865 0 351 451 33581409 33583649 754 102
YP 009513211.1 NC 006586.4 Canis lupus familiaris isolate Tasha breed boxer chromosome 4 alternate assembly Dog10K Boxer Tasha whole genome shotgun sequence 646.351 0 373 458 70408880 70410856 665 40
AAL38193.1 NC 006590.4 Canis lupus familiaris isolate Tasha breed boxer chromosome 8 alternate assembly Dog10K Boxer Tasha whole genome shotgun sequence 362.844 3.09044e102 231 311 72587951 72589447 513 43
AAD30048.1 NC 006585.4 Canis lupus familiaris isolate Tasha breed boxer chromosome 3 alternate assembly Dog10K Boxer Tasha whole genome shotgun sequence 373.244 8.02593e129 288 415 41518474 41520951 846 53
YP 009513211.1 NC 006583.4 Canis lupus familiaris isolate Tasha breed boxer chromosome 1 alternate assembly Dog10K Boxer Tasha whole genome shotgun sequence 270.011 7.62481e72 185 261 74944200 74945639 487 43
AAD30048.1 NC 006583.4 Canis lupus familiaris isolate Tasha breed boxer chromosome 1 alternate assembly Dog10K Boxer Tasha whole genome shotgun sequence 181.03 1.11871e43 149 223 26374405 26375850 501 33
AAD30048.1 NC 006621.4 Canis lupus familiaris isolate Tasha breed boxer chromosome X alternate assembly Dog10K Boxer Tasha whole genome shotgun sequence 103.219 3.33091e87 122 194 38798150 38799472 459 48
YP 009513211.1 NC 006590.4 Canis lupus familiaris isolate Tasha breed boxer chromosome 8 alternate assembly Dog10K Boxer Tasha whole genome shotgun sequence 776.163 0 471 577 7973689 7976382 901 31
AAL38193.1 NC 006583.4 Canis lupus familiaris isolate Tasha breed boxer chromosome 1 alternate assembly Dog10K Boxer Tasha whole genome shotgun sequence 337.421 2.11708e156 213 268 103491250 103492500 419 9
AAL38193.1 NC 006618.4 Canis lupus familiaris isolate Tasha breed boxer chromosome 36 alternate assembly Dog10K Boxer Tasha whole genome shotgun sequence 221.476 2.6351e56 185 255 3560156 3561601 509 53
AAL38193.1 NC 006588.4 Canis lupus familiaris isolate Tasha breed boxer chromosome 6 alternate assembly Dog10K Boxer Tasha whole genome shotgun sequence 72.0182 1.25529e23 105 157 1277216 1278331 416 79
AAL38193.1 NC 006585.4 Canis lupus familiaris isolate Tasha breed boxer chromosome 3 alternate assembly Dog10K Boxer Tasha whole genome shotgun sequence 423.32 2.3134e122 248 300 36216026 36217411 483 28
YP 009513211.1 NC 006591.4 Canis lupus familiaris isolate Tasha breed boxer chromosome 9 alternate assembly Dog10K Boxer Tasha whole genome shotgun sequence 397.512 0 260 330 11232815 11234449 575 70
AAL38193.1 NC 006621.4 Canis lupus familiaris isolate Tasha breed boxer chromosome X alternate assembly Dog10K Boxer Tasha whole genome shotgun sequence 516.538 4.85548e154 279 334 38799415 38800890 494 5
AAL38193.1 NC 006616.4 Canis lupus familiaris isolate Tasha breed boxer chromosome 34 alternate assembly Dog10K Boxer Tasha whole genome shotgun sequence 478.789 1.64503e144 366 486 6804368 6807016 924 69
YP 009513211.1 NC 006616.4 Canis lupus familiaris isolate Tasha breed boxer chromosome 34 alternate assembly Dog10K Boxer Tasha whole genome shotgun sequence 395.971 0 287 379 6804029 6805987 684 79
