# Supplementary material for: The Unique Immune System of Bats: An Evolutionary Analysis and Bibliometric Study
Source: Ecol Evol. 2024 Nov 24;14(11):e70614. doi: 10.1002/ece3.70614 (PMC11586106; doi:10.1002/ece3.70614)
Supplement: Supplementary file 1 — Data S1. [file ECE3-14-e70614-s001.zip › ece370614-sup-0001-DataS1 /Figure S11. The phylogenetic tree of the integrated ERVs in the Felis catus genome.pdf]

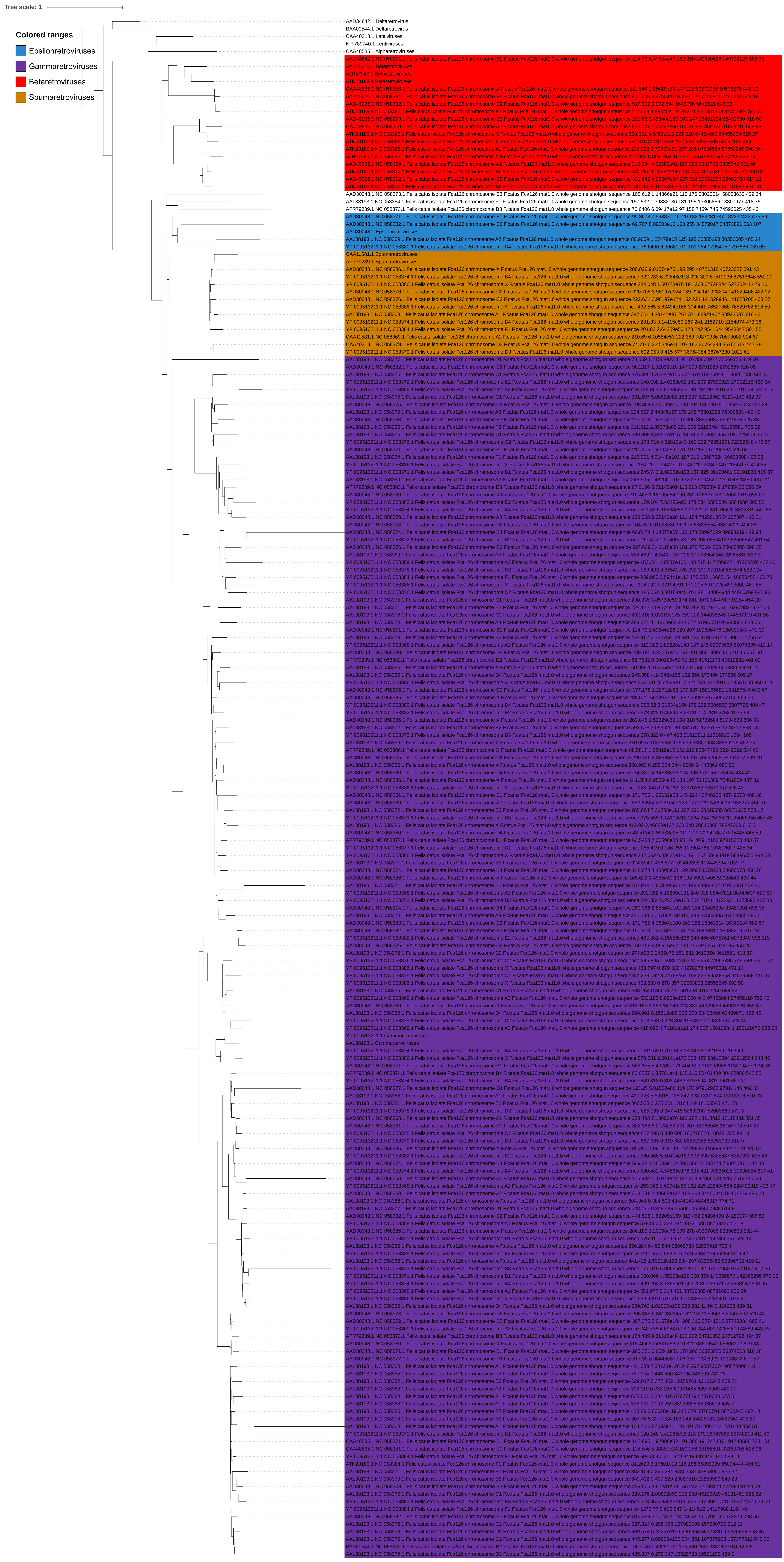

Tree scale: 1
Colored ranges
Epsilonretroviruses
Gammaretroviruses
Betaretroviruses
Spumaretroviruses
AAD34842.1 Deltaretrovirus
BAA00544.1 Deltaretrovirus
CAA40318.1 Lentiviruses
NP 789740.1 Lentiviruses
CAA48535.1 Alpharetroviruses
AAD45226.1 Betaretroviruses
AJR27940.1 Betaretroviruses
ATN28189.1 Betaretroviruses
AAD30048.1 Epsilonretroviruses
CAA11581.1 Spumaretroviruses
AFR79239.1 Spumaretroviruses
YP 009513211.1 Gammaretroviruses
AAL38193.1 Gammaretroviruses
