# Supplementary material for: The Unique Immune System of Bats: An Evolutionary Analysis and Bibliometric Study
Source: Ecol Evol. 2024 Nov 24;14(11):e70614. doi: 10.1002/ece3.70614 (PMC11586106; doi:10.1002/ece3.70614)
Supplement: Supplementary file 1 — Data S1. [file ECE3-14-e70614-s001.zip › ece370614-sup-0001-DataS1 /Supplemental materials.docx]

**Supplemental materials**

**INVENTORY OF SUPPLEMENTAL ITEMS**

1. **Supplemental Materials and Methods**

Supplementary Appendix 1.

1. **Seventeen supplemental figures:**

Figure S1. The divergence time tree of 28 mammals.

Figure S2. The phylogenetic tree of the integrated ERVs in the genome of *Hipposideros armiger.*

Figure S3. The phylogenetic tree of the integrated ERVs in the genome of *Rousettus aegyptiacus*.

Figure S4. The phylogenetic tree of the integrated ERVs in the genome of *Pteropus vampyrus*.

Figure S5. The phylogenetic tree of the integrated ERVs in the genome of *Pteropus alecto*.

Figure S6. The phylogenetic tree of the integrated ERVs in the genome of *Sturnira hondurensis*.

Figure S7. The phylogenetic tree of the integrated ERVs in the genome of *Phyllostomus discolor*.

Figure S8. The phylogenetic tree of the integrated ERVs in the genome of *Molossus molossus*.

Figure S9. The phylogenetic tree of the integrated ERVs in the genome of *Myotis myotis*.

Figure S10. The phylogenetic tree of the integrated ERVs in the genome of *Myotis lucifugus*.

Figure S11. The phylogenetic tree of the integrated ERVs in the genome of *Felis catus*.

Figure S12. The phylogenetic tree of the integrated ERVs in the genome of *Canis lupus familiaris*.

Figure S13. The phylogenetic tree of the integrated ERVs in the genome of *Equus caballus*.

Figure S14. The phylogenetic tree of the integrated ERVs in the genome of *Sus scrofa*.

Figure S15. The phylogenetic tree of the integrated ERVs in the genome of *Sorex araneus*.

Figure S16. The phylogenetic tree of the integrated ERVs in the genome of *Homo sapiens*.

Figure S17. The phylogenetic tree of the integrated ERVs in the genome of *Mus musculus*.

1. **Fifteen supplemental tables:**

Table S1. Information of 28 mammalian genomes used for comparative and phylogenetic analyses.

Table S2. The probe sequences for the identification of endogenous retroviruses.

Table S3. Endogenous retrovirus sequences integrated in mammal genomes.

Table S4. The expansion and contraction gene families of all the branches in the phylogenetic species tree.

Table S5. The expansive gene families in Chiroptera.

Table S6. The gene set analysis results of the expansive gene families in Chiroptera.

Table S7. The contracted gene families in Chiroptera.

Table S8. The gene set results of the contracted gene families in Chiroptera.

Table S9. Number of annual publications in the field of bat immune from 1970 to 2023.

Table S10. The publiction lists of 1054 papers.

Table S11. Top 10 countries based on count.

Table S12. Top 10 institutes based on count.

Table S13. Details of the top 10 clusters.

Table S14. Top 103 references with the strongest citation bursts.

Table S15. 32 references with the strongest citation bursts lasting until 2023.
