# Supplementary material for: The Unique Immune System of Bats: An Evolutionary Analysis and Bibliometric Study
Source: Ecol Evol. 2024 Nov 24;14(11):e70614. doi: 10.1002/ece3.70614 (PMC11586106; doi:10.1002/ece3.70614)
Supplement: Supplementary file 1 — Data S1. [file ECE3-14-e70614-s001.zip › ece370614-sup-0001-DataS1 /Figure S10. The phylogenetic tree of the integrated ERVs in the Myotis lucifugus genome.pdf]

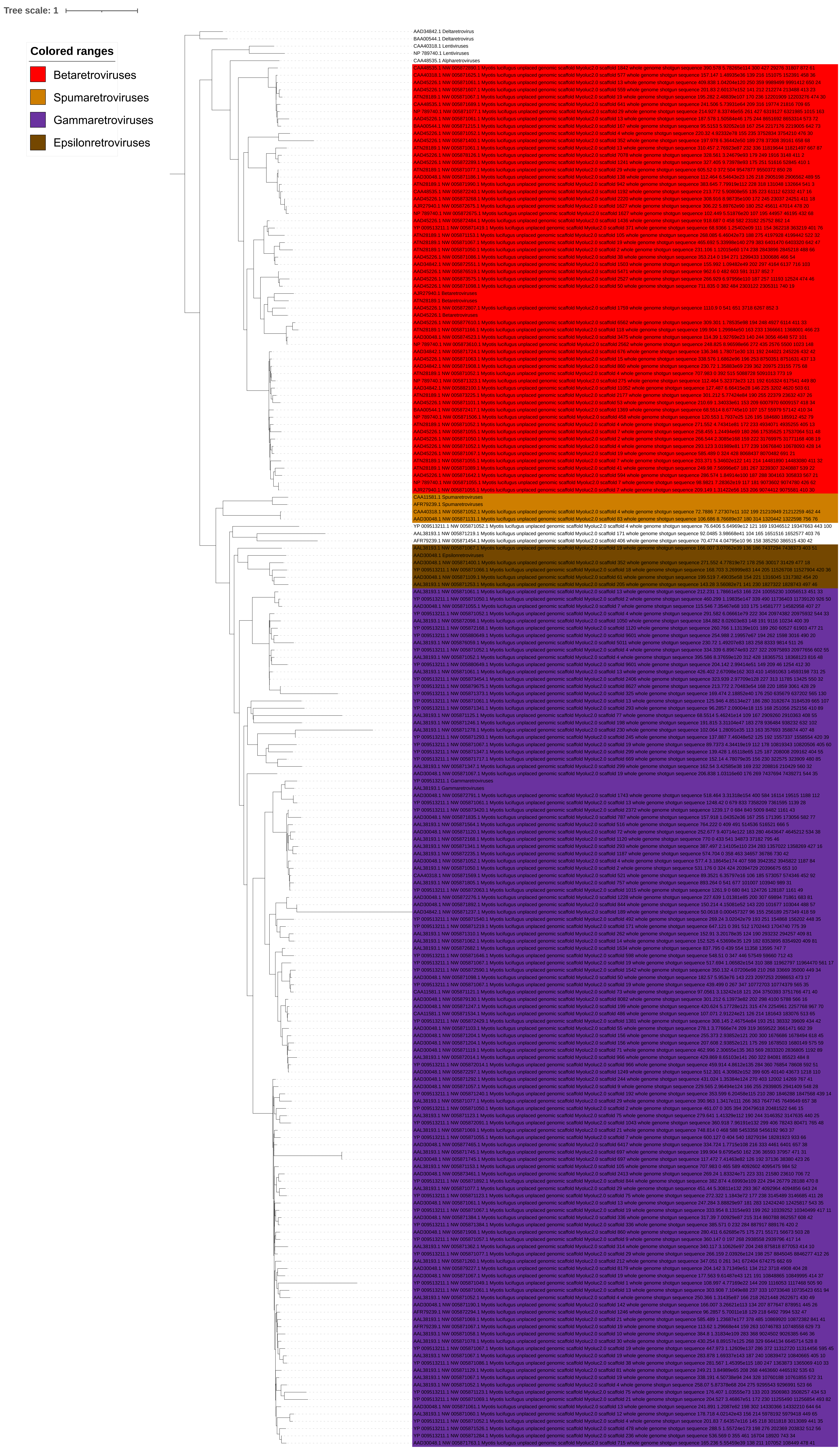
Tree scale: 1
Colored ranges
Betaretroviruses
Spumaretroviruses
Gammaretroviruses
Epsilonretroviruses
