# Supplementary figures and images for: The Unique Immune System of Bats: An Evolutionary Analysis and Bibliometric Study
Source: Ecol Evol. 2024 Nov 24;14(11):e70614. doi: 10.1002/ece3.70614 (PMC11586106; doi:10.1002/ece3.70614)

Tree scale: 1

## Colored ranges

- Spumaretroviruses
- Betaretroviruses
- Gammaretroviruses

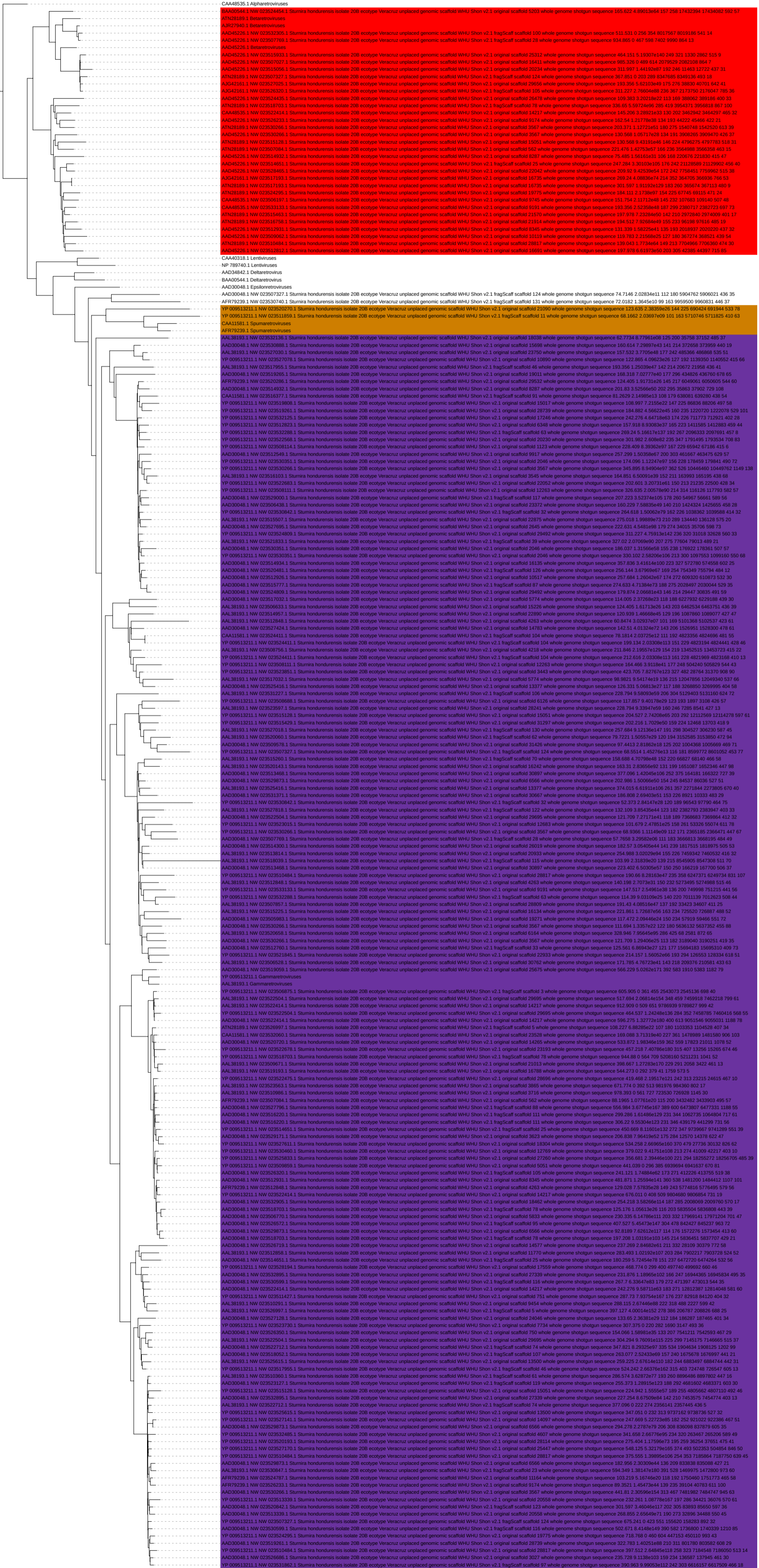

Supplement: Supplementary file 1 — Data S1. [file ECE3-14-e70614-s001.zip › ece370614-sup-0001-DataS1 /Figure S6. The phylogenetic tree of the integrated ERVs in the Sturnira hondurensis genome.pdf]

Tree scale: 1

Colored ranges

- Betaretroviruses
- Gammaretroviruses
- Epsilonretroviruses

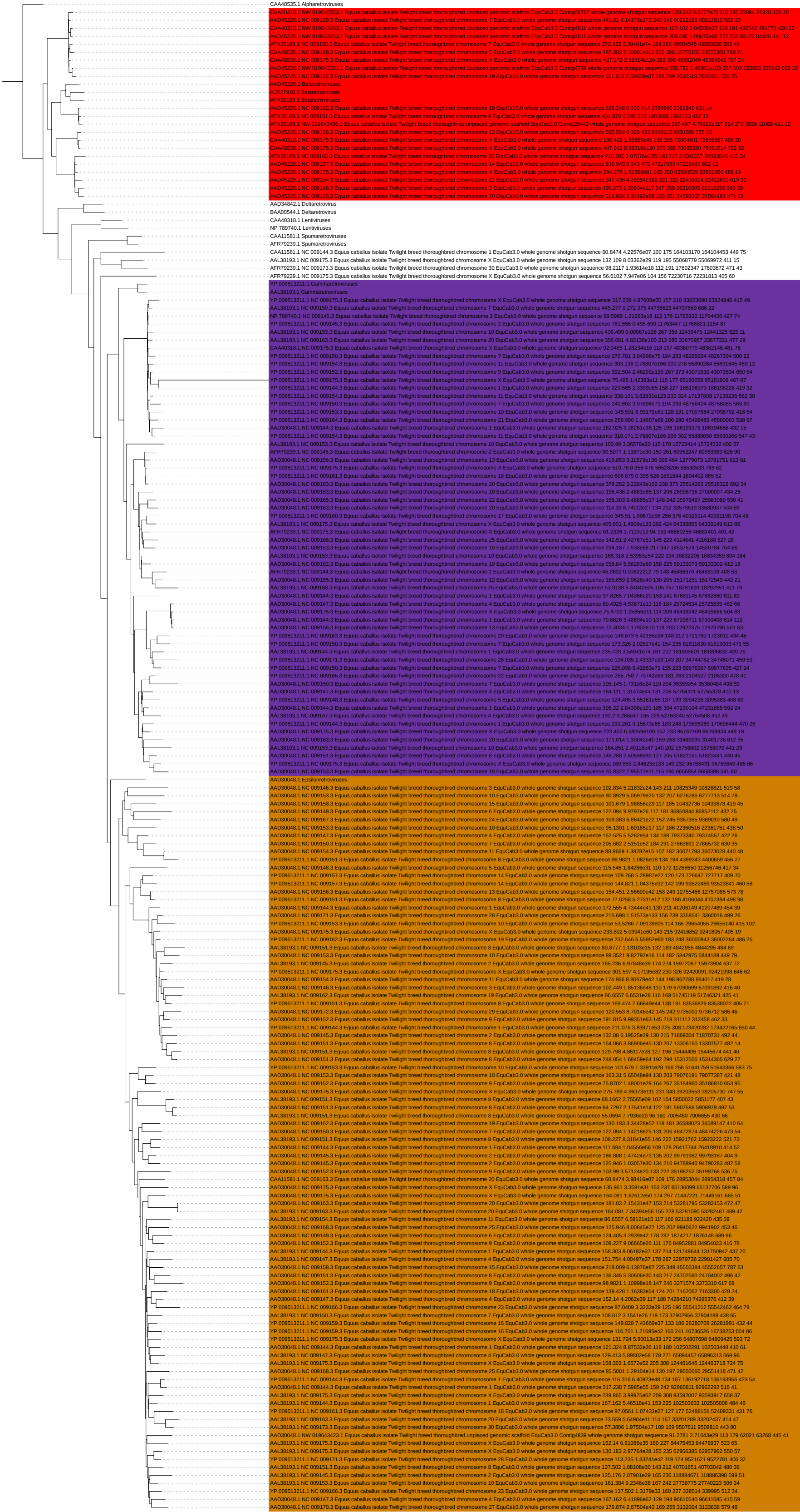

Supplement: Supplementary file 1 — Data S1. [file ECE3-14-e70614-s001.zip › ece370614-sup-0001-DataS1 /Figure S13. The phylogenetic tree of the integrated ERVs in the Equus caballus genome.pdf]

Tree scale: 1

Colored ranges

- Betaretroviruses
- Gammaretroviruses
- Spumaretroviruses

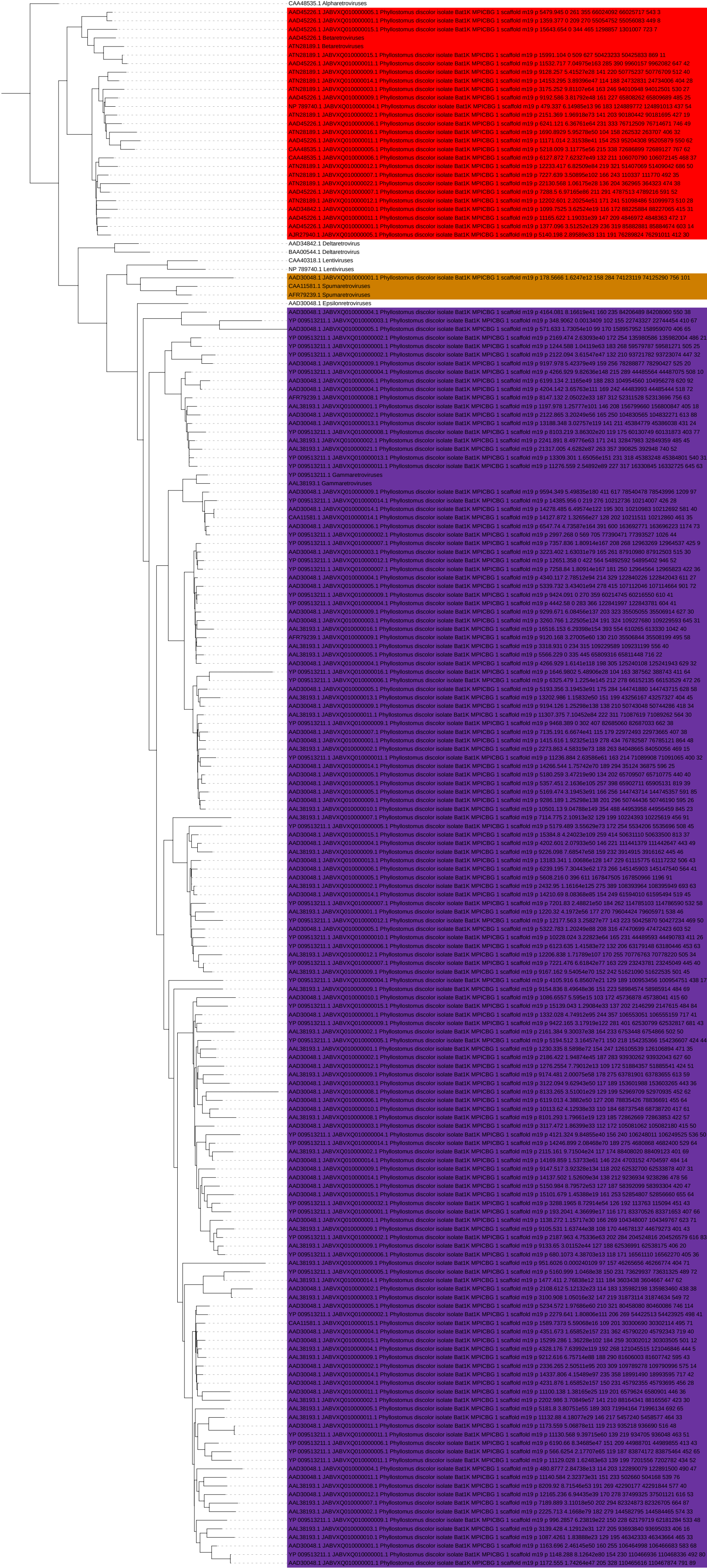

Supplement: Supplementary file 1 — Data S1. [file ECE3-14-e70614-s001.zip › ece370614-sup-0001-DataS1 /Figure S7. The phylogenetic tree of the integrated ERVs in the Phyllostomus discolor genome.pdf]

## Colored ranges

- Betaretroviruses
- Epsilonretroviruses
- Gammaretroviruses

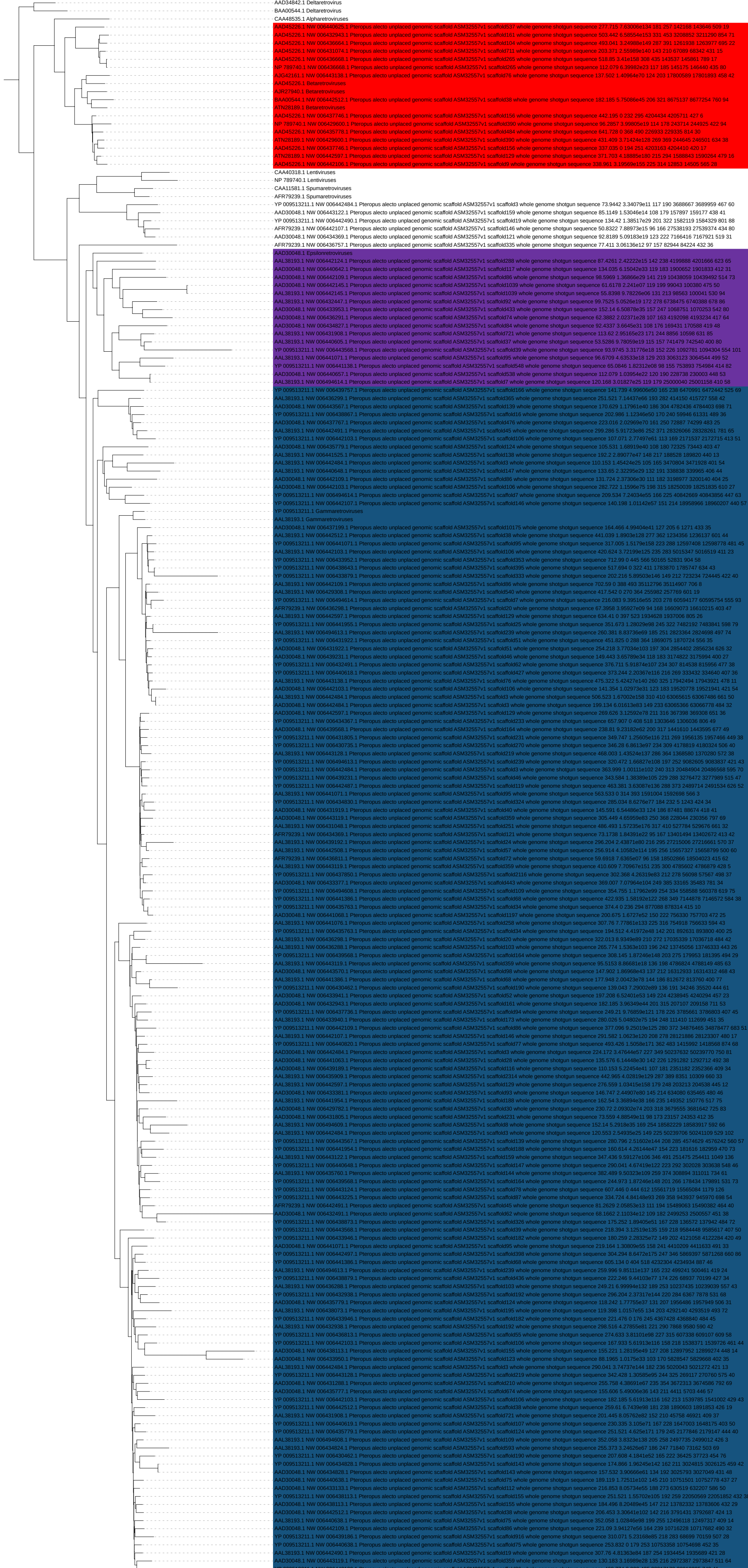

Supplement: Supplementary file 1 — Data S1. [file ECE3-14-e70614-s001.zip › ece370614-sup-0001-DataS1 /Figure S5. The phylogenetic tree of the integrated ERVs in the Pteropus alecto genome.pdf]

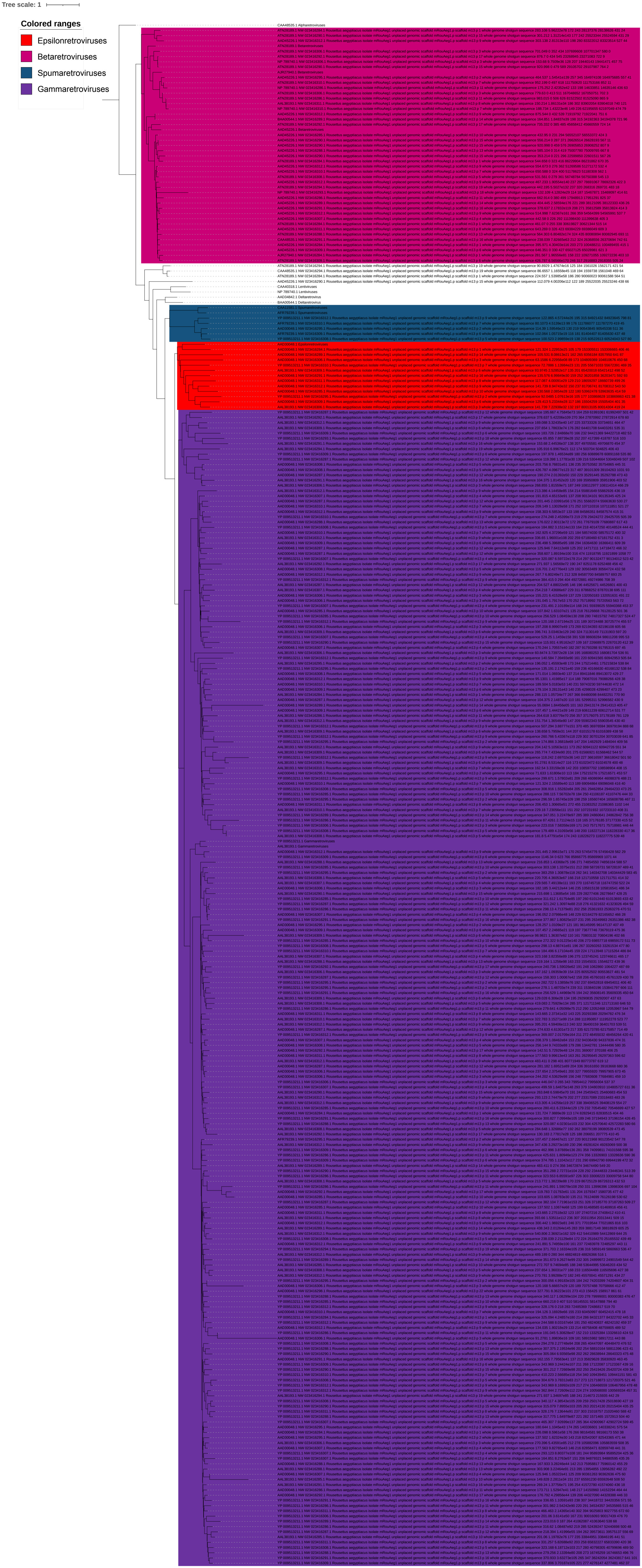

Supplement: Supplementary file 1 — Data S1. [file ECE3-14-e70614-s001.zip › ece370614-sup-0001-DataS1 /Figure S3. The phylogenetic tree of the integrated ERVs in the Rousettus aegyptiacus genome.pdf]

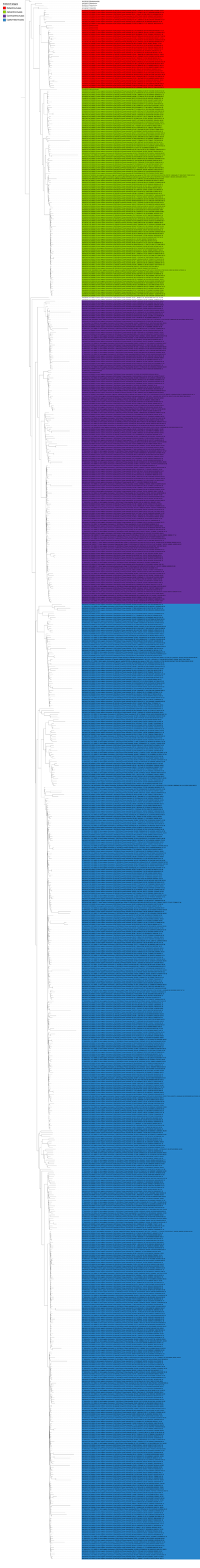

Supplement: Supplementary file 1 — Data S1. [file ECE3-14-e70614-s001.zip › ece370614-sup-0001-DataS1 /Figure S16. The phylogenetic tree of the integrated ERVs in the Homo sapiens genome.pdf]

Tree scale: 1

Colored ranges

- Epsilonretroviruses
- Betaretroviruses
- Gammaretroviruses

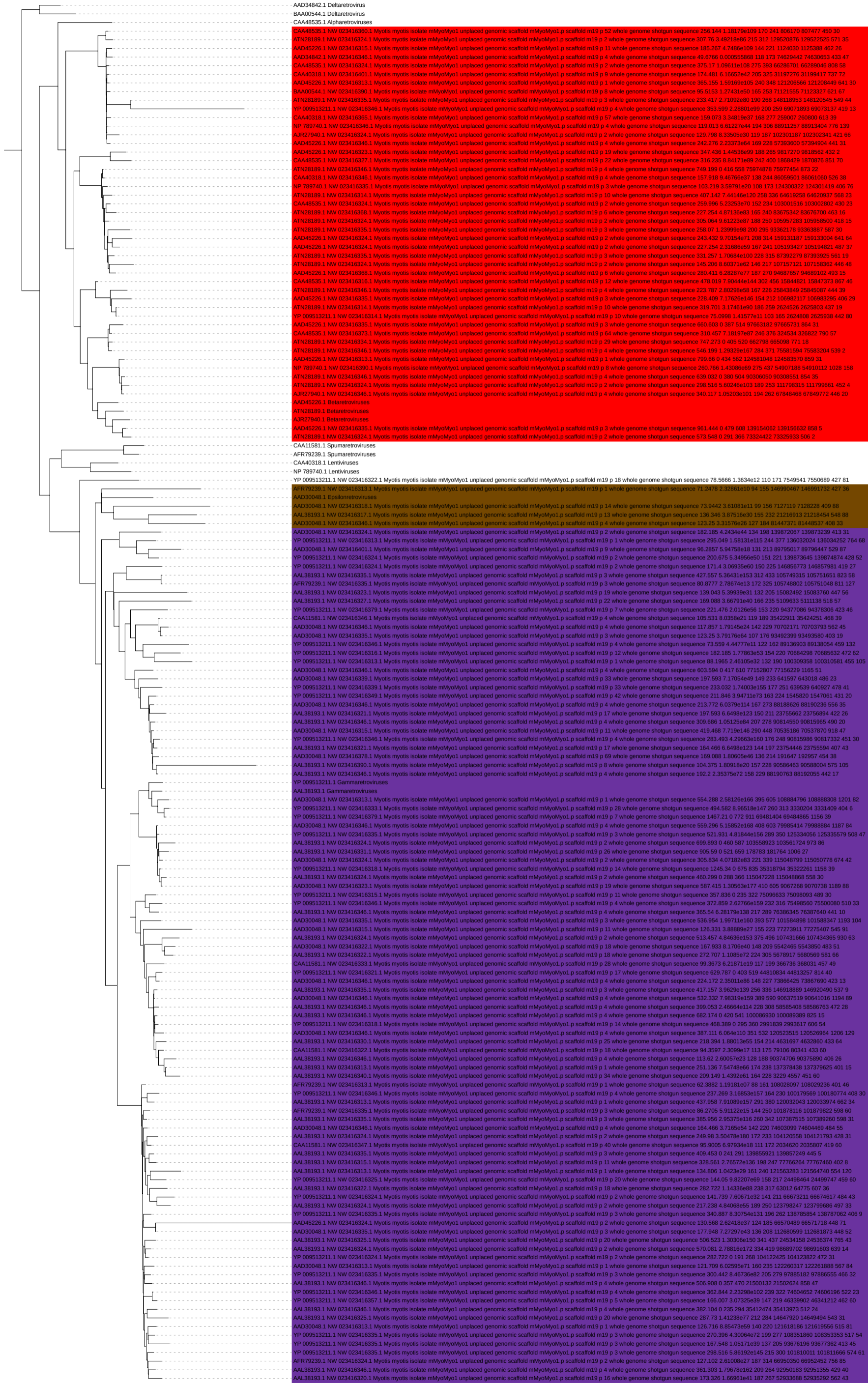

Supplement: Supplementary file 1 — Data S1. [file ECE3-14-e70614-s001.zip › ece370614-sup-0001-DataS1 /Figure S9. The phylogenetic tree of the integrated ERVs in the Myotis myotis genome.pdf]

Tree scale: 1

### Colored ranges

- Betaretroviruses
- Epsilonretroviruses
- Gammaretroviruses

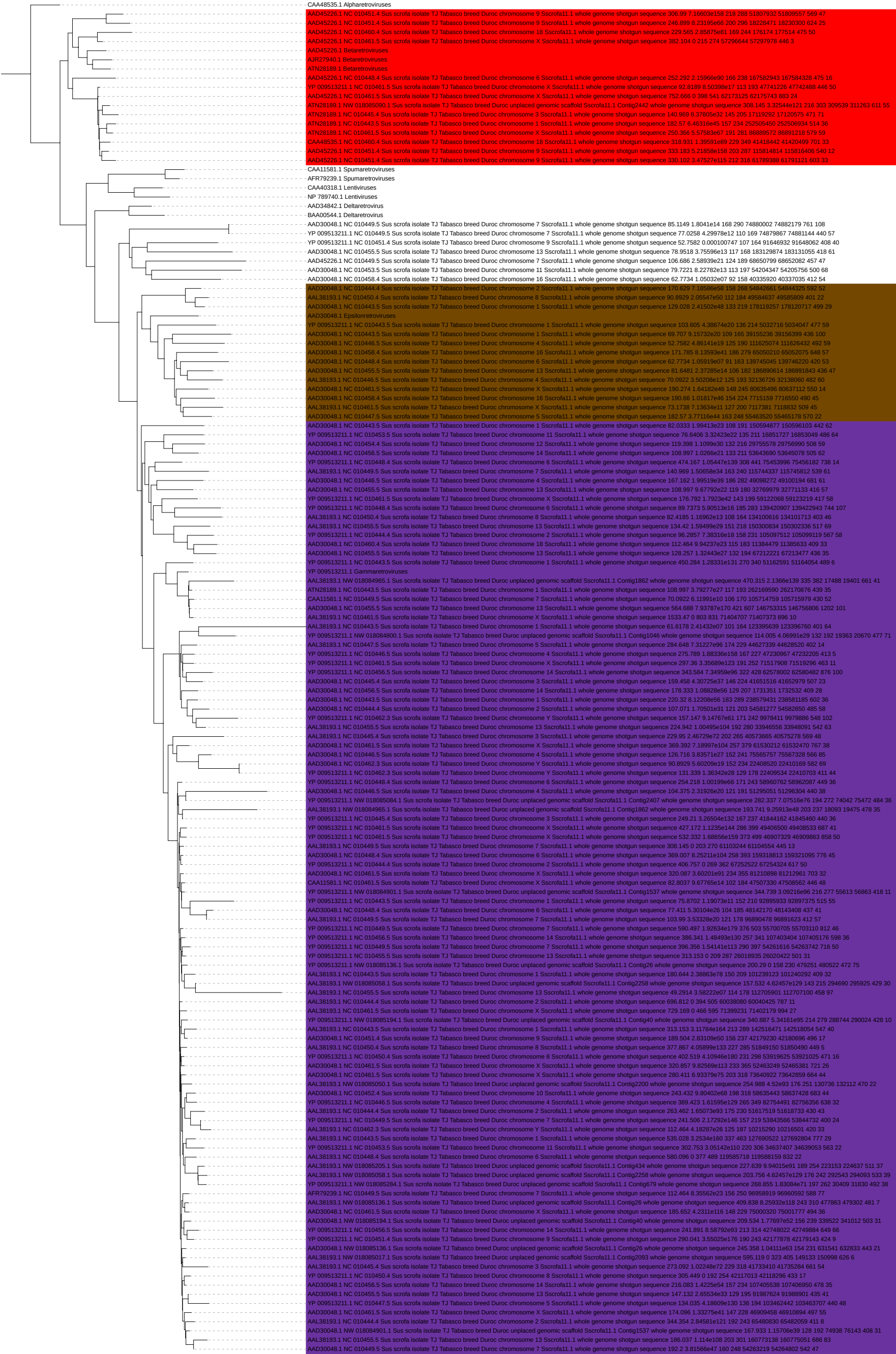

Supplement: Supplementary file 1 — Data S1. [file ECE3-14-e70614-s001.zip › ece370614-sup-0001-DataS1 /Figure S14. The phylogenetic tree of the integrated ERVs in the Sus scrofa genome.pdf]

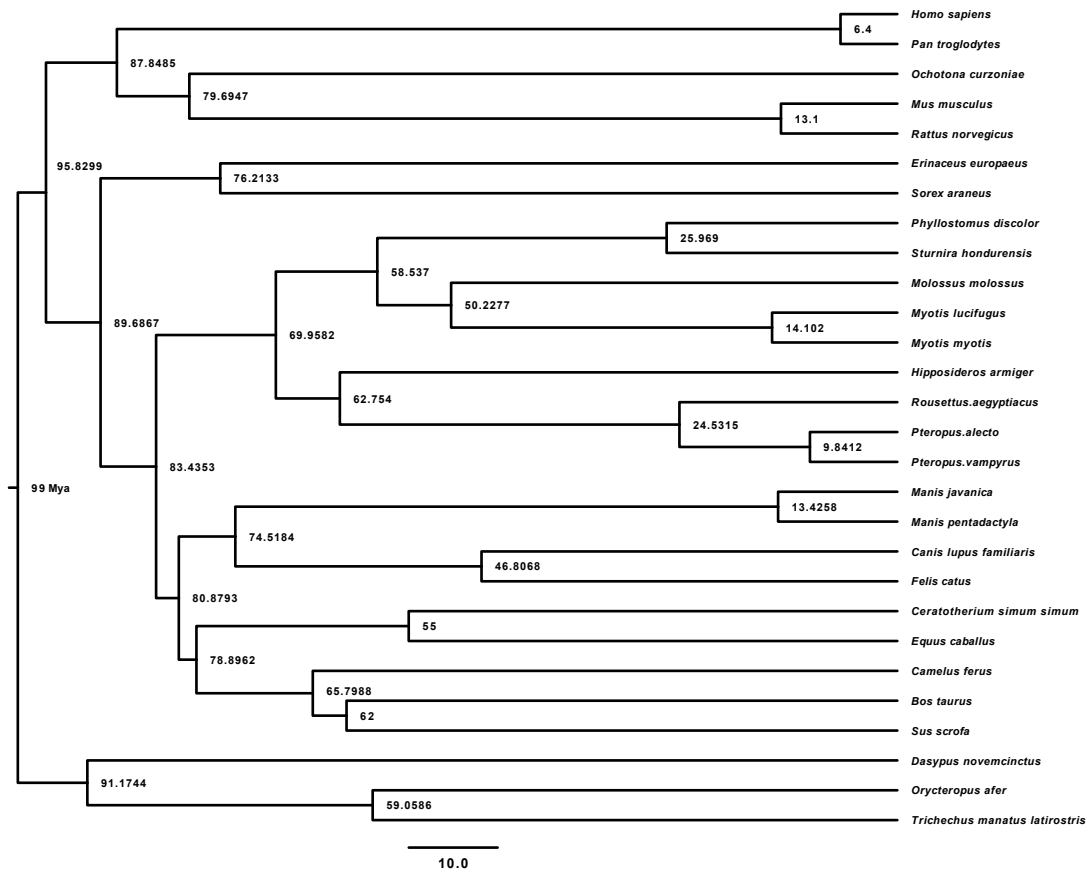

Supplement: Supplementary file 1 — Data S1. [file ECE3-14-e70614-s001.zip › ece370614-sup-0001-DataS1 /Figure S1. The divergence time tree of 28 mammals.pdf]

Tree scale: 1

Colored ranges

- Gammaretroviruses
- Spumaretroviruses
- Betaretroviruses

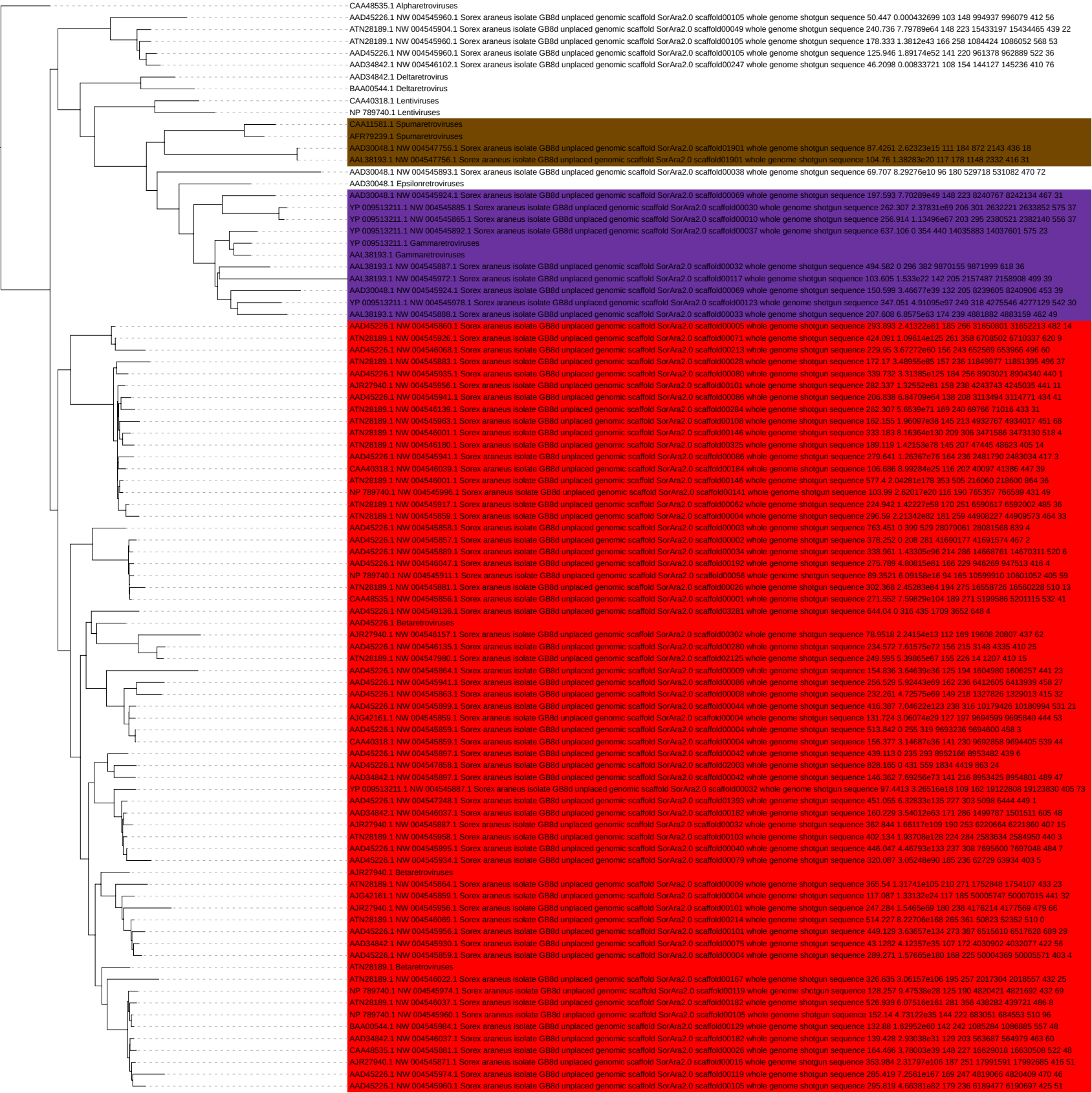

Supplement: Supplementary file 1 — Data S1. [file ECE3-14-e70614-s001.zip › ece370614-sup-0001-DataS1 /Figure S15. The phylogenetic tree of the integrated ERVs in the Sorex araneus genome.pdf]
