# Supplementary material for: The Unique Immune System of Bats: An Evolutionary Analysis and Bibliometric Study
Source: Ecol Evol. 2024 Nov 24;14(11):e70614. doi: 10.1002/ece3.70614 (PMC11586106; doi:10.1002/ece3.70614)
Supplement: Supplementary file 1 — Data S1. [file Supplemental data.pdf › ece370614-sup-0001-DataS1 /Supplemental data.pdf]

FN Clarivate Analytics Web of Science
VR 1.0
PT J
AU ROBBINS, LW
   SARICH, VM
AF ROBBINS, LW
   SARICH, VM
TI EVOLUTIONARY RELATIONSHIPS IN THE FAMILY EMBALLONURIDAE
(CHIROPTERA)
SO JOURNAL OF MAMMALOGY
LA English
DT Article
AB Protein electrophoresis and immunology were used to test
hypotheses concerning phylogenetic relationships within the family
Emballonuridae and the relationship of the family Rhinopomatidae to
the Emballonuridae. These data indicate the presence of three major
subdivisions within the family. The eight genera [Balantiopteryx,
Centronycteris, Cormura, Diclidurus, Peronymus, Peropteryx,
Rhynchonycteris and Saccoptery from the New World form one
monophyletic group, the Old World genera Taphozous and Saccolaimus
form the second, and Emballonura and Coleura the third. The
Emballonura-Coleura lineage is more closely related to the New World
genera than to the other Old World group. The family Rhinopomatidae
was found to be distantly related to both the Emballonuridae and the
Vespertilionidae.
C1 TEXAS TECH UNIV, DEPT BIOL SCI, LUBBOCK, TX 79409 USA.
   UNIV CALIF BERKELEY, DEPT ANTHROPOL & BIOCHEM, BERKELEY, CA 94720
USA.
C3 Texas Tech University System; Texas Tech University; University
of
   California System; University of California Berkeley
CR [Anonymous], 1972, Studies in Genetics, University of Texas
Publication
   [Anonymous], 1958, CIENCIAS ZOOLOGICAS
   ARNOW D, 1982, J CHEM PHYS, V77, P1
   AYALA FJ, 1972, GENETICS, V70, P113
   BAKER RJ, 1981, J MAMMAL, V62, P665, DOI 10.2307/1380587
   Barghoorn S.F., 1977, American Museum Novitates, V2618, P1
   BAVERSTOCK PR, 1979, SYST ZOOL, V28, P214, DOI 10.2307/2412524
   Champion A.B., 1974, P397
   CORBET GB, 1980, BRIT MUS NAT HIST LO
   CRONIN JE, 1976, NATURE, V260, P700, DOI 10.1038/260700a0
   CRONIN JE, 1975, J HUM EVOL, V4, P357, DOI
10.1016/0047-2484(75)90018-4
   DAWSON MR, 1976, SCIENCE, V192, P781, DOI 10.1126/
science.192.4241.781
   FARRIS JS, 1972, AM NAT, V106, P645, DOI 10.1086/282802
   FITCH WM, 1967, SCIENCE, V155, P279, DOI 10.1126/
science.155.3760.279
   GREENBAUM IF, 1976, SYST ZOOL, V25, P15, DOI 10.2307/2412775
   Hall E.R., 1981, The mammals of North America, V1, P1
   HIGHTON R, 1979, SYST ZOOL, V28, P597
   HILL JE, 1974, BRIT MUS NAT HIST ZO, V27, P304
   Honacki J.H., 1982, Mammal species of the world: A taxonomic and

geographic reference, Vfirst
HONEYCUTT RL, 1981, J MAMMAL, V62, P805, DOI 10.2307/1380602
HOOD CS, 1986, J MAMMAL, V67, P705, DOI 10.2307/1381131
KOOPMAN KF, 1982, SPECIAL PUBLICATIONS, V6, P273
KOOPMAN KF, 1970, BATS, P22
MAXSON LR, 1979, EVOLUTION, V33, P1057, DOI 10.1111/j.1558-5646.1979.tb04761.x
MAXSON LR, 1974, SCIENCE, V185, P66, DOI 10.1126/science.185.4145.66
MAXSON LR, 1975, NATURE, V255, P397, DOI 10.1038/255397a0
MILLER GS, 1907, B US NAT MUS, V56, P1
NAKANISHI M, 1969, SCIENCE, V163, P681, DOI 10.1126/science.163.3868.681
NEI M, 1972, AM NAT, V106, P283, DOI 10.1086/282771
OJASTI J, 1972, ACTA BIOL VENEZ, V7, P421
PATTON JC, 1978, SYST ZOOL, V27, P449, DOI 10.2307/2412927
PRAGER EM, 1971, J BIOL CHEM, V246, P5978
PRAGER EM, 1971, J BIOL CHEM, V246, P7010
ROBBINS LW, 1983, THESIS TEXAS TU LUBB
SARICH VM, 1973, NATURE, V245, P218, DOI 10.1038/245218a0
SARICH VM, 1967, P NATL ACAD SCI USA, V58, P142, DOI 10.1073/pnas.58.1.142
SARICH VM, 1977, NATURE, V265, P24, DOI 10.1038/265024a0
SARICH VM, 1969, SYST ZOOL, V18, P286, DOI 10.2307/2412325
SARICH VM, 1969, SYST ZOOL, V18, P416, DOI 10.2307/2412185
SARICH VM, 1977, MOL ANTHR, P141
Selander RK, 1971, U TEXAS PUBL, V7103, P49
SIMPSON GG, 1945, B AM MUS NAT HIST, V85, P1
Smith J.D., 1976, BIOL BATS NEW WORLD, V10, P49
Sneath P. H. A., NUMERICAL TAXONOMY
SOKAL RR, 1981, SYST ZOOL, V30, P309, DOI 10.2307/2413252
STARRETT ANDREW, 1964, ZOOLOGICA [NEW YORK], V49, P53
WATROUS LE, 1981, SYST ZOOL, V30, P1, DOI 10.2307/2992297
NR 47
TC 22
Z9 26
U1 1
U2 5
PU OXFORD UNIV PRESS INC
PI CARY
PA JOURNALS DEPT, 2001 EVANS RD, CARY, NC 27513 USA
SN 0022-2372
EI 1545-1542
J9 J MAMMAL
JI J. Mammal.
PD FEB 25
PY 1988
VL 69
IS 1
BP 1
EP 13
DI 10.2307/1381742
PG 13
WC Zoology

WE Science Citation Index Expanded (SCI-EXPANDED)
SC Zoology
GA M3632
UT WOS:A1988M363200001
DA 2024-10-21
ER

PT J
AU Miller-Butterworth, CM
   Murphy, WJ
   O'Brien, SJ
   Jacobs, DS
   Springer, MS
   Teeling, EC
AF Miller-Butterworth, Cassandra M.
   Murphy, William J.
   O'Brien, Stephen J.
   Jacobs, David S.
   Springer, Mark S.
   Teeling, Emma C.
TI A family matter:: Conclusive resolution of the taxonomic position of the
   long-fingered bats, <i>Miniopterus</i>
SO MOLECULAR BIOLOGY AND EVOLUTION
LA English
DT Article
DE Miniopterus; long-fingered bats; Vespertilionidae; Molossidae;
   Chiroptera; phylogeny; molecular dating
ID SCHREIBERSII VESPERTILIONIDAE; PHYLOGENETIC-RELATIONSHIPS;
   REPRODUCTIVE-CYCLE; VOMERONASAL ORGAN; EVOLUTION; CHIROPTERA;
   ECHOLOCATION; MORPHOLOGY; BIOGEOGRAPHY; MAMMALIA
AB The long-fingered bats (Miniopterus sp.) are among the most widely distributed mammals in the world. However, despite recent focus on the systematics of these bats, their taxonomic position has not been resolved. Traditionally, they are considered to be sole members of Miniopterinae, I of 5 subfamilies within the largest family of bats, the Vespertilionidae. However, this classification has increasingly been called into question. Miniopterines differ extensively from other vespertilionids in numerous aspects of morphology, embryology, immunology, and, most recently, genetics. Recent molecular studies have proposed that the miniopterines are sufficiently distinct from vespertilionids that Miniopterinae should be elevated to full familial status. However, controversy remains regarding the relationship of the putative family, Miniopteridae to existing Vespertilionidae and to the closely related free-tailed bats, the Molossidae. We report here the first conclusive analysis of the taxonomic position of Miniopterus relative to all other bat families. We generated one of the largest chiropteran data sets to date, incorporating similar to 11 kb of sequence data from 16 nuclear genes, from representatives of all bat families and 2 Miniopterus species. Our data confirm the distinctiveness of Miniopterus, and we support previous recommendations to elevate these bats to full familial status. We estimate that they diverged from all other bat species approximately 49-38 MYA, which is

comparable to most other bat families. Furthermore, we find very strong support from all phylogenetic methods for a sister group relationship between Miniopteridae and Vespertilionidae. The Molossidae diverged from these lineages approximately 54-43 MYA and form a sister group to the Miniopteridae-Vespertilionidae clade.
C1 Univ Pittsburgh, Grad Sch Publ Hlth, Dept Human Genet, Pittsburgh, PA 15260 USA.
Texas A&M Univ, Coll Vet Med, College Stn, TX 77843 USA.
Natl Canc Inst, Lab Genom Divers, Frederick, MD USA.
Univ Cape Town, Dept Zool, ZA-7700 Rondebosch, South Africa.
Univ Calif Riverside, Dept Biol, Riverside, CA 92521 USA.
Univ Coll Dublin, Sch Biol & Environm Sci, Dublin 2, Ireland.
C3 Pennsylvania Commonwealth System of Higher Education (PCSHE); University
of Pittsburgh; Texas A&M University System; Texas A&M University College
Station; National Institutes of Health (NIH) - USA; NIH National Cancer
Institute (NCI); University of Cape Town; University of California
System; University of California Riverside; University College Dublin
RP Miller-Butterworth, CM (corresponding author), Univ Pittsburgh, Grad Sch Publ Hlth, Dept Human Genet, Pittsburgh, PA 15260 USA.
EM cbutterworth@hgen.pitt.edu; emma.teeling@ucd.ie
RI Miller-Butterworth, Cassandra/H-5994-2013; OBRIEN,
STEPHEN/ABD-1346-2020; Jacobs, David/L-9118-2013
OI OBRIEN, STEPHEN/0000-0001-7857-0757; Jacobs, David/0000-0002-3243-8571;
Teeling, Emma/0000-0002-3309-1346
FU NCI NIH HHS [N01-CO-12400] Funding Source: Medline
CR AGRAWAL V C, 1973, Anatomischer Anzeiger, V133, P180
[Anonymous], 1907, B US NATL MUSEUM
[Anonymous], 1998, MAMMALS AUSTR
[Anonymous], THESIS U CALIFORNIA
Archibald J. David, 2001, Journal of Mammalian Evolution, V8, P107, DOI 10.1023/A:1011317930838
BERNARD R T F, 1980, Annals of the Transvaal Museum, V32, P55
Bernard RTF, 1996, J ZOOL, V238, P13, DOI 10.1111/j.1469-7998.1996.tb05376.x
Bhatnagar KP, 2001, ACTA CHIROPTEROL, V3, P119
BREED WG, 1985, MAMMALIA, V49, P105, DOI 10.1515/mamm.1985.49.1.105
CAPANNA E, 1971, CARYOLOGIA, V24, P471, DOI 10.1080/00087114.1971.10796455
COOPER JG, 1976, J ANAT, V122, P571
DEQUEIROZ A, 1993, SYST BIOL, V42, P368
DOBSON G.E., 1875, Annals and Magazine of Natural History, V16, P345, DOI DOI 10.1080/00222937508681865
DWYER P. D., 1963, AUSTRALIAN JOUR ZOOL, V11, P219, DOI 10.1071/ZO9630219
DWYER P. D., 1966, AUSTRALIAN J ZOOL, V14, P1073, DOI 10.1071/ZO9661073
Eick GN, 2005, MOL BIOL EVOL, V22, P1869, DOI 10.1093/molbev/

msi180
FELSENSTEIN J, 1984, EVOLUTION, V38, P16, DOI 10.1111/j.1558-5646.1984.tb00255.x
FINDLEY JS, 1972, J MAMMAL, V53, P429, DOI 10.2307/1379035
GOPALAKRISHNA A, 1983, CURR SCI INDIA, V52, P1176
Gregory TR, 2007, NUCLEIC ACIDS RES, V35, pD332, DOI 10.1093/nar/gkl828
Gunnell Gregg F., 2005, Journal of Mammalian Evolution, V12, P209, DOI 10.1007/s10914-005-6945-2
Hayman R. W, 1971, P1
Hoofer SR, 2003, ACTA CHIROPTEROL, V5, P1, DOI 10.3161/001.005.s101
Hutcheon James M., 2004, Journal of Mammalian Evolution, V11, P17, DOI 10.1023/B:JOMM.0000029144.80747.d2
Hutson A, 2001, Microchiropteran bats: global status survey and conservation action plan, volume56
Jacobs DS, 1999, ACTA CHIROPTEROL, V1, P93
Jones G, 2006, TRENDS ECOL EVOL, V21, P149, DOI 10.1016/j.tree.2006.01.001
Jones KE, 2005, EVOLUTION, V59, P2243, DOI 10.1111/j.0014-3820.2005.tb00932.x
Kawai K, 2002, J MOL EVOL, V55, P284, DOI 10.1007/s00239-002-2326-0
Kishino H, 2001, MOL BIOL EVOL, V18, P352, DOI 10.1093/oxfordjournals.molbev.a003811
KISHINO H, 1989, J MOL EVOL, V29, P170, DOI 10.1007/BF02100115
KOOPMAN KF, 1994, HDB ZOOLOGY MAMAMLIA, V8
KRUTZSCH PH, 1990, ACTA ANAT, V139, P109
Maeda K., 1982, Honyurui Kagaku (Mammalian Science) Supplement, V1, P1
MCDONALD JT, 1990, S AFR J WILDL RES, V20, P133
McKenna M., 1997, CLASSIFICATION MAMMA
MCWILLIAM AN, 1988, J ZOOL, V216, P119, DOI 10.1111/j.1469-7998.1988.tb02419.x
MEIN P, 1977, MAMMALIA, V41, P207, DOI 10.1515/mamm.1977.41.2.207
Miller-Butterworth CM, 2005, J MAMMAL, V86, P1121, DOI 10.1644/05-MAMM-A-021R1.1
Miller-Butterworth CM, 2003, NATURE, V424, P187, DOI 10.1038/nature01742
NORBERG UM, 1987, PHILOS T R SOC B, V316, P337
Nowak RM, 1994, WALKERS BATS WORLD
Palmeirim JM, 1995, SYM ZOOL S, P219
Posada D, 1998, BIOINFORMATICS, V14, P817, DOI 10.1093/bioinformatics/14.9.817
Racey Paul A., 2000, P363, DOI 10.1016/B978-012195670-7/50010-2
RAMBAUT A, 1996, AL SEQUENCE ALIGNMEN
Reep Roger L., 2000, P93, DOI 10.1017/CBO9780511541872.004
RICHARDSON EG, 1977, J ZOOL, V183, P353
RICHARZ K, 1993, WORLD BATS FLYING GO
Ronquist F, 2003, BIOINFORMATICS, V19, P1572, DOI 10.1093/bioinformatics/btg180
Ronquist F., 2005, MRBAYES V 3 1 MANUAL
Shimodaira H, 1999, MOL BIOL EVOL, V16, P1114, DOI 10.1093/oxfordjournals.molbev.a026201

Simmons Nancy B., 1998, P3
Simmons NB, 1998, B AM MUS NAT HIST, P1
Simmons NB, 2005, MAMMAL SPECIES WORLD, V1, P312, DOI [DOI 10.1111/J.1365-2656.2010.01742.X, 10.1093/acprof]
Skinner J.D., 1990, The mammals of the southern African subregion, P814
Swofford D.L., 2003, PAUP 4 0 PHYLOGENETI
Teeling EC, 2002, P NATL ACAD SCI USA, V99, P1431, DOI 10.1073/pnas.022477199
Teeling EC, 2005, SCIENCE, V307, P580, DOI 10.1126/science.1105113
Thompson JD, 1997, NUCLEIC ACIDS RES, V25, P4876, DOI 10.1093/nar/25.24.4876
Thorne JL, 1998, MOL BIOL EVOL, V15, P1647, DOI 10.1093/oxfordjournals.molbev.a025892
Tiunov M.P., 1989, P67
Van den Bussche RA, 2004, J MAMMAL, V85, P321, DOI 10.1644/1545-1542(2004)085<0321:PRARCF>2.0.CO;2
van der Mewe M., 1986, Cimbebasia Series A, V8, P107
VANDERMERWE M, 1975, S AFR J SCI, V71, P237
VOLLETH M, 1994, Z ZOOL SYST EVOL, V32, P11
NR 66
TC 150
Z9 172
U1 1
U2 44
PU OXFORD UNIV PRESS
PI OXFORD
PA GREAT CLARENDON ST, OXFORD OX2 6DP, ENGLAND
SN 0737-4038
EI 1537-1719
J9 MOL BIOL EVOL
JI Mol. Biol. Evol.
PD JUL
PY 2007
VL 24
IS 7
BP 1553
EP 1561
DI 10.1093/molbev/msm076
PG 9
WC Biochemistry & Molecular Biology; Evolutionary Biology; Genetics &
Heredity
WE Science Citation Index Expanded (SCI-EXPANDED)
SC Biochemistry & Molecular Biology; Evolutionary Biology; Genetics &
Heredity
GA 188TV
UT WOS:000247943100012
PM 17449895
DA 2024-10-21
ER

PT J
AU Kammerer, R
Mansfeld, M
Hänske, J
Missbach, S
He, XC
Kollner, B
Mouchantat, S
Zimmermann, W
AF Kammerer, Robert
Mansfeld, Martin
Haenske, Jana
Missbach, Sophie
He, Xiaocui
Kollner, Bernd
Mouchantat, Susan
Zimmermann, Wolfgang
TI Recent expansion and adaptive evolution of the carcinoembryonic antigen
family in bats of the Yangochiroptera subgroup
SO BMC GENOMICS
LA English
DT Article
DE Carcinoembryonic antigen gene family; Pregnancy-specific glycoproteins;
Positive selection; Chiroptera; Immunoglobulin superfamily; Bats
ID RECEPTOR; SUSCEPTIBILITY; IDENTIFICATION; EXPRESSION; RESPONSES;
MOLECULE; CELLS; TOOL; MHV
AB Background: Expansions of gene families are predictive for ongoing genetic adaptation to environmental cues. We describe such an expansion of the carcinoembryonic antigen (CEA) gene family in certain bat families. Members of the CEA family in humans and mice are exploited as cellular receptors by a number of pathogens, possibly due to their function in immunity and reproduction. The CEA family is composed of CEA-related cell adhesion molecules (CEACAMs) and secreted pregnancy-specific glycoproteins (PSGs). PSGs are almost exclusively expressed by trophoblast cells at the maternal-fetal interface. The reason why PSGs exist only in a minority of mammals is still unknown.

Results: Analysis of the CEA gene family in bats revealed that in certain bat families, belonging to the subgroup Yangochiroptera but not the Yinpterochiroptera subgroup an expansion of the CEA gene family took place, resulting in approximately one hundred CEA family genes in some species of the Vespertilionidae. The majority of these genes encode secreted PSG-like proteins (further referred to as PSG). Remarkably, we found strong evidence that the ligand-binding domain (IgV-like domain) of PSG is under diversifying positive selection indicating that bat PSGs may interact with structurally highly variable ligands. Such ligands might represent bacterial or viral pathogen adhesins. We have identified two distinct clusters of PSGs in three Myotis species. The two PSG cluster differ in the amino acids under positive selection. One cluster was only expanded in members of the Vespertilionidae while the other was found to be expanded in addition in members of the Miniopteridae and

Mormoopidae. Thus one round of PSG expansion may have occurred in an ancestry of all three families and a second only in Vespertilionidae. Although maternal ligands of PSGs may exist selective challenges by two distinct pathogens seem to be likely responsible for the expansion of PSGs in Vespertilionidae.
Conclusions: The rapid expansion of PSGs in certain bat species together with selection for diversification suggest that bat PSGs could be part of a pathogen defense system by serving as decoy receptors and/or regulators of feto-maternal interactions.
C1 [Kammerer, Robert; Mansfeld, Martin; Haenske, Jana; Missbach, Sophie; He, Xiaocui; Kollner, Bernd] Friedrich Loeffler Inst, Inst Immunol, Greifswald, Germany.
[Missbach, Sophie] Deutsch Primatenzentrum GmbH, Plattform Degenerat Erkrankungen, Gottingen, Germany.
[He, Xiaocui] Max Planck Inst Immunobiol & Epigenet, Dept Mol Immunol, Freiburg, Germany.
[Mouchantat, Susan] Friedrich Loeffler Inst, Jr Res Grp Wildlife Dis, Greifswald, Germany.
[Mouchantat, Susan] Univ Med Greifswald, Inst Diagnost Radiol & Neuroradiol, Greifswald, Germany.
[Zimmermann, Wolfgang] Ludwig Maximilians Univ Munchen, Univ Clin, LIFE Ctr, Tumor Immunol Lab, Munich, Germany.
[Zimmermann, Wolfgang] Ludwig Maximilians Univ Munchen, Univ Clin, Dept Urol, Munich, Germany.
C3 Friedrich Loeffler Institute; Deutsches Primatenzentrum (DPZ); Max
Planck Society; Friedrich Loeffler Institute; Greifswald Medical School;
University of Munich; University of Munich
RP Kammerer, R (corresponding author), Friedrich Loeffler Inst, Inst Immunol, Greifswald, Germany.
EM Robert.Kammerer@fli.de
RI Kammerer, Robert/AAQ-1338-2021; Zimmermann, Wolfgang/L-9277-2013
OI Kammerer, Robert/0000-0003-3038-8205
FU BMWi [KF2875802UL2]; GIZ [81170269, 13.1432.7-001.00]; DFG [HE 6249/4-1]; FAO
FX This study was supported by the BMWi (KF2875802UL2), GIZ (Contract no.
81170269; Project No. 13.1432.7-001.00) and DFG (HE 6249/4-1) (to R.K.)
and FAO (to S.M.)
CR Agnarsson I, 2011, PLOS CURR-TREE LIFE, DOI 10.1371/currents.RRN1212
Aleksic D, 2016, REPROD CONVERGENT EV
Baker ML, 2013, ZOONOSES PUBLIC HLTH, V60, P104, DOI 10.1111/j.1863-2378.2012.01528.x
Berger CN, 2004, MOL MICROBIOL, V52, P963, DOI 10.1111/j.1365-2958.2004.04033.x
Brandao PE, 2008, BRAZ J INFECT DIS, V12, P466, DOI 10.1590/S1413-86702008000600003
Carter AM, 2008, J EXP ZOOL PART B, V310B, P428, DOI 10.1002/jez.b.21216
Chuong EB, 2010, MOL BIOL EVOL, V27, P1221, DOI 10.1093/molbev/msq034

Conesa A, 2005, BIOINFORMATICS, V21, P3674, DOI 10.1093/bioinformatics/bti610
DVEKSLER GS, 1991, J VIROL, V65, P6881, DOI 10.1128/JVI.65.12.6881-6891.1991
GOPALAKRISHNA A, 1979, J REPROD FERTIL, V56, P417
Grabherr MG, 2011, NAT BIOTECHNOL, V29, P644, DOI 10.1038/nbt.1883
Grosser O., 1909, VERGLEICHENDE ANATOM
Herzig CTA, 2010, BMC EVOL BIOL, V10, DOI 10.1186/1471-2148-10-181
Hill DJ, 2001, MOL MICROBIOL, V39, P850, DOI 10.1046/j.1365-2958.2001.02233.x
Huang YH, 2015, NATURE, V517, P386, DOI 10.1038/nature13848
Javaheri A, 2017, NAT MICROBIOL, V2, DOI 10.1038/nmicrobiol.2016.189
Kammerer R, 2004, GENE, V339, P99, DOI 10.1016/j.gene.2004.06.023
Kammerer R, 2001, J IMMUNOL, V166, P6537, DOI 10.4049/jimmunol.166.11.6537
Kammerer R, 1998, EUR J IMMUNOL, V28, P3664, DOI 10.1002/(SICI)1521-4141(199811)28:11<3664::AID-IMMU3664>3.0.CO;2-D
Kammerer R, 2007, BMC EVOL BIOL, V7, DOI 10.1186/1471-2148-7-196
Kammerer R, 2010, BMC BIOL, V8, DOI 10.1186/1741-7007-8-12
Kawai K, 2003, MOL PHYLOGENET EVOL, V28, P297, DOI 10.1016/S1055-7903(03)00121-0
Königer V, 2017, NAT MICROBIOL, V2, DOI 10.1038/nmicrobiol.2016.188
Lei M, 2016, SCI REP-UK, V6, DOI 10.1038/srep27726
Miller-Butterworth CM, 2007, MOL BIOL EVOL, V24, P1553, DOI 10.1093/molbev/msm076
Moore T, 2014, INT J DEV BIOL, V58, P273, DOI 10.1387/ijdb.130329gd
Muenzner P, 2010, SCIENCE, V329, P1197, DOI 10.1126/science.1190892
Murrell B, 2015, MOL BIOL EVOL, V32, P1365, DOI 10.1093/molbev/msv035
Murrell B, 2012, PLOS GENET, V8, DOI 10.1371/journal.pgen.1002764
Papenfuss AT, 2012, BMC GENOMICS, V13, DOI 10.1186/1471-2164-13-261
Pavlopoulou A, 2014, GENOME BIOL EVOL, V6, P1314, DOI 10.1093/gbe/evu103
Platt RN, 2016, MOBILE DNA-UK, V7, DOI 10.1186/s13100-016-0071-y
Pond SLK, 2006, BIOINFORMATICS, V22, P3096, DOI 10.1093/bioinformatics/btl474
Schountz T, 2014, VIRUSES-BASEL, V6, P4880, DOI 10.3390/v6124880
Singer BB, 2014, PLOS ONE, V9, DOI 10.1371/journal.pone.0094106
Sintsova A, 2015, INFECT IMMUN, V83, P1372, DOI 10.1128/IAI.03123-14
Slevogt H, 2008, NAT IMMUNOL, V9, P1270, DOI 10.1038/ni.1661
Taguchi F, 2012, FRONT MICROBIOL, V3, DOI 10.3389/fmicb.2012.00068
Tchoupa AK, 2014, CELL COMMUN SIGNAL, V12, DOI 10.1186/1478-811X-12-27
Teeling EC, 2005, SCIENCE, V307, P580, DOI 10.1126/science.1105113

   Teeling EC, 2009, TRENDS ECOL EVOL, V24, P351, DOI 10.1016/
j.tree.2009.02.012
   Ye J, 2006, NUCLEIC ACIDS RES, V34, pW293, DOI 10.1093/nar/gkl031
   Zhang GJ, 2013, SCIENCE, V339, P456, DOI 10.1126/science.1230835
   Zimmermann W, 2016, BMC GENOMICS, V17, DOI 10.1186/
s12864-016-3279-9
NR 44
TC 15
Z9 15
U1 0
U2 9
PU BMC
PI LONDON
PA CAMPUS, 4 CRINAN ST, LONDON N1 9XW, ENGLAND
SN 1471-2164
J9 BMC GENOMICS
JI BMC Genomics
PD SEP 11
PY 2017
VL 18
AR 717
DI 10.1186/s12864-017-4106-7
PG 14
WC Biotechnology & Applied Microbiology; Genetics & Heredity
WE Science Citation Index Expanded (SCI-EXPANDED)
SC Biotechnology & Applied Microbiology; Genetics & Heredity
GA FG3MA
UT WOS:000410063800008
PM 28893191
OA Green Published, gold
DA 2024-10-21
ER

PT J
AU Yousefi, S
   Sharifi, M
AF Yousefi, Shetav
   Sharifi, Mozafar
TI Neonates of the Mediterranean horseshoe bat, <i>Rhinolophus
euryale</i>,
   pay no cost in body mass or length-adjusted body mass on account
of the
   load of the ectoparasite <i>Eyndhovenia euryalis</i>
SO EVOLUTIONARY ECOLOGY RESEARCH
LA English
DT Article
DE body-condition index; Eyndhovenia euryalis; nursing colony;
parasite
   load; Rhinolophus eutyale
ID MOUSE-EARED BAT; MITE SPINTURNIX-MYOTI; CHIROPTERA RHINOLOPHIDAE;
   MAMMALIA CHIROPTERA; COMMUNITY STRUCTURE; POSTNATAL-GROWTH;
   IMMUNE-RESPONSE; LIFE-HISTORY; HOST; PARASITE
AB Background: Bats are generally highly gregarious, forming large
colonies known to favour the transmission and reproduction of

ectoparasites. Exposure to parasites is responsible for the natural selection of very diverse behavioural and immune system adaptations that enable the hosts to survive and reproduce. A classic interaction between host and parasite is usually predicted, i.e. selection for more resistant hosts, which in turn leads to selection for more infective parasites. However, among bats and their ectoparasites, there are few examples of the host and parasite driving each other's evolution in this way.
Site of experiment: Kerend Cave, Kermanshah Province, western Iran.
Organisms: The Mediterranean horseshoe hat, Rhinolophus euryale and a mite, Eyndhovenia euryalis.
Question: Does the interaction of host and parasite (horseshoe bat and mite) result in a parasitism-induced cost manifested as reduced host body mass or reduced host body-condition index?
Methods: Simultaneous measurement of the percent prevalence, parasite load, and intensity of E. euryalis, and the body mass and body-condition index of neonates of R. euryale during the postnatal period in Kerend Cave.
Results: The parasite load of R. euryale neonates increases rapidly and peaks during the first three weeks of life, before decreasing steadily to its lowest value at the end of the post-natal period. We found no association between changes in parasite load and the body mass of R. eutyale. This indicates that the interaction between the bat and the mite is regulated so that the bat hosts, during their post-natal growth, resist parasites without paying a cost of parasitism in temis either of reduced body mass or body-condition index.
C1 [Yousefi, Shetav; Sharifi, Mozafar] Razi Univ, Dept Biol, Baghabrisham 6714967346, Kermanshah, Iran.
C3 Razi University
RP Sharifi, M (corresponding author), Razi Univ, Dept Biol, Baghabrisham 6714967346, Kermanshah, Iran.
EM sharifimozafar2012@gmail.com
CR Bartonicka T, 2007, PARASITOL RES, V100, P1323, DOI 10.1007/s00436-006-0414-6
Begon M., 2020, ECOLOGY INDIVIDUALS
Benda Petr, 2012, Acta Societatis Zoologicae Bohemicae, V76, P163
Brown CR, 2004, BEHAV ECOL SOCIOBIOL, V56, P498, DOI 10.1007/s00265-004-0813-6
BULL CM, 1993, OECOLOGIA, V96, P415, DOI 10.1007/BF00317513
Christe P, 2000, ECOL LETT, V3, P207
Christe P, 2007, J ANIM ECOL, V76, P703, DOI 10.1111/j.1365-2656.2007.01255.x
CLAYTON DH, 1994, P ROY SOC B-BIOL SCI, V256, P211, DOI 10.1098/rspb.1994.0072
DeBlase A. F, 1980, BATS IRAN SYSTEMATIC
Deerenberg C, 1997, P ROY SOC B-BIOL SCI, V264, P1021, DOI 10.1098/rspb.1997.0141
Eckstein RA, 2000, APPL ANIM BEHAV SCI, V68, P141, DOI 10.1016/S0168-1591(00)00095-2
Eghbali H, 2018, MAMMALIA, V82, P276, DOI 10.1515/mammalia-2017-0006
Fenner AL, 2008, ECOL RES, V23, P897, DOI 10.1007/

s11284-007-0453-1
Fitze PS, 2004, J ANIM ECOL, V73, P216, DOI 10.1111/j.0021-8790.2004.00799.x
Galván I, 2012, J AVIAN BIOL, V43, P273, DOI 10.1111/j.1600-048X.2012.05686.x
HAIG D, 1993, Q REV BIOL, V68, P495, DOI 10.1086/418300
Hassan V, 2010, ASIAN PAC J TROP MED, V3, P371, DOI 10.1016/S1995-7645(10)60090-9
Hawlena H, 2005, OECOLOGIA, V146, P200, DOI 10.1007/s00442-005-0187-0
HOGARTH P. J., 1982, IMMUNOLOGICAL ASPECT
Hutson A, 2001, Microchiropteran bats: global status survey and conservation action plan, volume56
Imaz E., 1999, Miscel.lania Zoologica, V22, P21
JOHNSON LS, 1993, OIKOS, V66, P255, DOI 10.2307/3544812
Khokhlova IS, 2002, J ZOOL, V258, P349, DOI 10.1017/S0952836902001498
Klein SL, 2004, PARASITE IMMUNOL, V26, P247, DOI 10.1111/j.0141-9838.2004.00710.x
Kristofik Jan, 2012, Vespertilio, V16, P167
Lourenco S. D. N., 2008, THESIS
Lourenço SI, 2007, J ZOOL, V273, P161, DOI 10.1111/j.1469-7998.2007.00322.x
Lucan RK, 2006, FOLIA PARASIT, V53, P147, DOI 10.14411/fp.2006.019
Marshall A.G., 1982, P369
Moller AP, 2004, OIKOS, V104, P299, DOI 10.1111/j.0030-1299.2004.12844.x
Moshkin MP, 1998, OIKOS, V82, P131, DOI 10.2307/3546923
Moura MO, 2003, MEM I OSWALDO CRUZ, V98, P811, DOI 10.1590/S0074-02762003000600017
Mouritsen KN, 2002, PARASITOLOGY, V124, pS101, DOI 10.1017/S0031182002001476
Najafi N, 2019, MITOCHONDRIAL DNA A, V30, P135, DOI 10.1080/24701394.2018.1463375
Neuhaus P, 2003, P ROY SOC B-BIOL SCI, V270, pS213, DOI 10.1098/rsbl.2003.0073
Patterson BD, 2008, J TROP ECOL, V24, P387, DOI 10.1017/S0266467408005117
Perez-Orella C, 2005, CAN J ZOOL, V83, P1381, DOI [10.1139/z05-126, 10.1139/Z05-126]
Postawa T, 2014, PARASITOL RES, V113, P1803, DOI 10.1007/s00436-014-3826-8
Reiczigel J., 2005, QUANTITATIVE PARASIT
RUDNICK ALBERT, 1960, UNIV CALIFORNIA PUBL ENT, V17, P157
Sachanowicz K, 2014, J NAT HIST, V48, P2661, DOI 10.1080/00222933.2014.939729
Sharifi M, 2004, ACTA CHIROPTEROL, V6, P155, DOI 10.3161/001.006.0112
Sharifi M., 2000, Myotis, V38, P61
Sharifi M, 2008, J PARASITOL, V94, P622, DOI 10.1645/GE-1334.1
Sharifi M, 2012, ZOOL STUD, V51, P1235
Sharifi M, 2013, ZOOL MIDDLE EAST, V59, P1, DOI 10.1080/09397140.2013.795053

   Sharifi M, 2013, ACTA PARASITOL, V58, P180, DOI 10.2478/s11686-013-0122-1
   ter Hofstede HM, 2005, J ZOOL, V266, P333, DOI 10.1017/S095283690500693X
   Theron A, 1998, PARASITOL RES, V84, P727, DOI 10.1007/s004360050477
   TOFT CA, 1990, TRENDS ECOL EVOL, V5, P326, DOI 10.1016/0169-5347(90)90179-H
   Tompkins DM, 1996, FUNCT ECOL, V10, P733, DOI 10.2307/2390508
   Uchikawa K., 1978, Bulletin of the National Science Museum, A, V4, P245
   Webber QMR, 2015, BEHAVIOUR, V152, P83, DOI 10.1163/1568539X-00003233
   Whiteman NK, 2005, ANIM CONSERV, V8, P175, DOI 10.1017/S1367943005001915
   Yousefi S, 2018, ACTA CHIROPTEROL, V20, P187, DOI 10.3161/15081109ACC2018.20.1.014
   Zahn A, 2004, J ZOOL, V262, P383, DOI 10.1017/S0952836903004722
   Zhang LB, 2010, J MAMMAL, V91, P136, DOI [10.1644/08-MAMM-A-306R2.1, 10.1644/08-MAMM-A-306R2.1.]
   Zuk M, 1996, ECOLOGY, V77, P1037, DOI 10.2307/2265574
NR 58
TC 0
Z9 0
U1 0
U2 1
PU EVOLUTIONARY ECOLOGY LTD
PI TUCSON
PA UNIV ARIZONA, 321 BIOSCIENCES WEST, TUCSON, AZ 85721 USA
SN 1522-0613
EI 1937-3791
J9 EVOL ECOL RES
JI Evol. Ecol. Res.
PD JUL
PY 2019
VL 20
IS 4
BP 437
EP 449
PG 13
WC Ecology; Evolutionary Biology; Genetics & Heredity
WE Science Citation Index Expanded (SCI-EXPANDED)
SC Environmental Sciences & Ecology; Evolutionary Biology; Genetics &
   Heredity
GA PG9IM
UT WOS:000600040100005
DA 2024-10-21
ER

PT J
AU Orlova, MV
   Kazakov, DV
   Orlov, OL

   Mishchenko, VA
   Zhigalin, AV
AF Orlova, Maria V.
   Kazakov, Denis V.
   Orlov, Oleg L.
   Mishchenko, Vladimir A.
   Zhigalin, Alexandr V.
TI The first data on the infestation of the parti-coloured bat,
   <i>Vespertilio murinus</i> (Chiroptera, Vespertilionidae), with
gamasid
   mites, <i>Steatonyssus spinosus</i> (Mesostigmata, Gamasina,
   Macronyssidae)
SO RUSSIAN JOURNAL OF THERIOLOGY
LA English
DT Article
ID REPRODUCTIVE-CYCLE; MYOTIS-DAUBENTONII; SPINTURNIX-MYOTI;
   SEX-DIFFERENCES; CELL-ACTIVITY; HOST; PREGNANCY; PARASITE;
   ECTOPARASITES; ENERGETICS
AB This article presents one of the very few records of a
macronyssid mite (Mesostigmata, Gamasina, Macronyssidae) infestation
of vesper bats (Chiroptera, Vespertilionidae). It is the first
report of the influence of host parameters on the infestation of the
parti-coloured bat, Vespertilio murinus, by the mite Steatonyssus
spinosus. It has been shown that the infestation varies considerably
throughout the host's occupation of summer roosts and the highest
infestation was observed in the post-lactation period. Female bats
are infested significantly more intensively than male bats due to
changes in their immune status during pregnancy and lactation. The
infestation decreases in the period when the breeding colony
disbands due to both roost switching and the intensification of
grooming during this period.
C1 [Orlova, Maria V.] Natl Res Tomsk State Univ, Av Lenina 36, Tomsk
634050, Russia.
   [Orlova, Maria V.] Tyumen State Univ, Volodarskogo Str 6, Tyumen
625003, Russia.
   [Kazakov, Denis V.] Irkutsk State Univ, Karl Marks Str 1, Irkutsk
664003, Russia.
   [Orlov, Oleg L.] Ural State Pedag Univ, Av Kosmonavtov 26,
Ekaterinburg 620017, Russia.
   [Mishchenko, Vladimir A.] Ural Fed Univ, Mira Str 19,
Ekaterinburg, Russia.
   [Zhigalin, Alexandr V.] Natl Res Tomsk State Univ, Av Lenina 36,
Tomsk 634050, Russia.
C3 Tomsk State University; Tyumen State University; Irkutsk State
   University; Ural State Pedagogical University; Ural Federal
University;
   Tomsk State University
RP Orlova, MV (corresponding author), Natl Res Tomsk State Univ, Av
Lenina 36, Tomsk 634050, Russia.; Orlova, MV (corresponding author),
Tyumen State Univ, Volodarskogo Str 6, Tyumen 625003, Russia.
EM masha_orlova@mail.ru; kazakov.denis.95@mail.ru;
o_l_orlov@mail.ru;
   innamoramento23@yandex.ru; alex-zhigalin@mail.ru
RI Kazakov, Denis/ABD-3918-2021; Mishchenko, Vladimir/ABG-3129-2020;

Zhigalin, Alexander/AAB-3260-2019; Orlova, Maria/E-9927-2014
OI Zhigalin, Alexander/0000-0003-4661-0560; Misenko,
Vladimir/0000-0003-4280-283X; Orlova, Maria/0000-0001-7811-4364;
Kazakov, Denis/0000-0001-7408-4698
FU project 'Biotic components of ecosystems, their properties, resource
potential, and dynamics under the conditions of transformation of the
natural environment of Western Siberia' [6.567.2014/k]; project for
raising the competitiveness of the leading Russian universities among
the world's research and education centres [5100]
FX We thank the heads of the State Nature Biosphere Reserve Daursky for
their valuable contributions during the fieldwork. This study was
supported by the project 'Biotic components of ecosystems, their
properties, resource potential, and dynamics under the conditions of
transformation of the natural environment of Western Siberia' (state
contract no. 6.567.2014/k) and the project for raising the
competitiveness of the leading Russian universities among the world's
research and education centres (Project 5100).
CR [Anonymous], ANIMAL PLANT PARASIT
[Anonymous], 2014, Randomization and bootstrap: statistical analysis in biology and ecology using R
[Anonymous], 2009, PARASITISM TICKS INS
[Anonymous], STAT ANAL DATA VISUA
[Anonymous], THESIS
[Anonymous], 1973, PARASITIC GAMASID MI
[Anonymous], BIOL J ARMENIA
[Anonymous], IMMUNOLOGY HOST ECTO
[Anonymous], 1997, RUDOLSTADTER NATURHI
[Anonymous], MAMMALS BATS
[Anonymous], 2013, ENTOMOL REV, DOI DOI 10.1134/S0013873813090169
BALEY JE, 1985, J IMMUNOL, V134, P3042
Benda Petr, 2012, Acta Societatis Zoologicae Bohemicae, V76, P163
Benda Petr, 1998, Acta Societatis Zoologicae Bohemicae, V62, P255
BOOTH DT, 1993, P ROY SOC B-BIOL SCI, V253, P125, DOI 10.1098/rspb.1993.0091
Christe P, 2003, J ANIM ECOL, V72, P866, DOI 10.1046/j.1365-2656.2003.00759.x
Christe P, 2000, ECOL LETT, V3, P207
Christe P, 2007, J ANIM ECOL, V76, P703, DOI 10.1111/j.1365-2656.2007.01255.x
Clayton D.H., 1991, Oxford Ornithology Series, P258
Dick CW, 2006, MICROMAMMALS AND MACROPARASITES: FROM EVOLUTIONARY ECOLOGY TO MANAGEMENT, P179, DOI 10.1007/978-4-431-36025-4_11
DUSBABEK F, 1972, Folia Parasitologica (Ceske Budejovice), V19, P139
Encarnaçao JA, 2012, ACTA CHIROPTEROL, V14, P153, DOI 10.3161/150811012X654367

Faraway J. J., 2016, C&H TEXT STAT SCI, V2nd
FURUKAWA K, 1984, J REPROD IMMUNOL, V6, P353, DOI 10.1016/0165-0378(84)90045-7
Giorgi MS, 2001, P ROY SOC B-BIOL SCI, V268, P2071, DOI 10.1098/rspb.2001.1686
GROSSMAN CJ, 1985, SCIENCE, V227, P257, DOI 10.1126/science.3871252
Haitlinger R., 1978, Wiadomosci Parazytologiczne, V24, P475
Hart Benjamin L., 1997, P59
Hosken DJ, 2001, ETHOL ECOL EVOL, V13, P173
Klein SL, 2004, PARASITE IMMUNOL, V26, P247, DOI 10.1111/j.0141-9838.2004.00710.x
Klein SL, 2000, NEUROSCI BIOBEHAV R, V24, P627, DOI 10.1016/S0149-7634(00)00027-0
KURTA A, 1989, PHYSIOL ZOOL, V62, P804, DOI 10.1086/physzool.62.3.30157928
KURTA A, 1990, J MAMMAL, V71, P59, DOI 10.2307/1381316
Lourenço S, 2008, PARASITOL RES, V104, P127, DOI 10.1007/s00436-008-1170-6
Lucan RK, 2006, FOLIA PARASIT, V53, P147, DOI 10.14411/fp.2006.019
Marshall A.G., 1981, ECOLOGY ECTOPARASITI
McKay LI, 1999, ENDOCR REV, V20, P435, DOI 10.1210/er.20.4.435
McLean JA, 1997, ANIM BEHAV, V54, P1193, DOI 10.1006/anbe.1997.0498
Micherdzinski W, 1980, TAXONOMISCHE ANAL FA
Miller L, 1996, LIFE SCI, V59, P1, DOI 10.1016/0024-3205(96)00122-1
MOLLER AP, 1994, OECOLOGIA, V98, P263, DOI 10.1007/BF00324213
MOORING MS, 1995, BEHAV ECOL, V6, P94, DOI 10.1093/beheco/6.1.94
Morales-Montor J, 2004, MICROBES INFECT, V6, P475, DOI 10.1016/j.micinf.2004.02.001
Orlova M. V., 2015, Vestnik Zoologii, V49, P333, DOI 10.1515/vzoo-2015-0036
Orlova MV, 2012, RUSS J ECOL+, V43, P328, DOI 10.1134/S1067413612040108
Orlova M.V., 2016, Gamasid mites (Mesostigmata: Gamasina) parasitizing bats (Chiroptera: Rhinolophidae, Vespertilionidae, Molossidae) of Palaearctic boreal zone (Russia and adjacent countries)
Orlova Maria, 2015, Zoology and Ecology, V25, P314, DOI 10.1080/21658005.2015.1082718
R Development Core Team, 2011, R: A language and envi- ronment for statistical computing
Racey Paul A., 2009, P249
Reckardt K, 2009, OIKOS, V118, P183, DOI 10.1111/j.1600-0706.2008.16950.x
Roulin A, 2003, J ANIM ECOL, V72, P75, DOI 10.1046/j.1365-2656.2003.00677.x
Rupp Doris, 2004, Spixiana, V27, P185
RYBIN SN, 1983, PARAZITOLOGIYA+, V17, P355
Rydell Jens, 1994, Mammalian Species, V467, P1
Savita, 1998, COMP BIOCHEM PHYS C, V119, P199, DOI 10.1016/S0742-8413(97)00207-7

   Sharifi M, 2008, J PARASITOL, V94, P622, DOI 10.1645/GE-1334.1
   STANJUKOVICH MK, 1990, PARAZITOLOGIYA+, V24, P193
   Sundari AA, 2012, J PARASITOL, V98, P60, DOI 10.1645/GE-2833.1
   Teng K.-f., 1980, Acta Zootaxonomica Sinica, V5, P59
   Till W. M., 1964, Bulletin of the British Museum (Natural History) Zoology, V11, P511
   TODER V, 1984, J CLIN LAB IMMUNOL, V14, P129
   Whitaker J.O. Jr, 1988, P459
   Zahn A, 2004, J ZOOL, V262, P383, DOI 10.1017/S0952836903004722
   Zhang LB, 2010, J MAMMAL, V91, P136, DOI [10.1644/08-MAMM-A-306R2.1, 10.1644/08-MAMM-A-306R2.1.]
NR 64
TC 8
Z9 8
U1 1
U2 11
PU KMK SCIENTIFIC PRESS LTD, MOSCOW STATE UNIV
PI MOSCOW
PA ZOOLOGICAL MUSEUM, BOLSHAYA NIKITSKAYA STREET 6, MOSCOW, 00000, RUSSIA
SN 1682-3559
J9 RUSS J THERIOL
JI Russ. J. Theriol.
PD JUN
PY 2017
VL 16
IS 1
BP 66
EP 73
DI 10.15298/rusjtheriol.16.1.06
PG 8
WC Zoology
WE Science Citation Index Expanded (SCI-EXPANDED)
SC Zoology
GA EW4BW
UT WOS:000402448000006
OA gold, Green Published
DA 2024-10-21
ER

PT J
AU Jiang, TL
   Huang, XB
   Wu, H
   Feng, J
AF Jiang, Tinglei
   Huang, Xiaobin
   Wu, Hui
   Feng, Jiang
TI Size and quality information in acoustic signals of <i>Rhinolophus</i>
   <i>ferrumequinum</i> in distress situations
SO PHYSIOLOGY & BEHAVIOR
LA English

DT Article
DE Acoustic communication; Bats; Prey quality; Distress calls; Honest
signal
ID ECHOLOCATION CALL FREQUENCY; IMMUNE-RESPONSE; BODY-SIZE; BAT; BEHAVIOR;
VESPERTILIONIDAE; CHIROPTERA; PREDATORS; SELECTION; BIRD
AB Many animals produce alarm or distress calls when they encounter predators. Previous studies have shown that the distress calls of some birds can also signal the quality of the bird as prey to predators. In this case, both predator and prey may benefit from sharing information about prey's ability to escape. However, little is known about whether echolocation pulses and distress calls in bats convey size and quality information in distress situations. This study investigates the relationship between echolocation, distress calls, and the health of the callers to determine whether these signals are reliable indicators of sender's attributes and quality. The spectro-temporal structure of echolocation pulses and distress calls from captured greater horseshoe bats, Rhinolophus ferrumequinum, were found to be correlated to their body size, body condition, and T-cell-mediated immunocompetence. The peak frequency of echolocation pulses was found to be positively correlated with the bats' forearm length. However, regression analysis has shown that no significant relationship exists between distress calls and overall body size, or between distress calls and overall health. These results suggest that the peak frequency of echolocation pulses may be a reliable index signal to attract conspecifics, but distress calls of bats may not convey information about their size or overall quality as conspecifics or prey. These results indicate that distress calls in bats may only convey their emotional state, to attract conspecifics and facilitate estimation of predation risk.
(C) 2017 Elsevier Inc. All rights reserved.
C1 [Jiang, Tinglei; Huang, Xiaobin; Wu, Hui; Feng, Jiang] Northeast Normal Univ, Jilin Key Lab Anim Resource Conservat & Utilizat, Jingyue St 2555, Changchun 130024, Peoples R China.
[Wu, Hui] Jilin Agr Univ, Coll Anim Sci & Technol, Xincheng ST 2888, Changchun 130118, Peoples R China.
C3 Northeast Normal University - China; Jilin Agricultural University
RP Feng, J (corresponding author), Northeast Normal Univ, Jilin Key Lab Anim Resource Conservat & Utilizat, Jingyue St 2555, Changchun 130024, Peoples R China.
EM fengj@nenu.edu.cn
RI Jiang, Tinglei/GQQ-9485-2022
FU National Natural Science Foundation of China [31470457, 31670390,
31100280]; Specialized Research Fund for the Doctoral Program of Higher
Education [20120043130002]; Fundamental Research Funds for the Central
Universities [2412016KJ045]; Jilin Provincial Key Laboratory of Animal
Resource Conservation and Utilization [130028600]; Northeast Normal
University

FX We would like to thank Diansheng Tang and his family for their help with
the field work. We would like to thank Accdon for its linguistic
assistance during the preparation of this manuscript. This research was
supported by the National Natural Science Foundation of China (Grant No.
31470457, 31670390, 31100280), Specialized Research Fund for the
Doctoral Program of Higher Education (No. 20120043130002), the
Fundamental Research Funds for the Central Universities (No.
2412016KJ045), and the open project program of Jilin Provincial Key
Laboratory of Animal Resource Conservation and Utilization (Grant No.
130028600), Northeast Normal University.
CR Allen LC, 2009, J COMP PHYSIOL B, V179, P315, DOI 10.1007/s00360-008-0315-3
Ardia DR, 2005, ECOLOGY, V86, P2040, DOI 10.1890/04-1619
AUGUST PV, 1985, SOUTHWEST NAT, V30, P371, DOI 10.2307/3671269
BARCLAY RMR, 1991, AM NAT, V137, P693, DOI 10.1086/285188
Bergstrom CT, 2001, ANIM BEHAV, V61, P535, DOI 10.1006/anbe.2000.1636
Bradbury J. W., 2011, PRINCIPLES ANIMAL CO
Carter G, 2015, PLOS ONE, V10, DOI 10.1371/journal.pone.0136146
Chen SF, 2009, P ROY SOC B-BIOL SCI, V276, P3901, DOI 10.1098/rspb.2009.1185
Christe P, 2000, ECOL LETT, V3, P207
DAWKINS R, 1979, PROC R SOC SER B-BIO, V205, P489, DOI 10.1098/rspb.1979.0081
de Bellocq JG, 2006, COMP BIOCHEM PHYS A, V145, P554, DOI 10.1016/j.cbpa.2006.08.045
Ding T, 2013, PLOS ONE, V8, DOI 10.1371/journal.pone.0066751
FENTON MB, 1976, CAN J ZOOL, V54, P1443, DOI 10.1139/z76-167
Fenton MB., 2016, Bat Bioacoustics
Furlow FB, 1997, EVOL HUM BEHAV, V18, P175, DOI 10.1016/S1090-5138(97)00006-8
Gervasi SS, 2008, FUNCT ECOL, V22, P100, DOI 10.1111/j.1365-2435.2007.01340.x
Goedert D, 2014, ANIM BEHAV, V91, P161, DOI 10.1016/j.anbehav.2014.03.012
GRAFEN A, 1990, J THEOR BIOL, V144, P517, DOI 10.1016/S0022-5193(05)80088-8
Griffin DR, 1958, LISTENING DARK
Hill GE, 2011, ECOL LETT, V14, P625, DOI 10.1111/j.1461-0248.2011.01622.x
HOGSTEDT G, 1983, AM NAT, V121, P562, DOI 10.1086/284083
Huang XB, 2015, PLOS ONE, V10, DOI 10.1371/journal.pone.0132817
Jiang TL, 2010, J ACOUST SOC AM, V128, P2204, DOI 10.1121/1.3478855
JOHNSTONE RA, 1995, BIOL REV, V70, P1, DOI 10.1111/j.1469-185X.1995.tb01439.x
Kahn PF, 2007, COPEIA, P614, DOI 10.1643/0045-8511(2007)2007[614:HBSATC]2.0.CO;2
KANWAL JS, 1994, J ACOUST SOC AM, V96, P1229, DOI

10.1121/1.410273
Knoernschild Mirjam, 2012, Chiroptera Neotropical, V18, P1132
Knörnschild M, 2012, P ROY SOC B-BIOL SCI, V279, P4827, DOI 10.1098/rspb.2012.1995
Kunz TH, 2003, BAT ECOLOGY, P3
Laiolo P, 2004, P ROY SOC B-BIOL SCI, V271, pS513, DOI 10.1098/rsbl.2004.0239
Laiolo P, 2007, BEHAV ECOL, V18, P507, DOI 10.1093/beheco/arm008
Lehmer EM, 2007, PHYSIOL BIOCHEM ZOOL, V80, P514, DOI 10.1086/520128
Lima SL, 2013, BIOL REV, V88, P626, DOI 10.1111/brv.12021
Lin HJ, 2015, ZOOLOGY, V118, P192, DOI 10.1016/j.zool.2014.12.005
Lucan RK, 2013, MAMMALIA, V77, P235, DOI 10.1515/mammalia-2012-0067
Luo B, 2013, J COMP PHYSIOL A, V199, P325, DOI 10.1007/s00359-013-0793-y
Mariappan S, 2016, J COMP PHYSIOL A, V202, P55, DOI 10.1007/s00359-015-1053-0
Mariappan S, 2013, J COMP PHYSIOL A, V199, P775, DOI 10.1007/s00359-013-0838-2
Maynard-Smith J., 2003, Animal Signals
MORTON ES, 1977, AM NAT, V111, P855, DOI 10.1086/283219
Pisanski K, 2014, ANIM BEHAV, V95, P89, DOI 10.1016/j.anbehav.2014.06.011
Puechmaille SJ, 2014, PLOS ONE, V9, DOI 10.1371/journal.pone.0103452
R Core Development Team, 2013, R LANG ENV STAT COMP
Racey P.A., 1982, P57
ROHWER S, 1976, AM MIDL NAT, V96, P418, DOI 10.2307/2424080
Russ JM, 2005, ANIM BEHAV, V70, P397, DOI 10.1016/j.anbehav.2004.11.006
Russ JM, 2004, ANIM BEHAV, V67, P1005, DOI 10.1016/j.anbehav.2003.09.003
Russ JM, 1998, ANIM BEHAV, V55, P705, DOI 10.1006/anbe.1997.0665
Ruxton GD, 2011, J EVOLUTION BIOL, V24, P2574, DOI 10.1111/j.1420-9101.2011.02386.x
RYAN JM, 1985, J MAMMAL, V66, P179, DOI 10.2307/1380980
RYDELL J, 1995, BIOL J LINN SOC, V54, P183, DOI 10.1006/bijl.1995.0011
Searcy W. A., 2010, The evolution of animal communication: Reliability and deception in signaling systems
Siemers BM, 2005, ACTA CHIROPTEROL, V7, P259, DOI 10.3161/1733-5329(2005)7[259:ISISAO]2.0.CO;2
Smits JE, 1999, FUNCT ECOL, V13, P567, DOI 10.1046/j.1365-2435.1999.00338.x
Stevens M, 2013, SENSORY ECOLOGY, BEHAVIOUR, AND EVOLUTION, P1, DOI 10.1093/acprof:oso/9780199601776.001.0001
Stoffberg S, 2011, J MAMM EVOL, V18, P117, DOI 10.1007/s10914-011-9158-x
Suzuki TN, 2012, ANIM BEHAV, V84, P53, DOI 10.1016/j.anbehav.2012.03.030
Turmelle AS, 2010, J COMP PHYSIOL B, V180, P1155, DOI 10.1007/s00360-010-0486-6
Vehrencamp L, 2009, ENCY NEUROSCIENCE, P373

   Wu H, 2015, J ZOOL, V297, P211, DOI 10.1111/jzo.12265
   ZAHAVI A, 1975, J THEOR BIOL, V53, P205, DOI
10.1016/0022-5193(75)90111-3
NR 61
TC 15
Z9 15
U1 3
U2 27
PU PERGAMON-ELSEVIER SCIENCE LTD
PI OXFORD
PA THE BOULEVARD, LANGFORD LANE, KIDLINGTON, OXFORD OX5 1GB, ENGLAND
SN 0031-9384
J9 PHYSIOL BEHAV
JI Physiol. Behav.
PD MAY 1
PY 2017
VL 173
BP 252
EP 257
DI 10.1016/j.physbeh.2017.02.025
PG 6
WC Psychology, Biological; Behavioral Sciences
WE Science Citation Index Expanded (SCI-EXPANDED); Social Science
Citation Index (SSCI)
SC Psychology; Behavioral Sciences
GA EP9LW
UT WOS:000397696100032
PM 28238774
DA 2024-10-21
ER

PT J
AU CHAKRABORTY, AK
   CHAKRAVARTY, AK
AF CHAKRABORTY, AK
   CHAKRAVARTY, AK
TI ANTIBODY-MEDIATED IMMUNE-RESPONSE IN THE BAT, PTEROPUS-GIGANTEUS
SO DEVELOPMENTAL AND COMPARATIVE IMMUNOLOGY
LA English
DT Article
AB Among mammals, the Chiroptera (Bats) were perhaps the least
studied with respect to systematic accounts of immune responses. To
resolve this omission in comparative immunology, aspects of immunity
of the large frugivorous bat, P. giganteus were analyzed with
respect to antibody secreting plaque forming cells [PFC] and serum
antibodies as measured by hemagglutination [HA] titers. Homologous
complement was more effective than guinea pig or rabbit complement
in lysing SRBC [sheep red blood cells] for developing plaques.
Kinetic of antibody response to 3 different doses of SRBC was
studied. A peak primary response and its decay were observed after a
single antigenic challenge which were notably delayed. A same dose
of antigen could produce 2-Mercaptoethanol resistant PFC and HA-
titers.
C1 UNIV N BENGAL, CTR LIFE SCI, IMMUNOL & CELL BIOL LAB, SILIGURI

734430, W BENGAL, INDIA.
CR [Anonymous], BIOL BATS

CHAKRABORTY AK, 1983, INDIAN J EXP BIOL, V21, P5
COLBERT EH, 1961, EVOLUTION VERTEBRATE, P261
Constantine D.G., 1970, Biology of bats, P320
CORRISTAN EC, 1956, FED PROC, V15, P584
CUNNINGHAM AJ, 1968, IMMUNOLOGY, V14, P599
DAVIS BD, 1970, PRINCIPLES MICROBIOL, P431
HATTEN BA, 1968, J IMMUNOL, V101, P141
HECK FC, 1965, THESIS TEXAS U TEXAS
HENSHAW RE, 1970, BATS, P188
ITO TAIICHI, 1952, JAPANESE JOUR BACT, V7, P617
JENSEN JA, 1968, FED PROC, V27, P491
Jepsen G. L., 1970, BIOL BATS, V1, P1
KANAKAMBIKA P, 1972, J IMMUNOL, V109, P415
KWAPINSKI JBG, 1972, METHODOLOGY IMMUNOCH, P322
LEONARD LL, 1968, J IMMUNOL, V101, P1168
LYMAN CP, 1970, BIOLOGY BATS, V1, P301
PLOTZ PH, 1968, J IMMUNOL, V100, P744
SETO F, 1980, DEV COMP IMMUNOL, V4, P111, DOI 10.1016/S0145-305X(80)80013-9
SULKIN SE, 1962, PROG MED VIROL, V4, P157
SULKIN SE, 1966, AM J TROP MED HYG, V15, P418, DOI 10.4269/ajtmh.1966.15.418
SULKIN SE, 1970, BATS CHIROPTERAN BIO, P284
WILLIAMS CA, 1976, METHODS IMMUNOLOGY I, P345

NR 23
TC 24
Z9 28
U1 0
U2 1

PU ELSEVIER SCI LTD
PI London
PA 125 London Wall, London, ENGLAND
SN 0145-305X
EI 1879-0089
J9 DEV COMP IMMUNOL
JI Dev. Comp. Immunol.
PY 1984
VL 8
IS 2
BP 415
EP 423
DI 10.1016/0145-305X(84)90048-X
PG 9

WC Fisheries; Immunology; Veterinary Sciences; Zoology
WE Science Citation Index Expanded (SCI-EXPANDED)
SC Fisheries; Immunology; Veterinary Sciences; Zoology
GA SW247
UT WOS:A1984SW24700017
PM 6376191
DA 2024-10-21
ER

PT J
AU Tian, SL
Zeng, JM
Jiao, HW
Zhang, DJ
Zhang, LB
Lei, CQ
Rossiter, SJ
Zhao, HB
AF Tian, Shilin
Zeng, Jiaming
Jiao, Hengwu
Zhang, Dejing
Zhang, Libiao
Lei, Cao-qi
Rossiter, Stephen J.
Zhao, Huabin
TI Comparative analyses of bat genomes identify distinct evolution of
immunity in Old World fruit bats
SO SCIENCE ADVANCES
LA English
DT Article
ID PTEROPID BATS; PHYLOGENETIC ANALYSIS; ADAPTIVE EVOLUTION; READ
ALIGNMENT; NIPAH VIRUS; IDENTIFICATION; ANNOTATION; GENE; INNATE;
TOOL
AB Bats have been identified as natural reservoir hosts of several zoonotic viruses, prompting suggestions that they have unique immunological adaptations. Among bats, Old World fruit bats (Pteropodidae) have been linked to multiple spillovers. To test for lineage-specific molecular adaptations in these bats, we developed a new assem -bly pipeline to generate a reference-quality genome of the fruit bat Cynopterus sphinx and used this in compar-ative analyses of 12 bat species, including six pteropodids. Our results reveal that immunity-related genes have higher evolutionary rates in pteropodids than in other bats. Several lineage-specific genetic changes were shared across pteropodids, including the loss of NLRP1, duplications of PGLYRP1 and C5AR2, and amino acid replacements in MyD88. We introduced MyD88 transgenes containing Pteropodidae-specific residues into bat and human cell lines and found evidence of dampened inflammatory responses. By uncovering distinct immune adaptations, our results could help explain why pteropodids are frequently identified as viral hosts.
C1 [Tian, Shilin; Zeng, Jiaming; Jiao, Hengwu; Lei, Cao-qi; Zhao, Huabin] Wuhan Univ, Coll Life Sci, Frontier Sci Ctr Immunol & Metab, Hubei Key Lab Cell Homeostasis, Wuhan 430072, Peoples R China.
[Zhang, Dejing] Novogene Bioinformat Inst, Beijing 100015, Peoples R China.
[Zhang, Libiao] Guangdong Acad Sci, Inst Zool, Guangzhou 510260, Peoples R China.
[Rossiter, Stephen J.] Queen Mary Univ London, Sch Biol & Chem Sci, London, England.
C3 Wuhan University; Guangdong Academy of Sciences; Institute of Zoology,

Guangdong Academy of Sciences; University of London; Queen Mary University London
RP Zhao, HB (corresponding author), Wuhan Univ, Coll Life Sci, Frontier Sci Ctr Immunol & Metab, Hubei Key Lab Cell Homeostasis, Wuhan 430072, Peoples R China.
EM huabinzhao@whu.edu.cn
RI Zhao, Huabin/AAS-7513-2020; Jiao, Hengwu/JBJ-5213-2023; Tian, Shilin/AAP-9998-2020
OI Jiao, Hengwu/0000-0002-0951-0432; Tian, Shilin/0000-0001-8958-1806
FU National Key Research and Development Program of China [2021YFF0702004];
Fundamental Research Funds for the Central Universities [2042021kf0217];
National Natural Science Foundation of China [31722051]; Natural Science
Foundation of the Hubei Province [2019CFA075]
FX Funding: This study was supported in part by the National Key Research
and Development Program of China (2021YFF0702004) , Fundamental Research
Funds for the Central Universities (2042021kf0217) , National Natural
Science Foundation of China (31722051) , Natural Science Foundation of
the Hubei Province (2019CFA075) to H.Z.
CR Ahn M, 2019, NAT MICROBIOL, V4, P789, DOI 10.1038/s41564-019-0371-3
ALTSCHUL SF, 1990, J MOL BIOL, V215, P403, DOI 10.1016/S0022-2836(05)80360-2
[Anonymous], 2018, NAT BIOTECHNOL, V36, P1121, DOI 10.1038/nbt.4318
Apweiler R, 2004, NUCLEIC ACIDS RES, V32, pD115, DOI [10.1093/nar/gkw1099, 10.1093/nar/gkh131]
Baker ML, 2013, ZOONOSES PUBLIC HLTH, V60, P104, DOI 10.1111/j.1863-2378.2012.01528.x
Banerjee A, 2020, FRONT IMMUNOL, V11, DOI 10.3389/fimmu.2020.00026
Bao WD, 2015, MOBILE DNA-UK, V6, DOI 10.1186/s13100-015-0041-9
Basith S, 2011, PLOS ONE, V6, DOI 10.1371/journal.pone.0023989
Benson G, 1999, NUCLEIC ACIDS RES, V27, P573, DOI 10.1093/nar/27.2.573
Benton MJ, 2007, MOL BIOL EVOL, V24, P26, DOI 10.1093/molbev/msl150
Bergman CM, 2007, BRIEF BIOINFORM, V8, P382, DOI 10.1093/bib/bbm048
Birney E, 2004, GENOME RES, V14, P988, DOI 10.1101/gr.1865504
Boyer JA, 2020, SCIENCE, V370, P450, DOI 10.1126/science.abd0609
Broz P, 2016, NAT REV IMMUNOL, V16, P407, DOI 10.1038/nri.2016.58
Burge C, 1997, J MOL BIOL, V268, P78, DOI 10.1006/jmbi.1997.0951
CAPANNA E, 1970, CARYOLOGIA, V23, P79, DOI 10.1080/00087114.1970.10796365
Chattopadhyay B, 2020, GENOME BIOL EVOL, V12, P259, DOI 10.1093/gbe/evaa030

Chen SF, 2018, BIOINFORMATICS, V34, P884, DOI 10.1093/bioinformatics/bty560
Cheng ZL, 2020, SIGNAL TRANSDUCT TAR, V5, DOI 10.1038/s41392-020-0198-7
Chua KB, 2002, MICROBES INFECT, V4, P145, DOI 10.1016/S1286-4579(01)01522-2
Dai J, 2019, CELL, V176, P1447, DOI 10.1016/j.cell.2019.01.016
De Bie T, 2006, BIOINFORMATICS, V22, P1269, DOI 10.1093/bioinformatics/btl097
Dong D, 2017, MOL BIOL EVOL, V34, P20, DOI 10.1093/molbev/msw231
Eckalbar W. L., NAT GENET
Edgar RC, 2005, BIOINFORMATICS, V21, pI152, DOI 10.1093/bioinformatics/bti1003
Ellinghaus D, 2008, BMC BIOINFORMATICS, V9, DOI 10.1186/1471-2105-9-18
EZEKOWITZ R A B, 1991, Current Biology, V1, P60
Finn RD, 2016, NUCLEIC ACIDS RES, V44, pD279, DOI 10.1093/nar/gkv1344
Gao DX, 2013, SCIENCE, V341, P903, DOI 10.1126/science.1240933
Gerard NP, 2005, J BIOL CHEM, V280, P39677, DOI 10.1074/jbc.C500287200
Grandvaux N, 2002, CURR OPIN INFECT DIS, V15, P259, DOI 10.1097/00001432-200206000-00008
Gu J, 2007, AM J PATHOL, V170, P1136, DOI 10.2353/ajpath.2007.061088
Guigó R, 1998, J COMPUT BIOL, V5, P681, DOI 10.1089/cmb.1998.5.681
Guito JC, 2021, CURR BIOL, V31, P257, DOI 10.1016/j.cub.2020.10.015
Haas BJ, 2003, NUCLEIC ACIDS RES, V31, P5654, DOI 10.1093/nar/gkg770
Haggadone MD, 2016, MEDIAT INFLAMM, V2016, DOI 10.1155/2016/1340156
Halpin K, 2000, J GEN VIROL, V81, P1927, DOI 10.1099/0022-1317-81-8-1927
Halpin K, 2011, AM J TROP MED HYG, V85, P946, DOI 10.4269/ajtmh.2011.10-0567
Han KD, 2005, NUCLEIC ACIDS RES, V33, P4040, DOI 10.1093/nar/gki718
Han YL, 2019, FRONT MICROBIOL, V10, DOI 10.3389/fmicb.2019.01900
Harris RS., 2007, IMPROVED PAIRWISE AL
Hu J, 2020, BIOINFORMATICS, V36, P2253, DOI 10.1093/bioinformatics/btz891
Huddleston J, 2014, GENOME RES, V24, P688, DOI 10.1101/gr.168450.113
Jebb D, 2020, NATURE, V583, P578, DOI 10.1038/s41586-020-2486-3
Jurka J, 2007, ANNU REV GENOM HUM G, V8, P241, DOI 10.1146/annurev.genom.8.080706.092416
Kanehisa M, 2014, NUCLEIC ACIDS RES, V42, pD199, DOI 10.1093/nar/gkt1076
Kim D, 2013, GENOME BIOL, V14, DOI 10.1186/gb-2013-14-4-r36
Kimura T, 2015, J CELL BIOL, V210, P973, DOI 10.1083/jcb.201503023
Korf I, 2004, BMC BIOINFORMATICS, V5, DOI 10.1186/1471-2105-5-59

Kranzusch PJ, 2013, CELL REP, V3, P1362, DOI 10.1016/j.celrep.2013.05.008
Kumar S, 2017, MOL BIOL EVOL, V34, P1812, DOI 10.1093/molbev/msx116
Laing ED, 2018, EMERG INFECT DIS, V24, P122, DOI 10.3201/eid2401.170401
Langmead B, 2012, NAT METHODS, V9, P357, DOI [10.1038/NMETH.1923, 10.1038/nmeth.1923]
Leroy EM, 2005, NATURE, V438, P575, DOI 10.1038/438575a
Li H, 2018, BIOINFORMATICS, V34, P3094, DOI 10.1093/bioinformatics/bty191
Li H, 2009, BIOINFORMATICS, V25, P1754, DOI 10.1093/bioinformatics/btp324
Li L, 2003, GENOME RES, V13, P2178, DOI 10.1101/gr.1224503
Li WD, 2005, SCIENCE, V310, P676, DOI 10.1126/science.1118391
Lin JS, 2017, METHODS MOL BIOL, V1615, P211, DOI 10.1007/978-1-4939-7033-9_17
Lindblad-Toh K, 2011, NATURE, V478, P476, DOI 10.1038/nature10530
Majoros WH, 2004, BIOINFORMATICS, V20, P2878, DOI 10.1093/bioinformatics/bth315
Mantovani A, 2011, NAT REV IMMUNOL, V11, P519, DOI 10.1038/nri3024
Mao XZ, 2005, BIOINFORMATICS, V21, P3787, DOI 10.1093/bioinformatics/bti430
Marcais G, 2011, BIOINFORMATICS, V27, P764, DOI 10.1093/bioinformatics/btr011
Martinon F, 2002, MOL CELL, V10, P417, DOI 10.1016/S1097-2765(02)00599-3
Middleton DJ, 2007, J COMP PATHOL, V136, P266, DOI 10.1016/j.jcpa.2007.03.002
Santillán DDM, 2021, MOL ECOL, V30, P6449, DOI 10.1111/mec.16027
Mount David W, 2007, CSH Protoc, V2007, DOI 10.1101/pdb.top17
Munster VJ, 2016, SCI REP-UK, V6, DOI 10.1038/srep21878
Myers EW, 2005, BIOINFORMATICS, V21, P79, DOI 10.1093/bioinformatics/bti1114
Naghib M, 2022, VIRAL IMMUNOL, V35, P15, DOI 10.1089/vim.2021.0141
Oliveira-Nascimento L, 2012, FRONT IMMUNOL, V3, DOI 10.3389/fimmu.2012.00079
Omatsu T, 2008, VET IMMUNOL IMMUNOP, V124, P169, DOI 10.1016/j.vetimm.2008.03.004
Pavlovich SS, 2018, CELL, V173, P1098, DOI 10.1016/j.cell.2018.03.070
Paweska JT, 2016, VIRUSES-BASEL, V8, DOI 10.3390/v8020029
Posada D, 1998, BIOINFORMATICS, V14, P817, DOI 10.1093/bioinformatics/14.9.817
Price AL, 2005, BIOINFORMATICS, V21, pI351, DOI 10.1093/bioinformatics/bti1018
Probst OC, 2013, BIOCHEM J, V451, P91, DOI 10.1042/BJ20121422
Rhie A, 2020, GENOME BIOL, V21, DOI 10.1186/s13059-020-02134-9
Rice P, 2000, TRENDS GENET, V16, P276, DOI 10.1016/S0168-9525(00)02024-2
Roy R., 2007, COMPREHENSIVE GLYCOS, P821
Samonte RV, 2002, NAT REV GENET, V3, P65, DOI 10.1038/nrg705

Schad J, 2016, IMMUNOGENETICS, V68, P783, DOI 10.1007/s00251-016-0940-z
Schountz T, 2014, VIRUSES-BASEL, V6, P4880, DOI 10.3390/v6124880
Schuh AJ, 2017, SCI REP-UK, V7, DOI 10.1038/s41598-017-07824-2
Scola AM, 2009, MOL IMMUNOL, V46, P1149, DOI 10.1016/j.molimm.2008.11.001
Seim I, 2013, NAT COMMUN, V4, DOI 10.1038/ncomms3212
Sheahan T, 2008, PLOS PATHOG, V4, DOI 10.1371/journal.ppat.1000240
Simao FA, 2015, BIOINFORMATICS, V31, P3210, DOI 10.1093/bioinformatics/btv351
Skirmuntt EC, 2019, VIRUS RES, V270, DOI 10.1016/j.virusres.2019.197645
Stamatakis A, 2014, BIOINFORMATICS, V30, P1312, DOI 10.1093/bioinformatics/btu033
Stanke M, 2003, BIOINFORMATICS, V19, pII215, DOI 10.1093/bioinformatics/btg1080
Steinbiss S, 2009, NUCLEIC ACIDS RES, V37, P7002, DOI 10.1093/nar/gkp759
Swanson WJ, 2003, MOL BIOL EVOL, V20, P18, DOI 10.1093/oxfordjournals.molbev.a004233
Talavera G, 2007, SYST BIOL, V56, P564, DOI 10.1080/10635150701472164
Teeling EC, 2005, SCIENCE, V307, P580, DOI 10.1126/science.1105113
TheGeneOntologyConsortium, 2017, Nucleic Acids Res, V45, pD331, DOI 10.1093/nar/gkw1108
Towner JS, 2007, PLOS ONE, V2, DOI 10.1371/journal.pone.0000764
Trapnell C, 2012, NAT PROTOC, V7, P562, DOI 10.1038/nprot.2012.016
Tsagkogeorga G, 2017, SCI REP-UK, V7, DOI 10.1038/s41598-017-00132-9
Tsu BV, 2021, ELIFE, V10, DOI 10.7554/eLife.60609
Veidenberg A, 2016, MOL BIOL EVOL, V33, P1126, DOI 10.1093/molbev/msv333
Wacharapluesadee S, 2015, VIROL J, V12, DOI 10.1186/s12985-015-0289-1
Wang K, 2020, MOL ECOL, V29, P4366, DOI 10.1111/mec.15542
Wang LF, 2021, NAT REV IMMUNOL, V21, P269, DOI 10.1038/s41577-021-00523-0
Wang YP, 2012, NUCLEIC ACIDS RES, V40, DOI 10.1093/nar/gkr1293
Wang YT, 2017, IMMUNITY, V46, P393, DOI 10.1016/j.immuni.2017.02.011
Wingett Steven, 2015, F1000Res, V4, P1310, DOI 10.12688/f1000research.7334.1
Xie C, 2011, NUCLEIC ACIDS RES, V39, pW316, DOI 10.1093/nar/gkr483
Xie JZ, 2018, CELL HOST MICROBE, V23, P297, DOI 10.1016/j.chom.2018.01.006
Xie W, 2019, P NATL ACAD SCI USA, V116, P11946, DOI 10.1073/pnas.1905013116
Xu Z, 2007, NUCLEIC ACIDS RES, V35, pW265, DOI 10.1093/nar/gkm286
Yang JY, 2015, NAT METHODS, V12, P7, DOI 10.1038/nmeth.3213
Yang ZH, 2007, MOL BIOL EVOL, V24, P1586, DOI 10.1093/molbev/

msm088
   Mendoza MLZ, 2018, NAT ECOL EVOL, V2, P659, DOI 10.1038/
s41559-018-0476-8
   Zhang GJ, 2013, SCIENCE, V339, P456, DOI 10.1126/science.1230835
   Zhang XT, 2019, NAT PLANTS, V5, P833, DOI 10.1038/
s41477-019-0487-8
   Zheng M, 2021, NAT IMMUNOL, V22, P829, DOI 10.1038/
s41590-021-00937-x
   Zhou P., 2020, NATURE, V579, P270, DOI DOI 10.1038/
s41586-020-2012-7
   Zhou P., P NATL ACAD SCI
NR 120
TC 13
Z9 14
U1 3
U2 28
PU AMER ASSOC ADVANCEMENT SCIENCE
PI WASHINGTON
PA 1200 NEW YORK AVE, NW, WASHINGTON, DC 20005 USA
SN 2375-2548
J9 SCI ADV
JI Sci. Adv.
PD MAY 3
PY 2023
VL 9
IS 18
AR eadd0141
DI 10.1126/sciadv.add0141
PG 17
WC Multidisciplinary Sciences
WE Science Citation Index Expanded (SCI-EXPANDED)
SC Science & Technology - Other Topics
GA G3NP9
UT WOS:000988268100005
PM 37146151
OA Green Published, gold
DA 2024-10-21
ER

PT J
AU Seidlova, V
   Zukal, J
   Brichta, J
   Anisimov, N
   Apoznanski, G
   Bandouchova, H
   Bartonicka, T
   Berková, H
   Botvinkin, AD
   Heger, T
   Dundarova, H
   Kokurewicz, T
   Linhart, P
   Orlov, OL

Piacek, V
   Presetnik, P
   Shumkina, AP
   Tiunov, MP
   Treml, F
   Pikula, J
AF Seidlova, Veronika
   Zukal, Jan
   Brichta, Jiri
   Anisimov, Nikolay
   Apoznanski, Grzegorz
   Bandouchova, Hana
   Bartonicka, Tomas
   Berkova, Hana
   Botvinkin, Alexander D.
   Heger, Tomas
   Dundarova, Heliana
   Kokurewicz, Tomasz
   Linhart, Petr
   Orlov, Oleg L.
   Piacek, Vladimir
   Presetnik, Primoz
   Shumkina, Alexandra P.
   Tiunov, Mikhail P.
   Treml, Frantisek
   Pikula, Jiri
TI Active surveillance for antibodies confirms circulation of lyssaviruses
   in Palearctic bats
SO BMC VETERINARY RESEARCH
LA English
DT Article
DE Chiroptera; rabies; blood samples; seroprevalence; Europe; Siberia
ID WHITE-NOSE SYNDROME; PSEUDOGYMNOASCUS-DESTRUCTANS; RABIES VACCINATION;
   IMMUNE-RESPONSES; INFECTION; ASIA; VESPERTILIONIDAE; PERSISTENCE;
   HIBERNATION; VETERINARY
AB Background: Palearctic bats host a diversity of lyssaviruses, though not the classical rabies virus (RABV). As surveillance for bat rabies over the Palearctic area covering Central and Eastern Europe and Siberian regions of Russia has been irregular, we lack data on geographic and seasonal patterns of the infection.
   Results: To address this, we undertook serological testing, using non-lethally sampled blood, on 1027 bats of 25 species in Bulgaria, the Czech Republic, Poland, Russia and Slovenia between 2014 and 2018. The indirect enzyme-linked immunosorbent assay (ELISA) detected rabies virus anti-glycoprotein antibodies in 33 bats, giving an overall seroprevalence of 3.2%. Bat species exceeding the seroconversion threshold included Myotis blythii, Myotis gracilis, Myotis petax, Myotis myotis, Murina hilgendorfi, Rhinolophus ferrumequinum and Vespertilio murinus. While Myotis species (84.8%) and adult females (48.5%) dominated in seropositive bats, juveniles of both sexes showed no difference in seroprevalence. Higher numbers

tested positive when sampled during the active season (10.5%), as compared with the hibernation period (0.9%). Bat rabies seroprevalence was significantly higher in natural habitats (4.0%) compared with synanthropic roosts (1.2%). Importantly, in 2018, we recorded 73.1% seroprevalence in a cave containing a M. blythii maternity colony in the Altai Krai of Russia.

Conclusions: Identification of such "hotspots" of non-RABV lyssavirus circulation not only provides important information for public health protection, it can also guide research activities aimed at more in-depth bat rabies studies.

C1 [Seidlova, Veronika; Brichta, Jiri; Bandouchova, Hana; Heger, Tomas; Linhart, Petr; Piacek, Vladimir; Pikula, Jiri] Univ Vet & Pharmaceut Sci Brno, Dept Ecol & Dis Game Fish & Bees, Palackeho Tr 1946-1, Brno 61242, Czech Republic.

[Zukal, Jan; Berkova, Hana] Czech Acad Sci, Inst Vertebrate Biol, Kvetna 8, Brno 60365, Czech Republic.

[Zukal, Jan; Bartonicka, Tomas] Masaryk Univ, Dept Bot & Zool, Kotlarska 267/2, Brno 61137, Czech Republic.

[Anisimov, Nikolay; Orlov, Oleg L.] Tyumen State Univ, Int Complex Res Lab Study Climate Change, Land Use & Biodivers, Volodarckogo 6, Tyumen 625003, Russia.

[Apoznanski, Grzegorz; Kokurewicz, Tomasz] Wroclaw Univ Environm & Life Sci, Inst Biol, Dept Vertebrate Ecol & Palaeontol, Wroclaw, Poland.

[Botvinkin, Alexander D.] Irkutsk State Med Univ, Krasnogo Vosstania St 1, Irkutsk 664003, Russia.

[Dundarova, Heliana] Inst Biodivers & Ecosyst Res, Dept Ecosyst Res Environm Risk Assessment & Conve, Tsar Osvoboditel 1, Sofia 1000, Bulgaria.

[Orlov, Oleg L.] Ural State Med Univ, Dept Biochem, Repina 3, Ekaterinburg 620014, Russia.

[Presetnik, Primoz] Ctr Cartog Fauna & Flora, Antoliciceva 1, SI-2204 Miklavz Dravskem Polju, Slovenia.

[Shumkina, Alexandra P.] Fed State Budgetary Inst Zapovednoe Pribaikalye, Western Baikal Protected Areas, Baikalskaya St 291B, Irkutsk 664050, Russia.

[Tiunov, Mikhail P.] Russian Acad Sci, Far East Branch, Inst Biol & Soil Sci, Pr T 100-Letiya Vladivostoka 159, Vladivostok 690022, Russia.

[Treml, Frantisek] Univ Vet & Pharmaceut Sci Brno, Dept Infect Dis & Microbiol, Palackeho Tr 1946-1, Brno 61242, Czech Republic.

[Pikula, Jiri] Univ Vet & Pharmaceut Sci Brno, CEITEC Cent European Inst Technol, Brno, Czech Republic.

C3 University of Veterinary Sciences Brno; Czech Academy of Sciences;
Institute of Vertebrate Biology of the Czech Academy of Sciences;
Masaryk University Brno; Tyumen State University; Wroclaw University of
Environmental & Life Sciences; Irkutsk State Medical University;
Bulgarian Academy of Sciences; Ural State Medical University; Russian
Academy of Sciences; Federal Scientific Center of the East Asia
Terrestrial Biodiversity, Far Eastern Branch of the Russian Academy of

Sciences; University of Veterinary Sciences Brno; University of
Veterinary Sciences Brno
RP Seidlova, V (corresponding author), Univ Vet & Pharmaceut Sci
Brno, Dept Ecol & Dis Game Fish & Bees, Palackeho Tr 1946-1, Brno
61242, Czech Republic.
EM seidlovav@vfu.cz
RI Tiunov, Yavada7+1/HHD-3340-2022; Bandouchova, Hana/AAI-1213-2020;
Kovacova, Veronika/V-1815-2019; Anisimov, Nikolay/ABD-5186-2021;
Kokurewicz, Tomasz/C-7729-2017; Brichta, Jiri/Q-8681-2017;
Piaček,
Vladimír/AAI-1231-2020; Apoznański, Grzegorz/HZK-5222-2023;
Berkova,
Hana/G-1108-2014; Dundarova, Heliana/HNI-7801-2023; Alexander,
Botvinkin/AAQ-3750-2021; Bartonička, Tomáš/J-2756-2014; Zukal,
Jan/G-1180-2014; Pikula, Jiri/V-8620-2018
OI Kokurewicz, Tomasz/0000-0002-3906-2124; Apoznanski,
Grzegorz/0000-0002-3433-5402; Dundarova, Heliana/
0000-0002-3136-6457;
Aleksandr, Botvinkin/0000-0002-1324-7374; Bandouchova,
Hana/0000-0003-0373-7940; Zukal, Jan/0000-0003-4967-6880;
Anisimov,
Nikolay/0000-0003-1573-3373; Presetnik, Primoz/
0000-0003-4599-0493;
Pikula, Jiri/0000-0001-8747-9365
FU Czech Science Foundation [1720286S]; Nacional Science Fund,
Ministry of
Education and Sciences, Bulgaria
FX This study was supported by the Czech Science Foundation (Grant
No.
1720286S) and Nacional Science Fund, Ministry of Education and
Sciences,
Bulgaria (Grant No. K.-06-H31/14). The funders had no role in the
study
design, data analysis, and decision to publish, or preparation of
the
manuscript.
CR Amengual B, 2007, PLOS ONE, V2, DOI 10.1371/journal.pone.0000566
Nguyen ATK, 2014, EMERG INFECT DIS, V20, P161, DOI 10.3201/
eid2001.130813
Ceballos NA, 2013, EMERG INFECT DIS, V19, P793, DOI 10.3201/
eid1905.121071
Baker ML, 2013, ZOONOSES PUBLIC HLTH, V60, P104, DOI 10.1111/
j.1863-2378.2012.01528.x
Bandouchova H, 2015, TRANSBOUND EMERG DIS, V62, P1, DOI 10.1111/
tbed.12282
Bandouchova H, 2018, SCI REP-UK, V8, DOI 10.1038/
s41598-018-24461-5
Banyard AC, 2014, VIRUSES-BASEL, V6, P2974, DOI 10.3390/v6082974
Blackwood JC, 2013, P NATL ACAD SCI USA, V110, P20837, DOI
10.1073/pnas.1308817110
Botvinkin AD, 2003, EMERG INFECT DIS, V9, P1623, DOI 10.3201/
eid0912.030374
Bouma HR, 2010, J LEUKOCYTE BIOL, V88, P619, DOI 10.1189/
jlb.0310174

Brookes SM, 2006, DEV BIOLOGICALS, V125, P185
Brookes SM, 2005, VACCINE, V23, P4101, DOI 10.1016/j.vaccine.2005.03.037
Brunet-Rossinni Anja K., 2009, P315
Colombi D, 2019, SCI REP-UK, V9, DOI 10.1038/s41598-018-36485-y
Datzmann T, 2012, ACTA CHIROPTEROL, V14, P243, DOI 10.3161/150811012X661594
Davy CM, 2020, VIRULENCE, V11, P781, DOI 10.1080/21505594.2020.1768018
Flieger M, 2016, SCI REP-UK, V6, DOI 10.1038/srep33200
Fooks AR, 2003, EPIDEMIOL INFECT, V131, P1029, DOI 10.1017/S0950268803001481
Freuling CM, 2011, EMERG INFECT DIS, V17, P1519, DOI 10.3201/eid1708.110201
Hajkova P, 2007, VET REC, V161, P139, DOI 10.1136/vr.161.4.139
Hampson K, 2015, PLOS NEGLECT TROP D, V9, DOI [10.1371/journal.pntd.0003709, 10.1371/journal.pntd.0003786]
He XC, 2014, PLOS ONE, V9, DOI 10.1371/journal.pone.0105373
Heger T, 2020, ACTA VET BRNO, V89, P79, DOI 10.2754/avb202089010079
Johnson N, 2003, VET REC, V152, P383, DOI 10.1136/vr.152.13.383
Johnson N, 2010, VET MICROBIOL, V142, P151, DOI 10.1016/j.vetmic.2010.02.001
Kovacova V, 2018, BMC VET RES, V14, DOI 10.1186/s12917-018-1521-1
Kuzmin IV, 2003, VIRUS RES, V97, P65, DOI 10.1016/S0168-1702(03)00217-X
Leopardi S, 2019, EPIDEMIOL INFECT, V147, DOI [10.1017/s0950268818003072, 10.1017/S0950268818003072]
Malerczyk C, 2014, HUM VACC IMMUNOTHER, V10, P2799, DOI 10.4161/21645515.2014.972741
Malerczyk C, 2009, VACCINE, V27, P5320, DOI 10.1016/j.vaccine.2009.06.095
Martínková N, 2018, VIRULENCE, V9, P1734, DOI 10.1080/21505594.2018.1548685
Matveev VA, 2005, ACTA CHIROPTEROL, V7, P23, DOI 10.3161/1733-5329(2005)7[23:ROMPHA]2.0.CO;2
Nokireki T, 2013, BMC VET RES, V9, DOI 10.1186/1746-6148-9-174
Picard-Meyer E, 2011, VET MICROBIOL, V151, P390, DOI 10.1016/j.vetmic.2011.03.034
Pikula J, 2020, BMC VET RES, V16, DOI 10.1186/s12917-020-02450-z
Pikula J, 2017, PLOS ONE, V12, DOI 10.1371/journal.pone.0180435
Pikula Jiri, 2017, Veterinary Clinics of North America Exotic Animal Practice, V20, P665, DOI 10.1016/j.cvex.2016.11.013
Pikula J, 2012, J WILDLIFE DIS, V48, P207, DOI 10.7589/0090-3558-48.1.207
Reynes JM, 2004, EMERG INFECT DIS, V10, P2231, DOI 10.3201/eid1012.040459
Robardet E, 2019, TROP MED INFECT DIS, V4, DOI 10.3390/tropicalmed4040124
Robardet E, 2017, PLOS NEGLECT TROP D, V11, DOI 10.1371/journal.pntd.0006048
Ruedi M, 2013, MOL PHYLOGENET EVOL, V69, P437, DOI 10.1016/j.ympev.2013.08.011
Schatz J, 2014, VET MICROBIOL, V169, P33, DOI 10.1016/

j.vetmic.2013.12.004
Schatz J, 2013, ZOONOSES PUBLIC HLTH, V60, P22, DOI 10.1111/zph.12002
Serra-Cobo J, 2002, EMERG INFECT DIS, V8, P413, DOI 10.3201/eid0804.010263
Serra-Cobo J, 2013, PLOS ONE, V8, DOI 10.1371/journal.pone.0064467
Servat A, 2007, J IMMUNOL METHODS, V318, P1, DOI 10.1016/j.jim.2006.07.026
Simic I, 2018, BMC VET RES, V14, DOI 10.1186/s12917-018-1592-z
Slate D, 2005, VIRUS RES, V111, P68, DOI 10.1016/j.virusres.2005.03.012
Slate D, 2009, PLOS NEGLECT TROP D, V3, DOI 10.1371/journal.pntd.0000549
Smits GP, 2012, CLIN VACCINE IMMUNOL, V19, P396, DOI 10.1128/CVI.05537-11
Streicker DG, 2016, P NATL ACAD SCI USA, V113, P10926, DOI 10.1073/pnas.1606587113
Van der Poel WHM, 2005, EMERG INFECT DIS, V11, P1854, DOI 10.3201/eid1112.041200
Vos A, 2007, ACTA CHIROPTEROL, V9, P283, DOI 10.3161/1733-5329(2007)9[283:EBLAEE]2.0.CO;2
Wasniewski M, 2016, J VIROL METHODS, V238, P77, DOI 10.1016/j.jviromet.2016.10.006
Wasniewski M, 2014, J VIROL METHODS, V195, P211, DOI 10.1016/j.jviromet.2013.10.021
Zahradníková A, 2018, TRANSBOUND EMERG DIS, V65, P303, DOI 10.1111/tbed.12773
Zukal J, 2016, SCI REP-UK, V6, DOI 10.1038/srep19829
Zukal J, 2014, PLOS ONE, V9, DOI 10.1371/journal.pone.0097224
NR 59
TC 4
Z9 4
U1 0
U2 17
PU BMC
PI LONDON
PA CAMPUS, 4 CRINAN ST, LONDON N1 9XW, ENGLAND
EI 1746-6148
J9 BMC VET RES
JI BMC Vet. Res.
PD DEC 10
PY 2020
VL 16
IS 1
AR 482
DI 10.1186/s12917-020-02702-y
PG 9
WC Veterinary Sciences
WE Science Citation Index Expanded (SCI-EXPANDED)
SC Veterinary Sciences
GA PG8OT
UT WOS:000599988800002
PM 33302915

OA Green Published, gold
DA 2024-10-21
ER

PT J
AU Schoen, A
   Hölzer, M
   Müller, MA
   Wallerang, KB
   Drosten, C
   Marz, M
   Lamp, B
   Weber, F
AF Schoen, Andreas
   Hoelzer, Martin
   Mueller, Marcel A.
   Wallerang, Kai B.
   Drosten, Christian
   Marz, Manja
   Lamp, Benjamin
   Weber, Friedemann
TI Functional comparisons of the virus sensor RIG-I from humans, the
   microbat <i>Myotis daubentonii</i>, and the megabat <i>Rousettus
   aegyptiacus</i>, and their response to SARS-CoV-2 infection
SO JOURNAL OF VIROLOGY
LA English
DT Article
DE bat; Chiroptera; interferon system; retinoic acid-inducible gene-
I;
   RIG-I; Myotis daubentonii; Yangochiroptera; Rousettus
aegyptiacus;
   Yinpterochiroptera; SARS-CoV-2
ID MOLECULAR CHARACTERIZATION; INTERFERON SYSTEM; STRUCTURAL BASIS;
   INFLUENZA-VIRUS; BETA-INTERFERON; NS1 PROTEIN; RNA;
TRANSCRIPTION;
   ACTIVATION; RECOGNITION
AB Bats (order Chiroptera) are a major reservoir for emerging and
re-emerging zoonotic viruses. Their tolerance toward highly
pathogenic human viruses led to the hypothesis that bats may possess
an especially active antiviral interferon (IFN) system. Here, we
cloned and functionally characterized the virus RNA sensor, retinoic
acid-inducible gene-I (RIG-I), from the "microbat" Myotis
daubentonii (suborder Yangochiroptera) and the "megabat" Rousettus
aegyptiacus (suborder Yinpterochiroptera) and compared them to the
human ortholog. Our data show that the overall sequence and domain
organization are highly conserved and that all three RIG-I orthologs
can mediate a similar IFN induction in response to viral RNA at 37
degrees and 39 degrees C but not at 30 degrees C. Like human RIG-I,
bat RIG-Is were optimally activated by double stranded RNA
containing a 5'-triphosphate end and required mitochondrial
antiviral-signaling protein (MAVS) for antiviral signaling.
Moreover, the RIG-I orthologs of humans and of R. aegyptiacus, but
not of M. daubentonii, enable innate immune sensing of SARS-CoV-2
infection. Our results thus show that microbats and megabats express

a RIG-I that is not substantially different from the human counterpart with respect to function, temperature dependency, antiviral signaling, and RNA ligand properties, and that human and megabat RIG-I are able to sense SARS-CoV-2 infection.

IMPORTANCE A common hypothesis holds that bats (order Chiroptera) are outstanding reservoirs for zoonotic viruses because of a special antiviral interferon (IFN) system. However, functional studies about key components of the bat IFN system are rare. RIG-I is a cellular sensor for viral RNA signatures that activates the antiviral signaling chain to induce IFN. We cloned and functionally characterized RIG-I genes from two species of the suborders Yangochiroptera and Yinpterochiroptera. The bat RIG-Is were conserved in their sequence and domain organization, and similar to human RIG-I in (i) mediating virus- and IFN-activated gene expression, (ii) antiviral signaling, (iii) temperature dependence, and (iv) recognition of RNA ligands. Moreover, RIG-I of Rousettus aegyptiacus (suborder Yinpterochiroptera) and of humans were found to recognize SARS-CoV-2 infection. Thus, members of both bat suborders encode RIG-Is that are comparable to their human counterpart. The ability of bats to harbor zoonotic viruses therefore seems due to other features.
C1 [Schoen, Andreas; Wallerang, Kai B.; Lamp, Benjamin; Weber, Friedemann] Justus Liebig Univ, Vet Med FB10, Inst Virol, Giessen, Germany.

[Hoelzer, Martin; Marz, Manja] Friedrich Schiller Univ Jena, RNA Bioinformat & High Throughput Anal, Jena, Germany.

[Hoelzer, Martin; Drosten, Christian; Marz, Manja; Weber, Friedemann] European Virus Bioinformat Ctr, Jena, Germany.

[Mueller, Marcel A.; Drosten, Christian; Weber, Friedemann] German Ctr Infect Res DZIF, Partner Sites Giessen & Charite, Berlin, Germany.

[Drosten, Christian] Charite Univ Med Berlin, Inst Virol, Berlin, Germany.

[Drosten, Christian] Free Univ Berlin, Berlin, Germany.

[Drosten, Christian] Humboldt Univ, Berlin, Germany.

[Drosten, Christian] Berlin Inst Hlth, Berlin, Germany.

C3 Justus Liebig University Giessen; Friedrich Schiller University of Jena;
German Center for Infection Research; Berlin Institute of Health; Berlin
Institute of Health; Free University of Berlin; Humboldt University of
Berlin; Charite Universitatsmedizin Berlin; Free University of Berlin;
Humboldt University of Berlin; Berlin Institute of Health; Berlin
Institute of Health

RP Weber, F (corresponding author), Justus Liebig Univ, Vet Med FB10, Inst Virol, Giessen, Germany.; Weber, F (corresponding author), European Virus Bioinformat Ctr, Jena, Germany.; Weber, F (corresponding author), German Ctr Infect Res DZIF, Partner Sites Giessen & Charite, Berlin, Germany.

EM friedemann.weber@vetmed.uni-giessen.de

RI Lamp, Benjamin/A-8053-2015; Hölzer, Martin/J-4725-2019; Drosten, Christian/AFF-8529-2022; Müller, Marcel/O-2425-2019; Weber,

Friedemann/AAB-3425-2019
OI Schoen, Andreas/0000-0002-1711-4800; Weber,
Friedemann/0000-0001-9737-337X; Mueller, Marcel/
0000-0003-2242-5117
FU Deutsche Forschungsgemeinschaft (DFG) [197785619, 2616/7-2, DR
772/10-2]; Bundesministerium fur Bildung und Forschung (BMBF)
[01KI1723E, 01KI20158, 01KI2006A]; Volkswagen Foundation
(VolkswagenStiftung) [AZ93345]
FX Deutsche Forschungsgemeinschaft (DFG) 197785619 Friedemann Weber
Deutsche Forschungsgemeinschaft (DFG) We 2616/7-2 Friedemann
Weber
Bundesministerium fur Bildung und Forschung (BMBF) 01KI1723E
Friedemann
Weber Bundesministerium fur Bildung und Forschung (BMBF)
01KI20158
Friedemann Weber Bundesministerium fur Bildung und Forschung
(BMBF)
01KI2006A Christian Drosten Deutsche Forschungsgemeinschaft (DFG)
DR
772/10-2 Christian Drosten Volkswagen Foundation
(VolkswagenStiftung)
AZ93345 Marcel A. Muller
CR Ahn M, 2019, NAT MICROBIOL, V4, P789, DOI 10.1038/
s41564-019-0371-3
Ahn M, 2016, SCI REP-UK, V6, DOI 10.1038/srep21722
Arai Y, 2023, ISCIENCE, V26, DOI 10.1016/j.isci.2022.105742
Banerjee A, 2021, ISCIENCE, V24, DOI 10.1016/j.isci.2021.102477
Banerjee A, 2020, ISCIENCE, V23, DOI 10.1016/j.isci.2020.100958
Banerjee A, 2017, SCI REP-UK, V7, DOI 10.1038/s41598-017-01513-w
Barral PM, 2009, VIROLOGY, V391, P171, DOI 10.1016/
j.virol.2009.06.045
Bird BH, 2007, J CLIN MICROBIOL, V45, P3506, DOI 10.1128/
JCM.00936-07
Blakqori G, 2007, J VIROL, V81, P4991, DOI 10.1128/JVI.01933-06
Bondet V, 2021, FRONT IMMUNOL, V12, DOI 10.3389/fimmu.2021.735866
Brook CE, 2015, TRENDS MICROBIOL, V23, P172, DOI 10.1016/
j.tim.2014.12.004
Burbank RC, 1934, J PHYSIOL-LONDON, V82, P459, DOI 10.1113/
jphysiol.1934.sp003197
Clayton E, 2020, FRONT CELL INFECT MI, V10, DOI 10.3389/
fcimb.2020.527921
Corman VM, 2020, EUROSURVEILLANCE, V25, P23, DOI
10.2807/1560-7917.ES.2020.25.3.2000045
Cowled C, 2012, DEV COMP IMMUNOL, V36, P657, DOI 10.1016/
j.dci.2011.11.008
Cui XY, 2023, NAT COMMUN, V14, DOI 10.1038/s41467-023-38202-4
De La Cruz-Rivera PC, 2018, J IMMUNOL, V200, P209, DOI 10.4049/
jimmunol.1701214
Fan L, 2010, J VIROL, V84, P1785, DOI 10.1128/JVI.01362-09
Field KA, 2018, MOL ECOL, V27, P3727, DOI 10.1111/mec.14827
Fuchs J, 2017, J VIROL, V91, DOI [10.1128/JVI.00361-17, 10.1128/
jvi.00361-17]
García-López MA, 2001, J CLIN ENDOCR METAB, V86, P5008, DOI
10.1210/jc.86.10.5008

Glennon NB, 2015, J VIROL, V89, P7550, DOI 10.1128/JVI.00302-15
Habjan M, 2008, PLOS ONE, V3, DOI 10.1371/journal.pone.0002032
Haller O, 2015, TRENDS MICROBIOL, V23, P154, DOI 10.1016/j.tim.2014.12.003
Harazim M, 2023, BMC IMMUNOL, V24, DOI 10.1186/s12865-023-00542-7
Hawkins JA, 2019, P NATL ACAD SCI USA, V116, P11351, DOI 10.1073/pnas.1814995116
Hayward JA, 2022, J VIROL, V96, DOI 10.1128/jvi.01152-22
He XC, 2014, PLOS ONE, V9, DOI 10.1371/journal.pone.0109795
Hess RD, 2012, VACCINE, V30, P2715, DOI 10.1016/j.vaccine.2012.02.015
Hölzer M, 2019, ISCIENCE, V19, P647, DOI 10.1016/j.isci.2019.08.016
Hölzer M, 2019, GIGASCIENCE, V8, DOI 10.1093/gigascience/giz039
Hölzer M, 2016, SCI REP-UK, V6, DOI 10.1038/srep34589
Hu B, 2017, PLOS PATHOG, V13, DOI 10.1371/journal.ppat.1006698
Irving AT, 2021, NATURE, V589, P363, DOI 10.1038/s41586-020-03128-0
Jacquet S, 2022, SCI ADV, V8, DOI 10.1126/sciadv.add7540
Jureka AS, 2015, STRUCTURE, V23, P2001, DOI 10.1016/j.str.2015.08.007
Kainulainen M, 2014, J VIROL, V88, P3464, DOI 10.1128/JVI.02914-13
Kolakofsky D, 2012, RNA, V18, P2118, DOI 10.1261/rna.035949.112
Kowalinski E, 2011, CELL, V147, P423, DOI 10.1016/j.cell.2011.09.039
Lane WC, 2018, MBIO, V9, DOI 10.1128/mBio.00535-18
Lazear HM, 2019, IMMUNITY, V50, P907, DOI 10.1016/j.immuni.2019.03.025
Lee HC, 2019, EXP MOL MED, V51, DOI 10.1038/s12276-019-0299-y
Lee JH, 2023, MED MICROBIOL IMMUN, V212, P125, DOI 10.1007/s00430-022-00734-9
Leventhal SS, 2021, VIRUSES-BASEL, V13, DOI 10.3390/v13020314
Li JJ, 2015, GENE, V561, P115, DOI 10.1016/j.gene.2015.02.013
Ling Z, 2009, J VIROL, V83, P3734, DOI 10.1128/JVI.02434-08
Liu GQ, 2022, CURR OPIN IMMUNOL, V78, DOI 10.1016/j.coi.2022.102252
Liu GQ, 2020, IMMUNITY, V53, P26, DOI 10.1016/j.immuni.2020.03.017
Liu YZ, 2021, MBIO, V12, DOI 10.1128/mBio.02335-21
Livak KJ, 2001, METHODS, V25, P402, DOI 10.1006/meth.2001.1262
Luis AD, 2013, P ROY SOC B-BIOL SCI, V280, DOI 10.1098/rspb.2012.2753
Luo DH, 2011, CELL, V147, P409, DOI 10.1016/j.cell.2011.09.023
Matrosovich M, 2006, VIROL J, V3, DOI 10.1186/1743-422X-3-63
Mibayashi M, 2007, J VIROL, V81, P514, DOI 10.1128/JVI.01265-06
Mostajo NF, 2020, NAR GENOM BIOINFORM, V2, DOI 10.1093/nargab/lqz006
Notredame C, 2000, J MOL BIOL, V302, P205, DOI 10.1006/jmbi.2000.4042
Olival KJ., 2015, Bats and viruses: a new frontier of emerging infectious diseases, P281, DOI DOI 10.1002/9781118818824.CH11
Onomoto K, 2021, CELL MOL IMMUNOL, V18, P539, DOI 10.1038/s41423-020-00602-7

Papies J, 2022, PLOS ONE, V17, DOI 10.1371/journal.pone.0264450
Parisien JP, 2002, J VIROL, V76, P6435, DOI 10.1128/JVI.76.13.6435-6441.2002
Paulson M, 2002, NAT CELL BIOL, V4, P140, DOI 10.1038/ncb747
Pavlovich SS, 2020, FRONT IMMUNOL, V11, DOI 10.3389/fimmu.2020.00435
Pavlovich SS, 2018, CELL, V173, P1098, DOI 10.1016/j.cell.2018.03.070
Prescott J, 2019, MSPHERE, V4, DOI 10.1128/mSphere.00728-19
Rebendenne A, 2021, J VIROL, V95, DOI 10.1128/JVI.02415-20
Reher S, 2021, P ROY SOC B-BIOL SCI, V288, DOI 10.1098/rspb.2020.2059
Rehwinkel J, 2020, NAT REV IMMUNOL, V20, P537, DOI 10.1038/s41577-020-0288-3
Rehwinkel J, 2010, CELL, V140, P397, DOI 10.1016/j.cell.2010.01.020
Rothe C, 2020, NEW ENGL J MED, V382, P970, DOI 10.1056/NEJMc2001468
Sampaio NG, 2021, SCI REP-UK, V11, DOI 10.1038/s41598-021-92940-3
Sánchez-Aparicio MT, 2018, J VIROL, V92, DOI 10.1128/JVI.01960-17
Santiago FW, 2014, J VIROL, V88, P4572, DOI 10.1128/JVI.03021-13
Sarkis S, 2018, DEV COMP IMMUNOL, V81, P1, DOI 10.1016/j.dci.2017.10.023
Savellini GG, 2019, PLOS PATHOG, V15, DOI 10.1371/journal.ppat.1008186
Schmid S, 2010, J BIOL CHEM, V285, P42013, DOI 10.1074/jbc.M110.165936
Schoen A, 2020, J VIROL, V94, DOI 10.1128/JVI.02134-19
Schuberth-Wagner C, 2015, IMMUNITY, V43, P41, DOI 10.1016/j.immuni.2015.06.015
Shaw AE, 2017, PLOS BIOL, V15, DOI 10.1371/journal.pbio.2004086
Stawski C, 2014, J ZOOL, V292, P86, DOI 10.1111/jzo.12105
Tarigan R, 2021, VIRUS GENES, V57, P510, DOI 10.1007/s11262-021-01865-6
Tarigan R, 2020, BIOCHEM BIOPH RES CO, V527, P1, DOI 10.1016/j.bbrc.2020.04.060
Teeling EC, 2018, ANNU REV ANIM BIOSCI, V6, P23, DOI 10.1146/annurev-animal-022516-022811
Thorne LG, 2021, EMBO J, V40, DOI 10.15252/embj.2021107826
Towner JS, 2007, PLOS ONE, V2, DOI 10.1371/journal.pone.0000764
Van Brussel K, 2022, CURR OPIN VIROL, V52, P192, DOI 10.1016/j.coviro.2021.12.008
Waterhouse AM, 2009, BIOINFORMATICS, V25, P1189, DOI 10.1093/bioinformatics/btp033
Weber M, 2015, CELL HOST MICROBE, V17, P309, DOI 10.1016/j.chom.2015.01.005
Weber M, 2014, CYTOKINE GROWTH F R, V25, P621, DOI 10.1016/j.cytogfr.2014.05.004
Weber M, 2013, CELL HOST MICROBE, V13, P336, DOI 10.1016/j.chom.2013.01.012
Wuerth JD, 2016, VIRUSES-BASEL, V8, DOI 10.3390/v8060174
Xie JZ, 2018, CELL HOST MICROBE, V23, P297, DOI 10.1016/j.chom.2018.01.006
Yamada T, 2021, NAT IMMUNOL, V22, P820, DOI 10.1038/

s41590-021-00942-0
   Yin X, 2021, CELL REP, V34, DOI 10.1016/j.celrep.2020.108628
   Yoneyama M, 1998, EMBO J, V17, P1087, DOI 10.1093/emboj/17.4.1087
   Yoneyama M, 2015, CURR OPIN IMMUNOL, V32, P48, DOI 10.1016/
j.coi.2014.12.012
   Yoshikawa Rokusuke, 2023, J Biol Chem, V299, P104819, DOI
10.1016/j.jbc.2023.104819
   Zhang GJ, 2013, SCIENCE, V339, P456, DOI 10.1126/science.1230835
   Zheng Y, 2020, SIGNAL TRANSDUCT TAR, V5, DOI 10.1038/
s41392-020-00438-7
   Zhou H, 2021, CELL, V184, P4380, DOI 10.1016/j.cell.2021.06.008
   Zhou P, 2016, P NATL ACAD SCI USA, V113, P2696, DOI 10.1073/
pnas.1518240113
   Zhou P, 2014, PLOS ONE, V9, DOI 10.1371/journal.pone.0103875
   Zou JZ, 2009, CELL STEM CELL, V5, P97, DOI 10.1016/
j.stem.2009.05.023
NR 102
TC 1
Z9 1
U1 1
U2 2
PU AMER SOC MICROBIOLOGY
PI WASHINGTON
PA 1752 N ST NW, WASHINGTON, DC 20036-2904 USA
SN 0022-538X
EI 1098-5514
J9 J VIROL
JI J. Virol.
PD OCT
PY 2023
VL 97
IS 10
AR e00205-23
DI 10.1128/jvi.00205-23
PG 24
WC Virology
WE Science Citation Index Expanded (SCI-EXPANDED)
SC Virology
GA MB7J1
UT WOS:001191228600001
PM 37728614
OA Green Submitted, hybrid, Green Published
DA 2024-10-21
ER

PT J
AU Vandewege, MW
   Sotero-Caio, CG
   Phillips, CD
AF Vandewege, Michael W.
   Sotero-Caio, Cibele G.
   Phillips, Caleb D.
TI Positive Selection and Gene Expression Analyses from Salivary
Glands

Reveal Discrete Adaptations within the Ecologically Diverse Bat Family
Phyllostomidae
SO GENOME BIOLOGY AND EVOLUTION
LA English
DT Article
DE submandibular glands; selection; molecular evolution; immunity; Chiroptera
ID NONALCOHOLIC STEATOHEPATITIS; PHYLOGENETIC ANALYSIS; SUBMAXILLARY-GLANDS; PROTEIN FAMILIES; EVOLUTION; TRANSCRIPTOME; INSULIN; HOST; MICE; DIVERSIFICATION
AB The leaf-nosed bats (Phyllostomidae) are outliers among chiropterans with respect to the unusually high diversity of dietary strategies within the family. Salivary glands, owing to their functions and high ultrastructural variability among lineages, are proposed to have played an important role during the phyllostomid radiation. To identify genes underlying salivary gland functional diversification, we sequenced submandibular gland transcriptomes from phyllostomid species representative of divergent dietary strategies. From the assembled transcriptomes, we performed an array of selection tests and gene expression analyses to identify signatures of adaptation. Overall, we identified an enrichment of immunity-related gene ontology terms among 53 genes evolving under positive selection. Lineage-specific selection tests revealed several endomembrane system genes under selection in the vampire bat. Many genes that respond to insulin were under selection and differentially expressed genes pointed to modifications of amino acid synthesis pathways in plant-visitors. Results indicate salivary glands have diversified in various ways across a functional diverse clade of mammals in response to niche specializations.
C1 [Vandewege, Michael W.; Sotero-Caio, Cibele G.; Phillips, Caleb D.] Texas Tech Univ, Dept Biol Sci, Lubbock, TX 79409 USA.
[Vandewege, Michael W.] Eastern New Mexico Univ, Dept Biol, Portales, NM 88130 USA.
[Sotero-Caio, Cibele G.] Wellcome Sanger Inst, Wellcome Genome Campus, Cambridge, England.
C3 Texas Tech University System; Texas Tech University; Eastern New Mexico
University; Wellcome Trust Sanger Institute
RP Vandewege, MW (corresponding author), Texas Tech Univ, Dept Biol Sci, Lubbock, TX 79409 USA.; Vandewege, MW (corresponding author), Eastern New Mexico Univ, Dept Biol, Portales, NM 88130 USA.
EM mike.vandewege@gmail.com
OI Sotero-Caio, Cibele/0000-0002-3112-0000
FU Conselho Nacional de Desenvolvimento Cientifico e Tecnologico (CNPq);
Coordenacao de Aperfeicoamento de Pessoal de Nivel Superior (CAPES),
Brazil
FX We would like to thank Robert Baker and Carleton Phillips for previous
work that inspired this research. This work was not possible without the
previous efforts of TTU students, faculty, and staff toward

sample
collection and curation. Therefore, we are thankful for Nicte
Ordonez-Garza, Maria Sagot, Heath Garner, Robert Bradley, and the Texas
Tech University Natural Science Research Laboratory (TTU NSRL) for
tissue loans. We also acknowledge the High Performance Computing Center
(HPCC) at Texas Tech University at Lubbock for providing HPC resources.
URL: http://cmsdev.ttu.edu/hpcc.C.G.S.C.was supported with a fellowship
(PDE) by Conselho Nacional de Desenvolvimento Cientifico e Tecnologico
(CNPq), and by a postdoctoral fellowship (PNPD) from Coordenacao de
Aperfeicoamento de Pessoal de Nivel Superior (CAPES), Brazil during the
development of this study. These funding agencies had no role in any
experimental aspect of this study.
CR Altschul SF, 1997, NUCLEIC ACIDS RES, V25, P3389, DOI 10.1093/nar/25.17.3389
Baker RJ, 2016, ACTA CHIROPTEROL, V18, P1, DOI 10.3161/15081109ACC2016.18.1.001
Bateman A, 2002, NUCLEIC ACIDS RES, V30, P276, DOI [10.1093/nar/gkh121, 10.1093/nar/gkr1065, 10.1093/nar/gkp985]
Bloss J, 2002, J CHEM ECOL, V28, P819, DOI 10.1023/A:1015296928423
Bolger AM, 2014, BIOINFORMATICS, V30, P2114, DOI 10.1093/bioinformatics/btu170
Calisher CH, 2006, CLIN MICROBIOL REV, V19, P531, DOI 10.1128/CMR.00017-06
Cirranello A, 2016, ACTA CHIROPTEROL, V18, P39, DOI 10.3161/15081109ACC2016.18.1.002
Datzmann T, 2010, BMC EVOL BIOL, V10, DOI 10.1186/1471-2148-10-165
Daugherty MD, 2012, ANNU REV GENET, V46, P677, DOI 10.1146/annurev-genet-110711-155522
Dimmer EC, 2012, NUCLEIC ACIDS RES, V40, pD565, DOI 10.1093/nar/gkr1048
Ding KY, 2005, MOL BIOL EVOL, V22, P148, DOI 10.1093/molbev/msh266
Dobrosielski-Vergona K., 1993, BIOL SALIVARY GLANDS
Dumont ER, 2012, P ROY SOC B-BIOL SCI, V279, P1797, DOI 10.1098/rspb.2011.2005
Eddy SR, 2011, PLOS COMPUT BIOL, V7, DOI 10.1371/journal.pcbi.1002195
Fábián TK, 2012, INT J MOL SCI, V13, P4295, DOI 10.3390/ijms13044295
Francischetti IMB, 2013, J PROTEOMICS, V82, P288, DOI 10.1016/j.jprot.2013.01.009
Fujiwara Y, 2007, J PHYSIOL-LONDON, V584, P235, DOI 10.1113/jphysiol.2007.136481

GOLDMAN N, 1994, MOL BIOL EVOL, V11, P725
Grabherr MG, 2011, NAT BIOTECHNOL, V29, P644, DOI 10.1038/nbt.1883
Haas BJ, 2013, NAT PROTOC, V8, P1494, DOI 10.1038/nprot.2013.084
Hawkins JA, 2019, P NATL ACAD SCI USA, V116, P11351, DOI 10.1073/pnas.1814995116
Hayden S, 2014, MOL BIOL EVOL, V31, P917, DOI 10.1093/molbev/msu043
Hedrick BP, 2020, J MAMM EVOL, V27, P563, DOI 10.1007/s10914-019-09472-x
Huang DW, 2009, NAT PROTOC, V4, P44, DOI 10.1038/nprot.2008.211
Huang W, 2011, BMC BIOINFORMATICS, V12, DOI 10.1186/1471-2105-12-1
Ibáñez C, 2001, P NATL ACAD SCI USA, V98, P9700, DOI 10.1073/pnas.171140598
Jiggins FM, 2007, J EVOLUTION BIOL, V20, P965, DOI 10.1111/j.1420-9101.2007.01305.x
Katoh Kazutaka, 2013, Mol Biol Evol, V30, P772, DOI 10.1093/molbev/mst010
Kosiol C, 2008, PLOS GENET, V4, DOI 10.1371/journal.pgen.1000144
Krogh A, 2001, J MOL BIOL, V305, P567, DOI 10.1006/jmbi.2000.4315
Langmead B, 2009, GENOME BIOL, V10, DOI 10.1186/gb-2009-10-3-r25
LASKA M, 1990, Z SAUGETIERKD, V55, P49
Lehto M, 2005, EXP CELL RES, V310, P445, DOI 10.1016/j.yexcr.2005.08.003
Li L, 2003, GENOME RES, V13, P2178, DOI 10.1101/gr.1224503
Liu Y, 2013, AM J PATHOL, V182, P1519, DOI 10.1016/j.ajpath.2013.01.050
Liu YY, 2019, ACTA PHARM SIN B, V9, P745, DOI 10.1016/j.apsb.2019.02.006
LOVE MI, 2014, GENOME BIOL, V15, DOI DOI 10.1186/S13059-014-0550-8
Matthijs G, 1997, NAT GENET, V16, P88, DOI 10.1038/ng0597-88
Mi HY, 2016, NUCLEIC ACIDS RES, V44, pD336, DOI 10.1093/nar/gkv1194
Monteiro LR, 2011, BMC EVOL BIOL, V11, DOI 10.1186/1471-2148-11-137
Ng MM, 2013, MOL BIOL CELL, V24, P796, DOI 10.1091/mbc.E12-07-0525
Olival KJ, 2017, NATURE, V546, P646, DOI 10.1038/nature22975
Pécheur EI, 2002, BIOCHEMISTRY-US, V41, P9813, DOI 10.1021/bi0259195
Petersen TN, 2011, NAT METHODS, V8, P785, DOI 10.1038/nmeth.1701
Phillips CD, 2015, FRONT ECOL EVOL, V3, DOI 10.3389/fevo.2015.00122
Phillips CJ, 2014, PLOS ONE, V9, DOI 10.1371/journal.pone.0083512
Phillips CJ, 1996, EUR J MORPHOL, V34, P155, DOI 10.1076/ejom.34.3.155.13040
Phillips CJ, 1998, EUR J MORPHOL, V36, P19
Platt RN, 2014, MOL BIOL EVOL, V31, P1536, DOI 10.1093/molbev/msu112
Polge C, 2015, FRONT PHYSIOL, V6, DOI 10.3389/fphys.2015.00059
Pritham EJ, 2007, P NATL ACAD SCI USA, V104, P1895, DOI 10.1073/pnas.0609601104

Prokopovic V, 2014, ARCH ORAL BIOL, V59, P302, DOI 10.1016/j.archoralbio.2013.12.005
Protzek AOP, 2010, COMP BIOCHEM PHYS A, V157, P142, DOI 10.1016/j.cbpa.2010.05.016
Ray DA, 2007, MOL BIOL EVOL, V24, P632, DOI 10.1093/molbev/msl192
Revell LJ, 2012, METHODS ECOL EVOL, V3, P217, DOI 10.1111/j.2041-210X.2011.00169.x
Rojas D, 2016, SYST BIOL, V65, P432, DOI 10.1093/sysbio/syw011
Rojas D, 2011, MOL ECOL, V20, P2217, DOI 10.1111/j.1365-294X.2011.05082.x
Rossoni DM, 2017, SCI REP-UK, V7, DOI 10.1038/s41598-017-08989-6
Roux J, 2014, MOL BIOL EVOL, V31, P1661, DOI 10.1093/molbev/msu141
Safi K, 2003, ANIM BEHAV, V65, P363, DOI 10.1006/anbe.2003.2067
Shen YY, 2010, P NATL ACAD SCI USA, V107, P8666, DOI 10.1073/pnas.0912613107
Smith I, 2013, CURR OPIN VIROL, V3, P84, DOI 10.1016/j.coviro.2012.11.006
Sotero-Caio CG, 2015, BMC EVOL BIOL, V15, DOI 10.1186/s12862-015-0494-y
Stamatakis A, 2014, BIOINFORMATICS, V30, P1312, DOI 10.1093/bioinformatics/btu033
Storey JD, 2003, P NATL ACAD SCI USA, V100, P9440, DOI 10.1073/pnas.1530509100
Storey JD, 2021, QVALUE Q VALUE ESTIM
Supek F, 2011, PLOS ONE, V6, DOI 10.1371/journal.pone.0021800
Talley HM, 2001, EVOLUTION, V55, P631, DOI 10.1554/0014-3820(2001)055[0631:FPFMSI]2.0.CO;2
Tandler B, 1998, FRONT ORAL BIOL, V10, P21
Tandler B, 2001, ANAT RECORD, V264, P121, DOI 10.1002/ar.1108
TUTTLE MD, 1981, SCIENCE, V214, P677, DOI 10.1126/science.214.4521.677
van der Lee R, 2017, NUCLEIC ACIDS RES, V45, P10634, DOI 10.1093/nar/gkx704
Vandewege MW, 2013, J MOL EVOL, V76, P324, DOI 10.1007/s00239-013-9561-4
Viljakainen L, 2009, MOL BIOL EVOL, V26, P1791, DOI 10.1093/molbev/msp086
Wang PX, 2017, NAT MED, V23, P439, DOI 10.1038/nm.4290
WING SS, 1994, AM J PHYSIOL, V267, pE39, DOI 10.1152/ajpendo.1994.267.1.E39
Xiao J, 2015, FISH SHELLFISH IMMUN, V44, P60, DOI 10.1016/j.fsi.2015.01.022
Yang ZH, 2002, MOL BIOL EVOL, V19, P908, DOI 10.1093/oxfordjournals.molbev.a004148
Yang ZH, 1998, MOL BIOL EVOL, V15, P568, DOI 10.1093/oxfordjournals.molbev.a025957
Yang ZH, 2007, MOL BIOL EVOL, V24, P1586, DOI 10.1093/molbev/msm088
Yassine HN, 2009, J GERONTOL A-BIOL, V64, P90, DOI 10.1093/gerona/gln032
Zhang GJ, 2013, SCIENCE, V339, P456, DOI 10.1126/science.1230835
NR 82
TC 5

Z9 5
U1 1
U2 6
PU OXFORD UNIV PRESS
PI OXFORD
PA GREAT CLARENDON ST, OXFORD OX2 6DP, ENGLAND
SN 1759-6653
J9 GENOME BIOL EVOL
JI Genome Biol. Evol.
PD AUG
PY 2020
VL 12
IS 8
BP 1419
EP 1428
DI 10.1093/gbe/evaa151
PG 10
WC Evolutionary Biology; Genetics & Heredity
WE Science Citation Index Expanded (SCI-EXPANDED)
SC Evolutionary Biology; Genetics & Heredity
GA OH6JE
UT WOS:000582695200015
PM 32697843
OA gold, Green Published
DA 2024-10-21
ER

PT J
AU Reeves, WK
AF Reeves, WK
TI Bionomics of <i>Cimex adjunctus</i> (Heteroptera: Cimicidae) in a
   maternity cave of <i>Myotis austroriparius</i> (Chiroptera:
   Vespertilionidae) (South Carolina, USA)
SO JOURNAL OF ENTOMOLOGICAL SCIENCE
LA English
DT Article
DE Cimex adjunctus; Myotis austroriparius; bionomics; sex ratio; Cimicidae;
   bats; Vespertilionidae
ID FREE-TAILED BATS; ACARI
AB Cimex adjunctus Barber was collected with pitfall traps and by hand in Santee Caves, South Carolina, USA. Santee Caves contain a maternity colony of Myotis austroriparius (Rhoads), the Southeastern Myotis bat. Cimex adjunctus was found only in the maternity chamber. My collection represents the first record of C. adjunctus in association with M, austroriparius. The sex ratio in the C. adjunctus population collected was 1:1 with 128 males, 128 females, and 755 nymphs. Predators of C. adjunctus in the cave environment included three spiders (Azilia affinis Chamberlin, Eidmannella pallida (Emerton), and Gaucelmus augustinus Keyserling). The pathogenic fungus Aspergillus sp. killed C. adjunctus in the cave and laboratory. The mite Histiostoma sp. was phoretic on C. adjunctus. Cimex adjunctus also fed on human blood and elicited an immune response after initial exposure.

C1 Clemson Univ, Dept Entomol, Clemson, SC 29634 USA.
C3 Clemson University
RP Reeves, WK (corresponding author), Clemson Univ, Dept Entomol, 114 Long Hall, Clemson, SC 29634 USA.
CR [Anonymous], 1966, MONOGRAPH CIMICIDAE
BOWER SM, 1981, CAN J ZOOL, V59, P546, DOI 10.1139/z81-078
CHENG T.C., 1964, BIOL ANIMAL PARASITE
Constantine D.G., 1970, Biology of bats, P320
DURDEN LA, 1992, J MED ENTOMOL, V29, P507, DOI 10.1093/jmedent/29.3.507
ELEY SM, 1987, J GEN VIROL, V68, P195, DOI 10.1099/0022-1317-68-1-195
Harvey M.J., 1999, BATS US
HASE A, 1917, Z ANGEW ENTOMOL, V4, P1
MARSHALL AG, 1981, ECOL ENTOMOL, V6, P155, DOI 10.1111/j.1365-2311.1981.tb00602.x
PATERSON WB, 1984, CAN J ZOOL, V62, P1581, DOI 10.1139/z84-231
PETERSON BV, 1987, RES BRANCH AGR CAN M, V28, P1283
RYCKMAN R E, 1981, Bulletin of the Society of Vector Ecologists, V6, P93
Spears RE, 1999, J MED ENTOMOL, V36, P481, DOI 10.1093/jmedent/36.4.481
NR 13
TC 6
Z9 8
U1 0
U2 8
PU GEORGIA ENTOMOLOGICAL SOC INC
PI TIFTON
PA PO BOX 748 DEPT ENTOMOLOGY COASTAL PLAIN EXPT STATION, TIFTON, GA 31793-0748 USA
SN 0749-8004
J9 J ENTOMOL SCI
JI J. Entomol. Sci.
PD JAN
PY 2001
VL 36
IS 1
BP 74
EP 77
DI 10.18474/0749-8004-36.1.74
PG 4
WC Entomology
WE Science Citation Index Expanded (SCI-EXPANDED)
SC Entomology
GA 413AX
UT WOS:000167588700009
DA 2024-10-21
ER

PT J
AU Uhrin, M
Kanuch, P
Kristofík, J

Paule, L
AF Uhrin, Marcel
Kanuch, Peter
Kristofik, Jan
Paule, Ladislav
TI Phenotypic plasticity in the greater mouse-eared bat in extremely different roost conditions
SO ACTA THERIOLOGICA
LA English
DT Article
DE Chiroptera; ecology; immunity; parasites; population genetics
ID MITE SPINTURNIX-MYOTI; DWELLING BAT; GLEANING BAT; POPULATION; SELECTION; VESPERTILIONIDAE; MITOCHONDRIAL; AVAILABILITY; MICROCLIMATE; TEMPERATURE
AB Bats use various roost types with a wide spectrum of ecological features. The greater mouse-eared bat Myotis myotis (Borkhausen, 1797), creates nurseries in attics and caves in Central Europe. The stable low temperature and high humidity cave microclimate contrasts that of attics, which may alter species adaptations and life strategies. We analysed population characteristics (composition, body condition, parasite load, and immune response) and genetic relatedness of two proximal M. myotis populations. Age, sexual and parasite species composition were similar between the cave and attic sites. However, a significantly higher parasite load and body condition was detected in the post-partum females and juveniles of the cave colony (n = 263 bats from the cave, 231 from the attic), with the cave colony females having a significantly stronger immune response (n = 2 caves and 2 attics, 20 females per site). There was no evidence for genetic divergence between cave and attic populations (n = 3 caves and 3 attics, 24 females per site), indicating that different population characteristics are not genetically based and that M. myotis is an example of a species with rather unique phenotypic plasticity.
C1 [Uhrin, Marcel; Kanuch, Peter] Inst Forest Ecol SAS, Zvolen 96053, Slovakia.
[Uhrin, Marcel] Charles Univ Prague, Fac Sci, Dept Zool, CR-12844 Prague, Czech Republic.
[Kristofik, Jan] Inst Zool SAS, Bratislava 84506, Slovakia.
[Paule, Ladislav] Tech Univ Zvolen, Fac Forestry, Zvolen 96053, Slovakia.
C3 Slovak Academy of Sciences; Charles University Prague; Technical University Zvolen
RP Uhrin, M (corresponding author), Inst Forest Ecol SAS, Sturova 2, Zvolen 96053, Slovakia.
EM kanuch@netopiere.sk
RI Paule, Ladislav/K-5373-2012; Kanuch, Peter/A-5416-2011; Uhrin, Marcel/C-7211-2011
OI Kanuch, Peter/0000-0003-2001-4541; Paule, Ladislav/0000-0002-5097-7472;
Kristofik, Jan/0000-0002-7149-1164
FU Czech Bat Conservation Trust; Slovak Grant Agency VEGA [2/0130/08];
Slovak Research and Development Agency [APVV-18-032105]; Czech

Science
   Foundation [206/09/0888]
FX We would like to acknowledge many colleagues who assisted with the field
   work (in alphabetical order): T. Bartonicka, S. Belanova, P. Benda, P.
   Bryndza, M. Fulin, E. Hapl, D. Horacek, S. Matis, M. Oleksak, M. Sevcik,
   A. Trebulova, J. Uhrinova, R. Uhrinova., M. Varga, Cumberland Bat Group,
   and others. P. Masan helped with mite determination and M. Straka with
   the lab work. We are grateful to P. Benda and two anonymous referees for
   providing valuable and helpful comments to the previous version of the
   manuscript and to S. Phillips and B. Kiehl for the linguistic revision.
   Field work and analysis of the sampled material was funded by grants
   from the Czech Bat Conservation Trust, Slovak Grant Agency VEGA
   (2/0130/08), Slovak Research and Development Agency (APVV-18-032105) and
   from the Czech Science Foundation (206/09/0888). This study was
   conducted under licences from the Ministry of Environment of the Slovak
   Republic (2598/715/03-5.1pil and 7121/2008-2.1pil).
CR [Anonymous], HDB SAUGETIERE EUR 1
   Arlettaz R, 1997, J ANIM ECOL, V66, P897, DOI 10.2307/6005
   Arlettaz R, 2001, OIKOS, V95, P105, DOI 10.1034/j.1600-0706.2001.950112.x
   ARLETTAZ R, 1993, MYOTIS, V29, P7
   Berthier P, 2006, P ROY SOC B-BIOL SCI, V273, P3101, DOI 10.1098/rspb.2006.3680
   Bogdanowicz W, 2009, ACTA CHIROPTEROL, V11, P289, DOI 10.3161/150811009X485530
   BUCS S, 2007, ANN W U TIMISOARA SE, V16, P13
   Burland TM, 1999, P ROY SOC B-BIOL SCI, V266, P975, DOI 10.1098/rspb.1999.0732
   Castella V, 2001, J EVOLUTION BIOL, V14, P708, DOI 10.1046/j.1420-9101.2001.00331.x
   Christe P, 2003, J ANIM ECOL, V72, P866, DOI 10.1046/j.1365-2656.2003.00759.x
   Christe P, 2000, ECOL LETT, V3, P207
   Corander J, 2007, B MATH BIOL, V69, P797, DOI 10.1007/s11538-006-9161-1
   Entwistle AC, 2000, J ZOOL, V252, P11, DOI 10.1111/j.1469-7998.2000.tb00815.x
   Giorgi MS, 2001, P ROY SOC B-BIOL SCI, V268, P2071, DOI 10.1098/rspb.2001.1686
   Godfrey LR, 2007, FOLIA PRIMATOL, V78, P405, DOI 10.1159/000105152
   Heidinger F., 1989, European bat research, P189
   Horacek I., 1985, Acta Universitatis Carolinae-Biologica, V1981,

P161
Horacek I., 1984, Myotis, V2122, P138
HORN J, 2005, NYCTALUS, V10, P108
Hutterer Rainer, 2005, P1
Kalinowski ST, 2007, MOL ECOL, V16, P1099, DOI 10.1111/j.1365-294X.2007.03089.x
Kunz T.H., 1982, P1
Kunz TH, 2003, BAT ECOLOGY, P3
Lausen CL, 2006, J MAMMAL, V87, P362, DOI 10.1644/05-MAMM-A-127R1.1
Law BS, 2007, AUST J ZOOL, V55, P49, DOI 10.1071/ZO06069
Lourenço SI, 2007, J ZOOL, V273, P161, DOI 10.1111/j.1469-7998.2007.00322.x
Lourenço S, 2008, PARASITOL RES, V104, P127, DOI 10.1007/s00436-008-1170-6
Marshall A.G., 1982, P369
Martinez MC, 2006, EDUC POLICY, V20, P299, DOI 10.1177/0895904805285017
Mitchell-Jones A.J., 1999, The Atlas of European Mammals, P1
Palmeirim JM, 1995, SYM ZOOL S, P219
Petri B, 1997, MOL ECOL, V6, P235, DOI 10.1046/j.1365-294X.1997.00176.x
Pitala N, 2007, BIOL LETTERS, V3, P418, DOI 10.1098/rsbl.2007.0135
Racey P.A., 1982, P57
Ransome RD, 1995, PHILOS T ROY SOC B, V350, P153, DOI 10.1098/rstb.1995.0149
Rodrigues L, 2003, MAMMALIA, V67, P321, DOI 10.1515/mamm.2003.67.3.321
Rousset F, 2008, MOL ECOL RESOUR, V8, P103, DOI 10.1111/j.1471-8286.2007.01931.x
Ruczynski I, 2005, J MAMMAL, V86, P921, DOI 10.1644/1545-1542(2005)86[921:RCSBNN]2.0.CO;2
Ruedi M, 2003, MOL ECOL, V12, P1527, DOI 10.1046/j.1365-294X.2003.01828.x
Ruedi M, 2008, MOL ECOL, V17, P1801, DOI 10.1111/j.1365-294X.2008.03702.x
Seckerdieck A, 2005, MAMM BIOL, V70, P201, DOI 10.1016/j.mambio.2004.10.002
Sedgeley JA, 2001, J APPL ECOL, V38, P425, DOI 10.1046/j.1365-2664.2001.00607.x
Shiel Caroline B., 1999, Myotis, V37, P41
Simmons NB, 2005, MAMMAL SPECIES WORLD, V1, P312, DOI [DOI 10.1111/J.1365-2656.2010.01742.X, 10.1093/acprof]
Solick DI, 2006, J MAMMAL, V87, P1020, DOI 10.1644/06-MAMM-A-044R1.1
WALSH PS, 1991, BIOTECHNIQUES, V10, P506, DOI 10.2144/000114018
Winemiller KO, 2005, CAN J FISH AQUAT SCI, V62, P872, DOI 10.1139/F05-040
Wojciechowski MS, 2007, COMP BIOCHEM PHYS A, V147, P828, DOI 10.1016/j.cbpa.2006.06.039
Zahn A, 2006, J ZOOL, V269, P486, DOI 10.1111/j.1469-7998.2006.00081.x
Zahn A, 2004, J ZOOL, V262, P383, DOI 10.1017/S0952836903004722

Zahn A, 1999, J ZOOL, V247, P275, DOI 10.1111/
j.1469-7998.1999.tb00991.x
Zahn A, 1997, J ZOOL, V243, P659, DOI 10.1111/
j.1469-7998.1997.tb01968.x
Zahn A, 2007, ACTA CHIROPTEROL, V9, P115, DOI
10.3161/1733-5329(2007)9[115:CTOTYF]2.0.CO;2
[No title captured], DOI DOI 10.1046/J.1365-294X.2000.00939.X
NR 54
TC 18
Z9 19
U1 1
U2 25
PU SPRINGER HEIDELBERG
PI HEIDELBERG
PA TIERGARTENSTRASSE 17, D-69121 HEIDELBERG, GERMANY
SN 0001-7051
EI 2190-3743
J9 ACTA THERIOL
JI Acta Theriol.
PD APR
PY 2010
VL 55
IS 2
BP 153
EP 164
DI 10.4098/j.at.0001-7051.073.2009
PG 12
WC Zoology
WE Science Citation Index Expanded (SCI-EXPANDED)
SC Zoology
GA 596LW
UT WOS:000277687500006
DA 2024-10-21
ER

PT J
AU Hosken, DJ
O'Shea, JE
AF Hosken, DJ
O'Shea, JE
TI Sperm production and immune function in two Australian bats,
<i>Chalinolobus morio</i> and <i>Nyctophilus geoffroyi</i>
SO ETHOLOGY ECOLOGY & EVOLUTION
LA English
DT Article
DE Chiroptera; immunocompetence; spleen size; testis size
ID SEXUAL SELECTION; SIZE; VESPERTILIONIDAE; IMMUNOCOMPETENCE;
TESTOSTERONE; TEMPERATURE; COMPETITION; CHIROPTERA; PATERNITY;
PARASITES
AB One assumption of the immunocompetence handicap hypothesis is
that androgens increase the expression of secondary sex characters
while concomitantly suppressing immune function. Primary sex
function, such as spermatogenesis, may also benefit from immune
down-regulation since spermatozoa are not recognised as self by the

immune system. Males with greater parasite resistance should therefore be able to experience higher androgens levels with a resultant increase in spermatogenic investment and ejaculate quality, plus greater suppression of the immune system. We investigate this possibility in two species of Australian bat. Testis size was used as a measure of ejaculate investment and quality, and spleen size was used as an indicator of immune function. Significant negative relationships were found between spleen and testis mass in one species and a negative trend was noted in a second. When the overall effect size was calculated, the negative association across both species was statistically significant. Our results are consistent with the immunocompetence handicap hypothesis since males able to withstand greater immune system down-regulation had larger testes.
C1 Univ Zurich, Zool Museum, CH-8057 Zurich, Switzerland.
Univ Western Australia, Dept Zool, Nedlands, WA 6907, Australia.
C3 University of Zurich; University of Western Australia
RP Univ Zurich, Zool Museum, Winterthurerstr 190, CH-8057 Zurich, Switzerland.
EM hosken@zoolmus.unizh.ch
CR Birkhead TR, 1998, SPERM COMPETITION SE
Calder WA, 1984, SIZE FUNCTION LIFE H
Churchill S., 1998, Australian bats
Deerenberg C, 1997, P ROY SOC B-BIOL SCI, V264, P1021, DOI 10.1098/rspb.1997.0141
Demas GE, 1998, J BIOL RHYTHM, V13, P253, DOI 10.1177/074873098129000093
Dziuk PJ, 1996, ANIM REPROD SCI, V43, P65, DOI 10.1016/0378-4320(95)01463-2
Emerson SB, 1996, GEN COMP ENDOCR, V103, P220, DOI 10.1006/gcen.1996.0113
Evans MR, 2000, BEHAV ECOL SOCIOBIOL, V47, P156, DOI 10.1007/s002650050006
FOLSTAD I, 1992, AM NAT, V139, P603, DOI 10.1086/285346
Folstad I, 1997, BEHAV ECOL, V8, P109, DOI 10.1093/beheco/8.1.109
GUSTAFSON AW, 1987, AM J ANAT, V178, P312, DOI 10.1002/aja.1001780403
Hillgarth N, 1997, BEHAV ECOL, V8, P108, DOI 10.1093/beheco/8.1.108
Hosken D. J., 1996, Journal of the Royal Society of Western Australia, V79, P211
Hosken DJ, 1997, P ROY SOC B-BIOL SCI, V264, P385, DOI 10.1098/rspb.1997.0055
Hosken DJ, 1998, J ZOOL, V245, P261, DOI 10.1017/S0952836998007031
Hosken DJ, 1998, J ZOOL, V245, P93, DOI 10.1017/S0952836998005111
Hosken DJ, 1997, J COMP PHYSIOL B, V167, P71, DOI 10.1007/s003600050049
KENAGY GJ, 1986, J MAMMAL, V67, P1, DOI 10.2307/1380997
KITCHENER DJ, 1981, AUST J ZOOL, V29, P305, DOI 10.1071/ZO9810305
Liljedal S, 1999, P ROY SOC B-BIOL SCI, V266, P1893, DOI 10.1098/rspb.1999.0863
Merilä J, 1999, BEHAV ECOL SOCIOBIOL, V45, P115, DOI 10.1007/s002650050545

Moller AP, 1997, P ROY SOC B-BIOL SCI, V264, P561, DOI 10.1098/rspb.1997.0080
MOLLER AP, 1991, P ROY SOC B-BIOL SCI, V246, P237, DOI 10.1098/rspb.1991.0150
Moller AP, 1998, J EVOLUTION BIOL, V11, P703, DOI 10.1007/s000360050114
Moller AP, 1999, Q REV BIOL, V74, P3, DOI 10.1086/392949
MOLLER AP, 1987, FUNCTIONAL ECOLOGY, V3, P91
Rosenthal R, 1991, METAANALYTIC PROCEDU
Saino N, 1997, J ANIM ECOL, V66, P827, DOI 10.2307/5998
Sheldon BC, 1996, TRENDS ECOL EVOL, V11, P317, DOI 10.1016/0169-5347(96)10039-2
Steiniger B., 1999, ADV ANAT EMBRYOL CEL, V151, P1
Stockley P, 1996, BEHAV ECOL, V7, P334, DOI 10.1093/beheco/7.3.334
WEDEKIND C, 1994, AM NAT, V143, P936, DOI 10.1086/285641
Withers P.C., 1992, COMP ANIMAL PHYSL
Zuk M, 1998, P ROY SOC B-BIOL SCI, V265, P1631, DOI 10.1098/rspb.1998.0481
NR 34
TC 16
Z9 17
U1 0
U2 8
PU TAYLOR & FRANCIS LTD
PI ABINGDON
PA 2-4 PARK SQUARE, MILTON PARK, ABINGDON OR14 4RN, OXON, ENGLAND
SN 0394-9370
EI 1828-7131
J9 ETHOL ECOL EVOL
JI Ethol. Ecol. Evol.
PD JUN
PY 2001
VL 13
IS 2
BP 173
EP 180
PG 8
WC Behavioral Sciences; Zoology
WE Science Citation Index Expanded (SCI-EXPANDED)
SC Behavioral Sciences; Zoology
GA 437TP
UT WOS:000169009900006
DA 2024-10-21
ER

PT J
AU Lin, HH
Horie, M
Tomonaga, K
AF Lin, Hsien-Hen
Horie, Masayuki
Tomonaga, Keizo
TI A comprehensive profiling of innate immune responses in

<i>Eptesicus</i>
   bat cells
SO MICROBIOLOGY AND IMMUNOLOGY
LA English
DT Article
DE bat; Eptesicus; high-throughput sequencing; innate immunity; poly (I; C)
ID READ ALIGNMENT; EXPRESSION; GENES; GENERATION; KNOWLEDGE; ALPHA
AB Bats (Order: Chiroptera), including those of the genus Eptesicus, have been reported to serve as reservoirs of several zoonotic viruses. Notably, bats have been reported to lack obvious symptoms of infection with such viruses and are thought to have unique immune system responses. However, the responses of their innate immune system, the first line of immunity, remain largely unclear. Here, we comprehensively analyzed the expression profiles in two Eptesicus bat cell lines to investigate their innate immune responses. The gene expression profiles after polyinosinic:polycytidylic acid [poly (I:C)] induction were similar between the two bat cell lines, but uniquely upregulated differentially expressed genes were also identified. We also revealed that the upregulated genes of Eptesicus bat cells were distinct from those of human epithelial cells in response to induction. Moreover, the basal expression levels of several immune-related genes were higher in bat cells than in human cells. We also identified unannotated novel transcripts that were upregulated after induction and novel microRNAs expressed in bat cells, some of which were upregulated by poly (I:C) treatment. This is the first report to illustrate the innate immune response in Eptesicus bat cells; therefore, this study provides basic and novel insights into bat innate immunity. Our data represent a valuable resource for future studies into bat immunity and the biology of Eptesicus bats.
C1 [Lin, Hsien-Hen; Horie, Masayuki; Tomonaga, Keizo] Kyoto Univ, Inst Frontier Life & Med Sci InFRONT, Dept Virus Res, Lab RNA Viruses, Kyoto, Japan.
   [Lin, Hsien-Hen; Tomonaga, Keizo] Kyoto Univ, Grad Sch Biostudies, Dept Mammalian Regulatory Network, Kyoto, Japan.
   [Horie, Masayuki] Kyoto Univ, Hakubi Ctr Adv Res, Kyoto, Japan.
   [Horie, Masayuki] Osaka Prefecture Univ, Grad Sch Life & Environm Sci, Div Vet Sci, Lab Vet Microbiol, Izumisano, Japan.
   [Tomonaga, Keizo] Kyoto Univ, Grad Sch Med, Dept Mol Virol, Kyoto, Japan.
C3 Kyoto University; Kyoto University; Kyoto University; Osaka Metropolitan
   University; Kyoto University
RP Horie, M; Tomonaga, K (corresponding author), Kyoto Univ, Inst Frontier Life & Med Sci InFRONT, Dept Virus Res, Lab RNA Viruses, Kyoto, Japan.
EM mhorie@vet.osakafu-u.ac.jp; tomonaga@infront.kyoto-u.ac.jp
OI Lin, Hsien-Hen/0000-0003-3018-7753; Horie, Masayuki/0000-0003-4682-7698
FU JSPS KAKENHI [JP20H05682, JP18K19443]; MEXT KAKENHI [JP16H06429,
   JP16K21723, JP16H0643, JP17H05821, JP19H04833]; Hakubi project at Kyoto
   University; Grants-in-Aid for Scientific Research [20H05682]

Funding
Source: KAKEN
FX We are grateful to Dr Takefumi Kondo and Ms. Yukari Sando for performing
the NextSeq sequencing. We thank Dr Keiko Takemoto (Institute for
Frontier Life and Medical Sciences, Kyoto University, Japan) for
technical support. This study was supported by JSPS KAKENHI JP20H05682
(Keizo Tomonaga) and JP18K19443 (Masayuki Horie); MEXT KAKENHI
JP16H06429 (Keizo Tomonaga), JP16K21723 (Keizo Tomonaga), JP16H0643
(Keizo Tomonaga), JP17H05821 (Masayuki Horie) and JP19H04833 (Masayuki
Horie); and the Hakubi project at Kyoto University (Masayuki Horie).
CR Ahn M, 2016, SCI REP-UK, V6, DOI 10.1038/srep21722
Banerjee A, 2020, SCI REP-UK, V10, DOI 10.1038/s41598-020-64264-1
Banerjee A, 2019, VIRUSES-BASEL, V11, DOI 10.3390/v11020152
Banerjee A, 2017, SCI REP-UK, V7, DOI 10.1038/s41598-017-01513-w
Banerjee A, 2016, J VIROL METHODS, V237, P166, DOI 10.1016/j.jviromet.2016.09.008
Becker DJ, 2019, INTEGR COMP BIOL, V59, P1176, DOI 10.1093/icb/icz007
Camacho C, 2009, BMC BIOINFORMATICS, V10, DOI 10.1186/1471-2105-10-421
Caracausi M, 2017, MOL MED REP, V16, P2397, DOI 10.3892/mmr.2017.6944
Chandan K, 2020, FRONT IMMUNOL, V10, DOI 10.3389/fimmu.2019.03081
Chen SF, 2018, BIOINFORMATICS, V34, P884, DOI 10.1093/bioinformatics/bty560
Chuong EB, 2016, SCIENCE, V351, P1083, DOI 10.1126/science.aad5497
Dobin A, 2013, BIOINFORMATICS, V29, P15, DOI 10.1093/bioinformatics/bts635
Fenton M.B., 2015, Bats: a world of science and mystery
Flynn JM, 2020, P NATL ACAD SCI USA, V117, P9451, DOI 10.1073/pnas.1921046117
Friedländer MR, 2012, NUCLEIC ACIDS RES, V40, P37, DOI 10.1093/nar/gkr688
Grabherr MG, 2011, NAT BIOTECHNOL, V29, P644, DOI 10.1038/nbt.1883
Grow EJ, 2015, NATURE, V522, P221, DOI 10.1038/nature14308
Haas BJ, 2013, NAT PROTOC, V8, P1494, DOI 10.1038/nprot.2013.084
Hadjicharalambous MR, 2019, NON-CODING RNA, V5, DOI 10.3390/ncrna5020034
Horie M, 2016, J VET MED SCI, V78, P1727, DOI 10.1292/jvms.16-0274
Irving AT, 2021, NATURE, V589, P363, DOI 10.1038/s41586-020-03128-0
Irving AT, 2020, CELL REP, V33, DOI 10.1016/j.celrep.2020.108345
Janardhana V, 2012, DEV COMP IMMUNOL, V36, P610, DOI 10.1016/j.dci.2011.11.001
Jin Y, 2015, BIOINFORMATICS, V31, P3593, DOI 10.1093/bioinformatics/btv422

Kohl C, 2021, VACCINES-BASEL, V9, DOI 10.3390/vaccines9070690
Kolde R., 2019, PHEATMAP PRETTY HEAT
Kozomara A, 2019, NUCLEIC ACIDS RES, V47, pD155, DOI 10.1093/nar/gky1141
Kumar S, 2017, MOL BIOL EVOL, V34, P1812, DOI 10.1093/molbev/msx116
Langmead B, 2012, NAT METHODS, V9, P357, DOI [10.1038/NMETH.1923, 10.1038/nmeth.1923]
Lee AJ, 2018, FRONT IMMUNOL, V9, DOI 10.3389/fimmu.2018.02061
Leroy EM, 2005, NATURE, V438, P575, DOI 10.1038/438575a
Li B, 2011, BMC BIOINFORMATICS, V12, DOI 10.1186/1471-2105-12-323
Li H, 2009, BIOINFORMATICS, V25, P1094, DOI [10.1093/bioinformatics/btp100, 10.1093/bioinformatics/btp324]
Liao Y, 2014, BIOINFORMATICS, V30, P923, DOI 10.1093/bioinformatics/btt656
LOVE MI, 2014, GENOME BIOL, V15, DOI DOI 10.1186/S13059-014-0550-8
Macchietto MG, 2020, LIFE SCI ALLIANCE, V3, DOI 10.26508/lsa.201900536
Mootha VK, 2003, NAT GENET, V34, P267, DOI 10.1038/ng1180
Moratelli R, 2015, MEM I OSWALDO CRUZ, V110, P1
Mu X, 2016, ADV IMMUNOL, V132, P47, DOI 10.1016/bs.ai.2016.07.001
Munster VJ, 2016, SCI REP-UK, V6, DOI 10.1038/srep21878
Nejad C, 2018, FEBS J, V285, P3695, DOI 10.1111/febs.14482
Olival KJ, 2014, VIRUSES-BASEL, V6, P1759, DOI 10.3390/v6041759
Omatsu T, 2008, VET IMMUNOL IMMUNOP, V124, P169, DOI 10.1016/j.vetimm.2008.03.004
Platt RN, 2014, MOL BIOL EVOL, V31, P1536, DOI 10.1093/molbev/msu112
Quinlan AR, 2010, BIOINFORMATICS, V26, P841, DOI 10.1093/bioinformatics/btq033
Rhein BA, 2015, PLOS PATHOG, V11, DOI 10.1371/journal.ppat.1005263
Rose SA, 2021, NAT IMMUNOL, V22, P914, DOI 10.1038/s41590-021-00944-y
Roulois D, 2015, CELL, V162, P961, DOI 10.1016/j.cell.2015.07.056
Rusinova I, 2013, NUCLEIC ACIDS RES, V41, pD1040, DOI 10.1093/nar/gks1215
Schoggins JW, 2011, CURR OPIN VIROL, V1, P519, DOI 10.1016/j.coviro.2011.10.008
Shen KL, 2020, WORLD J PEDIATR, V16, P232, DOI 10.1007/s12519-020-00362-4
Smit A.F.A., 2013, RepeatMasker Open-4.0
Subramanian A, 2005, P NATL ACAD SCI USA, V102, P15545, DOI 10.1073/pnas.0506580102
Nguyen TH, 2018, FRONT IMMUNOL, V9, DOI 10.3389/fimmu.2018.01541
Vandewege MW, 2016, GENOME BIOL EVOL, V8, P1327, DOI 10.1093/gbe/evw078
Xie JZ, 2018, CELL HOST MICROBE, V23, P297, DOI 10.1016/j.chom.2018.01.006
Yu G., 2020, enrichplot: Visualization of Functional Enrichment Result
Zhou P, 2016, P NATL ACAD SCI USA, V113, P2696, DOI 10.1073/pnas.1518240113

NR 58
TC 8
Z9 8
U1 0
U2 12
PU WILEY
PI HOBOKEN
PA 111 RIVER ST, HOBOKEN 07030-5774, NJ USA
SN 0385-5600
EI 1348-0421
J9 MICROBIOL IMMUNOL
JI Microbiol. Immunol.
PD MAR
PY 2022
VL 66
IS 3
BP 97
EP 112
DI 10.1111/1348-0421.12952
EA JAN 2022
PG 16
WC Immunology; Microbiology
WE Science Citation Index Expanded (SCI-EXPANDED)
SC Immunology; Microbiology
GA ZR0HM
UT WOS:000748511000001
PM 34842304
DA 2024-10-21
ER

PT J
AU Ruoss, S
   Becker, NI
   Otto, MS
   Czirják, GA
   Encarnaçao, JA
AF Ruoss, Sara
   Becker, Nina, I
   Otto, Matthias S.
   Czirjak, Gabor A.
   Encarnacao, Jorge A.
TI Effect of sex and reproductive status on the immunity of the temperate
   bat <i>Myotis daubentonii</i>
SO MAMMALIAN BIOLOGY
LA English
DT Article
DE Immunity; Chiroptera; Immunoglobulins; Leukocyte counts; Trade-off
ID LIFE-HISTORY; TRADE-OFFS; ECOLOGICAL IMMUNOLOGY; HUMORAL IMMUNITY;
   VIRUS-INFECTION; BROWN BAT; TESTOSTERONE; HORMONES; SYSTEM; STRESS
AB Studies of immunity in bat species are rare. However, it is

important to determine immunological variations to identify factors influencing the health status of these endangered mammals from an evolutionary, ecological, conservation, and public health point of view. Immunity is highly variable and can be influenced by both internal (e.g. hormone levels, energy demand) and external factors (e.g. pathogens, climate). As bats have some peculiar ecological, energetic, and putative immunological characteristics, they are outstanding study organisms for ecoimmunological studies. We tested if (i) female bats have a higher immunity than males similar to most other mammalian species and (ii) individuals differ according to their energy demand (e.g. reproductive status). To study these questions, we sampled female and male Myotis daubentonii with different reproductive states and estimated their bacterial killing activity, hemolysis/hemagglutination titer, immunoglobulin G (IgG) concentration, and total and differential white blood cell counts. These methods characterize the cellular and humoral branches of both the adaptive and the innate immune responses of these individuals. Reproductively active males had lower cellular immunity compared to non-reproductive individuals. Pregnant females had increased IgG concentrations while hemolysis was enhanced during lactation. No clear trade-off between immunity and reproduction was found; instead immunity of males and female bats seems to be modulated differently due to varying hormonal and energetic states. Our data suggest that both adaptive and innate immunity as well as individual differences (i.e. sex and reproductive state) need to be considered to get a comprehensive overall picture of immunity in wild mammals. (C) 2018 Deutsche Gesellschaft far Saugetierkunde. Published by Elsevier GmbH. All rights reserved.
C1 [Ruoss, Sara; Otto, Matthias S.; Encarnacao, Jorge A.] Justus Liebig Univ Giessen, Dept Anim Ecol & Systemat, Mammalian Ecol Grp, Heinrich Buff Ring 26 IFZ, D-35392 Giessen, Germany.
[Becker, Nina, I; Encarnacao, Jorge A.] Inst Appl Anim Ecol & Ecoinformat inat, Im Bruhl 2, D-35457 Lollar, Germany.
[Czirjak, Gabor A.] Leibniz Inst Zoo & Wildlife Res, Dept Wildlife Dis, Alfred Kowalke Str 17, D-10315 Berlin, Germany.
C3 Justus Liebig University Giessen; Leibniz Institut fur Zoo und Wildtierforschung
RP Encarnaçao, JA (corresponding author), Justus Liebig Univ Giessen, Dept Anim Ecol & Systemat, Mammalian Ecol Grp, Heinrich Buff Ring 26 IFZ, D-35392 Giessen, Germany.
EM J.Encarnacao@bio.uni-giessen.de
RI Czirják, Gábor/F-5440-2011; Encarnação, Jorge/I-8644-2018
OI Czirjak, Gabor Arpad/0000-0001-9488-0069
CR Adamo SA, 2004, ANIM BEHAV, V68, P1443, DOI 10.1016/j.anbehav.2004.05.005
Al-Afaleq AI, 1998, IMMUNOPHARM IMMUNOT, V20, P315, DOI 10.3109/08923979809038547
ALEXANDER J, 1988, PARASITOL TODAY, V4, P189, DOI 10.1016/0169-4758(88)90077-4
Allen LC, 2009, J COMP PHYSIOL B, V179, P315, DOI 10.1007/s00360-008-0315-3
[Anonymous], 2009, Bats of Britain, Europe and Northwest Africa
Anthony E.L. P., 1988, Ecological and Behavioral Methods for the Study of Bats, P1

ANTHONY ELP, 1981, OECOLOGIA, V51, P151, DOI 10.1007/BF00540593
ANTHONY ELP, 1977, ECOLOGY, V58, P775, DOI 10.2307/1936213
Apanius V, 1998, ADV STUD BEHAV, V27, P133
BAIN BJ, 1975, BRIT MED J, V1, P306, DOI 10.1136/bmj.1.5953.306
BARCLAY RMR, 1991, J ANIM ECOL, V60, P165, DOI 10.2307/5452
Barclay RMR, 2003, BAT ECOLOGY, P209
Becker DJ, 2018, PHILOS T R SOC B, V373, DOI 10.1098/rstb.2017.0089
Becker DJ, 2017, ROY SOC OPEN SCI, V4, DOI 10.1098/rsos.170073
Becker NI, 2013, ECOL RES, V28, P249, DOI 10.1007/s11284-012-1010-0
Becker NI, 2013, PHYSIOL BIOCHEM ZOOL, V86, P361, DOI 10.1086/670527
Boonstra R, 2001, ECOLOGY, V82, P1930, DOI 10.2307/2680058
Borghesi J., 2014, Open Journal of Animal Sciences, V4, P323
Bouma HR, 2010, J LEUKOCYTE BIOL, V88, P619, DOI 10.1189/jlb.0310174
Bouman A, 2005, HUM REPROD UPDATE, V11, P411, DOI 10.1093/humupd/dmi008
Buchanan KL, 2003, BEHAV ECOL SOCIOBIOL, V55, P50, DOI 10.1007/s00265-003-0682-4
Butterworth M, 1967, NATURE, V214, P1224
Christe P, 2000, ECOL LETT, V3, P207
Da Silva JAP, 1999, ANN NY ACAD SCI, V876, P102
Demas G., 2012, ECOIMMUNOLOGY, P259
Demas GE, 2011, J ANIM ECOL, V80, P710, DOI 10.1111/j.1365-2656.2011.01813.x
DEMPSTER G, 1966, J CELL PHYSIOL, V67, P443, DOI 10.1002/jcp.1040670309
Drazen DL, 2003, CAN J ZOOL, V81, P1406, DOI [10.1139/z03-120, 10.1139/Z03-120]
Drexler JF, 2011, EMERG INFECT DIS, V17, P449, DOI 10.3201/eid1703.100526
Duffy DL, 2000, BEHAV ECOL, V11, P654, DOI 10.1093/beheco/11.6.654
Ellison PT, 2003, AM J HUM BIOL, V15, P342, DOI 10.1002/ajhb.10152
Encarnaçao JA, 2004, MAMM BIOL, V69, P163, DOI 10.1078/1616-5047-00131
Encarnaçao JA, 2006, EUR J WILDLIFE RES, V52, P221, DOI 10.1007/s10344-006-0046-2
Encarnaçao JA, 2012, J ETHOL, V30, P271, DOI 10.1007/s10164-011-0323-8
Epstein JH, 2013, PLOS ONE, V8, DOI 10.1371/journal.pone.0067584
Fokidis HB, 2008, J AVIAN BIOL, V39, P300, DOI [10.1111/j.2008.0908-8857.04248.x, 10.1111/j.0908-8857.2008.04248.x]
FOLSTAD I, 1992, AM NAT, V139, P603, DOI 10.1086/285346
French SS, 2007, AM NAT, V170, P79, DOI 10.1086/518569
French SS, 2012, BIOL OPEN, V1, P482, DOI 10.1242/bio.2012919
Geiser F, 2004, ANNU REV PHYSIOL, V66, P239, DOI 10.1146/annurev.physiol.66.032102.115105
Georgea DB, 2011, P NATL ACAD SCI USA, V108, P10208, DOI 10.1073/pnas.1010875108
GITTLEMAN JL, 1988, AM ZOOL, V28, P863, DOI 10.1093/icb/28.3.863

Gloza-Rausch F, 2008, EMERG INFECT DIS, V14, P626, DOI 10.3201/eid1404.071439
Greiner S, 2010, GEN COMP ENDOCR, V168, P466, DOI 10.1016/j.ygcen.2010.06.008
Grindstaff JL, 2008, J EXP BIOL, V211, P654, DOI 10.1242/jeb.012344
Hammond KA, 1997, NATURE, V386, P457, DOI 10.1038/386457a0
Hayman DTS, 2013, ZOONOSES PUBLIC HLTH, V60, P2, DOI 10.1111/zph.12000
Heinrich SK, 2017, SCI REP-UK, V7, DOI 10.1038/srep44837
Heinrich SK, 2016, BIOL OPEN, V5, P550, DOI 10.1242/bio.014902
Hsieh MM, 2007, ANN INTERN MED, V146, P486, DOI 10.7326/0003-4819-146-7-200704030-00004
Klein SL, 2000, NEUROSCI BIOBEHAV R, V24, P627, DOI 10.1016/S0149-7634(00)00027-0
Krutzsch Philip H., 2000, P91, DOI 10.1016/B978-012195670-7/50005-9
KUNZ T H., 1994, Walker's Bats of the World, P1
Kunz TH, 1998, ECOSCIENCE, V5, P8, DOI 10.1080/11956860.1998.11682443
Lee KA, 2006, INTEGR COMP BIOL, V46, P1000, DOI 10.1093/icb/icl049
Lemke H, 2003, VACCINE, V21, P3428, DOI 10.1016/S0264-410X(03)00394-3
Lochmiller RL, 2000, OIKOS, V88, P87, DOI 10.1034/j.1600-0706.2000.880110.x
Love OP, 2008, AM NAT, V172, pE99, DOI 10.1086/589521
Lucan RK, 2009, FOREST ECOL MANAG, V258, P1301, DOI 10.1016/j.foreco.2009.06.032
Martin Len, 2000, P27, DOI 10.1016/B978-012195670-7/50003-5
Martin LB, 2008, PHILOS T R SOC B, V363, P321, DOI 10.1098/rstb.2007.2142
Matson KD, 2006, P ROY SOC B-BIOL SCI, V273, P815, DOI 10.1098/rspb.2005.3376
Matson KD, 2005, DEV COMP IMMUNOL, V29, P275, DOI 10.1016/j.dci.2004.07.006
Millet S, 2007, DEV COMP IMMUNOL, V31, P188, DOI 10.1016/j.dci.2006.05.013
Moore IT, 2009, INTEGR COMP BIOL, V49, P441, DOI 10.1093/icb/icp081
Moore MS, 2011, PLOS ONE, V6, DOI 10.1371/journal.pone.0027430
Mühldorfer K, 2011, PLOS ONE, V6, DOI 10.1371/journal.pone.0029773
Nelson R.J., 2002, SEASONAL PATTERNS ST
Norris K, 2000, BEHAV ECOL, V11, P19, DOI 10.1093/beheco/11.1.19
O'Shea TJ, 2014, EMERG INFECT DIS, V20, P741, DOI 10.3201/eid2005.130539
Ochsenbein AF, 1999, SCIENCE, V286, P2156, DOI 10.1126/science.286.5447.2156
Otto MS, 2013, MAMM BIOL, V78, P212, DOI 10.1016/j.mambio.2012.07.156
Pap PL, 2010, NATURWISSENSCHAFTEN, V97, P891, DOI 10.1007/s00114-010-0706-7
Plowright RK, 2008, P ROY SOC B-BIOL SCI, V275, P861, DOI

10.1098/rspb.2007.1260
Prendergast BJ, 2002, AM J PHYSIOL-REG I, V282, pR1054, DOI 10.1152/ajpregu.00562.2001
RACEY PA, 2009, ECOLOGICAL BEHAV MET
Roberts ML, 2004, ANIM BEHAV, V68, P227, DOI 10.1016/j.anbehav.2004.05.001
Schneeberger K, 2014, PLOS ONE, V9, DOI 10.1371/journal.pone.0108268
Schneeberger K, 2013, J EXP BIOL, V216, P4514, DOI 10.1242/jeb.090837
Schneeberger K, 2013, PLOS ONE, V8, DOI 10.1371/journal.pone.0054023
SCHUURS AHWM, 1990, J STEROID BIOCHEM, V35, P157, DOI 10.1016/0022-4731(90)90270-3
Seltmann A, 2017, CONSERV PHYSIOL, V5, DOI 10.1093/conphys/cox020
Sparkman AM, 2009, J ANIM ECOL, V78, P1242, DOI 10.1111/j.1365-2656.2009.01587.x
Speakman JR, 2008, PHILOS T R SOC B, V363, P375, DOI 10.1098/rstb.2007.2145
Strobel S, 2015, MAMM BIOL, V80, P312, DOI 10.1016/j.mambio.2015.02.005
Stubbe M., 2008, IUCN RED LIST THREAT, DOI [10.2305/IUCN.UK.2008.RLTS.T14128A4400742.en, DOI 10.2305/IUCN.UK.2008.RLTS.T14128A4400742.EN]
Telfer S, 2005, OIKOS, V109, P317, DOI 10.1111/j.0030-1299.2005.13734.x
Thompson S. D, 1993, MAMMALIAN ENERGETICS, P213
VALDIMARSSON H, 1983, CLIN EXP IMMUNOL, V53, P437
Zipfel PF, 2009, IMMUNOL LETT, V126, P1, DOI 10.1016/j.imlet.2009.07.005
Zuk M, 1996, INT J PARASITOL, V26, P1009, DOI [10.1016/S0020-7519(96)00086-0, 10.1016/S0020-7519(96)80001-4]
Zuk M, 2002, AM NAT, V160, pS9, DOI 10.1086/342131
NR 92
TC 16
Z9 19
U1 1
U2 25
PU SPRINGER HEIDELBERG
PI HEIDELBERG
PA TIERGARTENSTRASSE 17, D-69121 HEIDELBERG, GERMANY
SN 1616-5047
EI 1618-1476
J9 MAMM BIOL
JI Mamm. Biol.
PD JAN
PY 2019
VL 94
IS 1
BP 120
EP 126
DI 10.1016/j.mambio.2018.05.010
PG 7
WC Zoology

WE Science Citation Index Expanded (SCI-EXPANDED)
SC Zoology
GA HK7JX
UT WOS:000458165800017
PM 32218715
OA Green Published
DA 2024-10-21
ER

PT J
AU Herrera, MLG
Hernández-Arciga, U
González-Carcacia, JA
Nassar, JM
AF Herrera, M. L. Gerardo
Hernandez-Arciga, Ulalume
Gonzalez-Carcacia, Jose Antonio
Nassar, Jafet M.
TI Olfactory cues of mate quality in mammals: inflammatory response is
higher in males of long-nosed bats with odorous dorsal patch
SO MAMMAL RESEARCH
LA English
DT Article
DE Bats; Immune response; Mate choice; Nectarivorous bats;
Phytohemagglutinin
ID LEPTONYCTERIS-CURASOAE CHIROPTERA; IMMUNE FUNCTION; PHYLLOSTOMIDAE;
IMMUNOCOMPETENCE; GLOSSOPHAGINAE; COLORATION; BACTERIA; STATE
AB Bats use olfactory signals to provide information of species and individual identity and quality. Males of two species of long-nosed bats, Leptonycteris curasoae and L. yerbabuenae, display an odoriferous dorsal patch during the mating season that is involved in mate choice. The dorsal patch is documented to serve as an indicator of male quality; for instance, males with dorsal patch present larger testes and lower ectoparasite loads. We evaluated the role of the dorsal patch in males of L. curasoae and L. yerbabuenae as an indicator of inflammatory response 6 and 12 h after being injected with phytohemagglutinin (PHA). We found that the inflammatory response of males of both species was not different between reproductively active bats with or without dorsal patch 6 h after PHA injection, but the response was higher in bats with dorsal patch 12 h after the injection. Our study adds to the evidence that the dorsal patch exhibited by males of long-nosed bats indicates their immune quality for mating females. Further work including other branches of the immune system and the potential role of microbiota in the strength of the inflammatory response is warranted.
C1 [Herrera, M. L. Gerardo] Univ Nacl Autonoma Mexico, Estn Biol Chamela, Inst Biol, Apartado Postal 21, San Patricio 48980, Jalisco, Mexico.
[Hernandez-Arciga, Ulalume] Univ Nacl Autonoma Mexico, Posgrado Ciencias Biol, Unidad Posgrado, Edificio D,1 Piso,Circuito Posgrados,Ciudad Univ, Mexico City 04510, Mexico.

[Hernandez-Arciga, Ulalume] Univ Pittsburgh, Aging Inst, Bridgeside Point 1 100 Technol Dr, Pittsburgh, PA 15219 USA.
[Gonzalez-Carcacia, Jose Antonio; Nassar, Jafet M.] Inst Venezolano Invest Cient, Ctr Ecol, Altos Pipe, Miranda 1020A, Venezuela.
C3 Universidad Nacional Autonoma de Mexico; Universidad Nacional Autonoma
de Mexico; Pennsylvania Commonwealth System of Higher Education (PCSHE);
University of Pittsburgh; Venezuelan Institute Science Research
RP Herrera, MLG (corresponding author), Univ Nacl Autonoma Mexico, Estn Biol Chamela, Inst Biol, Apartado Postal 21, San Patricio 48980, Jalisco, Mexico.; Nassar, JM (corresponding author), Inst Venezolano Invest Cient, Ctr Ecol, Altos Pipe, Miranda 1020A, Venezuela.
EM gherrera@ib.unam.mx; herarc_ula9@hotmail.com; jagcarcacia@hotmail.com;
jafet.nassar@gmail.com
CR Adams DM, 2018, J CHEM ECOL, V44, P975, DOI 10.1007/s10886-018-1003-8
Allen LC, 2009, J COMP PHYSIOL B, V179, P315, DOI 10.1007/s00360-008-0315-3
[Anonymous], 2011, Statistica Data Analysis Software System
Bílková B, 2015, J EXP ZOOL PART A, V323, P767, DOI 10.1002/jez.1990
Blount JD, 2003, SCIENCE, V300, P125, DOI 10.1126/science.1082142
Chaverri G, 2018, BIOL REV, V93, P1938, DOI 10.1111/brv.12427
Christe P, 2000, ECOL LETT, V3, P207
de Veerdonk FLV, 2011, TRENDS IMMUNOL, V32, P110, DOI 10.1016/j.it.2011.01.003
Faivre B, 2003, ANIM BEHAV, V65, P1125, DOI 10.1006/anbe.2003.2142
Galindo C, 2004, BIOTROPICA, V36, P382, DOI 10.1646/03107
Gannon WL, 2007, J MAMMAL, V88, P809, DOI 10.1644/06-MAMM-F-185R1.1
Gaona O, 2019, PLOS ONE, V14, DOI 10.1371/journal.pone.0226239
Garvin JC, 2008, BEHAV ECOL, V19, P54, DOI 10.1093/beheco/arm099
Greenman CG, 2005, PHYSIOL BIOCHEM ZOOL, V78, P60, DOI 10.1086/425194
Iglesias-Carrasco M, 2016, BMC EVOL BIOL, V16, DOI 10.1186/s12862-016-0706-0
Ingala MR, 2019, ECOL EVOL, V9, P6508, DOI 10.1002/ece3.5228
Johansson BG, 2007, BIOL REV, V82, P265, DOI 10.1111/j.1469-185X.2007.00009.x
Martín J, 2010, NATURWISSENSCHAFTEN, V97, P545, DOI 10.1007/s00114-010-0669-8
Martin LB, 2006, FUNCT ECOL, V20, P290, DOI 10.1111/j.1365-2435.2006.01094.x
Merlo JL, 2014, COMP BIOCHEM PHYS A, V175, P90, DOI 10.1016/j.cbpa.2014.05.021
Merrill L, 2014, AUK, V131, P3, DOI 10.1642/AUK-13-020-R1.1
Miller GA, 2001, J ABNORM PSYCHOL, V110, P40, DOI 10.1037//0021-843X.110.1.40
Mougeot F, 2009, J AVIAN BIOL, V40, P67, DOI 10.1111/

j.1600-048X.2008.04439.x
Muñoz-Romo M, 2021, MAMMAL REV, V51, P155, DOI 10.1111/mam.12232
Muñoz-Romo M, 2020, BEHAV ECOL SOCIOBIOL, V74, DOI 10.1007/s00265-020-02914-0
Muñoz-Romo M, 2012, ACTA CHIROPTEROL, V14, P213, DOI 10.3161/150811012X654411
Muñoz-Romo M, 2011, ACTA CHIROPTEROL, V13, P207, DOI 10.3161/150811011X578769
Muñoz-Romo M, 2011, BEHAVIOUR, V148, P461, DOI 10.1163/000579511X564287
Muñoz-Romo M, 2009, J MAMMAL, V90, P1139, DOI 10.1644/08-MAMM-A-324.1
Nassar JM, 2008, ZOOLOGY, V111, P363, DOI 10.1016/j.zool.2007.10.006
Nunn CL, 2000, SCIENCE, V290, P1168, DOI 10.1126/science.290.5494.1168
Otálora-Ardila A, 2016, PLOS ONE, V11, DOI 10.1371/journal.pone.0164938
Plasman M, 2015, BEHAV ECOL SOCIOBIOL, V69, P765, DOI 10.1007/s00265-015-1892-2
Rincón-Vargas F, 2013, J MAMMAL, V94, P488, DOI 10.1644/11-MAMM-A-357.1
Smits JE, 1999, FUNCT ECOL, V13, P567, DOI 10.1046/j.1365-2435.1999.00338.x
Trigo S, 2015, BEHAV ECOL SOCIOBIOL, V69, P481, DOI 10.1007/s00265-014-1860-2
Turmelle AS, 2010, J COMP PHYSIOL B, V180, P1155, DOI 10.1007/s00360-010-0486-6
Vinkler M, 2014, J AVIAN BIOL, V45, P43, DOI 10.1111/j.1600-048X.2011.05860.x
Vinkler M, 2012, ANIM BEHAV, V83, P17, DOI 10.1016/j.anbehav.2011.10.001
Vinkler M, 2011, J AVIAN BIOL, V42, P490, DOI 10.1111/j.1600-048X.2011.05499.x
Vinkler M, 2010, FUNCT ECOL, V24, P1081, DOI 10.1111/j.1365-2435.2010.01711.x
Voigt CC, 2005, J MAMMAL, V86, P745, DOI 10.1644/1545-1542(2005)086[0745:BBABSS]2.0.CO;2
Voigt Christian C., 2014, P409
Voigt CC, 2008, J MAMMAL, V89, P1401, DOI 10.1644/08-MAMM-S-060.1
Vroonen J, 2013, AMPHIBIA-REPTILIA, V34, P539, DOI 10.1163/15685381-00002916
York JE, 2016, ECOL EVOL, V6, P1008, DOI 10.1002/ece3.1938
Zhang CM, 2022, ANN NY ACAD SCI, V1509, P74, DOI 10.1111/nyas.14712
NR 47
TC 0
Z9 0
U1 0
U2 6
PU SPRINGER HEIDELBERG
PI HEIDELBERG
PA TIERGARTENSTRASSE 17, D-69121 HEIDELBERG, GERMANY
SN 2199-2401

EI 2199-241X
J9 MAMMAL RES
JI Mammal Res.
PD JUL
PY 2023
VL 68
IS 3
BP 367
EP 373
DI 10.1007/s13364-023-00684-4
EA APR 2023
PG 7
WC Zoology
WE Science Citation Index Expanded (SCI-EXPANDED)
SC Zoology
GA I4JF2
UT WOS:000968660100002
OA hybrid
DA 2024-10-21
ER

PT J
AU Fuchs, J
   Hölzer, M
   Schilling, M
   Patzina, C
   Schoen, A
   Hoenen, T
   Zimmer, G
   Marz, M
   Weber, F
   Müller, MA
   Kochs, G
AF Fuchs, Jonas
   Hoelzer, Martin
   Schilling, Mirjam
   Patzina, Corinna
   Schoen, Andreas
   Hoenen, Thomas
   Zimmer, Gert
   Marz, Manja
   Weber, Friedemann
   Mueller, Marcel A.
   Kochs, Georg
TI Evolution and Antiviral Specificities of Interferon-Induced Mx
Proteins
   of Bats against Ebola, Influenza, and Other RNA Viruses
SO JOURNAL OF VIROLOGY
LA English
DT Article
DE Mx protein; bat; bunyavirus; Ebola virus; influenza; interferons;
   orthomyxovirus; vesicular stomatitis virus
ID BORNE THOGOTO-VIRUS; BLACK FLYING FOX; LA-CROSSE VIRUS; A VIRUS;
   MOLECULAR CHARACTERIZATION; PHYLOGENETIC ANALYSIS; NUCLEOCAPSID

PROTEIN;
   GTPASE ACTIVATION; IMMUNE-RESPONSES; RIG-I
AB Bats serve as a reservoir for various, often zoonotic viruses, including significant human pathogens such as Ebola and influenza viruses. However, for unknown reasons, viral infections rarely cause clinical symptoms in bats. A tight control of viral replication by the host innate immune defense might contribute to this phenomenon. Transcriptomic studies revealed the presence of the interferon-induced antiviral myxovirus resistance (Mx) proteins in bats, but detailed functional aspects have not been assessed. To provide evidence that bat Mx proteins might act as key factors to control viral replication we cloned Mx1 cDNAs from three bat families, Pteropodidae, Phyllostomidae, and Vespertilionidae. Phylogenetically these bat Mx1 genes cluster closely with their human ortholog MxA. Using transfected cell cultures, minireplicon systems, virus-like particles, and virus infections, we determined the antiviral potential of the bat Mx1 proteins. Bat Mx1 significantly reduced the polymerase activity of viruses circulating in bats, including Ebola and influenza A-like viruses. The related Thogoto virus, however, which is not known to infect bats, was not inhibited by bat Mx1. Further, we provide evidence for positive selection in bat Mx1 genes that might explain species-specific antiviral activities of these proteins. Together, our data suggest a role for Mx1 in controlling these viruses in their bat hosts.
   IMPORTANCE Bats are a natural reservoir for various viruses that rarely cause clinical symptoms in bats but are dangerous zoonotic pathogens, like Ebola or rabies virus. It has been hypothesized that the interferon system might play a key role in controlling viral replication in bats. We speculate that the interferon-induced Mx proteins might be key antiviral factors of bats and have coevolved with bat-borne viruses. This study evaluated for the first time a large set of bat Mx1 proteins spanning three major bat families for their antiviral potential, including activity against Ebola virus and bat influenza A-like virus, and we describe here their phylogenetic relationship, revealing patterns of positive selection that suggest a coevolution with viral pathogens. By understanding the molecular mechanisms of the innate resistance of bats against viral diseases, we might gain important insights into how to prevent and fight human zoonotic infections caused by bat-borne viruses.
C1 [Fuchs, Jonas; Schilling, Mirjam; Patzina, Corinna; Kochs, Georg] Univ Freiburg, Med Ctr, Inst Virol, Freiburg, Germany.
   [Fuchs, Jonas; Kochs, Georg] Univ Freiburg, Fac Med, Freiburg, Germany.
   [Hoelzer, Martin; Marz, Manja] Friedrich Schiller Univ Jena, Fac Math & Comp Sci, Jena, Germany.
   [Schoen, Andreas; Weber, Friedemann] Justus Liebig Univ Giessen, FB Vet Med 10, Inst Virol, Giessen, Germany.
   [Hoenen, Thomas] Greifswald Insel Riems, Friedrich Loeffler Inst, Sydney, NSW, Germany.
   [Hoenen, Thomas] NIAID, Virol Lab, Div Intramural Res, NIH, Hamilton, MT USA.
   [Weber, Friedemann] FLI Leibniz Inst Age Res, Jena, Germany.
   [Mueller, Marcel A.; Kochs, Georg] Univ Med Ctr Bonn, Inst Virol, Bonn, Germany.

C3 University of Freiburg; University of Freiburg; Friedrich Schiller
University of Jena; Justus Liebig University Giessen; Friedrich Loeffler
Institute; National Institutes of Health (NIH) - USA; NIH National
Institute of Allergy & Infectious Diseases (NIAID); Leibniz Institut fur
Alternsforschung - Fritz-Lipmann-Institut (FLI); University of Bonn
RP Kochs, G (corresponding author), Univ Freiburg, Med Ctr, Inst Virol, Freiburg, Germany.; Kochs, G (corresponding author), Univ Freiburg, Fac Med, Freiburg, Germany.
EM georg.kochs@uniklinik-freiburg.de
RI Schilling, Mirjam/GZN-1748-2022; Hölzer, Martin/J-4725-2019; Weber,
Friedemann/AAB-3425-2019; Müller, Marcel/O-2425-2019; Hoenen,
Thomas/H-8990-2019
OI Fuchs, Jonas/0000-0003-1974-212X; Mueller, Marcel/0000-0003-2242-5117;
Schilling, Mirjam/0000-0001-5386-3609; Zimmer, Gert/0000-0002-2708-2507;
Holzer, Martin/0000-0001-7090-8717; Hoenen, Thomas/0000-0002-5829-6305;
Marz, Manja/0000-0003-4783-8823; Weber, Friedemann/0000-0001-9737-337X;
Schoen, Andreas/0000-0002-1711-4800
FU Deutsche Forschungsgemeinschaft [SSP1596/2, KO1579/9-2, WE2616/7-2,
MA5082/7-1, MU3564/1-1]; Intramural Research Program of the National
Institutes of Health, NIAID
FX This work was supported by funding from the Deutsche
Forschungsgemeinschaft, priority program-SSP1596/2, to Georg Kochs
(KO1579/9-2), Friedemann Weber (WE2616/7-2), Manja Marz (MA5082/7-1),
and Marcel A. Muller (MU3564/1-1) and by the Intramural Research Program
of the National Institutes of Health, NIAID, to Thomas Hoenen.
CR Abascal F, 2010, NUCLEIC ACIDS RES, V38, pW7, DOI 10.1093/nar/gkq291
ALBANESE M, 1972, ACTA VIROL, V16, P267
Alcaide M, 2009, PLOS ONE, V4, DOI 10.1371/journal.pone.0007092
Almeida MF, 2005, EPIDEMIOL INFECT, V133, P523, DOI 10.1017/S0950268804003656
Andersson I, 2004, J VIROL, V78, P4323, DOI 10.1128/JVI.78.8.4323-4329.2004
Arguin PM, 2002, EMERG INFECT DIS, V8, P258, DOI 10.3201/eid0803.010330
ARTHUR DR, 1956, J PARASITOL, V42, P180, DOI 10.2307/3274734
Baker ML, 2013, ZOONOSES PUBLIC HLTH, V60, P104, DOI 10.1111/j.1863-2378.2012.01528.x
Biesold SE, 2011, PLOS ONE, V6, DOI 10.1371/journal.pone.0028131

Binger T, 2015, J VIROL, V89, P4588, DOI 10.1128/JVI.02932-14
Bouloy M, 2001, J VIROL, V75, P1371, DOI 10.1128/JVI.75.3.1371-1377.2001
Busnadiego I, 2014, J VIROL, V88, P7738, DOI 10.1128/JVI.00214-14
Calisher CH, 2006, CLIN MICROBIOL REV, V19, P531, DOI 10.1128/CMR.00017-06
Cowled C, 2012, DEV COMP IMMUNOL, V36, P657, DOI 10.1016/j.dci.2011.11.008
Cowled C, 2011, DEV COMP IMMUNOL, V35, P7, DOI 10.1016/j.dci.2010.07.006
Dick A, 2015, J BIOL CHEM, V290, P12779, DOI 10.1074/jbc.M115.650325
Dittmann J, 2008, J VIROL, V82, P3624, DOI 10.1128/JVI.01753-07
Edgar RC, 2004, NUCLEIC ACIDS RES, V32, P1792, DOI 10.1093/nar/gkh340
Field H, 1999, EMERG INFECT DIS, V5, P438, DOI 10.3201/eid0503.990316
Flohr F, 1999, FEBS LETT, V463, P24, DOI 10.1016/S0014-5793(99)01598-7
FRESE M, 1995, J VIROL, V69, P3904, DOI 10.1128/JVI.69.6.3904-3909.1995
Frese M, 1996, J VIROL, V70, P915, DOI 10.1128/JVI.70.2.915-923.1996
Fricke T, 2014, RETROVIROLOGY, V11, DOI 10.1186/s12977-014-0068-x
Gao S, 2011, IMMUNITY, V35, P514, DOI 10.1016/j.immuni.2011.07.012
Götz V, 2016, SCI REP-UK, V6, DOI 10.1038/srep23138
Grabherr MG, 2011, NAT BIOTECHNOL, V29, P644, DOI 10.1038/nbt.1883
Guo WZ, 2013, INT J DISTRIB SENS N, DOI 10.1155/2013/985410
Habjan M, 2009, VIROLOGY, V385, P400, DOI 10.1016/j.virol.2008.12.011
HALLER O, 1995, J VIROL, V69, P2596, DOI 10.1128/JVI.69.4.2596-2601.1995
Haller O, 2015, TRENDS MICROBIOL, V23, P154, DOI 10.1016/j.tim.2014.12.003
Hayman DTS, 2010, PLOS ONE, V5, DOI 10.1371/journal.pone.0011978
Hefti HP, 1999, J VIROL, V73, P6984, DOI 10.1128/JVI.73.8.6984-6991.1999
Holzinger D, 2007, J VIROL, V81, P7776, DOI 10.1128/JVI.00546-06
HORISBERGER MA, 1987, J GEN VIROL, V68, P945, DOI 10.1099/0022-1317-68-3-945
Jones KE, 2002, BIOL REV, V77, P223, DOI 10.1017/S1464793101005899
Juozapaitis M, 2014, NAT COMMUN, V5, DOI 10.1038/ncomms5448
KISHINO H, 1989, J MOL EVOL, V29, P170, DOI 10.1007/BF02100115
Klompen JSH, 1996, ANNU REV ENTOMOL, V41, P141, DOI 10.1146/annurev.en.41.010196.001041
Kochs G, 2002, P NATL ACAD SCI USA, V99, P3153, DOI 10.1073/pnas.052430399
Kochs G, 1999, P NATL ACAD SCI USA, V96, P2082, DOI 10.1073/pnas.96.5.2082
Kopp A, 2013, MBIO, V4, DOI 10.1128/mBio.00322-13
Pond SLK, 2006, MOL BIOL EVOL, V23, P1891, DOI 10.1093/molbev/

msl051
Kühl A, 2011, J INFECT DIS, V204, pS840, DOI 10.1093/infdis/jir306
Leroy EM, 2005, NATURE, V438, P575, DOI 10.1038/438575a
Li JJ, 2015, GENE, V561, P115, DOI 10.1016/j.gene.2015.02.013
Li WZ, 2006, BIOINFORMATICS, V22, P1658, DOI 10.1093/bioinformatics/btl158
Luis AD, 2013, P ROY SOC B-BIOL SCI, V280, DOI 10.1098/rspb.2012.2753
Mänz B, 2013, PLOS PATHOG, V9, DOI 10.1371/journal.ppat.1003279
Mandl JN, 2015, CELL, V160, P20, DOI 10.1016/j.cell.2014.12.003
Martin DP, 2015, VIRUS EVOL, V1, DOI 10.1093/ve/vev003
McBee RM, 2015, MOL BIOL EVOL, V32, P1091, DOI 10.1093/molbev/msu399
Mitchell PS, 2015, PLOS PATHOG, V11, DOI 10.1371/journal.ppat.1005304
Mitchell PS, 2013, CURR OPIN MICROBIOL, V16, P493, DOI 10.1016/j.mib.2013.04.005
Mitchell PS, 2012, CELL HOST MICROBE, V12, P598, DOI 10.1016/j.chom.2012.09.005
Mordstein M, 2008, PLOS PATHOG, V4, DOI 10.1371/journal.ppat.1000151
Müller MA, 2016, SCI REP-UK, V6, DOI 10.1038/srep26637
Nigg PE, 2015, J BIOL CHEM, V290, P29893, DOI 10.1074/jbc.M115.681494
NIWA H, 1991, GENE, V108, P193, DOI 10.1016/0378-1119(91)90434-D
Papenfuss AT, 2012, BMC GENOMICS, V13, DOI 10.1186/1471-2164-13-261
PAVLOVIC J, 1990, J VIROL, V64, P3370, DOI 10.1128/JVI.64.7.3370-3375.1990
PAVLOVIC J, 1992, J VIROL, V66, P2564, DOI 10.1128/JVI.66.4.2564-2569.1992
Pond SLK, 2005, BIOINFORMATICS, V21, P676, DOI 10.1093/bioinformatics/bti079
Pourrut X, 2009, BMC INFECT DIS, V9, DOI 10.1186/1471-2334-9-159
Reichelt M, 2004, TRAFFIC, V5, P772, DOI 10.1111/j.1600-0854.2004.00219.x
Rentsch MB, 2011, PLOS ONE, V6, DOI 10.1371/journal.pone.0025858
Schulz MH, 2012, BIOINFORMATICS, V28, P1086, DOI 10.1093/bioinformatics/bts094
SCHWEMMLE M, 1995, VIROLOGY, V206, P545, DOI 10.1016/S0042-6822(95)80071-9
Shaw TI, 2012, PLOS ONE, V7, DOI 10.1371/journal.pone.0048472
Simmons NB, 2005, SCIENCE, V307, P527, DOI 10.1126/science.1108871
Stamatakis A, 2014, BIOINFORMATICS, V30, P1312, DOI 10.1093/bioinformatics/btu033
Stertz S, 2006, J INTERF CYTOK RES, V26, P650, DOI 10.1089/jir.2006.26.650
Tong SX, 2013, PLOS PATHOG, V9, DOI 10.1371/journal.ppat.1003657
Tong SX, 2012, P NATL ACAD SCI USA, V109, P4269, DOI 10.1073/pnas.1116200109
Verhelst J, 2012, J VIROL, V86, P13445, DOI 10.1128/JVI.01682-12
Wang LF, 2011, CURR OPIN VIROL, V1, P649, DOI 10.1016/

j.coviro.2011.10.013
   Watt A, 2014, J VIROL, V88, P10511, DOI 10.1128/JVI.01272-14
   Weiss S, 2012, EMERG INFECT DIS, V18, P159, DOI 10.3201/
eid1801.111026
   Wynne JW, 2013, PLOS PATHOG, V9, DOI 10.1371/journal.ppat.1003651
   Xiao H, 2013, J VIROL, V87, P13053, DOI 10.1128/JVI.02220-13
   Xie YL, 2014, BIOINFORMATICS, V30, P1660, DOI 10.1093/
bioinformatics/btu077
   Yang ZH, 2007, MOL BIOL EVOL, V24, P1586, DOI 10.1093/molbev/
msm088
   Zerbino DR, 2008, GENOME RES, V18, P821, DOI 10.1101/
gr.074492.107
   Zhou P, 2016, P NATL ACAD SCI USA, V113, P2696, DOI 10.1073/
pnas.1518240113
   Zhou P, 2013, DEV COMP IMMUNOL, V40, P240, DOI 10.1016/
j.dci.2013.03.006
   Zimmermann P, 2011, J VIROL, V85, P8133, DOI 10.1128/JVI.00712-11
NR 85
TC 45
Z9 52
U1 2
U2 34
PU AMER SOC MICROBIOLOGY
PI WASHINGTON
PA 1752 N ST NW, WASHINGTON, DC 20036-2904 USA
SN 0022-538X
EI 1098-5514
J9 J VIROL
JI J. Virol.
PD AUG
PY 2017
VL 91
IS 15
AR e00361
DI 10.1128/JVI.00361-17
PG 20
WC Virology
WE Science Citation Index Expanded (SCI-EXPANDED)
SC Virology
GA FB0WV
UT WOS:000405866600011
PM 28490593
OA Green Published, Bronze
DA 2024-10-21
ER

PT J
AU Rivera-Ruiz, DA
   Flores-Martínez, JJ
   Rosales, C
   Montalvo, LGH
AF Rivera-Ruiz, David A.
   Flores-Martinez, Jose Juan
   Rosales, Carlos

Herrera Montalvo, Luis Gerardo
TI Constitutive Innate Immunity of Migrant and Resident Long-Nosed Bats
(<i>Leptonycteris yerbabuenae</i>) in the Drylands of Mexico
SO DIVERSITY-BASEL
LA English
DT Article
DE bactericidal activity; bats; immune response; migratory habits; nectarivorous bats
ID NECTAR-FEEDING BATS; CURASOAE CHIROPTERA; POPULATION-DYNAMICS; NECTARIVOROUS BATS; SMEARING BEHAVIOR; ANIMAL MIGRATION; NORMAL-PREGNANCY; DORSAL PATCH; COMPLEMENT; PHYLLOSTOMIDAE
AB In contrast to birds, the relationship between migration and immunity has been scarcely studied in bats. We examined how the expression of the humoral portion of the constitutive immunity varied in a bat with partial, sex-biased migration: the lesser long-nosed bat (Leptonycteris yerbabuenae (Phyllostomidae)). The lesser long-nosed bat is a nectarivorous species distributed in the arid and semi-arid regions of North and Central America. We evaluated the bacteria-killing abilities (BKAs) of the plasma of male and female lesser long-nosed bats on the Pacific coast in different periods of the year. Because adult males are resident, they were used to explore the effect of reproductive activity on BKA, and we predicted higher values in mating males (i.e., individuals presenting scrotal testicles and a fresh dorsal patch). In contrast to males, most females migrate to cactus deserts in northern Mexico during pregnancy and lactation, and then return to the dry forests of west-central Mexico to mate. We predicted that the combined effect of breeding and migration would have an adverse effect on BKA; therefore, migratory pregnant and lactating females were expected to exhibit a lower BKA than mating females in west-central Mexico. We compared the BKAs of females captured in October and December in central Mexico, and we predicted that migratory females that had recently arrived in October should exhibit a lower BKA than females captured two months later. In addition, we compared the BKAs between lactating females and young in northern Mexico and predicted lower values in recently born individuals. We found that the BKAs of males were higher in reproductive individuals than in non-reproductive individuals. We found a significant difference in the BKAs between females at the two extremes of their migratory range: the values of pregnant females in Sonora and females in December were higher than those of females captured in October. Finally, we found no difference in BKAs between lactating females and young individuals. Our findings indicate that the basal levels of the innate humoral component are heightened in mating males, that this response is reduced in females that recently returned to their mating grounds, and that the constitutive immunity of young individuals matures early, probably in anticipation of the potential to encounter pathogens during their migration to west-central Mexico.
C1 [Rivera-Ruiz, David A.] Unidad Posgrad, Posgrad Ciencias Biol, Edificio D,1 Piso,Circuito Posgrad,Ciudad Univ, Mexico City 04510, Mexico.
[Rivera-Ruiz, David A.; Herrera Montalvo, Luis Gerardo] Univ Nacl Autonoma Mexico, Estn Biol Chamela, Inst Biol, Apartado Postal 21,

San Patricio 48989, Jalisco, Mexico.
[Flores-Martinez, Jose Juan] Univ Nacl Autonoma Mexico, Dept Zool, Ins Biol, Lab Sistemas Informac Geograf, Ciudad Univ, Mexico City 04510, Mexico.
[Rosales, Carlos] Univ Nacl Autonoma Mexico, Dept Inmunol, Inst Invest Biomed, Circuito Exterior S-N,Ciudad Univ, Mexico City 04510, Mexico.
C3 Universidad Nacional Autonoma de Mexico; Universidad Nacional Autonoma
de Mexico; Universidad Nacional Autonoma de Mexico
RP Montalvo, LGH (corresponding author), Univ Nacl Autonoma Mexico, Estn Biol Chamela, Inst Biol, Apartado Postal 21, San Patricio 48989, Jalisco, Mexico.
EM david.rivera@correounivalle.edu.co; jj@ib.unam.mx;
carosal@iibiomedicas.unam.mx; gherrera@ib.unam.mx
RI ROSALES, CARLOS/AAA-5206-2019
OI FLORES MARTINEZ, JOSE JUAN/0000-0001-7178-8294; ROSALES,
CARLOS/0000-0003-2568-5723
FU Programa de Apoyo a Proyectos de Investigacion e Innovacion Tecnologica,
Direccion General de Asuntos del Personal Academico-UNAM [IN204219];
Consejo Nacional de Ciencia y Tecnologia
FX The research was funded by the Programa de Apoyo a Proyectos de
Investigacion e Innovacion Tecnologica, Direccion General de Asuntos del
Personal Academico-UNAM (#IN204219) to L.G.H.M. The Consejo Nacional de
Ciencia y Tecnologia supported D.A.R.-R. with a graduate student grant.
CR Aastrup C, 2021, DEV COMP IMMUNOL, V117, DOI 10.1016/j.dci.2020.103967
Abbassy Magda M, 2007, Egypt J Immunol, V14, P43
Allen LC, 2009, J COMP PHYSIOL B, V179, P315, DOI 10.1007/s00360-008-0315-3
Altizer S, 2011, SCIENCE, V331, P296, DOI 10.1126/science.1194694
Anthony E.L.P., 1988, P47
Avgar T, 2014, CAN J ZOOL, V92, P481, DOI 10.1139/cjz-2013-0076
Bagherimoghaddam A, 2022, HEALTH SCI REP-US, V5, DOI 10.1002/hsr2.519
Becker DJ, 2019, PHYSIOL BIOCHEM ZOOL, V92, P37, DOI 10.1086/701069
Becker DJ, 2018, PHILOS T R SOC B, V373, DOI 10.1098/rstb.2017.0089
Becker DJ, 2017, ROY SOC OPEN SCI, V4, DOI 10.1098/rsos.170073
Brooks ME, 2017, R J, V9, P378, DOI 10.32614/RJ-2017-066
Buehler DM, 2010, INTEGR COMP BIOL, V50, P346, DOI 10.1093/icb/icq055
Burke RA, 2019, DIVERS DISTRIB, V25, P1399, DOI 10.1111/ddi.12950
Cacho NT, 2017, FRONT IMMUNOL, V8, DOI 10.3389/fimmu.2017.00584
Calisher CH, 2006, CLIN MICROBIOL REV, V19, P531, DOI 10.1128/CMR.00017-06
CAREN LD, 1966, SCIENCE, V152, P782, DOI 10.1126/science.152.3723.782

Ceballos G, 1997, J MAMMAL, V78, P1220, DOI 10.2307/1383065
Christe P, 2000, ECOL LETT, V3, P207
COCKRUM E L, 1991, Anales del Instituto de Biologia Universidad Nacional Autonoma de Mexico Serie Zoologia, V62, P181
Delignette-Muller ML, 2015, J STAT SOFTW, V64, P1, DOI 10.18637/jss.v064.i04
Demas G., 2012, ECOIMMUNOLOGY, P259
Derzsy Z, 2010, MOL IMMUNOL, V47, P1500, DOI 10.1016/j.molimm.2010.01.021
Dingle H, 2007, BIOSCIENCE, V57, P113, DOI 10.1641/B570206
Dutra DD, 2021, INT J PARASITOL, V51, P877, DOI 10.1016/j.ijpara.2021.03.001
Eikenaar C, 2020, CURR ZOOL, V66, P21, DOI 10.1093/cz/zoz009
Eikenaar C, 2018, ECOL EVOL, V8, P2721, DOI 10.1002/ece3.3756
Eikenaar C, 2016, BIOL LETTERS, V12, DOI 10.1098/rsbl.2016.0078
Ezenwa VO, 2012, FUNCT ECOL, V26, P123, DOI 10.1111/j.1365-2435.2011.01919.x
Fleming TH, 2003, BAT ECOLOGY, P156
FLEMING TH, 1993, OECOLOGIA, V94, P72, DOI 10.1007/BF00317304
Foo YZ, 2017, BIOL REV, V92, P551, DOI 10.1111/brv.12243
Fox J., 2011, An {R} companion to applied regression second
French SS, 2012, BIOL OPEN, V1, P482, DOI 10.1242/bio.2012919
Friedrichs V, 2022, CELL REP, V40, DOI 10.1016/j.celrep.2022.111305
Gaona O, 2019, PLOS ONE, V14, DOI 10.1371/journal.pone.0226239
Gilot-Fromont E, 2012, PLOS ONE, V7, DOI 10.1371/journal.pone.0045576
Hall RJ, 2022, ECOL EVOL INFECT DIS, P111, DOI 10.1093/oso/9780192895561.003.0007
Hartig Florian, 2022, CRAN
He YD, 2020, AM J REPROD IMMUNOL, V83, DOI 10.1111/aji.13202
Heesterbeek DAC, 2018, J INNATE IMMUN, V10, P455, DOI 10.1159/000491439
Hegemann A, 2019, J ORNITHOL, V160, P893, DOI 10.1007/s10336-019-01648-9
Hernández-Arciga U, 2018, PLOS ONE, V13, DOI 10.1371/journal.pone.0190047
Hernández-Castellano LE, 2019, J DAIRY SCI, V102, P8478, DOI 10.3168/jds.2019-16731
Herrera M L.G., MAMMAL RES
HOPKINSON ND, 1992, J CLIN PATHOL, V45, P66, DOI 10.1136/jcp.45.1.66
Hothorn T, 2008, BIOMETRICAL J, V50, P346, DOI 10.1002/bimj.200810425
Ingala MR, 2019, ECOL EVOL, V9, P6508, DOI 10.1002/ece3.5228
Kelly TR, 2017, J EXP ZOOL PART A, V327, P284, DOI 10.1002/jez.2088
Kuzmin IV, 2017, J VIROL, V91, DOI [10.1128/JVI.02471-16, 10.1128/jvi.02471-16]
Liebl AL, 2009, FUNCT ECOL, V23, P1091, DOI 10.1111/j.1365-2435.2009.01592.x
Lochmiller RL, 2000, OIKOS, V88, P87, DOI 10.1034/j.1600-0706.2000.880110.x
Mauck RA, 2005, FUNCT ECOL, V19, P1001, DOI 10.1111/

j.1365-2435.2005.01060.x
Medellin R., 2016, IUCN RED LIST THREAT, DOI [10.2305/IUCN.UK.2016-1.RLTS.T136659A21988965.en, DOI 10.2305/IUCN.UK.2016-1.RLTS.T136659A21988965.EN]
Menchaca A, 2020, GLOB ECOL CONSERV, V23, DOI 10.1016/j.gecco.2020.e01164
Merchant ME, 2003, COMP BIOCHEM PHYS B, V136, P505, DOI 10.1016/S1096-4959(03)00256-2
Miller GA, 2001, J ABNORM PSYCHOL, V110, P40, DOI 10.1037//0021-843X.110.1.40
Millet S, 2007, DEV COMP IMMUNOL, V31, P188, DOI 10.1016/j.dci.2006.05.013
Moore MS, 2011, PLOS ONE, V6, DOI 10.1371/journal.pone.0027430
Morales-Garza MR, 2007, J ARID ENVIRON, V68, P248, DOI 10.1016/j.jaridenv.2006.05.009
Mühldorfer K, 2013, ZOONOSES PUBLIC HLTH, V60, P93, DOI 10.1111/j.1863-2378.2012.01536.x
Muñoz-Romo M, 2011, ACTA CHIROPTEROL, V13, P207, DOI 10.3161/150811011X578769
Muñoz-Romo M, 2011, BEHAVIOUR, V148, P461, DOI 10.1163/000579511X564287
Muñoz-Romo M, 2009, J MAMMAL, V90, P1139, DOI 10.1644/08-MAMM-A-324.1
Nassar JM, 2008, ZOOLOGY, V111, P363, DOI 10.1016/j.zool.2007.10.006
Nassar JM, 2016, MAMM BIOL, V81, P623, DOI 10.1016/j.mambio.2016.08.003
Neuweiler G., 2000, BIOL BATS
Nunn CL, 2000, SCIENCE, V290, P1168, DOI 10.1126/science.290.5494.1168
Otálora-Ardila A, 2022, DIVERSITY-BASEL, V14, DOI 10.3390/d14100781
Owen JC, 2008, J ETHOL, V26, P383, DOI 10.1007/s10164-008-0092-1
Paweska JT, 2016, VIRUSES-BASEL, V8, DOI 10.3390/v8020029
Popa-Lisseanu AG, 2009, J MAMMAL, V90, P1283, DOI 10.1644/09-MAMM-S-130R2.1
Poulin R, 2021, BIOL REV, V96, P1331, DOI 10.1111/brv.12704
R Core Team, 2022, R LANG ENV STAT COMP
Richani K, 2005, J MATERN-FETAL NEO M, V17, P239, DOI 10.1080/14767050500072722
Rincón-Vargas F, 2013, J MAMMAL, V94, P488, DOI 10.1644/11-MAMM-A-357.1
Rogers EJ, 2022, J ANIM ECOL, V91, P858, DOI 10.1111/1365-2656.13681
Rojas-Martínez A, 1999, J BIOGEOGR, V26, P1065, DOI 10.1046/j.1365-2699.1999.00354.x
Ruiz M, 2010, HORM BEHAV, V57, P134, DOI 10.1016/j.yhbeh.2009.09.019
Ruoss S, 2019, MAMM BIOL, V94, P120, DOI 10.1016/j.mambio.2018.05.010
Schneeberger K, 2014, PLOS ONE, V9, DOI 10.1371/journal.pone.0108268
Shaw AK, 2020, J ANIM ECOL, V89, P1448, DOI 10.1111/1365-2656.13195

   Shaw AK, 2016, AM NAT, V187, P491, DOI 10.1086/685386
   Sikes RS, 2011, J MAMMAL, V92, P235, DOI 10.1644/10-MAMM-F-355.1
   Simon AK, 2015, P ROY SOC B-BIOL SCI, V282, DOI 10.1098/rspb.2014.3085
   Stoner KE, 2003, BIODIVERS CONSERV, V12, P357, DOI 10.1023/A:1021963819751
   Strobel S, 2015, MAMM BIOL, V80, P312, DOI 10.1016/j.mambio.2015.02.005
   Tieleman BI, 2005, P ROY SOC B-BIOL SCI, V272, P1715, DOI 10.1098/rspb.2005.3155
   Trejo-Salazar RE, 2021, PEERJ, V9, DOI 10.7717/peerj.12168
   ValienteBanuet A, 1996, J TROP ECOL, V12, P103, DOI 10.1017/S0266467400009330
   Voigt CC, 2020, SCI REP-UK, V10, DOI 10.1038/s41598-020-74473-3
   Walker William H, 2011, Spermatogenesis, V1, P116
   WEINTRAU.RM, 1966, SCIENCE, V152, P783, DOI 10.1126/science.152.3723.783
   Wilkinson GS, 2021, NAT COMMUN, V12, DOI 10.1038/s41467-021-21900-2
   Wilkinson GS, 1996, MOL ECOL, V5, P329
NR 94
TC 0
Z9 0
U1 1
U2 6
PU MDPI
PI BASEL
PA ST ALBAN-ANLAGE 66, CH-4052 BASEL, SWITZERLAND
EI 1424-2818
J9 DIVERSITY-BASEL
JI Diversity-Basel
PD APR
PY 2023
VL 15
IS 4
AR 530
DI 10.3390/d15040530
PG 15
WC Biodiversity Conservation; Ecology
WE Science Citation Index Expanded (SCI-EXPANDED)
SC Biodiversity & Conservation; Environmental Sciences & Ecology
GA F0FB6
UT WOS:000979181000001
OA gold
DA 2024-10-21
ER

PT J
AU Nunes, H
   Rocha, FL
   Cordeiro-Estrela, P
AF Nunes, Hannah
   Rocha, Fabiana Lopes
   Cordeiro-Estrela, Pedro

TI Bats in urban areas of Brazil: roosts, food resources and parasites in
disturbed environments
SO URBAN ECOSYSTEMS
LA English
DT Article
DE Anthropogenic impacts; Bat diversity; Chiroptera; Zoonosis; Urbanization
ID CHIROPTERA PHYLLOSTOMIDAE; INSECTIVOROUS BATS; ATLANTIC FOREST; URBANIZATION; CONSERVATION; BIODIVERSITY; RESERVOIRS; ABUNDANCE; DIPTERA; CLIMATE
AB Urbanization is a widespread intense land use that generally results in biodiversity decline. Among the taxa capable to adapt to urban landscapes, bats are particularly ubiquitous. Brazil has one of the world's largest diversity of bat species and one of the highest urbanization rates of the world. Yet, few studies have synthesized the biology of bats in urban environments, especially in Brazil. To fill this gap, we systematically reviewed the published scientific literature on the bat fauna found in urban areas of Brazil. The knowledge of urban bats is still incipient and heterogeneously spatially distributed, mostly concentrated in the southeastern region of the country. The assembled list of 84 urban species, of which nineteen are new species records for urban areas (including one new family), represents 47% of the bat richness registered in the country. Thirty-one bat species (37%) were captured exclusively inside forest fragments. Moreover, we provide information on the resources used within the urban matrix by summarizing the roosting sites for 38 bat species, as well as 31 plants consumed by at least twelve bat species. Regarding parasitological aspects, we listed eleven zoonotic parasites hosted by 27 bat species and discussed their potential to become a public health threat. Likewise, we considered the different features linked to urbanization, including impacts on immunity, body condition and susceptibility to acquiring parasites, as possible bat conservation issues. Finally, we defined an agenda for bat studies in urban areas of Brazil.
C1 [Nunes, Hannah; Cordeiro-Estrela, Pedro] Univ Fed Paraiba, Dept Sistemat & Ecol, Cidade Jardim Univ S-N, BR-58051900 Joao Pessoa, Paraiba, Brazil.
[Rocha, Fabiana Lopes] Univ Fed Paraiba, Dept Engn & Meio Ambiente, Campus 4 Litoral Norte,Rua Mangueira S-N, BR-58297000 Rio Tinto, Paraiba, Brazil.
C3 Universidade Federal da Paraiba; Universidade Federal da Paraiba
RP Nunes, H (corresponding author), Univ Fed Paraiba, Dept Sistemat & Ecol, Cidade Jardim Univ S-N, BR-58051900 Joao Pessoa, Paraiba, Brazil.
EM hannahlarissa@gmail.com
RI Rocha, Fabiana/J-5471-2015; Cordeiro-Estrela, Pedro/A-2913-2015; Nunes,
Hannah/K-1420-2014
OI Nunes, Hannah/0000-0001-9190-5881; Rocha, Fabiana
Lopes/0000-0003-0819-6290; Cordeiro-Estrela, Pedro/
0000-0003-3383-571X
FU CAPES (Coordenacao de Aperfeicoamento de Pessoal de Nivel

Superior);
PNPD scholarship; Ministry of Health through PPSUS (Programa de Pesquisa
para o SUS - Edital - PPSUS/FAPESQ/MS/CNPq) [EFP_ 00008705]; Ministry of
Science and Technology (MCT-CNPq) through Programa de Pesquisa em
Biodiversidade (PPBIO Mata Atlantica); Rede BioM. A. Inventarios:
Padroes de diversidade, biogeografia e endemismo de especies de
mamiferos, aves, anfibios, drosofilas e parasitos na Mata Atlantica
[CNPq: 457524/2012-0]
FX We thank CAPES (Coordenacao de Aperfeicoamento de Pessoal de Nivel
Superior) for the PhD scholarship to Hannah Nunes and for the PNPD
scholarship to Fabiana Rocha. The Ministry of Health provided financial
support through the PPSUS program (Programa de Pesquisa para o SUS -
Edital 01/2013 - PPSUS/FAPESQ/MS/CNPq, EFP_ 00008705). The Ministry of
Science and Technology (MCT-CNPq) provided support through Programa de
Pesquisa em Biodiversidade (PPBIO Mata Atlantica), Rede BioM. A.
Inventarios: Padroes de diversidade, biogeografia e endemismo de
especies de mamiferos, aves, anfibios, drosofilas e parasitos na Mata
Atlantica (Processo CNPq: 457524/2012-0). We are also grateful to
Anderson Feijo, Emmanuel Messias Vilar, Geadelande Delgado and Mayara
Beltrao, who provided valuable comments that improved the manuscript.
CR Aguiar LMS, 2014, ZOOLOGIA-CURITIBA, V31, P223
Albuquerque Paloma, 2012, Revista Biociencias (Taubate), V18, P5
[Anonymous], 2014, World Urbanization Prospects: The 2014 Revision, P1
[Anonymous], 2007, MAMMALS S AM
Araujo Perini Fernando, 2003, Chiroptera Neotropical, V9, P169
Avila-Flores R, 2005, J MAMMAL, V86, P1193, DOI 10.1644/04-MAMM-A-085R1.1
Barros Ronald Souza Monteiro de, 2006, Biota Neotrop., V6, P0, DOI 10.1590/S1676-06032006000100012
Battin J, 2004, CONSERV BIOL, V18, P1482, DOI 10.1111/j.1523-1739.2004.00417.x
Bello-Gutiérrez J, 2010, J WILDLIFE DIS, V46, P1000, DOI 10.7589/0090-3558-46.3.1000
Bergallo HG, 2003, BIOTROPICA, V35, P278, DOI 10.1111/j.1744-7429.2003.tb00286.x
Bernard E, 2003, BIOTROPICA, V35, P262, DOI 10.1111/j.1744-7429.2003.tb00285.x
Bernardi Itiberê Piaia, 2009, Biota Neotrop., V9, P349, DOI 10.1590/S1676-06032009000300029
Bianchini E, 2015, BRAZ J BIOL, V75, pS206, DOI 10.1590/1519-6984.10614

Blehert DS, 2009, SCIENCE, V323, P227, DOI 10.1126/science.1163874
Bobrowiec Paulo Estefano D., 2010, Chiroptera Neotropical, V16, P595
Bredt Angelika, 1996, Chiroptera Neotropical, V2, P54
Brooks TM, 2006, SCIENCE, V313, P58, DOI 10.1126/science.1127609
Buczkowski G, 2012, PLOS ONE, V7, DOI 10.1371/journal.pone.0041729
Calisher CH, 2006, CLIN MICROBIOL REV, V19, P531, DOI 10.1128/CMR.00017-06
Campos GS, 2015, EMERG INFECT DIS, V21, P1885, DOI 10.3201/eid2110.150847
Coleman JL, 2012, URBAN ECOSYST, V15, P87, DOI 10.1007/s11252-011-0181-8
Constantine D.G., 1988, P491
Costa Luciana de Moraes, 2012, Pap. Avulsos Zool. (São Paulo), V52, P7, DOI 10.1590/S0031-10492012000200001
Cottontail VM, 2009, PARASITOLOGY, V136, P1133, DOI 10.1017/S0031182009990485
Czech B, 2000, BIOSCIENCE, V50, P593, DOI 10.1641/0006-3568(2000)050[0593:EAACOS]2.0.CO;2
Dar PA, 2014, CONTEMP PROBL ECOL+, V7, P123, DOI 10.1134/S1995425514020103
Ramírez JD, 2014, INFECT GENET EVOL, V22, P250, DOI 10.1016/j.meegid.2013.06.022
De Lima H, 2008, MEM I OSWALDO CRUZ, V103, P412, DOI 10.1590/S0074-02762008000400018
Dietrich M, 2015, PLOS PATHOG, V11, DOI 10.1371/journal.ppat.1005176
Dietrich M, 2015, ENVIRON MICROBIOL, V17, P4280, DOI 10.1111/1462-2920.12766
Rabinovich JE, 2011, MEM I OSWALDO CRUZ, V106, P479, DOI 10.1590/S0074-02762011000400016
Esb?rard C.E.L., 1999, REV BRAS MED VET, V21, P17
Esberard C. E. L., 2003, REV BRASILEIRA ZOOCI, V5, P189
Esbérard CEL, 2014, IHERINGIA SER ZOOL, V104, P59, DOI 10.1590/1678-4766201410415969
Eva H. D., 2002, 20159 EUR EN EUR COM
Ferreira Alvino Pedrosa, 2013, Check List, V9, P793
Bessa TAF, 2010, AM J TROP MED HYG, V82, P315, DOI 10.4269/ajtmh.2010.09-0227
Gaisler J, 1998, J ZOOL, V244, P439, DOI 10.1111/j.1469-7998.1998.tb00048.x
Garden J, 2006, AUSTRAL ECOL, V31, P126, DOI 10.1111/j.1442-9993.2006.01578.x
Gargas A, 2009, MYCOTAXON, V108, P147, DOI 10.5248/108.147
GEGGIE JF, 1985, CAN J ZOOL, V63, P263, DOI 10.1139/z85-040
Gonsalves L, 2013, PLOS ONE, V8, DOI 10.1371/journal.pone.0077183
Hoekstra JM, 2005, ECOL LETT, V8, P23, DOI 10.1111/j.1461-0248.2004.00686.x
Jung K, 2010, J MAMMAL, V91, P144, DOI [10.1644/08-MAMM-A-313R.1, 10.1644/08-MAMM-A-313R.1.]
Kalko Elisabeth K.V., 1996, P503, DOI 10.1016/B978-012178075-3/50017-9

Kasso M., 2013, ISRN BIODIVERSITY, P1, DOI [10.1155/2013/187415, DOI 10.1155/2013/187415]
KUNZ TH, 1995, OECOLOGIA, V101, P407, DOI 10.1007/BF00329419
Kunz TH, 2003, BAT ECOLOGY, P3
Kunz TH, 2011, ANN NY ACAD SCI, V1223, P1, DOI 10.1111/j.1749-6632.2011.06004.x
Kuzmin Ivan V, 2011, Emerg Health Threats J, V4, P7159, DOI 10.3402/ehtj.v4i0.7159
Lampo M, 2000, AM J TROP MED HYG, V62, P718, DOI 10.4269/ajtmh.2000.62.718
Leroy EM, 2005, NATURE, V438, P575, DOI 10.1038/438575a
Leroy EM, 2009, VECTOR-BORNE ZOONOT, V9, P723, DOI 10.1089/vbz.2008.0167
Lima IP, 2008, ECOLOGIA DE MORCEGOS, P71
Luis AD, 2013, P ROY SOC B-BIOL SCI, V280, DOI 10.1098/rspb.2012.2753
Maia-Elkhoury ANS, 2008, CAD SAUDE PUBLICA, V24, P2941, DOI 10.1590/S0102-311X2008001200024
Marcili A, 2013, J PARASITOL, V99, P1071, DOI 10.1645/12-156.1
McDonald RI, 2006, BIOL INVASIONS, V8, P1049, DOI 10.1007/s10530-005-5227-5
McKinney ML, 2006, BIOL CONSERV, V127, P247, DOI 10.1016/j.biocon.2005.09.005
McKinney ML, 2002, BIOSCIENCE, V52, P883, DOI 10.1641/0006-3568(2002)052[0883:UBAC]2.0.CO;2
Newbold T, 2014, P ROY SOC B-BIOL SCI, V281, DOI 10.1098/rspb.2014.1371
Nogueira Marcelo Rodrigues, 2014, Check List, V10, P808
Nunes Hannah Larissa de F.L., 2013, Check List, V9, P436
Nunes Mariane de Souza, 2007, Revista Brasileira de Zoociencias, V9, P213
OIE, 2016, OIE WORLDW MON SYST
Pacheco Susi M., 2010, Chiroptera Neotropical, V16, P629
Parlos J.A., 2014, OCCAS PAP MUS TEXAS, V320, P1
Pereira Adriana Fernandes, 2009, Revista Brasileira de Zoociencias, V11, P19
Reis NR, 2006, PARANA REV BRASILEIR, V19, P739, DOI DOI 10.1590/S0101-81752002000300011
Reiskind MH, 2009, J MED ENTOMOL, V46, P1037, DOI 10.1603/033.046.0510
Roche B, 2013, AM NAT, V181, P1, DOI 10.1086/668591
Roque Andre Luiz R., 2014, International Journal for Parasitology Parasites and Wildlife, V3, P251, DOI 10.1016/j.ijppaw.2014.08.004
Russo D, 2015, MAMM BIOL, V80, P205, DOI 10.1016/j.mambio.2014.10.003
Sartore ER, 2013, MAMMALIA, V77, P141, DOI 10.1515/mammalia-2012-0055
Scarano FR, 2015, BIODIVERS CONSERV, V24, P2319, DOI 10.1007/s10531-015-0972-y
Seto K.C., 2013, GLOBAL OUTLOOK URBAN, P1, DOI DOI 10.1007/978-94-007-7088-1_1
Shochat E, 2010, BIOSCIENCE, V60, P199, DOI 10.1525/bio.2010.60.3.6
Sih A, 2011, EVOL APPL, V4, P367, DOI 10.1111/

j.1752-4571.2010.00166.x
Simmons NB, 1998, B AM MUS NAT HIST, P1
Teixeira CP, 2006, P ROY SOC B-BIOL SCI, V273, P1007, DOI 10.1098/rspb.2005.3386
Tomassini A, 2014, J BIOGEOGR, V41, P944, DOI 10.1111/jbi.12248
Velazco PM, 2010, ZOOL J LINN SOC-LOND, V159, P785, DOI 10.1111/j.1096-3642.2009.00610.x
Velazco PM, 2008, MOL PHYLOGENET EVOL, V49, P749, DOI 10.1016/j.ympev.2008.09.015
Vercoza FC, 2012, NATUREZA, V10, P7
de Araújo MLVS, 2016, URBAN ECOSYST, V19, P287, DOI 10.1007/s11252-015-0487-z
Vilar Emmanuel Messias, 2015, Check List, V11, P1503
Voss RS, 1996, B AM MUS NAT HIST, P3
Walsh AL, 1996, J APPL ECOL, V33, P519, DOI 10.2307/2404981
Warnecke L, 2012, P NATL ACAD SCI USA, V109, P6999, DOI 10.1073/pnas.1200374109
Wood JLN, 2012, PHILOS T R SOC B, V367, P2881, DOI 10.1098/rstb.2012.0228
NR 90
TC 54
Z9 62
U1 0
U2 66
PU SPRINGER
PI DORDRECHT
PA VAN GODEWIJCKSTRAAT 30, 3311 GZ DORDRECHT, NETHERLANDS
SN 1083-8155
EI 1573-1642
J9 URBAN ECOSYST
JI Urban Ecosyst.
PD AUG
PY 2017
VL 20
IS 4
BP 953
EP 969
DI 10.1007/s11252-016-0632-3
PG 17
WC Biodiversity Conservation; Ecology; Environmental Sciences; Urban Studies
WE Science Citation Index Expanded (SCI-EXPANDED)
SC Biodiversity & Conservation; Environmental Sciences & Ecology; Urban
Studies
GA FC3YY
UT WOS:000406776700020
PM 32214783
OA Green Published
DA 2024-10-21
ER

PT J
AU HONEYCUTT, RL

SARICH, VM
AF HONEYCUTT, RL
SARICH, VM
TI ALBUMIN EVOLUTION AND SUBFAMILIAL RELATIONSHIPS AMONG NEW-WORLD
LEAF-NOSED BATS (FAMILY PHYLLOSTOMIDAE)
SO JOURNAL OF MAMMALOGY
LA English
DT Article
AB Subfamilial relationships within the New World leaf-nosed bats, family Phyllostomidae, and intergeneric relationships within the subfamily Phyllostominae are examined with the use of albumin immunology. Phylogenetic relationships indicated by the albumin data show a high level of congruence with those suggested by both anatomical and chromosomal studies. Immunological data indicate that the subfamily Phyllostominae is a paraphyletic unit containing lineages from which other phyllostomid subfamilies have been derived. The albumin data further suggest that diversification within the Phyllostomidae proceeded rather rapidly, with three and possibly four extant lineages originating as part of the basal phyllostomid radiation. The phylogenetic associations of currently recognized subfamilies and genera revealed by the immunological data together with chromosomal and morphological data suggest the need for a taxonomic revision of the family Phyllostomidae.
C1 HARVARD UNIV, MUSEUM COMPARAT ZOOL, CAMBRIDGE, MA 02138 USA.
UNIV CALIF BERKELEY, DEPT BIOCHEM, BERKELEY, CA 94720 USA.
UNIV CALIF BERKELEY, DEPT ANTHROPOL, BERKELEY, CA 94720 USA.
C3 Harvard University; University of California System; University of
California Berkeley; University of California System; University of
California Berkeley
RP HARVARD UNIV, DEPT ORGANISM & EVOLUT BIOL, 26 OXFORD ST, CAMBRIDGE, MA 02138 USA.
CR [Anonymous], 1972, MISCELLANEOUS PUBLIC
ARNOW D, 1982, J CHEM PHYS, V77, P1
BAKER RJ, 1979, J MAMMAL, V60, P364, DOI 10.2307/1379808
BAKER RJ, 1980, SYST ZOOL, V29, P239, DOI 10.2307/2412660
BAKER RJ, 1981, J MAMMAL, V62, P665, DOI 10.2307/1380587
BAKER RJ, 1979, EVOLUTION, V33, P220, DOI 10.1111/j.1558-5646.1979.tb04676.x
Champion A.B., 1974, P397
CRONIN JE, 1975, J HUM EVOL, V5, P357
FARRIS J S, 1985, Cladistics, V1, P67, DOI 10.1111/j.1096-0031.1985.tb00411.x
FARRIS JS, 1972, AM NAT, V106, P645, DOI 10.1086/282802
FARRIS JS, 1979, SYST ZOOL, V28, P627, DOI 10.2307/2412573
FARRIS JS, 1981, ADV CLADISTICS, P3
FITCH WM, 1967, SCIENCE, V155, P279, DOI 10.1126/science.155.3760.279
FORMAN GL, 1968, SYST ZOOL, V17, P417, DOI 10.2307/2412040
Gardner A.L., 1977, BIOL BATS NEW WORLD, V16, P293
GARDNER AL, 1977, SYST ZOOL, V26, P300, DOI 10.2307/2412677
Griffiths T.A., 1982, Am. Mus. Novit, V2742, P1, DOI DOI 10.1097/
HAIDUK MW, 1982, SYST ZOOL, V31, P252, DOI 10.2307/2413231

HONEYCUTT RL, 1981, J MAMMAL, V62, P805, DOI 10.2307/1380602
HOOD CS, 1982, SYST ZOOL, V31, P241, DOI 10.2307/2413230
JONES ND, 1976, MATH SYST THEORY, V10, P1, DOI 10.1007/BF01683259
MAXSON LR, 1974, SCIENCE, V185, P66, DOI 10.1126/science.185.4145.66
Miller G.S., 1907, P US NATL MUS, V57, pxvii + 282, DOI [https://doi.org/10.5962/bhl.title.16306, DOI 10.5479/SI.00963801.57-2311.205]
PATTON JC, 1978, SYST ZOOL, V27, P449, DOI 10.2307/2412927
Phillips C. J., 1971, Musc Publ Mus nat Hist Univ Kans, VNo. 54, P1
PRAGER EM, 1978, J MOL EVOL, V11, P129, DOI 10.1007/BF01733889
PRAGER EM, 1971, J BIOL CHEM, V246, P7010
Sarich V.M, 1976, P141
SARICH VM, 1967, P NATL ACAD SCI USA, V58, P142, DOI 10.1073/pnas.58.1.142
SARICH VM, 1966, SCIENCE, V154, P1563, DOI 10.1126/science.154.3756.1563
SARICH VM, 1969, SYST ZOOL, V18, P286, DOI 10.2307/2412325
Slaughter B.H., 1970, P51
SMITH J. D., 1976, SPECIAL PUBLICATIONS, V10, p[1, 49]
SMITH JD, 1984, SYST ZOOL, V33, P435, DOI 10.2307/2413096
SWOFFORD DL, 1981, ADV CLADISTICS, P25
WALTON D W, 1968, Journal of the Graduate Research Center Southern Methodist University, V37, P1
WILSON AC, 1977, ANNU REV BIOCHEM, V46, P573, DOI 10.1146/annurev.bi.46.070177.003041
NR 37
TC 21
Z9 23
U1 0
U2 3
PU OXFORD UNIV PRESS INC
PI CARY
PA JOURNALS DEPT, 2001 EVANS RD, CARY, NC 27513 USA
SN 0022-2372
EI 1545-1542
J9 J MAMMAL
JI J. Mammal.
PD AUG 28
PY 1987
VL 68
IS 3
BP 508
EP 517
DI 10.2307/1381587
PG 10
WC Zoology
WE Science Citation Index Expanded (SCI-EXPANDED)
SC Zoology
GA J8461
UT WOS:A1987J846100005
DA 2024-10-21
ER

PT J
AU Costantini, D
   Schad, J
   Czirják, GA
   Voigt, CC
AF Costantini, David
   Schad, Julia
   Czirjak, Gabor a.
   Voigt, Christian C.
TI Oxidative damage varies in response to bacterial, fungal and viral
   antigen challenges in bats
SO JOURNAL OF EXPERIMENTAL BIOLOGY
LA English
DT Article
DE Antioxidants; Immunity; Infection; Oxidative stress; Vertebrates
ID ANTIOXIDANT STATUS; IMMUNE-RESPONSE; TRADE-OFFS; STRESS; DISEASE;
   SUPEROXIDE; IMMUNOLOGY; INFECTION; LPS
AB Myotis myotis
C1 [Costantini, David] Tuscia Univ, Dept Ecol & Biol Sci, Largo Univ Snc, I-01100 Viterbo, Italy.
   [Schad, Julia; Czirjak, Gabor a.; Voigt, Christian C.] Leibniz Inst Zoo & Wildlife Res, Alfred Kowalke Str 17, D-10315 Berlin, Germany.
C3 Tuscia University; Leibniz Institut fur Zoo und Wildtierforschung
RP Costantini, D (corresponding author), Tuscia Univ, Dept Ecol & Biol Sci, Largo Univ Snc, I-01100 Viterbo, Italy.; Voigt, CC (corresponding author), Leibniz Inst Zoo & Wildlife Res, Alfred Kowalke Str 17, D-10315 Berlin, Germany.
EM david.costantini@unitus.it; voigt@izw-berlin.de
RI Czirják, Gábor/F-5440-2011; Costantini, David/C-6006-2013
OI Voigt, Christian/0000-0002-0706-3974; Costantini,
   David/0000-0002-8140-8790; Czirjak, Gabor Arpad/0000-0001-9488-0069
FU Leibniz Institute for Zoo and Wildlife Research; German Research
   Foundation; DFG; [GO2091/2-1]
FX This work was supported by institutional funds of the Leibniz Institute
   for Zoo and Wildlife Research and the German Research Foundation (DFG
   Priority Programme 1596) . Funding for the research station in Tabachka
   was provided by the DFG to Holger R. Goerlitz (Emmy Noether Program
   GO2091/2-1) .
CR Ahn M, 2019, NAT MICROBIOL, V4, P789, DOI 10.1038/s41564-019-0371-3
   Arsenault D, 2014, BRAIN BEHAV IMMUN, V38, P77, DOI 10.1016/j.bbi.2013.12.016
   Babior BM, 2004, CURR OPIN IMMUNOL, V16, P42, DOI 10.1016/j.coi.2003.12.001
   BABIOR BM, 1973, J CLIN INVEST, V52, P741, DOI 10.1172/JCI107236
   Baker ML, 2013, ZOONOSES PUBLIC HLTH, V60, P104, DOI 10.1111/

j.1863-2378.2012.01528.x
Brook CE, 2015, TRENDS MICROBIOL, V23, P172, DOI 10.1016/j.tim.2014.12.004
Clayton E, 2020, FRONT CELL INFECT MI, V10, DOI 10.3389/fcimb.2020.527921
Cohen A, 2007, COMP BIOCHEM PHYS B, V147, P110, DOI 10.1016/j.cbpb.2006.12.015
Coon CAC, 2011, AM J PHYSIOL-REG I, V300, pR1418, DOI 10.1152/ajpregu.00187.2010
Costantini, 2014, OXIDATIVE STRESS HOR, DOI DOI 10.1007/978-3-642-54663-1
Costantini D, 2022, CONSERV PHYSIOL, V10, DOI 10.1093/conphys/coac028
Costantini D, 2022, CONSERV PHYSIOL, V10, DOI 10.1093/conphys/coac018
Costantini D, 2006, COMP BIOCHEM PHYS A, V145, P137, DOI 10.1016/j.cbpa.2006.06.002
Costantini D, 2019, J EXP BIOL, V222, DOI 10.1242/jeb.194688
Delhaye J, 2016, MALARIA J, V15, DOI 10.1186/s12936-016-1579-9
Dokka S, 2001, AM J PHYSIOL-LUNG C, V280, pL1196, DOI 10.1152/ajplung.2001.280.6.L1196
Fritze M, 2021, DEV COMP IMMUNOL, V119, DOI 10.1016/j.dci.2021.104017
Fritze M, 2019, BIOL OPEN, V8, DOI 10.1242/bio.046078
Georgieva NV, 2006, VET J, V172, P488, DOI 10.1016/j.tvjl.2005.07.016
Gombart AF, 2020, NUTRIENTS, V12, DOI 10.3390/nu12010236
Gorbunova V, 2020, CELL METAB, V32, P31, DOI 10.1016/j.cmet.2020.06.013
Guito JC, 2021, CURR BIOL, V31, P257, DOI 10.1016/j.cub.2020.10.015
Halliwell B., 2015, Free radicals in biology and medicine
Hasselquist D, 2012, ANIM BEHAV, V83, P1303, DOI 10.1016/j.anbehav.2012.03.025
He YB, 2021, SCI REP-UK, V11, DOI 10.1038/s41598-021-89777-1
Huang ZX, 2019, NAT ECOL EVOL, V3, P1110, DOI 10.1038/s41559-019-0913-3
Kacprzyk J, 2017, ACTA CHIROPTEROL, V19, P219, DOI [10.3161/15081109ACC2017.19.2.001, 10.3161/15081109acc2017.19.2.001]
Klasing Kirk C., 2004, Acta Zoologica Sinica, V50, P961
Koinarski V, 2005, REV MED VET-TOULOUSE, V156, P498
Lips KR, 2006, P NATL ACAD SCI USA, V103, P3165, DOI 10.1073/pnas.0506889103
Lochmiller RL, 2000, OIKOS, V88, P87, DOI 10.1034/j.1600-0706.2000.880110.x
Luis AD, 2013, P ROY SOC B-BIOL SCI, V280, DOI 10.1098/rspb.2012.2753
Maizels RM, 2013, NAT IMMUNOL, V14, P879, DOI 10.1038/ni.2643
Marri V, 2015, COMP BIOCHEM PHYS A, V179, P192, DOI 10.1016/j.cbpa.2014.10.013
McCallum H, 2009, ECOLOGY, V90, P3379, DOI 10.1890/08-1763.1
Mollentze N, 2020, P NATL ACAD SCI USA, V117, P9423, DOI 10.1073/pnas.1919176117
Santillán DDM, 2021, MOL ECOL, V30, P6449, DOI 10.1111/mec.16027

Nathan C, 2013, NAT REV IMMUNOL, V13, P349, DOI 10.1038/nri3423
Pasková V, 2008, SCI TOTAL ENVIRON, V398, P34, DOI 10.1016/j.scitotenv.2008.03.001
Periasamy P, 2019, FRONT IMMUNOL, V10, DOI 10.3389/fimmu.2019.00489
Schneeberger K, 2013, J EXP BIOL, V216, P4514, DOI 10.1242/jeb.090837
Segal AW, 2005, ANNU REV IMMUNOL, V23, P197, DOI 10.1146/annurev.immunol.23.021704.115653
Seltmann A, 2022, SCI REP-UK, V12, DOI 10.1038/s41598-022-18240-6
Sheldon BC, 1996, TRENDS ECOL EVOL, V11, P317, DOI 10.1016/0169-5347(96)10039-2
Sorci G, 2009, PHILOS T R SOC B, V364, P71, DOI 10.1098/rstb.2008.0151
Tobler M, 2015, PLOS ONE, V10, DOI 10.1371/journal.pone.0126155
Tobler M, 2011, BIOL LETTERS, V7, P906, DOI 10.1098/rsbl.2011.0350
Triana-Llanos C, 2019, BIOLOGIA, V74, P667, DOI 10.2478/s11756-019-00204-3
van de Crommenacker J., 2011, PhD thesis
van de Crommenacker J, 2010, J EXP BIOL, V213, P3527, DOI 10.1242/jeb.045591
Voigt CC, 2020, SCI REP-UK, V10, DOI 10.1038/s41598-020-74473-3
Wibbelt G, 2010, BIOL LETTERS, V6, P438, DOI 10.1098/rsbl.2010.0267
Wilkinson GS, 2021, NAT COMMUN, V12, DOI 10.1038/s41467-021-21900-2
Wu L, 2017, FISH SHELLFISH IMMUN, V71, P28, DOI 10.1016/j.fsi.2017.09.072
NR 54
TC 1
Z9 1
U1 3
U2 8
PU COMPANY BIOLOGISTS LTD
PI CAMBRIDGE
PA BIDDER BUILDING, STATION RD, HISTON, CAMBRIDGE CB24 9LF, ENGLAND
SN 0022-0949
EI 1477-9145
J9 J EXP BIOL
JI J. Exp. Biol.
PD NOV
PY 2023
VL 226
IS 21
AR jeb246332
DI 10.1242/jeb.246332
PG 8
WC Biology; Zoology
WE Science Citation Index Expanded (SCI-EXPANDED)
SC Life Sciences & Biomedicine - Other Topics; Zoology
GA CO2U5
UT WOS:001126131800009
PM 37823235

DA 2024-10-21
ER

PT J
AU Ruhs, EC
   Becker, DJ
   Oakey, SJ
   Ogunsina, O
   Fenton, MB
   Simmons, NB
   Martin, LB
   Downs, CJ
AF Ruhs, Emily Cornelius
   Becker, Daniel J.
   Oakey, Samantha J.
   Ogunsina, Ololade
   Fenton, M. Brock
   Simmons, Nancy B.
   Martin, Lynn B.
   Downs, Cynthia J.
TI Body size affects immune cell proportions in birds and non-volant
   mammals, but not bats
SO JOURNAL OF EXPERIMENTAL BIOLOGY
LA English
DT Article
DE Immunology; Leukocytes; Life history; Flight; Allometry;
Chiroptera;
   Scaling
ID LIFE-HISTORY; LONGEVITY; FLIGHT; EVOLUTION; STRESS; VIRUS;
RESERVOIRS;
   MIGRATION; RESPONSES; DEFENSES
AB Powered flight has evolved several times in vertebrates and
constrains morphology and physiology in ways that likely have shaped
how organisms cope with infections. Some of these constraints
probably have impacts on aspects of immunology, such that larger
fliers might prioritize risk reduction and safety. Addressing how
the evolution of flight may have driven relationships between body
size and immunity could be particularly informative for
understanding the propensity of some taxa to harbor many virulent
and sometimes zoonotic pathogens without showing clinical disease.
Here, we used a comparative framework to quantify scaling
relationships between body mass and the proportions of two types of
white blood cells - lymphocytes and granulocytes (neutrophils/
heterophils) - across 63 bat species, 400 bird species and 251
nonvolant mammal species. By using phylogenetically informed
statistical models on field-collected data from wild Neotropical
bats and from captive bats, non-volant mammals and birds, we show
that lymphocyte and neutrophil proportions do not vary
systematically with body mass among bats. In contrast, larger birds
and non-volant mammals have disproportionately higher granulocyte
proportions than expected for their body size. Our inability to
distinguish bat lymphocyte scaling from birds and bat granulocyte
scaling from all other taxa suggests there may be other ecological
explanations (i.e. not flight related) for the cell proportion

scaling patterns. Future comparative studies of wild bats, birds and non-volant mammals of similar body mass should aim to further differentiate evolutionary effects and other aspects of life history on immune defense and its role in the tolerance of (zoonotic) infections.
C1 [Ruhs, Emily Cornelius; Oakey, Samantha J.; Ogunsina, Ololade; Martin, Lynn B.] Univ S Florida, Global Hlth & Infect Dis Res, Tampa, FL 33612 USA.
[Becker, Daniel J.] Univ Oklahoma, Dept Biol, Norman, OK 73019 USA.
[Fenton, M. Brock] Western Univ, Dept Biol, London, ON N6A 5B7, Canada.
[Simmons, Nancy B.] Amer Museum Nat Hist, Div Vertebrate Zool, Dept Mammal, New York, NY 10024 USA.
[Downs, Cynthia J.] SUNY Coll Environm Sci & Forestry, Dept Environm & Forest Biol, Syracuse, NY 13210 USA.
C3 State University System of Florida; University of South Florida; University of Oklahoma System; University of Oklahoma - Norman; Western University (University of Western Ontario); American Museum of Natural History (AMNH); State University of New York (SUNY) System; State University of New York (SUNY) College of Environmental Science & Forestry
RP Ruhs, EC (corresponding author), Univ S Florida, Global Hlth & Infect Dis Res, Tampa, FL 33612 USA.
EM ecruhs@usf.edu
RI Martin, Lynn/C-2037-2009; Becker, Daniel/A-6466-2014; Downs, Cynthia/K-3758-2012
OI Becker, Daniel/0000-0003-4315-8628; Cornelius Ruhs, Emily/0000-0002-0546-6593; Simmons, Nancy B./0000-0001-8807-7499; Downs, Cynthia/0000-0001-9435-0623
FU Achievement Rewards for College Scientists Foundation; Explorers Club; American Museum of Natural History (Theodore Roosevelt Memorial Fund); National Science Foundation [IOS 1656618, IOS 1656551]
FX This work was funded by the Achievement Rewards for College Scientists Foundation and The Explorers Club (D.J.B.), American Museum of Natural History (Theodore Roosevelt Memorial Fund to D.J.B., Taxonomic Mammalogy Fund to N.B. S.), and National Science Foundation (IOS 1656618 to L.B.M., IOS 1656551 to C.J.D.).
CR Ahn M, 2019, NAT MICROBIOL, V4, P789, DOI 10.1038/s41564-019-0371-3
Amman BR, 2015, J WILDLIFE DIS, V51, P113, DOI 10.7589/2014-08-198
Baker ML, 2013, ZOONOSES PUBLIC HLTH, V60, P104, DOI 10.1111/j.1863-2378.2012.01528.x
Banyard AC, 2011, ADV VIRUS RES, V79, P239, DOI 10.1016/B978-0-12-387040-7.00012-3

Becker DJ, 2021, J APPL ECOL, V58, P879, DOI 10.1111/1365-2664.13809
Becker DJ, 2021, TRANSBOUND EMERG DIS, V68, P870, DOI 10.1111/tbed.13754
Becker DJ, 2020, J ANIM ECOL, V89, P972, DOI 10.1111/1365-2656.13166
Becker DJ, 2019, PHYSIOL BIOCHEM ZOOL, V92, P37, DOI 10.1086/701069
Brace AJ, 2017, J EXP ZOOL PART A, V327, P254, DOI 10.1002/jez.2084
Brook CE, 2020, ELIFE, V9, DOI 10.7554/eLife.48401
Brook CE, 2015, TRENDS MICROBIOL, V23, P172, DOI 10.1016/j.tim.2014.12.004
Brown JH, 2004, ECOLOGY, V85, P1771, DOI 10.1890/03-9000
Brown JH, 2000, SFI S SCI C, P1
Brunet-Rossinni AK, 2004, MECH AGEING DEV, V125, P11, DOI 10.1016/j.mad.2003.09.003
Calder WA, 1984, SIZE FUNCTION LIFE H
Clayton Dale H., 2010, Open Ornithology Journal, V3, P41, DOI 10.2174/1874453201003010041
COHN M, 1990, IMMUNOL REV, V115, P7, DOI 10.1111/j.1600-065X.1990.tb00783.x
Davis AK, 2008, FUNCT ECOL, V22, P760, DOI 10.1111/j.1365-2435.2008.01467.x
Dingli D, 2006, PLOS ONE, V1, DOI 10.1371/journal.pone.0000002
DOBSON AP, 1986, TRENDS ECOL EVOL, V1, P11, DOI 10.1016/0169-5347(86)90060-1
Downs C. J., 2020, 242107 BIORXIV, DOI [10.1101/2020.09.04.242107, DOI 10.1101/2020.09.04.242107]
Downs CJ, 2020, AM NAT, V195, P107, DOI 10.1086/706235
Downs CJ, 2019, TRENDS PARASITOL, V35, P182, DOI 10.1016/j.pt.2018.12.002
Dumont ER, 2010, P ROY SOC B-BIOL SCI, V277, P2193, DOI 10.1098/rspb.2010.0117
Dunning JB, 2007, CRC HDB AVIAN BODY M
Feduccia A., 1999, The Origin and Evolution of Birds, V2nd
Fischer CP, 2019, CONSERV PHYSIOL, V7, DOI 10.1093/conphys/coz093
Gunnell GF, 2012, CAM STUD MORPHOL MOL, P1, DOI 10.1017/CBO9781139045599
Guth S, 2019, PHILOS T R SOC B, V374, DOI 10.1098/rstb.2019.0296
Hadfield JD, 2010, J STAT SOFTW, V33, P1, DOI 10.18637/jss.v033.i02
Halpin K, 2011, AM J TROP MED HYG, V85, P946, DOI 10.4269/ajtmh.2011.10-0567
Han BA, 2015, J ANIM ECOL, V84, P637, DOI 10.1111/1365-2656.12336
Harrison JF, 2017, TRENDS ECOL EVOL, V32, P653, DOI 10.1016/j.tree.2017.05.008
Hasselquist D, 2007, J EXP BIOL, V210, P1123, DOI 10.1242/jeb.02712
HATTEN BA, 1973, J EXP ZOOL, V186, P193, DOI 10.1002/jez.1401860210
Healy K, 2014, P ROY SOC B-BIOL SCI, V281, DOI 10.1098/rspb.2014.0298
Herrera JP, 2019, AM J PRIMATOL, V81, DOI 10.1002/ajp.22991

Herrera JP, 2018, ACTA CHIROPTEROL, V20, P147, DOI 10.3161/15081109ACC2018.20.1.011
Housworth EA, 2004, AM NAT, V163, P84, DOI 10.1086/380570
Ingala MR, 2018, MSPHERE, V3, DOI 10.1128/mSphere.00397-18
Johnstone CP, 2012, J COMP PHYSIOL B, V182, P861, DOI 10.1007/s00360-012-0656-9
Jones G, 2006, TRENDS ECOL EVOL, V21, P149, DOI 10.1016/j.tree.2006.01.001
Jones T., 1997, ANIMAL DIVERSITY WEB
Kacprzyk J, 2017, ACTA CHIROPTEROL, V19, P219, DOI [10.3161/15081109ACC2017.19.2.001, 10.3161/15081109acc2017.19.2.001]
Kerth G, 2008, BIOSCIENCE, V58, P737, DOI 10.1641/B580810
KLEIBER MAX, 1932, HILGARDIA, V6, P315
Kunz T.H., 1982, P1
Lanier LL, 2013, NAT REV IMMUNOL, V13, P73, DOI 10.1038/nri3389
Lee KA, 2006, INTEGR COMP BIOL, V46, P1000, DOI 10.1093/icb/icl049
Letunic I, 2015, phyloT: a phylogenetic tree generator
Levesque DL, 2021, J WILDLIFE DIS, V57, P238, DOI 10.7589/JWD-D-20-00079
Li WD, 2005, SCIENCE, V310, P676, DOI 10.1126/science.1118391
Luis AD, 2013, P ROY SOC B-BIOL SCI, V280, DOI 10.1098/rspb.2012.2753
Maurer BA, 2004, EVOL ECOL RES, V6, P783
McGuire LP, 2009, J MAMMAL, V90, P1290, DOI 10.1644/09-MAMM-S-084R.1
Minias P, 2017, EVOLUTION, V71, P432, DOI 10.1111/evo.13142
Mollentze N, 2020, P NATL ACAD SCI USA, V117, P9423, DOI 10.1073/pnas.1919176117
Muijres FT, 2012, PLOS ONE, V7, DOI 10.1371/journal.pone.0037335
Munshi-South J, 2010, AGEING RES REV, V9, P12, DOI 10.1016/j.arr.2009.07.006
Nakagawa S, 2013, METHODS ECOL EVOL, V4, P133, DOI 10.1111/j.2041-210x.2012.00261.x
Nebel S, 2012, J EXP BIOL, V215, P272, DOI 10.1242/jeb.057885
O'Shea TJ, 2014, EMERG INFECT DIS, V20, P741, DOI 10.3201/eid2005.130539
Paradis E, 2004, BIOINFORMATICS, V20, P289, DOI [10.1093/bioinformatics/btg412, 10.1093/bioinformatics/bty633]
Parr Cynthia S, 2014, Biodivers Data J, pe1079, DOI 10.3897/BDJ.2.e1079
Pavlovich SS, 2018, CELL, V173, P1098, DOI 10.1016/j.cell.2018.03.070
RAYNER JMV, 1988, BIOL J LINN SOC, V34, P269, DOI 10.1111/j.1095-8312.1988.tb01963.x
Reid FA., 1997, FIELD GUIDE MAMMALS, P334
Richards SA, 2005, ECOLOGY, V86, P2805, DOI 10.1890/05-0074
Ruhs E. C., 2020, IMPACTS BODY MASS IM
Salmon AB, 2009, FASEB J, V23, P2317, DOI 10.1096/fj.08-122523
Schmidt-Nielsen K., 1984, SCALING WHY IS ANIMA, DOI 10.1017/CBO9781139167826
SCHMIDTNIELSEN K, 1972, SCIENCE, V177, P222, DOI 10.1126/science.177.4045.222
Schneeberger K, 2013, PLOS ONE, V8, DOI 10.1371/

journal.pone.0054023
Shaw AE, 2017, PLOS BIOL, V15, DOI 10.1371/journal.pbio.2004086
Sikes RS, 2011, J MAMMAL, V92, P235, DOI 10.1644/10-MAMM-F-355.1
Simmons N.B., 2020, Bat species of the world: A taxonomic and geographic database
SMITH JA, 1994, J LEUKOCYTE BIOL, V56, P672, DOI 10.1002/jlb.56.6.672
STANLEY SM, 1973, EVOLUTION, V27, P1, DOI 10.1111/j.1558-5646.1973.tb05912.x
Stockmaier S, 2015, BIOL LETTERS, V11, DOI 10.1098/rsbl.2015.0576
Swartz S., 2007, AER SCI M 45 AIAA AE
Tacutu R, 2013, NUCLEIC ACIDS RES, V41, pD1027, DOI 10.1093/nar/gks1155
THOMAS SP, 1975, J EXP BIOL, V63, P273
Tian JD, 2015, FUNCT ECOL, V29, P823, DOI 10.1111/1365-2435.12394
Tian XD, 2006, BIOINSPIR BIOMIM, V1, pS10, DOI 10.1088/1748-3182/1/4/S02
Viney M.E., 2014, Eco-immunology: Evolutive Aspects and Future Perspectives, P1
Voigt CC, 2020, SCI REP-UK, V10, DOI 10.1038/s41598-020-74473-3
Webber QMR, 2017, ECOHEALTH, V14, P652, DOI 10.1007/s10393-017-1276-3
Wiegel FW, 2004, IMMUNOL CELL BIOL, V82, P127, DOI 10.1046/j.0818-9641.2004.01229.x
Wilhelm D, 2007, COMP BIOCHEM PHYS C, V146, P214, DOI 10.1016/j.cbpc.2006.11.015
Wilkinson GS, 2019, BIOL LETTERS, V15, DOI 10.1098/rsbl.2018.0860
Wilkinson GS, 2002, AGING CELL, V1, P124, DOI 10.1046/j.1474-9728.2002.00020.x
Williamson MM, 2000, J COMP PATHOL, V122, P201, DOI 10.1053/jcpa.1999.0364
Wilman H., 2014, Ecology, V95, P2027, DOI [DOI 10.1890/13-1917.1, 10.1890/13-1917.1]
Xie JZ, 2018, CELL HOST MICROBE, V23, P297, DOI 10.1016/j.chom.2018.01.006
Zhang GJ, 2013, SCIENCE, V339, P456, DOI 10.1126/science.1230835
Zhou P, 2016, P NATL ACAD SCI USA, V113, P2696, DOI 10.1073/pnas.1518240113
NR 96
TC 7
Z9 7
U1 0
U2 15
PU COMPANY BIOLOGISTS LTD
PI CAMBRIDGE
PA BIDDER BUILDING, STATION RD, HISTON, CAMBRIDGE CB24 9LF, ENGLAND
SN 0022-0949
EI 1477-9145
J9 J EXP BIOL
JI J. Exp. Biol.
PD JUL
PY 2021
VL 224
IS 13

AR jeb241109
DI 10.1242/jeb.241109
PG 10
WC Biology; Zoology
WE Science Citation Index Expanded (SCI-EXPANDED)
SC Life Sciences & Biomedicine - Other Topics; Zoology
GA TV0CW
UT WOS:000681397200026
PM 34104965
OA Green Submitted, Bronze
DA 2024-10-21
ER

PT J
AU Mühldorfer, K
   Speck, S
   Kurth, A
   Lesnik, R
   Freuling, C
   Müller, T
   Kramer-Schadt, S
   Wibbelt, G
AF Muehldorfer, Kristin
   Speck, Stephanie
   Kurth, Andreas
   Lesnik, Rene
   Freuling, Conrad
   Mueller, Thomas
   Kramer-Schadt, Stephanie
   Wibbelt, Gudrun
TI Diseases and Causes of Death in European Bats: Dynamics in Disease
   Susceptibility and Infection Rates
SO PLOS ONE
LA English
DT Article
ID HENDRA VIRUS; HOST; RABIES; PCR; LYSSAVIRUS; VESPERTILIONIDAE;
   CORONAVIRUSES; ECTOPARASITES; CHIROPTERA; PREVALENCE
AB Background: Bats receive increasing attention in infectious disease studies, because of their well recognized status as reservoir species for various infectious agents. This is even more important, as bats with their capability of long distance dispersal and complex social structures are unique in the way microbes could be spread by these mammalian species. Nevertheless, infection studies in bats are predominantly limited to the identification of specific pathogens presenting a potential health threat to humans. But the impact of infectious agents on the individual host and their importance on bat mortality is largely unknown and has been neglected in most studies published to date.
   Methodology/Principal Findings: Between 2002 and 2009, 486 deceased bats of 19 European species (family Vespertilionidae) were collected in different geographic regions in Germany. Most animals represented individual cases that have been incidentally found close to roosting sites or near human habitation in urban and urban-like

environments. The bat carcasses were subjected to a post-mortem examination and investigated histo-pathologically, bacteriologically and virologically. Trauma and disease represented the most important causes of death in these bats. Comparative analysis of pathological findings and microbiological results show that microbial agents indeed have an impact on bats succumbing to infectious diseases, with fatal bacterial, viral and parasitic infections found in at least 12% of the bats investigated.

Conclusions/Significance: Our data demonstrate the importance of diseases and infectious agents as cause of death in European bat species. The clear seasonal and individual variations in disease prevalence and infection rates indicate that maternity colonies are more susceptible to infectious agents, underlining the possible important role of host physiology, immunity and roosting behavior as risk factors for infection of bats.
C1 [Muehldorfer, Kristin; Kramer-Schadt, Stephanie; Wibbelt, Gudrun] Leibniz Inst Zoo & Wildlife Res, Berlin, Germany.
[Speck, Stephanie] Bundeswehr Inst Microbiol, Munich, Germany.
[Kurth, Andreas; Lesnik, Rene] Robert Koch Inst, Berlin, Germany.
[Freuling, Conrad; Mueller, Thomas] Friedrich Loeffler Inst, Wusterhausen, Germany.
C3 Leibniz Institut fur Zoo und Wildtierforschung; Bundeswehr Institute of
Microbiology (IMB); Robert Koch Institute; Friedrich Loeffler Institute
RP Mühldorfer, K (corresponding author), Leibniz Inst Zoo & Wildlife Res, Berlin, Germany.
EM muehldorfer@izw-berlin.de
RI Müller, Thomas/JKJ-5544-2023; Kramer-Schadt, Stephanie/A-8778-2013
OI Kramer-Schadt, Stephanie/0000-0002-9269-4446
FU Adolf and Hildegard Isler-Stiftung; FAZIT-Stiftung; Klara Samariter-Stiftung
FX This study was supported by the Adolf and Hildegard Isler-Stiftung, the
FAZIT-Stiftung (fellowship to KM) and the Klara Samariter-Stiftung. The
funders had no role in study design, data collection and analysis,
decision to publish, or preparation of the manuscript.
CR Aguirre LF, 2003, FUNCT ECOL, V17, P201, DOI 10.1046/j.1365-2435.2003.00721.x
Akaike H., 1973, 2 INT S INFORM THEOR, P267, DOI DOI 10.1007/978-1-4612-1694-0_15
[Anonymous], THESIS U LONDON
[Anonymous], 2005, Systematic bacteriology. The Proteobacteria, Part C: The Alpha-, Beta-, Delta-, and Epsilonproteobacteria
[Anonymous], 2007, Rabies
Bisping W., 1988, COLOUR ATLAS DIAGNOS
Bouma HR, 2010, J LEUKOCYTE BIOL, V88, P619, DOI 10.1189/jlb.0310174
Bradley CA, 2007, TRENDS ECOL EVOL, V22, P95, DOI 10.1016/j.tree.2006.11.001
Christe P, 2000, ECOL LETT, V3, P207

Christe P, 2007, J ANIM ECOL, V76, P703, DOI 10.1111/j.1365-2656.2007.01255.x
Chua KB, 2002, MICROBES INFECT, V4, P145, DOI 10.1016/S1286-4579(01)01522-2
Ciechanowski M, 2010, ACTA THERIOL, V55, P301, DOI 10.4098/j.at.0001-7051.093.2009
Dacheux L, 2009, EMERG INFECT DIS, V15, P280, DOI 10.3201/eid1502.080637
Luna LKD, 2007, J CLIN MICROBIOL, V45, P1049, DOI 10.1128/JCM.02426-06
Dean D.J., 1996, Laboratory Techniques in Rabies, V4th, P47
Duignan P., 2003, Surveillance (Wellington), V30, P15
Duzgunes N, 2016, MEDICAL MICROBIOLOGY AND IMMUNOLOGY FOR DENTISTRY, P55
Evans NJ, 2009, EMERG INFECT DIS, V15, P1331, DOI 10.3201/eid1508.090475
Feldhamer GA, 2009, AM MIDL NAT, V162, P43, DOI 10.1674/0003-0031-162.1.43
Freuling C, 2008, VET MICROBIOL, V131, P26, DOI 10.1016/j.vetmic.2008.02.028
Freuling CM, 2011, EMERG INFECT DIS, V17, P1519, DOI 10.3201/eid1708.110201
Gaisler J, 1998, J ZOOL, V244, P439, DOI 10.1111/j.1469-7998.1998.tb00048.x
Georgea DB, 2011, P NATL ACAD SCI USA, V108, P10208, DOI 10.1073/pnas.1010875108
Gloza-Rausch F, 2008, EMERG INFECT DIS, V14, P626, DOI 10.3201/eid1404.071439
Hajkova P, 2007, VET REC, V161, P139, DOI 10.1136/vr.161.4.139
Halpin K, 2000, J GEN VIROL, V81, P1927, DOI 10.1099/0022-1317-81-8-1927
Harris SL, 2006, BIOL CONSERV, V131, P193, DOI 10.1016/j.biocon.2006.04.006
HART BL, 1992, J PARASITOL, V78, P256, DOI 10.2307/3283472
Johnson N, 2010, VET MICROBIOL, V142, P151, DOI 10.1016/j.vetmic.2010.02.001
Johnson PTJ, 2010, TRENDS ECOL EVOL, V25, P362, DOI 10.1016/j.tree.2010.01.005
Kanuch P, 2007, ACTA CHIROPTEROL, V9, P277, DOI 10.3161/1733-5329(2007)9[277:ARPTFS]2.0.CO;2
Klempa B, 2006, EMERG INFECT DIS, V12, P838, DOI 10.3201/eid1205.051487
Kunz TH, 2011, ANN NY ACAD SCI, V1223, P1, DOI 10.1111/j.1749-6632.2011.06004.x
Kuzmin Ivan V, 2011, Emerg Health Threats J, V4, P7159, DOI 10.3402/ehtj.v4i0.7159
Leroy EM, 2005, NATURE, V438, P575, DOI 10.1038/438575a
Li WD, 2005, SCIENCE, V310, P676, DOI 10.1126/science.1118391
Linacre Adrian, 2005, Methods Mol Biol, V297, P45
Lucan RK, 2006, FOLIA PARASIT, V53, P147, DOI 10.14411/fp.2006.019
Mickleburgh SP, 2002, ORYX, V36, P18, DOI 10.1017/S0030605301000011
Mühldorfer K, 2011, BMC VET RES, V7, DOI 10.1186/1746-6148-7-61

Mühldorfer K, 2011, VET MICROBIOL, V149, P163, DOI 10.1016/j.vetmic.2010.10.002
Mühldorfer K, 2010, EMERG INFECT DIS, V16, P578, DOI 10.3201/eid1603.091035
Müller T, 2007, ARCH VIROL, V152, P273, DOI 10.1007/s00705-006-0853-5
O'Shea TJ, 2011, URBAN ECOSYST, V14, P665, DOI 10.1007/s11252-011-0182-7
Patterson BD, 2007, J TROP ECOL, V23, P177, DOI 10.1017/S0266467406003816
Pearce RD, 2007, J PARASITOL, V93, P518, DOI 10.1645/GE-973R.1
Plowright RK, 2008, P ROY SOC B-BIOL SCI, V275, P861, DOI 10.1098/rspb.2007.1260
Rihtaric D, 2010, ARCH VIROL, V155, P507, DOI 10.1007/s00705-010-0612-5
Sánchez-Seco MP, 2005, J VIROL METHODS, V126, P101, DOI 10.1016/j.jviromet.2005.01.025
Schipper J, 2008, SCIENCE, V322, P225, DOI 10.1126/science.1165115
Schneider L. G., 1985, Rabies in the tropics. Proceedings of an international conference on rabies control in the tropics, Tunis, Oct. 3-6, 1983, P47
Schrenzel MD, 2008, EMERG INFECT DIS, V14, P1616, DOI 10.3201/eid1410.080703
Simpson VR, 2000, RES VET SCI, V69, P11, DOI 10.1053/rvsc.2000.0384
Sonntag M, 2009, EMERG INFECT DIS, V15, P2052, DOI 10.3201/eid1512.090646
Spackman E, 2002, J CLIN MICROBIOL, V40, P3256, DOI 10.1128/JCM.40.9.3256-3260.2002
Streicker DG, 2010, SCIENCE, V329, P676, DOI 10.1126/science.1188836
Talan DA, 1999, NEW ENGL J MED, V340, P85, DOI 10.1056/NEJM199901143400202
ter Hofstede HM, 2005, J ZOOL, V266, P333, DOI 10.1017/S095283690500693X
THIRY E, 1988, J WILDLIFE DIS, V24, P268, DOI 10.7589/0090-3558-24.2.268
Towner JS, 2009, PLOS PATHOG, V5, DOI 10.1371/journal.ppat.1000536
Webster W. A., 1996, P96
Wibbelt G, 2007, J GEN VIROL, V88, P2651, DOI 10.1099/vir.0.83045-0
Wibbelt G, 2010, BIOL LETTERS, V6, P438, DOI 10.1098/rsbl.2010.0267
Wibbelt Gudrun, 2009, P775
Wong S, 2007, REV MED VIROL, V17, P67, DOI 10.1002/rmv.520
Zahn A, 2004, J ZOOL, V262, P383, DOI 10.1017/S0952836903004722
NR 66
TC 80
Z9 87
U1 0
U2 76
PU PUBLIC LIBRARY SCIENCE

PI SAN FRANCISCO
PA 1160 BATTERY STREET, STE 100, SAN FRANCISCO, CA 94111 USA
SN 1932-6203
J9 PLOS ONE
JI PLoS One
PD DEC 28
PY 2011
VL 6
IS 12
AR e29773
DI 10.1371/journal.pone.0029773
PG 12
WC Multidisciplinary Sciences
WE Science Citation Index Expanded (SCI-EXPANDED)
SC Science & Technology - Other Topics
GA 897SK
UT WOS:000300676300083
PM 22216354
OA Green Published, Green Submitted, gold
DA 2024-10-21
ER

PT J
AU Li, AQ
   Leng, HX
   Li, ZL
   Jin, LR
   Sun, KP
   Feng, J
AF Li, Aoqiang
   Leng, Haixia
   Li, Zhongle
   Jin, Longru
   Sun, Keping
   Feng, Jiang
TI Temporal dynamics of the bat wing transcriptome: Insight into
   gene-expression changes that enable protection against pathogen
SO VIRULENCE
LA English
DT Article
DE Wing transcriptome; immune defence; Pseudogymnoascus destructans;
   Rhinolophus ferrumequinum; hibernation
ID WHITE-NOSE SYNDROME; PSEUDOGYMNOASCUS-DESTRUCTANS; SKIN;
HIBERNATION;
   RESISTANCE; INFECTION; RESPONSES; GEOMYCES; REVEALS
AB Skin acts as a mechanical barrier between the body and its
surrounding environment and plays an important role in resistance to
pathogens. However, we still know little regarding skin responses to
physiological changes, particularly with regard to responses against
potential pathogens. We herein executed RNA-seq on the wing of the
Rhinolophus ferrumequinum to assess gene-expression variations at
four physiological stages: pre-hibernation, hibernation (early-
hibernation and late-hibernation), and post-hibernation, as well as
the gene-expression patterns of infected and uninfected bats with

the Pseudogymnoascus destructans (Pd). Our results showed that a greater number of differentially expressed genes between the more disparate physiological stages. Functional enrichment analysis showed that the down-regulated response pathways in hibernating bats included phosphorus metabolism and immune response, indicating metabolic suppression and decreased whole immune function. We also found up-regulated genes in post-hibernating bats that included C-type lectin receptor signalling, Toll-like receptor signalling pathway, and cell adhesion, suggesting that the immune response and skin integrity of the wing were improved after bats emerged from their hibernation and that this facilitated clearing Pd from the integument. Additionally, we found that the genes involved in cytokine or chemokine activity were up-regulated in late-hibernation compared to early-hibernation and that FOSB regulation of immune cell activation was differentially expressed in bats infected with Pd during late-hibernation, implying that the host's innate immune function was enhanced during late-hibernation so as to resist pathogenic infection. Our findings highlight the concept that maintenance of intrinsic immunity provides protection against pathogenic infections in highly resistant bats.
C1 [Li, Aoqiang; Leng, Haixia; Li, Zhongle; Jin, Longru; Sun, Keping; Feng, Jiang] Northeast Normal Univ, Jilin Prov Key Lab Anim Resource Conservat & Utili, Changchun, Peoples R China.
[Li, Aoqiang] Cent China Normal Univ, Sch Life Sci, Wuhan, Peoples R China.
[Li, Zhongle; Feng, Jiang] Jilin Agr Univ, Coll Life Sci, Changchun, Peoples R China.
C3 Northeast Normal University - China; Central China Normal University;
Jilin Agricultural University
RP Sun, KP; Feng, J (corresponding author), Northeast Normal Univ, Jilin Prov Key Lab Anim Resource Conservat & Utili, Changchun, Peoples R China.; Feng, J (corresponding author), Jilin Agr Univ, Coll Life Sci, Changchun, Peoples R China.
EM sunkp129@nenu.edu.cn; fengj@nenu.edu.cn
RI sun, ke/KMX-1265-2024
CR Anders S, 2015, BIOINFORMATICS, V31, P166, DOI 10.1093/bioinformatics/btu638
Beekman C, 2018, BIOL CHEM, V399, P1375, DOI 10.1515/hsz-2018-0240
BENJAMINI Y, 1995, J R STAT SOC B, V57, P289, DOI 10.1111/j.2517-6161.1995.tb02031.x
Bernard Francois-Xavier, 2012, J Allergy (Cairo), V2012, P718725, DOI 10.1155/2012/718725
Bolger AM, 2014, BIOINFORMATICS, V30, P2114, DOI 10.1093/bioinformatics/btu170
Bouma HR, 2010, J LEUKOCYTE BIOL, V88, P619, DOI 10.1189/jlb.0310174
Carey HV, 2003, PHYSIOL REV, V83, P1153, DOI 10.1152/physrev.00008.2003
Cryan PM, 2010, BMC BIOL, V8, DOI 10.1186/1741-7007-8-135
Davy CM, 2020, VIRULENCE, V11, P781, DOI 10.1080/21505594.2020.1768018
Davy CM, 2017, ECOL EVOL, V7, P7161, DOI 10.1002/ece3.3234

Dobin A, 2013, BIOINFORMATICS, V29, P15, DOI 10.1093/bioinformatics/bts635
Drees KP, 2017, MBIO, V8, DOI 10.1128/mBio.01941-17
Ellison AR, 2015, GENOME BIOL EVOL, V7, P286, DOI 10.1093/gbe/evu285
Eyerich S, 2018, TRENDS IMMUNOL, V39, P315, DOI 10.1016/j.it.2018.02.004
Faherty SL, 2018, MOL ECOL, V27, P709, DOI 10.1111/mec.14483
Fedorov VB, 2011, BMC GENOMICS, V12, DOI 10.1186/1471-2164-12-171
Field KA, 2018, MOL ECOL, V27, P3727, DOI 10.1111/mec.14827
Field KA, 2015, PLOS PATHOG, V11, DOI 10.1371/journal.ppat.1005168
Flieger M, 2016, SCI REP-UK, V6, DOI 10.1038/srep33200
Frank CL, 2018, MOLECULES, V23, DOI 10.3390/molecules23081986
Fritze M, 2021, DEV COMP IMMUNOL, V119, DOI 10.1016/j.dci.2021.104017
Geiser F, 2004, ANNU REV PHYSIOL, V66, P239, DOI 10.1146/annurev.physiol.66.032102.115105
Geiser F, 2013, CURR BIOL, V23, pR188, DOI 10.1016/j.cub.2013.01.062
Harazim M, 2018, BMC ZOOL, V3, DOI 10.1186/s40850-018-0035-4
Harris MA, 2004, NUCLEIC ACIDS RES, V32, pD258, DOI 10.1093/nar/gkh036
Hoyt JR, 2021, NAT REV MICROBIOL, V19, P196, DOI 10.1038/s41579-020-00493-5
Hoyt JR, 2020, P NATL ACAD SCI USA, V117, P7255, DOI 10.1073/pnas.1914794117
Hoyt JR, 2016, P ROY SOC B-BIOL SCI, V283, DOI 10.1098/rspb.2015.2861
Hu YZ, 2017, FISH SHELLFISH IMMUN, V66, P398, DOI 10.1016/j.fsi.2017.05.042
Janseng HT, 2019, COMMUN BIOL, V2, DOI 10.1038/s42003-019-0574-4
Kanehisa M, 2000, NUCLEIC ACIDS RES, V28, P27, DOI 10.1093/nar/28.1.27
Langwig KE, 2015, P ROY SOC B-BIOL SCI, V282, DOI 10.1098/rspb.2014.2335
Lei M, 2014, PLOS ONE, V9, DOI 10.1371/journal.pone.0107746
Li AQ, 2022, ENVIRON MICROBIOL, V24, P1484, DOI 10.1111/1462-2920.15754
Li B, 2011, BMC BIOINFORMATICS, V12, DOI 10.1186/1471-2105-12-323
Lilley TM, 2019, OECOLOGIA, V191, P295, DOI 10.1007/s00442-019-04499-6
Lorch JM, 2011, NATURE, V480, P376, DOI 10.1038/nature10590
Love MI, 2014, GENOME BIOL, V15, DOI 10.1186/s13059-014-0550-8
Matejuk A, 2018, ARCH IMMUNOL THER EX, V66, P45, DOI 10.1007/s00005-017-0477-3
McGuire LP, 2018, J MAMMAL, V99, P1065, DOI 10.1093/jmammal/gyy103
Meteyer CU, 2012, VIRULENCE, V3, P583, DOI 10.4161/viru.22330
Meteyer CU, 2009, J VET DIAGN INVEST, V21, P411, DOI 10.1177/104063870902100401
Minnis AM, 2013, FUNGAL BIOL-UK, V117, P638, DOI 10.1016/j.funbio.2013.07.001
Mortazavi A, 2008, NAT METHODS, V5, P621, DOI 10.1038/nmeth.1226

   Muller LK, 2013, MYCOLOGIA, V105, P253, DOI 10.3852/12-242
   Pannkuk EL, 2015, PLOS ONE, V10, DOI 10.1371/journal.pone.0120508
   Poorten TJ, 2016, MOL ECOL, V25, P5663, DOI 10.1111/mec.13871
   Rosenblum EB, 2012, MOL ECOL, V21, P3110, DOI 10.1111/
j.1365-294X.2012.05481.x
   Roy BA, 2000, EVOLUTION, V54, P51, DOI 10.1111/
j.0014-3820.2000.tb00007.x
   Schwartz C, 2013, PLOS ONE, V8, DOI 10.1371/journal.pone.0058427
   Song XY, 2015, IMMUNITY, V43, P488, DOI 10.1016/
j.immuni.2015.06.024
   Team RC, R LANG ENV STAT COMP
   Wang AY, 2013, CELL MICROBIOL, V15, P779, DOI 10.1111/cmi.12071
   Williams DR, 2005, PHYSIOL GENOMICS, V24, P13, DOI 10.1152/
physiolgenomics.00301.2004
   Xiao YH, 2015, PLOS ONE, V10, DOI 10.1371/journal.pone.0145702
   Xu Z, 2013, P NATL ACAD SCI USA, V110, P13097, DOI 10.1073/
pnas.1304319110
   Yan J, 2008, PHYSIOL GENOMICS, V32, P170, DOI 10.1152/
physiolgenomics.00075.2007
   Zukal J, 2016, SCI REP-UK, V6, DOI 10.1038/srep19829
NR 58
TC 6
Z9 6
U1 7
U2 57
PU TAYLOR & FRANCIS INC
PI PHILADELPHIA
PA 530 WALNUT STREET, STE 850, PHILADELPHIA, PA 19106 USA
SN 2150-5594
EI 2150-5608
J9 VIRULENCE
JI Virulence
PD DEC 31
PY 2023
VL 14
IS 1
AR 2156185
DI 10.1080/21505594.2022.2156185
PG 13
WC Immunology; Infectious Diseases; Microbiology
WE Science Citation Index Expanded (SCI-EXPANDED)
SC Immunology; Infectious Diseases; Microbiology
GA 7O1KB
UT WOS:000907789400001
PM 36599840
OA gold, Green Published
DA 2024-10-21
ER

PT J
AU Scheben, A
   Ramos, OM
   Kramer, M
   Goodwin, S

Oppenheim, S
   Becker, DJ
   Schatz, MC
   Simmons, NB
   Siepel, A
   McCombie, WR
AF Scheben, Armin
   Ramos, Olivia Mendivil
   Kramer, Melissa
   Goodwin, Sara
   Oppenheim, Sara
   Becker, Daniel J.
   Schatz, Michael C.
   Simmons, Nancy B.
   Siepel, Adam
   McCombie, W. Richard
TI Long-Read Sequencing Reveals Rapid Evolution of Immunity- and
   Cancer-Related Genes in Bats
SO GENOME BIOLOGY AND EVOLUTION
LA English
DT Article
DE cancer resistance; Chiroptera; comparative genomics; immunity;
long
   reads; viral tolerance
ID TOLL-LIKE RECEPTORS; POSITIVE SELECTION; MESSENGER-RNA; I
INTERFERONS;
   GENOMES; ALIGNMENT; PROTEINS; EXPRESSION; ACTIVATION; UNC93B1
AB Bats are exceptional among mammals for their powered flight,
extended lifespans, and robust immune systems and therefore have
been of particular interest in comparative genomics. Using the
Oxford Nanopore Technologies long-read platform, we sequenced the
genomes of two bat species with key phylogenetic positions, the
Jamaican fruit bat (Artibeus jamaicensis) and the Mesoamerican
mustached bat (Pteronotus mesoamericanus), and carried out a
comprehensive comparative genomic analysis with a diverse collection
of bats and other mammals. The high-quality, long-read genome
assemblies revealed a contraction of interferon (IFN)-alpha at the
immunity-related type I IFN locus in bats, resulting in a shift in
relative IFN-alpha and IFN-alpha copy numbers. Contradicting
previous hypotheses of constitutive expression of IFN-alpha being a
feature of the bat immune system, three bat species lost all IFN-
alpha genes. This shift to IFN-omega could contribute to the
increased viral tolerance that has made bats a common reservoir for
viruses that can be transmitted to humans. Antiviral genes
stimulated by type I IFNs also showed evidence of rapid evolution,
including a lineage-specific duplication of IFN-induced
transmembrane genes and positive selection in IFIT2. In addition, 33
tumor suppressors and 6 DNA-repair genes showed signs of positive
selection, perhaps contributing to increased longevity and reduced
cancer rates in bats. The robust immune systems of bats rely on both
bat-wide and lineagespecific evolution in the immune gene
repertoire, suggesting diverse immune strategies. Our study provides
new genomic resources for bats and sheds new light on the
extraordinary molecular evolution in this critically important group

of mammals.
C1 [Scheben, Armin; Schatz, Michael C.; Siepel, Adam] Cold Spring Harbor Lab, Simons Ctr Quantitat Biol, POB 100, Cold Spring Harbor, NY 11724 USA.
[Ramos, Olivia Mendivil; Kramer, Melissa; Goodwin, Sara; McCombie, W. Richard] Cold Spring Harbor Lab, POB 100, Cold Spring Harbor, NY 11724 USA.
[Oppenheim, Sara] Amer Museum Nat Hist, Inst Comparat Genom, New York, NY USA.
[Becker, Daniel J.] Univ Oklahoma, Sch Biol Sci, Norman, OK USA.
[Schatz, Michael C.] Johns Hopkins Univ, Dept Comp Sci, Baltimore, MD USA.
[Schatz, Michael C.] Johns Hopkins Univ, Dept Biol, Baltimore, MD USA.
[Simmons, Nancy B.] Amer Museum Nat Hist, Div Vertebrate Zool, Dept Mammal, New York, NY USA.
[Ramos, Olivia Mendivil] OneThree Biotech, 335 Madison Ave, New York, NY USA.
C3 Cold Spring Harbor Laboratory; Cold Spring Harbor Laboratory; American
Museum of Natural History (AMNH); University of Oklahoma System;
University of Oklahoma - Norman; Johns Hopkins University; Johns Hopkins
University; American Museum of Natural History (AMNH)
RP Siepel, A (corresponding author), Cold Spring Harbor Lab, Simons Ctr Quantitat Biol, POB 100, Cold Spring Harbor, NY 11724 USA.; McCombie, WR (corresponding author), Cold Spring Harbor Lab, POB 100, Cold Spring Harbor, NY 11724 USA.
EM asiepel@cshl.edu; mccombie@cshl.edu
RI McCombie, William/ACF-2412-2022; Schatz, Michael/L-2690-2019; Becker,
Daniel/A-6466-2014; Oppenheim, Sara/A-4641-2014
OI Siepel, Adam/0000-0002-3557-7219; Simmons, Nancy B./0000-0001-8807-7499;
Becker, Daniel/0000-0003-4315-8628; Goodwin, Sara/0000-0002-6110-7296;
Schatz, Michael/0000-0002-4118-4446; Oppenheim, Sara/0000-0003-1109-3639
FU US National Institutes of Health (NIH) [P30-CA045508, R35-GM127070];
Simons Center for Quantitative Biology; NIH [S10OD028632-01]; National
Science Foundation [BII 2213854]
FX This research was supported, in part, by US National Institutes of
Health (NIH) grants P30-CA045508 (to D. Tuveson and colleagues) and
R35-GM127070 (to Ad.S.) and by the Simons Center for Quantitative
Biology. Computational work was performed with assistance from the NIH
Grant S10OD028632-01. We would further like to acknowledge funding
support from the CSHL/Northwell Health Affiliation for purchase of the

ONT PromethION sequencer used in this study. We thank the CSHL Cancer
Center and Director David Tuveson for support as well as use of the
Sequencing Technologies and Analysis Shared Resource. The genome browser
was set up with help from Ritika Ramani. W.R.M. is the Davis Family
Professor of Human Genetics at CSHL. D.J.B. was supported by the
National Science Foundation (BII 2213854). We also thank Semir Beyaz for
helpful comments on the manuscript. Finally, we thank Brock Fenton, Neil
Duncan, the staff at the Lamanai Field Research Center, and many other
colleagues who assisted with the fieldwork necessary for this study. The
content is solely the responsibility of the authors and does not
necessarily represent the official views of the NIH or other funding
sources.
CR Abrusán G, 2009, BIOINFORMATICS, V25, P1329, DOI 10.1093/bioinformatics/btp084
Ahn M, 2019, NAT MICROBIOL, V4, P789, DOI 10.1038/s41564-019-0371-3
Ahn M, 2016, SCI REP-UK, V6, DOI 10.1038/srep21722
Alteber Z, 2018, IMMUNOL CELL BIOL, V96, P284, DOI 10.1111/imcb.12000
Amman BR, 2012, PLOS PATHOG, V8, DOI 10.1371/journal.ppat.1002877
Andersen KG, 2020, NAT MED, V26, P450, DOI 10.1038/s41591-020-0820-9
Anisimova M, 2002, MOL BIOL EVOL, V19, P950, DOI 10.1093/oxfordjournals.molbev.a004152
Aricò E, 2019, CANCERS, V11, DOI 10.3390/cancers11121943
Armstrong J, 2020, NATURE, V587, P246, DOI 10.1038/s41586-020-2871-y
Bailey CC, 2014, ANNU REV VIROL, V1, P261, DOI 10.1146/annurev-virology-031413-085537
Baker Z, 2017, ELIFE, V6, DOI 10.7554/eLife.24133
Banerjee A, 2020, ISCIENCE, V23, DOI 10.1016/j.isci.2020.100958
Banerjee A, 2017, SCI REP-UK, V7, DOI 10.1038/s41598-017-01513-w
Becker DJ, 2022, FRONT VIROL-LAUSANNE, V2, DOI 10.3389/fviro.2022.862961
Belotserkovskaya R, 2020, NAT COMMUN, V11, DOI 10.1038/s41467-020-14563-y
Benfield CTO, 2020, LIFE SCI ALLIANCE, V3, DOI 10.26508/lsa.201900542
Bigot Thomas, 2019, F1000Res, V8, P530, DOI 10.12688/f1000research.18776.1
Blum M, 2021, NUCLEIC ACIDS RES, V49, pD344, DOI 10.1093/nar/gkaa977
Blumer M, 2022, SCI ADV, V8, DOI 10.1126/sciadv.abm6494
Brass AL, 2009, CELL, V139, P1243, DOI 10.1016/j.cell.2009.12.017
Cabrera-Romo S, 2014, AM J TROP MED HYG, V91, P1227, DOI 10.4269/

ajtmh.14-0361
Calisher CH, 2006, CLIN MICROBIOL REV, V19, P531, DOI 10.1128/CMR.00017-06
Cantalapiedra CP, 2021, MOL BIOL EVOL, V38, P5825, DOI 10.1093/molbev/msab293
Cantarel BL, 2008, GENOME RES, V18, P188, DOI 10.1101/gr.6743907
Cavassim MIA, 2022, P NATL ACAD SCI USA, V119, DOI 10.1073/pnas.2114401119
Chesarino NM, 2017, EMBO REP, V18, P1740, DOI 10.15252/embr.201744100
Cock PJA, 2009, BIOINFORMATICS, V25, P1422, DOI 10.1093/bioinformatics/btp163
da Fonseca RR, 2010, FEBS LETT, V584, P469, DOI 10.1016/j.febslet.2009.12.022
David Q, 2022, VIRUSES-BASEL, V14, DOI 10.3390/v14020238
De Bie T, 2006, BIOINFORMATICS, V22, P1269, DOI 10.1093/bioinformatics/btl097
de Weerd NA, 2007, J BIOL CHEM, V282, P20053, DOI 10.1074/jbc.R700006200
Decout A, 2021, NAT REV IMMUNOL, V21, P548, DOI 10.1038/s41577-021-00524-z
Desai TM, 2014, PLOS PATHOG, V10, DOI 10.1371/journal.ppat.1004048
Deveryshetty J, 2019, ELIFE, V8, DOI 10.7554/eLife.44063
Diamond MS, 2013, NAT REV IMMUNOL, V13, P46, DOI 10.1038/nri3344
Doronina L, 2017, GENOME RES, V27, P997, DOI 10.1101/gr.210948.116
Dudchenko O, 2017, SCIENCE, V356, P92, DOI 10.1126/science.aal3327
Durinck S, 2005, BIOINFORMATICS, V21, P3439, DOI 10.1093/bioinformatics/bti525
Emms DM, 2019, GENOME BIOL, V20, DOI 10.1186/s13059-019-1832-y
Enard D, 2016, ELIFE, V5, DOI 10.7554/eLife.12469
Escalera-Zamudio M, 2015, MOL ECOL, V24, P5899, DOI 10.1111/mec.13431
Fensterl V, 2015, J VIROL, V89, P2462, DOI 10.1128/JVI.02744-14
Fitzgerald KA, 2020, CELL, V180, P1044, DOI 10.1016/j.cell.2020.02.041
Fleming TH, 2020, Phyllostomid bats: a un�ique mammalian radiation
Forbes SA, 2011, NUCLEIC ACIDS RES, V39, pD945, DOI 10.1093/nar/gkq929
Foster TL, 2016, CELL HOST MICROBE, V20, P429, DOI 10.1016/j.chom.2016.08.006
Fu LM, 2012, BIOINFORMATICS, V28, P3150, DOI 10.1093/bioinformatics/bts565
Fuchs J, 2017, J VIROL, V91, DOI [10.1128/JVI.00361-17, 10.1128/jvi.00361-17]
Furth N, 2017, CELL DEATH DIFFER, V24, P1488, DOI 10.1038/cdd.2017.99
Garlanda C, 2013, IMMUNITY, V39, P1003, DOI 10.1016/j.immuni.2013.11.010
Glorieux C, 2017, BIOL CHEM, V398, P1095, DOI 10.1515/hsz-2017-0131

Goh G, 2020, P NATL ACAD SCI USA, V117, P28939, DOI 10.1073/pnas.2003352117
Gonzalez H, 2018, GENE DEV, V32, P1267, DOI 10.1101/gad.314617.118
Gorbunova V, 2020, CELL METAB, V32, P31, DOI 10.1016/j.cmet.2020.06.013
Gouravani M, 2020, EXPERT REV CLIN IMMU, V16, P175, DOI 10.1080/1744666X.2020.1713755
Grabherr MG, 2011, NAT BIOTECHNOL, V29, P644, DOI 10.1038/nbt.1883
Guan DF, 2020, BIOINFORMATICS, V36, P2896, DOI 10.1093/bioinformatics/btaa025
Guy L, 2010, BIOINFORMATICS, V26, P2334, DOI 10.1093/bioinformatics/btq413
Haas BJ, 2003, NUCLEIC ACIDS RES, V31, P5654, DOI 10.1093/nar/gkg770
Haas BJ, 2008, GENOME BIOL, V9, DOI 10.1186/gb-2008-9-1-r7
Haas BJ, 2013, NAT PROTOC, V8, P1494, DOI 10.1038/nprot.2013.084
Halo JV, 2021, P NATL ACAD SCI USA, V118, DOI 10.1073/pnas.2016274118
Hawkins JA, 2019, P NATL ACAD SCI USA, V116, P11351, DOI 10.1073/pnas.1814995116
Hayward JA, 2022, bioRxiv, DOI [10.1101/2020.04.08.031203, 10.1101/2020.04.08.031203, DOI 10.1101/2020.04.08.031203]
Hayward JA, 2018, MOL BIOL EVOL, V35, P1626, DOI 10.1093/molbev/msy048
He K, 2021, GENOME BIOL EVOL, V13, DOI 10.1093/gbe/evaa270
Hennessy EJ, 2010, NAT REV DRUG DISCOV, V9, P293, DOI 10.1038/nrd3203
Holmes EC, 2021, CELL, V184, P4848, DOI 10.1016/j.cell.2021.08.017
Hu J, 2023, An Efficient Error Correction and Accurate Assembly Tool for Noisy Long Reads, DOI DOI 10.1101/2023.03.09.531669
Huang DCS, 2000, CELL, V103, P839, DOI 10.1016/S0092-8674(00)00187-2
Hur J, 2006, CANCER RES, V66, P10153, DOI 10.1158/0008-5472.CAN-05-3696
Irving AT, 2021, NATURE, V589, P363, DOI 10.1038/s41586-020-03128-0
Jaks E, 2007, J MOL BIOL, V366, P525, DOI 10.1016/j.jmb.2006.11.053
Jebb D, 2020, NATURE, V583, P578, DOI 10.1038/s41586-020-2486-3
Jiang HY, 2017, IMMUNOGENETICS, V69, P271, DOI 10.1007/s00251-016-0966-2
Johri P, 2023, MOL BIOL EVOL, V40, DOI 10.1093/molbev/msad100
Johri P, 2022, PLOS BIOL, V20, DOI 10.1371/journal.pbio.3001669
Kacprzyk J, 2021, AGING-US, V13, P7998, DOI 10.18632/aging.202852
Kang MY, 2013, CELL DEATH DIFFER, V20, P117, DOI 10.1038/cdd.2012.102
Kent WJ, 2002, GENOME RES, V12, P656, DOI [10.1101/gr.229202. Article published online before March 2002, 10.1101/gr.229202]
Kepler TB, 2010, BMC GENOMICS, V11, DOI 10.1186/1471-2164-11-444
Kim D, 2019, NAT BIOTECHNOL, V37, P907, DOI 10.1038/s41587-019-0201-4

Kim YM, 2008, NATURE, V452, P234, DOI 10.1038/nature06726
Kirilenko BM, 2023, SCIENCE, V380, P368, DOI 10.1126/science.abn3107
Kolmogorov M, 2019, NAT BIOTECHNOL, V37, P540, DOI 10.1038/s41587-019-0072-8
Korf I, 2004, BMC BIOINFORMATICS, V5, DOI 10.1186/1471-2105-5-59
Kosiol C, 2008, PLOS GENET, V4, DOI 10.1371/journal.pgen.1000144
Kovaka S, 2019, GENOME BIOL, V20, DOI 10.1186/s13059-019-1910-1
Kumar S, 2002, P NATL ACAD SCI USA, V99, P803, DOI 10.1073/pnas.022629899
Kumar S, 2017, MOL BIOL EVOL, V34, P1812, DOI 10.1093/molbev/msx116
Larsen PA, 2007, J MAMMAL, V88, P712, DOI 10.1644/06-MAMM-A-125R.1
Lee BL, 2013, ELIFE, V2, DOI [10.1155/2013/961957, 10.7554/eLife.00291]
Lee HE, 2019, SCI REP-UK, V9, DOI 10.1038/s41598-019-48794-x
Li DW, 2011, CLIN CANCER RES, V17, P3558, DOI 10.1158/1078-0432.CCR-10-2729
Li H., 2013, PREPRINT, DOI DOI 10.48550/ARXIV.1303.3997
Li H, 2018, BIOINFORMATICS, V34, P3094, DOI 10.1093/bioinformatics/bty191
Li SF, 2017, INT IMMUNOPHARMACOL, V52, P253, DOI 10.1016/j.intimp.2017.08.028
Li WD, 2005, SCIENCE, V310, P676, DOI 10.1126/science.1118391
Lomsadze A, 2005, NUCLEIC ACIDS RES, V33, P6494, DOI 10.1093/nar/gki937
López I, 2017, CELL DEATH DIFFER, V24, P1717, DOI 10.1038/cdd.2017.96
Löytynoja A, 2014, METHODS MOL BIOL, V1079, P155, DOI 10.1007/978-1-62703-646-7_10
Lu CW, 2019, J IMMUNOTHER CANCER, V7, DOI 10.1186/s40425-019-0635-8
Majoros WH, 2004, BIOINFORMATICS, V20, P2878, DOI 10.1093/bioinformatics/bth315
Marín-Aguilar F, 2020, AGING CELL, V19, DOI 10.1111/acel.13050
McNab F, 2015, NAT REV IMMUNOL, V15, P87, DOI 10.1038/nri3787
Moossavi M, 2018, MOL CANCER, V17, DOI 10.1186/s12943-018-0900-3
Santillán DDM, 2021, MOL ECOL, V30, P6449, DOI 10.1111/mec.16027
Moss CF, 2001, J ACOUST SOC AM, V110, P2207, DOI 10.1121/1.1398051
Munster VJ, 2016, SCI REP-UK, V6, DOI 10.1038/srep21878
Murrell B, 2012, PLOS GENET, V8, DOI 10.1371/journal.pgen.1002764
Nielsen R, 2005, PLOS BIOL, V3, P976, DOI 10.1371/journal.pbio.0030170
O'Connor EA, 2022, TRENDS GENET, V38, P650, DOI 10.1016/j.tig.2022.02.014
Olival KJ, 2017, NATURE, V546, P646, DOI 10.1038/nature22975
Oliveira-Nascimento L, 2012, FRONT IMMUNOL, V3, DOI 10.3389/fimmu.2012.00079
Ostrand-Rosenberg S, 2008, CURR OPIN GENET DEV, V18, P11, DOI 10.1016/j.gde.2007.12.007
Paigen K, 2018, TRENDS GENET, V34, P291, DOI 10.1016/j.tig.2017.12.017

Panchanathan R, 2013, INT IMMUNOL, V25, P521, DOI 10.1093/intimm/dxt015
Pavlovich SS, 2018, CELL, V173, P1098, DOI 10.1016/j.cell.2018.03.070
Pekar JE, 2022, SCIENCE, V377, P960, DOI 10.1126/science.abp8337
Pelka K, 2018, IMMUNITY, V48, P911, DOI 10.1016/j.immuni.2018.04.011
Pidugu VK, 2019, FRONT MOL BIOSCI, V6, DOI 10.3389/fmolb.2019.00148
Pond SLK, 2020, MOL BIOL EVOL, V37, P295, DOI 10.1093/molbev/msz197
Potter JHT, 2021, MOL BIOL EVOL, V38, P3864, DOI 10.1093/molbev/msab028
Pulliam JRC, 2012, J R SOC INTERFACE, V9, P89, DOI 10.1098/rsif.2011.0223
Rambaut A, 2018, SYST BIOL, V67, P901, DOI 10.1093/sysbio/syy032
Real PJ, 2006, FEBS LETT, V580, P5905, DOI 10.1016/j.febslet.2006.08.088
Reumers J, 2005, NUCLEIC ACIDS RES, V33, pD527
Rhie A, 2021, NATURE, V592, P737, DOI 10.1038/s41586-021-03451-0
Rhie A, 2020, GENOME BIOL, V21, DOI 10.1186/s13059-020-02134-9
Rojas D, 2016, SYST BIOL, V65, P432, DOI 10.1093/sysbio/syw011
Ruan J, 2020, NAT METHODS, V17, P155, DOI 10.1038/s41592-019-0669-3
Scheben A., 2021, figshare., DOI [10.6084/m9.figshare.15223014.v2, DOI 10.6084/M9.FIGSHARE.15223014.V2]
Schwartz JJ, 2014, NAT COMMUN, V5, DOI 10.1038/ncomms5370
Seim I, 2013, NAT COMMUN, V4, DOI 10.1038/ncomms3212
Shafin K, 2021, NAT METHODS, V18, P1322, DOI 10.1038/s41592-021-01299-w
Shafin K, 2020, NAT BIOTECHNOL, V38, P1044, DOI 10.1038/s41587-020-0503-6
Shen W, 2019, bioRxiv, DOI [10.1101/513523, DOI 10.1101/513523]
Siegrist F, 2011, J INTERF CYTOK RES, V31, P183, DOI 10.1089/jir.2010.0112
Sikes RS, 2016, J MAMMAL, V97, P663, DOI 10.1093/jmammal/gyw078
Simao FA, 2015, BIOINFORMATICS, V31, P3210, DOI 10.1093/bioinformatics/btv351
Simmons NB, 2008, NATURE, V451, P818, DOI 10.1038/nature06549
Simmons NB, 1998, Bulletin of the AMNH; no. 235. Relationships of Eocene bats
Slodkowicz G, 2020, P NATL ACAD SCI USA, V117, P5977, DOI 10.1073/pnas.1916786117
Smith MD, 2015, MOL BIOL EVOL, V32, P1342, DOI 10.1093/molbev/msv022
Sotero-Caio CG, 2013, BMC EVOL BIOL, V13, DOI 10.1186/1471-2148-13-276
Stamatakis A, 2014, BIOINFORMATICS, V30, P1312, DOI 10.1093/bioinformatics/btu033
Stanke M, 2006, NUCLEIC ACIDS RES, V34, pW435, DOI 10.1093/nar/gkl200
Sy SMH, 2009, P NATL ACAD SCI USA, V106, P7155, DOI 10.1073/pnas.0811159106
Tarailo-Graovac Maja, 2009, Curr Protoc Bioinformatics, VChapter

4, DOI 10.1002/0471250953.bi0410s25
Taylor DJ, 2011, BMC EVOL BIOL, V11, DOI 10.1186/1471-2148-11-336
Teeling EC, 2018, ANNU REV ANIM BIOSCI, V6, P23, DOI 10.1146/annurev-animal-022516-022811
Tejada-Martinez D, 2021, P ROY SOC B-BIOL SCI, V288, DOI 10.1098/rspb.2020.2592
Tengesdal IW, 2021, P NATL ACAD SCI USA, V118, DOI 10.1073/pnas.2000915118
Thomas C, 2011, CELL, V146, P621, DOI 10.1016/j.cell.2011.06.048
Tollis M, 2017, CURR OPIN GENET DEV, V42, P40, DOI 10.1016/j.gde.2016.12.004
Tsan MF, 2009, J LEUKOCYTE BIOL, V85, P905, DOI 10.1189/jlb.0109005
Velazquez-Salinas L, 2019, FRONT MICROBIOL, V10, DOI 10.3389/fmicb.2019.01057
Veldhoen M, 2017, NAT IMMUNOL, V18, P612, DOI 10.1038/ni.3742
Vicens A, 2018, GENES-BASEL, V9, DOI 10.3390/genes9120582
Vollger MR, 2019, NAT METHODS, V16, P88, DOI 10.1038/s41592-018-0236-3
Wang LF, 2021, NAT REV IMMUNOL, V21, P269, DOI 10.1038/s41577-021-00523-0
Wang LF, 2011, CURR OPIN VIROL, V1, P649, DOI 10.1016/j.coviro.2011.10.013
Wicker T, 2007, NAT REV GENET, V8, P973, DOI 10.1038/nrg2165
Wilkinson GS, 2019, BIOL LETTERS, V15, DOI 10.1098/rsbl.2018.0860
Wu TD, 2005, BIOINFORMATICS, V21, P1859, DOI 10.1093/bioinformatics/bti310
Xie JZ, 2018, CELL HOST MICROBE, V23, P297, DOI 10.1016/j.chom.2018.01.006
Xie X, 2019, FRONT IMMUNOL, V10, DOI 10.3389/fimmu.2019.00836
Xing JJ, 2021, NAT COMMUN, V12, DOI 10.1038/s41467-021-23003-4
Yan HD, 2020, BIOINFORMATICS, V36, P4269, DOI 10.1093/bioinformatics/btaa519
Yang Y, 2019, CELL DEATH DIS, V10, DOI 10.1038/s41419-019-1413-8
Yang ZH, 2002, MOL BIOL EVOL, V19, P908, DOI 10.1093/oxfordjournals.molbev.a004148
Yang ZL, 2012, CELL RES, V22, P1328, DOI 10.1038/cr.2012.111
Yang ZH, 2011, MOL BIOL EVOL, V28, P1217, DOI 10.1093/molbev/msq303
Youm YH, 2013, CELL METAB, V18, P519, DOI 10.1016/j.cmet.2013.09.010
Yu T, 2015, PROTEIN CELL, V6, P6, DOI 10.1007/s13238-014-0122-4
Yu WN, 2019, FRONT ONCOL, V9, DOI 10.3389/fonc.2019.00989
Yu X, 2018, NAT COMMUN, V9, DOI 10.1038/s41467-018-07384-7
Mendoza MLZ, 2018, NAT ECOL EVOL, V2, P659, DOI 10.1038/s41559-018-0476-8
Zhang GJ, 2013, SCIENCE, V339, P456, DOI 10.1126/science.1230835
Zhang JZ, 2005, MOL BIOL EVOL, V22, P2472, DOI 10.1093/molbev/msi237
Zhang Y, 2015, NAT IMMUNOL, V16, P1215, DOI 10.1038/ni.3279
Zhao M, 2016, NUCLEIC ACIDS RES, V44, pD1023, DOI 10.1093/nar/gkv1268
Zhao X, 2009, J INTERF CYTOK RES, V29, P687, DOI 10.1089/jir.2008.0060

   Zheng M, 2021, NAT IMMUNOL, V22, P829, DOI 10.1038/
s41590-021-00937-x
   Zhou P, 2016, P NATL ACAD SCI USA, V113, P2696, DOI 10.1073/
pnas.1518240113
   Zhu HF, 2021, TRENDS MICROBIOL, V29, P776, DOI 10.1016/
j.tim.2021.01.002
   Zimin AV, 2020, PLOS COMPUT BIOL, V16, DOI 10.1371/
journal.pcbi.1007981
   Zou YY, 2002, CANCER RES, V62, P8
NR 188
TC 12
Z9 12
U1 5
U2 7
PU OXFORD UNIV PRESS
PI OXFORD
PA GREAT CLARENDON ST, OXFORD OX2 6DP, ENGLAND
SN 1759-6653
J9 GENOME BIOL EVOL
JI Genome Biol. Evol.
PD SEP 4
PY 2023
VL 15
IS 9
DI 10.1093/gbe/evad148
PG 19
WC Evolutionary Biology; Genetics & Heredity
WE Science Citation Index Expanded (SCI-EXPANDED)
SC Evolutionary Biology; Genetics & Heredity
GA LM3K2
UT WOS:001187176200001
PM 37728212
OA Green Accepted, Green Submitted, Green Published, gold
DA 2024-10-21
ER

PT J
AU Katayama, M
   Murakami, S
   Matsugo, H
   Kamiki, H
   Fujii, M
   Takenaka-Uema, A
   Horimoto, T
AF Katayama, Misa
   Murakami, Shin
   Matsugo, Hiromichi
   Kamiki, Haruhiko
   Fujii, Marina
   Takenaka-Uema, Akiko
   Horimoto, Taisuke
TI Complete genome sequence of a novel bat mastadenovirus C strain
isolated
   from <i>Rhinolophus cornutus</i> in Japan

SO ARCHIVES OF VIROLOGY
LA English
DT Article
ID ADENOVIRUSES; SARS-COV-2; VIRUS
AB Here, we report a novel bat adenovirus strain isolated from apparently healthy bats of the species Rhinolophus cornutus in Japan. The genome of the isolate was 36,506 bp in length and encoded at least 33 proteins. Phylogenetic analysis of the DNA polymerase amino acid sequence, which provides one demarcation criterion for adenoviral species, indicated that the isolate belongs to the species Bat mastadenovirus C in the genus Mastadenovirus. Most of the encoded proteins shared high sequence similarity with those of known bat adenovirus C strains detected in different species of Rhinolophus, whereas the fiber protein and some E3- and E4-related proteins shared moderate similarity, and only the large E3 protein, which contains several host immune-suppression-related motifs, showed considerably lower similarity.
C1 [Katayama, Misa; Murakami, Shin; Matsugo, Hiromichi; Kamiki, Haruhiko; Fujii, Marina; Takenaka-Uema, Akiko; Horimoto, Taisuke] Univ Tokyo, Grad Sch Agr & Life Sci, Lab Vet Microbiol, Bunkyo Ku, 1-1-1 Yayoi, Tokyo 1138657, Japan.
[Matsugo, Hiromichi] Univ Tokyo, Grad Sch Agr & Life Sci, Lab Vet Publ Hlth, Tokyo, Japan.
C3 University of Tokyo; University of Tokyo
RP Murakami, S; Horimoto, T (corresponding author), Univ Tokyo, Grad Sch Agr & Life Sci, Lab Vet Microbiol, Bunkyo Ku, 1-1-1 Yayoi, Tokyo 1138657, Japan.
EM shin-murakami@g.ecc.u-tokyo.ac.jp; taihorimoto@g.ecc.u-tokyo.ac.jp
OI , Taisuke/0000-0002-9201-9424
FU Japan Agency for Medical Research and Development (AMED) [JP21fk0108602,
JP21fk0108615]
FX This work was supported by the Japan Agency for Medical Research and
Development (AMED) under Grant numbers JP21fk0108602 and JP21fk0108615.
CR Benko M., 2005, VIRUS TAXONOMY, P213
Borkenhagen LK, 2019, EMERG MICROBES INFEC, V8, P1679, DOI 10.1080/22221751.2019.1690953
Epstein JH, 2020, P NATL ACAD SCI USA, V117, P29190, DOI 10.1073/pnas.2000429117
Hassanin A, 2021, SCI REP-UK, V11, DOI 10.1038/s41598-021-93738-z
Johnson N, 2010, VET MICROBIOL, V142, P151, DOI 10.1016/j.vetmic.2010.02.001
Kobayashi T, 2019, SCI REP-UK, V9, DOI 10.1038/s41598-018-37224-z
Kuzmin IV, 2010, EMERG INFECT DIS, V16, P352, DOI 10.3201/eid1602.091269
Lau SKP, 2005, P NATL ACAD SCI USA, V102, P14040, DOI 10.1073/pnas.0506735102
Lau SKP, 2020, EMERG INFECT DIS, V26, P1542, DOI 10.3201/eid2607.200092
Li LL, 2021, EMERG MICROBES INFEC, V10, P1683, DOI 10.1080/22221751.2021.1964925

Murakami S, 2020, EMERG INFECT DIS, V26, P3025, DOI 10.3201/eid2612.203386
Ogawa H, 2017, VIRUSES-BASEL, V9, DOI 10.3390/v9120371
Shirogane Y, 2008, J VIROL, V82, P8942, DOI 10.1128/JVI.00676-08
Tan B, 2016, J GEN VIROL, V97, P1625, DOI 10.1099/jgv.0.000470
Wacharapluesadee S, 2021, NAT COMMUN, V12, DOI 10.1038/s41467-021-21240-1
Walker PJ, 2020, ARCH VIROL, V165, P2737, DOI 10.1007/s00705-020-04752-x
Zhou H, 2020, CURR BIOL, V30, P2196, DOI 10.1016/j.cub.2020.05.023
Zhou P., [No title captured]
NR 18
TC 4
Z9 4
U1 0
U2 4
PU SPRINGER WIEN
PI Vienna
PA Prinz-Eugen-Strasse 8-10, A-1040 Vienna, AUSTRIA
SN 0304-8608
EI 1432-8798
J9 ARCH VIROL
JI Arch. Virol.
PD MAR
PY 2022
VL 167
IS 3
BP 979
EP 982
DI 10.1007/s00705-021-05357-8
EA FEB 2022
PG 4
WC Virology
WE Science Citation Index Expanded (SCI-EXPANDED)
SC Virology
GA ZK4XF
UT WOS:000749980300002
PM 35112204
OA Green Published, Bronze
DA 2024-10-21
ER

PT J
AU Irving, AT
Rozario, P
Kong, PS
Luko, K
Gorman, JJ
Hastie, ML
Chia, WN
Mani, S
Lee, BPYH
Smith, GJD

Mendenhall, IH
   Larman, HB
   Elledge, SJ
   Wang, LF
AF Irving, Aaron T.
   Rozario, Pritisha
   Kong, Pui-San
   Luko, Katarina
   Gorman, Jeffrey J.
   Hastie, Marcus L.
   Chia, Wan Ni
   Mani, Shailendra
   Lee, Benjamin P. Y-H.
   Smith, Gavin J. D.
   Mendenhall, Ian H.
   Larman, H. Benjamin
   Elledge, Stephen J.
   Wang, Lin-Fa
TI Robust dengue virus infection in bat cells and limited innate immune
   responses coupled with positive serology from bats in IndoMalaya and
   Australasia
SO CELLULAR AND MOLECULAR LIFE SCIENCES
LA English
DT Article
DE Flavivirus; Immunity; Zoonosis; Pteropus; Disease; Bats
ID ENCEPHALITIS-VIRUS; PTEROPUS-ALECTO; TRANSMISSION; RESERVOIR; HOST;
   MEGACHIROPTERA; SUSCEPTIBILITY; PTEROPODIDAE; ACTIVATION; MOSQUITOS
AB Natural reservoir hosts can sustain infection of pathogens without succumbing to overt disease. Multiple bat species host a plethora of viruses, pathogenic to other mammals, without clinical symptoms. Here, we detail infection of bat primary cells, immune cells, and cell lines with Dengue virus. While antibodies and viral RNA were previously detected in wild bats, their ability to sustain infection is not conclusive. Old-world fruitbat cells can be infected, producing high titres of virus with limited cellular responses. In addition, there is minimal interferon (IFN) response in cells infected with MOIs leading to dengue production. The ability to support in vitro replication/production raises the possibility of bats as a transient host in the life cycle of dengue or similar flaviviruses. New antibody serology evidence from Asia/ Pacific highlights the previous exposure and raises awareness that bats may be involved in flavivirus dynamics and infection of other hosts.
C1 [Irving, Aaron T.; Rozario, Pritisha; Kong, Pui-San; Luko, Katarina; Chia, Wan Ni; Mani, Shailendra; Smith, Gavin J. D.; Mendenhall, Ian H.; Wang, Lin-Fa] Duke NUS Med Sch, Singapore, Singapore.
   [Gorman, Jeffrey J.; Hastie, Marcus L.] QIMR Berghofer Med Res Inst, Herston, Qld, Australia.
   [Lee, Benjamin P. Y-H.] Natl Pk Board, Conservat Div, Singapore,

Singapore.
[Larman, H. Benjamin] Johns Hopkins Univ, Baltimore, MD 21205 USA.
[Elledge, Stephen J.; Wang, Lin-Fa] Harvard Univ, Med Sch, Boston, MA 02115 USA.
[Elledge, Stephen J.; Wang, Lin-Fa] Brigham & Womens Hosp, Howard Hughes Med Inst, Boston, MA 02115 USA.
C3 National University of Singapore; QIMR Berghofer Medical Research Institute; Johns Hopkins University; Harvard University; Harvard Medical School; Howard Hughes Medical Institute; Harvard University; Brigham & Women's Hospital
RP Irving, AT; Wang, LF (corresponding author), Duke NUS Med Sch, Singapore, Singapore.
EM aaron.irving@duke-nus.edu.sg; Linfa.wang@duke-nus.edu.sg
RI Lee, Benjamin/IYT-2976-2023; Irving, Aaron/AAE-8874-2021; Chia, Wan Ni/HCH-7319-2022; Elledge, Stephen/HJX-9765-2023; Irving, Aaron/I-6754-2013; Hastie, Marcus/L-4366-2016
OI Irving, Aaron/0000-0002-0196-1570; Hastie, Marcus/0000-0002-8386-5549;
Mani, Shailendra/0000-0001-8541-8315; Wang, Lin-Fa/0000-0003-2752-0535;
Mendenhall, Ian/0000-0003-4250-6459
CR Ashford RW, 2003, EMERG INFECT DIS, V9, P1495
Balinsky CA, 2017, J VIROL, V91, DOI [10.1128/JVI.01606-16, 10.1128/jvi.01606-16]
Bean AGD, 2013, NAT REV IMMUNOL, V13, P851, DOI 10.1038/nri3551
Best SM, 2017, J VIROL, V91, DOI [10.1128/JVI.01970-16, 10.1128/jvi.01970-16]
Breed AC, 2010, ECOHEALTH, V7, P204, DOI 10.1007/s10393-010-0332-z
Ramirez JAC, 2015, J INTERF CYTOK RES, V35, P421, DOI 10.1089/jir.2014.0129
Chatel-Chaix L, 2016, CELL HOST MICROBE, V20, P342, DOI 10.1016/j.chom.2016.07.008
Cheng MS, 2015, BIOSENS BIOELECTRON, V70, P74, DOI 10.1016/j.bios.2015.03.018
Crameri G, 2009, PLOS ONE, V4, DOI 10.1371/journal.pone.0008266
Davis A, 2005, AM J TROP MED HYG, V73, P467, DOI 10.4269/ajtmh.2005.73.467
de Thoisy B, 2009, VECTOR-BORNE ZOONOT, V9, P157, DOI 10.1089/vbz.2007.0280
Dick OB, 2012, AM J TROP MED HYG, V87, P584, DOI 10.4269/ajtmh.2012.11-0770
Fallahi P, 2016, CLIN TER, V167, pE186, DOI 10.7417/CT.2016.1966
Green AM, 2014, J MOL BIOL, V426, P1148, DOI 10.1016/j.jmb.2013.11.023
Gubler DJ, 1998, CLIN MICROBIOL REV, V11, P480, DOI 10.1128/CMR.11.3.480
Gubler Duane J, 2006, Novartis Found Symp, V277, P3, DOI 10.1002/0470058005.ch2
Gunnell GF, 2017, PLOS ONE, V12, DOI 10.1371/journal.pone.0172621

Hamilton PB, 2012, MOL PHYLOGENET EVOL, V63, P521, DOI 10.1016/j.ympev.2012.01.007
Helbig KJ, 2013, PLOS NEGLECT TROP D, V7, DOI 10.1371/journal.pntd.0002178
HERBOLD JR, 1983, AM J VET RES, V44, P1889
Hodgkison R, 2004, BIOTROPICA, V36, P522, DOI 10.1646/1593
Hsieh JT, 2019, NAT COMMUN, V10, DOI 10.1038/s41467-019-08641-z
Hsu YL, 2013, PLOS ONE, V8, DOI 10.1371/journal.pone.0079518
Jain B, 2014, MICROB PATHOGENESIS, V69-70, P45, DOI 10.1016/j.micpath.2014.03.004
Jing Y, 2017, J THEOR BIOL, V416, P161, DOI 10.1016/j.jtbi.2016.12.014
Jones M, 2005, J VIROL, V79, P5414, DOI 10.1128/JVI.79.9.5414-5420.2005
Kading Rebekah C., 2018, Infection Ecology & Epidemiology, V8, P1439215, DOI 10.1080/20008686.2018.1439215
Kading RC, 2016, AM J TROP MED HYG, V95, P993, DOI 10.4269/ajtmh.16-0625
Kuzmin IV, 2017, J VIROL, V91, DOI [10.1128/JVI.02471-16, 10.1128/jvi.02471-16]
LAMOTTE LC, 1958, AM J HYG, V67, P101, DOI 10.1093/oxfordjournals.aje.a119912
Larman HB, 2011, NAT BIOTECHNOL, V29, P535, DOI 10.1038/nbt.1856
Lei M, 2016, SCI REP-UK, V6, DOI 10.1038/srep27726
Lin RJ, 2009, J IMMUNOL, V183, P8035, DOI 10.4049/jimmunol.0902728
Ludert JE, 2008, VIROL J, V5, DOI 10.1186/1743-422X-5-51
Machain-Williams C, 2013, J WILDLIFE DIS, V49, P684, DOI 10.7589/2012-12-318
Mandl JN, 2015, CELL, V160, P20, DOI 10.1016/j.cell.2014.12.003
Maximova OA, 2018, ANNU REV VIROL, V5, P255, DOI 10.1146/annurev-virology-092917-043439
Mishra A, 2017, INT REV IMMUNOL, V36, P89, DOI 10.1080/08830185.2017.1291640
Mohan D, 2018, NAT PROTOC, V13, P1958, DOI 10.1038/s41596-018-0025-6
Mok L, 2017, BMC GENOMICS, V18, DOI 10.1186/s12864-017-3994-x
Moreira-Soto A, 2017, COMP IMMUNOL MICROB, V50, P101, DOI 10.1016/j.cimid.2016.12.004
Mozzi A, 2015, GENOME BIOL EVOL, V7, P1016, DOI 10.1093/gbe/evv046
Mustafá YM, 2019, FRONT MICROBIOL, V10, DOI 10.3389/fmicb.2019.00525
Ooi EE, 2009, CAD SAUDE PUBLICA, V25, pS115, DOI 10.1590/S0102-311X2009001300011
Pan XB, 2012, PLOS ONE, V7, DOI 10.1371/journal.pone.0051033
Pattanakitsakul S, 2007, J PROTEOME RES, V6, P4592, DOI 10.1021/pr070366b
Pepin KM, 2015, PARASITE VECTOR, V8, DOI 10.1186/s13071-015-0659-y
Perea-Martínez L, 2013, COMP IMMUNOL MICROB, V36, P193, DOI 10.1016/j.cimid.2012.12.002
Ploquin MJ, 2016, CURR OPIN HIV AIDS, V11, P201, DOI 10.1097/COH.0000000000000238

Puccioni-Sohler M, 2015, ARQ NEURO-PSIQUIAT, V73, P698, DOI 10.1590/0004-282X20150074
REAGAN RL, 1952, J INFECT DIS, V91, P145, DOI 10.1093/infdis/91.2.145
Reller ME, 2012, EMERG INFECT DIS, V18, P256, DOI 10.3201/eid1802.110962
Romo H, 2018, EMERG MICROBES INFEC, V7, DOI 10.1038/s41426-018-0180-4
ROSEN L, 1985, AM J TROP MED HYG, V34, P603, DOI 10.4269/ajtmh.1985.34.603
Ryan PA, 1997, AM J TROP MED HYG, V57, P476, DOI 10.4269/ajtmh.1997.57.476
Shen B, 2013, PLOS ONE, V8, DOI 10.1371/journal.pone.0062307
Shen B, 2012, PLOS ONE, V7, DOI 10.1371/journal.pone.0033197
Shepard DS, 2013, PLOS NEGLECT TROP D, V7, DOI [10.1371/journal.pntd.0002055, 10.1371/journal.pntd.0002056]
SHEPHERD R. C, 1964, ZOONOSES RES, V3, P125
SIMPSON DIH, 1968, ANN TROP MED PARASIT, V62, P422, DOI 10.1080/00034983.1968.11686579
Sprokholt J, 2018, FUTURE VIROL, V13, P287, DOI 10.2217/fvl-2017-0146
Sprokholt JK, 2017, PLOS PATHOG, V13, DOI 10.1371/journal.ppat.1006738
Stabell AC, 2018, ELIFE, V7, DOI [10.7554/elife.31919, 10.7554/eLife.31919]
Thiemmeca S, 2016, J IMMUNOL, V197, P4053, DOI 10.4049/jimmunol.1600323
Thongyuan S, 2017, PLOS ONE, V12, DOI 10.1371/journal.pone.0180013
Bui TT, 2018, J GEN VIROL, V99, P1044, DOI 10.1099/jgv.0.001092
Urcuqui-Inchima S, 2017, ANTIVIR RES, V147, P47, DOI 10.1016/j.antiviral.2017.09.017
van den Hurk AF, 2009, AM J TROP MED HYG, V81, P457, DOI 10.4269/ajtmh.2009.81.457
Vasilakis N, 2011, NAT REV MICROBIOL, V9, P532, DOI 10.1038/nrmicro2595
Vicente-Santos A, 2017, PLOS NEGLECT TROP D, V11, DOI 10.1371/journal.pntd.0005537
Wang WK, 2002, J CLIN MICROBIOL, V40, P4472, DOI 10.1128/JCM.40.12.4472-4478.2002
Xie JZ, 2018, CELL HOST MICROBE, V23, P297, DOI 10.1016/j.chom.2018.01.006
Yin QY, 2016, PLOS ONE, V11, DOI 10.1371/journal.pone.0146274
Zheng TT, 2018, J THEOR BIOL, V443, P82, DOI 10.1016/j.jtbi.2018.01.017
Zhou P, 2016, SCI REP-UK, V6, DOI 10.1038/srep38597
Zompi S, 2012, VIRUSES-BASEL, V4, P62, DOI 10.3390/v4010062
NR 76
TC 10
Z9 12
U1 0
U2 10
PU SPRINGER BASEL AG
PI BASEL

PA PICASSOPLATZ 4, BASEL, 4052, SWITZERLAND
SN 1420-682X
EI 1420-9071
J9 CELL MOL LIFE SCI
JI Cell. Mol. Life Sci.
PD APR
PY 2020
VL 77
IS 8
BP 1607
EP 1622
DI 10.1007/s00018-019-03242-x
PG 16
WC Biochemistry & Molecular Biology; Cell Biology
WE Science Citation Index Expanded (SCI-EXPANDED)
SC Biochemistry & Molecular Biology; Cell Biology
GA LD8UB
UT WOS:000526301600010
PM 31352533
DA 2024-10-21
ER

PT J
AU Springer, MS
   Gatesy, J
AF Springer, Mark S.
   Gatesy, John
TI Pinniped Diphyly and Bat Triphyly: More Homology Errors Drive Conflicts
   in the Mammalian Tree
SO JOURNAL OF HEREDITY
LA English
DT Article
DE Chiroptera; homology; Mammalia; phylogenomics; Pinnipedia
ID MULTISPECIES COALESCENT MODEL; LONG-BRANCH ATTRACTION; MOLECULAR
   PHYLOGENY; GENE TREES; LIKELIHOOD SUPPORT; PLACENTAL MAMMALS; HIDDEN
   SUPPORT; SPECIES TREES; DATA SETS; CARNIVORA
AB Homology is perhaps the most central concept of phylogenetic biology. At difficult to resolve polytomies that are deep in the Tree of Life, a few homology errors in phylogenomic data can drive spurious phylogenetic results. Feijoo and Parada (2017) assembled three phylogenomic data sets for mammals and reported methodological discrepancies and unexpected results that contradict the monophyly of well-established clades in Pinnipedia and Yangochiroptera. Examination of Feijoo and Parada's (2017) data sets reveals extensive homology errors (paralogous sequences, alignments of different exons to each other) and cross-contamination of sequences from different species. These problems predictably result in distorted estimates of gene trees, species trees, bootstrap support, and branch lengths. Correction of these errors resulted in robust support for conventional relationships in Pinnipedia and Yangochiroptera. Phylogenomic data sets are not immune to the problems of homology errors in sequence alignments. Rather, sequence

alignments underlie all inferences in molecular phylogenetics and evolution and should be spot-checked for obvious errors via manual inspection of alignments and gene trees.
C1 [Springer, Mark S.] Univ Calif Riverside, Dept Evolut Ecol & Organismal Biol, Riverside, CA 92521 USA.
[Gatesy, John] Amer Museum Nat Hist, Div Vertebrate Zool, New York, NY 10024 USA.
C3 University of California System; University of California Riverside;
American Museum of Natural History (AMNH)
RP Springer, MS (corresponding author), Univ Calif Riverside, Dept Evolut Ecol & Organismal Biol, Riverside, CA 92521 USA.; Gatesy, J (corresponding author), Amer Museum Nat Hist, Div Vertebrate Zool, New York, NY 10024 USA.
EM springer@ucr.edu; jgatesy@amnh.org
FU National Science Foundation [DEB-1457735]; Direct For Biological Sciences [1457735] Funding Source: National Science Foundation; Division Of Environmental Biology [1457735] Funding Source: National Science Foundation
FX This work was supported by National Science Foundation grant DEB-1457735 to J.G. and M.S.S.
CR Amador LI, 2018, J MAMM EVOL, V25, P37, DOI 10.1007/s10914-016-9363-8
Arnason U, 2006, MOL PHYLOGENET EVOL, V41, P345, DOI 10.1016/j.ympev.2006.05.022
Bergsten J, 2005, CLADISTICS, V21, P163, DOI 10.1111/j.1096-0031.2005.00059.x
Minh BQ, 2013, MOL BIOL EVOL, V30, P1188, DOI 10.1093/molbev/mst024
Chernomor O, 2016, SYST BIOL, V65, P997, DOI 10.1093/sysbio/syw037
Chiari Y, 2012, BMC BIOL, V10, DOI 10.1186/1741-7007-10-65
de Queiroz A, 2007, TRENDS ECOL EVOL, V22, P34, DOI 10.1016/j.tree.2006.10.002
Degnan JH, 2006, PLOS GENET, V2, P762, DOI 10.1371/journal.pgen.0020068
Delsuc F, 2006, NATURE, V439, P965, DOI 10.1038/nature04336
Doronina L, 2015, MOL BIOL EVOL, V32, P3194, DOI 10.1093/molbev/msv188
dos Reis M, 2012, P ROY SOC B-BIOL SCI, V279, P3491, DOI 10.1098/rspb.2012.0683
Douzery EJP, 2014, MOL BIOL EVOL, V31, P1923, DOI 10.1093/molbev/msu132
Edwards SV, 2016, ZOOL SCR, V45, P63, DOI 10.1111/zsc.12210
Edwards SV, 2016, MOL PHYLOGENET EVOL, V94, P447, DOI 10.1016/j.ympev.2015.10.027
Edwards SV, 2009, EVOLUTION, V63, P1, DOI 10.1111/j.1558-5646.2008.00549.x
Eick GN, 2005, MOL BIOL EVOL, V22, P1869, DOI 10.1093/molbev/msi180
Emerling CA, 2015, P ROY SOC B-BIOL SCI, V282, DOI 10.1098/

rspb.2015.1817
Feijoo M, 2017, MOL PHYLOGENET EVOL, V113, P76, DOI 10.1016/j.ympev.2017.05.004
Flynn JJ, 2005, SYST BIOL, V54, P317, DOI 10.1080/10635150590923326
Foley NM, 2016, PHILOS T R SOC B, V371, DOI 10.1098/rstb.2015.0140
Fulton TL, 2006, MOL PHYLOGENET EVOL, V41, P165, DOI 10.1016/j.ympev.2006.05.025
Gatesy J, 1999, CLADISTICS, V15, P271, DOI 10.1111/j.1096-0031.1999.tb00268.x
Gatesy J, 2005, SYST BIOL, V54, P483, DOI 10.1080/10635150590945368
Gatesy J, 2017, CLADISTICS, V33, P295, DOI 10.1111/cla.12170
Gatesy J, 2014, MOL PHYLOGENET EVOL, V80, P231, DOI 10.1016/j.ympev.2014.08.013
Gatesy J, 2013, P NATL ACAD SCI USA, V110, pE1179, DOI 10.1073/pnas.1221121110
Heled J, 2010, MOL BIOL EVOL, V27, P570, DOI 10.1093/molbev/msp274
Kalyaanamoorthy S, 2017, NAT METHODS, V14, P587, DOI [10.1038/NMETH.4285, 10.1038/nmeth.4285]
Kearse M, 2012, BIOINFORMATICS, V28, P1647, DOI 10.1093/bioinformatics/bts199
Kolaczkowski B, 2004, NATURE, V431, P980, DOI 10.1038/nature02917
Kumar V, 2013, PLOS ONE, V8, DOI 10.1371/journal.pone.0060019
Lack JB, 2010, J MAMMAL, V91, P976, DOI 10.1644/09-MAMM-A-192.1
Nguyen LT, 2015, MOL BIOL EVOL, V32, P268, DOI 10.1093/molbev/msu300
Lee MSY, 2003, SYST BIOL, V52, P15, DOI 10.1080/10635150390132650
Luan PT, 2013, MOL PHYLOGENET EVOL, V66, P748, DOI 10.1016/j.ympev.2012.10.023
Mason VC, 2016, SCI ADV, V2, DOI 10.1126/sciadv.1600633
McCormack JE, 2012, GENOME RES, V22, P746, DOI 10.1101/gr.125864.111
Meredith RW, 2011, SCIENCE, V334, P521, DOI 10.1126/science.1211028
Miller MA, 2010, GAT COMP ENV WORKSH, V1, P1, DOI [DOI 10.1109/GCE.2010.5676129, 10.1109/gce.2010.5676129]
Patel S., 2013, J PHYLOGENET EVOL BI, V01, P1000110, DOI DOI 10.4172/2329-9002.1000110
Philippe H, 2005, BMC EVOL BIOL, V5, DOI 10.1186/1471-2148-5-50
Philippe H, 2017, EUR J TAXON, V283, P1, DOI 10.5852/ejt.2017.283
Ranwez V, 2007, BMC EVOL BIOL, V7, DOI 10.1186/1471-2148-7-241
Rokas A, 2003, NATURE, V425, P798, DOI 10.1038/nature02053
Romiguier J, 2013, MOL BIOL EVOL, V30, P2134, DOI 10.1093/molbev/mst116
Rosenberg NA, 2008, SYST BIOL, V57, P131, DOI 10.1080/10635150801905535
Scornavacca C, 2017, SYST BIOL, V66, P112, DOI 10.1093/sysbio/syw082
Shaw TI, 2012, PLOS ONE, V7, DOI 10.1371/journal.pone.0048472
Shen XX, 2017, NAT ECOL EVOL, V1, DOI 10.1038/s41559-017-0126
Simmons MP, 2016, MOL PHYLOGENET EVOL, V97, P76, DOI 10.1016/

j.ympev.2015.12.013
   Simmons MP, 2015, MOL PHYLOGENET EVOL, V91, P98, DOI 10.1016/
j.ympev.2015.05.011
   Song S, 2012, P NATL ACAD SCI USA, V109, P14942, DOI 10.1073/
pnas.1211733109
   Springer MS, 2016, MOL PHYLOGENET EVOL, V94, P1, DOI 10.1016/
j.ympev.2015.07.018
   Springer MS, 2014, TRENDS PLANT SCI, V19, P267, DOI 10.1016/
j.tplants.2014.02.012
   Stamatakis A, 2006, BIOINFORMATICS, V22, P2688, DOI 10.1093/
bioinformatics/btl446
   Sullivan Jack, 1997, Journal of Mammalian Evolution, V4, P77, DOI
10.1023/A:1027314112438
   Swofford DL, 2002, PHYLOGENETIC ANAL US
   Tarver JE, 2016, GENOME BIOL EVOL, V8, P330, DOI 10.1093/gbe/
evv261
   Teeling EC, 2005, SCIENCE, V307, P580, DOI 10.1126/
science.1105113
   Teeling EC, 2012, CAM STUD MORPHOL MOL, P1
NR 60
TC 9
Z9 10
U1 1
U2 36
PU OXFORD UNIV PRESS INC
PI CARY
PA JOURNALS DEPT, 2001 EVANS RD, CARY, NC 27513 USA
SN 0022-1503
EI 1465-7333
J9 J HERED
JI J. Hered.
PD MAY
PY 2018
VL 109
IS 3
BP 297
EP 307
DI 10.1093/jhered/esx089
PG 11
WC Evolutionary Biology; Genetics & Heredity
WE Science Citation Index Expanded (SCI-EXPANDED)
SC Evolutionary Biology; Genetics & Heredity
GA FZ8GE
UT WOS:000427844900008
PM 29077895
OA Bronze
DA 2024-10-21
ER

PT J
AU HATTEN, BA
   ALLEN, R
   SULKIN, SE
AF HATTEN, BA

ALLEN, R
SULKIN, SE
TI STUDIES ON IMMUNE CAPABILITIES OF CHIROPTERA .1. QUANTITATIVE AND QUALITATIVE NATURE OF IMMUNE RESPONSES IN BATS TO BACTERIOPHAGE PHICHI174
SO JOURNAL OF IMMUNOLOGY
LA English
DT Article
CR ADAMS MH, 1959, BACTERIOPHAGES, P463
BYERS VS, 1968, J EXP MED, V127, P307, DOI 10.1084/jem.127.2.307
CEROTTINI JC, 1969, J IMMUNOL, V103, P268
Dresser D W, 1968, Adv Immunol, V8, P129, DOI 10.1016/S0065-2776(08)60466-6
FINKELSTEIN MS, 1966, J IMMUNOL, V97, P565
FLETCHER MA, 1962, TEX REP BIOL MED, V20, P142
GOIDL EA, 1968, J IMMUNOL, V100, P371
GOOD RA, 1969, IMMUNOLOGICAL TOLERA, P215
HAMPAR B, 1968, J IMMUNOL, V100, P586
HATTEN BA, 1968, J IMMUNOL, V101, P141
JARSLOW BW, 1968, P NAT ACAD SCI, V61, P69
LEONARD LL, 1968, J IMMUNOL, V101, P1168
MCKENNA JM, 1968, P SOC EXP BIOL MED, V129, P720
MILLER JFA, 1969, IMMUNOLOGICAL TOLERA, P53
MOLLER G, 1968, J EXP MED, V127, P291, DOI 10.1084/jem.127.2.291
SULKIN SE, 1963, AM J TROP MED HYG, V12, P800, DOI 10.4269/ajtmh.1963.12.800
SULKIN SE, 1966, AM J TROP MED HYG, V15, P418, DOI 10.4269/ajtmh.1966.15.418
SULKIN SE, 1962, PROGRESS MEDICAL VIR, V4, P158
SVEHAG SE, 1968, PROG MED VIROL, V10, P1
Uhr J W, 1968, Adv Immunol, V8, P81, DOI 10.1016/S0065-2776(08)60465-4
UHR JW, 1962, J EXP MED, V115, P655, DOI 10.1084/jem.115.3.655
UHR JW, 1963, J EXP MED, V117, P457, DOI 10.1084/jem.117.3.457
NR 22
TC 10
Z9 11
U1 0
U2 1
PU AMER ASSOC IMMUNOLOGISTS
PI BETHESDA
PA 9650 ROCKVILLE PIKE, BETHESDA, MD 20814
SN 0022-1767
J9 J IMMUNOL
JI J. Immunol.
PY 1970
VL 105
IS 4
BP 872
EP &
PG 0
WC Immunology
WE Science Citation Index Expanded (SCI-EXPANDED)
SC Immunology

GA H5945
UT WOS:A1970H594500011
PM 5470524
DA 2024-10-21
ER

PT J
AU Wibbelt, G
   Moore, MS
   Schountz, T
   Voigt, CC
AF Wibbelt, Gudrun
   Moore, Marianne S.
   Schountz, Tony
   Voigt, Christian C.
TI Emerging diseases in Chiroptera: why bats?
SO BIOLOGY LETTERS
LA English
DT Article
DE immunology; infectious disease; virus ecology; white-nose syndrome;
   conservation; zoonosis
ID WHITE-NOSE SYNDROME; VIRUS; RESERVOIRS
AB A conference entitled '2nd International Berlin Bat Meeting: Bat Biology and Infectious Diseases' was held between the 19 and 21 of February 2010 in Berlin, Germany. Researchers from two major disciplines, bat biologists and disease specialists, met for the first time in an interdisciplinary event to share their knowledge about bat-associated diseases. The focus of the meeting was to understand why in particular bats are the hosts of so many of the most virulent diseases globally. During several sessions, key note speakers and participants discussed infectious diseases associated with bats, including viral diseases caused by Henipa-, Filo-, Corona- and Lyssaviruses, the spread of whitenose syndrome in North American bats, bat immunology/immunogenetics, bat parasites, and finally, conservation and human health issues.
C1 [Wibbelt, Gudrun; Voigt, Christian C.] Leibniz Inst Zoo & Wildlife Res, D-10315 Berlin, Germany.
   [Moore, Marianne S.] Boston Univ, Dept Biol, Ctr Ecol & Conservat Biol, Boston, MA 02215 USA.
   [Schountz, Tony] Univ No Colorado, Sch Biol Sci, Greeley, CO 80639 USA.
C3 Leibniz Institut fur Zoo und Wildtierforschung; Boston University;
   University of Northern Colorado
RP Voigt, CC (corresponding author), Leibniz Inst Zoo & Wildlife Res, Alfred Kowalke Str 17, D-10315 Berlin, Germany.
EM voigt@izw-berlin.de
RI Schountz, Tony/I-2161-2017
FU Eurobats; Deutsche Forschungsgemeinschaft [Vo 890/19]; Direct For
   Biological Sciences; Division Of Environmental Biology [0808487] Funding
   Source: National Science Foundation
FX We thank the Deutsche Forschungsgemeinschaft (Vo 890/19) and

Eurobats
   for financial support.
CR Blehert DS, 2009, SCIENCE, V323, P227, DOI 10.1126/
science.1163874
   Chua KB, 2002, MICROBES INFECT, V4, P145, DOI 10.1016/
S1286-4579(01)01522-2
   Epstein JH, 2009, J APPL ECOL, V46, P991, DOI 10.1111/
j.1365-2664.2009.01699.x
   Halpin K, 2007, CLIN INFECT DIS, V44, P711, DOI 10.1086/511078
   Lau SKP, 2005, P NATL ACAD SCI USA, V102, P14040, DOI 10.1073/
pnas.0506735102
   Leroy EM, 2005, NATURE, V438, P575, DOI 10.1038/438575a
   Li WD, 2005, SCIENCE, V310, P676, DOI 10.1126/science.1118391
   LORCH JM, J VET DIAGN IN PRESS
   Puechmaille SJ, 2010, EMERG INFECT DIS, V16, P290, DOI 10.3201/
eid1602.091391
   Reichard JD, 2009, ACTA CHIROPTEROL, V11, P457, DOI
10.3161/150811009X485684
   Towner JS, 2007, PLOS ONE, V2, DOI 10.1371/journal.pone.0000764
   Turner Gregory G., 2009, Bat Research News, V50, P47
NR 12
TC 52
Z9 57
U1 0
U2 66
PU ROYAL SOC
PI LONDON
PA 6-9 CARLTON HOUSE TERRACE, LONDON SW1Y 5AG, ENGLAND
SN 1744-9561
J9 BIOL LETTERS
JI Biol. Lett.
PD AUG 23
PY 2010
VL 6
IS 4
BP 438
EP 440
DI 10.1098/rsbl.2010.0267
PG 3
WC Biology; Ecology; Evolutionary Biology
WE Science Citation Index Expanded (SCI-EXPANDED)
SC Life Sciences & Biomedicine - Other Topics; Environmental
Sciences &
   Ecology; Evolutionary Biology
GA 623GB
UT WOS:000279725700039
PM 20427329
OA Green Published
DA 2024-10-21
ER

PT J
AU Strobel, S
   Becker, NI

   Encarnaçao, JA
AF Strobel, Sara
   Becker, Nina I.
   Encarnacao, Jorge A.
TI No short-term effect of handling and capture stress on immune responses
   of bats assessed by bacterial killing assay
SO MAMMALIAN BIOLOGY
LA English
DT Article
DE Acute stress; Bacterial killing assay; Constitutive immunity; Innate
   immune system; Chiroptera
ID BASE-LINE; IMMUNOLOGY; GLUCOCORTICOIDS; CORTICOSTERONE; MYOTIS; BLOOD
AB Ecoimmunology of wild animals becomes increasingly important. However, there are methodical limitations, especially when working on small mammals, e.g. small sample volume and acute stress associated with capture, handling and sampling that can influence immune parameters. The plasma bacterial killing assay measures innate humoral immune responses, mainly complement activity. It is a powerful tool with many methodical advantages. To avoid investigation of artefacts in future ecoimmunological studies the influence of acute stress on the bacterial killing activity was assessed.
   Bats (Nyctalus noctula, n = 9) were repeatedly sampled in three time intervals up to 97 min after capture. Bacterial killing activity against Escherichia coil was measured using a microplate absorbance reader. Bacterial killing activity was not influenced by capture, handling and sampling. Hence, released stress hormones did not affect circulating complement activity. To conclude, the plasma bacterial killing assay is reliable and efficient ecoimmunological tool in wildlife studies even of small mammals. (C) 2015 Deutsche Gesellschaft fur Saugetierkunde. Published by Elsevier GmbH. All rights reserved.
C1 [Strobel, Sara; Becker, Nina I.; Encarnacao, Jorge A.] Univ Giessen, Mammalian Ecol Grp, Dept Anim Ecol & Systemat, D-35392 Giessen, Germany.
C3 Justus Liebig University Giessen
RP Strobel, S (corresponding author), Univ Giessen, Mammalian Ecol Grp, Dept Anim Ecol & Systemat, Heinrich Buff Ring 26 IFZ, D-35392 Giessen, Germany.
EM Sara.Strobel@allzool.bio.uni-giessen.de;
   Nina.I.Becker@allzool.bio.uni-giessen.de;
   J.Encarnacao@bio.uni-giessen.de
RI Encarnacao, Jorge A./I-8644-2018
OI Encarnacao, Jorge A./0000-0001-8766-0607
CR [Anonymous], 2009, Bats of Britain, Europe and Northwest Africa
   Boughton RK, 2011, FUNCT ECOL, V25, P81, DOI 10.1111/j.1365-2435.2010.01817.x
   Buehler DM, 2008, PHYSIOL BIOCHEM ZOOL, V81, P673, DOI 10.1086/588591
   Charles A, 2001, PRINCIPLES INNATE AD
   Csorba G., 2008, NYCTALUS NOCTULA IUC

Demas GE., 2012, Ecoimmunology, P3
Derting TL, 2003, PHYSIOL BIOCHEM ZOOL, V76, P744, DOI 10.1086/375662
Dhabhar FS, 2002, INTEGR COMP BIOL, V42, P556, DOI 10.1093/icb/42.3.556
Encarnaçao JA, 2004, MAMM BIOL, V69, P163, DOI 10.1078/1616-5047-00131
French SS, 2012, BIOL OPEN, V1, P482, DOI 10.1242/bio.2012919
Grandin T, 1997, J ANIM SCI, V75, P249
KEUSCH GT, 1975, J INFECT DIS, V131, P584, DOI 10.1093/infdis/131.5.584
Korte SM, 2005, NEUROSCI BIOBEHAV R, V29, P3, DOI 10.1016/j.neubiorev.2004.08.009
Kurth A, 2012, PLOS ONE, V7, DOI 10.1371/journal.pone.0038688
Lee KA, 2006, INTEGR COMP BIOL, V46, P1000, DOI 10.1093/icb/icl049
Liebl AL, 2009, FUNCT ECOL, V23, P1091, DOI 10.1111/j.1365-2435.2009.01592.x
Lochmiller RL, 2000, OIKOS, V88, P87, DOI 10.1034/j.1600-0706.2000.880110.x
Luis AD, 2013, P ROY SOC B-BIOL SCI, V280, DOI 10.1098/rspb.2012.2753
Martin LB, 2009, GEN COMP ENDOCR, V163, P70, DOI 10.1016/j.ygcen.2009.03.008
Matson KD, 2006, P ROY SOC B-BIOL SCI, V273, P815, DOI 10.1098/rspb.2005.3376
Merchant ME, 2003, COMP BIOCHEM PHYS B, V136, P505, DOI 10.1016/S1096-4959(03)00256-2
Millet S, 2007, DEV COMP IMMUNOL, V31, P188, DOI 10.1016/j.dci.2006.05.013
Pilosof S, 2014, MAMM BIOL, V79, P183, DOI 10.1016/j.mambio.2013.10.005
RACEY PA, 1974, J REPROD FERTIL, V41, P169
Reeder DM, 2004, GEN COMP ENDOCR, V136, P260, DOI 10.1016/j.ygcen.2003.12.020
Reusken CBEM, 2010, VECTOR-BORNE ZOONOT, V10, P785, DOI 10.1089/vbz.2009.0173
Ricklin D, 2010, NAT IMMUNOL, V11, P785, DOI 10.1038/ni.1923
Romero LM, 2005, COMP BIOCHEM PHYS A, V140, P73, DOI 10.1016/j.cbpb.2004.11.004
Sapolsky RM, 2000, ENDOCR REV, V21, P55, DOI 10.1210/er.21.1.55
Segerstrom SC, 2007, CURR DIR PSYCHOL SCI, V16, P326, DOI 10.1111/j.1467-8721.2007.00522.x
Wibbelt G, 2007, J GEN VIROL, V88, P2651, DOI 10.1099/vir.0.83045-0
WIDMAIER EP, 1994, J EXP ZOOL, V269, P442, DOI 10.1002/jez.1402690507
WIDMAIER EP, 1993, J EXP ZOOL, V265, P533, DOI 10.1002/jez.1402650509
NR 33
TC 13
Z9 14
U1 1
U2 41

PU ELSEVIER GMBH
PI MUNICH
PA HACKERBRUCKE 6, 80335 MUNICH, GERMANY
SN 1616-5047
EI 1618-1476
J9 MAMM BIOL
JI Mamm. Biol.
PD AUG
PY 2015
VL 80
IS 4
BP 312
EP 315
DI 10.1016/j.mambio.2015.02.005
PG 4
WC Zoology
WE Science Citation Index Expanded (SCI-EXPANDED)
SC Zoology
GA CN5KU
UT WOS:000358469000011
PM 32218714
OA Green Published
DA 2024-10-21
ER

PT J
AU Eckhart, L
   Sipos, W
AF Eckhart, Leopold
   Sipos, Wolfgang
TI Differential Loss of OAS Genes Indicates Diversification of Antiviral
   Immunity in Mammals
SO VACCINES
LA English
DT Article
DE oligoadenylate synthetase; innate immunity; evolution; zoonoses;
   SARS-CoV-2; camel; bat; gene loss; gene duplication; gene family
ID EVOLUTIONARY RELATIONSHIPS; SYNTHETASE; PROTEINS; CONSEQUENCES;
   CDD/SPARCLE; FAMILY; VIRUS; CGAS; BATS
AB One of the main mechanisms of inducing an antiviral response depends on 2 '-5 '-oligoadenylate synthetases (OAS), which sense double-stranded RNA in the cytoplasm and activate RNase L. Mutations leading to the loss of functional OAS1 and OAS2 genes have been identified as important modifiers of the human immune response against severe acute respiratory syndrome coronavirus 2 (SARS-CoV-2). Here, we performed comparative genomics to search for inactivating mutations of OAS genes in other species of mammals and to establish a model for the diversifying evolution of the OAS gene family. We found that a recombination of the OAS and OAS-like (OASL) loci has led to the loss of OAS2 in camelids, which also lack OAS3. Both paralogs of OASL and OAS3 are absent in Asian pangolins. An evolutionarily ancient OAS paralog, which we tentatively name OAS4, has been lost in pangolins, bats and humans. A previously unknown

OAS gene, tentatively named OAS5, is present in Yangochiroptera, a suborder of bats. These differences in the OAS gene repertoire may affect innate immune responses to coronaviruses and other RNA viruses.
C1 [Eckhart, Leopold] Med Univ Vienna, Dept Dermatol, A-1090 Vienna, Austria.
[Sipos, Wolfgang] Univ Vet Med Vienna, Clin Dept Farm Anim & Herd Management, A-1210 Vienna, Austria.
C3 Medical University of Vienna; University of Veterinary Medicine Vienna
RP Eckhart, L (corresponding author), Med Univ Vienna, Dept Dermatol, A-1090 Vienna, Austria.
EM leopold.eckhart@meduniwien.ac.at
RI Eckhart, Leopold/AFJ-8308-2022
OI Eckhart, Leopold/0000-0002-5645-2036
CR Ablasser A, 2020, NAT IMMUNOL, V21, P17, DOI 10.1038/s41590-019-0556-1
ALTSCHUL SF, 1990, J MOL BIOL, V215, P403, DOI 10.1016/S0022-2836(05)80360-2
Azamor T, 2021, VIRUSES-BASEL, V13, DOI 10.3390/v13010096
Banday AR, 2022, NAT GENET, V54, P1103, DOI 10.1038/s41588-022-01113-z
Banerjee A, 2020, FRONT IMMUNOL, V11, DOI 10.3389/fimmu.2020.00026
Bignon E, 2022, CHEM COMMUN, V58, P2176, DOI 10.1039/d1cc07006a
Carelli FN, 2016, GENOME RES, V26, P301, DOI 10.1101/gr.198473.115
Carey CM, 2019, CELL HOST MICROBE, V25, P336, DOI 10.1016/j.chom.2019.01.001
Drappier M, 2015, CURR OPIN VIROL, V15, P19, DOI 10.1016/j.coviro.2015.07.002
Drosten C, 2014, NEW ENGL J MED, V371, P1359, DOI 10.1056/NEJMc1409847
Du TF, 2017, TRENDS MICROBIOL, V25, P968, DOI 10.1016/j.tim.2017.06.001
Eckhart L, 2006, MOL BIOL EVOL, V23, P2081, DOI 10.1093/molbev/msl077
Ehrlich F, 2020, MOL BIOL EVOL, V37, P982, DOI 10.1093/molbev/msz279
Fischer H, 2020, APOPTOSIS, V25, P474, DOI 10.1007/s10495-020-01614-4
Fischer H, 2020, FRONT IMMUNOL, V11, DOI 10.3389/fimmu.2020.00939
Ghosh A, 2019, IMMUNITY, V50, P51, DOI 10.1016/j.immuni.2018.12.013
Glidden CK, 2021, CURR BIOL, V31, pR1342, DOI 10.1016/j.cub.2021.08.070
Gonzalez V, 2022, ISCIENCE, V25, DOI 10.1016/j.isci.2022.104779
Haley PJ, 2022, INNATE IMMUN-LONDON, V28, P107, DOI 10.1177/17534259221093120
Han BA, 2021, TRENDS PARASITOL, V37, P1096, DOI 10.1016/j.pt.2021.08.006
Hancks DC, 2015, PLOS GENET, V11, DOI 10.1371/journal.pgen.1005203
Hedges SB, 2015, MOL BIOL EVOL, V32, P835, DOI 10.1093/molbev/

msv037
Holmes EC, 2021, CELL, V184, P4848, DOI 10.1016/j.cell.2021.08.017
Holthaus KB, 2021, SCI REP-UK, V11, DOI 10.1038/s41598-021-91863-3
Hornung V, 2014, NAT REV IMMUNOL, V14, P521, DOI 10.1038/nri3719
Hu JX, 2018, BMC EVOL BIOL, V18, DOI 10.1186/s12862-018-1315-x
Hur S, 2019, ANNU REV IMMUNOL, V37, P349, DOI 10.1146/annurev-immunol-042718-041356
Indik S, 2005, VET MICROBIOL, V107, P171, DOI 10.1016/j.vetmic.2005.01.024
Irving AT, 2021, NATURE, V589, P363, DOI 10.1038/s41586-020-03128-0
Jacquet S, 2022, SCI ADV, V8, DOI 10.1126/sciadv.add7540
Kjaer KH, 2009, J MOL EVOL, V69, P612, DOI 10.1007/s00239-009-9299-1
Kumar S, 2000, MOL BIOL EVOL, V17, P738, DOI 10.1093/oxfordjournals.molbev.a026352
Lam TTY, 2020, NATURE, V583, P282, DOI 10.1038/s41586-020-2169-0
Lee D, 2023, SCIENCE, V379, P554, DOI 10.1126/science.abo3627
Li WD, 2005, SCIENCE, V310, P676, DOI 10.1126/science.1118391
Liu G, 2023, ANIMALS-BASEL, V13, DOI 10.3390/ani13020209
Liu HL, 2020, INFECT GENET EVOL, V85, DOI 10.1016/j.meegid.2020.104438
Lu SN, 2020, NUCLEIC ACIDS RES, V48, pD265, DOI 10.1093/nar/gkz991
MacNeill AL, 2022, PATHOGENS, V11, DOI 10.3390/pathogens11080892
Malathi K, 2007, NATURE, V448, P816, DOI 10.1038/nature06042
Marchler-Bauer A, 2017, NUCLEIC ACIDS RES, V45, pD200, DOI 10.1093/nar/gkw1129
Mashimo T, 2003, GENOMICS, V82, P537, DOI 10.1016/S0888-7543(03)00176-9
McDougal MB, 2022, CURR OPIN VIROL, V53, DOI 10.1016/j.coviro.2022.101202
Medina RA, 2011, NAT REV MICROBIOL, V9, P590, DOI 10.1038/nrmicro2613
Mollentze N, 2020, P NATL ACAD SCI USA, V117, P9423, DOI 10.1073/pnas.1919176117
Mozzi A, 2015, GENOME BIOL EVOL, V7, P1016, DOI 10.1093/gbe/evv046
Perelygin AA, 2006, J MOL EVOL, V63, P562, DOI 10.1007/s00239-006-0073-3
Rebouillat D, 1999, J INTERF CYTOK RES, V19, P295, DOI 10.1089/107999099313992
Rong EG, 2018, FRONT IMMUNOL, V9, DOI 10.3389/fimmu.2018.01398
Sadler AJ, 2008, NAT REV IMMUNOL, V8, P559, DOI 10.1038/nri2314
Salova M, 2022, IMMUNOGENETICS, V74, P261, DOI 10.1007/s00251-021-01230-9
SHORT EC, 1987, J CLIN MICROBIOL, V25, P1735, DOI 10.1128/JCM.25.9.1735-1740.1987
Sipos W, 2003, VIRAL IMMUNOL, V16, P335, DOI 10.1089/088282403322396136
Sipos W, 2021, TIERAERZTL PRAX G N, V49, P256, DOI 10.1055/a-1516-3404

   Souvorov A., 2010, Natl. Cent. Biotechnol. Inf, V25, P1
   Tian J, 2022, CELL REP, V39, DOI 10.1016/j.celrep.2022.110969
   Tsagkogeorga G, 2013, CURR BIOL, V23, P2262, DOI 10.1016/j.cub.2013.09.014
   Wang XX, 2022, SCI CHINA LIFE SCI, V65, P1395, DOI 10.1007/s11427-021-2002-y
   Wickenhagen A, 2021, SCIENCE, V374, DOI 10.1126/science.abj3624
   Xie JZ, 2018, CELL HOST MICROBE, V23, P297, DOI 10.1016/j.chom.2018.01.006
   Yao YL, 2019, CYTOKINE, V114, P106, DOI 10.1016/j.cyto.2018.11.009
   Zhu JZ, 2014, IMMUNITY, V40, P936, DOI 10.1016/j.immuni.2014.05.007
NR 62
TC 1
Z9 1
U1 2
U2 3
PU MDPI
PI BASEL
PA ST ALBAN-ANLAGE 66, CH-4052 BASEL, SWITZERLAND
EI 2076-393X
J9 VACCINES-BASEL
JI Vaccines
PD FEB
PY 2023
VL 11
IS 2
AR 419
DI 10.3390/vaccines11020419
PG 11
WC Immunology; Medicine, Research & Experimental
WE Science Citation Index Expanded (SCI-EXPANDED)
SC Immunology; Research & Experimental Medicine
GA 9J0XX
UT WOS:000939922200001
PM 36851296
OA gold, Green Published
DA 2024-10-21
ER

PT J
AU Santillán, DDM
   Lama, TM
   Guerrero, YTG
   Brown, AM
   Donat, P
   Zhao, HB
   Rossiter, SJ
   Yohe, LR
   Potter, JH
   Teeling, EC
   Vernes, SC
   Davies, KTJ

   Myers, E
   Hughes, GM
   Huang, ZX
   Hoffmann, F
   Corthals, AP
   Ray, DA
   Dávalos, LM
AF Moreno Santillan, Diana D.
   Lama, Tanya M.
   Gutierrez Guerrero, Yocelyn T.
   Brown, Alexis M.
   Donat, Paul
   Zhao, Huabin
   Rossiter, Stephen J.
   Yohe, Laurel R.
   Potter, Joshua H.
   Teeling, Emma C.
   Vernes, Sonja C.
   Davies, Kalina T. J.
   Myers, Eugene
   Hughes, Graham M.
   Huang, Zixia
   Hoffmann, Federico
   Corthals, Angelique P.
   Ray, David A.
   Davalos, Liliana M.
TI Large-scale genome sampling reveals unique immunity and metabolic
   adaptations in bats
SO MOLECULAR ECOLOGY
LA English
DT Article
DE adaptive immunity; gene family evolution; inflammatory pathway;
innate
   immunity; metabolism; viral tolerance
ID PHYLOGENETIC ANALYSIS; INTERFERON-GAMMA; NKG2D; GENE; EXPRESSION;
   EVOLUTION; SELECTION; FLIGHT; HOST; LIGANDS
AB Comprising more than 1,400 species, bats possess adaptations
unique among mammals including powered flight, unexpected longevity,
and extraordinary immunity. Some of the molecular mechanisms
underlying these unique adaptations includes DNA repair, metabolism
and immunity. However, analyses have been limited to a few divergent
lineages, reducing the scope of inferences on gene family evolution
across the Order Chiroptera. We conducted an exhaustive comparative
genomic study of 37 bat species, one generated in this study,
encompassing a large number of lineages, with a particular emphasis
on multi-gene family evolution across immune and metabolic genes. In
agreement with previous analyses, we found lineage-specific
expansions of the APOBEC3 and MHC-I gene families, and loss of the
proinflammatory PYHIN gene family. We inferred more than 1,000 gene
losses unique to bats, including genes involved in the regulation of
inflammasome pathways such as epithelial defence receptors, the
natural killer gene complex and the interferon-gamma induced
pathway. Gene set enrichment analyses revealed genes lost in bats
are involved in defence response against pathogen-associated

molecular patterns and damage-associated molecular patterns. Gene family evolution and selection analyses indicate bats have evolved fundamental functional differences compared to other mammals in both innate and adaptive immune system, with the potential to enhance antiviral immune response while dampening inflammatory signalling. In addition, metabolic genes have experienced repeated expansions related to convergent shifts to plant-based diets. Our analyses support the hypothesis that, in tandem with flight, ancestral bats had evolved a unique set of immune adaptations whose functional implications remain to be explored.
C1 [Moreno Santillan, Diana D.; Ray, David A.] Texas Tech Univ, Dept Biol Sci, Lubbock, TX 79409 USA.
[Lama, Tanya M.; Brown, Alexis M.; Donat, Paul; Davalos, Liliana M.] SUNY Stony Brook, Dept Ecol & Evolut, Stony Brook, NY 11794 USA.
[Gutierrez Guerrero, Yocelyn T.] Univ Nacl Autonoma Mexico, Inst Ecol, Dept Ecol Evolut, Ciudad Univ, Mexico City, DF, Mexico.
[Zhao, Huabin] Wuhan Univ, Coll Life Sci, Hubei Key Lab Cell Homeostasis, Tibetan Ctr Ecol & Conservat WHU TU,Dept Ecol, Wuhan, Peoples R China.
[Rossiter, Stephen J.; Potter, Joshua H.; Davies, Kalina T. J.] Queen Mary Univ London, Sch Biol & Chem Sci, London, England.
[Yohe, Laurel R.] Yale Univ, Dept Earth & Planetary Sci, New Haven, CT USA.
[Teeling, Emma C.; Hughes, Graham M.; Huang, Zixia] Univ Coll Dublin, Sch Biol & Environm Sci, Dublin, Ireland.
[Vernes, Sonja C.] Max Planck Inst Psycholinguist, Neurogenet Vocal Commun Grp, Nijmegen, Netherlands.
[Vernes, Sonja C.] Donders Inst Brain Cognit & Behav, Nijmegen, Netherlands.
[Vernes, Sonja C.] Univ St Andrews, Sch Biol, St Andrews, Fife, Scotland.
[Myers, Eugene] Max Planck Inst Mol Cell Biol & Genet, Dresden, Germany.
[Hoffmann, Federico] Mississippi State Univ, Dept Biochem Mol Biol Entomol & Plant Pathol, Mississippi State, MS 39762 USA.
[Corthals, Angelique P.] John Jay Coll Criminal Justice, Dept Sci, New York, NY USA.
[Davalos, Liliana M.] SUNY Stony Brook, Consortium Interdisciplinary Environm Res, Stony Brook, NY 11794 USA.
C3 Texas Tech University System; Texas Tech University; State University of
New York (SUNY) System; Stony Brook University; Universidad Nacional
Autonoma de Mexico; Wuhan University; University of London; Queen Mary
University London; Yale University; University College Dublin; Max
Planck Society; Radboud University Nijmegen; University of St Andrews;
Max Planck Society; Mississippi State University; City University of New
York (CUNY) System; State University of New York (SUNY) System; Stony
Brook University

RP Ray, DA (corresponding author), Texas Tech Univ, Dept Biol Sci,
Lubbock, TX 79409 USA.; Dávalos, LM (corresponding author), SUNY
Stony Brook, Dept Ecol & Evolut, Stony Brook, NY 11794 USA.
EM david.a.ray@ttu.edu
RI Davalos, Liliana/AEE-7879-2022; Rossiter, Stephen/E-2111-2011;
Zhao,
Huabin/AAS-7513-2020; Huang, Zixia/AAO-6936-2021; Yohe,
Laurel/ABE-5464-2021; Vernes, Sonja/E-8454-2012
OI Potter, Joshua/0000-0002-3785-1656; Hughes, Graham/
0000-0003-3088-345X;
Huang, Zixia/0000-0002-1298-0486; Vernes, Sonja/
0000-0003-0305-4584;
Corthals, Angelique/0000-0002-5610-2992; Teeling,
Emma/0000-0002-3309-1346; Yohe, Laurel/0000-0003-1567-8749;
Moreno-Santillan, Diana D./0000-0003-2153-0732; Davalos,
Liliana/0000-0002-4327-7697; Davies, Kalina/0000-0002-4258-4775;
Lama,
Tanya/0000-0002-7372-8081
FU Irish Research Council [IRCLA/2017/58]; UKRI Future Leaders
Fellowship
[MR/T021985/1]; Human Frontiers Science Program Grant
[RGP0058/2016];
National Science Foundation [NSF-DBI 1812035, NSF-DEB 1442142,
NSF-DEB
1838273, NSF-DEB 1838283, NSF-DGE 1633299, NSF-IOS 2031906, NSF-
IOS
2031926, NSF-IOS 2032006, NSF-IOS 2032011, NSF-IOS 2032063, NSF-
IOS
2032073, NSF-PRFB 2010853]; Irish Research Council (IRC) [IRCLA/
2017/58]
Funding Source: Irish Research Council (IRC); FLF [MR/T021985/1]
Funding
Source: UKRI
FX Irish Research Council, Grant/Award Number: IRCLA/2017/58; UKRI
Future
Leaders Fellowship (, Grant/Award Number: MR/T021985/1; Human
Frontiers
Science Program Grant, Grant/Award Number: RGP0058/2016; National
Science Foundation, Grant/Award Number: NSF-DBI 1812035, NSF-DEB
1442142, NSF-DEB 1838273, NSF-DEB 1838283, NSF-DGE 1633299, NSF-
IOS
2031906, NSF-IOS 2031926, NSF-IOS 2032006, NSF-IOS 2032011, NSF-
IOS
2032063, NSF-IOS 2032073 and NSF-PRFB 2010853
CR Ahn M, 2019, NAT MICROBIOL, V4, P789, DOI 10.1038/
s41564-019-0371-3
Ahn M, 2016, SCI REP-UK, V6, DOI 10.1038/srep21722
Akbar AN, 2020, SCIENCE, V369, P256, DOI 10.1126/science.abb0762
Amitai O, 2010, J EXP BIOL, V213, P2693, DOI 10.1242/jeb.043505
Arapovic J, 2009, J VIROL, V83, P8198, DOI 10.1128/JVI.02549-08
Ashburner M, 2000, NAT GENET, V25, P25, DOI 10.1038/75556
AUSTAD SN, 1991, J GERONTOL, V46, pB47, DOI 10.1093/geronj/
46.2.B47
Ayala-Berdon J, 2011, PHYSIOL BIOCHEM ZOOL, V84, P458, DOI

10.1086/661541
Babic AM, 2004, MOL MED, V10, P72, DOI 10.2119/2004-00038.Wagner
Baker M.L., 2018, ADV COMP IMMUNOLOGY, P869, DOI [10.1007/978-3-319-76768-0_23, DOI 10.1007/978-3-319-76768-0_23]
Banerjee A, 2020, FRONT IMMUNOL, V11, DOI 10.3389/fimmu.2020.00026
Banerjee A, 2017, SCI REP-UK, V7, DOI 10.1038/s41598-017-01513-w
Banerjee M, 2002, EUR J IMMUNOL, V32, P1947, DOI 10.1002/1521-4141(200207)32:7<1947::AID-IMMU1947>3.0.CO;2-1
Basler C., 2020, BATS VIRUSES CURRENT, P119, DOI [10.21775/9781912530144.08, DOI 10.21775/9781912530144.08]
Bateman A, 2019, NUCLEIC ACIDS RES, V47, pD506, DOI 10.1093/nar/gky1049
Bay RA, 2017, SCI ADV, V3, DOI 10.1126/sciadv.1701413
Berg J.M., 2002, BIOCHEMISTRY
Bernatchez L, 2003, J EVOLUTION BIOL, V16, P363, DOI 10.1046/j.1420-9101.2003.00531.x
Bhattacharya S, 2014, IMMUNOL RES, V58, P234, DOI 10.1007/s12026-014-8516-1
Boni MF, 2020, NAT MICROBIOL, V5, P1408, DOI 10.1038/s41564-020-0771-4
Brook CE, 2020, ELIFE, V9, DOI 10.7554/eLife.48401
Brook CE, 2015, TRENDS MICROBIOL, V23, P172, DOI 10.1016/j.tim.2014.12.004
Caillat-Zucman S, 2006, HUM IMMUNOL, V67, P204, DOI 10.1016/j.humimm.2006.02.013
Camacho C, 2009, BMC BIOINFORMATICS, V10, DOI 10.1186/1471-2105-10-421
Carbon S, 2019, NUCLEIC ACIDS RES, V47, pD330, DOI 10.1093/nar/gky1055
Costantini D, 2019, CURR ZOOL, V65, P147, DOI 10.1093/cz/zoy039
Czaplewski Nicholas J., 2010, P240
Czaplewski Nicolas J., 2003, Revista de la Academia Colombiana de Ciencias Exactas Fisicas y Naturales, V27, P263
Darriba D, 2011, BIOINFORMATICS, V27, P1164, DOI 10.1093/bioinformatics/btr088
Davalos L.M., 2020, Phyllostomid Bats: A Unique Mammalian Radiation, P87
Dávalos LM, 2014, SYST BIOL, V63, P582, DOI 10.1093/sysbio/syu022
Davies KTJ, 2020, MOL ECOL, V29, P1839, DOI 10.1111/mec.15445
Delves PJ, 2017, Roitt's essential immunology, V13th
Denton JF, 2014, PLOS COMPUT BIOL, V10, DOI 10.1371/journal.pcbi.1003998
Ding KY, 2005, MOL BIOL EVOL, V22, P148, DOI 10.1093/molbev/msh266
Djelloul M, 2016, BRAIN BEHAV IMMUN, V58, P209, DOI 10.1016/j.bbi.2016.07.147
Dong D, 2017, MOL BIOL EVOL, V34, P20, DOI 10.1093/molbev/msw231
Dong ZM, 1997, P NATL ACAD SCI USA, V94, P7526, DOI 10.1073/pnas.94.14.7526
Drexler JF, 2012, NAT COMMUN, V3, DOI 10.1038/ncomms1796
Dumont ER, 2012, P ROY SOC B-BIOL SCI, V279, P1797, DOI 10.1098/rspb.2011.2005
Eckalbar WL, 2016, NAT GENET, V48, P528, DOI 10.1038/ng.3537

Eiting TP, 2009, J MAMM EVOL, V16, P151, DOI 10.1007/s10914-009-9118-x
Emms DM, 2019, GENOME BIOL, V20, DOI 10.1186/s13059-019-1832-y
Feng CG, 2008, NAT IMMUNOL, V9, P1279, DOI 10.1038/ni.1653
Fenton M.B., 2015, Bats: a world of science and mystery
Ferber IA, 1996, J IMMUNOL, V156, P5
Fjell CD, 2008, PROTEINS, V73, P420, DOI 10.1002/prot.22059
Fleischer T, 2017, SCI REP-UK, V7, DOI 10.1038/s41598-017-06392-9
Foley NM, 2018, SCI ADV, V4, DOI 10.1126/sciadv.aao0926
Genereux DP, 2020, NATURE, V587, P240, DOI 10.1038/s41586-020-2876-6
Goebel J, 2017, BMC GENOMICS, V18, DOI 10.1186/s12864-017-3839-7
Goldstein T, 2018, NAT MICROBIOL, V3, P1084, DOI 10.1038/s41564-018-0227-2
Gorbunova V, 2020, CELL METAB, V32, P31, DOI 10.1016/j.cmet.2020.06.013
Grigat J, 2007, J IMMUNOL, V179, P3958, DOI 10.4049/jimmunol.179.6.3958
Guerra N, 2013, CLIN IMMUNOL, V149, P432, DOI 10.1016/j.clim.2013.09.003
Gunnell Gregg F., 2005, Journal of Mammalian Evolution, V12, P209, DOI 10.1007/s10914-005-6945-2
Gutiérrez-Guerrero YT, 2020, GIGASCIENCE, V9, DOI 10.1093/gigascience/giaa059
Hahn MW, 2005, GENOME RES, V15, P1153, DOI 10.1101/gr.3567505
Han HJ, 2015, VIRUS RES, V205, P1, DOI 10.1016/j.virusres.2015.05.006
Han MV, 2013, MOL BIOL EVOL, V30, P1987, DOI 10.1093/molbev/mst100
Han YJ, 2015, BMC EVOL BIOL, V15, DOI 10.1186/s12862-015-0373-6
Harper JM, 2007, AGING CELL, V6, P1, DOI 10.1111/j.1474-9726.2006.00255.x
Hayden S, 2014, MOL BIOL EVOL, V31, P917, DOI 10.1093/molbev/msu043
Hayman DTS, 2019, NAT MICROBIOL, V4, P728, DOI 10.1038/s41564-019-0430-9
Hayward JA, 2018, MOL BIOL EVOL, V35, P1626, DOI 10.1093/molbev/msy048
Healy K, 2014, P ROY SOC B-BIOL SCI, V281, DOI 10.1098/rspb.2014.0298
Holt C, 2011, BMC BIOINFORMATICS, V12, DOI 10.1186/1471-2105-12-491
Hu B, 2017, PLOS PATHOG, V13, DOI 10.1371/journal.ppat.1006698
Huang RL, 2019, FRONT PHARMACOL, V10, DOI 10.3389/fphar.2019.00445
Huang ZX, 2020, AGING-US, V12, DOI 10.18632/aging.103725
Huang ZX, 2019, NAT ECOL EVOL, V3, P1110, DOI 10.1038/s41559-019-0913-3
Huelsmann M, 2019, SCI ADV, V5, DOI 10.1126/sciadv.aaw6671
Hughes GM, 2018, MOL BIOL EVOL, V35, P1390, DOI 10.1093/molbev/msy028
Irving AT, 2021, NATURE, V589, P363, DOI 10.1038/s41586-020-03128-0
Jebb D, 2020, NATURE, V583, P578, DOI 10.1038/s41586-020-2486-3

Jones KE, 2005, EVOLUTION, V59, P2243, DOI 10.1111/j.0014-3820.2005.tb00932.x
Jones P, 2014, BIOINFORMATICS, V30, P1236, DOI 10.1093/bioinformatics/btu031
Kacprzyk J, 2017, ACTA CHIROPTEROL, V19, P219, DOI [10.3161/15081109ACC2017.19.2.001, 10.3161/15081109acc2017.19.2.001]
Katoh Kazutaka, 2013, Mol Biol Evol, V30, P772, DOI 10.1093/molbev/mst010
Kearse M, 2012, BIOINFORMATICS, V28, P1647, DOI 10.1093/bioinformatics/bts199
Kelm DH, 2011, P ROY SOC B-BIOL SCI, V278, P3490, DOI 10.1098/rspb.2011.0465
Kolmogorov M, 2019, NAT BIOTECHNOL, V37, P540, DOI 10.1038/s41587-019-0072-8
Korf I, 2004, BMC BIOINFORMATICS, V5, DOI 10.1186/1471-2105-5-59
Lau SKP, 2020, EMERG INFECT DIS, V26, P1542, DOI 10.3201/eid2607.200092
Lenartic M, 2017, J IMMUNOL, V198, P1531, DOI 10.4049/jimmunol.1600461
Lewin HA, 2018, P NATL ACAD SCI USA, V115, P4325, DOI 10.1073/pnas.1720115115
Liberles SD, 2006, NATURE, V442, P645, DOI 10.1038/nature05066
Loo TM, 2017, CANCER DISCOV, V7, P522, DOI 10.1158/2159-8290.CD-16-0932
McHeyzer-Williams M, 2012, NAT REV IMMUNOL, V12, P24, DOI 10.1038/nri3128
Meakin PJ, 2015, PLOS ONE, V10, DOI 10.1371/journal.pone.0138872
Memish ZA, 2020, LANCET, V395, P1063, DOI 10.1016/S0140-6736(19)33221-0
Meng FX, 2016, SCI REP-UK, V6, DOI 10.1038/srep29960
Merad M, 2020, NAT REV IMMUNOL, V20, P355, DOI 10.1038/s41577-020-0331-4
Mi HY, 2019, NUCLEIC ACIDS RES, V47, pD419, DOI 10.1093/nar/gky1038
Miller MR, 2016, J INFECT DIS, V214, pS297, DOI 10.1093/infdis/jiw199
Minias P, 2019, GENOME BIOL EVOL, V11, P17, DOI 10.1093/gbe/evy253
Mollentze N, 2020, P NATL ACAD SCI USA, V117, P9423, DOI 10.1073/pnas.1919176117
Moratelli R, 2015, MEM I OSWALDO CRUZ, V110, P1
Muntasell A, 2010, BLOOD, V115, P5170, DOI 10.1182/blood-2009-11-256479
Nagaoka I, 2008, INT IMMUNOL, V20, P543, DOI 10.1093/intimm/dxn012
Ng JHJ, 2016, SCI REP-UK, V6, DOI 10.1038/srep21256
Niyonsaba F, 2010, J IMMUNOL, V184, P3526, DOI 10.4049/jimmunol.0900712
Nogueira R.M.R., 2018, NO UPDATED CHECKLIST
Nowak MD, 2021, MOL ECOL RESOUR, V21, P661, DOI 10.1111/1755-0998.13280
Olival KJ, 2017, NATURE, V546, P646, DOI 10.1038/nature22975
Papenfuss AT, 2012, BMC GENOMICS, V13, DOI 10.1186/1471-2164-13-261

Papkou A, 2019, P NATL ACAD SCI USA, V116, P923, DOI 10.1073/pnas.1810402116
Parham P, 2013, NAT REV IMMUNOL, V13, P133, DOI 10.1038/nri3370
Parker J, 2013, NATURE, V502, P228, DOI 10.1038/nature12511
Parthasarathy R, 1997, BRAIN RES, V778, P99, DOI 10.1016/S0006-8993(97)01042-1
Pavlovich SS, 2018, CELL, V173, P1098, DOI 10.1016/j.cell.2018.03.070
Pickering AM, 2015, J GERONTOL A-BIOL, V70, P791, DOI 10.1093/gerona/glu115
Pierce CA, 2020, SCI TRANSL MED, V12, DOI 10.1126/scitranslmed.abd5487
Podlutsky AJ, 2005, J GERONTOL A-BIOL, V60, P1366, DOI 10.1093/gerona/60.11.1366
Pomaznoy M, 2018, BMC BIOINFORMATICS, V19, DOI 10.1186/s12859-018-2533-3
Pond SLK, 2005, BIOINFORMATICS, V21, P676, DOI 10.1093/bioinformatics/bti079
POTTER JHT, 2021, MOL BIOL EVOL
Qurkhuli T, 2019, IMMUNOGENETICS, V71, P575, DOI 10.1007/s00251-019-01128-7
Rafikov R, 2019, AM J PHYSIOL-LUNG C, V316, pL428, DOI 10.1152/ajplung.00393.2018
Rapista A, 2011, RETROVIROLOGY, V8, DOI 10.1186/1742-4690-8-45
Ravel A, 2016, GEODIVERSITAS, V38, P355, DOI 10.5252/g2016n3a3
Reddy ST, 2010, NAT BIOTECHNOL, V28, P965, DOI 10.1038/nbt.1673
Rhie A., 2020, Towards complete and error-free genome assemblies of all vertebrate species, DOI DOI 10.1101/2020.05.22.110833
Rojas D, 2018, ECOL LETT, V21, P402, DOI 10.1111/ele.12911
Salmon AB, 2009, FASEB J, V23, P2317, DOI 10.1096/fj.08-122523
Santos PSC, 2016, SCI REP-UK, V6, DOI 10.1038/srep38490
Scheben A., 2020, UNRAVELING MOL MECH, DOI 10.1101/2020.09.09.290502
Schountz T, 2017, FRONT IMMUNOL, V8, DOI 10.3389/fimmu.2017.01098
Schroder K, 2004, J LEUKOCYTE BIOL, V75, P163, DOI 10.1189/jlb.0603252
Seim I, 2013, NAT COMMUN, V4, DOI 10.1038/ncomms3212
Semple F, 2012, J INNATE IMMUN, V4, P337, DOI 10.1159/000336619
Sharma V, 2018, PLOS BIOL, V16, DOI 10.1371/journal.pbio.2005293
Sharma V, 2018, NAT COMMUN, V9, DOI 10.1038/s41467-018-03667-1
Shen B, 2013, PLOS ONE, V8, DOI 10.1371/journal.pone.0062307
Shen B, 2012, PLOS ONE, V7, DOI 10.1371/journal.pone.0033197
Shlomchik M.J., 2001, IMMUNOBIOLOGY IMMUNE
Shultz AJ, 2019, ELIFE, V8, DOI 10.7554/eLife.41815
Simmons NB, 2008, NATURE, V451, P818, DOI 10.1038/nature06549
Sironi M, 2015, NAT REV GENET, V16, P224, DOI 10.1038/nrg3905
Slavuljica I, 2011, FRONT IMMUNOL, V2, DOI 10.3389/fimmu.2011.00085
Smith MD, 2015, MOL BIOL EVOL, V32, P1342, DOI 10.1093/molbev/msv022
Stamatakis A, 2014, BIOINFORMATICS, V30, P1312, DOI 10.1093/bioinformatics/btu033
Stanke M, 2003, BIOINFORMATICS, V19, pII215, DOI 10.1093/bioinformatics/btg1080

Stolberg VR, 2014, AM J PATHOL, V184, P454, DOI 10.1016/j.ajpath.2013.10.017
Storch Gerhard, 2002, Palaeontologische Zeitschrift, V76, P189
Subudhi S, 2019, VIRUSES-BASEL, V11, DOI 10.3390/v11020192
Suyama M, 2006, NUCLEIC ACIDS RES, V34, pW609, DOI 10.1093/nar/gkl315
Takada A, 2008, J IMMUNOL, V180, P1678, DOI 10.4049/jimmunol.180.3.1678
Teeling EC, 2005, SCIENCE, V307, P580, DOI 10.1126/science.1105113
Teeling EC, 2018, ANNU REV ANIM BIOSCI, V6, P23, DOI 10.1146/annurev-animal-022516-022811
Towner JS, 2007, PLOS ONE, V2, DOI 10.1371/journal.pone.0000764
Tsagkogeorga G, 2017, SCI REP-UK, V7, DOI 10.1038/s41598-017-00132-9
Tu JB, 2015, EVOL BIOINFORM, V11, DOI 10.4137/EBO.S25580
Vadstrup K, 2017, EXP MOL PATHOL, V103, P56, DOI 10.1016/j.yexmp.2017.06.010
van Oosterhout C, 2009, P ROY SOC B-BIOL SCI, V276, P657, DOI 10.1098/rspb.2008.1299
Voigt CC, 2007, FUNCT ECOL, V21, P913, DOI 10.1111/j.1365-2435.2007.01321.x
Vosseberg J, 2021, NAT ECOL EVOL, V5, DOI 10.1038/s41559-020-01320-z
Wang K, 2020, MOL ECOL, V29, P4366, DOI 10.1111/mec.15542
Wang P, 2020, GENOME BIOL, V21, DOI 10.1186/s13059-020-01959-8
Welch KC, 2008, J EXP BIOL, V211, P310, DOI 10.1242/jeb.012252
Wiens ME, 2014, PLOS PATHOG, V10, DOI 10.1371/journal.ppat.1004186
Wilkinson GS, 2019, BIOL LETTERS, V15, DOI 10.1098/rsbl.2018.0860
Wortham BW, 2012, J IMMUNOL, V188, P4468, DOI 10.4049/jimmunol.1102643
Xiao YJ, 2004, BMC GENOMICS, V5, DOI 10.1186/1471-2164-5-56
Xie JZ, 2018, CELL HOST MICROBE, V23, P297, DOI 10.1016/j.chom.2018.01.006
Xu D, 2020, FRONT IMMUNOL, V11, DOI 10.3389/fimmu.2020.00764
Yang ZH, 2005, MOL BIOL EVOL, V22, P1107, DOI 10.1093/molbev/msi097
Yang ZH, 2007, MOL BIOL EVOL, V24, P1586, DOI 10.1093/molbev/msm088
Yates AD, 2020, NUCLEIC ACIDS RES, V48, pD682, DOI 10.1093/nar/gkz966
Yohe LR, 2019, JOVE-J VIS EXP, DOI 10.3791/59505
You XX, 2014, NAT COMMUN, V5, DOI 10.1038/ncomms6594
Yu GC, 2017, METHODS ECOL EVOL, V8, P28, DOI 10.1111/2041-210X.12628
Zafirova B, 2009, IMMUNITY, V31, P270, DOI 10.1016/j.immuni.2009.06.017
Mendoza MLZ, 2018, NAT ECOL EVOL, V2, P659, DOI 10.1038/s41559-018-0476-8
Zhang GJ, 2013, SCIENCE, V339, P456, DOI 10.1126/science.1230835
Zheng DP, 2020, CELL DISCOV, V6, DOI 10.1038/s41421-020-0167-x
Zhi L, 2010, BIOCHEMISTRY-US, V49, P2443, DOI 10.1021/bi902130j
Zhou P., 2020, BATS VIRUSES CURRENT, P135, DOI

[10.21775/9781912530144.09, DOI 10.21775/9781912530144.09]
   Zhou P, 2016, P NATL ACAD SCI USA, V113, P2696, DOI 10.1073/
pnas.1518240113
   Zhou XM, 2020, CELL REP, V32, DOI 10.1016/j.celrep.2020.107949
   Zimin AV, 2013, BIOINFORMATICS, V29, P2669, DOI 10.1093/
bioinformatics/btt476
NR 181
TC 42
Z9 45
U1 4
U2 48
PU WILEY
PI HOBOKEN
PA 111 RIVER ST, HOBOKEN 07030-5774, NJ USA
SN 0962-1083
EI 1365-294X
J9 MOL ECOL
JI Mol. Ecol.
PD DEC
PY 2021
VL 30
IS 23
SI SI
BP 6449
EP 6467
DI 10.1111/mec.16027
EA JUL 2021
PG 19
WC Biochemistry & Molecular Biology; Ecology; Evolutionary Biology
WE Science Citation Index Expanded (SCI-EXPANDED)
SC Biochemistry & Molecular Biology; Environmental Sciences &
Ecology;
   Evolutionary Biology
GA XE8NF
UT WOS:000669313500001
PM 34146369
OA Green Submitted, Green Accepted
DA 2024-10-21
ER

PT J
AU Liu, BY
   Chen, XL
   Zhou, L
   Li, JY
   Wang, D
   Yang, WT
   Wu, H
   Yao, JY
   Yang, GL
   Wang, CF
   Feng, J
   Jiang, TL
AF Liu, Boyu

Chen, Xiaolei
   Zhou, Lei
   Li, Junyi
   Wang, Dan
   Yang, Wentao
   Wu, Hui
   Yao, Jiyuan
   Yang, Guilian
   Wang, Chunfeng
   Feng, Jiang
   Jiang, Tinglei
TI The gut microbiota of bats confers tolerance to influenza virus (H1N1)
   infection in mice
SO TRANSBOUNDARY AND EMERGING DISEASES
LA English
DT Article
DE bat; Chiroptera; immunity; isoflavone; microbiome; viral infection
ID IMMUNE-RESPONSES; GENE-EXPRESSION; IN-VITRO; A VIRUS; HOST; METABOLISM;
   RESERVOIRS; EVOLUTION; SITE; CORONAVIRUS
AB Pathogens from wild animals cause approximately 60% of emerging infectious diseases (EIDs). Studies on the immune systems of natural hosts are helpful for preventing the spread of EIDs. Bats are natural hosts for many emerging infectious pathogens and have a unique immune system that often coexists with pathogens without infection. Previous studies have shown that some genes and proteins may help bats fight virus infection, but little is known about the function of the bat gut microbiome on immunity. Here, we transplanted gut microbiota from wild bats (Great Himalayan Leaf-nosed bats, Hipposideros armiger) into antibiotic-treated mice, and found that the gut microbiota from bats regulated the immune system faster than mice after antibiotic treatment. Moreover, we infected mice with H1N1, and found that the gut microbiota of bats could effectively protect mice, leading to decreased inflammatory response and increased survival rate. Finally, metabolomics analysis showed that the gut microbiota of bats produced more flavonoid and isoflavones. Our results demonstrate that the quick-start innate immune response endowed by bat gut microbiota and the regulatory and antiviral effects of gut microbiota metabolites successfully ensured mouse survival after viral challenge. To our knowledge, our study was the first to use fecal microbiota transplantation (FMT) to transplant the gut microbiota of bats into mice, and the first to provide evidence that the gut microbiota of bats confers tolerance to viral infections.
C1 [Liu, Boyu; Chen, Xiaolei; Zhou, Lei; Li, Junyi; Wang, Dan; Yang, Wentao; Yao, Jiyuan; Yang, Guilian; Wang, Chunfeng] Jilin Agr Univ, Coll Vet Med, Changchun, Peoples R China.
   [Wu, Hui; Feng, Jiang] Jilin Agr Univ, Coll Life Sci, Changchun, Peoples R China.
   [Chen, Xiaolei; Li, Junyi; Wang, Dan; Yang, Wentao; Yang, Guilian; Wang, Chunfeng] Jilin Agr Univ, Jilin Prov Engn Res Ctr Anim Probiot, Changchun, Peoples R China.

[Feng, Jiang; Jiang, Tinglei] Northeast Normal Univ, Jilin Prov Key Lab Anim Resource Conservat & Util, Changchun, Peoples R China.
C3 Jilin Agricultural University; Jilin Agricultural University; Jilin
Agricultural University; Northeast Normal University - China
RP Feng, J (corresponding author), Jilin Agr Univ, Coll Life Sci, Changchun, Peoples R China.; Jiang, TL (corresponding author), Northeast Normal Univ, Jilin Prov Key Lab Anim Resource Conservat & Util, Changchun, Peoples R China.
EM fengj@nenu.edu.cn; jiangtl730@nenu.edu.cn
RI Chen, Xiaolei/ABD-6837-2021; Jiang, Tinglei/GQQ-9485-2022; Liu, Song/KCX-6842-2024
OI Li, Junyi/0000-0001-8435-2262; Liu, Haibo/0000-0002-4213-2883
FU National Natural Science Foundation of China [31922050, 32071492, 32171489]; Jilin Province Science and Technology Development Project
[20200201186JC]
FX National Natural Science Foundation of China, Grant/Award Numbers:
31922050, 32071492, 32171489; The Jilin Province Science and Technology
Development Project, Grant/Award Number: 20200201186JC
CR Abt MC, 2012, IMMUNITY, V37, P158, DOI 10.1016/j.immuni.2012.04.011
Agus A, 2018, CELL HOST MICROBE, V23, P716, DOI 10.1016/j.chom.2018.05.003
Ahn M, 2019, NAT MICROBIOL, V4, P789, DOI 10.1038/s41564-019-0371-3
Ahn M, 2016, SCI REP-UK, V6, DOI 10.1038/srep21722
Allen T, 2017, NAT COMMUN, V8, DOI 10.1038/s41467-017-00923-8
Alvarez P, 2006, NUTRITION, V22, P913, DOI 10.1016/j.nut.2005.12.012
Andres A, 2009, J NUTR BIOCHEM, V20, P563, DOI 10.1016/j.jnutbio.2009.04.004
Arabyan E, 2018, ANTIVIR RES, V156, P128, DOI 10.1016/j.antiviral.2018.06.014
Baker ML, 2013, ZOONOSES PUBLIC HLTH, V60, P104, DOI 10.1111/j.1863-2378.2012.01528.x
Ball HC, 2018, PLOS ONE, V13, DOI 10.1371/journal.pone.0196154
Banerjee A, 2020, TRENDS MICROBIOL, V28, P703, DOI 10.1016/j.tim.2020.04.006
Banerjee A, 2020, FRONT IMMUNOL, V11, DOI 10.3389/fimmu.2020.00026
Bernatoniene J, 2021, INT J MOL SCI, V22, DOI 10.3390/ijms22115656
Bokulich NA, 2018, MICROBIOME, V6, DOI 10.1186/s40168-018-0470-z
Bolyen E, 2019, NAT BIOTECHNOL, V37, P852, DOI 10.1038/s41587-019-0209-9
Brook CE, 2015, TRENDS MICROBIOL, V23, P172, DOI 10.1016/j.tim.2014.12.004
Burrello C, 2018, NAT COMMUN, V9, DOI 10.1038/s41467-018-07359-8
Calisher CH, 2006, CLIN MICROBIOL REV, V19, P531, DOI 10.1128/CMR.00017-06
Caviedes-Vidal E, 2007, P NATL ACAD SCI USA, V104, P19132, DOI

10.1073/pnas.0703159104
Chan JFW, 2013, TRENDS MICROBIOL, V21, P544, DOI 10.1016/j.tim.2013.05.005
Ciminski K, 2019, NAT MICROBIOL, V4, P2298, DOI 10.1038/s41564-019-0556-9
Biancatelli RMLC, 2020, FRONT IMMUNOL, V11, DOI 10.3389/fimmu.2020.01451
Cryan JF, 2019, PHYSIOL REV, V99, P1877, DOI 10.1152/physrev.00018.2018
De La Cruz-Rivera PC, 2018, J IMMUNOL, V200, P209, DOI 10.4049/jimmunol.1701214
Dong D, 2017, MOL BIOL EVOL, V34, P20, DOI 10.1093/molbev/msw231
Ekmekciu I, 2017, FRONT IMMUNOL, V8, DOI 10.3389/fimmu.2017.00397
Flerlage T, 2021, NAT REV MICROBIOL, V19, P425, DOI 10.1038/s41579-021-00542-7
Ganai AA, 2015, BIOMED PHARMACOTHER, V76, P30, DOI 10.1016/j.biopha.2015.10.026
Ge XY, 2013, NATURE, V503, P535, DOI 10.1038/nature12711
Ge XY, 2012, J VIROL, V86, P4620, DOI 10.1128/JVI.06671-11
Gómez-Zorita S, 2020, NUTRIENTS, V12, DOI 10.3390/nu12123853
Gong LX, 2021, MSPHERE, V6, DOI 10.1128/mSphere.00467-21
Guo J, 2013, RETROVIROLOGY, V10, DOI 10.1186/1742-4690-10-62
Halpin K, 2000, J GEN VIROL, V81, P1927, DOI 10.1099/0022-1317-81-8-1927
He G. D., 2020, PNEUMONIA OUTBREAK A, V27, P609
Herlemann DPR, 2011, ISME J, V5, P1571, DOI 10.1038/ismej.2011.41
Hölzer M, 2019, ISCIENCE, V19, P647, DOI 10.1016/j.isci.2019.08.016
Irving AT, 2021, NATURE, V589, P363, DOI 10.1038/s41586-020-03128-0
Jin YB, 2018, APPL MICROBIOL BIOT, V102, P8403, DOI 10.1007/s00253-018-9205-0
Jones KE, 2008, NATURE, V451, P990, DOI 10.1038/nature06536
Karakus U, 2019, NATURE, V567, P109, DOI 10.1038/s41586-019-0955-3
Kepler TB, 2010, BMC GENOMICS, V11, DOI 10.1186/1471-2164-11-444
KLITE PD, 1965, J BACTERIOL, V90, P375, DOI 10.1128/JB.90.2.375-379.1965
Kong SS, 2018, BIOMED RES INT, V2018, DOI 10.1155/2018/2819154
Kumaki Y, 2011, ANTIVIR RES, V92, P329, DOI 10.1016/j.antiviral.2011.09.001
Le Roy T, 2019, FRONT MICROBIOL, V9, DOI 10.3389/fmicb.2018.03289
Leroy EM, 2005, NATURE, V438, P575, DOI 10.1038/438575a
Ley RE, 2008, SCIENCE, V320, P1647, DOI 10.1126/science.1155725
Li BW, 2014, BIOORGAN MED CHEM, V22, P3146, DOI 10.1016/j.bmc.2014.04.016
Li EG, 2000, J BIOL CHEM, V275, P14729, DOI 10.1074/jbc.275.19.14729
Li WD, 2005, SCIENCE, V310, P676, DOI 10.1126/science.1118391
Li Y, 2016, NUTRIENTS, V8, DOI 10.3390/nu8030167
Liu C, 2019, FRONT MICROBIOL, V10, DOI 10.3389/fmicb.2019.01116
Luis AD, 2013, P ROY SOC B-BIOL SCI, V280, DOI 10.1098/rspb.2012.2753
Lyu SY, 2005, ARCH PHARM RES, V28, P1293, DOI 10.1007/BF02978215

Ma C, 2018, MICROBIOME, V6, DOI 10.1186/s40168-018-0578-1
Ma Wenjun, 2008, J Mol Genet Med, V3, P158
Magoc T, 2011, BIOINFORMATICS, V27, P2957, DOI 10.1093/bioinformatics/btr507
Malmlov A, 2019, PLOS NEGLECT TROP D, V13, DOI 10.1371/journal.pntd.0007071
Mandl JN, 2018, FRONT IMMUNOL, V9, DOI 10.3389/fimmu.2018.02112
Marchesi JR, 2016, GUT, V65, P330, DOI 10.1136/gutjnl-2015-309990
Michaudel C, 2020, CELL METAB, V32, P514, DOI 10.1016/j.cmet.2020.09.004
Miles SL, 2014, NUTR REV, V72, P720, DOI 10.1111/nure.12152
Morens DM, 2012, MBIO, V3, DOI 10.1128/mBio.00494-12
MORSE SS, 1995, EMERG INFECT DIS, V1, P7, DOI 10.3201/eid0101.950102
Nagai E, 2019, PLANT FOOD HUM NUTR, V74, P538, DOI 10.1007/s11130-019-00773-3
Nair MPN, 2002, BBA-MOL CELL RES, V1593, P29, DOI 10.1016/S0167-4889(02)00328-2
Neumann G, 2009, NATURE, V459, P931, DOI 10.1038/nature08157
Olival KJ, 2017, NATURE, V546, P646, DOI 10.1038/nature22975
Pandey A, 2010, CYTOKINE, V49, P114, DOI 10.1016/j.cyto.2009.08.015
Paramsothy S, 2017, LANCET, V389, P1218, DOI 10.1016/S0140-6736(17)30182-4
Pavlovich SS, 2018, CELL, V173, P1098, DOI 10.1016/j.cell.2018.03.070
Periasamy P, 2019, FRONT IMMUNOL, V10, DOI 10.3389/fimmu.2019.00489
Pickard JM, 2017, IMMUNOL REV, V279, P70, DOI 10.1111/imr.12567
Pike J, 2014, P NATL ACAD SCI USA, V111, P18519, DOI 10.1073/pnas.1412661112
Poole DS, 2014, J VIROL, V88, P12572, DOI 10.1128/JVI.01857-14
Price ER, 2015, PHYSIOLOGY, V30, P69, DOI 10.1152/physiol.00020.2014
Pruesse E, 2007, NUCLEIC ACIDS RES, V35, P7188, DOI 10.1093/nar/gkm864
Pulendran B, 2020, SCIENCE, V369, P1582, DOI 10.1126/science.aay4014
Rooks MG, 2016, NAT REV IMMUNOL, V16, P341, DOI 10.1038/nri.2016.42
Rosshart SP, 2017, CELL, V171, P1015, DOI 10.1016/j.cell.2017.09.016
Rouse BT, 2010, NAT REV IMMUNOL, V10, P514, DOI 10.1038/nri2802
Segata N, 2011, GENOME BIOL, V12, DOI 10.1186/gb-2011-12-6-r60
Shaw AE, 2017, PLOS BIOL, V15, DOI 10.1371/journal.pbio.2004086
Shi CW, 2020, FRONT MICROBIOL, V11, DOI 10.3389/fmicb.2020.607735
Sommer F, 2016, CELL REP, V14, P1655, DOI 10.1016/j.celrep.2016.01.026
Song SJ, 2020, MBIO, V11, DOI 10.1128/mBio.02901-19
Stantchev TS, 2007, VIRUS RES, V123, P178, DOI 10.1016/j.virusres.2006.09.004
Steed AL, 2017, SCIENCE, V357, P498, DOI 10.1126/science.aam5336
Subudhi S, 2019, VIRUSES-BASEL, V11, DOI 10.3390/v11020192
Sun DL, 2020, FRONT MICROBIOL, V11, DOI 10.3389/fmicb.2020.01040

Sun HL, 2020, P NATL ACAD SCI USA, V117, P17204, DOI 10.1073/pnas.1921186117
Swanepoel R, 1996, EMERG INFECT DIS, V2, P321, DOI 10.3201/eid0204.960407
Taubenberger JK, 2008, ANNU REV PATHOL-MECH, V3, P499, DOI 10.1146/annurev.pathmechdis.3.121806.154316
Tong SX, 2012, P NATL ACAD SCI USA, V109, P4269, DOI 10.1073/pnas.1116200109
Trompette A, 2014, NAT MED, V20, P159, DOI 10.1038/nm.3444
Valdes Ana M, 2018, BMJ, V361, pk2179, DOI 10.1136/bmj.k2179
van der Giessen J, 2020, GUT, V69, P473, DOI 10.1136/gutjnl-2019-318263
van Sadelhoff JHJ, 2020, FRONT IMMUNOL, V11, DOI 10.3389/fimmu.2020.01007
Watanabe S, 2010, EMERG INFECT DIS, V16, P1217, DOI 10.3201/eid1608.100208
Wu WJ, 2016, VIRUSES-BASEL, V8, DOI 10.3390/v8010006
Wu ZQ, 2016, ISME J, V10, P609, DOI 10.1038/ismej.2015.138
Wynne JW, 2016, J IMMUNOL, V196, P4468, DOI 10.4049/jimmunol.1502062
Xiao YH, 2019, PEERJ, V7, DOI 10.7717/peerj.6844
Xie JZ, 2018, CELL HOST MICROBE, V23, P297, DOI 10.1016/j.chom.2018.01.006
Xu T, 2006, AM J RESP CRIT CARE, V174, P1011, DOI 10.1164/rccm.200511-1751OC
Yuan BF, 2018, ANAL CHEM, V90, P3512, DOI 10.1021/acs.analchem.7b05355
Zhang CH, 2010, ISME J, V4, P232, DOI 10.1038/ismej.2009.112
Zhang GJ, 2013, SCIENCE, V339, P456, DOI 10.1126/science.1230835
Zhong GX, 2020, J VIROL, V94, DOI 10.1128/JVI.01416-19
Zhou Hong, 2020, Curr Biol, V30, P2196, DOI 10.1016/j.cub.2020.05.023
Zhou P, 2016, P NATL ACAD SCI USA, V113, P2696, DOI 10.1073/pnas.1518240113
Zhou P, 2011, J IMMUNOL, V186, P3138, DOI 10.4049/jimmunol.1003115
NR 113
TC 6
Z9 8
U1 6
U2 44
PU WILEY-HINDAWI
PI LONDON
PA ADAM HOUSE, 3RD FL, 1 FITZROY SQ, LONDON, WIT 5HE, ENGLAND
SN 1865-1674
EI 1865-1682
J9 TRANSBOUND EMERG DIS
JI Transbound. Emerg. Dis.
PD SEP
PY 2022
VL 69
IS 5
BP E1469
EP E1487

DI 10.1111/tbed.14478
EA FEB 2022
PG 19
WC Infectious Diseases; Veterinary Sciences
WE Science Citation Index Expanded (SCI-EXPANDED)
SC Infectious Diseases; Veterinary Sciences
GA 4V0LT
UT WOS:000761315000001
PM 35156318
OA gold
DA 2024-10-21
ER

PT J
AU Jebb, D
   Huang, ZX
   Pippel, M
   Hughes, GM
   Lavrichenko, K
   Devanna, P
   Winkler, S
   Jermiin, LS
   Skirmuntt, EC
   Katzourakis, A
   Burkitt-Gray, L
   Ray, DA
   Sullivan, KAM
   Roscito, JG
   Kirilenko, BM
   Davalos, LM
   Corthals, AP
   Power, ML
   Jones, G
   Ransome, RD
   Dechmann, DKN
   Locatelli, AG
   Puechmaille, SJ
   Fedrigo, O
   Jarvis, ED
   Hiller, M
   Vernes, SC
   Myers, EW
   Teeling, EC
AF Jebb, David
   Huang, Zixia
   Pippel, Martin
   Hughes, Graham M.
   Lavrichenko, Ksenia
   Devanna, Paolo
   Winkler, Sylke
   Jermiin, Lars S.
   Skirmuntt, Emilia C.
   Katzourakis, Aris
   Burkitt-Gray, Lucy

Ray, David A.
Sullivan, Kevin A. M.
Roscito, Juliana G.
Kirilenko, Bogdan M.
Davalos, Liliana M.
Corthals, Angelique P.
Power, Megan L.
Jones, Gareth
Ransome, Roger D.
Dechmann, Dina K. N.
Locatelli, Andrea G.
Puechmaille, Sebastien J.
Fedrigo, Olivier
Jarvis, Erich D.
Hiller, Michael
Vernes, Sonja C.
Myers, Eugene W.
Teeling, Emma C.
TI Six reference-quality genomes reveal evolution of bat adaptations
SO NATURE
LA English
DT Article
ID PHYLOGENETIC ANALYSIS; HEARING-LOSS; CD-HIT; ALIGNMENT; PROTEIN; TOOL;
FAMILY; CLASSIFICATION; DUPLICATION; ANNOTATION
AB Bats possess extraordinary adaptations, including flight, echolocation, extreme longevity and unique immunity. High-quality genomes are crucial for understanding the molecular basis and evolution of these traits. Here we incorporated long-read sequencing and state-of-the-art scaffolding protocols(1)to generate, to our knowledge, the first reference-quality genomes of six bat species (Rhinolophus ferrumequinum,Rousettus aegyptiacus,Phyllostomus discolor,Myotis myotis,Pipistrellus kuhliiandMolossus molossus). We integrated gene projections from our 'Tool to infer Orthologs from Genome Alignments' (TOGA) software with de novo and homology gene predictions as well as short- and long-read transcriptomics to generate highly complete gene annotations. To resolve the phylogenetic position of bats within Laurasiatheria, we applied several phylogenetic methods to comprehensive sets of orthologous protein-coding and noncoding regions of the genome, and identified a basal origin for bats within Scrotifera. Our genome-wide screens revealed positive selection on hearing-related genes in the ancestral branch of bats, which is indicative of laryngeal echolocation being an ancestral trait in this clade. We found selection and loss of immunity-related genes (including pro-inflammatory NF-kappa B regulators) and expansions of anti-viral APOBEC3 genes, which highlights molecular mechanisms that may contribute to the exceptional immunity of bats. Genomic integrations of diverse viruses provide a genomic record of historical tolerance to viral infection in bats. Finally, we found and experimentally validated bat-specific variation in microRNAs, which may regulate bat-specific gene-expression programs. Our reference-quality bat genomes provide the resources required to uncover and validate the genomic basis of adaptations of bats, and stimulate new avenues of

research that are directly relevant to human health and disease(1).
Reference-quality genomes for six bat species shed light on the phylogenetic position of Chiroptera, and provide insight into the genetic underpinnings of the unique adaptations of this clade.

C1 [Jebb, David; Pippel, Martin; Winkler, Sylke; Roscito, Juliana G.; Kirilenko, Bogdan M.; Hiller, Michael; Myers, Eugene W.] Max Planck Inst Mol Cell Biol & Genet, Dresden, Germany.
[Jebb, David; Roscito, Juliana G.; Kirilenko, Bogdan M.; Hiller, Michael] Max Planck Inst Phys Komplexer Syst, Dresden, Germany.
[Jebb, David; Pippel, Martin; Roscito, Juliana G.; Kirilenko, Bogdan M.; Hiller, Michael; Myers, Eugene W.] Ctr Syst Biol Dresden, Dresden, Germany.
[Huang, Zixia; Hughes, Graham M.; Jermiin, Lars S.; Power, Megan L.; Locatelli, Andrea G.; Teeling, Emma C.] Univ Coll Dublin, Sch Biol & Environm Sci, Dublin, Ireland.
[Lavrichenko, Ksenia; Devanna, Paolo; Vernes, Sonja C.] Max Planck Inst Psycholinguist, Neurogenet Vocal Commun Grp, Nijmegen, Netherlands.
[Jermiin, Lars S.] Australian Natl Univ, Res Sch Biol, Canberra, ACT, Australia.
[Jermiin, Lars S.] Univ Coll Dublin, Earth Inst, Dublin, Ireland.
[Skirmuntt, Emilia C.; Katzourakis, Aris] Univ Oxford, Dept Zool, Peter Medawar Bldg Pathogen Res, Oxford, England.
[Burkitt-Gray, Lucy] Univ Coll Dublin, Conway Inst Biomol & Biomed Sci, Dublin, Ireland.
[Ray, David A.; Sullivan, Kevin A. M.] Texas Tech Univ, Dept Biol Sci, Lubbock, TX 79409 USA.
[Davalos, Liliana M.] SUNY Stony Brook, Dept Ecol & Evolut, Stony Brook, NY 11794 USA.
[Davalos, Liliana M.] SUNY Stony Brook, Consortium Interdisciplinary Environm Res, Stony Brook, NY 11794 USA.
[Corthals, Angelique P.] John Jay Coll Criminal Justice, Dept Sci, New York, NY USA.
[Jones, Gareth; Ransome, Roger D.] Univ Bristol, Sch Biol Sci, Bristol, Avon, England.
[Dechmann, Dina K. N.] Max Planck Inst Anim Behav, Dept Migrat, Radolfzell am Bodensee, Germany.
[Dechmann, Dina K. N.] Univ Konstanz, Dept Biol, Constance, Germany.
[Dechmann, Dina K. N.] Smithsonian Trop Res Inst, Panama City, Panama.
[Puechmaille, Sebastien J.] Univ Montpellier, ISEM, Montpellier, France.
[Puechmaille, Sebastien J.] Univ Greifswald, Zool Inst & Museum, Greifswald, Germany.
[Fedrigo, Olivier; Jarvis, Erich D.] Rockefeller Univ, Vertebrate Genomes Lab, 1230 York Ave, New York, NY 10021 USA.
[Jarvis, Erich D.] Rockefeller Univ, Lab Neurogenet Language, 1230 York Ave, New York, NY 10021 USA.
[Jarvis, Erich D.] Howard Hughes Med Inst, Chevy Chase, MD USA.
[Vernes, Sonja C.] Donders Inst Brain Cognit & Behav, Nijmegen, Netherlands.
[Myers, Eugene W.] Tech Univ Dresden, Fac Comp Sci, Dresden, Germany.

C3 Max Planck Society; Max Planck Society; University College
Dublin; Max
Planck Society; Australian National University; University
College
Dublin; University of Oxford; University College Dublin; Texas
Tech
University System; Texas Tech University; State University of New
York
(SUNY) System; Stony Brook University; State University of New
York
(SUNY) System; Stony Brook University; City University of New
York
(CUNY) System; University of Bristol; Max Planck Society;
University of
Konstanz; Smithsonian Institution; Smithsonian Tropical Research
Institute; Centre National de la Recherche Scientifique (CNRS);
Institut
de Recherche pour le Developpement (IRD); Universite de
Montpellier;
Universitat Greifswald; Rockefeller University; Rockefeller
University;
Howard Hughes Medical Institute; Radboud University Nijmegen;
Technische
Universitat Dresden
RP Hiller, M; Myers, EW (corresponding author), Max Planck Inst Mol
Cell Biol & Genet, Dresden, Germany.; Hiller, M (corresponding
author), Max Planck Inst Phys Komplexer Syst, Dresden, Germany.;
Hiller, M; Myers, EW (corresponding author), Ctr Syst Biol Dresden,
Dresden, Germany.; Teeling, EC (corresponding author), Univ Coll
Dublin, Sch Biol & Environm Sci, Dublin, Ireland.; Vernes, SC
(corresponding author), Max Planck Inst Psycholinguist, Neurogenet
Vocal Commun Grp, Nijmegen, Netherlands.; Vernes, SC (corresponding
author), Donders Inst Brain Cognit & Behav, Nijmegen, Netherlands.;
Myers, EW (corresponding author), Tech Univ Dresden, Fac Comp Sci,
Dresden, Germany.
EM hiller@mpi-cbg.de; sonja.vernes@mpi.nl; gene@mpi-cbg.de;
emma.teeling@ucd.ie
RI Huang, Zixia/AAO-6936-2021; Skirmuntt, Emilia/HKV-2122-2023;
Locatelli,
Andrea Giacomo/HPD-3342-2023; Katzourakis, Aris/K-4488-2012;
Davalos,
Liliana/AEE-7879-2022; Juan, David/AAA-2614-2020; Dechmann,
Dina/A-1827-2010; Jarvis, Erich/A-2319-2008; Pippel,
Martin/AGA-8156-2022; roscito, juliana/AAI-8461-2021; Vernes,
Sonja/E-8454-2012; Jermiin, Lars/C-2458-2009; Puechmaille,
Sebastien/D-1612-2010
OI Skirmuntt, Emilia/0000-0002-7302-0665; Vernes,
Sonja/0000-0003-0305-4584; Locatelli, Andrea
Giacomo/0000-0002-3763-7967; Jermiin, Lars/0000-0002-9619-3809;
Huang,
Zixia/0000-0002-1298-0486; Corthals, Angelique/
0000-0002-5610-2992;
Burkitt-Gray, Lucy/0000-0001-5170-0638; Puechmaille,
Sebastien/0000-0001-9517-5775; Ray, David/0000-0002-3340-3987;

Jones,
Gareth/0000-0002-1904-3735; Davalos, Liliana/0000-0002-4327-7697;
Pippel, Martin/0000-0002-8134-5929; Power, Megan/
0000-0001-7402-3254;
Teeling, Emma/0000-0002-3309-1346
CR Abrusán G, 2009, BIOINFORMATICS, V25, P1329, DOI 10.1093/
bioinformatics/btp084
Aken BL, 2016, DATABASE-OXFORD, DOI 10.1093/database/baw093
ALTSCHUL SF, 1990, J MOL BIOL, V215, P403, DOI 10.1016/
S0022-2836(05)80360-2
[Anonymous], 2018, NAT BIOTECHNOL, V36, P1121, DOI 10.1038/
nbt.4318
Banerjee A, 2020, FRONT MICROBIOL, V11, DOI 10.3389/
fmicb.2020.00733
Barker BR, 2011, CURR OPIN IMMUNOL, V23, P591, DOI 10.1016/
j.coi.2011.07.005
Bejerano G, 2004, SCIENCE, V304, P1321, DOI 10.1126/
science.1098119
Bridgewood C, 2017, EXP DERMATOL, V26, P402, DOI 10.1111/
exd.13228
Chou J, 2015, BMC GENOMICS, V16, DOI 10.1186/1471-2164-16-S10-S2
De Bie T, 2006, BIOINFORMATICS, V22, P1269, DOI 10.1093/
bioinformatics/btl097
Devanna P, 2018, MOL PSYCHIATR, V23, P1375, DOI 10.1038/
mp.2017.30
Devanna P, 2018, HUM GENET, V137, P717, DOI 10.1007/
s00439-018-1925-9
Dong D, 2017, MOL BIOL EVOL, V34, P20, DOI 10.1093/molbev/msw231
Doronina L, 2017, GENOME RES, V27, P997, DOI 10.1101/
gr.210948.116
Eckalbar WL, 2016, NAT GENET, V48, P528, DOI 10.1038/ng.3537
Flo TH, 2004, NATURE, V432, P917, DOI 10.1038/nature03104
Foley NM, 2016, PHILOS T R SOC B, V371, DOI 10.1098/
rstb.2015.0140
Fu LM, 2012, BIOINFORMATICS, V28, P3150, DOI 10.1093/
bioinformatics/bts565
Gremme G, 2005, INFORM SOFTWARE TECH, V47, P965, DOI 10.1016/
j.infsof.2005.09.005
Gunn MD, 1998, NATURE, V391, P799, DOI 10.1038/35876
Haas BJ, 2008, GENOME BIOL, V9, DOI 10.1186/gb-2008-9-1-r7
Hase K, 2009, NATURE, V462, P226, DOI 10.1038/nature08529
Hayward A, 2013, P NATL ACAD SCI USA, V110, P20146, DOI 10.1073/
pnas.1315419110
Hayward JA, 2018, MOL BIOL EVOL, V35, P1626, DOI 10.1093/molbev/
msy048
Hecker N, 2020, GIGASCIENCE, V9, DOI 10.1093/gigascience/giz159
Heimberg AM, 2008, P NATL ACAD SCI USA, V105, P2946, DOI 10.1073/
pnas.0712259105
Ho SYW, 2004, SYST BIOL, V53, P623, DOI 10.1080/10635150490503035
Hoff KJ, 2016, BIOINFORMATICS, V32, P767, DOI 10.1093/
bioinformatics/btv661
Huang ZX, 2019, NAT ECOL EVOL, V3, P1110, DOI 10.1038/
s41559-019-0913-3
Jermiin LS, 2020, NAR GENOM BIOINFORM, V2, DOI 10.1093/nargab/

lqaa041
Jermiin LS, 2005, SCIENCE, V310, P1910, DOI 10.1126/science.1122440
Johnston A, 2011, J IMMUNOL, V186, P2613, DOI 10.4049/jimmunol.1003162
Jones G, 2013, FRONT PHYSIOL, V4, DOI 10.3389/fphys.2013.00117
Kalyaanamoorthy S, 2017, NAT METHODS, V14, P587, DOI [10.1038/NMETH.4285, 10.1038/nmeth.4285]
Kantarci S, 2007, NAT GENET, V39, P957, DOI 10.1038/ng2063
Katzourakis A, 2007, P NATL ACAD SCI USA, V104, P6261, DOI 10.1073/pnas.0700471104
Katzourakis A, 2010, PLOS GENET, V6, DOI 10.1371/journal.pgen.1001191
Kent WJ, 2003, P NATL ACAD SCI USA, V100, P11484, DOI 10.1073/pnas.1932072100
Kent WJ, 2002, GENOME RES, V12, P656, DOI [10.1101/gr.229202. Article published online before March 2002, 10.1101/gr.229202]
Kim D, 2019, NAT BIOTECHNOL, V37, P907, DOI 10.1038/s41587-019-0201-4
Kuo R. I., 2019, ILLUMINATING DARK SI, DOI DOI 10.1101/780015V1
Larsson A, 2014, BIOINFORMATICS, V30, P3276, DOI 10.1093/bioinformatics/btu531
Li WZ, 2006, BIOINFORMATICS, V22, P1658, DOI 10.1093/bioinformatics/btl158
Mi HY, 2005, NUCLEIC ACIDS RES, V33, pD284, DOI 10.1093/nar/gki078
Moran Y, 2017, NAT ECOL EVOL, V1, DOI 10.1038/s41559-016-0027
Münk C, 2012, BMC EVOL BIOL, V12, DOI 10.1186/1471-2148-12-71
Nawrocki EP, 2009, BIOINFORMATICS, V25, P1335, DOI 10.1093/bioinformatics/btp157
Nguyen Lam-Tung, 2015, Mol Biol Evol, V32, P268, DOI 10.1093/molbev/msu300
Nishida A, 2016, INFLAMM BOWEL DIS, V22, P303, DOI 10.1097/MIB.0000000000000654
Nishihara H, 2006, P NATL ACAD SCI USA, V103, P9929, DOI 10.1073/pnas.0603797103
Nowoshilow S, 2018, NATURE, V554, P50, DOI 10.1038/nature25458
Pace JK, 2007, GENOME RES, V17, P422, DOI 10.1101/gr.5826307
Parker J, 2013, NATURE, V502, P228, DOI 10.1038/nature12511
Pavlovich SS, 2018, CELL, V173, P1098, DOI 10.1016/j.cell.2018.03.070
Luong P, 2018, ELIFE, V7, DOI 10.7554/eLife.38539
Philippe H, 2011, PLOS BIOL, V9, DOI 10.1371/journal.pbio.1000602
Platt RN, 2016, GENOME BIOL EVOL, V8, P403, DOI 10.1093/gbe/evw009
Pond SLK, 2005, BIOINFORMATICS, V21, P676, DOI 10.1093/bioinformatics/bti079
Rhie A., 2020, Towards complete and error-free genome assemblies of all vertebrate species, DOI DOI 10.1101/2020.05.22.110833
Roper N, 2019, CELL REP, V26, P2651, DOI 10.1016/j.celrep.2019.02.028
Saddawi-Konefka R, 2016, CELL REP, V16, P2348, DOI 10.1016/j.celrep.2016.07.075
Salter JD, 2016, TRENDS BIOCHEM SCI, V41, P578, DOI 10.1016/

j.tibs.2016.05.001
Seim I, 2013, NAT COMMUN, V4, DOI 10.1038/ncomms3212
Sharma V, 2018, NAT COMMUN, V9, DOI 10.1038/s41467-018-03667-1
Sharma V, 2017, BIOINFORMATICS, V33, P3985, DOI 10.1093/bioinformatics/btx527
Sharma V, 2017, NUCLEIC ACIDS RES, V45, P8369, DOI 10.1093/nar/gkx554
Simmons N.B., 2020, Bat species of the world: A taxonomic and geographic database
Skirmuntt EC, 2019, VIRUS RES, V270, DOI 10.1016/j.virusres.2019.197645
Smit A.F.A., 2013, RepeatMasker Open-4.0
Smith MD, 2015, MOL BIOL EVOL, V32, P1342, DOI 10.1093/molbev/msv022
Springer M. S., 2019, ABBA BABA TEST INTRO, DOI DOI 10.1101/709477V1
Springer MS, 2018, SYST BIODIVERS, V16, P210, DOI 10.1080/14772000.2017.1401016
Stamatakis A, 2014, BIOINFORMATICS, V30, P1312, DOI 10.1093/bioinformatics/btu033
Stanke M, 2008, BIOINFORMATICS, V24, P637, DOI 10.1093/bioinformatics/btn013
Tabari Ehsan, 2017, Big Data Anal, V2, DOI 10.1186/s41044-016-0019-8
Tan J, 2013, AM J PATHOL, V183, P49, DOI 10.1016/j.ajpath.2013.03.009
Tarver JE, 2016, GENOME BIOL EVOL, V8, P330, DOI 10.1093/gbe/evv261
Taylor DJ, 2011, BMC EVOL BIOL, V11, DOI 10.1186/1471-2148-11-336
Teeling EC, 2005, SCIENCE, V307, P580, DOI 10.1126/science.1105113
Teeling EC, 2018, ANNU REV ANIM BIOSCI, V6, P23, DOI 10.1146/annurev-animal-022516-022811
Tischler G., 2017, NONHYBRID LONG READ, DOI DOI 10.1101/106252V1
Tischler G., 2019, COMPUTATIONAL INTELL, P103
Tsagkogeorga G, 2013, CURR BIOL, V23, P2262, DOI 10.1016/j.cub.2013.09.014
Vendelin J, 2006, HUM MOL GENET, V15, P2923, DOI 10.1093/hmg/ddl234
Vernes SC, 2020, PHILOS T R SOC B, V375, DOI 10.1098/rstb.2019.0061
Walsh T, 2010, AM J HUM GENET, V87, P101, DOI 10.1016/j.ajhg.2010.05.011
Wang Z, 2017, NAT ECOL EVOL, V1, DOI 10.1038/s41559-016-0021
Waterhouse Robert M, 2018, Mol Biol Evol, V35, P543, DOI 10.1093/molbev/msx319
Wei W, 2003, BIOCHEM BIOPH RES CO, V305, P981, DOI 10.1016/S0006-291X(03)00876-3
Wen M, 2018, GIGASCIENCE, V7, DOI 10.1093/gigascience/giy116
Wicker T, 2007, NAT REV GENET, V8, P973, DOI 10.1038/nrg2165
Wilkinson GS, 2019, BIOL LETTERS, V15, DOI 10.1098/rsbl.2018.0860
Xu XY, 2018, PLOS PATHOG, V14, DOI 10.1371/journal.ppat.1007072
Yang JY, 2015, NAT METHODS, V12, P7, DOI 10.1038/nmeth.3213
Yang ZH, 2007, MOL BIOL EVOL, V24, P1586, DOI 10.1093/molbev/

msm088
   Mendoza MLZ, 2018, NAT ECOL EVOL, V2, P659, DOI 10.1038/
s41559-018-0476-8
   Zhang GJ, 2013, SCIENCE, V339, P456, DOI 10.1126/science.1230835
NR 97
TC 182
Z9 196
U1 9
U2 91
PU NATURE PORTFOLIO
PI BERLIN
PA HEIDELBERGER PLATZ 3, BERLIN, 14197, GERMANY
SN 0028-0836
EI 1476-4687
J9 NATURE
JI Nature
PD JUL 23
PY 2020
VL 583
IS 7817
BP 578
EP +
DI 10.1038/s41586-020-2486-3
PG 26
WC Multidisciplinary Sciences
WE Science Citation Index Expanded (SCI-EXPANDED)
SC Science & Technology - Other Topics
GA MP1EM
UT WOS:000551954700010
PM 32699395
OA Green Submitted, hybrid, Green Published
DA 2024-10-21
ER

PT J
AU Cabrera-Martínez, LV
   Herrera, LG
   Cruz-Neto, AP
AF Cabrera-Martinez, Lucia V.
   Herrera M, L. Gerardo
   Cruz-Neto, Ariovaldo P.
TI The energetic cost of mounting an immune response for Pallas's
   long-tongued bat (Glossophaga soricina)
SO PEERJ
LA English
DT Article
DE Immune response; Metabolic rate; Caloric cost;
Phytohaemagglutinin;
   Phyllostomidae; Acute phase response; Lipopolysacharide
ID ACUTE-PHASE RESPONSE; TRADE-OFFS; OXIDATIVE STRESS; FOOD; LIFE;
   IMMUNOLOGY; DEFENSES; BEHAVIOR; SPARROW; ECOLOGY
AB The acute phase response (APR) is the first line of defense of
the vertebrate immune system against pathogens. Mounting an immune
response is believed to be energetically costly but direct measures

of metabolic rate during immune challenges contradict this assumption. The energetic cost of APR for birds is higher than for rodents, suggesting that this response is less expensive for mammals. However, the particularly large increase in metabolic rate after APR activation for a piscivorous bat (Myotis vivesi) suggests that immune response might be unusually costly for bats. Here we quantified the energetic cost and body mass change associated with APR for the nectarivorous Pallas's long-tongued bat (Glossophaga soricina). Activation of the APR resulted in a short-term decrease in body mass and an increase in resting metabolic rate (RMR) with a total energy cost of only 2% of the total energy expenditure estimated for G. soricina. This increase in RMR was far from the large increase measured for piscivorous bats; rather, it was similar to the highest values reported for birds. Overall, our results suggest that the costs of APR for bats may vary interspecifically. Measurement of the energy cost of vertebrate immune response is limited to a few species and further work is warranted to evaluate its significance for an animal's energy budget.
C1 [Cabrera-Martinez, Lucia V.; Cruz-Neto, Ariovaldo P.] Univ Estadual Paulista, Inst Biociencias, Dept Zool, Rio Claro, SP, Brazil.
[Herrera M, L. Gerardo] Univ Nacl Autonoma Mexico, Inst Biol, Estn Biol Chamela, San Patricio, Jalisco, Mexico.
C3 Universidade Estadual Paulista; Universidad Nacional Autonoma de Mexico
RP Herrera, LG (corresponding author), Univ Nacl Autonoma Mexico, Inst Biol, Estn Biol Chamela, San Patricio, Jalisco, Mexico.
EM gherrera@ib.unam.mx
RI cruz-neto, ariovaldo/Y-7225-2019; Cabrera-Martinez, Lucia/JKI-1730-2023;
Cruz-Neto, Ariovaldo/B-2958-2013
OI Cabrera-Martinez, Lucia/0000-0002-5487-9837; Herrera Montalvo, Luis
Gerardo/0000-0001-9348-5040; Cruz-Neto, Ariovaldo/0000-0001-5270-7276
FU CNPq/CONACYT [490586/2013-9]; Conselho Nacional de Pesquisa e
Desenvolvimento Tecnologico [161027/2014-8]; Direccion General de
Asuntos del Personal Academico (DGAPA) [IN202113]; PASPA-DGAPA program
of the Universidad Nacional Autonoma de Mexico [062-2014]; Fundacao de
Amparo a Pesquisa do Estado de Sao Paulo [2008/57687-0]
FX This work was supported by a grant from the bilateral program
CNPq/CONACYT (# 490586/2013-9) to L. Gerardo Herrera M. and Ariovaldo P.
Cruz-Neto. Lucia V. Cabrera-Martinez received a master grant from the
Conselho Nacional de Pesquisa e Desenvolvimento Tecnologico
(#161027/2014-8). L. Gerardo Herrera M. was supported by research grants
from Direccion General de Asuntos del Personal Academico (DGAPA
#IN202113) and by the PASPA-DGAPA program (#062-2014) of the Universidad
Nacional Autonoma de Mexico. Ariovaldo P. Cruz-Neto was funded by

a
grant from Fundacao de Amparo a Pesquisa do Estado de Sao Paulo
(#2008/57687-0). The funders had no role in study design, data
collection and analysis, decision to publish, or preparation of
the
manuscript.
CR Alexander C, 2001, J ENDOTOXIN RES, V7, P167, DOI
10.1177/09680519010070030101
Allen LC, 2009, J COMP PHYSIOL B, V179, P315, DOI 10.1007/
s00360-008-0315-3
[Anonymous], 2008, MEASURING METABOLIC
[Anonymous], ECOLOGICAL BEHAV MET
Ashley N.T., 2012, Ecoimmunology, P45
Aubert A, 1997, BRAIN BEHAV IMMUN, V11, P229, DOI 10.1006/
brbi.1997.0503
Baker ML, 2013, ZOONOSES PUBLIC HLTH, V60, P104, DOI 10.1111/
j.1863-2378.2012.01528.x
Bonneaud C, 2003, AM NAT, V161, P367, DOI 10.1086/346134
Buehler DM, 2009, PHYSIOL BIOCHEM ZOOL, V82, P561, DOI
10.1086/603635
Burness G, 2010, J EXP BIOL, V213, P1386, DOI 10.1242/jeb.027011
Christe P, 2000, ECOL LETT, V3, P207
Cray C, 2009, COMPARATIVE MED, V59, P517
Cruz-Neto AP, 2006, FUNCTIONAL EVOLUTION, P58
Cruz-Neto Ariovaldo P., 1997, Revista Brasileira de Biologia,
V57, P203
Demas GE, 2011, J ANIM ECOL, V80, P710, DOI 10.1111/
j.1365-2656.2011.01813.x
Hasselquist D, 2012, ANIM BEHAV, V83, P1303, DOI 10.1016/
j.anbehav.2012.03.025
Kelm DH, 2007, J COMP PHYSIOL B, V177, P667, DOI 10.1007/
s00360-007-0164-5
King MO, 2013, J EXP BIOL, V216, P2097, DOI 10.1242/jeb.079574
Lee KA, 2006, INTEGR COMP BIOL, V46, P1000, DOI 10.1093/icb/
icl049
Lochmiller RL, 2000, OIKOS, V88, P87, DOI 10.1034/
j.1600-0706.2000.880110.x
MacDonald L, 2012, PHYSIOL BEHAV, V107, P762, DOI 10.1016/
j.physbeh.2012.06.009
Marais M, 2011, J THERM BIOL, V36, P116, DOI 10.1016/
j.jtherbio.2010.12.004
Martin LB, 2017, J EXP BIOL, V220, P2228, DOI 10.1242/jeb.154716
McNab BK, 2003, COMP BIOCHEM PHYS A, V135, P357, DOI 10.1016/
S1095-6433(03)00090-4
Otálora-Ardila A, 2017, MAMM BIOL, V82, P41, DOI 10.1016/
j.mambio.2016.11.002
Otálora-Ardila A, 2016, PLOS ONE, V11, DOI 10.1371/
journal.pone.0164938
Owen-Ashley NT, 2008, BRAIN BEHAV IMMUN, V22, P614, DOI 10.1016/
j.bbi.2007.12.005
Schneeberger K, 2014, NATURWISSENSCHAFTEN, V101, P285, DOI
10.1007/s00114-014-1155-5
Schneeberger K, 2013, J EXP BIOL, V216, P4514, DOI 10.1242/
jeb.090837

Schneeberger K, 2013, PLOS ONE, V8, DOI 10.1371/
journal.pone.0054023
Sheldon BC, 1996, TRENDS ECOL EVOL, V11, P317, DOI
10.1016/0169-5347(96)10039-2
Sikes RS, 2011, J MAMMAL, V92, P235, DOI 10.1644/10-MAMM-F-355.1
Sköld-Chiriac S, 2014, PHYSIOL BIOCHEM ZOOL, V87, P288, DOI
10.1086/674789
Speakman JR, 2010, J ANIM ECOL, V79, P726, DOI 10.1111/
j.1365-2656.2010.01689.x
Stockmaier S, 2015, BIOL LETTERS, V11, DOI 10.1098/rsbl.2015.0576
Strobel S, 2015, MAMM BIOL, V80, P312, DOI 10.1016/
j.mambio.2015.02.005
TAI MM, 1994, DIABETES CARE, V17, P152, DOI 10.2337/
diacare.17.2.152
Tieleman BI, 2005, P ROY SOC B-BIOL SCI, V272, P1715, DOI
10.1098/rspb.2005.3155
Vallès A, 2000, AM J PHYSIOL-REG I, V279, pR1138, DOI 10.1152/
ajpregu.2000.279.3.R1138
Voigt CC, 2006, J COMP PHYSIOL B, V176, P213, DOI 10.1007/
s00360-005-0042-y
Weise P, 2017, PEERJ, V5, DOI 10.7717/peerj.3570
Welch KC, 2008, J EXP BIOL, V211, P310, DOI 10.1242/jeb.012252
NR 42
TC 14
Z9 15
U1 1
U2 15
PU PEERJ INC
PI LONDON
PA 341-345 OLD ST, THIRD FLR, LONDON, EC1V 9LL, ENGLAND
SN 2167-8359
J9 PEERJ
JI PeerJ
PD JUN 5
PY 2018
VL 6
AR e4627
DI 10.7717/peerj.4627
PG 13
WC Multidisciplinary Sciences
WE Science Citation Index Expanded (SCI-EXPANDED)
SC Science & Technology - Other Topics
GA GI2XP
UT WOS:000434235500001
PM 29888121
OA Green Published, gold
DA 2024-10-21
ER

PT J
AU Li, JJ
Zhang, GX
Cheng, DL
Ren, H

Qian, M
Du, B
AF Li, Jinju
Zhang, Guangxu
Cheng, Dalong
Ren, Hua
Qian, Min
Du, Bing
TI Molecular characterization of RIG-I, STAT-1 and IFN-beta in the horseshoe bat
SO GENE
LA English
DT Article
DE Bat; RIG-I; STAT-1; IFN-beta; VSV
ID SARS-LIKE CORONAVIRUSES; BLACK FLYING FOX; PTEROPUS-ALECTO; RESERVOIR;
RECEPTORS; HELICASES; VIRUSES; ROLES
AB Wild Chinese horseshoe bats have been proven to be natural reservoirs of SARS-like coronaviruses. However, the molecular characterization of key proteins in bats still needs to be explored further. In this study, we used cloning and bioinformatics to analyze the sequence of RIG-I, STAT-1 and IFN-beta in the immortalized cell lines from Rhinolophus affinis and Rhinolophus sinicus. Then, we treated different bat cells, mouse embryonic fibroblasts (MEF) and splenocytes with polyinosinic-polycytidylic acid (polyI:C) and vesicular stomatitis virus (VSV) to assess and compare antiviral immune responses between bats and mice. Our results demonstrated that bat RIG-I, STAT-1 and IFN-beta showed close homology with human, mouse, pig and rhesus monkey. RIG-I and STAT-1 were both highly expressed in bat spleen. Furthermore, IFN-beta was induced by polyI:C and VSV in both bat and mouse cells. These findings have provided new insight into the potential characteristics of the bat innate immune system against viral infection. (C) 2015 Elsevier B.V. All rights reserved.
C1 E China Normal Univ, Inst Biomed Sci, Shanghai Key Lab Regulatory Biol, Shanghai 200241, Peoples R China.
E China Normal Univ, Sch Life Sci, Shanghai 200241, Peoples R China.
C3 East China Normal University; East China Normal University
RP Du, B (corresponding author), E China Normal Univ, Inst Biomed Sci, 500 Dongchuan Rd, Shanghai 200241, Peoples R China.
EM bdu@bio.ecnu.edu.cn
OI Du, Bing/0000-0002-5402-6527
FU National Basic Research Program of China [2012CB910400]; National Natural Science Foundation of China [81172816, 81272369]; Science and
Technology Commission of Shanghai Municipality [11DZ2260300]
FX This work was supported by the National Basic Research Program of China
[2012CB910400], the National Natural Science Foundation of China
[81172816 and 81272369], and the Science and Technology Commission of
Shanghai Municipality [11DZ2260300].
CR Biesold SE, 2011, PLOS ONE, V6, DOI 10.1371/journal.pone.0028131

Calisher CH, 2006, CLIN MICROBIOL REV, V19, P531, DOI 10.1128/CMR.00017-06
Chan PKS, 2013, J THORAC DIS, V5, pS118, DOI 10.3978/j.issn.2072-1439.2013.06.19
Chen XM, 1998, CELL, V93, P827, DOI 10.1016/S0092-8674(00)81443-9
Cowled C, 2012, DEV COMP IMMUNOL, V36, P657, DOI 10.1016/j.dci.2011.11.008
Cowled C, 2011, DEV COMP IMMUNOL, V35, P7, DOI 10.1016/j.dci.2010.07.006
Fujii H, 2010, DEV COMP IMMUNOL, V34, P598, DOI 10.1016/j.dci.2010.01.004
Kato H, 2006, NATURE, V441, P101, DOI 10.1038/nature04734
Lau SKP, 2005, P NATL ACAD SCI USA, V102, P14040, DOI 10.1073/pnas.0506735102
Lau SKP, 2010, J VIROL, V84, P2808, DOI 10.1128/JVI.02219-09
Leroy EM, 2005, NATURE, V438, P575, DOI 10.1038/438575a
Li WD, 2005, SCIENCE, V310, P676, DOI 10.1126/science.1118391
Liao JY, 2000, P NATL ACAD SCI USA, V97, P5267, DOI 10.1073/pnas.97.10.5267
Loo YM, 2011, IMMUNITY, V34, P680, DOI 10.1016/j.immuni.2011.05.003
Luis AD, 2013, P ROY SOC B-BIOL SCI, V280, DOI 10.1098/rspb.2012.2753
Omatsu T, 2008, VET IMMUNOL IMMUNOP, V124, P169, DOI 10.1016/j.vetimm.2008.03.004
Pichlmair A, 2006, SCIENCE, V314, P997, DOI 10.1126/science.1132998
Quan PL, 2013, P NATL ACAD SCI USA, V110, P8194, DOI 10.1073/pnas.1303037110
Towner JS, 2009, PLOS PATHOG, V5, DOI 10.1371/journal.ppat.1000536
Virtue ER, 2011, PLOS ONE, V6, DOI 10.1371/journal.pone.0022488
Yuan JF, 2010, J GEN VIROL, V91, P1058, DOI 10.1099/vir.0.016378-0
Zhou P, 2011, J IMMUNOL, V186, P3138, DOI 10.4049/jimmunol.1003115
NR 22
TC 10
Z9 12
U1 1
U2 20
PU ELSEVIER SCIENCE BV
PI AMSTERDAM
PA PO BOX 211, 1000 AE AMSTERDAM, NETHERLANDS
SN 0378-1119
EI 1879-0038
J9 GENE
JI Gene
PD APR 25
PY 2015
VL 561
IS 1
BP 115
EP 123

DI 10.1016/j.gene.2015.02.013
PG 9
WC Genetics & Heredity
WE Science Citation Index Expanded (SCI-EXPANDED)
SC Genetics & Heredity
GA CE6VS
UT WOS:000351976900017
PM 25680291
OA Green Published
DA 2024-10-21
ER

PT J
AU Meza, DK
   Mollentze, N
   Broos, A
   Tello, C
   Valderrama, W
   Recuenco, S
   Carrera, JE
   Shiva, C
   Falcon, N
   Viana, M
   Streicker, DG
AF Meza, Diana K.
   Mollentze, Nardus
   Broos, Alice
   Tello, Carlos
   Valderrama, William
   Recuenco, Sergio
   Carrera, Jorge E.
   Shiva, Carlos
   Falcon, Nestor
   Viana, Mafalda
   Streicker, Daniel G.
TI Ecological determinants of rabies virus dynamics in vampire bats and
   spillover to livestock
SO PROCEEDINGS OF THE ROYAL SOCIETY B-BIOLOGICAL SCIENCES
LA English
DT Article
DE Desmodus rotundus; Chiroptera; metapopulation maintenance; zoonoses;
   eco-immunology; wildlife
ID NIPAH VIRUS; POPULATION; TRANSMISSION; REPRODUCTION; INFECTION;
   IMMUNITY; RISK
AB The pathogen transmission dynamics in bat reservoirs underpin efforts to reduce risks to human health and enhance bat conservation, but are notoriously challenging to resolve. For vampire bat rabies, the geographical scale of enzootic cycles, whether environmental factors modulate baseline risk, and how within-host processes affect population-level dynamics remain unresolved. We studied patterns of rabies exposure using an 11-year, spatially replicated sero-survey of 3709 Peruvian vampire bats and

co-occurring outbreaks in livestock. Seroprevalence was correlated among nearby sites but fluctuated asynchronously at larger distances. A generalized additive mixed model confirmed spatially compartmentalized transmission cycles, but no effects of bat demography or environmental context on seroprevalence. Among 427 recaptured bats, we observed long-term survival following rabies exposure and antibody waning, supporting hypotheses that immunological mechanisms influence viral maintenance. Finally, seroprevalence in bats was only weakly correlated with outbreaks in livestock, reinforcing the challenge of spillover prediction even with extensive data. Together our results suggest that rabies maintenance requires transmission among multiple, nearby bat colonies which may be facilitated by waning of protective immunity. However, the likelihood of incursions and dynamics of transmission within bat colonies appear largely independent of bat ecology. The implications of these results for spillover anticipation and controlling transmission at the source are discussed.
C1 [Meza, Diana K.; Mollentze, Nardus; Broos, Alice; Viana, Mafalda; Streicker, Daniel G.] Univ Glasgow, Sch Biodivers One Hlth & Vet Med, Glasgow, Scotland.
[Meza, Diana K.; Mollentze, Nardus; Broos, Alice; Streicker, Daniel G.] Univ Glasgow, Med Res Council, Ctr Virus Res, Glasgow, Scotland.
[Tello, Carlos; Valderrama, William] Asociac Desarrollo & Conservac Recursos Nat, ILLARIY, Lima, Peru.
[Tello, Carlos] Yunkawasi, Lima, Peru.
[Valderrama, William; Shiva, Carlos; Falcon, Nestor] Univ Peruana Cayetano Heredia, Lima, Peru.
[Recuenco, Sergio] Univ Nacl Mayor de San Marcos, Fac Med San Fernando, Lima, Peru.
[Carrera, Jorge E.] Univ Nacl Mayor de San Marcos, Dept Mastozool, Museo Hist Nat, Lima, Peru.
[Carrera, Jorge E.] Programa Conservac Murcielagos Peru, Lima, Peru.
C3 University of Glasgow; University of Glasgow; UK Research & Innovation
(UKRI); Medical Research Council UK (MRC); Universidad Peruana Cayetano
Heredia; Universidad Nacional Mayor de San Marcos; Universidad Nacional
Mayor de San Marcos
RP Meza, DK (corresponding author), Univ Glasgow, Sch Biodivers One Hlth & Vet Med, Glasgow, Scotland.; Meza, DK (corresponding author), Univ Glasgow, Med Res Council, Ctr Virus Res, Glasgow, Scotland.
EM d.villa-meza.1@research.gla.ac.uk
RI Meza, Diana/GYQ-9537-2022
OI Tello, Carlos/0000-0003-3758-265X; Shiva Ramayoni,
carlos/0000-0002-8473-0128; Broos, Alice/0000-0001-7593-1000; Valderrama
Bazan, William/0000-0003-4626-1444; Carrera Guardia, Jorge
Eduardo/0000-0001-6644-4518; Recuenco-Cabrera,
Sergio/0000-0002-8446-7411; Mollentze, Nardus/
0000-0002-2452-6416; Meza,
Diana K./0000-0001-9796-6706

CR Amman BR, 2014, EMERG INFECT DIS, V20, P1761, DOI 10.3201/eid2010.140696
[Anonymous], 2013, MuMIn: Multi-model inference. R package version 1.10.0. 1
[Anonymous], 2020, R: A language and environment for statistical computing
Bakker KM, 2019, NAT ECOL EVOL, V3, P1697, DOI 10.1038/s41559-019-1032-x
Becker DJ, 2021, TRANSBOUND EMERG DIS, V68, P870, DOI 10.1111/tbed.13754
Becker DJ, 2018, PHILOS T R SOC B, V373, DOI 10.1098/rstb.2017.0089
Benavides JA, 2020, VIRUSES-BASEL, V12, DOI 10.3390/v12091002
Benavides JA, 2017, PLOS NEGLECT TROP D, V11, DOI 10.1371/journal.pntd.0006105
Benavides JA, 2016, P ROY SOC B-BIOL SCI, V283, DOI 10.1098/rspb.2016.0328
Blackwood JC, 2013, P NATL ACAD SCI USA, V110, P20837, DOI 10.1073/pnas.1308817110
Bohmann K, 2018, MOL ECOL RESOUR, V18, P1050, DOI 10.1111/1755-0998.12891
Cárdenas-Canales EM, 2020, TROP MED INFECT DIS, V5, DOI 10.3390/tropicalmed5010034
Davis AD, 2012, J WILDLIFE DIS, V48, P508, DOI 10.7589/0090-3558-48.2.508
Delpietro H.A., 1989, Revue Scientifique et Technique Office International des Epizooties, V8, P177, DOI 10.20506/rst.8.1.401
Delpietro HA, 1996, REV SCI TECH OIE, V15, P971, DOI 10.20506/rst.15.3.964
Donnelly CA, 2003, NATURE, V426, P834, DOI 10.1038/nature02192
Edson D, 2015, PLOS ONE, V10, DOI 10.1371/journal.pone.0125881
Gilbert M, 2018, SCI DATA, V5, DOI 10.1038/sdata.2018.227
Glennon EE, 2019, PHILOS T R SOC B, V374, DOI 10.1098/rstb.2019.0021
Griffiths ME, 2020, NAT COMMUN, V11, DOI 10.1038/s41467-020-19832-4
Griffiths MM, 2022, PLOS BIOL, V20, DOI 10.1371/journal.pbio.3001580
Hijmans RJ., 2020 RASTER GEOGRAPH
Hoyt JR, 2019, SCI REP-UK, V9, DOI 10.1038/s41598-019-45453-z
Jarvis A., 2008, Hole-filled SRTM for the globe Version 4
Lloyd-Smith JO, 2005, TRENDS ECOL EVOL, V20, P511, DOI 10.1016/j.tree.2005.07.004
Lord R.D., 1975, Bulletin of the Pan American Health Organization, V9, P189
LORD RD, 1992, J WILDLIFE DIS, V28, P292, DOI 10.7589/0090-3558-28.2.292
Luby SP, 2009, EMERG INFECT DIS, V15, P1229, DOI 10.3201/eid1508.081237
Meza Diana, 2021, Zenodo, DOI 10.5281/ZENODO.5013655
Meza DK, 2021, TRANSBOUND EMERG DIS, V68, P1564, DOI 10.1111/tbed.13826
Meza DK., 2022, FIGSHARE, DOI [10.6084/m9.figshare.c.6168147, DOI 10.6084/M9.FIGSHARE.C.6168147]

Mollentze N, 2020, P NATL ACAD SCI USA, V117, P9423, DOI 10.1073/pnas.1919176117
Nakagawa S, 2017, J R SOC INTERFACE, V14, DOI 10.1098/rsif.2017.0213
Oksanen J., 2022, Vegan: Community ecology package
Pedersen EJ, 2019, PEERJ, V7, DOI 10.7717/peerj.6876
Plowright RK, 2008, P ROY SOC B-BIOL SCI, V275, P861, DOI 10.1098/rspb.2007.1260
Plowright RK, 2016, PLOS NEGLECT TROP D, V10, DOI 10.1371/journal.pntd.0004796
Plowright RK, 2015, P ROY SOC B-BIOL SCI, V282, DOI 10.1098/rspb.2014.2124
Rocke TE, 2019, SCI REP-UK, V9, DOI 10.1038/s41598-019-43210-w
Sohayati AR, 2011, EPIDEMIOL INFECT, V139, P1570, DOI 10.1017/S0950268811000550
Streicker DG, 2019, P ROY SOC B-BIOL SCI, V286, DOI 10.1098/rspb.2019.1527
Streicker DG, 2016, P NATL ACAD SCI USA, V113, P10926, DOI 10.1073/pnas.1606587113
Streicker DG, 2012, P ROY SOC B-BIOL SCI, V279, P3384, DOI 10.1098/rspb.2012.0538
Trajano E, 1996, BIOTROPICA, V28, P121, DOI 10.2307/2388777
Turmelle AS, 2010, J GEN VIROL, V91, P2360, DOI 10.1099/vir.0.020073-0
Wood S. N., 2017, Generalized Additive Models: An Introduction with R, DOI [10.1201/9781315370279, DOI 10.1201/9781315370279]
NR 46
TC 10
Z9 10
U1 3
U2 14
PU ROYAL SOC
PI LONDON
PA 6-9 CARLTON HOUSE TERRACE, LONDON SW1Y 5AG, ENGLAND
SN 0962-8452
EI 1471-2954
J9 P ROY SOC B-BIOL SCI
JI Proc. R. Soc. B-Biol. Sci.
PD SEP 14
PY 2022
VL 289
IS 1982
AR 20220860
DI 10.1098/rspb.2022.0860
PG 10
WC Biology; Ecology; Evolutionary Biology
WE Science Citation Index Expanded (SCI-EXPANDED)
SC Life Sciences & Biomedicine - Other Topics; Environmental Sciences &
Ecology; Evolutionary Biology
GA 6Q5CB
UT WOS:000891629300001
PM 36069012
OA Green Published, Green Accepted, hybrid

DA 2024-10-21
ER

PT J
AU Berman, TS
   Weinberg, M
   Moreno, KR
   Czirják, GA
   Yovel, Y
AF Berman, Tali S.
   Weinberg, Maya
   Moreno, Kelsey R.
   Czirjak, Gabor A.
   Yovel, Yossi
TI In sickness and in health: the dynamics of the fruit bat gut microbiota
   under a bacterial antigen challenge and its association with the immune
   response
SO FRONTIERS IN IMMUNOLOGY
LA English
DT Article
DE immune response; Chiroptera; ecoimmunology; Weissella; gut microbiota
   resilience; 16s RNA; SILVA database; host-pathogen interactions
ID LIPOPOLYSACCHARIDE; DIVERSITY; IMPACTS; DIET
AB IntroductionInteractions between the gut microbiome (GM) and the immune system influence host health and fitness. However, few studies have investigated this link and GM dynamics during disease in wild species. Bats (Mammalia: Chiroptera) have an exceptional ability to cope with intracellular pathogens and a unique GM adapted to powered flight. Yet, the contribution of the GM to bat health, especially immunity, or how it is affected by disease, remains unknown. MethodsHere, we examined the dynamics of the Egyptian fruit bats' (Rousettus aegyptiacus) GM during health and disease. We provoked an inflammatory response in bats using lipopolysaccharides (LPS), an endotoxin of Gram-negative bacteria. We then measured the inflammatory marker haptoglobin, a major acute phase protein in bats, and analyzed the GM (anal swabs) of control and challenged bats using high-throughput 16S rRNA sequencing, before the challenge, 24h and 48h post challenge. ResultsWe revealed that the antigen challenge causes a shift in the composition of the bat GM (e.g., Weissella, Escherichia, Streptococcus). This shift was significantly correlated with haptoglobin concentration, but more strongly with sampling time. Eleven bacterial sequences were correlated with haptoglobin concentration and nine were found to be potential predictors of the strength of the immune response, and implicit of infection severity, notably Weissella and Escherichia. The bat GM showed high resilience, regaining the colony's group GM composition rapidly, as bats resumed foraging and social activities. ConclusionOur results demonstrate a tight link between bat immune response and changes in their GM, and emphasize the importance of integrating microbial ecology in ecoimmunological studies of wild species. The resilience of the GM may provide this species with an

adaptive advantage to cope with infections and maintain colony health.
C1 [Berman, Tali S.; Weinberg, Maya; Moreno, Kelsey R.; Yovel, Yossi] Tel Aviv Univ, Dept Zool, Tel Aviv, Israel.
[Czirjak, Gabor A.] Leibniz Inst Zoo & Wildlife Res, Dept Wildlife Dis, Berlin, Germany.
[Yovel, Yossi] Tel Aviv Univ, Sagol Sch Neurosci, Tel Aviv, Israel.
C3 Tel Aviv University; Leibniz Institut fur Zoo und Wildtierforschung; Tel
Aviv University
RP Berman, TS; Weinberg, M (corresponding author), Tel Aviv Univ, Dept Zool, Tel Aviv, Israel.
EM talisberman@gmail.com; mayababa@gmail.com
RI Berman, Tali/K-7010-2019; Czirják, Gábor/F-5440-2011
OI Berman, Tali/0000-0003-4006-794X; Czirjak, Gabor
Arpad/0000-0001-9488-0069
FU European Research Council: Behaviour-Island
FX Funding This research was supported by the European Research Council:
Behaviour-Island-1010019933. GC was supported by internal funds from the
Leibniz Institute for Zoo and Wildlife Research, Germany.
CR Anand AAP, 2004, COMP BIOCHEM PHYS A, V139, P65, DOI 10.1016/j.cbpb.2004.07.006
Archie EA, 2019, NAT ECOL EVOL, V3, P18, DOI 10.1038/s41559-018-0749-2
Benskin CMH, 2009, BIOL REV, V84, P349, DOI 10.1111/j.1469-185X.2008.00076.x
Bolnick DI, 2014, MOL ECOL, V23, P4831, DOI 10.1111/mec.12846
Bozzi D, 2021, ANIM MICROBIOME, V3, DOI 10.1186/s42523-021-00096-2
Brennan CA, 2019, NAT REV MICROBIOL, V17, P156, DOI 10.1038/s41579-018-0129-6
Callahan BJ, 2016, NAT METHODS, V13, P581, DOI [10.1038/NMETH.3869, 10.1038/nmeth.3869]
Carthey AJR, 2020, FUNCT ECOL, V34, P764, DOI 10.1111/1365-2435.13504
Costantini D, 2022, CONSERV PHYSIOL, V10, DOI 10.1093/conphys/coac028
Costantini D, 2019, SCI TOTAL ENVIRON, V671, P1077, DOI 10.1016/j.scitotenv.2019.03.398
Davies CS, 2022, MICROBIOME, V10, DOI 10.1186/s40168-022-01233-y
DeGruttola AK, 2016, INFLAMM BOWEL DIS, V22, P1137, DOI 10.1097/MIB.0000000000000750
Dietrich M, 2018, ROY SOC OPEN SCI, V5, DOI 10.1098/rsos.180041
Dietrich M, 2015, ENVIRON MICROBIOL, V17, P4280, DOI 10.1111/1462-2920.12766
Dimkic I, 2021, APPL MICROBIOL BIOT, V105, P1407, DOI 10.1007/s00253-021-11143-y
Dousse F, 2008, J VET DIAGN INVEST, V20, P716, DOI 10.1177/104063870802000602
Drexler JF, 2011, EMERG INFECT DIS, V17, P449, DOI 10.3201/eid1703.100526

Duvallet C, 2017, NAT COMMUN, V8, DOI 10.1038/s41467-017-01973-8
Elkin Lisa A., 2021, UIST '21: The 34th Annual ACM Symposium on User Interface Software and Technology, P754, DOI 10.1145/3472749.3474784
Field KA, 2015, PLOS PATHOG, V11, DOI 10.1371/journal.ppat.1005168
Fleischer R, 2022, MOL ECOL, V31, P3342, DOI 10.1111/mec.16491
Flint HJ, 2015, P NUTR SOC, V74, P13, DOI 10.1017/S0029665114001463
Flint HJ, 2012, NAT REV GASTRO HEPAT, V9, P577, DOI 10.1038/nrgastro.2012.156
Fritze M, 2021, DEV COMP IMMUNOL, V119, DOI 10.1016/j.dci.2021.104017
Fritze M, 2019, BIOL OPEN, V8, DOI 10.1242/bio.046078
Fusco V, 2015, FRONT MICROBIOL, V6, DOI 10.3389/fmicb.2015.00155
Gerardo NM, 2020, PHILOS T R SOC B, V375, DOI 10.1098/rstb.2019.0601
GUTHRIE LA, 1984, J EXP MED, V160, P1656, DOI 10.1084/jem.160.6.1656
Haghshenas B, 2015, INT J FOOD SCI TECH, V50, P1056, DOI 10.1111/ijfs.12718
Haghshenas B, 2015, LWT-FOOD SCI TECHNOL, V60, P690, DOI 10.1016/j.lwt.2014.09.058
Hammer Oyvind, 2001, Palaeontologia Electronica, V4, pUnpaginated
Hoyt JR, 2015, PLOS ONE, V10, DOI 10.1371/journal.pone.0121329
Hu YJ, 2020, BIOINFORMATICS, V36, P4106, DOI 10.1093/bioinformatics/btaa260
Humbert MV, 2020, PATHOGENS, V9, DOI 10.3390/pathogens9010010
Ingala MR, 2019, ECOL EVOL, V9, P6508, DOI 10.1002/ece3.5228
Ingala MR, 2018, MSPHERE, V3, DOI 10.1128/mSphere.00397-18
Jones DN, 2022, TRENDS MICROBIOL, V30, P632, DOI 10.1016/j.tim.2021.12.009
Khan MAW, 2019, PLOS ONE, V14, DOI 10.1371/journal.pone.0215946
Kolodny O, 2019, NAT ECOL EVOL, V3, P116, DOI 10.1038/s41559-018-0731-z
Korine C, 1999, BIOL CONSERV, V88, P301, DOI 10.1016/S0006-3207(98)00126-8
Kreisinger J, 2018, ECOL EVOL, V8, P9793, DOI 10.1002/ece3.4454
Lewin-Epstein O, 2017, NAT COMMUN, V8, DOI 10.1038/ncomms14040
Li AQ, 2022, ENVIRON MICROBIOL, V24, P1484, DOI 10.1111/1462-2920.15754
Li JJ, 2021, FRONT MICROBIOL, V12, DOI 10.3389/fmicb.2021.735122
Lo BC, 2021, INT IMMUNOL, V33, P197, DOI 10.1093/intimm/dxaa079
Luo JH, 2021, ANIM BIOTELEM, V9, DOI 10.1186/s40317-021-00268-6
Lutz HL, 2019, MSYSTEMS, V4, DOI 10.1128/mSystems.00511-19
Madden AA, 2022, J EXP BIOL, V225, DOI 10.1242/jeb.243176
Matheen MIA, 2022, EMU, V122, P255, DOI 10.1080/01584197.2022.2114088
McFall-Ngai M, 2013, P NATL ACAD SCI USA, V110, P3229, DOI 10.1073/pnas.1218525110
McKenzie VJ, 2017, INTEGR COMP BIOL, V57, P690, DOI 10.1093/icb/icx090
Mohr AE, 2022, FEBS LETT, V596, P849, DOI 10.1002/1873-3468.14328
Moreno KR, 2021, ANN NY ACAD SCI, V1505, P178, DOI 10.1111/

nyas.14600
Namasivayam S, 2019, MBIO, V10, DOI 10.1128/mBio.01018-19
Naqib A, 2018, METHODS MOL BIOL, V1783, P149, DOI
10.1007/978-1-4939-7834-2_7
Newman MM, 2018, FRONT MICROBIOL, V9, DOI 10.3389/
fmicb.2018.00914
Ofek T, 2022, ANIM MICROBIOME, V4, DOI 10.1186/s42523-022-00201-z
Pabari K, 2020, PROBIOTICS ANTIMICRO, V12, P1126, DOI 10.1007/
s12602-019-09630-w
Poppleton DI, 2017, FRONT MICROBIOL, V8, DOI 10.3389/
fmicb.2017.01215
Quast Christian, 2013, Nucleic Acids Res, V41, pD590, DOI
10.1093/nar/gks1219
Seltmann A, 2022, SCI REP-UK, V12, DOI 10.1038/s41598-022-18240-6
Sommer F, 2017, NAT REV MICROBIOL, V15, P630, DOI 10.1038/
nrmicro.2017.58
Sun Y, 2019, ANN MICROBIOL, V69, P1407, DOI 10.1007/
s13213-019-01524-0
TEDMAN RA, 1985, AUST J ZOOL, V33, P625, DOI 10.1071/ZO9850625
UTZURRUM RCB, 1991, BIOTROPICA, V23, P311, DOI 10.2307/2388211
Víquez-R L, 2021, MICROBIOL SPECTR, V9, DOI 10.1128/
Spectrum.01525-21
Voigt CC, 2005, J MAMMAL, V86, P745, DOI
10.1644/1545-1542(2005)086[0745:BBABSS]2.0.CO;2
Wallen ZD, 2020, NPJ PARKINSONS DIS, V6, DOI 10.1038/
s41531-020-0112-6
Wasimuddin, 2018, ISME J, V12, P2883, DOI 10.1038/
s41396-018-0239-1
Xiao GH, 2019, FRONT MICROBIOL, V10, DOI 10.3389/fmicb.2019.02247
Yin ZW, 2020, PEERJ, V8, DOI 10.7717/peerj.9003
Zheng DP, 2020, CELL RES, V30, P492, DOI 10.1038/
s41422-020-0332-7
NR 72
TC 3
Z9 3
U1 0
U2 5
PU FRONTIERS MEDIA SA
PI LAUSANNE
PA AVENUE DU TRIBUNAL FEDERAL 34, LAUSANNE, CH-1015, SWITZERLAND
SN 1664-3224
J9 FRONT IMMUNOL
JI Front. Immunol.
PD APR 11
PY 2023
VL 14
AR 1152107
DI 10.3389/fimmu.2023.1152107
PG 11
WC Immunology
WE Science Citation Index Expanded (SCI-EXPANDED)
SC Immunology
GA E2IT1
UT WOS:000973842300001

PM 37114064
OA gold, Green Published
DA 2024-10-21
ER

PT J
AU Rodhain, F
AF Rodhain, Francois
TI Bats and viruses: what relationships ? What consequences
SO BULLETIN DE L ACADEMIE NATIONALE DE MEDECINE
LA French
DT Article
DE CHIROPTERA; VIRUS; IMMUNE TOLERANCE; RESERVOIR; EPIDEMIOLOGY
ID CORONAVIRUS
AB More than 1 240 species of bats are known; many of them are insectivorous, others are frugivorous and some american species are haematophagous. More than 100 different viruses are associated with these mammals, particularly Rhabdoviridae (Lyssavirus like rabies virus), Paramyxoviridae (like Nipah and Hendra viruses), Filoviridae (Ebola and Marburg viruses) and Coronaviridae (viruses causing SARS and MERS). These infections are usually asymptomatic in bats but the mechanism of this tolerance is not yet understood. For those viruses, bats are efficient reservoirs and disseminators. So, they represent a significative risk for human and animal public health, that justifies to set up surveillance of batasso ciated viruses and research programs about the particular immunity mechanisms of bats.
CR Blackwood JC, 2013, P NATL ACAD SCI USA, V110, P20837, DOI 10.1073/pnas.1308817110
Calisher CH, 2006, CLIN MICROBIOL REV, V19, P531, DOI 10.1128/CMR.00017-06
Daszak P, 2013, P NATL ACAD SCI USA, V110, P3681, DOI 10.1073/pnas.1201243109
Drexler JF, 2012, NAT COMMUN, V3, DOI 10.1038/ncomms1796
Hahn MB, 2014, AM J TROP MED HYG, V90, P247, DOI 10.4269/ajtmh.13-0256
Li WD, 2005, SCIENCE, V310, P676, DOI 10.1126/science.1118391
Memish ZA, 2013, EMERG INFECT DIS, V19, P1819, DOI 10.3201/eid1911.131172
MOUTOU F, 2007, VENGEANCE CIVETTE MA
Nadin-Davis SA, 2011, ADV VIRUS RES, V79, P203, DOI 10.1016/B978-0-12-387040-7.00011-1
O'Shea TJ, 2014, EMERG INFECT DIS, V20, P741, DOI 10.3201/eid2005.130539
Picard-Meyer E, 2014, PLOS ONE, V9, DOI 10.1371/journal.pone.0098622
Shankar V, 2005, VECTOR-BORNE ZOONOT, V5, P330, DOI 10.1089/vbz.2005.5.330
Towner JS, 2007, PLOS ONE, V2, DOI 10.1371/journal.pone.0000764
Vabret A, 2013, VIROLOGIE, V17, P211, DOI 10.1684/vir.2013.0514
van der Poel WHM, 2006, VECTOR-BORNE ZOONOT, V6, P315, DOI 10.1089/vbz.2006.6.315
Zhang GJ, 2013, SCIENCE, V339, P456, DOI 10.1126/science.1230835
NR 16
TC 0

Z9 0
U1 0
U2 11
PU ELSEVIER MASSON SAS EDITEUR
PI ISSY LES MOULINEAUX CEDEX
PA 62, RUE CAMILLE DESMOULINS, ISSY LES MOULINEAUX CEDEX, 92442,
FRANCE
SN 0001-4079
J9 B ACAD NAT MED PARIS
JI Bull. Acad. Natl. Med.
PD OCT
PY 2014
VL 198
IS 7
BP 1423
EP 1436
DI 10.1016/S0001-4079(19)31238-5
PG 14
WC Medicine, General & Internal
WE Science Citation Index Expanded (SCI-EXPANDED)
SC General & Internal Medicine
GA V43VJ
UT WOS:000209708600021
PM 27120912
OA Bronze, Green Published
DA 2024-10-21
ER

PT J
AU Luo, DS
   Li, B
   Shen, XR
   Jiang, RD
   Zhu, Y
   Wu, J
   Fan, Y
   Bourhy, H
   Hu, B
   Ge, XY
   Shi, ZL
   Dacheux, L
AF Luo, Dong-Sheng
   Li, Bei
   Shen, Xu-Rui
   Jiang, Ren-Di
   Zhu, Yan
   Wu, Jia
   Fan, Yi
   Bourhy, Herve
   Hu, Ben
   Ge, Xing-Yi
   Shi, Zheng-Li
   Dacheux, Laurent
TI Characterization of Novel Rhabdoviruses in Chinese Bats

SO VIRUSES-BASEL
LA English
DT Article
DE bat; rhabdovirus; lyssavirus; vesiculovirus; ledantevirus; China;
   RT-qPCR; phylogeny; brain; complete genome
ID LYSSAVIRUS INFECTION; VESICULAR STOMATITIS; JAPANESE PIPISTRELLE;
   OROPHARYNGEAL SWABS; HUMAN RABIES; EVOLUTION; CHIROPTERA;
RESERVOIRS;
   EMERGENCE; CORONAVIRUS
AB Bats, the second largest order of mammals worldwide, harbor specific characteristics such as sustaining flight, a special immune system, unique habits, and ecological niches. In addition, they are the natural reservoirs of a variety of emerging or re-emerging zoonotic pathogens. Rhabdoviridae is one of the most diverse families of RNA viruses, which consists of 20 ecologically diverse genera, infecting plants, mammals, birds, reptiles, and fish. To date, three bat-related genera are described, named Lyssavirus, Vesiculovirus, and Ledantevirus. However, the prevalence and the distribution of these bat-related rhabdoviruses remain largely unknown, especially in China. To fill this gap, we performed a large molecular retrospective study based on the real-time reverse transcription polymerase chain reaction (RT-qPCR) detection of lyssavirus in bat samples (1044 brain and 3532 saliva samples, from 63 different bat species) originating from 21 provinces of China during 2006-2018. None of them were positive for lyssavirus, but six bat brains (0.6%) of Rhinolophus bat species, originating from Hubei and Hainan provinces, were positive for vesiculoviruses or ledanteviruses. Based on complete genomes, these viruses were phylogenetically classified into three putative new species, tentatively named Yinshui bat virus (YSBV), Taiyi bat virus (TYBV), and Qiongzhong bat virus (QZBV). These results indicate the novel rhabdoviruses circulated in different Chinese bat populations.
C1 [Luo, Dong-Sheng; Li, Bei; Shen, Xu-Rui; Jiang, Ren-Di; Zhu, Yan; Wu, Jia; Fan, Yi; Hu, Ben; Shi, Zheng-Li] Chinese Acad Sci, Wuhan Inst Virol, CAS Key Lab Special Pathogens & Biosafety, Wuhan 430071, Peoples R China.
   [Luo, Dong-Sheng; Shen, Xu-Rui; Jiang, Ren-Di; Fan, Yi; Shi, Zheng-Li] Univ Chinese Acad Sci, Beijing 100049, Peoples R China.
   [Luo, Dong-Sheng; Bourhy, Herve; Dacheux, Laurent] Inst Pasteur, Lyssavirus Epidemiol & Neuropathol Unit, F-75724 Paris, France.
   [Ge, Xing-Yi] Hunan Univ, Coll Biol, Hunan Prov Key Lab Med Virol, Changsha 410082, Peoples R China.
C3 Chinese Academy of Sciences; Wuhan Institute of Virology, CAS;
Chinese
   Academy of Sciences; University of Chinese Academy of Sciences,
CAS;
   Pasteur Network; Universite Paris Cite; Institut Pasteur Paris;
Hunan
   University
RP Shi, ZL (corresponding author), Chinese Acad Sci, Wuhan Inst Virol, CAS Key Lab Special Pathogens & Biosafety, Wuhan 430071, Peoples R China.; Shi, ZL (corresponding author), Univ Chinese Acad Sci, Beijing 100049, Peoples R China.; Dacheux, L (corresponding author), Inst Pasteur, Lyssavirus Epidemiol & Neuropathol Unit,

F-75724 Paris, France.
EM dongshengluo@outlook.com; libei@wh.iov.cn; pukulovesu@163.com;
drteamwind1004@163.com; zhuyan@wh.iov.cn; wuj@wh.iov.cn;
yifanfs0224@163.com; herve.bourhy@pasteur.fr; huben@wh.iov.cn;
xyge@hnu.edu.cn; zlshi@wh.iov.cn; laurent.dacheux@pasteur.fr
RI Hu, Ben/HCH-4477-2022; Jiang, Rendi/AAH-7358-2021; Fan,
Yi/HNI-9386-2023; Dacheux, Laurent/AAJ-5372-2020; Jiang,
Rendi/KEI-2247-2024; Shi, Zheng-Li/A-1013-2013
OI ge, xing yi/0000-0003-3964-5140; Bourhy, Herve/
0000-0002-2608-5589;
Jiang, Rendi/0000-0003-4235-6964; Dacheux, Laurent/
0000-0002-4401-3706;
Shi, Zheng-Li/0000-0001-8089-163X
FU Campus France; China Scholarship Council through the PHC Cai
Yuanpei
2016 program [36724VF]; National Natural Science Foundation of
China
[81290341]; Scientific and technological basis special project
[2013FY113500]; Institut Pasteur, Paris
FX This work was jointly funded by Campus France and China
Scholarship
Council (D.S.L.) through the PHC Cai Yuanpei 2016 program under
grant
number 36724VF. This study also received funding from the
National
Natural Science Foundation of China (81290341), the Scientific
and
technological basis special project (2013FY113500), and Institut
Pasteur, Paris.
CR Ang BSP, 2018, J CLIN MICROBIOL, V56, DOI 10.1128/JCM.01875-17
Ceballos NA, 2013, EMERG INFECT DIS, V19, P793, DOI 10.3201/
eid1905.121071
Aznar-Lopez C, 2013, J GEN VIROL, V94, P69, DOI 10.1099/
vir.0.046490-0
Badrane H, 2001, J VIROL, V75, P8096, DOI 10.1128/
JVI.75.17.8096-8104.2001
Banyard AC, 2014, VIRUSES-BASEL, V6, P2974, DOI 10.3390/v6082974
Banyard AC, 2011, ADV VIRUS RES, V79, P239, DOI 10.1016/
B978-0-12-387040-7.00012-3
Bennett AJ, 2020, MICROORGANISMS, V8, DOI 10.3390/
microorganisms8050750
Binger T, 2015, J VIROL, V89, P4588, DOI 10.1128/JVI.02932-14
Blasdell KR, 2015, AM J TROP MED HYG, V92, P405, DOI 10.4269/
ajtmh.14-0606
Brook CE, 2015, TRENDS MICROBIOL, V23, P172, DOI 10.1016/
j.tim.2014.12.004
Calisher CH, 2006, CLIN MICROBIOL REV, V19, P531, DOI 10.1128/
CMR.00017-06
CHENIK M, 1995, J VIROL, V69, P707, DOI 10.1128/
JVI.69.2.707-712.1995
Dacheux L, 2019, TRANSBOUND EMERG DIS, V66, P2601, DOI 10.1111/
tbed.13323
Dacheux L, 2016, PLOS NEGLECT TROP D, V10, DOI 10.1371/
journal.pntd.0004812

Dacheux L, 2014, PLOS ONE, V9, DOI 10.1371/journal.pone.0087194
Dacheux Laurent, 2011, Infectious Disorders - Drug Targets, V11, P251
de Thoisy B, 2016, PLOS NEGLECT TROP D, V10, DOI 10.1371/journal.pntd.0004378
Delmas O, 2008, PLOS ONE, V3, DOI 10.1371/journal.pone.0002057
Fischer M, 2014, VIROL J, V11, DOI 10.1186/s12985-014-0196-x
Fort P, 2012, MOL BIOL EVOL, V29, P381, DOI 10.1093/molbev/msr226
Ge XY, 2013, NATURE, V503, P535, DOI 10.1038/nature12711
Goldberg TL, 2017, SCI REP-UK, V7, DOI 10.1038/s41598-017-05236-w
Grard G, 2012, PLOS PATHOG, V8, DOI 10.1371/journal.ppat.1002924
Guillen-Servent A, 2006, ACTA CHIROPTEROL, V8, P39, DOI 10.3161/1733-5329(2006)8[39:ANSOBO]2.0.CO;2
Guindon S, 2010, SYST BIOL, V59, P307, DOI 10.1093/sysbio/syq010
Gunawardena PS, 2016, EMERG INFECT DIS, V22, P1456, DOI 10.3201/eid2208.151986
Guo H, 2020, J VIROL, V94, DOI 10.1128/JVI.00902-20
Hayman DTS, 2016, ANNU REV VIROL, V3, P77, DOI 10.1146/annurev-virology-110615-042203
Hu B, 2017, PLOS PATHOG, V13, DOI 10.1371/journal.ppat.1006698
Hu SC, 2018, EMERG INFECT DIS, V24, P782, DOI 10.3201/eid2404.171696
Iwasaki T, 2004, ARCH VIROL, V149, P1139, DOI 10.1007/s00705-003-0271-x
Jiang Y, 2010, VECTOR-BORNE ZOONOT, V10, P177, DOI 10.1089/vbz.2008.0212
Jiang Zhigang, 2017, Biodiversity Science, V25, P886, DOI 10.17520/biods.2017098
Johnson N, 2010, VET MICROBIOL, V142, P151, DOI 10.1016/j.vetmic.2010.02.001
Jones G, 2006, TRENDS ECOL EVOL, V21, P149, DOI 10.1016/j.tree.2006.01.001
Kessler MK, 2018, ANN NY ACAD SCI, V1429, P78, DOI 10.1111/nyas.13910
Kumar S, 2016, MOL BIOL EVOL, V33, P1870, DOI [10.1093/molbev/msw054, 10.1093/molbev/msv279]
Kumin IV, 2009, INFECT GENET EVOL, V9, P541, DOI 10.1016/j.meegid.2009.02.005
Larkin MA, 2007, BIOINFORMATICS, V23, P2947, DOI 10.1093/bioinformatics/btm404
Lau SKP, 2013, J VIROL, V87, P8638, DOI 10.1128/JVI.01055-13
Lelli D, 2018, VIROL J, V15, DOI 10.1186/s12985-018-0949-z
Leroy EM, 2005, NATURE, V438, P575, DOI 10.1038/438575a
Li C, 2015, ELIFE, V4, DOI [10.7554/eLife.05378, 10.7554/eLife.06587]
Li WD, 2005, SCIENCE, V310, P676, DOI 10.1126/science.1118391
Liu Y, 2013, PLOS NEGLECT TROP D, V7, DOI 10.1371/journal.pntd.0002097
Luis AD, 2013, P ROY SOC B-BIOL SCI, V280, DOI 10.1098/rspb.2012.2753
Mao XG, 2013, PLOS ONE, V8, DOI 10.1371/journal.pone.0056786
Mareuil F., 2017, F1000 Research, V6, DOI [DOI 10.7490/F1000RESEARCH.1114334.1, 10.7490/f1000research.1114334.1]
Mbu'u CM, 2019, VECTOR-BORNE ZOONOT, V19, P455, DOI 10.1089/

vbz.2018.2365
METSELAAR D, 1969, ARCH GES VIRUSFORSCH, V26, P183, DOI 10.1007/BF01241186
Milne I, 2013, BRIEF BIOINFORM, V14, P193, DOI 10.1093/bib/bbs012
Nel LH, 2007, CRIT REV MICROBIOL, V33, P301, DOI 10.1080/10408410701647602
Ng TFF, 2013, EMERG INFECT DIS, V19, P1978, DOI 10.3201/eid1912.121506
Nokireki T, 2018, TRANSBOUND EMERG DIS, V65, P593, DOI 10.1111/tbed.12833
Pallandre L, 2020, VIRUSES-BASEL, V12, DOI 10.3390/v12060649
Rodríguez LL, 2002, VIRUS RES, V85, P211, DOI 10.1016/S0168-1702(02)00026-6
Rupprecht CE, 2011, CURR OPIN VIROL, V1, P662, DOI 10.1016/j.coviro.2011.10.014
Sapkal Gajanan N, 2018, Open Virol J, V12, P44, DOI 10.2174/1874357901812010044
Schatz J, 2014, EPIDEMIOL INFECT, V142, P1155, DOI 10.1017/S0950268813002185
Serra-Cobo J, 2002, EMERG INFECT DIS, V8, P413, DOI 10.3201/eid0804.010263
Shi ZL, 2013, SCI CHINA LIFE SCI, V56, P678, DOI 10.1007/s11427-013-4517-x
Shipley R, 2019, TROP MED INFECT DIS, V4, DOI 10.3390/tropicalmed4010031
Simmons NB, 2005, MAMMAL SPECIES WORLD, V1, P312, DOI [DOI 10.1111/J.1365-2656.2010.01742.X, 10.1093/acprof]
SPIROPOULOU CF, 1993, J VIROL, V67, P3103, DOI 10.1128/JVI.67.6.3103-3110.1993
Teeling EC, 2005, SCIENCE, V307, P580, DOI 10.1126/science.1105113
Towner JS, 2009, PLOS PATHOG, V5, DOI 10.1371/journal.ppat.1000536
Troupin C, 2016, PLOS PATHOG, V12, DOI 10.1371/journal.ppat.1006041
Velasco-Villa A, 2017, ANTIVIR RES, V146, P221, DOI 10.1016/j.antiviral.2017.03.013
Walker PJ, 2018, J GEN VIROL, V99, P447, DOI 10.1099/jgv.0.001020
Walker PJ, 2015, PLOS PATHOG, V11, DOI 10.1371/journal.ppat.1004664
Walker PJ, 2011, VIRUS RES, V162, P110, DOI 10.1016/j.virusres.2011.09.004
Wang LF, 2019, CURR OPIN VIROL, V34, P79, DOI 10.1016/j.coviro.2018.12.007
Wibbelt G, 2010, BIOL LETTERS, V6, P438, DOI 10.1098/rsbl.2010.0267
Xie JZ, 2018, CELL HOST MICROBE, V23, P297, DOI 10.1016/j.chom.2018.01.006
Xu L, 2018, VIRUS RES, V244, P208, DOI 10.1016/j.virusres.2017.11.028
Zhang GJ, 2013, SCIENCE, V339, P456, DOI 10.1126/science.1230835
Zhou P., 2020, NATURE, V579, P270, DOI DOI 10.1038/s41586-020-2012-7
Zhou P, 2016, P NATL ACAD SCI USA, V113, P2696, DOI 10.1073/

pnas.1518240113
NR 78
TC 14
Z9 14
U1 2
U2 13
PU MDPI
PI BASEL
PA ST ALBAN-ANLAGE 66, CH-4052 BASEL, SWITZERLAND
EI 1999-4915
J9 VIRUSES-BASEL
JI Viruses-Basel
PD JAN
PY 2021
VL 13
IS 1
AR 64
DI 10.3390/v13010064
PG 22
WC Virology
WE Science Citation Index Expanded (SCI-EXPANDED)
SC Virology
GA PY2AA
UT WOS:000611850400001
PM 33466539
OA Green Published, gold
DA 2024-10-21
ER

PT J
AU Becker, DJ
Czirják, GA
Rynda-Apple, A
Plowright, RK
AF Becker, Daniel J.
Czirjak, Gabor A.
Rynda-Apple, Agnieszka
Plowright, Raina K.
TI Handling Stress and Sample Storage Are Associated with Weaker
Complement-Mediated Bactericidal Ability in Birds but Not Bats
SO PHYSIOLOGICAL AND BIOCHEMICAL ZOOLOGY
LA English
DT Article
DE acute stress; bacterial killing assay; bacterial killing ability;
ecoimmunology; Escherichia coli ATCC 8739; innate immunity;
wildlife
ID IMMUNE-RESPONSES; RESERVOIR HOSTS; VIRUS-INFECTION; PTEROPID
BATS;
LIFE-HISTORY; DISEASE; PLASMA; METAANALYSIS; EVOLUTION; FLIGHT
AB Variation in immune defense influences infectious disease
dynamics within and among species. Understanding how variation in
immunity drives pathogen transmission among species is especially
important for animals that are reservoir hosts for zoonotic
pathogens. Bats, in particular, have a propensity to host serious

viral zoonoses without developing clinical disease themselves. The immunological adaptations that allow bats to host viruses without disease may be related to their adaptations for flight (e.g., in metabolism and mediation of oxidative stress). A number of analyses report greater richness of zoonotic pathogens in bats than in other taxa, such as birds (i.e., mostly volant vertebrates) and rodents (i.e., nonvolant small mammals), but immunological comparisons between bats and these other taxa are rare. To examine interspecific differences in bacterial killing ability (BKA), a functional measure of overall constitutive innate immunity, we use a phylogenetic meta-analysis to compare how BKA responds to the acute stress of capture and to storage time of frozen samples across the orders Aves and Chiroptera. After adjusting for host phylogeny, sample size, and total microbe colony-forming units, we find preliminary evidence that the constitutive innate immune defense of bats may be more resilient to handling stress and storage time than that of birds. This pattern was also similar when we analyzed the proportion of nonnegative and positive effect sizes per species, using phylogenetic comparative methods. We discuss potential physiological and evolutionary mechanisms by which complement proteins may differ between species orders and suggest future avenues for comparative field studies of immunity between sympatric bats, birds, and rodents in particular.
C1 [Becker, Daniel J.; Rynda-Apple, Agnieszka; Plowright, Raina K.] Montana State Univ, Dept Microbiol & Immunol, Bozeman, MT 59717 USA.
[Becker, Daniel J.] Univ Georgia, Ctr Ecol Infect Dis, Athens, GA 30602 USA.
[Czirjak, Gabor A.] Leibniz Inst Zoo & Wildlife Res, Dept Wildlife Dis, Berlin, Germany.
C3 Montana State University System; Montana State University Bozeman;
University System of Georgia; University of Georgia; Leibniz Institut
fur Zoo und Wildtierforschung
RP Becker, DJ (corresponding author), Montana State Univ, Dept Microbiol & Immunol, Bozeman, MT 59717 USA.; Becker, DJ (corresponding author), Univ Georgia, Ctr Ecol Infect Dis, Athens, GA 30602 USA.
EM daniel.becker3@montana.edu
RI Czirják, Gábor/F-5440-2011; Becker, Daniel/A-6466-2014
OI Czirjak, Gabor Arpad/0000-0001-9488-0069; Becker,
Daniel/0000-0003-4315-8628
FU Defense Advanced Research Projects Agency Young Faculty Award
[D16AP00113]; National Science Foundation [DEB-1716698]; National
Institutes of Health (NIH) Institutional Development Award (IDeA)
Program [P20GM103474, P30GM110732]; Strategic Environmental Research and
Development Program [RC-2633]; Leibniz Institute for Zoo and Wildlife
Research; NIH IDeA Program [P30GM110732, P20GM104417]; NIH/ National
Institute of Allergy and Infectious Diseases award [R01AI04905]; Montana
State University Agricultural Experiment Station

FX D.J.B. and R.K.P. were supported by a Defense Advanced Research Projects
Agency Young Faculty Award (D16AP00113) and National Science Foundation
award DEB-1716698; R.K.P. was also funded by the National Institutes of
Health (NIH) Institutional Development Award (IDeA) Program (P20GM103474
and P30GM110732) and the Strategic Environmental Research and
Development Program(RC-2633). G.A.C. was supported by funds from the
Leibniz Institute for Zoo and Wildlife Research. A.R.-A. was supported
by the NIH IDeA Program (P20GM104417, P30GM110732), NIH/National
Institute of Allergy and Infectious Diseases award R01AI04905, and the
Montana State University Agricultural Experiment Station. We thank
Ulalume Hernandez-Arciga for providing assay clarification and three
anonymous reviewers for helpful comments on a previous version of the
manuscript. The authors declare no conflicts of interest.
CR Allen LC, 2009, J COMP PHYSIOL B, V179, P315, DOI 10.1007/s00360-008-0315-3
Amman BR, 2015, J WILDLIFE DIS, V51, P113, DOI 10.7589/2014-08-198
[Anonymous], PHYSL BIOCH ZOOL DRY
[Anonymous], 2014, Modern Phylogenetic Comparative Methods and Their Application in Evolutionary Biology: Concepts and Practice
López-López JA, 2014, BRIT J MATH STAT PSY, V67, P30, DOI 10.1111/bmsp.12002
Arnold TW, 2010, J WILDLIFE MANAGE, V74, P1175, DOI 10.2193/2009-367
Baker ML, 2013, ZOONOSES PUBLIC HLTH, V60, P104, DOI 10.1111/j.1863-2378.2012.01528.x
Bean AGD, 2013, NAT REV IMMUNOL, V13, P851, DOI 10.1038/nri3551
Beck ML, 2017, J EXP ZOOL PART A, V327, P293, DOI 10.1002/jez.2089
Becker DJ, 2018, PLOS NEGLECT TROP D, V12, DOI 10.1371/journal.pntd.0006786
Becker DJ, 2018, PHILOS T R SOC B, V373, DOI 10.1098/rstb.2017.0089
Becker DJ, 2017, ROY SOC OPEN SCI, V4, DOI 10.1098/rsos.170073
Bentz AB, 2016, ROY SOC OPEN SCI, V3, DOI 10.1098/rsos.160499
Borenstein M., 2009, HDB RES SYNTHESIS ME, P221, DOI DOI 10.7758/9781610441384
Brook CE, 2015, TRENDS MICROBIOL, V23, P172, DOI 10.1016/j.tim.2014.12.004
Bundle MW, 2007, J EXP BIOL, V210, P1075, DOI 10.1242/jeb.02727
Burnham K.P., 1998, Model Selection and Multimodel Inference: A Practical Information-theoretic Approach
Cabrera-Martínez LV, 2018, PEERJ, V6, DOI 10.7717/peerj.4627
Cohn LD, 2003, PSYCHOL METHODS, V8, P243, DOI

10.1037/1082-989X.8.3.243
Delvaeye M, 2009, BLOOD, V114, P2367, DOI 10.1182/blood-2009-05-199208
Demas GE, 2011, J ANIM ECOL, V80, P710, DOI 10.1111/j.1365-2656.2011.01813.x
Dodds AW, 2007, IMMUNOBIOLOGY, V212, P233, DOI 10.1016/j.imbio.2006.11.009
Duval S, 2000, BIOMETRICS, V56, P455, DOI 10.1111/j.0006-341X.2000.00455.x
Ecker EE, 1938, J BIOL CHEM, V123, P351
Egger M, 1997, BMJ-BRIT MED J, V315, P629, DOI 10.1136/bmj.315.7109.629
Fassbinder-Orth CA, 2014, INTEGR COMP BIOL, V54, P396, DOI 10.1093/icb/icu023
Flies AS, 2016, FUNCT ECOL, V30, P1549, DOI 10.1111/1365-2435.12638
Foley NM, 2018, SCI ADV, V4, DOI 10.1126/sciadv.aao0926
French SS, 2012, BIOL OPEN, V1, P482, DOI 10.1242/bio.2012919
GRAFEN A, 1989, PHILOS T R SOC B, V326, P119, DOI 10.1098/rstb.1989.0106
Guerrero-Chacón AL, 2018, MAMMAL RES, V63, P397, DOI 10.1007/s13364-018-0375-z
Halpin K, 2011, AM J TROP MED HYG, V85, P946, DOI 10.4269/ajtmh.2011.10-0567
Han BA, 2016, TRENDS PARASITOL, V32, P565, DOI 10.1016/j.pt.2016.04.007
HATTEN BA, 1973, J EXP ZOOL, V186, P193, DOI 10.1002/jez.1401860210
Hawley DM, 2011, FUNCT ECOL, V25, P48, DOI 10.1111/j.1365-2435.2010.01753.x
Heinrich SK, 2016, BIOL OPEN, V5, P550, DOI 10.1242/bio.014902
Hinchliff CE, 2015, P NATL ACAD SCI USA, V112, P12764, DOI 10.1073/pnas.1423041112
Huber-Lang M, 2006, NAT MED, V12, P682, DOI 10.1038/nm1419
Jacobs AC, 2016, COMP BIOCHEM PHYS A, V191, P115, DOI 10.1016/j.cbpa.2015.10.004
Jenkins Rosalind E., 2008, V425, P15, DOI 10.1007/978-1-60327-210-0_2
Johnson JB, 2015, J VIROL, V89, P989, DOI 10.1128/JVI.02427-14
Johnson PTJ, 2012, ECOL LETT, V15, P235, DOI 10.1111/j.1461-0248.2011.01730.x
Jolles AE, 2015, PARASITE IMMUNOL, V37, P255, DOI 10.1111/pim.12153
Kacprzyk J, 2017, ACTA CHIROPTEROL, V19, P219, DOI [10.3161/15081109ACC2017.19.2.001, 10.3161/15081109acc2017.19.2.001]
Konstantopoulos S, 2011, RES SYNTH METHODS, V2, P61, DOI 10.1002/jrsm.35
Korte SM, 2005, NEUROSCI BIOBEHAV R, V29, P3, DOI 10.1016/j.neubiorev.2004.08.009
Ksiazek A, 2012, PHYSIOL BIOCHEM ZOOL, V85, P51, DOI 10.1086/663696
Lee KA, 2006, INTEGR COMP BIOL, V46, P1000, DOI 10.1093/icb/icl049
Li WD, 2005, SCIENCE, V310, P676, DOI 10.1126/science.1118391

Liebl AL, 2009, FUNCT ECOL, V23, P1091, DOI 10.1111/j.1365-2435.2009.01592.x
Luis AD, 2013, P ROY SOC B-BIOL SCI, V280, DOI 10.1098/rspb.2012.2753
Lynch NJ, 2005, J IMMUNOL, V174, P4998, DOI 10.4049/jimmunol.174.8.4998
Martin LB, 2007, ECOLOGY, V88, P2516, DOI 10.1890/07-0060.1
Matson KD, 2006, PHYSIOL BIOCHEM ZOOL, V79, P556, DOI 10.1086/501057
MCNAB BK, 1994, AM NAT, V144, P628, DOI 10.1086/285697
Michonneau F, 2016, METHODS ECOL EVOL, V7, P1476, DOI 10.1111/2041-210X.12593
Middleton DJ, 2007, J COMP PATHOL, V136, P266, DOI 10.1016/j.jcpa.2007.03.002
Millet S, 2007, DEV COMP IMMUNOL, V31, P188, DOI 10.1016/j.dci.2006.05.013
Moher D, 2015, SYST REV-LONDON, V4, DOI [10.1186/2046-4053-4-1, 10.1371/journal.pmed.1000097, 10.1136/bmj.b2700, 10.1136/bmj.b2535, 10.1016/j.ijsu.2010.07.299, 10.1016/j.ijsu.2010.02.007, 10.1136/bmj.i4086]
Moore MS, 2011, PLOS ONE, V6, DOI 10.1371/journal.pone.0027430
Mühldorfer K, 2013, ZOONOSES PUBLIC HLTH, V60, P93, DOI 10.1111/j.1863-2378.2012.01536.x
Munshi-South J, 2010, AGEING RES REV, V9, P12, DOI 10.1016/j.arr.2009.07.006
Nakao M, 2016, EVOLUTION OF THE IMMUNE SYSTEM: CONSERVATION AND DIVERSIFICATION, P151, DOI 10.1016/B978-0-12-801975-7.00006-2
Noris M, 2013, SEMIN NEPHROL, V33, P479, DOI 10.1016/j.semnephrol.2013.08.001
O'Shea TJ, 2014, EMERG INFECT DIS, V20, P741, DOI 10.3201/eid2005.130539
Olival KJ, 2017, NATURE, V546, P646, DOI 10.1038/nature22975
Padgett DA, 2003, TRENDS IMMUNOL, V24, P444, DOI 10.1016/S1471-4906(03)00173-X
Pagel M, 1999, NATURE, V401, P877, DOI 10.1038/44766
2004, BIOINFORMATICS, V20, P289, DOI [DOI 10.1093/bioinformatics/btg412, DOI 10.1093/BIOINFORMATICS/BTG412]
Paull SH, 2012, FRONT ECOL ENVIRON, V10, P75, DOI 10.1890/110111
PILLEMER L, 1953, J EXP MED, V97, P573, DOI 10.1084/jem.97.4.573
Pinheiro Jos., 2010, Mixed-Effects Models in S and SPLUS
Plowright RK, 2016, PLOS NEGLECT TROP D, V10, DOI 10.1371/journal.pntd.0004796
Plowright RK, 2015, P ROY SOC B-BIOL SCI, V282, DOI 10.1098/rspb.2014.2124
Previtali MA, 2012, OIKOS, V121, P1483, DOI 10.1111/j.1600-0706.2012.020215.x
R Development Core Team, 2019, R: A Language and Environment for Statistical Computing, V1, P409, DOI [10.1007/978-3-540-74686-7, DOI 10.1007/978-3-540-74686-7]
Reed Kurt D, 2003, Clin Med Res, V1, P5
Reeder DM, 2006, J COMP PHYSIOL B, V176, P513, DOI 10.1007/s00360-006-0073-z
Reeder DM, 2004, GEN COMP ENDOCR, V136, P260, DOI 10.1016/j.ygcen.2003.12.020

ROSENTHAL R, 1979, PSYCHOL BULL, V86, P638, DOI 10.1037/0033-2909.86.3.638
Rosenthal R, 2001, ANNU REV PSYCHOL, V52, P59, DOI 10.1146/annurev.psych.52.1.59
Schneeberger K, 2013, J EXP BIOL, V216, P4514, DOI 10.1242/jeb.090837
Schneeberger K, 2013, PLOS ONE, V8, DOI 10.1371/journal.pone.0054023
Schountz T, 2017, FRONT IMMUNOL, V8, DOI 10.3389/fimmu.2017.01098
Selvaraju S, 2012, ELECTROPHORESIS, V33, P74, DOI 10.1002/elps.201100431
Shaw AE, 2017, PLOS BIOL, V15, DOI 10.1371/journal.pbio.2004086
Speakman JR, 2003, BAT ECOLOGY, P430
Stephens PR, 2016, ECOL LETT, V19, P1159, DOI 10.1111/ele.12644
Sterne JAC, 2005, PUBLICATION BIAS IN META-ANALYSIS: PREVENTION, ASSESSMENT AND ADJUSTMENTS, P99
Stockmaier S, 2015, BIOL LETTERS, V11, DOI 10.1098/rsbl.2015.0576
Strobel S, 2015, MAMM BIOL, V80, P312, DOI 10.1016/j.mambio.2015.02.005
Tian JD, 2015, FUNCT ECOL, V29, P823, DOI 10.1111/1365-2435.12394
Tieleman BI, 2005, P ROY SOC B-BIOL SCI, V272, P1715, DOI 10.1098/rspb.2005.3155
Uzun L, 2013, COLLOID SURFACE B, V112, P1, DOI 10.1016/j.colsurfb.2013.07.010
VanderWaal KL, 2016, FUNCT ECOL, V30, P1606, DOI 10.1111/1365-2435.12645
Veikkolainen V, 2014, EMERG INFECT DIS, V20, P960, DOI 10.3201/eid2006.130956
Viechtbauer W, 2010, J STAT SOFTW, V36, P1, DOI 10.18637/jss.v036.i03
Wang LF, 2011, CURR OPIN VIROL, V1, P649, DOI 10.1016/j.coviro.2011.10.013
Weise P, 2017, PEERJ, V5, DOI 10.7717/peerj.3570
Williamson MM, 2000, J COMP PATHOL, V122, P201, DOI 10.1053/jcpa.1999.0364
Wilman H., 2014, Ecology, V95, P2027, DOI [DOI 10.1890/13-1917.1, 10.1890/13-1917.1]
Wolf F. M., 1986, METAANALYSIS QUANTIT
Wright NA, 2016, P NATL ACAD SCI USA, V113, P4765, DOI 10.1073/pnas.1522931113
Xie JZ, 2018, CELL HOST MICROBE, V23, P297, DOI 10.1016/j.chom.2018.01.006
Zhang GJ, 2013, SCIENCE, V339, P456, DOI 10.1126/science.1230835
Zhou P, 2016, P NATL ACAD SCI USA, V113, P2696, DOI 10.1073/pnas.1518240113
Zylberberg M, 2015, J EXP BIOL, V218, P757, DOI 10.1242/jeb.111716
NR 107
TC 15
Z9 17
U1 0
U2 31
PU UNIV CHICAGO PRESS
PI CHICAGO

PA 1427 E 60TH ST, CHICAGO, IL 60637-2954 USA
SN 1522-2152
EI 1537-5293
J9 PHYSIOL BIOCHEM ZOOL
JI Physiol. Biochem. Zool.
PD JAN-FEB
PY 2019
VL 92
IS 1
BP 37
EP 48
DI 10.1086/701069
PG 12
WC Physiology; Zoology
WE Science Citation Index Expanded (SCI-EXPANDED); Social Science Citation Index (SSCI)
SC Physiology; Zoology
GA HB8EP
UT WOS:000451319100001
PM 30481115
OA Bronze, Green Accepted
DA 2024-10-21
ER

PT J
AU Tarigan, R
   Shimoda, H
   Doysabas, KCC
   Ken, M
   Iida, A
   Hondo, E
AF Tarigan, Ronald
   Shimoda, Hiroshi
   Doysabas, Karla Cristine C.
   Ken, Maeda
   Iida, Atsuo
   Hondo, Eiichi
TI Role of pattern recognition receptors and interferon-beta in protecting
   bat cell lines from encephalomyocarditis virus and Japanese encephalitis
   virus infection
SO BIOCHEMICAL AND BIOPHYSICAL RESEARCH COMMUNICATIONS
LA English
DT Article
DE Bat; EMCV; JEV; PRRs; IFN-beta
ID TOLL-LIKE RECEPTORS; BLACK FLYING FOX; MOLECULAR CHARACTERIZATION;
   RIG-I; RESPONSES; HELICASES; ACID
AB Bats are potential natural hosts of Encephalomyocarditis virus (EMCV) and Japanese encephalitis virus (JEV). Bats appear to have some unique features in their innate immune system that inhibit viral replication causing limited clinical symptoms, and thus, contributing to the virus spill over to humans. Here, kidney

epithelial cell lines derived from four bat species (Pteropus dasymallus, Rousettus leschenaultii, Rhinolophus ferrumequinum, and Miniopterus fuliginosus) and two non-bat species (Homo sapiens and Mesocricetus auratus) were infected with EMCV and JEV. The replication of EMCV and JEV was lower in the bat cell lines derived from R. leschenaultii, R. ferrumequinum, and M. fuliginosus with a higher expression level of pattern recognition receptors (PRRs) (TLR3, RIG-I, and MDA5) and interferon-beta (IFN-beta) than that in the non-bat cell lines and a bat cell line derived from P. dasymallus. The knockdown of TLR3, RIG-I, and MDA5 in Rhinolophus bat cell line using antisense RNA oligonucleotide led to decrease IFN-b expression and increased viral replication. These results suggest that TLR3, RIG-I, and MDA5 are important for antiviral response against EMCV and JEV in Rhinolophus bats. (c) 2020 Elsevier Inc. All rights reserved.
C1 [Tarigan, Ronald; Doysabas, Karla Cristine C.; Iida, Atsuo; Hondo, Eiichi] Nagoya Univ, Grad Sch Bioagr Sci, Lab Anim Morphol, Nagoya, Aichi, Japan.
[Shimoda, Hiroshi] Yamaguchi Univ, Joint Fac Vet Med, Lab Vet Microbiol, Yamaguchi, Japan.
[Ken, Maeda] Natl Inst Infect Dis, Div Vet Sci, Tokyo, Japan.
C3 Nagoya University; Yamaguchi University; National Institute of Infectious Diseases (NIID)
RP Hondo, E (corresponding author), Nagoya Univ, Grad Sch Bioagr Sci, Lab Anim Morphol, Nagoya, Aichi, Japan.
EM ehondo@agr.nagoya-u.ac.jp
RI Tarigan, Ronald/GPW-7141-2022
OI IIDA, Atsuo/0000-0002-0138-5366; Tarigan, Ronald/0000-0002-6058-0591
CR Ahn M, 2019, NAT MICROBIOL, V4, P789, DOI 10.1038/s41564-019-0371-3
Baker ML, 2013, ZOONOSES PUBLIC HLTH, V60, P104, DOI 10.1111/j.1863-2378.2012.01528.x
Banerjee A, 2019, VIRUSES-BASEL, V11, DOI 10.3390/v11020152
Banerjee A, 2017, SCI REP-UK, V7, DOI 10.1038/s41598-017-01513-w
Basu Anirban, 2017, F1000Res, V6, P259, DOI 10.12688/f1000research.9561.1
Bean AGD, 2013, NAT REV IMMUNOL, V13, P851, DOI 10.1038/nri3551
Carocci M, 2012, VIRULENCE, V3, P351, DOI 10.4161/viru.20573
Cowled C, 2012, DEV COMP IMMUNOL, V36, P657, DOI 10.1016/j.dci.2011.11.008
Cowled C, 2011, DEV COMP IMMUNOL, V35, P7, DOI 10.1016/j.dci.2010.07.006
Doysabas KCC, 2019, VIRUS RES, V259, P62, DOI 10.1016/j.virusres.2018.10.020
Fagre AC, 2019, VIRUSES-BASEL, V11, DOI 10.3390/v11030215
Gitlin L, 2006, P NATL ACAD SCI USA, V103, P8459, DOI 10.1073/pnas.0603082103
Hardarson HS, 2007, AM J PHYSIOL-HEART C, V292, pH251, DOI 10.1152/ajpheart.00398.2006
Höbel S, 2010, METHODS MOL BIOL, V623, P283, DOI 10.1007/978-1-60761-588-0_18
Iha K, 2010, J VET MED SCI, V72, P217, DOI 10.1292/jvms.09-0050
Jiang R, 2014, J IMMUNOL RES, V2014, DOI 10.1155/2014/787023

Li JJ, 2015, GENE, V561, P115, DOI 10.1016/j.gene.2015.02.013
Liu WJ, 2006, J VIROL, V80, P2396, DOI 10.1128/JVI.80.5.2396-2404.2006
Maeda K, 2008, EMERG INFECT DIS, V14, P347, DOI 10.3201/eid1402.070932
Maruyama J, 2014, J VIROL, V88, P99, DOI 10.1128/JVI.02265-13
Meylan E, 2006, MOL CELL, V22, P561, DOI 10.1016/j.molcel.2006.05.012
Nazmi A, 2011, PLOS ONE, V6, DOI 10.1371/journal.pone.0021761
Nerome R, 2007, J GEN VIROL, V88, P2762, DOI 10.1099/vir.0.82941-0
Omatsu T, 2007, COMP IMMUNOL MICROB, V30, P357, DOI 10.1016/j.cimid.2007.05.006
Papon L, 2009, VIROLOGY, V393, P311, DOI 10.1016/j.virol.2009.08.009
Sandekian V, 2013, VIRUS RES, V173, P327, DOI 10.1016/j.virusres.2013.01.010
Sarkis S, 2018, DEV COMP IMMUNOL, V81, P1, DOI 10.1016/j.dci.2017.10.023
Schountz T, 2017, FRONT IMMUNOL, V8, DOI 10.3389/fimmu.2017.01098
Teeling EC, 2005, SCIENCE, V307, P580, DOI 10.1126/science.1105113
van den Hurk AF, 2009, AM J TROP MED HYG, V81, P457, DOI 10.4269/ajtmh.2009.81.457
Zhang HL, 2017, J VIROL, V91, DOI [10.1128/JVI.02396-16, 10.1128/jvi.02396-16]
Zhou DY, 2018, VIROL SIN, V33, P515, DOI 10.1007/s12250-018-0067-5
NR 32
TC 10
Z9 11
U1 4
U2 24
PU ACADEMIC PRESS INC ELSEVIER SCIENCE
PI SAN DIEGO
PA 525 B ST, STE 1900, SAN DIEGO, CA 92101-4495 USA
SN 0006-291X
EI 1090-2104
J9 BIOCHEM BIOPH RES CO
JI Biochem. Biophys. Res. Commun.
PD JUN 18
PY 2020
VL 527
IS 1
BP 1
EP 7
DI 10.1016/j.bbrc.2020.04.060
PG 7
WC Biochemistry & Molecular Biology; Biophysics
WE Science Citation Index Expanded (SCI-EXPANDED)
SC Biochemistry & Molecular Biology; Biophysics
GA LR8RP
UT WOS:000535964300001
PM 32446351

OA Green Published
DA 2024-10-21
ER

PT J
AU Bondet, V
Le Baut, M
Le Poder, S
Lécu, A
Petit, T
Wedlarski, R
Duffy, D
Le Roux, D
AF Bondet, Vincent
Le Baut, Maxime
Le Poder, Sophie
Lecu, Alexis
Petit, Thierry
Wedlarski, Rudy
Duffy, Darragh
Le Roux, Delphine
TI Constitutive IFNα Protein Production in Bats
SO FRONTIERS IN IMMUNOLOGY
LA English
DT Article
DE Chiroptera; bats; type I IFN; Simoa digital ELISA; protein levels;
antiviral immunity
ID FRUIT BATS; INTERFERON-ALPHA; RESERVOIR HOSTS; VIRUS-INFECTION; PTEROPID
BATS; HENDRA VIRUS
AB Bats are the only mammals with self-powered flight and account for 20% of all extant mammalian diversity. In addition, they harbor many emerging and reemerging viruses, including multiple coronaviruses, several of which are highly pathogenic in other mammals, but cause no disease in bats. How this symbiotic relationship between bats and viruses exists is not yet fully understood. Existing evidence supports a specific role for the innate immune system, in particular type I interferon (IFN) responses, a major component of antiviral immunity. Previous studies in bats have shown that components of the IFN pathway are constitutively activated at the transcriptional level. In this study, we tested the hypothesis that the type I IFN response in bats is also constitutively activated at the protein level. For this, we utilized highly sensitive Single Molecule (Simoa) digital ELISA assays, previously developed for humans that we adapted to bat samples. We prospectively sampled four non-native chiroptera species from French zoos. We identified a constitutive expression of IFN alpha protein in the circulation of healthy bats, and concentrations that are physiologically active in humans. Expression levels differed according to the species examined, but were not associated with age, sex, or health status suggesting constitutive IFN alpha protein expression independent of disease. These results confirm a unique IFN response in bat species that may explain their ability to

coexist with multiple viruses in the absence of pathology. These results may help to manage potential zoonotic viral reservoirs and potentially identify new anti-viral strategies.
C1 [Bondet, Vincent; Duffy, Darragh] Univ Paris, Inst Pasteur, Translat Immunol Lab, Paris, France.
[Le Baut, Maxime; Le Poder, Sophie; Le Roux, Delphine] Ecole Natl Vet Alfort, BioPole Alfort, Maisons Alfort, France.
[Le Poder, Sophie] Anses, UMR Virol, Lab Sante Anim, Ecole Natl Vet Alfort,INRAE, Maisons Alfort, France.
[Lecu, Alexis] Museum Natl Hist Nat, Parc Zool Paris, Paris, France.
[Petit, Thierry] Parc Zool La Palmyre, Les Mathes, France.
[Wedlarski, Rudy] Bioparc Zoo Doue La Fontaine, Doue La Fontaine, France.
[Le Roux, Delphine] Ansas, UMR BIPAR, Ecole Natl Vet Alfort, INRAE,Lab Sante Anim, Maisons Alfort, France.
C3 Pasteur Network; Universite Paris Cite; Institut Pasteur Paris; Ecole Nationale Veterinaire d'Alfort (ENVA); Agence Nationale de Securite Sanitaire de l'Alimentation, de l'Environnement du Travail (ANSES); Laboratoire de Sante Animale de Maisons-Alfort; Ecole Nationale Veterinaire d'Alfort (ENVA); INRAE; Museum National d'Histoire Naturelle (MNHN); Ecole Nationale Veterinaire d'Alfort (ENVA); INRAE; Agence Nationale de Securite Sanitaire de l'Alimentation, de l'Environnement du Travail (ANSES); Laboratoire de Sante Animale de Maisons-Alfort
RP Duffy, D (corresponding author), Univ Paris, Inst Pasteur, Translat Immunol Lab, Paris, France.; Le Roux, D (corresponding author), Ecole Natl Vet Alfort, BioPole Alfort, Maisons Alfort, France.; Le Roux, D (corresponding author), Ansas, UMR BIPAR, Ecole Natl Vet Alfort, INRAE,Lab Sante Anim, Maisons Alfort, France.
EM darragh.duffy@pasteur.fr; delphine.le-roux@vet-alfort.fr
OI le roux, delphine/0000-0003-3252-9155; Duffy, Darragh/0000-0002-8875-2308
FU Agence Nationale pour la Recherche (ANR) [CE17001002]
FX Funding This work was funded by the Agence Nationale pour la Recherche (ANR), grant number CE17001002.
CR Amador LI, 2018, J MAMM EVOL, V25, P37, DOI 10.1007/s10914-016-9363-8
Badrane H, 2001, J VIROL, V75, P8096, DOI 10.1128/JVI.75.17.8096-8104.2001
Baker KS, 2013, J VIROL, V87, P1348, DOI 10.1128/JVI.01202-12
Baker ML, 2013, ZOONOSES PUBLIC HLTH, V60, P104, DOI 10.1111/j.1863-2378.2012.01528.x
Bastard P, 2020, SCIENCE, V370, P423, DOI 10.1126/science.abd4585
Bondet V, 2021, CYTOKINE, V144, DOI 10.1016/j.cyto.2021.155533
Brook CE, 2015, TRENDS MICROBIOL, V23, P172, DOI 10.1016/j.tim.2014.12.004
Calisher CH, 2006, CLIN MICROBIOL REV, V19, P531, DOI 10.1128/

CMR.00017-06
Chua KB, 2002, ARCH VIROL, V147, P1323, DOI 10.1007/s00705-002-0815-5
de Weerd NA, 2012, IMMUNOL CELL BIOL, V90, P483, DOI 10.1038/icb.2012.9
Drexler JF, 2012, NAT COMMUN, V3, DOI 10.1038/ncomms1796
Frémond ML, 2021, J CLIN IMMUNOL, V41, P501, DOI 10.1007/s10875-021-00974-z
Gouilh MA, 2018, VIROLOGY, V517, P88, DOI 10.1016/j.virol.2018.01.014
Hadjadj J, 2020, SCIENCE, V369, P718, DOI 10.1126/science.abc6027
Halpin K, 2000, J GEN VIROL, V81, P1927, DOI 10.1099/0022-1317-81-8-1927
Hauser P, 2004, GASTROENTEROL CLIN N, V33, pS37, DOI 10.1016/j.gtc.2003.12.005
Horowitz B, 2004, WHO TECH REP SER, V924, P1
Kühnel D, 2017, TRANSFUSION, V57, P802, DOI 10.1111/trf.13964
Lau SKP, 2005, P NATL ACAD SCI USA, V102, P14040, DOI 10.1073/pnas.0506735102
Leroy EM, 2005, NATURE, V438, P575, DOI 10.1038/438575a
Li WD, 2005, SCIENCE, V310, P676, DOI 10.1126/science.1118391
Luis AD, 2013, P ROY SOC B-BIOL SCI, V280, DOI 10.1098/rspb.2012.2753
McNab F, 2015, NAT REV IMMUNOL, V15, P87, DOI 10.1038/nri3787
Middleton DJ, 2007, J COMP PATHOL, V136, P266, DOI 10.1016/j.jcpa.2007.03.002
Omatsu T, 2008, VET IMMUNOL IMMUNOP, V124, P169, DOI 10.1016/j.vetimm.2008.03.004
Pavlovich SS, 2018, CELL, V173, P1098, DOI 10.1016/j.cell.2018.03.070
Picard C, 2017, AUTOIMMUN REV, V16, P897, DOI 10.1016/j.autrev.2017.07.001
Pourrut X, 2009, BMC INFECT DIS, V9, DOI 10.1186/1471-2334-9-159
Rodero MP, 2017, NAT COMMUN, V8, DOI 10.1038/s41467-017-01932-3
Rodero MP, 2017, J EXP MED, V214, P1547, DOI 10.1084/jem.20161451
Rodhain E., 2015, Bulletin de la Societe de Pathologie Exotique, V108, P272, DOI 10.1007/s13149-015-0448-z
Sadier A, 2020, GENET MOL BIOL, V43, DOI [10.1590/1678-4685-gmb-2019-0146, 10.1590/1678-4685-GMB-2019-0146]
Schountz T, 2017, FRONT IMMUNOL, V8, DOI 10.3389/fimmu.2017.01098
Sétien AA, 1998, VACCINE, V16, P1122, DOI 10.1016/S0264-410X(98)80108-4
Simmons NB, 2005, SCIENCE, V307, P527, DOI 10.1126/science.1108871
Swanepoel R, 2007, EMERG INFECT DIS, V13, P1847, DOI 10.3201/eid1312.071115
Teeling EC, 2005, SCIENCE, V307, P580, DOI 10.1126/science.1105113
Towner JS, 2007, PLOS ONE, V2, DOI 10.1371/journal.pone.0000764
Towner JS, 2009, PLOS PATHOG, V5, DOI 10.1371/journal.ppat.1000536
Wacharapluesadee S, 2021, NAT COMMUN, V12, DOI 10.1038/s41467-021-21240-1
Wang LF, 2011, CURR OPIN VIROL, V1, P649, DOI 10.1016/

j.coviro.2011.10.013
   Williamson MM, 2000, J COMP PATHOL, V122, P201, DOI 10.1053/
jcpa.1999.0364
   Wong S, 2007, REV MED VIROL, V17, P67, DOI 10.1002/rmv.520
   Zhou P, 2016, P NATL ACAD SCI USA, V113, P2696, DOI 10.1073/
pnas.1518240113
NR 44
TC 11
Z9 12
U1 0
U2 11
PU FRONTIERS MEDIA SA
PI LAUSANNE
PA AVENUE DU TRIBUNAL FEDERAL 34, LAUSANNE, CH-1015, SWITZERLAND
SN 1664-3224
J9 FRONT IMMUNOL
JI Front. Immunol.
PD NOV 1
PY 2021
VL 12
AR 735866
DI 10.3389/fimmu.2021.735866
PG 10
WC Immunology
WE Science Citation Index Expanded (SCI-EXPANDED)
SC Immunology
GA XI8NN
UT WOS:000726361100001
PM 34790193
OA Green Submitted, Green Published, gold
DA 2024-10-21
ER

PT J
AU Becker, DJ
   Chumchal, MM
   Bentz, AB
   Platt, SG
   Czirják, GA
   Rainwater, TR
   Altizer, S
   Streicker, DG
AF Becker, Daniel J.
   Chumchal, Matthew M.
   Bentz, Alexandra B.
   Platt, Steven G.
   Czirjak, Gabor A.
   Rainwater, Thomas R.
   Altizer, Sonia
   Streicker, Daniel G.
TI Predictors and immunological correlates of sublethal mercury exposure in
   vampire bats
SO ROYAL SOCIETY OPEN SCIENCE

LA English
DT Article
DE agriculture; Chiroptera; ecoimmunology; ecotoxicology; spatio-temporal;
wildlife health
ID DESMODUS-ROTUNDUS; STABLE-ISOTOPES; IMMUNE FUNCTION; ORGANOCHLORINE
PESTICIDES; MYOTIS-LUCIFUGUS; MAYA DIET; LAND-USE; WILDLIFE; HEALTH;
SOILS
AB Mercury (Hg) is a pervasive heavy metal that often enters the environment from anthropogenic sources such as gold mining and agriculture. Chronic exposure to Hg can impair immune function, reducing the ability of animals to resist or recover from infections. How Hg influences immunity and susceptibility remains unknown for bats, which appear immunologically distinct from other mammals and are reservoir hosts of many pathogens of importance to human and animal health. We here quantify total Hg (THg) in hair collected from common vampire bats (Desmodus rotundus), which feed on blood and are the main reservoir hosts of rabies virus in Latin America. We examine how diet, sampling site and year, and bat demography influence THg and test the consequences of this variation for eight immune measures. In two populations from Belize, THg concentrations in bats were best explained by an interaction between long-term diet inferred from stable isotopes and year. Bats that foraged more consistently on domestic animals exhibited higher THg.
C1 [Becker, Daniel J.; Altizer, Sonia; Streicker, Daniel G.] Univ Georgia, Odum Sch Ecol, Athens, GA 30602 USA.
[Becker, Daniel J.; Altizer, Sonia] Univ Georgia, Ctr Ecol Infect Dis, Athens, GA 30602 USA.
[Bentz, Alexandra B.] Univ Georgia, Dept Poultry Sci, Athens, GA 30602 USA.
[Chumchal, Matthew M.] Texas Christian Univ, Dept Biol, Ft Worth, TX 76129 USA.
[Platt, Steven G.] Myanmar Program, Wildlife Conservat Soc, Yangon, Myanmar.
[Czirjak, Gabor A.] Leibniz Inst Zoo & Wildlife Res, Dept Wildlife Dis, Berlin, Germany.
[Rainwater, Thomas R.] Clemson Univ, Tom Yawkey Wildlife Ctr, Georgetown, SC USA.
[Rainwater, Thomas R.] Clemson Univ, Belle W Baruch Inst Coastal Ecol & Forest Sci, Georgetown, SC USA.
[Streicker, Daniel G.] Univ Glasgow, Inst Biodivers Anim Hlth & Comparat Med, Glasgow G12 8QQ, Lanark, Scotland.
[Streicker, Daniel G.] Univ Glasgow, MRC, Ctr Virus Res, Glasgow G61 1QH, Lanark, Scotland.
C3 University System of Georgia; University of Georgia; University System
of Georgia; University of Georgia; University System of Georgia;
University of Georgia; Texas Christian University; Leibniz Institut fur
Zoo und Wildtierforschung; Clemson University; Clemson University;
University of Glasgow; University of Glasgow

RP Becker, DJ (corresponding author), Univ Georgia, Odum Sch Ecol, Athens, GA 30602 USA.; Becker, DJ (corresponding author), Univ Georgia, Ctr Ecol Infect Dis, Athens, GA 30602 USA.
EM dbecker@uga.edu
RI Czirják, Gábor/F-5440-2011; Platt, Steven/KFB-1259-2024; Becker, Daniel/A-6466-2014
OI Chumchal, Matthew/0000-0001-7160-3157; Becker, Daniel/0000-0003-4315-8628; Czirjak, Gabor Arpad/0000-0001-9488-0069; Streicker, Daniel/0000-0001-7475-2705; Bentz, Alexandra/0000-0003-2445-4886
FU NSF Graduate Research Fellowship; ARCS Foundation Award; Sigma Xi; Odum School of Ecology; UGA Graduate School; Explorer's Club; UGA Biomedical and Health Sciences Institute; NSF [DEB-1020966, DEB-1518611]; NSF Research Coordination Network in Ecoimmunology [NSF IOS-0947177]; Texas Christian University Research and Creative Activities Fund Award; Wildlife Conservation Society; Leibniz Institute for Zoo and Wildlife Research; Yawkey Foundation; Tom Yawkey Wildlife Center and Clemson University; Sir Henry Dale Fellowship; Wellcome Trust and Royal Society [102507/Z/13/Z]; MRC [MC_UU_12014/8] Funding Source: UKRI
FX D.J.B. was funded by an NSF Graduate Research Fellowship, ARCS Foundation Award, Sigma Xi, the Odum School of Ecology, UGA Graduate School, Explorer's Club and UGA Biomedical and Health Sciences Institute; fieldwork in Belize was also supported by NSF DEB-1020966. Training in immune assays at the Leibniz Institute for Zoo and Wildlife Research was supported by the NSF Research Coordination Network in Ecoimmunology (NSF IOS-0947177). M.M.C. was supported by a Texas Christian University Research and Creative Activities Fund Award, A.B.B. was supported by an NSF Graduate Research Fellowship, S.G.P. was supported by the Wildlife Conservation Society, and G.A.C. was supported by the Leibniz Institute for Zoo and Wildlife Research. T.R.R. was supported by the Yawkey Foundation, Tom Yawkey Wildlife Center and Clemson University; this paper represents technical contribution number 6529 of the Clemson University Experiment Station. S.A. acknowledges support from NSF DEB-1518611, and D.G.S. was supported by a Sir Henry Dale Fellowship, jointly funded by the Wellcome Trust and Royal

Society
(102507/Z/13/Z).

CR Alonso ML, 2003, ENVIRON POLLUT, V125, P173, DOI 10.1016/S0269-7491(03)00073-3
[Anonymous], 2013, MuMIn: Multi-model inference. R package version 1.10.0. 1
[Anonymous], 1988, The Natural History of Vampire Bats
ARNASON T, 1982, AGRO-ECOSYSTEMS, V8, P27, DOI 10.1016/0304-3746(82)90012-9
Bagenstose LM, 1999, IMMUNOL RES, V20, P67, DOI 10.1007/BF02786508
Baker ML, 2013, ZOONOSES PUBLIC HLTH, V60, P104, DOI 10.1111/j.1863-2378.2012.01528.x
Bechshoft T, 2015, ECOTOXICOLOGY, V24, P1315, DOI 10.1007/s10646-015-1506-9
Becker DJ, 2015, ECOL LETT, V18, P483, DOI 10.1111/ele.12428
Becker DJ, 2014, BIOL LETTERS, V10, DOI 10.1098/rsbl.2014.0309
Becker DJ, 2017, DRYAD DIGITAL REPOSI
Beldomenico PM, 2008, P ROY SOC B-BIOL SCI, V275, P1753, DOI 10.1098/rspb.2008.0147
BENJAMINI Y, 1995, J R STAT SOC B, V57, P289, DOI 10.1111/j.2517-6161.1995.tb02031.x
Bond AL, 2015, P ROY SOC B-BIOL SCI, V282, DOI 10.1098/rspb.2015.0032
Brook CE, 2015, TRENDS MICROBIOL, V23, P172, DOI 10.1016/j.tim.2014.12.004
Burnham K. P., 2002, Model Selection and Multimodel Inference: A Practical InformationTheoretic Approach, VSecond edition, DOI [10.1007/b97636, DOI 10.1007/B97636]
Chan HM, 2003, HUM ECOL RISK ASSESS, V9, P867, DOI 10.1080/713610013
Chaplin DD, 2010, J ALLERGY CLIN IMMUN, V125, pS3, DOI 10.1016/j.jaci.2009.12.980
Chumchal MM, 2011, ENVIRON TOXICOL CHEM, V30, P1153, DOI 10.1002/etc.477
Cribari-Neto F, 2010, J STAT SOFTW, V34, P1
DAUM JR, 1993, INT J IMMUNOPHARMACO, V15, P383, DOI 10.1016/0192-0561(93)90049-5
Davis AK, 2008, FUNCT ECOL, V22, P760, DOI 10.1111/j.1365-2435.2008.01467.x
Day RD, 2007, ENVIRON HEALTH PERSP, V115, P1421, DOI 10.1289/ehp.9918
DELPIETRO HA, 1992, PREV VET MED, V14, P13, DOI 10.1016/0167-5877(92)90080-Y
Delpietro VA, 2002, MAMM BIOL, V67, P65, DOI 10.1078/1616-5047-00011
DENIRO MJ, 1978, GEOCHIM COSMOCHIM AC, V42, P495, DOI 10.1016/0016-7037(78)90199-0
Dixon P, 2003, J VEG SCI, V14, P927, DOI 10.1111/j.1654-1103.2003.tb02228.x
Elbert RA, 1998, ENVIRON TOXICOL CHEM, V17, P210, DOI 10.1002/etc.5620170212
Ellington AA, 2010, CLIN CHEM, V56, P186, DOI 10.1373/clinchem.2009.127514

Evers D., 2008, MERCURY TERRESTRIAL
Fallacara DM, 2011, ENVIRON TOXICOL CHEM, V30, P1320, DOI 10.1002/etc.494
Farella N, 2006, SCI TOTAL ENVIRON, V368, P19, DOI 10.1016/j.scitotenv.2006.04.025
Finkelstein ME, 2007, ENVIRON TOXICOL CHEM, V26, P1896, DOI 10.1897/06-505R.1
Flache L, 2015, MAMM BIOL, V80, P178, DOI 10.1016/j.mambio.2015.01.007
French SS, 2012, BIOL OPEN, V1, P482, DOI 10.1242/bio.2012919
Frick WF, 2015, GLOBAL ECOL BIOGEOGR, V24, P741, DOI 10.1111/geb.12290
George BM, 2008, ENVIRON POLLUT, V152, P484, DOI 10.1016/j.envpol.2007.04.030
GILL TS, 1985, WATER AIR SOIL POLL, V24, P165
Heinrich SK, 2016, BIOL OPEN, V5, P550, DOI 10.1242/bio.014902
Henriques B, 2013, ENVIRON INT, V59, P408, DOI 10.1016/j.envint.2013.07.006
Hickey MBC, 2001, B ENVIRON CONTAM TOX, V66, P699
Karouna-Renier NK, 2014, ECOTOXICOLOGY, V23, P1419, DOI 10.1007/s10646-014-1284-9
Kupfer JA, 2004, AGR ECOSYST ENVIRON, V103, P509, DOI 10.1016/j.agee.2003.11.011
Lalancette A, 2003, DEV COMP IMMUNOL, V27, P735, DOI 10.1016/S0145-305X(03)00038-7
Lewis CA, 2013, ARCH ENVIRON CON TOX, V64, P327, DOI 10.1007/s00244-012-9830-z
Mahaffey K., 1997, Mercury study report to Congress. Volume 7. Characterization of human health and wildlife risks from mercury exposure in the United States
McCartor A., 2010, World's Worst Pollution Problems Report
Meerman JC, 2012, BIODIVERSITY ENV RES
Moore MS, 2011, PLOS ONE, V6, DOI 10.1371/journal.pone.0027430
Murray MH, 2016, BIOL CONSERV, V204, P163, DOI 10.1016/j.biocon.2016.10.034
Nam DH, 2012, ECOTOXICOLOGY, V21, P1094, DOI 10.1007/s10646-012-0864-9
Nicholson FA, 2003, SCI TOTAL ENVIRON, V311, P205, DOI 10.1016/S0048-9697(03)00139-6
OHI G, 1976, B ENVIRON CONTAM TOX, V15, P175, DOI 10.1007/BF01685157
PALHETA D, 1995, SCI TOTAL ENVIRON, V168, P63, DOI 10.1016/0048-9697(95)04533-7
Parnell AC, 2013, ENVIRONMETRICS, V24, P387, DOI 10.1002/env.2221
Parnell AC, 2010, PLOS ONE, V5, DOI 10.1371/journal.pone.0009672
PETERSON BJ, 1987, ANNU REV ECOL SYST, V18, P293, DOI 10.1146/annurev.es.18.110187.001453
Post DM, 2002, ECOLOGY, V83, P703, DOI 10.2307/3071875
Raats M. M., 1992, Food Quality and Preference, V3, P89, DOI 10.1016/0950-3293(91)90028-D
Rainwater TR, 2007, SCI TOTAL ENVIRON, V373, P146, DOI 10.1016/j.scitotenv.2006.11.010
Rainwater TR, 2005, ENVIRON TOXICOL CHEM, V24, P665, DOI 10.1897/04-223R.1

Rainwater TR, 2002, ARCH ENVIRON CON TOX, V42, P319, DOI 10.1007/s00244-001-0020-7
Richards SA, 2005, ECOLOGY, V86, P2805, DOI 10.1890/05-0074
Robinson Timothy P, 2007, Vet Ital, V43, P745
Scheulhammer AM, 2007, AMBIO, V36, P12, DOI 10.1579/0044-7447(2007)36[12:EOEMOT]2.0.CO;2
Schneeberger K, 2014, PLOS ONE, V9, DOI 10.1371/journal.pone.0108268
Schneeberger K, 2013, PLOS ONE, V8, DOI 10.1371/journal.pone.0054023
Schneider MC, 2009, REV PANAM SALUD PUBL, V25, P260, DOI 10.1590/S1020-49892009000300010
Silbergeld EK, 2000, IMMUNOPHARM IMMUNOT, V22, P685, DOI 10.3109/08923970009016432
Simas AB, 2010, COMPUT STAT DATA AN, V54, P348, DOI 10.1016/j.csda.2009.08.017
Smithson M, 2006, PSYCHOL METHODS, V11, P54, DOI 10.1037/1082-989X.11.1.54
Smyth GK, 1999, ENVIRONMETRICS, V10, P695, DOI 10.1002/(SICI)1099-095X(199911/12)10:6<695::AID-ENV385>3.0.CO;2-M
Streicker DG, 2016, J APPL ECOL, V53, P1280, DOI 10.1111/1365-2664.12690
Swenson JJ, 2011, PLOS ONE, V6, DOI 10.1371/journal.pone.0018875
Syaripuddin K, 2014, ECOTOXICOLOGY, V23, P1164, DOI 10.1007/s10646-014-1258-y
Tieleman BI, 2005, P ROY SOC B-BIOL SCI, V272, P1715, DOI 10.1098/rspb.2005.3155
TIESZEN LL, 1993, J ARCHAEOL SCI, V20, P25, DOI 10.1006/jasc.1993.1002
Undeger U, 1996, TOXICOLOGY, V109, P167, DOI 10.1016/0300-483X(96)03333-1
USEPA, 1998, MERC SOL SOL THERM D
Vas J, 2008, ANN NY ACAD SCI, V1143, P240, DOI 10.1196/annals.1443.022
Voigt CC, 2006, J MAMMAL, V87, P1, DOI 10.1644/05-MAMM-F-276R1.1
Voigt CC, 2012, ETHOLOGY, V118, P260, DOI 10.1111/j.1439-0310.2011.02004.x
Wada H, 2010, ECOTOXICOLOGY, V19, P1277, DOI 10.1007/s10646-010-0513-0
Wayland M, 2002, ENVIRON RES, V90, P47, DOI 10.1006/enrs.2002.4384
WHITE CD, 1993, J ANTHROPOL RES, V49, P347, DOI 10.1086/jar.49.4.3630154
WHITE CD, 1989, J ARCHAEOL SCI, V16, P451, DOI 10.1016/0305-4403(89)90068-X
Wimsatt W. A., 1962, Journal of Mammalogy, V43, P17, DOI 10.2307/1376876
Wolfe MF, 1998, ENVIRON TOXICOL CHEM, V17, P146, DOI [10.1897/1551-5028(1998)017<0146:EOMOWA>2.3.CO;2, 10.1002/etc.5620170203]
Wray AK, 2016, ECOHEALTH, V13, P761, DOI 10.1007/s10393-016-1183-z
Yates DE, 2014, ECOTOXICOLOGY, V23, P45, DOI 10.1007/s10646-013-1150-1

Zahir F, 2005, ENVIRON TOXICOL PHAR, V20, P351, DOI 10.1016/j.etap.2005.03.007
Zetun CB, 2009, J VENOM ANIM TOXINS, V15, P546, DOI 10.1590/S1678-91992009000300014
Zhou P, 2016, P NATL ACAD SCI USA, V113, P2696, DOI 10.1073/pnas.1518240113
Zukal J, 2015, MAMM BIOL, V80, P220, DOI 10.1016/j.mambio.2015.01.001
NR 93
TC 43
Z9 48
U1 1
U2 47
PU ROYAL SOC
PI LONDON
PA 6-9 CARLTON HOUSE TERRACE, LONDON SW1Y 5AG, ENGLAND
SN 2054-5703
J9 ROY SOC OPEN SCI
JI R. Soc. Open Sci.
PD APR
PY 2017
VL 4
IS 4
AR 170073
DI 10.1098/rsos.170073
PG 13
WC Multidisciplinary Sciences
WE Science Citation Index Expanded (SCI-EXPANDED); Social Science Citation Index (SSCI)
SC Science & Technology - Other Topics
GA ET8CZ
UT WOS:000400527200033
PM 28484633
OA Green Accepted, Green Published, gold
DA 2024-10-21
ER

PT J
AU Nawae, W
Sonthirod, C
Yoocha, T
Waiyamitra, P
Soisook, P
Tangphatsornruang, S
Pootakham, W
AF Nawae, Wanapinun
Sonthirod, Chutima
Yoocha, Thippawan
Waiyamitra, Pitchaporn
Soisook, Pipat
Tangphatsornruang, Sithichoke
Pootakham, Wirulda
TI Genome assembly of the Pendlebury's roundleaf bat, Hipposideros pendleburyi, revealed the expansion of Tc1/Mariner DNA

transposons in
   Rhinolophoidea
SO DNA RESEARCH
LA English
DT Article
DE Pendlebury's roundleaf bat; threatened animals; genome assembly; 10x
   linked-reads; transposable elements
ID HORIZONTAL TRANSFER; SELECTION; COMPLEX; PROTEIN; EVOLUTION; GRANZYME;
   MAMMALS; SINES; TOOL
AB Bats (Chiroptera) constitute the second largest order of mammals and have several distinctive features, such as true self-powered flight and strong immunity. The Pendlebury's roundleaf bat, Hipposideros pendleburyi, is endemic to Thailand and listed as a vulnerable species. We employed the 10x Genomics linked-read technology to obtain a genome assembly of H. pendleburyi. The assembly size was 2.17 Gb with a scaffold N50 length of 15,398,518 bases. Our phylogenetic analysis placed H. pendleburyi within the rhinolophoid clade of the suborder Yinpterochiroptera. A synteny analysis showed that H. pendleburyi shared conserved chromosome segments (up to 105 Mb) with Rhinolophus ferrumequinum and Phyllostomus discolor albeit having different chromosome numbers and belonging different families. We found positive selection signals in genes involved in inflammation, spermatogenesis and Wnt signalling. The analyses of transposable elements suggested the contraction of short interspersed nuclear elements (SINEs) and the accumulation of young mariner DNA transposons in the analysed hipposiderids. Distinct mariners were likely horizontally transferred to hipposiderid genomes over the evolution of this family. The lineage-specific profiles of SINEs and mariners might involve in the evolution of hipposiderids and be associated with the phylogenetic separations of these bats from other bat families.
C1 [Nawae, Wanapinun; Sonthirod, Chutima; Yoocha, Thippawan; Waiyamitra, Pitchaporn; Tangphatsornruang, Sithichoke; Pootakham, Wirulda] Natl Sci & Technol Dev Agcy, Natl Omics Ctr NOC, Thailand Sci Pk, Pathum Thani, Thailand.
   [Soisook, Pipat] Prince Songkla Univ, Princess Maha Chakri Sirindhorn Nat Hist Museum, Hat Yai, Thailand.
C3 National Science & Technology Development Agency - Thailand; Prince of
   Songkla University
RP Pootakham, W (corresponding author), Natl Sci & Technol Dev Agcy, Natl Omics Ctr NOC, Thailand Sci Pk, Pathum Thani, Thailand.
EM wirulda@alumni.stanford.edu
RI Pootakham, Wirulda/Q-9366-2019; Yoocha, Thippawan/D-8086-2012; SOISOOK,
   PIPAT/HPH-3274-2023; Tangphatsornruang, Sithichoke/G-3633-2010
OI Tangphatsornruang, Sithichoke/0000-0003-2673-0012; Nawae,
   Wanapinun/0000-0001-9228-6963
FU National Science and Technology Development Agency (NSTDA), Thailand
   [P-19-52245]; Thailand Research Fund (TRF) [DBG6180028]
FX This work was supported by the National Science and Technology

Development Agency (NSTDA), Thailand, grant number: P-19-52245 and
Thailand Research Fund (TRF), grant number: DBG6180028.
CR Anisimova M, 2001, MOL BIOL EVOL, V18, P1585, DOI 10.1093/oxfordjournals.molbev.a003945
Arafat M, 2017, J MED GENET, V54, P633, DOI 10.1136/jmedgenet-2017-104514
Banerjee A, 2020, FRONT IMMUNOL, V11, DOI 10.3389/fimmu.2020.00026
Bateman A, 2019, NUCLEIC ACIDS RES, V47, pD506, DOI 10.1093/nar/gky1049
Bloomquist RF, 2015, P NATL ACAD SCI USA, V112, pE5954, DOI 10.1073/pnas.1514298112
Borodulina OR, 2005, GENE, V349, P197, DOI 10.1016/j.gene.2004.12.035
Borodulina OR, 1999, FEBS LETT, V457, P409, DOI 10.1016/S0014-5793(99)01059-5
Bouallègue M, 2017, BMC GENOMICS, V18, DOI 10.1186/s12864-017-3856-6
Bruna T, 2021, NAR GENOM BIOINFORM, V3, DOI 10.1093/nargab/lqaa108
Butelli E, 2017, PLANT PHYSIOL, V173, P2225, DOI 10.1104/pp.16.01701
Cantrell MA, 2008, GENETICS, V178, P393, DOI 10.1534/genetics.107.080275
Capella-Gutiérrez S, 2009, BIOINFORMATICS, V25, P1972, DOI 10.1093/bioinformatics/btp348
Chang JHT, 1999, HUM MOL GENET, V8, P575, DOI 10.1093/hmg/8.4.575
Chau BN, 2000, MOL CELL, V6, P31, DOI 10.1016/S1097-2765(00)00005-8
Cordaux R, 2006, P NATL ACAD SCI USA, V103, P8101, DOI 10.1073/pnas.0601161103
Darriba D, 2020, MOL BIOL EVOL, V37, P291, DOI 10.1093/molbev/msz189
Dewannieux M, 2003, NAT GENET, V35, P41, DOI 10.1038/ng1223
Dong D, 2017, MOL BIOL EVOL, V34, P20, DOI 10.1093/molbev/msw231
Donninelli G, 2020, J NEUROINFLAMM, V17, DOI 10.1186/s12974-020-01770-z
Dupeyron M, 2020, MOBILE DNA-UK, V11, DOI 10.1186/s13100-020-00212-0
Edgar RC, 2004, BMC BIOINFORMATICS, V5, P1, DOI 10.1186/1471-2105-5-113
Emms DM, 2019, GENOME BIOL, V20, DOI 10.1186/s13059-019-1832-y
Flynn JM, 2020, P NATL ACAD SCI USA, V117, P9451, DOI 10.1073/pnas.1921046117
Foley NM, 2015, MOL BIOL EVOL, V32, P313, DOI 10.1093/molbev/msu329
Gao FL, 2019, ECOL EVOL, V9, P3891, DOI 10.1002/ece3.5015
Gilbert C, 2010, NATURE, V464, P1347, DOI 10.1038/nature08939
Gogolevsky KP, 2009, GENOMICS, V93, P494, DOI 10.1016/j.ygeno.2009.02.001
Goh G, 2020, P NATL ACAD SCI USA, V117, P28939, DOI 10.1073/pnas.2003352117
Haas BJ, 2008, GENOME BIOL, V9, DOI 10.1186/gb-2008-9-1-r7

Holt C, 2011, BMC BIOINFORMATICS, V12, DOI 10.1186/1471-2105-12-491
IRMLER M, 1995, J EXP MED, V181, P1917, DOI 10.1084/jem.181.5.1917
IUCN, 2015, IUCN RED LIST THREAT, Patent No. [eT158085A760192, 158085760192]
IUCN Species Survival Commission (SSC), 2012, IUCN Red List categories and criteria, Vsecond ed.
Jebb D, 2020, NATURE, V583, P578, DOI 10.1038/s41586-020-2486-3
Juuri E, 2012, DEV CELL, V23, P317, DOI 10.1016/j.devcel.2012.05.012
Koito A, 2013, FRONT MICROBIOL, V4, DOI 10.3389/fmicb.2013.00028
Kozlov AM, 2019, BIOINFORMATICS, V35, P4453, DOI 10.1093/bioinformatics/btz305
Kramerov DA, 2011, HEREDITY, V107, P487, DOI 10.1038/hdy.2011.43
Kriventseva EV, 2019, NUCLEIC ACIDS RES, V47, pD807, DOI 10.1093/nar/gky1053
Kumar S, 2017, MOL BIOL EVOL, V34, P1812, DOI 10.1093/molbev/msx116
Lavery TH, 2014, ZOOL SCR, V43, P429, DOI 10.1111/zsc.12068
Lee SH, 2005, P NATL ACAD SCI USA, V102, P18075, DOI 10.1073/pnas.0503676102
Lefort V, 2017, MOL BIOL EVOL, V34, P2422, DOI 10.1093/molbev/msx149
Lemoine F, 2019, NUCLEIC ACIDS RES, V47, pW260, DOI 10.1093/nar/gkz303
Li H, 2018, BIOINFORMATICS, V34, P3094, DOI 10.1093/bioinformatics/bty191
Li WZ, 2006, BIOINFORMATICS, V22, P1658, DOI 10.1093/bioinformatics/btl158
Li Y, 2008, GENE DEV, V22, P3050, DOI 10.1101/gad.1687308
Li-Hawkins J, 2002, J CLIN INVEST, V110, P1191, DOI 10.1172/JCI200216309
Lieberman J, 2010, IMMUNOL REV, V235, P93, DOI 10.1111/j.0105-2896.2010.00902.x
Linton DM, 2020, SCI REP-UK, V10, DOI 10.1038/s41598-020-63538-y
Machovsky-Capuska GE, 2018, J ANIM ECOL, V87, P1286, DOI 10.1111/1365-2656.12856
Matsuyama M, 2009, PLOS GENET, V5, DOI 10.1371/journal.pgen.1000427
Monadjem A., HDB MAMMALS WORLD, V9, P227
Myers P., 2022, The animal diversity web
Nikaido M, 2020, DNA RES, V27, DOI 10.1093/dnares/dsaa021
Novick P, 2010, GENE, V449, P85, DOI 10.1016/j.gene.2009.08.017
Oliveira SG, 2012, MOBILE DNA-UK, V3, DOI 10.1186/1759-8753-3-14
Pace JK, 2008, P NATL ACAD SCI USA, V105, P17023, DOI 10.1073/pnas.0806548105
Pfeiffer B, 2013, J ZOOL, V289, P77, DOI 10.1111/j.1469-7998.2012.00970.x
Platt RN, 2016, MOBILE DNA-UK, V7, DOI 10.1186/s13100-016-0071-y
Platt RN, 2014, MOL BIOL EVOL, V31, P1536, DOI 10.1093/molbev/msu112
Price MN, 2010, PLOS ONE, V5, DOI 10.1371/journal.pone.0009490
Ray DA, 2008, GENOME RES, V18, P717, DOI 10.1101/gr.071886.107

Ray DA, 2007, MOL BIOL EVOL, V24, P632, DOI 10.1093/molbev/msl192
Sadier A, 2020, GENET MOL BIOL, V43, DOI [10.1590/1678-4685-gmb-2019-0146, 10.1590/1678-4685-GMB-2019-0146]
Satoh W, 2008, GENESIS, V46, P92, DOI 10.1002/dvg.20369
Seppey M, 2019, METHODS MOL BIOL, V1962, P227, DOI 10.1007/978-1-4939-9173-0_14
Shang YL, 2017, ELIFE, V6, DOI [10.7554/eLife.28199, 10.7554/eLife.28199.001]
Shen D, 2020, GENOME BIOL EVOL, V12, P1060, DOI 10.1093/gbe/evaa135
Sikes RS, 2016, J MAMMAL, V97, P663, DOI 10.1093/jmammal/gyw078
Smirnov DG, 2014, BIOL BULL+, V41, P60, DOI 10.1134/S1062359014010105
Soderlund C, 2006, GENOME RES, V16, P1159, DOI 10.1101/gr.5396706
Soderlund C, 2011, NUCLEIC ACIDS RES, V39, DOI 10.1093/nar/gkr123
Sotero-Caio CG, 2017, GENES-BASEL, V8, DOI 10.3390/genes8100272
Teeling EC, 2005, SCIENCE, V307, P580, DOI 10.1126/science.1105113
Teeling EC, 2018, ANNU REV ANIM BIOSCI, V6, P23, DOI 10.1146/annurev-animal-022516-022811
Tempel Sebastien, 2012, Methods Mol Biol, V859, P29, DOI 10.1007/978-1-61779-603-6_2
Thomas J, 2011, GENE, V474, P52, DOI 10.1016/j.gene.2010.12.007
van Lith M, 2007, MOL BIOL CELL, V18, P2795, DOI 10.1091/mbc.E07-02-0147
Varshney D, 2015, NAT COMMUN, V6, DOI 10.1038/ncomms7569
Thong VD, 2012, MAMMAL REV, V42, P166, DOI 10.1111/j.1365-2907.2011.00202.x
Wang H, 2020, GENOME BIOL EVOL, V12, P3740, DOI 10.1093/gbe/evz250
Webb AE, 2017, PEERJ COMPUT SCI, DOI 10.7717/peed-cs.118
WHITAKER JO, 1976, J MAMMAL, V57, P199, DOI 10.2307/1379533
Wilson D.E., HDB MAMMALS WORLD, V9
Yang L, 2019, MOBILE DNA-UK, V10, DOI 10.1186/s13100-019-0164-5
Yang ZH, 2005, MOL BIOL EVOL, V22, P1107, DOI 10.1093/molbev/msi097
Yang ZH, 2007, MOL BIOL EVOL, V24, P1586, DOI 10.1093/molbev/msm088
Zhang JZ, 2005, MOL BIOL EVOL, V22, P2472, DOI 10.1093/molbev/msi237
NR 89
TC 0
Z9 0
U1 0
U2 3
PU OXFORD UNIV PRESS
PI OXFORD
PA GREAT CLARENDON ST, OXFORD OX2 6DP, ENGLAND
SN 1340-2838
EI 1756-1663
J9 DNA RES
JI DNA Res.
PD AUG 23
PY 2022

VL 29
IS 5
AR dsac026
DI 10.1093/dnares/dsac026
PG 13
WC Genetics & Heredity
WE Science Citation Index Expanded (SCI-EXPANDED)
SC Genetics & Heredity
GA 5E3GE
UT WOS:000865515400001
PM 36214371
OA Green Published
DA 2024-10-21
ER

PT J
AU He, CZ
   Wei, YJ
   Zhu, YB
   Xia, Y
   Irwin, DM
   Liu, Y
AF He, Chunzheng
   Wei, Yujia
   Zhu, Yubo
   Xia, Yu
   Irwin, David M.
   Liu, Yang
TI Adaptive Evolution of C-Type Lysozyme in Vampire Bats
SO JOURNAL OF MOLECULAR EVOLUTION
LA English
DT Article
DE Chiroptera; Vampire bat; Chicken-type lysozyme; Positive selection;
   Sanguivorous
ID DETECTING POSITIVE SELECTION; STOMACH LYSOZYMES; FUNCTIONAL DIVERGENCE;
   DESMODUS-ROTUNDUS; LIKELIHOOD METHOD; CONVERGENCE; TESTS; GENE; DIET
AB In mammals, chicken-type (c-type) lysozymes are part of the innate immune system, killing bacteria by degrading peptidoglycan in their cell walls. Many of the studies on the evolution of c-type lysozymes have focused on its new digestive function, including the duplicated stomach lysozymes in ruminants. Similarly, in bats, gene duplications and subsequent adaptive evolution of c-type lysozyme have been reported in a clade of insectivorous species, which might have been driven by the need to digest chitin. However, no studies on the evolution of c-type lysozyme have been carried out in the second largest and dietary diverse bat family Phyllostomidae, which includes insectivorous, frugivorous, nectarivorous and sanguivorous species. Here, we sequenced and analyzed c-type lysozyme genes from four phyllostomid bats, the common vampire bat, the white-winged vampire bat, the lesser long-nosed bat and the big fruit-eating bat. Only a single lysozyme gene was identified in each of these species.

Evidence for positive selection on mature lysozyme was found on lineages leading to vampire bats, but not other bats with single copy lysozyme genes. Moreover, several amino acid substitutions found in mature lysozymes from the sanguivorous clade are predicted to have functional impacts, adding further evidence for the adaptive evolution of lysozyme in vampire bats. Functional adaptation of vampire bat lysozymes could be associated with anti-microbial defense, possibly driven by the specialized sanguivory-related habits of vampire bats.
C1 [He, Chunzheng; Wei, Yujia; Zhu, Yubo; Xia, Yu; Liu, Yang] Shenyang Agr Univ, Coll Anim Sci & Vet Med, Key Lab Zoonosis Liaoning Prov, Shenyang 110866, Liaoning, Peoples R China.
[Irwin, David M.] Univ Toronto, Dept Lab Med & Pathobiol, Toronto, ON M5S 18, Canada.
C3 Shenyang Agricultural University; University of Toronto
RP Liu, Y (corresponding author), Shenyang Agr Univ, Coll Anim Sci & Vet Med, Key Lab Zoonosis Liaoning Prov, Shenyang 110866, Liaoning, Peoples R China.
EM yliu@syau.edu.cn
RI Wei, Yujia/IAQ-8917-2023
OI Liu, Yang/0000-0002-9258-7237
CR Pacheco MA, 2007, COMP BIOCHEM PHYS A, V147, P808, DOI 10.1016/j.cbpa.2006.07.010
Becker DJ, 2018, PHILOS T R SOC B, V373, DOI 10.1098/rstb.2017.0089
Benavides JA, 2018, ZOONOSES PUBLIC HLTH, V65, P454, DOI 10.1111/zph.12456
Bielawski JP, 2004, J MOL EVOL, V59, P121, DOI 10.1007/s00239-004-2597-8
Bobrowiec PED, 2015, J MAMMAL, V96, P54, DOI 10.1093/jmammal/gyu002
Callewaert L, 2010, J BIOSCIENCES, V35, P127, DOI 10.1007/s12038-010-0015-5
CAMARA VM, 1984, AM J PHYSIOL, V247, pG19, DOI 10.1152/ajpgi.1984.247.1.G19
Carter G, 2015, PLOS ONE, V10, DOI 10.1371/journal.pone.0138430
Carter GG, 2015, P ROY SOC B-BIOL SCI, V282, DOI 10.1098/rspb.2015.2524
Carter GG, 2006, ACTA CHIROPTEROL, V8, P255, DOI 10.3161/1733-5329(2006)8[255:AHDIFT]2.0.CO;2
Choi Y, 2015, BIOINFORMATICS, V31, P2745, DOI 10.1093/bioinformatics/btv195
Darriba D, 2012, NAT METHODS, V9, P772, DOI 10.1038/nmeth.2109
Datzmann T, 2010, BMC EVOL BIOL, V10, DOI 10.1186/1471-2148-10-165
DOBSON DE, 1984, J BIOL CHEM, V259, P1607
Elizalde-Arellano C, 2007, ACTA CHIROPTEROL, V9, P314, DOI 10.3161/1733-5329(2007)9[314:FSBITH]2.0.CO;2
Francischetti IMB, 2013, J PROTEOMICS, V82, P288, DOI 10.1016/j.jprot.2013.01.009
Guex N, 1997, ELECTROPHORESIS, V18, P2714, DOI 10.1002/elps.1150181505
Gunnell Gregg F., 2005, Journal of Mammalian Evolution, V12, P209, DOI 10.1007/s10914-005-6945-2

HAMMER MF, 1987, J MOL EVOL, V24, P272, DOI 10.1007/BF02111240
Irwin DM, 2004, GENOME, V47, P1082, DOI 10.1139/G04-075
IRWIN DM, 1989, J BIOL CHEM, V264, P11387
Ito F, 2016, ACTA CHIROPTEROL, V18, P509, DOI 10.3161/15081109ACC2016.18.2.017
Kumar S, 2016, MOL BIOL EVOL, V33, P1870, DOI [10.1093/molbev/msw054, 10.1093/molbev/msv279]
Kunz TH, 2003, BAT ECOLOGY, P3
Leelapaibul W, 2005, ACTA CHIROPTEROL, V7, P111, DOI 10.3161/1733-5329(2005)7[111:DOWFBT]2.0.CO;2
Liu Y, 2014, MOL BIOL EVOL, V31, P2829, DOI 10.1093/molbev/msu240
Miller-Butterworth CM, 2007, MOL BIOL EVOL, V24, P1553, DOI 10.1093/molbev/msm076
Monteiro LR, 2011, BMC EVOL BIOL, V11, DOI 10.1186/1471-2148-11-137
Ronquist F, 2003, BIOINFORMATICS, V19, P1572, DOI 10.1093/bioinformatics/btg180
SIMMONS N.B., 2005, Mammal Species of the World: a taxonomic and geographic reference, V3rd, P1
Stamatakis A, 2006, BIOINFORMATICS, V22, P2688, DOI 10.1093/bioinformatics/btl446
STEWART CB, 1987, COLD SPRING HARB SYM, V52, P891, DOI 10.1101/SQB.1987.052.01.097
Stockmaier S, 2018, ANIM BEHAV, V140, P141, DOI 10.1016/j.anbehav.2018.04.021
SWANSON KW, 1991, J MOL EVOL, V33, P418, DOI 10.1007/BF02103133
Takano K, 2000, BIOCHEMISTRY-US, V39, P8655, DOI 10.1021/bi9928694
Teeling EC, 2005, SCIENCE, V307, P580, DOI 10.1126/science.1105113
THOMPSON JD, 1994, NUCLEIC ACIDS RES, V22, P4673, DOI 10.1093/nar/22.22.4673
Waterhouse A, 2018, NUCLEIC ACIDS RES, V46, pW296, DOI 10.1093/nar/gky427
Weadick CJ, 2012, MOL BIOL EVOL, V29, P1297, DOI 10.1093/molbev/msr311
Wen Y, 1999, MOL PHYLOGENET EVOL, V13, P474, DOI 10.1006/mpev.1999.0651
WILKINSON GS, 1986, ANIM BEHAV, V34, P1880, DOI 10.1016/S0003-3472(86)80274-3
WILKINSON GS, 1984, NATURE, V308, P181, DOI 10.1038/308181a0
Yang ZH, 1998, MOL BIOL EVOL, V15, P568, DOI 10.1093/oxfordjournals.molbev.a025957
Yang ZH, 2007, MOL BIOL EVOL, V24, P1586, DOI 10.1093/molbev/msm088
Mendoza MLZ, 2018, NAT ECOL EVOL, V2, P659, DOI 10.1038/s41559-018-0476-8
Zhang JZ, 2005, MOL BIOL EVOL, V22, P2472, DOI 10.1093/molbev/msi237
NR 46
TC 5
Z9 6
U1 1
U2 24

PU SPRINGER
PI NEW YORK
PA 233 SPRING ST, NEW YORK, NY 10013 USA
SN 0022-2844
EI 1432-1432
J9 J MOL EVOL
JI J. Mol. Evol.
PD DEC
PY 2019
VL 87
IS 9-10
BP 309
EP 316
DI 10.1007/s00239-019-09910-7
PG 8
WC Biochemistry & Molecular Biology; Evolutionary Biology; Genetics &
   Heredity
WE Science Citation Index Expanded (SCI-EXPANDED)
SC Biochemistry & Molecular Biology; Evolutionary Biology; Genetics &
   Heredity
GA JN1JB
UT WOS:000496658200004
PM 31506780
DA 2024-10-21
ER

PT J
AU Cowled, C
   Baker, M
   Tachedjian, M
   Zhou, P
   Bulach, D
   Wang, LF
AF Cowled, Christopher
   Baker, Michelle
   Tachedjian, Mary
   Zhou, Peng
   Bulach, Dieter
   Wang, Lin-Fa
TI Molecular characterisation of Toll-like receptors in the black flying
   fox <i>Pteropus alecto</i>
SO DEVELOPMENTAL AND COMPARATIVE IMMUNOLOGY
LA English
DT Article
DE Innate immunity; Viral infection; Toll-like receptor; Chiroptera;
   Pteropus alecto; Fruit bat
ID DNA TRANSPOSON ACTIVITY; HENDRA VIRUS; NIPAH VIRUS; FRUIT BATS;
   TRANSMISSION; RECOGNITION; SEQUENCE; PROTEIN; MORBILLIVIRUS; CHIROPTERA
AB Bats are believed to be reservoir hosts for a number of emerging
and re-emerging viruses, many of which are responsible for illness

and mortality in humans, livestock and other animals. In other vertebrates, early responses to viral infection involve engagement of Toll-like receptors (TLRs). which induce changes in gene expression collectively leading to an "antiviral state". In this study we report the cloning and bioinformatic analysis of a complete set of TLRs from the black flying fox Pteropus alecto, and perform quantitative tissue expression analysis of the nucleic acid-sensing TLRs 3, 7, 8 and 9. Full-length mRNA transcripts from TLRs homologous to human TLRs 1-10 were sequenced, as well as a nearly intact TLR13 pseudogene that was spliced and polyadenylated. This prototype data can now be used to design functional studies of the bat innate immune system. Crown Copyright (C) 2010 Published by Elsevier Ltd. All rights reserved.
C1 [Cowled, Christopher; Baker, Michelle; Tachedjian, Mary; Bulach, Dieter; Wang, Lin-Fa] CSIRO Livestock Ind, Australian Anim Hlth Lab, Geelong, Vic 3220, Australia.
[Baker, Michelle] Univ New Mexico, Dept Biol, Ctr Evolutionary & Theoret Immunol, Albuquerque, NM 87131 USA.
[Zhou, Peng] Chinese Acad Sci, Wuhan Inst Virol, State Key Lab Virol, Wuhan, Peoples R China.
[Wang, Lin-Fa] Australian Biosecur Cooperat Res Ctr Emerging Inf, Geelong, Vic 3220, Australia.
C3 Commonwealth Scientific & Industrial Research Organisation (CSIRO);
University of New Mexico; Chinese Academy of Sciences; Wuhan Institute
of Virology, CAS; Commonwealth Scientific & Industrial Research
Organisation (CSIRO)
RP Baker, M (corresponding author), CSIRO Livestock Ind, Australian Anim Hlth Lab, 5 Portarlington Rd, Geelong, Vic 3220, Australia.
EM michelle.baker@csiro.au
RI Zhou, Peng/I-8578-2015; lu, yuan/JZD-0832-2024; Baker,
Michelle/C-9694-2013; Cowled, Christopher/H-5308-2013; Bulach,
Dieter/D-5793-2011
OI Wang, Lin-Fa/0000-0003-2752-0535; Baker, Michelle/0000-0002-7993-9971;
Cowled, Christopher/0000-0002-1969-9371; Bulach,
Dieter/0000-0001-9823-6078
FU CSIRO
FX The authors wish to acknowledge support from a CSIRO CEO Science Leaders
award to L.-F.W. We thank Craig Smith, Carol De Jong, Hume Field, Gary
Crameri and Deborah Middleton for provision of bat tissues.
CR ALTSCHUL SF, 1990, J MOL BIOL, V215, P403, DOI 10.1016/S0022-2836(05)80360-2
Baker ML, 2010, IMMUNOGENETICS, V62, P173, DOI 10.1007/s00251-010-0425-4
Bieback K, 2002, J VIROL, V76, P8729, DOI 10.1128/JVI.76.17.8729-8736.2002
Blehert DS, 2009, SCIENCE, V323, P227, DOI 10.1126/science.1163874
Brinkmann MM, 2007, J CELL BIOL, V177, P265, DOI 10.1083/jcb.200612056

Calisher CH, 2006, CLIN MICROBIOL REV, V19, P531, DOI 10.1128/CMR.00017-06
Choe J, 2005, SCIENCE, V309, P581, DOI 10.1126/science.1115253
Chua KB, 2007, P NATL ACAD SCI USA, V104, P11424, DOI 10.1073/pnas.0701372104
Chua KB, 2000, SCIENCE, V288, P1432, DOI 10.1126/science.288.5470.1432
Comeron JM, 1999, BIOINFORMATICS, V15, P763, DOI 10.1093/bioinformatics/15.9.763
Crameri G, 2009, PLOS ONE, V4, DOI 10.1371/journal.pone.0008266
Field HE, 2007, CURR TOP MICROBIOL, V315, P133
Halpin K, 2000, J GEN VIROL, V81, P1927, DOI 10.1099/0022-1317-81-8-1927
Hasan U, 2005, J IMMUNOL, V174, P2942, DOI 10.4049/jimmunol.174.5.2942
Hofmann K., 1993, BIOL CHEM HOPPESEYLE, V374, P166, DOI [DOI 10.1515/BCHM3.1993.374.1-6.143, DOI 10.1016/J.JTBI.2009.11.002]
Hurst LD, 2002, TRENDS GENET, V18, P486, DOI 10.1016/S0168-9525(02)02722-1
Iha K, 2010, J VET MED SCI, V72, P217, DOI 10.1292/jvms.09-0050
Kim YM, 2008, NATURE, V452, P234, DOI 10.1038/nature06726
Krogh A, 2001, J MOL BIOL, V305, P567, DOI 10.1006/jmbi.2000.4315
Kurt-Jones EA, 2000, NAT IMMUNOL, V1, P398, DOI 10.1038/80833
Lau SKP, 2005, P NATL ACAD SCI USA, V102, P14040, DOI 10.1073/pnas.0506735102
Leroy EM, 2005, NATURE, V438, P575, DOI 10.1038/438575a
Letunic I, 2009, NUCLEIC ACIDS RES, V37, pD229, DOI 10.1093/nar/gkn808
Li WD, 2005, SCIENCE, V310, P676, DOI 10.1126/science.1118391
Lund O., 2002, CASP5 C ABSTRACTS, pA102
Madsen O, 2001, NATURE, V409, P610, DOI 10.1038/35054544
Matsushima N, 2007, BMC GENOMICS, V8, DOI 10.1186/1471-2164-8-124
Middleton DJ, 2007, J COMP PATHOL, V136, P266, DOI 10.1016/j.jcpa.2007.03.002
Murphy WJ, 2001, NATURE, V409, P614, DOI 10.1038/35054550
MURRAY K, 1995, EMERG INFECT DIS, V1, P31, DOI 10.3201/eid0101.950107
Nakajima T, 2008, IMMUNOGENETICS, V60, P727, DOI 10.1007/s00251-008-0332-0
Nielsen R, 2005, PLOS BIOL, V3, P976, DOI 10.1371/journal.pbio.0030170
Nikaido M, 2000, J MOL EVOL, V51, P318, DOI 10.1007/s002390010094
Nishihara H, 2006, P NATL ACAD SCI USA, V103, P9929, DOI 10.1073/pnas.0603797103
Omatsu T, 2008, VET IMMUNOL IMMUNOP, V124, P169, DOI 10.1016/j.vetimm.2008.03.004
Omatsu T, 2006, VET IMMUNOL IMMUNOP, V111, P309, DOI 10.1016/j.vetimm.2005.12.002
Opsal MA, 2006, GENE, V384, P45, DOI 10.1016/j.gene.2006.06.027
Patin E, 2009, PLOS GENET, V5, DOI 10.1371/journal.pgen.1000448
Ray DA, 2008, GENOME RES, V18, P717, DOI 10.1101/gr.071886.107
Ray DA, 2007, MOL BIOL EVOL, V24, P632, DOI 10.1093/molbev/msl192
Roach JC, 2005, P NATL ACAD SCI USA, V102, P9577, DOI 10.1073/pnas.0502272102

Roeder A, 2004, TRENDS MICROBIOL, V12, P44, DOI 10.1016/j.tim.2003.11.003
RZHETSKY A, 1992, J MOL EVOL, V35, P367, DOI 10.1007/BF00161174
Smith JDL, 2009, BIOL LETTERS, V5, P347, DOI 10.1098/rsbl.2009.0016
Speare R, 1997, Commun Dis Intell, V21, P117
Sulkin S E, 1974, Monogr Virol, V8, P1
Tabeta K, 2006, NAT IMMUNOL, V7, P156, DOI 10.1038/ni1297
Tamura K, 2007, MOL BIOL EVOL, V24, P1596, DOI 10.1093/molbev/msm092
THOMPSON JD, 1994, NUCLEIC ACIDS RES, V22, P4673, DOI 10.1093/nar/22.22.4673
Towner JS, 2009, PLOS PATHOG, V5, DOI 10.1371/journal.ppat.1000536
Tusnády GE, 2001, BIOINFORMATICS, V17, P849, DOI 10.1093/bioinformatics/17.9.849
Uematsu S, 2007, J BIOL CHEM, V282, P15319, DOI 10.1074/jbc.R700009200
Uematsu S, 2006, J MOL MED-JMM, V84, P712, DOI 10.1007/s00109-006-0084-y
van den Hurk AF, 2009, AM J TROP MED HYG, V81, P457, DOI 10.4269/ajtmh.2009.81.457
van der Poel WHM, 2006, VECTOR-BORNE ZOONOT, V6, P315, DOI 10.1089/vbz.2006.6.315
Williamson MM, 1998, AUST VET J, V76, P813, DOI 10.1111/j.1751-0813.1998.tb12335.x
Wong S, 2007, REV MED VIROL, V17, P67, DOI 10.1002/rmv.520
Xiao T, 2009, IMMUNOL RES, V43, P98, DOI 10.1007/s12026-008-8053-x
Xu YW, 2000, NATURE, V408, P111, DOI 10.1038/35040600
Yarovinsky F, 2006, INT J PARASITOL, V36, P255, DOI 10.1016/j.ijpara.2005.12.003
NR 60
TC 49
Z9 61
U1 1
U2 25
PU ELSEVIER SCI LTD
PI OXFORD
PA THE BOULEVARD, LANGFORD LANE, KIDLINGTON, OXFORD OX5 1GB, OXON, ENGLAND
SN 0145-305X
EI 1879-0089
J9 DEV COMP IMMUNOL
JI Dev. Comp. Immunol.
PD JAN
PY 2011
VL 35
IS 1
BP 7
EP 18
DI 10.1016/j.dci.2010.07.006
PG 12
WC Fisheries; Immunology; Veterinary Sciences; Zoology

WE Science Citation Index Expanded (SCI-EXPANDED)
SC Fisheries; Immunology; Veterinary Sciences; Zoology
GA 690RW
UT WOS:000285024400002
PM 20692287
OA Green Published
DA 2024-10-21
ER

PT J
AU Wu, JW
Zhang, LB
Shen, C
Sin, SYW
Lei, CQ
Zhao, HB
AF Wu, Jinwei
Zhang, Libiao
Shen, Chao
Sin, Simon Yung Wa
Lei, Caoqi
Zhao, Huabin
TI Comparative transcriptome analysis reveals molecular adaptations underlying distinct immunity and inverted resting posture in bats
SO INTEGRATIVE ZOOLOGY
LA English
DT Article
DE adaptation; bats; immunity; resting posture; transcriptome
ID GENOMES; EVOLUTION; ALIGNMENTS; SIZE
AB Understanding how natural selection shapes unique traits in mammals is a central topic in evolutionary biology. The mammalian order Chiroptera (bats) is attractive for biologists as well as the general public due to their specific traits of extraordinary immunity and inverted resting posture. However, genomic resources for bats that occupy key phylogenetic positions are not sufficient, which hinders comprehensive investigation of the molecular mechanisms underpinning the origin of specific traits in bats. Here, we sequenced the transcriptomes of 5 bats that are phylogenetically divergent and occupy key positions in the phylogenetic tree of bats. In combination with the available genomes of 19 bats and 21 other mammals, we built a database consisting of 10 918 one-to-one ortholog genes and reconstructed phylogenetic relationships of these mammals. We found that genes related to immunity, bone remodeling, and cardiovascular system are targets of natural selection along the ancestral branch of bats. Further analyses revealed that the T cell receptor signaling pathway involved in immune adaptation is specifically enriched in bats. Moreover, molecular adaptations of bone remodeling, cardiovascular system, and balance sensing may help to explain the reverted resting posture in bats. Our study provides valuable transcriptome resources, enabling us to tentatively identify genetic changes associated with bat-specific traits. This work is among the first to advance our understanding of the molecular underpinnings of inverted resting posture in bats, which could provide insight into healthcare applications such as

hypertension in humans.
C1 [Wu, Jinwei] China Three Gorges Univ, Engn Res Ctr Ecoenvironm Three Gorges Reservoir R, Minist Educ, Yichang, Peoples R China.
[Zhang, Libiao] Guangdong Acad Sci, Inst Zool, Guangdong Key Lab Anim Conservat & Resource Utili, Guangdong Publ Lab Wild Anim Conservat & Utilizat, Guangzhou, Peoples R China.
[Shen, Chao; Lei, Caoqi; Zhao, Huabin] Wuhan Univ, Coll Life Sci, Wuhan, Peoples R China.
[Sin, Simon Yung Wa] Univ Hong Kong, Sch Biol Sci, Hong Kong, Peoples R China.
C3 China Three Gorges University; Guangdong Academy of Sciences; Institute
of Zoology, Guangdong Academy of Sciences; Wuhan University; University
of Hong Kong
RP Zhao, HB (corresponding author), Wuhan Univ, Coll Life Sci, Dept Ecol, Wuhan 430072, Hubei, Peoples R China.
EM huabinzhao@whu.edu.cn
RI Zhao, Huabin/AAS-7513-2020
OI Wu, Jinwei/0000-0001-9920-4609; Sin, Simon Yung Wa/ 0000-0003-2484-2897
FU National Natural Science Foundation of China [31722051]; National Key
Research and Development Program of China [2021YFF0702004]; Fundamental
Research Funds for the Central Universities [2042021kf0217]
FX This work was supported in part by the National Natural Science
Foundation of China (31722051), the National Key Research and
Development Program of China (2021YFF0702004), and the Fundamental
Research Funds for the Central Universities (2042021kf0217) to H.Z.
CR Altringham J., 1996, Bats: Biology and Behaviour
Banerjee A, 2020, FRONT IMMUNOL, V11, DOI 10.3389/ fimmu.2020.00026
Bu DC, 2021, NUCLEIC ACIDS RES, V49, pW317, DOI 10.1093/nar/ gkab447
Calisher CH, 2006, CLIN MICROBIOL REV, V19, P531, DOI 10.1128/ CMR.00017-06
Canals M, 2005, ACTA CHIROPTEROL, V7, P65, DOI 10.3161/1733-5329(2005)7[65:RSOHAL]2.0.CO;2
Castresana J, 2000, MOL BIOL EVOL, V17, P540, DOI 10.1093/ oxfordjournals.molbev.a026334
Chen L, 2019, SCIENCE, V364, P1152, DOI 10.1126/science.aav6202
Currie SE, 2018, J COMP PHYSIOL B, V188, P695, DOI 10.1007/ s00360-018-1158-1
Darriba D, 2020, MOL BIOL EVOL, V37, P291, DOI 10.1093/molbev/ msz189
Donoghue M, 2000, CIRC RES, V87, pE1, DOI 10.1161/01.res.87.5.e1
Doronina L, 2017, GENOME RES, V27, P997, DOI 10.1101/ gr.210948.116
FISH FE, 1991, MAMMAL REV, V21, P181, DOI 10.1111/ j.1365-2907.1991.tb00292.x
Foley NM, 2016, PHILOS T R SOC B, V371, DOI 10.1098/

rstb.2015.0140
Grabherr MG, 2011, NAT BIOTECHNOL, V29, P644, DOI 10.1038/nbt.1883
Gutiérrez EG, 2021, INTEGR ZOOL, V16, P646, DOI 10.1111/1749-4877.12514
Hall L.S., 2000, Flying foxes: fruit and blossom bats of Australia
HEFFNER RS, 1993, J COMP NEUROL, V331, P418, DOI 10.1002/cne.903310311
Hu YB, 2017, P NATL ACAD SCI USA, V114, P1081, DOI 10.1073/pnas.1613870114
Irie N, 2009, J BIOL CHEM, V284, P14637, DOI 10.1074/jbc.M807598200
Irving AT, 2021, NATURE, V589, P363, DOI 10.1038/s41586-020-03128-0
Ishii M, 2009, NATURE, V458, P524, DOI 10.1038/nature07713
Jebb D, 2020, NATURE, V583, P578, DOI 10.1038/s41586-020-2486-3
Jiao HW, 2021, P NATL ACAD SCI USA, V118, DOI 10.1073/pnas.2021516118
Jiao HW, 2019, MOL BIOL EVOL, V36, P2171, DOI 10.1093/molbev/msz127
Kelley LA, 2015, NAT PROTOC, V10, P845, DOI 10.1038/nprot.2015.053
Kozlov AM, 2019, BIOINFORMATICS, V35, P4453, DOI 10.1093/bioinformatics/btz305
Li WD, 2005, SCIENCE, V310, P676, DOI 10.1126/science.1118391
Liu C, 2021, SCI ADV, V7, DOI 10.1126/sciadv.abe9459
Löytynoja A, 2005, P NATL ACAD SCI USA, V102, P10557, DOI 10.1073/pnas.0409137102
Lu Q, 2021, INTEGR ZOOL, V16, P659, DOI 10.1111/1749-4877.12509
Machado JP, 2016, BMC GENOMICS, V17, DOI 10.1186/s12864-016-2681-7
Mistry J, 2021, NUCLEIC ACIDS RES, V49, pD412, DOI 10.1093/nar/gkaa913
Santillán DDM, 2021, MOL ECOL, V30, P6449, DOI 10.1111/mec.16027
MORRISON DW, 1980, OECOLOGIA, V45, P270, DOI 10.1007/BF00346469
Pavlovich SS, 2018, CELL, V173, P1098, DOI 10.1016/j.cell.2018.03.070
Priatel JJ, 2002, IMMUNITY, V17, P617, DOI 10.1016/S1074-7613(02)00451-X
Ramírez-Fráncel LA, 2022, INTEGR ZOOL, V17, P2, DOI 10.1111/1749-4877.12552
Reidenerg JS, 2007, ANAT REC, V290, P507, DOI 10.1002/ar.20541
Roy E, 2010, P NATL ACAD SCI USA, V107, P15529, DOI 10.1073/pnas.1009743107
Scheben A., 2020, BIORXIV
Sercan Ö, 2010, J IMMUNOL, V184, P2855, DOI 10.4049/jimmunol.0902708
Shah K, 2021, SIGNAL TRANSDUCT TAR, V6, DOI 10.1038/s41392-021-00823-w
Shannon P, 2003, GENOME RES, V13, P2498, DOI 10.1101/gr.1239303
Simmons NB, 2005, MAMMAL SPECIES WORLD, V1, P312, DOI [DOI 10.1111/J.1365-2656.2010.01742.X, 10.1093/acprof]
Stathopoulos S, 2014, PLOS ONE, V9, DOI 10.1371/

journal.pone.0093336
Sun CN, 2022, INTEGR ZOOL, V17, P731, DOI 10.1111/1749-4877.12545
Sun ZP, 2021, CIRCULATION, V144, P788, DOI 10.1161/
CIRCULATIONAHA.121.053255
Suyama M, 2006, NUCLEIC ACIDS RES, V34, pW609, DOI 10.1093/nar/
gkl315
Szklarczyk D, 2019, NUCLEIC ACIDS RES, V47, pD607, DOI [10.1093/
nar/gky1131, 10.1093/nar/gkac1000]
Teeling EC, 2018, ANNU REV ANIM BIOSCI, V6, P23, DOI 10.1146/
annurev-animal-022516-022811
Towner JS, 2009, PLOS PATHOG, V5, DOI 10.1371/
journal.ppat.1000536
Vermeren M, 2017, MAMM GENOME, V28, P498, DOI 10.1007/
s00335-017-9718-3
Wang K, 2020, MOL ECOL, V29, P4366, DOI 10.1111/mec.15542
Xi ZX, 2014, SYST BIOL, V63, P919, DOI 10.1093/sysbio/syu055
Xu HS, 2004, DEVELOPMENT, V131, P3217, DOI 10.1242/dev.01174
Yan H, 2021, NAT ECOL EVOL, V5, P600, DOI 10.1038/
s41559-021-01407-1
Yang ZH, 2007, MOL BIOL EVOL, V24, P1586, DOI 10.1093/molbev/
msm088
Yu Y, 2015, BMC GENOMICS, V16, DOI 10.1186/1471-2164-16-S10-S10
Zhang C, 2018, BMC BIOINFORMATICS, V19, DOI 10.1186/
s12859-018-2129-y
Zhang GJ, 2013, SCIENCE, V339, P456, DOI 10.1126/science.1230835
Zou DH, 2021, MOL BIOL EVOL, V38, P3649, DOI 10.1093/molbev/
msab130
NR 61
TC 3
Z9 3
U1 5
U2 31
PU WILEY
PI HOBOKEN
PA 111 RIVER ST, HOBOKEN 07030-5774, NJ USA
SN 1749-4877
EI 1749-4869
J9 INTEGR ZOOL
JI Integr. Zool.
PD MAY
PY 2023
VL 18
IS 3
BP 493
EP 505
DI 10.1111/1749-4877.12676
EA SEP 2022
PG 13
WC Zoology
WE Science Citation Index Expanded (SCI-EXPANDED)
SC Zoology
GA AS2W5
UT WOS:000858343000001
PM 36049759

DA 2024-10-21
ER

PT J
AU Omatsu, T
   Watanabe, S
   Akashi, H
   Yoshikawa, Y
AF Omatsu, Tsutomu
   Watanabe, Shumpei
   Akashi, Hiroomi
   Yoshikawa, Yasuhiro
TI Biological characters of bats in relation to natural reservoir of
   emerging viruses
SO COMPARATIVE IMMUNOLOGY MICROBIOLOGY AND INFECTIOUS DISEASES
LA French
DT Article
DE Chiroptera; mitochondrial DNA; immune factors; body temperature;
Spleen;
   Retina; ELISA; YOKV
ID INFECTION; MICROCHIROPTERA; ECHOLOCATION; CHIROPTERA; INTERFERON;
   LYSSAVIRUS; ANTIBODIES; PHYLOGENY; EVOLUTION; ORIGIN
AB Many investigators focused on bats (Chiroptera) for their
specific character, i.e. echolocation system, phylogenic tree, food
practice and unique reproduction. However, most of basic information
about the vital functions related to anti-viral activity has been
unclear. For evaluating some animals as a natural reservoir or host
of infectious pathogens, it is necessary that not only their immune
system but also their biology, the environment of their living, food
habits and physiological features should be clarified and they
should be analyzed from these multi-view points. The majority of
current studies on infectious diseases have been conducted for the
elucidation of viral virulence using experimental animals or viral
gene function in vitro, but in a few case, researchers focused on
wild animal itself. In this paper, we described basic information
about bats as follows; genetic background, character of the
immunological factors, histological character of immune organs, the
physiological function and sensitivity of bat cells to viral
infection. (C) 2007 Published by Elsevier Ltd.
C1 Univ Tokyo, Grad Sch Agr & Life Sci, Dept Biomed Sci, Bunkyo Ku,
Tokyo 1138657, Japan.
   Univ Tokyo, Grad Sch Agr & Life Sci, Dept Vet Microbiol, Bunkyo
Ku, Tokyo 1138657, Japan.
C3 University of Tokyo; University of Tokyo
RP Omatsu, T (corresponding author), Univ Tokyo, Grad Sch Agr & Life
Sci, Dept Biomed Sci, Bunkyo Ku, 1-1-1 Yayoi, Tokyo 1138657, Japan.
EM aa37157@mail.ecc.u-tokyo.ac.jp
RI Omatsu, Tsutomu/C-8344-2013
CR BADWAIK NK, 1975, PLACENTA, V182, P124
   Bartels W, 1998, J COMP PHYSIOL B, V168, P233, DOI 10.1007/
s003600050141
   Basler CF, 2003, J VIROL, V77, P7945, DOI 10.1128/
JVI.77.14.7945-7956.2003
   BLEIER WJ, 1975, ANAT REC, V182, P237, DOI 10.1002/ar.1091820208

BUNDO K, 1985, J VIROL METHODS, V11, P15, DOI 10.1016/0166-0934(85)90120-X
CALISHER CH, 2005, CLIN MICROBIOL REV, V292, P531
CHANDA D, 2002, BIOTECH HISTOCHEM, V292, P129
Chua KB, 2003, J CLIN VIROL, V26, P265, DOI 10.1016/S1386-6532(02)00268-8
Chua KB, 2002, MICROBES INFECT, V4, P145, DOI 10.1016/S1286-4579(01)01522-2
COTTER JA, 1976, J COMP NEUROL, V115, P381
COVEY E, 2003, ANAT REC PART A, V13, P1103
Davis A, 2005, AM J TROP MED HYG, V73, P467, DOI 10.4269/ajtmh.2005.73.467
de Jong CE, 2005, THERIOGENOLOGY, V64, P1072, DOI 10.1016/j.theriogenology.2005.02.016
FIELD H, 2000, MICROBES INFECT, V172, P307
FUJIMOTO K, 1966, JPN J MED SCI BIOL, V151, P131
GAUNT MW, 1998, J GEN VIROL, V72, P1867
GOODMAN M, 1987, HUM BIOL, V40, P879
GOODMAN M, 1987, HUM BIOL, V40, P9
HAFLEIGH AS, 1966, SCIENCE, V151, P1530, DOI 10.1126/science.151.3717.1530
HALPIN K, 1998, J GEN VIROL, V4, P1927
Hance P, 2006, Med Trop (Mars), V66, P119
Hanna JN, 2000, MED J AUSTRALIA, V172, P597, DOI 10.5694/j.1326-5377.2000.tb124126.x
Hertzog PJ, 2003, TRENDS IMMUNOL, V24, P534, DOI 10.1016/j.it.2003.08.006
Honda K, 2005, INT IMMUNOL, V17, P1367, DOI 10.1093/intimm/dxh318
Hughes GJ, 2006, ARCH VIROL, V151, P2021, DOI 10.1007/s00705-005-0785-0
Inoue M, 2003, J VIROL, V77, P3238, DOI 10.1128/JVI.77.5.3238-3246.2003
Iwasaki A, 2004, NAT IMMUNOL, V5, P987, DOI 10.1038/ni1112
Iwasaki T, 2004, ARCH VIROL, V149, P1139, DOI 10.1007/s00705-003-0271-x
Johnson N, 2003, VET REC, V152, P383, DOI 10.1136/vr.152.13.383
KASHIWAZAKI Y, 1985, J VIROL METHODS, V11, P259
Kuno G, 1998, J VIROL, V72, P73, DOI 10.1128/JVI.72.1.73-83.1998
Lancaster WC, 2001, J EXP BIOL, V204, P4185
LAU SK, 2004, EMERG INFECT DIS, V121, P1108
Lau SKP, 2005, P NATL ACAD SCI USA, V102, P14040, DOI 10.1073/pnas.0506735102
Leroy EM, 2005, NATURE, V438, P575, DOI 10.1038/438575a
Lin YH, 2001, MOL BIOL EVOL, V18, P684, DOI 10.1093/oxfordjournals.molbev.a003850
Mackenzie JS, 2003, J APPL MICROBIOL, V94, p59S
MACKENZIE JS, 2004, ARCH VIROL S, V18, P97
Marom S, 2006, PHYSIOL BIOCHEM ZOOL, V79, P944, DOI 10.1086/505999
MILDE KF, 1993, BIOCHIM BIOPHYS ACTA, V1172, P315, DOI 10.1016/0167-4781(93)90220-8
MOSS CF, 2001, CURR OPIN NEUROBI 24, V204, P751
Murphy WJ, 2001, SCIENCE, V294, P2348, DOI 10.1126/science.1067179

Murphy WJ, 2004, TRENDS GENET, V20, P631, DOI 10.1016/j.tig.2004.09.005
Nikaido M, 2001, J MOL EVOL, V53, P508, DOI 10.1007/s002390010241
Nikaido M, 2000, J MOL EVOL, V51, P318, DOI 10.1007/s002390010094
Nishihara H, 2006, P NATL ACAD SCI USA, V103, P9929, DOI 10.1073/pnas.0603797103
Normile D, 2005, SCIENCE, V309, P2154, DOI 10.1126/science.309.5744.2154
NOVACEK MJ, 1992, NATURE, V356, P121, DOI 10.1038/356121a0
Omatsu T, 2003, EXP ANIM TOKYO, V52, P425, DOI 10.1538/expanim.52.425
Ozato K, 2002, BIOTECHNIQUES, P66
PECORARO MR, 1993, IMMUNOLOGY, V1172, P127
PENTNEY RP, 1976, BRAIN RES, V115, P479, DOI 10.1016/0006-8993(76)90363-2
Philbey AW, 1998, EMERG INFECT DIS, V4, P269, DOI 10.3201/eid0402.980214
Phung HTT, 2003, J VIROL METHODS, V109, P125, DOI 10.1016/S0166-0934(03)00062-4
REID JE, 2000, J NEUROVIROL, V122, P511
REID SP, 2003, J VIROL, V77, P5156
ROZASDENNIS GS, 2001, MEM I O CRUZ, V42, P427
Samuel CE, 2001, CLIN MICROBIOL REV, V14, P778, DOI 10.1128/CMR.14.4.778-809.2001
SARKAR SK, 1991, DEV COMP IMMUNOL, V15, P423, DOI 10.1016/0145-305X(91)90034-V
Sen GC, 2001, ANNU REV MICROBIOL, V55, P255, DOI 10.1146/annurev.micro.55.1.255
Sétien AA, 1998, VACCINE, V16, P1122, DOI 10.1016/S0264-410X(98)80108-4
SHOSHANI J, 1992, MOL PHYLOGENET EVOL, V356, P572
Singh UP, 2002, J EXP ZOOL, V292, P384, DOI 10.1002/jez.10086
SWAIN SL, 1984, J IMMUNOL, V132, P1118
Swanepoel R, 1996, EMERG INFECT DIS, V2, P321, DOI 10.3201/eid0204.960407
Tajima S, 2005, VIROLOGY, V332, P38, DOI 10.1016/j.virol.2004.06.052
Teeling EC, 2000, NATURE, V403, P188, DOI 10.1038/35003188
Tennant B C, 2001, ILAR J, V42, P89
THOMAS Y, 1984, J MOL CELL IMMUNOL, V1, P103
VARELASWESLEY I, 1982, AM J TROP MED HYG, V31, P1273, DOI 10.4269/ajtmh.1982.31.1273
VEILLETTE A, 1988, MOL CELL BIOL, V8, P4353, DOI 10.1128/MCB.8.10.4353
VEILLETTE A, 1984, CELL, V1, P301
Wacharapluesadee S, 2005, EMERG INFECT DIS, V11, P1949, DOI 10.3201/eid1112.050613
WADDELL PJ, 2000, GENOME INFORM, V51, P141
WARRILOW D, 2000, EMERG INFECT DIS 8, V81, P262
WARRILOW D, 2004, CURR TOP MICROBIOL I, V18, P25
WILLIAMSON MM, 1998, J COMP PATHOL, V16, P201
NR 77
TC 33
Z9 36

U1 0
U2 38
PU ELSEVIER SCI LTD
PI OXFORD
PA THE BOULEVARD, LANGFORD LANE, KIDLINGTON, OXFORD OX5 1GB, OXON,
ENGLAND
SN 0147-9571
EI 1878-1667
J9 COMP IMMUNOL MICROB
JI Comp. Immunol. Microbiol. Infect. Dis.
PD SEP
PY 2007
VL 30
IS 5-6
BP 357
EP 374
DI 10.1016/j.cimid.2007.05.006
PG 18
WC Immunology; Microbiology; Veterinary Sciences
WE Science Citation Index Expanded (SCI-EXPANDED)
SC Immunology; Microbiology; Veterinary Sciences
GA 225TK
UT WOS:000250540300008
PM 17706776
OA Green Published
DA 2024-10-21
ER

PT J
AU Li, J
   Sun, KP
   Dai, WT
   Leng, HX
   Li, AQ
   Feng, J
AF Li, Jun
   Sun, Keping
   Dai, Wentao
   Leng, Haixia
   Li, Aoqiang
   Feng, Jiang
TI Extensive Adaptive Variation in Gene Expression within and
between
   Closely Related Horseshoe Bats (Chiroptera, <i>Rhinolophus</i>)
Revealed
   by Three Organs
SO ANIMALS
LA English
DT Article
DE adaptive expression variation; natural selection; closely related
   species; phenotype; bat
ID HYBRID ZONE; EVOLUTION; DIVERGENCE; DIFFERENTIATION;
CONSERVATION;
   WIDESPREAD; CARRION; BIOLOGY; MODEL; CROW

AB Simple Summary The role of natural selection on evolution at the gene expression level is still not well-understood. However, it is important for better understanding the molecular mechanisms of species differentiation. By identifying differentially expressed genes with variation forced by natural selection, we found that expression variation and variation related to phenotypic divergence were extensively adaptive between both species and subspecies for three closely related horseshoe bats. In addition, despite the immune-related adaptive expression variation found both within and between species, there were different functional patterns with respect to adaptive expression variation between two taxonomic scales as well. These results could be helpful for better comprehending the role of natural selection on evolution, and providing scientific basis for the protection of bat species. In the process of species differentiation and adaption, the relative influence of natural selection on gene expression variation often remains unclear (especially its impact on phenotypic divergence). In this study, we used differentially expressed genes from brain, cochlea, and liver samples collected from two species of bats to determine the gene expression variation forced by natural selection when comparing at the interspecific (Rhinolophus siamensis and R. episcopus episcopus) and the intraspecific (R. e. episcopus and R. episcopus spp.) levels. In both cases, gene expression variation was extensively adaptive (>66.0%) and mainly governed by directional selection, followed by stabilizing selection, and finally balancing selection. The expression variation related to acoustic signals (resting frequency, RF) and body size (forearm length, FA) was also widely governed by natural selection (>69.1%). Different functional patterns of RF- or FA-related adaptive expression variation were found between the two comparisons, which manifested as abundant immune-related regulations between subspecies (indicating a relationship between immune response and phenotypic adaption). Our study verifies the extensive adaptive expression variation between both species and subspecies and provides insight into the effects of natural selection on species differentiation and adaptation as well as phenotypic divergence at the expression level.
C1 [Li, Jun; Sun, Keping; Dai, Wentao; Leng, Haixia; Li, Aoqiang; Feng, Jiang] Northeast Normal Univ, Jilin Prov Key Lab Anim Resource Conservat & Utili, Changchun 130117, Peoples R China.
[Li, Jun; Sun, Keping; Dai, Wentao; Leng, Haixia; Li, Aoqiang] Minist Educ, Key Lab Vegetat Ecol, Changchun 130024, Peoples R China.
[Feng, Jiang] Jilin Agr Univ, Coll Life Sci, Changchun 130118, Peoples R China.
C3 Northeast Normal University - China; Jilin Agricultural University
RP Sun, KP; Feng, J (corresponding author), Northeast Normal Univ, Jilin Prov Key Lab Anim Resource Conservat & Utili, Changchun 130117, Peoples R China.; Sun, KP (corresponding author), Minist Educ, Key Lab Vegetat Ecol, Changchun 130024, Peoples R China.; Feng, J (corresponding author), Jilin Agr Univ, Coll Life Sci, Changchun 130118, Peoples R China.
EM sunkp129@nenu.edu.cn; fengj@nenu.edu.cn
RI sun, ke/KMX-1265-2024

OI Li, Jun/0000-0002-7083-5359
CR Achieng AO, 2019, EBIOMEDICINE, V45, P278, DOI 10.1016/j.ebiom.2019.06.040
Blaner WS, 2020, CRIT REV BIOCHEM MOL, V55, P197, DOI 10.1080/10409238.2020.1768207
Blekhman R, 2008, PLOS GENET, V4, DOI 10.1371/journal.pgen.1000271
Brawand D, 2011, NATURE, V478, P343, DOI 10.1038/nature10532
Bryk J, 2013, ECOL EVOL, V3, P558, DOI 10.1002/ece3.447
Burdette SC, 2003, P NATL ACAD SCI USA, V100, P3605, DOI 10.1073/pnas.0637711100
Castro CN, 2020, J EXP MED, V217, DOI 10.1084/jem.20192275
Chan Esther T., 2009, Journal of Biology (London), V8, P33, DOI 10.1186/jbiol130
Chapman MG, 1999, MAR ECOL PROG SER, V180, P257, DOI 10.3354/meps180257
Cheng YL, 2017, FRONT ZOOL, V14, DOI 10.1186/s12983-017-0245-6
Chien Y.H., 2004, HUM GENET, V115, P532
Cortes-Hernandez J, 2004, EUR J IMMUNOL, V34, P3713, DOI 10.1002/eji.200425616
Danilovich S, 2019, SCI ADV, V5, DOI 10.1126/sciadv.aaw6503
Dave DD, 2018, INT J BIOMATH, V11, DOI 10.1142/S1793524518500882
Davies KTJ, 2013, FRONT ZOOL, V10, DOI 10.1186/1742-9994-10-2
DeCoster MA, 1995, ADV NEUROIMMUNOL, V5, P233, DOI 10.1016/0960-5428(95)00015-T
Delmonte OM, 2021, BLOOD, V138, P1019, DOI 10.1182/blood.2020008629
Enard W, 2002, SCIENCE, V296, P340, DOI 10.1126/science.1068996
Fraser HB, 2011, BIOESSAYS, V33, P469, DOI 10.1002/bies.201000094
Fraser HB, 2010, P NATL ACAD SCI USA, V107, P2977, DOI 10.1073/pnas.0912245107
Fushan AA, 2015, AGING CELL, V14, P352, DOI 10.1111/acel.12283
Galván I, 2020, J ZOOL SYST EVOL RES, V58, P519, DOI 10.1111/jzs.12329
Gilad Y, 2006, TRENDS GENET, V22, P456, DOI 10.1016/j.tig.2006.06.002
Goutte S, 2018, J EVOLUTION BIOL, V31, P148, DOI 10.1111/jeb.13210
Guo J, 2016, MOL BIOL EVOL, V33, P62, DOI 10.1093/molbev/msv196
Haas F, 2005, IBIS, V147, P649, DOI 10.1111/j.1474-919x.2005.00422.x
Haas F, 2009, MOL ECOL, V18, P294, DOI 10.1111/j.1365-294X.2008.04017.x
Hart T, 2013, BMC GENOMICS, V14, DOI 10.1186/1471-2164-14-778
He LZ, 2018, J IMMUNOL RES, V2018, DOI 10.1155/2018/5214187
He TH, 2019, J INTEGR PLANT BIOL, V61, P417, DOI 10.1111/jipb.12697
Hrynkow SH, 1998, NEUROSCIENCE, V87, P401, DOI 10.1016/S0306-4522(98)00155-9
Hughes AC, 2010, ACTA CHIROPTEROL, V12, P311, DOI 10.3161/150811010X537891
Khaitovich P, 2004, PLOS BIOL, V2, P682, DOI 10.1371/journal.pbio.0020132
Khaitovich P, 2006, NAT REV GENET, V7, P693, DOI 10.1038/nrg1940

Kuroiwa S, 2000, AM J OPHTHALMOL, V130, P672, DOI 10.1016/S0002-9394(00)00765-0
Leman JC, 2009, J EVOLUTION BIOL, V22, P163, DOI 10.1111/j.1420-9101.2008.01636.x
Lencer ES, 2017, BMC GENOMICS, V18, DOI 10.1186/s12864-017-3810-7
Li J, 2023, J MAMMAL, V104, P62, DOI 10.1093/jmammal/gyac103
Liang Yan, 2018, Oncotarget, V9, P14862, DOI 10.18632/oncotarget.23206
Lin Peter Bor-Chian, 2021, Alzheimers Dement, V17 Suppl 2, pe058724, DOI 10.1002/alz.058724
Liu T., 2020, J ZOOL SYST EVOL RES, V59, P249
Liu T, 2019, MOL PHYLOGENET EVOL, V139, DOI 10.1016/j.ympev.2019.106544
Luo B, 2019, BEHAV ECOL SOCIOBIOL, V73, DOI 10.1007/s00265-019-2766-9
Marambaud P, 2009, MOL NEURODEGENER, V4, DOI 10.1186/1750-1326-4-20
Matthews LP, 2021, MAR POLLUT BULL, V173, DOI 10.1016/j.marpolbul.2021.113043
Pavey SA, 2010, ANN NY ACAD SCI, V1206, P110, DOI 10.1111/j.1749-6632.2010.05765.x
Puechmaille SJ, 2011, NAT COMMUN, V2, DOI 10.1038/ncomms1582
Qin HY, 2023, INT J NEUROSCI, V133, P89, DOI 10.1080/00207454.2021.1886098
Ricard-Blum S, 2022, CURR OPIN STRUC BIOL, V74, DOI 10.1016/j.sbi.2022.102355
Shibkov AA, 2001, ZOOL ZH, V80, P454
Simoes BF, 2019, MOL BIOL EVOL, V36, P54, DOI 10.1093/molbev/msy192
Skokowa J, 2012, NAT MED, V18, P1550, DOI 10.1038/nm.2958
Sun KP, 2016, SCI REP-UK, V6, DOI 10.1038/srep35417
Sun KP, 2013, PLOS ONE, V8, DOI 10.1371/journal.pone.0070368
Szklarczyk D, 2019, NUCLEIC ACIDS RES, V47, pD607, DOI [10.1093/nar/gky1131, 10.1093/nar/gkac1000]
Trapnell C, 2010, NAT BIOTECHNOL, V28, P511, DOI 10.1038/nbt.1621
Udayanga KGS, 2016, INT IMMUNOL, V28, P611, DOI 10.1093/intimm/dxw047
Uebbing S, 2016, MOL ECOL, V25, P2015, DOI 10.1111/mec.13596
Vannatta JM, 2022, MAMMALIA, V86, P280, DOI 10.1515/mammalia-2021-0033
Wang H, 2020, GENOME BIOL EVOL, V12, P3740, DOI 10.1093/gbe/evz250
Wang Z, 2017, NAT ECOL EVOL, V1, DOI 10.1038/s41559-016-0021
Whitehead A, 2006, P NATL ACAD SCI USA, V103, P5425, DOI 10.1073/pnas.0507648103
Wolf JBW, 2010, MOL ECOL, V19, P162, DOI 10.1111/j.1365-294X.2009.04471.x
Yang JR, 2017, MOL BIOL EVOL, V34, P2125, DOI 10.1093/molbev/msx171
Zhao HB, 2019, FRONT ZOOL, V16, DOI 10.1186/s12983-019-0336-7
Zhao HB, 2009, P NATL ACAD SCI USA, V106, P8980, DOI 10.1073/pnas.0813201106
NR 66
TC 0

Z9 0
U1 1
U2 10
PU MDPI
PI BASEL
PA ST ALBAN-ANLAGE 66, CH-4052 BASEL, SWITZERLAND
SN 2076-2615
J9 ANIMALS-BASEL
JI Animals
PD DEC
PY 2022
VL 12
IS 23
AR 3432
DI 10.3390/ani12233432
PG 15
WC Agriculture, Dairy & Animal Science; Veterinary Sciences; Zoology
WE Science Citation Index Expanded (SCI-EXPANDED)
SC Agriculture; Veterinary Sciences; Zoology
GA 6V9HP
UT WOS:000895349500001
PM 36496954
OA gold, Green Published
DA 2024-10-21
ER

PT J
AU Voigt, CC
Fritze, M
Lindecke, O
Costantini, D
Petersons, G
Czirják, GA
AF Voigt, Christian C.
Fritze, Marcus
Lindecke, Oliver
Costantini, David
Petersons, Gunars
Czirjak, Gabor A.
TI The immune response of bats differs between pre-migration and migration
seasons
SO SCIENTIFIC REPORTS
LA English
DT Article
ID OXIDATIVE STRESS; COSTS; IMMUNOLOGY; LONGEVITY; DEFENSES; THRUSHES;
DISEASES; FLIGHT; BIRDS; HOST
AB Maintaining a competent immune system is energetically costly and thus immunity may be traded against other costly traits such as seasonal migration. Here, we tested in long-distance migratory Nathusius' pipistrelles (Pipistrellus nathusii), if selected branches of immunity are expressed differently in response to the energy demands and oxidative stress of aerial migration. During the

migration period, we observed higher baseline lymphocyte and lower neutrophil levels than during the pre-migration period, but no stronger response of cellular effectors to an antigen challenge. Baseline plasma haptoglobin, as a component of the humoral innate immunity, remained similar during both seasons, yet baseline plasma haptoglobin levels increased by a factor of 7.8 in migratory bats during an immune challenge, whereas they did not change during the pre-migration period. Oxidative stress was higher during migration than during pre-migration, yet there was no association between blood oxidative status and immune parameters, and immune challenge did not trigger any changes in oxidative stress, irrespective of season. Our findings suggest that humoral effectors of the acute phase response may play a stronger role in the first-line defense against infections for migrating bats compared to non-migrating bats. We conclude that Nathusius' pipistrelles allocate resources differently into the branches of their immune system, most likely following current demands resulting from tight energy budgets during migration.
C1 [Voigt, Christian C.; Fritze, Marcus; Lindecke, Oliver; Czirjak, Gabor A.] Leibniz Inst Zoo & Wildlife Res, Alfred Kowalke Str 17, D-10315 Berlin, Germany.
[Fritze, Marcus; Lindecke, Oliver] Free Univ Berlin, Inst Biol, Takustr 6, D-14895 Berlin, Germany.
[Costantini, David] CNRS, Museum Natl Hist Nat, Unite Physiol Mol & Adaptat PhyMA, 57 Rue Cuvier,CP32, F-75005 Paris, France.
[Petersons, Gunars] Latvia Univ Life Sci & Technol, Fac Vet Med, K Helmana St 8, LV-3004 Jelgava, Latvia.
C3 Leibniz Institut fur Zoo und Wildtierforschung; Free University of
Berlin; Centre National de la Recherche Scientifique (CNRS); Museum
National d'Histoire Naturelle (MNHN); Latvia University of Life Sciences
& Technologies
RP Voigt, CC (corresponding author), Leibniz Inst Zoo & Wildlife Res, Alfred Kowalke Str 17, D-10315 Berlin, Germany.
EM voigt@izw-berlin.de
RI Costantini, David/C-6006-2013; Czirják, Gábor/F-5440-2011; Fritze,
Marcus/H-3597-2015; Lindecke, Oliver/Z-4304-2019
OI Czirjak, Gabor Arpad/0000-0001-9488-0069; Lindecke,
Oliver/0000-0002-2545-9999; , Gunars/0000-0003-3914-3554
FU Projekt DEAL
FX Open Access funding enabled and organized by Projekt DEAL.
CR Altizer S, 2011, SCIENCE, V331, P296, DOI 10.1126/science.1194694
[Anonymous], 2004, 40th anniversary Bat Marking Centre Dresden-evaluation of methods and overview of results
[Anonymous], 2004, Myotis
Brunet-Rossinni AK, 2004, MECH AGEING DEV, V125, P11, DOI 10.1016/j.mad.2003.09.003
Buehler DM, 2008, PHILOS T R SOC B, V363, P247, DOI 10.1098/rstb.2007.2138
Canale CI, 2011, FUNCT ECOL, V25, P557, DOI 10.1111/j.1365-2435.2010.01815.x

Case AJ, 2011, FREE RADICAL BIO MED, V50, P448, DOI 10.1016/j.freeradbiomed.2010.11.025
Costantini D, 2008, J EXP BIOL, V211, P377, DOI 10.1242/jeb.012856
Costantini D, 2019, SCI TOTAL ENVIRON, V671, P1077, DOI 10.1016/j.scitotenv.2019.03.398
Costantini D, 2019, CURR ZOOL, V65, P147, DOI 10.1093/cz/zoy039
Costantini D, 2009, COMP BIOCHEM PHYS A, V153, P339, DOI 10.1016/j.cbpa.2009.03.010
Cray C, 2009, COMPARATIVE MED, V59, P517
Demas GE, 1997, AM J PHYSIOL-REG I, V273, pR1631, DOI 10.1152/ajpregu.1997.273.5.R1631
Dierschke V, 2005, BEHAV ECOL SOCIOBIOL, V57, P470, DOI 10.1007/s00265-004-0872-8
Drexler JF, 2012, NAT COMMUN, V3, DOI 10.1038/ncomms1796
Eikenaar C, 2018, ECOL EVOL, V8, P2721, DOI 10.1002/ece3.3756
Eikenaar C, 2016, BIOL LETTERS, V12, DOI 10.1098/rsbl.2016.0078
Field KA, 2015, PLOS PATHOG, V11, DOI 10.1371/journal.ppat.1005168
Fritze M, 2019, BIOL OPEN, V8, DOI 10.1242/bio.046078
Hasselquist D, 2007, J ORNITHOL, V148, pS571, DOI 10.1007/s10336-007-0201-x
Hasselquist D, 2012, ANIM BEHAV, V83, P1303, DOI 10.1016/j.anbehav.2012.03.025
Hayman DTS, 2013, ZOONOSES PUBLIC HLTH, V60, P2, DOI 10.1111/zph.12000
Hegemann A, 2018, OECOLOGIA, V188, P1011, DOI 10.1007/s00442-018-4291-3
Jenni-Eiermann S, 2014, PLOS ONE, V9, DOI 10.1371/journal.pone.0097650
Klasing Kirk C., 2004, Acta Zoologica Sinica, V50, P961
Kozak W.I.E.S., 1994, AM J PHYSIOL-REG I, V266, pR125
Krauel Jennifer J., 2013, P293
Lee KA, 2006, INTEGR COMP BIOL, V46, P1000, DOI 10.1093/icb/icl049
Lochmiller RL, 2000, OIKOS, V88, P87, DOI 10.1034/j.1600-0706.2000.880110.x
Luis AD, 2013, P ROY SOC B-BIOL SCI, V280, DOI 10.1098/rspb.2012.2753
Martin LB, 2003, P ROY SOC B-BIOL SCI, V270, P153, DOI 10.1098/rspb.2002.2185
McGuire LP, 2014, PLOS ONE, V9, DOI 10.1371/journal.pone.0115724
Moller AP, 1998, EVOL ECOL, V12, P945, DOI 10.1023/A:1006516222343
Olival KJ, 2017, NATURE, V546, P646, DOI 10.1038/nature22975
Otálora-Ardila A, 2016, PLOS ONE, V11, DOI 10.1371/journal.pone.0164938
Owen JC, 2008, CAN J ZOOL, V86, P638, DOI 10.1139/Z08-038
Owen JC, 2008, J ETHOL, V26, P383, DOI 10.1007/s10164-008-0092-1
Owen JC, 2006, CONDOR, V108, P389, DOI 10.1650/0010-5422(2006)108[389:SDIICO]2.0.CO;2
Popa-Lisseanu AG, 2009, J MAMMAL, V90, P1283, DOI 10.1644/09-MAMM-S-130R2.1
Roberts BJ, 2012, PLOS ONE, V7, DOI 10.1371/journal.pone.0042532

Salmon AB, 2009, FASEB J, V23, P2317, DOI 10.1096/fj.08-122523
Schneeberger K, 2014, NATURWISSENSCHAFTEN, V101, P285, DOI 10.1007/s00114-014-1155-5
Schneeberger K, 2013, J EXP BIOL, V216, P4514, DOI 10.1242/jeb.090837
Schneeberger K, 2013, PLOS ONE, V8, DOI 10.1371/journal.pone.0054023
Sikes RS, 2011, J MAMMAL, V92, P235, DOI 10.1644/10-MAMM-F-355.1
Speakman JR, 2003, BAT ECOLOGY, P430
Stockmaier S, 2015, BIOL LETTERS, V11, DOI 10.1098/rsbl.2015.0576
Svensson E, 1998, FUNCT ECOL, V12, P912, DOI 10.1046/j.1365-2435.1998.00271.x
Tobler M, 2015, PLOS ONE, V10, DOI 10.1371/journal.pone.0126155
Troxell SA, 2019, J EXP BIOL, V222, DOI 10.1242/jeb.176396
Voigt CC, 2012, J EXP BIOL, V215, P4340, DOI 10.1242/jeb.076224
Wang DT, 2010, J IMMUNOL, V184, P582, DOI 10.4049/jimmunol.0902352
Weber TP, 2007, EMERG INFECT DIS, V13, P1139, DOI 10.3201/eid1308.070319
Weise P, 2017, PEERJ, V5, DOI 10.7717/peerj.3570
Wibbelt G, 2010, BIOL LETTERS, V6, P438, DOI 10.1098/rsbl.2010.0267
Wikelski M, 2003, NATURE, V423, P704, DOI 10.1038/423704a
Wilhelm D, 2007, COMP BIOCHEM PHYS C, V146, P214, DOI 10.1016/j.cbpc.2006.11.015
Zhang GJ, 2013, SCIENCE, V339, P456, DOI 10.1126/science.1230835
NR 58
TC 21
Z9 23
U1 0
U2 19
PU NATURE PORTFOLIO
PI BERLIN
PA HEIDELBERGER PLATZ 3, BERLIN, 14197, GERMANY
SN 2045-2322
J9 SCI REP-UK
JI Sci Rep
PD OCT 15
PY 2020
VL 10
IS 1
AR 17384
DI 10.1038/s41598-020-74473-3
PG 11
WC Multidisciplinary Sciences
WE Science Citation Index Expanded (SCI-EXPANDED)
SC Science & Technology - Other Topics
GA OH6MF
UT WOS:000582705100022
PM 33060711
OA Green Published, gold
DA 2024-10-21
ER

PT J
AU Peel, AJ
   Baker, KS
   Hayman, DTS
   Broder, CC
   Cunningham, AA
   Fooks, AR
   Garnier, R
   Wood, JLN
   Restif, O
AF Peel, Alison J.
   Baker, Kate S.
   Hayman, David T. S.
   Broder, Christopher C.
   Cunningham, Andrew A.
   Fooks, Anthony R.
   Garnier, Romain
   Wood, James L. N.
   Restif, Olivier
TI Support for viral persistence in bats from age-specific serology and
   models of maternal immunity
SO SCIENTIFIC REPORTS
LA English
DT Article
ID COLORED FRUIT BATS; VIRUS-INFECTION; ANTIBODIES; RABIES; DYNAMICS;
   MEASLES; ASSAYS; HOST
AB Spatiotemporally-localised prediction of virus emergence from wildlife requires focused studies on the ecology and immunology of reservoir hosts in their native habitat. Reliable predictions from mathematical models remain difficult in most systems due to a dearth of appropriate empirical data. Our goal was to study the circulation and immune dynamics of zoonotic viruses in bat populations and investigate the effects of maternally-derived and acquired immunity on viral persistence. Using rare age-specific serological data from wild-caught Eidolon helvum fruit bats as a case study, we estimated viral transmission parameters for a stochastic infection model. We estimated mean durations of around 6 months for maternally-derived immunity to Lagos bat virus and African henipavirus, whereas acquired immunity was long-lasting (Lagos bat virus: mean 12 years, henipavirus: mean 4 years). In the presence of a seasonal birth pulse, the effect of maternally-derived immunity on virus persistence within modelled bat populations was highly dependent on transmission characteristics. To explain previous reports of viral persistence within small natural and captive E. helvum populations, we hypothesise that some bats must experience prolonged infectious periods or within-host latency. By further elucidating plausible mechanisms of virus persistence in bat populations, we contribute to guidance of future field studies.
C1 [Peel, Alison J.; Baker, Kate S.; Hayman, David T. S.; Garnier, Romain; Wood, James L. N.; Restif, Olivier] Univ Cambridge, Dept Vet Med, Dis Dynam Unit, Cambridge CB3 0ES, England.
   [Peel, Alison J.; Baker, Kate S.; Hayman, David T. S.;

Cunningham, Andrew A.] Zool Soc London, Inst Zool, Regents Pk, London NW1 4RY, England.
[Peel, Alison J.] Griffith Univ, Environm Futures Res Inst, Brisbane, Qld 4111, Australia.
[Baker, Kate S.] Univ Liverpool, Inst Integrat Biol, Liverpool L69 7ZB, Merseyside, England.
[Hayman, David T. S.; Fooks, Anthony R.] APHA, Addlestone KT15 3NB, Surrey, England.
[Hayman, David T. S.] Massey Univ, Hopkirk Res Inst, Mol Epidemiol & Publ Hlth Lab, Palmerston North 4442, New Zealand.
[Broder, Christopher C.] Uniformed Serv Univ Hlth Sci, Dept Microbiol & Immunol, Bethesda, MD 20814 USA.
C3 University of Cambridge; Zoological Society of London; Griffith University; University of Liverpool; Massey University; Uniformed Services University of the Health Sciences - USA
RP Peel, AJ (corresponding author), Univ Cambridge, Dept Vet Med, Dis Dynam Unit, Cambridge CB3 0ES, England.; Peel, AJ (corresponding author), Zool Soc London, Inst Zool, Regents Pk, London NW1 4RY, England.; Peel, AJ (corresponding author), Griffith Univ, Environm Futures Res Inst, Brisbane, Qld 4111, Australia.
EM alisonpeel@gmail.com
RI Broder, Christopher/E-1169-2013; Fooks, Anthony/F-5418-2010; Wood, James/A-1626-2008; , orestif/AAA-1532-2019; Fuentes, José/JZT-9323-2024; Cunningham, Andrew/E-7536-2010; Peel, Alison/I-3202-2012; Baker, Kate/P-2087-2016
OI Hayman, David/0000-0003-0087-3015; Wood, James/0000-0002-0258-3188; Peel, Alison/0000-0003-3538-3550; Baker, Kate/0000-0001-5850-1949; Restif, Olivier/0000-0001-9158-853X
FU Isaac Newton Trust; Queensland Accelerate Postdoctoral Research Fellowship; Wellcome Trust Clinical Research Career Development Fellowship [106690/A/14/Z]; Alborada Trust; European Union [278976]; Royal Society Wolfson Research Merit Award; Royal Society University Research Fellowship
FX We acknowledge Raina Plowright for thoughtful comments on an earlier draft of this manuscript. The authors thank the governments of Ghana, Tanzania, Malawi, Zambia, Uganda, Equatorial Guinea, and Sao Tome and Principe for facilitating this research. For their invaluable support in planning and implementing the field work, we also thank Guy-Crispin Gembu; Tiziana Lembo; Inaki Rodriguez-Prieto; Alex Torrance; Lucrecia Bile Osa Ahara; Heidi Ruffler; Ricardo Castro Cesar de Sa; Ricardo Faustino de Lima; Mariana Carvalho; Richard Suu-Ire; Andy

Alhassan; and
Meyir Ziekah. Funding for this study was provided by the Isaac Newton
Trust (AJP), a Queensland Accelerate Postdoctoral Research Fellowship
(AJP), a Wellcome Trust Clinical Research Career Development Fellowship
(106690/A/14/Z), the Alborada Trust (JLNW, OR), a European Union FP7
project ANTIGONE (Anticipating Global Onset of Novel Epidemics 278976)
(ACB, JLNW, AAC), a Royal Society Wolfson Research Merit Award (AAC),
and a Royal Society University Research Fellowship (OR).
CR [Anonymous], POWELL POWELLS UOBYQ
[Anonymous], ADAPTIVETAU TAU LEAP
[Anonymous], SOCIALITY BATS
[Anonymous], SCI REP
[Anonymous], NATURE COMMUNICATION
[Anonymous], 1993, A laboratory manual for cementum age determination of Alaska brown bear first premolar teeth
[Anonymous], SCI REP
Baker KS, 2014, J ANIM ECOL, V83, P415, DOI 10.1111/1365-2656.12153
BARTLETT MS, 1957, J R STAT SOC SER A-G, V120, P48, DOI 10.2307/2342553
Bates D, 2015, J STAT SOFTW, V67, P1, DOI 10.18637/jss.v067.i01
Blackwood JC, 2013, P NATL ACAD SCI USA, V110, P20837, DOI 10.1073/pnas.1308817110
Borremans B, 2016, PLOS COMPUT BIOL, V12, DOI 10.1371/journal.pcbi.1004882
Bossart KN, 2007, J VIROL METHODS, V142, P29, DOI 10.1016/j.jviromet.2007.01.003
Boulinier T, 2008, TRENDS ECOL EVOL, V23, P282, DOI 10.1016/j.tree.2007.12.006
Cador C, 2016, PLOS ONE, V11, DOI 10.1371/journal.pone.0163672
Cao Y, 2007, J CHEM PHYS, V126, DOI 10.1063/1.2745299
Clutton-Brock T.H., 2004, Soay Sheep
CONSTANTINE DG, 1968, PUBLIC HEALTH REP, V83, P303, DOI 10.2307/4593275
Diekmann O., 2000, Mathematical Epidemiology of Infectious Diseases: Model Building, Analysis and Interpretation
Dietrich M, 2015, ENVIRON MICROBIOL, V17, P4280, DOI 10.1111/1462-2920.12766
Drexler JF, 2012, NAT COMMUN, V3, DOI 10.1038/ncomms1796
Epstein JH, 2013, PLOS ONE, V8, DOI 10.1371/journal.pone.0067584
Field H, 2015, PLOS ONE, V10, DOI 10.1371/journal.pone.0144055
Fouchet D, 2006, J THEOR BIOL, V242, P81, DOI 10.1016/j.jtbi.2006.02.003
Franka R, 2008, J GEN VIROL, V89, P1998, DOI 10.1099/vir.0.83688-0
Georgea DB, 2011, P NATL ACAD SCI USA, V108, P10208, DOI 10.1073/pnas.1010875108
Gilbert AT, 2013, ECOHEALTH, V10, P298, DOI 10.1007/

s10393-013-0856-0
Grindstaff JL, 2010, J ORNITHOL, V151, P423, DOI 10.1007/s10336-009-0472-5
Halpin K, 2011, AM J TROP MED HYG, V85, P946, DOI 10.4269/ajtmh.2011.10-0567
Hasselquist D, 2009, PHILOS T R SOC B, V364, P51, DOI 10.1098/rstb.2008.0137
Hayman DTS, 2008, EMERG INFECT DIS, V14, P926, DOI 10.3201/eid1406.071421
Hayman DTS, 2016, BIOL CONSERV, V200, P131, DOI 10.1016/j.biocon.2016.06.003
Hayman DTS, 2015, P ROY SOC B-BIOL SCI, V282, DOI 10.1098/rspb.2014.2591
Hayman DTS, 2012, J MAMMAL, V93, P1393, DOI 10.1644/11-MAMM-A-270.1
Hayman DTS, 2008, PLOS ONE, V3, DOI 10.1371/journal.pone.0002739
Kuzmin IV, 2015, Bats and Viruses: A New Frontier of Emerging Infectious Diseases, P47, DOI DOI 10.1002/9781118818824.CH3
Lloyd-Smith JO, 2005, TRENDS ECOL EVOL, V20, P511, DOI 10.1016/j.tree.2005.07.004
Nelson DD, 2016, FRONT MICROBIOL, V6, DOI 10.3389/fmicb.2015.01415
O'Shea TJ, 2014, PLOS ONE, V9, DOI 10.1371/journal.pone.0086261
Peel AJ, 2014, P ROY SOC B-BIOL SCI, V281, DOI 10.1098/rspb.2013.2962
Peel AJ, 2017, ACTA CHIROPTEROL, V19, P77, DOI 10.3161/15081109ACC2017.19.1.006
Peel AJ, 2016, SCI DATA, V3, DOI 10.1038/sdata.2016.49
Peel AJ, 2013, J VIROL METHODS, V193, P295, DOI 10.1016/j.jviromet.2013.06.030
Peel AJ, 2012, PLOS ONE, V7, DOI 10.1371/journal.pone.0030346
Pepin KM, 2017, ECOL LETT, V20, P275, DOI 10.1111/ele.12732
Pinheiro J., 2018, R Package Version, V3, P1
PITCHERWILMOTT RW, 1980, CLIN EXP IMMUNOL, V41, P303
Plowright RK, 2016, PLOS NEGLECT TROP D, V10, DOI 10.1371/journal.pntd.0004796
Plowright RK, 2011, P ROY SOC B-BIOL SCI, V278, P3703, DOI 10.1098/rspb.2011.0522
Pulliam JRC, 2007, PLOS ONE, V2, DOI 10.1371/journal.pone.0000165
Pulliam JRC, 2012, J R SOC INTERFACE, V9, P89, DOI 10.1098/rsif.2011.0223
R Core Team, 2016, LANG ENV STAT COMP
Restif O, 2012, ECOL LETT, V15, P1083, DOI 10.1111/j.1461-0248.2012.01836.x
SATO H, 1979, AM J DIS CHILD, V133, P1240, DOI 10.1001/archpedi.1979.02130120032005
Schuh AJ, 2017, NAT COMMUN, V8, DOI 10.1038/ncomms14446
Sohayati AR, 2011, EPIDEMIOL INFECT, V139, P1570, DOI 10.1017/S0950268811000550
Suu-Ire RD, 2017, TROP MED INFECT DIS, V2, DOI 10.3390/tropicalmed2030025
Turmelle AS, 2010, J GEN VIROL, V91, P2360, DOI 10.1099/vir.0.020073-0
Venables W.N., 2002, Modern Applied Statistics with S, DOI [DOI

10.1007/978-0-387-21706-2, 10.1007/978-0-387-21706-2]
   Wells K, 2015, J R SOC INTERFACE, V12, DOI 10.1098/rsif.2014.1184
   Zuur Alain F., 2009, P1
NR 61
TC 33
Z9 37
U1 1
U2 19
PU NATURE PORTFOLIO
PI BERLIN
PA HEIDELBERGER PLATZ 3, BERLIN, 14197, GERMANY
SN 2045-2322
J9 SCI REP-UK
JI Sci Rep
PD MAR 1
PY 2018
VL 8
AR 3859
DI 10.1038/s41598-018-22236-6
PG 11
WC Multidisciplinary Sciences
WE Science Citation Index Expanded (SCI-EXPANDED)
SC Science & Technology - Other Topics
GA FX8OL
UT WOS:000426354200031
PM 29497106
OA Green Published, gold, Green Submitted
DA 2024-10-21
ER

PT J
AU Omatsu, T
   Nishimura, Y
   Bak, EJ
   Ishii, Y
   Tohya, Y
   Kyuwa, S
   Akashi, H
   Yoshikawa, Y
AF Omatsu, Tsutomu
   Nishimura, Yorihiro
   Bak, Eun Jung
   Ishii, Yoshiyuki
   Tohya, Yukinobu
   Kyuwa, Shigeru
   Akashi, Hiroomi
   Yoshikawa, Yasuhiro
TI Molecular cloning and sequencing of the cDNA encoding the bat CD4
SO VETERINARY IMMUNOLOGY AND IMMUNOPATHOLOGY
LA English
DT Article
DE bat; CD4; cDNA; cloning; Ig-like C-type 1 region
ID VIRUS
AB Chiroptera is thought to be a vector or a natural reservoir of

various pathogenic microbes. However, there are few basic studies on the subject of chiroptera immune systems. This is the first report to determine the sequence of bat CD4 cDNA. Comparison with other animals' CD4 and phylogenetic analysis have shown that bat CD4 had a higher homology to cat and dog CD4 than to human and mouse CD4. Moreover, from the analysis of the structure of the CD4 Ig-like C-type I region, in bat CD4 there was an insertion of 18 extra amino acids. In addition, bat CD4 lacked cystein, which suggested that the disulfide bond could not be formed. Human, monkey and mouse CD4 have the cystein and the disulfide bond, but pig, cat, whale and dog CD4, like that of the bat, lacked the cystein. We conducted the present study in order to help elucidate the infectious diseases derived from the bat as well as bat immune systems. (c) 2006 Elsevier B.V. All rights reserved.
C1 Univ Tokyo, Dept Biomed Sci, Grad Sch Agr & Life Sci, Bunkyo Ku, Tokyo 1138657, Japan.
   Univ Tokyo, Dept Vet Microbiol, Grad Sch Agr & Life Sci, Bunkyo Ku, Tokyo 1138657, Japan.
C3 University of Tokyo; University of Tokyo
RP Omatsu, T (corresponding author), Univ Tokyo, Dept Biomed Sci, Grad Sch Agr & Life Sci, Bunkyo Ku, 1-1-1 Yayoi, Tokyo 1138657, Japan.
EM aa37157@mail.ecc.u-tokyo.ac.jp
RI Omatsu, Tsutomu/C-8344-2013
CR Chua KB, 2002, MICROBES INFECT, V4, P145, DOI 10.1016/S1286-4579(01)01522-2
   Field H, 2004, ARCH VIROL, P113
   Field H, 2001, MICROBES INFECT, V3, P307, DOI 10.1016/S1286-4579(01)01384-3
   Hanna JN, 2000, MED J AUSTRALIA, V172, P597, DOI 10.5694/j.1326-5377.2000.tb124126.x
   Higgins DG, 1996, METHOD ENZYMOL, V266, P383
   Johnson N, 2002, VET REC, V151, P455
   Kumar S, 2001, BIOINFORMATICS, V17, P1244, DOI 10.1093/bioinformatics/17.12.1244
   Lin YH, 2001, MOL BIOL EVOL, V18, P684, DOI 10.1093/oxfordjournals.molbev.a003850
   Messenger SL, 2002, CLIN INFECT DIS, V35, P738, DOI 10.1086/342387
   MILDE KF, 1993, BIOCHIM BIOPHYS ACTA, V1172, P315, DOI 10.1016/0167-4781(93)90220-8
   Nikaido M, 2001, J MOL EVOL, V53, P508, DOI 10.1007/s002390010241
   NORIMINE J, 1992, IMMUNOLOGY, V75, P74
   Pilipski JD, 2004, J WILDLIFE DIS, V40, P335, DOI 10.7589/0090-3558-40.2.335
   SULKIN SE, 1966, SCIENCE, V152, P223, DOI 10.1126/science.152.3719.223
   SWAIN SL, 1984, J IMMUNOL, V132, P1118
   THOMPSON PG, 1984, MISS AFES RES REP, V8, P1
   VEILLETTE A, 1988, MOL CELL BIOL, V8, P4353, DOI 10.1128/MCB.8.10.4353
   VEILLETTE A, 1988, CELL, V55, P301, DOI 10.1016/0092-8674(88)90053-0
NR 18

TC 9
Z9 10
U1 0
U2 7
PU ELSEVIER
PI AMSTERDAM
PA RADARWEG 29, 1043 NX AMSTERDAM, NETHERLANDS
SN 0165-2427
EI 1873-2534
J9 VET IMMUNOL IMMUNOP
JI Vet. Immunol. Immunopathol.
PD JUN 15
PY 2006
VL 111
IS 3-4
BP 309
EP 313
DI 10.1016/j.vetimm.2005.12.002
PG 5
WC Immunology; Veterinary Sciences
WE Science Citation Index Expanded (SCI-EXPANDED)
SC Immunology; Veterinary Sciences
GA 044IW
UT WOS:000237666700017
PM 16480776
DA 2024-10-21
ER

PT J
AU Moratelli, R
   Calisher, CH
AF Moratelli, Ricardo
   Calisher, Charles H.
TI Bats and zoonotic viruses: can we confidently link bats with emerging
   deadly viruses?
SO MEMORIAS DO INSTITUTO OSWALDO CRUZ
LA English
DT Article
DE bat-borne viruses; biosafety; Chiroptera; bat immunology; emerging
   infectious diseases; zoonosis
ID RESPIRATORY SYNDROME CORONAVIRUS; WHITE-NOSE SYNDROME; NIPAH VIRUS;
   RESERVOIR HOSTS; RABIES VIRUS; FRUIT BATS; IMMUNE-RESPONSES; FLYING
   FOXES; EBOLA-VIRUS; EVOLUTION
AB An increasingly asked question is 'can we confidently link bats with emerging viruses?'. No, or not yet, is the qualified answer based on the evidence available. Although more than 200 viruses-some of them deadly zoonotic viruses -have been isolated from or otherwise detected in bats, the supposed connections between bats, bat viruses and human diseases have been raised more on speculation than on evidence supporting their direct or indirect roles in the

epidemiology of diseases (except for rabies). However, we are convinced that the evidence points in that direction and that at some point it will be proved that bats are competent hosts for at least a few zoonotic viruses. In this review, we cover aspects of bat biology, ecology and evolution that might be relevant in medical investigations and we provide a historical synthesis of some disease outbreaks causally linked to bats. We provide evolutionary-based hypotheses to tentatively explain the viral transmission route through mammalian intermediate hosts and to explain the geographic concentration of most outbreaks, but both are no more than speculations that still require formal assessment.
C1 [Moratelli, Ricardo] Fiocruz Mata Atlantica, Rio De Janeiro, RJ, Brazil.
[Calisher, Charles H.] Colorado State Univ, Coll Vet Med & Biomed Sci, Dept Microbiol Immunol & Pathol, Arthropod Borne & Infect Dis Lab, Ft Collins, CO 80523 USA.
C3 Fundacao Oswaldo Cruz; Colorado State University
RP Moratelli, R (corresponding author), Natl Museum Nat Hist, Smithsonian Inst, Div Mammals, Washington, DC 20560 USA.
EM rmoratelli@fiocruz.br
RI Moratelli, Ricardo/A-5735-2013
OI Moratelli, Ricardo/0000-0003-0942-6633
FU PAPES; FIOCRUZ; CNPq [407623/2012-4]; CNPq/CsF [202612/2012]; FAPERJ
[E-26/111.288/2014]; Smithsonian Institution (USA)
FX PAPES, FIOCRUZ, CNPq (407623/2012-4), CNPq/CsF (202612/2012), FAPERJ
(E-26/111.288/2014), Smithsonian Institution (USA)
CR Adams R.A., 2003, BATS ROCKY MOUNTAIN
Adney DR, 2014, EMERG INFECT DIS, V20, P1999, DOI 10.3201/eid2012.141280
Agnelli P, 2011, HYSTRIX, V22, P215, DOI 10.4404/Hystrix-22.1-4540
Altringham J., 1996, Bats: Biology and Behaviour
[Anonymous], 2011, BATS EVOLUTION CONSE
AUSTAD SN, 1991, J GERONTOL, V46, pB47, DOI 10.1093/geronj/46.2.B47
Baer GeorgeM., 1991, The Natural History of Rabies
Baize S, 2014, NEW ENGL J MED, V371, P1418, DOI 10.1056/NEJMoa1404505
Baker KS, 2014, J ANIM ECOL, V83, P415, DOI 10.1111/1365-2656.12153
Baker ML, 2013, ZOONOSES PUBLIC HLTH, V60, P104, DOI 10.1111/j.1863-2378.2012.01528.x
Barr JA, 2012, J GEN VIROL, V93, P2590, DOI 10.1099/vir.0.045385-0
Bermingham A, 2012, EUROSURVEILLANCE, V17, P6
Blackwood JC, 2013, P NATL ACAD SCI USA, V110, P20837, DOI 10.1073/pnas.1308817110
Blehert DS, 2009, SCIENCE, V323, P227, DOI 10.1126/science.1163874
Boonman A, 2014, CURR BIOL, V24, DOI 10.1016/j.cub.2014.10.077
Bouma HR, 2010, J LEUKOCYTE BIOL, V88, P619, DOI 10.1189/jlb.0310174

Bourliere F., 1958, J Gerontol, V13, P16, DOI [DOI 10.1093/GERONJ/13.SUPPL_1.16, 10.1093/geronj/13.Suppl_1.16]
Brook CE, 2015, TRENDS MICROBIOL, V23, P172, DOI 10.1016/j.tim.2014.12.004
Butler JE, 2011, DEV COMP IMMUNOL, V35, P273, DOI 10.1016/j.dci.2010.08.011
Calisher CH, 2015, BATS VIRUSES PATHOGE
Calisher CH, 2006, CLIN MICROBIOL REV, V19, P531, DOI 10.1128/CMR.00017-06
Calisher CH, 2012, TRAVEL MED INFECT DI, V10, P69, DOI 10.1016/j.tmaid.2012.01.003
Carini A., 1911, Ann. Institut Pasteur, V25, P843
Ceballos G, 2006, P NATL ACAD SCI USA, V103, P19374, DOI 10.1073/pnas.0609334103
Chadha MS, 2006, EMERG INFECT DIS, V12, P235, DOI 10.3201/eid1202.051247
Chua KB, 2000, SCIENCE, V288, P1432, DOI 10.1126/science.288.5470.1432
Chua KB, 2002, MICROBES INFECT, V4, P145, DOI 10.1016/S1286-4579(01)01522-2
Daszak P, 2013, P NATL ACAD SCI USA, V110, P3681, DOI 10.1073/pnas.1201243109
Davis A, 2012, VECTOR-BORNE ZOONOT, V12, P55, DOI 10.1089/vbz.2011.0674
Dobson AP, 2006, SCIENCE, V311, P1099
DOBSON G.E., 1875, Annals and Magazine of Natural History, V16, P345, DOI DOI 10.1080/00222937508681865
Drexler JF, 2011, EMERG INFECT DIS, V17, P449, DOI 10.3201/eid1703.100526
Drexler JF, 2009, PLOS ONE, V4, DOI 10.1371/journal.pone.0006367
Epstein JH, 2013, PLOS ONE, V8, DOI 10.1371/journal.pone.0067584
Fenton M.B., 2015, Bats: a world of science and mystery
Fenton MB, 2006, SCIENCE, V311, P1098
Fenton MB., 1983, Just Bats
Fraser GC, 1996, EMERG INFECT DIS, V2, P327, DOI 10.3201/eid0204.960408
Gardner AL, 2008, MAMMALS S AM, V1, P402
Gire SK, 2014, SCIENCE, V345, P1369, DOI 10.1126/science.1259657
GOLDWASSER RA, 1958, P SOC EXP BIOL MED, V98, P219, DOI 10.3181/00379727-98-23996
GOULD E, 1988, J MAMMAL, V69, P378, DOI 10.2307/1381392
Hall L.S., 2000, Flying foxes: fruit and blossom bats of Australia
Halpin K, 2000, J GEN VIROL, V81, P1927, DOI 10.1099/0022-1317-81-8-1927
Hayman DTS, 2008, PLOS ONE, V3, DOI 10.1371/journal.pone.0002739
Ithete NL, 2013, EMERG INFECT DIS, V19, P1697, DOI 10.3201/eid1910.130946
JAHRLING PB, 1990, LANCET, V335, P502, DOI 10.1016/0140-6736(90)90737-P
JOHNSON KM, 1977, LANCET, V1, P569
Jones Gareth, 2009, Endangered Species Research, V8, P93, DOI 10.3354/esr00182
Koopman K.F., 1984, Bat Research News, V25, P25

Koopman KF, 1994, HDB ZOOLOGIE 60, V8
Ksiazek TG, 2003, NEW ENGL J MED, V348, P1953, DOI 10.1056/NEJMoa030781
KUNZ T H., 1994, Walker's Bats of the World, P1
Kunz TH, 2011, ANN NY ACAD SCI, V1223, P1, DOI 10.1111/j.1749-6632.2011.06004.x
Kupferschmidt K, 2013, SCIENCE, V341, P948, DOI 10.1126/science.341.6149.948
Lau SKP, 2005, P NATL ACAD SCI USA, V102, P14040, DOI 10.1073/pnas.0506735102
LEGUENNO B, 1995, LANCET, V345, P1271, DOI 10.1016/S0140-6736(95)90925-7
Leroy EM, 2005, NATURE, V438, P575, DOI 10.1038/438575a
Li WD, 2005, SCIENCE, V310, P676, DOI 10.1126/science.1118391
Lima EQ, 1934, REV DEP NAC PROD ANI, V1, P165
Lobova TA., 2009, Seed Dispersal by Bats in the Neotropics
Luckett W.P., 1980, Proceedings International Bat Research Conference, V5, P245
Luis AD, 2013, P ROY SOC B-BIOL SCI, V280, DOI 10.1098/rspb.2012.2753
Mandl JN, 2015, CELL, V160, P20, DOI 10.1016/j.cell.2014.12.003
Marsh GA, 2012, PLOS PATHOG, V8, DOI 10.1371/journal.ppat.1002836
MARSHALL LG, 1982, SCIENCE, V215, P1351, DOI 10.1126/science.215.4538.1351
Melaun C, 2014, PARASITOL RES MG, V5, P25, DOI 10.1007/978-3-642-39333-4_3
Memish ZA, 2013, EMERG INFECT DIS, V19, P1819, DOI 10.3201/eid1911.131172
Meteyer CU, 2012, VIRULENCE, V3, P583, DOI 10.4161/viru.22330
Mickleburgh SP., 1992, Old World fruit bats: an action plan for their conservation
Mills J.N., 1995, Methods for Trapping and Sampling Small Mammals for Virologic Testing
Mühldorfer K, 2011, PLOS ONE, V6, DOI 10.1371/journal.pone.0029773
Muscarella R, 2007, BIOL REV, V82, P573, DOI 10.1111/j.1469-185X.2007.00026.x
Myers N, 2000, NATURE, V403, P853, DOI 10.1038/35002501
Negri A., 1903, Zentralbl. Bakteriol. Parasitenkd. Infektionskr. Hyg, V43, P507
Nowak RM, 1994, WALKERS BATS WORLD
O'Leary MA, 2013, SCIENCE, V339, P662, DOI 10.1126/science.1229237
O'Shea TJ, 2014, EMERG INFECT DIS, V20, P741, DOI 10.3201/eid2005.130539
Olival KJ, 2014, VIRUSES-BASEL, V6, P1759, DOI 10.3390/v6041759
Osborne C, 2011, PLOS ONE, V6, DOI 10.1371/journal.pone.0019156
Papenfuss AT, 2012, BMC GENOMICS, V13, DOI 10.1186/1471-2164-13-261
Parrish CR, 2008, MICROBIOL MOL BIOL R, V72, P457, DOI 10.1128/MMBR.00004-08
Pawan J. L., 1936, Annals of Tropical Medicine and Parasitology, V30, P101
PAWAN JL, 1948, ANN TROP MED PARASIT, V42, P173, DOI

10.1080/00034983.1948.11685359
Pettigrew JD, 1995, SYM ZOOL S, P3
Plowright RK, 2015, P ROY SOC B-BIOL SCI, V282, DOI 10.1098/rspb.2014.2124
Podlutsky AJ, 2005, J GERONTOL A-BIOL, V60, P1366, DOI 10.1093/gerona/60.11.1366
Poon LLM, 2005, J VIROL, V79, P2001, DOI 10.1128/JVI.79.4.2001-2009.2005
Raj VS, 2014, EMERG INFECT DIS, V20, P1339, DOI 10.3201/eid2008.140663
Reynes JM, 2005, EMERG INFECT DIS, V11, P1042, DOI 10.3201/eid1107.041350
Rota PA, 2003, SCIENCE, V300, P1394, DOI 10.1126/science.1085952
Ryan PA, 1997, AM J TROP MED HYG, V57, P476, DOI 10.4269/ajtmh.1997.57.476
RYDELL J, 1995, BIOL J LINN SOC, V54, P183, DOI 10.1006/bijl.1995.0011
Saéz AM, 2015, EMBO MOL MED, V7, P17, DOI 10.15252/emmm.201404792
SAZIMA I, 1989, PLANT SYST EVOL, V168, P167, DOI 10.1007/BF00936097
Sazima M, 2003, ANN BOT-LONDON, V92, P725, DOI 10.1093/aob/mcg190
Seim I, 2013, NAT COMMUN, V4, DOI 10.1038/ncomms3212
SELVEY LA, 1995, MED J AUSTRALIA, V162, P642, DOI 10.5694/j.1326-5377.1995.tb126050.x
SEYMOUR C, 1978, AM J TROP MED HYG, V27, P297, DOI 10.4269/ajtmh.1978.27.297
SEYMOUR C, 1978, AM J TROP MED HYG, V27, P290, DOI 10.4269/ajtmh.1978.27.290
Shaw TI, 2012, PLOS ONE, V7, DOI 10.1371/journal.pone.0048472
Simmons NB, 2008, NATURE, V451, P818, DOI 10.1038/nature06549
Simmons Nancy B., 1998, Bulletin of the American Museum of Natural History, V235, P1
Simmons Nancy B., 1994, American Museum Novitates, V3103, P1
Simmons Nancy B., 1993, P1
Simmons Nancy B., 1998, P3
Simmons Nancy B., 1998, Bulletin of the American Museum of Natural History, V237, P1
Simmons NB, 1995, SYM ZOOL S, P27
SIMMONS NB, 1991, SYST ZOOL, V40, P239, DOI 10.2307/2992261
Simmons NB, 2005, MAMMAL SPECIES WORLD, V1, P312, DOI [DOI 10.1111/J.1365-2656.2010.01742.X, 10.1093/acprof]
Simon R, 2011, SCIENCE, V333, P631, DOI 10.1126/science.1204210
Siu KL, 2014, J VIROL, V88, P4866, DOI 10.1128/JVI.03649-13
Springer MS, 2001, P NATL ACAD SCI USA, V98, P6241, DOI 10.1073/pnas.111551998
STEECE RS, 1989, J WILDLIFE DIS, V25, P329, DOI 10.7589/0090-3558-25.3.329
SULKIN SE, 1966, AM J TROP MED HYG, V15, P418, DOI 10.4269/ajtmh.1966.15.418
Swanepoel R, 1996, EMERG INFECT DIS, V2, P321, DOI 10.3201/eid0204.960407
Swanepoel R, 2007, EMERG INFECT DIS, V13, P1847, DOI 10.3201/eid1312.071115
Teeling EC, 2012, CAM STUD MORPHOL MOL, P1

Towner JS, 2009, PLOS PATHOG, V5, DOI 10.1371/
journal.ppat.1000536
Towner JS, 2008, PLOS PATHOG, V4, DOI 10.1371/
journal.ppat.1000212
Van den Bussche RA, 2004, J MAMMAL, V85, P321, DOI
10.1644/1545-1542(2004)085<0321:PRARCF>2.0.CO;2
Voigt CC, 2011, BIOTROPICA, V43, P108, DOI 10.1111/
j.1744-7429.2010.00632.x
Voss Robert S., 2001, Bulletin of the American Museum of Natural
History, V263, P1
Wacharapluesadee S, 2005, EMERG INFECT DIS, V11, P1949, DOI
10.3201/eid1112.050613
Wibbelt G, 2010, BIOL LETTERS, V6, P438, DOI 10.1098/
rsbl.2010.0267
WIBLE J R, 1988, American Museum Novitates, P1
Wilkinson GS, 2002, AGING CELL, V1, P124, DOI 10.1046/
j.1474-9728.2002.00020.x
WILSON DE, 1973, SYST ZOOL, V22, P14, DOI 10.2307/2412374
Wilson DE, 2005, MAMMAL SPECIES WORLD
Wilson DonE., 1997, Bats in Question: The Smithsonian Answer Book
WITTE EJ, 1954, AM J PUBLIC HEALTH, V44, P186
Wynne JW, 2013, PLOS PATHOG, V9, DOI 10.1371/journal.ppat.1003651
Zhang GJ, 2013, SCIENCE, V339, P456, DOI 10.1126/science.1230835
Zhang HJ, 2012, J VIROL, V86, P8014, DOI 10.1128/JVI.00723-12
Zhou P, 2014, PLOS ONE, V9, DOI 10.1371/journal.pone.0103875
NR 135
TC 136
Z9 158
U1 1
U2 133
PU FUNDACO OSWALDO CRUZ
PI RIO DE JANEIRO, RJ
PA AV BRASIL 4365, 21045-900 RIO DE JANEIRO, RJ, BRAZIL
SN 0074-0276
EI 1678-8060
J9 MEM I OSWALDO CRUZ
JI Mem. Inst. Oswaldo Cruz
PD FEB
PY 2015
VL 110
IS 1
BP 1
EP 22
DI 10.1590/0074-02760150048
PG 22
WC Parasitology; Tropical Medicine
WE Science Citation Index Expanded (SCI-EXPANDED)
SC Parasitology; Tropical Medicine
GA CC9OB
UT WOS:000350699000001
PM 25742261
OA Green Published, gold, Green Submitted
DA 2024-10-21
ER

PT J
AU Turmelle, AS
Ellison, JA
Mendonça, MT
McCracken, GF
AF Turmelle, Amy S.
Ellison, James A.
Mendonca, Mary T.
McCracken, Gary F.
TI Histological assessment of cellular immune response to the phytohemagglutinin skin test in Brazilian free-tailed bats (Tadarida brasiliensis)
SO JOURNAL OF COMPARATIVE PHYSIOLOGY B-BIOCHEMICAL SYSTEMIC AND ENVIRONMENTAL PHYSIOLOGY
LA English
DT Article
DE Bat; Cellular immune response; Phytohemagglutinin
ID MEDIATED-IMMUNITY; LIFE-HISTORY; RABIES VIRUS; FRUIT BATS; IMMUNOCOMPETENCE; CORONAVIRUSES; DYNAMICS; ANTIBODIES; CHICKENS; ECOLOGY
AB Bats are known reservoirs for numerous emerging infectious diseases, occupy unique ecological niches, and occur globally except for Antarctica. Given their impact on human and agricultural health, it is critical to understand the mechanisms underlying immunocompetence in this reservoir host. To date, few studies have examined immune function in the Order Chiroptera, particularly among natural colonies of bats. The phytohemagglutinin (PHA) skin test has been widely used to measure delayed-type cellular immune response in a wide variety of vertebrates, and has been routinely employed in immunoecological studies. Although this test is frequently described as a measure of T cell proliferation, recent studies indicate it may represent a combination of immune responses. In mammals, the immune response is differentially, temporally and spatially regulated, therefore, we characterized the infiltrating leukocyte response to the PHA skin test in bats by examining a time-series of histological sections from PHA and saline injection areas in 41 Brazilian free-tailed bats (Tadarida brasiliensis). Results suggest that bats exhibit diverse leukocyte traffic within 6 h, and up to 24 h following subcutaneous PHA injection. There was a significant presence of lymphocytes and neutrophils, as well as eosinophils, basophils, and macrophages observed in the PHA-injected tissues, compared with saline-injected control tissues. We observed a highly significant negative correlation between the number of lymphocytes and neutrophils in PHA-injected tissue, with peak lymphocyte response at 12 h, and peak neutrophil response at 24 h post-injection. These results indicate substantial variation in the immune response of individuals, and may aid our understanding of disease emergence in natural populations of bats.
C1 [Ellison, James A.; Mendonca, Mary T.] Auburn Univ, Dept Biol Sci, Auburn, AL 36849 USA.
[Turmelle, Amy S.; McCracken, Gary F.] Univ Tennessee, Dept Ecol & Evolutionary Biol, Knoxville, TN 37996 USA.

C3 Auburn University System; Auburn University; University of Tennessee
System; University of Tennessee Knoxville
RP Turmelle, AS (corresponding author), Ctr Dis Control & Prevent, 1600 Clifton Rd NE,Mailstop G-33, Atlanta, GA 30333 USA.
EM ATurmelle@cdc.gov
RI Ellison, James/C-1402-2013
OI McCracken, Gary/0000-0002-2493-8103; Ellison, James/0000-0003-4492-4857;
Gilbert, Amy T./0000-0002-8256-0081
FU National Science Foundation-National Institutes of Health Ecology of
Infectious Disease [0430418]; Environmental Protection Agency
FX This research was supported in part by a National Science
Foundation-National Institutes of Health Ecology of Infectious Disease
grant (#0430418 to G.F.M.), and also an Environmental Protection Agency
Science-To-Achieve-Results (STAR) Fellowship to AST. The authors wish to
thank Bill Cofer for granting access to his property, and S. Duncan for
invaluable assistance in the field. The authors also express thanks to
B. French (Bat World), B. Landreth and L. Rascoe (Auburn University
College of Veterinary Medicine) for technical support. We also thank two
anonymous reviewers for providing constructive comments that improved
this manuscript.
CR Allen LC, 2010, J ZOOL, V280, P8, DOI 10.1111/j.1469-7998.2009.00636.x
Allen LC, 2009, J COMP PHYSIOL B, V179, P315, DOI 10.1007/s00360-008-0315-3
Altizer S, 2006, ECOL LETT, V9, P467, DOI 10.1111/j.1461-0248.2005.00879.x
[Anonymous], 2007, Rabies
[Anonymous], ECOLOGICAL BEHAV MET
Berger S, 2005, HORM BEHAV, V47, P419, DOI 10.1016/j.yhbeh.2004.11.011
BONFORTE RJ, 1972, J PEDIATR-US, V81, P775, DOI 10.1016/S0022-3476(72)80101-X
Carrington CVF, 2008, EMERG INFECT DIS, V14, P1890, DOI 10.3201/eid1412.080642
Chappuis G, 1998, VACCINE, V16, P1468, DOI 10.1016/S0264-410X(98)00110-8
Christe P, 2000, ECOL LETT, V3, P207
Chua KB, 2001, VIROLOGY, V283, P215, DOI 10.1006/viro.2000.0882
CONSTANTINE DG, 1968, PUBLIC HEALTH REP, V83, P303, DOI 10.2307/4593275
CONSTANTINE DG, 1986, J WILDLIFE DIS, V22, P249, DOI 10.7589/0090-3558-22.2.249
de Bellocq JG, 2006, COMP BIOCHEM PHYS A, V145, P554, DOI

10.1016/j.cbpa.2006.08.045
de Bellocq JG, 2007, PARASITOL RES, V100, P321, DOI 10.1007/s00436-006-0273-1
Dimitrov Dobromir T., 2009, Journal of Biological Dynamics, V3, P164, DOI 10.1080/17513750802702443
Dominguez SR, 2007, EMERG INFECT DIS, V13, P1295, DOI 10.3201/eid1309.070491
Drazen DL, 2003, CAN J ZOOL, V81, P1406, DOI [10.1139/z03-120, 10.1139/Z03-120]
Gannon WL, 2007, J MAMMAL, V88, P809, DOI 10.1644/06-MAMM-F-185R1.1
Gervasi SS, 2008, FUNCT ECOL, V22, P100, DOI 10.1111/j.1365-2435.2007.01340.x
GILLIS S, 1981, J CLIN INVEST, V67, P937, DOI 10.1172/JCI110143
Gloza-Rausch F, 2008, EMERG INFECT DIS, V14, P626, DOI 10.3201/eid1404.071439
GOTO N, 1978, POULTRY SCI, V57, P246, DOI 10.3382/ps.0570246
Halpin K., 1996, Communicable Diseases Intelligence, V20, P476
Halpin K, 2007, CLIN INFECT DIS, V44, P711, DOI 10.1086/511078
Johara MY, 2001, EMERG INFECT DIS, V7, P439
Kahn PF, 2007, COPEIA, P614, DOI 10.1643/0045-8511(2007)2007[614:HBSATC]2.0.CO;2
Keeley ATH, 2004, J MAMMAL, V85, P113, DOI 10.1644/BME-004
Kennedy MW, 2006, TRENDS ECOL EVOL, V21, P653, DOI 10.1016/j.tree.2006.09.017
Klasing Kirk C., 2004, Acta Zoologica Sinica, V50, P961
KUNZ TH, 1995, J MAMMAL, V76, P769, DOI 10.2307/1382746
LAWLOR GJ, 1973, J ALLERGY CLIN IMMUN, V52, P31, DOI 10.1016/0091-6749(73)90118-8
Lee KA, 2006, INTEGR COMP BIOL, V46, P1000, DOI 10.1093/icb/icl049
Lehmer EM, 2007, PHYSIOL BIOCHEM ZOOL, V80, P514, DOI 10.1086/520128
Leroy EM, 2005, NATURE, V438, P575, DOI 10.1038/438575a
LEROY EM, 2009, VECTOR BORNE ZOONOTI
Li WD, 2005, SCIENCE, V310, P676, DOI 10.1126/science.1118391
LOCHMILLER RL, 1993, AUK, V110, P503, DOI 10.2307/4088414
Martin LB, 2006, FUNCT ECOL, V20, P290, DOI 10.1111/j.1365-2435.2006.01094.x
MCCORKLE F, 1980, POULTRY SCI, V59, P616, DOI 10.3382/ps.0590616
Müller MA, 2007, EMERG INFECT DIS, V13, P1367, DOI 10.3201/eid1309.070342
Müller TF, 2001, AM J VET RES, V62, P1154, DOI 10.2460/ajvr.2001.62.1154
NASPITZ CK, 1968, PROG ALLERGY, V12, P1, DOI 10.1159/000287256
Navara KJ, 2006, PHYSIOL BIOCHEM ZOOL, V79, P550, DOI 10.1086/501054
Nelson R.J., 2002, SEASONAL PATTERNS ST
Nelson RJ, 1996, Q REV BIOL, V71, P511, DOI 10.1086/419555
Reichard JD, 2009, J MAMMAL, V90, P1478, DOI 10.1644/08-MAMM-A-266R1.1
SCHUURS AHWM, 1990, J STEROID BIOCHEM, V35, P157, DOI 10.1016/0022-4731(90)90270-3
Smits JE, 1999, FUNCT ECOL, V13, P567, DOI 10.1046/

j.1365-2435.1999.00338.x
   STADECKER MJ, 1977, J IMMUNOL, V118, P1564
   Swanepoel R, 2007, EMERG INFECT DIS, V13, P1847, DOI 10.3201/eid1312.071115
   Tella JL, 2002, P ROY SOC B-BIOL SCI, V269, P1059, DOI 10.1098/rspb.2001.1951
   Tella JL, 2008, PLOS ONE, V3, DOI 10.1371/journal.pone.0003295
   Towner JS, 2009, PLOS PATHOG, V5, DOI 10.1371/journal.ppat.1000536
   Tschirren B, 2003, J ANIM ECOL, V72, P839, DOI 10.1046/j.1365-2656.2003.00755.x
   Turmelle AS, 2010, VECTOR-BORNE ZOONOT, V10, P165, DOI 10.1089/vbz.2008.0163
   Vos A, 2003, VET REC, V153, P16, DOI 10.1136/vr.153.1.16
   XIANG ZQ, 1992, VIRUS RES, V24, P297, DOI 10.1016/0168-1702(92)90125-S
   Zuk M, 1996, ECOLOGY, V77, P1037, DOI 10.2307/2265574
NR 59
TC 28
Z9 37
U1 1
U2 27
PU SPRINGER HEIDELBERG
PI HEIDELBERG
PA TIERGARTENSTRASSE 17, D-69121 HEIDELBERG, GERMANY
SN 0174-1578
J9 J COMP PHYSIOL B
JI J. Comp. Physiol. B-Biochem. Syst. Environ. Physiol.
PD NOV
PY 2010
VL 180
IS 8
BP 1155
EP 1164
DI 10.1007/s00360-010-0486-6
PG 10
WC Physiology; Zoology
WE Science Citation Index Expanded (SCI-EXPANDED)
SC Physiology; Zoology
GA 666SO
UT WOS:000283139900004
PM 20549214
OA Green Published, Bronze
DA 2024-10-21
ER

PT J
AU Cooper, LN
   Ansari, MY
   Capshaw, G
   Galazyuk, A
   Lauer, AM
   Moss, CF
   Sears, KE

Stewart, M
Teeling, EC
Wilkinson, GS
Wilson, RC
Zwaka, TP
Orman, R
AF Cooper, Lisa Noelle
Ansari, Mohammad Y.
Capshaw, Grace
Galazyuk, Alex
Lauer, Amanda M.
Moss, Cynthia F.
Sears, Karen E.
Stewart, Mark
Teeling, Emma C.
Wilkinson, Gerald S.
Wilson, Rachel C.
Zwaka, Thomas P.
Orman, Rena
TI Bats as instructive animal models for studying longevity and aging
SO ANNALS OF THE NEW YORK ACADEMY OF SCIENCES
LA English
DT Article; Early Access
DE Chiroptera; hearing; immunity; lifespan; neural system; reproductive
physiology; skeletal system
ID CAUDATE-NUCLEUS; FRUIT BATS; BROWN BAT; PARKINSONS-DISEASE; CALCIUM
HYPOTHESIS; ALZHEIMERS-DISEASE; RHESUS-MONKEY; AGE; PROSUBICULUM;
DYSFUNCTION
AB Bats (order Chiroptera) are emerging as instructive animal models for aging studies. Unlike some common laboratory species, they meet a central criterion for aging studies: they live for a long time in the wild or in captivity, for 20, 30, and even >40 years. Healthy aging (i.e., healthspan) in bats has drawn attention to their potential to improve the lives of aging humans due to bat imperviousness to viral infections, apparent low rate of tumorigenesis, and unique ability to repair DNA. At the same time, bat longevity also permits the accumulation of age-associated systemic pathologies that can be examined in detail and manipulated, especially in captive animals. Research has uncovered additional and critical advantages of bats. In multiple ways, bats are better analogs to humans than are rodents. In this review, we highlight eight diverse areas of bat research with relevance to aging: genome sequencing, telomeres, and DNA repair; immunity and inflammation; hearing; menstruation and menopause; skeletal system and fragility; neurobiology and neurodegeneration; stem cells; and senescence and mortality. These examples demonstrate the broad relevance of the bat as an animal model and point to directions that are particularly important for human aging studies.
C1 [Cooper, Lisa Noelle; Ansari, Mohammad Y.; Galazyuk, Alex] Northeast Ohio Med Univ, Dept Anat & Neurobiol, 4209 State Route 44, Rootstown, OH 44272 USA.

[Capshaw, Grace; Moss, Cynthia F.] Johns Hopkins Univ, Dept Psychol & Brain Sci, Baltimore, MD USA.
[Lauer, Amanda M.] Johns Hopkins Univ, Sch Med, Dept Otolaryngol HNS, Baltimore, MD USA.
[Sears, Karen E.] Univ Calif Los Angeles, Dept Ecol & Evolutionary Biol, Dept Mol Cellular & Dev Biol, Los Angeles, CA USA.
[Stewart, Mark; Orman, Rena] SUNY Downstate Hlth Sci Univ, Dept Physiol & Pharmacol, 450 Clarkson Ave MSC31, Brooklyn, NY 11203 USA.
[Teeling, Emma C.] Univ Coll Dublin, Sci Ctr East, Sch Biol & Environm Sci, Dublin, Ireland.
[Wilkinson, Gerald S.] Univ Maryland Coll Pk, Dept Biol, College Pk, MD USA.
[Wilson, Rachel C.] Whitman Coll, Dept Biol, Walla Walla, WA USA.
[Zwaka, Thomas P.] Icahn Sch Med Mt Sinai, Black Family Stem Cell Inst, Huffington Ctr Cell Based Res Parkinsons Dis, Dept Cell Dev & Regenerat Biol, New York, NY USA.
C3 University System of Ohio; Northeast Ohio Medical University (NEOMED);
Johns Hopkins University; Johns Hopkins University; University of California System; University of California Los Angeles; State University of New York (SUNY) System; SUNY Downstate Health Sciences
University; University College Dublin; University System of Maryland;
University of Maryland College Park; Whitman College; Icahn School of
Medicine at Mount Sinai
RP Cooper, LN (corresponding author), Northeast Ohio Med Univ, Dept Anat & Neurobiol, 4209 State Route 44, Rootstown, OH 44272 USA.; Orman, R (corresponding author), SUNY Downstate Hlth Sci Univ, Dept Physiol & Pharmacol, 450 Clarkson Ave MSC31, Brooklyn, NY 11203 USA.
EM lcooper@neomed.edu; rena.orman@downstate.edu
FU National Science Foundation [1256423, 1537745, 1707519, 1734744,
1854469, 2017803, 2213824]; National Institutes of Health [AG075644,
AG078474, AG078784, AI135972, AI169516, AI171399, DC000023, GM129329,
HD100518, NS118462, NS121413]; Open Philanthropy Project Fund
[2021-244135(5384)]; David M. Rubenstein Fund for Hearing Research; SUNY
Downstate SVPR 2023 Seed Grant; Science Foundation Ireland Future
Frontiers Awards [19/FFP/6790]; Center for Research on Influenza
Pathogenesis and Response (CRIPT); Office of Naval Research Global
[N00014-17-1-2736, N00014-22-1-2793]
FX National Science Foundation, Grant/Award Numbers: 1256423, 1537745,
1707519, 1734744, 1854469, 2017803, 2213824; National Institutes of
Health, Grant/Award Numbers: AG075644, AG078474, AG078784, AI135972,
AI169516, AI171399, DC000023, GM129329, HD100518, NS118462, NS121413;

Open Philanthropy Project Fund, Grant/Award Number: 2021-244135(5384);
David M. Rubenstein Fund for Hearing Research; SUNY Downstate SVPR 2023
Seed Grant; Science Foundation Ireland Future <EM><STRONG>
</STRONG></EM>Frontiers Awards, Grant/Award Number: 19/FFP/6790; Center
for Research on Influenza Pathogenesis and Response (CRIPT); Office of
Naval Research Global, Grant/Award Numbers: N00014-17-1-2736,
N00014-22-1-2793
CR Ahn M, 2023, CELL, V186, P2144, DOI 10.1016/j.cell.2023.03.036
Ahn M, 2019, NAT MICROBIOL, V4, P789, DOI 10.1038/s41564-019-0371-3
Lagunas-Rangel FA, 2020, BIOGERONTOLOGY, V21, P1, DOI 10.1007/s10522-019-09840-3
Almeida OP, 2003, DEMENT GERIATR COGN, V16, P57, DOI 10.1159/000070676
Ansari MY, 2021, OSTEOARTHR CARTILAGE, V29, P100, DOI 10.1016/j.joca.2020.08.014
Ansari MY, 2020, J CELL SCI, V133, DOI 10.1242/jcs.247353
Atlan G, 2018, CURR BIOL, V28, P2752, DOI 10.1016/j.cub.2018.06.068
Austad SN, 2010, J COMP PATHOL, V141, pS10, DOI 10.1016/j.jcpa.2009.10.024
AUSTAD SN, 1991, J GERONTOL, V46, pB47, DOI 10.1093/geronj/46.2.B47
Baglietto-Vargas D, 2010, J ALZHEIMERS DIS, V21, P119, DOI 10.3233/JAD-2010-100066
Bai LL, 2024, FEBS OPEN BIO, V14, P598, DOI 10.1002/2211-5463.13781
Baid K, 2024, TRENDS IMMUNOL, V45, P188, DOI 10.1016/j.it.2024.01.007
Ball HC, 2016, STEM CELL RES, V17, P54, DOI 10.1016/j.scr.2016.05.009
Balsters JH, 2020, ELIFE, V9, DOI 10.7554/eLife.53680
Banerjee A, 2020, FRONT IMMUNOL, V11, DOI 10.3389/fimmu.2020.00026
Bao JX, 2010, HEARING RES, V264, P93, DOI 10.1016/j.heares.2009.10.009
Barnard S. M., 2011, Bats in captivity, volume 3: Diet and feeding Environment and housing
Barnard S. M., 2010, Bats in captivity, volume 2: Aspects of rehabilitation
Barnard S. M., 2009, Bats in captivity, volume 1: Biological and medical aspects
Beetz MJ, 2017, ENEURO, V4, DOI 10.1523/ENEURO.0314-17.2017
Benningfield MM, 2014, DEV COGN NEUROS-NETH, V7, P43, DOI 10.1016/j.dcn.2013.10.009
Berrío-Martínez J, 2019, PEERJ, V7, DOI 10.7717/peerj.7448
Biesold SE, 2011, PLOS ONE, V6, DOI 10.1371/journal.pone.0028131
Bishop CV, 2022, BIOL REPROD, DOI 10.1093/biolre/ioac183
Blatt GJ, 1998, J COMP NEUROL, V392, P92, DOI 10.1002/(SICI)1096-9861(19980302)392:1<92::AID-CNE7>3.0.CO;2-K

Bondet V, 2021, FRONT IMMUNOL, V12, DOI 10.3389/fimmu.2021.735866
Bosch JLHR, 2013, J UROLOGY, V189, pS86, DOI 10.1016/j.juro.2012.11.033
Bovee S, 2024, INT J MOL SCI, V25, DOI 10.3390/ijms25105391
Bowl MR, 2019, CSH PERSPECT MED, V9, DOI 10.1101/cshperspect.a033217
Braak H, 2020, J NEUROPATH EXP NEUR, V79, P163, DOI 10.1093/jnen/nlz123
Brenner RM, 2012, REV ENDOCR METAB DIS, V13, P309, DOI 10.1007/s11154-012-9225-5
BRION JP, 1994, ACTA NEUROPATHOL, V88, P33, DOI 10.1007/BF00294357
Brunet-Rossinni AK, 2004, BIOGERONTOLOGY, V5, P211, DOI 10.1023/B:BGEN.0000038022.65024.d8
Busche MA, 2016, PHILOS T R SOC B, V371, DOI 10.1098/rstb.2015.0429
Carvalho VS, 2021, TISSUE CELL, V71, DOI 10.1016/j.tice.2021.101507
CASPARY DM, 1995, EXP GERONTOL, V30, P349, DOI 10.1016/0531-5565(94)00052-5
Catalini L, 2020, BIOL REPROD, V102, P1160, DOI 10.1093/biolre/ioaa029
Chan LLY, 2023, EMERG MICROBES INFEC, V12, DOI 10.1080/22221751.2022.2148561
Chen M, 2014, FRONT AGING NEUROSCI, V6, DOI 10.3389/fnagi.2014.00329
Clayton BA, 2013, ZOONOSES PUBLIC HLTH, V60, P69, DOI 10.1111/j.1863-2378.2012.01501.x
COTTER JR, 1990, J HIRNFORSCH, V31, P87
Counter CM, 1996, MUTAT RES-REV GENET, V366, P45, DOI 10.1016/S0165-1110(96)90006-8
Crick FC, 2005, PHILOS T R SOC B, V360, P1271, DOI 10.1098/rstb.2005.1661
Dani H, 2016, NAT REV UROL, V13, P573, DOI 10.1038/nrurol.2016.134
Davies KTJ, 2014, GENE, V549, P228, DOI 10.1016/j.gene.2014.07.061
De La Cruz-Rivera PC, 2018, J IMMUNOL, V200, P209, DOI 10.4049/jimmunol.1701214
Déjosez M, 2023, CELL, V186, P957, DOI 10.1016/j.cell.2023.01.011
Delpietro HA, 2017, ROY SOC OPEN SCI, V4, DOI 10.1098/rsos.160959
Ding SL, 2013, J COMP NEUROL, V521, P4145, DOI 10.1002/cne.23416
Doi T, 2020, ELIFE, V9, DOI 10.7554/eLife.56694
Eckerle I, 2014, PLOS ONE, V9, DOI 10.1371/journal.pone.0084679
EilamAltstadter R., 2021, Stereotaxic brain atlas of the Egyptian fruit bat
Fan YS, 2024, J NEUROSCI, V44, DOI 10.1523/JNEUROSCI.0902-23.2023
Fine Noam D, 2017, F1000Res, V6, P1627, DOI 10.12688/f1000research.11979.1
Foley NM, 2018, SCI ADV, V4, DOI 10.1126/sciadv.aao0926
Foskolos I, 2022, J EXP BIOL, V225, DOI [10.1242/jeb.242957, 10.1242/jeb.244494]
Galazyuk AV, 1997, J COMP PHYSIOL A, V180, P301, DOI 10.1007/s003590050050

Gatome CW, 2010, BRAIN BEHAV EVOLUT, V76, P116, DOI 10.1159/000320210
Geva S, 2020, NEUROIMAGE-CLIN, V28, DOI 10.1016/j.nicl.2020.102429
Giardini MA, 2014, PROG MOL BIOL TRANSL, V125, P1, DOI 10.1016/B978-0-12-397898-1.00001-3
Giesers NK, 2020, ASN NEURO, V12, DOI 10.1177/1759091420925356
Goldberg RL, 1998, HEARING RES, V122, P71, DOI 10.1016/S0378-5955(98)00078-1
Goll Y, 2015, TRENDS NEUROSCI, V38, P486, DOI 10.1016/j.tins.2015.05.006
Gorbunova V, 2020, CELL METAB, V32, P31, DOI 10.1016/j.cmet.2020.06.013
GRIFFITHS PD, 1994, NEUROSCI LETT, V169, P68, DOI 10.1016/0304-3940(94)90358-1
Haile LM, 2021, LANCET, V397, P996, DOI 10.1016/S0140-6736(21)00516-X
Hargett SE, 2024, LAB ANIMAL, V53, P93, DOI 10.1038/s41684-024-01349-8
Harper JM, 2007, AGING CELL, V6, P1, DOI 10.1111/j.1474-9726.2006.00255.x
Havill LM, 2003, BONE, V33, P877, DOI 10.1016/S8756-3282(03)00231-X
Hieronymus TL, 2024, ANAT REC, V307, P2084, DOI 10.1002/ar.25368
Hölzer M, 2019, ISCIENCE, V19, P647, DOI 10.1016/j.isci.2019.08.016
Hua R, 2024, NAT COMMUN, V15, DOI 10.1038/s41467-024-45767-1
Huang ZX, 2020, AGING-US, V12, DOI 10.18632/aging.103725
Huang ZX, 2019, NAT ECOL EVOL, V3, P1110, DOI 10.1038/s41559-019-0913-3
Huang ZX, 2016, BMC GENOMICS, V17, DOI 10.1186/s12864-016-3227-8
Huguin M, 2018, J HERED, V109, P393, DOI 10.1093/jhered/esx111
Jafri MA, 2016, GENOME MED, V8, DOI 10.1186/s13073-016-0324-x
Jebb D, 2020, NATURE, V583, P578, DOI 10.1038/s41586-020-2486-3
Jilka RL, 2013, J GERONTOL A-BIOL, V68, P1209, DOI 10.1093/gerona/glt046
Johnstone RA, 2019, CURR BIOL, V29, pR112, DOI 10.1016/j.cub.2018.12.048
Kacprzyk J, 2017, ACTA CHIROPTEROL, V19, P219, DOI [10.3161/15081109ACC2017.19.2.001, 10.3161/15081109acc2017.19.2.001]
Kedo O., 2016, Clinical Medicine Image Library, V2, P034
Kessler MJ, 2016, AM J PRIMATOL, V78, P152, DOI 10.1002/ajp.22399
KHACHATURIAN ZS, 1994, ANN NY ACAD SCI, V747, P1
KIMURA M, 1993, ADV NEUROL, V60, P62
Kolkert H, 2020, ECOL EVOL, V10, P371, DOI 10.1002/ece3.5901
Kujawa SG, 2006, J NEUROSCI, V26, P2115, DOI 10.1523/JNEUROSCI.4985-05.2006
Kujawa SG, 2015, HEARING RES, V330, P191, DOI 10.1016/j.heares.2015.02.009
Kurtzman JT, 2016, CURR OPIN UROL, V26, P315, DOI 10.1097/MOU.0000000000000287
LANDFIELD PW, 1987, NEUROBIOL AGING, V8, P346, DOI 10.1016/0197-4580(87)90074-1
Lansdorp PM, 2022, BLOOD, V139, P813, DOI 10.1182/

blood.2021014299
Leng K, 2021, NAT NEUROSCI, V24, P276, DOI 10.1038/s41593-020-00764-7
Leroy EM, 2005, NATURE, V438, P575, DOI 10.1038/438575a
Li AQ, 2023, VIRULENCE, V14, DOI 10.1080/21505594.2022.2156185
Li WD, 2005, SCIENCE, V310, P676, DOI 10.1126/science.1118391
Li X, 2023, SIGNAL TRANSDUCT TAR, V8, DOI 10.1038/s41392-023-01502-8
LILES SL, 1969, J NEUROPHYSIOL, V32, P564, DOI 10.1152/jn.1969.32.4.564
Lin HH, 2022, MICROBIOL IMMUNOL, V66, P97, DOI 10.1111/1348-0421.12952
Liu Z, 2021, J GENET GENOMICS, V48, P984, DOI 10.1016/j.jgg.2021.06.007
Luo JH, 2015, SCI REP-UK, V5, DOI 10.1038/srep18556
Ii JMA, 2022, CELLS-BASEL, V11, DOI 10.3390/cells11040647
Mai J.K., 2004, Atlas of the Human Brain, V2nd
Makary CA, 2011, JARO-J ASSOC RES OTO, V12, P711, DOI 10.1007/s10162-011-0283-2
Marshall GA, 2004, J NEUROL NEUROSUR PS, V75, P1396, DOI 10.1136/jnnp.2003.030007
Martel-Pelletier J, 2016, NAT REV DIS PRIMERS, V2, DOI 10.1038/nrdp.2016.72
Mathur BN, 2014, FRONT SYST NEUROSCI, V8, DOI 10.3389/fnsys.2014.00048
Mikkonen M, 1999, NEUROSCIENCE, V92, P515, DOI 10.1016/S0306-4522(99)00047-0
Morello T, 2022, HIPPOCAMPUS, V32, P752, DOI 10.1002/hipo.23464
Morello T, 2022, BRAIN STRUCT FUNCT, V227, P1615, DOI 10.1007/s00429-022-02459-0
Nakabayashi J, 1998, J NEUROPATH EXP NEUR, V57, P343, DOI 10.1097/00005072-199804000-00007
Nakamura K, 2012, J NEUROSCI, V32, P15963, DOI 10.1523/JNEUROSCI.1518-12.2012
Napier JE, 2009, J ZOO WILDLIFE MED, V40, P437, DOI 10.1638/2007-0161.1
Nguyen DT, 2015, J BIOMED MATER RES A, V103, P420, DOI 10.1002/jbm.a.35139
Nikoletopoulou Vassiliki, 2012, Frontiers in Genetics, V3, P200, DOI 10.3389/fgene.2012.00200
Nixon RA, 2003, AGEING RES REV, V2, P407, DOI 10.1016/S1568-1637(03)00029-1
O'Shea TJ, 2011, J MAMMAL, V92, P433, DOI 10.1644/10-MAMM-A-162.1
Olds JE, 2015, J ZOO WILDLIFE MED, V46, P325, DOI 10.1638/2014-0069R2.1
Ong KL, 2019, CLINICOECONOMIC OUTC, V11, P245, DOI 10.2147/CEOR.S170119
Orman R, 2017, J COMP NEUROL, V525, P1459, DOI 10.1002/cne.24036
Ou HL, 2018, BLOOD, V131, P488, DOI 10.1182/blood-2017-07-746396
Palop JJ, 2006, NATURE, V443, P768, DOI 10.1038/nature05289
Paredes F, 2021, CANCER LETT, V502, P133, DOI 10.1016/j.canlet.2020.12.020
Paxinos G., 2004, The rat nervous system, Vxvii, P1309
PEARCE BR, 1984, NEUROCHEM PATHOL, V2, P221

Phillips CD, 2013, PLOS ONE, V8, DOI 10.1371/journal.pone.0057649
Pickering AM, 2015, J GERONTOL A-BIOL, V70, P791, DOI 10.1093/gerona/glu115
Podlutsky AJ, 2005, J GERONTOL A-BIOL, V60, P1366, DOI 10.1093/gerona/60.11.1366
Potter JHT, 2021, MOL BIOL EVOL, V38, P3864, DOI 10.1093/molbev/msab028
Power ML, 2023, P ROY SOC B-BIOL SCI, V290, DOI 10.1098/rspb.2023.1589
Pride H, 2015, BIOCHEM BIOPH RES CO, V457, P669, DOI 10.1016/j.bbrc.2015.01.046
Racey Paul A., 2000, P363, DOI 10.1016/B978-012195670-7/50010-2
RASWEILER JJ, 1992, J REPROD FERTIL, V95, P231
Reginster JY, 2014, CURR MED RES OPIN, V30, P1165, DOI 10.1185/03007995.2014.890927
Reinwald S, 2008, J BONE MINER RES, V23, P1353, DOI 10.1359/JBMR.080516
Richards P. J., 2014, Drug Discovery Today: Disease Models, V13, P17
Rossiello F, 2022, NAT CELL BIOL, V24, P135, DOI 10.1038/s41556-022-00842-x
Ryan NS, 2013, BRAIN, V136, P1399, DOI 10.1093/brain/awt065
Scalia F., 2013, Forebrain atlas of the shorttailed fruit bat, carollia perpicillata
SCHULTE BA, 1992, HEARING RES, V61, P35, DOI 10.1016/0378-5955(92)90034-K
Seim I, 2013, NAT COMMUN, V4, DOI 10.1038/ncomms3212
SELDEN N, 1994, NEUROSCIENCE, V60, P621, DOI 10.1016/0306-4522(94)90491-X
Sergeyenko Y, 2013, J NEUROSCI, V33, P13686, DOI 10.1523/JNEUROSCI.1783-13.2013
Shi AQ, 2020, CEREB CORTEX, V30, P1272, DOI 10.1093/cercor/bhz165
Simmons JA, 2017, J ACOUST SOC AM, V142, P2942, DOI 10.1121/1.5010159
Skrinyer A.J., 2017, Laboratory Animal Science Professional June, P24
Small SA, 2018, COLD SH Q B, V83, P193, DOI 10.1101/sqb.2018.83.036889
Smith JB, 2019, FRONT NEUROANAT, V13, DOI 10.3389/fnana.2019.00064
Smith JB, 2019, J COMP NEUROL, V527, P476, DOI 10.1002/cne.24537
Soloway MS, 2016, CURR UROL REP, V17, DOI 10.1007/s11934-016-0632-0
Stanborough RO, 2022, RADIOL CLIN N AM, V60, P593, DOI 10.1016/j.rcl.2022.03.003
Stember DS, 2007, INT J CLIN PRACT, V61, P17, DOI 10.1111/j.1742-1241.2007.01462.x
Stewart M, 2021, BIOMEDICINES, V9, DOI 10.3390/biomedicines9101454
Subbaraman N, 2021, NATURE, V595, P633, DOI 10.1038/d41586-021-01894-z
Sudah Suleiman Y, 2023, JSES Rev Rep Tech, V3, P21, DOI 10.1016/j.xrrt.2022.09.005
Sümer S, 2009, J COMP PHYSIOL A, V195, P463, DOI 10.1007/

s00359-009-0424-9
Sullivan IR, 2022, P ROY SOC B-BIOL SCI, V289, DOI 10.1098/rspb.2022.0635
Swartz SM, 2008, CELLS TISSUES ORGANS, V187, P59, DOI 10.1159/000109964
Swartz SM, 2012, CAM STUD MORPHOL MOL, P317
Swartz Sharon M., 2005, P110
Syed FA, 2010, ANN NY ACAD SCI, V1211, P95, DOI 10.1111/j.1749-6632.2010.05813.x
Tacutu R, 2013, NUCLEIC ACIDS RES, V41, pD1027, DOI 10.1093/nar/gks1155
Tarnovsky YC, 2023, LIFE SCI ALLIANCE, V6, DOI 10.26508/lsa.202201847
Teeling EC, 2018, ANNU REV ANIM BIOSCI, V6, P23, DOI 10.1146/annurev-animal-022516-022811
Thibault O, 2007, AGING CELL, V6, P307, DOI 10.1111/j.1474-9726.2007.00295.x
Tressler J, 2009, J COMP PHYSIOL A, V195, P923, DOI 10.1007/s00359-009-0468-x
Tsamis KI, 2013, CURR ALZHEIMER RES, V10, P469, DOI 10.2174/1567205011310050002
Tschapka M, 1999, Z SAUGETIERKD, V64, P239
Vernes SC, 2022, ANN NY ACAD SCI, V1517, P125, DOI 10.1111/nyas.14884
Verret L, 2012, CELL, V149, P708, DOI 10.1016/j.cell.2012.02.046
Vincze O, 2022, NATURE, V601, P263, DOI 10.1038/s41586-021-04224-5
VOGT BA, 1976, J COMP NEUROL, V169, P63, DOI 10.1002/cne.901690105
Voytenko SV, 2008, NEUROSCIENCE, V155, P923, DOI 10.1016/j.neuroscience.2008.06.031
Wang LF, 2021, NAT REV IMMUNOL, V21, P269, DOI 10.1038/s41577-021-00523-0
Ward HL, 2014, PLOS ONE, V9, DOI 10.1371/journal.pone.0087199
Weinberg MM, 2021, HEARING RES, V412, DOI 10.1016/j.heares.2021.108377
White MG, 2018, BRAIN STRUCT FUNCT, V223, P3945, DOI 10.1007/s00429-018-1731-0
Wilkinson GS, 2021, NAT COMMUN, V12, DOI 10.1038/s41467-021-21900-2
Wilkinson GS, 2019, BIOL LETTERS, V15, DOI 10.1098/rsbl.2018.0860
WILKINSON GS, 1984, NATURE, V308, P181, DOI 10.1038/308181a0
Wilkinson GS, 2002, AGING CELL, V1, P124, DOI 10.1046/j.1474-9728.2002.00020.x
Wilson D. E., 2019, Handbook of the mammals of the world, V9, P1
Wong ACY, 2015, FRONT AGING NEUROSCI, V7, DOI 10.3389/fnagi.2015.00058
Wu PZ, 2020, J NEUROSCI, V40, P6357, DOI 10.1523/JNEUROSCI.0937-20.2020
Xie DH, 1998, HEARING RES, V124, P60, DOI 10.1016/S0378-5955(98)00122-1
Zallo F, 2018, NEUROSCI LETT, V681, P19, DOI 10.1016/j.neulet.2018.05.027
Zhang D, 2023, FRONT CELL INFECT MI, V13, DOI 10.3389/

fcimb.2023.1224532
   Zhang GJ, 2013, SCIENCE, V339, P456, DOI 10.1126/science.1230835
   Zhao BZ, 2023, FRONT ENDOCRINOL, V14, DOI 10.3389/
fendo.2023.1161356
   Zhou J, 2020, NAT MED, V26, P1077, DOI 10.1038/s41591-020-0912-6
   Zhou P, 2016, SCI REP-UK, V6, DOI 10.1038/srep38597
NR 190
TC 0
Z9 0
U1 0
U2 0
PU WILEY
PI HOBOKEN
PA 111 RIVER ST, HOBOKEN 07030-5774, NJ USA
SN 0077-8923
EI 1749-6632
J9 ANN NY ACAD SCI
JI Ann. N.Y. Acad. Sci.
PD 2024 OCT 4
PY 2024
DI 10.1111/nyas.15233
EA OCT 2024
PG 14
WC Multidisciplinary Sciences
WE Science Citation Index Expanded (SCI-EXPANDED)
SC Science & Technology - Other Topics
GA I2P7T
UT WOS:001328734300001
PM 39365995
OA hybrid
DA 2024-10-21
ER

PT J
AU Zhou, H
   Ma, L
   Liu, LY
   Yao, XS
AF Zhou, Hao
   Ma, Long
   Liu, Longyu
   Yao, Xinsheng
TI TR Locus Annotation and Characteristics of <i>Rhinolophus
   ferrumequinum</i>
SO FRONTIERS IN IMMUNOLOGY
LA English
DT Article
DE TR loci; annotation; IMGT; bat; T-cell receptor; immunogenomics
ID CHAIN DIVERSITY; EVOLUTION; BAT; IMMUNOGLOBULIN; SHEEP; TCR;
GENOMES;
   GENE; RECOMBINATION; ORGANIZATION
AB T-cell antigen receptors (TRs) in vertebrates can be divided into
alpha beta or gamma delta, encoded by TRA/D, TRG, or TRB loci. TRs
play a central role in mammal cellular immunity, which occurs by

rearrangement of V, D, J, and C genes in the loci. The bat is the only mammal with flying ability and is considered the main host of zoonotic viruses, an important public health concern. However, at present, little is known about the composition of bat TR genes. Based on the whole genome sequence of the greater horseshoe bat (Rhinolophus ferrumequinum) and referring to the TR/IG annotation rules formulated by the international ImMunoGeneTics information system (IMGT), we present a complete annotation of TRA/D, TRG, and TRB loci of R. ferrumequinum. A total of 128 V segments, three D segments, 85 J segments, and 6 C segments were annotated and compared with other known mammalian data. The characteristics of the TR locus and germline genes of R. ferrumequinum are analyzed.
C1 [Zhou, Hao; Ma, Long; Liu, Longyu; Yao, Xinsheng] Zunyi Med Univ, Ctr Immunomol Engn Innovat & Practice Base Grad S, Dept Immunol, Zunyi, Guizhou, Peoples R China.
C3 Zunyi Medical University
RP Ma, L; Yao, XS (corresponding author), Zunyi Med Univ, Ctr Immunomol Engn Innovat & Practice Base Grad S, Dept Immunol, Zunyi, Guizhou, Peoples R China.
EM immunology01@126.com; 164849671@qq.com
RI Ma, Linan/GYR-2386-2022
FU National Natural Science Foundation of China [31860257]; Guizhou
Provincial High-level Innovative Talents Project [(2018) 5637]
FX The National Natural Science Foundation of China (31860257) and Guizhou
Provincial High-level Innovative Talents Project [No. (2018) 5637]
funded this study.
CR Ahn M, 2019, NAT MICROBIOL, V4, P789, DOI 10.1038/s41564-019-0371-3
[Anonymous], 2012, JANEWAYS IMMUNOBIOLO
Antonacci R, 2007, VET RES COMMUN, V31, P977, DOI 10.1007/s11259-006-0202-x
Antonacci R, 2020, GENES-BASEL, V11, DOI 10.3390/genes11060624
Baker ML, 2010, IMMUNOGENETICS, V62, P173, DOI 10.1007/s00251-010-0425-4
Banerjee A, 2020, FRONT IMMUNOL, V11, DOI 10.3389/fimmu.2020.00026
Bratsch S, 2011, DEV COMP IMMUNOL, V35, P421, DOI 10.1016/j.dci.2010.06.004
Burgin CJ, 2018, J MAMMAL, V99, P1, DOI 10.1093/jmammal/gyx147
Butler JE, 2011, DEV COMP IMMUNOL, V35, P273, DOI 10.1016/j.dci.2010.08.011
Connelley TK, 2014, BMC GENOMICS, V15, DOI 10.1186/1471-2164-15-994
Conrad ML, 2007, VET IMMUNOL IMMUNOP, V115, P346, DOI 10.1016/j.vetimm.2006.10.019
Csorba G., 2003, HORSESHOE BATSS WORL
EARLY P, 1980, CELL, V19, P981, DOI 10.1016/0092-8674(80)90089-6
Giudicelli V, 1999, BIOINFORMATICS, V15, P1047, DOI 10.1093/bioinformatics/15.12.1047
Giudicelli Veronique, 2012, Frontiers in Genetics, V3, P79, DOI 10.3389/fgene.2012.00079
Giudicelli W, 2005, NUCLEIC ACIDS RES, V33, pD256, DOI 10.1093/

nar/gki010
Hawkins JA, 2019, P NATL ACAD SCI USA, V116, P11351, DOI 10.1073/pnas.1814995116
Jebb D, 2020, NATURE, V583, P578, DOI 10.1038/s41586-020-2486-3
Krebs JE., 2011, LEWINS GENES, P490
Lane J, 2010, BMC BIOINFORMATICS, V11, DOI 10.1186/1471-2105-11-223
Lei M, 2016, SCI REP-UK, V6, DOI 10.1038/srep27726
Litman GW, 2010, NAT REV IMMUNOL, V10, P543, DOI 10.1038/nri2807
Massari S, 2009, MOL IMMUNOL, V46, P2728, DOI 10.1016/j.molimm.2009.05.008
Massari S, 1998, CHROMOSOME RES, V6, P419, DOI 10.1023/A:1009245830804
Massari S, 2021, GENES-BASEL, V12, DOI 10.3390/genes12040544
MCBLANE JF, 1995, CELL, V83, P387, DOI 10.1016/0092-8674(95)90116-7
Mehdizadeh R, 2020, MITOCHONDRIAL DNA A, V31, P87, DOI 10.1080/24701394.2020.1741562
Miccoli MC, 2003, J MOL EVOL, V57, P52, DOI 10.1007/s00239-002-2451-9
Miccoli MC, 2001, IMMUNOGENETICS, V53, P416, DOI 10.1007/s002510100340
Nei M, 2005, ANNU REV GENET, V39, P121, DOI 10.1146/annurev.genet.39.073003.112240
Nei M, 1997, P NATL ACAD SCI USA, V94, P7799, DOI 10.1073/pnas.94.15.7799
Parra ZE, 2008, BMC GENOMICS, V9, DOI 10.1186/1471-2164-9-111
Parra ZE, 2007, P NATL ACAD SCI USA, V104, P9776, DOI 10.1073/pnas.0609106104
Parra ZE, 2006, DEV COMP IMMUNOL, V30, P699, DOI 10.1016/j.dci.2005.10.002
Parra ZE, 2012, MOL BIOL EVOL, V29, P3205, DOI 10.1093/molbev/mss128
Parra ZE, 2010, EUR J IMMUNOL, V40, P2319, DOI 10.1002/eji.201040515
Parra ZE, 2009, J IMMUNOL, V182, P154, DOI 10.4049/jimmunol.182.1.154
Pavlovich SS, 2018, CELL, V173, P1098, DOI 10.1016/j.cell.2018.03.070
Pégorier P, 2021, GENES-BASEL, V12, DOI 10.3390/genes12010030
Pégorier P, 2020, FRONT IMMUNOL, V11, DOI 10.3389/fimmu.2020.00821
Piccinni B, 2015, BMC GENOMICS, V16, DOI 10.1186/s12864-015-1790-z
Radtanakatikanon A, 2020, BMC GENOMICS, V21, DOI 10.1186/s12864-019-6431-5
Richards MH, 2000, MOL BIOL EVOL, V17, P146, DOI 10.1093/oxfordjournals.molbev.a026227
Simmons N. B., Bat Species of the World: A taxonomic and geographic database
Simmons NB, 2008, NATURE, V451, P818, DOI 10.1038/nature06549
Su C, 2001, MOL BIOL EVOL, V18, P503, DOI 10.1093/oxfordjournals.molbev.a003829
Teeling EC, 2005, SCIENCE, V307, P580, DOI 10.1126/

science.1105113
   TJOELKER LW, 1990, P NATL ACAD SCI USA, V87, P7856, DOI 10.1073/
pnas.87.20.7856
   Vaccarelli G, 2005, GENE, V357, P103, DOI 10.1016/
j.gene.2005.05.033
   Vaccarelli G, 2012, EUR J IMMUNOL, V42, P3416, DOI 10.1002/
eji.201142176
   Wang XX, 2011, J IMMUNOL, V187, P5246, DOI 10.4049/
jimmunol.1101113
   Wynne JW, 2013, PLOS ONE, V8, DOI 10.1371/journal.pone.0052930
   Yazawa R, 2008, MOL IMMUNOL, V45, P2150, DOI 10.1016/
j.molimm.2007.12.007
   Zhou P, 2016, P NATL ACAD SCI USA, V113, P2696, DOI 10.1073/
pnas.1518240113
NR 54
TC 5
Z9 5
U1 1
U2 5
PU FRONTIERS MEDIA SA
PI LAUSANNE
PA AVENUE DU TRIBUNAL FEDERAL 34, LAUSANNE, CH-1015, SWITZERLAND
SN 1664-3224
J9 FRONT IMMUNOL
JI Front. Immunol.
PD SEP 30
PY 2021
VL 12
AR 741408
DI 10.3389/fimmu.2021.741408
PG 18
WC Immunology
WE Science Citation Index Expanded (SCI-EXPANDED)
SC Immunology
GA WH2GE
UT WOS:000707502400001
PM 34659234
OA Green Submitted, Green Published, gold
DA 2024-10-21
ER

PT J
AU Wang, XR
   Ding, PW
   Sun, CC
   Wang, DX
   Zhu, JC
   Wu, WD
   Wei, Y
   Xiang, R
   Ding, XN
   Luo, LH
   Li, ML
   Zhang, WS

Jin, X
Sun, J
Liu, H
Chen, DS
AF Wang, Xiran
Ding, Peiwen
Sun, Chengcheng
Wang, Daxi
Zhu, Jiacheng
Wu, Wendi
Wei, Yanan
Xiang, Rong
Ding, Xiangning
Luo, Lihua
Li, Meiling
Zhang, Wensheng
Jin, Xin
Sun, Jian
Liu, Huan
Chen, Dongsheng
TI Comparative analysis of single cell lung atlas of bat, cat, tiger, and
pangolin
SO CELL BIOLOGY AND TOXICOLOGY
LA English
DT Article
DE Single cell; Bat; Cross species; Comparative analysis
AB Horseshoe bats (Rhinolophus sinicus) might help maintain coronaviruses severely affecting human health, such as severe acute respiratory syndrome coronavirus (SARS-CoV). Bats may be more tolerant of viral infection than other mammals due to their unique immune system, but the exact mechanism remains to be fully explored. During the coronavirus disease 2019 (COVID-19) pandemic, multiple animal species were diseased by coronavirus infection, especially in the respiratory system. Herein, a comparative analysis with single nucleus transcriptomic data of the lungs across four species, including horseshoe bat, cat, tiger, and pangolin, were conducted. The distribution of entry factors for twenty-eight respiratory viruses was characterized for the four species. Our findings might increase our understanding of the immune background of horseshoe bats.
C1 [Wang, Xiran; Sun, Jian] South China Agr Univ, Natl Risk Assessment Lab Antimicrobial Resistance, Guangzhou, Peoples R China.
[Wang, Xiran; Sun, Jian] Guangdong Lab Lingnan Modern Agr, Guangzhou, Peoples R China.
[Ding, Peiwen; Sun, Chengcheng; Wang, Daxi; Zhu, Jiacheng; Wu, Wendi; Wei, Yanan; Xiang, Rong; Ding, Xiangning; Luo, Lihua; Li, Meiling; Jin, Xin; Liu, Huan; Chen, Dongsheng] BGI Shenzhen, Shenzhen 518083, Peoples R China.
[Ding, Peiwen; Sun, Chengcheng; Zhu, Jiacheng; Wu, Wendi; Wei, Yanan; Ding, Xiangning; Luo, Lihua; Liu, Huan; Chen, Dongsheng] Univ Chinese Acad Sci, Coll Life Sci, Beijing 100049, Peoples R China.
[Sun, Chengcheng; Wu, Wendi; Wei, Yanan] Qingdao Univ, Sch Basic Med, Qingdao, Peoples R China.

[Wang, Daxi] BGI Shenzhen, Shenzhen Key Lab Unknown Pathogen Identificat, Shenzhen 518083, Peoples R China.
[Zhang, Wensheng] Binzhou Med Univ, Sch Basic Med Sci, 346 Guanhai Rd, Shandong, Peoples R China.
C3 South China Agricultural University; Guangdong Laboratory for Lingnan Modern Agriculture; Beijing Genomics Institute (BGI); Chinese Academy of Sciences; University of Chinese Academy of Sciences, CAS; Qingdao University; Beijing Genomics Institute (BGI); Binzhou Medical University
RP Sun, J (corresponding author), South China Agr Univ, Natl Risk Assessment Lab Antimicrobial Resistance, Guangzhou, Peoples R China.; Liu, H; Chen, DS (corresponding author), BGI Shenzhen, Shenzhen 518083, Peoples R China.; Liu, H; Chen, DS (corresponding author), Univ Chinese Acad Sci, Coll Life Sci, Beijing 100049, Peoples R China.
EM jiansun@scau.edu.cn; liuhuan@genomics.cn; chendongsheng@genomics.cn
RI Jiang, Yuan/JED-3759-2023; XIANG, Rong/B-2589-2008; zhang, xinyi/JWA-0980-2024; Wang, Xiran/HCG-9631-2022; liu, huan/JKI-3764-2023;
Wang, Peilin/JWP-6008-2024; Li, Meiling/AAF-1917-2020
OI Liu, Huan/0000-0003-3909-0931; Sun, Chengcheng/0000-0003-1122-1952
FU Guangdong Major Project of Basic and Applied Basic Research [2020B0301030007]; Local Innovative and Research Teams Project of Guangdong Pearl River Talents Program [2019BT02N054]; Program for Changjiang Scholars and Innovative Research Team in University of Ministry of Education of China [IRT_17R39]; Innovation Team Project of Guangdong University [2019KCXTD001]
FX This work was supported by the Guangdong Major Project of Basic and Applied Basic Research (Grant 2020B0301030007), the Local Innovative and Research Teams Project of Guangdong Pearl River Talents Program (Grant 2019BT02N054), the Program for Changjiang Scholars and Innovative Research Team in University of Ministry of Education of China (Grant No. IRT_17R39), the Innovation Team Project of Guangdong University (Grant No. 2019KCXTD001).
CR Banerjee A, 2020, FRONT IMMUNOL, V11, DOI 10.3389/fimmu.2020.00026
Chen DS, 2022, NUCLEIC ACIDS RES, V50, pD934, DOI 10.1093/nar/gkab894
Chen DS, 2021, NAT COMMUN, V12, DOI 10.1038/s41467-021-27162-2
Gadient RA, 1999, STEM CELLS, V17, P127, DOI 10.1002/stem.170127
Ge XY, 2013, NATURE, V503, P535, DOI 10.1038/nature12711
Glennon NB, 2015, J VIROL, V89, P7550, DOI 10.1128/JVI.00302-15
Hu B, 2017, PLOS PATHOG, V13, DOI 10.1371/journal.ppat.1006698
Irving AT, 2021, NATURE, V589, P363, DOI 10.1038/

s41586-020-03128-0
   Lam TTY, 2020, NATURE, V583, P282, DOI 10.1038/s41586-020-2169-0
   Li WD, 2005, SCIENCE, V310, P676, DOI 10.1126/science.1118391
   Liu P, 2019, VIRUSES-BASEL, V11, DOI 10.3390/v11110979
   McAloose D, 2020, MBIO, V11, DOI 10.1128/mBio.02220-20
   Newman A, 2020, MMWR-MORBID MORTAL W, V69, P710, DOI 10.15585/mmwr.mm6923e3
   Papenfuss AT, 2012, BMC GENOMICS, V13, DOI 10.1186/1471-2164-13-261
   Tisoncik JR, 2012, MICROBIOL MOL BIOL R, V76, P16, DOI 10.1128/MMBR.05015-11
   Wynne JW, 2014, GENOME BIOL, V15, DOI 10.1186/s13059-014-0532-x
   Xie JZ, 2018, CELL HOST MICROBE, V23, P297, DOI 10.1016/j.chom.2018.01.006
   Zhang Q, 2020, EMERG MICROBES INFEC, V9, P2013, DOI 10.1080/22221751.2020.1817796
   Zhou H, 2021, CELL, V184, P4380, DOI 10.1016/j.cell.2021.06.008
   Zhou P, 2020, NATURE, V579, P270, DOI [10.1038/s41586-020-2012-7, 10.1038/s41586-020-2951-z]
   Zhou P, 2016, P NATL ACAD SCI USA, V113, P2696, DOI 10.1073/pnas.1518240113
NR 21
TC 2
Z9 2
U1 1
U2 13
PU SPRINGER
PI DORDRECHT
PA VAN GODEWIJCKSTRAAT 30, 3311 GZ DORDRECHT, NETHERLANDS
SN 0742-2091
EI 1573-6822
J9 CELL BIOL TOXICOL
JI Cell Biol. Toxicol.
PD OCT
PY 2023
VL 39
IS 5
BP 2431
EP 2435
DI 10.1007/s10565-022-09771-9
EA SEP 2022
PG 5
WC Cell Biology; Toxicology
WE Science Citation Index Expanded (SCI-EXPANDED)
SC Cell Biology; Toxicology
GA T7MW5
UT WOS:000861200000001
PM 36169743
OA Bronze, Green Submitted, Green Published
DA 2024-10-21
ER

PT J
AU Delbos, V

Abgueguen, P
Chennebault, JM
Pichard, E
AF Delbos, V.
Abgueguen, P.
Chennebault, J. -M.
Pichard, E.
TI A new case of bat rabies in France
SO REVUE DE MEDECINE INTERNE
LA French
DT Article
DE rabies; zoonosis; chiroptera; postexposure treatment
AB Introduction. - Bat rabies represents an emerging zoonosis in Europe and the only endemic cause of rabies in France.
Case record. - A 29 year-old woman was bitten at the hand by a bat. The diagnosis of bat rabies was positive and the viral strain was an European Bat Lyssavirus 1a. A combination of rabies vaccine and human rabies immune globulin was provided to the patient.
Discussion. - Any direct contact with a bat must be avoided. In case of exposure to bats, the postexposure treatment must associate the rabies vaccine and human rabies immune globulin because of antigenic diversity of Lyssavirus circulating in bat species. (C) 2006 Elsevier SAS. Tous droits reserves.
C1 CHU Angers, Serv Malad Infect & Trop, F-49933 Angers 09, France.
C3 Universite d'Angers; Centre Hospitalier Universitaire d'Angers
RP Delbos, V (corresponding author), CHU Angers, Serv Malad Infect & Trop, 4 Rue Larrey, F-49933 Angers 09, France.
EM VaDelbos@chu-angers.fr
CR *CDCP, 1997, MMWR-MORBID MORTAL W, V46, P770
Centers for Disease Control and Prevention (CDC), 2000, MMWR Morb Mortal Wkly Rep, V49, P1111
Fooks AR, 2003, EPIDEMIOL INFECT, V131, P1029, DOI 10.1017/S0950268803001481
NANCY A, 2000, CAS RAGE CHAUVES SOU, P30
NANCY A, 2003, RAPPORT RAGE CHIROPE, P1
Nathwani D, 2003, CLIN INFECT DIS, V37, P598, DOI 10.1086/376641
ROTIVEL Y, 2001, B EPIDEMIOL HEB, V39
NR 7
TC 0
Z9 0
U1 0
U2 3
PU ELSEVIER FRANCE-EDITIONS SCIENTIFIQUES MEDICALES ELSEVIER
PI ISSY-LES-MOULINEAUX
PA 65 RUE CAMILLE DESMOULINS, CS50083, 92442 ISSY-LES-MOULINEAUX, FRANCE
SN 0248-8663
J9 REV MED INTERNE
JI Rev. Med. Interne
PD JUL
PY 2006
VL 27
IS 7
BP 575

EP 577
DI 10.1016/j.revmed.2006.04.002
PG 3
WC Medicine, General & Internal
WE Science Citation Index Expanded (SCI-EXPANDED)
SC General & Internal Medicine
GA 067TH
UT WOS:000239325000015
PM 16797796
DA 2024-10-21
ER

PT J
AU Weaver, KN
   Alfano, SE
   Kronquist, AR
   Reeder, DM
AF Weaver, Kimberly N.
   Alfano, Sara E.
   Kronquist, Amanda R.
   Reeder, DeeAnn M.
TI Healing rates of wing punch wounds in free-ranging little brown myotis
   (<i>Myotis lucifugus</i>)
SO ACTA CHIROPTEROLOGICA
LA English
DT Article
DE Chiroptera; energy balance; immune function; Myotis lucifugus; wing
   biopsy; wing damage; wound healing
ID WHITE-NOSE SYNDROME; SEASONAL-CHANGES; PALLID BATS; STRESS
C1 [Weaver, Kimberly N.; Alfano, Sara E.; Kronquist, Amanda R.; Reeder, DeeAnn M.] Bucknell Univ, Dept Biol, Lewisburg, PA 17837 USA.
C3 Bucknell University
RP Reeder, DM (corresponding author), Bucknell Univ, Dept Biol, Lewisburg, PA 17837 USA.
EM dreeder@bucknell.edu
OI Reeder, DeeAnn/0000-0001-8651-2012
FU Bucknell University Undergraduate Summer Research Fellowships
FX This work was supported by Bucknell University Undergraduate Summer
   Research Fellowships in the Biological Sciences to KNW, SEA, and ARK.
   Additional field assistance was provided by J. B. Kobilis, K. J. Piatt,
   and R. Jacob.
CR [Anonymous], 1996, BAT RES NEWS
   Blehert DS, 2009, SCIENCE, V323, P227, DOI 10.1126/science.1163874
   BOGDANOWICZ W, 1986, ACTA THERIOL, V31, P180, DOI 10.4098/AT.arch.86-19
   BONACCORSO FJ, 1976, J MAMMAL, V57, P181, DOI 10.2307/1379526
   BUCKNALL TE, 1980, BRIT J SURG, V67, P851, DOI 10.1002/

bjs.1800671205
Christian LM, 2006, NEUROIMMUNOMODULAT, V13, P337, DOI 10.1159/000104862
CHURCH JCT, 1968, BRIT J SURG, V55, P26, DOI 10.1002/bjs.1800550108
DAVIS R, 1972, J MAMMAL, V53, P377, DOI 10.2307/1379180
DAVIS R, 1968, AM MIDL NAT, V79, P388, DOI 10.2307/2423184
Gannon WL, 2007, J MAMMAL, V88, P809, DOI 10.1644/06-MAMM-F-185R1.1
Kerth G, 2000, MOL ECOL, V9, P793, DOI 10.1046/j.1365-294x.2000.00934.x
Kerth G, 2002, MOL ECOL, V11, P1491, DOI 10.1046/j.1365-294X.2002.01528.x
Kinsey SG, 2003, PHYSIOL BEHAV, V78, P205, DOI 10.1016/S0031-9384(02)00967-8
KURTA A, 1989, PHYSIOL ZOOL, V62, P804, DOI 10.1086/physzool.62.3.30157928
Martin LB, 2008, PHILOS T R SOC B, V363, P321, DOI 10.1098/rstb.2007.2142
Nelson RJ, 1996, Q REV BIOL, V71, P511, DOI 10.1086/419555
Reeder DeeAnn M., 2008, Bat Research News, V49, P75
NR 17
TC 33
Z9 41
U1 0
U2 35
PU MUSEUM & INST ZOOLOGY PAS-POLISH ACAD SCIENCES
PI WARSAW
PA WILCZA STREET 64, 00-679 WARSAW, POLAND
SN 1508-1109
EI 1733-5329
J9 ACTA CHIROPTEROL
JI Acta Chiropt.
PY 2009
VL 11
IS 1
BP 220
EP 223
DI 10.3161/150811009X465866
PG 4
WC Zoology
WE Science Citation Index Expanded (SCI-EXPANDED)
SC Zoology
GA 471QG
UT WOS:000268072600022
DA 2024-10-21
ER

PT J
AU van Tol, S
Hage, A
Rajsbaum, R
Freiberg, AN
AF van Tol, Sarah

Hage, Adam
Rajsbaum, Ricardo
Freiberg, Alexander N.
TI <i>Pteropus vampyrus</i> TRIM40 Is an Interferon-Stimulated Gene That
Antagonizes RIG-I-like Receptors
SO VIRUSES-BASEL
LA English
DT Article
DE tripartite motif (TRIM) proteins; RIG-I-like receptors; MDA5; RIG-I;
Nipah virus (NiV); TRIM40; inflammatory responses in bats and humans;
innate immunity; type-I interferons (IFNs)
ID NIPAH VIRUS-INFECTION; HENDRA VIRUS; FLYING FOXES; BATS; REVEALS; MOTIF;
DETERMINANTS; EXPRESSION; EVOLUTION; RESERVOIR
AB Nipah virus (NiV; genus: Henipavirus; family: Paramyxoviridae)
naturally infects Old World fruit bats (family Pteropodidae) without
causing overt disease. Conversely, NiV infection in humans and other
mammals can be lethal. Comparing bat antiviral responses with those
of humans may illuminate the mechanisms that facilitate bats'
tolerance. Tripartite motif proteins (TRIMs), a large family of E3-
ubiquitin ligases, fine-tune innate antiviral immune responses, and
two human TRIMs interact with Henipavirus proteins. We hypothesize
that NiV infection induces the expression of an immunosuppressive
TRIM in bat, but not human cells, to promote tolerance. Here, we
show that TRIM40 is an interferon-stimulated gene (ISG) in
pteropodid but not human cells. Knockdown of bat TRIM40 increases
gene expression of IFN beta, ISGs, and pro-inflammatory cytokines
following poly(I:C) transfection. In Pteropus vampyrus, but not
human cells, NiV induces TRIM40 expression within 16 h after
infection, and knockdown of TRIM40 correlates with reduced NiV
titers as compared to control cells. Bats may have evolved to
express TRIM40 in response to viral infections to control
immunopathogenesis.
C1 [van Tol, Sarah; Hage, Adam; Rajsbaum, Ricardo] Univ Texas Med
Branch, Dept Microbiol & Immunol, Galveston, TX 77555 USA.
[Rajsbaum, Ricardo] Rutgers State Univ, Ctr Virus Host Innate
Immun, New Jersey Med Sch, RBHS Inst Infect & Inflammatory Dis,
Newark, NJ 07102 USA.
[Rajsbaum, Ricardo; Freiberg, Alexander N.] Univ Texas Med
Branch, Sealy & Smith Fdn, Inst Human Infect & Immun, Galveston, TX
77555 USA.
[Freiberg, Alexander N.] Univ Texas Med Branch, Dept Pathol,
Galveston, TX 77555 USA.
[Freiberg, Alexander N.] Univ Texas Med Branch, Ctr Biodef &
Emerging Infect Dis, Galveston, TX 77555 USA.
C3 University of Texas System; University of Texas Medical Branch
Galveston; Rutgers University System; Rutgers University New Brunswick;
Rutgers University Biomedical & Health Sciences; Rutgers University
Newark; University of Texas System; University of Texas Medical

Branch
Galveston; University of Texas System; University of Texas Medical
Branch Galveston; University of Texas System; University of Texas
Medical Branch Galveston
RP Rajsbaum, R (corresponding author), Univ Texas Med Branch, Dept Microbiol & Immunol, Galveston, TX 77555 USA.; Rajsbaum, R (corresponding author), Rutgers State Univ, Ctr Virus Host Innate Immun, New Jersey Med Sch, RBHS Inst Infect & Inflammatory Dis, Newark, NJ 07102 USA.; Rajsbaum, R; Freiberg, AN (corresponding author), Univ Texas Med Branch, Sealy & Smith Fdn, Inst Human Infect & Immun, Galveston, TX 77555 USA.; Freiberg, AN (corresponding author), Univ Texas Med Branch, Dept Pathol, Galveston, TX 77555 USA.; Freiberg, AN (corresponding author), Univ Texas Med Branch, Ctr Biodef & Emerging Infect Dis, Galveston, TX 77555 USA.
EM sarah.vantol@nih.gov; adam.hage@nih.gov; ricardo.rajsbaum@rutgers.edu;
anfreibe@utmb.edu
RI Rajsbaum, Ricardo/JVO-9819-2024
OI Rajsbaum, Ricardo/0000-0002-5058-5444; Freiberg, Alexander
N/0000-0001-8413-8960
FU NIH/NIAID
FX We thank Megan Shaw for providing the PVK4 cells and Ayato Takada for
providing the ZFBK13-75A cells.
CR Ahn M, 2019, NAT MICROBIOL, V4, P789, DOI 10.1038/s41564-019-0371-3
Ahn M, 2016, SCI REP-UK, V6, DOI 10.1038/srep21722
Amaya M, 2020, ANNU REV VIROL, V7, P447, DOI 10.1146/annurev-virology-021920-113833
Bharaj P, 2016, PLOS PATHOG, V12, DOI 10.1371/journal.ppat.1005880
Burroughs AL, 2016, PLOS ONE, V11, DOI 10.1371/journal.pone.0155252
Chua KB, 2000, SCIENCE, V288, P1432, DOI 10.1126/science.288.5470.1432
Chua KB, 2002, MICROBES INFECT, V4, P145, DOI 10.1016/S1286-4579(01)01522-2
De La Cruz-Rivera PC, 2018, J IMMUNOL, V200, P209, DOI 10.4049/jimmunol.1701214
Dong JH, 2020, CELL, V183, P1536, DOI 10.1016/j.cell.2020.11.023
Edson D, 2015, PLOS ONE, V10, DOI 10.1371/journal.pone.0140670
Esposito D, 2017, BIOCHEM SOC T, V45, P183, DOI 10.1042/BST20160325
Geisbert TW, 2021, NPJ VACCINES, V6, DOI 10.1038/s41541-021-00284-w
Geisbert TW, 2012, CURR TOP MICROBIOL, V359, P153, DOI 10.1007/82_2012_208
Glennon NB, 2015, J VIROL, V89, P7550, DOI 10.1128/JVI.00302-15
Goldspink LK, 2015, PLOS ONE, V10, DOI 10.1371/journal.pone.0128835
Gonzalez V, 2022, ISCIENCE, V25, DOI 10.1016/j.isci.2022.104779
Guth S, 2022, P NATL ACAD SCI USA, V119, DOI 10.1073/pnas.2113628119

Hahn MB, 2014, J APPL ECOL, V51, P376, DOI 10.1111/1365-2664.12212
Halpin K, 2000, J GEN VIROL, V81, P1927, DOI 10.1099/0022-1317-81-8-1927
Halpin K, 2011, AM J TROP MED HYG, V85, P946, DOI 10.4269/ajtmh.2011.10-0567
Huang ZX, 2019, NAT ECOL EVOL, V3, P1110, DOI 10.1038/s41559-019-0913-3
Islam MS, 2016, EMERG INFECT DIS, V22, P664, DOI 10.3201/eid2204.151747
Jebb D, 2020, NATURE, V583, P578, DOI 10.1038/s41586-020-2486-3
Koliopoulos MG, 2016, EMBO J, V35, P1204, DOI 10.15252/embj.201593741
McNab F, 2015, NAT REV IMMUNOL, V15, P87, DOI 10.1038/nri3787
Middleton DJ, 2007, J COMP PATHOL, V136, P266, DOI 10.1016/j.jcpa.2007.03.002
Nor MNM, 2000, REV SCI TECH OIE, V19, P160
Ogawa H, 2017, VIRUSES-BASEL, V9, DOI 10.3390/v9120371
Pavlovich SS, 2020, FRONT IMMUNOL, V11, DOI 10.3389/fimmu.2020.00435
Pavlovich SS, 2018, CELL, V173, P1098, DOI 10.1016/j.cell.2018.03.070
Pelissier Rodolphe, 2019, F1000Res, V8, DOI 10.12688/f1000research.19975.1
Plowright RK, 2008, P ROY SOC B-BIOL SCI, V275, P861, DOI 10.1098/rspb.2007.1260
Rajsbaum R, 2014, IMMUNITY, V40, P880, DOI 10.1016/j.immuni.2014.04.018
Reymond A, 2001, EMBO J, V20, P2140, DOI 10.1093/emboj/20.9.2140
Reynes JM, 2005, EMERG INFECT DIS, V11, P1042, DOI 10.3201/eid1107.041350
Rusinova I, 2013, NUCLEIC ACIDS RES, V41, pD1040, DOI 10.1093/nar/gks1215
Sánchez-Aparicio MT, 2018, J VIROL, V92, DOI 10.1128/JVI.01960-17
Satterfield BA, 2015, NAT COMMUN, V6, DOI 10.1038/ncomms8483
Schountz T, 2017, FRONT IMMUNOL, V8, DOI 10.3389/fimmu.2017.01098
SELVEY LA, 1995, MED J AUSTRALIA, V162, P642, DOI 10.5694/j.1326-5377.1995.tb126050.x
Shaw AE, 2017, PLOS BIOL, V15, DOI 10.1371/journal.pbio.2004086
Shen Y, 2021, CELL REP, V35, DOI 10.1016/j.celrep.2021.109272
Short KM, 2006, J BIOL CHEM, V281, P8970, DOI 10.1074/jbc.M512755200
SIMMONS N B., 2022, Bat species of the world: A taxonomic and geographic database
Stachowiak B, 2012, PLOS ONE, V7, DOI 10.1371/journal.pone.0030855
van Tol S, 2017, VACCINES-BASEL, V5, DOI 10.3390/vaccines5030023
Versteeg GA, 2014, CYTOKINE GROWTH F R, V25, P563, DOI 10.1016/j.cytogfr.2014.08.001
Versteeg GA, 2013, IMMUNITY, V38, P384, DOI 10.1016/j.immuni.2012.11.013
Woon AP, 2020, PLOS PATHOG, V16, DOI 10.1371/journal.ppat.1008412
Wynne JW, 2014, GENOME BIOL, V15, DOI 10.1186/s13059-014-0532-x
Xie JZ, 2018, CELL HOST MICROBE, V23, P297, DOI 10.1016/

j.chom.2018.01.006
   Yun T, 2015, J VIROL, V89, P1242, DOI 10.1128/JVI.02583-14
   Zhang GJ, 2013, SCIENCE, V339, P456, DOI 10.1126/science.1230835
   Zhao CY, 2017, CELL REP, V21, P1613, DOI 10.1016/
j.celrep.2017.10.020
   Zhou P, 2016, P NATL ACAD SCI USA, V113, P2696, DOI 10.1073/
pnas.1518240113
NR 55
TC 2
Z9 2
U1 0
U2 1
PU MDPI
PI BASEL
PA ST ALBAN-ANLAGE 66, CH-4052 BASEL, SWITZERLAND
EI 1999-4915
J9 VIRUSES-BASEL
JI Viruses-Basel
PD NOV
PY 2023
VL 15
IS 11
AR 2147
DI 10.3390/v15112147
PG 17
WC Virology
WE Science Citation Index Expanded (SCI-EXPANDED)
SC Virology
GA Z8MI1
UT WOS:001114555800001
PM 38005825
OA gold, Green Published
DA 2024-10-21
ER

PT J
AU Zheng, XS
   Wang, Q
   Xie, TT
   Si, HR
   Zhang, W
   Zhu, Y
   Li, A
   Su, J
   Shi, ZL
   Zhou, P
AF Zheng, Xiao-Shuang
   Wang, Qi
   Xie, Ting-ting
   Si, Hao-rui
   Zhang, Wei
   Zhu, Yan
   Li, Ang
   Su, Jia

Shi, Zheng-Li
Zhou, Peng
TI Assessment and sero-diagnosis for coronaviruses with risk of human
spillover
SO EMERGING MICROBES & INFECTIONS
LA English
DT Article
DE Coronavirus; spillover; zoonosis; risk assessment; bats; serology
ID PANGOLINS; HOST
AB Zoonotic coronaviruses (CoVs) caused major human outbreaks in the last two decades. One of the biggest challenges during future CoV disease is ensuring rapid detection and diagnosis at the early phase of a zoonotic event, and active surveillance to the zoonotic high-risk CoVs appears the best way at the present time to provide early warnings. However, there is neither an evaluation of spillover potential nor diagnosis tools for the majority of CoVs. Here, we analyzed the viral traits, including population, genetic diversity, receptor and host species for all 40 alpha- and beta-CoV species, where the human-infecting CoVs are from. Our analysis proposed 20 high-risk CoV species, including 6 of which jumped to human, 3 with evidence of spillover but not to human and 11 without evidence of spillover yet, which prediction were further supported by an analysis of the history of CoV zoonosis. We also found three major zoonotic sources: multiple bat-origin CoV species, the rodent-origin sub-genus Embecovirus and the CoV species AlphaCoV1. Moreover, the Rhinolophidae and Hipposideridae bats harbour a significantly higher proportion of human-threatening CoV species, whereas camel, civet, swine and pangolin could be important intermediate hosts during CoV zoonotic transmission. Finally, we established quick and sensitive serologic tools for a list of proposed high-risk CoVs and validated the methods in serum cross-reaction assays using hyper-immune rabbit sera or clinical samples. By comprehensive risk assessment of the potential human-infecting CoVs, our work provides a theoretical or practical basis for future CoV disease preparedness.
C1 [Zheng, Xiao-Shuang; Wang, Qi; Xie, Ting-ting; Si, Hao-rui; Zhang, Wei; Zhu, Yan; Li, Ang; Su, Jia; Shi, Zheng-Li; Zhou, Peng] Chinese Acad Sci, Wuhan Inst Virol, CAS Key Lab Special Pathogens, Wuhan, Peoples R China.
[Zheng, Xiao-Shuang; Wang, Qi; Xie, Ting-ting; Si, Hao-rui; Li, Ang; Su, Jia] Univ Chinese Acad Sci, Beijing, Peoples R China.
[Zhou, Peng] Guangzhou Int Bio Isl, Guangzhou Lab, 9 XingDaoHuanBei Rd, Guangzhou 510005, Guangdong, Peoples R China.
C3 Chinese Academy of Sciences; Wuhan Institute of Virology, CAS; Chinese
Academy of Sciences; University of Chinese Academy of Sciences, CAS;
Guangzhou Laboratory
RP Shi, ZL; Zhou, P (corresponding author), Chinese Acad Sci, Wuhan Inst Virol, CAS Key Lab Special Pathogens, Wuhan, Peoples R China.; Zhou, P (corresponding author), Guangzhou Int Bio Isl, Guangzhou Lab, 9 XingDaoHuanBei Rd, Guangzhou 510005, Guangdong, Peoples R China.
EM zlshi@wh.iov.cn; peng.zhou@wh.iov.cn

RI Peng, Ziyi/JTT-8210-2023
FU National Key R&D program of China [2021YFC2300901]; R&D Program of
   Guangzhou Laboratory [SPRG22-001]; Strategic Priority Research Program
   of Chinese Academy of Sciences [XDB29010101]
FX This work was supported by National Key R&D program of China: [Grant
   Number 2021YFC2300901]; R&D Program of Guangzhou Laboratory: [Grant
   Number SPRG22-001]; the Strategic Priority Research Program of Chinese
   Academy of Sciences: [Grant Number XDB29010204]; the Strategic Priority
   Research Program of Chinese Academy of Sciences: [Grant Number
   XDB29010101].
NR 0
TC 0
Z9 0
U1 0
U2 0
PU TAYLOR & FRANCIS LTD
PI ABINGDON
PA 2-4 PARK SQUARE, MILTON PARK, ABINGDON OR14 4RN, OXON, ENGLAND
EI 2222-1751
J9 EMERG MICROBES INFEC
JI Emerg. Microbes Infect.
PD DEC 8
PY 2023
VL 12
IS 2
AR 2225932
DI 10.1080/22221751.2023.2225932
PG 10
WC Immunology; Infectious Diseases; Microbiology
WE Science Citation Index Expanded (SCI-EXPANDED)
SC Immunology; Infectious Diseases; Microbiology
GA C4H1T
UT WOS:001288977100003
OA gold, Green Published
DA 2024-10-21
ER

PT J
AU Zheng, XS
   Wang, Q
   Xie, TT
   Si, HR
   Zhang, W
   Zhu, Y
   Li, A
   Su, J
   Shi, ZL
   Zhou, P

AF Zheng, Xiao-Shuang
   Wang, Qi
   Xie, Ting-ting
   Si, Hao-rui
   Zhang, Wei
   Zhu, Yan
   Li, Ang
   Su, Jia
   Shi, Zheng-Li
   Zhou, Peng
TI Assessment and sero-diagnosis for coronaviruses with risk of human
   spillover
SO EMERGING MICROBES & INFECTIONS
LA English
DT Article
DE Coronavirus; spillover; zoonosis; risk assessment; bats; serology; >
ID PANGOLINS; HOST
AB Zoonotic coronaviruses (CoVs) caused major human outbreaks in the last two decades. One of the biggest challenges during future CoV disease is ensuring rapid detection and diagnosis at the early phase of a zoonotic event, and active surveillance to the zoonotic high-risk CoVs appears the best way at the present time to provide early warnings. However, there is neither an evaluation of spillover potential nor diagnosis tools for the majority of CoVs. Here, we analyzed the viral traits, including population, genetic diversity, receptor and host species for all 40 alpha- and beta-CoV species, where the human-infecting CoVs are from. Our analysis proposed 20 high-risk CoV species, including 6 of which jumped to human, 3 with evidence of spillover but not to human and 11 without evidence of spillover yet, which prediction were further supported by an analysis of the history of CoV zoonosis. We also found three major zoonotic sources: multiple bat-origin CoV species, the rodent-origin sub-genus Embecovirus and the CoV species AlphaCoV1. Moreover, the Rhinolophidae and Hipposideridae bats harbour a significantly higher proportion of human-threatening CoV species, whereas camel, civet, swine and pangolin could be important intermediate hosts during CoV zoonotic transmission. Finally, we established quick and sensitive serologic tools for a list of proposed high-risk CoVs and validated the methods in serum cross-reaction assays using hyper-immune rabbit sera or clinical samples. By comprehensive risk assessment of the potential human-infecting CoVs, our work provides a theoretical or practical basis for future CoV disease preparedness.
C1 [Zheng, Xiao-Shuang; Wang, Qi; Xie, Ting-ting; Si, Hao-rui; Zhang, Wei; Zhu, Yan; Li, Ang; Su, Jia; Shi, Zheng-Li; Zhou, Peng] Chinese Acad Sci, Wuhan Inst Virol, CAS Key Lab Special Pathogens, Wuhan, Peoples R China.
   [Zheng, Xiao-Shuang; Wang, Qi; Xie, Ting-ting; Si, Hao-rui; Li, Ang; Su, Jia] Univ Chinese Acad Sci, Beijing, Peoples R China.
   [Zhou, Peng] Guangzhou Int Bio Isl, Guangzhou Lab, 9 XingDaoHuanBei Rd, Guangzhou 510005, Guangdong, Peoples R China.
C3 Chinese Academy of Sciences; Wuhan Institute of Virology, CAS; Chinese

Academy of Sciences; University of Chinese Academy of Sciences, CAS;
Guangzhou Laboratory
RP Shi, ZL; Zhou, P (corresponding author), Chinese Acad Sci, Wuhan Inst Virol, CAS Key Lab Special Pathogens, Wuhan, Peoples R China.
EM zlshi@wh.iov.cn; peng.zhou@wh.iov.cn
RI wu, p/JDW-5015-2023; Shi, Zhengli/A-1013-2013; Li, Shiyue/KFA-3709-2024;
Peng, Ziyi/JTT-8210-2023; li, cheng/KCZ-0615-2024; Wang, Ling/KBA-9814-2024
OI Wang, Ling/0000-0003-0272-2974
FU National Key Ramp;D program of China [2021YFC2300901]; Ramp;D Program of
Guangzhou Laboratory [SPRG22-001]; Strategic Priority Research Program
of Chinese Academy of Sciences [XDB29010204, XDB29010101]
FX This work was supported by National Key R&D program of China: [Grant
Number 2021YFC2300901]; R&D Program of Guangzhou Laboratory: [Grant
Number SPRG22-001]; the Strategic Priority Research Program of Chinese
Academy of Sciences: [Grant Number XDB29010204]; the Strategic Priority
Research Program of Chinese Academy of Sciences: [Grant Number XDB29010101].
CR Burbelo PD, 2009, CLIN VACCINE IMMUNOL, V16, P366, DOI 10.1128/CVI.00350-08
Chen J, 2023, CELL, V186, P850, DOI 10.1016/j.cell.2023.01.019
Corman VM, 2018, ADV VIRUS RES, V100, P163, DOI 10.1016/bs.aivir.2018.01.001
Corman VM, 2016, P NATL ACAD SCI USA, V113, P9864, DOI 10.1073/pnas.1604472113
Cui J, 2019, NAT REV MICROBIOL, V17, P181, DOI 10.1038/s41579-018-0118-9
Forni D, 2017, TRENDS MICROBIOL, V25, P35, DOI 10.1016/j.tim.2016.09.001
Geoghegan JL, 2016, P NATL ACAD SCI USA, V113, P4170, DOI 10.1073/pnas.1521582113
Grange ZL, 2021, P NATL ACAD SCI USA, V118, DOI 10.1073/pnas.2002324118
Guan Y, 2003, SCIENCE, V302, P276, DOI 10.1126/science.1087139
Han YL, 2019, FRONT MICROBIOL, V10, DOI 10.3389/fmicb.2019.01900
He WT, 2022, CELL, V185, P1117, DOI 10.1016/j.cell.2022.02.014
Hu B, 2021, NAT REV MICROBIOL, V19, P141, DOI 10.1038/s41579-020-00459-7
Hu B, 2017, PLOS PATHOG, V13, DOI 10.1371/journal.ppat.1006698
ictv.global, 2022, taxonomy I
iucnredlist, 2022, REDL I
Lam TTY, 2020, NATURE, V583, P282, DOI 10.1038/s41586-020-2169-0
Latinne Alice, 2020, Nat Commun, V11, P4235, DOI [10.1038/s41467-020-17687-3, 10.1101/2020.05.31.116061]
Lau SKP, 2015, J VIROL, V89, P3076, DOI 10.1128/JVI.02420-14
Luo CM, 2018, J VIROL, V92, DOI 10.1128/JVI.00116-18

Luo Y, 2021, VIROL SIN, V36, P559, DOI 10.1007/s12250-020-00321-3
Mehand MS, 2018, ANTIVIR RES, V159, P63, DOI 10.1016/j.antiviral.2018.09.009
Meyer B, 2014, VIRUS RES, V194, P175, DOI 10.1016/j.virusres.2014.03.018
Mollentze N, 2020, P NATL ACAD SCI USA, V117, P9423, DOI 10.1073/pnas.1919176117
Olival KJ, 2017, NATURE, V546, P646, DOI 10.1038/nature22975
Reusken CBEM, 2013, LANCET INFECT DIS, V13, P859, DOI 10.1016/S1473-3099(13)70164-6
Shi WQ, 2022, NAT MICROBIOL, V7, P1259, DOI 10.1038/s41564-022-01181-1
Tan CW, 2021, CURR OPIN VIROL, V49, P68, DOI 10.1016/j.coviro.2021.04.013
Tao Y, 2017, J VIROL, V91, DOI 10.1128/JVI.01953-16
Vlasova AN, 2022, CLIN INFECT DIS, V74, P446, DOI 10.1093/cid/ciab456
Vora NM, 2022, NATURE, V605, P419, DOI 10.1038/d41586-022-01312-y
Wang LF., 2016, Choice: Curr Rev Acad Lib, V53, P901
Warren Cody J, 2019, PLoS Biol, V17, pe3000217, DOI 10.1371/journal.pbio.3000217
Woo PCY, 2016, INT J MOL SCI, V17, DOI 10.3390/ijms17050691
Yang Y, 2014, P NATL ACAD SCI USA, V111, P12516, DOI 10.1073/pnas.1405889111
Zhang W, 2019, EMERG MICROBES INFEC, V8, P1528, DOI 10.1080/22221751.2019.1679610
Zhou P., 2020, NATURE, V579, P270, DOI DOI 10.1038/s41586-020-2012-7
Zhou P, 2018, NATURE, V556, P255, DOI 10.1038/s41586-018-0010-9
NR 37
TC 0
Z9 0
U1 0
U2 0
PU TAYLOR & FRANCIS LTD
PI ABINGDON
PA 2-4 PARK SQUARE, MILTON PARK, ABINGDON OR14 4RN, OXON, ENGLAND
EI 2222-1751
J9 EMERG MICROBES INFEC
JI Emerg. Microbes Infect.
PD DEC 8
PY 2023
VL 12
IS 2
AR 2225932
DI 10.1080/22221751.2023.2225932
PG 10
WC Immunology; Infectious Diseases; Microbiology
WE Science Citation Index Expanded (SCI-EXPANDED)
SC Immunology; Infectious Diseases; Microbiology
GA L5SC7
UT WOS:001023852600001
OA gold, Green Published
DA 2024-10-21

ER

PT J
AU Schaer, J
   Perkins, SL
   Decher, J
   Leendertz, FH
   Fahr, J
   Weber, N
   Matuschewski, K
AF Schaer, Juliane
   Perkins, Susan L.
   Decher, Jan
   Leendertz, Fabian H.
   Fahr, Jakob
   Weber, Natalie
   Matuschewski, Kai
TI High diversity of West African bat malaria parasites and a tight link
   with rodent <i>Plasmodium</i> taxa
SO PROCEEDINGS OF THE NATIONAL ACADEMY OF SCIENCES OF THE UNITED STATES OF
   AMERICA
LA English
DT Article
DE Haemosporida; Chiroptera; vector-borne disease; molecular phylogeny;
   host-pathogen coevolution
ID CYTOCHROME-B; EVOLUTION; PHYLOGENY; SEQUENCE; HOSTS
AB As the only volant mammals, bats are captivating for their high taxonomic diversity, for their vital roles in ecosystems-particularly as pollinators and insectivores-and, more recently, for their important roles in the maintenance and transmission of zoonotic viral diseases. Genome sequences have identified evidence for a striking expansion of and positive selection in gene families associated with immunity. Bats have also been known to be hosts of malaria parasites for over a century, and as hosts, they possess perhaps the most phylogenetically diverse set of hemosporidian genera and species. To provide a molecular framework for the study of these parasites, we surveyed bats in three remote areas of the Upper Guinean forest ecosystem. We detected four distinct genera of hemosporidian parasites: Plasmodium, Polychromophilus, Nycteria, and Hepatocystis. Intriguingly, the two species of Plasmodium in bats fall within the clade of rodent malaria parasites, indicative of multiple host switches across mammalian orders. We show that Nycteria species form a very distinct phylogenetic group and that Hepatocystis parasites display an unusually high diversity and prevalence in epauletted fruit bats. The diversity and high prevalence of novel lineages of chiropteran hemosporidians underscore the exceptional position of bats among all other mammalian hosts of hemosporidian parasites and support hypotheses of pathogen tolerance consistent with the exceptional immunology of bats.
C1 [Schaer, Juliane; Matuschewski, Kai] Max Planck Inst Infect Biol,

Parasitol Unit, D-10117 Berlin, Germany.
[Schaer, Juliane] Leibniz Inst Res Evolut & Biodivers, Museum Nat Kunde, D-10115 Berlin, Germany.
[Perkins, Susan L.] Amer Museum Nat Hist, Sackler Inst Comparat Genom, New York, NY 10024 USA.
[Decher, Jan] Zool Forsch Museum A Konig, Sect Mammals, D-53113 Bonn, Germany.
[Leendertz, Fabian H.] Robert Koch Inst, D-13302 Berlin, Germany.
[Fahr, Jakob] Tech Univ Carolo Wilhelmina Braunschweig, Inst Zool, D-38106 Braunschweig, Germany.
[Fahr, Jakob] Max Planck Inst Ornithol, Dept Migrat & Immunoecol, D-78315 Radolfzell am Bodensee, Germany.
[Weber, Natalie] Univ Ulm, Inst Expt Ecol, D-89069 Ulm, Germany.
[Matuschewski, Kai] Humboldt Univ, Inst Biol, D-10117 Berlin, Germany.
C3 Max Planck Society; Leibniz Institut fur Evolutions und Biodiversitatsforschung; American Museum of Natural History (AMNH);
Robert Koch Institute; Braunschweig University of Technology; Max Planck Society; Ulm University; Humboldt University of Berlin
RP Schaer, J (corresponding author), Max Planck Inst Infect Biol, Parasitol Unit, D-10117 Berlin, Germany.
EM schaer@mpiib-berlin.mpg.de; perkins@amnh.org
RI Leendertz, Fabian/H-8902-2019; Schaer, Juliane/AAR-1024-2020; Perkins, Susan/D-3408-2018; Fahr, Jakob/E-8831-2012
OI Matuschewski, Kai/0000-0001-6147-8591; Schaer, Juliane/0000-0001-6714-5771; Perkins, Susan/0000-0002-5400-5662; Fahr, Jakob/0000-0002-9174-1204; Weber, Natalie/0000-0003-0390-1229
FU Research Coordination grant from the US National Science Foundation; Annette Kade Fellowship from the American Museum of Natural History; Max Planck Society
FX We thank Stefan Pettersson and Siv Aina Leendertz, who captured the bats in Cote d'Ivoire; Rainer Hutterer, for identifications and preservation of the bat vouchers from Guinea at the Zoological Museum Koenig in Bonn; Kevin Olival for early contributions to the study; and Frieder Mayer for discussions. We also thank SNC-Lavalin Group Inc.; RioTinto; the Putu Iron Ore Project, which permitted sampling during the consulting jobs of J.S. and J.D. in Guinea, and J.S. in Liberia; the Guinean Ministere en charge de l'Agriculture de l'Elevage de L'Environnement et des Eaux et Forets; the Liberian Forest Development Authority; the authorities in

Cote d'Ivoire for long-term support, especially the Ministry of
Environment and Forests and the Ministry of Research; the
directorship
of the Tai National Park; the Office Ivoirien des Parcs et
Reserves; and
the Swiss Research Center in Abidjan. This research was supported
by a
Research Coordination grant from the US National Science
Foundation (see
http://malariarcn.org), the Annette Kade Fellowship from the
American
Museum of Natural History, and the Max Planck Society.
CR [Anonymous], 1988, PROTOZOAN PHYLUM API
Bueno MG, 2013, ECOHEALTH, V10, P48, DOI 10.1007/s10393-012-0809-z
Calisher CH, 2006, CLIN MICROBIOL REV, V19, P531, DOI 10.1128/CMR.00017-06
Carlton JM, 2002, NATURE, V419, P512, DOI 10.1038/nature01099
Decher Jan, 2005, Mammalian Species, P1
Dionisi A, 1898, ATTI R ACCAD LINCEI, V7, P214
Duval L, 2007, MALARIA J, V6, DOI 10.1186/1475-2875-6-157
Edgar RC, 2004, NUCLEIC ACIDS RES, V32, P1792, DOI 10.1093/nar/gkh340
Eick GN, 2005, MOL BIOL EVOL, V22, P1869, DOI 10.1093/molbev/msi180
Escalante AA, 1998, P NATL ACAD SCI USA, V95, P8124, DOI 10.1073/pnas.95.14.8124
Garnham P. C. C., 1966, MALARIA PARASITES OT
Hagner SC, 2007, PARASITOL RES, V101, P493, DOI 10.1007/s00436-007-0499-6
Janse CJ, 2011, TRENDS PARASITOL, V27, P31, DOI 10.1016/j.pt.2010.06.016
Kaiser M, 2010, EMERG INFECT DIS, V16, P1956, DOI 10.3201/eid1612.100424
Kalka MB, 2008, SCIENCE, V320, P71, DOI 10.1126/science.1153352
LANDAU I, 1978, ANN PARASIT HUM COMP, V53, P247, DOI 10.1051/parasite/1978533247
Leroy EM, 2005, NATURE, V438, P575, DOI 10.1038/438575a
Liu WM, 2010, NATURE, V467, P420, DOI 10.1038/nature09442
Martinsen ES, 2008, MOL PHYLOGENET EVOL, V47, P261, DOI 10.1016/j.ympev.2007.11.012
Martinsen ES, 2013, MALARIA PARASITES: COMPARATIVE GENOMICS , EVOLUTION AND MOLECULAR BIOLOGY, P1
Matuschewski K, 2013, HUM VACC IMMUNOTHER, V9, P450, DOI 10.4161/hv.23218
Nylander JAA, 2008, BIOINFORMATICS, V24, P581, DOI 10.1093/bioinformatics/btm388
Outlaw DC, 2011, P NATL ACAD SCI USA, V108, P13183, DOI 10.1073/pnas.1109153108
Perkins SL, 2002, J PARASITOL, V88, P972, DOI 10.1645/0022-3395(2002)088[0972:AMPOMP]2.0.CO;2
Prugnolle F, 2011, PLOS PATHOG, V7, DOI 10.1371/journal.ppat.1001283
Prugnolle F, 2010, P NATL ACAD SCI USA, V107, P1458, DOI 10.1073/

pnas.0914440107
Reece SE, 2008, NATURE, V453, P609, DOI 10.1038/nature06954
Ronquist F, 2012, SYST BIOL, V61, P539, DOI 10.1093/sysbio/sys029
Rosevear D.R., 1965, BATS W AFRICA
Silvestro D, 2012, ORG DIVERS EVOL, V12, P335, DOI 10.1007/s13127-011-0056-0
Singh B, 2004, LANCET, V363, P1017, DOI 10.1016/S0140-6736(04)15836-4
Singh B, 2009, EMERG INFECT DIS, V15, P1657, DOI 10.3201/eid1510.090364
Stamatakis A, 2008, SYST BIOL, V57, P758, DOI 10.1080/10635150802429642
Sutherland CJ, 2010, J INFECT DIS, V201, P1544, DOI 10.1086/652240
VANDERKAAY HJ, 1964, ANN TROP MED PARASIT, V58, P261, DOI 10.1080/00034983.1964.11686241
Vincke JH, 1948, ANN SOC BELG MED TR, V28, P97
Witsenburg F, 2012, MALARIA J, V11, DOI 10.1186/1475-2875-11-53
Wozencraft CW., 2005, MAMMAL SPECIES WORLD
Zhang GJ, 2013, SCIENCE, V339, P456, DOI 10.1126/science.1230835
NR 39
TC 98
Z9 107
U1 0
U2 49
PU NATL ACAD SCIENCES
PI WASHINGTON
PA 2101 CONSTITUTION AVE NW, WASHINGTON, DC 20418 USA
SN 0027-8424
J9 P NATL ACAD SCI USA
JI Proc. Natl. Acad. Sci. U. S. A.
PD OCT 22
PY 2013
VL 110
IS 43
BP 17415
EP 17419
DI 10.1073/pnas.1311016110
PG 5
WC Multidisciplinary Sciences
WE Science Citation Index Expanded (SCI-EXPANDED)
SC Science & Technology - Other Topics
GA 238JU
UT WOS:000325943300058
PM 24101466
OA Green Published, Bronze
DA 2024-10-21
ER

PT J
AU Schountz, T
AF Schountz, Tony
TI Immunology of Bats and Their Viruses: Challenges and Opportunities

SO VIRUSES-BASEL
LA English
DT Article
DE chiroptera; reservoir host; virus ecology; zoonosis; emerging infectious
disease; RNA-seq; metabolomics
ID RESPIRATORY SYNDROME CORONAVIRUS; SIN NOMBRE HANTAVIRUS; TOLL-LIKE
RECEPTORS; BLACK FLYING FOX; NIPAH-VIRUS; EXPERIMENTAL-INFECTION;
NATURAL RESERVOIR; PTEROPID BATS; MOLECULAR CHARACTERIZATION; EXPRESSION
ANALYSIS
AB Bats are reservoir hosts of several high-impact viruses that cause significant human diseases, including Nipah virus, Marburg virus and rabies virus. They also harbor many other viruses that are thought to have caused disease in humans after spillover into intermediate hosts, including SARS and MERS coronaviruses. As is usual with reservoir hosts, these viruses apparently cause little or no pathology in bats. Despite the importance of bats as reservoir hosts of zoonotic and potentially zoonotic agents, virtually nothing is known about the host/virus relationships; principally because few colonies of bats are available for experimental infections, a lack of reagents, methods and expertise for studying bat antiviral responses and immunology, and the difficulty of conducting meaningful field work. These challenges can be addressed, in part, with new technologies that are species-independent that can provide insight into the interactions of bats and viruses, which should clarify how the viruses persist in nature, and what risk factors might facilitate transmission to humans and livestock.
C1 Colorado State Univ, Arthropod Borne & Infect Dis Lab, Dept Microbiol Immunol & Pathol, Coll Vet Med, Ft Collins, CO 80524 USA.
C3 Colorado State University
RP Schountz, T (corresponding author), Colorado State Univ, Arthropod Borne & Infect Dis Lab, Dept Microbiol Immunol & Pathol, Coll Vet Med, Ft Collins, CO 80524 USA.
EM tony.schountz@colostate.edu
RI Schountz, Tony/I-2161-2017
CR Abraham J, 2010, NAT STRUCT MOL BIOL, V17, P438, DOI 10.1038/nsmb.1772
Albariño CG, 2014, EMERG INFECT DIS, V20, P211, DOI 10.3201/eid2002.131620
Alcami A, 2000, TRENDS MICROBIOL, V8, P410, DOI 10.1016/S0966-842X(00)01830-8
Amman BR, 2015, J WILDLIFE DIS, V51, P113, DOI 10.7589/2014-08-198
Anders S, 2010, GENOME BIOL, V11, DOI 10.1186/gb-2010-11-10-r106
Anders S, 2013, NAT PROTOC, V8, P1765, DOI 10.1038/nprot.2013.099
[Anonymous], 2001, MAMMAL SPECIES, DOI DOI 10.2307/0.662.1
[Anonymous], 2008, Microbe
[Anonymous], ZOONOSES PUBLIC HLTH
Badrane H, 2001, J VIROL, V75, P8096, DOI 10.1128/JVI.75.17.8096-8104.2001
Bai Y, 2011, EMERG INFECT DIS, V17, P1269, DOI 10.3201/eid1707.101867

Baker ML, 2013, ZOONOSES PUBLIC HLTH, V60, P104, DOI 10.1111/j.1863-2378.2012.01528.x
Baker ML, 2010, IMMUNOGENETICS, V62, P173, DOI 10.1007/s00251-010-0425-4
Barbalat R, 2011, ANNU REV IMMUNOL, V29, P185, DOI 10.1146/annurev-immunol-031210-101340
Barr J, 2015, J GEN VIROL, V96, P24, DOI 10.1099/vir.0.068106-0
Biesold SE, 2011, PLOS ONE, V6, DOI 10.1371/journal.pone.0028131
Billeter SA, 2012, PARASITOLOGY, V139, P324, DOI 10.1017/S0031182011002113
Blehert DS, 2009, SCIENCE, V323, P227, DOI 10.1126/science.1163874
Botten J, 2000, P NATL ACAD SCI USA, V97, P10578, DOI 10.1073/pnas.180197197
Botten J, 2003, J VIROL, V77, P1540, DOI 10.1128/JVI.77.2.1540-1550.2002
Bratsch S, 2011, DEV COMP IMMUNOL, V35, P421, DOI 10.1016/j.dci.2010.06.004
Breed AC, 2010, ECOHEALTH, V7, P204, DOI 10.1007/s10393-010-0332-z
Butler JE, 2011, DEV COMP IMMUNOL, V35, P273, DOI 10.1016/j.dci.2010.08.011
Calisher CH, 2006, CLIN MICROBIOL REV, V19, P531, DOI 10.1128/CMR.00017-06
Calisher CH, 2012, TRAVEL MED INFECT DI, V10, P69, DOI 10.1016/j.tmaid.2012.01.003
Carrington CVF, 2008, EMERG INFECT DIS, V14, P1890, DOI 10.3201/eid1412.080642
CHAKRABORTY AK, 1983, INDIAN J EXP BIOL, V21, P5
CHAKRAVARTY AK, 1994, LYMPHOLOGY, V27, P97
Charrel RN, 2002, BIOCHEM BIOPH RES CO, V296, P1118, DOI 10.1016/S0006-291X(02)02053-3
Chen G, 2009, ANNU REV PATHOL-MECH, V4, P365, DOI 10.1146/annurev.pathol.4.110807.092239
Ching S, 2007, J NEUROSCI, V27, P10476, DOI 10.1523/JNEUROSCI.3357-07.2007
Chu DKW, 2008, J VIROL, V82, P9107, DOI 10.1128/JVI.00857-08
Chua KB, 2002, MICROBES INFECT, V4, P145, DOI 10.1016/S1286-4579(01)01522-2
Chukkapalli V, 2012, CURR OPIN MICROBIOL, V15, P512, DOI 10.1016/j.mib.2012.05.013
Cogswell-Hawkinson A, 2012, J VIROL, V86, P5791, DOI 10.1128/JVI.00201-12
Cowled C, 2014, BMC GENOMICS, V15, DOI 10.1186/1471-2164-15-682
Cowled C, 2012, DEV COMP IMMUNOL, V36, P657, DOI 10.1016/j.dci.2011.11.008
Cowled C, 2011, DEV COMP IMMUNOL, V35, P7, DOI 10.1016/j.dci.2010.07.006
Davenport B.J., 2004, BMC IMMUNOL, V5, pe23
Davis AD, 2013, J VIROL, V87, P9008, DOI 10.1128/JVI.03554-12
Dennehy JJ, 2014, ANNU REV MICROBIOL, V68, P117, DOI 10.1146/annurev-micro-091313-103436
Dennis Jr G., 2003, GENOME BIOL, V4, peP3
Diamond DL, 2010, PLOS PATHOG, V6, DOI 10.1371/

journal.ppat.1000719
DIAZ MO, 1994, GENOMICS, V22, P540, DOI 10.1006/geno.1994.1427
Dominguez SR, 2007, EMERG INFECT DIS, V13, P1295, DOI 10.3201/eid1309.070491
DOWNS WG, 1963, AM J TROP MED HYG, V12, P640, DOI 10.4269/ajtmh.1963.12.640
Easterbrook JD, 2007, P NATL ACAD SCI USA, V104, P15502, DOI 10.1073/pnas.0707453104
Evans VC, 2012, NAT METHODS, V9, P1207, DOI [10.1038/NMETH.2227, 10.1038/nmeth.2227]
Forster SC, 2013, BIOINFORMATICS, V29, P810, DOI 10.1093/bioinformatics/btt034
Frick WF, 2010, SCIENCE, V329, P679, DOI 10.1126/science.1188594
Fujii H, 2010, DEV COMP IMMUNOL, V34, P598, DOI 10.1016/j.dci.2010.01.004
Fulhorst CF, 1999, J INFECT DIS, V180, P966, DOI 10.1086/315029
Gard G., 2009, COMMUNICATION
Gargas A, 2009, MYCOTAXON, V108, P147, DOI 10.5248/108.147
Glenn TC, 2011, MOL ECOL RESOUR, V11, P759, DOI 10.1111/j.1755-0998.2011.03024.x
Grandvaux N, 2002, CURR OPIN INFECT DIS, V15, P259, DOI 10.1097/00001432-200206000-00008
GREER DL, 1981, AM J TROP MED HYG, V30, P653, DOI 10.4269/ajtmh.1981.30.653
Griseri P, 2014, J INTERF CYTOK RES, V34, P242, DOI 10.1089/jir.2013.0140
Haas BJ, 2013, NAT PROTOC, V8, P1494, DOI 10.1038/nprot.2013.084
Halpin K, 2000, J GEN VIROL, V81, P1927, DOI 10.1099/0022-1317-81-8-1927
Halpin K, 2011, AM J TROP MED HYG, V85, P946, DOI 10.4269/ajtmh.2011.10-0567
Heaton NS, 2011, TRENDS MICROBIOL, V19, P368, DOI 10.1016/j.tim.2011.03.007
HIGGINS DG, 1988, GENE, V73, P237, DOI 10.1016/0378-1119(88)90330-7
Holmes EC, 2002, VIROLOGY, V292, P247, DOI 10.1006/viro.2001.1271
Iha K, 2010, J VET MED SCI, V72, P217, DOI 10.1292/jvms.09-0050
Janardhana V, 2012, DEV COMP IMMUNOL, V36, P610, DOI 10.1016/j.dci.2011.11.001
Kosoy M, 2010, EMERG INFECT DIS, V16, P1875, DOI 10.3201/eid1612.100601
Krysko DV, 2011, TRENDS IMMUNOL, V32, P157, DOI 10.1016/j.it.2011.01.005
Kuzmin I.V., 2011, EMERG HLTH THREATS J, V4, pe7159
Lau SKP, 2005, P NATL ACAD SCI USA, V102, P14040, DOI 10.1073/pnas.0506735102
Leroy EM, 2005, NATURE, V438, P575, DOI 10.1038/438575a
Li B, 2011, BMC BIOINFORMATICS, V12, DOI 10.1186/1471-2105-12-323
Ligtenberg WPA, 2013, J BIOINF COMPUT BIOL, V11, DOI 10.1142/S0219720013500042
Luis AD, 2013, P ROY SOC B-BIOL SCI, V280, DOI 10.1098/rspb.2012.2753
Lutz MB, 1999, J IMMUNOL METHODS, V223, P77, DOI 10.1016/S0022-1759(98)00204-X

Medzhitov R, 1997, CELL, V91, P295, DOI 10.1016/S0092-8674(00)80412-2
Memish ZA, 2013, EMERG INFECT DIS, V19, P1819, DOI 10.3201/eid1911.131172
Morse SF, 2012, INFECT GENET EVOL, V12, P1717, DOI 10.1016/j.meegid.2012.06.009
Mühldorfer K, 2011, PLOS ONE, V6, DOI 10.1371/journal.pone.0029773
Mühldorfer K, 2011, BMC VET RES, V7, DOI 10.1186/1746-6148-7-61
Mühldorfer K, 2010, EMERG INFECT DIS, V16, P578, DOI 10.3201/eid1603.091035
Negredo A, 2011, PLOS PATHOG, V7, DOI 10.1371/journal.ppat.1002304
O'Shea TJ, 2014, EMERG INFECT DIS, V20, P741, DOI 10.3201/eid2005.130539
Omatsu T, 2007, COMP IMMUNOL MICROB, V30, P357, DOI 10.1016/j.cimid.2007.05.006
Papenfuss AT, 2012, BMC GENOMICS, V13, DOI 10.1186/1471-2164-13-261
PAUL BN, 1986, DEV COMP IMMUNOL, V10, P55, DOI 10.1016/0145-305X(86)90044-3
Paweska JT, 2012, PLOS ONE, V7, DOI 10.1371/journal.pone.0045479
Pearce EL, 2013, IMMUNITY, V38, P633, DOI 10.1016/j.immuni.2013.04.005
Pulliam JRC, 2012, J R SOC INTERFACE, V9, P89, DOI 10.1098/rsif.2011.0223
Quan PL, 2013, P NATL ACAD SCI USA, V110, P8194, DOI 10.1073/pnas.1303037110
Reeves WK, 2006, J VECTOR ECOL, V31, P386, DOI 10.3376/1081-1710(2006)31[386:SSOEFF]2.0.CO;2
Rodriguez JJ, 2004, VIRAL IMMUNOL, V17, P210, DOI 10.1089/0882824041310568
Rodriguez JJ, 2004, J VIROL, V78, P5358, DOI 10.1128/JVI.78.10.5358-5367.2004
Rodriguez JJ, 2002, J VIROL, V76, P11476, DOI 10.1128/JVI.76.22.11476-11483.2002
RUPPRECHT CE, 1995, EMERG INFECT DIS, V1, P107, DOI 10.3201/eid0104.950401
Rupprecht CE, 2011, CURR OPIN VIROL, V1, P662, DOI 10.1016/j.coviro.2011.10.014
SARKAR SK, 1991, DEV COMP IMMUNOL, V15, P423, DOI 10.1016/0145-305X(91)90034-V
Schountz T, 2007, P NATL ACAD SCI USA, V104, P15496, DOI 10.1073/pnas.0707454104
Schountz T, 2014, J VIROL, V88, P8319, DOI 10.1128/JVI.00004-14
Schountz T, 2013, BMC IMMUNOL, V14, DOI 10.1186/1471-2172-14-18
Schulz MH, 2012, BIOINFORMATICS, V28, P1086, DOI 10.1093/bioinformatics/bts094
Shaw ML, 2009, VIRUSES-BASEL, V1, P1190, DOI 10.3390/v1031190
Shaw ML, 2004, J VIROL, V78, P5633, DOI 10.1128/JVI.78.11.5633-5641.2004
Shaw TI, 2012, PLOS ONE, V7, DOI 10.1371/journal.pone.0048472
Sheppard P, 2003, NAT IMMUNOL, V4, P63, DOI 10.1038/ni873
Takeda K, 2003, ANNU REV IMMUNOL, V21, P335, DOI 10.1146/

annurev.immunol.21.120601.141126
   Tikasingh E., 2011, COMMUNICATION
   Towner JS, 2007, PLOS ONE, V2, DOI 10.1371/journal.pone.0000764
   Turmelle AS, 2010, J GEN VIROL, V91, P2360, DOI 10.1099/
vir.0.020073-0
   Valmas C, 2010, PLOS PATHOG, V6, DOI 10.1371/journal.ppat.1000721
   Vastrik I, 2007, GENOME BIOL, V8, DOI 10.1186/gb-2007-8-3-r39
   Virtue ER, 2011, PLOS ONE, V6, DOI 10.1371/journal.pone.0022488
   Wang LF, 2011, CURR OPIN VIROL, V1, P649, DOI 10.1016/
j.coviro.2011.10.013
   Wang Lin-Fa, 2009, Virologica Sinica, V24, P93, DOI 10.1007/
s12250-009-3032-5
   Weitzman MD, 2014, CELL HOST MICROBE, V15, P283, DOI 10.1016/
j.chom.2014.02.010
   Wibbelt G, 2010, BIOL LETTERS, V6, P438, DOI 10.1098/
rsbl.2010.0267
   Wilhelms DB, 2014, J NEUROSCI, V34, P11684, DOI 10.1523/
JNEUROSCI.1838-14.2014
   Wood JLN, 2012, PHILOS T R SOC B, V367, P2881, DOI 10.1098/
rstb.2012.0228
   Wynne JW, 2014, GENOME BIOL, V15, DOI 10.1186/s13059-014-0532-x
   Wynne JW, 2013, PLOS ONE, V8, DOI 10.1371/journal.pone.0052930
   Xiao JP, 2011, ARCH VIROL, V156, P1415, DOI 10.1007/
s00705-011-1011-2
   YANAGIHARA R, 1985, J VIROL, V55, P34, DOI 10.1128/
JVI.55.1.34-38.1985
   Yanagihara R, 2014, VIRUS RES, V187, P6, DOI 10.1016/
j.virusres.2013.12.038
   Yuan LH, 2014, VIRUSES-BASEL, V6, P2138, DOI 10.3390/v6052138
   Zhang GJ, 2013, SCIENCE, V339, P456, DOI 10.1126/science.1230835
   Zhou P, 2013, DEV COMP IMMUNOL, V40, P240, DOI 10.1016/
j.dci.2013.03.006
   Zhou P, 2011, PLOS ONE, V6, DOI 10.1371/journal.pone.0025385
   Zhou P, 2011, J IMMUNOL, V186, P3138, DOI 10.4049/
jimmunol.1003115
NR 128
TC 72
Z9 83
U1 1
U2 99
PU MDPI
PI BASEL
PA ST ALBAN-ANLAGE 66, CH-4052 BASEL, SWITZERLAND
EI 1999-4915
J9 VIRUSES-BASEL
JI Viruses-Basel
PD DEC
PY 2014
VL 6
IS 12
BP 4880
EP U410
DI 10.3390/v6124880
PG 23

WC Virology
WE Science Citation Index Expanded (SCI-EXPANDED)
SC Virology
GA AX3JA
UT WOS:000346834500005
PM 25494448
OA gold, Green Published, Green Submitted
DA 2024-10-21
ER

PT J
AU Yang, DB
Xu, YC
Wang, DH
AF Yang, Deng-Bao
Xu, Yan-Chao
Wang, De-Hua
TI Partial removal of brown adipose tissue enhances humoral immunity in
warm-acclimated Mongolian gerbils (<i>Meriones unguiculatus</i>)
SO GENERAL AND COMPARATIVE ENDOCRINOLOGY
LA English
DT Article
DE Brown adipose tissue; Immunity; Cold acclimation; Thermoregulation;
Trade-offs; Mongolian gerbil
ID UNCOUPLING PROTEIN EXPRESSION; NONSHIVERING THERMOGENESIS; BODY-MASS;
SEASONAL-CHANGES; METABOLIC-RATE; COLD-STRESS; FOOD-INTAKE; DJUNGARIAN
HAMSTERS; AMBIENT-TEMPERATURE; ENERGY-BALANCE
AB Temperate rodent species experience marked seasonal fluctuations in environmental temperatures. High thermoregulatory demands during winter usually weaken immune function. Brown adipose tissue (BAT) plays a crucial role in adaptive thermoregulatory process. Thus, we proposed the hypothesis that BAT might participate in the regulation of seasonal changes in immune function. The present study examined the trade-off between thermoregulation and immune function and the potential role of BAT in regulating seasonal changes in immune function in Mongolian gerbils. Specifically, surgical removal of interscapular BAT (34% of total BAT) was performed in male gerbils, and subsequently acclimated to either warm (23 +/- 1 degrees C) or cold (4 +/- 1 degrees C) conditions. Gerbils were then challenged with innocuous antigens and the immune responses were measured. Resting metabolic rate (RMR) and nonshivering thermogenesis (NST) were increased under cold conditions. However, the cost of thermoregulation during cold acclimation did not suppress T-cell mediated immunity and humoral immunity or decrease spleen mass, thymus mass and white blood cells. Partial removal of BAT significantly enhanced humoral immunity in warm-acclimated, but not in cold-acclimated gerbils. T-cell mediated immunity, white blood cells and immune organs were not affected by BAT removal under both warm and cold conditions. Collectively, our results imply that BAT has a suppressive effect on humoral immunity in warm-acclimated

gerbils and differential effects of BAT on humoral immunity under different temperatures (e.g., summer and winter) might be benefit to their survival. (C) 2011 Elsevier Inc. All rights reserved.
C1 [Yang, Deng-Bao; Xu, Yan-Chao; Wang, De-Hua] Chinese Acad Sci, Inst Zool, State Key Lab Integrated Management Pest Insects, Beijing 100101, Peoples R China.
[Yang, Deng-Bao; Xu, Yan-Chao] Chinese Acad Sci, Grad Sch, Beijing 100049, Peoples R China.
C3 Chinese Academy of Sciences; Institute of Zoology, CAS; Chinese Academy
of Sciences; University of Chinese Academy of Sciences, CAS
RP Wang, DH (corresponding author), Chinese Acad Sci, Inst Zool, State Key Lab Integrated Management Pest Insects, 1 Beichen W Rd, Beijing 100101, Peoples R China.
EM wangdh@ioz.ac.cn
RI Xu, Yanchao/KQU-4135-2024; yang, dengbao/KIG-4663-2024; Dehua, Wang/AFL-4468-2022
FU Ministry of Science and Technology [2007BC109103]; Chinese Academy of
Sciences [KSCX2-EW-N-005]; National Natural Science Foundation of China
[31071930]
FX Many thanks to the two anonymous reviewers for their comments and
suggestions. We thank all the members of Animal Physiological Ecology
Group for their assistance. This study was supported by Grants from the
Ministry of Science and Technology (2007BC109103), Chinese Academy of
Sciences (KSCX2-EW-N-005), and National Natural Science Foundation of
China (31071930).
CR Abbas A.K., 2000, CELL MOL IMMUNOL, V4th, P161
Abelenda M, 2003, J THERM BIOL, V28, P525, DOI 10.1016/S0306-4565(03)00053-6
Ahima RS, 1996, NATURE, V382, P250, DOI 10.1038/382250a0
Arvaniti K, 1998, ENDOCRINOLOGY, V139, P4000, DOI 10.1210/en.139.9.4000
ATANASSOV CL, 1995, COMP BIOCHEM PHYS C, V112, P93, DOI 10.1016/0742-8413(95)00000-3
BHATNAGAR S, 1995, PHYSIOL BEHAV, V57, P633, DOI 10.1016/0031-9384(94)00161-8
BIANCO AC, 1987, J CLIN INVEST, V79, P295, DOI 10.1172/JCI112798
Bowers SL, 2008, BRAIN BEHAV IMMUN, V22, P105, DOI 10.1016/j.bbi.2007.07.012
Bronson Franklin H., 1994, P541
Calder PC, 2002, BRIT J NUTR, V88, pS165, DOI 10.1079/BJN2002682
Cancello R, 1998, ENDOCRINOLOGY, V139, P4747, DOI 10.1210/en.139.11.4747
Cannon B, 2004, PHYSIOL REV, V84, P277, DOI 10.1152/physrev.00015.2003
Chen WH, 2002, GEN COMP ENDOCR, V126, P90, DOI 10.1006/gcen.2001.7772
Chen Z. Z., 1988, Inner Mongolia grassland ecosystem research

station, V3, P13
Chi QS, 2011, J COMP PHYSIOL B, V181, P91, DOI 10.1007/s00360-010-0506-6
Cichon M, 2002, P ROY SOC B-BIOL SCI, V269, P1493, DOI 10.1098/rspb.2002.2054
Commins SP, 1999, ENDOCRINOLOGY, V140, P292, DOI 10.1210/en.140.1.292
Cyr NE, 2007, GEN COMP ENDOCR, V151, P82, DOI 10.1016/j.ygcen.2006.12.003
Dallman MF, 2004, ENDOCRINOLOGY, V145, P2633, DOI 10.1210/en.2004-0037
de Bellocq JG, 2006, COMP BIOCHEM PHYS A, V145, P554, DOI 10.1016/j.cbpa.2006.08.045
de Jesus LA, 2001, J CLIN INVEST, V108, P1379, DOI 10.1172/JCI200113803
Demas GE, 2005, P ROY SOC B-BIOL SCI, V272, P1845, DOI 10.1098/rspb.2005.3126
Demas GE, 1998, J BIOL RHYTHM, V13, P253, DOI 10.1177/074873098129000093
Demas GE, 1997, AM J PHYSIOL-REG I, V272, pR1762, DOI 10.1152/ajpregu.1997.272.6.R1762
Demas GE, 2003, P ROY SOC B-BIOL SCI, V270, P905, DOI 10.1098/rspb.2003.2341
Demas GE, 2004, HORM BEHAV, V45, P173, DOI 10.1016/j.yhbeh.2003.11.002
Demas GE, 1997, AM J PHYSIOL-REG I, V273, pR1631, DOI 10.1152/ajpregu.1997.273.5.R1631
Demas GE, 2010, GEN COMP ENDOCR, V166, P314, DOI 10.1016/j.ygcen.2009.10.011
Derting TL, 2003, PHYSIOL BIOCHEM ZOOL, V76, P744, DOI 10.1086/375662
Doufas Anthony G, 2003, Best Pract Res Clin Anaesthesiol, V17, P535, DOI 10.1016/S1521-6896(03)00052-1
Drahota Z., 1970, BROWN ADIPOSE TISSUE, P25
Dratman MB, 1996, THYROID, V6, P639, DOI 10.1089/thy.1996.6.639
Drazen DL, 2001, ENDOCRINOLOGY, V142, P2768, DOI 10.1210/en.142.7.2768
Drazen DL, 2000, AM J PHYSIOL-REG I, V278, pR1401, DOI 10.1152/ajpregu.2000.278.6.R1401
Enerback S, 1997, NATURE, V387, P90, DOI 10.1038/387090a0
Friedman JM, 1998, NATURE, V395, P763, DOI 10.1038/27376
HAMMOND KA, 1992, PHYSIOL ZOOL, V65, P952, DOI 10.1086/physzool.65.5.30158552
HAYWARD JS, 1972, CAN J PHYSIOL PHARM, V50, P168, DOI 10.1139/y72-024
HELDMAIER G, 1982, J COMP PHYSIOL, V149, P1, DOI 10.1007/BF00735708
HELDMAIER G, 1982, INT J BIOMETEOROL, V26, P339, DOI 10.1007/BF02219505
HELDMAIER G, 1985, J COMP PHYSIOL B, V156, P237, DOI 10.1007/BF00695778
Himms-Hagen J., 1986, BROWN ADIPOSE TISSUE, P214
Himms-Hagen J., 1972, HDB EXP PHARMAK, P363
HORWITZ BA, 1972, EXPERIENTIA, V28, P284, DOI 10.1007/BF01928691

HULL D, 1965, J PHYSIOL-LONDON, V181, P449, DOI 10.1113/jphysiol.1965.sp007773
JANKOVIC BD, 1975, IMMUNOLOGY, V28, P597
JANSKY L, 1966, FED PROC, V25, P1297
Klingenspor M, 2003, EXP PHYSIOL, V88, P141, DOI 10.1113/eph8802508
Klingenspor M, 1996, BIOCHEM J, V316, P607, DOI 10.1042/bj3160607
KONARZEWSKI M, 1994, PHYSIOL ZOOL, V67, P1186, DOI 10.1086/physzool.67.5.30163889
Król E, 2007, J ENDOCRINOL, V192, P271, DOI 10.1677/JOE-06-0074
Ksiazek A, 2004, PHYSIOL BIOCHEM ZOOL, V77, P890, DOI 10.1086/425190
Ksiazek A, 2003, P ROY SOC B-BIOL SCI, V270, P2025, DOI 10.1098/rspb.2003.2474
Li XS, 2007, PHYSIOL BIOCHEM ZOOL, V80, P326, DOI 10.1086/513189
Li XS, 2005, HORM BEHAV, V48, P321, DOI 10.1016/j.yhbeh.2005.04.004
Li XS, 2005, J COMP PHYSIOL B, V175, P593, DOI 10.1007/s00360-005-0022-2
Lochmiller RL, 2000, OIKOS, V88, P87, DOI 10.1034/j.1600-0706.2000.880110.x
Lord GM, 1998, NATURE, V394, P897, DOI 10.1038/29795
Martin LB, 2005, GEN COMP ENDOCR, V140, P126, DOI 10.1016/j.ygcen.2004.10.010
Martin LB, 2008, J COMP PHYSIOL B, V178, P623, DOI 10.1007/s00360-008-0253-0
Martin LB, 2008, PHILOS T R SOC B, V363, P321, DOI 10.1098/rstb.2007.2142
MCDEVITT RM, 1994, PHYSIOL ZOOL, V67, P1117, DOI 10.1086/physzool.67.5.30163885
McEwen BS, 1997, BRAIN RES REV, V23, P79, DOI 10.1016/S0165-0173(96)00012-4
MORISCOT A, 1993, AM J PHYSIOL, V265, pE81, DOI 10.1152/ajpendo.1993.265.1.E81
MURPHY HM, 1992, PEPTIDES, V13, P373, DOI 10.1016/0196-9781(92)90123-K
Nelson R.J., 2002, SEASONAL PATTERNS ST
Nelson RJ, 1996, Q REV BIOL, V71, P511, DOI 10.1086/419555
Nelson RJ, 2004, TRENDS IMMUNOL, V25, P187, DOI 10.1016/j.it.2004.02.001
Rich EL, 2005, AM J PHYSIOL-REG I, V288, pR1628, DOI 10.1152/ajpregu.00484.2004
Romero LM, 2004, TRENDS ECOL EVOL, V19, P249, DOI 10.1016/j.tree.2004.03.008
Sapolsky RM, 2000, ENDOCR REV, V21, P55, DOI 10.1210/er.21.1.55
Scarpace PJ, 1997, AM J PHYSIOL-ENDOC M, V273, pE226, DOI 10.1152/ajpendo.1997.273.1.E226
Sheldon BC, 1996, TRENDS ECOL EVOL, V11, P317, DOI 10.1016/0169-5347(96)10039-2
Shephard RJ, 1998, CAN J PHYSIOL PHARM, V76, P828, DOI 10.1139/cjpp-76-9-828
SIDKY YA, 1969, P SOC EXP BIOL MED, V132, P760
SIDKY YA, 1972, IMMUNOL COMMUN, V1, P579, DOI 10.3109/08820137209022966

Smits JE, 1999, FUNCT ECOL, V13, P567, DOI 10.1046/j.1365-2435.1999.00338.x
Stearns S.C., 1992, pi
STRACK AM, 1995, AM J PHYSIOL-REG I, V268, pR183, DOI 10.1152/ajpregu.1995.268.1.R183
Svensson E, 1998, FUNCT ECOL, V12, P912, DOI 10.1046/j.1365-2435.1998.00271.x
Van Sant MJ, 2008, PHYSIOL BIOCHEM ZOOL, V81, P605, DOI 10.1086/588175
WADE GN, 1992, NEUROSCI BIOBEHAV R, V16, P235, DOI 10.1016/S0149-7634(05)80183-6
Walker E.P., 1968, Mammals of the world, V2d
Wang D.-H, 2003, Comparative Biochemistry and Physiology Part A Molecular & Integrative Physiology, V134A, pS96
Wang DH, 2000, ACTA THERIOL, V45, P183, DOI 10.4098/AT.arch.00-21
Wunder Bruce A., 1996, P131
Xu DL, 2011, PHYSIOL BIOCHEM ZOOL, V84, P87, DOI 10.1086/657687
Xu DL, 2010, COMP BIOCHEM PHYS A, V155, P25, DOI 10.1016/j.cbpa.2009.09.003
Zhang ZQ, 2007, COMP BIOCHEM PHYS A, V148, P346, DOI 10.1016/j.cbpa.2007.05.012
Zhang Zhiqiang, 2006, Acta Theriologica Sinica, V26, P338
Zhao ZJ, 2006, J THERM BIOL, V31, P323, DOI 10.1016/j.jtherbio.2006.01.001
NR 91
TC 5
Z9 7
U1 0
U2 12
PU ACADEMIC PRESS INC ELSEVIER SCIENCE
PI SAN DIEGO
PA 525 B ST, STE 1900, SAN DIEGO, CA 92101-4495 USA
SN 0016-6480
EI 1095-6840
J9 GEN COMP ENDOCR
JI Gen. Comp. Endocrinol.
PD JAN 1
PY 2012
VL 175
IS 1
BP 144
EP 152
DI 10.1016/j.ygcen.2011.10.012
PG 9
WC Endocrinology & Metabolism; Zoology
WE Science Citation Index Expanded (SCI-EXPANDED)
SC Endocrinology & Metabolism; Zoology
GA 875WL
UT WOS:000299065800017
PM 22080042
DA 2024-10-21
ER

PT J

AU Nabi, G
Wang, Y
Lü, L
Jiang, C
Ahmad, S
Wu, YF
Li, DM
AF Nabi, Ghulam
Wang, Yang
Lu, Liang
Jiang, Chuan
Ahmad, Shahid
Wu, Yuefeng
Li, Dongming
TI Bats and birds as viral reservoirs: A physiological and ecological
perspective
SO SCIENCE OF THE TOTAL ENVIRONMENT
LA English
DT Article
DE Bird; Bat; Flight; Physiology; Immunology; Viral transmission
ID H5N1 INFLUENZA-VIRUSES; GENOME SIZE; BODY-SIZE; WILD BIRDS; CELL-SIZE;
C-VALUE; EVOLUTION; CORONAVIRUSES; URBANIZATION; EPIDEMIOLOGY
AB The birds (class Aves) and bats (order Chiroptera, class Mammalia) are well known natural reservoirs of a diverse range of viruses, including some zoonoses. The only extant volant vertebrates, bats and birds have undergone dramatic adaptive radiations that have allowed them to occupy diverse ecological niches and colonize most of the planet. However, few studies have compared the physiology and ecology of these ecologically, and medically, important taxa. Here, we review convergent traits in the physiology, immunology, flight-related ecology of birds and bats that might enable these taxa to act as viral reservoirs and asymptomatic carriers. Many species of birds and bats are well adapted to urban environments and may host more zoonotic pathogens than species that do not colonize anthropogenic habitats. These convergent traits in birds and bats and their ecological interactions with domestic animals and humans increase the potential risk of viral spillover transmission and facilitate the emergence of novel viruses that most likely sources of zoonoses with the potential to cause global pandemics. (C) 2020 Elsevier B.V. All rights reserved.
C1 [Nabi, Ghulam; Wang, Yang; Lu, Liang; Jiang, Chuan; Ahmad, Shahid; Wu, Yuefeng; Li, Dongming] Hebei Normal Univ, Coll Life Sci, Key Lab Anim Physiol Biochem & Mol Biol Hebei Pro, Shijiazhuang, Hebei, Peoples R China.
C3 Hebei Normal University
RP Li, DM (corresponding author), Hebei Normal Univ, Coll Life Sci, 20 East Rd 2nd South Ring, Shijiazhuang 050024, Hebei, Peoples R China.
EM lidongming@hebtu.edu.cn
RI Nabi, Ghulam/AGQ-2183-2022; ahmad, shahid/AAE-3976-2022; zhang, ying/HJB-1230-2022

OI Nabi, Ghulam/0000-0001-5256-0083; Li, Dongming/
0000-0003-2759-3435; ,
shahid/0000-0002-8015-7037; Wu, Yuefeng/0000-0001-5121-0049
FU National Natural Science Foundation of China (NSFC) [31672292,
31971413,
31770445]; Ministry of Ecology and Environment of China
FX We appreciate the valuable comments from three anonymous
reviewers for
the improvement of our original manuscript. This study was
supported by
the National Natural Science Foundation of China (NSFC, 31672292,
31971413) to D.M.L., NSFC (31770445) to Y.F.W., the biodiversity
investigation, observation, and assessment program (2019-2023) of
Ministry of Ecology and Environment of China to D.M.L.
CR Abulreesh Hussein H., 2007, Ringing & Migration, V23, P193
Afelt A, 2018, FRONT MICROBIOL, V9, DOI 10.3389/fmicb.2018.00702
Aguirre AA, 2020, WORLD MED HEALTH POL, V12, P256, DOI 10.1002/
wmh3.348
Alexander DJ, 2007, VACCINE, V25, P5637, DOI 10.1016/
j.vaccine.2006.10.051
Allocati N, 2016, CELL DEATH DISCOV, V2, DOI 10.1038/
cddiscovery.2016.48
Anderson D.E., 2020, BIORXIV
[Anonymous], 2016, BATS ANTHROPOCENE CO
[Anonymous], 2018, DIVERSITY-BASEL, DOI DOI 10.3390/d10030049
[Anonymous], 2017, F1000RESEARCH, DOI DOI 10.12688/
F1000RESEARCH.10416.1
AUSTAD SN, 1991, J GERONTOL, V46, pB47, DOI 10.1093/geronj/
46.2.B47
Bahl J, 2013, PLOS PATHOG, V9, DOI 10.1371/journal.ppat.1003570
Baker ML, 2013, ZOONOSES PUBLIC HLTH, V60, P104, DOI 10.1111/
j.1863-2378.2012.01528.x
Baker ML, 2010, IMMUNOGENETICS, V62, P173, DOI 10.1007/
s00251-010-0425-4
Banerjee A, 2020, FRONT IMMUNOL, V11, DOI 10.3389/
fimmu.2020.00026
Brook CE, 2015, TRENDS MICROBIOL, V23, P172, DOI 10.1016/
j.tim.2014.12.004
Brown JH, 2014, J BIOGEOGR, V41, P8, DOI 10.1111/jbi.12228
Brunotte L, 2016, CURR OPIN VIROL, V16, P114, DOI 10.1016/
j.coviro.2016.02.003
Bui C, 2016, TRANSBOUND EMERG DIS, V63, P602, DOI 10.1111/
tbed.12327
Butchart SHM, 2008, BIRD CONSERV INT, V18, pS245, DOI 10.1017/
S095927090800035X
Calisher CH, 2006, CLIN MICROBIOL REV, V19, P531, DOI 10.1128/
CMR.00017-06
Camargo PHSA, 2016, WILSON J ORNITHOL, V128, P903
Cash R., 2020, LANCET, V5, P2020
Caviedes-Vidal E, 2007, P NATL ACAD SCI USA, V104, P19132, DOI
10.1073/pnas.0703159104
CDC, 2019, SPEC DEAD BIRDS WHIC
Chamings A, 2018, SCI REP-UK, V8, DOI 10.1038/s41598-018-24407-x
Chan JFW, 2015, CURR OPIN VIROL, V10, P63, DOI 10.1016/

j.coviro.2015.01.006
Chan JFW, 2013, TRENDS MICROBIOL, V21, P544, DOI 10.1016/j.tim.2013.05.005
Chen H, 2006, P NATL ACAD SCI USA, V103, P2845, DOI 10.1073/pnas.0511120103
Chen LH, 2014, DATABASE-OXFORD, DOI 10.1093/database/bau021
Chu DKW, 2011, J VIROL, V85, P12815, DOI 10.1128/JVI.05838-11
Costantini D, 2008, ECOL LETT, V11, P1238, DOI 10.1111/j.1461-0248.2008.01246.x
Cui J, 2019, NAT REV MICROBIOL, V17, P181, DOI 10.1038/s41579-018-0118-9
Drexler JF, 2012, NAT COMMUN, V3, DOI 10.1038/ncomms1796
Duchamp JE, 2008, LANDSCAPE ECOL, V23, P849, DOI 10.1007/s10980-008-9241-8
FENTON M B, 1976, Biotropica, V8, P104, DOI 10.2307/2989629
Figuerola J, 2000, EVOL ECOL, V14, P143, DOI 10.1023/A:1011009419264
GAMMELIN M, 1990, MOL BIOL EVOL, V7, P194
Georgopoulou Ioanna, 2008, Vet Ital, V44, P671
Goldingay RL, 2009, WILDLIFE RES, V36, P394, DOI 10.1071/WR08172
Gregory TR, 2009, P R SOC B, V276, P3753, DOI 10.1098/rspb.2009.1004
Gregory TR, 2002, EVOLUTION, V56, P121
Gregory TR, 2001, BLOOD CELL MOL DIS, V27, P830, DOI 10.1006/bcmd.2001.0457
Guernier V, 2004, PLOS BIOL, V2, P740, DOI 10.1371/journal.pbio.0020141
Guigueno MF, 2019, COMP BIOCHEM PHYS A, V235, P193, DOI 10.1016/j.cbpa.2019.06.003
Hassell JM, 2017, TRENDS ECOL EVOL, V32, P55, DOI 10.1016/j.tree.2016.09.012
Hayman DTS, 2016, ANNU REV VIROL, V3, P77, DOI 10.1146/annurev-virology-110615-042203
Healy K, 2014, P ROY SOC B-BIOL SCI, V281, DOI 10.1098/rspb.2014.0298
Hill NJ, 2016, ECOL LETT, V19, P915, DOI 10.1111/ele.12629
Hill NJ, 2012, VECTOR-BORNE ZOONOT, V12, P243, DOI 10.1089/vbz.2010.0246
Holmes DJ, 2003, EXP GERONTOL, V38, P1365, DOI 10.1016/j.exger.2003.10.018
Hughes LA, 2009, EMERG INFECT DIS, V15, P1091, DOI 10.3201/eid1507.090067
Hulse-Post DJ, 2005, P NATL ACAD SCI USA, V102, P10682, DOI 10.1073/pnas.0504662102
Ibáñez C, 2001, P NATL ACAD SCI USA, V98, P9700, DOI 10.1073/pnas.171140598
Isaksson C., 2018, BIRD SPECIES FASCINA
Johnson MTJ, 2017, SCIENCE, V358, DOI 10.1126/science.aam8327
Kaiser P, 2010, AVIAN PATHOL, V39, P309, DOI 10.1080/03079457.2010.508777
Kapusta A, 2017, P NATL ACAD SCI USA, V114, pE1460, DOI 10.1073/pnas.1616702114
Kirby JS, 2008, BIRD CONSERV INT, V18, pS49, DOI 10.1017/S0959270908000439

Krauel J.J., 2013, BAT EVOLUTION ECOLOG
Kuiken T, 2013, P ROY SOC B-BIOL SCI, V280, DOI 10.1098/rspb.2013.0990
Laurin M, 2004, SYST BIOL, V53, P594, DOI 10.1080/10635150490445706
LINDSTEDT SL, 1981, Q REV BIOL, V56, P1, DOI 10.1086/412080
Lucas T.C., 2016, THESIS
Lycett SJ, 2019, PHILOS T R SOC B, V374, DOI 10.1098/rstb.2018.0257
MacGregor-Fors I, 2012, STUD AVIAN BIOL, V45, P33
Magle SB, 2012, BIOL CONSERV, V155, P23, DOI 10.1016/j.biocon.2012.06.018
MAHASE E, 2020, BMJ-BRIT MED J, V369, DOI DOI 10.1136/BMJ.M1327
MCCRACKEN GF, 1991, ETHOLOGY, V89, P305
McGuire LP, 2009, J MAMMAL, V90, P1290, DOI 10.1644/09-MAMM-S-084R.1
MEDELLIN RA, 1988, J MAMMAL, V69, P841, DOI 10.2307/1381644
Mikula P, 2016, MAMMAL REV, V46, P160, DOI 10.1111/mam.12060
Mildenstein T., 2016, BATS ANTHROPOCENE CO
Milek J, 2018, J VET RES, V62, P249, DOI 10.2478/jvetres-2018-0035
Minias P, 2016, CURR ZOOL, V62, P79, DOI 10.1093/cz/zow034
Mollentze N, 2020, P NATL ACAD SCI USA, V117, P9423, DOI 10.1073/pnas.1919176117
Morse SS, 2012, LANCET, V380, P1956, DOI 10.1016/S0140-6736(12)61684-5
Munshi-South J, 2010, AGEING RES REV, V9, P12, DOI 10.1016/j.arr.2009.07.006
Myczko L, 2017, IBIS, V159, P235, DOI 10.1111/ibi.12434
Myhrvold N.P., 2015, ECOLOGY, V96, P3109
Nabi G, 2020, ENVIRON RES, V185, DOI 10.1016/j.envres.2020.109460
Newton I, 2008, MIGRATION ECOLOGY OF BIRDS, P1
Norberg U M., 1990, Vertebrate Flight: Mechanics, Physiology, Morphology, Ecology and Evolution, Vvol 27
Olival KJ, 2017, NATURE, V546, P646, DOI 10.1038/nature22975
Olsen B, 2006, SCIENCE, V312, P384, DOI 10.1126/science.1122438
Organ CL, 2007, NATURE, V446, P180, DOI 10.1038/nature05621
Pavlovich SS, 2018, CELL, V173, P1098, DOI 10.1016/j.cell.2018.03.070
Perrella DF, 2020, J AVIAN BIOL, V51, DOI 10.1111/jav.02277
Plowright RK, 2015, P ROY SOC B-BIOL SCI, V282, DOI 10.1098/rspb.2014.2124
PRINZINGER R, 1993, COMP BIOCHEM PHYS A, V105, P609, DOI 10.1016/0300-9629(93)90260-B
Prosser D.J., 2013, VIRAL INFECT GLOBAL, P151
Quan PL, 2013, P NATL ACAD SCI USA, V110, P8194, DOI 10.1073/pnas.1303037110
Reed Kurt D, 2003, Clin Med Res, V1, P5
Satterfield DA, 2018, PHILOS T R SOC B, V373, DOI 10.1098/rstb.2017.0094
Satu H., 2017, INFECT ECOL EPIDEMIO, V7
Schat KA., 2014, AVIAN IMMUNOLOGY, V2
Scholtissek C, 1995, VIRUS GENES, V11, P209, DOI 10.1007/BF01728660

Sehgal RNM, 2010, J EXP BIOL, V213, P955, DOI 10.1242/jeb.037663
Seo SH, 2002, J VIROL, V76, P4886, DOI 10.1128/JVI.76.10.4886-4890.2002
Song SJ, 2020, MBIO, V11, DOI 10.1128/mBio.02901-19
Speakman JR, 2005, J EXP BIOL, V208, P1717, DOI 10.1242/jeb.01556
Sturm-Ramirez KM, 2004, J VIROL, V78, P4892, DOI 10.1128/JVI.78.9.4892-4901.2004
Teeling EC, 2018, ANNU REV ANIM BIOSCI, V6, P23, DOI 10.1146/annurev-animal-022516-022811
THOMAS SP, 1972, J EXP BIOL, V57, P317
TIERSCH TR, 1991, J HERED, V82, P363, DOI 10.1093/oxfordjournals.jhered.a111105
Tong SX, 2013, PLOS PATHOG, V9, DOI 10.1371/journal.ppat.1003657
Van Cakenberghe V., 2019, HDB MAMMALS WORLD, V9, P875
Vanderven HA, 2012, MOL IMMUNOL, V51, P316, DOI 10.1016/j.molimm.2012.03.034
Venkatesh D, 2018, J VIROL, V92, DOI 10.1128/JVI.00433-18
Verhagen JH, 2015, SCIENCE, V347, P616, DOI 10.1126/science.aaa6724
Veselka N, 2010, NATURE, V463, P939, DOI 10.1038/nature08737
Voigt CC., 2016, BATS ANTHROPOCENE CO
Walsh MG, 2017, SCI REP-UK, V7, DOI 10.1038/s41598-017-08065-z
Wang D., 2020, COLD SPRING HARB PER
WARR GW, 1995, IMMUNOL TODAY, V16, P392, DOI 10.1016/0167-5699(95)80008-5
WEBSTER RG, 1992, MICROBIOL REV, V56, P152, DOI 10.1128/MMBR.56.1.152-179.1992
Weiss P, 2020, LANCET, V395, P1014, DOI 10.1016/S0140-6736(20)30633-4
Wigley P., 2017, ELS, P1, DOI [DOI 10.1002/9780470015902.A, 10.1002/9780470015902.a]
Wilkinson GS, 2002, AGING CELL, V1, P124, DOI 10.1046/j.1474-9728.2002.00020.x
Wong ACP, 2019, VIRUSES-BASEL, V11, DOI 10.3390/v11020174
Woo PCY, 2012, J VIROL, V86, P3995, DOI 10.1128/JVI.06540-11
Woolhouse MEJ, 2018, SCI DATA, V5, DOI 10.1038/sdata.2018.17
World Health Organization, 2020, WHO consolidated guidelines on tuberculosis. Module 4: treatment-drug-resistant tuberculosis treatmentM
Zhang GJ, 2014, SCIENCE, V346, P1311, DOI 10.1126/science.1251385
Zhang GJ, 2013, SCIENCE, V339, P456, DOI 10.1126/science.1230835
Zhang XY, 2017, BIOL REV, V92, P2144, DOI 10.1111/brv.12325
Zhou B, 2014, PLOS PATHOG, V10, DOI 10.1371/journal.ppat.1004420
Zhou P, 2020, NATURE, V579, P270, DOI [10.1038/s41586-020-2012-7, 10.1038/s41586-020-2951-z]
Zhu XY, 2013, P NATL ACAD SCI USA, V110, P1458, DOI 10.1073/pnas.1218509110
NR 128
TC 24
Z9 24
U1 5
U2 83
PU ELSEVIER
PI AMSTERDAM

PA RADARWEG 29, 1043 NX AMSTERDAM, NETHERLANDS
SN 0048-9697
EI 1879-1026
J9 SCI TOTAL ENVIRON
JI Sci. Total Environ.
PD FEB 1
PY 2021
VL 754
AR 142372
DI 10.1016/j.scitotenv.2020.142372
PG 6
WC Environmental Sciences
WE Science Citation Index Expanded (SCI-EXPANDED)
SC Environmental Sciences & Ecology
GA OX9WY
UT WOS:000593907600006
PM 33254850
OA Green Published
DA 2024-10-21
ER

PT J
AU Becker, DJ
   Czirják, GA
   Volokhov, DV
   Bentz, AB
   Carrera, JE
   Camus, MS
   Navara, KJ
   Chizhikov, VE
   Fenton, MB
   Simmons, NB
   Recuenco, SE
   Gilbert, AT
   Altizer, S
   Streicker, DG
AF Becker, Daniel J.
   Czirjak, Gabor A.
   Volokhov, Dmitriy V.
   Bentz, Alexandra B.
   Carrera, Jorge E.
   Camus, Melinda S.
   Navara, Kristen J.
   Chizhikov, Vladimir E.
   Fenton, M. Brock
   Simmons, Nancy B.
   Recuenco, Sergio E.
   Gilbert, Amy T.
   Altizer, Sonia
   Streicker, Daniel G.
TI Livestock abundance predicts vampire bat demography, immune profiles and
   bacterial infection risk
SO PHILOSOPHICAL TRANSACTIONS OF THE ROYAL SOCIETY B-BIOLOGICAL

SCIENCES
LA English
DT Article
DE agriculture; Bartonella; ecoimmunology; haemoplasmas; resource
   provisioning; supplemental feeding
ID DESMODUS-ROTUNDUS; BARTONELLA-HENSELAE; PARASITE INFECTION;
   LIFE-HISTORY; TRANSMISSION; CHIROPTERA; DYNAMICS; DIVERSE;
HEALTH;
   RABIES
AB Human activities create novel food resources that can alter wildlife-pathogen interactions. If resources amplify or dampen, pathogen transmission probably depends on both host ecology and pathogen biology, but studies that measure responses to provisioning across both scales are rare. We tested these relationships with a 4-year study of 369 common vampire bats across 10 sites in Peru and Belize that differ in the abundance of livestock, an important anthropogenic food source. We quantified innate and adaptive immunity from bats and assessed infection with two common bacteria. We predicted that abundant livestock could reduce starvation and foraging effort, allowing for greater investments in immunity. Bats from high-livestock sites had higher microbicidal activity and proportions of neutrophils but lower immunoglobulin G and proportions of lymphocytes, suggesting more investment in innate relative to adaptive immunity and either greater chronic stress or pathogen exposure. This relationship was most pronounced in reproductive bats, which were also more common in high-livestock sites, suggesting feedbacks between demographic correlates of provisioning and immunity. Infection with both Bartonella and haemoplasmas were correlated with similar immune profiles, and both pathogens tended to be less prevalent in high-livestock sites, although effects were weaker for haemoplasmas. These differing responses to provisioning might therefore reflect distinct transmission processes. Predicting how provisioning alters host-pathogen interactions requires considering how both within-host processes and transmission modes respond to resource shifts.
   This article is part of the theme issue 'Anthropogenic resource subsidies and host-parasite dynamics in wildlife'.
C1 [Becker, Daniel J.; Altizer, Sonia; Streicker, Daniel G.] Univ Georgia, Odum Sch Ecol, Athens, GA 30602 USA.
   [Becker, Daniel J.; Altizer, Sonia] Univ Georgia, Ctr Ecol Infect Dis, Athens, GA 30602 USA.
   [Becker, Daniel J.] Montana State Univ, Dept Microbiol & Immunol, Bozeman, MT 59717 USA.
   [Czirjak, Gabor A.] Leibniz Inst Zoo & Wildlife Res, Dept Wildlife Dis, Berlin, Germany.
   [Volokhov, Dmitriy V.; Chizhikov, Vladimir E.] US FDA, Ctr Biol Evaluat & Res, Rockville, MD 20857 USA.
   [Bentz, Alexandra B.; Navara, Kristen J.] Univ Georgia, Dept Poultry Sci, Athens, GA 30602 USA.
   [Bentz, Alexandra B.] Indiana Univ, Dept Biol, Bloomington, IN USA.
   [Carrera, Jorge E.] Univ Nacl Piura, Fac Ciencias, Piura, Peru.
   [Carrera, Jorge E.] Programa Conservac Murcielagos Peru, Piura, Peru.

[Camus, Melinda S.] Univ Georgia, Coll Vet Med, Dept Pathol, Athens, GA 30602 USA.
[Fenton, M. Brock] Western Univ, Dept Biol, London, ON, Canada.
[Simmons, Nancy B.] Amer Museum Nat Hist, Dept Mammal, Div Vertebrate Zool, New York, NY 10024 USA.
[Recuenco, Sergio E.] Univ Nacl Mayor San Marcos, Fac Med, Dept Prevent Med & Publ Hlth, Lima, Peru.
[Gilbert, Amy T.] USDA, Natl Wildlife Res Ctr, Ft Collins, CO USA.
[Streicker, Daniel G.] Univ Glasgow, Inst Biodivers Anim Hlth & Comparat Med, Glasgow, Lanark, Scotland.
[Streicker, Daniel G.] Univ Glasgow, Ctr Virus Res, MRC, Glasgow, Lanark, Scotland.
C3 University System of Georgia; University of Georgia; University System of Georgia; University of Georgia; Montana State University System; Montana State University Bozeman; Leibniz Institut fur Zoo und Wildtierforschung; US Food & Drug Administration (FDA); University System of Georgia; University of Georgia; Indiana University System; Indiana University Bloomington; Universidad Nacional de Piura; University System of Georgia; University of Georgia; Western University (University of Western Ontario); American Museum of Natural History (AMNH); Universidad Nacional Mayor de San Marcos; United States Department of Agriculture (USDA); University of Glasgow; University of Glasgow
RP Becker, DJ (corresponding author), Univ Georgia, Odum Sch Ecol, Athens, GA 30602 USA.; Becker, DJ (corresponding author), Univ Georgia, Ctr Ecol Infect Dis, Athens, GA 30602 USA.; Becker, DJ (corresponding author), Montana State Univ, Dept Microbiol & Immunol, Bozeman, MT 59717 USA.
EM daniel.becker3@montana.edu
RI Sánchez, Melissa/A-8271-2011; Czirják, Gábor/F-5440-2011; Becker, Daniel/A-6466-2014
OI Becker, Daniel/0000-0003-4315-8628; Gilbert, Amy T./0000-0002-8256-0081; Carrera Guardia, Jorge Eduardo/0000-0001-6644-4518; Bentz, Alexandra/0000-0003-2445-4886; Streicker, Daniel/0000-0001-7475-2705; Czirjak, Gabor Arpad/0000-0001-9488-0069
FU National Science Foundation Graduate Research Fellowship; ARCS Foundation Award; Sigma Xi; Animal Behavior Society; American Society of Mammalogists; Odum School of Ecology; UGA Graduate School; UGA Latin American; Caribbean Studies Institute; UGA Biomedical and Health Sciences Institute; Explorer's Club; NSF Doctoral Dissertation Improvement Grant [DEB-1601052]; NSF [DEB-1020966, DEB-1518611]; Leibniz

Institute for Zoo and Wildlife Research; NSF Graduate Research
Fellowship; UGA Global Programs International Travel Award;
American
Museum of Natural History Taxonomic Mammalogy Fund; USDA National
Rabies
Management Program; Wellcome Trust; Royal Society [102507/Z/13/
Z];
Direct For Biological Sciences; Division Of Environmental Biology
[1601052] Funding Source: National Science Foundation; MRC
[MC_UU_12014/8] Funding Source: UKRI
FX D.J.B. was supported by a National Science Foundation Graduate
UGA Latin
American and Caribbean Studies InstituteResearch Fellowship, ARCS
Foundation Award, Sigma Xi, Animal Behavior Society, Bat
Conservation
International, American Society of Mammalogists, Odum School of
Ecology,
UGA Graduate School, UGA Latin American and Caribbean Studies
Institute,
UGA Biomedical and Health Sciences Institute, Explorer's Club and
NSF
Doctoral Dissertation Improvement Grant (DEB-1601052); fieldwork
was
also supported by NSF DEB-1020966. G.A.C. was supported by the
Leibniz
Institute for Zoo and Wildlife Research. A.B.B. was supported by
an NSF
Graduate Research Fellowship, the American Society of
Mammalogists and a
UGA Global Programs International Travel Award. KJN was funded by
NSF
DEB-1518611. Fieldwork by N.B.S. was supported by the American
Museum of
Natural History Taxonomic Mammalogy Fund. A.T.G. was supported by
the
USDA National Rabies Management Program. S.A. acknowledges
support from
NSF DEB-1518611, and D.G.S. was supported by a Sir Henry Dale
Fellowship, jointly funded by the Wellcome Trust and Royal
Society
(102507/Z/13/Z).
CR Altizer S, 2018, PHILOS T R SOC B, V373, DOI 10.1098/
rstb.2017.0102
[Anonymous], DRYAD DIGITAL REPOSI
[Anonymous], 2013, MuMIn: Multi-model inference. R package
version 1.10.0. 1
[Anonymous], ACTA ZOOL SIN
[Anonymous], 1988, The Natural History of Vampire Bats
[Anonymous], 2017, EPIDEMIOL INFECT, DOI DOI 10.1017/
S0950268817000966
[Anonymous], MODEL SELECTION MULT
Bai Y, 2016, J WILDLIFE DIS, V52, P844, DOI 10.7589/2016-01-015
Bai Ying, 2012, Am J Trop Med Hyg, V87, P518, DOI 10.4269/
ajtmh.2012.12-0097

Becker DJ, 2018, PHILOS T R SOC B, V373, DOI 10.1098/rstb.2017.0086
Becker DJ, 2018, J ANIM ECOL, V87, P511, DOI 10.1111/1365-2656.12765
Becker DJ, 2017, ROY SOC OPEN SCI, V4, DOI 10.1098/rsos.170073
Becker DJ, 2015, ECOL LETT, V18, P483, DOI 10.1111/ele.12428
Becker DJ, 2014, BIOL LETTERS, V10, DOI 10.1098/rsbl.2014.0309
BENJAMINI Y, 1995, J R STAT SOC B, V57, P289, DOI 10.1111/j.2517-6161.1995.tb02031.x
Blanco G, 2011, ECOL APPL, V21, P2469, DOI 10.1890/11-0038.1
Bobrowiec PED, 2015, J MAMMAL, V96, P54, DOI 10.1093/jmammal/gyu002
Bradley CA, 2008, ECOL APPL, V18, P1083, DOI 10.1890/07-0822.1
Brook CE, 2015, TRENDS MICROBIOL, V23, P172, DOI 10.1016/j.tim.2014.12.004
Brzek P, 2007, J EXP BIOL, V210, P2361, DOI 10.1242/jeb.003517
Budischak SA, 2015, INT J PARASITOL, V45, P455, DOI 10.1016/j.ijpara.2015.02.005
Buehler DM, 2011, PLOS ONE, V6, DOI 10.1371/journal.pone.0018592
Cade BS, 2015, ECOLOGY, V96, P2370, DOI 10.1890/14-1639.1
Carter GG, 2013, P ROY SOC B-BIOL SCI, V280, DOI 10.1098/rspb.2012.2573
Chaplin DD, 2010, J ALLERGY CLIN IMMUN, V125, pS3, DOI 10.1016/j.jaci.2009.12.980
Chomel BB, 1996, J CLIN MICROBIOL, V34, P1952, DOI 10.1128/JCM.34.8.1952-1956.1996
Clout MN, 2002, BIOL CONSERV, V107, P13, DOI 10.1016/S0006-3207(01)00267-1
Davis AK, 2008, FUNCT ECOL, V22, P760, DOI 10.1111/j.1365-2435.2008.01467.x
de Magalhaes JP, 2009, J EVOLUTION BIOL, V22, P1770, DOI 10.1111/j.1420-9101.2009.01783.x
Delpietro HA, 2017, ROY SOC OPEN SCI, V4, DOI 10.1098/rsos.160959
DELPIETRO HA, 1992, PREV VET MED, V14, P13, DOI 10.1016/0167-5877(92)90080-Y
Delpietro VA, 2002, MAMM BIOL, V67, P65, DOI 10.1078/1616-5047-00011
Diniz JAF, 2003, GLOBAL ECOL BIOGEOGR, V12, P53, DOI 10.1046/j.1466-822X.2003.00322.x
Eads DA, 2016, J MAMMAL, V97, P1044, DOI 10.1093/jmammal/gyw035
Ezenwa VO, 2012, FUNCT ECOL, V26, P123, DOI 10.1111/j.1365-2435.2011.01919.x
Forbes KM, 2016, BIOL LETTERS, V12, DOI 10.1098/rsbl.2016.0471
Freitas M. B., 2006, Braz. J. Biol., V66, P1051, DOI 10.1590/S1519-69842006000600012
Freitas MB, 2005, COMP BIOCHEM PHYS B, V140, P59, DOI 10.1016/j.cbpc.2004.09.023
French SS, 2012, BIOL OPEN, V1, P482, DOI 10.1242/bio.2012919
Galetti M, 2016, FRONT ECOL ENVIRON, V14, P505, DOI 10.1002/fee.1431
Garnier R, 2014, INTEGR COMP BIOL, V54, P363, DOI 10.1093/icb/icu022
Gilot-Fromont E, 2012, PLOS ONE, V7, DOI 10.1371/journal.pone.0045576

Gompper ME, 2005, J ZOOL, V266, P215, DOI 10.1017/S0952836905006813
Harms A, 2012, CLIN MICROBIOL REV, V25, P42, DOI 10.1128/CMR.05009-11
Hegglin D, 2007, FUNCT ECOL, V21, P552, DOI 10.1111/j.1365-2435.2007.01257.x
Hines AM, 2007, VET PARASITOL, V148, P350, DOI 10.1016/j.vetpar.2007.07.006
Horn JA, 2011, J WILDLIFE MANAGE, V75, P1177, DOI 10.1002/jwmg.145
Imai K, 2010, PSYCHOL METHODS, V15, P309, DOI 10.1037/a0020761
Judson SD, 2015, ZOONOSES PUBLIC HLTH, V62, P609, DOI 10.1111/zph.12188
Kabeya H, 2009, MICROBES INFECT, V11, P716, DOI 10.1016/j.micinf.2009.03.008
Koesling J, 2001, J IMMUNOL, V167, P11, DOI 10.4049/jimmunol.167.1.11
La Scola B, 2003, TRENDS MICROBIOL, V11, P318, DOI 10.1016/S0966-842X(03)00143-4
Lee KA, 2006, INTEGR COMP BIOL, V46, P1000, DOI 10.1093/icb/icl049
León B, 2008, IMMUNOL CELL BIOL, V86, P320, DOI 10.1038/icb.2008.14
Lochmiller RL, 2000, OIKOS, V88, P87, DOI 10.1034/j.1600-0706.2000.880110.x
Martin LB, 2007, AM J PHYSIOL-REG I, V292, pR316, DOI 10.1152/ajpregu.00386.2006
McDade TW, 2016, EVOL MED PUBLIC HLTH, P1, DOI 10.1093/emph/eov033
Messick JB, 2004, VET CLIN PATH, V33, P2, DOI 10.1111/j.1939-165X.2004.tb00342.x
Moller AP, 2006, OIKOS, V115, P463, DOI 10.1111/j.2006.0030-1299.15312.x
Moore MS, 2011, PLOS ONE, V6, DOI 10.1371/journal.pone.0027430
Moyers SC, 2018, PHILOS T R SOC B, V373, DOI 10.1098/rstb.2017.0090
Murray MH, 2018, PHILOS T R SOC B, V373, DOI 10.1098/rstb.2017.0100
Murray MH, 2016, BIOL CONSERV, V204, P163, DOI 10.1016/j.biocon.2016.10.034
Museux K, 2009, VET RES, V40, DOI 10.1051/vetres/2009028
Nakagawa S, 2013, METHODS ECOL EVOL, V4, P133, DOI 10.1111/j.2041-210x.2012.00261.x
Oro D, 2013, ECOL LETT, V16, P1501, DOI 10.1111/ele.12187
Post DM, 2002, ECOLOGY, V83, P703, DOI 10.2307/3071875
Pulliam JRC, 2012, J R SOC INTERFACE, V9, P89, DOI 10.1098/rsif.2011.0223
Raats M. M., 1992, Food Quality and Preference, V3, P89, DOI 10.1016/0950-3293(91)90028-D
Robinson TP, 2014, PLOS ONE, V9, DOI 10.1371/journal.pone.0096084
RODRIGUEZBARRADAS MC, 1995, INFECT IMMUN, V63, P2367, DOI 10.1128/IAI.63.6.2367-2370.1995
Rosing MN, 1998, J WILDLIFE MANAGE, V62, P380, DOI 10.2307/3802302

Ruiz M, 2010, HORM BEHAV, V57, P134, DOI 10.1016/j.yhbeh.2009.09.019
Schinnerl M, 2011, J ZOO WILDLIFE MED, V42, P382, DOI 10.1638/2010-0060.1
Schneeberger K, 2014, PLOS ONE, V9, DOI 10.1371/journal.pone.0108268
Schneeberger K, 2013, PLOS ONE, V8, DOI 10.1371/journal.pone.0054023
Seltmann A, 2017, CONSERV PHYSIOL, V5, DOI 10.1093/conphys/cox020
Semeniuk CAD, 2008, MAR ECOL PROG SER, V357, P271, DOI 10.3354/meps07299
Shochat E, 2004, OIKOS, V106, P622, DOI 10.1111/j.0030-1299.2004.13159.x
Simmons NB, 1998, B AM MUS NAT HIST, P1
Stoner-Duncan B, 2014, PLOS NEGLECT TROP D, V8, DOI 10.1371/journal.pntd.0002867
Strandin T, 2018, PHILOS T R SOC B, V373, DOI 10.1098/rstb.2017.0088
Streicker DG, 2016, J APPL ECOL, V53, P1280, DOI 10.1111/1365-2664.12690
Streicker DG, 2012, P ROY SOC B-BIOL SCI, V279, P3384, DOI 10.1098/rspb.2012.0538
Tella JL, 2001, P ROY SOC B-BIOL SCI, V268, P1455, DOI 10.1098/rspb.2001.1688
Tieleman BI, 2005, P ROY SOC B-BIOL SCI, V272, P1715, DOI 10.1098/rspb.2005.3155
Tingley D, 2014, J STAT SOFTW, V59
Trajano E, 1996, BIOTROPICA, V28, P121, DOI 10.2307/2388777
Turmelle AS, 2010, J GEN VIROL, V91, P2360, DOI 10.1099/vir.0.020073-0
Venables W. N., 2013, MODERN APPL STAT S P
Voigt CC, 2006, J MAMMAL, V87, P1, DOI 10.1644/05-MAMM-F-276R1.1
Voigt CC, 2008, J COMP PHYSIOL B, V178, P9, DOI 10.1007/s00360-007-0194-z
Volokhov DV, 2017, EPIDEMIOL INFECT, V145, P3154, DOI 10.1017/S095026881700231X
Volokhov DV, 2011, VET MICROBIOL, V149, P262, DOI 10.1016/j.vetmic.2010.10.026
Weise P, 2017, PEERJ, V5, DOI 10.7717/peerj.3570
Wellehan JFX, 2009, COMP IMMUNOL MICROB, V32, P379, DOI 10.1016/j.cimid.2007.11.002
Wilcoxen TE, 2015, CONSERV PHYSIOL, V3, DOI 10.1093/conphys/cov058
WILKINSON GS, 1986, ANIM BEHAV, V34, P1880, DOI 10.1016/S0003-3472(86)80274-3
WILKINSON GS, 1984, NATURE, V308, P181, DOI 10.1038/308181a0
Young HS, 2016, FUNCT ECOL, V30, P799, DOI 10.1111/1365-2435.12542
Zuur Alain F., 2009, P1
NR 101
TC 57
Z9 62
U1 0
U2 64

PU ROYAL SOC
PI LONDON
PA 6-9 CARLTON HOUSE TERRACE, LONDON SW1Y 5AG, ENGLAND
SN 0962-8436
EI 1471-2970
J9 PHILOS T R SOC B
JI Philos. Trans. R. Soc. B-Biol. Sci.
PD MAY 5
PY 2018
VL 373
IS 1745
AR 20170089
DI 10.1098/rstb.2017.0089
PG 12
WC Biology
WE Science Citation Index Expanded (SCI-EXPANDED)
SC Life Sciences & Biomedicine - Other Topics
GA FZ0VS
UT WOS:000427292400004
PM 29531144
OA Green Published, Green Accepted, Green Submitted, hybrid
DA 2024-10-21
ER

PT J
AU Fritze, M
   Puechmaille, SJ
   Costantini, D
   Fickel, J
   Voigt, CC
   Czirják, GA
AF Fritze, Marcus
   Puechmaille, Sebastien J.
   Costantini, David
   Fickel, Jorns
   Voigt, Christian C.
   Czirjak, Gabor A.
TI Determinants of defence strategies of a hibernating European bat species
   towards the fungal pathogen Pseudogymnoascus destructans
SO DEVELOPMENTAL AND COMPARATIVE IMMUNOLOGY
LA English
DT Article
DE White-nose disease; Hibernation immunology; Acute phase response;
   Haptoglobin; Oxidative stress; Tolerance; Chiroptera
ID WHITE-NOSE SYNDROME; MAMMALIAN HIBERNATION; GEOMYCES-DESTRUCTANS;
   MYOTIS-LUCIFUGUS; HAPTOGLOBIN; INFECTION; RESPONSES; TOLERANCE; IMPACTS;
   ORIGIN
AB Pseudogymnoascus destructans (Pd), the causative agent of white-nose syndrome in North America, has decimated bat populations within a decade. The fungus impacts bats during hibernation when physiological functions, including immune responses, are down-regulated. Studies have shown that Pd is native to Europe, where it

is not associated with mass mortalities. Moreover, genomic and proteomic studies indicated that European bats may have evolved an effective immune defence, which is lacking in North American bats. However, it is still unclear which defence strategy enables European bats to cope with the pathogen. Here, we analyzed selected physiological and immunological parameters in torpid, Pd infected European greater mouse-eared bats (Myotis myotis) showing three different levels of infection (asymptomatic, mild and severe symptoms). From a subset of the studied bats we tracked skin temperatures during one month of hibernation. Contrasting North American bats, arousal patterns remained unaffected by Pd infections in M. myotis. In general, heavier M. myotis aroused more often from hibernation and showed less severe disease symptoms than lean individuals; most likely because heavy bats were capable of reducing the Pd load more effectively than lean individuals. In the blood of severely infected bats, we found higher gene expression levels of an inflammatory cytokine (IL-1?), but lower levels of an acute phase protein (haptoglobin), reactive oxygen metabolites (ROMs) and plasma non-enzymatic antioxidant capacity (OXY) compared to conspecifics with lower levels of infection. We conclude that M. myotis, and possibly also other European bat species, tolerate Pd infections during torpor by using selected acute phase response parameters at baseline levels, yet without arousing from torpor and without synthesizing additional immune molecules.
C1 [Fritze, Marcus; Voigt, Christian C.; Czirjak, Gabor A.] Leibniz Inst Zoo & Wildlife Res, Alfred Kowalke Str 17, D-10315 Berlin, Germany.
[Fritze, Marcus; Voigt, Christian C.] Free Univ Berlin, Inst Biol, Takustr 6, D-14195 Berlin, Germany.
[Puechmaille, Sebastien J.] Univ Montpellier, CNRS, Inst Sci Evolut, EPHE,IRD, F-34095 Montpellier, France.
[Costantini, David] Museum Natl Hist Nat, Unite Physiol Mol & Adapt PhyMA, CNRS, CP32,57 Rue Cuvier, F-75005 Paris, France.
[Fickel, Jorns] Univ Potsdam, Inst Biochem & Biol, Karl Liebknecht Str 24-25, D-14476 Potsdam, Germany.
[Fritze, Marcus] Univ Greifswald, Appl Zool & Nat Conservat, Loitzer Str 26, D-17489 Greifswald, Germany.
C3 Leibniz Institut fur Zoo und Wildtierforschung; Free University of
Berlin; Centre National de la Recherche Scientifique (CNRS); Institut de
Recherche pour le Developpement (IRD); Universite de Montpellier;
Universite PSL; Ecole Pratique des Hautes Etudes (EPHE); Centre National
de la Recherche Scientifique (CNRS); Museum National d'Histoire
Naturelle (MNHN); University of Potsdam; Universitat Greifswald
RP Fritze, M (corresponding author), Leibniz Inst Zoo & Wildlife Res, Alfred Kowalke Str 17, D-10315 Berlin, Germany.; Fritze, M (corresponding author), Univ Greifswald, Appl Zool & Nat Conservat, Loitzer Str 26, D-17489 Greifswald, Germany.
EM marcus.fritze@uni-greifswald.de
RI Costantini, David/C-6006-2013; Fritze, Marcus/H-3597-2015; Czirják,
Gábor/F-5440-2011; Puechmaille, Sebastien/D-1612-2010

OI Czirjak, Gabor Arpad/0000-0001-9488-0069; Puechmaille,
Sebastien/0000-0001-9517-5775
FU Leibniz-Institute for Zoo and Wildlife Research
FX We thank Janina Radwainski, Susanne Holtze and Hanna Pruter for
veterinary support; Bernd Ohlendorf, Matthias Hammer, Bernhard Walk and
Carolin Stern for help during field work; Katja Pohle, Miriam Hahn and
Anke Schmidt for the laboratory work; Kseniia Kravchenko and Nicolas
Fasel for helpful comments concerning statistical analysis and Gudrun
Wibbelt and Alexander Scheuerlein for the helpful discussions. This
study was supported by funds from the Leibniz-Institute for Zoo and
Wildlife Research.
CR Bandouchova H, 2018, SCI REP-UK, V8, DOI 10.1038/s41598-018-24461-5
Bouma HR, 2010, J LEUKOCYTE BIOL, V88, P619, DOI 10.1189/jlb.0310174
Brockmann D., 2011, NYCTALUS, V16, P186
Brownlee-Bouboulis SA, 2013, J WILDLIFE DIS, V49, P850, DOI 10.7589/2012-10-242
Carey HV, 2003, PHYSIOL REV, V83, P1153, DOI 10.1152/physrev.00008.2003
Cheng TNL, 2019, J ANIM ECOL, V88, P591, DOI 10.1111/1365-2656.12954
Chow BA, 2013, PLOS ONE, V8, DOI 10.1371/journal.pone.0066119
COSTANTINI D., 2014, Oxidative stress and hormesis in evolutionary ecology and physiology: a marriage between mechanistic and evolutionary approaches
Costantini D, 2019, SCI TOTAL ENVIRON, V671, P1077, DOI 10.1016/j.scitotenv.2019.03.398
Costantini D, 2009, COMP BIOCHEM PHYS A, V153, P339, DOI 10.1016/j.cbpa.2009.03.010
Cray C, 2009, COMPARATIVE MED, V59, P517
Cryan PM, 2010, BMC BIOL, V8, DOI 10.1186/1741-7007-8-135
Davy CM, 2020, VIRULENCE, V11, P781, DOI 10.1080/21505594.2020.1768018
Drees KP, 2017, MBIO, V8, DOI 10.1128/mBio.01941-17
EATON JW, 1982, SCIENCE, V215, P691, DOI 10.1126/science.7036344
Field KA, 2018, MOL ECOL, V27, P3727, DOI 10.1111/mec.14827
Field KA, 2015, PLOS PATHOG, V11, DOI 10.1371/journal.ppat.1005168
Frank CL, 2019, FRONT ZOOL, V16, DOI 10.1186/s12983-019-0340-y
Frank CL, 2014, PLOS ONE, V9, DOI 10.1371/journal.pone.0113958
FRENCH AR, 1985, J COMP PHYSIOL B, V156, P13, DOI 10.1007/BF00692921
Frick WF, 2017, ECOLOGY, V98, P624, DOI 10.1002/ecy.1706
Frick WF, 2015, GLOBAL ECOL BIOGEOGR, V24, P741, DOI 10.1111/geb.12290
Fritze M., 2021, RAPID IN SITU MINIMA
Fritze M., 2012, NYCTALUS, V17, P146

Fritze M, 2019, BIOL OPEN, V8, DOI 10.1242/bio.046078
Fritze M, 2018, MAMMAL REV, V48, P224, DOI 10.1111/mam.12122
Fuller NW, 2020, J EXP BIOL, V223, DOI 10.1242/jeb.211912
Gelman A, 2008, STAT MED, V27, P2865, DOI 10.1002/sim.3107
Gruet Dr., 1949, Mammalia Paris, V13, P69
Harazim M, 2018, BMC ZOOL, V3, DOI 10.1186/s40850-018-0035-4
Hecht AM, 2015, SCI REP-UK, V5, DOI 10.1038/srep16604
Hecht-Höger AM, 2020, MOL ECOL, V29, P1745, DOI 10.1111/mec.15437
Hildebrand F, 2005, CYTOKINE, V31, P382, DOI 10.1016/j.cyto.2005.06.008
Hothorn T., 2019, PACKAGE MULTCOMP
Humphries MM, 2003, PHYSIOL BIOCHEM ZOOL, V76, P165, DOI 10.1086/367950
Johnson JS, 2015, ECOL EVOL, V5, P2203, DOI 10.1002/ece3.1502
Jonasson KA, 2011, PLOS ONE, V6, DOI 10.1371/journal.pone.0021061
Kacprzyk J, 2017, ACTA CHIROPTEROL, V19, P219, DOI [10.3161/15081109ACC2017.19.2.001, 10.3161/15081109acc2017.19.2.001]
Kuncewicz T, 2001, AM J PHYSIOL-RENAL, V281, pF326, DOI 10.1152/ajprenal.2001.281.2.F326
Kuznetsova A., 2019, PACKAGE LMER TEST
Langwig KE, 2017, PHILOS T R SOC B, V372, DOI 10.1098/rstb.2016.0044
Leopardi S, 2015, CURR BIOL, V25, pR217, DOI 10.1016/j.cub.2015.01.047
Lilley TM, 2017, P ROY SOC B-BIOL SCI, V284, DOI 10.1098/rspb.2016.2232
Lilley TM, 2019, OECOLOGIA, V191, P295, DOI 10.1007/s00442-019-04499-6
Lilley TM, 2016, FRONT ZOOL, V13, DOI 10.1186/s12983-016-0143-3
Lorch JM, 2016, PHILOS T R SOC B, V371, DOI 10.1098/rstb.2015.0457
Maniero GD, 2002, DEV COMP IMMUNOL, V26, P563, DOI 10.1016/S0145-305X(02)00006-X
Medzhitov R, 2012, SCIENCE, V335, P936, DOI 10.1126/science.1214935
Meierhofer MB, 2018, J WILDLIFE DIS, V54, P480, DOI 10.7589/2017-08-195
Meteyer CU, 2011, J WILDLIFE DIS, V47, P618, DOI 10.7589/0090-3558-47.3.618
Meteyer CU, 2009, J VET DIAGN INVEST, V21, P411, DOI 10.1177/104063870902100401
Mominoki K, 2005, COMP BIOCHEM PHYS A, V142, P472, DOI 10.1016/j.cbpa.2005.09.017
Moore MS, 2018, J COMP PHYSIOL B, V188, P163, DOI 10.1007/s00360-017-1109-2
Moore MS, 2013, PLOS ONE, V8, DOI 10.1371/journal.pone.0058976
Morin P, 2009, INT J DEV BIOL, V53, P433, DOI 10.1387/ijdb.082643pm
Nijsten MWN, 2000, CRIT CARE MED, V28, P458, DOI 10.1097/00003246-200002000-00028
O'Hanlon SJ, 2018, SCIENCE, V360, P621, DOI 10.1126/science.aar1965
Prendergast BJ, 2002, AM J PHYSIOL-REG I, V282, pR1054, DOI 10.1152/ajpregu.00562.2001

Puechmaille SJ, 2011, PLOS ONE, V6, DOI 10.1371/journal.pone.0019167
Reeder DM, 2012, PLOS ONE, V7, DOI 10.1371/journal.pone.0038920
Schneeberger K, 2014, PLOS ONE, V9, DOI 10.1371/journal.pone.0108268
Schneider DS, 2008, NAT REV IMMUNOL, V8, P889, DOI 10.1038/nri2432
Skovgaard K, 2009, VET RES, V40, DOI 10.1051/vetres/2009006
Theilgaard-Mönch K, 2006, BLOOD, V108, P353, DOI 10.1182/blood-2005-09-3890
Tizard I.R., 2008, VET IMMUNOLOGY
Turner GG, 2014, J WILDLIFE DIS, V50, P566, DOI 10.7589/2014-03-058
Verant Michelle L., 2014, BMC Physiology, V14, P10, DOI 10.1186/s12899-014-0010-4
Warnecke L, 2012, P NATL ACAD SCI USA, V109, P6999, DOI 10.1073/pnas.1200374109
Wei T, 2017, PACKAGE CORRPLOT
Wibbelt G, 2013, PLOS ONE, V8, DOI 10.1371/journal.pone.0074105
Zervanos SM, 2014, INTEGR COMP BIOL, V54, P443, DOI 10.1093/icb/ict100
Zukal J, 2016, SCI REP-UK, V6, DOI 10.1038/srep19829
NR 72
TC 15
Z9 15
U1 1
U2 21
PU ELSEVIER SCI LTD
PI OXFORD
PA THE BOULEVARD, LANGFORD LANE, KIDLINGTON, OXFORD OX5 1GB, OXON, ENGLAND
SN 0145-305X
EI 1879-0089
J9 DEV COMP IMMUNOL
JI Dev. Comp. Immunol.
PD JUN
PY 2021
VL 119
AR 104017
DI 10.1016/j.dci.2021.104017
EA JAN 2021
PG 9
WC Fisheries; Immunology; Veterinary Sciences; Zoology
WE Science Citation Index Expanded (SCI-EXPANDED)
SC Fisheries; Immunology; Veterinary Sciences; Zoology
GA WK1IJ
UT WOS:000709486400010
PM 33476670
DA 2024-10-21
ER

PT J
AU Xie, JZ
Tan, BW

Zhang, Y
AF Xie, Jiazheng
Tan, Bowen
Zhang, Yi
TI Positive Selection and Duplication of Bat TRIM Family Proteins
SO VIRUSES-BASEL
LA English
DT Article
DE Chiroptera; TRIM; antiviral immunity; positive selection; gene duplication
ID E3 UBIQUITIN LIGASE; RECOGNITION; EVOLUTION; GENOMES; VIRUS; GENES;
ROLES; ALPHA
AB Bats have received increasing attention because of some unique biological features they possess. TRIM is a large family of proteins that participate in diverse cellular functions, such as antiviral immunity, DNA damage repair, tumor suppression, and aging. These functional areas appear to be highly consistent with the special characteristics of bats, such as tolerance to viruses and DNA damage generated in flight, low cancer incidence, and longevity. However, there is still a lack of systematic study of the TRIM family in bats. Here, we explored the TRIM family of bats using the genomes of 16 representative species. The results showed that the bat TRIM family contains 70 members, with 24 under positive selection and 7 duplicated. Additional transcriptomic analysis revealed the tissue-specific expressions of TRIM9, 46, 54, 55, 63, and 72. Additionally, following interferon or viral stimulation, TRIM orthologs associated with antiviral immunity reported in humans were also upregulated in bat cells. The present study systematically analyzed the composition, evolution, and expression of bat TRIM genes. It may provide a theoretical basis for studies of bat TRIM in the fields of antiviral immunity, longevity, and tolerance to DNA damage.
C1 [Xie, Jiazheng; Tan, Bowen; Zhang, Yi] Chongqing Univ Posts & Telecommun, Chongqing Key Lab Big Data Bio Intelligence, Chongqing 400065, Peoples R China.
C3 Chongqing University of Posts & Telecommunications
RP Xie, JZ (corresponding author), Chongqing Univ Posts & Telecommun, Chongqing Key Lab Big Data Bio Intelligence, Chongqing 400065, Peoples R China.
EM xiejz@cqupt.edu.cn
RI xie, jiazheng/KOC-8979-2024; Tan, Bowen/LDE-8670-2024
OI Xie, Jiazheng/0000-0003-4341-5575
FU National Natural Science Foundation of China [31900152]; Science and
Technology Research Program of Chongqing Municipal Education Commission
[KJQN202100632]
FX This research was funded by the National Natural Science Foundation of
China (grant No. 31900152) and the Science and Technology Research
Program of Chongqing Municipal Education Commission (grant No.
KJQN202100632).
NR 0

TC 2
Z9 2
U1 0
U2 8
PU MDPI
PI BASEL
PA ST ALBAN-ANLAGE 66, CH-4052 BASEL, SWITZERLAND
EI 1999-4915
J9 VIRUSES-BASEL
JI Viruses-Basel
PD APR
PY 2023
VL 15
IS 4
AR 875
DI 10.3390/v15040875
PG 11
WC Virology
WE Science Citation Index Expanded (SCI-EXPANDED)
SC Virology
GA E8KR4
UT WOS:000977971900001
PM 37112854
OA gold, Green Published
DA 2024-10-21
ER

PT J
AU Salmier, A
   de Thoisy, B
   Crouau-Roy, B
   Lacoste, V
   Lavergne, A
AF Salmier, Arielle
   de Thoisy, Benoit
   Crouau-Roy, Brigitte
   Lacoste, Vincent
   Lavergne, Anne
TI Spatial pattern of genetic diversity and selection in the MHC class II
   <i>DRB</i> of three Neotropical bat species
SO BMC EVOLUTIONARY BIOLOGY
LA English
DT Article
DE MHC; DRB polymorphism; Selection; Carollia perspicillata; Desmodus
   rotundus; Molossus molossus
ID MAJOR-HISTOCOMPATIBILITY-COMPLEX; AMINO-ACID SITES; PHYLOGENETIC
   ANALYSIS; EVOLUTION; PARASITE; POLYMORPHISM; ADAPTATION; ORIGIN;
   DIFFERENTIATION; FRAGMENTATION
AB Background: Although bats are natural reservoirs of many pathogens, few studies have been conducted on the genetic variation and detection of selection in major histocompatibility complex (MHC) genes. These genes are critical for resistance and susceptibility to

diseases, and host-pathogen interactions are major determinants of their extensive polymorphism. Here we examined spatial patterns of diversity of the expressed MHC class II DRB gene of three sympatric Neotropical bats, Carollia perspicillata and Desmodus rotundus (Phyllostomidae), and Molossus molossus (Molossidae), all of which use the same environments (e.g., forests, edge habitats, urban areas). Comparison with neutral marker (mtDNA D-loop) diversity was performed at the same time.

Results: Twenty-three DRB alleles were identified in 19 C. perspicillata, 30 alleles in 35 D. rotundus and 20 alleles in 28 M. molossus. The occurrence of multiple DRB loci was found for the two Phyllostomidae species. The DRB polymorphism was high in all sampling sites and different signatures of positive selection were detected depending on the environment. The patterns of DRB diversity were similar to those of neutral markers for C. perspicillata and M. molossus. In contrast, these patterns were different for D. rotundus for which a geographical structure was highlighted. A heterozygote advantage was also identified for this species. No recombination or gene conversion event was found and phylogenetic relationships showed a trans-species mode of evolution in the Phyllostomids.

Conclusions: This study of MHC diversity demonstrated the strength of the environment and contrasting pathogen pressures in shaping DRB diversity. Differences between positively selected sites identified in bat species highlighted the potential role of gut microbiota in shaping immune responses. Furthermore, multiple geographic origins and/or population admixtures observed in C. perspicillata and M. molossus populations acted as an additional force in shaping DRB diversity. In contrast, DRB diversity of D. rotundus was shaped by environment rather than demographic history.
C1 [Salmier, Arielle; de Thoisy, Benoit; Lacoste, Vincent; Lavergne, Anne] Inst Pasteur, Lab Interact Virus Hotes, 23 Ave Pasteur,BP 6010, Cayenne 97306, French Guiana.
[Crouau-Roy, Brigitte] Univ Toulouse 3, UPS, CNRS, Lab Evolut & Diversite Biol,ENFA,UMR 5174 EDB, F-31062 Toulouse, France.
C3 Universite Paris Cite; Universite de Toulouse; Universite Toulouse III -
Paul Sabatier; Centre National de la Recherche Scientifique (CNRS); CNRS
- Institute of Ecology & Environment (INEE); Universite Federale
Toulouse Midi-Pyrenees (ComUE); Ecole Nationale Formation Agronomique
(ENSFEA)
RP Lavergne, A (corresponding author), Inst Pasteur, Lab Interact Virus Hotes, 23 Ave Pasteur,BP 6010, Cayenne 97306, French Guiana.
EM alavergne@pasteur-cayenne.fr
RI LACOSTE, Vincent/V-2321-2019
OI Lavergne, Anne/0000-0002-5304-5574; de thoisy, benoit/0000-0002-8420-5112; Lacoste, Vincent/0000-0002-3173-4053
FU European funds (PO FSE); Agence Nationale de la Recherche (CEBA) [ANR-10- LABEX-25-01]; European funds (ERDF/FEDER); European Commission
grant within the FP7 [REGPOT-CT-2011-285837-STRonGer]; "Investissement
d'Avenir" grants - Agence Nationale de la Recherche (CEBA)

[ANR-10-
LABEX-25-01]
FX A. Salmier was supported by a grant from European funds (PO FSE
2007-2013) and "Investissement d'Avenir" managed by Agence
Nationale de
la Recherche (CEBA, Ref. ANR-10- LABEX-25-01). This study was
conducted
within the CAROLIA program supported by European funds (ERDF/
FEDER) and
assistance from Region Guyane and Direction Regionale pour la
Recherche
et la Technologie. It also received a European Commission
"REGPOT-CT-2011-285837-STRonGer" grant within the FP7 and
"Investissement d'Avenir" grants managed by the Agence Nationale
de la
Recherche (CEBA, Ref. ANR-10- LABEX-25-01). The funders had no
role in
study design, data collection and analysis, decision to publish,
or
preparation of the manuscript.
CR Alcaide M, 2010, MOL ECOL, V19, P3842, DOI 10.1111/
j.1365-294X.2010.04772.x
[Anonymous], 2015, ZOONOSES INFECT AFFE
Babik W, 2009, MOL ECOL, V18, P769, DOI 10.1111/
j.1365-294X.2008.04057.x
Becker DJ, 2015, ECOL LETT, V18, P483, DOI 10.1111/ele.12428
Bowen L, 2004, IMMUNOGENETICS, V56, P12, DOI 10.1007/
s00251-004-0655-4
Brook CE, 2015, TRENDS MICROBIOL, V23, P172, DOI 10.1016/
j.tim.2014.12.004
Brosset A, 1996, CAN J ZOOL, V74, P1974, DOI 10.1139/z96-224
BROWN JH, 1993, NATURE, V364, P33, DOI 10.1038/364033a0
Carrillo-Araujo M, 2015, FRONT MICROBIOL, V6, DOI 10.3389/
fmicb.2015.00447
Castro-Prieto A, 2011, MOL BIOL EVOL, V28, P1455, DOI 10.1093/
molbev/msq330
Catzeflis Francois, 2013, Rhinolophe, V19, P89
Clozato CL, 2015, ECOL EVOL, V5, P4932, DOI 10.1002/ece3.1656
Consuegra S, 2011, EVOL APPL, V4, P660, DOI 10.1111/
j.1752-4571.2011.00189.x
Darriba D, 2012, NAT METHODS, V9, P772, DOI 10.1038/nmeth.2109
de Bellocq JG, 2009, IMMUNOGENETICS, V61, P131, DOI 10.1007/
s00251-008-0349-4
de Thoisy B, 2016, PLOS NEGLECT TROP D, V10, DOI 10.1371/
journal.pntd.0004378
Del Real-Monroy M, 2014, ACTA CHIROPTEROL, V16, P301, DOI
10.3161/150811014X687260
Delaval M, 2006, REV ECOL-TERRE VIE, V61, P343
Delport W, 2010, BIOINFORMATICS, V26, P2455, DOI 10.1093/
bioinformatics/btq429
Dionne M, 2007, EVOLUTION, V61, P2154, DOI 10.1111/
j.1558-5646.2007.00178.x
Ditchkoff SS, 2001, EVOLUTION, V55, P616, DOI
10.1554/0014-3820(2001)055[0616:MHCAVI]2.0.CO;2

Drummond AJ, 2005, MOL BIOL EVOL, V22, P1185, DOI [10.1093/molbev/msi103, 10.1093/molbev/mss075]
Dunn RR, 2010, P ROY SOC LOND B BIO, V14
Excoffier L, 2010, MOL ECOL RESOUR, V10, P564, DOI 10.1111/j.1755-0998.2010.02847.x
Fenton M.B., 2015, Bats: a world of science and mystery
Froeschke G, 2014, EVOL ECOL, V28, P1169, DOI 10.1007/s10682-014-9731-x
Froeschke G, 2014, PARASITE VECTOR, V7, DOI 10.1186/1756-3305-7-393
Fuller CA, 2011, ECOL ENTOMOL, V36, P459, DOI 10.1111/j.1365-2311.2011.01289.x
Fumagalli M, 2011, PLOS GENET, V7, DOI 10.1371/journal.pgen.1002355
Gannon WL, 2007, J MAMMAL, V88, P809, DOI 10.1644/06-MAMM-F-185R1.1
Gay Noellie, 2014, International Journal for Parasitology Parasites and Wildlife, V3, P161, DOI 10.1016/j.ijppaw.2014.06.003
Goudet J, FSTAT PROGRAM ESTIMA
Guindon S, 2003, SYST BIOL, V52, P696, DOI 10.1080/10635150390235520
Hayman DTS, 2016, SCI ADV, V2, DOI 10.1126/sciadv.1500831
Hedrick PW, 2002, EVOLUTION, V56, P1902, DOI 10.1111/j.0014-3820.2002.tb00116.x
HEDRICK PW, 1988, GENETICS, V119, P199
Huelsenbeck JP, 2001, BIOINFORMATICS, V17, P754, DOI 10.1093/bioinformatics/17.8.754
Hughes A, 1999, ADAPTIVE EVOLUTION G, P54
Hughes AL, 1998, ANNU REV GENET, V32, P415, DOI 10.1146/annurev.genet.32.1.415
Hughes Austin L., 1998, Frontiers in Bioscience, V3, pD509
Jensen JL, 2005, BMC GENET, V6, DOI 10.1186/1471-2156-6-13
Johnson JS, 2014, PLOS ONE, V9, DOI 10.1371/journal.pone.0112502
Johnson M, 2008, NUCLEIC ACIDS RES, V36, pW5, DOI 10.1093/nar/gkn201
Jones KE, 2008, NATURE, V451, P990, DOI 10.1038/nature06536
JUKES T H, 1969, P21
Kamiya T, 2014, ECOGRAPHY, V37, P689, DOI 10.1111/j.1600-0587.2013.00571.x
Katoh K, 2002, NUCLEIC ACIDS RES, V30, P3059, DOI 10.1093/nar/gkf436
Kearse M, 2012, BIOINFORMATICS, V28, P1647, DOI 10.1093/bioinformatics/bts199
Kennedy LJ, 2002, IMMUNOGENETICS, V54, P348, DOI 10.1007/s00251-002-0465-5
KLEIN J, 1987, HUM IMMUNOL, V19, P155, DOI 10.1016/0198-8859(87)90066-8
KLEIN J, 1990, IMMUNOGENETICS, V31, P217, DOI 10.1007/BF00204890
Kloch A, 2010, MOL ECOL, V19, P255, DOI 10.1111/j.1365-294X.2009.04476.x
Pond SLK, 2006, MOL BIOL EVOL, V23, P1891, DOI 10.1093/molbev/msl051
Kupfermann H, 1999, J MOL EVOL, V48, P663, DOI 10.1007/PL00006510
Leigh JW, 2015, METHODS ECOL EVOL, V6, P1110, DOI

10.1111/2041-210X.12410
Lenz TL, 2013, MOL ECOL, V22, P774, DOI 10.1111/j.1365-294X.2012.05756.x
Librado P, 2009, BIOINFORMATICS, V25, P1451, DOI 10.1093/bioinformatics/btp187
Marsden CD, 2012, MOL ECOL, V21, P1379, DOI 10.1111/j.1365-294X.2012.05477.x
Martins FM, 2009, BMC EVOL BIOL, V9, DOI 10.1186/1471-2148-9-294
Mayer F, 2007, HEREDITY, V99, P257, DOI 10.1038/sj.hdy.6800989
Meyer-Lucht Y, 2008, BMC GENET, V9, DOI 10.1186/1471-2156-9-39
Miller HC, 2010, MOL ECOL, V19, P3894, DOI 10.1111/j.1365-294X.2010.04771.x
Monzón-Argüello C, 2013, ECOL EVOL, V3, P3359, DOI 10.1002/ece3.701
Murrell B, 2012, PLOS GENET, V8, DOI 10.1371/journal.pgen.1002764
Myers N, 2000, NATURE, V403, P853, DOI 10.1038/35002501
Nei M, 1997, P NATL ACAD SCI USA, V94, P7799, DOI 10.1073/pnas.94.15.7799
NEI M, 1986, MOL BIOL EVOL, V3, P418
Palmer JM, 2016, PEER J PREPR, V4
Pavan AC, 2011, BIOL J LINN SOC, V102, P527, DOI 10.1111/j.1095-8312.2010.01601.x
Pechouskova E, 2015, IMMUNOGENETICS, V67, P229, DOI 10.1007/s00251-015-0827-4
Pedersen AB, 2009, ECOHEALTH, V6, P496, DOI 10.1007/s10393-010-0284-3
Penn D.J., 2005, Encyclopedia of Life Science, P1
Piertney SB, 2006, HEREDITY, V96, P7, DOI 10.1038/sj.hdy.6800724
Pond SLK, 2005, MOL BIOL EVOL, V22, P1208, DOI 10.1093/molbev/msi105
Pond SLK, 2005, BIOINFORMATICS, V21, P676, DOI 10.1093/bioinformatics/bti079
Poulin R, 2014, INT J PARASITOL, V44, P581, DOI 10.1016/j.ijpara.2014.02.003
Prugnolle F, 2005, CURR BIOL, V15, P1022, DOI 10.1016/j.cub.2005.04.050
Richman AD, 2010, INT J IMMUNOGENET, V37, P401, DOI 10.1111/j.1744-313X.2010.00941.x
Rivero-de Aguilar J, 2013, ECOL EVOL, V3, P1878, DOI 10.1002/ece3.598
Rojas D, 2016, SYST BIOL, V65, P432, DOI 10.1093/sysbio/syw011
Rousset F, 1997, GENETICS, V145, P1219
SAWYER S, 1989, MOL BIOL EVOL, V6, P526
Schad J, 2011, HEREDITY, V107, P115, DOI 10.1038/hdy.2010.173
Schad J, 2012, IMMUNOGENETICS, V64, P535, DOI 10.1007/s00251-012-0609-1
Schaschl H, 2006, HEREDITY, V97, P427, DOI 10.1038/sj.hdy.6800892
Sommer Simone, 2005, Frontiers in Zoology, V2, P1, DOI 10.1186/1742-9994-2-16
Tamura K, 2013, MOL BIOL EVOL, V30, P2725, DOI [10.1093/molbev/mst197, 10.1093/molbev/msr121]
Tesicky M, 2015, J IMMUNOL RES, V2015, DOI 10.1155/2015/838035
van Oosterhout C, 2006, EVOLUTION, V60, P2562, DOI 10.1111/j.0014-3820.2006.tb01890.x

WILKINSON GS, 1991, GENETICS, V128, P607
Winternitz JC, 2013, ECOL EVOL, V3, P1552, DOI 10.1002/ece3.567
Wlasiuk G, 2010, MOL BIOL EVOL, V27, P2172, DOI 10.1093/molbev/msq104
Yang ZH, 2000, GENETICS, V155, P431
Yang ZH, 2005, MOL BIOL EVOL, V22, P1107, DOI 10.1093/molbev/msi097
Yang ZH, 2007, MOL BIOL EVOL, V24, P1586, DOI 10.1093/molbev/msm088
Yao G, 2015, ANIM GENET, V46, P535, DOI 10.1111/age.12336
Yu FJ, 2015, SCI BULL, V60, P136, DOI 10.1007/s11434-014-0686-7
Zarlenga DS, 2014, J PARASITOL, V100, P756, DOI 10.1645/14-622.1
Zeisset I, 2014, PLOS ONE, V9, DOI 10.1371/journal.pone.0100176
Zukal J, 2014, PLOS ONE, V9, DOI 10.1371/journal.pone.0097224
NR 100
TC 14
Z9 16
U1 0
U2 43
PU BMC
PI LONDON
PA CAMPUS, 4 CRINAN ST, LONDON N1 9XW, ENGLAND
SN 1471-2148
J9 BMC EVOL BIOL
JI BMC Evol. Biol.
PD OCT 26
PY 2016
VL 16
AR 229
DI 10.1186/s12862-016-0802-1
PG 19
WC Evolutionary Biology; Genetics & Heredity
WE Science Citation Index Expanded (SCI-EXPANDED)
SC Evolutionary Biology; Genetics & Heredity
GA DZ7EU
UT WOS:000386028400001
PM 27782798
OA Green Published, gold
DA 2024-10-21
ER

PT J
AU Becker, DJ
Nachtmann, C
Argibay, HD
Botto, G
Escalera-Zamudio, M
Carrera, JE
Tello, C
Winiarski, E
Greenwood, AD
Méndez-Ojeda, ML
Loza-Rubio, E
Lavergne, A

de Thoisy, B
   Czirják, GA
   Plowright, RK
   Altizer, S
   Streicker, DG
AF Becker, Daniel J.
   Nachtmann, Cecilia
   Argibay, Hernan D.
   Botto, German
   Escalera-Zamudio, Marina
   Carrera, Jorge E.
   Tello, Carlos
   Winiarski, Erik
   Greenwood, Alex D.
   Mendez-Ojeda, Maria L.
   Loza-Rubio, Elizabeth
   Lavergne, Anne
   de Thoisy, Benoit
   Czirjak, Gabor A.
   Plowright, Raina K.
   Altizer, Sonia
   Streicker, Daniel G.
TI Leukocyte Profiles Reflect Geographic Range Limits in a
Widespread
   Neotropical Bat
SO INTEGRATIVE AND COMPARATIVE BIOLOGY
LA English
DT Article; Proceedings Paper
CT Symposium on Allometry, Scaling, and Ontogeny of Form / Symposium
on
   Scale of Sickness - How Immune Variation across Space and Species
   Affects Infectious Disease Dynamics at the Annual Meeting of the
   Society-of-Integrative-and-Comparative-Biology
CY JAN 03-07, 2019
CL Tampa, FL
SP Soc Integrat & Comparat BIol
ID VAMPIRE BATS; ECOLOGICAL IMMUNOLOGY; PARASITE INFECTION; IMMUNE
   FUNCTION; DISEASE; STRESS; BLOOD; VERTEBRATES; EXPRESSION;
RESPONSES
AB Synopsis Quantifying how the environment shapes host immune
defense is important for understanding which wild populations may be
more susceptible or resistant to pathogens. Spatial variation in
parasite risk, food and predator abundance, and abiotic conditions
can each affect immunity, and these factors can also manifest at
both local and biogeographic scales. Yet identifying predictors and
the spatial scale of their effects is limited by the rarity of
studies that measure immunity across many populations of broadly
distributed species. We analyzed leukocyte profiles from 39 wild
populations of the common vampire bat (Desmodus rotundus) across its
wide geographic range throughout the Neotropics. White blood cell
differentials varied spatially, with proportions of neutrophils and
lymphocytes varying up to six-fold across sites. Leukocyte profiles
were spatially autocorrelated at small and very large distances,
suggesting that local environment and large-scale biogeographic

factors influence cellular immunity. Generalized additive models showed that bat populations closer to the northern and southern limits of the species range had more neutrophils, monocytes, and basophils, but fewer lymphocytes and eosinophils, than bats sampled at the core of their distribution. Habitats with access to more livestock also showed similar patterns in leukocyte profiles, but large-scale patterns were partly confounded by time between capture and sampling across sites. Our findings suggest that populations at the edge of their range experience physiologically limiting conditions that predict higher chronic stress and greater investment in cellular innate immunity. High food abundance in livestock-dense habitats may exacerbate such conditions by increasing bat density or diet homogenization, although future spatially and temporally coordinated field studies with common protocols are needed to limit sampling artifacts. Systematically assessing immune function and response over space will elucidate how environmental conditions influence traits relevant to epidemiology and help predict disease risks with anthropogenic disturbance, land conversion, and climate change.
C1 [Becker, Daniel J.; Nachtmann, Cecilia; Altizer, Sonia; Streicker, Daniel G.] Univ Georgia, Odum Sch Ecol, Athens, GA 30602 USA.
[Becker, Daniel J.; Altizer, Sonia] Univ Georgia, Ctr Ecol Infect Dis, Athens, GA 30602 USA.
[Becker, Daniel J.] Indiana Univ, Dept Biol, Bloomington, IN 47405 USA.
[Argibay, Hernan D.] Univ Buenos Aires, Fac Ciencias Exactas & Nat, Dept Ecol Genet & Evoluc, C1428EGA, Buenos Aires, DF, Argentina.
[Botto, German; Plowright, Raina K.] Montana State Univ, Dept Microbiol & Immunol, Bozeman, MT 59715 USA.
[Botto, German] Univ Republica, Fac Med, Dept Metodos Cuantitat, Montevideo 11800, Uruguay.
[Escalera-Zamudio, Marina; Czirjak, Gabor A.] Leibniz Inst Zoo & Wildlife Res, Dept Wildlife Dis, D-10315 Berlin, Germany.
[Escalera-Zamudio, Marina] Univ Oxford, Dept Zool, Oxford OX1 3SY, England.
[Carrera, Jorge E.] Univ Nacl Piura, Fac Ciencias, Piura 20009, Peru.
[Carrera, Jorge E.] Programa Conservac Murcielagos, Lima 1, Peru.
[Tello, Carlos] Assoc Conservat & Dev Nat Resources, Lima 15037, Peru.
[Tello, Carlos] Yunkawasi, Lima 15049, Peru.
[Winiarski, Erik] Univ Republica, Fac Med, Dept Histol, Montevideo 11800, Uruguay.
[Greenwood, Alex D.] Free Univ Berlin, Dept Vet Med, D-14163 Berlin, Germany.
[Mendez-Ojeda, Maria L.] Univ Veracruzana, Fac Med Vet & Zootecnia, Xalapa 91710, Veracruz, Mexico.
[Loza-Rubio, Elizabeth] Inst Nacl Invest Forestales Agr & Pecuarias, Ctr Nacl Invest Disciplinaria Microbiol Anim, Mexico City 05110, DF, Mexico.
[Lavergne, Anne; de Thoisy, Benoit] Inst Pasteur Guyane, Lab Interact Virus Hotes, F-97300 Cayenne, French Guiana, France.

[Streicker, Daniel G.] Univ Glasgow, Coll Med Vet & Life Sci, Inst Biodivers Anim Hlth & Comparat Med, Glasgow G12 8QQ, Lanark, Scotland.
[Streicker, Daniel G.] Univ Glasgow, Ctr Virus Res, MRC, Glasgow G61 1QH, Lanark, Scotland.
C3 University System of Georgia; University of Georgia; University System
of Georgia; University of Georgia; Indiana University System; Indiana
University Bloomington; University of Buenos Aires; Montana State
University System; Montana State University Bozeman; Universidad de la
Republica, Uruguay; Leibniz Institut fur Zoo und Wildtierforschung;
University of Oxford; Universidad Nacional de Piura; Universidad de la
Republica, Uruguay; Free University of Berlin; Universidad Veracruzana;
Universite Paris Cite; University of Glasgow; University of Glasgow
RP Becker, DJ (corresponding author), Univ Georgia, Odum Sch Ecol, Athens, GA 30602 USA.; Becker, DJ (corresponding author), Univ Georgia, Ctr Ecol Infect Dis, Athens, GA 30602 USA.; Becker, DJ (corresponding author), Indiana Univ, Dept Biol, Bloomington, IN 47405 USA.
EM danbeck@iu.edu
RI Czirják, Gábor/F-5440-2011; Botto Nuñez, German/HKN-3801-2023;
Greenwood, Alex/GWU-8292-2022; Argibay, Hernan Dario/IUO-5497-2023;
Becker, Daniel/A-6466-2014
OI Carrera Guardia, Jorge Eduardo/0000-0001-6644-4518; Czirjak, Gabor
Arpad/0000-0001-9488-0069; de thoisy, benoit/0000-0002-8420-5112;
Argibay, Hernan Dario/0000-0001-7640-961X; Botto Nunez,
German/0000-0002-4055-9277; Becker, Daniel/0000-0003-4315-8628; Tello,
Carlos/0000-0003-3758-265X; Lavergne, Anne/0000-0002-5304-5574;
Escalera-Zamudio, Marina/0000-0002-4773-2773
FU National Science Foundation [DEB 1601052, DEB-1020966, DEB-1716698];
ARCS Foundation; Sigma Xi; Animal Behavior Society; Bat Conservation
International; American Society of Mammalogists; University of Georgia
(UGA); Explorer's Club; Roemmers Foundation; Fulbright Commission;
Deutsche Forschungsgemeinschaft [GR 3924/9-1]; Consejo Nacional de
Ciencia y Tecnologia of Mexico; German Academic Exchange Service
[311664]; Leibniz Institute for Zoo and Wildlife Research; National
Institutes of Health IDeA Program [P20GM103474, P30GM110732]; Defense
Advanced Research Projects Agency [D16AP00113, D18AC00031]; Sir

Henry
   Dale Fellowship (Wellcome Trust) [102507/Z/13/Z]; Sir Henry Dale
   Fellowship (Royal Society) [102507/Z/13/Z]; MRC [MC_UU_12014/8]
Funding
   Source: UKRI
FX This work was supported by the National Science Foundation
(Graduate
   Research Fellowship, DEB 1601052, DEB-1020966, DEB-1716698), ARCS
   Foundation, Sigma Xi, Animal Behavior Society, Bat Conservation
   International, American Society of Mammalogists, University of
Georgia
   (UGA), Explorer's Club, Roemmers Foundation, Fulbright
Commission,
   Deutsche Forschungsgemeinschaft (GR 3924/9-1), Consejo Nacional
de
   Ciencia y Tecnologia of Mexico and German Academic Exchange
Service
   (311664), Leibniz Institute for Zoo and Wildlife Research,
National
   Institutes of Health IDeA Program (P20GM103474 and P30GM110732),
Defense
   Advanced Research Projects Agency (Young Faculty Award D16AP00113
and
   PREEMPT award D18AC00031; the content of the information does not
   necessarily reflect the position or the policy of the U.S.
government,
   and no official endorsement should be inferred), and a Sir Henry
Dale
   Fellowship (jointly funded by the Wellcome Trust and Royal
Society;
   102507/Z/13/Z).
CR Acevedo-Whitehouse K, 2009, PHILOS T R SOC B, V364, P3429, DOI
10.1098/rstb.2009.0128
   Adelman JS, 2013, AM NAT, V181, P674, DOI 10.1086/670024
   Albery GF, 2018, BIORXIV
   Allen LC, 2009, J COMP PHYSIOL B, V179, P315, DOI 10.1007/
s00360-008-0315-3
   [Anonymous], 2013, MuMIn: Multi-model inference. R package
version 1.10.0. 1
   [Anonymous], 2010, BIOGEOGRAPHY HOST PA
   Ardia DR, 2005, ECOLOGY, V86, P2040, DOI 10.1890/04-1619
   Argibay HD, 2016, PARASITOLOGY, V143, P1358, DOI 10.1017/
S0031182016000925
   Baptista M, 1997, REV CIENT I PESQ GON, V3, P11
   Barquez R, 2015, IUCN RED LIST THREAT
   Becker DJ, 2019, PHYSIOL BIOCHEM ZOOL, V92, P37, DOI
10.1086/701069
   Becker DJ, 2018, PLOS NEGLECT TROP D, V12, DOI 10.1371/
journal.pntd.0006786
   Becker DJ, 2018, PHILOS T R SOC B, V373, DOI 10.1098/
rstb.2017.0089
   Beechler BR, 2012, PHYSIOL BIOCHEM ZOOL, V85, P255, DOI
10.1086/665276
   Bergner LM, 2019, MOL ECOL RESOUR, V19, P128, DOI

10.1111/1755-0998.12946
Bivand RS, 2008, USE R, P1
Bjornstad ON, 2001, ENVIRON ECOL STAT, V8, P53, DOI 10.1023/A:1009601932481
Bobrowiec PED, 2015, J MAMMAL, V96, P54, DOI 10.1093/jmammal/gyu002
Bokony V, 2009, AM NAT, V173, P589, DOI 10.1086/597610
Brearley G, 2013, BIOL REV, V88, P427, DOI 10.1111/brv.12009
BREIDENSTEIN CP, 1982, J MAMMAL, V63, P482, DOI 10.2307/1380446
Brook CE, 2015, TRENDS MICROBIOL, V23, P172, DOI 10.1016/j.tim.2014.12.004
Buehler DM, 2011, PLOS ONE, V6, DOI 10.1371/journal.pone.0018592
Corby-Harris V, 2008, J ANIM ECOL, V77, P768, DOI 10.1111/j.1365-2656.2008.01399.x
Couch CS, 2008, MAR BIOL, V155, P281, DOI 10.1007/s00227-008-1024-x
Davis AK, 2008, FUNCT ECOL, V22, P760, DOI 10.1111/j.1365-2435.2008.01467.x
de Thoisy B, 2016, PLOS NEGLECT TROP D, V10, DOI 10.1371/journal.pntd.0004378
Eberle JU, 2016, SEMIN IMMUNOPATHOL, V38, P605, DOI 10.1007/s00281-016-0563-3
Escalera-Zamudio M, 2018, ECOHEALTH, V15, P132, DOI 10.1007/s10393-017-1297-y
Ezenwa VO, 2004, INT J PARASITOL, V34, P535, DOI 10.1016/j.ijpara.2003.11.012
Fassbinder-Orth CA, 2014, INTEGR COMP BIOL, V54, P396, DOI 10.1093/icb/icu023
Frick WF, 2015, GLOBAL ECOL BIOGEOGR, V24, P741, DOI 10.1111/geb.12290
Garamszegi LZ., 2014, Modern phylogenetic comparative methods and their application in evolutionary biology
Gilbert M, 2018, SCI DATA, V5, DOI 10.1038/sdata.2018.227
GREENHALL A M, 1983, Mammalian Species, P1, DOI 10.2307/3503895
Hawley DM, 2011, FUNCT ECOL, V25, P48, DOI 10.1111/j.1365-2435.2010.01753.x
Hijmans RJ, 2005, INT J CLIMATOL, V25, P1965, DOI 10.1002/joc.1276
Houwen B., 2001, LAB HEMATOL, V7, P89, DOI DOI 10.1182/ASHEDUCATION-2012.1.475
Jolles AE, 2015, PARASITE IMMUNOL, V37, P255, DOI 10.1111/pim.12153
Jones Gareth, 2009, Endangered Species Research, V8, P93, DOI 10.3354/esr00182
Keeling MJ, 1997, SCIENCE, V275, P65, DOI 10.1126/science.275.5296.65
Koenig WD, 1999, TRENDS ECOL EVOL, V14, P22, DOI 10.1016/S0169-5347(98)01533-X
Kradin RL., 2017, DIAGNOSTIC PATHOLOGY
Krama T, 2013, J ORNITHOL, V154, P1019, DOI 10.1007/s10336-013-0969-9
Lee DN, 2012, PLOS ONE, V7, DOI 10.1371/journal.pone.0042466
Lewontin R., 2000, Capitalism Nat. Social, V11, P103
Lochmiller RL, 2000, OIKOS, V88, P87, DOI 10.1034/

j.1600-0706.2000.880110.x
Maceda-Veiga A, 2015, SCI TOTAL ENVIRON, V514, P322, DOI 10.1016/j.scitotenv.2015.02.004
Martin LB, 2010, ANN NY ACAD SCI, V1195, P129, DOI 10.1111/j.1749-6632.2010.05454.x
Martin LB, 2009, GEN COMP ENDOCR, V163, P70, DOI 10.1016/j.ygcen.2009.03.008
Martinez L., 1939, 2 CONTRIBUCION ACERC, P109
Matson KD, 2006, P ROY SOC B-BIOL SCI, V273, P815, DOI 10.1098/rspb.2005.3376
MAXWELL MH, 1992, BRIT POULTRY SCI, V33, P177, DOI 10.1080/00071669208417455
MCNAB BK, 1973, J MAMMAL, V54, P131, DOI 10.2307/1378876
Minias P, 2018, FUNCT ECOL, V32, P369, DOI 10.1111/1365-2435.12991
Moller AP, 1998, OIKOS, V82, P265, DOI 10.2307/3546966
Murray MH, 2016, BIOL CONSERV, V204, P163, DOI 10.1016/j.biocon.2016.10.034
Paradis E, 2004, BIOINFORMATICS, V20, P289, DOI [10.1093/bioinformatics/btg412, 10.1093/bioinformatics/bty633]
Phelps KL, 2018, OECOLOGIA, V188, P41, DOI 10.1007/s00442-018-4179-2
Raats M. M., 1992, Food Quality and Preference, V3, P89, DOI 10.1016/0950-3293(91)90028-D
Romero LM, 2005, COMP BIOCHEM PHYS A, V140, P73, DOI 10.1016/j.cbpb.2004.11.004
Romero LM, 2000, GEN COMP ENDOCR, V118, P113, DOI 10.1006/gcen.1999.7446
Schinnerl M, 2011, J ZOO WILDLIFE MED, V42, P382, DOI 10.1638/2010-0060.1
Schneeberger K, 2013, PLOS ONE, V8, DOI 10.1371/journal.pone.0054023
Schneider MC, 2009, REV PANAM SALUD PUBL, V25, P260, DOI 10.1590/S1020-49892009000300010
Seltmann A, 2017, CONSERV PHYSIOL, V5, DOI 10.1093/conphys/cox020
Stephens PR, 2016, ECOL LETT, V19, P1159, DOI 10.1111/ele.12644
STEVENS GC, 1989, AM NAT, V133, P240, DOI 10.1086/284913
Streicker DG, 2016, P NATL ACAD SCI USA, V113, P10926, DOI 10.1073/pnas.1606587113
Streicker DG, 2016, J APPL ECOL, V53, P1280, DOI 10.1111/1365-2664.12690
Streicker DG, 2010, SCIENCE, V329, P676, DOI 10.1126/science.1188836
Tizard I.R., 2012, Veterinary Immunology
TOBLER WR, 1970, ECON GEOGR, V46, P234, DOI 10.2307/143141
Tong SX, 2013, PLOS PATHOG, V9, DOI 10.1371/journal.ppat.1003657
Tschirren B, 2015, BIOL LETTERS, V11, DOI 10.1098/rsbl.2015.0263
Vilar TD, 2005, REV U RURAL, V25, P57
Volokhov D V, 2017, Epidemiol Infect, V145, P3154, DOI [10.1017/s095026881700231x, 10.1017/S095026881700231X]
Walsh Sr, 2005, J SURG ONCOL, V91, P181, DOI 10.1002/jso.20329
Wood S. N., 2017, Generalized Additive Models: An Introduction with R, DOI [10.1201/9781315370279, DOI 10.1201/9781315370279]
Zhang ZQ, 2015, INTEGR ZOOL, V10, P302, DOI

10.1111/1749-4877.12133
   Zhou P, 2016, P NATL ACAD SCI USA, V113, P2696, DOI 10.1073/
pnas.1518240113
NR 81
TC 20
Z9 21
U1 0
U2 12
PU OXFORD UNIV PRESS INC
PI CARY
PA JOURNALS DEPT, 2001 EVANS RD, CARY, NC 27513 USA
SN 1540-7063
EI 1557-7023
J9 INTEGR COMP BIOL
JI Integr. Comp. Biol.
PD NOV
PY 2019
VL 59
IS 5
BP 1176
EP 1189
DI 10.1093/icb/icz007
PG 14
WC Zoology
WE Science Citation Index Expanded (SCI-EXPANDED); Conference
Proceedings Citation Index - Science (CPCI-S)
SC Zoology
GA KD1BY
UT WOS:000507607800005
PM 30873523
OA Green Published, hybrid, Green Accepted
DA 2024-10-21
ER

PT J
AU Moreno-Santillán, DD
   Machain-Williams, C
   Hernández-Montes, G
   Ortega, J
AF Moreno-Santillan, Diana D.
   Machain-Williams, Carlos
   Hernandez-Montes, Georgina
   Ortega, Jorge
TI Transcriptomic analysis elucidates evolution of the major
   histocompatibility complex class I in neotropical bats
SO JOURNAL OF MAMMALOGY
LA English
DT Article
DE bats; immune response; major histocompatibility complex; positive
   selection; trans-specific polymorphism
ID FRUIT BATS; PERFORMANCE; RESERVOIRS; SELECTION; PLATFORM;
PROTEIN;
   GENOME
AB The Order Chiroptera comprises more than 1,400 species, each with

its evolutionary history and under unique selective pressures, among which are the host-pathogen interactions. Bats have coped with complex interactions with a broad spectrum of microbes throughout their evolutionary history, prompting the development of unique adaptations that allow them to co-exist with microbes with pathogenic potential more efficiently than other nonadapted species. In this sense, an extraordinary immune system with unique adaptations has been hypothesized in bats. To explore this, we focused on the major histocompatibility complex (MHC), which plays a crucial role in pathogen recognition and presentation to T cells to trigger the adaptive immune response. We analyzed MHC class I transcripts in five species, each from different families of New World bats. From RNA-seq data, we assembled a partial region of the MHC-I comprising the alpha 1 and alpha 2 domains, which are responsible for peptide binding and recognition. We described five putative functional variants, two of which have two independent insertions at the alpha 2 domain. Our results suggest that this insertion appeared after the divergence of the order Chiroptera and may have an adaptive function in the defense against intracellular pathogens, providing evidence of positive selection and trans-specific polymorphism on the peptide-binding sites.
C1 [Moreno-Santillan, Diana D.] Univ Calif Berkeley, Dept Integrat Biol, Berkeley, CA 94720 USA.
[Machain-Williams, Carlos] Univ Autanoma Yucatan, Lab Arbovirol, Merida 97000, Yucatan, Mexico.
[Hernandez-Montes, Georgina] Univ Nacl Autonoma Mexico, Red Apoyo Invest, Coordinac Invest Cient Entre Univ & Red Apoyo, Ciudad De Mexico 14080, Mexico.
[Ortega, Jorge] Inst Politecn Nacl, Escuela Nacl Ciencias Biol, Dept Zool, Posgrad Ciencias Quimicobiol, Ciudad De Mexico 11350, Mexico.
C3 University of California System; University of California Berkeley;
Universidad Autonoma de Yucatan; Universidad Nacional Autonoma de Mexico; Instituto Politecnico Nacional - Mexico
RP Ortega, J (corresponding author), Inst Politecn Nacl, Escuela Nacl Ciencias Biol, Dept Zool, Posgrad Ciencias Quimicobiol, Ciudad De Mexico 11350, Mexico.
EM artibeus2@aol.com
RI Hernńadez, Georgina/JDC-7211-2023
CR Ahn M, 2016, SCI REP-UK, V6, DOI 10.1038/srep21722
Allocati N, 2016, CELL DEATH DISCOV, V2, DOI 10.1038/cddiscovery.2016.48
Andrews S., 2010, FastQC: A Quality Control Tool for High Throughput Sequence Data
[Anonymous], 2000, Revista Mexicana de Mastozoologa
Azevedo L, 2015, HUM GENOMICS, V9, DOI 10.1186/s40246-015-0043-1
Banerjee A, 2019, VIRUSES-BASEL, V11, DOI 10.3390/v11010041
Banerjee A, 2017, SCI REP-UK, V7, DOI 10.1038/s41598-017-01513-w
Calisher CH, 2006, CLIN MICROBIOL REV, V19, P531, DOI 10.1128/CMR.00017-06
Chattopadhyay B, 2020, GENOME BIOL EVOL, V12, P259, DOI 10.1093/gbe/evaa030
Finn RD, 2016, NUCLEIC ACIDS RES, V44, pD279, DOI 10.1093/nar/

gkv1344
Grubaugh ND, 2019, NAT MICROBIOL, V4, P10, DOI 10.1038/s41564-018-0296-2
Guindon S, 2010, SYST BIOL, V59, P307, DOI 10.1093/sysbio/syq010
Haas BJ, 2013, NAT PROTOC, V8, P1494, DOI 10.1038/nprot.2013.084
Hughes AL, 1998, ANNU REV GENET, V32, P415, DOI 10.1146/annurev.genet.32.1.415
Janeway C A., 2012, Immunobiology: The Immune System in Health and Disease, V8th
Jebb D, 2020, NATURE, V583, P578, DOI 10.1038/s41586-020-2486-3
Katoh Kazutaka, 2013, Mol Biol Evol, V30, P772, DOI 10.1093/molbev/mst010
Kearse M, 2012, BIOINFORMATICS, V28, P1647, DOI 10.1093/bioinformatics/bts199
Klein J, 1998, ANNU REV ECOL SYST, V29, P1, DOI 10.1146/annurev.ecolsys.29.1.1
Kunz TH, 2011, ANN NY ACAD SCI, V1223, P1, DOI 10.1111/j.1749-6632.2011.06004.x
Leendertz SAJ, 2016, ECOHEALTH, V13, P18, DOI 10.1007/s10393-015-1053-0
Lefort V, 2017, MOL BIOL EVOL, V34, P2422, DOI 10.1093/molbev/msx149
Leroy EM, 2005, NATURE, V438, P575, DOI 10.1038/438575a
Leroy EM, 2009, VECTOR-BORNE ZOONOT, V9, P723, DOI 10.1089/vbz.2008.0167
Letko M, 2020, NAT REV MICROBIOL, V18, P461, DOI 10.1038/s41579-020-0394-z
Li B, 2011, BMC BIOINFORMATICS, V12, DOI 10.1186/1471-2105-12-323
Li WZ, 2006, BIOINFORMATICS, V22, P1658, DOI 10.1093/bioinformatics/btl158
Lu D, 2019, PLOS BIOL, V17, DOI 10.1371/journal.pbio.3000436
Maccari G, 2018, IMMUNOGENETICS, V70, P619, DOI 10.1007/s00251-018-1072-4
Martin M., 2011, EMBNET J, V17, P10, DOI [10.14806/ej.17.1.200, DOI 10.14806/EJ.17.1.200, 10.14806/EJ.17.1.200]
Meredith RW, 2011, SCIENCE, V334, P521, DOI 10.1126/science.1211028
Santillán DDM, 2021, MOL ECOL, V30, P6449, DOI 10.1111/mec.16027
Moreno-Santillán DD, 2019, SCI REP-UK, V9, DOI 10.1038/s41598-019-42560-9
Moreno-Santillán DD, 2016, PLOS ONE, V11, DOI 10.1371/journal.pone.0141296
Murrell B, 2012, PLOS GENET, V8, DOI 10.1371/journal.pgen.1002764
Negredo A, 2011, PLOS PATHOG, V7, DOI 10.1371/journal.ppat.1002304
Ng JHJ, 2016, SCI REP-UK, V6, DOI 10.1038/srep21256
Papenfuss AT, 2012, BMC GENOMICS, V13, DOI 10.1186/1471-2164-13-261
Pavlovich SS, 2018, CELL, V173, P1098, DOI 10.1016/j.cell.2018.03.070
Penn DJ., 2002, Encycl. Life Sci, P1
Peterson AT, 2004, EMERG INFECT DIS, V10, P40, DOI 10.3201/eid1001.030125
Qu ZH, 2019, J IMMUNOL, V202, P3493, DOI 10.4049/jimmunol.1900001

Rached L. Abi, 1999, Immunological Reviews, V167, P33, DOI 10.1111/j.1600-065X.1999.tb01380.x
Radwan J, 2020, TRENDS GENET, V36, P298, DOI 10.1016/j.tig.2020.01.008
Mello MAR, 2015, OIKOS, V124, P1031, DOI 10.1111/oik.01613
Riccucci Marco, 2014, Vespertilio, V17, P161
Sanchez R., 2007, J NAT HIST, V41, P25
Schountz T, 2014, VIRUSES-BASEL, V6, P4880, DOI 10.3390/v6124880
Shapiro JT, 2021, VIRUSES-BASEL, V13, DOI 10.3390/v13071356
Sikes RS, 2016, J MAMMAL, V97, P663, DOI 10.1093/jmammal/gyw078
Sommer Simone, 2005, Frontiers in Zoology, V2, P1, DOI 10.1186/1742-9994-2-16
Teeling E.C., 2008, HDB SIGNAL PROCESSIN, P1835
Teeling EC, 2005, SCIENCE, V307, P580, DOI 10.1126/science.1105113
Tesicky M, 2015, J IMMUNOL RES, V2015, DOI 10.1155/2015/838035
van Oosterhout Cock, 2009, Communicative & Integrative Biology, V2, P408
Wynne JW, 2016, J IMMUNOL, V196, P4468, DOI 10.4049/jimmunol.1502062
Yang ZH, 2005, MOL BIOL EVOL, V22, P1107, DOI 10.1093/molbev/msi097
Yang ZH, 2007, MOL BIOL EVOL, V24, P1586, DOI 10.1093/molbev/msm088
Zhang GJ, 2013, SCIENCE, V339, P456, DOI 10.1126/science.1230835
Zhang T, 2020, CURR BIOL, V30, P1346, DOI 10.1016/j.cub.2020.03.022
Zhou P, 2020, NATURE, V579, P270, DOI [10.1038/s41586-020-2012-7, 10.1038/s41586-020-2951-z]
Zhou P, 2016, P NATL ACAD SCI USA, V113, P2696, DOI 10.1073/pnas.1518240113
NR 62
TC 0
Z9 0
U1 0
U2 2
PU OXFORD UNIV PRESS INC
PI CARY
PA JOURNALS DEPT, 2001 EVANS RD, CARY, NC 27513 USA
SN 0022-2372
EI 1545-1542
J9 J MAMMAL
JI J. Mammal.
PD OCT 14
PY 2022
VL 103
IS 5
BP 1084
EP 1093
DI 10.1093/jmammal/gyac052
EA JUN 2022
PG 10
WC Zoology
WE Science Citation Index Expanded (SCI-EXPANDED)

SC Zoology
GA 5H7XH
UT WOS:000856506200001
DA 2024-10-21
ER

PT J
AU Kumar, V
AF Kumar, V
TI Understanding the complexities of SARS-CoV2 infection and its
   immunology: A road to immune-based therapeutics
SO INTERNATIONAL IMMUNOPHARMACOLOGY
LA English
DT Article
DE SARS-CoV2; COVID-19; Immune response; Immunomodulation; Cytokines
ID RESPIRATORY SYNDROME CORONAVIRUS; NF-KAPPA-B; CONVERTING ENZYME TYPE-2;
   IFN-GAMMA-PRODUCTION; DENDRITIC CELLS; INFLAMMATORY RESPONSES;
   ENDOPLASMIC-RETICULUM; STRUCTURAL BASIS; BAT CORONAVIRUS; CLEAVAGE SITE
AB Emerging infectious diseases always pose a threat to humans along with plant and animal life. SARS-CoV2 is the recently emerged viral infection that originated from Wuhan city of the Republic of China in December 2019. Now, it has become a pandemic. Currently, SARS-CoV2 has infected more than 27.74 million people worldwide, and taken 901,928 human lives. It was named first 'WH 1 Human CoV' and later changed to 2019 novel CoV (2019-nCoV). Scientists have established it as a zoonotic viral disease emerged from Chinese horseshoe bats, which do not develop a severe infection. For example, Rhinolophus Chinese horseshoe bats harboring severe acute respiratory syndrome-related coronavirus (SARSr-CoV) or SARSr-Rh-BatCoV appear healthy and clear the virus within 2-4 months period. The article introduces first the concept of EIDs and some past EIDs, which have affected human life. Next section discusses mysteries regarding SARS-CoV2 origin, its evolution, and human transfer. Third section describes COVID-19 clinical symptoms and factors affecting susceptibility or resistance. The fourth section introduces the SARS-CoV2 entry in the host cell, its replication, and the establishment of productive infection. Section five describes the host's immune response associated with asymptomatic, symptomatic, mild to moderate, and severe COVID-19. The subsequent seventh and eighth sections mention the immune status in COVID-19 convalescent patients and re-emergence of COVID-19 in them. Thereafter, the eighth section describes viral strategies to hijack the host antiviral immune response and generate the "cytokine storm". The ninth section describes about transgenic humane ACE2 (hACE2) receptor expressing mice to study immunity, drugs, and vaccines. The article ends with the development of different immunomodulatory and immunotherapeutics strategies, including vaccines waiting for their approval in humans as prophylaxis or treatment measures.
C1 [Kumar, V] Univ Queensland, Fac Med, Sch Clin Med, Childrens Hlth Queensland Clin Unit,Mater Res, Brisbane, Qld 4078, Australia.
   [Kumar, V] Univ Queensland, Fac Med, Sch Biomed Sci, Brisbane, Qld 4078, Australia.

C3 University of Queensland; Mater Research; University of Queensland
RP Kumar, V (corresponding author), Univ Queensland, Fac Med, Childrens Hlth Clin Unit, Mater Res, Brisbane, Qld 4078, Australia.
EM vij_tox@yahoo.com
RI Kumar, Vijay/O-6315-2014
OI Kumar, Vijay/0000-0001-9741-3597
CR Ahn J, 2019, EXP MOL MED, V51, DOI 10.1038/s12276-019-0333-0
Akhtar H, 2020, GYNECOL OBSTET INVES, V85, P295, DOI 10.1159/000509290
Al-Omari A, 2019, DIAGN MICR INFEC DIS, V93, P265, DOI 10.1016/j.diagmicrobio.2018.10.011
Allen IC, 2011, IMMUNITY, V34, P854, DOI 10.1016/j.immuni.2011.03.026
Allocati N, 2016, CELL DEATH DISCOV, V2, DOI 10.1038/cddiscovery.2016.48
[Anonymous], 2020, NAT MED, DOI DOI 10.1038/s41591-020-0944-y
[Anonymous], 2020, BIORXIV
Arunachalam PS, 2020, SCIENCE, V369, P1210, DOI 10.1126/science.abc6261
Atyeo C., IMMUNITY
BAGGIOLINI M, 1994, ADV IMMUNOL, V55, P97
Bais C, 1998, NATURE, V391, P86, DOI 10.1038/34193
Bao L, 2020, NATURE, V583, P830
Bao Y, 2014, CELL RES, V24, P161, DOI 10.1038/cr.2013.155
Barnes CO, 2020, CELL, V182, P828, DOI [10.1016/j.cell.2020.06.025, 10.1101/2020.05.28.121533]
BARRESINOUSSI F, 1983, SCIENCE, V220, P868, DOI 10.1126/science.6189183
Barron E, 2020, LANCET DIABETES ENDO, V8, P813, DOI 10.1016/S2213-8587(20)30272-2
Baseler L, 2017, ANNU REV PATHOL-MECH, V12, P387, DOI 10.1146/annurev-pathol-052016-100506
Baud D, 2020, JAMA-J AM MED ASSOC, V323, P2198, DOI 10.1001/jama.2020.7233
Behr FM, 2019, FRONT IMMUNOL, V10, DOI 10.3389/fimmu.2019.00400
Beigel JH, 2020, NEW ENGL J MED, V383, P1813, DOI 10.1056/NEJMoa2007764
Bevan MJ, 2004, NAT REV IMMUNOL, V4, P595, DOI 10.1038/nri1413
Bian H, 2020, MEPLAZUMAB TREATS CO, DOI 10.1101/2020.03.21.20040691
Blanco-Melo D, 2020, CELL, V181, P1036, DOI 10.1016/j.cell.2020.04.026
Bloch EM, 2020, J CLIN INVEST, V130, P2757, DOI 10.1172/JCI138745
Boada-Romero E, 2020, NAT REV MOL CELL BIO, V21, P398, DOI 10.1038/s41580-020-0232-1
Boni MF, 2020, NAT MICROBIOL, V5, P1408, DOI 10.1038/s41564-020-0771-4
Bosch BJ, 2008, J VIROL, V82, P8887, DOI 10.1128/JVI.00415-08
Bosque A, 2005, EUR J IMMUNOL, V35, P1812, DOI 10.1002/eji.200526046
Bost P, 2020, CELL, V181, P1475, DOI 10.1016/j.cell.2020.05.006
Boulware DR, 2020, NEW ENGL J MED, V383, P517, DOI 10.1056/NEJMoa2016638

Braga FAV, 2015, EUR J IMMUNOL, V45, P2945, DOI 10.1002/eji.201545650
Branch DR, 2015, TRANSFUSION, V55, pS74, DOI 10.1111/trf.13087
Brann DH, 2020, SCI ADV, V6, DOI 10.1126/sciadv.abc5801
Braun J, 2020, NATURE, V587, P270, DOI 10.1038/s41586-020-2598-9
Bronte V, 2020, J CLIN INVEST, V130, P6409, DOI 10.1172/JCI141772
Brook CE, 2015, TRENDS MICROBIOL, V23, P172, DOI 10.1016/j.tim.2014.12.004
Brumeanu T.D., 2020, BIORXIV
Byun HJ, 2007, CELL BIOL INT, V31, P257, DOI 10.1016/j.cellbi.2006.11.002
Cagliani R, 2020, J VIROL, V94, DOI 10.1128/JVI.00411-20
Cao WCC, 2007, NEW ENGL J MED, V357, P1162, DOI 10.1056/NEJMc070348
Casadevall A, 2020, J CLIN INVEST, V130, P1545, DOI 10.1172/JCI138003
Case JB, 2020, CELL HOST MICROBE, V28, P465, DOI 10.1016/j.chom.2020.07.018
Cavalcanti AB, 2020, NEW ENGL J MED, V383, P2041, DOI 10.1056/NEJMoa2019014
Centers for Disease Control (CDC), 1981, MMWR Morb Mortal Wkly Rep, V30, P305
Chai X, 2020, bioRxiv, DOI [10.1101/2020.02.03.931766, 10.1101/2020.02.03.931766, DOI 10.1101/2020.02.03.931766, 10.1101/2020.02.03.931766v1]
Chang SC, 2005, CLIN DIAGN LAB IMMUN, V12, P1455, DOI 10.1128/CDLI.12.12.1455-1457.2005
Chen C., 2020, CLIN TRANSL MED
Chen G, 2020, J CLIN INVEST, V130, P2620, DOI 10.1172/JCI137244
Chen HJ, 2020, LANCET, V395, P809, DOI 10.1016/S0140-6736(20)30360-3
Chen IY, 2015, TRENDS MICROBIOL, V23, P55, DOI 10.1016/j.tim.2014.09.007
Chen N., 2020, Lancet, V395, P507, DOI [10.1016/S0140-6736(20)30211-7, DOI 10.1016/S0140-6736(20)30211-7]
Chen T, 2020, BMJ-BRIT MED J, V368, DOI [10.1136/bmj.m1295, 10.1136/bmj.m1091]
Cheng YF, 2005, JAMA-J AM MED ASSOC, V293, P1450, DOI 10.1001/jama.293.12.1450-c
Cho E, 2012, ELECTROCHEM COMMUN, V22, P1, DOI 10.1016/j.elecom.2012.05.018
Cordova ZM, 2016, ONCOTARGET, V7, P54392, DOI 10.18632/oncotarget.11106
Cottam EM, 2011, AUTOPHAGY, V7, P1335, DOI 10.4161/auto.7.11.16642
Coutard B, 2020, ANTIVIR RES, V176, DOI 10.1016/j.antiviral.2020.104742
Cugno M., J ALLERGY CLIN IMMUN
Cush SS, 2007, J IMMUNOL, V179, P141, DOI 10.4049/jimmunol.179.1.141
da Silva HB, 2020, IMMUNITY, V53, P158, DOI 10.1016/j.immuni.2020.06.010
da Silva IP, 2014, CANCER IMMUNOL RES, V2, P410, DOI 10.1158/2326-6066.CIR-13-0171

Davis SM, 2018, J NEUROINFLAMM, V15, DOI 10.1186/s12974-018-1326-y
de Lartigue J, 2009, TRAFFIC, V10, P883, DOI 10.1111/j.1600-0854.2009.00915.x
De Luca G., LANCET RHEUMATOLOGY
Di Mascio D, 2020, AM J OBST GYNEC MFM, V2, DOI 10.1016/j.ajogmf.2020.100107
Diao B, 2020, FRONT IMMUNOL, V11, DOI 10.3389/fimmu.2020.00827
Dimopoulos G, 2020, CELL HOST MICROBE, V28, P117, DOI 10.1016/j.chom.2020.05.007
Dinnon KH III, 2020, NATURE, V586, P560, DOI 10.1038/s41586-020-2708-8
Dolin R, 2020, NEW ENGL J MED, V383, P1886, DOI 10.1056/NEJMe2018715
Dong C, 2008, NAT REV IMMUNOL, V8, P337, DOI 10.1038/nri2295
Dong R, 2020, GENES-BASEL, V11, DOI 10.3390/genes11060637
Eaglesham JB, 2020, CURR OPIN IMMUNOL, V66, P27, DOI 10.1016/j.coi.2020.04.002
Eastman RT, 2020, ACS CENTRAL SCI, V6, P672, DOI 10.1021/acscentsci.0c00489
Ellinghaus D, 2020, NEW ENGL J MED, V383, P1522, DOI 10.1056/NEJMoa2020283
Escher R, 2020, THROMB RES, V190, P62, DOI 10.1016/j.thromres.2020.04.014
Fan C, 2020, ACE2 EXPRESSION KIDN, DOI 10.1101/2020.02.12.20022418
Fauver JR, 2020, CELL, V181, P990, DOI 10.1016/j.cell.2020.04.021
Feeley EM, 2011, PLOS PATHOG, V7, DOI 10.1371/journal.ppat.1002337
Ferguson CJ, 2009, HUM MOL GENET, V18, P4868, DOI 10.1093/hmg/ddp460
Fleming DT, 1997, NEW ENGL J MED, V337, P1105, DOI 10.1056/NEJM199710163371601
Folegatti PM, 2020, LANCET, V396, P467, DOI 10.1016/S0140-6736(20)31604-4
Forsman A, 2008, J VIROL, V82, P12069, DOI 10.1128/JVI.01379-08
Fransen F, 2015, IMMUNITY, V43, P527, DOI 10.1016/j.immuni.2015.08.011
Franzetti M, 2020, INT J INFECT DIS, V97, P215, DOI 10.1016/j.ijid.2020.05.050
Frucht DM, 2001, TRENDS IMMUNOL, V22, P556, DOI 10.1016/S1471-4906(01)02005-1
Frumento G, 2019, FRONT IMMUNOL, V10, DOI 10.3389/fimmu.2019.00468
Fung TS, 2019, ANNU REV MICROBIOL, V73, P529, DOI 10.1146/annurev-micro-020518-115759
GALILI U, 1988, INFECT IMMUN, V56, P1730, DOI 10.1128/IAI.56.7.1730-1737.1988
Gao DX, 2013, SCIENCE, V341, P903, DOI 10.1126/science.1240933
Gao Q, 2020, SCIENCE, V369, P77, DOI 10.1126/science.abc1932
Gao T., 2020, HIGHLY PATHOGENIC CO, DOI [DOI 10.1101/2020.03.29.20041962, 10.1101/2020.03.29.20041962]
Ge XY, 2013, NATURE, V503, P535, DOI 10.1038/nature12711
Giamarellos-Bourboulis EJ, 2020, CELL HOST MICROBE, V27, P992,

DOI 10.1016/j.chom.2020.04.009
Girbl T, 2018, IMMUNITY, V49, P1062, DOI 10.1016/j.immuni.2018.09.018
Giudice V, 2020, FRONT PHARMACOL, V11, DOI 10.3389/fphar.2020.00857
Glezen WP, 1996, EPIDEMIOL REV, V18, P64, DOI 10.1093/oxfordjournals.epirev.a017917
Goel S, 2015, NAT MED, V21, P314, DOI 10.1038/nm.3812
Goldstein T, 2018, NAT MICROBIOL, V3, P1084, DOI 10.1038/s41564-018-0227-2
Gong F, 2020, J CLIN INVEST, V130, P6588, DOI 10.1172/JCI141054
Gordon David E, 2020, bioRxiv, DOI 10.1101/2020.03.22.002386
Goubau D, 2013, IMMUNITY, V38, P855, DOI 10.1016/j.immuni.2013.05.007
Gralinski LE, 2018, MBIO, V9, DOI 10.1128/mBio.01753-18
Grein J, 2020, NEW ENGL J MED, V382, P2327, DOI 10.1056/NEJMoa2007016
Grifoni A, 2020, CELL, V181, P1489, DOI 10.1016/j.cell.2020.05.015
Grifoni A, 2020, CELL HOST MICROBE, V27, P671, DOI 10.1016/j.chom.2020.03.002
Gruber C, 2020, NAT REV IMMUNOL, DOI 10.1038/s41577-020-0335-0
Gu JY, 2020, GASTROENTEROLOGY, V158, P1518, DOI 10.1053/j.gastro.2020.02.054
Guan WJ, 2020, NEW ENGL J MED, DOI 10.1056/NEJMoa2002032
Guaraldi G., LANCET RHEUMATOLOGY
Gudbjartsson DF, 2020, NEW ENGL J MED, V383, P1724, DOI 10.1056/NEJMoa2026116
Guo JY, 2020, J AM HEART ASSOC, V9, DOI 10.1161/JAHA.120.016219
Hachim MY, 2020, FRONT IMMUNOL, V11, DOI 10.3389/fimmu.2020.01372
Hadjadj J, 2020, SCIENCE, V369, P718, DOI 10.1126/science.abc6027
Hamilton JA, 2008, NAT REV IMMUNOL, V8, P533, DOI 10.1038/nri2356
Harjunpää H, 2020, CLIN EXP IMMUNOL, V200, P108, DOI 10.1111/cei.13407
Hartmann G, 2017, ADV IMMUNOL, V133, P121, DOI 10.1016/bs.ai.2016.11.001
Hassan AO, 2020, CELL, V182, P744, DOI 10.1016/j.cell.2020.06.011
Hegerova L, 2020, BLOOD, V136, P759, DOI 10.1182/blood.2020006964
Hoffmann M, 2020, CELL, V181, P271, DOI 10.1016/j.cell.2020.02.052
Hoffmann M, 2020, MOL CELL, V78, P779, DOI 10.1016/j.molcel.2020.04.022
Hosier H, 2020, J CLIN INVEST, V130, P4947, DOI 10.1172/JCI139569
Hou YXJ, 2020, CELL, V182, P429, DOI 10.1016/j.cell.2020.05.042
Hu D, 2018, EMERG MICROBES INFEC, V7, DOI 10.1038/s41426-018-0155-5
Huang FH, 2019, MATER CHEM FRONT, V3, P10, DOI 10.1039/c8qm90057d
Huang IC, 2011, PLOS PATHOG, V7, DOI 10.1371/journal.ppat.1001258
Huet T, 2020, LANCET RHEUMATOL, V2, pE393, DOI 10.1016/S2665-9913(20)30164-8
Hung IFN, 2020, LANCET INFECT DIS, V20, P1051, DOI 10.1016/S1473-3099(20)30364-9
Hung IFN, 2020, LANCET, V395, P1695, DOI 10.1016/S0140-6736(20)31042-4

Hung SC, 2018, MICROBES INFECT, V20, P615, DOI 10.1016/j.micinf.2017.09.014
Huo JD, 2020, CELL HOST MICROBE, V28, P445, DOI [10.1016/j.chom.2020.06.010, 10.1016/j.chom.2020.07.002]
Huo JD, 2020, NAT STRUCT MOL BIOL, V27, P846, DOI [10.1038/s41594-020-0469-6, 10.1038/s41594-020-00527-9]
Ibarrondo FJ, 2020, NEW ENGL J MED, V383, P1085, DOI 10.1056/NEJMc2025179
Ishikawa H, 2008, NATURE, V455, P674, DOI 10.1038/nature07317
Ishikawa H, 2009, NATURE, V461, P788, DOI 10.1038/nature08476
Iwanaga Naoki, 2020, bioRxiv, DOI 10.1101/2020.06.15.152157
Jackson LA, 2020, NEW ENGL J MED, V383, P1920, DOI 10.1056/NEJMoa2022483
Jaigirdar SA, 2017, FRONT IMMUNOL, V8, DOI 10.3389/fimmu.2017.01627
Janssen EM, 2005, NATURE, V434, P88, DOI 10.1038/nature03337
Janssens K, 2014, J NEUROIMMUNOL, V275, P155, DOI 10.1016/j.jneuroim.2014.08.414
Ji HL, 2020, PHYSIOL REV, V100, P1065, DOI 10.1152/physrev.00013.2020
Ji W, 2020, J MED VIROL, V92, P433, DOI 10.1002/jmv.25682
Jiang RD, 2020, CELL, V182, P50, DOI 10.1016/j.cell.2020.05.027
Jin Jian-Min, 2020, Front Public Health, V8, P152, DOI 10.3389/fpubh.2020.00152
Jin Z, 2020, FRONT IMMUNOL, V11, DOI 10.3389/fimmu.2020.00263
Jones KE, 2008, NATURE, V451, P990, DOI 10.1038/nature06536
Joyce M Gordon, 2020, bioRxiv, DOI 10.1101/2020.03.15.992883
Joyner MJ, 2020, J CLIN INVEST, V130, P4791, DOI [10.1172/JCI140200, 10.1101/2020.05.12.20099879]
Juan J, 2020, ULTRASOUND OBST GYN, V56, P15, DOI 10.1002/uog.22088
Junttila IS, 2018, FRONT IMMUNOL, V9, DOI 10.3389/fimmu.2018.00888
Kaech SM, 2001, NAT IMMUNOL, V2, P415, DOI 10.1038/87720
Kajani R, 2020, BRAIN BEHAV IMMUN, V88, P28, DOI 10.1016/j.bbi.2020.05.042
Kamitaki N, 2020, NATURE, V582, P577, DOI 10.1038/s41586-020-2277-x
Kasper LH, 2014, ANN CLIN TRANSL NEUR, V1, P622, DOI 10.1002/acn3.84
Kelvin AA, 2020, LANCET INFECT DIS, V20, P633, DOI 10.1016/S1473-3099(20)30236-X
Khan A, 2017, CRIT CARE, V21, DOI 10.1186/s13054-017-1823-x
Khan M, 2020, FRONT IMMUNOL, V11, DOI 10.3389/fimmu.2020.00167
Kilbourne ED, 2006, EMERG INFECT DIS, V12, P9, DOI 10.3201/eid1201.051254
Kindhauser MK, 2016, B WORLD HEALTH ORGAN, V94, P675, DOI 10.2471/BLT.16.171082
Klann K., MOL CELL
Klein Sabra L, 2020, J Clin Invest, V130, P6141, DOI [10.1172/JCI142004, 10.1101/2020.06.26.20139063]
Komatsu T, 1998, CYTOKINE GROWTH F R, V9, P277, DOI 10.1016/S1359-6101(98)00017-3
Kondo T, 2012, BIOCHEM BIOPH RES CO, V422, P501, DOI 10.1016/

j.bbrc.2012.05.028
Kopp E, 1999, GENE DEV, V13, P2059, DOI 10.1101/gad.13.16.2059
Korber B, 2020, CELL, V182, P812, DOI 10.1016/j.cell.2020.06.043
Kragten NAM, 2018, EUR J IMMUNOL, V48, P1644, DOI 10.1002/eji.201847771
Kulkarni R, 2021, INFECTION, V49, P339, DOI 10.1007/s15010-020-01493-6
Kumagai Y, 2011, IMMUNITY, V35, P320, DOI 10.1016/j.immuni.2011.09.003
Kumar V, 2010, INT IMMUNOPHARMACOL, V10, P1325, DOI 10.1016/j.intimp.2010.08.012
Kumar V, 2009, J CHEMOTHERAPY, V21, P159, DOI 10.1179/joc.2009.21.2.159
Kumar V, 2008, EUR J PHARMACOL, V592, P146, DOI 10.1016/j.ejphar.2008.07.019
Kumar V, 2021, INT REV IMMUNOL, V40, P5, DOI 10.1080/08830185.2020.1800688
Kumar V, 2020, FRONT IMMUNOL, V11, DOI 10.3389/fimmu.2020.01722
Kumar V, 2020, INT REV IMMUNOL, V39, P118, DOI 10.1080/08830185.2020.1732958
Kumar V, 2019, J LEUKOCYTE BIOL, V106, P171, DOI 10.1002/JLB.4MIR1018-397RR
Kumar V, 2010, INT IMMUNOPHARMACOL, V10, P777, DOI 10.1016/j.intimp.2010.04.008
Kuri-Cervantes L, 2020, SCI IMMUNOL, V5, DOI 10.1126/sciimmunol.abd7114
Laing AG, 2020, NAT MED, V26, P1623, DOI 10.1038/s41591-020-1038-6
Lam TTY, 2020, NATURE, V583, P282, DOI 10.1038/s41586-020-2169-0
Lamers MM, 2020, SCIENCE, V369, P50, DOI 10.1126/science.abc1669
Lane BR, 2002, J VIROL, V76, P11570, DOI 10.1128/JVI.76.22.11570-11583.2002
Lane BR, 2001, J VIROL, V75, P5812, DOI 10.1128/JVI.75.13.5812-5822.2001
LANGMUIR AD, 1961, AM REV RESPIR DIS, V83, P2
Lau SKP, 2010, J VIROL, V84, P2808, DOI 10.1128/JVI.02219-09
Lee PY, 2013, J LEUKOCYTE BIOL, V94, P585, DOI 10.1189/jlb.0113014
Lee WB, 2019, BMB REP, V52, P133, DOI 10.5483/BMBRep.2019.52.2.129
Lei CQ, 2015, J INNATE IMMUN, V7, P153, DOI 10.1159/000365971
Letko M, 2020, NAT MICROBIOL, V5, P562, DOI [10.1038/s41564-020-0688-y, 10.1101/2020.01.22.915660]
Leverkus M, 2000, BLOOD, V96, P2628
Li XD, 2013, SCIENCE, V341, P1390, DOI 10.1126/science.1244040
Li XG, 2020, J MED VIROL, V92, P602, DOI 10.1002/jmv.25731
Li Y, 2013, MICROBES INFECT, V15, P88, DOI 10.1016/j.micinf.2012.10.008
Li YZ, 2016, P NATL ACAD SCI USA, V113, P2241, DOI 10.1073/pnas.1519657113
Lian SJ, 2016, MEDICINE, V95, DOI 10.1097/MD.0000000000004328
Liao MF, 2020, NAT MED, V26, P842, DOI 10.1038/s41591-020-0901-9
Ligthart S, 2014, ARTERIOSCL THROM VAS, V34, P2695, DOI 10.1161/ATVBAHA.114.304306

Lim Yvonne Xinyi, 2016, Diseases, V4, DOI 10.3390/diseases4030026
Liu JY, 2020, J TRANSL MED, V18, DOI 10.1186/s12967-020-02374-0
Liu K, 2020, CELL DISCOV, V6, DOI 10.1038/s41421-019-0132-8
Liu L, 2020, MICROBES INFECT, V22, P206, DOI 10.1016/j.micinf.2020.05.008
Liu P, 2020, PLOS PATHOG, V16, DOI 10.1371/journal.ppat.1008421
Lo MK, 2017, SCI REP-UK, V7, DOI 10.1038/srep43395
Long Q.X., 2020, NAT MED, V26, P1200, DOI DOI 10.1038/S41591-020-0965-6
LoPresti M, 2020, AM J HUM GENET, V107, P381, DOI 10.1016/j.ajhg.2020.08.007
Lu RJ, 2020, LANCET, V395, P565, DOI 10.1016/S0140-6736(20)30251-8
Luo HB, 2006, INT J BIOCHEM CELL B, V38, P589, DOI 10.1016/j.biocel.2005.10.022
Ma Y, 2020, MICROBES INFECT, V22, P212, DOI 10.1016/j.micinf.2020.04.011
MacLean O A., 2020, bioRxiv, DOI 10.1101/2020.05.28.122366.
Magram J, 1996, IMMUNITY, V4, P471, DOI 10.1016/S1074-7613(00)80413-6
Magro C, 2020, TRANSL RES, V220, P1, DOI 10.1016/j.trsl.2020.04.007
Maier HJ, 2012, VIRUSES-BASEL, V4, P3440, DOI 10.3390/v4123440
Mangalmurti N, 2020, IMMUNITY, V53, P19, DOI 10.1016/j.immuni.2020.06.017
Mao L, 2020, JAMA NEUROL, V77, P683, DOI 10.1001/jamaneurol.2020.1127
Martinelli I, 2020, THROMB RES, V191, P36, DOI 10.1016/j.thromres.2020.04.022
Mastellos DC, 2019, NAT REV DRUG DISCOV, V18, P707, DOI 10.1038/s41573-019-0031-6
MAURER FD, 1962, J AM VET MED ASSOC, V141, P699
Mazzoni A, 2020, J CLIN INVEST, V130, P4694, DOI [10.1172/JCI138554, 10.1172/JCI138554.]
McCray PB, 2007, J VIROL, V81, P813, DOI 10.1128/JVI.02012-06
Meckiff Benjamin J, 2020, SSRN, P3641939, DOI [10.1101/2020.06.12.148916, 10.2139/ssrn.3641939]
Menachery VD, 2015, PLOS ONE, V10, DOI 10.1371/journal.pone.0131451
Metcalfe SM, 2011, GENES IMMUN, V12, P157, DOI 10.1038/gene.2011.9
MINTY A, 1993, NATURE, V362, P248, DOI 10.1038/362248a0
Mizumoto K, 2020, EUROSURVEILLANCE, V25, P2, DOI 10.2807/1560-7917.ES.2020.25.10.2000180
Monteil V, 2020, CELL, V181, P905, DOI 10.1016/j.cell.2020.04.004
Monteiro JM, 1998, J VIROL, V72, P4825, DOI 10.1128/JVI.72.6.4825-4831.1998
Moreno-Eutimio MA, 2020, MICROBES INFECT, V22, P226, DOI 10.1016/j.micinf.2020.04.009
Morita R, 2011, IMMUNITY, V34, P108, DOI 10.1016/j.immuni.2010.12.012
Morokata T, 1999, IMMUNOLOGY, V98, P345, DOI 10.1046/j.1365-2567.1999.00890.x
MORRIS SC, 1994, J IMMUNOL, V152, P1047

Nagai-Singer MA, 2019, FRONT IMMUNOL, V10, DOI 10.3389/fimmu.2019.02419
Ndow G., 2019, Sociocultural Dimensions of Emerging Infectious Diseases in Africa, P31, DOI [DOI 10.1007/978-3-030-17474-3_3, https://doi.org/10.1007/978-3-030-17474-3_3, DOI 10.1007/978-3-030-17474-3]
Neuman BW, 2014, TRENDS MICROBIOL, V22, P642, DOI 10.1016/j.tim.2014.06.003
Neuman BW, 2011, J STRUCT BIOL, V174, P11, DOI 10.1016/j.jsb.2010.11.021
Ni GX, 2018, PLOS PATHOG, V14, DOI 10.1371/journal.ppat.1007148
Ni L, 2020, IMMUNITY, V52, P971, DOI 10.1016/j.immuni.2020.04.023
Nie Q, 2020, VIRUS RES, V287, DOI 10.1016/j.virusres.2020.198098
Nielsen S.C.A., 2020, Res Sq
O'Connor BP, 2003, IMMUNOL REV, V194, P61, DOI 10.1034/j.1600-065X.2003.00055.x
ORANGE JS, 1994, J IMMUNOL, V152, P1253
Ostrowski LE, 2003, MOL THER, V8, P637, DOI 10.1016/S1525-0016(03)00221-1
Ou XY, 2020, NAT COMMUN, V11, DOI 10.1038/s41467-020-15562-9
Oxenius A, 1999, J IMMUNOL, V162, P965
Pallanti S, 2020, COMPR PSYCHIAT, V100, DOI 10.1016/j.comppsych.2020.152184
Panigada M, 2020, J THROMB HAEMOST, V18, P1738, DOI 10.1111/jth.14850
Papillion A, 2019, SCI IMMUNOL, V4, DOI 10.1126/sciimmunol.aaw7636
Peckham H., 2020, RES SQUARE, DOI [10.21203/rs.3.rs-23651/v1, DOI 10.21203/RS.3.RS-23651/V1]
Pedersen SF, 2020, J CLIN INVEST, V130, P2202, DOI 10.1172/JCI137647
Pelonero AL, 1998, PSYCHIATR SERV, V49, P1163, DOI 10.1176/ps.49.9.1163
Pesu M, 2006, BLOOD, V108, P983, DOI 10.1182/blood-2005-09-3824
Pique-Regi R, 2020, ELIFE, V9, DOI 10.7554/eLife.58716
Pizzorno A, 2020, CELL REP MED, V1, DOI 10.1016/j.xcrm.2020.100059
Pontelli Marjorie C, 2020, bioRxiv, DOI 10.1101/2020.07.28.225912
Post M, 2017, FRONT IMMUNOL, V8, DOI 10.3389/fimmu.2017.00535
Prentice E, 2004, J VIROL, V78, P9977, DOI 10.1128/JVI.78.18.9977-9986.2004
Prentice E, 2004, J BIOL CHEM, V279, P10136, DOI 10.1074/jbc.M306124200
Preston JA, 2004, EUR RESPIR J, V23, P224, DOI 10.1183/09031936.03.00081403
PUDDINGTON L, 1994, IMMUNITY, V1, P733, DOI 10.1016/S1074-7613(94)80015-4
Rabb H, 2020, J CLIN INVEST, V130, P2749, DOI 10.1172/JCI138871
Radzikowska U, 2020, ALLERGY, V75, P2829, DOI 10.1111/all.14429
Ramasamy S, 2016, P NATL ACAD SCI USA, V113, P1871, DOI 10.1073/pnas.1511380113
Ramlall V, 2020, NAT MED, V26, P1609, DOI 10.1038/s41591-020-1021-2
Ratajczak MZ, 2020, STEM CELL REV REP, V16, P1, DOI 10.1007/

s12015-019-09951-x
Rehwinkel J, 2020, NAT REV IMMUNOL, V20, P537, DOI 10.1038/s41577-020-0288-3
Reis FM, 2011, FERTIL STERIL, V95, P176, DOI 10.1016/j.fertnstert.2010.06.060
Ren L., 2020, Lancet, V395, P497, DOI [10.1016/S0140-6736(20)30183-5, DOI 10.1016/S0140-6736(20)30183-5]
Renshaw SA, 2003, J IMMUNOL, V170, P1027, DOI 10.4049/jimmunol.170.2.1027
RICHMOND A, 1986, J CELL PHYSIOL, V129, P375, DOI 10.1002/jcp.1041290316
Risitano AM, 2020, NAT REV IMMUNOL, V20, P343, DOI 10.1038/s41577-020-0320-7
Robbiani DF, 2020, NATURE, V584, P437, DOI 10.1038/s41586-020-2456-9
Rodrigues-Diez RR, 2020, BRIT J PHARMACOL, V177, P4873, DOI 10.1111/bph.15166
Rodriguez L, 2020, CELL REP MED, V1, DOI 10.1016/j.xcrm.2020.100078
Roschewski M, 2020, SCI IMMUNOL, V5, DOI 10.1126/sciimmunol.abd0110
Rowe JA, 2007, P NATL ACAD SCI USA, V104, P17471, DOI 10.1073/pnas.0705390104
Rutherford AC, 2006, J CELL SCI, V119, P3944, DOI 10.1242/jcs.03153
Sage PT, 2016, NAT IMMUNOL, V17, P1436, DOI 10.1038/ni.3578
Sage PT, 2014, J CLIN INVEST, V124, P5191, DOI 10.1172/JCI76861
Sánchez-Cerrillo I, 2020, J CLIN INVEST, V130, P6290, DOI 10.1172/JCI140335
Sattler A, 2020, J CLIN INVEST, V130, DOI 10.1172/JCI140965
Schafer S, 2017, NATURE, V552, P110, DOI 10.1038/nature24676
Schijns VECJ, 1998, J IMMUNOL, V160, P3958
Schlottau K., LANCET MICROBE
Schmidt Fabian, 2020, bioRxiv, DOI [10.1084/jem.20201181, 10.1101/2020.06.08.140871]
Schoch-Spana M, 2001, PUBLIC HEALTH REP, V116, P32, DOI 10.1093/phr/116.S2.32
Schultheiss C, 2020, IMMUNITY, V53, P442, DOI 10.1016/j.immuni.2020.06.024
Schutyser E, 2005, J LEUKOCYTE BIOL, V78, P14, DOI 10.1189/jlb.1204712
Schwartz DA, 2017, ARCH PATHOL LAB MED, V141, P18, DOI [10.5858/arpa.2016-0429-ED, 10.5858/arpa.2016-04]
Scully EP, 2020, NAT REV IMMUNOL, V20, P442, DOI 10.1038/s41577-020-0348-8
Sehra ST, 2020, CLIN INFECT DIS, V71, P2482, DOI 10.1093/cid/ciaa681
Sekine T., 2020, ROBUST T CELL IMMUNI, DOI [10.1101/2020.06.29.174888, DOI 10.1016/J.CELL.2020.08.017]
Sevajol M, 2014, VIRUS RES, V194, P90, DOI 10.1016/j.virusres.2014.10.008
Seydoux E., ANAL SARS COV 2 INFE
Shang J, 2020, NATURE, V581, P221, DOI 10.1038/s41586-020-2179-y
Sharma A, 2020, CELL REP MED, V1, DOI [10.1016/

j.xcrm.2020.100052, 10.1101/2020.04.21.051912]
Sheahan TP, 2020, SCI TRANSL MED, V12, DOI 10.1126/scitranslmed.abb5883
Sheahan TP, 2017, SCI TRANSL MED, V9, DOI 10.1126/scitranslmed.aal3653
Shen CG, 2020, JAMA-J AM MED ASSOC, V323, P1582, DOI 10.1001/jama.2020.4783
Shi R, 2020, NATURE, V584, P120, DOI 10.1038/s41586-020-2381-y
Shisheva A, 2008, CELL BIOL INT, V32, P591, DOI 10.1016/j.cellbi.2008.01.006
Shubin NJ, 2013, CRIT CARE, V17, DOI 10.1186/cc13131
Shubin NJ, 2012, J LEUKOCYTE BIOL, V92, P593, DOI 10.1189/jlb.1211641
Silvin A., Cell
Singh Komudi, 2020, bioRxiv, DOI 10.1101/2020.05.13.092536
Sisman J., PEDIAT INFECT DIS J
Siu YL, 2008, J VIROL, V82, P11318, DOI 10.1128/JVI.01052-08
Skendros P, 2020, J CLIN INVEST, V130, P6151, DOI 10.1172/JCI141374
Smith NL, 2011, CIRCULATION, V123, P1864, DOI 10.1161/CIRCULATIONAHA.110.009480
Song JW, 2020, CELL METAB, V32, P188, DOI 10.1016/j.cmet.2020.06.016
Spence JS, 2019, NAT CHEM BIOL, V15, P259, DOI 10.1038/s41589-018-0213-2
Starr Tyler N, 2020, bioRxiv, DOI [10.1016/j.cell.2020.08.012, 10.1101/2020.06.17.157982]
Sun J, 2020, CELL, V182, P734, DOI 10.1016/j.cell.2020.06.010
Sun SH, 2020, CELL HOST MICROBE, V28, P124, DOI 10.1016/j.chom.2020.05.020
Sungnak W, 2020, NAT MED, V26, P681, DOI 10.1038/s41591-020-0868-6
Suthar MS, 2020, CELL REP MED, V1, DOI [10.1016/j.xcrm.2020.100040, 10.1101/2020.05.03.20084442]
Tabata S, 2020, LANCET INFECT DIS, V20, P1043, DOI 10.1016/S1473-3099(20)30482-5
Takahashi T., 2020, NATURE, V588, P315, DOI [10.1038/s41586-020-2700-3, DOI 10.1038/s41586-020-2700-3]
Tan CW, 2020, NAT BIOTECHNOL, V38, P1073, DOI 10.1038/s41587-020-0631-z
Tang FSM, 2016, RESPIROLOGY, V21, P172, DOI 10.1111/resp.12657
Tang W., 2020, High Performance Computing for Geospatial Applications, P1, DOI [10.1101/2020.04.10.20060558, 10.1136/bmj.m1849]
Tchesnokov EP, 2019, VIRUSES-BASEL, V11, DOI 10.3390/v11040326
Thevarajan I, 2020, NAT MED, V26, P453, DOI 10.1038/s41591-020-0819-2
Tilocca B, 2020, MICROBES INFECT, V22, P182, DOI 10.1016/j.micinf.2020.05.013
Timoteo RP, 2017, MEDIAT INFLAMM, V2017, DOI 10.1155/2017/7151285
Tong L, 2020, ADV THEOR SIMUL, V3, DOI 10.1002/adts.202000024
Torabi A, 2020, ACS CHEM NEUROSCI, V11, P1909, DOI 10.1021/acschemneuro.0c00249
Totura AL, 2015, MBIO, V6, DOI 10.1128/mBio.00638-15

Ulrich H, 2020, STEM CELL REV REP, V16, P434, DOI 10.1007/s12015-020-09976-7
Unger JP, 2021, INT J HEALTH SERV, V51, P37, DOI 10.1177/0020731420946590
Upadhyay G, 2019, FRONT IMMUNOL, V10, DOI 10.3389/fimmu.2019.00819
Ursini-Siegel J, 2002, J IMMUNOL, V169, P5505, DOI 10.4049/jimmunol.169.10.5505
V'kovski P, 2015, VIRUS RES, V202, P33, DOI 10.1016/j.virusres.2014.12.021
Vaira LA, 2020, INT FORUM ALLERGY RH, V10, P1103, DOI 10.1002/alr.22593
van Gisbergen KPJM, 2005, FEBS LETT, V579, P6159, DOI 10.1016/j.febslet.2005.09.089
van Gisbergen KPJM, 2005, J EXP MED, V201, P1281, DOI 10.1084/jem.20041276
Vaz-Silva J, 2009, REPROD SCI, V16, P247, DOI 10.1177/1933719108327593
Viboud C, 2016, J INFECT DIS, V213, P738, DOI 10.1093/infdis/jiv534
Vijay K, 2018, INT IMMUNOPHARMACOL, V59, P391, DOI 10.1016/j.intimp.2018.03.002
Vivanti AJ, 2020, NAT COMMUN, V11, DOI 10.1038/s41467-020-17436-6
Wakeham J, 2000, INFECT IMMUN, V68, P6946, DOI 10.1128/IAI.68.12.6946-6953.2000
Walls A C., 2020, Cell
Walls AC, 2020, CELL, V181, P281, DOI [10.1016/j.cell.2020.02.058, 10.1016/j.cell.2020.11.032]
Wan YS, 2020, J VIROL, V94, DOI [10.1128/JVI.00127-20, 10.1128/JVI.02015-19]
Wang C, 2009, NAT IMMUNOL, V10, P744, DOI 10.1038/ni.1742
[王科雯 Wang Kewen], 2020, [测绘通报, Bulletin of Surveying and Mapping], P1
Wang N., CELL HOST MICROBE
Wang Q, 2020, CELL, V182, P417, DOI 10.1016/j.cell.2020.05.034
Wang YQ, 2020, J CLIN INVEST, V130, P5235, DOI [10.1172/JCI138759, 10.1172/JCI138759.]
Wang YM, 2020, LANCET, V395, P1569, DOI 10.1016/S0140-6736(20)31022-9
Warren TK, 2016, NATURE, V531, P381, DOI 10.1038/nature17180
Wauters E., 2020, Discriminating Mild from, DOI 10.1101/2020.07.09.196519
Wee YS, 2012, INNATE IMMUN-LONDON, V18, P834, DOI 10.1177/1753425912443392
Weiskopf D, 2020, SCI IMMUNOL, V5, DOI 10.1126/sciimmunol.abd2071
West NR, 2019, FRONT IMMUNOL, V10, DOI 10.3389/fimmu.2019.01093
Wi SM, 2014, J BIOL CHEM, V289, P35205, DOI 10.1074/jbc.M114.597187
Williamson BN, 2020, NATURE, V585, P273, DOI 10.1038/s41586-020-2423-5
Winkler ES, 2020, NAT IMMUNOL, V21, P1327, DOI 10.1038/s41590-020-0778-2
Wolf Y, 2020, NAT REV IMMUNOL, V20, P173, DOI 10.1038/s41577-019-0224-6

Woolhouse M, 2007, CRIT REV MICROBIOL, V33, P231, DOI 10.1080/10408410701647560
Wrapp D, 2020, CELL, V181, P1004, DOI 10.1016/j.cell.2020.04.031
Wrapp D, 2020, SCIENCE, V367, P1260, DOI [10.1126/science.abb2507, 10.1101/2020.02.11.944462]
Wu AP, 2020, CELL HOST MICROBE, V27, P325, DOI 10.1016/j.chom.2020.02.001
Wu F., 2020, NEUTRALIZING ANTIBOD, DOI [10.1101/2020.03.30.20047365, 10.2139/ssrn.3566211]
Wu F, 2020, NATURE, V579, P265, DOI 10.1038/s41586-020-2008-3
Wu JF, 2020, BMJ OPEN DIAB RES CA, V8, DOI 10.1136/bmjdrc-2020-001476
Wu JJ, 2020, IMMUNITY, V53, P115, DOI 10.1016/j.immuni.2020.06.009
Wu JJ, 2019, J EXP MED, V216, P867, DOI 10.1084/jem.20182192
Wu ZY, 2020, JAMA-J AM MED ASSOC, V323, P1239, DOI 10.1001/jama.2020.2648
Xia S, 2020, CELL MOL IMMUNOL, V17, P765, DOI 10.1038/s41423-020-0374-2
Xia XJ, 2011, IMMUNITY, V34, P843, DOI 10.1016/j.immuni.2011.02.022
Xia XY, 2020, BLOOD, V136, P755, DOI 10.1182/blood.2020007079
Xu H, 2020, INT J ORAL SCI, V12, DOI [10.1038/s41368-020-0074-X, 10.1007/s40820-020-00498-y, 10.1038/s41368-020-0074-x]
Ye S, 2012, J BIOL CHEM, V287, P26740, DOI 10.1074/jbc.M112.383265
Yoshimoto FK, 2020, PROTEIN J, V39, P198, DOI 10.1007/s10930-020-09901-4
Young BE, 2020, LANCET, V396, P603, DOI 10.1016/S0140-6736(20)31757-8
Yuan M, 2020, ISCIENCE, V23, DOI 10.1016/j.isci.2020.101273
Yuan M, 2020, SCIENCE, V368, P630, DOI 10.1126/science.abb7269
Yuen KS, 2020, CELL BIOSCI, V10, DOI 10.1186/s13578-020-00404-4
Zang RC, 2020, SCI IMMUNOL, V5, DOI 10.1126/sciimmunol.abc3582
Zhang BC, 2020, FRONT MOL BIOSCI, V7, DOI 10.3389/fmolb.2020.00157
Zhang H, 2020, MATH PROBL ENG, V2020, DOI 10.1155/2020/7204859
Zhang JR, 2020, B ENG GEOL ENVIRON, V79, P959, DOI 10.1007/s10064-019-01595-y
Zhang Lizhou, 2020, bioRxiv, DOI 10.1101/2020.06.12.148726
ZHANG Q, 2019, FRONT ENDOCRINOL, V11, P525, DOI DOI 10.1080/17565529.2018.1442808
Zhang T, 2020, J MATER CIVIL ENG, V32, DOI 10.1061/(ASCE)MT.1943-5533.0003063
Zhang T, 2020, CURR BIOL, V30, P1346, DOI 10.1016/j.cub.2020.03.022
Zhang Y., 2020, ORF8 PROTEIN SARS CO, DOI [DOI 10.1101/2020.05.24.111823, 10.1101/2020.05.24.111823]
Zhang Y., 2020, MATTER, V2, P1, DOI DOI 10.1007/s42768-020-00033-0
Zhang ZN, 2020, MOL IMMUNOL, V122, P38, DOI 10.1016/j.molimm.2020.03.022
Zhao B, 2020, ADV FUNCT MATER, V30, DOI 10.1002/adfm.202002019
Zhao CY, 2020, FRONT IMMUNOL, V11, DOI 10.3389/fimmu.2020.00211

Zhao XS, 2020, J VIROL, V94, DOI 10.1128/JVI.00562-20
Zheng MJ, 2020, CELL MOL IMMUNOL, V17, P533, DOI 10.1038/s41423-020-0402-2
Zhou DM, 2020, NAT STRUCT MOL BIOL, V27, P950, DOI 10.1038/s41594-020-0480-y
Zhou F, 2020, LANCET, V395, P1054, DOI 10.1016/S0140-6736(20)30566-3
Zhou H, 2020, CURR BIOL, V30, P2196, DOI 10.1016/j.cub.2020.05.023
Zhou J, 2020, NAT MED, V26, P1077, DOI 10.1038/s41591-020-0912-6
Zhou Run-hong, 2020, Immunity, V53, P864, DOI 10.1016/j.immuni.2020.07.026
Zhou Y., 2020, medRxiv, DOI [10.1101/2020.02.12. 945576, 10.1101/2020.02.12.945576]
Zhou Z, 2020, CELL HOST MICROBE, V27, P883, DOI 10.1016/j.chom.2020.04.017
Zhu FC, 2020, LANCET, V396, P479, DOI 10.1016/S0140-6736(20)31605-6
Zhu L, 2020, CARDIORENAL MED, V10, P470, DOI 10.1159/000510916
Zhu LX, 2020, MOL IMMUNOL, V122, P124, DOI 10.1016/j.molimm.2020.04.006
Ziegler CGK, 2020, CELL, V181, P1016, DOI 10.1016/j.cell.2020.04.035
Zlotnik A, 2012, IMMUNITY, V36, P705, DOI 10.1016/j.immuni.2012.05.008
Zou X, 2020, FRONT MED-PRC, V14, P185, DOI 10.1007/s11684-020-0754-0
NR 403
TC 26
Z9 28
U1 0
U2 26
PU ELSEVIER
PI AMSTERDAM
PA RADARWEG 29, 1043 NX AMSTERDAM, NETHERLANDS
SN 1567-5769
EI 1878-1705
J9 INT IMMUNOPHARMACOL
JI Int. Immunopharmacol.
PD NOV
PY 2020
VL 88
AR 106980
DI 10.1016/j.intimp.2020.106980
PG 37
WC Immunology; Pharmacology & Pharmacy
WE Science Citation Index Expanded (SCI-EXPANDED)
SC Immunology; Pharmacology & Pharmacy
GA OP6AD
UT WOS:000588166300130
PM 33182073
OA Green Published
DA 2024-10-21
ER

PT J
AU Sun, HJ
Wang, JY
Xing, YT
Pan, YH
Mao, XG
AF Sun, Haijian
Wang, Jiaying
Xing, Yutong
Pan, Yi-Hsuan
Mao, Xiuguang
TI Gut transcriptomic changes during hibernation in the greater horseshoe
bat (<i>Rhinolophus ferrumequinum</i>)
SO FRONTIERS IN ZOOLOGY
LA English
DT Article
DE RNA-Seq; Food intake; Immune system; Intestine; Mammals
ID GROUND-SQUIRREL; FOOD-INTAKE; IMMUNE-SYSTEM; AGRP NEURONS; PROTEIN;
GENE; EXPRESSION; PEPTIDE; PACKAGE; LEPTIN
AB Background The gut is the major organ for nutrient absorption and immune response in the body of animals. Although effects of fasting on the gut functions have been extensively studied in model animals (e.g. mice), little is known about the response of the gut to fasting in a natural condition (e.g. hibernation). During hibernation, animals endure the long term of fasting and hypothermia. Results Here we generated the first gut transcriptome in a wild hibernating bat (Rhinolophus ferrumequinum). We identified 1614 differentially expressed genes (DEGs) during four physiological states (Torpor, Arousal, Winter Active and Summer Active). Gene co-expression network analysis assigns 926 DEGs into six modules associated with Torpor and Arousal. Our results reveal that in response to the stress of luminal nutrient deficiency during hibernation, the gut helps to reduce food intake by overexpressing genes (e.g.CCKandGPR17) that regulate the sensitivity to insulin and leptin. At the same time, the gut contributes energy supply by overexpressing genes that increase capacity for ketogenesis (HMGCS2) and selective autophagy (TEX264). Furthermore, we identified separate sets of multiple DEGs upregulated in Torpor and Arousal whose functions are involved in innate immunity. Conclusion This is the first gut transcriptome of a hibernating mammal. Our study identified candidate genes associated with regulation of food intake and enhance of innate immunity in the gut during hibernation. By comparing with previous studies, we found that two DEGs (CPEandHSPA8) were also significantly elevated during torpor in liver and brain ofR. ferrumequinumand several DEGs (e.g.TXNIPandPDK1/4) were commonly upregulated during torpor in multiple tissues of different mammals. Our results support that shared expression changes may underlie the hibernation phenotype by most mammals.
C1 [Sun, Haijian; Mao, Xiuguang] East China Normal Univ, Sch Ecol & Environm Sci, Shanghai 200062, Peoples R China.

[Wang, Jiaying; Xing, Yutong] East China Normal Univ, Inst Estuarine & Coastal Res, Shanghai 200062, Peoples R China.
[Pan, Yi-Hsuan] East China Normal Univ, Sch Life Sci, Minist Educ, Key Lab Brain Funct Genom, Shanghai 200062, Peoples R China.
[Mao, Xiuguang] East China Normal Univ, Inst Ecochongming IEC, Shanghai 200062, Peoples R China.
C3 East China Normal University; East China Normal University; East China
Normal University; East China Normal University
RP Mao, XG (corresponding author), East China Normal Univ, Sch Ecol & Environm Sci, Shanghai 200062, Peoples R China.; Mao, XG (corresponding author), East China Normal Univ, Inst Ecochongming IEC, Shanghai 200062, Peoples R China.
EM xgmao6721@gmail.com
RI Pan, Yi-Hsuan/ABG-6271-2020
OI Pan, Yi-Hsuan/0000-0001-5156-9887; mao, xiuguang/ 0000-0002-3432-7509
FU National Natural Science Foundation of China [31570378]
FX This work was supported by the National Natural Science Foundation of
China (No. 31570378).
CR An H, 2019, MOL CELL, V74, P891, DOI 10.1016/j.molcel.2019.03.034
Andrews MT, 2019, J EXP BIOL, V222, DOI 10.1242/jeb.160606
[Anonymous], 2003, HORSESHOE BATS WORLD
Ashburner M, 2000, NAT GENET, V25, P25, DOI 10.1038/75556
Bado A, 1998, NATURE, V394, P790, DOI 10.1038/29547
Bai L, 2019, CELL, V179, P1129, DOI 10.1016/j.cell.2019.10.031
Banerjee S, 2018, ANTIOXIDANTS-BASEL, V7, DOI 10.3390/ antiox7040055
Banoth B, 2015, ELIFE, V4, DOI 10.7554/eLife.05648
Barrachina MD, 1997, P NATL ACAD SCI USA, V94, P10455, DOI 10.1073/pnas.94.19.10455
Bogren LK, 2017, J COMP PHYSIOL B, V187, P735, DOI 10.1007/ s00360-017-1073-x
Bohr J, 2014, CLIN EXP GASTROENTER, V7, P273, DOI 10.2147/ CEG.S63905
Bolger AM, 2014, BIOINFORMATICS, V30, P2114, DOI 10.1093/ bioinformatics/btu170
Bouma HR, 2010, J LEUKOCYTE BIOL, V88, P619, DOI 10.1189/ jlb.0310174
Buchfink B, 2015, NAT METHODS, V12, P59, DOI 10.1038/nmeth.3176
Cain BM, 1997, ENDOCRINOLOGY, V138, P4034, DOI 10.1210/ en.138.9.4034
CARACENI P, 1994, AM J PHYSIOL, V266, pG799, DOI 10.1152/ ajpgi.1994.266.5.G799
Carey HV, 2014, J THERM BIOL, V44, P78, DOI 10.1016/ j.jtherbio.2014.02.015
Carey HV, 2013, AM J PHYSIOL-REG I, V304, pR33, DOI 10.1152/ ajpregu.00387.2012
Carey HV, 2003, PHYSIOL REV, V83, P1153, DOI 10.1152/ physrev.00008.2003
Carey HV, 2001, GUT, V49, P459, DOI 10.1136/gut.49.4.459
CAREY HV, 1991, AM J PHYSIOL, V261, pG37, DOI 10.1152/ ajpgi.1991.261.1.G37

Chino H, 2019, MOL CELL, V74, P909, DOI 10.1016/j.molcel.2019.03.033
Choi JS, 2015, MUCOSAL IMMUNOL, V8, P1285, DOI 10.1038/mi.2015.19
Cooper ST, 2016, PHYSIOL GENOMICS, V48, P513, DOI 10.1152/physiolgenomics.00120.2015
Cordomi A, 2016, CURR OPIN PHARMACOL, V31, P63, DOI 10.1016/j.coph.2016.09.001
CRAWLEY JN, 1994, PEPTIDES, V15, P731, DOI 10.1016/0196-9781(94)90104-X
Cummings DE, 2007, J CLIN INVEST, V117, P13, DOI 10.1172/JCI30227
Delanghe JR, 2001, CLIN CHIM ACTA, V312, P13, DOI 10.1016/S0009-8981(01)00586-1
Dockray GJ, 2013, CURR OPIN PHARMACOL, V13, P954, DOI 10.1016/j.coph.2013.09.007
Donaldson GP, 2016, NAT REV MICROBIOL, V14, P20, DOI 10.1038/nrmicro3552
Ebersole JL, 2000, PERIODONTOL 2000, V23, P19, DOI 10.1034/j.1600-0757.2000.2230103.x
Faherty SL, 2018, MOL ECOL, V27, P709, DOI 10.1111/mec.14483
Faherty SL, 2016, GENOME BIOL EVOL, V8, P2413, DOI 10.1093/gbe/evw163
Field KA, 2018, MOL ECOL, V27, P3727, DOI 10.1111/mec.14827
Florant GL, 2012, J COMP PHYSIOL B, V182, P451, DOI 10.1007/s00360-011-0630-y
Geiser F, 2004, ANNU REV PHYSIOL, V66, P239, DOI 10.1146/annurev.physiol.66.032102.115105
Grabherr MG, 2011, NAT BIOTECHNOL, V29, P644, DOI 10.1038/nbt.1883
Grosse J, 2014, P NATL ACAD SCI USA, V111, P11133, DOI 10.1073/pnas.1411413111
Hahm S, 1999, NEURON, V23, P537, DOI 10.1016/S0896-6273(00)80806-5
Hahm S, 2002, J NEUROSCI, V22, P6929
Harris RA, 2002, ADV ENZYME REGUL, V42, P249, DOI 10.1016/S0065-2571(01)00061-9
Hooper LV, 2002, ANNU REV NUTR, V22, P283, DOI 10.1146/annurev.nutr.22.011602.092259
Huang BL, 2002, DIABETES, V51, P276, DOI 10.2337/diabetes.51.2.276
HURLBERT SH, 1984, ECOL MONOGR, V54, P187, DOI 10.2307/1942661
Janseng HT, 2019, COMMUN BIOL, V2, DOI 10.1038/s42003-019-0574-4
Jethwa PH, 2007, ENDOCRINOLOGY, V148, P4044, DOI 10.1210/en.2007-0038
Kim MS, 2006, NAT NEUROSCI, V9, P901, DOI 10.1038/nn1731
Kitamura T, 2006, NAT MED, V12, P534, DOI 10.1038/nm1392
KRAEHENBUHL JP, 1992, PHYSIOL REV, V72, P853, DOI 10.1152/physrev.1992.72.4.853
Kurtz CC, 2007, DEV COMP IMMUNOL, V31, P415, DOI 10.1016/j.dci.2006.07.003
Langfelder P, 2008, BIOINFORMATICS, V24, P719, DOI 10.1093/bioinformatics/btm563
Langfelder P, 2008, BMC BIOINFORMATICS, V9, DOI 10.1186/1471-2105-9-559
Langfelder P, 2013, PLOS ONE, V8, DOI 10.1371/

journal.pone.0061505
Langmead B, 2012, NAT METHODS, V9, P357, DOI [10.1038/NMETH.1923, 10.1038/nmeth.1923]
Leek JT, 2012, BIOINFORMATICS, V28, P882, DOI 10.1093/bioinformatics/bts034
Lei M, 2014, PLOS ONE, V9, DOI 10.1371/journal.pone.0107746
Levi A, 2004, CELL MOL NEUROBIOL, V24, P517, DOI 10.1023/B:CEMN.0000023627.79947.22
Li B, 2011, BMC BIOINFORMATICS, V12, DOI 10.1186/1471-2105-12-323
Li WZ, 2006, BIOINFORMATICS, V22, P1658, DOI 10.1093/bioinformatics/btl158
Lilley TM, 2019, OECOLOGIA, V191, P295, DOI 10.1007/s00442-019-04499-6
Long AT, 2016, J THROMB HAEMOST, V14, P427, DOI 10.1111/jth.13235
Love MI, 2014, GENOME BIOL, V15, DOI 10.1186/s13059-014-0550-8
Maniero GD, 2002, DEV COMP IMMUNOL, V26, P563, DOI 10.1016/S0145-305X(02)00006-X
Marakasova ES, 2017, BIOESSAYS, V39, DOI 10.1002/bies.201700014
Martin SL, 2008, AM J PHYSIOL-REG I, V295, pR316, DOI 10.1152/ajpregu.00418.2007
Mathew AG, 2001, BIOTECHNOLOGY FEED I
McCue MD, 2010, COMP BIOCHEM PHYS A, V156, P1, DOI 10.1016/j.cbpa.2010.01.002
MORAN TH, 1995, CRIT REV NEUROBIOL, V9, P1
Mugahid DA, 2019, SCI REP-UK, V9, DOI 10.1038/s41598-019-56007-8
Nespolo RF, 2018, MOL ECOL, V27, P4489, DOI 10.1111/mec.14876
Olivier E, 2000, BIOCHEM J, V350, P589, DOI 10.1042/0264-6021:3500589
Ombrellino M, 2001, SHOCK, V15, P181, DOI 10.1097/00024382-200115030-00004
Paksuz EP, 2014, ACTA HISTOCHEM, V116, P1480, DOI 10.1016/j.acthis.2014.10.004
PEIKIN SR, 1989, GASTROENTEROL CLIN N, V18, P757
Plum L, 2009, NAT MED, V15, P1195, DOI 10.1038/nm.2026
Prendergast BJ, 2002, AM J PHYSIOL-REG I, V282, pR1054, DOI 10.1152/ajpregu.00562.2001
Quaye IK, 2008, T ROY SOC TROP MED H, V102, P735, DOI 10.1016/j.trstmh.2008.04.010
Ren HX, 2012, CELL, V149, P1314, DOI 10.1016/j.cell.2012.04.032
Robinson MD, 2010, BIOINFORMATICS, V26, P139, DOI 10.1093/bioinformatics/btp616
SCARPIGNATO C, 1993, J PHYSIOL-PARIS, V87, P291, DOI 10.1016/0928-4257(93)90035-R
Schwartz C, 2013, PLOS ONE, V8, DOI 10.1371/journal.pone.0058427
Schwartz MW, 2000, NATURE, V404, P661, DOI 10.1038/35007534
Shamay A, 2005, J DAIRY SCI, V88, P569, DOI 10.3168/jds.S0022-0302(05)72719-3
Simao FA, 2015, BIOINFORMATICS, V31, P3210, DOI 10.1093/bioinformatics/btv351
Stawski C, 2014, J ZOOL, V292, P86, DOI 10.1111/jzo.12105
Stutchfield BM, 2015, GASTROENTEROLOGY, V149, P1896, DOI 10.1053/j.gastro.2015.08.053
Szklarczyk D, 2019, NUCLEIC ACIDS RES, V47, pD607, DOI [10.1093/nar/gky1131, 10.1093/nar/gkac1000]

   Tseng CF, 2004, PROTEOMICS, V4, P2221, DOI 10.1002/pmic.200300787
   van Breukelen F, 2015, PHYSIOLOGY, V30, P273, DOI 10.1152/physiol.00010.2015
   Varela L, 2012, EMBO REP, V13, P1079, DOI 10.1038/embor.2012.174
   Volkow ND, 2011, TRENDS COGN SCI, V15, P37, DOI 10.1016/j.tics.2010.11.001
   Wang YH, 1997, AM J PHYSIOL-REG I, V273, pR833, DOI 10.1152/ajpregu.1997.273.2.R833
   Wijenayake S, 2017, J THERM BIOL, V69, P199, DOI 10.1016/j.jtherbio.2017.07.010
   Xiao GH, 2019, FRONT MICROBIOL, V10, DOI 10.3389/fmicb.2019.02247
   Xiao YH, 2015, PLOS ONE, V10, DOI 10.1371/journal.pone.0145702
   Yu GC, 2012, OMICS, V16, P284, DOI 10.1089/omi.2011.0118
   Zhang L, 2014, CELL BIOCHEM BIOPHYS, V70, P233, DOI 10.1007/s12013-014-9887-6
   Zhou YD, 2020, CELL DISCOV, V6, DOI 10.1038/s41421-020-0153-3
NR 98
TC 3
Z9 4
U1 2
U2 25
PU BMC
PI LONDON
PA CAMPUS, 4 CRINAN ST, LONDON N1 9XW, ENGLAND
SN 1742-9994
J9 FRONT ZOOL
JI Front. Zool.
PD JUL 17
PY 2020
VL 17
IS 1
AR 21
DI 10.1186/s12983-020-00366-w
PG 14
WC Zoology
WE Science Citation Index Expanded (SCI-EXPANDED)
SC Zoology
GA MP7IX
UT WOS:000552376200001
PM 32690984
OA gold, Green Published
DA 2024-10-21
ER

PT J
AU Harazim, M
   Perrot, J
   Varet, H
   Bourhy, H
   Lannoy, J
   Pikula, J
   Seidlová, V
   Dacheux, L
   Martínková, N

AF Harazim, Marketa
   Perrot, Juliette
   Varet, Hugo
   Bourhy, Herve
   Lannoy, Julien
   Pikula, Jiri
   Seidlova, Veronika
   Dacheux, Laurent
   Martinkova, Natalia
TI Transcriptomic responses of bat cells to European bat lyssavirus 1
   infection under conditions simulating euthermia and hibernation
SO BMC IMMUNOLOGY
LA English
DT Article
DE Chiroptera; Myotis myotis; Hibernation; Lyssaviruses; In vitro infection
   model; EBLV-1; Innate immunity; Heat shock proteins (HSPs); Antiviral
   state; Transcriptome
ID PSEUDOGYMNOASCUS-DESTRUCTANS; EPTESICUS-FUSCUS; IMMUNE-SYSTEM; TGF-BETA;
   RABIES; SURVEILLANCE; MECHANISMS; EVOLUTION; TYPE-1
AB BackgroundCoevolution between pathogens and their hosts decreases host morbidity and mortality. Bats host and can tolerate viruses which can be lethal to other vertebrate orders, including humans. Bat adaptations to infection include localized immune response, early pathogen sensing, high interferon expression without pathogen stimulation, and regulated inflammatory response. The immune reaction is costly, and bats suppress high-cost metabolism during torpor. In the temperate zone, bats hibernate in winter, utilizing a specific behavioural adaptation to survive detrimental environmental conditions and lack of energy resources. Hibernation torpor involves major physiological changes that pose an additional challenge to bat-pathogen coexistence. Here, we compared bat cellular reaction to viral challenge under conditions simulating hibernation, evaluating the changes between torpor and euthermia.ResultsWe infected the olfactory nerve-derived cell culture of Myotis myotis with an endemic bat pathogen, European bat lyssavirus 1 (EBLV-1). After infection, the bat cells were cultivated at two different temperatures, 37 degrees C and 5 degrees C, to examine the cell response during conditions simulating euthermia and torpor, respectively. The mRNA isolated from the cells was sequenced and analysed for differential gene expression attributable to the temperature and/or infection treatment. In conditions simulating euthermia, infected bat cells produce an excess signalling by multitude of pathways involved in apoptosis and immune regulation influencing proliferation of regulatory cell types which can, in synergy with other produced cytokines, contribute to viral tolerance. We found no up- or down-regulated genes expressed in infected cells cultivated at conditions simulating torpor compared to non-infected cells cultivated under the same conditions. When studying the reaction of uninfected cells to the temperature treatment, bat cells show an increased production of heat shock

proteins (HSPs) with chaperone activity, improving the bat's ability to repair molecular structures damaged due to the stress related to the temperature change.ConclusionsThe lack of bat cell reaction to infection in conditions simulating hibernation may contribute to the virus tolerance or persistence in bats. Together with the cell damage repair mechanisms induced in response to hibernation, the immune regulation may promote bats' ability to act as reservoirs of zoonotic viruses such as lyssaviruses.
C1 [Harazim, Marketa; Martinkova, Natalia] Czech Acad Sci, Inst Vertebrate Biol, Kvetna 8, Brno 60300, Czech Republic.
[Harazim, Marketa] Masaryk Univ, Dept Bot & Zool, Kotlarska 2, Brno 61137, Czech Republic.
[Perrot, Juliette; Bourhy, Herve; Lannoy, Julien; Dacheux, Laurent] Univ Paris Cite Lyssavirus, Inst Pasteur, Epidemiol & Neuropathol Unit, 28 rue Docteur Roux, F-75724 Paris 15, France.
[Varet, Hugo] Univ Paris Cite, Inst Pasteur, Bioinformat & Biostat Hub, 28 rue Docteur Roux, F-75724 Paris 15, France.
[Pikula, Jiri; Seidlova, Veronika] Univ Vet Sci Brno, Dept Ecol & Dis Zoo Anim Game Fish & Bees, Palackeho trida 1946-1, Brno 61242, Czech Republic.
[Martinkova, Natalia] Masaryk Univ, RECETOX, Kotlarska 2, Brno 61137, Czech Republic.
C3 Czech Academy of Sciences; Institute of Vertebrate Biology of the Czech
Academy of Sciences; Masaryk University Brno; Pasteur Network;
Universite Paris Cite; Institut Pasteur Paris; Pasteur Network;
Universite Paris Cite; Institut Pasteur Paris; University of Veterinary
Sciences Brno; Masaryk University Brno
RP Harazim, M (corresponding author), Czech Acad Sci, Inst Vertebrate Biol, Kvetna 8, Brno 60300, Czech Republic.; Harazim, M (corresponding author), Masaryk Univ, Dept Bot & Zool, Kotlarska 2, Brno 61137, Czech Republic.
EM harazim@ivb.cz
RI Seidlova, Veronika/HNQ-0598-2023; Dacheux, Laurent/AAJ-5372-2020;
Pikula, Jiri/V-8620-2018; Martínková, Natália/C-2939-2008
FU Czech Science Foundation [17-20286S]; Masaryk University grant
[MUNI/A/1098/2019]; RECETOX Research Infrastructure - Ministry of
Education, Youth and Sports [LM2018121]; Operational Programme Research,
Development and Education (the CETOCOEN EXCELLENCE)
[CZ.02.1.01/0.0/0.0/17_043/0009632]; Institut Pasteur; Ministry of
Education, Youth and Sports of the Czech Republic [e-INFRA CZ
LM2018140]; Barrande fellowship program by the French Ministry of
Foreign Affairs
FX This study was supported by The Czech Science Foundation (17-20286S),
Masaryk University grant MUNI/A/1098/2019, RECETOX Research
Infrastructure (No. LM2018121) financed by the Ministry of Education,
Youth and Sports, and the Operational Programme Research, Development
and Education (the CETOCOEN EXCELLENCE project No.

CZ.02.1.01/0.0/0.0/17_043/0009632), and Institut Pasteur. MetaCentrum
computational resources were supplied by the project"e-Infrastruktura
CZ"(e-INFRA CZ LM2018140) supported by the Ministry of Education, Youth
and Sports of the Czech Republic. MH's stay at Institut Pasteur was
supported by the Barrande fellowship program provided by the French
Ministry of Foreign Affairs.
CR Ahn M, 2019, NAT MICROBIOL, V4, P789, DOI 10.1038/s41564-019-0371-3
Amengual B, 2007, PLOS ONE, V2, DOI 10.1371/journal.pone.0000566
Banerjee A, 2017, SCI REP-UK, V7, DOI 10.1038/s41598-017-01513-w
Begeman L, 2020, PLOS NEGLECT TROP D, V14, DOI 10.1371/journal.pntd.0008898
Bouma HR, 2010, J LEUKOCYTE BIOL, V88, P619, DOI 10.1189/jlb.0310174
Calisher CH, 2006, CLIN MICROBIOL REV, V19, P531, DOI 10.1128/CMR.00017-06
Chang YF, 2007, ANNU REV BIOCHEM, V76, P51, DOI 10.1146/annurev.biochem.76.050106.093909
Chionh YT, 2019, CELL STRESS CHAPERON, V24, P835, DOI 10.1007/s12192-019-01013-y
Clayton E, 2020, FRONT CELL INFECT MI, V10, DOI 10.3389/fcimb.2020.527921
Cokelaer T., 2017, J Open Source Softw, V2, P352, DOI DOI 10.21105/JOSS.00352
Colombi D, 2019, SCI REP-UK, V9, DOI 10.1038/s41598-018-36485-y
Currie SE, 2018, J EXP BIOL, V221, DOI 10.1242/jeb.170894
Dacheux L, 2019, TRANSBOUND EMERG DIS, V66, P2601, DOI 10.1111/tbed.13323
Dacheux L, 2009, EMERG INFECT DIS, V15, P280, DOI 10.3201/eid1502.080637
Davis AD, 2007, J INFECT DIS, V195, P1144, DOI 10.1086/512616
Davis W. H., 1970, P265
Davy CM, 2020, VIRULENCE, V11, P781, DOI 10.1080/21505594.2020.1768018
Delmas O, 2008, PLOS ONE, V3, DOI 10.1371/journal.pone.0002057
Dobin A, 2013, BIOINFORMATICS, V29, P15, DOI 10.1093/bioinformatics/bts635
Eggerbauer E, 2017, PLOS NEGLECT TROP D, V11, DOI 10.1371/journal.pntd.0005668
Fabregat A, 2017, BMC BIOINFORMATICS, V18, DOI 10.1186/s12859-017-1559-2
Fadok VA, 1998, J CLIN INVEST, V101, P890, DOI 10.1172/JCI1112
Faherty SL, 2018, MOL ECOL, V27, P709, DOI 10.1111/mec.14483
Feldman EL, 1997, NEUROBIOL DIS, V4, P201, DOI 10.1006/nbdi.1997.0156
Field KA, 2018, MOL ECOL, V27, P3727, DOI 10.1111/mec.14827
Field KA, 2015, PLOS PATHOG, V11, DOI 10.1371/journal.ppat.1005168
Franka R, 2008, J GEN VIROL, V89, P1998, DOI 10.1099/

vir.0.83688-0
Freuling C, 2009, J GEN VIROL, V90, P2493, DOI 10.1099/vir.0.011510-0
Fritze M, 2019, BIOL OPEN, V8, DOI 10.1242/bio.046078
Fuoco NL, 2018, ANTIVIR RES, V160, P94, DOI 10.1016/j.antiviral.2018.10.023
Ganeshan K, 2019, CELL, V177, P399, DOI 10.1016/j.cell.2019.01.050
Gerow CM, 2019, TRANSBOUND EMERG DIS, V66, P1054, DOI 10.1111/tbed.13102
Gómez JMM, 2016, SCI REP-UK, V6, DOI 10.1038/srep37796
Guito JC, 2021, CURR BIOL, V31, P257, DOI 10.1016/j.cub.2020.10.015
Harazim M, 2021, EVOL ECOL, V35, P291, DOI 10.1007/s10682-020-10096-4
Harazim M, 2018, BMC ZOOL, V3, DOI 10.1186/s40850-018-0035-4
He XC, 2014, PLOS ONE, V9, DOI 10.1371/journal.pone.0109795
Hicks DJ, 2013, J COMP PATHOL, V149, P446, DOI 10.1016/j.jcpa.2013.04.001
Hicks DJ, 2009, J COMP PATHOL, V140, P113, DOI 10.1016/j.jcpa.2008.09.001
Irving AT, 2020, CELL REP, V33, DOI 10.1016/j.celrep.2020.108345
Kurtz CC, 2007, DEV COMP IMMUNOL, V31, P415, DOI 10.1016/j.dci.2006.07.003
Kurtz CC, 2021, COMP BIOCHEM PHYS A, V253, DOI 10.1016/j.cbpa.2020.110875
KUZMIN IV, 1994, VOP VIRUSOL+, V39, P17
Kuzmin IV, 2017, J VIROL, V91, DOI [10.1128/JVI.02471-16, 10.1128/jvi.02471-16]
Lei M, 2014, PLOS ONE, V9, DOI 10.1371/journal.pone.0107746
Leopardi S, 2019, EPIDEMIOL INFECT, P147
Liao Y, 2014, BIOINFORMATICS, V30, P923, DOI 10.1093/bioinformatics/btt656
Logan SM, 2021, CELL SIGNAL, V82, DOI 10.1016/j.cellsig.2021.109975
Love MI, 2014, GENOME BIOL, V15, DOI 10.1186/s13059-014-0550-8
Lyman C. P., 1970, P301
Mareuil F., 2017, F1000Res. 10
Martin M., 2011, EMBNET J, V17, P10, DOI DOI 10.14806/EJ.17.1.200
Martínková N, 2018, VIRULENCE, V9, P1734, DOI 10.1080/21505594.2018.1548685
Müller T, 2004, J VET MED B, V51, P49, DOI 10.1111/j.1439-0450.2003.00725.x
Parize P, 2020, ZOONOSES PUBLIC HLTH, V67, P772, DOI 10.1111/zph.12747
Peirce MJ, 2010, PLOS ONE, V5, DOI 10.1371/journal.pone.0011465
Pfaffl MW, 2001, NUCLEIC ACIDS RES, V29, DOI 10.1093/nar/29.9.e45
Regnault B, 2022, CLIN INFECT DIS, V74, P461, DOI 10.1093/cid/ciab443
Robardet E, 2017, PLOS NEGLECT TROP D, V11, DOI 10.1371/journal.pntd.0006048
Schad J, 2016, IMMUNOGENETICS, V68, P783, DOI 10.1007/s00251-016-0940-z
Schuster N, 2002, CELL TISSUE RES, V307, P1, DOI 10.1007/

s00441-001-0479-6
Seidlova V, 2020, BMC VET RES, V16, DOI 10.1186/s12917-020-02702-y
Serra-Cobo J, 2002, EMERG INFECT DIS, V8, P413, DOI 10.3201/eid0804.010263
Simic I, 2018, BMC VET RES, V14, DOI 10.1186/s12917-018-1592-z
Tjornehoj K, 2006, J COMP PATHOL, V134, P190, DOI 10.1016/j.jcpa.2005.10.005
Troupin C, 2017, GENOME BIOL EVOL, V9, P3202, DOI 10.1093/gbe/evx236
Turmelle AS, 2010, J GEN VIROL, V91, P2360, DOI 10.1099/vir.0.020073-0
Van Brussel K, 2022, CURR OPIN VIROL, V52, P192, DOI 10.1016/j.coviro.2021.12.008
Vandesompele J, 2002, GENOME BIOL, V3, DOI 10.1186/gb-2002-3-7-research0034
Wang ZW, 2005, J VIROL, V79, P12554, DOI 10.1128/JVI.79.19.12554-12565.2005
Xie JZ, 2018, CELL HOST MICROBE, V23, P297, DOI 10.1016/j.chom.2018.01.006
Yang BH, 2019, CELL REP, V27, P3629, DOI 10.1016/j.celrep.2019.05.061
Zhang GJ, 2013, SCIENCE, V339, P456, DOI 10.1126/science.1230835
Zhou P, 2016, P NATL ACAD SCI USA, V113, P2696, DOI 10.1073/pnas.1518240113
NR 74
TC 3
Z9 3
U1 0
U2 5
PU BMC
PI LONDON
PA CAMPUS, 4 CRINAN ST, LONDON N1 9XW, ENGLAND
EI 1471-2172
J9 BMC IMMUNOL
JI BMC Immunol.
PD APR 21
PY 2023
VL 24
IS 1
AR 7
DI 10.1186/s12865-023-00542-7
PG 13
WC Immunology
WE Science Citation Index Expanded (SCI-EXPANDED)
SC Immunology
GA E6NU9
UT WOS:000976695400001
PM 37085747
OA gold, Green Published
DA 2024-10-21
ER

PT J

AU Costantini, D
Weinberg, M
Jordán, L
Moreno, KR
Yovel, Y
Czirjak, GA
AF Costantini, David
Weinberg, Maya
Jordan, Lilla
Moreno, Kelsey R.
Yovel, Yossi
Czirjak, Gabor A.
TI Induced bacterial sickness causes inflammation but not blood oxidative
stress in Egyptian fruit bats (<i>Rousettus aegyptiacus</i>)
SO CONSERVATION PHYSIOLOGY
LA English
DT Article
DE oxidative stress; innate immunity; inflammation; infection;
extracellular pathogen; ecoimmunology; bats; antioxidant
ID LIFE-HISTORY; IMMUNE; CHIROPTERA; LONGEVITY; DISEASE; BIRDS
AB Relying on a simulated bacterial infection in Egyptian fruit bats, we identify haptoglobin and lysozyme as potential diagnostic markers for extracellular infections in bats and find no relationships between inflammation and oxidative stress.

Bats are particularly interesting vertebrates in their response to pathogens owing to extremes in terms of tolerance and resistance. Oxidation is often a by-product of processes involved in the acute phase response, which may result in antimicrobial or self-damaging effects. We measured the immunological and oxidative status responses of Egyptian fruit bats (Rousettus aegyptiacus) to a simulated bacterial infection using lipopolysaccharide injection. As expected, experimental bats exhibited increases in two humoral immunological markers. However, they surprisingly did not show any effects across two markers of oxidative damage and four antioxidant markers. We propose that this lack of effects on oxidative status may be due to a reduction in cell metabolism through sickness behaviours or given life history traits, such as a long lifespan and a frugivorous diet. Finally, the consistency in the pattern of elevation in haptoglobin and lysozyme between current and previous findings highlights their utility as diagnostic markers for extracellular infections in bats.
C1 [Costantini, David] CNRS, Museum Natl Hist Nat, Unite Physiol Mol & Adaptat PhyMA, UMR 7221, CP32,57 Rue Cuvier, F-75005 Paris, France.

[Weinberg, Maya; Moreno, Kelsey R.; Yovel, Yossi] Tel Aviv Univ, Dept Zool, IL-6997801 Tel Aviv, Israel.

[Jordan, Lilla; Czirjak, Gabor A.] Leibniz Inst Zoo & Wildlife Res, Dept Wildlife Dis, Alfred Kowalke Str 17, D-10315 Berlin, Germany.

[Jordan, Lilla] Eotvos Lorand Univ, Dept Systemat Zool & Ecol, Behav Ecol Grp, Pazmany Peter Setany 1-C, H-1117 Budapest, Hungary.

[Yovel, Yossi] Tel Aviv Univ, Sagol Sch Neurosci, IL-6997801 Tel Aviv, Israel.

C3 Museum National d'Histoire Naturelle (MNHN); Centre National de la
Recherche Scientifique (CNRS); CNRS - National Institute for Biology
(INSB); Tel Aviv University; Leibniz Institut fur Zoo und
Wildtierforschung; Eotvos Lorand University; Tel Aviv University
RP Costantini, D (corresponding author), CNRS, Museum Natl Hist Nat,
Unite Physiol Mol & Adaptat, UMR 7221, CP32,57 Rue Cuvier, F-75005
Paris, France.
EM david.costantini@mnhn.fr
RI Czirják, Gábor/F-5440-2011; Jordán, Lilla/KIB-2545-2024; Costantini,
David/C-6006-2013
OI Czirjak, Gabor Arpad/0000-0001-9488-0069
FU Erasmus+ scholarship from the Eotvos Lorand University, Budapest,
Hungary; Leibniz Institute for Zoo and Wildlife Research, Berlin;
Zuckerman STEM Leadership Program
FX LJ received an Erasmus+ scholarship from the Eotvos Lorand University,
Budapest, Hungary. GAC was supported by funds from the Leibniz Institute
for Zoo and Wildlife Research, Berlin. KRM was supported by the
Zuckerman STEM Leadership Program.
CR Babior BM, 2004, CURR OPIN IMMUNOL, V16, P42, DOI 10.1016/j.coi.2003.12.001
Baker ML, 2013, ZOONOSES PUBLIC HLTH, V60, P104, DOI 10.1111/j.1863-2378.2012.01528.x
Brook CE, 2015, TRENDS MICROBIOL, V23, P172, DOI 10.1016/j.tim.2014.12.004
Brunet-Rossinni AK, 2004, MECH AGEING DEV, V125, P11, DOI 10.1016/j.mad.2003.09.003
Costantini, 2014, OXIDATIVE STRESS HOR, DOI DOI 10.1007/978-3-642-54663-1
Costantini D, 2019, SCI TOTAL ENVIRON, V671, P1077, DOI 10.1016/j.scitotenv.2019.03.398
Costantini D, 2009, COMP BIOCHEM PHYS A, V153, P339, DOI 10.1016/j.cbpa.2009.03.010
Field KA, 2015, PLOS PATHOG, V11, DOI 10.1371/journal.ppat.1005168
Foley NM, 2018, SCI ADV, V4, DOI 10.1126/sciadv.aao0926
Fritze M, 2021, DEV COMP IMMUNOL, V119, DOI 10.1016/j.dci.2021.104017
Fritze M, 2019, BIOL OPEN, V8, DOI 10.1242/bio.046078
He CZ, 2019, J MOL EVOL, V87, P309, DOI 10.1007/s00239-019-09910-7
Huang ZX, 2020, AGING-US, V12, DOI 10.18632/aging.103725
Huber N, 2017, PHYSIOL BEHAV, V182, P46, DOI 10.1016/j.physbeh.2017.09.017
Jebb D, 2018, SCI REP-UK, V8, DOI 10.1038/s41598-018-31093-2
Lee KA, 2006, INTEGR COMP BIOL, V46, P1000, DOI 10.1093/icb/icl049
Lips KR, 2006, P NATL ACAD SCI USA, V103, P3165, DOI 10.1073/pnas.0506889103
Mandl JN, 2018, FRONT IMMUNOL, V9, DOI 10.3389/fimmu.2018.02112

   McCallum H, 2009, ECOLOGY, V90, P3379, DOI 10.1890/08-1763.1
   Moreno KR, 2021, ANN NY ACAD SCI, V1505, P178, DOI 10.1111/
nyas.14600
   Santillán DDM, 2021, MOL ECOL, V30, P6449, DOI 10.1111/mec.16027
   Ohmer MEB, 2021, CONSERV PHYSIOL, V9, DOI 10.1093/conphys/coab074
   Pavlovich SS, 2018, CELL, V173, P1098, DOI 10.1016/
j.cell.2018.03.070
   Pikula J, 2020, BMC VET RES, V16, DOI 10.1186/s12917-020-02450-z
   R Core Team, 2020, R LANG ENV STAT COMP
   Schneeberger K, 2014, NATURWISSENSCHAFTEN, V101, P285, DOI
10.1007/s00114-014-1155-5
   Schneeberger K, 2013, J EXP BIOL, V216, P4514, DOI 10.1242/
jeb.090837
   Voigt CC, 2020, SCI REP-UK, V10, DOI 10.1038/s41598-020-74473-3
   Wibbelt G, 2010, BIOL LETTERS, V6, P438, DOI 10.1098/
rsbl.2010.0267
   Wilkinson GS, 2021, NAT COMMUN, V12, DOI 10.1038/
s41467-021-21900-2
   Wilkinson GS, 2002, AGING CELL, V1, P124, DOI 10.1046/
j.1474-9728.2002.00020.x
   Xie JZ, 2018, CELL HOST MICROBE, V23, P297, DOI 10.1016/
j.chom.2018.01.006
   York HA, 2009, J MAMMAL, V90, P1469, DOI 10.1644/08-MAMM-A-382R.1
NR 33
TC 5
Z9 5
U1 1
U2 4
PU OXFORD UNIV PRESS
PI OXFORD
PA GREAT CLARENDON ST, OXFORD OX2 6DP, ENGLAND
SN 2051-1434
J9 CONSERV PHYSIOL
JI Conserv. Physiol.
PD JAN 1
PY 2022
VL 10
IS 1
AR coac028
DI 10.1093/conphys/coac028
PG 7
WC Biodiversity Conservation; Ecology; Environmental Sciences;
Physiology
WE Science Citation Index Expanded (SCI-EXPANDED)
SC Biodiversity & Conservation; Environmental Sciences & Ecology;
   Physiology
GA 0U0YY
UT WOS:000787386100003
PM 35492418
OA Green Published
DA 2024-10-21
ER

PT J

AU Yong, KSM
   Ng, JHJ
   Her, Z
   Hey, YY
   Tan, SY
   Tan, WWS
   Irac, SE
   Liu, M
   Chan, XY
   Gunawan, M
   Foo, RJH
   Low, DHW
   Mendenhall, IH
   Chionh, YT
   Dutertre, CA
   Chen, QF
   Wang, LF
AF Yong, Kylie Su Mei
   Ng, Justin Han Jia
   Her, Zhisheng
   Hey, Ying Ying
   Tan, Sue Yee
   Tan, Wilson Wei Sheng
   Irac, Sergio Erdal
   Liu, Min
   Chan, Xue Ying
   Gunawan, Merry
   Foo, Randy Jee Hiang
   Low, Dolyce Hong Wen
   Mendenhall, Ian Hewitt
   Chionh, Yok Teng
   Dutertre, Charles-Antoine
   Chen, Qingfeng
   Wang, Lin-Fa
TI Bat-mouse bone marrow chimera: a novel animal model for dissecting the
   uniqueness of the bat immune system
SO SCIENTIFIC REPORTS
LA English
DT Article
ID VERSUS-HOST-DISEASE; STEM-CELL TRANSPLANTATION; CD4(+) T-CELLS;
   HUMANIZED MICE; RESERVOIRS; ESTABLISHMENT; PATHOGENESIS; GENERATION;
   IMMUNOLOGY; EVOLUTION
AB Bats are an important animal model with long lifespans, low incidences of tumorigenesis and an ability to asymptomatically harbour pathogens. Currently, in vivo studies of bats are hampered due to their low reproduction rates. To overcome this, we transplanted bat cells from bone marrow (BM) and spleen into an immunodeficient mouse strain NOD-scid IL-2R(-/-) (NSG), and have successfully established stable, long-term reconstitution of bat immune cells in mice (bat-mice). Immune functionality of our bat-mouse model was demonstrated through generation of antigen-specific antibody response by bat cells following immunization. Post-

engraftment of total bat BM cells and splenocytes, bat immune cells survived, expanded and repopulated the mouse without any observable clinical abnormalities. Utilizing bat's remarkable immunological functions, this novel model has a potential to be transformed into a powerful platform for basic and translational research.
C1 [Yong, Kylie Su Mei; Her, Zhisheng; Tan, Sue Yee; Tan, Wilson Wei Sheng; Liu, Min; Chan, Xue Ying; Gunawan, Merry; Chen, Qingfeng] ASTAR, Inst Mol & Cell Biol, Singapore 138673, Singapore.
[Ng, Justin Han Jia; Hey, Ying Ying; Irac, Sergio Erdal; Foo, Randy Jee Hiang; Low, Dolyce Hong Wen; Mendenhall, Ian Hewitt; Chionh, Yok Teng; Dutertre, Charles-Antoine; Wang, Lin-Fa] Duke NUS Med Sch, Programme Emerging Infect Dis, Singapore 169857, Singapore.
[Dutertre, Charles-Antoine] ASTAR, Singapore Immunol Network SIgN, Singapore 138648, Singapore.
[Chen, Qingfeng] Natl Univ Singapore, Yong Loo Lin Sch Med, Dept Microbiol & Immunol, Singapore 119228, Singapore.
[Chen, Qingfeng] Guangzhou Med Univ, Affiliated Hosp 3, Key Lab Major Obstet Dis Guangdong Prov, Guangzhou 510150, Guangdong, Peoples R China.
C3 Agency for Science Technology & Research (A*STAR); A*STAR - Institute of
Molecular & Cell Biology (IMCB); National University of Singapore;
Agency for Science Technology & Research (A*STAR); A*STAR - Singapore
Immunology Network (SIgN); National University of Singapore; Guangzhou
Medical University
RP Chen, QF (corresponding author), ASTAR, Inst Mol & Cell Biol, Singapore 138673, Singapore.; Wang, LF (corresponding author), Duke NUS Med Sch, Programme Emerging Infect Dis, Singapore 169857, Singapore.; Chen, QF (corresponding author), Natl Univ Singapore, Yong Loo Lin Sch Med, Dept Microbiol & Immunol, Singapore 119228, Singapore.; Chen, QF (corresponding author), Guangzhou Med Univ, Affiliated Hosp 3, Key Lab Major Obstet Dis Guangdong Prov, Guangzhou 510150, Guangdong, Peoples R China.
EM qchen@imcb.a-star.edu.sg; linfa.wang@duke-nus.edu.sg
RI Dutertre, Charles Antoine/JRX-2224-2023; Irac, Sergio Erdal/G-6690-2014
OI Mendenhall, Ian/0000-0003-4250-6459; Dutertre,
Charles-Antoine/0000-0001-7950-3652; Irac, Sergio
Erdal/0000-0001-5622-1409; Tan, Wilson Wei Sheng/0000-0003-3027-1162;
Ng, Justin Han Jia/0000-0002-1076-6013; Yong, Kylie/0000-0002-2424-2835;
FOO, RANDY/0000-0001-9661-8662
FU National Research Foundation [NRF2012NRF-CRP001-056,
NMRC/BNIG/2026/2014, NMRC/BNIG/2005/2013]; National Medical Research
Council in Singapore; Joint Council Office Development Programme, Agency
for Science, Technology and Research (A*STAR), Singapore [1334k00082];
Singapore National Research Foundation [NRF-NRFF2017-03]

FX We thank Aaron Irving for helpful technical discussion. This study was
supported by grants NRF2012NRF-CRP001-056 (L.-F.W.), NMRC/BNIG/2026/2014
(C-A.D)and NMRC/BNIG/2005/2013 (I.H.M) from the National Research
Foundation and National Medical Research Council in Singapore, and by
the Joint Council Office Development Programme 1334k00082, Agency for
Science, Technology and Research (A*STAR), Singapore (Q.C.). Q.C. is
also supported by Singapore National Research Foundation Fellowship
NRF-NRFF2017-03.
CR Ahn M, 2016, SCI REP-UK, V6, DOI 10.1038/srep21722
Allocati N, 2016, CELL DEATH DISCOV, V2, DOI 10.1038/cddiscovery.2016.48
Anderson BE, 2003, J CLIN INVEST, V112, P101, DOI 10.1172/JCI200317601
[Anonymous], 1986, AVIAN PHYSIOL
Ball LM, 2008, BONE MARROW TRANSPL, V41, pS58, DOI 10.1038/bmt.2008.56
Banerjee A, 2016, J VIROL METHODS, V237, P166, DOI 10.1016/j.jviromet.2016.09.008
BOERMANS HJ, 1992, VET IMMUNOL IMMUNOP, V34, P273, DOI 10.1016/0165-2427(92)90170-U
Bouchlaka MN, 2010, IMMUNOTHERAPY-UK, V2, P399, DOI 10.2217/IMT.10.20
Brehm MA, 2010, CURR OPIN ENDOCRINOL, V17, P120, DOI 10.1097/MED.0b013e328337282f
Buckley RH, 2011, IMMUNOL RES, V49, P25, DOI 10.1007/s12026-010-8191-9
Calisher CH, 2006, CLIN MICROBIOL REV, V19, P531, DOI 10.1128/CMR.00017-06
Chen Q, 2013, BIO-PROTOCOL, V3, pe992, DOI DOI 10.21769/BioProtoc.992
Chen QF, 2009, P NATL ACAD SCI USA, V106, P21783, DOI 10.1073/pnas.0912274106
Chu YW, 2008, BIOL BLOOD MARROW TR, V14, P365, DOI 10.1016/j.bbmt.2007.12.002
Crameri G, 2009, PLOS ONE, V4, DOI 10.1371/journal.pone.0008266
Czechowicz A, 2010, IMMUNOL ALLERGY CLIN, V30, P159, DOI 10.1016/j.iac.2010.03.003
Drake AC, 2012, CELL MOL IMMUNOL, V9, P215, DOI 10.1038/cmi.2012.6
Fox J. G., 2007, The mouse in biomedical research, V2nd
GALE RP, 1986, LANCET, V1, P1468
Gannon WL, 2007, J MAMMAL, V88, P809, DOI 10.1644/06-MAMM-F-185R1.1
Ge XY, 2013, NATURE, V503, P535, DOI 10.1038/nature12711
Gómez JMM, 2016, SCI REP-UK, V6, DOI 10.1038/srep37796
HAMILTON IM, 1994, CAN J ZOOL, V72, P744, DOI 10.1139/z94-100
Hess AD, 2006, BIOL BLOOD MARROW TR, V12, P13, DOI 10.1016/j.bbmt.2005.11.002

Ito M, 2002, BLOOD, V100, P3175, DOI 10.1182/blood-2001-12-0207
Ito R, 2012, CELL MOL IMMUNOL, V9, P208, DOI 10.1038/cmi.2012.2
Justice MJ, 2016, DIS MODEL MECH, V9, P101, DOI 10.1242/dmm.024547
Keng CT, 2016, GUT, V65, P1744, DOI 10.1136/gutjnl-2014-307856
Kim EJ, 2011, IMMUNOL CELL BIOL, V89, P897, DOI 10.1038/icb.2011.8
KORNGOLD R, 1978, J EXP MED, V148, P1687
Leroy EM, 2005, NATURE, V438, P575, DOI 10.1038/438575a
Li WD, 2005, SCIENCE, V310, P676, DOI 10.1126/science.1118391
Luis AD, 2013, P ROY SOC B-BIOL SCI, V280, DOI 10.1098/rspb.2012.2753
Marsh GA, 2012, PLOS PATHOG, V8, DOI 10.1371/journal.ppat.1002836
Mattsson J, 2008, BIOL BLOOD MARROW TR, V14, P165, DOI 10.1016/j.bbmt.2007.10.025
MOSIER DE, 1988, NATURE, V335, P256, DOI 10.1038/335256a0
Mourya DT, 2013, INDIAN J MED RES, V138, P224
Ng JHJ, 2016, SCI REP-UK, V6, DOI 10.1038/srep21256
Niu HT, 2008, EUR J IMMUNOL, V38, P3028, DOI 10.1002/eji.200838417
Olival KJ, 2017, NATURE, V546, P646, DOI 10.1038/nature22975
PLOEMACHER RE, 1989, BLOOD, V74, P2755
Reddy P, 2008, BIOL BLOOD MARROW TR, V14, P129, DOI 10.1016/j.bbmt.2007.10.021
REVILLA Y, 1994, J GEN VIROL, V75, P1983, DOI 10.1099/0022-1317-75-8-1983
Rosenthal N, 2007, NAT CELL BIOL, V9, P993, DOI 10.1038/ncb437
Schountz T, 2014, VIRUSES-BASEL, V6, P4880, DOI 10.3390/v6124880
Schroeder MA, 2011, DIS MODEL MECH, V4, P318, DOI 10.1242/dmm.006668
Shultz LD, 2007, NAT REV IMMUNOL, V7, P118, DOI 10.1038/nri2017
Shultz LD, 2012, NAT REV IMMUNOL, V12, P786, DOI 10.1038/nri3311
Socié G, 2009, BLOOD, V114, P4327, DOI 10.1182/blood-2009-06-204669
TASWELL C, 1981, J IMMUNOL, V126, P1614
Towner JS, 2009, PLOS PATHOG, V5, DOI 10.1371/journal.ppat.1000536
TRUITT RL, 1991, BONE MARROW TRANSPL, V8, P51
Wang L. M, 2013, FIELDS VIROLOGY, P1070
Wang LF, 2011, CURR OPIN VIROL, V1, P649, DOI 10.1016/j.coviro.2011.10.013
Wilkinson GS, 2002, AGING CELL, V1, P124, DOI 10.1046/j.1474-9728.2002.00020.x
Wynne JW, 2013, PLOS PATHOG, V9, DOI 10.1371/journal.ppat.1003651
Yong K. S. M., 2018, ARCH IMMUNOL THER EX, P1
Yong KSM, 2016, CELL MOL IMMUNOL, V13, P605, DOI 10.1038/cmi.2015.40
Zeiser R, 2016, BLOOD, V127, P3117, DOI 10.1182/blood-2016-02-699082
Zhang GJ, 2013, SCIENCE, V339, P456, DOI 10.1126/science.1230835
Zhou P, 2016, SCI REP-UK, V6, DOI 10.1038/srep38597
NR 61
TC 12
Z9 12

U1 0
U2 16
PU NATURE PORTFOLIO
PI BERLIN
PA HEIDELBERGER PLATZ 3, BERLIN, 14197, GERMANY
SN 2045-2322
J9 SCI REP-UK
JI Sci Rep
PD MAR 16
PY 2018
VL 8
AR 4726
DI 10.1038/s41598-018-22899-1
PG 10
WC Multidisciplinary Sciences
WE Science Citation Index Expanded (SCI-EXPANDED)
SC Science & Technology - Other Topics
GA FZ4VC
UT WOS:000427589100003
PM 29549333
OA Green Published, gold
DA 2024-10-21
ER

PT J
AU Rashid, N
   Irfan, M
   Nadeem, MS
   Shabbir, A
AF Rashid, Nosheen
   Irfan, Muhammad
   Nadeem, Muhammad Sajid
   Shabbir, Asghar
TI Comparative Seasonal Haematology of Two Bat Species,
<i>Scotophilus
   heathii</i> and <i>Pipistrellus pipistrellus</i>, in a
Subtropical Area
   of Pakistan
SO PAKISTAN JOURNAL OF ZOOLOGY
LA English
DT Article
DE Seasonal variations; Haematological parameters; Vespertilionid
bat; S.
   heathii
ID TRADE-OFFS; ECOLOGICAL IMMUNOLOGY; BLOOD; UNDERNUTRITION;
HEMOGLOBIN;
   COMPONENTS; NITROGEN; GLUCOSE; SERUM; DEER
AB Various haematological parameters of two bat species belonging to
the family Vespertilionidae (Scotophilus healthii and Pipistrellus
pipistrellus) were examined on the seasonal basis. Blood samples
were taken from 47 bats (23 S. heathii and 24 P. pipistrellus)
captured from Potohar region. Results showed that P. pipistrellus
exhibited significantly (P<0.05) higher mean counts of red blood
cells (RBCs), white blood cells (WBCs), platelets, haemoglobin (Hb),

and packed cell volume (PCV) as compared to S. heathii. However,
mean corpuscular volume (MCV), mean corpuscular haemoglobin (MCH)
and mean corpuscular haemoglobin concentration (MCHC) levels were
significantly ($P<0.05$) higher in S. heathii as compared to P.
pipistrellus. Seasonal comparisons in both the species depicted
lowest levels of RBCs, WBCs, MCV, MCH and PCV in spring when bats
are least active just after hibernation. Highest mean estimates of
RBCs, Hb, and PCV were observed in summer (bats are most active) in
male and female bats belonging to both species. However, WBCs levels
were at peak during autumn when bats are preparing to go into
hibernation.
C1 [Rashid, Nosheen; Irfan, Muhammad; Nadeem, Muhammad Sajid] Pir
Mehr Ali Shah Arid Agr Univ, Dept Zool, Rawalpindi, Pakistan.
[Shabbir, Asghar] COMSAT Inst Informat Technol, Biosci,
Islamabad, Pakistan.
C3 Arid Agriculture University; COMSATS University Islamabad (CUI)
RP Irfan, M (corresponding author), Pir Mehr Ali Shah Arid Agr Univ,
Dept Zool, Rawalpindi, Pakistan.
EM muhammadirfan11@gmail.com
RI Nadeem, Muhammad/HKV-5114-2023; Shabbir, Asghar/AAA-3171-2022;
Irfan,
Muhammad/F-1517-2014
FU Pir Mehr Ali Shah, Arid Agriculture University Rawalpindi [PMAS-
AAUR/DR
454]
FX This work was supported by Pir Mehr Ali Shah, Arid Agriculture
University Rawalpindi under Grant No. PMAS-AAUR/DR 454.
CR ALFARO V, 1994, COMP BIOCHEM PHYS A, V107, P149, DOI
10.1016/0300-9629(94)90287-9
AREVALO F, 1992, ARCH INT PHYSIOL BIO, V100, P385, DOI
10.3109/13813459209000730
AREVALO F, 1987, COMP BIOCHEM PHYS A, V88, P447, DOI
10.1016/0300-9629(87)90061-2
BATES P. J. J., 1997, BATS INDIAN SUBCONTI
Biino G, 2011, HAEMATOL-HEMATOL J, V96, P96, DOI 10.3324/
haematol.2010.029934
Buckley MF, 2000, THROMB HAEMOSTASIS, V83, P480
Campbell T.W., 1992, AVIAN HEMATOLOGY CYT
Dawson RD, 1997, COMP BIOCHEM PHYS A, V117, P383, DOI 10.1016/
S0300-9629(96)00364-7
DELGIUDICE GD, 1994, J WILDLIFE MANAGE, V58, P430, DOI
10.2307/3809313
Eates K.R., 1968, INTRO VERTEBRATE FAU, P33
Folstad I, 1997, BEHAV ECOL, V8, P109, DOI 10.1093/beheco/8.1.109
Gulland F. M. D., 1990, Veterinary Annual, V30, P126
HARDIG J, 1983, COMP BIOCHEM PHYS A, V75, P35, DOI
10.1016/0300-9629(83)90040-3
Harshman LG, 2007, TRENDS ECOL EVOL, V22, P80, DOI 10.1016/
j.tree.2006.10.008
Heard DJ, 1997, J ZOO WILDLIFE MED, V28, P464
HELLGREN EC, 1989, CAN J ZOOL, V67, P1837, DOI 10.1139/z89-262
JURGENS KD, 1981, RESP PHYSIOL, V45, P243, DOI
10.1016/0034-5687(81)90009-8
Kim J.-C., 2002, Comp. Clin. Pathol., V11, P98, DOI [10.1007/

s005800200006, DOI 10.1007/S005800200006]
KOOPMAN HN, 1995, MAR MAMMAL SCI, V11, P123, DOI 10.1111/j.1748-7692.1995.tb00512.x
Korine C, 1999, J COMP PHYSIOL B, V169, P280, DOI 10.1007/s003600050222
KORINE C, 1993, J THERM BIOL, V18, P61, DOI 10.1016/0306-4565(93)90018-O
Kortet R, 2004, BIOL J LINN SOC, V81, P111, DOI 10.1111/j.1095-8312.2004.00275.x
LEWIS JH, 1977, COMP BIOCHEM PHYS A, V58, P103, DOI 10.1016/0300-9629(77)90023-8
Lochmiller RL, 2000, OIKOS, V88, P87, DOI 10.1034/j.1600-0706.2000.880110.x
LURIE S, 1993, GYNECOL OBSTET INVES, V36, P141, DOI 10.1159/000292613
Mahmood-ul-Hassan M., 2006, JAPS, Journal of Animal and Plant Sciences, V16, P38
MINEMATSU S, 1995, EXP ANIM TOKYO, V44, P223, DOI 10.1538/expanim.44.223
MIRZA Z B, 1967, Pakistan Journal of Science, V19, P193
Mstat C., 1991, MANUAL MICROSTATISTI
Murray J.A., 1884, VERTEBRATE ZOOLOGY S, P476
Nadeem MS, 2013, PAK J ZOOL, V45, P565
Nieminen N., 1979, COMP BIOCH PHYSL A, V66, P399
Norris K, 2000, BEHAV ECOL, V11, P19, DOI 10.1093/beheco/11.1.19
Pehlivanoglu B, 2001, Arch Physiol Biochem, V109, P383, DOI 10.1076/apab.109.3.383.9338
REWKIEWICZDZIARSKA A, 1975, B ACAD POL SCI BIOL, V23, P481
Roberts TJ, 1997, MAMMALS OF PAKISTAN
Roff Derek A., 1992
Schmid-Hempel P, 2003, TRENDS ECOL EVOL, V18, P27, DOI 10.1016/S0169-5347(02)00013-7
Sheldon BC, 1996, TRENDS ECOL EVOL, V11, P317, DOI 10.1016/0169-5347(96)10039-2
Skau PA, 2005, BEHAV ECOL, V16, P410, DOI 10.1093/beheco/ari004
Stearns S.C., 1992, pi
VANDERWESTHUYZEN J, 1978, S AFR J SCI, V74, P99
Viljoen M, 1997, S AFR J SCI, V93, P414
Walker S., 2003, SUMMARY STATUS S ASI, P23
WIDMAIER EP, 1993, J EXP ZOOL, V265, P533, DOI 10.1002/jez.1402650509
WIGHTMAN J, 1987, COMP BIOCHEM PHYS B, V88, P305, DOI 10.1016/0305-0491(87)90119-2
Wintrobe MM., 1933, FOLIA HAEMATOL, V51, P32, DOI DOI 10.1007/S00580-011-1316-2
WOLKERS H, 1994, CAN J ZOOL, V72, P1291, DOI 10.1139/z94-172
NR 48
TC 4
Z9 8
U1 0
U2 4
PU ZOOLOGICAL SOC PAKISTAN
PI LAHORE
PA UNIV PUNJAB, NEW CAMPUS, C/O DEPT ZOOLOGY, LAHORE, PAKISTAN

SN 0030-9923
J9 PAK J ZOOL
JI Pak. J. Zool.
PD OCT
PY 2016
VL 48
IS 5
BP 1503
EP 1510
PG 8
WC Zoology
WE Science Citation Index Expanded (SCI-EXPANDED)
SC Zoology
GA DY0KU
UT WOS:000384786500037
DA 2024-10-21
ER

PT J
AU Chen, WL
   Mao, XG
AF Chen, Wenli
   Mao, Xiuguang
TI Impacts of seasonality on gene expression in the Chinese horseshoe bat
SO ECOLOGY AND EVOLUTION
LA English
DT Article
DE bats; circannual; immunity; seasonal changes; transcriptome
ID MEIOTIC ARREST; NEURONS; SEX; HYPOTHALAMUS; PLASTICITY
AB Seasonality can cause changes in many environmental factors which potentially affects gene expression. Here, we used a bat species (Rhinolophus sinicus) from eastern China as a model to explore the molecular mechanisms of seasonal effects, in particular during phenological shifts in the spring and autumn. Based on the analysis of 45 RNA-seq samples, we found strong seasonal effects on gene expression, with a large number of genes identified as either specific or biased to each season. Weighted gene co-expression network analysis also identified multiple modules significantly associated with each season. These seasonal genes were further enriched into different functional categories. Consistent with effects of phenological shifts on bats, we found that genes related to promoting food intake were highly expressed in both autumn and spring. In addition, immunity genes were also highly expressed in both seasons although this seasonal immune response had tissue specificity in different seasons. In female bats, genes related to the delay of ovulation (e.g., NPPC, natriuretic peptide precursor type C) were highly expressed in October, while genes associated with the promotion of reproduction (e.g., DIO2, iodothyronine deiodinase 2) were biasedly expressed in April. Lastly, we found multiple known core clock genes in both October-biased and April-biased expressed genes, which may be involved in regulating the start and end of hibernation, respectively. Overall, together with studies in other land and aquatic animals, our work supports that

seasonal gene expression variations may be a general evolutionary response to environmental changes in wild animals.
C1 [Chen, Wenli; Mao, Xiuguang] East China Normal Univ, Sch Ecol & Environm Sci, Shanghai 200062, Peoples R China.
C3 East China Normal University
RP Mao, XG (corresponding author), East China Normal Univ, Sch Ecol & Environm Sci, Shanghai 200062, Peoples R China.
EM xgmao@sklec.ecnu.edu.cn
OI Chen, Wenli/0000-0003-3129-3662; mao, xiuguang/
0000-0002-3432-7509
FU Scientific and Technological Innovation Plan of Shanghai Science and
Technology Committee [20ZR1417000]
FX The authors thank Sun Haijian, Wang JY, Ding YT, and Li Qianqian for
assistance with field data collection. This work was supported by The
Scientific and Technological Innovation Plan of Shanghai Science and
Technology Committee (20ZR1417000). The authors are grateful to two
anonymous reviewers whose comments significantly improve this
manuscript.
CR Abhilasha DC, 2003, PHYSIOL BIOCHEM ZOOL, V76, P271, DOI 10.1086/367941
BENJAMINI Y, 1995, J R STAT SOC B, V57, P289, DOI 10.1111/j.2517-6161.1995.tb02031.x
Bolger AM, 2014, BIOINFORMATICS, V30, P2114, DOI 10.1093/bioinformatics/btu170
Bresnahan ST, 2022, MOL ECOL, V31, P174, DOI 10.1111/mec.16220
Calvez J, 2017, BRIT J PHARMACOL, V174, P1049, DOI 10.1111/bph.13530
Cheviron ZA, 2017, INTEGR COMP BIOL, V57, P1040, DOI 10.1093/icb/icx118
Daniels EV, 2014, MOL ECOL, V23, P6123, DOI 10.1111/mec.12988
De Jong S, 2014, HUM MOL GENET, V23, P2721, DOI 10.1093/hmg/ddt665
Dopico XC, 2015, NAT COMMUN, V6, DOI 10.1038/ncomms8000
Duong HA, 2011, SCIENCE, V332, P1436, DOI 10.1126/science.1196766
Gillen AE, 2021, FRONT PHYSIOL, V12, DOI 10.3389/fphys.2021.662132
Glass D, 2013, GENOME BIOL, V14, DOI 10.1186/gb-2013-14-7-r75
Goldinger A, 2015, PLOS ONE, V10, DOI 10.1371/journal.pone.0126995
Grilliot ME, 2014, ACTA CHIROPTEROL, V16, P379, DOI 10.3161/150811014X687332
Harrison PW, 2012, SEMIN CELL DEV BIOL, V23, P222, DOI 10.1016/j.semcdb.2011.12.004
Heideman Paul D, 2003, BMC Ecol, V3, P11, DOI 10.1186/1472-6785-3-11
Helm B, 2013, P ROY SOC B-BIOL SCI, V280, DOI 10.1098/rspb.2013.0016
HOCHBERG Y, 1990, STAT MED, V9, P811, DOI 10.1002/sim.4780090710
Hoering F, 2021, MAR GENOM, V56, DOI 10.1016/j.margen.2020.100806

Johnston RA, 2016, MOL ECOL, V25, P5680, DOI 10.1111/mec.13879
Kim D, 2015, NAT METHODS, V12, P357, DOI [10.1038/NMETH.3317, 10.1038/nmeth.3317]
Kiyosu C, 2012, REPRODUCTION, V144, P187, DOI 10.1530/REP-12-0050
Koch M, 2015, NATURE, V519, P45, DOI 10.1038/nature14260
Kolde R., 2019, PHEATMAP PRETTY HEAT
Langfelder P, 2008, BMC BIOINFORMATICS, V9, DOI 10.1186/1471-2105-9-559
Lei M, 2014, PLOS ONE, V9, DOI 10.1371/journal.pone.0107746
Levy O, 2016, ECOL LETT, V19, P620, DOI 10.1111/ele.12595
Li H, 2009, BIOINFORMATICS, V25, P2078, DOI 10.1093/bioinformatics/btp352
Liao Y, 2014, BIOINFORMATICS, V30, P923, DOI 10.1093/bioinformatics/btt656
Lisovski S, 2017, INTEGR COMP BIOL, V57, P934, DOI 10.1093/icb/icx040
Love MI, 2014, GENOME BIOL, V15, DOI 10.1186/s13059-014-0550-8
Luquet S, 2005, SCIENCE, V310, P683, DOI 10.1126/science.1115524
Macias-Muñoz A, 2016, MOL BIOL EVOL, V33, P79, DOI 10.1093/molbev/msv197
Miller KE, 2016, HEARING RES, V341, P91, DOI 10.1016/j.heares.2016.07.014
Mondal MS, 2003, ENDOCRINOLOGY, V144, P4729, DOI 10.1210/en.2003-0536
Mure LS, 2018, SCIENCE, V359, P1232, DOI 10.1126/science.aao0318
Nowack J, 2017, J COMP PHYSIOL B, V187, P889, DOI 10.1007/s00360-017-1100-y
Oostra V, 2018, NAT COMMUN, V9, DOI 10.1038/s41467-018-03384-9
OXBERRY BA, 1979, J REPROD FERTIL, V56, P359
Pei HJ, 2014, MOL METAB, V3, P209, DOI 10.1016/j.molmet.2013.12.006
Sailani MR, 2020, NAT COMMUN, V11, DOI 10.1038/s41467-020-18758-1
Schwartz C, 2013, CURR TOP DEV BIOL, V105, P247, DOI 10.1016/B978-0-12-396968-2.00009-9
Schwartz C, 2013, PLOS ONE, V8, DOI 10.1371/journal.pone.0058427
Seale LA, 2020, AM J CLIN NUTR, V112, P447, DOI 10.1093/ajcn/nqaa177
Sendor T, 2003, J ANIM ECOL, V72, P308, DOI 10.1046/j.1365-2656.2003.00702.x
Sharma A, 2020, MOL CELL ENDOCRINOL, V508, DOI 10.1016/j.mce.2020.110794
Simmons N.B., 2020, Bat species of the world: A taxonomic and geographic database
Somel M, 2006, CURR BIOL, V16, pR359, DOI 10.1016/j.cub.2006.04.024
Sun HJ, 2020, FRONT ZOOL, V17, DOI 10.1186/s12983-020-00366-w
Suzuki R, 2006, BIOINFORMATICS, V22, P1540, DOI 10.1093/bioinformatics/btl117
Van Dolah FM, 2015, PLOS ONE, V10, DOI 10.1371/journal.pone.0130934
Wang Z, 2008, ZOOL STUD, V47, P215
Webb PI, 1996, CAN J ZOOL, V74, P761, DOI 10.1139/z96-087
Wilkinson GS, 2021, NAT COMMUN, V12, DOI 10.1038/s41467-021-21900-2

   Williams CM, 2017, INTEGR COMP BIOL, V57, P921, DOI 10.1093/icb/icx122
   Wucher V, 2022, bioRxiv, DOI [10.1101/2021.02.28.433266, DOI 10.1101/2021.02.28.433266]
   Xiao YH, 2015, PLOS ONE, V10, DOI 10.1371/journal.pone.0145702
   Zhang MJ, 2010, SCIENCE, V330, P366, DOI 10.1126/science.1193573
   Zhao XQ, 2016, MOL BIOL EVOL, V33, P707, DOI 10.1093/molbev/msv263
   Zhou YY, 2019, NAT COMMUN, V10, DOI 10.1038/s41467-019-09234-6
NR 60
TC 4
Z9 4
U1 1
U2 11
PU WILEY
PI HOBOKEN
PA 111 RIVER ST, HOBOKEN 07030-5774, NJ USA
SN 2045-7758
J9 ECOL EVOL
JI Ecol. Evol.
PD MAY
PY 2022
VL 12
IS 5
AR e8923
DI 10.1002/ece3.8923
PG 12
WC Ecology; Evolutionary Biology
WE Science Citation Index Expanded (SCI-EXPANDED)
SC Environmental Sciences & Ecology; Evolutionary Biology
GA 1F8FA
UT WOS:000795396700001
PM 35592062
OA Green Submitted, Green Published
DA 2024-10-21
ER

PT J
AU de Oliveira, MB
   Bonvicino, CR
AF de Oliveira, Marcione B.
   Bonvicino, Cibele R.
TI Incidence of viruses in Neotropical bats
SO ACTA CHIROPTEROLOGICA
LA English
DT Article
DE Chiroptera; DNA and RNA viruses; host; Neotropical region; zoonosis
ID NON-HEMATOPHAGOUS BATS; CROSS-SPECIES TRANSMISSION; SAO-PAULO STATE;
   RIO-DE-JANEIRO; RABIES VIRUS; GENETIC-CHARACTERIZATION; MOLECULAR
   EPIDEMIOLOGY; INSECTIVOROUS BATS; DESMODUS-ROTUNDUS; VAMPIRE BATS
AB The diversity of bat species and some of their distinct biological and ecological characteristics contribute to their wide

distribution, being generally abundant and common in a variety of habitats, factors that can influence their association with different emerging and reemerging viruses. Studies have shown that bats have characteristics which contribute to the evolution of a unique set of antiviral immune responses that control the replication of viruses, such as their gregarious social organization and ability to fly. In this paper we review the incidence of viruses in Neotropical bats, and the role of species as hosts or vectors of these diseases. A total of 110 species belonging to seven families were found to have been detected carrying virus in the region. Although Chiroptera is the second most diverse order of mammals. the diversity of bat-hosted viruses has only recently come into focus, as the result of improved wildlife surveillance. Diverse viruses have been detected in bats, however, in the majority of the cases, there is no evidence that the virus is transmitted from bats to other animals or causes disease in humans.
C1 [de Oliveira, Marcione B.; Bonvicino, Cibele R.] Univ Fed Rio de Janeiro, Grad Program Zool, Natl Museum, Quinta Boa Vista S-N, BR-20940040 Rio De Janeiro, RJ, Brazil.
[Bonvicino, Cibele R.] Inst Nacl Canc, Genet Div, Rua Andre Cavalcanti 37,4 Andar, BR-20231050 Rio De Janeiro, RJ, Brazil.
[Bonvicino, Cibele R.] Fiocruz MS, Lab Biol & Parasitol Mamiferos, Inst Oswaldo Cruz, BR-21040360 Rio De Janeiro, RJ, Brazil.
C3 Universidade Federal do Rio de Janeiro; National Cancer Institute (Inca); Fundacao Oswaldo Cruz
RP de Oliveira, MB (corresponding author), Univ Fed Rio de Janeiro, Grad Program Zool, Natl Museum, Quinta Boa Vista S-N, BR-20940040 Rio De Janeiro, RJ, Brazil.
EM oliveira01marcione@gmail.com
RI de Oliveira, Marcione/HJH-0994-2022
FU CNPq fellowship; FAPERJ
FX MBO received a CNPq fellowship and CRB received a CNPq fellowship and
financial support from FAPERJ. We thank Maria Elaine A. de Oliveira and
Camila L. Nacif for their advice and contributions to the manuscript.
CR ACHA PN, 1968, B OFIC SANIT PANAM, V64, P411
Aguilar-Setién A, 2008, EPIDEMIOL INFECT, V136, P1678, DOI 10.1017/S0950268808000460
Albas A, 2011, REV SOC BRAS MED TRO, V44, P678, DOI 10.1590/S0037-86822011000600006
Albas A, 2011, REV SOC BRAS MED TRO, V44, P201, DOI 10.1590/S0037-86822011005000001
Albas A, 2009, REV SOC BRAS MED TRO, V42, P15, DOI 10.1590/S0037-86822009000100004
Albuquerque Paloma, 2012, Revista Biociencias (Taubate), V18, P5
Allendorf SD, 2012, VIRUS RES, V165, P119, DOI 10.1016/j.virusres.2012.01.011
Campos ACA, 2019, EMERG INFECT DIS, V25, P333, DOI 10.3201/eid2502.181246
Almeida MF, 2005, EPIDEMIOL INFECT, V133, P523, DOI 10.1017/S0950268804003656

AMORIM A. F., 1970, PESQUI AGROPECU BRAS, V5, P433
[Anonymous], 2003, SEMINA
Anthony SJ, 2013, J GEN VIROL, V94, P1028, DOI 10.1099/vir.0.049759-0
Anthony SJ, 2017, VIRUS EVOL, V3, DOI 10.1093/ve/vex012
Asano KM, 2016, ARCH VIROL, V161, P3225, DOI 10.1007/s00705-016-3010-9
Avila-Flores Rafael, 2002, Mammalian Species, V697, P1, DOI 10.1644/1545-1410(2002)697<0001:NL>2.0.CO;2
Badrane H, 2001, J VIROL, V75, P8096, DOI 10.1128/JVI.75.17.8096-8104.2001
Baillie GJ, 2004, J VIROL, V78, P5784, DOI 10.1128/JVI.78.11.5784-5798.2004
Baker ML, 2013, ZOONOSES PUBLIC HLTH, V60, P104, DOI 10.1111/j.1863-2378.2012.01528.x
Banerjee A, 2020, FRONT MICROBIOL, V11, DOI 10.3389/fmicb.2020.00733
Barbosa TFS, 2007, PUBLIC HLTH NOTEB, V15, P329
de Almeida MAB, 2012, AM J PRIMATOL, V74, P68, DOI 10.1002/ajp.21010
BASTIDA-GONZALEZ F. G., 2011, DATABASE BAT ASS VIR
Bennett M, 2006, EPIDEMIOL INFECT, V134, P905, DOI 10.1017/S0950268806006674
Góes LGB, 2016, INFECT GENET EVOL, V44, P510, DOI 10.1016/j.meegid.2016.07.034
Góes LGB, 2013, EMERG INFECT DIS, V19, P1711, DOI 10.3201/eid1910.130525
Bernardi F, 2005, J GEN VIROL, V86, P3153, DOI 10.1099/vir.0.81223-0
BIANCONI G. V., 2005, 2 C BRAS MAST AR AN, P68
Bittar C, 2020, MICROB ECOL, V79, P203, DOI 10.1007/s00248-019-01391-x
Bolatti EM, 2020, VIRUSES-BASEL, V12, DOI 10.3390/v12040422
BRANDAO P. E., 2018, DATABASE BAT ASS VIR
Brandao PE, 2008, BRAZ J INFECT DIS, V12, P466, DOI 10.1590/S1413-86702008000600003
Brook CE, 2015, TRENDS MICROBIOL, V23, P172, DOI 10.1016/j.tim.2014.12.004
Cabral CC, 2012, REV SOC BRAS MED TRO, V45, P180, DOI 10.1590/S0037-86822012000200008
Calderón A, 2019, VECTOR-BORNE ZOONOT, V19, P747, DOI 10.1089/vbz.2018.2324
Calderón A, 2019, ANN CLIN MICROB ANTI, V18, DOI 10.1186/s12941-019-0308-y
Calisher CH, 2006, CLIN MICROBIOL REV, V19, P531, DOI 10.1128/CMR.00017-06
Canuti M, 2011, PLOS ONE, V6, DOI 10.1371/journal.pone.0029140
Cárdenas-Canales EM, 2020, TROP MED INFECT DIS, V5, DOI 10.3390/tropicalmed5010034
Carneiro AJB, 2010, EMERG INFECT DIS, V16, P2004, DOI 10.3201/eid1612.100726
Carnieli P, 2012, VIRUS GENES, V45, P76, DOI 10.1007/s11262-012-0743-8
Carnieli P, 2010, ARCH VIROL, V155, P941, DOI 10.1007/

s00705-010-0671-7
Carrington CVF, 2008, EMERG INFECT DIS, V14, P1890, DOI 10.3201/eid1412.080642
CARVALHO CD, 2011, VET ZOOTECNIA, V18, P490
CARVALHO-NETO F. G., 2017, DATABASE BAT ASS VIR
Castilho JG, 2010, J WILDLIFE DIS, V46, P1335, DOI 10.7589/0090-3558-46.4.1335
Castilho JG, 2008, REV INST MED TROP SP, V50, P95, DOI 10.1590/S0036-46652008000200006
Chen LH, 2014, DATABASE-OXFORD, DOI 10.1093/database/bau021
Cibulski SP, 2014, GENOME ANNOUNCEMENTS, V2, DOI 10.1128/genomeA.01028-14
Cirranello A, 2016, ACTA CHIROPTEROL, V18, P39, DOI 10.3161/15081109ACC2016.18.1.002
Cisterna D, 2005, VIRUS RES, V109, P139, DOI 10.1016/j.virusres.2004.10.013
Clayton BA, 2017, CURR OPIN VIROL, V22, P97, DOI 10.1016/j.coviro.2016.12.003
Constantine D. G., 2009, Bat rabies and other lyssavirus infections
CORMAN V. M., 2014, DATABASE BAT ASS VIR
Corman VM, 2013, J GEN VIROL, V94, P1984, DOI 10.1099/vir.0.054841-0
Correa M.M.O., 2013, Boletim da Sociedade Brasileira de Mastozoologia, V67, P23
CORREAGIRON P, 1972, SCIENCE, V175, P546, DOI 10.1126/science.175.4021.546
Costa LJC, 2013, T ROY SOC TROP MED H, V107, P684, DOI 10.1093/trstmh/trt080
Crawford DorothyH., 2018, VIRUSES VERY SHORT I
CUEVAS-ROMERO S., 2013, DATABASE BAT ASS VIR
Cunha EMS, 2005, REV SAUDE PUBL, V39, P683, DOI 10.1590/S0034-89102005000400025
da Rosa AR, 2011, REV SOC BRAS MED TRO, V44, P146, DOI 10.1590/S0037-86822011005000018
da Silva LHQ, 1999, REV SAUDE PUBL, V33, P626, DOI 10.1590/S0034-89101999000600016
da Silva MV, 2007, REV SOC BRAS MED TRO, V40, P479, DOI 10.1590/S0037-86822007000400022
Dabanch P Jeannette, 2003, Rev. chil. infectol., V20, P47
DANTAS JR J. V, 2006, DATABASE BAT ASS VIR
de Almeida MF, 2011, REV SOC BRAS MED TRO, V44, P140, DOI 10.1590/S0037-86822011005000011
de Almeida MF, 2011, REV INST MED TROP SP, V53, P31, DOI 10.1590/S0036-46652011000100006
de Araujo Jansen, 2012, BMC Research Notes, V5, P690, DOI 10.1186/1756-0500-5-690
Carneiro NFD, 2009, REV SOC BRAS MED TRO, V42, P449, DOI 10.1590/S0037-86822009000400017
de Mattos CA, 2000, J WILDLIFE DIS, V36, P231, DOI 10.7589/0090-3558-36.2.231
DE SALES L., 2015, PLOS ONE, V10
DE SALES L., 2013, VIRUS GENES, V47, P164
Lima FED, 2015, ARCH VIROL, V160, P1831, DOI 10.1007/

s00705-015-2447-6
Lima FED, 2013, VIRUS GENES, V47, P378, DOI 10.1007/s11262-013-0947-6
DE SOUZA A. J. S., 2016, DATA BASE BAT ASS VI
de Souza WM, 2018, VIRUSES-BASEL, V10, DOI 10.3390/v10040143
de Souza WM, 2018, VIROLOGY, V514, P9, DOI 10.1016/j.virol.2017.11.001
de Souza WM, 2017, J GEN VIROL, V98, P225, DOI 10.1099/jgv.0.000671
de Thoisy B, 2009, VECTOR-BORNE ZOONOT, V9, P157, DOI 10.1089/vbz.2007.0280
Delmas O, 2008, PLOS ONE, V3, DOI 10.1371/journal.pone.0002057
Delpietro HA, 2009, J WILDLIFE DIS, V45, P1169, DOI 10.7589/0090-3558-45.4.1169
deMattos CA, 1996, J CLIN MICROBIOL, V34, P1553, DOI 10.1128/JCM.34.6.1553-1558.1996
Dobson AP, 2005, SCIENCE, V310, P628, DOI 10.1126/science.1120872
DOWNS WG, 1963, AM J TROP MED HYG, V12, P640, DOI 10.4269/ajtmh.1963.12.640
Drexler JF, 2015, P NATL ACAD SCI USA, V112, P15190, DOI 10.1073/pnas.1516992112
Drexler JF, 2013, P NATL ACAD SCI USA, V110, P16151, DOI 10.1073/pnas.1308049110
Drexler JF, 2012, J VIROL, V86, P9134, DOI 10.1128/JVI.00800-12
Drexler JF, 2012, NAT COMMUN, V3, DOI 10.1038/ncomms1796
Drexler JF, 2009, PLOS ONE, V4, DOI 10.1371/journal.pone.0006367
DUPONT P. M., DATABASE BAT ASS VIR
Easterbrook JD, 2008, PLOS PATHOG, V4, DOI 10.1371/journal.ppat.1000172
Condori-Condori RE, 2013, EMERG INFECT DIS, V19, P1463, DOI 10.3201/eid1909.130083
Ellison JA, 2014, PLOS NEGLECT TROP D, V8, DOI 10.1371/journal.pntd.0003070
Enríquez-Acevedo T, 2020, AGRON SUSTAIN DEV, V40, DOI 10.1007/s13593-020-00645-0
Epstein JH, 2010, PLOS PATHOG, V6, DOI 10.1371/journal.ppat.1000972
Escalera-Zamudio M, 2018, ECOHEALTH, V15, P132, DOI 10.1007/s10393-017-1297-y
Escalera-Zamudio M, 2016, MBIO, V7, DOI [10.1128/mbio.01425-16, 10.1128/mBio.01425-16]
Escalera-Zamudio M, 2015, J VIROL, V89, P5180, DOI 10.1128/JVI.03452-14
Escobar LE, 2015, EPIDEMIOL INFECT, V143, P1486, DOI 10.1017/S095026881400226X
Escobar LE, 2015, REV INST MED TROP SP, V57, P63, DOI 10.1590/S0036-46652015000100009
ESPINOZA-GOMEZ A., 2019, DATABASE BAT ASS VIR
Fagrouch Z, 2012, J GEN VIROL, V93, P2652, DOI 10.1099/vir.0.044149-0
Fahl WO, 2012, BRAZ J INFECT DIS, V16, P545, DOI 10.1016/j.bjid.2012.07.002
Favi M, 1999, ARCH MED VET, V31, P157
Favoretto SR, 2013, EPIDEMIOL INFECT, V141, P1552, DOI 10.1017/

S0950268813000198
FAVORETTO S. R., 2011, DATABASE BAT ASS VIR
FAVORETTO Silvana Regina, 2002, Rev. Inst. Med. trop. S. Paulo, V44, P91, DOI 10.1590/S0036-46652002000200007
Ferraz Claudia, 2007, Rev. Inst. Med. trop. S. Paulo, V49, P389, DOI 10.1590/S0036-46652007000600010
Findley J.S., 1993, BATS COMMUNITY PERSP
Finoketti F, 2019, ARCH VIROL, V164, P1015, DOI 10.1007/s00705-018-04129-1
Fumagalli MJ, 2019, INFECT GENET EVOL, V75, DOI 10.1016/j.meegid.2019.104036
Gardner A. L, 2007, Marsupials, Xenarthrans, Shrews, and Bats, V1, P690
Gibb R, 2020, NATURE, V584, P398, DOI 10.1038/s41586-020-2562-8
Gilbert AT, 2012, AM J TROP MED HYG, V87, P206, DOI 10.4269/ajtmh.2012.11-0689
Gomes A.A.B., 2012, Arq. Inst. Biol., V79, P611
Gonçalves Marcio A.S., 2002, Rev. Soc. Bras. Med. Trop., V35, P453, DOI 10.1590/S0037-86822002000500006
GONZALEZ J. C., 2009, VETERINARIA, V45, P31
GOODWIN GEORGE G., 1961, BULL AMER MUS NAT HIST, V122, P187
Guarino H, 2013, VIRUS RES, V173, P415, DOI 10.1016/j.virusres.2012.12.013
Dohmen FG, 2009, REV SCI TECH OIE, V28, P987, DOI 10.20506/rst.28.3.1943
Guzmán C, 2019, ACTA TROP, V191, P178, DOI 10.1016/j.actatropica.2018.12.016
Hayman DTS, 2013, ZOONOSES PUBLIC HLTH, V60, P2, DOI 10.1111/zph.12000
Heinemann MB, 2002, EPIDEMIOL INFECT, V128, P503, DOI 10.1017/S095026880200688X
Duarte NFH, 2020, ZOONOSES PUBLIC HLTH, V67, P186, DOI 10.1111/zph.12670
Huang ZX, 2019, NAT ECOL EVOL, V3, P1110, DOI 10.1038/s41559-019-0913-3
Ito M, 2003, J CLIN VIROL, V26, P317, DOI 10.1016/S1386-6532(02)00048-3
IUCN, 2021, IUCN RED LIST THREAT
James S, 2020, INFECT GENET EVOL, V84, DOI 10.1016/j.meegid.2020.104367
Jebb D, 2020, NATURE, V583, P578, DOI 10.1038/s41586-020-2486-3
JOHNSON HN, 1948, AM J HYG, V47, P189, DOI 10.1093/oxfordjournals.aje.a119196
Johnson N, 2014, VIRUSES-BASEL, V6, P1911, DOI 10.3390/v6051911
KANUKA M. L., 2018, DATABASE BAT ASS VIR
KIMURA L. M. S., 2006, DATABASE BAT ASS VIR
Kobayashi Y, 2007, ARCH VIROL, V152, P1995, DOI 10.1007/s00705-007-1033-y
Kobayashi Y, 2005, J VET MED SCI, V67, P647, DOI 10.1292/jvms.67.647
KOBAYASHI Y., 2009, DATABASE BAT ASS VIR
Kobayashi Y, 2007, J VET MED SCI, V69, P1145, DOI 10.1292/jvms.69.1145
Kohl C, 2012, J VIROL, V86, P1888, DOI 10.1128/JVI.05974-11

Kojaoghlanian T, 2003, REV MED VIROL, V13, P155, DOI 10.1002/rmv.386
Krumbholz A, 2009, INFECT GENET EVOL, V9, P784, DOI 10.1016/j.meegid.2009.04.008
Kunz TH, 2003, BAT ECOLOGY, P3
Langoni H., 2005, J. Venom. Anim. Toxins incl. Trop. Dis, V11, P84, DOI 10.1590/S1678-91992005000100010
Larsen PA, 2007, J MAMMAL, V88, P712, DOI 10.1644/06-MAMM-A-125R.1
LIANG E., 2015, DATABASE BAT ASS VIR
López-Baucells A, 2018, MAMMAL REV, V48, P62, DOI 10.1111/mam.12110
Loureiro LO, 2018, ZOOSYSTEMA, V40, P425, DOI 10.5252/zoosystema2018v40a18
Loza-Rubio E, 2005, EPIDEMIOL INFECT, V133, P927, DOI 10.1017/S095026880500405X
MACEDO C. I., 2004, DATABASE BAT ASS VIR
Maine JJ, 2015, P NATL ACAD SCI USA, V112, P12438, DOI 10.1073/pnas.1505413112
Mantilla-Meluk Hugo, 2014, Occasional Papers Museum of Texas Tech University, V325, P1
Simas PVM, 2015, EMERG INFECT DIS, V21, P729, DOI 10.3201/eid2104.141783
MARTORELLI LFA, 1995, REV SAUDE PUBL, V29, P140, DOI 10.1590/S0034-89101995000200009
Martorelli LFA, 1996, REV SAUDE PUBL, V30, P101, DOI 10.1590/S0034-89101996000100013
McDonough MM, 2008, J MAMMAL, V89, P1306, DOI 10.1644/07-MAMM-A-349.1
da Silva LAM, 2011, REV SOC BRAS MED TRO, V44, P526, DOI 10.1590/S0037-86822011000400027
Menozzi BD, 2020, ZOONOSES PUBLIC HLTH, V67, P138, DOI 10.1111/zph.12663
Menozzi BD, 2017, ARCH VIROL, V162, P1201, DOI 10.1007/s00705-017-3220-9
MINISTERIO DA SAUDE. SECRETARIA DE VIGILANCIA EM SAUDE - SVS, TAB 2 CAS RAIV HUM E
MOCHIZUKI N., 2011, DATABASE BAT ASS VIR
Mochizuki N, 2011, J VET MED SCI, V73, P759, DOI 10.1292/jvms.10-0238
Mollentze N, 2020, P NATL ACAD SCI USA, V117, P9423, DOI 10.1073/pnas.1919176117
MORALES A. A., 1968, CALDASIA, V10, P167
Moras LM, 2015, MAMMALIA, V79, P439, DOI 10.1515/mammalia-2014-0057
Moreira-Soto A, 2015, ZOONOSES PUBLIC HLTH, V62, P501, DOI 10.1111/zph.12181
MOTA S. C., 2010, BRAZ J VET RES ANIM, V47, P74
Mouinga-Ondémé A, 2012, J VIROL, V86, P1255, DOI 10.1128/JVI.06016-11
Murray SM, 2006, J MED PRIMATOL, V35, P225, DOI 10.1111/j.1600-0684.2006.00171.x
Nadin-Davis SA, 2006, EPIDEMIOL INFECT, V134, P1313, DOI 10.1017/S0950268806006297

Nadin-Davis SA, 2002, VIROLOGY, V298, P286, DOI 10.1006/viro.2002.1492
Nadin-Davis SA, 2001, VIRUS RES, V74, P139, DOI 10.1016/S0168-1702(00)00259-8
Nadin-Davis SA, 2006, VIRUS RES, V117, P215, DOI 10.1016/j.virusres.2005.10.006
Nishiyama S, 2014, J GEN VIROL, V95, P1544, DOI 10.1099/vir.0.065219-0
Nogueira Marcelo Rodrigues, 2014, Check List, V10, P808
Okamoto H, 2002, J GEN VIROL, V83, P1291, DOI 10.1099/0022-1317-83-6-1291
Oliveira RD, 2010, VIROLOGY, V405, P352, DOI 10.1016/j.virol.2010.05.030
ONE HEALTH INSTITUTE & KAREN C DRAYER WILDLIFE HEALTH CENTER, 2018, DAT BAT ASS VIR
Pacheco S. M., 2010, Revista Brasileira de Biociencias, V8, P61
Páez A, 2003, J GEN VIROL, V84, P795, DOI 10.1099/vir.0.18899-0
Páez A, 2007, VIRUS RES, V130, P172, DOI 10.1016/j.virusres.2007.06.008
PASSOS E. D. C., 1999, BRAZILIAN J VET RES, V36
Passos EC, 1998, REV SAUDE PUBL, V32, P74, DOI 10.1590/S0034-89101998000100011
Pavan AC, 2018, J MAMMAL, V99, P624, DOI 10.1093/jmammal/gyy048
Pavan AC, 2017, MOL PHYLOGENET EVOL, V108, P61, DOI 10.1016/j.ympev.2017.01.017
Pawan J. L., 1936, Annals of Tropical Medicine and Parasitology, V30, P101
Pereira AD, 2017, VECTOR-BORNE ZOONOT, V17, P576, DOI 10.1089/vbz.2016.2010
Vieira LFP, 2013, VIRUS GENES, V46, P330, DOI 10.1007/s11262-012-0866-y
Petersen L.R., 2017, Infectious Diseases, VVolume 2, P1493
Piñero C, 2012, PLOS NEGLECT TROP D, V6, DOI 10.1371/journal.pntd.0001635
Platt KB, 2000, J MED ENTOMOL, V37, P965, DOI 10.1603/0022-2585-37.6.965
PRICE JL, 1978, AM J TROP MED HYG, V27, P153, DOI 10.4269/ajtmh.1978.27.153
Quan PL, 2013, P NATL ACAD SCI USA, V110, P8194, DOI 10.1073/pnas.1303037110
QUEIROZ L. H., 2010, DATABASE BAT ASS VIR
Queiroz LH, 2012, ARCH VIROL, V157, P93, DOI 10.1007/s00705-011-1146-1
Queiroz LH, 2009, REV SOC BRAS MED TRO, V42, P9, DOI 10.1590/S0037-86822009000100003
RASCHE A., 2015, DATABASE BAT ASS VIR
Razafindratsimandresy R, 2009, J GEN VIROL, V90, P44, DOI 10.1099/vir.0.006825-0
Sabino-Santos G Jr, 2018, SCI REP-UK, V8, DOI 10.1038/s41598-018-27442-w
Sabino-Santos G, 2015, AM J TROP MED HYG, V93, P404, DOI 10.4269/ajtmh.15-0032
Lima FES, 2015, GENOME ANNOUNCEMENTS, V3, DOI 10.1128/genomeA.01393-15

Salmier A, 2017, PLOS ONE, V12, DOI [10.1371/Journal.pone.0186943, 10.1371/journal.pone.0186943]
Salmón-Mulanovich G, 2009, EMERG INFECT DIS, V15, P1308, DOI 10.3201/eid1508.081522
Sato G, 2009, VIRUS GENES, V38, P74, DOI 10.1007/s11262-008-0290-5
Sato G, 2006, ARCH VIROL, V151, P2243, DOI 10.1007/s00705-006-0770-7
SATO G., 2011, DATABASE BAT ASS VIR
Schaefer R, 2005, VET MICROBIOL, V107, P161, DOI 10.1016/j.vetmic.2005.01.023
Scheffer KC, 2007, REV SAUDE PUBL, V41, P389, DOI 10.1590/S0034-89102007000300010
Seetahal JFR, 2020, VIRUSES-BASEL, V12, DOI 10.3390/v12020178
Cunha EMS, 2006, REV SAUDE PUBL, V40, P1082, DOI 10.1590/S0034-89102006000700017
SEYMOUR C, 1978, AM J TROP MED HYG, V27, P290, DOI 10.4269/ajtmh.1978.27.290
Sheeler-Gordon LL, 2001, J WILDLIFE DIS, V37, P582, DOI 10.7589/0090-3558-37.3.582
Shoji Y, 2006, ACTA VIROL, V50, P33
Shoji Y, 2004, J VET MED SCI, V66, P1271, DOI 10.1292/jvms.66.1271
SILEONI S, 1971, B OFIC SANIT PANAM, V70, P456
Simmons N.B., 2020, Bat species of the world: A taxonomic and geographic database
Simmons NB, 2005, MAMMAL SPECIES WORLD, V1, P312, DOI [DOI 10.1111/J.1365-2656.2010.01742.X, 10.1093/acprof]
SIMSEK C., 2019, DATABASE BAT ASS VIR
SMITH A., 2016, PORCINE RUBULAVIRUS
Sodre Miriam M., 2007, Chiroptera Neotropical, V13, P307
Sotomayor-Bonilla J, 2017, J WILDLIFE DIS, V53, P657, DOI 10.7589/2016-11-249
Sotomayor-Bonilla J, 2014, AM J TROP MED HYG, V91, P129, DOI 10.4269/ajtmh.13-0524
Streicker DG, 2019, P ROY SOC B-BIOL SCI, V286, DOI 10.1098/rspb.2019.1527
Stutz WH, 2004, CHIROPT NEOTROP, V10, P188
Sugay W, 1966, Bol Oficina Sanit Panam, V60, P310
Tao Y, 2013, J GEN VIROL, V94, P738, DOI 10.1099/vir.0.047928-0
THOISY B., 2016, PLOS NEGLECT TROP D, V10
Tiwari R, 2020, VET QUART, V40, P169, DOI 10.1080/01652176.2020.1766725
Tomaz Leonardo A.G., 2007, Chiroptera Neotropical, V13, P309
Tong SX, 2013, PLOS PATHOG, V9, DOI 10.1371/journal.ppat.1003657
Tong SX, 2012, P NATL ACAD SCI USA, V109, P4269, DOI 10.1073/pnas.1116200109
Torres C, 2014, MOL ECOL, V23, P2340, DOI 10.1111/mec.12728
Towner JS, 2009, PLOS PATHOG, V5, DOI 10.1371/journal.ppat.1000536
Uieda W, 1998, REV SAUDE PUBL, V32, P484, DOI 10.1590/S0034-89101998000500012
Uieda W, 1995, REV SAUDE PUBL, V29, P393, DOI 10.1590/S0034-89101995000500009

Uieda Wilson, 2005, Chiroptera Neotropical, V11, P224
Velasco-Villa A, 2002, J CLIN MICROBIOL, V40, P951, DOI 10.1128/JCM.40.3.951-958.2002
Velasco-Villa A, 2006, J CLIN MICROBIOL, V44, P1697, DOI 10.1128/JCM.44.5.1697-1710.2006
Velazco PM, 2019, B AM MUS NAT HIST, P1, DOI 10.1206/0003-0090.429.1.1
Velazco PM, 2013, MOL PHYLOGENET EVOL, V68, P683, DOI 10.1016/j.ympev.2013.04.016
Velazco PM, 2011, AM MUS NOVIT, P1, DOI 10.1206/3710.2
de Barros BDV, 2018, PLOS ONE, V13, DOI 10.1371/journal.pone.0209005
VERA C. S. M., 2014, REV I MED TROPICAL, V2, P7
Vicente-Santos A, 2017, PLOS NEGLECT TROP D, V11, DOI 10.1371/journal.pntd.0005537
Vieira LFP, 2010, ARQ BRAS MED VET ZOO, V62, P343, DOI 10.1590/S0102-09352010000200014
Walker PJ, 2018, J GEN VIROL, V99, P447, DOI 10.1099/jgv.0.001020
Wang LF, 2007, ARCH VIROL, V152, P1259, DOI 10.1007/s00705-007-0959-4
Wang LF, 2011, CURR OPIN VIROL, V1, P649, DOI 10.1016/j.coviro.2011.10.013
Warner CK, 1999, AM J TROP MED HYG, V60, P502, DOI 10.4269/ajtmh.1999.60.502
White RJ, 2020, MAMMAL REV, V50, P336, DOI 10.1111/mam.12201
World Health Organization, 2016, AMBIENT AIR POLLUTIO
Wray AK, 2016, ECOHEALTH, V13, P761, DOI 10.1007/s10393-016-1183-z
Yung V, 2012, EPIDEMIOL INFECT, V140, P2157, DOI 10.1017/S0950268812000520
Yung V, 2002, ARCH VIROL, V147, P2197, DOI 10.1007/s00705-002-0894-3
Zhang GJ, 2013, SCIENCE, V339, P456, DOI 10.1126/science.1230835
NR 253
TC 1
Z9 1
U1 0
U2 15
PU MUSEUM & INST ZOOLOGY PAS-POLISH ACAD SCIENCES
PI WARSAW
PA WILCZA STREET 64, 00-679 WARSAW, POLAND
SN 1508-1109
EI 1733-5329
J9 ACTA CHIROPTEROL
JI Acta Chiropt.
PD DEC
PY 2020
VL 22
IS 2
BP 461
EP 489
DI 10.3161/15081109ACC2020.22.2.018
PG 29
WC Zoology

WE Science Citation Index Expanded (SCI-EXPANDED); Social Science Citation Index (SSCI)
SC Zoology
GA QR8CS
UT WOS:000625441600018
DA 2024-10-21
ER

PT J
AU Castellanos, FX
   Moreno-Santillan, D
   Hughes, GM
   Paulat, NS
   Sipperly, N
   Brown, AM
   Martin, KR
   Poterewicz, GM
   Lim, MCW
   Russell, AL
   Moore, MS
   Johnson, MG
   Corthals, AP
   Ray, DA
   Davalos, LM
AF Castellanos, Francisco X.
   Moreno-Santillan, Diana
   Hughes, Graham M.
   Paulat, Nicole S.
   Sipperly, Nicolette
   Brown, Alexis M.
   Martin, Katherine R.
   Poterewicz, Gregory M.
   Lim, Marisa C. W.
   Russell, Amy L.
   Moore, Marianne S.
   Johnson, Matthew G.
   Corthals, Angelique P.
   Ray, David A.
   Davalos, Liliana M.
TI The evolution of antimicrobial peptides in Chiroptera
SO FRONTIERS IN IMMUNOLOGY
LA English
DT Article
DE innate immunity; defensins; bioinformatics pipelines; non-model
   organisms; gene annotation; transposable elements
ID TRANSPOSABLE ELEMENTS; DEFENSINS; GENES; PREDICTION; UBIQUITIN; REVEALS;
   VIRUSES; MODELS; BATS
AB High viral tolerance coupled with an extraordinary regulation of the immune response makes bats a great model to study host-pathogen evolution. Although many immune-related gene gains and losses have been previously reported in bats, important gene families such as antimicrobial peptides (AMPs) remain understudied. We built an exhaustive bioinformatic pipeline targeting the major gene families

of defensins and cathelicidins to explore AMP diversity and analyze their evolution and distribution across six bat families. A combination of manual and automated procedures identified 29 AMP families across queried species, with alpha-, beta-defensins, and cathelicidins representing around 10% of AMP diversity. Gene duplications were inferred in both alpha-defensins, which were absent in five species, and three beta-defensin gene subfamilies, but cathelicidins did not show significant shifts in gene family size and were absent in Anoura caudifer and the pteropodids. Based on lineage-specific gains and losses, we propose diet and diet-related microbiome evolution may determine the evolution of alpha- and beta-defensins gene families and subfamilies. These results highlight the importance of building species-specific libraries for genome annotation in non-model organisms and shed light on possible drivers responsible for the rapid evolution of AMPs. By focusing on these understudied defenses, we provide a robust framework for explaining bat responses to pathogens.
C1 [Castellanos, Francisco X.; Paulat, Nicole S.; Johnson, Matthew G.; Ray, David A.] Texas Tech Univ, Dept Biol Sci, Lubbock, TX 79409 USA.
[Moreno-Santillan, Diana] Univ Calif Berkeley, Dept Integrat Biol, Berkeley, CA 94720 USA.
[Hughes, Graham M.] Univ Coll Dublin, Sch Biol & Environm Sci, Dublin, Ireland.
[Sipperly, Nicolette; Brown, Alexis M.; Poterewicz, Gregory M.; Lim, Marisa C. W.; Davalos, Liliana M.] SUNY Stony Brook, Dept Ecol & Evolut, Stony Brook, NY 11794 USA.
[Martin, Katherine R.] Univ Cent Florida, Dept Biol, Orlando, FL USA.
[Russell, Amy L.] Grand Valley State Univ, Dept Biol, Allendale, MI USA.
[Moore, Marianne S.] Univ Virgin Isl, Coll Sci & Math, St Thomas, VI USA.
[Corthals, Angelique P.] John Jay Coll Criminal Justice, Dept Sci, New York, NY USA.
[Davalos, Liliana M.] SUNY Stony Brook, Consortium Interdisciplinary Environm Res, Stony Brook, NY 11794 USA.
C3 Texas Tech University System; Texas Tech University; University of
California System; University of California Berkeley; University College
Dublin; State University of New York (SUNY) System; Stony Brook
University; State University System of Florida; University of Central
Florida; Grand Valley State University; University of the Virgin
Islands; City University of New York (CUNY) System; State University of
New York (SUNY) System; Stony Brook University
RP Ray, DA (corresponding author), Texas Tech Univ, Dept Biol Sci, Lubbock, TX 79409 USA.; Moreno-Santillan, D (corresponding author), Univ Calif Berkeley, Dept Integrat Biol, Berkeley, CA 94720 USA.; Davalos, LM (corresponding author), SUNY Stony Brook, Dept Ecol & Evolut, Stony Brook, NY 11794 USA.; Davalos, LM (corresponding author), SUNY Stony Brook, Consortium Interdisciplinary Environm

Res, Stony Brook, NY 11794 USA.
EM diana.moreno@berkeley.edu; david.4.ray@gmail.com;
liliana.davalos@stonybrook.edu
RI Davalos, Liliana/AEE-7879-2022; Martin, Katherine/HJY-7977-2023
OI Castellanos, Francisco/0000-0003-0955-8185; Davalos,
Liliana/0000-0002-4327-7697; Martin, Katherine/
0000-0002-1634-3484;
Johnson, Matthew/0000-0002-1958-6334
FU This work was funded in part by US Fish and Wildlife Service grant
F14AP00737 to MM, LMD, and ALR. LMD was supported in part by NSF-IOS
2032063 and 2031906, APC was supported in part by NSF-IOS 2031926 and
2032011; NS was supported by a charitable donation [F14AP00737]; US Fish
and Wildlife Service [2031926, 2032011]; NSF-IOS [1838283]; NSF-DEB; IOS
- Texas Tech Department of Biological Sciences
FX The authors thank the High-Performance Computer Center at Texas Tech
University for their unconditional technical support and infrastructure
access throughout the work.This work was funded in part by US Fish and
Wildlife Service grant F14AP00737 to MM, LMD, and ALR. LMD was supported
in part by NSF-IOS 2032063 and 2031906, APC was supported in part by
NSF-IOS 2031926 and 2032011; NS was supported by a charitable donation
by Pamela Hurst-Della Pietra to the Davalos Lab; DAR and DM-S were
supported by NSF-DEB 1838283 and IOS 2032006. FXC was funded by a
fellowship granted by the Texas Tech Department of Biological Sciences.
GMH was funded by a UCD Ad Astra scholarship.r This work was funded in
part by US Fish and Wildlife Service grant F14AP00737 to MM, LMD, and
ALR. LMD was supported in part by NSF-IOS 2032063 and 2031906, APC was
supported in part by NSF-IOS 2031926 and 2032011; NS was supported by a
charitable donation by Pamela Hurst-Della Pietra to the Davalos Lab; DAR
and DM-S were supported by NSF-DEB 1838283 and IOS 2032006. FXC was
funded by a fellowship granted by the Texas Tech Department of
Biological Sciences. GMH was funded by a UCD Ad Astra scholarship.
CR Ahn M, 2019, NAT MICROBIOL, V4, P789, DOI 10.1038/s41564-019-0371-3
Andrews S., 2010, FASTQC QUALITY CONTR

Barreiro LB, 2010, NAT REV GENET, V11, P17, DOI 10.1038/nrg2698
Baxter AA, 2017, CELL DEATH DIS, V8, DOI 10.1038/cddis.2017.69
Camacho C, 2009, BMC BIOINFORMATICS, V10, DOI 10.1186/1471-2105-10-421
Cerbin S, 2018, CURR OPIN GENET DEV, V49, P63, DOI 10.1016/j.gde.2018.03.005
Ch'ng L, 2022, TRANSBOUND EMERG DIS, V69, P3917, DOI 10.1111/tbed.14762
Chang CI, 2006, ANTIMICROB AGENTS CH, V50, P185, DOI 10.1128/AAC.50.1.185-195.2006
Chen SF, 2018, BIOINFORMATICS, V34, P884, DOI 10.1093/bioinformatics/bty560
Chen XY, 2020, MAR DRUGS, V18, DOI 10.3390/md18010030
Cheng M, 2015, CLIN EXP GASTROENTER, V8, P13, DOI 10.2147/CEG.S70906
Chinchar VG, 2004, VIROLOGY, V323, P268, DOI 10.1016/j.virol.2004.02.029
Cho JH, 2009, BBA-BIOMEMBRANES, V1788, P1564, DOI 10.1016/j.bbamem.2008.10.025
Choi M, 2022, ANTIBIOTICS-BASEL, V11, DOI 10.3390/antibiotics11080989
Conibear AC, 2014, ANGEW CHEM INT EDIT, V53, P10612, DOI 10.1002/anie.201402167
Delves PJ, 2017, Roitt's essential immunology, V13th
Dong YJ, 2019, BIOMOLECULES, V9, DOI 10.3390/biom9070254
Dorin J.R., 2015, Molecular Medical Microbiology, VVolume 1, P539, DOI DOI 10.1016/C2010-1-67744-9
Edson D, 2019, EPIDEMIOL INFECT, V147, P1, DOI [10.1017/S0950268819001237, 10.1017/s0950268819001237]
Emms DM, 2019, GENOME BIOL, V20, DOI 10.1186/s13059-019-1832-y
Esselstyn JA, 2017, GENOME BIOL EVOL, V9, P2308, DOI 10.1093/gbe/evx168
Fingerhut LCHW, 2020, BIOINFORMATICS, V36, P5262, DOI 10.1093/bioinformatics/btaa653
Ge XY, 2012, J VIROL, V86, P4620, DOI 10.1128/JVI.06671-11
Hadfield JD, 2010, J STAT SOFTW, V33, P1, DOI 10.18637/jss.v033.i02
Hanson MA, 2019, FRONT IMMUNOL, V10, DOI 10.3389/fimmu.2019.02620
Holt C, 2011, BMC BIOINFORMATICS, V12, DOI 10.1186/1471-2105-12-491
Howe KL, 2021, NUCLEIC ACIDS RES, V49, pD884, DOI 10.1093/nar/gkaa942
Ingala MR, 2021, ANIM MICROBIOME, V3, DOI 10.1186/s42523-021-00139-8
Ingala MR, 2018, MSPHERE, V3, DOI 10.1128/mSphere.00397-18
Jebb D, 2020, NATURE, V583, P578, DOI 10.1038/s41586-020-2486-3
Jones P, 2014, BIOINFORMATICS, V30, P1236, DOI 10.1093/bioinformatics/btu031
Katoh K, 2002, NUCLEIC ACIDS RES, V30, P3059, DOI 10.1093/nar/gkf436
Kieffer AE, 2003, FASEB J, V17, P776, DOI 10.1096/fj.02-0699fje
Kines KJ, 2012, FRONT BIOSCI-LANDMRK, V17, P1329, DOI 10.2741/3990
Kuhn M, 2008, J STAT SOFTW, V28, P1, DOI 10.18637/jss.v028.i05

Lazzaro BP, 2020, SCIENCE, V368, P487, DOI 10.1126/science.aau5480
Li CH, 2007, FISH SHELLFISH IMMUN, V22, P663, DOI 10.1016/j.fsi.2006.08.013
Li CK, 2022, BMC GENOMICS, V23, DOI 10.1186/s12864-022-08310-4
Li X, 2022, FRONT IMMUNOL, V13, DOI 10.3389/fimmu.2022.1030610
MaManes MD, 2018, PEERJ, V6, DOI 10.7717/peerj.5428
McDermott Alison M, 2004, Ocul Surf, V2, P229
Meade KG, 2019, FRONT IMMUNOL, V9, DOI 10.3389/fimmu.2018.03072
Mendes FK, 2020, BIOINFORMATICS, V36, P5516, DOI 10.1093/bioinformatics/btaa1022
Minh BQ, 2020, MOL BIOL EVOL, V37, P1530, DOI 10.1093/molbev/msaa015
Santillán DDM, 2021, MOL ECOL, V30, P6449, DOI 10.1111/mec.16027
Nakatsuji T, 2012, J INVEST DERMATOL, V132, P887, DOI 10.1038/jid.2011.387
Nam BH, 2015, FISH SHELLFISH IMMUN, V45, P167, DOI 10.1016/j.fsi.2015.03.035
O'Leary NA, 2016, NUCLEIC ACIDS RES, V44, pD733, DOI 10.1093/nar/gkv1189
Okonechnikov K, 2012, BIOINFORMATICS, V28, P1166, DOI 10.1093/bioinformatics/bts091
Osmanski AB, 2023, SCIENCE, V380, P371, DOI 10.1126/science.abn1430
Pavlovich SS, 2018, CELL, V173, P1098, DOI 10.1016/j.cell.2018.03.070
de la Lastra JMP, 2021, MOLECULES, V26, DOI 10.3390/molecules26061811
Pizzo E, 2018, CURR PHARM DESIGN, V24, P1054, DOI 10.2174/1381612824666180327165012
Purdy GE, 2009, MOL MICROBIOL, V73, P844, DOI 10.1111/j.1365-2958.2009.06801.x
Quinlan AR, 2010, BIOINFORMATICS, V26, P841, DOI 10.1093/bioinformatics/btq033
Randolph HE, 2018, IMMUNITY, V48, P1074, DOI 10.1016/j.immuni.2018.05.016
Ranwez V, 2018, MOL BIOL EVOL, V35, P2582, DOI 10.1093/molbev/msy159
Rognes T, 2016, PEERJ, V4, DOI 10.7717/peerj.2584
Rojas D, 2018, ECOL LETT, V21, P402, DOI 10.1111/ele.12911
Salzman NH, 2010, NAT IMMUNOL, V11, P76, DOI 10.1038/ni.1825
Sanderson MJ, 2003, BIOINFORMATICS, V19, P301, DOI 10.1093/bioinformatics/19.2.301
Sankaran-Walters S, 2017, FRONT MICROBIOL, V8, DOI 10.3389/fmicb.2017.00647
Shen W, 2016, PLOS ONE, V11, DOI 10.1371/journal.pone.0163962
Sievers F, 2018, PROTEIN SCI, V27, P135, DOI 10.1002/pro.3290
Simmons N. B., 2023, BAT SPECIES WORLD TA
Sironi M, 2015, NAT REV GENET, V16, P224, DOI 10.1038/nrg3905
Slater GS, 2005, BMC BIOINFORMATICS, V6, DOI 10.1186/1471-2105-6-31
Solanki SS, 2021, MICROB PATHOGENESIS, V155, DOI 10.1016/j.micpath.2021.104930
Sousa FH., 2016, Antiviral host defence peptides, P57, DOI

[10.1007/978-3-319-32949-9_3, DOI 10.1007/978-3-319-32949-9_3]
Steenwyk JL, 2021, G3-GENES GENOM GENET, V11, DOI 10.1093/g3journal/jkab250
Teeling EC, 2005, SCIENCE, V307, P580, DOI 10.1126/science.1105113
van Dijk A, 2023, MOL IMMUNOL, V157, P53, DOI 10.1016/j.molimm.2023.03.011
Van Dongen S, 2008, SIAM J MATRIX ANAL A, V30, P121, DOI 10.1137/040608635
Wang GS, 2016, NUCLEIC ACIDS RES, V44, pD1087, DOI 10.1093/nar/gkv1278
Waterhouse RM, 2018, MOL BIOL EVOL, V35, P543, DOI 10.1093/molbev/msx319
Whittington CM, 2008, GENOME RES, V18, P986, DOI 10.1101/gr.7149808
Wilkins D, 2023, Gggenes: Draw Gene Arrow Maps in ggplot2
Wu TD, 2010, BIOINFORMATICS, V26, P873, DOI 10.1093/bioinformatics/btq057
Yang D, 2002, TRENDS IMMUNOL, V23, P291, DOI 10.1016/S1471-4906(02)02246-9
Ye ML, 2020, P NATL ACAD SCI USA, V117, P7905, DOI 10.1073/pnas.1912008117
Yuan JF, 2010, J GEN VIROL, V91, P1058, DOI 10.1099/vir.0.016378-0
Zhang LJ, 2016, CURR BIOL, V26, pR14, DOI 10.1016/j.cub.2015.11.017
Zhang Y, 2011, PLOS COMPUT BIOL, V7, DOI 10.1371/journal.pcbi.1002046
Zheng DP, 2020, CELL RES, V30, P492, DOI 10.1038/s41422-020-0332-7
Zhu S, 2017, HEREDITY, V118, P453, DOI 10.1038/hdy.2016.117
NR 85
TC 1
Z9 1
U1 8
U2 17
PU FRONTIERS MEDIA SA
PI LAUSANNE
PA AVENUE DU TRIBUNAL FEDERAL 34, LAUSANNE, CH-1015, SWITZERLAND
SN 1664-3224
J9 FRONT IMMUNOL
JI Front. Immunol.
PD SEP 26
PY 2023
VL 14
AR 1250229
DI 10.3389/fimmu.2023.1250229
PG 14
WC Immunology
WE Science Citation Index Expanded (SCI-EXPANDED)
SC Immunology
GA T8XG0
UT WOS:001080753200001
PM 37822944

OA Green Published, gold
DA 2024-10-21
ER

PT J
AU Siew, ZY
   Lai, ZJ
   Ho, QY
   Ter, HC
   Ho, SH
   Wong, ST
   Gani, M
   Leong, PP
   Voon, K
AF Siew, Z. Y.
   Lai, Z. J.
   Ho, Q. Y.
   Ter, H. C.
   Ho, S. H.
   Wong, S. T.
   Gani, M.
   Leong, P. P.
   Voon, K.
TI Bat coronavirus was detected positive from insectivorous bats in Krau
   Wildlife Reserve Forest
SO TROPICAL BIOMEDICINE
LA English
DT Article
DE Bat diversity; Coronavirus; Paramyxovirus; Pteropine orthoreovirus;
   Dengue virus
AB Bats are flying mammals with unique immune systems that allow them to hold many pathogens. Hence, they are recognised as the reservoir of many zoonotic pathogens. In this study, we performed molecular detection to detect coronaviruses, paramyxoviruses, pteropine orthoreoviruses and dengue viruses from samples collected from insectivorous bats in Krau Reserve Forest. One faecal sample from Rhinolophus spp. was detected positive for coronavirus. Based on BLASTN, phylogenetic analysis and pairwise alignment-based sequence identity calculation, the detected bat coronavirus is most likely to be a bat betacoronavirus lineage slightly different from coronavirus from China, Philippines, Thailand and Luxembourg. In summary, continuous surveillance of bat virome should be encouraged, as Krau Reserve Forest reported a wide spectrum of biodiversity of insectivorous and fruit bats. Moreover, the usage of primers for the broad detection of viruses should be reconsidered because geographical variations might possibly affect the sensitivity of primers in a molecular approach.
C1 [Siew, Z. Y.; Voon, K.] Univ Nottingham Malaysia, Semenyih 43500, Selangor, Malaysia.
   [Lai, Z. J.; Ho, Q. Y.; Ter, H. C.; Ho, S. H.; Wong, S. T.; Voon, K.] Int Med Univ, Kuala Lumpur 57000, Malaysia.
   [Gani, M.] Dept Wildlife & Natl Pk Peninsular Malaysia, Kuala

Lumpur 56100, Malaysia.
[Leong, P. P.] Univ Tunku Abdul Rahman, Sg Long 43000, Selangor, Malaysia.
C3 University of Nottingham Malaysia; International Medical University
Malaysia; Universiti Tunku Abdul Rahman (UTAR)
RP Voon, K (corresponding author), Univ Nottingham Malaysia, Semenyih 43500, Selangor, Malaysia.; Voon, K (corresponding author), Int Med Univ, Kuala Lumpur 57000, Malaysia.
EM Kenny.Voon@nottingham.edu.my
RI Siew, Zhen Yun/JYO-9716-2024
OI Siew, Zhen Yun/0000-0001-7598-6781
FU MyOHUNgrant, FRGS 2013-1 [ID1763]; IMU grant [BMSc I-2018 (03), BMSI-2018 (04)]; UNM pump priming grant [BMSI-2018 (04)]
FX This study was supported in part by the MyOHUNgrant, FRGS 2013-1 (Project ID1763) grant, UNM pump priming grant (F0013.54.04) , IMU grant
BMSc I-2018 (03) and IMU grant BMSI-2018 (04) .
CR Ahmad C.B., 2018, Asian Journal of Environment-Behaviour Studies, V3, P33, DOI [10.21834/aje-bs.v3i8.277, DOI 10.21834/AJE-BS.V3I8.277]
Banerjee A, 2020, FRONT IMMUNOL, V11, DOI 10.3389/fimmu.2020.00026
Biswas R, 2020, REV SCI TECH OIE, V39, P1077, DOI 10.20506/rst.39.3.3198
Boyles JG, 2011, SCIENCE, V332, P41, DOI 10.1126/science.1201366
Chua KB, 2001, VIROLOGY, V283, P215, DOI 10.1006/viro.2000.0882
Edgar RC, 2004, NUCLEIC ACIDS RES, V32, P1792, DOI 10.1093/nar/gkh340
Flaquer C, 2007, J MAMMAL, V88, P526, DOI 10.1644/06-MAMM-A-135R1.1
Habeebur-Rahman SP, 2023, VET MED SCI, V9, P2634, DOI 10.1002/vms3.1251
Hall T., 1999, NUCL ACIDS S SER, P95, DOI [DOI 10.14344/IOC.ML.11.1, 10.1021/bk-1999-0734.ch008]
Howley P M., 2020, Fields virology: Emerging viruses
Kingston T., 2006, Bats of Krau Wildlife Reserve
Letko M, 2020, NAT REV MICROBIOL, V18, P461, DOI 10.1038/s41579-020-0394-z
Maine JJ, 2015, P NATL ACAD SCI USA, V112, P12438, DOI 10.1073/pnas.1505413112
Muhire BM, 2014, PLOS ONE, V9, DOI 10.1371/journal.pone.0108277
Poon Leo L. M., 2008, V454, P13, DOI 10.1007/978-1-59745-181-9_2
Sakoui Souraya, 2020, International Journal of Ecology, V2020, P9872532
Shi ZL, 2013, SCI CHINA LIFE SCI, V56, P678, DOI 10.1007/s11427-013-4517-x
Siew ZY, 2023, DNA CELL BIOL, V42, P289, DOI 10.1089/dna.2022.0561
Tamura K, 2021, MOL BIOL EVOL, V38, P3022, DOI 10.1093/molbev/msab120
Tan CS, 2023, VIROL J, V20, DOI 10.1186/s12985-023-02133-9
Tan CS, 2021, BMC RES NOTES, V14, DOI 10.1186/s13104-021-05880-6
Tee KK, 2023, J MED VIROL, V95, DOI 10.1002/jmv.28520

Teeling EC, 2018, ANNU REV ANIM BIOSCI, V6, P23, DOI 10.1146/annurev-animal-022516-022811
Teo CHJ, 2017, TROP BIOMED, V34, P583
Tippmann HF, 2004, BRIEF BIOINFORM, V5, P82, DOI 10.1093/bib/5.1.82
Tong S, 2008, J CLIN MICROBIOL, V46, P2652, DOI 10.1128/JCM.00192-08
Wang J, 2023, NAT COMMUN, V14, DOI 10.1038/s41467-023-39835-1
NR 27
TC 0
Z9 0
U1 1
U2 3
PU MALAYSIAN SOC PARASITOLOGY TROPICAL MEDICINE
PI KUALA LUMPUR
PA 21-5-2, BLOCK, E, DIAMOND SQUARE, JALAN 3-50, OFF JALAN GOMBAK, KUALA
LUMPUR, MALAYSIA
SN 0127-5720
J9 TROP BIOMED
JI Trop. Biomed.
PD DEC
PY 2023
VL 40
IS 4
BP 462
EP 470
DI 10.47665/tb.40.4.012
PG 9
WC Parasitology; Tropical Medicine
WE Science Citation Index Expanded (SCI-EXPANDED)
SC Parasitology; Tropical Medicine
GA GR1N6
UT WOS:001154307400001
PM 38308834
OA Bronze
DA 2024-10-21
ER

PT J
AU Melhado, G
Herrera, LG
da Cruz-Neto, AP
AF Melhado, Gabriel
Herrera M., L. Gerardo
da Cruz-Neto, Ariovaldo P.
TI Bats respond to simulated bacterial infection during the active phase by
reducing food intake
SO JOURNAL OF EXPERIMENTAL ZOOLOGY PART A-ECOLOGICAL AND INTEGRATIVE
PHYSIOLOGY
LA English
DT Article
DE bat; Carollia perspicillata; fever; immune response;

leukocytosis; LPS;
   sickness behavior
ID SICKNESS BEHAVIOR; BODY-TEMPERATURE; IMMUNITY; FEVER; COST; IMMUNOLOGY;
   ECOLOGY; STRESS; NIGHT
AB Sickness triggers a series of behavioral and physiological processes collectively known as acute phase response (APR). Bats are known as reservoirs of a broad variety of pathogens and the physiological changes resulting from APR activation have been tested predominantly during the resting phase (daytime) in several species exposed to lipopolysaccharide (LPS). In contrast, behavioral consequences of sickness for bats and other wild mammals have received less attention. We examined the physiological and behavioral consequences of APR activation in a fruit-eating bat (Carollia perspicillata) challenged with LPS during the active phase (nighttime). We measured changes in food intake, body mass, body temperature, total white blood cell counts, and the neutrophil/lymphocyte ratio (N/L). No fever and leukocytosis were observed in bats injected with LPS, but food intake decreased, bats lost body mass and their N/L ratio increased. The effect of LPS on daily energy balance is remarkable and, along with the increase in N/L ratio, it is assumed to be beneficial to fight disease. On the basis of our findings and those with other bats, it is probable that the physiological and behavioral components of the immune response to LPS follow circadian rhythms, but a formal test of this hypothesis is warranted.
C1 [Melhado, Gabriel; da Cruz-Neto, Ariovaldo P.] Univ Estadual Paulista, Dept Biodiversidade, Inst Biociencias, Rio Claro, SP, Brazil.
   [Herrera M., L. Gerardo] Univ Nacl Autonoma Mexico, Inst Biol, Estn Biol Chamela, San Patricio, Jalisco, Mexico.
C3 Universidade Estadual Paulista; Universidad Nacional Autonoma de Mexico
RP da Cruz-Neto, AP (corresponding author), Univ Estadual Paulista, Dept Biodiversidade, Inst Biociencias, Rio Claro, SP, Brazil.; Herrera, LG (corresponding author), Univ Nacl Autonoma Mexico, Inst Biol, Estn Biol Chamela, San Patricio, Jalisco, Mexico.
EM gherrera@ib.unam.mx; ap.cruz-neto@unesp.br
RI Melhado, Gabriel/GRS-6030-2022; Cruz-Neto, Ariovaldo/B-2958-2013
OI Cruz-Neto, Ariovaldo/0000-0001-5270-7276; Melhado,
   Gabriel/0000-0001-7638-6841
FU Direccion General de Asuntos del Personal Academico, Universidad
   Nacional Autonoma de Mexico [814-2018]; Consejo Nacional de Ciencia y
   Tecnologia [490586/2013-9]; Conselho Nacional de Pesquisa e
   Desenvolvimento Tecnologico [43486, 490586/20139]; Fundacao de Amparo a
   Pesquisa do Estado de Sao Paulo [2014/17607-4, 2017-17607-6]
FX Direccion General de Asuntos del Personal Academico, Universidad
   Nacional Autonoma de Mexico, Grant/Award Number: 814-2018; Consejo
   Nacional de Ciencia y Tecnologia, Grant/Award Number: 490586/2013-9;
   Conselho Nacional de Pesquisa e Desenvolvimento Tecnologico,

Grant/Award
   Numbers: 43486, 490586/20139; Fundacao de Amparo a Pesquisa do Estado de
   Sao Paulo, Grant/Award Numbers: 2014/17607-4, 2017-17607-6
CR ADER R, 1993, ANNU REV PSYCHOL, V44, P53, DOI 10.1146/annurev.ps.44.020193.000413
   [Anonymous], 2017, EPIDEMIOL INFECT, DOI DOI 10.1017/S0950268817000966
   Ashley N.T., 2012, Ecoimmunology, P45
   BANET M, 1981, EXPERIENTIA, V37, P1302, DOI 10.1007/BF01948375
   Bilbo SD, 2002, P ROY SOC B-BIOL SCI, V269, P447, DOI 10.1098/rspb.2001.1915
   Bohn SJ, 2016, BEHAVIOUR, V153, P981, DOI 10.1163/1568539X-00003384
   Bonneaud C, 2003, AM NAT, V161, P367, DOI 10.1086/346134
   Burness G, 2010, J EXP BIOL, V213, P1386, DOI 10.1242/jeb.027011
   Cabrera-Martinez LV, 2019, COMP BIOCHEM PHYS A, V229, P93, DOI 10.1016/j.cbpa.2018.12.003
   Cabrera-Martínez LV, 2018, PEERJ, V6, DOI 10.7717/peerj.4627
   Calisher CH, 2006, CLIN MICROBIOL REV, V19, P531, DOI 10.1128/CMR.00017-06
   Carlton ED, 2017, GEN COMP ENDOCR, V242, P83, DOI 10.1016/j.ygcen.2015.11.001
   Davis AK, 2008, FUNCT ECOL, V22, P760, DOI 10.1111/j.1365-2435.2008.01467.x
   Guerrero-Chacón AL, 2018, MAMMAL RES, V63, P397, DOI 10.1007/s13364-018-0375-z
   Hart B.L., 2019, Encyclopedia of Animal Behaviour, V1, DOI DOI 10.1016/B978-0-12-809633-8.20750-4
   Hawley DM, 2011, FUNCT ECOL, V25, P48, DOI 10.1111/j.1365-2435.2010.01753.x
   Johnson RW, 2002, VET IMMUNOL IMMUNOP, V87, P443, DOI 10.1016/S0165-2427(02)00069-7
   Lochmiller RL, 2000, OIKOS, V88, P87, DOI 10.1034/j.1600-0706.2000.880110.x
   Luker FI, 2000, AM J PHYSIOL-REG I, V279, pR610, DOI 10.1152/ajpregu.2000.279.2.R610
   Man K, 2016, SCIENCE, V354, P999, DOI 10.1126/science.aah4966
   Moratelli R, 2015, MEM I OSWALDO CRUZ, V110, P1
   Mühldorfer K, 2013, ZOONOSES PUBLIC HLTH, V60, P93, DOI 10.1111/j.1863-2378.2012.01536.x
   MURRAY MJ, 1979, AM J CLIN NUTR, V32, P593, DOI 10.1093/ajcn/32.3.593
   Nathan C, 2006, NAT REV IMMUNOL, V6, P173, DOI 10.1038/nri1785
   Otálora-Ardila A, 2017, MAMM BIOL, V82, P41, DOI 10.1016/j.mambio.2016.11.002
   Otálora-Ardila A, 2016, PLOS ONE, V11, DOI 10.1371/journal.pone.0164938
   Paksuz S., 2009, TRAKYA U J SCI, V10, P55
   Pollmacher T, 1996, J INFECT DIS, V174, P1040, DOI 10.1093/infdis/174.5.1040
   Scheiermann C, 2013, NAT REV IMMUNOL, V13, P190, DOI 10.1038/nri3386
   Schneeberger K, 2013, J EXP BIOL, V216, P4514, DOI 10.1242/

jeb.090837
   Schneeberger K, 2013, PLOS ONE, V8, DOI 10.1371/
journal.pone.0054023
   Siopes TD, 2008, GEN COMP ENDOCR, V158, P245, DOI 10.1016/
j.ygcen.2008.07.008
   Sköld-Chiriac S, 2015, J EXP BIOL, V218, P2961, DOI 10.1242/
jeb.122150
   Sköld-Chiriac S, 2014, PHYSIOL BIOCHEM ZOOL, V87, P288, DOI
10.1086/674789
   Speakman JR, 2010, J ANIM ECOL, V79, P726, DOI 10.1111/
j.1365-2656.2010.01689.x
   Steiger M, 1999, J ANIM SCI, V77, P2523
   Stockmaier S, 2018, ANIM BEHAV, V140, P141, DOI 10.1016/
j.anbehav.2018.04.021
   Stockmaier S, 2015, BIOL LETTERS, V11, DOI 10.1098/rsbl.2015.0576
   Weise P, 2017, PEERJ, V5, DOI 10.7717/peerj.3570
   Wilckens T, 1997, IMMUNOL TODAY, V18, P418, DOI 10.1016/
S0167-5699(97)01111-0
   Williams JB, 2009, ARDEA, V97, P120, DOI 10.5253/078.097.0115
   WING EJ, 1980, INFECT IMMUN, V28, P771
NR 42
TC 9
Z9 9
U1 0
U2 4
PU WILEY
PI HOBOKEN
PA 111 RIVER ST, HOBOKEN 07030-5774, NJ USA
SN 2471-5638
EI 2471-5646
J9 J EXP ZOOL PART A
JI J. Exp. Zool. Part A-Ecol. Integr. Physiol.
PD OCT
PY 2020
VL 333
IS 8
BP 536
EP 542
DI 10.1002/jez.2399
EA JUL 2020
PG 7
WC Zoology
WE Science Citation Index Expanded (SCI-EXPANDED)
SC Zoology
GA NN7QW
UT WOS:000550434800001
PM 32691525
DA 2024-10-21
ER

PT J
AU Fooks, AR
   Shipley, R
   Markotter, W

Tordo, N
Freuling, CM
Müller, T
McElhinney, LM
Banyard, AC
Rupprecht, CE
AF Fooks, Anthony R.
Shipley, Rebecca
Markotter, Wanda
Tordo, Noel
Freuling, Conrad M.
Mueller, Thomas
McElhinney, Lorraine M.
Banyard, Ashley C.
Rupprecht, Charles E.
TI Renewed Public Health Threat from Emerging Lyssaviruses
SO VIRUSES-BASEL
LA English
DT Article
DE rabies; lyssavirus; bats; emerging; novel; encephalitis; prophylaxis;
zoonoses
ID LONG-FINGERED BATS; RABIES BIOLOGICS; IMMUNE-RESPONSES; VIRUS; VACCINE;
CHIROPTERA; PHYLOGENY; EMERGENCE
AB Pathogen discovery contributes to our knowledge of bat-borne viruses and is linked to the heightened interest globally in bats as recognised reservoirs of zoonotic agents. The transmission of lyssaviruses from bats-to-humans, domestic animals, or other wildlife species is uncommon, but interest in these pathogens remains due to their ability to cause an acute, progressive, invariably fatal encephalitis in humans. Consequently, the detection and characterisation of bat lyssaviruses continues to expand our knowledge of their phylogroup definition, viral diversity, host species association, geographical distribution, evolution, mechanisms for perpetuation, and the potential routes of transmission. Although the opportunity for lyssavirus cross-species transmission seems rare, adaptation in a new host and the possibility of onward transmission to humans requires continued investigation. Considering the limited efficacy of available rabies biologicals it is important to further our understanding of protective immunity to minimize the threat from these pathogens to public health. Hence, in addition to increased surveillance, the development of a niche pan-lyssavirus vaccine or therapeutic biologics for post-exposure prophylaxis for use against genetically divergent lyssaviruses should be an international priority as these emerging lyssaviruses remain a concern for global public health.
C1 [Fooks, Anthony R.; Shipley, Rebecca; McElhinney, Lorraine M.; Banyard, Ashley C.] Who Collaborating Ctr Rabies, OIE Reference Lab Rabies, Anim & Plant Hlth Agcy APHA, Weybridge KT15 3NB, Surrey, England.
[Markotter, Wanda] Univ Pretoria, Fac Hlth Sci, Ctr Viral Zoonoses, Dept Med Virol, ZA-0001 Pretoria, South Africa.
[Tordo, Noel] Inst Pasteur Guinee, Route Donka,BP 4416, Conakry,

Guinea.
   [Freuling, Conrad M.; Mueller, Thomas] WHO Collaborating Ctr
Rabies Surveillance & Res R, OIE Reference Lab, Friedrich Loeffler
Inst FLI, D-17493 Greifswald, Germany.
   [Rupprecht, Charles E.] LYSSA LLC, Cumming, GA 30044 USA.
C3 Animal & Plant Health Agency UK; University of Pretoria;
Friedrich
   Loeffler Institute
RP Fooks, AR (corresponding author), Who Collaborating Ctr Rabies,
OIE Reference Lab Rabies, Anim & Plant Hlth Agcy APHA, Weybridge
KT15 3NB, Surrey, England.
EM Tony.Fooks@apha.gov.uk; rebecca.shipley@apha.gov.uk;
   wanda.markotter@up.ac.za; ntordo@pasteur.fr;
conrad.freuling@fli.de;
   Thomas.Mueller@fli.de; Lorraine.McElhinney@apha.gov.uk;
   ashley.banyard@apha.gov.uk; charles_rupprecht@yahoo.com
RI Rupprecht, Charles/AAI-1544-2020; TORDO, Noël/GQQ-9089-2022;
Markotter,
   Wanda/A-2129-2010; Banyard, Ashley C/C-7998-2011; McElhinney,
   Lorraine/C-7997-2011
OI Muller, Thomas/0000-0002-0959-3653; Banyard, Ashley
   C/0000-0002-1286-9825; Markotter, Wanda/0000-0002-7550-0080;
McElhinney,
   Lorraine/0000-0002-6022-348X; Shipley, Rebecca/
0000-0001-7039-9596;
   Freuling, Conrad/0000-0002-1076-398X; Fooks,
   Anthony/0000-0002-3243-6154; TORDO, Noel/0000-0001-5873-059X
FU Defra [SE0431]; Scottish Government [SV3500]; Welsh Government
[SV3500];
   European Union's Horizon 2020 research and innovation program
under
   RABYD-VAX grant [733176]; South African Research Chair Initiative
   (Department of Science and Innovation) [98339]
FX Members from APHA were supported by Defra, the Scottish
Government and
   Welsh Government through grant SV3500 and by the European Union's
   Horizon 2020 research and innovation program under RABYD-VAX
grant
   agreement No. 733176. R.S. was supported by a PhD studentship
under
   Defra grant SE0431. WM was supported by the South African
Research Chair
   Initiative (of the Department of Science and Innovation and
administered
   by the National Research Foundation of South Africa (UID: 98339).
The
   authors' thank Bernadette Abela-Ridder (World Health
Organization,
   Geneva, Switzerland) for insightful ideas and encouragement.
CR [Anonymous], 2017, F1000RESEARCH, DOI DOI 10.12688/
F1000RESEARCH.10416.1
   Ceballos NA, 2013, EMERG INFECT DIS, V19, P793, DOI 10.3201/
eid1905.121071
   Athingo R, 2020, TROP MED INFECT DIS, V5, DOI 10.3390/

tropicalmed5010012
Badrane H, 2001, J VIROL, V75, P8096, DOI 10.1128/JVI.75.17.8096-8104.2001
Badrane H, 2001, J VIROL, V75, P3268, DOI 10.1128/JVI.75.7.3268-3276.2001
Bahloul C, 1998, VACCINE, V16, P417, DOI 10.1016/S0264-410X(97)00204-1
Banyard A.C., 2020, BAT RABIES, V231, P276
Banyard AC, 2018, J GEN VIROL, V99, P1590, DOI 10.1099/jgv.0.001068
Bonnaud EM, 2019, PLOS PATHOG, V15, DOI 10.1371/journal.ppat.1007799
Botvinkin AD, 2003, EMERG INFECT DIS, V9, P1623, DOI 10.3201/eid0912.030374
Brookes SM, 2005, VACCINE, V23, P4101, DOI 10.1016/j.vaccine.2005.03.037
Coertse J, 2021, VIRUSES-BASEL, V13, DOI 10.3390/v13040576
Coertse J, 2020, EMERG INFECT DIS, V26, P3056, DOI 10.3201/eid2612.203592
Coertse J, 2017, BMC VET RES, V13, DOI 10.1186/s12917-017-0948-0
Davis R, 2013, VIRUS RES, V174, P27, DOI 10.1016/j.virusres.2013.02.008
Fisher CR, 2020, CELL REP, V32, DOI 10.1016/j.celrep.2020.107920
Fooks AR, 2017, NAT REV DIS PRIMERS, V3, DOI 10.1038/nrdp.2017.91
Goodman SM, 2007, J MAMMAL, V88, P1216, DOI 10.1644/06-MAMM-A-285R1.1
Grobler CS, 2021, MICROBIOL RESOUR ANN, V10, DOI 10.1128/MRA.00241-21
Hampson K, 2015, PLOS NEGLECT TROP D, V9, DOI [10.1371/journal.pntd.0003709, 10.1371/journal.pntd.0003786]
Hanlon CA, 2005, VIRUS RES, V111, P44, DOI 10.1016/j.virusres.2005.03.009
Hayman DTS, 2016, PLOS NEGLECT TROP D, V10, DOI 10.1371/journal.pntd.0005266
Horton DL, 2010, J VIROL, V84, P11841, DOI 10.1128/JVI.01153-10
Jallet C, 1999, J VIROL, V73, P225, DOI 10.1128/JVI.73.1.225-233.1999
Johnson N, 2014, VIRUSES-BASEL, V6, P1911, DOI 10.3390/v6051911
Kulzmin IV, 2008, EMERG INFECT DIS, V14, P1887, DOI 10.3201/eid1412.080750
Kuzmin IV, 2012, PLOS PATHOG, V8, DOI 10.1371/journal.ppat.1002786
Letko M, 2020, NAT REV MICROBIOL, V18, P461, DOI 10.1038/s41579-020-0394-z
Liu Y, 2013, J CLIN MICROBIOL, V51, P3499, DOI 10.1128/JCM.01565-13
Malerczyk C, 2014, HUM VACC IMMUNOTHER, V10, P2799, DOI 10.4161/21645515.2014.972741
Malerczyk C, 2009, VACCINE, V27, P5320, DOI 10.1016/j.vaccine.2009.06.095
Markotter W, 2008, VIRUS RES, V135, P10, DOI 10.1016/j.virusres.2008.02.001
Marston DA, 2018, CURR OPIN VIROL, V28, P68, DOI 10.1016/j.coviro.2017.11.007

Marston DA, 2017, GENOME ANNOUNCEMENTS, V5, DOI [10.1128/genomea.01427-16, 10.1128/genomeA.01427-16]
Miller-Butterworth CM, 2007, MOL BIOL EVOL, V24, P1553, DOI 10.1093/molbev/msm076
Miller-Butterworth CM, 2005, J MAMMAL, V86, P1121, DOI 10.1644/05-MAMM-A-021R1.1
Mollentze N, 2020, P NATL ACAD SCI USA, V117, P28859, DOI 10.1073/pnas.2006778117
Mollentze N, 2014, CURR OPIN VIROL, V8, P68, DOI 10.1016/j.coviro.2014.07.004
Nokireki T, 2018, TRANSBOUND EMERG DIS, V65, P593, DOI 10.1111/tbed.12833
Perrin P, 2000, Dev Biol (Basel), V104, P151
Picard-Meyer E, 2019, ZOONOSES PUBLIC HLTH, V66, P254, DOI 10.1111/zph.12535
Quetglas J., 2003, Quercus, V203, P50
Regnault B, 2022, CLIN INFECT DIS, V74, P461, DOI 10.1093/cid/ciab443
Schatz J, 2013, ZOONOSES PUBLIC HLTH, V60, P22, DOI 10.1111/zph.12002
Shipley R, 2021, VIRUSES-BASEL, V13, DOI 10.3390/v13050947
Streicker DG, 2010, SCIENCE, V329, P676, DOI 10.1126/science.1188836
Taylor E, 2019, VACCINE, V37, P2285, DOI 10.1016/j.vaccine.2019.03.037
Troupin C, 2016, PLOS PATHOG, V12, DOI 10.1371/journal.ppat.1006041
Troupin C, 2017, GENOME BIOL EVOL, V9, P3202, DOI 10.1093/gbe/evx236
Walker PJ, 2020, ARCH VIROL, V165, P2737, DOI 10.1007/s00705-020-04752-x
Wallace RM, 2014, PLOS ONE, V9, DOI 10.1371/journal.pone.0107539
Weyer J, 2008, EPIDEMIOL INFECT, V136, P670, DOI 10.1017/S0950268807008965
World Hlth Org, 2018, VACCINE, V36, P5500, DOI 10.1016/j.vaccine.2018.06.061
NR 53
TC 18
Z9 19
U1 0
U2 13
PU MDPI
PI BASEL
PA ST ALBAN-ANLAGE 66, CH-4052 BASEL, SWITZERLAND
EI 1999-4915
J9 VIRUSES-BASEL
JI Viruses-Basel
PD SEP
PY 2021
VL 13
IS 9
AR 1769
DI 10.3390/v13091769
PG 10

WC Virology
WE Science Citation Index Expanded (SCI-EXPANDED)
SC Virology
GA UZ2VI
UT WOS:000702067600001
PM 34578350
OA Green Published, gold
DA 2024-10-21
ER

PT J
AU Marosi, A
AF Marosi, A.
TI Current knowledge on pathogenesis and immunology of rabies
SO MAGYAR ALLATORVOSOK LAPJA
LA Hungarian
DT Article
ID CENTRAL-NERVOUS-SYSTEM; BLOOD-BRAIN-BARRIER; VIRUS-P-PROTEIN; INNATE
IMMUNE-RESPONSES; NITRIC-OXIDE SYNTHASE; BAT RABIES; INTERFERON
ANTAGONIST; MATRIX PROTEIN; PHOSPHOPROTEIN-P; DENDRITIC CELLS
C1 [Marosi, A.] ATE Jarvanytam & Mikrobiol Tanszek, Hungana Krt 23-25, H-1143 Budapest, Hungary.
RP Marosi, A (corresponding author), ATE Jarvanytam & Mikrobiol Tanszek, Hungana Krt 23-25, H-1143 Budapest, Hungary.
EM marosi.andras@umivet.hu
CR Abela-Ridder B, 2016, LANCET GLOB HEALTH, V4, pE780, DOI 10.1016/S2214-109X(16)30245-5
Alter A, 2003, J IMMUNOL, V170, P4497, DOI 10.4049/jimmunol.170.9.4497
[Anonymous], 2017, F1000RESEARCH, DOI DOI 10.12688/F1000RESEARCH.10416.1
Appolinário CM, 2016, AM J TROP MED HYG, V94, P378, DOI 10.4269/ajtmh.15-0361
Baloul L, 2004, J NEUROVIROL, V10, P372, DOI 10.1080/13550280490521122
Baloul L, 2003, BIOCHIMIE, V85, P777, DOI 10.1016/S0300-9084(03)00137-8
Barkhouse DA, 2015, J VIROL, V89, P312, DOI 10.1128/JVI.01572-14
Bauer A, 2014, J VIROL, V88, P14172, DOI 10.1128/JVI.02254-14
Ben Khalifa Y, 2016, SCI REP-UK, V6, DOI 10.1038/srep39420
Both L, 2013, FASEB J, V27, P2055, DOI 10.1096/fj.12-219964
Brzózka K, 2005, J VIROL, V79, P7673, DOI 10.1128/JVI.79.12.7673-7681.2005
Brzózka K, 2006, J VIROL, V80, P2675, DOI 10.1128/JVI.80.6.2675-2683.2006
Caillet-Saguy C, 2015, PROG BIOPHYS MOL BIO, V119, P53, DOI 10.1016/j.pbiomolbio.2015.02.007
Carosella ED, 2001, TRENDS IMMUNOL, V22, P553, DOI 10.1016/S1471-4906(01)02007-5
Chai QQ, 2014, J VIROL, V88, P4698, DOI 10.1128/JVI.03149-13
CHENIK M, 1995, J VIROL, V69, P707, DOI 10.1128/JVI.69.2.707-712.1995
Chopy D, 2011, J NEUROVIROL, V17, P353, DOI 10.1007/

s13365-011-0041-6
Chopy D, 2011, J VIROL, V85, P6657, DOI 10.1128/JVI.00302-11
Davis AD, 2007, J INFECT DIS, V195, P1144, DOI 10.1086/512616
Dietzschold B, 2008, FUTURE VIROL, V3, P481, DOI 10.2217/17460794.3.5.481
Dorfmeier CL, 2013, J VIROL, V87, P9217, DOI 10.1128/JVI.00995-13
Faber M, 2002, J VIROL, V76, P3374, DOI 10.1128/JVI.76.7.3374-3381.2002
Faul EJ, 2010, PLOS PATHOG, V6, DOI 10.1371/journal.ppat.1001016
Fernandes ER, 2011, VIRUS RES, V156, P121, DOI 10.1016/j.virusres.2011.01.006
Finke S, 2003, J VIROL, V77, P12074, DOI 10.1128/JVI.77.22.12074-12082.2003
Finke S, 2003, J GEN VIROL, V84, P1613, DOI 10.1099/vir.0.19128-0
Fooks AR, 2017, NAT REV DIS PRIMERS, V3, DOI 10.1038/nrdp.2017.91
Forró B, 2018, MAGY ALLATORVOSOK, V140, P485
Gluska S, 2014, PLOS PATHOG, V10, DOI 10.1371/journal.ppat.1004348
Gnanadurai CW, 2013, PLOS NEGLECT TROP D, V7, DOI 10.1371/journal.pntd.0002375
Hemachudha T, 2013, LANCET NEUROL, V12, P498, DOI 10.1016/S1474-4422(13)70038-3
Hooper DC, 2009, PLOS NEGLECT TROP D, V3, DOI 10.1371/journal.pntd.0000535
Hooper DC, 1998, J VIROL, V72, P3711, DOI 10.1128/JVI.72.5.3711-3719.1998
Hornung V, 2006, SCIENCE, V314, P994, DOI 10.1126/science.1132505
Horowitz A, 2010, J IMMUNOL, V185, P2808, DOI 10.4049/jimmunol.1000844
Huang JH, 2017, ANTIVIR RES, V141, P1, DOI 10.1016/j.antiviral.2017.01.022
IMPROTA T, 1992, J INTERFERON RES, V12, P87, DOI 10.1089/jir.1992.12.87
Ito N, 2010, J VIROL, V84, P6699, DOI 10.1128/JVI.00011-10
Jackson AC, 2003, J NEUROVIROL, V9, P253, DOI 10.1080/13550280390193975
Jackson AC, 2008, J NEUROVIROL, V14, P368, DOI 10.1080/13550280802216502
Johnson N, 2006, J MED MICROBIOL, V55, P785, DOI 10.1099/jmm.0.46370-0
Kammouni W, 2015, J NEUROVIROL, V21, P370, DOI 10.1007/s13365-015-0320-8
Kawai T, 2008, ANN NY ACAD SCI, V1143, P1, DOI 10.1196/annals.1443.020
Kip E, 2017, CELL DEATH DISCOV, V3, DOI 10.1038/cddiscovery.2017.12
Klingen Y, 2008, J VIROL, V82, P237, DOI 10.1128/JVI.01342-07
Koraka P, 2018, FRONT MICROBIOL, V9, DOI 10.3389/fmicb.2018.00397
Lafon M, 2005, CURR TOP MICROBIOL, V289, P239
Lafon M, 2005, J NEUROVIROL, V11, P82, DOI 10.1080/13550280590900427
LAFON M., 2007, RABIES, P500
Lafon M, 2008, J IMMUNOL, V180, P7506, DOI 10.4049/jimmunol.180.11.7506

Lebrun A, 2015, J IMMUNOL, V195, P4358, DOI 10.4049/jimmunol.1501274
Li JW, 2008, VACCINE, V26, P419, DOI 10.1016/j.vaccine.2007.10.072
Li JW, 2012, J VIROL, V86, P3200, DOI 10.1128/JVI.06699-11
Li XQ, 2005, J VIROL, V79, P10063, DOI 10.1128/JVI.79.15.10063-10068.2005
Liao PH, 2012, PATHOL INT, V62, P619, DOI 10.1111/j.1440-1827.2012.02846.x
Lieu KG, 2013, J VIROL, V87, P8261, DOI 10.1128/JVI.00989-13
Luco S, 2012, PLOS PATHOG, V8, DOI 10.1371/journal.ppat.1003060
Lytle AG, 2015, J VIROL, V89, P2842, DOI 10.1128/JVI.03409-14
MacLachlan NJ, 2011, FENNER'S VETERINARY VIROLOGY, 4TH EDITION, P327, DOI 10.1016/B978-0-12-375158-4.00018-3
Madhu B. P., 2016, VirusDisease, V27, P387, DOI 10.1007/s13337-016-0343-7
Maier T, 2010, CLIN INFECT DIS, V50, P1112, DOI 10.1086/651267
Marosi A, 2019, VACCINE, V37, P4724, DOI 10.1016/j.vaccine.2018.05.066
Masatani T, 2011, VIRUS RES, V155, P168, DOI 10.1016/j.virusres.2010.09.016
Masatani T, 2010, J VIROL, V84, P4002, DOI 10.1128/JVI.02220-09
Matsumoto M, 1999, BIOL CHEM, V380, P699, DOI 10.1515/BC.1999.087
Mehta S., 2015, VirusDisease, V26, P163, DOI 10.1007/s13337-015-0269-5
Messenger SL, 2002, CLIN INFECT DIS, V35, P738, DOI 10.1086/342387
Miao FM, 2017, ARCH VIROL, V162, P247, DOI 10.1007/s00705-016-3081-7
Michlmayr D, 2014, J VIROL, V88, P9553, DOI 10.1128/JVI.03421-13
Mitrabhakdi E, 2005, J NEUROL SCI, V238, P3, DOI 10.1016/j.jns.2005.05.004
Moretta A, 2008, CELL DEATH DIFFER, V15, P226, DOI 10.1038/sj.cdd.4402170
Müller T, 2009, PLOS NEGLECT TROP D, V3, DOI 10.1371/journal.pntd.0000542
Nakamichi K, 2005, J VIROL, V79, P11801, DOI 10.1128/JVI.79.18.11801-11812.2005
Nakamichi K, 2004, J VIROL, V78, P9376, DOI 10.1128/JVI.78.17.9376-9388.2004
Niu XF, 2013, ARCH VIROL, V158, P2297, DOI 10.1007/s00705-013-1743-2
Pasdeloup D, 2005, VIROLOGY, V334, P284, DOI 10.1016/j.virol.2005.02.005
Phares TW, 2006, J IMMUNOL, V176, P7666, DOI 10.4049/jimmunol.176.12.7666
Phares TW, 2010, J IMMUNOL, V185, P5607, DOI 10.4049/jimmunol.1001984
Phoolcharoen W, 2017, PLANT BIOTECHNOL J, V15, P1331, DOI 10.1111/pbi.12719
Piccinotti S, 2016, PLOS PATHOG, V12, DOI 10.1371/journal.ppat.1005753
Pollin R, 2013, CELL MICROBIOL, V15, P200, DOI 10.1111/cmi.12037
Préhaud C, 2003, J VIROL, V77, P10537, DOI 10.1128/

JVI.77.19.10537-10547.2003
Préhaud C, 2010, SCI SIGNAL, V3, DOI 10.1126/scisignal.2000510
Prendergast Catriona T., 2009, Endocrine Metabolic & Immune Disorders-Drug Targets, V9, P315
Roche S, 2004, J VIROL, V78, P8746, DOI 10.1128/JVI.78.16.8746-8752.2004
Roy A, 2007, J VIROL, V81, P7993, DOI 10.1128/JVI.00710-07
Roy A, 2007, J VIROL, V81, P1110, DOI 10.1128/JVI.01964-06
Russo MV, 2015, TRENDS IMMUNOL, V36, P637, DOI 10.1016/j.it.2015.08.002
Katz ISS, 2017, ARCH VIROL, V162, P3251, DOI 10.1007/s00705-017-3484-0
Sarmento L, 2005, J NEUROVIROL, V11, P571, DOI 10.1080/13550280500385310
Schnell MJ, 2010, NAT REV MICROBIOL, V8, P51, DOI 10.1038/nrmicro2260
Senba K, 2013, SPRINGERPLUS, V2, DOI 10.1186/2193-1801-2-419
Shimizu K, 2006, MICROBIOL IMMUNOL, V50, P975, DOI 10.1111/j.1348-0421.2006.tb03875.x
SMRECZAK M., 2018, VACCINE
Solanki A, 2009, J NEUROIMMUNOL, V217, P85, DOI 10.1016/j.jneuroim.2009.09.019
Srinivasan A, 2005, NEW ENGL J MED, V352, P1103, DOI 10.1056/NEJMoa043018
Suja M S, 2011, Patholog Res Int, V2011, P374286, DOI 10.4061/2011/374286
Terryn S, 2014, PLOS ONE, V9, DOI 10.1371/journal.pone.0109367
Thoulouze MI, 1998, J VIROL, V72, P7181, DOI 10.1128/JVI.72.9.7181-7190.1998
Tsekoa TL, 2016, PLOS ONE, V11, DOI 10.1371/journal.pone.0159313
Tuffereau C, 1998, EMBO J, V17, P7250, DOI 10.1093/emboj/17.24.7250
Ubol S, 2001, J MED MICROBIOL, V50, P238, DOI 10.1099/0022-1317-50-3-238
Velandia-Romero M.L., 2013, J NEUROVIROL
Vidy A, 2007, J VIROL, V81, P4255, DOI 10.1128/JVI.01930-06
Viktor M, 2008, MAGY ALLATORVOSOK, V130, P629
Wallace RM, 2017, FRONT VET SCI, V4, DOI 10.3389/fvets.2017.00009
Wang LH, 2013, PROTEIN CELL, V4, P901, DOI 10.1007/s13238-013-3918-8
Wang ZW, 2005, J VIROL, V79, P12554, DOI 10.1128/JVI.79.19.12554-12565.2005
Weli SC, 2006, J VIROL, V80, P10270, DOI 10.1128/JVI.01272-06
Wiltzer L, 2014, J INFECT DIS, V209, P1744, DOI 10.1093/infdis/jit829
Yamaoka S, 2013, J VIROL, V87, P12327, DOI 10.1128/JVI.02132-13
Yan XZ, 2001, J NEUROVIROL, V7, P518, DOI 10.1080/135502801753248105
Yang Y, 2015, J VIROL, V89, P2157, DOI 10.1128/JVI.02092-14
Zampieri N, 2014, NEURON, V81, P766, DOI 10.1016/j.neuron.2013.12.033
Zhang GQ, 2013, VET MICROBIOL, V162, P601, DOI 10.1016/j.vetmic.2012.11.031
Zhang R, 2017, ONCOTARGET, V8, P43822, DOI 10.18632/

oncotarget.16517
NR 116
TC 1
Z9 1
U1 2
U2 10
PU MEZOGAZDA KIADO KFT
PI BUDAPEST
PA PO BOX 16, 1631 BUDAPEST, HUNGARY
SN 0025-004X
J9 MAGY ALLATORVOSOK
JI Magy. Allatorv. Lapja
PD OCT
PY 2019
VL 141
IS 10
BP 607
EP 622
PG 16
WC Veterinary Sciences
WE Science Citation Index Expanded (SCI-EXPANDED)
SC Veterinary Sciences
GA JI6KH
UT WOS:000493575700004
DA 2024-10-21
ER

PT J
AU Mortlock, M
   Geldenhuys, M
   Dietrich, M
   Epstein, JH
   Weyer, J
   Paweska, JT
   Markotter, W
AF Mortlock, Marinda
   Geldenhuys, Marike
   Dietrich, Muriel
   Epstein, Jonathan H.
   Weyer, Jacqueline
   Paweska, Janusz T.
   Markotter, Wanda
TI Seasonal shedding patterns of diverse henipavirus-related
   paramyxoviruses in Egyptian rousette bats
SO SCIENTIFIC REPORTS
LA English
DT Article
ID FRUIT BAT; NIPAH VIRUS; MOLECULAR-DETECTION; MARBURG VIRUS;
   SOUTH-AFRICA; AEGYPTIACUS; INFECTION; REPRODUCTION; PTEROPODIDAE;
   SPILLOVER
AB Bat-borne viruses in the Henipavirus genus have been associated
with zoonotic diseases of high morbidity and mortality in Asia and
Australia. In Africa, the Egyptian rousette bat species (Rousettus
aegyptiacus) is an important viral host in which Henipavirus-related

viral sequences have previously been identified. We expanded these
findings by assessing the viral dynamics in a southern African bat
population. A longitudinal study of henipavirus diversity and
excretion dynamics identified 18 putative viral species circulating
in a local population, three with differing seasonal dynamics, and
the winter and spring periods posing a higher risk of virus
spillover and transmission. The annual peaks in virus excretion are
most likely driven by subadults and may be linked to the waning of
maternal immunity and recolonization of the roost in early spring.
These results provide insightful information into the bat-host
relationship that can be extrapolated to other populations across
Africa and be communicated to at-risk communities as a part of
evidence-based public health education and prevention measures
against pathogen spillover threats.
C1 [Mortlock, Marinda; Geldenhuys, Marike; Epstein, Jonathan H.;
Weyer, Jacqueline; Paweska, Janusz T.; Markotter, Wanda] Univ
Pretoria, Dept Med Virol, Ctr Viral Zoonoses, ZA-0001 Pretoria,
South Africa.
[Dietrich, Muriel] UMR Processus Infectieux Milieu Insulaire
Trop, F-97490 Sainte Clotilde, Reunion, France.
[Epstein, Jonathan H.] EcoHlth Alliance, New York, NY 10001 USA.
[Weyer, Jacqueline; Paweska, Janusz T.] Natl Inst Communicable
Dis, Ctr Emerging Zoonot & Parasit Dis, Natl Hlth Lab Serv, ZA-2131
Johannesburg, South Africa.
[Weyer, Jacqueline; Paweska, Janusz T.] Univ Witwatersrand, Sch
Pathol, Dept Microbiol & Infect Dis, ZA-2131 Johannesburg, South
Africa.
C3 University of Pretoria; EcoHealth Alliance; National Institute
for
Communicable Diseases (NICD); National Health Laboratory Service;
University of Witwatersrand
RP Markotter, W (corresponding author), Univ Pretoria, Dept Med
Virol, Ctr Viral Zoonoses, ZA-0001 Pretoria, South Africa.
EM wanda.markotter@up.ac.za
RI Markotter, Wanda/A-2129-2010; dietrich, muriel/A-6430-2015;
Geldenhuys,
Marike/AAL-4868-2020
OI Dietrich, Muriel/0000-0002-2665-7970; Geldenhuys,
Marike/0000-0003-4005-118X
FU National Research Foundation (NRF) of South Africa: the DSI-NRF
South
African Research Chair [98339]; NRF Doctoral bursary [82938]; NRF
[UID:78566]; Poliomyelitis Research Foundation [14/16, 13/47];
Department of the Defense, Defense Threat Reduction Agency
[HDTRA1-20-1-0025]; South African Medical Research Council
(SA-MRC)-Self-Initiated Research (SIR) Grant; University of
Pretoria
FX We would like to thank all the staff and students at the
Biosurveillance
and Ecology of Emerging Zoonoses Research group at the University
of
Pretoria and the Centre for Emerging Zoonotic and Parasitic
Diseases at
the National Institute for Communicable Diseases for their

assistance
with the monthly collection of samples. Additionally, we thank the South
African Weather Service for providing us with weather data. We
acknowledge Marinda Mortlock and Marike Geldenhuys for the images used
in the manuscript. This work was financially supported in part by the
National Research Foundation (NRF) of South Africa: the DSI-NRF South
African Research Chair held by W.M. Grant No. 98339 (including
postdoctoral fellowship funding), an NRF Doctoral bursary (Grant No:
82938). The NRF is also thanked for funding the equipment based at the
DNA Sanger sequencing facility in the Faculty of Natural and
Agricultural Sciences, University of Pretoria (UID:78566), which was
used to generate Sanger sequencing data presented in this work. Opinions
expressed and conclusions arrived at are those of the author and are not
necessarily to be attributed to the NRF. Research and student support
were also provided by the Poliomyelitis Research Foundation through a
research grant (No: 14/16) and student support grant (No: 13/47). The
project or effort depicted was or is sponsored by the Department of the
Defense, Defense Threat Reduction Agency (HDTRA1-20-1-0025). The content
of the information does not necessarily reflect the position or the
policy of the federal government, and no official endorsement should be
inferred. Additional funding was provided by the South African Medical
Research Council (SA-MRC)-Self-Initiated Research (SIR) Grant in support
of this research and publication. M.G. and M.M. were also supported by
the University of Pretoria's postdoctoral funding program.
CR ACR, 2020, AFR CHIR REP 2020
Albarino CG., 2014, EMERG INFECT DIS, V20
Amman BR, 2020, NAT COMMUN, V11, DOI 10.1038/s41467-020-14327-8
Amman BR, 2015, J WILDLIFE DIS, V51, P774, DOI 10.7589/2015-02-044
Anthony SJ, 2017, VIRUS EVOL, V3, DOI 10.1093/ve/vex012
Arunkumar G, 2019, J INFECT DIS, V219, P1867, DOI 10.1093/infdis/jiy612
Baker KS, 2012, J GEN VIROL, V93, P850, DOI 10.1099/vir.0.039339-0
Baker KS, 2014, J ANIM ECOL, V83, P415, DOI

10.1111/1365-2656.12153
Barclay RMR, 2011, CAN J ZOOL, V89, P466, DOI 10.1139/Z11-013
Benda P, 2012, BIOLOGIA, V67, P1230, DOI 10.2478/s11756-012-0105-y
Bouckaert R., 2018, bioRxiv, DOI 10.1101/012401
Chua KB, 2002, MICROBES INFECT, V4, P145, DOI 10.1016/S1286-4579(01)01522-2
Conrardy C, 2014, AM J TROP MED HYG, V91, P258, DOI 10.4269/ajtmh.13-0664
Cumming GS, 1997, OECOLOGIA, V111, P309, DOI 10.1007/s004420050240
Darriba D., 2015, NAT METHODS, V9, P1
Devleesschauwer B., 2014, PREVALENCE PACKAGE, P1
Dietrich M, 2018, ROY SOC OPEN SCI, V5, DOI 10.1098/rsos.180041
Drexler JF, 2012, NAT COMMUN, V3, DOI 10.1038/ncomms1796
Epstein JH, 2020, P NATL ACAD SCI USA, V117, P29190, DOI 10.1073/pnas.2000429117
Epstein JH, 2009, J APPL ECOL, V46, P991, DOI 10.1111/j.1365-2664.2009.01699.x
Field H, 2015, PLOS ONE, V10, DOI 10.1371/journal.pone.0144055
Field H, 2011, PLOS ONE, V6, DOI 10.1371/journal.pone.0028678
Geldenhuys M, 2018, PLOS ONE, V13, DOI 10.1371/journal.pone.0194527
Gibson L, 2021, VIRUSES-BASEL, V13, DOI 10.3390/v13081659
Gokhale MD, 2021, J INFECT PUBLIC HEAL, V14, P1010, DOI 10.1016/j.jiph.2021.05.001
Gurley ES, 2007, EMERG INFECT DIS, V13, P1031, DOI 10.3201/eid1307.061128
Hall T. A., NUCL ACIDS S SER, V41, P95
Halpin K., 2015, Zoonoses - Infections Affecting Humans and Animals, P997, DOI DOI 10.1007/978-94-017-9457-2_40
JACOBSEN NHG, 1976, S AFR J SCI, V72, P270
Jolma ER, 2021, VIRUSES-BASEL, V13, DOI 10.3390/v13081654
Kalenga OI, 2019, NEW ENGL J MED, V381, P373, DOI 10.1056/NEJMsr1904253
Kandeil A, 2019, J VIROL, V93, DOI 10.1128/JVI.01059-18
Lucan RK, 2016, J MAMMAL, V97, P815, DOI 10.1093/jmammal/gyw014
Lucan RK, 2014, J MAMMAL, V95, P1036, DOI 10.1644/14-MAMM-A-035
Markotter W, 2019, TROP MED INFECT DIS, V4, DOI 10.3390/tropicalmed4030099
Martin G, 2018, ECOHEALTH, V15, P526, DOI 10.1007/s10393-017-1309-y
McKee CD, 2021, VIRUSES-BASEL, V13, DOI 10.3390/v13020169
Mélade J, 2016, SCI REP-UK, V6, DOI 10.1038/srep23752
Mollentze N, 2020, P NATL ACAD SCI USA, V117, P9423, DOI 10.1073/pnas.1919176117
Mortlock M, 2019, VIRUSES-BASEL, V11, DOI 10.3390/v11010037
Mortlock M, 2015, EMERG INFECT DIS, V21, P1840, DOI 10.3201/eid2110.140368
Muleya W, 2014, J VET MED SCI, V76, P611, DOI 10.1292/jvms.13-0518
MUTERE F A, 1968, Acta Tropica, V25, P97
Olival KJ, 2017, NATURE, V546, P646, DOI 10.1038/nature22975
Paez DJ, 2017, EPIDEMIOL INFECT, V145, P3143, DOI 10.1017/

S0950268817002138
Paweska JT, 2020, EMERG INFECT DIS, V26, P3051, DOI 10.3201/eid2612.202108
Paweska JT, 2018, EMERG INFECT DIS, V24, P1134, DOI 10.3201/eid2406.172165
Peel AJ, 2019, EMERG MICROBES INFEC, V8, P1314, DOI 10.1080/22221751.2019.1661217
Peel AJ, 2018, SCI REP-UK, V8, DOI 10.1038/s41598-018-22236-6
Pernet O, 2014, NAT COMMUN, V5, DOI 10.1038/ncomms6342
Pinky L, 2016, PLOS ONE, V11, DOI 10.1371/journal.pone.0155589
Plowright RK, 2008, P ROY SOC B-BIOL SCI, V275, P861, DOI 10.1098/rspb.2007.1260
R Core Team, 2022, R LANG ENV STAT COMP
Sasaki M, 2018, INFECT GENET EVOL, V63, P104, DOI 10.1016/j.meegid.2018.05.016
Strachinis I, 2018, MAMMALIA, V82, P611, DOI 10.1515/mammalia-2017-0063
THOMAS DW, 1984, J ZOOL, V202, P265
Tong S, 2008, J CLIN MICROBIOL, V46, P2652, DOI 10.1128/JCM.00192-08
van Vuren PJ, 2018, SCI REP-UK, V8, DOI 10.1038/s41598-018-27836-w
Waruhiu C, 2017, VIROL SIN, V32, P101, DOI 10.1007/s12250-016-3930-2
Weiss S, 2012, EMERG INFECT DIS, V18, P1536, DOI 10.3201/eid1809.111607
Wilkinson DA, 2014, J VIROL, V88, P8268, DOI 10.1128/JVI.01211-14
NR 61
TC 9
Z9 10
U1 1
U2 13
PU NATURE PORTFOLIO
PI BERLIN
PA HEIDELBERGER PLATZ 3, BERLIN, 14197, GERMANY
SN 2045-2322
J9 SCI REP-UK
JI Sci Rep
PD DEC 20
PY 2021
VL 11
IS 1
AR 24262
DI 10.1038/s41598-021-03641-w
PG 12
WC Multidisciplinary Sciences
WE Science Citation Index Expanded (SCI-EXPANDED)
SC Science & Technology - Other Topics
GA XT0IT
UT WOS:000733281900026
PM 34930962
OA gold, Green Published
DA 2024-10-21
ER

PT J
AU Zhou, P
   Chionh, YT
   Irac, SE
   Ahn, M
   Ng, JHJ
   Fossum, E
   Bogen, B
   Ginhoux, F
   Irving, AT
   Dutertre, CA
   Wang, LF
AF Zhou, Peng
   Chionh, Yok Teng
   Irac, Sergio Erdal
   Ahn, Matae
   Ng, Justin Han Jia
   Fossum, Even
   Bogen, Bjarne
   Ginhoux, Florent
   Irving, Aaron T.
   Dutertre, Charles-Antoine
   Wang, Lin-Fa
TI Unlocking bat immunology: establishment of <i>Pteropus alecto</i> bone
   marrow-derived dendritic cells and macrophages
SO SCIENTIFIC REPORTS
LA English
DT Article
ID NIPAH VIRUS; FRUIT BAT; CYTOKINE; GENERATION; RESERVOIRS; INFECTION;
   DISTINCT; NUMBERS; DCS
AB Bats carry and shed many emerging infectious disease agents including Ebola virus and SARS-like Coronaviruses, yet they rarely display clinical symptoms of infection. Bat epithelial or fibroblast cell lines were previously established to study the bat immune response against viral infection. However, the lack of professional immune cells such as dendritic cells (DC) and macrophages has greatly limited the significance of current investigations. Using Pteropus alecto (P. alecto) GM-CSF plus IL4, FLT3L and CSF-1, we successfully generated bat bone marrow-derived DC and macrophages. Cells with the phenotype, morphology and functional features of monocyte-derived DC, bona fide DC or macrophages were obtained in GM-CSF/IL4, FLT3L or CSF-1 cultures, respectively. The successful generation of the first bat bone marrow-derived immune cells paves the way to unlocking the immune mechanisms that confer host resilience to pathogens in bats.
C1 [Zhou, Peng; Chionh, Yok Teng; Irac, Sergio Erdal; Ahn, Matae; Ng, Justin Han Jia; Ginhoux, Florent; Irving, Aaron T.; Dutertre, Charles-Antoine; Wang, Lin-Fa] Duke NUS Med Sch, Programme Emerging Infect Dis, 8 Coll Rd, Singapore 169857, Singapore.
   [Fossum, Even; Bogen, Bjarne] Univ Oslo, Oslo Univ Hosp, KG Jebsen Ctr Res Influenza Vaccines, N-0027 Oslo, Norway.

[Bogen, Bjarne] Univ Oslo, Oslo Univ Hosp, Rikshosp, Ctr Immune Regulat,Inst Immunol, N-0424 Oslo, Norway.
[Ginhoux, Florent; Dutertre, Charles-Antoine] ASTAR, BIOPOLIS, Singapore Immunol Network SIgN, 8A Biomed Grove,IMMUNOS Bldg 3-4, Singapore 138648, Singapore.
C3 National University of Singapore; University of Oslo; University of
Oslo; National Hospital Norway; Agency for Science Technology & Research
(A*STAR); A*STAR - Singapore Immunology Network (SIgN)
RP Irving, AT; Dutertre, CA (corresponding author), Duke NUS Med Sch, Programme Emerging Infect Dis, 8 Coll Rd, Singapore 169857, Singapore.; Dutertre, CA (corresponding author), ASTAR, BIOPOLIS, Singapore Immunol Network SIgN, 8A Biomed Grove,IMMUNOS Bldg 3-4, Singapore 138648, Singapore.
EM aaron.irving@duke-nus.edu.sg; charles.dutertre@duke-nus.edu.sg
RI Ginhoux, Florent/AFN-0385-2022; Fossum, Even/W-9136-2019; Peng,
Ziyi/JTT-8210-2023; Irving, Aaron/AAE-8874-2021; Dutertre, Charles
Antoine/JRX-2224-2023; Irac, Sergio Erdal/G-6690-2014; Irving,
Aaron/I-6754-2013
OI Ahn, Matae/0000-0003-2114-8250; Wang, Lin-Fa/0000-0003-2752-0535; Irac,
Sergio Erdal/0000-0001-5622-1409; Fossum, Even/ 0000-0001-7064-0327;
Dutertre, Charles-Antoine/0000-0001-7950-3652; Irving,
Aaron/0000-0002-0196-1570
FU National Research Foundation in Singapore [NRF2012NRF-CRP001-056];
National Research Foundation Singapore [NMRC/BNIG/2026/2014,
NMRC/BNIG/2040/2015]; Research Council of Norway [220642/H10]; Jebsen
Foundation
FX We thank Charlene Foong Shu Fen from the SingHealth Flow Cytometry Core
Platform. We thank Antonio Bertoletti and Anthony T. Tan for the use of
and help with the Amnis Imagestream. We thank members of the CSIRO
Australian Animal Health Laboratory team led by Gary Crameri and the
Queensland Animal Science Precinct team led by Hume Field for their
advice and help in catching and handling bats. This work was supported
by grant NRF2012NRF-CRP001-056 from the National Research Foundation in
Singapore (F.G. and L-F.W.), by the National Research Foundation
Singapore under its cooperative basic research grant new investigator
grant (NMRC/BNIG/2026/2014 for C-A.D and NMRC/BNIG/2040/2015 for A.T.I)
and administered by the Singapore Ministry of Health's National Medical

Research Council, The Research Council of Norway (grant: 220642/H10,
E.F.) and the Jebsen Foundation (B.B.).
CR AKAGAWA KS, 1988, J IMMUNOL, V141, P3383
Banchereau J, 2000, ANNU REV IMMUNOL, V18, P767, DOI 10.1146/annurev.immunol.18.1.767
Biesold SE, 2011, PLOS ONE, V6, DOI 10.1371/journal.pone.0028131
Calisher CH, 2006, CLIN MICROBIOL REV, V19, P531, DOI 10.1128/CMR.00017-06
Chua KB, 2002, MICROBES INFECT, V4, P145, DOI 10.1016/S1286-4579(01)01522-2
Crameri G, 2009, PLOS ONE, V4, DOI 10.1371/journal.pone.0008266
Dutertre CA, 2014, CELL IMMUNOL, V291, P3, DOI 10.1016/j.cellimm.2014.08.006
Eisenberg E, 2013, TRENDS GENET, V29, P569, DOI 10.1016/j.tig.2013.05.010
Fossum E, 2015, EUR J IMMUNOL, V45, P624, DOI 10.1002/eji.201445080
Ge XY, 2013, NATURE, V503, P535, DOI 10.1038/nature12711
Ginhoux F, 2016, NAT IMMUNOL, V17, P34, DOI 10.1038/ni.3324
Ginhoux F, 2014, NAT REV IMMUNOL, V14, P392, DOI 10.1038/nri3671
Halpin K, 2000, J GEN VIROL, V81, P1927, DOI 10.1099/0022-1317-81-8-1927
Harrington LE, 2005, NAT IMMUNOL, V6, P1123, DOI 10.1038/ni1254
INABA K, 1992, J EXP MED, V176, P1693, DOI 10.1084/jem.176.6.1693
Jordan I, 2009, VIRUS RES, V145, P54, DOI 10.1016/j.virusres.2009.06.007
Krüger N, 2013, J VIROL, V87, P13889, DOI 10.1128/JVI.02458-13
Kühl A, 2011, J INFECT DIS, V204, pS840, DOI 10.1093/infdis/jir306
Lauterbach H, 2010, J EXP MED, V207, P2703, DOI 10.1084/jem.20092720
Leroy EM, 2005, NATURE, V438, P575, DOI 10.1038/438575a
Maraskovsky E, 1996, J EXP MED, V184, P1953, DOI 10.1084/jem.184.5.1953
Menachery VD, 2015, NAT MED, V21, P1508, DOI 10.1038/nm.3985
Middleton DJ, 2007, J COMP PATHOL, V136, P266, DOI 10.1016/j.jcpa.2007.03.002
Mildner A, 2013, ADV IMMUNOL, V120, P69, DOI 10.1016/B978-0-12-417028-5.00003-X
Oppmann B, 2000, IMMUNITY, V13, P715, DOI 10.1016/S1074-7613(00)00070-4
Oynebråten I, 2012, SCAND J IMMUNOL, V75, P379, DOI 10.1111/j.1365-3083.2011.02639.x
Pfaffl MW, 2001, NUCLEIC ACIDS RES, V29, DOI 10.1093/nar/29.9.e45
Schlitzer A, 2015, SEMIN CELL DEV BIOL, V41, P9, DOI 10.1016/j.semcdb.2015.03.011
Schlitzer A, 2013, IMMUNITY, V38, P970, DOI 10.1016/j.immuni.2013.04.011
Sétien AA, 1998, VACCINE, V16, P1122, DOI 10.1016/S0264-410X(98)80108-4
Swanepoel R, 1996, EMERG INFECT DIS, V2, P321, DOI 10.3201/eid0204.960407
TRINCHIERI G, 1995, ANNU REV IMMUNOL, V13, P251, DOI 10.1146/

annurev.immunol.13.1.251
   Varol C, 2015, ANNU REV IMMUNOL, V33, P643, DOI 10.1146/annurev-immunol-032414-112220
   Villadangos JA, 2005, IMMUNOL REV, V207, P191, DOI 10.1111/j.0105-2896.2005.00317.x
   Virtue ER, 2011, PLOS ONE, V6, DOI 10.1371/journal.pone.0022488
   Wang LF, 2011, CURR OPIN VIROL, V1, P649, DOI 10.1016/j.coviro.2011.10.013
   Yan ZQ, 2007, IMMUNOL REV, V219, P187, DOI 10.1111/j.1600-065X.2007.00554.x
   Zhang GJ, 2013, SCIENCE, V339, P456, DOI 10.1126/science.1230835
   Zhou P, 2014, PLOS ONE, V9, DOI 10.1371/journal.pone.0103875
   Zhou P, 2011, J IMMUNOL, V186, P3138, DOI 10.4049/jimmunol.1003115
NR 40
TC 25
Z9 26
U1 1
U2 37
PU NATURE PORTFOLIO
PI BERLIN
PA HEIDELBERGER PLATZ 3, BERLIN, 14197, GERMANY
SN 2045-2322
J9 SCI REP-UK
JI Sci Rep
PD DEC 9
PY 2016
VL 6
AR 38597
DI 10.1038/srep38597
PG 10
WC Multidisciplinary Sciences
WE Science Citation Index Expanded (SCI-EXPANDED)
SC Science & Technology - Other Topics
GA EE7RO
UT WOS:000389820900001
PM 27934903
OA Green Published, gold
DA 2024-10-21
ER

PT J
AU Li, J
   Sun, KP
   Dai, WT
   Leng, HX
   Feng, J
AF Li, Jun
   Sun, Keping
   Dai, Wentao
   Leng, Haixia
   Feng, Jiang
TI Divergence in interspecific and intersubspecific gene expression between

two closely related horseshoe bats (<i>Rhinolophus</i>)
SO JOURNAL OF MAMMALOGY
LA English
DT Article
DE closely related species; comparative transcriptomics; gene expression;
phenotypic variation; Rhinolophus; species differentiation
ID HYBRID ZONE; EVOLUTION; DIFFERENTIATION; POPULATIONS; TRANSCRIPTOMICS;
COEXISTENCE; MECHANISMS; CHEMOKINES; PLASTICITY; TELEOSTEI
AB Closely related species have been used as representative systems to investigate the genetic mechanisms involved in the early stages of species differentiation. Previous studies have indicated that variation in gene expression might be a sensitive indicator of initial species divergence, although the role of expression divergence, and especially that associated with phenotypic variation remained relatively undefined. For three organs (cochlea, brain, and liver) from two closely related bat species (Rhinolophus siamensis and R. episcopus), the interspecific and intersubspecific gene expression profiles were compared using transcriptomics in this study. Striking organ specificity of expression was observed, and expression profiles exhibited similarities between cochlea and brain tissues. Numerous differentially expressed genes (DEGs) were identified for each organ in the interspecific comparison (cochlea/brain/liver: 1,069/647/692) and intersubspecific comparison (608/528/368). Functional enrichment analysis indicated vital variation in expression related to the immune system, ion activities, neuronal function, and multisensory system regulation in both comparisons. DEGs relevant to the variation in echolocation calls (RF) were found, and some of them were involved in the pivotal patterns of expression variation. The regulation of immune, ion channel, neural activity, and sophisticated sensory functions at the expression level might be key mechanisms in the early species divergence of bats, and the expression variation related to acoustical signal could have played a crucial part. This study expands our knowledge of gene expression and patterns of variation for three key organs to echolocation at both the interspecific and intersubspecific levels. Further, the framework described here provides insight into the genetic basis of phenotypic variation during the incipient stage of species differentiation.
C1 [Li, Jun; Sun, Keping; Dai, Wentao; Leng, Haixia; Feng, Jiang] Northeast Normal Univ, Jilin Prov Key Lab Anim Resource Conservat & Utili, Changchun 130117, Peoples R China.
[Li, Jun; Sun, Keping; Dai, Wentao; Leng, Haixia] Minist Educ, Key Lab Vegetat Ecol, Changchun 130024, Peoples R China.
[Feng, Jiang] Jilin Agr Univ, Coll Life Sci, Changchun 130118, Peoples R China.
C3 Northeast Normal University - China; Jilin Agricultural University
RP Sun, KP (corresponding author), Northeast Normal Univ, Jilin Prov Key Lab Anim Resource Conservat & Utili, Changchun 130117, Peoples R China.; Sun, KP (corresponding author), Minist Educ, Key Lab Vegetat Ecol, Changchun 130024, Peoples R China.
EM sunkp129@nenu.edu.cn

RI sun, ke/KMX-1265-2024
OI Li, Jun/0000-0002-7083-5359
CR Alvarez M, 2015, MOL ECOL, V24, P710, DOI 10.1111/mec.13055
Balla T, 2006, J ENDOCRINOL, V188, P135, DOI 10.1677/joe.1.06595
Brawand D, 2011, NATURE, V478, P343, DOI 10.1038/nature10532
Bruun JM, 2002, MOL CELL ENDOCRINOL, V190, P91, DOI 10.1016/S0303-7207(02)00007-2
Campbell P, 2007, J MAMMAL, V88, P105, DOI 10.1644/06-MAMM-A-160R1.1
Chan Esther T., 2009, Journal of Biology (London), V8, P33, DOI 10.1186/jbiol130
Chaves JA, 2016, MOL ECOL, V25, P5282, DOI 10.1111/mec.13743
Chen SF, 2018, BIOINFORMATICS, V34, P884, DOI 10.1093/bioinformatics/bty560
Cheng YL, 2017, FRONT ZOOL, V14, DOI 10.1186/s12983-017-0245-6
Davies KTJ, 2013, FRONT ZOOL, V10, DOI 10.1186/1742-9994-10-2
Dayan DI, 2015, MOL ECOL, V24, P3345, DOI 10.1111/mec.13188
Fukushima K, 2020, NAT COMMUN, V11, DOI 10.1038/s41467-020-18090-8
Fushan AA, 2015, AGING CELL, V14, P352, DOI 10.1111/acel.12283
Galván I, 2020, J ZOOL SYST EVOL RES, V58, P519, DOI 10.1111/jzs.12329
Galván I, 2016, P NATL ACAD SCI USA, V113, P10932, DOI 10.1073/pnas.1609724113
Gilad Y, 2006, TRENDS GENET, V22, P456, DOI 10.1016/j.tig.2006.06.002
Goetz F, 2010, MOL ECOL, V19, P176, DOI 10.1111/j.1365-294X.2009.04481.x
Haas F, 2005, IBIS, V147, P649, DOI 10.1111/j.1474-919x.2005.00422.x
Haas F, 2009, MOL ECOL, V18, P294, DOI 10.1111/j.1365-294X.2008.04017.x
Hart T, 2013, BMC GENOMICS, V14, DOI 10.1186/1471-2164-14-778
Huang RC, 2018, BIOMED J, V41, P5, DOI 10.1016/j.bj.2018.02.003
Jebb D, 2020, NATURE, V583, P578, DOI 10.1038/s41586-020-2486-3
Jeukens J, 2010, MOL ECOL, V19, P5389, DOI 10.1111/j.1365-294X.2010.04934.x
Jiang TL, 2008, PROG NAT SCI-MATER, V18, P523, DOI 10.1016/j.pnsc.2007.12.005
Jones G, 2013, FRONT PHYSIOL, V4, DOI 10.3389/fphys.2013.00117
Khaitovich P, 2006, NAT REV GENET, V7, P693, DOI 10.1038/nrg1940
Kim TJ, 2008, MOL CELLS, V26, P373
KING MC, 1975, SCIENCE, V188, P107, DOI 10.1126/science.1090005
Kramer B, 2014, J NAT HIST, V48, P429, DOI 10.1080/00222933.2013.807950
Langfelder P, 2007, BMC SYST BIOL, V1, DOI 10.1186/1752-0509-1-54
Langfelder P, 2008, BMC BIOINFORMATICS, V9, DOI 10.1186/1471-2105-9-559
Langmead B, 2012, NAT METHODS, V9, P357, DOI [10.1038/NMETH.1923, 10.1038/nmeth.1923]
Lencer ES, 2017, BMC GENOMICS, V18, DOI 10.1186/s12864-017-3810-7
Li D, 2001, CELL MOL LIFE SCI, V58, P2085, DOI 10.1007/PL00000838
Li Y, 2010, CURR BIOL, V20, pR55, DOI 10.1016/j.cub.2009.11.042
Liao BY, 2006, MOL BIOL EVOL, V23, P1119, DOI 10.1093/molbev/

msj119
Liu T, 2021, J ZOOL SYST EVOL RES, V59, P294, DOI 10.1111/jzs.12394
Liu T, 2019, MOL PHYLOGENET EVOL, V139, DOI 10.1016/j.ympev.2019.106544
Liu Z, 2011, PLOS ONE, V6, DOI 10.1371/journal.pone.0026618
Mokodongan DF, 2018, MOL PHYLOGENET EVOL, V118, P194, DOI 10.1016/j.ympev.2017.10.005
Nowick K, 2009, P NATL ACAD SCI USA, V106, P22358, DOI 10.1073/pnas.0911376106
Oleksiak MF, 2002, NAT GENET, V32, P261, DOI 10.1038/ng983
Papakostas S, 2014, NAT COMMUN, V5, DOI 10.1038/ncomms5071
Passow CN, 2017, MOL ECOL, V26, P6384, DOI 10.1111/mec.14360
Pavey SA, 2010, ANN NY ACAD SCI, V1206, P110, DOI 10.1111/j.1749-6632.2010.05765.x
Puechmaille SJ, 2011, NAT COMMUN, V2, DOI 10.1038/ncomms1582
Qiao Q, 2016, FRONT PLANT SCI, V7, DOI 10.3389/fpls.2016.01839
Rendón-Anaya M, 2017, GENOME BIOL, V18, DOI 10.1186/s13059-017-1190-6
Restif O, 2010, P ROY SOC B-BIOL SCI, V277, P2247, DOI 10.1098/rspb.2010.0188
Rohlfs RV, 2014, MOL BIOL EVOL, V31, P201, DOI 10.1093/molbev/mst190
Romero IG, 2012, NAT REV GENET, V13, P505, DOI 10.1038/nrg3229
Rose NH, 2018, EVOLUTION, V72, P82, DOI 10.1111/evo.13385
Salorio-Corbetto M, 2017, INT J AUDIOL, V56, P106, DOI 10.1080/14992027.2016.1234071
Schmid-Hempel P, 2003, P ROY SOC B-BIOL SCI, V270, P357, DOI 10.1098/rspb.2002.2265
Shao Y, 2015, SCI REP-UK, V5, DOI 10.1038/srep17262
Sikes RS, 2016, J MAMMAL, V97, P663, DOI 10.1093/jmammal/gyw078
Simoes BF, 2019, MOL BIOL EVOL, V36, P54, DOI 10.1093/molbev/msy192
Sun HJ, 2020, P ROY SOC B-BIOL SCI, V287, DOI 10.1098/rspb.2020.0875
Sun KP, 2016, SCI REP-UK, V6, DOI 10.1038/srep35417
Szklarczyk D, 2019, NUCLEIC ACIDS RES, V47, pD607, DOI [10.1093/nar/gky1131, 10.1093/nar/gkac1000]
Teeling EC, 2018, ANNU REV ANIM BIOSCI, V6, P23, DOI 10.1146/annurev-animal-022516-022811
Trapnell C, 2010, NAT BIOTECHNOL, V28, P511, DOI 10.1038/nbt.1621
Trapnell C, 2009, BIOINFORMATICS, V25, P1105, DOI 10.1093/bioinformatics/btp120
Uebbing S, 2016, MOL ECOL, V25, P2015, DOI 10.1111/mec.13596
Voolstra C, 2007, GENOME RES, V17, P42, DOI 10.1101/gr.5683806
Wang H, 2020, GENOME BIOL EVOL, V12, P3740, DOI 10.1093/gbe/evz250
Wang H, 2018, SCI REP-UK, V8, DOI 10.1038/s41598-018-34333-7
Whitehead A, 2006, P NATL ACAD SCI USA, V103, P5425, DOI 10.1073/pnas.0507648103
Winter PM, 2004, J INFECT DIS, V190, P1618, DOI 10.1086/423328
Wittkopp PJ, 2008, NAT GENET, V40, P346, DOI 10.1038/ng.77
Wolf JBW, 2010, MOL ECOL, V19, P162, DOI 10.1111/j.1365-294X.2009.04471.x

   Yang JR, 2017, MOL BIOL EVOL, V34, P2125, DOI 10.1093/molbev/
msx171
   Zarbock A, 2008, AM J PATHOL, V172, P1, DOI 10.2353/
ajpath.2008.070502
   Zhao HB, 2019, FRONT ZOOL, V16, DOI 10.1186/s12983-019-0336-7
   Zhou XM, 2016, MOL BIOL EVOL, V33, P2670, DOI 10.1093/molbev/
msw150
   Zukowska WB, 2016, J SYST EVOL, V54, P17, DOI 10.1111/jse.12177
NR 76
TC 1
Z9 1
U1 0
U2 5
PU OXFORD UNIV PRESS INC
PI CARY
PA JOURNALS DEPT, 2001 EVANS RD, CARY, NC 27513 USA
SN 0022-2372
EI 1545-1542
J9 J MAMMAL
JI J. Mammal.
PD FEB 17
PY 2023
VL 104
IS 1
BP 62
EP 75
DI 10.1093/jmammal/gyac103
EA DEC 2022
PG 14
WC Zoology
WE Science Citation Index Expanded (SCI-EXPANDED)
SC Zoology
GA 9D4CT
UT WOS:000894936500001
DA 2024-10-21
ER

PT J
AU Corduneanu, A
   Mihalca, AD
   Sándor, AD
   Hornok, S
   Malmberg, M
   Viso, NP
   Bongcam-Rudloff, E
AF Corduneanu, Alexandra
   Mihalca, Andrei Daniel
   Sandor, Attila D.
   Hornok, Sandor
   Malmberg, Maja
   Viso, Natalia Pin
   Bongcam-Rudloff, Erik
TI The heart microbiome of insectivorous bats from Central and South
   Eastern Europe

SO COMPARATIVE IMMUNOLOGY MICROBIOLOGY AND INFECTIOUS DISEASES
LA English
DT Article
DE Bacteria; Chiroptera; Diversity; Zoonotic; Parasite
AB Host associated microbiome not only may affect the individual health-status or provide insights into the species or group specific bacterial communities but may act as early warning signs in the assessment of zoonotic reservoirs, offering clues to predict, prevent and control possible episodes of emerging zoonoses. Bats may be carriers and reservoirs of multiple pathogens such as viruses, bacteria and parasites, showing in the same time robust immunity against many of them. The microbiota plays a fundamental role on the induction, training and function of the host immune system and the immune system has largely evolved in order to maintain the symbiotic relationship of the host with these diverse microbes. Thus, expanding our knowledge on bat-associated microbiome it can be usefully in understanding bats? outstanding immune capacities. The aim of this study was to investigate the presence of different bacterial communities in heart tissue of insectivorous bats, Nyctalus noctula, Pipistrellus pipistrellus and Rhinoplophus hipposideros, from Central and Eastern Europe using high throughput sequencing of variable regions of the 16S rRNA. In addition, species-specific PCRs were used to validate the presence of the vector-borne pathogens Bartonella spp. and Rickettsia spp. In this study we identified a wide variety of bacterial groups, with the most abundant phyla being Proteobacteria and Firmicutes. The results showed that at individual level, the year or location had no effect on the diversity and composition of the microbiome, however host species determined both structure and abundance of the bacterial community. We report the presence of vector-borne bacteria Bartonella spp. in samples of N. noctula and indications of Rickettsia spp. in R. hipposideros. Our results provide a first insight into the bacterial community found in heart tissue of bats from Central and South Eastern Europe.
C1 [Corduneanu, Alexandra; Mihalca, Andrei Daniel; Sandor, Attila D.] Univ Agr Sci & Vet Med Cluj Napoca, Dept Parasitol & Parasit Dis, Cluj Napoca, Romania.
[Sandor, Attila D.; Hornok, Sandor] Univ Vet Med, Dept Parasitol & Zool, Budapest, Hungary.
[Malmberg, Maja] Swedish Univ Agr Sci, Dept Biomed Sci & Vet Publ Hlth, Sect Virol, Box 7028, S-75007 Uppsala, Sweden.
[Malmberg, Maja; Bongcam-Rudloff, Erik] Swedish Univ Agr Sci, Dept Anim Breeding & Genet, SLU Global Bioinformat Ctr, Box 7023, S-75007 Uppsala, Sweden.
[Viso, Natalia Pin] Consejo Nacl Invest Cient & Tecn, Godoy Cruz 2290, RA-1425 Buenos Aires, DF, Argentina.
[Viso, Natalia Pin] INTA CONICET, Inst Agrobiotecnol & Biol Mol, IABiMo, Calle Las Cabahas & Reseros S-N,Casilla Correo 25, RA-1712 Buenos Aires, DF, Argentina.
C3 University of Agricultural Sciences & Veterinary Medicine Cluj Napoca;
University of Veterinary Medicine Budapest; Swedish University of Agricultural Sciences; Swedish University of Agricultural Sciences;

Consejo Nacional de Investigaciones Cientificas y Tecnicas
(CONICET);
Instituto Nacional de Tecnologia Agropecuaria (INTA)
RP Sándor, AD (corresponding author), Univ Agr Sci & Vet Med Cluj
Napoca, Dept Parasitol & Parasit Dis, Cluj Napoca, Romania.
EM attila.sandor@usamvcluj.ro
RI Corduneanu, Alexandra/HSF-4508-2023; Mihalca, Andrei/A-1561-2008;
Malmberg, Maja/H-1890-2014; Sandor, Attila/A-4782-2009
OI Malmberg, Maja/0000-0002-4506-0408; Sandor, Attila/
0000-0001-8852-8341;
Alexandra, Corduneanu/0000-0002-6682-1827; Hornok,
Sandor/0000-0002-1125-5178
FU National Genomics Infrastructure (NGI)/Uppsala Genome Center
(RFI/VR,
Sweden); National Genomics Infrastructure (NGI)/Uppsala Genome
Center
(Science for Life Laboratory, Sweden); RFI/VR, Sweden; Science
for Life
Laboratory, Sweden; COST Action [TD1303]; ERASMUS+program; Janos
Bolyai
Research Scholarship of Hungarian Academy of Science; UNKP New
National
Excellence Program of the MIT [19-4-ATE-10]; Swedish Research
Council
for Environment, Agricultural Sciences and Spatial Planning,
Formas
[221-2012-586]; Marie Curie International Research Staff Exchange
Scheme
within the 7th European Community Framework Program
[PIRSES-GA-2013-612583-DEANN]; [NKFI132794]; [NKFI130216]
FX The authors would like to acknowledge the support of the National
Genomics Infrastructure (NGI)/Uppsala Genome Center (funded by
RFI/VR
and Science for Life Laboratory, Sweden)and the SLU
Bioinformatics
Infrastructure (SLUBI) for providing assistance in massive
parallel
sequencing and computational infrastructure. Work performed at
NGI/Uppsala Genome Center has been funded by RFI/VR and Science
for Life
Laboratory, Sweden. AC was supported by the COST Action TD1303:
European
Network for Neglected Vectors and VectorBorne Infections
(EURNEGVEC) and
ERASMUS+program. The Janos Bolyai Research Scholarship of
Hungarian
Academy of Science, the UNKP 19-4-ATE-10 New National Excellence
Program
of the MIT and NKFI132794 provided financial resources to ADS,
while SH
received funding from NKFI130216. MM received financial support
from the
Swedish Research Council for Environment, Agricultural Sciences
and

Spatial Planning, Formas (Grant Number 221-2012-586). NPV received
funding from the Marie Curie International Research Staff Exchange
Scheme within the 7th European Community Framework Program under grant
agreement No PIRSES-GA-2013-612583-DEANN. The authors would like to
thank for the kind contribution of Peter Estok, Sandor Boldogh
(Hungary), Juliette Hayer and Tomas Klingstr.m (Sweden).
CR [Anonymous], 2009, Bats of Britain, Europe and Northwest Africa
Asangba AE, 2019, AM J PRIMATOL, V81, DOI 10.1002/ajp.23045
Avena CV, 2016, FRONT MICROBIOL, V7, DOI 10.3389/fmicb.2016.01753
Bai Y, 2017, PLOS ONE, V12, DOI 10.1371/journal.pone.0171175
Barb JJ, 2016, PLOS ONE, V11, DOI 10.1371/journal.pone.0148047
Belák S, 2013, VET MICROBIOL, V165, P95, DOI 10.1016/j.vetmic.2013.01.022
Blomström AL, 2011, VET QUART, V31, P107, DOI 10.1080/01652176.2011.604971
Blomström AL, 2010, J CLIN MICROBIOL, V48, P4392, DOI 10.1128/JCM.01040-10
Bolger AM, 2014, BIOINFORMATICS, V30, P2114, DOI 10.1093/bioinformatics/btu170
Carrillo-Araujo M, 2015, FRONT MICROBIOL, V6, DOI 10.3389/fmicb.2015.00447
Cheng J, 2019, INT J INFECT DIS, V83, P148, DOI 10.1016/j.ijid.2019.03.007
Cheng J, 2018, ANN CLIN MICROB ANTI, V17, DOI 10.1186/s12941-018-0294-5
Cicuttin GL, 2017, COMP IMMUNOL MICROB, V52, P1, DOI 10.1016/j.cimid.2017.04.004
Concannon R, 2005, PARASITOLOGY, V131, P489, DOI 10.1017/S0031182005008097
Corduneanu A, 2018, PARASITE VECTOR, V11, DOI 10.1186/s13071-018-3070-7
Dacheux L, 2014, PLOS ONE, V9, DOI 10.1371/journal.pone.0087194
Davoust B, 2016, VECTOR-BORNE ZOONOT, V16, P516, DOI 10.1089/vbz.2015.1918
De Mandal S, 2015, GENOM DATA, V4, P99, DOI 10.1016/j.gdata.2015.04.001
Decuypere S, 2016, PLOS NEGLECT TROP D, V10, DOI 10.1371/journal.pntd.0004470
Dietrich M, 2019, ECOL EVOL, V9, P1730, DOI 10.1002/ece3.4842
Dietrich M, 2017, FEMS MICROBIOL LETT, V364, DOI 10.1093/femsle/fnw284
Donaldson EF, 2010, J VIROL, V84, P13004, DOI 10.1128/JVI.01255-10
Dufkova L, 2015, VECTOR-BORNE ZOONOT, V15, P518, DOI 10.1089/vbz.2015.1813
Edgar RC, 2013, NAT METHODS, V10, P996, DOI [10.1038/NMETH.2604, 10.1038/nmeth.2604]
Fischer K, 2016, INFECT GENET EVOL, V37, P108, DOI 10.1016/j.meegid.2015.11.010
Foley NM, 2018, SCI ADV, V4, DOI 10.1126/sciadv.aao0926

Fouhy F, 2016, BMC MICROBIOL, V16, DOI 10.1186/s12866-016-0738-z
Franco LC, 2019, MICROBIOL RESOUR ANN, V8, DOI [10.1128/MRA.01422-18, 10.1128/mra.01422-18]
Ge XY, 2012, J VIROL, V86, P4620, DOI 10.1128/JVI.06671-11
Han HJ, 2017, PLOS ONE, V12, DOI 10.1371/journal.pone.0167915
Hayman DTS, 2019, NAT MICROBIOL, V4, P728, DOI 10.1038/s41564-019-0430-9
He B, 2013, PLOS ONE, V8, DOI 10.1371/journal.pone.0061950
Holz PH, 2019, INT J PARASITOL-PAR, V8, P10, DOI 10.1016/j.ijppaw.2018.11.008
Hornok S, 2019, PARASITE VECTOR, V12, DOI 10.1186/s13071-019-3303-4
Hornok S, 2016, PLOS ONE, V11, DOI 10.1371/journal.pone.0167735
Hyde ER, 2013, PLOS ONE, V8, DOI 10.1371/journal.pone.0077733
Judson SD, 2015, ZOONOSES PUBLIC HLTH, V62, P609, DOI 10.1111/zph.12188
Kamani J, 2014, VECTOR-BORNE ZOONOT, V14, P625, DOI 10.1089/vbz.2013.1541
Kennedy NA, 2014, PLOS ONE, V9, DOI 10.1371/journal.pone.0088982
Kim M, 2011, J MICROBIOL METH, V84, P81, DOI 10.1016/j.mimet.2010.10.020
Kuczynski Justin, 2012, Curr Protoc Microbiol, VChapter 1, DOI [10.1002/0471250953.bi1007s36, 10.1002/9780471729259.mc01e05s27]
Leli C, 2014, DIAGN MICR INFEC DIS, V79, P98, DOI 10.1016/j.diagmicrobio.2013.12.011
Lemieux-Labonté V, 2016, PEERJ, V4, DOI 10.7717/peerj.2430
Li LL, 2010, J VIROL, V84, P6955, DOI 10.1128/JVI.00501-10
Lilley TM, 2017, VECTOR-BORNE ZOONOT, V17, P243, DOI 10.1089/vbz.2016.2080
Lozupone C, 2011, ISME J, V5, P169, DOI 10.1038/ismej.2010.133
McKee CD, 2019, FRONT ECOL EVOL, V7, DOI 10.3389/fevo.2019.00069
Metcalf JL, 2016, SCIENCE, V351, P158, DOI 10.1126/science.aad2646
Millán J, 2015, COMP IMMUNOL MICROB, V39, P9, DOI 10.1016/j.cimid.2015.01.002
Morse SF, 2012, INFECT GENET EVOL, V12, P1717, DOI 10.1016/j.meegid.2012.06.009
Mühldorfer K, 2011, BMC VET RES, V7, DOI 10.1186/1746-6148-7-61
Muszer M, 2015, ARCH IMMUNOL THER EX, V63, P287, DOI 10.1007/s00005-015-0332-3
Oh J, 2012, GENOME MED, V4, DOI 10.1186/gm378
Pendleton KM, 2017, AM J RESP CRIT CARE, V196, P1610, DOI 10.1164/rccm.201703-0537LE
Pylro VS, 2014, J MICROBIOL METH, V107, P30, DOI 10.1016/j.mimet.2014.08.018
Rogers EJ, 2019, PHYSIOL BIOCHEM ZOOL, V92, P386, DOI 10.1086/704107
Rutanga JP, 2018, EXPERT REV MOL DIAGN, V18, P749, DOI 10.1080/14737159.2018.1498786
Salter SJ, 2014, BMC BIOL, V12, DOI 10.1186/s12915-014-0087-z
Sándor AD, 2018, MICROB ECOL, V76, P1076, DOI 10.1007/s00248-018-1195-z
Stuckey MJ, 2017, EMERG INFECT DIS, V23, P539, DOI 10.3201/eid2303.160934

Tengs T, 2017, DEV COMP IMMUNOL, V75, P109, DOI 10.1016/j.dci.2017.01.025
Tse H, 2012, PLOS ONE, V7, DOI 10.1371/journal.pone.0043986
Urushadze L, 2017, PLOS NEGLECT TROP D, V11, DOI 10.1371/journal.pntd.0005428
Veikkolainen V, 2014, EMERG INFECT DIS, V20, P960, DOI 10.3201/eid2006.130956
Watanabe N, 2018, PLOS ONE, V13, DOI 10.1371/journal.pone.0202049
Wilkinson DA, 2016, APPL ENVIRON MICROB, V82, P1778, DOI 10.1128/AEM.03505-15
Winter AS, 2017, PEERJ, V5, DOI 10.7717/peerj.3944
NR 67
TC 7
Z9 7
U1 0
U2 13
PU ELSEVIER SCI LTD
PI OXFORD
PA THE BOULEVARD, LANGFORD LANE, KIDLINGTON, OXFORD OX5 1GB, OXON, ENGLAND
SN 0147-9571
EI 1878-1667
J9 COMP IMMUNOL MICROB
JI Comp. Immunol. Microbiol. Infect. Dis.
PD APR
PY 2021
VL 75
AR 101605
DI 10.1016/j.cimid.2020.101605
EA JAN 2021
PG 11
WC Immunology; Microbiology; Veterinary Sciences
WE Science Citation Index Expanded (SCI-EXPANDED)
SC Immunology; Microbiology; Veterinary Sciences
GA RC0AK
UT WOS:000632466200012
PM 33421678
OA Green Submitted
DA 2024-10-21
ER

PT J
AU Papenfuss, AT
Baker, ML
Feng, ZP
Tachedjian, M
Crameri, G
Cowled, C
Ng, J
Janardhana, V
Field, HE
Wang, LF
AF Papenfuss, Anthony T.
Baker, Michelle L.

Feng, Zhi-Ping
Tachedjian, Mary
Crameri, Gary
Cowled, Chris
Ng, Justin
Janardhana, Vijaya
Field, Hume E.
Wang, Lin-Fa
TI The immune gene repertoire of an important viral reservoir, the Australian black flying fox
SO BMC GENOMICS
LA English
DT Article
ID LEUKOCYTE RECEPTOR COMPLEX; KILLER-CELL RECEPTORS; FRUIT BATS; RIG-I;
MOLECULAR CHARACTERIZATION; PHYLOGENETIC POSITION; SIGNALING PATHWAYS;
VIRUS-INFECTION; MX-PROTEIN; SEQUENCE
AB Background: Bats are the natural reservoir host for a range of emerging and re-emerging viruses, including SARS-like coronaviruses, Ebola viruses, henipaviruses and Rabies viruses. However, the mechanisms responsible for the control of viral replication in bats are not understood and there is little information available on any aspect of antiviral immunity in bats. Massively parallel sequencing of the bat transcriptome provides the opportunity for rapid gene discovery. Although the genomes of one megabat and one microbat have now been sequenced to low coverage, no transcriptomic datasets have been reported from any bat species. In this study, we describe the immune transcriptome of the Australian flying fox, Pteropus alecto, providing an important resource for identification of genes involved in a range of activities including antiviral immunity.

Results: Towards understanding the adaptations that have allowed bats to coexist with viruses, we have de novo assembled transcriptome sequence from immune tissues and stimulated cells from P. alecto. We identified about 18,600 genes involved in a broad range of activities with the most highly expressed genes involved in cell growth and maintenance, enzyme activity, cellular components and metabolism and energy pathways. 3.5% of the bat transcribed genes corresponded to immune genes and a total of about 500 immune genes were identified, providing an overview of both innate and adaptive immunity. A small proportion of transcripts found no match with annotated sequences in any of the public databases and may represent bat-specific transcripts.

Conclusions: This study represents the first reported bat transcriptome dataset and provides a survey of expressed bat genes that complement existing bat genomic data. In addition, these data provide insight into genes relevant to the antiviral responses of bats, and form a basis for examining the roles of these molecules in immune response to viral infection.
C1 [Baker, Michelle L.; Tachedjian, Mary; Crameri, Gary; Cowled, Chris; Ng, Justin; Janardhana, Vijaya; Wang, Lin-Fa] CSIRO Livestock Ind, Australian Anim Hlth Lab, Geelong, Vic 3220, Australia.
[Baker, Michelle L.] Univ New Mexico, Dept Biol, Ctr Evolutionary & Theoret Immunol, Albuquerque, NM 87131 USA.

[Papenfuss, Anthony T.; Feng, Zhi-Ping] Walter & Eliza Hall Inst Med Res, Bioinformat Div, Parkville, Vic 3052, Australia.
[Papenfuss, Anthony T.] Univ Melbourne, Dept Math & Stat, Melbourne, Vic 3010, Australia.
[Feng, Zhi-Ping] Univ Melbourne, Dept Med Biol, Melbourne, Vic 3010, Australia.
[Field, Hume E.; Wang, Lin-Fa] Australian Biosecur Cooperat Res Ctr Emerging Inf, Brisbane, Qld, Australia.
[Field, Hume E.] Biosecur Queensland, Queensland Primary Ind & Fisheries, Brisbane, Qld, Australia.
C3 Commonwealth Scientific & Industrial Research Organisation (CSIRO);
University of New Mexico; Walter & Eliza Hall Institute; University of
Melbourne; University of Melbourne; Queensland Department of Agriculture
& Fisheries
RP Baker, ML (corresponding author), CSIRO Livestock Ind, Australian Anim Hlth Lab, PO Bag 24, Geelong, Vic 3220, Australia.
EM Michelle.Baker@csiro.au; Linfa.Wang@csiro.au
RI Crameri, Gary/H-8441-2013; Papenfuss, Anthony/C-6297-2008; lu,
yuan/JZD-0832-2024; Feng, Zhi-Ping/L-2083-2016; Tachedjian,
Mary/H-4572-2013; Cowled, Christopher/H-5308-2013; Baker,
Michelle/C-9694-2013
OI Tachedjian, Mary/0000-0002-8936-6564; Wang, Lin-Fa/0000-0003-2752-0535;
Papenfuss, Anthony/0000-0002-1102-8506; Cowled,
Christopher/0000-0002-1969-9371; Baker, Michelle/0000-0002-7993-9971
FU Australian Biosecurity Cooperative Research Centre for Emerging
Infectious Diseases [2.051R]; CSIRO CEO Science Leaders award; NHMRC
Career Development Fellowship
FX We thank Craig Smith, Carol de Jong, Deborah Middleton and Susanne
Wilson for provision of bat tissues and peripheral blood used for this
study and Andrew Bean for advice on mitogen stimulation of bat cells.
The work was supported by funding from the Australian Biosecurity
Cooperative Research Centre for Emerging Infectious Diseases to L-F. W
and H. F. (Project 2.051R), a CSIRO CEO Science Leaders award to L-F. W
and an NHMRC Career Development Fellowship to ATP.
CR Altschul SF, 1997, NUCLEIC ACIDS RES, V25, P3389, DOI 10.1093/nar/25.17.3389
Anderson SK, 2001, IMMUNOL REV, V181, P79, DOI 10.1034/j.1600-065X.2001.1810106.x
[Anonymous], J MOL EVOL
ARNHEITER H, 1990, CELL, V62, P51, DOI 10.1016/0092-8674(90)90239-B
Bairoch A, 2005, NUCLEIC ACIDS RES, V33, pD154, DOI 10.1093/nar/gki070

Baker ML, 2010, IMMUNOGENETICS, V62, P173, DOI 10.1007/s00251-010-0425-4
Bateman A, 2002, NUCLEIC ACIDS RES, V30, P276, DOI [10.1093/nar/gkh121, 10.1093/nar/gkr1065, 10.1093/nar/gkp985]
Beck S, 1999, IMMUNOL REV, V167, P201, DOI 10.1111/j.1600-065X.1999.tb01393.x
Belov K, 2007, GENOME RES, V17, P982, DOI 10.1101/gr.6121807
BERNASCONI D, 1995, J INTERF CYTOK RES, V15, P47, DOI 10.1089/jir.1995.15.47
BODMER JG, 1990, HUM IMMUNOL, V28, P326, DOI 10.1016/0198-8859(90)90060-3
BROWN JH, 1993, NATURE, V364, P33, DOI 10.1038/364033a0
Brown MG, 2008, SEMIN IMMUNOL, V20, P361, DOI 10.1016/j.smim.2008.06.004
Butler JE, 2011, DEV COMP IMMUNOL, V35, P273, DOI 10.1016/j.dci.2010.08.011
Calisher CH, 2006, CLIN MICROBIOL REV, V19, P531, DOI 10.1128/CMR.00017-06
Chevreux B, 2004, GENOME RES, V14, P1147, DOI 10.1101/gr.1917404
Cowled C, 2012, DEV COMP IMMUNOL, V36, P657, DOI 10.1016/j.dci.2011.11.008
Cowled C, 2011, DEV COMP IMMUNOL, V35, P7, DOI 10.1016/j.dci.2010.07.006
Crameri G, 2009, PLOS ONE, V4, DOI 10.1371/journal.pone.0008266
Field H, 1999, EMERG INFECT DIS, V5, P438, DOI 10.3201/eid0503.990316
Flicek P, 2011, NUCLEIC ACIDS RES, V39, pD800, DOI 10.1093/nar/gkq1064
Franchi L, 2009, IMMUNOL REV, V227, P106, DOI 10.1111/j.1600-065X.2008.00734.x
Gao S, 2010, NATURE, V465, P502, DOI 10.1038/nature08972
Haller O, 2007, MICROBES INFECT, V9, P1636, DOI 10.1016/j.micinf.2007.09.010
HEIN WR, 1991, IMMUNOL TODAY, V12, P30, DOI 10.1016/0167-5699(91)90109-7
Hu Z-L., 2008, ONLINE J BIOINFORMAT, V9, P108
HUGHES AL, 1990, MOL BIOL EVOL, V7, P491
Jones KE, 2002, BIOL REV, V77, P223, DOI 10.1017/S1464793101005899
Kato H, 2006, NATURE, V441, P101, DOI 10.1038/nature04734
Kelley J, 2005, PLOS GENET, V1, P129, DOI 10.1371/journal.pgen.0010027
Lamkanfi M, 2012, IMMUNOBIOLOGY, V217, P13, DOI 10.1016/j.imbio.2011.08.011
Lassmann T, 2009, BIOINFORMATICS, V25, P2839, DOI 10.1093/bioinformatics/btp527
Laun K, 2006, PLOS GENET, V2, P707, DOI 10.1371/journal.pgen.0020073
LeMaoult J, 2003, TISSUE ANTIGENS, V62, P273, DOI 10.1034/j.1399-0039.2003.00143.x
Leroy EM, 2005, NATURE, V438, P575, DOI 10.1038/438575a
Leroy EM, 2009, VECTOR-BORNE ZOONOT, V9, P723, DOI 10.1089/vbz.2008.0167
Li WZ, 2006, BIOINFORMATICS, V22, P1658, DOI 10.1093/

bioinformatics/btl158
Madsen O, 2001, NATURE, V409, P610, DOI 10.1038/35054544
Mayer F, 2007, HEREDITY, V99, P257, DOI 10.1038/sj.hdy.6800989
McColl KA, 2002, AUST VET J, V80, P636, DOI 10.1111/j.1751-0813.2002.tb10973.x
Middleton DJ, 2007, J COMP PATHOL, V136, P266, DOI 10.1016/j.jcpa.2007.03.002
Mouchaty SK, 2000, MOL BIOL EVOL, V17, P60, DOI 10.1093/oxfordjournals.molbev.a026238
Murphy WJ, 2001, SCIENCE, V294, P2348, DOI 10.1126/science.1067179
Murphy WJ, 2001, NATURE, V409, P614, DOI 10.1038/35054550
Nikaido M, 2001, J MOL EVOL, V53, P508, DOI 10.1007/s002390010241
Nikaido M, 2000, J MOL EVOL, V51, P318, DOI 10.1007/s002390010094
Nishihara H, 2006, P NATL ACAD SCI USA, V103, P9929, DOI 10.1073/pnas.0603797103
NOTEBORN M, 1987, J INTERFERON RES, V7, P657, DOI 10.1089/jir.1987.7.657
Prasad AB, 2008, MOL BIOL EVOL, V25, P1795, DOI 10.1093/molbev/msn104
Richman AD, 2010, INT J IMMUNOGENET, V37, P401, DOI 10.1111/j.1744-313X.2010.00941.x
Sadler AJ, 2008, NAT REV IMMUNOL, V8, P559, DOI 10.1038/nri2314
SAITOU N, 1987, MOL BIOL EVOL, V4, P406, DOI 10.1093/oxfordjournals.molbev.a040454
Schad J, 2011, HEREDITY, V107, P115, DOI 10.1038/hdy.2010.173
Simmons NB, 2005, MAMMAL SPECIES WORLD, V1, P312, DOI [DOI 10.1111/J.1365-2656.2010.01742.X, 10.1093/acprof]
Six A, 1996, P NATL ACAD SCI USA, V93, P15329, DOI 10.1073/pnas.93.26.15329
Springer MS, 2004, TRENDS ECOL EVOL, V19, P430, DOI 10.1016/j.tree.2004.05.006
Storset AK, 2003, EUR J IMMUNOL, V33, P980, DOI 10.1002/eji.200323710
SULKIN SE, 1966, AM J TROP MED HYG, V15, P418, DOI 10.4269/ajtmh.1966.15.418
Swanepoel R, 1996, EMERG INFECT DIS, V2, P321, DOI 10.3201/eid0204.960407
Takahashi T, 2004, EUR J IMMUNOL, V34, P773, DOI 10.1002/eji.200324695
Tamura K, 2007, MOL BIOL EVOL, V24, P1596, DOI 10.1093/molbev/msm092
Teeling EC, 2005, SCIENCE, V307, P580, DOI 10.1126/science.1105113
Teeling EC, 2009, TIMETREE LIFE
Thompson JD, 2002, CURRENT PROTOCOLS BI
Tomasello E, 2000, SEMIN IMMUNOL, V12, P139, DOI 10.1006/smim.2000.0216
Tschopp J, 2010, NAT REV IMMUNOL, V10, P210, DOI 10.1038/nri2725
Vacic V, 2007, BMC BIOINFORMATICS, V8, DOI 10.1186/1471-2105-8-211
Vaidya SV, 2006, IMMUNOL LETT, V105, P180, DOI 10.1016/j.imlet.2006.02.006
Van Den Bussche RA, 2002, MOL PHYLOGENET EVOL, V22, P333, DOI

10.1006/mpev.2001.1051
Viertlboeck BC, 2009, J IMMUNOL, V182, P6985, DOI 10.4049/jimmunol.0803060
VIHINEN M, 1994, PROTEINS, V19, P141, DOI 10.1002/prot.340190207
Virtue ER, 2011, PLOS ONE, V6, DOI 10.1371/journal.pone.0022488
von der Malsburg A, 2011, J BIOL CHEM, V286, P37858, DOI 10.1074/jbc.M111.249037
Williamson MM, 1998, AUST VET J, V76, P813, DOI 10.1111/j.1751-0813.1998.tb12335.x
Williamson MM, 2000, J COMP PATHOL, V122, P201, DOI 10.1053/jcpa.1999.0364
Wong ESW, 2009, IMMUNOGENETICS, V61, P565, DOI 10.1007/s00251-009-0386-7
Wong S, 2007, REV MED VIROL, V17, P67, DOI 10.1002/rmv.520
WOOTTON JC, 1993, COMPUT CHEM, V17, P149, DOI 10.1016/0097-8485(93)85006-X
Yoder JA, 2011, IMMUNOGENETICS, V63, P123, DOI 10.1007/s00251-010-0506-4
Yoneyama M, 2005, J IMMUNOL, V175, P2851, DOI 10.4049/jimmunol.175.5.2851
Zerbino DR, 2008, GENOME RES, V18, P821, DOI 10.1101/gr.074492.107
Zhou P, 2011, PLOS ONE, V6, DOI 10.1371/journal.pone.0025385
Zhou P, 2011, J IMMUNOL, V186, P3138, DOI 10.4049/jimmunol.1003115
NR 83
TC 90
Z9 102
U1 1
U2 4
PU BMC
PI LONDON
PA CAMPUS, 4 CRINAN ST, LONDON N1 9XW, ENGLAND
SN 1471-2164
J9 BMC GENOMICS
JI BMC Genomics
PD JUN 20
PY 2012
VL 13
AR 261
DI 10.1186/1471-2164-13-261
PG 17
WC Biotechnology & Applied Microbiology; Genetics & Heredity
WE Science Citation Index Expanded (SCI-EXPANDED)
SC Biotechnology & Applied Microbiology; Genetics & Heredity
GA 001RD
UT WOS:000308476900001
PM 22716473
OA Green Published, Green Accepted, gold
DA 2024-10-21
ER

PT J
AU Shi, ZL

AF Shi, Zhengli
TI Bat and virus
SO PROTEIN & CELL
LA English
DT Article
AB Bat, the only flying mammal and count more than 20% of the extant mammals on earth, were recently identified as a natural reservoir of emerging and reemerging infectious pathogens. Astonishing amount (more than 70) and genetic diversity of viruses isolated from the bat have been identified in different populations throughout the world. Many studies focus on bat viruses that caused severe domestic and human diseases. However, many viruses were found in apparently healthy bats, suggesting that bats may have a specific immune system or antiviral activity against virus infections. Therefore, basic researches for bat immunology and virus-host interactions are important for understanding bat-derived infectious diseases.
C1 Chinese Acad Sci, Wuhan Inst Virol, State Key Lab Virol, Wuhan 430071, Peoples R China.
C3 Chinese Academy of Sciences; Wuhan Institute of Virology, CAS
RP Shi, ZL (corresponding author), Chinese Acad Sci, Wuhan Inst Virol, State Key Lab Virol, Wuhan 430071, Peoples R China.
EM zlshi@wh.iov.cn
RI Shi, Zheng-Li/A-1013-2013
OI Shi, Zheng-Li/0000-0001-8089-163X
CR Aguirre LF, 2003, FUNCT ECOL, V17, P201, DOI 10.1046/j.1365-2435.2003.00721.x
Almeida MF, 2005, EPIDEMIOL INFECT, V133, P523, DOI 10.1017/S0950268804003656
Badilla X, 2003, EMERG INFECT DIS, V9, P721
Badrane H, 2001, J VIROL, V75, P8096, DOI 10.1128/JVI.75.17.8096-8104.2001
Biek R, 2006, PLOS PATHOG, V2, P885, DOI 10.1371/journal.ppat.0020090
Blanton JD, 2008, JAVMA-J AM VET MED A, V233, P884, DOI 10.2460/javma.233.6.884
BOURHY H, 1993, VIROLOGY, V194, P70, DOI 10.1006/viro.1993.1236
Calisher CH, 2006, CLIN MICROBIOL REV, V19, P531, DOI 10.1128/CMR.00017-06
Carrington CVF, 2008, EMERG INFECT DIS, V14, P1890, DOI 10.3201/eid1412.080642
Chadha MS, 2006, EMERG INFECT DIS, V12, P235, DOI 10.3201/eid1202.051247
Chan-Yeung M, 2003, RESPIROLOGY, V8, pS9, DOI 10.1046/j.1440-1843.2003.00518.x
Chu DKW, 2008, J VIROL, V82, P9107, DOI 10.1128/JVI.00857-08
Chu DKW, 2006, J GEN VIROL, V87, P2461, DOI 10.1099/vir.0.82203-0
Chua KB, 2007, P NATL ACAD SCI USA, V104, P11424, DOI 10.1073/pnas.0701372104
Chua KB, 2000, SCIENCE, V288, P1432, DOI 10.1126/science.288.5470.1432
Chua KB, 2002, MICROBES INFECT, V4, P145, DOI 10.1016/S1286-4579(01)01522-2
Dominguez SR, 2007, EMERG INFECT DIS, V13, P1295, DOI 10.3201/eid1309.070491

Eaton BT, 2006, NAT REV MICROBIOL, V4, P23, DOI 10.1038/nrmicro1323
EATON BT, 2007, FIELDS VIROLOGY
EMOND RTD, 1977, BMJ-BRIT MED J, V2, P541, DOI 10.1136/bmj.2.6086.541
Favi M, 2002, EMERG INFECT DIS, V8, P79, DOI 10.3201/eid0801.010108
Field H., 2008, VIROLOGICA SINICA, V24, P105
Fooks AR, 2003, J MED VIROL, V71, P281, DOI 10.1002/jmv.10481
Gloza-Rausch F, 2008, EMERG INFECT DIS, V14, P626, DOI 10.3201/eid1404.071439
Gonzalez JP, 2007, CURR TOP MICROBIOL, V315, P363
Halpin K, 1999, VET MICROBIOL, V68, P83, DOI 10.1016/S0378-1135(99)00063-2
Hanna JN, 2000, MED J AUSTRALIA, V172, P597, DOI 10.5694/j.1326-5377.2000.tb124126.x
Hon CC, 2008, J VIROL, V82, P1819, DOI 10.1128/JVI.01926-07
Hughes GJ, 2006, ARCH VIROL, V151, P2021, DOI 10.1007/s00705-005-0785-0
Iehlé C, 2007, EMERG INFECT DIS, V13, P159, DOI 10.3201/eid1301.060791
Jones KE, 2008, NATURE, V451, P990, DOI 10.1038/nature06536
Lau SKP, 2005, P NATL ACAD SCI USA, V102, P14040, DOI 10.1073/pnas.0506735102
Lau SKP, 2007, VIROLOGY, V367, P428, DOI 10.1016/j.virol.2007.06.009
Leroy EM, 2005, NATURE, V438, P575, DOI 10.1038/438575a
Li WH, 2005, EMBO J, V24, P1634, DOI 10.1038/sj.emboj.7600640
Li Y, 2008, EMERG INFECT DIS, V14, P1974, DOI 10.3201/eid1412.080359
MALHERBE H, 1968, LANCET, V1, P1434
Middleton DJ, 2007, J COMP PATHOL, V136, P266, DOI 10.1016/j.jcpa.2007.03.002
Misra V, 2009, J GEN VIROL, V90, P2015, DOI 10.1099/vir.0.010694-0
Molnár V, 2008, ACTA VET HUNG, V56, P529, DOI 10.1556/AVet.56.2008.4.10
Müller MA, 2007, EMERG INFECT DIS, V13, P1367, DOI 10.3201/eid1309.070342
MURRAY K, 1995, SCIENCE, V268, P94, DOI 10.1126/science.7701348
Nathwani D, 2003, CLIN INFECT DIS, V37, P598, DOI 10.1086/376641
Paton NI, 1999, LANCET, V354, P1253, DOI 10.1016/S0140-6736(99)04379-2
Patterson BD, 2003, BAT ECOLOGY, P536
Paweska JT, 2006, EMERG INFECT DIS, V12, P1965
Pfefferle S, 2009, EMERG INFECT DIS, V15, P1377, DOI 10.3201/eid1509.090224
Poon LLM, 2005, J VIROL, V79, P2001, DOI 10.1128/JVI.79.4.2001-2009.2005
Pourrut X, 2007, J INFECT DIS, V196, pS176, DOI 10.1086/520541
Pourrut X, 2009, BMC INFECT DIS, V9, DOI 10.1186/1471-2334-9-159
Razafindratsimandresy R, 2009, J GEN VIROL, V90, P44, DOI 10.1099/vir.0.006825-0
Ren W, 2008, J VIROL, V82, P1899, DOI 10.1128/JVI.01085-07

Ren W, 2006, J GEN VIROL, V87, P3355, DOI 10.1099/vir.0.82220-0
Reynes JM, 2005, EMERG INFECT DIS, V11, P1042, DOI 10.3201/eid1107.041350
Sanchez A., 2007, FIELDS VIROLOGY, P1409
Sendow I, 2006, EMERG INFECT DIS, V12, P711, DOI 10.3201/eid1204.051181
Simmons NB, 2005, MAMMAL SPECIES WORLD, V1, P312, DOI [DOI 10.1111/J.1365-2656.2010.01742.X, 10.1093/acprof]
Sonntag M, 2009, EMERG INFECT DIS, V15, P2052, DOI 10.3201/eid1512.090646
Sulkin S., 1974, Monographs in Virology, V1
Swanepoel R, 2007, EMERG INFECT DIS, V13, P1847, DOI 10.3201/eid1312.071115
Tandler B, 1996, J COMP PATHOL, V114, P1, DOI 10.1016/S0021-9975(96)80057-0
Tang XC, 2006, J VIROL, V80, P7481, DOI 10.1128/JVI.00697-06
Teeling EC, 2005, SCIENCE, V307, P580, DOI 10.1126/science.1105113
Tjornehoj K, 2006, J COMP PATHOL, V134, P190, DOI 10.1016/j.jcpa.2005.10.005
Towner JS, 2007, PLOS ONE, V2, DOI 10.1371/journal.pone.0000764
Towner JS, 2009, PLOS PATHOG, V5, DOI 10.1371/journal.ppat.1000536
Van Thiel P.P., 2008, EURO SURVEILL, V13
Wacharapluesadee S, 2005, EMERG INFECT DIS, V11, P1949, DOI 10.3201/eid1112.050613
Watanabe S, 2009, VIRUS GENES, V39, P90, DOI 10.1007/s11262-009-0368-8
Wibbelt G, 2007, J GEN VIROL, V88, P2651, DOI 10.1099/vir.0.83045-0
Woo PCY, 2007, J VIROL, V81, P1574, DOI 10.1128/JVI.02182-06
Zhu HC, 2009, J GEN VIROL, V90, P883, DOI 10.1099/vir.0.007732-0
NR 72
TC 27
Z9 30
U1 0
U2 24
PU HIGHER EDUCATION PRESS
PI BEIJING
PA NO 4 DEWAI DAJIE, BEIJING 100120, PEOPLES R CHINA
SN 1674-800X
EI 1674-8018
J9 PROTEIN CELL
JI Protein Cell
PD FEB
PY 2010
VL 1
IS 2
BP 109
EP 114
DI 10.1007/s13238-010-0029-7
PG 6
WC Cell Biology
WE Science Citation Index Expanded (SCI-EXPANDED)

SC Cell Biology
GA V25XJ
UT WOS:000208510400002
PM 21203979
OA Green Published, Bronze
DA 2024-10-21
ER

PT J
AU Liang, YZ
   Wu, LJ
   Zhang, Q
   Zhou, P
   Wang, MN
   Yang, XL
   Ge, XY
   Wang, LF
   Shi, ZL
AF Liang, Ying-Zi
   Wu, Li-Jun
   Zhang, Qian
   Zhou, Peng
   Wang, Mei-Niang
   Yang, Xing-Lou
   Ge, Xing-Yi
   Wang, Lin-Fa
   Shi, Zheng-Li
TI Cloning, expression, and antiviral activity of interferon β from the
   Chinese microbat, <i>Myotis davidii</i>
SO VIROLOGICA SINICA
LA English
DT Article
DE bat; interferon; IFN-stimulated genes; antiviral activity
ID BLACK FLYING FOX; EMERGING INFECTIOUS-DISEASES; BATS
   PTEROPUS-POLIOCEPHALUS; HENDRA VIRUS-INFECTION; MOLECULAR
   CHARACTERIZATION; NIPAH VIRUS; FRUIT BATS; RIG-I; ALECTO;
REPERTOIRE
AB Bats are natural reservoir hosts for many viruses that produce no clinical symptoms in bats. Therefore, bats may have evolved effective mechanisms to control viral replication. However, little information is available on bat immune responses to viral infection. Type I interferon (IFN) plays a key role in controlling viral infections. In this study, we report the cloning, expression, and biological activity of interferon beta (IFN beta) from the Chinese microbat species, Myotis davidii. We demonstrated the upregulation of IFNB and IFN-stimulated genes in a kidney cell line derived from M. davidii after treatment with polyI:C or infection with Sendai virus. Furthermore, the recombinant IFN beta inhibited vesicular stomatitis virus and bat adenovirus replication in cell lines from two bat species, M. davidii and Rhinolophus sinicus. We provide the first in vitro evidence of IFN beta antiviral activity in microbats, which has important implications for virus interactions with these hosts.

C1 [Liang, Ying-Zi; Wu, Li-Jun; Zhang, Qian; Wang, Mei-Niang; Yang, Xing-Lou; Ge, Xing-Yi; Shi, Zheng-Li] Chinese Acad Sci, Wuhan Inst Virol, Key Lab Special Pathogens & Biosafety, Wuhan 430071, Peoples R China.
[Zhou, Peng; Wang, Lin-Fa] Duke Natl Univ Singapore, Grad Sch Med, Program Emerging Infect Dis, Singapore 169857, Singapore.
C3 Chinese Academy of Sciences; Wuhan Institute of Virology, CAS; National
University of Singapore
RP Shi, ZL (corresponding author), Chinese Acad Sci, Wuhan Inst Virol, Key Lab Special Pathogens & Biosafety, Wuhan 430071, Peoples R China.
EM zlshi@wh.iov.cn
RI Yang, Xinglou/AAW-6969-2020; Shi, Zheng-Li/A-1013-2013
OI Shi, Zheng-Li/0000-0001-8089-163X; Yang, Xing-Lou/ 0000-0002-5317-8983;
Wang, Lin-Fa/0000-0003-2752-0535
FU National Natural Science Foundation of China [31321001]
FX This work was funded by the National Natural Science Foundation of China
(No. 31321001).
CR Arnold K, 2006, BIOINFORMATICS, V22, P195, DOI 10.1093/ bioinformatics/bti770
Baker ML, 2013, ZOONOSES PUBLIC HLTH, V60, P104, DOI 10.1111/ j.1863-2378.2012.01528.x
Baker ML, 2010, IMMUNOGENETICS, V62, P173, DOI 10.1007/ s00251-010-0425-4
Biesold SE, 2011, PLOS ONE, V6, DOI 10.1371/journal.pone.0028131
Brook CE, 2015, TRENDS MICROBIOL, V23, P172, DOI 10.1016/ j.tim.2014.12.004
Chua KB, 2002, MICROBES INFECT, V4, P145, DOI 10.1016/ S1286-4579(01)01522-2
Cowled C, 2012, DEV COMP IMMUNOL, V36, P657, DOI 10.1016/ j.dci.2011.11.008
Cowled C, 2011, DEV COMP IMMUNOL, V35, P7, DOI 10.1016/ j.dci.2010.07.006
Crameri G, 2009, PLOS ONE, V4, DOI 10.1371/journal.pone.0008266
Fernandez M, 2002, J VIROL, V76, P895, DOI 10.1128/ JVI.76.2.895-904.2002
Ge XY, 2013, NATURE, V503, P535, DOI 10.1038/nature12711
Halpin K, 2000, J GEN VIROL, V81, P1927, DOI 10.1099/0022-1317-81-8-1927
Hayman DTS, 2013, ZOONOSES PUBLIC HLTH, V60, P2, DOI 10.1111/ zph.12000
He X, 2014, J VIROL, V88, P5444, DOI 10.1128/JVI.03403-13
Janardhana V, 2012, DEV COMP IMMUNOL, V36, P610, DOI 10.1016/ j.dci.2011.11.001
Jones KE, 2008, NATURE, V451, P990, DOI 10.1038/nature06536
Kepler TB, 2010, BMC GENOMICS, V11, DOI 10.1186/1471-2164-11-444
Lau SKP, 2005, P NATL ACAD SCI USA, V102, P14040, DOI 10.1073/ pnas.0506735102
Li JJ, 2015, GENE, V561, P115, DOI 10.1016/j.gene.2015.02.013
Li WD, 2005, SCIENCE, V310, P676, DOI 10.1126/science.1118391
Li Y, 2010, J VIROL, V84, P3889, DOI 10.1128/JVI.02497-09

Marchler-Bauer A, 2011, NUCLEIC ACIDS RES, V39, pD225, DOI 10.1093/nar/gkq1189
Marsh GA, 2012, CURR OPIN VIROL, V2, P242, DOI 10.1016/j.coviro.2012.03.006
Middleton DJ, 2007, J COMP PATHOL, V136, P266, DOI 10.1016/j.jcpa.2007.03.002
O'Shea TJ, 2014, EMERG INFECT DIS, V20, P741, DOI 10.3201/eid2005.130539
Omatsu T, 2008, VET IMMUNOL IMMUNOP, V124, P169, DOI 10.1016/j.vetimm.2008.03.004
Papenfuss AT, 2012, BMC GENOMICS, V13, DOI 10.1186/1471-2164-13-261
Plowright RK, 2008, P ROY SOC B-BIOL SCI, V275, P861, DOI 10.1098/rspb.2007.1260
Pourrut X, 2009, BMC INFECT DIS, V9, DOI 10.1186/1471-2334-9-159
Randall RE, 2008, J GEN VIROL, V89, P1, DOI 10.1099/vir.0.83391-0
Shi ZL, 2013, SCI CHINA LIFE SCI, V56, P678, DOI 10.1007/s11427-013-4517-x
THOMPSON JD, 1994, NUCLEIC ACIDS RES, V22, P4673, DOI 10.1093/nar/22.22.4673
Turmelle AS, 2010, J GEN VIROL, V91, P2360, DOI 10.1099/vir.0.020073-0
Virtue ER, 2011, PLOS ONE, V6, DOI 10.1371/journal.pone.0022488
Wang LF, 2011, CURR OPIN VIROL, V1, P649, DOI 10.1016/j.coviro.2011.10.013
Williamson MM, 1998, AUST VET J, V76, P813, DOI 10.1111/j.1751-0813.1998.tb12335.x
Williamson MM, 2000, J COMP PATHOL, V122, P201, DOI 10.1053/jcpa.1999.0364
Zhang GJ, 2013, SCIENCE, V339, P456, DOI 10.1126/science.1230835
Zhou P, 2011, PLOS ONE, V6, DOI 10.1371/journal.pone.0025385
Zhou P, 2011, J IMMUNOL, V186, P3138, DOI 10.4049/jimmunol.1003115
NR 40
TC 7
Z9 8
U1 0
U2 22
PU SPRINGER
PI NEW YORK
PA 233 SPRING ST, NEW YORK, NY 10013 USA
SN 1674-0769
EI 1995-820X
J9 VIROL SIN
JI Virol. Sin.
PD DEC
PY 2015
VL 30
IS 6
BP 425
EP 432
DI 10.1007/s12250-015-3668-2
PG 8
WC Virology

WE Science Citation Index Expanded (SCI-EXPANDED)
SC Virology
GA DA0XE
UT WOS:000367520100004
PM 26645237
OA Bronze, Green Published
DA 2024-10-21
ER

PT J
AU Tarigan, R
   Katta, T
   Takemae, H
   Shimoda, H
   Maeda, K
   Iida, A
   Hondo, E
AF Tarigan, Ronald
   Katta, Tetsufumi
   Takemae, Hitoshi
   Shimoda, Hiroshi
   Maeda, Ken
   Iida, Atsuo
   Hondo, Eiichi
TI Distinct interferon response in bat and other mammalian cell lines
   infected with <i>Pteropine orthoreovirus</i>
SO VIRUS GENES
LA English
DT Article
DE Bat; Pteropine orthoreovirus; Pattern recognition receptor; Interferon
ID FRUIT BATS; SEROLOGICAL EVIDENCE; GENE-EXPRESSION; NIPAH VIRUS; RIG-I;
   RECOGNITION; TRANSMISSION; RESERVOIRS; RECEPTORS; INDUCTION
AB Bats serve as natural hosts of Pteropine orthoreovirus (PRV), an emerging group of bat-borne, zoonotic viruses. Bats appear to possess unique innate immune system responses that can inhibit viral replication, thus reducing clinical symptoms. We examined the innate immune response against PRV and assessed viral replication in cell lines derived from four bat species (Miniopterus fuliginosus, Pteropus dasymallus, Rhinolophus ferrumequinum, and Rousettus leschenaultii), one rodent (Mesocricetous auratus), and human (Homo sapiens). The expression levels of pattern recognition receptors (PRRs) (TLR3, RIG-I, and MDA5) and interferons (IFNB1 and IFNL1) were higher and PRV replication was lower in cell lines derived from M. fuliginosus, R. ferrumequinum, and R. leschenaultii. Reduction of IFNB1 expression by the knockdown of PRRs in the cell line derived from R. ferrumequinum was associated with increased PRV replication. The knockdown of RIG-I led to the most significant reduction in viral replication for all cell lines. These results suggest that RIG-I production is important for antiviral response against PRV in R. ferrumequinum.
C1 [Tarigan, Ronald; Katta, Tetsufumi; Takemae, Hitoshi; Iida,

Atsuo; Hondo, Eiichi] Nagoya Univ, Grad Sch Bioagr Sci, Dept Anim Sci, Lab Anim Morphol,Chikusa Ku, Furo Cho, Nagoya, Aichi 4648601, Japan.
[Shimoda, Hiroshi] Yamaguchi Univ, Joint Fac Vet Med, Lab Vet Microbiol, Yamaguchi, Japan.
[Maeda, Ken] Natl Inst Infect Dis, Div Vet Sci, Tokyo, Japan.
C3 Nagoya University; Yamaguchi University; National Institute of Infectious Diseases (NIID)
RP Hondo, E (corresponding author), Nagoya Univ, Grad Sch Bioagr Sci, Dept Anim Sci, Lab Anim Morphol,Chikusa Ku, Furo Cho, Nagoya, Aichi 4648601, Japan.
EM ehondo@agr.nagoya-u.ac.jp
RI Takemae, Hitoshi/AEP-5503-2022; Tarigan, Ronald/GPW-7141-2022
OI Maeda, Ken/0000-0002-3488-5439; IIDA, Atsuo/0000-0002-0138-5366
FU Grants-in-Aid for Scientific Research [21H04637] Funding Source: KAKEN
CR Ahn M, 2019, NAT MICROBIOL, V4, P789, DOI 10.1038/s41564-019-0371-3
Banerjee A, 2020, ISCIENCE, V23, DOI 10.1016/j.isci.2020.100958
Banerjee A, 2019, VIRUSES-BASEL, V11, DOI 10.3390/v11020152
Banerjee A, 2017, SCI REP-UK, V7, DOI 10.1038/s41598-017-01513-w
Cheng P, 2009, J CLIN VIROL, V45, P79, DOI 10.1016/j.jcv.2009.03.001
Chua KB, 2008, PLOS ONE, V3, DOI 10.1371/journal.pone.0003803
Chua KB, 2002, MICROBES INFECT, V4, P145, DOI 10.1016/S1286-4579(01)01522-2
Ciechonska M, 2014, TRENDS MICROBIOL, V22, P715, DOI 10.1016/j.tim.2014.08.005
De La Cruz-Rivera PC, 2018, J IMMUNOL, V200, P209, DOI 10.4049/jimmunol.1701214
Egawa K, 2017, PLOS NEGLECT TROP D, V11, DOI 10.1371/journal.pntd.0006076
FARONE AL, 1993, J LEUKOCYTE BIOL, V53, P133, DOI 10.1002/jlb.53.2.133
Farone AL, 2006, VIRAL IMMUNOL, V19, P546, DOI 10.1089/vim.2006.19.546
GARD G, 1970, J VIROL, V6, P100, DOI 10.1128/JVI.6.1.100-106.1970
Halpin K, 2000, J GEN VIROL, V81, P1927, DOI 10.1099/0022-1317-81-8-1927
Hemann EA, 2017, FRONT IMMUNOL, V8, DOI 10.3389/fimmu.2017.01707
Höbel S, 2010, METHODS MOL BIOL, V623, P283, DOI 10.1007/978-1-60761-588-0_18
Hu TS, 2014, BMC MICROBIOL, V14, DOI 10.1186/s12866-014-0293-4
Irving AT, 2020, CELL REP, V33, DOI 10.1016/j.celrep.2020.108345
Irving AT, 2020, CELL MOL LIFE SCI, V77, P1607, DOI 10.1007/s00018-019-03242-x
Jiang RD, 2020, VIROLOGY, V551, P84, DOI 10.1016/j.virol.2020.05.014
Kanai Y, 2018, VIROLOGY, V514, P57, DOI 10.1016/j.virol.2017.10.021
Kato H, 2006, NATURE, V441, P101, DOI 10.1038/nature04734
Kato H, 2008, J EXP MED, V205, P1601, DOI 10.1084/jem.20080091
Katoh H, 2016, EMERG INFECT DIS, V22, P703, DOI 10.3201/eid2204.151116

Kawagishi T, 2016, PLOS PATHOG, V12, DOI 10.1371/journal.ppat.1005455
Kuzmin IV, 2017, J VIROL, V91, DOI [10.1128/JVI.02471-16, 10.1128/jvi.02471-16]
Leroy EM, 2005, NATURE, V438, P575, DOI 10.1038/438575a
Li JJ, 2015, GENE, V561, P115, DOI 10.1016/j.gene.2015.02.013
Li WD, 2005, SCIENCE, V310, P676, DOI 10.1126/science.1118391
Liu WJ, 2006, J VIROL, V80, P2396, DOI 10.1128/JVI.80.5.2396-2404.2006
Maeda K, 2008, EMERG INFECT DIS, V14, P347, DOI 10.3201/eid1402.070932
Maruyama J, 2016, VIROLOGY, V488, P43, DOI 10.1016/j.virol.2015.11.002
Maruyama J, 2014, J VIROL, V88, P99, DOI 10.1128/JVI.02265-13
McFarlane R, 2011, PLOS ONE, V6, DOI 10.1371/journal.pone.0028374
Memish ZA, 2013, EMERG INFECT DIS, V19, P1819, DOI 10.3201/eid1911.131172
Meylan E, 2006, MOL CELL, V22, P561, DOI 10.1016/j.molcel.2006.05.012
Middleton DJ, 2007, J COMP PATHOL, V136, P266, DOI 10.1016/j.jcpa.2007.03.002
Mok L, 2017, BMC GENOMICS, V18, DOI 10.1186/s12864-017-3994-x
Mok L, 2015, J GEN VIROL, V96, P1787, DOI 10.1099/vir.0.000112
Munster VJ, 2016, SCI REP-UK, V6, DOI 10.1038/srep21878
Nikolay B, 2019, NEW ENGL J MED, V380, P1804, DOI 10.1056/NEJMoa1805376
Paweska JT, 2016, VIRUSES-BASEL, V8, DOI 10.3390/v8020029
Pritchard LI, 2006, ARCH VIROL, V151, P229, DOI 10.1007/s00705-005-0644-4
Sarkis S, 2018, DEV COMP IMMUNOL, V81, P1, DOI 10.1016/j.dci.2017.10.023
Schmittgen TD, 2008, NAT PROTOC, V3, P1101, DOI 10.1038/nprot.2008.73
Sherry B, 2009, J INTERF CYTOK RES, V29, P559, DOI 10.1089/jir.2009.0072
Singh H, 2015, J MED VIROL, V87, P2145, DOI 10.1002/jmv.24274
Takadate Y, 2020, MICROORGANISMS, V8, DOI 10.3390/microorganisms8101530
Takadate Y, 2020, CELL REP, V30, P308, DOI 10.1016/j.celrep.2019.12.042
Takemae H, 2018, VIRUS GENES, V54, P823, DOI 10.1007/s11262-018-1603-y
Tan CW, 2019, EMERG MICROBES INFEC, V8, P787, DOI 10.1080/22221751.2019.1621668
Taniguchi S, 2017, ARCH VIROL, V162, P1529, DOI 10.1007/s00705-017-3251-2
Tarigan R, 2020, BIOCHEM BIOPH RES CO, V527, P1, DOI 10.1016/j.bbrc.2020.04.060
Towner JS, 2009, PLOS PATHOG, V5, DOI 10.1371/journal.ppat.1000536
Uehara A, 2019, J MED VIROL, V91, P707, DOI 10.1002/jmv.25355
Voon K, 2015, J MED VIROL, V87, P2149, DOI 10.1002/jmv.24304
Yamanaka A, 2014, PLOS ONE, V9, DOI 10.1371/journal.pone.0092777
Zhou P, 2013, DEV COMP IMMUNOL, V40, P240, DOI 10.1016/

j.dci.2013.03.006
   Zhou P, 2011, J IMMUNOL, V186, P3138, DOI 10.4049/
jimmunol.1003115
NR 59
TC 10
Z9 10
U1 0
U2 7
PU SPRINGER
PI DORDRECHT
PA VAN GODEWIJCKSTRAAT 30, 3311 GZ DORDRECHT, NETHERLANDS
SN 0920-8569
EI 1572-994X
J9 VIRUS GENES
JI Virus Genes
PD DEC
PY 2021
VL 57
IS 6
BP 510
EP 520
DI 10.1007/s11262-021-01865-6
EA AUG 2021
PG 11
WC Genetics & Heredity; Virology
WE Science Citation Index Expanded (SCI-EXPANDED)
SC Genetics & Heredity; Virology
GA WY6CW
UT WOS:000688369100001
PM 34432209
OA Green Published, Bronze
DA 2024-10-21
ER

PT S
AU Skirmuntt, EC
   Escalera-Zamudio, M
   Teeling, EC
   Smith, A
   Katzourakis, A
AF Skirmuntt, Emilia C.
   Escalera-Zamudio, Marina
   Teeling, Emma C.
   Smith, Adrian
   Katzourakis, Aris
BE Enquist, L
TI The Potential Role of Endogenous Viral Elements in the Evolution
of Bats
   as Reservoirs for Zoonotic Viruses
SO ANNUAL REVIEW OF VIROLOGY, VOL 7, 2020
SE Annual Review of Virology
LA English
DT Article; Book Chapter
DE bats; viruses; immunity; evolution; ERVs; EVEs

ID BORNA-DISEASE VIRUS; TOLL-LIKE RECEPTORS; IMMUNE-RESPONSES;
INTERFERON-ALPHA; HOST; INFECTION; DISCOVERY; RETROVIRUSES;
PARVOVIRUSES; EXPRESSION

AB Despite a small genome size, bats have comparable diversity of retroviral and non-retroviral endogenous sequences to other mammals. These include Class I and Class II retroviral sequences, foamy viruses, and deltaretro-viruses, as well as filovirus, bornavirus, and parvovirus endogenous viral elements. Some of these endogenous viruses are sufficiently preserved in bat genomes to be expressed, with potential effects for host biology. It is clear that the bat immune system differs when compared with other mammals, yet the role that virus-derived endogenous elements may have played in the evolution of bat immunity is poorly understood. In this review, we discuss some of the bat-specific immune mechanisms that may have resulted in a virus-tolerant phenotype and link these to the long-standing virus-host coevolution that may have allowed a large diversity of endogenous retroviruses and other endogenous viral elements to colonize bat genomes. We also consider the possible effects of endogenization in the evolution of the bat immune system.

C1 [Skirmuntt, Emilia C.; Escalera-Zamudio, Marina; Smith, Adrian; Katzourakis, Aris] Univ Oxford, Dept Zool, Oxford OX1 3PS, England.
[Teeling, Emma C.] Univ Coll Dublin, Sch Biol & Environm Sci, Dublin 4, Ireland.
C3 University of Oxford; University College Dublin
RP Katzourakis, A (corresponding author), Univ Oxford, Dept Zool, Oxford OX1 3PS, England.
EM aris.katzourakis@zoo.ox.ac.uk
RI Skirmuntt, Emilia/HKV-2122-2023; Katzourakis, Aris/K-4488-2012
OI Escalera-Zamudio, Marina/0000-0002-4773-2773; Skirmuntt, Emilia/0000-0002-7302-0665; Smith, Adrian/0000-0002-7657-6191; Teeling, Emma/0000-0002-3309-1346

FU Irish Research Council Consolidator Laureate Award; Leverhulme Trust ECR Fellowship [ECF-2019-542]
FX E.C.T. was funded by an Irish Research Council Consolidator Laureate Award. M.E.-Z. is currently supported by a Leverhulme Trust ECR Fellowship (ECF-2019-542).

CR Agnarsson I, 2011, PLOS CURR-TREE LIFE, DOI 10.1371/currents.RRN1212
Ahn M, 2016, SCI REP-UK, V6, DOI 10.1038/srep21722
Aiewsakun P, 2015, VIROLOGY, V479, P26, DOI 10.1016/j.virol.2015.02.011
Arnaud F, 2007, PLOS PATHOG, V3, P1716, DOI 10.1371/journal.ppat.0030170
Arriagada G, 2014, J VIROL, V88, P12158, DOI 10.1128/JVI.01173-14
Aswad A, 2012, TRENDS ECOL EVOL, V27, P627, DOI 10.1016/j.tree.2012.07.007
Baker ML, 2013, ZOONOSES PUBLIC HLTH, V60, P104, DOI 10.1111/j.1863-2378.2012.01528.x
Banerjee A, 2020, FRONT IMMUNOL, V11, DOI 10.3389/fimmu.2020.00026
Banyard AC, 2014, VIRUSES-BASEL, V6, P2974, DOI 10.3390/v6082974

Bat1K, 2019, BAT1K CONS
Becker DJ, 2019, INTEGR COMP BIOL, V59, P1176, DOI 10.1093/icb/icz007
Belyi VA, 2010, PLOS PATHOG, V6, DOI 10.1371/journal.ppat.1001030
Bill CA, 2004, P NATL ACAD SCI USA, V101, P11135, DOI 10.1073/pnas.0403925101
Boehme KW, 2004, J VIROL, V78, P7867, DOI 10.1128/JVI.78.15.7867-7873.2004
Bratsch S, 2011, DEV COMP IMMUNOL, V35, P421, DOI 10.1016/j.dci.2010.06.004
Broecker F, 2019, FRONT MICROBIOL, V10, DOI 10.3389/fmicb.2019.00051
Brook CE, 2020, ELIFE, V9, DOI 10.7554/eLife.48401
Brook CE, 2015, TRENDS MICROBIOL, V23, P172, DOI 10.1016/j.tim.2014.12.004
Calisher CH, 2006, CLIN MICROBIOL REV, V19, P531, DOI 10.1128/CMR.00017-06
Cárdenas WB, 2006, J VIROL, V80, P5168, DOI 10.1128/JVI.02199-05
Chen AY, 2010, FUTURE VIROL, V5, P731, DOI 10.2217/FVL.10.56
Chen JG, 2019, ONCOGENESIS, V8, DOI 10.1038/s41389-018-0114-y
Chuong EB, 2018, PLOS BIOL, V16, DOI 10.1371/journal.pbio.3000028
Chuong EB, 2016, SCIENCE, V351, P1083, DOI 10.1126/science.aad5497
Cotmore SF, 2014, ANNU REV VIROL, V1, P517, DOI 10.1146/annurev-virology-031413-085444
Cowled C, 2011, DEV COMP IMMUNOL, V35, P7, DOI 10.1016/j.dci.2010.07.006
Cui J, 2015, VIRUSES-BASEL, V7, P5792, DOI 10.3390/v7112906
Cui J, 2015, SCI REP-UK, V5, DOI 10.1038/srep16561
Cui J, 2012, J GEN VIROL, V93, P2037, DOI 10.1099/vir.0.043760-0
Cui J, 2012, J VIROL, V86, P4288, DOI 10.1128/JVI.06624-11
Drexler JF, 2012, NAT COMMUN, V3, DOI 10.1038/ncomms1796
Eisenstein M, 2018, LAB ANIMAL, V47, P97, DOI 10.1038/s41684-018-0029-4
Engel ME, 2010, NAT MED, V16, P517, DOI 10.1038/nm0510-517
Escalera-Zamudio M, 2016, MBIO, V7, DOI [10.1128/mbio.01425-16, 10.1128/mBio.01425-16]
Escalera-Zamudio M, 2015, MOL ECOL, V24, P5899, DOI 10.1111/mec.13431
Escalera-Zamudio M, 2015, J VIROL, V89, P5180, DOI 10.1128/JVI.03452-14
Fabozzi G, 2011, J VIROL, V85, P2512, DOI 10.1128/JVI.01160-10
Farkasová H, 2017, P NATL ACAD SCI USA, V114, P3145, DOI 10.1073/pnas.1621224114
Feschotte C, 2012, NAT REV GENET, V13, P283, DOI 10.1038/nrg3199
Fujino K, 2014, P NATL ACAD SCI USA, V111, P13175, DOI 10.1073/pnas.1407046111
Geib T, 2003, J VIROL, V77, P4283, DOI 10.1128/JVI.77.7.4283-4290.2003
Gifford R, 2005, J VIROL, V79, P6478, DOI 10.1128/JVI.79.10.6478-6486.2005
Gilbert C, 2010, PLOS BIOL, V8, DOI 10.1371/journal.pbio.1000495
Han GZ, 2015, MOL BIOL EVOL, V32, P211, DOI 10.1093/molbev/msu297
Harris RS, 2004, NAT REV IMMUNOL, V4, P868, DOI 10.1038/nri1489

Hayman DTS, 2013, ZOONOSES PUBLIC HLTH, V60, P2, DOI 10.1111/zph.12000
Hayman DTS, 2019, NAT MICROBIOL, V4, P728, DOI 10.1038/s41564-019-0430-9
Hayward A, 2015, P NATL ACAD SCI USA, V112, P464, DOI 10.1073/pnas.1414980112
Hayward JA, 2018, MOL BIOL EVOL, V35, P1626, DOI 10.1093/molbev/msy048
Hayward JA, 2013, RETROVIROLOGY, V10, DOI 10.1186/1742-4690-10-35
HERZOG S, 1991, J GEN VIROL, V72, P535, DOI 10.1099/0022-1317-72-3-535
Honda T, 2016, MOBILE GENET ELEM, V6, P1548
Horie M, 2016, SCI REP-UK, V6, DOI 10.1038/srep25873
Horie M, 2010, NATURE, V463, P84, DOI 10.1038/nature08695
Hron T, 2018, VIRUSES-BASEL, V10, DOI 10.3390/v10040185
Huang ZX, 2019, NAT ECOL EVOL, V3, P1110, DOI 10.1038/s41559-019-0913-3
Iha K, 2010, J VET MED SCI, V72, P217, DOI 10.1292/jvms.09-0050
Jebb D, 2020, BIORXIV, DOI [10.1101/836874, DOI 10.1101/836874]
Johara MY, 2001, EMERG INFECT DIS, V7, P439
Johnson WE, 2015, ANNU REV VIROL, V2, P135, DOI 10.1146/annurev-virology-100114-054945
Jones KE, 2005, EVOLUTION, V59, P2243, DOI 10.1111/j.0014-3820.2005.tb00932.x
Kapoor A, 2010, J VIROL, V84, P12628, DOI 10.1128/JVI.01732-10
Kassiotis G, 2016, NAT REV IMMUNOL, V16, P207, DOI 10.1038/nri.2016.27
Katzourakis A, 2007, P NATL ACAD SCI USA, V104, P6261, DOI 10.1073/pnas.0700471104
Katzourakis A, 2013, PHILOS T R SOC B, V368, DOI 10.1098/rstb.2012.0493
Katzourakis A, 2010, PLOS GENET, V6, DOI 10.1371/journal.pgen.1001191
Kinnunen PM, 2013, J GEN VIROL, V94, P247, DOI 10.1099/vir.0.046961-0
Kondoh T, 2017, PLOS ONE, V12, DOI 10.1371/journal.pone.0186450
Kuhn J.H., 2018, HARRISONS PRINCIPLES, V2, P1509
Kunz TH, 2011, ANN NY ACAD SCI, V1223, P1, DOI 10.1111/j.1749-6632.2011.06004.x
Kuzmin IV, 2017, J VIROL, V91, DOI [10.1128/JVI.02471-16, 10.1128/jvi.02471-16]
Lahouassa H, 2012, NAT IMMUNOL, V13, P223, DOI 10.1038/ni.2236
Lau SKP, 2017, J GEN VIROL, V98, P3046, DOI 10.1099/jgv.0.000969
Lauw FN, 2005, TRENDS IMMUNOL, V26, P509, DOI 10.1016/j.it.2005.08.006
Leroy EM, 2005, NATURE, V438, P575, DOI 10.1038/438575a
Leung DW, 2010, NAT STRUCT MOL BIOL, V17, P165, DOI 10.1038/nsmb.1765
Liu HQ, 2011, J VIROL, V85, P9863, DOI 10.1128/JVI.00828-11
Ludwig H, 2000, REV SCI TECH OIE, V19, P259, DOI 10.20506/rst.19.1.1217
Luis AD, 2013, P ROY SOC B-BIOL SCI, V280, DOI 10.1098/rspb.2012.2753
Maillard PV, 2013, SCIENCE, V342, P235, DOI 10.1126/

science.1241930
Mandl JN, 2018, FRONT IMMUNOL, V9, DOI 10.3389/fimmu.2018.02112
Meredith RW, 2011, SCIENCE, V334, P521, DOI 10.1126/science.1211028
Mi S, 2000, NATURE, V403, P785, DOI 10.1038/35001608
Mühldorfer K, 2011, PLOS ONE, V6, DOI 10.1371/journal.pone.0029773
Negredo A, 2011, PLOS PATHOG, V7, DOI 10.1371/journal.ppat.1002304
Ng CT, 2015, CELL HOST MICROBE, V17, P653, DOI 10.1016/j.chom.2015.04.005
Norley S, 2010, RETROVIRUSES: MOLECULAR BIOLOGY, GENOMICS AND PATHOGENESIS, P309
Olival KJ, 2017, NATURE, V546, P646, DOI 10.1038/nature22975
Omatsu T, 2008, VET IMMUNOL IMMUNOP, V124, P169, DOI 10.1016/j.vetimm.2008.03.004
Ophinni Y, 2019, TRENDS IMMUNOL, V40, P998, DOI 10.1016/j.it.2019.09.003
Ozato K, 2008, NAT REV IMMUNOL, V8, P849, DOI 10.1038/nri2413
Parrish NF, 2015, RNA, V21, P1691, DOI 10.1261/rna.052092.115
Pénzes JJ, 2018, VIRUS EVOL, V4, DOI 10.1093/ve/vey026
Philbin VJ, 2005, IMMUNOLOGY, V114, P507, DOI 10.1111/j.1365-2567.2005.02125.x
Picard-Meyer E, 2017, BMC VET RES, V13, DOI 10.1186/s12917-017-1303-1
Pourrut X, 2009, BMC INFECT DIS, V9, DOI 10.1186/1471-2334-9-159
Pritham EJ, 2007, P NATL ACAD SCI USA, V104, P1895, DOI 10.1073/pnas.0609601104
Ray DA, 2007, MOL BIOL EVOL, V24, P632, DOI 10.1093/molbev/msl192
SARKAR SK, 1991, DEV COMP IMMUNOL, V15, P423, DOI 10.1016/0145-305X(91)90034-V
Schountz T, 2017, FRONT IMMUNOL, V8, DOI 10.3389/fimmu.2017.01098
Shackelton LA, 2005, P NATL ACAD SCI USA, V102, P379, DOI 10.1073/pnas.0406765102
Skirmuntt EC, 2019, VIRUS RES, V270, DOI 10.1016/j.virusres.2019.197645
Subudhi S, 2019, VIRUSES-BASEL, V11, DOI 10.3390/v11020192
Tarlinton RE, 2006, NATURE, V442, P79, DOI 10.1038/nature04841
Tassetto M, 2019, ELIFE, V8, DOI 10.7554/eLife.41244
Taylor DJ, 2010, BMC EVOL BIOL, V10, DOI 10.1186/1471-2148-10-193
Teeling EC, 2005, SCIENCE, V307, P580, DOI 10.1126/science.1105113
Teeling EC, 2018, ANNU REV ANIM BIOSCI, V6, P23, DOI 10.1146/annurev-animal-022516-022811
Timothy F, 2015, BIOL PATHOGENSSIS RH, P427
Wang LF, 2019, CURR OPIN VIROL, V34, P79, DOI 10.1016/j.coviro.2018.12.007
Wang LF, 2011, CURR OPIN VIROL, V1, P649, DOI 10.1016/j.coviro.2011.10.013
Weiss RA, 2013, PHILOS T R SOC B, V368, DOI 10.1098/rstb.2012.0494
Whitfield ZJ, 2017, CURR BIOL, V27, P3511, DOI 10.1016/j.cub.2017.09.067
Xie JZ, 2018, CELL HOST MICROBE, V23, P297, DOI 10.1016/

j.chom.2018.01.006
   Xu W, 2014, CELL HOST MICROBE, V16, P187, DOI 10.1016/
j.chom.2014.07.008
   Yang XL, 2019, NAT MICROBIOL, V4, P390, DOI 10.1038/
s41564-018-0328-y
   Young GR, 2012, PLOS PATHOG, V8, DOI 10.1371/journal.ppat.1002709
   Mendoza MLZ, 2018, NAT ECOL EVOL, V2, P659, DOI 10.1038/
s41559-018-0476-8
   Zhang GJ, 2013, SCIENCE, V339, P456, DOI 10.1126/science.1230835
   Zhou P, 2016, P NATL ACAD SCI USA, V113, P2696, DOI 10.1073/
pnas.1518240113
   Zhuo XY, 2015, PLOS PATHOG, V11, DOI 10.1371/journal.ppat.1005279
   Zhuo XY, 2013, J VIROL, V87, P8493, DOI 10.1128/JVI.00892-13
   Zong J, 2009, GENE, V447, P29, DOI 10.1016/j.gene.2009.07.004
NR 123
TC 30
Z9 31
U1 0
U2 17
PU ANNUAL REVIEWS
PI PALO ALTO
PA 4139 EL CAMINO WAY, PO BOX 10139, PALO ALTO, CA 94303-0897 USA
SN 2327-056X
EI 2327-0578
J9 ANNU REV VIROL
PY 2020
VL 7
BP 103
EP 119
DI 10.1146/annurev-virology-092818-015613
PG 17
WC Virology
WE Book Citation Index – Science (BKCI-S); Science Citation Index
Expanded (SCI-EXPANDED)
SC Virology
GA BQ6ZW
UT WOS:000613942300006
PM 32432980
DA 2024-10-21
ER

PT J
AU Kim, Y
   Leopardi, S
   Scaravelli, D
   Zecchin, B
   Priori, P
   Festa, F
   Drzewnioková, P
   De Benedictis, P
   Nouvellet, P
AF Kim, Younjung
   Leopardi, Stefania
   Scaravelli, Dino

Zecchin, Barbara
Priori, Pamela
Festa, Francesca
Drzewniokova, Petra
De Benedictis, Paola
Nouvellet, Pierre
TI Transmission dynamics of lyssavirus in <i>Myotis myotis</i>: mechanistic
modelling study based on longitudinal seroprevalence data
SO PROCEEDINGS OF THE ROYAL SOCIETY B-BIOLOGICAL SCIENCES
LA English
DT Article
DE lyssavirus; EBLV-1; Myotis bats; bat-borne virus; zoonosis; transmission
dynamics
ID VIRUS-INFECTION; SEROTINE BATS; HUMAN RABIES; NIPAH VIRUS; SEASONALITY;
HIBERNATION; CHIROPTERA; RESERVOIRS; TYPE-1; HOST
AB We investigated the transmission dynamics of lyssavirus in Myotis myotis and Myotis blythii, using serological, virological, demographic and ecological data collected between 2015 and 2022 from two maternity colonies in northern Italian churches. Despite no lyssavirus detection in 556 bats sampled over 11 events by reverse transcription-polymerase chain reaction (RT-PCR), 36.3% of 837 bats sampled over 27 events showed neutralizing antibodies to European bat lyssavirus 1, with a significant increase in summers. By fitting sets of mechanistic models to seroprevalence data, we investigated factors that influenced lyssavirus transmission within and between years. Five models were selected as a group of final models: in one model, a proportion of exposed bats (median model estimate: 5.8%) became infectious and died while the other exposed bats recovered with immunity without becoming infectious; in the other four models, all exposed bats became infectious and recovered with immunity. The final models supported that the two colonies experienced seasonal outbreaks driven by: (i) immunity loss particularly during hibernation, (ii) density-dependent transmission, and (iii) a high transmission rate after synchronous birthing. These findings highlight the importance of understanding ecological factors, including colony size and synchronous birthing timing, and potential infection heterogeneities to enable more robust assessments of lyssavirus spillover risk.
C1 [Kim, Younjung; Nouvellet, Pierre] Univ Sussex, Sch Life Sci, Dept Evolut Behav & Environm, Brighton BN1 9RH, England.
[Leopardi, Stefania; Zecchin, Barbara; Festa, Francesca; Drzewniokova, Petra; De Benedictis, Paola] Ist Zooprofilatt Sperimentale Venezie, FAO, Viale Univ 10, I-35020 Legnaro, Italy.
[Leopardi, Stefania; Zecchin, Barbara; Festa, Francesca; Drzewniokova, Petra; De Benedictis, Paola] Ist Zooprofilatt Sperimentale Venezie, Natl Reference Ctr Rabies, Viale Univ 10, I-35020 Legnaro, Italy.
[Scaravelli, Dino; Priori, Pamela] STERNA, Via Pedrali 12, I-47121 Forli, Italy.
[Scaravelli, Dino; Priori, Pamela] Museo Ornitol F Foschi, Via Pedrali 12, I-47121 Forli, Italy.

[Scaravelli, Dino] Univ Bologna, Dept Biol Geol & Environm Sci, Via Selmi 3, I-40126 Bologna, Italy.
[Nouvellet, Pierre] Imperial Coll London, Sch Publ Hlth, Dept Infect Dis Epidemiol, London SW7 2AZ, England.
C3 University of Sussex; IZS delle Venezie; Food & Agriculture Organization
of the United Nations (FAO); IZS delle Venezie; University of Bologna;
Imperial College London
RP Nouvellet, P (corresponding author), Univ Sussex, Sch Life Sci, Dept Evolut Behav & Environm, Brighton BN1 9RH, England.; Nouvellet, P (corresponding author), Imperial Coll London, Sch Publ Hlth, Dept Infect Dis Epidemiol, London SW7 2AZ, England.
EM pierre.nouvellet@sussex.ac.uk
RI De Benedictis, Paola/T-3273-2019; Leopardi, Stefania/ABD-8236-2021
OI nouvellet, pierre/0000-0002-6094-5722
FU BBSRC, through the ERA-NET ICRAD program [BB/V019945/1]; Italian Ministry of Health [GR-2011-023505919]; Ricerca Corrente [RC-IZSVe-8/18]; Ricerca Corrente [RC-IZSVe-6/19]; BBSRC [BB/V019945/1]
Funding Source: UKRI
FX Y.K. and P.N. acknowledge support from the BBSRC, through the ERA-NET
ICRAD program (grant no. BB/V019945/1). Batsampling and laboratory
analyses were funded by the Italian Ministry of Health through grants
WFR GR-2011-023505919, Ricerca Corrente RC-IZSVe-8/18 and Ricerca Corrente RC-IZSVe-6/19.
CR Altizer S, 2006, ECOL LETT, V9, P467, DOI 10.1111/j.1461-0248.2005.00879.x
Amengual B, 2007, PLOS ONE, V2, DOI 10.1371/journal.pone.0000566
Amman BR, 2012, PLOS PATHOG, V8, DOI 10.1371/journal.ppat.1002877
[Anonymous], 1986, MMWR MORBIDITY MORTA, V35, P430
Arlettaz R, 1997, J ZOOL, V242, P45, DOI 10.1111/j.1469-7998.1997.tb02928.x
AUDET D, 1990, J MAMMAL, V71, P420, DOI 10.2307/1381955
Banyard AC, 2011, ADV VIRUS RES, V79, P239, DOI 10.1016/B978-0-12-387040-7.00012-3
Bates D, 2015, J STAT SOFTW, V67, P1, DOI 10.18637/jss.v067.i01
Blackwood JC, 2013, P NATL ACAD SCI USA, V110, P20837, DOI 10.1073/pnas.1308817110
Bouma HR, 2011, P NATL ACAD SCI USA, V108, P2052, DOI 10.1073/pnas.1008823108
Bouma HR, 2010, J LEUKOCYTE BIOL, V88, P619, DOI 10.1189/jlb.0310174
Breed AC, 2011, PLOS ONE, V6, DOI 10.1371/journal.pone.0028816
Brook CE, 2019, J ANIM ECOL, V88, P1001, DOI 10.1111/1365-2656.12985
Burnham KP, 2004, SOCIOL METHOD RES, V33, P261, DOI 10.1177/0049124104268644
Christe P, 2000, ECOL LETT, V3, P207
Colombi D, 2019, SCI REP-UK, V9, DOI 10.1038/s41598-018-36485-y

Dacheux L, 2009, EMERG INFECT DIS, V15, P280, DOI 10.3201/eid1502.080637
De Benedictis P, 2011, J CLIN MICROBIOL, V49, P1932, DOI 10.1128/JCM.02015-10
Drexler JF, 2011, EMERG INFECT DIS, V17, P449, DOI 10.3201/eid1703.100526
Epstein JH, 2020, P NATL ACAD SCI USA, V117, P29190, DOI 10.1073/pnas.2000429117
FitzJohn Rich, 2023, CRAN
Folly AJ, 2021, VIRUSES-BASEL, V13, DOI 10.3390/v13101979
Fooks AR, 2003, EPIDEMIOL INFECT, V131, P1029, DOI 10.1017/S0950268803001481
Franka R, 2008, J GEN VIROL, V89, P1998, DOI 10.1099/vir.0.83688-0
Freuling C, 2009, J GEN VIROL, V90, P2493, DOI 10.1099/vir.0.011510-0
Gentles AD, 2020, PATHOG GLOB HEALTH, V114, P407, DOI 10.1080/20477724.2020.1833161
Georgea DB, 2011, P NATL ACAD SCI USA, V108, P10208, DOI 10.1073/pnas.1010875108
Hayman DTS, 2018, PLOS ONE, V13, DOI 10.1371/journal.pone.0198563
Hayman DTS, 2015, P ROY SOC B-BIOL SCI, V282, DOI 10.1098/rspb.2014.2591
IRWIN DM, 1991, J MOL EVOL, V32, P128, DOI 10.1007/BF02515385
Jackson FR, 2008, J WILDLIFE DIS, V44, P612, DOI 10.7589/0090-3558-44.3.612
Joffrin L, 2022, ROY SOC OPEN SCI, V9, DOI 10.1098/rsos.211600
Johara MY, 2001, EMERG INFECT DIS, V7, P439
Johnson N, 2008, J GEN VIROL, V89, P2662, DOI 10.1099/vir.0.2008/003889-0
Kim Y., 2023, VIRUSES-BASEL, DOI [10.6084/m9.figshare.c.6486249, DOI 10.6084/M9.FIGSHARE.C.6486249]
Kunz T., 2009, Ecological and behavioral methods for the study of bats, V2nd, P901
Leopardi S, 2019, EPIDEMIOL INFECT, V147, DOI [10.1017/s0950268818003072, 10.1017/S0950268818003072]
Leroy EM, 2005, NATURE, V438, P575, DOI 10.1038/438575a
Li WD, 2005, SCIENCE, V310, P676, DOI 10.1126/science.1118391
LUMIO J, 1986, LANCET, V1, P378
Müller T, 2004, J VET MED B, V51, P49, DOI 10.1111/j.1439-0450.2003.00725.x
Nathwani D, 2003, CLIN INFECT DIS, V37, P598, DOI 10.1086/376641
Peel AJ, 2014, P ROY SOC B-BIOL SCI, V281, DOI 10.1098/rspb.2013.2962
Peel AJ, 2019, EMERG MICROBES INFEC, V8, P1314, DOI 10.1080/22221751.2019.1661217
Peel AJ, 2018, SCI REP-UK, V8, DOI 10.1038/s41598-018-22236-6
Picard-Meyer E, 2017, BMC VET RES, V13, DOI 10.1186/s12917-017-1303-1
Plowright RK, 2008, P ROY SOC B-BIOL SCI, V275, P861, DOI 10.1098/rspb.2007.1260
Plowright RK, 2016, PLOS NEGLECT TROP D, V10, DOI 10.1371/journal.pntd.0004796
Pons-Salort M, 2014, PLOS ONE, V9, DOI 10.1371/

journal.pone.0095610
R Core Team, 2022, R LANG ENV STAT COMP
Regnault B, 2022, CLIN INFECT DIS, V74, P461, DOI 10.1093/cid/ciab443
ROINE RO, 1988, BRAIN, V111, P1505, DOI 10.1093/brain/111.6.1505
Ruoss S, 2019, MAMM BIOL, V94, P120, DOI 10.1016/j.mambio.2018.05.010
Schatz J, 2013, ZOONOSES PUBLIC HLTH, V60, P22, DOI 10.1111/zph.12002
Serra-Cobo J, 2013, PLOS ONE, V8, DOI 10.1371/journal.pone.0064467
Shankar V, 2004, J WILDLIFE DIS, V40, P403, DOI 10.7589/0090-3558-40.3.403
Shipley R, 2019, TROP MED INFECT DIS, V4, DOI 10.3390/tropicalmed4010031
Simic I, 2018, BMC VET RES, V14, DOI 10.1186/s12917-018-1592-z
Swanepoel R, 2007, EMERG INFECT DIS, V13, P1847, DOI 10.3201/eid1312.071115
Tjornehoj K, 2006, J COMP PATHOL, V134, P190, DOI 10.1016/j.jcpa.2005.10.005
Wacharapluesadee S, 2018, VIROL J, V15, DOI 10.1186/s12985-018-0950-6
Wacharapluesadee S, 2010, VECTOR-BORNE ZOONOT, V10, P183, DOI 10.1089/vbz.2008.0105
WHO, 2018, WHO TECH REP SER, V1012, P1
Wood SN, 2011, J ROY STAT SOC B, V73, P3, DOI 10.1111/j.1467-9868.2010.00749.x
Woolhouse MEJ, 1997, P NATL ACAD SCI USA, V94, P338, DOI 10.1073/pnas.94.1.338
NR 65
TC 2
Z9 3
U1 1
U2 3
PU ROYAL SOC
PI LONDON
PA 6-9 CARLTON HOUSE TERRACE, LONDON SW1Y 5AG, ENGLAND
SN 0962-8452
EI 1471-2954
J9 P ROY SOC B-BIOL SCI
JI Proc. R. Soc. B-Biol. Sci.
PD APR 26
PY 2023
VL 290
IS 1997
AR 20230183
DI 10.1098/rspb.2023.0183
PG 12
WC Biology; Ecology; Evolutionary Biology
WE Science Citation Index Expanded (SCI-EXPANDED)
SC Life Sciences & Biomedicine - Other Topics; Environmental Sciences &
Ecology; Evolutionary Biology
GA E2YV4

UT WOS:000974262200009
PM 37072038
OA Green Published, hybrid
DA 2024-10-21
ER

PT J
AU Tovstuha, I
   Timofieieva, O
   Kovalov, V
   Vlaschenko, A
AF Tovstuha, Ihor
   Timofieieva, Olha
   Kovalov, Viktor
   Vlaschenko, Anton
TI Effect of ⁹⁰Sr and ¹³⁷Cs concentration on
   hematological parameters of bats in the Chernobyl Exclusion Zone
   (Ukraine)
SO ACTA CHIROPTEROLOGICA
LA English
DT Article
DE Chiroptera; radionuclides; hematological parameters; strontium;
caesium;
   eco-immunology
ID BLOOD
AB We examined the effects of the level of internal Sr-90 and Cs-137
concentrations on hematological parameters of bats captured in the
Chernobyl Exclusion Zone (Ukraine). In total, 68 animals of six bat
species were live-monitored with measurement of the whole-body
activity concentrations of the two radionuclides (Sr-90 and Cs-137)
and blood sampling for further analysis. We found statistically
significant positive correlations between the blood neutrophil count
and whole-body Sr-90 activity concentration in individuals of
Eptesicus serotinus; and between the neutrophil/lymphocyte ratio and
Sr-90 concentration, and between the red blood cell (RBC) count and
Cs-137 concentration in Vespertilio murinus. We did not observe any
correlation between concentrations of Sr-90 and Cs-137 and
hematological parameters in individuals of Pipistrellus nathusii.
Moreover. none of these three species showed significant sexual
(male versus female) or age (adult versus sexually immature)
differences in Sr-90 and Cs-137 concentrations. Our results
contribute to better knowledge and understanding of the eco-
immunology of free-ranging bats in one of the most radioactively
contaminated areas in the world - the Chernobyl Exclusion Zone. To
our knowledge, this is the first study investigating the
relationship between radionuclide contamination and hematological
parameters of bats.
C1 [Tovstuha, Ihor; Vlaschenko, Anton] Bat Rehabil Ctr Feldman
Ecopk, UA-62340 Lesnoye, Kharkiv Region, Ukraine.
   [Timofieieva, Olha] Jagiellonian Univ, Inst Environm Sci, Terr
Ecosyst & Ecotoxicologv Grp, Gronostajowa 7, PL-30387 Krakow,
Poland.
   [Kovalov, Viktor] Univ Zurich, Dept Evolutionary Biol & Environm
Studies, Winterthurerstr 190, CH-8057 Zurich, Switzerland.

[Vlaschenko, Anton] HS Skovoroda Kharkiv Natl Pedag Univ, Inst Nat Sci, Valentynivska St 2, UA-61168 Kharkiv, Ukraine.
C3 Jagiellonian University; University of Zurich; Ministry of Education &
Science of Ukraine; H.S. Skovoroda Kharkiv National Pedagogical University
RP Timofieieva, O (corresponding author), Jagiellonian Univ, Inst Environm Sci, Terr Ecosyst & Ecotoxicology Grp, Gronostajowa 7, PL-30387 Krakow, Poland.
EM olhatimofieieva@gmail.com
RI Vlaschenko, Anton/JMQ-2607-2023
OI Timofieieva, Olha/0000-0002-1049-8557
CR Baker ML., 2018, Advances in Comparative Immunology, P839
Beresford NA, 2020, J ENVIRON RADIOACTIV, V211, DOI 10.1016/j.jenvrad.2019.01.005
Beresford NA, 2016, J ENVIRON RADIOACTIV, V157, P77, DOI 10.1016/j.jenvrad.2016.02.003
El-Shanshoury H, 2016, J RADIAT RES APPL SI, V9, P282, DOI 10.1016/j.jrras.2016.01.001
GASHCHAK S., 2013, Problems of Chernobyl Exclusion Zone, V11, P56
GASHCHAK S. P., 2009, PROBLEMS CHERNOBYL E, V9, P102
Gashchak S, 2015, HYSTRIX, V26, P59, DOI 10.4404/hystrix-26.1-10624
Gashchak S, 2010, RADIAT ENVIRON BIOPH, V49, P635, DOI 10.1007/s00411-010-0322-0
Haskins DL, 2020, J ENVIRON RADIOACTIV, V213, DOI 10.1016/j.jenvrad.2019.106107
Jones Gareth, 2009, Endangered Species Research, V8, P93, DOI 10.3354/esr00182
LITTLE J. B., 2011, COMPR PHYSIOL, P127, DOI [10.1002/cphy.cp090108, DOI 10.1002/CPHY.CP090108]
Naidoo S, 2015, ECOTOX ENVIRON SAFE, V114, P304, DOI 10.1016/j.ecoenv.2014.04.043
Piraccini R., 2016, IUCN RED LIST THREAT, DOI DOI 10.2305/IUCN.UK.2016-2.RLTS.T2553A22029285.EN
Racey PA, 2011, ACTA CHIROPTEROL, V13, P223, DOI 10.3161/150811011X578787
Rodriguez-Caballero E, 2018, NAT GEOSCI, V11, P185, DOI 10.1038/s41561-018-0072-1
Ruiz SR, 2019, ECOTOX ENVIRON SAFE, V169, P93, DOI 10.1016/j.ecoenv.2018.10.083
Russo D, 2015, MAMM BIOL, V80, P157, DOI 10.1016/j.mambio.2015.03.005
Seltmann A, 2017, CONSERV PHYSIOL, V5, DOI 10.1093/conphys/cox020
Sikes RS, 2016, J MAMMAL, V97, P663, DOI 10.1093/jmammal/gyw078
Smith CS, 2011, ACTA CHIROPTEROL, V13, P224
Smith CS, 2010, ACTA CHIROPTEROL, V12, P255, DOI 10.3161/150811010X504752
Strobel S, 2015, MAMM BIOL, V80, P312, DOI 10.1016/j.mambio.2015.02.005
Vlaschenko A., 2020, Zoodiversity, V54, P53, DOI 10.15407/zoo2020.01.053
Vlaschenko A, 2016, TURK J ZOOL, V40, P876, DOI 10.3906/zoo-1508-56

   Voigt CC, 2020, SCI REP-UK, V10, DOI 10.1038/s41598-020-74473-3
   Warren RW, 2014, J ENVIRON RADIOACTIV, V129, P86, DOI 10.1016/
j.jenvrad.2013.11.005
   Zocche JJ, 2010, ENVIRON RES, V110, P684, DOI 10.1016/
j.envres.2010.06.003
NR 27
TC 0
Z9 0
U1 0
U2 4
PU MUSEUM & INST ZOOLOGY PAS-POLISH ACAD SCIENCES
PI WARSAW
PA WILCZA STREET 64, 00-679 WARSAW, POLAND
SN 1508-1109
EI 1733-5329
J9 ACTA CHIROPTEROL
JI Acta Chiropt.
PD DEC
PY 2021
VL 23
IS 2
BP 371
EP 376
DI 10.3161/15081109ACC2021.23.2.006
PG 6
WC Zoology
WE Science Citation Index Expanded (SCI-EXPANDED)
SC Zoology
GA ZE6RB
UT WOS:000759006400006
DA 2024-10-21
ER

PT J
AU Ingala, MR
   Becker, DJ
   Holm, JB
   Kristiansen, K
   Simmons, NB
AF Ingala, Melissa R.
   Becker, Daniel J.
   Holm, Jacob Bak
   Kristiansen, Karsten
   Simmons, Nancy B.
TI Habitat fragmentation is associated with dietary shifts and microbiota
   variability in common vampire bats
SO ECOLOGY AND EVOLUTION
LA English
DT Article
DE Desmodus rotundus; diet homogenization; land-use change; livestock;
   microbiota; resource provisioning
ID DESMODUS-ROTUNDUS; GUT MICROBIOME; HOST; COMMUNITIES; CHIROPTERA;

ECOIMMUNOLOGY; TRANSMISSION; DISRUPTION; PHYSIOLOGY; PREFERENCE
AB Host ecological factors and external environmental factors are known to influence the structure of gut microbial communities, but few studies have examined the impacts of environmental changes on microbiotas in free-ranging animals. Rapid land-use change has the potential to shift gut microbial communities in wildlife through exposure to novel bacteria and/or by changing the availability or quality of local food resources. The consequences of such changes to host health and fitness remain unknown and may have important implications for pathogen spillover between humans and wildlife. To better understand the consequences of land-use change on wildlife microbiotas, we analyzed long-term dietary trends, gut microbiota composition, and innate immune function in common vampire bats (Desmodus rotundus) in two nearby sites in Belize that vary in landscape structure. We found that vampire bats living in a small forest fragment had more homogenous diets indicative of feeding on livestock and shifts in microbiota heterogeneity, but not overall composition, compared to those living in an intact forest reserve. We also found that irrespective of sampling site, vampire bats which consumed relatively more livestock showed shifts in some core bacteria compared with vampire bats which consumed relatively less livestock. The relative abundance of some core microbiota members was associated with innate immune function, suggesting that future research should consider the role of the host microbiota in immune defense and its relationship to zoonotic infection dynamics. We suggest that subsequent homogenization of diet and habitat loss through livestock rearing in the Neotropics may lead to disruption to the microbiota that could have downstream impacts on host immunity and cross-species pathogen transmission.
C1 [Ingala, Melissa R.] Amer Museum Nat Hist, Richard Gilder Grad Sch, 79th St Cent Pk West, New York, NY 10024 USA.
[Ingala, Melissa R.; Simmons, Nancy B.] Amer Museum Nat Hist, Div Vertebrate Zool, Dept Mammal, New York, NY 10024 USA.
[Becker, Daniel J.] Univ Georgia, Odum Sch Ecol, Athens, GA 30602 USA.
[Becker, Daniel J.] Univ Georgia, Ctr Ecol Infect Dis, Athens, GA 30602 USA.
[Becker, Daniel J.] Indiana Univ, Dept Biol, Bloomington, IN USA.
[Holm, Jacob Bak; Kristiansen, Karsten] Univ Copenhagen, Dept Biol, Copenhagen, Denmark.
[Holm, Jacob Bak] Clin Microbi, Copenhagen, Denmark.
[Kristiansen, Karsten] BGI, Shenzhen, Peoples R China.
C3 American Museum of Natural History (AMNH); American Museum of Natural
History (AMNH); University System of Georgia; University of Georgia;
University System of Georgia; University of Georgia; Indiana University
System; Indiana University Bloomington; University of Copenhagen;
Beijing Genomics Institute (BGI)
RP Ingala, MR (corresponding author), Amer Museum Nat Hist, Richard Gilder Grad Sch, 79th St Cent Pk West, New York, NY 10024 USA.
EM ingala.melissar@gmail.com
RI Holm, Jacob/B-8677-2019; Kristiansen, Karsten/J-5148-2014;

Becker,
Daniel/A-6466-2014
OI Kristiansen, Karsten/0000-0002-6024-0917; Becker,
Daniel/0000-0003-4315-8628
FU Explorers Club; American Museum of Natural History; Achievement Rewards
for College Scientists Foundation; Danish National Science Research
Council; Carlsbergfondet; National Science Foundation; ARCS Foundation;
Sigma Xi
FX Explorers Club, Grant/Award Number: Research Grant; American Museum of
Natural History, Grant/Award Number: Graduate Research Fellowship;
Achievement Rewards for College Scientists Foundation; Danish National
Science Research Council; Carlsbergfondet; National Science Foundation,
Grant/Award Number: Graduate Research Fellowship; ARCS Foundation; Sigma
Xi, Grant/Award Number: Grant-in-Aid
CR Altizer S, 2018, PHILOS T R SOC B, V373, DOI 10.1098/rstb.2017.0102
Amato KR, 2015, MICROB ECOL, V69, P434, DOI 10.1007/s00248-014-0554-7
Amato KR, 2013, ISME J, V7, P1344, DOI 10.1038/ismej.2013.16
Amman BR, 2012, PLOS PATHOG, V8, DOI 10.1371/journal.ppat.1002877
[Anonymous], 1988, The Natural History of Vampire Bats
Antony-Babu S, 2017, SCI REP-UK, V7, DOI 10.1038/s41598-017-11363-1
Apprill A, 2015, AQUAT MICROB ECOL, V75, P129, DOI 10.3354/ame01753
Araújo-Pérez F, 2012, GUT MICROBES, V3, P530, DOI 10.4161/gmic.22157
Bahrndorff S, 2016, INT J GENOMICS, V2016, DOI 10.1155/2016/5304028
Barelli C, 2015, SCI REP-UK, V5, DOI 10.1038/srep14862
Becker CG, 2017, P ROY SOC B-BIOL SCI, V284, DOI 10.1098/rspb.2017.0582
Becker DJ, 2018, PLOS NEGLECT TROP D, V12, DOI 10.1371/journal.pntd.0006786
Becker DJ, 2018, PHILOS T R SOC B, V373, DOI 10.1098/rstb.2017.0089
Becker DJ, 2017, ROY SOC OPEN SCI, V4, DOI 10.1098/rsos.170073
Bobrowiec PED, 2015, J MAMMAL, V96, P54, DOI 10.1093/jmammal/gyu002
Bokulich NA, 2018, MICROBIOME, V6, DOI 10.1186/s40168-018-0470-z
Bolyen E, 2019, NAT BIOTECHNOL, V37, P852, DOI 10.1038/s41587-019-0209-9
Callahan BJ, 2016, NAT METHODS, V13, P581, DOI [10.1038/NMETH.3869, 10.1038/nmeth.3869]
Caporaso JG, 2011, P NATL ACAD SCI USA, V108, P4516, DOI 10.1073/pnas.1000080107

Caporaso JG, 2010, NAT METHODS, V7, P335, DOI 10.1038/nmeth.f.303
Carrillo-Araujo M, 2015, FRONT MICROBIOL, V6, DOI 10.3389/fmicb.2015.00447
Cheng YY, 2015, MICROBIOME, V3, DOI 10.1186/s40168-015-0143-0
Cherrington E.A., 2010, TECHNICAL REPORT
Clare EL, 2014, FUNCT ECOL, V28, P632, DOI 10.1111/1365-2435.12192
David LA, 2014, NATURE, V505, P559, DOI 10.1038/nature12820
Davis NM, 2018, MICROBIOME, V6, DOI 10.1186/s40168-018-0605-2
Delpietro HA, 2017, ROY SOC OPEN SCI, V4, DOI 10.1098/rsos.160959
Demas GE, 2011, J ANIM ECOL, V80, P710, DOI 10.1111/j.1365-2656.2011.01813.x
Condori-Condori RE, 2013, EMERG INFECT DIS, V19, P1463, DOI 10.3201/eid1909.130083
Evans JK, 2017, HORM BEHAV, V88, P112, DOI 10.1016/j.yhbeh.2016.12.003
Fassbinder-Orth CA, 2014, INTEGR COMP BIOL, V54, P396, DOI 10.1093/icb/icu023
FENTON MB, 1992, BIOTROPICA, V24, P440, DOI 10.2307/2388615
Goodwin GG, 1961, REV BATS TRINIDAD TO
GOWER JC, 1966, BIOMETRIKA, V53, P325, DOI 10.2307/2333639
GREENHALL A M, 1983, Mammalian Species, P1, DOI 10.2307/3503895
Greenhall AM., 1969, I BIOL UNAM ZOOL, V40, P245, DOI [10.2307/2989817, DOI 10.2307/2989817]
Hale VL, 2015, J MICROBIOL METH, V113, P16, DOI 10.1016/j.mimet.2015.03.021
Hanning I, 2015, MICROBIOME, V3, DOI 10.1186/s40168-015-0113-6
Herrera JP, 2018, ACTA CHIROPTEROL, V20, P147, DOI 10.3161/15081109ACC2018.20.1.011
HOARE CECIL A., 1965, ACTA TROP, V22, P204
Hooper LV, 2012, SCIENCE, V336, P1268, DOI 10.1126/science.1223490
Hughey MC, 2017, PEERJ, V5, DOI 10.7717/peerj.3688
Ichinohe T, 2011, P NATL ACAD SCI USA, V108, P5354, DOI 10.1073/pnas.1019378108
Ingala MR, 2018, FRONT MICROBIOL, V9, DOI 10.3389/fmicb.2018.00803
Jackson AL, 2011, J ANIM ECOL, V80, P595, DOI 10.1111/j.1365-2656.2011.01806.x
Karst S. M., 2016, BIORXIV, V10, P70771, DOI [10.1101/070771, DOI 10.1101/070771]
Kau AL, 2011, NATURE, V474, P327, DOI 10.1038/nature10213
Khosravi A, 2013, CURR OPIN MICROBIOL, V16, P221, DOI 10.1016/j.mib.2013.03.009
Koller M, 2016, J STAT SOFTW, V75, P1, DOI 10.18637/jss.v075.i06
Lemieux-Labonté V, 2017, MICROBIOME, V5, DOI 10.1186/s40168-017-0334-y
LOVE MI, 2014, GENOME BIOL, V15, DOI DOI 10.1186/S13059-014-0550-8
Lozupone C, 2006, BMC BIOINFORMATICS, V7, DOI 10.1186/1471-2105-7-371
Balcázar JL, 2007, BRIT J NUTR, V97, P522, DOI 10.1017/S0007114507432986
Macpherson AJ, 2004, NAT REV IMMUNOL, V4, P478, DOI 10.1038/

nri1373
Mayen F, 2003, J VET MED B, V50, P469, DOI 10.1046/j.1439-0450.2003.00713.x
McCord AI, 2014, AM J PRIMATOL, V76, P347, DOI 10.1002/ajp.22238
McKenzie VJ, 2017, INTEGR COMP BIOL, V57, P690, DOI 10.1093/icb/icx090
McMurdie PJ, 2014, PLOS COMPUT BIOL, V10, DOI 10.1371/journal.pcbi.1003531
McMurdie PJ, 2013, PLOS ONE, V8, DOI 10.1371/journal.pone.0061217
Moore MS, 2011, PLOS ONE, V6, DOI 10.1371/journal.pone.0027430
Muegge BD, 2011, SCIENCE, V332, P970, DOI 10.1126/science.1198719
MULLER HE, 1980, ZBL VET MED B, V27, P419
O'Sulliyan A, 2013, J PROTEOME RES, V12, P2833, DOI 10.1021/pr4001702
Olival KJ, 2017, NATURE, V546, P646, DOI 10.1038/nature22975
Pedersen AB, 2011, MOL ECOL, V20, P872, DOI 10.1111/j.1365-294X.2010.04938.x
Phillips CD, 2017, INTEGR COMP BIOL, V57, P743, DOI 10.1093/icb/icx011
Phillips CD, 2012, MOL ECOL, V21, P2617, DOI 10.1111/j.1365-294X.2012.05568.x
Plowright RK, 2015, P ROY SOC B-BIOL SCI, V282, DOI 10.1098/rspb.2014.2124
Post DM, 2002, ECOLOGY, V83, P703, DOI 10.2307/3071875
Schneider MC, 2009, REV PANAM SALUD PUBL, V25, P260, DOI 10.1590/S1020-49892009000300010
Sikes RS, 2016, J MAMMAL, V97, P663, DOI 10.1093/jmammal/gyw078
Stecher B, 2013, NAT REV MICROBIOL, V11, P277, DOI 10.1038/nrmicro2989
Streicker DG, 2016, J APPL ECOL, V53, P1280, DOI 10.1111/1365-2664.12690
Streicker DG, 2012, P ROY SOC B-BIOL SCI, V279, P3384, DOI 10.1098/rspb.2012.0538
Thaiss CA, 2016, NATURE, V535, P65, DOI 10.1038/nature18847
Thomason CA, 2017, SCI REP-UK, V7, DOI 10.1038/s41598-017-16393-3
Tieleman BI, 2005, P ROY SOC B-BIOL SCI, V272, P1715, DOI 10.1098/rspb.2005.3155
TIESZEN LL, 1983, OECOLOGIA, V57, P32, DOI 10.1007/BF00379558
Tong SX, 2013, PLOS PATHOG, V9, DOI 10.1371/journal.ppat.1003657
Trajano E, 1996, BIOTROPICA, V28, P121, DOI 10.2307/2388777
Trevelline BK, 2019, P ROY SOC B-BIOL SCI, V286, DOI 10.1098/rspb.2018.2448
Turner D., 1975, VAMPIRE BAT FIELD ST
Veikkolainen V, 2014, EMERG INFECT DIS, V20, P960, DOI 10.3201/eid2006.130956
Voigt CC, 2006, J MAMMAL, V87, P1, DOI 10.1644/05-MAMM-F-276R1.1
Volokhov D V, 2017, Epidemiol Infect, V145, P3154, DOI [10.1017/s095026881700231x, 10.1017/S095026881700231X]
Wei FW, 2019, SCI CHINA LIFE SCI, V62, P168, DOI 10.1007/s11427-018-9423-3
West AG, 2019, BIOL CONSERV, V229, P85, DOI 10.1016/j.biocon.2018.11.016
Williams CL, 2016, FRONT MICROBIOL, V7, DOI 10.3389/fmicb.2016.00661

Woodhams DC, 2014, PLOS ONE, V9, DOI 10.1371/journal.pone.0096375
Xiong T, 2020, SENSOR ACTUAT B-CHEM, V304, DOI [10.1016/j.snb.2019.127310, 10.1126/scitranslmed.3009124]
Zaneveld JR, 2017, NAT MICROBIOL, V2, DOI 10.1038/nmicrobiol.2017.121
Mendoza MLZ, 2018, NAT ECOL EVOL, V2, P659, DOI 10.1038/s41559-018-0476-8
Zetun CB, 2009, J VENOM ANIM TOXINS, V15, P546, DOI 10.1590/S1678-91992009000300014
Zinsstag J, 2011, PREV VET MED, V101, P148, DOI 10.1016/j.prevetmed.2010.07.003
Zuur Alain F., 2009, P1
NR 95
TC 57
Z9 64
U1 4
U2 39
PU WILEY
PI HOBOKEN
PA 111 RIVER ST, HOBOKEN 07030-5774, NJ USA
SN 2045-7758
J9 ECOL EVOL
JI Ecol. Evol.
PD JUN
PY 2019
VL 9
IS 11
BP 6508
EP 6523
DI 10.1002/ece3.5228
PG 16
WC Ecology; Evolutionary Biology
WE Science Citation Index Expanded (SCI-EXPANDED)
SC Environmental Sciences & Ecology; Evolutionary Biology
GA IE8KN
UT WOS:000472622400031
PM 31236240
OA Green Published, gold
DA 2024-10-21
ER

PT J
AU Periasamy, P
Hutchinson, PE
Chen, JM
Bonne, I
Hameed, SSS
Selvam, P
Hey, YY
Fink, K
Irving, AT
Dutertre, CA
Bakers, M
Crameri, G

Wang, LF
Alonso, S
AF Periasamy, Pravin
Hutchinson, Paul E.
Chen, Jinmiao
Bonne, Isabelle
Hameed, Shahana Shereene Shahul
Selvam, Pavithra
Hey, Ying Ying
Fink, Katja
Irving, Aaron T.
Dutertre, Charles-Antoine
Bakers, Michelle
Crameri, Gary
Wang, Lin-Fa
Alonso, Sylvie
TI Studies on B Cells in the Fruit-Eating Black Flying Fox
(<i>Pteropus
alecto</i>)
SO FRONTIERS IN IMMUNOLOGY
LA English
DT Article
DE bat; adaptive immunity; Pteropus alecto; Eonycteris spelaea;
cross-reactive antibodies
ID TOLL-LIKE RECEPTORS; CROSS-REACTIVITY; BATS; ACTIVATION;
CHALLENGES;
EVOLUTION; ECOLOGY; FLIGHT; HOST
AB The ability of bats to act as reservoir for viruses that are highly pathogenic to humans suggests unique properties and functional characteristics of their immune system. However, the lack of bat specific reagents, in particular antibodies, has limited our knowledge of bat's immunity. Here, we report a panel of cross-reactive antibodies against MHC-II, NK1.1, CD3, CD21, CD27, and immunoglobulin (Ig), that allows flow cytometry analysis of B, T and NK cell populations in two different fruit-eating bat species namely, Pteropus alecto and E. spelaea. Results confirmed predominance of T cells in the spleen and blood of bats, as previously reported by us. However, the percentages of B cells in bone marrow and NK cells in spleen varied greatly between wild caught P. alecto bats and E. spelaea colony bats, which may reflect inherent differences of their immune system or different immune status. Other features of bat B cells were investigated. A significant increase in slg(+) B cell population was observed in the spleen and blood from LPS-injected bats but not from poly I:C-injected bats, supporting T-independent polyclonal B cell activation by LPS. Furthermore, using an in vitro calcium release assay, P. alecto B cells exhibited significant calcium release upon cross-linking of their B cell receptor. Together, this work contributes to improve our knowledge of bat adaptive immunity in particular B cells.
C1 [Periasamy, Pravin; Hameed, Shahana Shereene Shahul; Selvam, Pavithra; Alonso, Sylvie] Natl Univ Singapore, Yong Loo Lin Sch Med, Dept Microbiol & Immunol, Singapore, Singapore.
[Periasamy, Pravin; Hutchinson, Paul E.; Bonne, Isabelle; Hameed,

Shahana Shereene Shahul; Selvam, Pavithra; Alonso, Sylvie] Natl Univ Singapore, Life Sci Inst, Immunol Programme, Singapore, Singapore.
[Chen, Jinmiao; Fink, Katja; Dutertre, Charles-Antoine] ASTAR, Singapore Immunol Network SIgN, Singapore, Singapore.
[Hey, Ying Ying; Irving, Aaron T.; Wang, Lin-Fa] Duke NUS Med Sch, Programme Emerging Infect Dis, Singapore, Singapore.
[Bakers, Michelle] CSIRO, Australian Anim Hlth Lab, Livestock Ind, Geelong, Vic, Australia.
[Crameri, Gary] Cramer Res Consulting, Geelong, Vic, Australia.
C3 National University of Singapore; National University of Singapore;
Agency for Science Technology & Research (A*STAR); A*STAR - Singapore
Immunology Network (SIgN); National University of Singapore;
Commonwealth Scientific & Industrial Research Organisation (CSIRO)
RP Alonso, S (corresponding author), Natl Univ Singapore, Yong Loo Lin Sch Med, Dept Microbiol & Immunol, Singapore, Singapore.; Alonso, S (corresponding author), Natl Univ Singapore, Life Sci Inst, Immunol Programme, Singapore, Singapore.
EM micas@nus.edu.sg
RI Fink, Katja/AAI-1881-2021; Irving, Aaron/AAE-8874-2021; Chen, Jinmiao/ABE-5601-2021; Dutertre, Charles Antoine/JRX-2224-2023; Irving, Aaron/I-6754-2013; Baker, Michelle/C-9694-2013
OI Irving, Aaron/0000-0002-0196-1570; GUERIN-BONNE, Isabelle/0000-0002-8261-6794; Dutertre, Charles-Antoine/0000-0001-7950-3652; Baker, Michelle/0000-0002-7993-9971; Wang, Lin-Fa/0000-0003-2752-0535
FU National Research Foundation [NRF-CRP10-2012-05]
FX This work was supported by the National Research Foundation (awarded to L-FW; NRF-CRP10-2012-05).
CR [Anonymous], MORBIDITY MORTALITY
AUSTAD SN, 1991, J GERONTOL, V46, pB47, DOI 10.1093/geronj/46.2.B47
Baker ML, 2013, ZOONOSES PUBLIC HLTH, V60, P104, DOI 10.1111/j.1863-2378.2012.01528.x
Baker ML, 2010, IMMUNOGENETICS, V62, P173, DOI 10.1007/s00251-010-0425-4
Banerjee A, 2018, VIRUS RES, V248, P5, DOI 10.1016/j.virusres.2018.02.013
Calisher CH, 2006, CLIN MICROBIOL REV, V19, P531, DOI 10.1128/CMR.00017-06
CHAKRABORTY AK, 1983, INDIAN J EXP BIOL, V21, P5
Conrad ML, 2007, CYTOM PART A, V71A, P925, DOI 10.1002/cyto.a.20435
Cowled C, 2011, DEV COMP IMMUNOL, V35, P7, DOI 10.1016/j.dci.2010.07.006
Farady CJ, 2009, BIOORG MED CHEM LETT, V19, P3744, DOI 10.1016/j.bmcl.2009.05.005
Fullerton JN, 2016, JOVE-J VIS EXP, DOI 10.3791/53913
Georgea DB, 2011, P NATL ACAD SCI USA, V108, P10208, DOI 10.1073/pnas.1010875108

Gómez JMM, 2016, SCI REP-UK, V6, DOI 10.1038/srep37796
Gorbunova V, 2014, NAT REV GENET, V15, P531, DOI 10.1038/nrg3728
HEMSLEY SW, 1995, IMMUNOL CELL BIOL, V73, P321, DOI 10.1038/icb.1995.49
Janardhana V, 2012, DEV COMP IMMUNOL, V36, P610, DOI 10.1016/j.dci.2011.11.001
JENNINGS LK, 1989, BLOOD, V74, P2674
Jiang HY, 2017, IMMUNOGENETICS, V69, P271, DOI 10.1007/s00251-016-0966-2
Ksiazek A, 2012, PHYSIOL BIOCHEM ZOOL, V85, P51, DOI 10.1086/663696
Lee AK, 2015, BMC GENOMICS, V16, DOI 10.1186/s12864-015-2124-x
Leong TM, 2011, P NAT SOC, P41
Liu JKH, 2014, ANN MED SURG, V3, P113, DOI 10.1016/j.amsu.2014.09.001
Majstoravich S, 2004, BLOOD, V104, P1396, DOI 10.1182/blood-2004-02-0437
Mamchak AA, 2000, IMMUNOL CELL BIOL, V78, P142, DOI 10.1046/j.1440-1711.2000.00896.x
Munford RS, 2010, J INFECT DIS, V201, P175, DOI 10.1086/649558
Ng JHJ, 2017, BMC GENOMICS, V18, DOI 10.1186/s12864-017-3760-0
O'Shea TJ, 2014, EMERG INFECT DIS, V20, P741, DOI 10.3201/eid2005.130539
Papenfuss AT, 2012, BMC GENOMICS, V13, DOI 10.1186/1471-2164-13-261
Patterson HCK, 2006, IMMUNITY, V25, P55, DOI 10.1016/j.immuni.2006.04.014
Podlutsky AJ, 2005, J GERONTOL A-BIOL, V60, P1366, DOI 10.1093/gerona/60.11.1366
Pone EJ, 2016, METHODS MOL BIOL, V1390, P229, DOI 10.1007/978-1-4939-3335-8_15
Schmidt KM, 1996, DEV CHARACTERIZATION, P1113
Schountz T, 2017, FRONT IMMUNOL, V8, DOI 10.3389/fimmu.2017.01098
Schountz T, 2014, VIRUSES-BASEL, V6, P4880, DOI 10.3390/v6124880
Shaw TI, 2012, PLOS ONE, V7, DOI 10.1371/journal.pone.0048472
Sulkin S E, 1974, Monogr Virol, V8, P1
Virtue ER, 2011, PLOS ONE, V6, DOI 10.1371/journal.pone.0022488
Wang L., 2015, Bats and Viruses: A New Frontier of Emerging Infectious Diseases
Wang LF, 2019, CURR OPIN VIROL, V34, P79, DOI 10.1016/j.coviro.2018.12.007
Wang LF, 2011, CURR OPIN VIROL, V1, P649, DOI 10.1016/j.coviro.2011.10.013
Wynne JW, 2013, PLOS ONE, V8, DOI 10.1371/journal.pone.0052930
Zhang GJ, 2013, SCIENCE, V339, P456, DOI 10.1126/science.1230835
Zhang Q, 2017, PLOS ONE, V12, DOI 10.1371/journal.pone.0182866
Zhou P, 2011, J IMMUNOL, V186, P3138, DOI 10.4049/jimmunol.1003115
NR 44
TC 20
Z9 22
U1 1
U2 11
PU FRONTIERS MEDIA SA

PI LAUSANNE
PA AVENUE DU TRIBUNAL FEDERAL 34, LAUSANNE, CH-1015, SWITZERLAND
SN 1664-3224
J9 FRONT IMMUNOL
JI Front. Immunol.
PD MAR 14
PY 2019
VL 10
AR 489
DI 10.3389/fimmu.2019.00489
PG 11
WC Immunology
WE Science Citation Index Expanded (SCI-EXPANDED)
SC Immunology
GA HO7OJ
UT WOS:000461136700001
PM 30930908
OA Green Published, gold
DA 2024-10-21
ER

PT J
AU Pikula, J
   Heger, T
   Bandouchova, H
   Kovacova, V
   Nemcova, M
   Papezikova, I
   Piacek, V
   Zajícková, R
   Zukal, J
AF Pikula, Jiri
   Heger, Tomas
   Bandouchova, Hana
   Kovacova, Veronika
   Nemcova, Monika
   Papezikova, Ivana
   Piacek, Vladimir
   Zajickova, Renata
   Zukal, Jan
TI Phagocyte activity reflects mammalian homeo- and hetero-thermic
   physiological states
SO BMC VETERINARY RESEARCH
LA English
DT Article
DE Bats; Torpor; Innate immunity; Blood; Phagocytosis; Respiratory
burst
ID WHITE-NOSE SYNDROME; IMMUNE-RESPONSE; ELECTROLYTE DEPLETION;
RESPIRATORY
   BURST; NYCTALUS-NOCTULA; SEASONAL-CHANGES; MYOTIS-MYOTIS; TORPOR
USE;
   BATS; HIBERNATION
AB Background Emergence of both viral zoonoses from bats and
diseases that threaten bat populations has highlighted the necessity

for greater insights into the functioning of the bat immune system. Particularly when considering hibernating temperate bat species, it is important to understand the seasonal dynamics associated with immune response. Body temperature is one of the factors that modulates immune functions and defence mechanisms against pathogenic agents in vertebrates. To better understand innate immunity mediated by phagocytes in bats, we measured respiratory burst and haematology and blood chemistry parameters in heterothermic greater mouse-eared bats (Myotis myotis) and noctules (Nyctalus noctula) and homeothermic laboratory mice (Mus musculus). Results Bats displayed similar electrolyte levels and time-related parameters of phagocyte activity, but differed in blood profile parameters related to metabolism and red blood cell count. Greater mouse-eared bats differed from mice in all phagocyte activity parameters and had the lowest phagocytic activity overall, while noctules had the same quantitative phagocytic values as mice. Homeothermic mice were clustered separately in a high phagocyte activity group, while both heterothermic bat species were mixed in two lower phagocyte activity clusters. Stepwise regression identified glucose, white blood cell count, haemoglobin, total dissolved carbon dioxide and chloride variables as the best predictors of phagocyte activity. White blood cell counts, representing phagocyte numbers available for respiratory burst, were the best predictors of both time-related and quantitative parameters of phagocyte activity. Haemoglobin, as a proxy variable for oxygen available for uptake by phagocytes, was important for the onset of phagocytosis. Conclusions Our comparative data indicate that phagocyte activity reflects the physiological state and blood metabolic and cellular characteristics of homeothermic and heterothermic mammals. However, further studies elucidating trade-offs between immune defence, seasonal lifestyle physiology, hibernation behaviour, roosting ecology and geographic patterns of immunity of heterothermic bat species will be necessary. An improved understanding of bat immune responses will have positive ramifications for wildlife and conservation medicine.
C1 [Pikula, Jiri; Heger, Tomas; Bandouchova, Hana; Kovacova, Veronika; Nemcova, Monika; Papezikova, Ivana; Piacek, Vladimir] Univ Vet & Pharmaceut Sci Brno, Dept Ecol & Dis Zoo Anim Game Fish & Bees, Palackeho Trida 1946-1, Brno 61242, Czech Republic.
[Pikula, Jiri] Univ Vet & Pharmaceut Sci Brno, CEITEC Cent European Inst Technol, Brno, Czech Republic.
[Zajickova, Renata; Zukal, Jan] Masaryk Univ, Dept Bot & Zool, Kotlarska 2, Brno 61137, Czech Republic.
[Zajickova, Renata] Masaryk Univ, Inst Biostat & Anal, Kamenice 3, Brno 62500, Czech Republic.
[Zukal, Jan] Czech Acad Sci, Inst Vertebrate Biol, Kvetna 8, Brno 60365, Czech Republic.
C3 University of Veterinary Sciences Brno; University of Veterinary Sciences Brno; Masaryk University Brno; Institute of Biostatistics & Analyses; Masaryk University Brno; Czech Academy of Sciences; Institute of Vertebrate Biology of the Czech Academy of Sciences
RP Pikula, J; Heger, T (corresponding author), Univ Vet & Pharmaceut Sci Brno, Dept Ecol & Dis Zoo Anim Game Fish & Bees, Palackeho Trida

1946-1, Brno 61242, Czech Republic.; Pikula, J (corresponding
author), Univ Vet & Pharmaceut Sci Brno, CEITEC Cent European Inst
Technol, Brno, Czech Republic.
EM pikulaj@vfu.cz; hegert4@gmail.com
RI Piaček, Vladimír/AAI-1231-2020; Kovacova, Veronika/V-1815-2019;
   Bandouchova, Hana/AAI-1213-2020; Divinova, Renata/KBA-8297-2024;
Pikula,
   Jiri/V-8620-2018; Zukal, Jan/G-1180-2014
OI Bandouchova, Hana/0000-0003-0373-7940; Divinova,
   Renata/0000-0001-8541-5536; Pikula, Jiri/0000-0001-8747-9365;
Zukal,
   Jan/0000-0003-4967-6880
FU Czech Science Foundation [17-20286S]
FX This study was supported by the Czech Science Foundation (Grant
No.
   17-20286S). The funder had no role in the study design, data
analysis,
   and decision to publish, or preparation of the manuscript.
CR Allen LC, 2009, J COMP PHYSIOL B, V179, P315, DOI 10.1007/
s00360-008-0315-3
   Andrews MT, 2007, BIOESSAYS, V29, P431, DOI 10.1002/bies.20560
   Babior BM, 2000, AM J MED, V109, P33, DOI 10.1016/
S0002-9343(00)00481-2
   Baker ML, 2013, ZOONOSES PUBLIC HLTH, V60, P104, DOI 10.1111/
j.1863-2378.2012.01528.x
   Bandouchova H, 2018, SCI REP-UK, V8, DOI 10.1038/
s41598-018-24461-5
   Bartonicka T, 2017, J THERM BIOL, V63, P119, DOI 10.1016/
j.jtherbio.2016.12.005
   Becker DJ, 2020, J ANIM ECOL, V89, P972, DOI
10.1111/1365-2656.13166
   Bergman A, 2010, MBIO, V1, DOI 10.1128/mBio.00212-10
   Blehert DS, 2009, SCIENCE, V323, P227, DOI 10.1126/
science.1163874
   Boratynski JS, 2012, ACTA CHIROPTEROL, V14, P417, DOI
10.3161/150811012X661738
   Bouma HR, 2013, J LEUKOCYTE BIOL, V94, P431, DOI 10.1189/
jlb.0611298
   Bouma HR, 2010, J LEUKOCYTE BIOL, V88, P619, DOI 10.1189/
jlb.0310174
   Boyer BB, 1999, BIOSCIENCE, V49, P713, DOI 10.2307/1313595
   Brook CE, 2015, TRENDS MICROBIOL, V23, P172, DOI 10.1016/
j.tim.2014.12.004
   Calisher CH, 2006, CLIN MICROBIOL REV, V19, P531, DOI 10.1128/
CMR.00017-06
   Canale CI, 2011, FUNCT ECOL, V25, P557, DOI 10.1111/
j.1365-2435.2010.01815.x
   Carey HV, 2003, PHYSIOL REV, V83, P1153, DOI 10.1152/
physrev.00008.2003
   Casadevall A, 1999, INFECT IMMUN, V67, P3703, DOI 10.1128/
IAI.67.8.3703-3713.1999
   Chapman ALP, 2002, J BIOL CHEM, V277, P9757, DOI 10.1074/
jbc.M106134200
   Cichon M, 2002, P ROY SOC B-BIOL SCI, V269, P1493, DOI 10.1098/

rspb.2002.2054
Costantini D, 2009, COMP BIOCHEM PHYS A, V153, P339, DOI 10.1016/j.cbpa.2009.03.010
Cryan PM, 2013, J WILDLIFE DIS, V49, P398, DOI 10.7589/2012-04-121
Dahlgren C, 1999, J IMMUNOL METHODS, V232, P3, DOI 10.1016/S0022-1759(99)00146-5
Dietz M, 2006, J COMP PHYSIOL B, V176, P223, DOI 10.1007/s00360-005-0043-x
Field KA, 2018, MOL ECOL, V27, P3727, DOI 10.1111/mec.14827
Flieger M, 2016, SCI REP-UK, V6, DOI 10.1038/srep33200
Gaisler J., 2003, VESPERTILIO, V7, P3
Gaut JP, 2001, P NATL ACAD SCI USA, V98, P11961, DOI 10.1073/pnas.211190298
Geiser F, 2011, HIBERNATION ENDOTHER
GLETTE J, 1982, ACTA PATH MICRO IM C, V90, P91
Hawley DM, 2012, FUNCT ECOL, V26, P701, DOI 10.1111/j.1365-2435.2012.01978.x
He XC, 2014, PLOS ONE, V9, DOI 10.1371/journal.pone.0105373
Heard DJ, 1998, J ZOO WILDLIFE MED, V29, P14
Hecht AM, 2015, SCI REP-UK, V5, DOI 10.1038/srep16604
Heger T, 2020, ACTA VET BRNO, V89, P79, DOI 10.2754/avb202089010079
Kacprzyk J, 2017, ACTA CHIROPTEROL, V19, P219, DOI [10.3161/15081109ACC2017.19.2.001, 10.3161/15081109acc2017.19.2.001]
Kokurewicz VT., 2019, Nyctalus, V19, P252
Kruger P, 2015, PLOS PATHOG, V11, DOI 10.1371/journal.ppat.1004651
Linhart P, 2020, ACTA VET BRNO, V89, P69, DOI 10.2754/avb202089010069
Lojek A, 1997, J BIOLUM CHEMILUM, V12, P225, DOI 10.1002/(SICI)1099-1271(199709/10)12:5<225::AID-BIO448>3.0.CO;2-X
Luis AD, 2006, FUNCT ECOL, V20, P471, DOI 10.1111/j.1365-2435.2006.01119.x
MAINA JN, 1984, J EXP BIOL, V111, P43
Mandl JN, 2018, FRONT IMMUNOL, V9, DOI 10.3389/fimmu.2018.02112
Mandl JN, 2015, CELL, V160, P20, DOI 10.1016/j.cell.2014.12.003
MARNILA P, 1995, COMP BIOCHEM PHYS A, V111, P609, DOI 10.1016/0300-9629(95)00054-B
Martínková N, 2019, J THEOR BIOL, V464, P98, DOI 10.1016/j.jtbi.2018.12.026
Martínková N, 2018, VIRULENCE, V9, P1734, DOI 10.1080/21505594.2018.1548685
Mayberry HW, 2018, J COMP PHYSIOL B, V188, P333, DOI 10.1007/s00360-017-1119-0
Medzhitov R, 2012, SCIENCE, V335, P936, DOI 10.1126/science.1214935
Meteyer CU, 2012, VIRULENCE, V3, P583, DOI 10.4161/viru.22330
Moore MS, 2011, PLOS ONE, V6, DOI 10.1371/journal.pone.0027430
MUSACCHIA XJ, 1971, AM J PHYSIOL, V221, P128, DOI 10.1152/ajplegacy.1971.221.1.128
Papezíková I, 2016, FISH SHELLFISH IMMUN, V57, P400, DOI 10.1016/j.fsi.2016.08.048
Parasuraman S, 2010, J PHARMACOL PHARMACO, V1, P87, DOI

10.4103/0976-500X.72350
Perry RW, 2013, ENVIRON REV, V21, P28, DOI 10.1139/er-2012-0042
Pikula J, 2017, PLOS ONE, V12, DOI 10.1371/journal.pone.0180435
Pikula Jiri, 2017, Veterinary Clinics of North America Exotic Animal Practice, V20, P665, DOI 10.1016/j.cvex.2016.11.013
Rodríguez-Durán A, 2008, CARIBB J SCI, V44, P375
Rosales C, 2017, BIOMED RES INT-UK, V2017, DOI 10.1155/2017/9042851
Roy VK, 2013, J EXP ZOOL PART A, V319, P517, DOI 10.1002/jez.1815
Ruczynski I, 2005, J MAMMAL, V86, P921, DOI 10.1644/1545-1542(2005)86[921:RCSBNN]2.0.CO;2
Smith LM, 2013, BIOCHEM SOC T, V41, P475, DOI 10.1042/BST20130014
Strelkov PP, 1969, ACTA ZOOL CRAC, V14, P93
Strobel S, 2015, MAMM BIOL, V80, P312, DOI 10.1016/j.mambio.2015.02.005
Thomas DC, 2017, IMMUNOL LETT, V192, P88, DOI 10.1016/j.imlet.2017.08.016
Venables W.N., 2002, Modern Applied Statistics with S, DOI [DOI 10.1007/978-0-387-21706-2, 10.1007/978-0-387-21706-2]
Vitula F, 2011, BMC VET RES, V7, DOI 10.1186/1746-6148-7-34
Voyles J, 2007, DIS AQUAT ORGAN, V77, P113, DOI 10.3354/dao01838
Wang LF, 2011, CURR OPIN VIROL, V1, P649, DOI 10.1016/j.coviro.2011.10.013
Wang MC, 2016, ENERG BUILDINGS, V127, P313, DOI 10.1016/j.enbuild.2016.05.065
Webb PI, 1996, CAN J ZOOL, V74, P761, DOI 10.1139/z96-087
Willis CKR, 2017, INTEGR COMP BIOL, V57, P1214, DOI 10.1093/icb/icx087
WINTERBOURN CC, 1987, J CLIN INVEST, V80, P1486, DOI 10.1172/JCI113230
Wojciechowski MS, 2007, COMP BIOCHEM PHYS A, V147, P828, DOI 10.1016/j.cbpa.2006.06.039
Xu YC, 2013, BMC GENOMICS, V14, DOI 10.1186/1471-2164-14-567
Zukal J, 2005, MAMM BIOL, V70, P271, DOI 10.1016/j.mambio.2005.03.003
Zukal J, 2016, SCI REP-UK, V6, DOI 10.1038/srep19829
NR 77
TC 10
Z9 10
U1 0
U2 13
PU BMC
PI LONDON
PA CAMPUS, 4 CRINAN ST, LONDON N1 9XW, ENGLAND
EI 1746-6148
J9 BMC VET RES
JI BMC Vet. Res.
PD JUL 6
PY 2020
VL 16
IS 1
AR 232
DI 10.1186/s12917-020-02450-z
PG 13

WC Veterinary Sciences
WE Science Citation Index Expanded (SCI-EXPANDED)
SC Veterinary Sciences
GA MM2SO
UT WOS:000550006900003
PM 32631329
OA Green Published, gold
DA 2024-10-21
ER

PT J
AU Friedrichs, V
Toussaint, C
Schäfer, A
Rissmann, M
Dietrich, O
Mettenleiter, TC
Pei, G
Balkema-Buschmann, A
Saliba, AE
Dorhoi, A
AF Friedrichs, Virginia
Toussaint, Christophe
Schaefer, Alexander
Rissmann, Melanie
Dietrich, Oliver
Mettenleiter, Thomas C.
Pei, Gang
Balkema-Buschmann, Anne
Saliba, Antoine-Emmanuel
Dorhoi, Anca
TI Landscape and age dynamics of immune cells in the Egyptian rousette bat
SO CELL REPORTS
LA English
DT Article
ID MARBURG HEMORRHAGIC-FEVER; RESERVOIR HOSTS; VIRUS-INFECTION; PTEROPID
BATS; FRUIT BATS; B-CELLS; EXPRESSION; ACTIVATION; RESPONSES; MEMORY
AB Bats harbor high-impact zoonotic viruses often in the absence of disease manifestation. This restriction and disease tolerance possibly rely on specific immunological features. In-depth molecular characterization of cellular immunity and imprinting of age on leukocyte compartments remained unexplored in bats. We employ single-cell RNA sequencing (scRNA-seq) and establish immunostaining panels to characterize the immune cell landscape in juvenile, subadult, and adult Egyptian rousette bats (ERBs). Transcriptomic and flow cytometry data reveal conserved subsets and substantial enrichments of CD79a(+)B cells and CD11b(+) T cells in juvenile animals, whereas neutrophils, CD206(+) myeloid cells, and CD3(+)T cells dominate as bats reach adult-hood. Despite differing frequencies, phagocytosis of circulating and tissue-resident myeloid cells and proliferation of peripheral and splenic lymphocytes are

analogous in juvenile and adult ERBs. We provide a comprehensive map
of the immune landscape in ERBs and show age-imprinted resilience
progression and find that variability in cellular immunity only
partly recapitulates mammalian archetypes.
C1 [Friedrichs, Virginia; Schaefer, Alexander; Pei, Gang; Dorhoi,
Anca] Friedrich Loeffler Inst, Inst Immunol, Greifswald, Germany.
[Toussaint, Christophe; Dietrich, Oliver; Saliba, Antoine-
Emmanuel] Helmholtz Ctr Infect Res HZI, Inst RNA Based Infect Res
HIRI, Wurzburg, Germany.
[Rissmann, Melanie; Balkema-Buschmann, Anne] Friedrich Loeffler
Inst, Inst Novel & Emerging Infect Dis, Greifswald, Germany.
[Mettenleiter, Thomas C.] Friedrich Loeffler Inst, Greifswald,
Germany.
[Dorhoi, Anca] Univ Greifswald, Fac Math & Nat Sci, Greifswald,
Germany.
[Friedrichs, Virginia] Friedrich Loeffler Inst, Inst Diagnost
Virol, Greifswald, Germany.
[Rissmann, Melanie] Erasmus MC, Rotterdam, Zuid Holland,
Netherlands.
C3 Friedrich Loeffler Institute; Helmholtz Association; Helmholtz-
Center
for Infection Research; Friedrich Loeffler Institute; Friedrich
Loeffler
Institute; Universitat Greifswald; Friedrich Loeffler Institute;
Erasmus
University Rotterdam; Erasmus MC
RP Dorhoi, A (corresponding author), Friedrich Loeffler Inst, Inst
Immunol, Greifswald, Germany.; Saliba, AE (corresponding author),
Helmholtz Ctr Infect Res HZI, Inst RNA Based Infect Res HIRI,
Wurzburg, Germany.; Dorhoi, A (corresponding author), Univ
Greifswald, Fac Math & Nat Sci, Greifswald, Germany.
EM emmanuel.saliba@helmholtz-hiri.de; anca.dorhoi@fli.de
RI Rissmann, Melanie/AAZ-6477-2021; Saliba, Antoine-Emmanuel/
AAC-1252-2022;
Mettenleiter, h./U-1016-2019; Pei, Gang/U-4740-2017; Schafer,
Alexander/I-8853-2018
OI Balkema-Buschmann, Anne/0000-0001-7613-9592; Toussaint,
Christophe/0000-0003-3419-490X; Pei, Gang/0000-0001-5517-8496;
Dorhoi,
Anca/0000-0003-1739-749X; Schafer, Alexander/0000-0002-4258-0651
FU Initialisierung- und Vernetzungsfonds furInfektionsforschung
Greifswald,
an initiative of Friedrich-Loeffler-Institut,Helmholtz Center for
Infection Research; Bundesministerium fur Bildung und For-schung
(BMBF)
[031L0289B]; University of Greifswald
FX This study was funded by intramural funds from the
Friedrich-Loeffler-Institutand was financially supported by
Initialisierung- und Vernetzungsfonds furInfektionsforschung
Greifswald,
an initiative of Friedrich-Loeffler-Institut,Helmholtz Center for
Infection Research and University of Greifswald, ProjectImmubat.
A.E.S.
and C.T. thank Bundesministerium fur Bildung und For-schung

(BMBF) for
support via the grant HOPARL (031L0289B). We thankMarcel Bokelmann for
assistance with blood sample collection as well as SilkeRehbein and Lisa
Loerzer for excellent technical assistance and all animalcaretakers for
maintaining ideal standards for the bat colony. We further
thankAlexander Leipold for supporting data analysis
CR Abolins S, 2017, NAT COMMUN, V8, DOI 10.1038/ncomms14811
Ahn M, 2019, NAT MICROBIOL, V4, P789, DOI 10.1038/s41564-019-0371-3
Ahn M, 2016, SCI REP-UK, V6, DOI 10.1038/srep21722
Amman BR, 2012, PLOS PATHOG, V8, DOI 10.1371/journal.ppat.1002877
Avery P, 2014, CURR VASC PHARMACOL, V12, P659, DOI 10.2174/1570161111666131219101226
Baker ML, 2013, ZOONOSES PUBLIC HLTH, V60, P104, DOI 10.1111/j.1863-2378.2012.01528.x
Banerjee A, 2020, FRONT IMMUNOL, V11, DOI 10.3389/fimmu.2020.00026
Bausch DG, 2006, NEW ENGL J MED, V355, P909, DOI 10.1056/NEJMoa051465
Becht E, 2019, NAT BIOTECHNOL, V37, P38, DOI 10.1038/nbt.4314
Becker DJ, 2019, INTEGR COMP BIOL, V59, P1176, DOI 10.1093/icb/icz007
Bondet V, 2021, FRONT IMMUNOL, V12, DOI 10.3389/fimmu.2021.735866
Büttner M, 2021, NAT COMMUN, V12, DOI 10.1038/s41467-021-27150-6
Burny W, 2017, FRONT IMMUNOL, V8, P14, DOI 10.3389/fimmu.2017.00943
Butcher SK, 2001, J LEUKOCYTE BIOL, V70, P881
CHAKRAVARTY AK, 1987, DEV COMP IMMUNOL, V11, P649, DOI 10.1016/0145-305X(87)90053-X
CHAKRAVARTY AK, 1994, LYMPHOLOGY, V27, P97
Channathodiyil P, 2021, PLOS ONE, V16, DOI 10.1371/journal.pone.0240769
Christensen JE, 2001, INT IMMUNOL, V13, P593, DOI 10.1093/intimm/13.4.593
Donath A, 2019, NUCLEIC ACIDS RES, V47, P10543, DOI 10.1093/nar/gkz833
Dutertre CA, 2019, IMMUNITY, V51, P573, DOI 10.1016/j.immuni.2019.08.008
Edson D, 2019, EPIDEMIOL INFECT, V147, P1, DOI [10.1017/S0950268819001237, 10.1017/s0950268819001237]
Gamage AM, 2020, SCI REP-UK, V10, DOI 10.1038/s41598-019-57212-1
Geirsdottir L, 2019, CELL, V179, P1609, DOI 10.1016/j.cell.2019.11.010
Goh G, 2020, P NATL ACAD SCI USA, V117, P28939, DOI 10.1073/pnas.2003352117
Gómez JMM, 2016, SCI REP-UK, V6, DOI 10.1038/srep37796
Guito JC, 2021, CURR BIOL, V31, P257, DOI 10.1016/j.cub.2020.10.015
Gupta-Wright A, 2017, FRONT IMMUNOL, V8, DOI 10.3389/fimmu.2017.01222
Halpin K, 2011, AM J TROP MED HYG, V85, P946, DOI 10.4269/

ajtmh.2011.10-0567
HATHCOCK KS, 1993, J IMMUNOL, V151, P6712
Haug T, 2019, FRONT IMMUNOL, V10, DOI 10.3389/fimmu.2019.00883
He X, 2014, J VIROL, V88, P5444, DOI 10.1128/JVI.03403-13
Hearps AC, 2012, AGING CELL, V11, P867, DOI 10.1111/j.1474-9726.2012.00851.x
Heaton H, 2020, NAT METHODS, V17, P615, DOI 10.1038/s41592-020-0820-1
Herrera-Uribe J, 2021, FRONT GENET, V12, DOI 10.3389/fgene.2021.689406
Hilton HG, 2019, PLOS BIOL, V17, DOI 10.1371/journal.pbio.3000528
Ince LM, 2019, FRONT IMMUNOL, V9, DOI 10.3389/fimmu.2018.03143
Irving AT, 2021, NATURE, V589, P363, DOI 10.1038/s41586-020-03128-0
Jacques Dainat D.H, 2021, NBISWEDENAGAT AGAT V
Jebb D, 2020, NATURE, V583, P578, DOI 10.1038/s41586-020-2486-3
Kandeil A, 2019, J VIROL, V93, DOI 10.1128/JVI.01059-18
Keresztes M, 2007, STRESS, V10, P271, DOI 10.1080/10253890701248079
Kingston Tigga, 2016, P539
Kollmann TR, 2017, IMMUNITY, V46, P350, DOI 10.1016/j.immuni.2017.03.009
Kunz T.H., 1982, P1
La Manno G, 2016, CELL, V167, P566, DOI 10.1016/j.cell.2016.09.027
Larson PA, 2021, CELL REP, V35, DOI 10.1016/j.celrep.2021.109140
Lee AK, 2015, BMC GENOMICS, V16, DOI 10.1186/s12864-015-2124-x
MARKOVIC SN, 1993, ANESTHESIOLOGY, V78, P700, DOI 10.1097/00000542-199304000-00013
MCFARLAND HI, 1992, J IMMUNOL, V149, P1326
Middleton DJ, 2007, J COMP PATHOL, V136, P266, DOI 10.1016/j.jcpa.2007.03.002
Moore PR, 2010, ANN TROP MED PARASIT, V104, P613, DOI 10.1179/136485910X12851868779948
Munster VJ, 2016, SCI REP-UK, V6, DOI 10.1038/srep21878
Nowak RM, 1994, WALKERS BATS WORLD
Papenfuss AT, 2012, BMC GENOMICS, V13, DOI 10.1186/1471-2164-13-261
Park CK, 1999, J KOREAN MED SCI, V14, P600, DOI 10.3346/jkms.1999.14.6.600
Patel RS, 2021, BMC BIOL, V19, DOI 10.1186/s12915-020-00947-5
Pavlovich SS, 2018, CELL, V173, P1098, DOI 10.1016/j.cell.2018.03.070
Paweska JT, 2016, VIRUSES-BASEL, V8, DOI 10.3390/v8020029
Periasamy P, 2019, FRONT IMMUNOL, V10, DOI 10.3389/fimmu.2019.00489
Piatosa B, 2010, CYTOM PART B-CLIN CY, V78B, P372, DOI 10.1002/cyto.b.20536
Plowden J, 2004, AGING CELL, V3, P161, DOI 10.1111/j.1474-9728.2004.00102.x
Ren L, 2020, bioRxiv, DOI [10.1101/2020.06.30.175778, 10.1101/2020.06.30.175778, DOI 10.1101/2020.06.30.175778]
SARKAR SK, 1991, DEV COMP IMMUNOL, V15, P423, DOI 10.1016/0145-305X(91)90034-V

Satija R, 2015, NAT BIOTECHNOL, V33, P495, DOI 10.1038/nbt.3192
Simell B, 2011, VACCINE, V29, P1929, DOI 10.1016/j.vaccine.2010.12.121
Simon AK, 2015, P ROY SOC B-BIOL SCI, V282, DOI 10.1098/rspb.2014.3085
Stewart A, 2021, FRONT IMMUNOL, V12, DOI 10.3389/fimmu.2021.602539
Storm N, 2018, VIRUSES-BASEL, V10, DOI 10.3390/v10020073
Stuart T, 2019, CELL, V177, P1888, DOI 10.1016/j.cell.2019.05.031
Swanepoel R, 2007, EMERG INFECT DIS, V13, P1847, DOI 10.3201/eid1312.071115
Timen A, 2009, EMERG INFECT DIS, V15, P1171, DOI [10.3201/eid1508.090051, 10.3201/eid1508.090015]
Towner JS, 2007, PLOS ONE, V2, DOI 10.1371/journal.pone.0000764
Towner JS, 2009, PLOS PATHOG, V5, DOI 10.1371/journal.ppat.1000536
Traag VA, 2019, SCI REP-UK, V9, DOI 10.1038/s41598-019-41695-z
Valiathan R, 2016, SCAND J IMMUNOL, V83, P255, DOI 10.1111/sji.12413
VANDERWESTHUYZEN J, 1988, COMP BIOCHEM PHYS A, V90, P117, DOI 10.1016/0300-9629(88)91015-8
Vidgen ME, 2015, J GEN VIROL, V96, P1619, DOI 10.1099/vir.0.000099
Wang LF, 2021, NAT REV IMMUNOL, V21, P269, DOI 10.1038/s41577-021-00523-0
Watanabe S, 2010, EMERG INFECT DIS, V16, P1217, DOI 10.3201/eid1608.100208
WATT SM, 1980, J HISTOCHEM CYTOCHEM, V28, P934, DOI 10.1177/28.9.7410816
Wein AN, 2019, J EXP MED, V216, P2748, DOI 10.1084/jem.20181308
Wenisch C, 2000, J LEUKOCYTE BIOL, V67, P40, DOI 10.1002/jlb.67.1.40
Williamson MM, 1998, AUST VET J, V76, P813, DOI 10.1111/j.1751-0813.1998.tb12335.x
Williamson MM, 2000, J COMP PATHOL, V122, P201, DOI 10.1053/jcpa.1999.0364
Xie JZ, 2018, CELL HOST MICROBE, V23, P297, DOI 10.1016/j.chom.2018.01.006
Zhang GJ, 2013, SCIENCE, V339, P456, DOI 10.1126/science.1230835
Zhou P, 2016, SCI REP-UK, V6, DOI 10.1038/srep38597
Zhou P, 2016, P NATL ACAD SCI USA, V113, P2696, DOI 10.1073/pnas.1518240113
NR 88
TC 18
Z9 19
U1 1
U2 6
PU CELL PRESS
PI CAMBRIDGE
PA 50 HAMPSHIRE ST, FLOOR 5, CAMBRIDGE, MA 02139 USA
SN 2211-1247
J9 CELL REP
JI Cell Reports
PD SEP 6

PY 2022
VL 40
IS 10
AR 111305
DI 10.1016/j.celrep.2022.111305
EA SEP 2022
PG 24
WC Cell Biology
WE Science Citation Index Expanded (SCI-EXPANDED)
SC Cell Biology
GA 5B0YA
UT WOS:000863301600006
PM 36070695
OA gold
DA 2024-10-21
ER

PT J
AU Warburton, EM
   Pearl, CA
   Vonhof, MJ
AF Warburton, Elizabeth M.
   Pearl, Christopher A.
   Vonhof, Maarten J.
TI Relationships between host body condition and immunocompetence, not host
   sex, best predict parasite burden in a bat-helminth system
SO PARASITOLOGY RESEARCH
LA English
DT Article
DE Sex-biased parasitism; Helminthiasis; Eptesicus fuscus;
   Immunocompetence; Body condition
ID MALE-BIASED PARASITISM; EPTESICUS-FUSCUS; IMMUNE DEFENSE; TRADE-OFFS;
   ECOLOGICAL IMMUNOLOGY; SOCIAL-STRUCTURE; SEASONAL-CHANGES; COSTS;
   TESTOSTERONE; POPULATIONS
AB Sex-biased parasitism highlights potentially divergent approaches to parasite resistance resulting in differing energetic trade-offs for males and females; however, trade-offs between immunity and self-maintenance could also depend on host body condition. We investigated these relationships in the big brown bat, Eptesicus fuscus, to determine if host sex or body condition better predicted parasite resistance, if testosterone levels predicted male parasite burdens, and if immune parameters could predict male testosterone levels. We found that male and female hosts had similar parasite burdens and female bats scored higher than males in only one immunological measure. Top models of helminth burden revealed interactions between body condition index and agglutination score as well as between agglutination score and host sex. Additionally, the strength of the relationships between sex, agglutination, and helminth burden is affected by body condition. Models of male parasite burden provided no support for testosterone predicting helminthiasis. Models that best predicted testosterone levels did not include parasite burden but instead consistently included month

of capture and agglutination score. Thus, in our system, body condition was a more important predictor of immunity and worm burden than host sex.
C1 [Warburton, Elizabeth M.; Pearl, Christopher A.; Vonhof, Maarten J.] Western Michigan Univ, Dept Biol Sci, 1903 West Michigan Ave, Kalamazoo, MI 49008 USA.
[Vonhof, Maarten J.] Western Michigan Univ, Environm & Sustainabil Studies Program, 1903 West Michigan Ave, Kalamazoo, MI 49008 USA.
[Pearl, Christopher A.] Grand Valley State Univ, Dept Biomed Sci, 1 Campus Dr, Allendale, MI 49401 USA.
C3 Western Michigan University; Western Michigan University; Grand Valley
State University
RP Warburton, EM (corresponding author), Western Michigan Univ, Dept Biol Sci, 1903 West Michigan Ave, Kalamazoo, MI 49008 USA.
EM elizabeth.m.warburton@wmich.edu
OI Warburton, Elizabeth/0000-0001-8125-2741
FU American Society of Mammalogists; American Society of Parasitologists;
Annual Midwestern Conference of Parasitologists; Western Michigan University
FX The authors would like to thank J.W. Warburton, C.J. Warburton, D.J.
Clarke, B.A. Hines, S.M. Warner, D.M. Courtney, B.K. Hubbard, L.M.
Vanbladeren, H.E. LaFore, and E.M. Freed for their assistance in the
field and in the lab. We appreciate the assistance of F.A. Jimenez in
nematode identification. We would also like to thank J. Glatz for
creating a map of our sites. Finally, we thank S.L. Kohler, S.A. Gill,
and J.M. Lotz as well as two anonymous reviewers for their constructive
comments on earlier versions of the manuscript. Funding was provided by
the American Society of Mammalogists, the American Society of
Parasitologists, the Annual Midwestern Conference of Parasitologists,
and Western Michigan University.
CR Allen DE, 2011, J EVOLUTION BIOL, V24, P224, DOI 10.1111/j.1420-9101.2010.02152.x
Anderson R. C., 2009, Keys to the nematode parasites of vertebrates: archival volume, P30, DOI 10.1079/9781845935726.0030
Barnett CA, 2015, J ZOOL, V296, P1, DOI 10.1111/jzo.12213
Biard C, 2015, METHODS ECOL EVOL, V6, P960, DOI 10.1111/2041-210X.12371
BLANKESPOOR H D, 1970, Proceedings of the Iowa Academy of Science, V77, P200
Boonekamp JJ, 2008, BIOL LETTERS, V4, P741, DOI 10.1098/rsbl.2008.0347
Bordes F, 2012, OECOLOGIA, V170, P123, DOI 10.1007/s00442-012-2300-5

Boyden S V, 1966, Adv Immunol, V5, P1
Bush AO, 1997, J PARASITOL, V83, P575, DOI 10.2307/3284227
Cai XQ, 2009, ZOOLOGY, V112, P69, DOI 10.1016/j.zool.2008.04.006
Chen J, 2009, J AUTOIMMUN, V32, P195, DOI 10.1016/j.jaut.2009.02.021
COGGINS JR, 1982, AM MIDL NAT, V107, P305, DOI 10.2307/2425381
Czaplinski B., 1994, P595
Dare OK, 2009, J PARASITOL, V95, P275, DOI 10.1645/GE-1713.1
Davis AK, 2008, FUNCT ECOL, V22, P760, DOI 10.1111/j.1365-2435.2008.01467.x
Degen AA, 2006, MICROMAMMALS AND MACROPARASITES: FROM EVOLUTIONARY ECOLOGY TO MANAGEMENT, P371, DOI 10.1007/978-4-431-36025-4_19
Downs CJ, 2013, P ROY SOC B-BIOL SCI, V280, DOI 10.1098/rspb.2012.2636
Ferrari N, 2004, ECOL LETT, V7, P88, DOI 10.1046/j.1461-0248.2003.00552.x
Field A., 2013, DISCOVERING STAT USI
FOLSTAD I, 1992, AM NAT, V139, P603, DOI 10.1086/285346
Forbes MR, 2007, TRENDS ECOL EVOL, V22, P111, DOI 10.1016/j.tree.2006.12.004
Forsman AM, 2008, J EVOLUTION BIOL, V21, P873, DOI 10.1111/j.1420-9101.2008.01503.x
Friesen OC, 2015, J MAMMAL, V96, P417, DOI 10.1093/jmammal/gyv043
Fujita T, 2004, IMMUNOL REV, V198, P185, DOI 10.1111/j.0105-2896.2004.0123.x
Gasque P, 2004, MOL IMMUNOL, V41, P1089, DOI 10.1016/j.molimm.2004.06.011
Giacomin PR, 2004, J IMMUNOL METHODS, V289, P201, DOI 10.1016/j.jim.2004.04.024
Gooderham K, 2011, BEHAV ECOL, V22, P1195, DOI 10.1093/beheco/arr112
GRINEVITCH L, 1995, J ZOOL, V235, P301, DOI 10.1111/j.1469-7998.1995.tb05146.x
Guerrero R, 2002, COMP PARASITOL, V69, P177, DOI 10.1654/1525-2647(2002)069[0177:NAKSOL]2.0.CO;2
Hasu T, 2006, OIKOS, V114, P322, DOI 10.1111/j.2006.0030-1299.14726.x
Hayes A. F., 2013, Introduction to Mediation, Moderation, and Conditional Process Analysis: A Regression-Based Approach, V51, P335
Hepworth MR, 2010, EUR J IMMUNOL, V40, P406, DOI 10.1002/eji.200939589
Hillegass MA, 2008, BEHAV ECOL, V19, P1006, DOI 10.1093/beheco/arn070
Honkavaara J, 2009, ENTOMOL EXP APPL, V132, P165, DOI 10.1111/j.1570-7458.2009.00877.x
Khan MN, 2010, PARASITOL RES, V107, P787, DOI 10.1007/s00436-010-1931-x
Kiffner C, 2013, OECOLOGIA, V173, P1009, DOI 10.1007/s00442-013-2664-1
Krasnov BR, 2012, MAMMALIA, V76, P1, DOI 10.1515/mammalia-2011-0108
Kumar V, 1999, LECITHODENDRIID INFE, P363
KUNZ TH, 1982, J MAMMAL, V63, P23, DOI 10.2307/1380667

KURTA A, 1990, J MAMMAL, V71, P59, DOI 10.2307/1381316
KURTA A, 1990, Mammalian Species, P1, DOI 10.2307/3504258
Lajeunesse MJ, 2004, OIKOS, V106, P501, DOI 10.1111/j.0030-1299.2004.13076.x
Lee KA, 2008, J ANIM ECOL, V77, P356, DOI 10.1111/j.1365-2656.2007.01347.x
Lochmiller RL, 2000, OIKOS, V88, P87, DOI 10.1034/j.1600-0706.2000.880110.x
Lotz J. M., 2008, Keys to the Trematoda, Volume 3, P527, DOI 10.1079/9780851995885.0527
LOTZ JM, 1983, P HELM SOC WASH, V50, P83
LOTZ JM, 1985, CAN J ZOOL, V63, P2969, DOI 10.1139/z85-445
MacIntosh AJJ, 2010, PRIMATES, V51, P353, DOI 10.1007/s10329-010-0211-9
Mallon EB, 2003, EVOLUTION, V57, P1444
Martin LB, 2006, FUNCT ECOL, V20, P290, DOI 10.1111/j.1365-2435.2006.01094.x
Martin LB, 2008, PHILOS T R SOC B, V363, P321, DOI 10.1098/rstb.2007.2142
Marzal A, 2007, BEHAV ECOL SOCIOBIOL, V61, P1573, DOI 10.1007/s00265-007-0389-z
Matson KD, 2005, DEV COMP IMMUNOL, V29, P275, DOI 10.1016/j.dci.2004.07.006
Mazerolle MJ, 2006, AMPHIBIA-REPTILIA, V27, P169, DOI 10.1163/156853806777239922
McAllister CT, 2009, COMP PARASITOL, V76, P117, DOI 10.1654/4354.1
McCurdy DG, 1998, OIKOS, V82, P303, DOI 10.2307/3546970
Meeusen ENT, 2000, PARASITOL TODAY, V16, P95, DOI 10.1016/S0169-4758(99)01607-5
Mendonca MT, 1996, HORM BEHAV, V30, P153, DOI 10.1006/hbeh.1996.0019
Mills SC, 2010, EVOLUTION, V64, P166, DOI 10.1111/j.1558-5646.2009.00820.x
Mills SC, 2009, AM NAT, V173, P475, DOI 10.1086/597222
Moore SL, 2002, SCIENCE, V297, P2015, DOI 10.1126/science.1074196
Moreno-Rueda G, 2011, ECOL RES, V26, P943, DOI 10.1007/s11284-011-0848-x
Muehlenbein MP, 2010, AM J HUM BIOL, V22, P546, DOI 10.1002/ajhb.21045
Nakazawa M, 1997, EXP PARASITOL, V85, P233, DOI 10.1006/expr.1997.4148
NICKEL PA, 1967, AM MIDL NAT, V78, P481, DOI 10.2307/2485245
Norris K, 2000, BEHAV ECOL, V11, P19, DOI 10.1093/beheco/11.1.19
Nunn CL, 2009, PHILOS T R SOC B, V364, P61, DOI 10.1098/rstb.2008.0148
Palacios MG, 2012, PHYSIOL BIOCHEM ZOOL, V85, P1, DOI 10.1086/663311
Pearce RD, 2008, ACTA CHIROPTEROL, V10, P153, DOI 10.3161/150811008X331171
Perkins SE, 2008, PARASITOLOGY, V135, P1561, DOI 10.1017/S0031182008000449
PISTOLE DH, 1988, P HELM SOC WASH, V55, P270
Porteous IS, 1998, PARASITOLOGY, V116, P269, DOI 10.1017/

S0031182097002205
Poulin R, 1996, INT J PARASITOL, V26, P1311, DOI 10.1016/S0020-7519(96)00108-7
RAUSCH RL, 1975, CAN J ZOOL, V53, P1537, DOI 10.1139/z75-189
Roberts ML, 2004, ANIM BEHAV, V68, P227, DOI 10.1016/j.anbehav.2004.05.001
Robinson SA, 2008, J AVIAN BIOL, V39, P272, DOI 10.1111/j.0908-8857.2008.04340.x
Robinson SA, 2010, WATERBIRDS, V33, P307, DOI 10.1675/063.033.0306
Rolff J, 2002, P ROY SOC B-BIOL SCI, V269, P867, DOI 10.1098/rspb.2002.1959
Rossin MA, 2010, PARASITOLOGY, V137, P1569, DOI 10.1017/S0031182010000351
Russo J, 2013, PHYSIOL BIOCHEM ZOOL, V86, P193, DOI 10.1086/669482
Schalk G, 1997, OIKOS, V78, P67, DOI 10.2307/3545801
Schell S.C., 1985, HDB TREMATODES N AM
Schmid-Hempel P, 2003, TRENDS ECOL EVOL, V18, P27, DOI 10.1016/S0169-5347(02)00013-7
Schmid-Hempel P, 2003, P ROY SOC B-BIOL SCI, V270, P357, DOI 10.1098/rspb.2002.2265
Schroderus E, 2010, AM NAT, V176, pE90, DOI 10.1086/656264
Schulte-Hostedde AI, 2005, ECOLOGY, V86, P155, DOI 10.1890/04-0232
Sheldon BC, 1996, TRENDS ECOL EVOL, V11, P317, DOI 10.1016/0169-5347(96)10039-2
Stoehr AM, 2006, BEHAV ECOL, V17, P751, DOI 10.1093/beheco/ark018
Superina M, 2008, J WILDLIFE DIS, V44, P902, DOI 10.7589/0090-3558-44.4.902
Tälleklint-Eisen L, 1999, EXP APPL ACAROL, V23, P731, DOI 10.1023/A:1006212323760
Tkach VV, 2000, SYST PARASITOL, V47, P9, DOI 10.1023/A:1006358524045
Tkach VV, 2008, KEYS TREMATODA, P296
Ujvari B, 2006, BEHAV ECOL, V17, P20, DOI 10.1093/beheco/ari091
Waterman JM, 2014, CAN J ZOOL, V92, P73, DOI 10.1139/cjz-2013-0151
WILLIAMS RUSSELL R., 1960, OHIO JOUR SCI, V60, P323
Zuk M, 1996, INT J PARASITOL, V26, P1009, DOI [10.1016/S0020-7519(96)00086-0, 10.1016/S0020-7519(96)80001-4]
Zuk M, 2009, PLOS PATHOG, V5, DOI 10.1371/journal.ppat.1000267
NR 97
TC 22
Z9 27
U1 0
U2 32
PU SPRINGER
PI NEW YORK
PA ONE NEW YORK PLAZA, SUITE 4600, NEW YORK, NY, UNITED STATES
SN 0932-0113
EI 1432-1955
J9 PARASITOL RES
JI Parasitol. Res.

PD JUN
PY 2016
VL 115
IS 6
BP 2155
EP 2164
DI 10.1007/s00436-016-4957-x
PG 10
WC Parasitology
WE Science Citation Index Expanded (SCI-EXPANDED)
SC Parasitology
GA DM1JH
UT WOS:000376101000004
PM 26898834
DA 2024-10-21
ER

PT J
AU Aso, H
   Ito, J
   Ozaki, H
   Kashima, Y
   Suzuki, Y
   Koyanagi, Y
   Sato, K
AF Aso, Hirofumi
   Ito, Jumpei
   Ozaki, Haruka
   Kashima, Yukie
   Suzuki, Yutaka
   Koyanagi, Yoshio
   Sato, Kei
TI Single-cell transcriptome analysis illuminating the characteristics of
   species-specific innate immune responses against viral infections
SO GIGASCIENCE
LA English
DT Article
DE innate immunity; mammal; virus infection; single-cell RNA sequencing;
   tensor
ID GENE-EXPRESSION; RECOGNITION; ACTIVATION; BATS; INTERFERON; RECEPTORS
AB Background: Bats harbor various viruses without severe symptoms and act as their natural reservoirs. The tolerance of bats against viral infections is assumed to originate from the uniqueness of their immune system. However, how immune responses vary between primates and bats remains unclear. Here, we characterized differences in the immune responses by peripheral blood mononuclear cells to various pathogenic stimuli between primates (humans, chimpanzees, and macaques) and bats (Egyptian fruit bats) using single-cell RNA sequencing.
   Results: We show that the induction patterns of key cytosolic DNA/RNA sensors and antiviral genes differed between primates and

bats. A novel subset of monocytes induced by pathogenic stimuli specifically in bats was identified. Furthermore, bats robustly respond to DNA virus infection even though major DNA sensors are dampened in bats.
Conclusions: Overall, our data suggest that immune responses are substantially different between primates and bats, presumably underlying the difference in viral pathogenicity among the mammalian species tested.
C1 [Sato, Kei] Univ Tokyo, Inst Med Sci, Div Syst Virol, Dept Microbiol & Immunol, Tokyo 1088639, Japan.
[Aso, Hirofumi; Koyanagi, Yoshio] Kyoto Univ, Inst Life & Med Sci, Kyoto 6068507, Japan.
[Aso, Hirofumi; Koyanagi, Yoshio] Kyoto Univ, Grad Sch Pharmaceut Sci, Kyoto 6068501, Japan.
[Aso, Hirofumi] Tokyo Med & Dent Univ, Dept AI Syst Med, M&D Data Sci Ctr, Tokyo 1138510, Japan.
[Ozaki, Haruka] Univ Tsukuba, Bioinformat Lab, Fac Med, Tsukuba 3050821, Japan.
[Ozaki, Haruka] Univ Tsukuba, Ctr Artificial Intelligence Res, Tsukuba 3058577, Japan.
[Kashima, Yukie; Suzuki, Yutaka] Univ Tokyo, Grad Sch Frontier Sci, Lab Syst Genom, Kashiwa 2778561, Japan.
[Sato, Kei] Univ Tokyo, Inst Med Sci, Int Res Ctr Infect Dis, Tokyo 1088639, Japan.
[Sato, Kei] Univ Tokyo, Inst Med Sci, Int Vaccine Design Ctr, Tokyo 1088639, Japan.
[Sato, Kei] Univ Tokyo, Grad Sch Med, Tokyo 1130033, Japan.
[Sato, Kei] Univ Tokyo, Grad Sch Frontier Sci, Kashiwa 2778561, Japan.
[Sato, Kei] Kumamoto Univ, Collaborat Unit Infect, Joint Res Ctr Human Retrovirus infect, Kumamoto 8600811, Japan.
[Sato, Kei] Japan Sci & Technol Agency, CREST, Kawaguchi 3320012, Japan.
C3 University of Tokyo; Kyoto University; Kyoto University; Institute of
Science Tokyo; Tokyo Medical & Dental University (TMDU); University of
Tsukuba; University of Tsukuba; University of Tokyo; University of
Tokyo; University of Tokyo; University of Tokyo; University of Tokyo;
Kumamoto University; Japan Science & Technology Agency (JST)
RP Sato, K (corresponding author), Univ Tokyo, Inst Med Sci, Div Syst Virol, Dept Microbiol & Immunol, Tokyo 1088639, Japan.
EM keisato@g.ecc.u-tokyo.ac.jp
RI Ozaki, Haruka/ADO-1642-2022
OI Aso, Hirofumi/0000-0002-2826-3836; Suzuki, Yutaka/0000-0003-4852-1879;
Sato, Kei/0000-0003-4431-1380; Ito, Jumpei/0000-0003-0440-8321;
Koyanagi, Yoshio/0000-0002-3007-6642; Ozaki, Haruka/0000-0002-1606-2762
FU Cooperative Research Program of the Primate Research Institute, Kyoto
University [2019-c9]

FX We thank Naoko Misawa, Akiko Oide, Mai Suganami, and Kazumi Abe (The
University of Tokyo) for technical support; Hiroo Imai (Kyoto
University) for providing primate PBMCs; Ayuko Morita (Kyoto City
Institute of Health and Environmental Sciences) for providing bat PBMCs;
Yasushi Kawaguchi (The University of Tokyo) for providing HSV-1; Takashi
Irie (Hiroshima University) for providing SeV; and Human Genome Center
(the Institute of Medical Science, the University of Tokyo) for
providing the super-computing resource SHIROKANE
(http://sc.hgc.jp/shirokane.html). This work was supported by the
Cooperative Research Program of the Primate Research Institute, Kyoto
University (2019-c9).
CR 10X Genomics, 2020, Build a custom reference (cellranger mkref)
Abcam, 2021, Chemokines and Their Receptors
Ahn M, 2019, NAT MICROBIOL, V4, P789, DOI 10.1038/s41564-019-0371-3
Ahn M, 2016, SCI REP-UK, V6, DOI 10.1038/srep21722
Akira S, 2006, CELL, V124, P783, DOI 10.1016/j.cell.2006.02.015
[Anonymous], 2022, MSigDB (version 7.3)
[Anonymous], 2022, rTensor. rTensor (version 1.4.8)
[Anonymous], 2018, Bat1K Project
Aso H, 2023, scRNA-seq_pbmc_animals_aso_et_al
Aso Hirofumi, 2023, GigaDB, DOI 10.5524/102448
Azimuth, 2020, Azimuth (version 0.4.3)
Banerjee A, 2020, FRONT IMMUNOL, V11, DOI 10.3389/fimmu.2020.00026
Banerjee A, 2017, SCI REP-UK, V7, DOI 10.1038/s41598-017-01513-w
Cell Ranger, 2021, Cell Ranger (version 6.0.1)
De La Cruz-Rivera PC, 2018, J IMMUNOL, V200, P209, DOI 10.4049/jimmunol.1701214
Dempsey A, 2015, VIROLOGY, V479, P146, DOI 10.1016/j.virol.2015.03.013
EJERCITO PM, 1968, J GEN VIROL, V2, P357, DOI 10.1099/0022-1317-2-3-357
Friedrichs V, 2022, CELL REP, V40, DOI 10.1016/j.celrep.2022.111305
Goh G, 2020, P NATL ACAD SCI USA, V117, P28939, DOI 10.1073/pnas.2003352117
Gorbunova V, 2020, CELL METAB, V32, P31, DOI 10.1016/j.cmet.2020.06.013
Guinney Justin, 2017, Bioconductor, DOI 10.18129/B9.BIOC.GSVA
Guito JC, 2021, CURR BIOL, V31, P257, DOI 10.1016/j.cub.2020.10.015
Hänzelmann S, 2013, BMC BIOINFORMATICS, V14, DOI 10.1186/1471-2105-14-7
Hansen JD, 2011, DEV COMP IMMUNOL, V35, P886, DOI 10.1016/j.dci.2011.01.008
Hao YH, 2021, CELL, V184, P3573, DOI 10.1016/j.cell.2021.04.048
Huff JL, 2003, EMERG INFECT DIS, V9, P246
Irving AT, 2020, CELL REP, V33, DOI 10.1016/j.celrep.2020.108345

Jebb D, 2020, NATURE, V583, P578, DOI 10.1038/s41586-020-2486-3
Kiyotani K, 2007, VIROLOGY, V359, P82, DOI 10.1016/j.virol.2006.08.053
Lafaille FG, 2012, NATURE, V491, P769, DOI 10.1038/nature11583
Letko M, 2020, NAT REV MICROBIOL, V18, P461, DOI 10.1038/s41579-020-0394-z
Li QJ, 2007, CELL, V129, P147, DOI 10.1016/j.cell.2007.03.008
Lin HH, 2022, MICROBIOL IMMUNOL, V66, P97, DOI 10.1111/1348-0421.12952
López-Cotarelo P, 2017, TRENDS IMMUNOL, V38, P927, DOI 10.1016/j.it.2017.08.004
Lu YZ, 2020, DEV COMP IMMUNOL, V102, DOI 10.1016/j.dci.2019.103468
McInnes L, 2020, Arxiv, DOI [arXiv:1802.03426, DOI 10.21105/JOSS.00861, 10.21105/joss.00861, 10.48550/arXiv.1802.03426]
National Center for Biotechnology Information, 2020, RefSeq
National Center for Biotechnology Information, 2021, The Report of Orthologous Genes
National Institutes of Health, 2020, The Human BioMolecular Atlas Program (HuBMAP)
Pavlovich SS, 2018, CELL, V173, P1098, DOI 10.1016/j.cell.2018.03.070
Poltorak A, 1998, SCIENCE, V282, P2085, DOI 10.1126/science.282.5396.2085
Posselt G, 2011, J IMMUNOL, V187, P2875, DOI 10.4049/jimmunol.1003348
Quinlan AR, 2010, BIOINFORMATICS, V26, P841, DOI 10.1093/bioinformatics/btq033
Sato Kei, 2023, Mendeley Data, V2, DOI 10.17632/KG3DFKYJV5.2
Sato R, 2018, NAT IMMUNOL, V19, P1071, DOI 10.1038/s41590-018-0203-2
Schountz T, 2017, FRONT IMMUNOL, V8, DOI 10.3389/fimmu.2017.01098
Seal S, 2021, ELIFE, V10, DOI 10.7554/eLife.68874
Seurat, 2021, Seurat (version 4.0.4)
Shah S, 2014, J BIOL CHEM, V289, P13667, DOI 10.1074/jbc.M113.540799
Shaw AE, 2017, PLOS BIOL, V15, DOI 10.1371/journal.pbio.2004086
Takeuchi O, 2010, CELL, V140, P805, DOI 10.1016/j.cell.2010.01.022
TensorLy, 2021, TensorLy (version 0.6.0)
Towner JS, 2009, PLOS PATHOG, V5, DOI 10.1371/journal.ppat.1000536
University of California Santa Cruz, 2000, UCSC Genome Browser
Xie JZ, 2018, CELL HOST MICROBE, V23, P297, DOI 10.1016/j.chom.2018.01.006
Yamada E, 2018, CELL HOST MICROBE, V23, P110, DOI 10.1016/j.chom.2017.12.009
Yoshida A, 2018, J VIROL, V92, DOI 10.1128/JVI.02094-17
Zheng GXY, 2017, NAT COMMUN, V8, DOI 10.1038/ncomms14049
Zhou P, 2016, P NATL ACAD SCI USA, V113, P2696, DOI 10.1073/pnas.1518240113
NR 59
TC 1
Z9 1

U1 1
U2 1
PU OXFORD UNIV PRESS
PI OXFORD
PA GREAT CLARENDON ST, OXFORD OX2 6DP, ENGLAND
SN 2047-217X
J9 GIGASCIENCE
JI GigaScience
PY 2023
VL 12
DI 10.1093/gigascience/giad086
PG 14
WC Biology; Multidisciplinary Sciences
WE Science Citation Index Expanded (SCI-EXPANDED)
SC Life Sciences & Biomedicine - Other Topics; Science & Technology - Other
Topics
GA LU0M0
UT WOS:001189196000012
PM 37848618
OA Green Submitted, gold, Green Published
DA 2024-10-21
ER

PT J
AU Zhou, P
Cowled, C
Mansell, A
Monaghan, P
Green, D
Wu, LJ
Shi, ZL
Wang, LF
Baker, ML
AF Zhou, Peng
Cowled, Chris
Mansell, Ashley
Monaghan, Paul
Green, Diane
Wu, Lijun
Shi, Zhengli
Wang, Lin-Fa
Baker, Michelle L.
TI IRF7 in the Australian Black Flying Fox, <i>Pteropus alecto</i>: Evidence for a Unique Expression Pattern and Functional Conservation
SO PLOS ONE
LA English
DT Article
ID REGULATORY FACTOR FAMILY; TOLL-LIKE RECEPTORS; MOLECULAR CHARACTERIZATION; POSITIVE FEEDBACK; FRUIT BATS; INTERFERON; VIRUS; INDUCTION; GENE; TRANSCRIPTION
AB As the only flying mammal, bats harbor a number of emerging and

re-emerging viruses, may of which cause severe diseases in humans and other mammals yet results in no clinical symptoms in bats. As the master regulator of the interferon (IFN)-dependent immune response, IFN regulatory factor 7 (IRF7) plays a central role in innate antiviral immunity. To explore the role of bat IRF7 in the regulation of IFN response, we performed sequence and functional analysis of IRF7 from the pteropid bat, Pteropus alecto. Our results demonstrate that bat IRF7 retains the ability to bind to MyD88 and activate the IFN response despite unique changes in the MyD88 binding domain. We also demonstrate that bat IRF7 has a unique expression pattern across noth immune and non-immune related tissues and is inducible by double-strand RNA. The broad tissue compared to other mammals. The importance of IRF7 in antiviral activity against the bat reovirus, pulau virus ws confirmed by siRNA knockdown of IRF7 in bat cells resulting in enhanced viral replication. Our results highlight the importance of IRF7 in innate antiviral immunity in bats.
C1 [Zhou, Peng; Cowled, Chris; Monaghan, Paul; Green, Diane; Wang, Lin-Fa; Baker, Michelle L.] CSIRO, Australian Anim Hlth Lab, Geelong, Vic, Australia.
[Mansell, Ashley] Monash Univ, Prince Henry Inst Med Res, Monash Inst Med Res, Ctr Innate Immun & Infect Dis, Clayton, Vic, Australia.
[Wu, Lijun; Shi, Zhengli] Chinese Acad Sci, Wuhan Inst Virol, Ctr Emerging Infect Dis, Wuhan, Peoples R China.
[Wang, Lin-Fa] Duke Natl Univ Singapore, Grad Sch Med, Program Emerging Infect Dis, Singapore, Singapore.
C3 Commonwealth Scientific & Industrial Research Organisation (CSIRO);
Monash Institute of Medical Research; Prince Henry's Institute of Medical Research; Monash University; Chinese Academy of Sciences; Wuhan
Institute of Virology, CAS; National University of Singapore
RP Baker, ML (corresponding author), CSIRO, Australian Anim Hlth Lab, Geelong, Vic, Australia.
EM Michelle.Baker@csiro.au
RI Monaghan, Paul/H-1973-2013; Zhou, Peng/I-8578-2015; Shi, Zheng-Li/A-1013-2013; Baker, Michelle/C-9694-2013; Mansell, Ashley/B-9553-2008; Cowled, Christopher/H-5308-2013
OI Shi, Zheng-Li/0000-0001-8089-163X; Wang, Lin-Fa/0000-0003-2752-0535;
Baker, Michelle/0000-0002-7993-9971; Mansell, Ashley/0000-0002-6540-1281; Cowled, Christopher/0000-0002-1969-9371
FU Australian Research Council Future Fellowship [FT110100234]; CSIRO OCE
Science Leaders award; Victorian Government's Operational Infrastructure
Support Program; Australian Research Council [FT110100234] Funding
Source: Australian Research Council
FX This work was supported by funding from the Australian Research Council
Future Fellowship (FT110100234) to M. L. B. and a CSIRO OCE

Science
   Leaders award to L. -F. W. and support from the Victorian
Government's
   Operational Infrastructure Support Program. The funders had no
role in
   study design, data collection and analysis, decision to publish,
or
   preparation of the manuscript.
CR Baker ML, 2013, ZOONOSES PUBLIC HLTH, V60, P104, DOI 10.1111/
j.1863-2378.2012.01528.x
   Bergan V, 2010, MOL IMMUNOL, V47, P2005, DOI 10.1016/
j.molimm.2010.04.015
   Bermingham A, 2012, EUROSURVEILLANCE, V17, P6
   Bidwell BN, 2012, NAT MED, V18, P1224, DOI 10.1038/nm.2830
   Calisher CH, 2006, CLIN MICROBIOL REV, V19, P531, DOI 10.1128/
CMR.00017-06
   Chua KB, 2000, SCIENCE, V288, P1432, DOI 10.1126/
science.288.5470.1432
   Civas A, 2002, BIOCHIMIE, V84, P643, DOI 10.1016/
S0300-9084(02)01431-1
   Cowled C, 2012, DEV COMP IMMUNOL, V36, P657, DOI 10.1016/
j.dci.2011.11.008
   Cowled C, 2011, DEV COMP IMMUNOL, V35, P7, DOI 10.1016/
j.dci.2010.07.006
   Crameri G, 2009, PLOS ONE, V4, DOI 10.1371/journal.pone.0008266
   FUJITA T, 1988, EMBO J, V7, P3397, DOI 10.1002/
j.1460-2075.1988.tb03213.x
   Ge XY, 2013, NATURE, V503, P535, DOI 10.1038/nature12711
   Génin P, 2009, CYTOKINE GROWTH F R, V20, P283, DOI 10.1016/
j.cytogfr.2009.07.005
   Halpin K, 2000, J GEN VIROL, V81, P1927, DOI
10.1099/0022-1317-81-8-1927
   Honda K, 2005, NATURE, V434, P772, DOI 10.1038/nature03464
   Honda K, 2004, P NATL ACAD SCI USA, V101, P15416, DOI 10.1073/
pnas.0406933101
   Honda K, 2006, IMMUNITY, V25, P349, DOI 10.1016/
j.immuni.2006.08.009
   Hoshino K, 2006, NATURE, V440, P949, DOI 10.1038/nature04641
   Hu GB, 2010, FISH SHELLFISH IMMUN, V29, P963, DOI 10.1016/
j.fsi.2010.08.002
   Ikeda F, 2007, EMBO J, V26, P3451, DOI 10.1038/sj.emboj.7601773
   Into T, 2010, J BIOL CHEM, V285, P35759, DOI 10.1074/
jbc.M110.126904
   Jia WZ, 2008, MOL IMMUNOL, V45, P2419, DOI 10.1016/
j.molimm.2007.11.011
   Kawai T, 2004, NAT IMMUNOL, V5, P1061, DOI 10.1038/ni1118
   Leroy EM, 2005, NATURE, V438, P575, DOI 10.1038/438575a
   Lin RT, 2000, J BIOL CHEM, V275, P34320, DOI 10.1074/
jbc.M002814200
   Lu RQ, 2000, J BIOL CHEM, V275, P31805, DOI 10.1074/
jbc.M005288200
   Mansell A, 2006, NAT IMMUNOL, V7, P148, DOI 10.1038/ni1299
   Marié I, 1998, EMBO J, V17, P6660, DOI 10.1093/emboj/17.22.6660
   Marié I, 2000, MOL CELL BIOL, V20, P8803, DOI 10.1128/

MCB.20.23.8803-8814.2000
Ning S, 2011, GENES IMMUN, V12, P399, DOI 10.1038/gene.2011.21
Nishiya T, 2007, FEBS LETT, V581, P3223, DOI 10.1016/j.febslet.2007.06.008
Österlund PI, 2007, J IMMUNOL, V179, P3434, DOI 10.4049/jimmunol.179.6.3434
Papenfuss AT, 2012, BMC GENOMICS, V13, DOI 10.1186/1471-2164-13-261
Paun A, 2007, BIOCHIMIE, V89, P744, DOI 10.1016/j.biochi.2007.01.014
Pritchard LI, 2006, ARCH VIROL, V151, P229, DOI 10.1007/s00705-005-0644-4
Randall RE, 2008, J GEN VIROL, V89, P1, DOI 10.1099/vir.0.83391-0
Sato M, 1998, FEBS LETT, V441, P106, DOI 10.1016/S0014-5793(98)01514-2
Strahle L, 2006, VIROLOGY, V351, P101, DOI 10.1016/j.virol.2006.03.022
Sun BJ, 2007, VET IMMUNOL IMMUNOP, V116, P26, DOI 10.1016/j.vetimm.2007.01.001
Tamura K, 2007, MOL BIOL EVOL, V24, P1596, DOI 10.1093/molbev/msm092
van der Poel WHM, 2006, VECTOR-BORNE ZOONOT, V6, P315, DOI 10.1089/vbz.2006.6.315
Vaquerizas JM, 2009, NAT REV GENET, V10, P252, DOI 10.1038/nrg2538
Zhang GJ, 2013, SCIENCE, V339, P456, DOI 10.1126/science.1230835
Zhang LW, 1997, MOL CELL BIOL, V17, P5748, DOI 10.1128/MCB.17.10.5748
Zhang YB, 2003, FISH SHELLFISH IMMUN, V15, P453, DOI 10.1016/S1050-4648(03)00025-1
Zhou P, 2013, DEV COMP IMMUNOL, V40, P240, DOI 10.1016/j.dci.2013.03.006
Zhou P, 2011, PLOS ONE, V6, DOI 10.1371/journal.pone.0025385
Zhou P, 2011, J IMMUNOL, V186, P3138, DOI 10.4049/jimmunol.1003115
NR 48
TC 45
Z9 51
U1 1
U2 21
PU PUBLIC LIBRARY SCIENCE
PI SAN FRANCISCO
PA 1160 BATTERY STREET, STE 100, SAN FRANCISCO, CA 94111 USA
SN 1932-6203
J9 PLOS ONE
JI PLoS One
PD AUG 6
PY 2014
VL 9
IS 8
AR e103875
DI 10.1371/journal.pone.0103875
PG 13
WC Multidisciplinary Sciences

WE Science Citation Index Expanded (SCI-EXPANDED)
SC Science & Technology - Other Topics
GA AM6SM
UT WOS:000339995100037
PM 25100081
OA Green Submitted, Green Published, gold
DA 2024-10-21
ER

PT J
AU Becker, DJ
Speer, KA
Korstian, JM
Volokhov, DV
Droke, HF
Brown, AM
Baijnauth, CL
Padgett-Stewart, T
Broders, HG
Plowright, RK
Rainwater, TR
Fenton, MB
Simmons, NB
Chumchal, MM
AF Becker, Daniel J.
Speer, Kelly A.
Korstian, Jennifer M.
Volokhov, Dmitriy V.
Droke, Hannah F.
Brown, Alexis M.
Baijnauth, Catherene L.
Padgett-Stewart, Ticha
Broders, Hugh G.
Plowright, Raina K.
Rainwater, Thomas R.
Fenton, M. Brock
Simmons, Nancy B.
Chumchal, Matthew M.
TI Disentangling interactions among mercury, immunity and infection in a
Neotropical bat community
SO JOURNAL OF APPLIED ECOLOGY
LA English
DT Article
DE bacterial pathogens; Chiroptera; disease ecology; ecoimmunology;
ecotoxicology; heavy metals; Latin America
ID R PACKAGE; LAND-USE; EXPOSURE; DIVERSE; BIOACCUMULATION; CONTAMINANTS;
MAMMALS; SAMPLES; REGION; AMAZON
AB Contaminants such as mercury are pervasive and can have immunosuppressive effects on wildlife. Impaired immunity could be important for forecasting pathogen spillover, as many land-use changes that generate mercury contamination also bring wildlife into close contact with humans and domestic animals. However, the

interactions among contaminants, immunity and infection are difficult to study in natural systems, and empirical tests of possible directional relationships remain rare.

We capitalized on extreme mercury variation in a diverse bat community in Belize to test association among contaminants, immunity and infection. By comparing a previous dataset of bats sampled in 2014 with new data from 2017, representing a period of rapid agricultural land conversion, we first confirmed bat species more reliant on aquatic prey had higher fur mercury. Bats in the agricultural habitat also had higher mercury in recent years. We then tested covariation between mercury and cellular immunity and determined if such relationships mediated associations between mercury and bacterial pathogens. As bat ecology can dictate exposure to mercury and pathogens, we also assessed species-specific patterns in mercury-infection relationships.

Across the bat community, individuals with higher mercury had fewer neutrophils but not lymphocytes, suggesting stronger associations with innate immunity. However, the odds of infection for haemoplasmas and Bartonella spp. were generally lowest in bats with high mercury, and relationships between mercury and immunity did not mediate infection patterns. Mercury also showed species- and clade-specific relationships with infection, being associated with especially low odds for haemoplasmas in Pteronotus mesoamericanus and Dermanura phaeotis. For Bartonella spp., mercury was associated with particularly low odds of infection in the genus Pteronotus but high odds in the subfamily Stenodermatinae.

Synthesis and application. Lower general infection risk in bats with high mercury despite weaker innate defense suggests contaminant-driven loss of pathogen habitat (i.e. anemia) or vector mortality as possible causes. Greater attention to these potential pathways could help disentangle relationships among contaminants, immunity and infection in anthropogenic habitats and help forecast disease risks. Our results also suggest that contaminants may increase infection risk in some taxa but not others, emphasizing the importance of considering surveillance and management at different phylogenetic scales.
C1 [Becker, Daniel J.] Univ Oklahoma, Dept Biol, Norman, OK 73019 USA.

[Speer, Kelly A.] Amer Museum Nat Hist, Richard Gilder Grad Sch, New York, NY 10024 USA.

[Speer, Kelly A.] Smithsonian Inst, Natl Museum Nat Hist, Dept Invertebrate Zool, Washington, DC 20560 USA.

[Speer, Kelly A.] Smithsonian Conservat Biol Inst, Ctr Conservat Genom, Natl Zool Pk, Washington, DC USA.

[Korstian, Jennifer M.] Texas Tech Univ, Dept Biol Sci, Lubbock, TX 79409 USA.

[Volokhov, Dmitriy V.] US FDA, Ctr Biol Evaluat & Res, Silver Spring, MD USA.

[Droke, Hannah F.] Univ S Florida, Dept Global & Planetary Hlth, Tampa, FL 33620 USA.

[Brown, Alexis M.] SUNY Stony Brook, Dept Ecol & Evolut, Stony Brook, NY 11794 USA.

[Baijnauth, Catherene L.] Amer Museum Nat Hist, Sackler Inst Comparat Genom, New York, NY 10024 USA.

[Padgett-Stewart, Ticha; Plowright, Raina K.] Montana State Univ, Dept Microbiol & Immunol, Bozeman, MT 59717 USA.
[Broders, Hugh G.] Univ Waterloo, Dept Biol, Waterloo, ON, Canada.
[Rainwater, Thomas R.] Clemson Univ, Dept Forestry & Environm Conservat, Clemson, SC USA.
[Rainwater, Thomas R.] Clemson Univ, Baruch Inst Coastal Ecol & Forest Sci, Georgetown, SC USA.
[Rainwater, Thomas R.] Tom Yawkey Wildlife Ctr, Georgetown, SC USA.
[Fenton, M. Brock] Western Univ, Dept Biol, London, ON, Canada.
[Simmons, Nancy B.] Amer Museum Nat Hist, Dept Mammal, Div Vertebrate Zool, New York, NY 10024 USA.
[Chumchal, Matthew M.] Texas Christian Univ, Dept Biol, Ft Worth, TX 76129 USA.
C3 University of Oklahoma System; University of Oklahoma - Norman; American
Museum of Natural History (AMNH); Smithsonian Institution; Smithsonian
National Museum of Natural History; Smithsonian Institution; Smithsonian
National Zoological Park & Conservation Biology Institute; Texas Tech
University System; Texas Tech University; US Food & Drug Administration
(FDA); State University System of Florida; University of South Florida;
State University of New York (SUNY) System; Stony Brook University;
American Museum of Natural History (AMNH); Montana State University
System; Montana State University Bozeman; University of Waterloo;
Clemson University; Clemson University; Western University (University
of Western Ontario); American Museum of Natural History (AMNH); Texas
Christian University
RP Becker, DJ (corresponding author), Univ Oklahoma, Dept Biol, Norman, OK 73019 USA.
EM danbeck@ou.edu
RI Broders, Hugh/C-9087-2011; Becker, Daniel/A-6466-2014
OI Speer, Kelly A./0000-0002-8775-7254; Plowright,
Raina/0000-0002-3338-6590; Chumchal, Matthew/0000-0001-7160-3157;
Volokhov, Dmitriy/0000-0003-3363-8361; Broders,
Hugh/0000-0002-6151-8079; Brown, Alexis/0000-0003-4258-0501; Becker,
Daniel/0000-0003-4315-8628
FU National Science Foundation [DEB-1716698]; Achievement Rewards for
College Scientists Foundation; American Museum of Natural History; U.S.
Department of Agriculture [1015891]; National Institutes of Health
[P20GM103474, P30GM110732]; Defense Advanced Research Projects

Agency
[D16AP00113, D18AC00031]; Richard Gilder Graduate School Student
Research Fellowship; Texas Christian University Research and
Creative
Activities Fund Award; Natural Sciences and Engineering Research
Council; Yawkey Foundation
FX National Science Foundation, Grant/Award Number: DEB-1716698;
Achievement Rewards for College Scientists Foundation; American
Museum
of Natural History; U.S. Department of Agriculture, Grant/Award
Number:
1015891; National Institutes of Health, Grant/Award Number:
P20GM103474
and P30GM110732; Defense Advanced Research Projects Agency,
Grant/Award
Number: D16AP00113 and D18AC00031; Richard Gilder Graduate School
Student Research Fellowship; Texas Christian University Research
and
Creative Activities Fund Award; Natural Sciences and Engineering
Research Council; Yawkey Foundation
CR [Anonymous], 2016, THESIS
Bai Y, 2016, J WILDLIFE DIS, V52, P844, DOI 10.7589/2016-01-015
Becker DJ, 2020, MOL ECOL, V29, P1534, DOI 10.1111/mec.15422
Becker DJ, 2020, J ANIM ECOL, V89, P972, DOI
10.1111/1365-2656.13166
Becker DJ, 2018, PLOS NEGLECT TROP D, V12, DOI 10.1371/
journal.pntd.0006786
Becker DJ, 2018, ENVIRON POLLUT, V233, P1076, DOI 10.1016/
j.envpol.2017.10.010
Becker DJ, 2017, ROY SOC OPEN SCI, V4, DOI 10.1098/rsos.170073
BECKER DJ, 2018, PHILOS T R SOC B, V373
Bergner LM, 2019, MOL ECOL RESOUR, V19, P128, DOI
10.1111/1755-0998.12946
BORREMANS B, 2019, PHILOS T R SOC B, V374
Brook CE, 2015, TRENDS MICROBIOL, V23, P172, DOI 10.1016/
j.tim.2014.12.004
Bürkner PC, 2017, J STAT SOFTW, V80, P1, DOI 10.18637/
jss.v080.i01
Carrasco-Rueda F, 2020, ECOTOXICOLOGY, V29, P1032, DOI 10.1007/
s10646-020-02195-3
Chamberlain Scott A, 2013, F1000Res, V2, P191, DOI 10.12688/
f1000research.2-191.v1
Chételat J, 2018, SCI TOTAL ENVIRON, V626, P668, DOI 10.1016/
j.scitotenv.2018.01.044
Chumchal MM, 2011, ENVIRON TOXICOL CHEM, V30, P1153, DOI 10.1002/
etc.477
Costantini D, 2019, SCI TOTAL ENVIRON, V671, P1077, DOI 10.1016/
j.scitotenv.2019.03.398
Cristol DA, 2008, SCIENCE, V320, P335, DOI 10.1126/
science.1154082
Davidson C, 2004, ECOL APPL, V14, P1892, DOI 10.1890/03-5224
Eeva T, 2013, PARASITOLOGY, V140, P1384, DOI 10.1017/
S0031182013000796
Farella N, 2007, AGR ECOSYST ENVIRON, V120, P449, DOI 10.1016/

j.agee.2006.11.003
Fisk AT, 2005, SCI TOTAL ENVIRON, V351, P57, DOI 10.1016/j.scitotenv.2005.01.051
Flache L, 2015, MAMM BIOL, V80, P178, DOI 10.1016/j.mambio.2015.01.007
Fritz S., 2013, R. Package Version, V5, P1
Gelman A, 2019, AM STAT, V73, P307, DOI 10.1080/00031305.2018.1549100
Graham CH, 2018, GLOBAL ECOL BIOGEOGR, V27, P175, DOI 10.1111/geb.12686
Grasman KA, 2002, INTEGR COMP BIOL, V42, P34, DOI 10.1093/icb/42.1.34
Gunnell GF, 2012, CAM STUD MORPHOL MOL, P1, DOI 10.1017/CBO9781139045599
Hanna P, 2018, SCI TOTAL ENVIRON, V644, P151, DOI 10.1016/j.scitotenv.2018.06.297
Heinze G, 2002, STAT MED, V21, P2409, DOI 10.1002/sim.1047
Herrera JP, 2018, ACTA CHIROPTEROL, V20, P147, DOI 10.3161/15081109ACC2018.20.1.011
Ikeda P, 2017, EPIDEMIOL INFECT, V145, P2038, DOI [10.1017/S0950268817000966, 10.1017/s0950268817000966]
Imai K, 2010, PSYCHOL METHODS, V15, P309, DOI 10.1037/a0020761
Judson SD, 2015, ZOONOSES PUBLIC HLTH, V62, P609, DOI 10.1111/zph.12188
Korstian JM, 2018, ENVIRON TOXICOL CHEM, V37, P160, DOI 10.1002/etc.3940
Lalancette A, 2003, DEV COMP IMMUNOL, V27, P735, DOI 10.1016/S0145-305X(03)00038-7
Lanier LL, 2013, NAT REV IMMUNOL, V13, P73, DOI 10.1038/nri3389
Lewis CA, 2013, ARCH ENVIRON CON TOX, V64, P327, DOI 10.1007/s00244-012-9830-z
Michonneau F, 2016, METHODS ECOL EVOL, V7, P1476, DOI 10.1111/2041-210X.12593
Millán J, 2021, TRANSBOUND EMERG DIS, V68, P3236, DOI 10.1111/tbed.13932
Nam DH, 2012, ECOTOXICOLOGY, V21, P1094, DOI 10.1007/s10646-012-0864-9
Ortega-Rodriguez CL, 2019, ENVIRON TOXICOL CHEM, V38, P2503, DOI 10.1002/etc.4579
PALHETA D, 1995, SCI TOTAL ENVIRON, V168, P63, DOI 10.1016/0048-9697(95)04533-7
Paradis E, 2004, BIOINFORMATICS, V20, P289, DOI [10.1093/bioinformatics/btg412, 10.1093/bioinformatics/bty633]
Rainwater TR, 2005, ENVIRON TOXICOL CHEM, V24, P665, DOI 10.1897/04-223R.1
Reid F.A., 1997, FIELD GUIDE MAMMALS
Rojas D, 2011, MOL ECOL, V20, P2217, DOI 10.1111/j.1365-294X.2011.05082.x
Ross PS, 2002, HUM ECOL RISK ASSESS, V8, P277, DOI 10.1080/20028091056917
Salinas-Ramos VB, 2015, MOL ECOL, V24, P5296, DOI 10.1111/mec.13386
Scheulhammer AM, 2007, AMBIO, V36, P12, DOI 10.1579/0044-7447(2007)36[12:EOEMOT]2.0.CO;2

Schneeberger K, 2014, PLOS ONE, V9, DOI 10.1371/journal.pone.0108268
Schneeberger K, 2013, PLOS ONE, V8, DOI 10.1371/journal.pone.0054023
Serieys LEK, 2018, P ROY SOC B-BIOL SCI, V285, DOI 10.1098/rspb.2017.2533
SHAW BP, 1991, ENVIRON POLLUT, V73, P43, DOI 10.1016/0269-7491(91)90095-E
Sikes RS, 2016, J MAMMAL, V97, P663, DOI 10.1093/jmammal/gyw078
Smith PN, 2007, ENVIRON POLLUT, V150, P41, DOI 10.1016/j.envpol.2007.06.009
Speir SL, 2014, ENVIRON TOXICOL CHEM, V33, P2506, DOI 10.1002/etc.2700
Streicker DG, 2016, J APPL ECOL, V53, P1280, DOI 10.1111/1365-2664.12690
ter Hofstede HM, 2004, CAN J ZOOL, V82, P616, DOI [10.1139/z04-030, 10.1139/Z04-030]
Vehtari A, 2017, STAT COMPUT, V27, P1433, DOI 10.1007/s11222-016-9709-3
Volokhov D V, 2017, Epidemiol Infect, V145, P3154, DOI [10.1017/s095026881700231x, 10.1017/S095026881700231X]
Wada H, 2010, ECOTOXICOLOGY, V19, P1277, DOI 10.1007/s10646-010-0513-0
Washburne AD, 2019, ECOL MONOGR, V89, DOI 10.1002/ecm.1353
Weise P, 2017, PEERJ, V5, DOI 10.7717/peerj.3570
Weiss B, 2011, TRENDS PARASITOL, V27, P514, DOI 10.1016/j.pt.2011.05.001
Wilman H., 2014, Ecology, V95, P2027, DOI [DOI 10.1890/13-1917.1, 10.1890/13-1917.1]
Yates DE, 2014, ECOTOXICOLOGY, V23, P45, DOI 10.1007/s10646-013-1150-1
NR 67
TC 14
Z9 16
U1 2
U2 30
PU WILEY
PI HOBOKEN
PA 111 RIVER ST, HOBOKEN 07030-5774, NJ USA
SN 0021-8901
EI 1365-2664
J9 J APPL ECOL
JI J. Appl. Ecol.
PD APR
PY 2021
VL 58
IS 4
BP 879
EP 889
DI 10.1111/1365-2664.13809
EA DEC 2020
PG 11
WC Biodiversity Conservation; Ecology
WE Science Citation Index Expanded (SCI-EXPANDED)

SC Biodiversity & Conservation; Environmental Sciences & Ecology
GA RJ3IY
UT WOS:000598259200001
PM 33911313
OA Green Submitted, Green Accepted
DA 2024-10-21
ER

PT J
AU Lytras, S
   Hughes, J
   Martin, D
   Swanepoel, P
   de Klerk, A
   Lourens, R
   Pond, SLK
   Xia, W
   Jiang, XW
   Robertson, DL
AF Lytras, Spyros
   Hughes, Joseph
   Martin, Darren
   Swanepoel, Phillip
   de Klerk, Arne
   Lourens, Rentia
   Pond, Sergei L. Kosakovsky
   Xia, Wei
   Jiang, Xiaowei
   Robertson, David L.
TI Exploring the Natural Origins of SARS-CoV-2 in the Light of
   Recombination
SO GENOME BIOLOGY AND EVOLUTION
LA English
DT Article
DE SARS-CoV-2; Sarbecoviruses; bats; origin; COVID-19; host range;
   coronaviruses; recombination; Rhinolophus; pangolins
ID DETECTING RECOMBINATION; MOSAIC STRUCTURE; DNA-SEQUENCES;
ALGORITHM
AB The lack of an identifiable intermediate host species for the
proximal animal ancestor of SARS-CoV-2, and the large geographical
distance between Wuhan and where the closest evolutionary related
coronaviruses circulating in horseshoe bats (members of the
Sarbecovirus subgenus) have been identified, is fueling speculation
on the natural origins of SARS-CoV-2. We performed a comprehensive
phylogenetic study on SARS-CoV-2 and all the related bat and
pangolin sarbecoviruses sampled so far. Determining the likely
recombination events reveals a highly reticulate evolutionary
history within this group of coronaviruses. Distribution of the
inferred recombination events is nonrandom with evidence that Spike,
the main target for humoral immunity, is beside a recombination
hotspot likely driving antigenic shift events in the ancestry of bat
sarbecoviruses. Coupled with the geographic ranges of their hosts
and the sampling locations, across southern China, and into
Southeast Asia, we confirm that horseshoe bats, Rhinolophus, are the

likely reservoir species for the SARS-CoV-2 progenitor. By tracing the recombinant sequence patterns, we conclude that there has been relatively recent geographic movement and cocirculation of these viruses' ancestors, extending across their bat host ranges in China and Southeast Asia over the last 100 years. We confirm that a direct proximal ancestor to SARS-CoV-2 has not yet been sampled, since the closest known relatives collected in Yunnan shared a common ancestor with SARS-CoV-2 approximately 40 years ago. Our analysis highlights the need for dramatically more wildlife sampling to: 1) pinpoint the exact origins of SARS-CoV-2's animal progenitor, 2) the intermediate species that facilitated transmission from bats to humans (if there is one), and 3) survey the extent of the diversity in the related sarbecoviruses' phylogeny that present high risk for future spillovers.
C1 [Lytras, Spyros; Hughes, Joseph; Robertson, David L.] Univ Glasgow, MRC, Ctr Virus Res, Glasgow, Lanark, Scotland.
[Martin, Darren; Swanepoel, Phillip; de Klerk, Arne] Univ Cape Town, Dept Integrat Biomed Sci, Computat Biol Div, Cape Town, South Africa.
[Lourens, Rentia] Univ Cape Town, Neurosci Inst, Dept Surg, Div Neurosurg, Cape Town, South Africa.
[Pond, Sergei L. Kosakovsky] Temple Univ, Dept Biol, Inst Genom & Evolutionary Med, Philadelphia, PA 19122 USA.
[Xia, Wei] South China Agr Univ, Natl Sch Agr Inst & Dev, Guangzhou, Peoples R China.
[Jiang, Xiaowei] Xian Jiaotong Liverpool Univ XJTLU, Dept Biol Sci, Suzhou, Peoples R China.
C3 University of Glasgow; University of Cape Town; University of Cape Town;
Pennsylvania Commonwealth System of Higher Education (PCSHE); Temple
University; South China Agricultural University; Xi'an
Jiaotong-Liverpool University
RP Robertson, DL (corresponding author), Univ Glasgow, MRC, Ctr Virus Res, Glasgow, Lanark, Scotland.
EM david.l.robertson@glasgow.ac.uk
RI Jiang, Xiaowei/D-1079-2010; Hughes, Joseph/B-7711-2009; Lytras,
Spyros/GPT-0655-2022; Robertson, David/KXQ-9544-2024; Pond,
Sergei/G-9830-2012; Martin, Darren/ABE-3614-2021
OI Jiang, Xiaowei/0000-0002-6030-1249; Robertson,
David/0000-0001-6338-0221; Hughes, Joseph/0000-0003-2556-2563; Martin,
Darren/0000-0002-8785-0870; Lytras, Spyros/0000-0003-4202-6682
FU UK Medical Research Council (MRC) [MC_UU_1201412]; Wellcome Trust (WT)
[220977/Z/20/Z]; MRC studentship; WT [222574/Z/21/Z]; National
Institutes of Health (NIH) [R01 AI134384]; Jiangsu Province High-level
Innovation and Entrepreneurship Talent Programme; National Institute of
Allergy and Infectious Diseases [R01AI134384] Funding Source: NIH
RePORTER; Wellcome Trust [222574/Z/21/Z] Funding Source: Wellcome Trust
FX We would like to thank all the authors who have kindly deposited

and
shared genome data on GISAID. Credit also needs to be given to the
surveillance projects for generating the genome data that is available
in GenBank and to the software developers for making the tools we have
used freely available. A table with genome sequence acknowledgments can
be found in the Supplementary Material online supplementary table S1,
Supplementary Material online). D.L.R. and J.H. are funded by the UK
Medical Research Council (MRC, MC_UU_1201412) and D.L.R. by the Wellcome
Trust (WT, 220977/Z/20/Z). S.L. is funded by an MRC studentship. D.M. is
funded by the WT (222574/Z/21/Z). S.L.K.P. was supported in part by a
grant fromthe National Institutes of Health (NIH, R01 AI134384
[NIH/NIAID]). X.J. is funded by the Jiangsu Province High-level
Innovation and Entrepreneurship Talent Programme.
CR Akaike H., 1973, Information Theory: Proceedings of the 2nd International Symposium ed. by, P267, DOI [10.1007/978-1-4612-1694-015, DOI 10.1007/978-1-4612-1694-015]
Bates P., 2019, RHINOLOPHUS MALAYANU
Bergner LM, 2021, VIRUSES-BASEL, V13, DOI 10.3390/v13020252
Bobay LM, 2020, PLOS GENET, V16, DOI 10.1371/journal.pgen.1009272
Boni MF, 2007, GENETICS, V176, P1035, DOI 10.1534/genetics.106.068874
Boni MF, 2020, NAT MICROBIOL, V5, P1408, DOI 10.1038/s41564-020-0771-4
Challender D., 2019, MANIS PENTADACTYLA I, DOI DOI 10.2305/IUCN.UK.2019-3.RLTS.T12764A123585318.EN
Conceicao C, 2020, PLOS BIOL, V18, DOI 10.1371/journal.pbio.3001016
de Klerk A, 2021, bioRxiv, DOI [10.1101/2021.11.21.469423, 10.1101/2021.11.21.469423v1, DOI 10.1101/2021.11.21.469423]
Drummond AJ, 2007, BMC EVOL BIOL, V7, DOI 10.1186/1471-2148-7-214
Gibbs MJ, 2000, BIOINFORMATICS, V16, P573, DOI 10.1093/bioinformatics/16.7.573
Goldstein SA, 2021, bioRxiv, DOI [10.1101/2021.02.03.429646, 10.1101/2021.02.03.429646, DOI 10.1101/2021.02.03.429646]
Gorbalenya AE, 2020, NAT MICROBIOL, V5, P536, DOI 10.1038/s41564-020-0695-z
Graham RL, 2010, J VIROL, V84, P3134, DOI 10.1128/JVI.01394-09
Hall T., 1999, NUCL ACIDS S SER, P95, DOI [DOI 10.14344/IOC.ML.11.1, 10.1021/bk-1999-0734.ch008]
Heath L, 2006, J VIROL, V80, P11827, DOI 10.1128/JVI.01100-06
Hoffmann RS., 2013, PRINCETON POCKET GUI
Holmes EC, 1999, MOL BIOL EVOL, V16, P405, DOI 10.1093/oxfordjournals.molbev.a026121
Holmes EC, 2021, CELL, V184, P4848, DOI 10.1016/j.cell.2021.08.017

Hu D, 2018, EMERG MICROBES INFEC, V7, DOI 10.1038/s41426-018-0155-5
Huerta-Cepas J, 2016, MOL BIOL EVOL, V33, P1635, DOI 10.1093/molbev/msw046
Jackson B, 2021, CELL, V184, P5179, DOI 10.1016/j.cell.2021.08.014
Jariani A, 2019, VIRUS EVOL, V5, DOI 10.1093/ve/vez003
Katoh K, 2013, MOL BIOL EVOL, V30, P772, DOI 10.1093/molbev/mst010
Lam TTY, 2020, NATURE, V583, P282, DOI 10.1038/s41586-020-2169-0
Nguyen LT, 2015, MOL BIOL EVOL, V32, P268, DOI 10.1093/molbev/msu300
Latinne Alice, 2020, Nat Commun, V11, P4235, DOI [10.1038/s41467-020-17687-3, 10.1101/2020.05.31.116061]
Lee J, 2020, ECOHEALTH, V17, P406, DOI 10.1007/s10393-020-01503-x
Li H., 2019, BIOSAF HLTH, V1, P84, DOI 10.1016/j.bsheal.2019.10.004
Li LL, 2021, EMERG MICROBES INFEC, V10, P1683, DOI 10.1080/22221751.2021.1964925
Li Q., 2020, NEW ENGL J MED, V382, P1199, DOI [10.1056/NEJMoa2001316, DOI 10.1056/NEJMoa2001316]
Li XJ, 2020, SCI ADV, V6, DOI 10.1126/sciadv.abb9153
Lin XD, 2017, VIROLOGY, V507, P1, DOI 10.1016/j.virol.2017.03.019
Liu P, 2019, VIRUSES-BASEL, V11, DOI 10.3390/v11110979
Luo JH, 2013, ORYX, V47, P526, DOI 10.1017/S0030605311001505
Lytras S, 2021, SCIENCE, V373, P968, DOI 10.1126/science.abh0117
MacLean OA, 2021, PLOS BIOL, V19, DOI 10.1371/journal.pbio.3001115
Mao XG, 2010, MOL ECOL, V19, P2754, DOI 10.1111/j.1365-294X.2010.04704.x
Martin D, 2000, BIOINFORMATICS, V16, P562, DOI 10.1093/bioinformatics/16.6.562
Martin DP, 2017, METHODS MOL BIOL, V1525, P433, DOI 10.1007/978-1-4939-6622-6_17
Martin DP, 2005, AIDS RES HUM RETROV, V21, P98, DOI 10.1089/aid.2005.21.98
Martin DP., 2021, VIRUS EVOL, V7, P087, DOI [DOI 10.1093/VE/VEAA087, 10.1093/ve/veaa087]
Munnink BBO, 2021, SCIENCE, V371, P172, DOI 10.1126/science.abe5901
Nikolaidis M, 2022, MOL BIOL EVOL, V39, DOI 10.1093/molbev/msab292
Padidam M, 1999, VIROLOGY, V265, P218, DOI 10.1006/viro.1999.0056
Piraccini R., 2016, Rhinolophus ferrumequinum, Greater Horsehoe Bat. IUCN Read List of Threatened species
Pond SLK, 2020, MOL BIOL EVOL, V37, P295, DOI 10.1093/molbev/msz197
Pond SLK, 2006, BIOINFORMATICS, V22, P3096, DOI 10.1093/bioinformatics/btl474
Posada D, 2002, MOL BIOL EVOL, V19, P708, DOI 10.1093/oxfordjournals.molbev.a004129
Posada D, 2001, P NATL ACAD SCI USA, V98, P13757, DOI 10.1073/pnas.241370698
Simon-Loriere E, 2009, PLOS PATHOG, V5, DOI 10.1371/

journal.ppat.1000418
SMITH JM, 1992, J MOL EVOL, V34, P126
Sola I, 2015, ANNU REV VIROL, V2, P265, DOI 10.1146/annurev-virology-100114-055218
Suchard MA, 2018, VIRUS EVOL, V4, DOI 10.1093/ve/vey016
Suyama M, 2006, NUCLEIC ACIDS RES, V34, pW609, DOI 10.1093/nar/gkl315
Temmam S., 2022, NATURE AVAILABLE
Wacharapluesadee S, 2021, NAT COMMUN, V12, DOI 10.1038/s41467-021-21240-1
Walls AC, 2020, CELL, V181, P281, DOI [10.1016/j.cell.2020.02.058, 10.1016/j.cell.2020.11.032]
Wang HR, 2021, VIRUS EVOL, V7, DOI 10.1093/ve/veaa098
Wang N, 2018, VIROL SIN, V33, P104, DOI 10.1007/s12250-018-0012-7
Wells HL., 2021, Virus Evol, V7, pv
WHO, 2021, WHO CONV GLOB STUD O
Worobey M, 2021, SCIENCE, V374, P1202, DOI 10.1126/science.abm4454
Wu F, 2020, NATURE, V579, P265, DOI 10.1038/s41586-020-2008-3
Xia W., 2021, ONE PANDEMIC LED ANO
Xiao KP, 2020, NATURE, V583, P286, DOI 10.1038/s41586-020-2313-x
Xiao X, 2021, SCI REP-UK, V11, DOI 10.1038/s41598-021-91470-2
Xu L., 2016, TRAFFIC
Yang YY, 2021, MOL BIOL EVOL, V38, P1241, DOI 10.1093/molbev/msaa281
Zhou H, 2021, CELL, V184, P4380, DOI 10.1016/j.cell.2021.06.008
Zhou H, 2020, CURR BIOL, V30, P2196, DOI 10.1016/j.cub.2020.05.023
Zhou P, 2020, NATURE, V579, P270, DOI [10.1038/s41586-020-2012-7, 10.1038/s41586-020-2951-z]
NR 72
TC 80
Z9 87
U1 3
U2 32
PU OXFORD UNIV PRESS
PI OXFORD
PA GREAT CLARENDON ST, OXFORD OX2 6DP, ENGLAND
SN 1759-6653
J9 GENOME BIOL EVOL
JI Genome Biol. Evol.
PD FEB 4
PY 2022
VL 14
IS 2
AR evac018
DI 10.1093/gbe/evac018
PG 14
WC Evolutionary Biology; Genetics & Heredity
WE Science Citation Index Expanded (SCI-EXPANDED)
SC Evolutionary Biology; Genetics & Heredity
GA 0E5QI
UT WOS:000776735600003
PM 35137080

OA Green Published, Green Accepted, Green Submitted, gold
DA 2024-10-21
ER

PT J
AU Mayer, F
   Brunner, A
AF Mayer, F.
   Brunner, A.
TI Non-neutral evolution of the major histocompatibility complex class II
   gene DRB1 in the sac-winged bat <i>Saccopteryx bilineata</i>
SO HEREDITY
LA English
DT Article
DE MHC class II; DRB locus; balancing selection; intergenic recombination;
   Chiroptera
ID HETEROZYGOTE-EXCESS; BALANCING SELECTION; NATURAL-SELECTION; EFFECTIVE
   NUMBER; MHC; DIVERSITY; POLYMORPHISM; LOCI; RECOMBINATION; ALLELES
AB The immune genes of the major histocompatibility complex (MHC) are classical examples for high levels of genetic diversity and non-neutral evolution. This is particularly true for the regions containing the antigen-binding sites as, for instance, in the exon 2 of the MHC class II gene DRB. We surveyed, for the first time in the order Chiroptera, the genetic diversity within this exon in the sac-winged bat Saccopteryx bilineata. We detected 11 alleles among 85 bats, of which 79 were sampled in one population. Pairwise comparisons revealed that interallelic sequence differences ranged between 3 and 22%, although nucleotide substitutions were not evenly distributed along the exon sequence. This was most probably the result of intragenic recombination. High levels of sequence divergence and significantly more nonsynonymous than synonymous substitutions (d(N)/d(S)> 1) suggest long-term balancing selection. Thus, the data are consistent with the hypothesis that recombination gives rise to new alleles at the DRB locus of the sac-winged bat, and these are maintained in the population through balancing selection. In this respect, the sac-winged bat closely resembles other mammalian species.
C1 Univ Erlangen Nurnberg, Dept Zool, D-91058 Erlangen, Germany.
C3 University of Erlangen Nuremberg
RP Mayer, F (corresponding author), Univ Erlangen Nurnberg, Dept Zool, Staudtstr 5, D-91058 Erlangen, Germany.
EM fmayer@biologie.uni-erlangen.de
CR Aguilar A, 2004, P NATL ACAD SCI USA, V101, P3490, DOI 10.1073/pnas.0306582101
   Amills M, 2004, HEREDITY, V93, P266, DOI 10.1038/sj.hdy.6800499
   ANDERSSON L, 1995, IMMUNOL REV, V143, P5, DOI 10.1111/j.1600-065X.1995.tb00667.x
   Balloux F, 2004, EVOLUTION, V58, P1891
   Bernatchez L, 2003, J EVOLUTION BIOL, V16, P363, DOI 10.1046/j.1420-9101.2003.00531.x

Borghans JAM, 2004, IMMUNOGENETICS, V55, P732, DOI 10.1007/s00251-003-0630-5
BROWN JH, 1993, NATURE, V364, P33, DOI 10.1038/364033a0
Charbonnel N, 2005, HEREDITY, V95, P377, DOI 10.1038/sj.hdy.6800735
Charlesworth D, 2006, PLOS GENET, V2, P379, DOI 10.1371/journal.pgen.0020064
CLARKE B, 1966, NATURE, V211, P999, DOI 10.1038/211999a0
Cliquet F, 2004, REV SCI TECH OIE, V23, P625, DOI 10.20506/rst.23.2.1514
Doherty P C, 1975, Nature, V256, P50
Fooks AR, 2003, EPIDEMIOL INFECT, V131, P1029, DOI 10.1017/S0950268803001481
Froeschke G, 2005, MOL BIOL EVOL, V22, P1254, DOI 10.1093/molbev/msi112
Garrigan D, 2003, EVOLUTION, V57, P1707
GYLLENSTEN U, 1991, J IMMUNOL, V146, P4368
Heckel G, 2003, MOL ECOL, V12, P219, DOI 10.1046/j.1365-294X.2003.01722.x
HEDRICK PW, 1991, P NATL ACAD SCI USA, V88, P5897, DOI 10.1073/pnas.88.13.5897
HEDRICK PW, 1983, GENETICS, V104, P449
HILL AVS, 1994, PHILOS T R SOC B, V346, P379, DOI 10.1098/rstb.1994.0155
Jukes T.H., 1969, MAMMALIAN PROTEIN ME, P21, DOI DOI 10.1093/BIOINFORMATICS/BTM404
Kelley J, 2005, IMMUNOGENETICS, V56, P683, DOI 10.1007/s00251-004-0717-7
Klein J., 1986, NATURAL HIST MAJOR H
Konnai S, 2003, EUR J IMMUNOGENET, V30, P275, DOI 10.1046/j.1365-2370.2003.00399.x
Kumar S, 2004, BRIEF BIOINFORM, V5, P150, DOI 10.1093/bib/5.2.150
Leroy EM, 2005, NATURE, V438, P575, DOI 10.1038/438575a
Li WD, 2005, SCIENCE, V310, P676, DOI 10.1126/science.1118391
Luikart G, 1999, GENETICS, V151, P1211
McClelland EE, 2003, INFECT IMMUN, V71, P2079, DOI 10.1128/IAI.71.4.2079-2086.2003
Mikko S, 1999, IMMUNOL REV, V167, P169, DOI 10.1111/j.1600-065X.1999.tb01390.x
MULLENBACH R, 1989, TRENDS GENET, V5, P391
Nei M, 2005, ANNU REV GENET, V39, P121, DOI 10.1146/annurev.genet.39.073003.112240
NEI M, 1986, MOL BIOL EVOL, V3, P418
Piertney SB, 2006, HEREDITY, V96, P7, DOI 10.1038/sj.hdy.6800724
Pudovkin AI, 1996, GENETICS, V144, P383
RAYMOND M, 1995, J HERED, V86, P248, DOI 10.1093/oxfordjournals.jhered.a111573
Reusch TBH, 2005, J MOL EVOL, V61, P531, DOI 10.1007/s00239-004-0340-0
Richman AD, 2003, GENET RES, V82, P89, DOI 10.1017/S0016672303006347
SAWYER S, 1989, MOL BIOL EVOL, V6, P526
SAWYER S, 1999, MOL BIOL COMPUTER PR
Schad J, 2004, CONSERV GENET, V5, P299, DOI 10.1023/

B:COGE.0000031137.50239.d3
   SCHARF SJ, 1988, HUM IMMUNOL, V22, P61, DOI 10.1016/0198-8859(88)90052-3
   Schaschl H, 2005, IMMUNOGENETICS, V57, P108, DOI 10.1007/s00251-005-0784-4
   Schneider S, 2000, ARLEQUIN SOFTWARE PO
   Sena L, 2003, ANIM GENET, V34, P1, DOI 10.1046/j.1365-2052.2003.00920.x
   SIMMONS NB, 2006, MAMMAL SPECIES WORLD
   Sommer S, 1999, MOL ECOL, V8, P1259, DOI 10.1046/j.1365-294X.1999.00687.x
   Sommer Simone, 2005, Frontiers in Zoology, V2, P1, DOI 10.1186/1742-9994-2-16
   Weber DS, 2004, MOL ECOL, V13, P711, DOI 10.1111/j.1365-294X.2004.02095.x
   Westerdahl H, 2004, J EVOLUTION BIOL, V17, P485, DOI 10.1111/j.1420-9101.2004.00711.x
   Wong S, 2007, REV MED VIROL, V17, P67, DOI 10.1002/rmv.520
   ZANGENBERG G, 1995, NAT GENET, V10, P407, DOI 10.1038/ng0895-407
NR 52
TC 21
Z9 25
U1 0
U2 15
PU SPRINGERNATURE
PI LONDON
PA CAMPUS, 4 CRINAN ST, LONDON, N1 9XW, ENGLAND
SN 0018-067X
EI 1365-2540
J9 HEREDITY
JI Heredity
PD SEP
PY 2007
VL 99
IS 3
BP 257
EP 264
DI 10.1038/sj.hdy.6800989
PG 8
WC Ecology; Evolutionary Biology; Genetics & Heredity
WE Science Citation Index Expanded (SCI-EXPANDED)
SC Environmental Sciences & Ecology; Evolutionary Biology; Genetics &
   Heredity
GA 202QP
UT WOS:000248918400004
PM 17519971
OA Bronze, Green Published
DA 2024-10-21
ER

PT J
AU Lilley, TM
   Stauffer, J

Kanerva, M
   Eeva, T
AF Lilley, T. M.
   Stauffer, J.
   Kanerva, M.
   Eeva, T.
TI Interspecific variation in redox status regulation and immune defence in
   five bat species: the role of ectoparasites
SO OECOLOGIA
LA English
DT Article
DE Chiroptera; Redox status; Oxidative damage; Immune defence;
   Ectoparasites
ID HISTORY TRADE-OFFS; OXIDATIVE STRESS; MYOTIS-DAUBENTONII; LIFE-HISTORY;
   ANTIOXIDANT DEFENSES; PROTEIN; ENZYME; HOST; LONGEVITY; TISSUE
AB Harmful reactive oxygen species (ROS) produced during metabolism and immune responses are neutralized in part by a powerful enzymatic antioxidant system. Inter-species variability in the baseline activity of antioxidant enzymes may be explained by a variety of life history traits. For instance, ectoparasites can elicit repeated immune responses, thus increasing the production of reactive oxygen species. The bat species studied so far have been acknowledged to have effective antioxidant defences. However, interspecific comparisons within the clade do not exist. The present study compares the antioxidant defence and immune function activities in five northern boreal bat species relative to their ectoparasite prevalence and intensity (wing mites and louse flies) to reveal inter-species differences. Antioxidant enzyme and immune defense activities, which differ between species, are positively associated, with total ectoparasite (mites and bat flies) frequencies, total ROS, and protein carbonylation in Daubenton's bats, but enzyme activities are also independently influenced by sampling date with activities increasing towards the autumn. Antioxidant activities are also positively associated with total reactive oxygen species and oxidative damage (protein carbonylation) in the Daubenton's bat. Our results suggest that antioxidant activities are associated with ecological factors such as parasite load and season, and we consider it likely that these may partly explain the observed interspecific variation.
C1 [Lilley, T. M.; Stauffer, J.; Kanerva, M.; Eeva, T.] Univ Turku, Dept Biol, Turku 20014, Finland.
C3 University of Turku
RP Lilley, TM (corresponding author), Univ Turku, Dept Biol, Turku 20014, Finland.
EM tmlill@utu.fi
RI Eeva, Tapio/V-8922-2018; Lilley, Thomas/F-2236-2015
OI Eeva, Tapio/0000-0002-0395-1536; Kanerva, Mirella/0000-0002-2463-2497;
   Lilley, Thomas/0000-0001-5864-4958
FU Maj and Tor Nessling Foundation; Academy of Finland [265859]
FX We thank Anna Kald for contributing to laboratory work. Our study was

financed by Maj and Tor Nessling Foundation (TL) and the Academy of
Finland (TE: project 265859).
CR Alamdari DH, 2005, FREE RADICAL BIO MED, V39, P1362, DOI 10.1016/j.freeradbiomed.2005.06.023
Allen LC, 2009, J COMP PHYSIOL B, V179, P315, DOI 10.1007/s00360-008-0315-3
[Anonymous], 2009, HDB BATS EUROPE NW A
[Anonymous], P BIOL SCI
Aznar-Lopez C, 2013, J GEN VIROL, V94, P69, DOI 10.1099/vir.0.046490-0
BOARD PG, 1981, FEBS LETT, V124, P163, DOI 10.1016/0014-5793(81)80127-5
BRADFORD MM, 1976, ANAL BIOCHEM, V72, P248, DOI 10.1016/0003-2697(76)90527-3
Brunet-Rossinni AK, 2004, MECH AGEING DEV, V125, P11, DOI 10.1016/j.mad.2003.09.003
Chavez J, 2006, ANAL CHEM, V78, P6847, DOI 10.1021/ac0607257
Chung WG, 2008, ELECTROPHORESIS, V29, P1317, DOI 10.1002/elps.200700606
Cohen A, 2007, COMP BIOCHEM PHYS B, V147, P110, DOI 10.1016/j.cbpb.2006.12.015
Costantini D, 2009, COMP BIOCHEM PHYS A, V153, P339, DOI 10.1016/j.cbpa.2009.03.010
Czenze Zenon J., 2011, Journal of Parasitology Research, V2011, DOI 10.1155/2011/341535
DEISSEROTH A, 1970, PHYSIOL REV, V50, P319, DOI 10.1152/physrev.1970.50.3.319
Encarnaçao JA, 2012, ACTA CHIROPTEROL, V14, P153, DOI 10.3161/150811012X654367
Encarnaçao JA, 2012, J ETHOL, V30, P271, DOI 10.1007/s10164-011-0323-8
Ferreira-Cravo M, 2007, CHEMOSPHERE, V66, P1367, DOI 10.1016/j.chemosphere.2006.06.050
Freitas M, 2009, ANAL CHIM ACTA, V649, P8, DOI 10.1016/j.aca.2009.06.063
Giorgi MS, 2001, P ROY SOC B-BIOL SCI, V268, P2071, DOI 10.1098/rspb.2001.1686
HABIG WH, 1974, J BIOL CHEM, V249, P7130
Halliwell B., 2000, FREE RADICAL BIO MED, V3rd
Hill DA, 2005, MAMMAL REV, V35, P116, DOI 10.1111/j.1365-2907.2005.00058.x
Hope PR, 2012, J COMP PHYSIOL B, V182, P569, DOI 10.1007/s00360-011-0631-x
Hornok S, 2012, VET MICROBIOL, V159, P541, DOI 10.1016/j.vetmic.2012.04.003
Jakava-Viljanen M, 2010, EPIDEMIOL INFECT, V138, P1581, DOI 10.1017/S0950268810000373
Kapfer G, 2008, MAMM BIOL, V73, P267, DOI 10.1016/j.mambio.2007.01.001
Kilpi MK, 2009, DEV COMP IMMUNOL, V33, P1102, DOI 10.1016/j.dci.2009.06.007
Koivula MJ, 2011, ENVIRON RES, V111, P362, DOI 10.1016/j.envres.2011.01.005

Krautz GM, 2000, PARASITOL TODAY, V16, P31, DOI 10.1016/S0169-4758(99)01581-1
Lilley TM, 2013, COMP BIOCHEM PHYS C, V157, P298, DOI 10.1016/j.cbpc.2013.01.003
Lucan RK, 2006, FOLIA PARASIT, V53, P147, DOI 10.14411/fp.2006.019
Luis AD, 2013, P ROY SOC B-BIOL SCI, V280, DOI 10.1098/rspb.2012.2753
McLean JA, 1997, ANIM BEHAV, V54, P1193, DOI 10.1006/anbe.1997.0498
Morin PJ, 2007, ARCH BIOCHEM BIOPHYS, V461, P59, DOI 10.1016/j.abb.2007.01.035
Mühldorfer K, 2013, ZOONOSES PUBLIC HLTH, V60, P93, DOI 10.1111/j.1863-2378.2012.01536.x
Munshi-South J, 2010, AGEING RES REV, V9, P12, DOI 10.1016/j.arr.2009.07.006
Murphy K., 2007, JANEWAYS IMMUNOBIOLO
Nikinmaa M., 1990, Vertebrate Red Blood Cells
NOLTMANN E, 1961, J BIOL CHEM, V236, P1225
Norris K, 2000, BEHAV ECOL, V11, P19, DOI 10.1093/beheco/11.1.19
Pamplona R, 2011, AM J PHYSIOL-REG I, V301, pR843, DOI 10.1152/ajpregu.00034.2011
Parsons KN, 2003, ANIM CONSERV, V6, P283, DOI 10.1017/S1367943003003342
Podlutsky AJ, 2005, J GERONTOL A-BIOL, V60, P1366, DOI 10.1093/gerona/60.11.1366
Raja-aho S, 2012, PHYSIOL BIOCHEM ZOOL, V85, P148, DOI 10.1086/664826
Rudneva I. I., 2004, Parazitologiya (St. Petersburg), V38, P557
Schneeberger K, 2013, PLOS ONE, V8, DOI 10.1371/journal.pone.0054023
Senior P, 2005, P ROY SOC B-BIOL SCI, V272, P2467, DOI 10.1098/rspb.2005.3237
Shiono H, 2003, FREE RADICAL RES, V37, P1181, DOI 10.1080/10715760310001607023
SMITH IK, 1988, ANAL BIOCHEM, V175, P408, DOI 10.1016/0003-2697(88)90564-7
SMITH PK, 1985, ANAL BIOCHEM, V150, P76, DOI 10.1016/0003-2697(85)90442-7
Socci DJ, 1999, EXP NEUROL, V155, P109, DOI 10.1006/exnr.1998.6969
Storey KB., 2005, Functional Metabolism: Regulation and Adaptation
Storey KB, 2010, GERONTOLOGY, V56, P220, DOI 10.1159/000228829
Uhrin M, 2010, ACTA THERIOL, V55, P153, DOI 10.4098/j.at.0001-7051.073.2009
Vaughan N, 1997, MAMMAL REV, V27, P77, DOI 10.1111/j.1365-2907.1997.tb00373.x
Vesterinen EJ, 2013, PLOS ONE, V8, DOI 10.1371/journal.pone.0082168
Viarengo A, 1999, AM J PHYSIOL-REG I, V277, pR1612, DOI 10.1152/ajpregu.1999.277.6.R1612
Wermundsen T, 2010, CENT EUR J BIOL, V5, P262, DOI 10.2478/s11535-009-0063-8

   Wikel SK, 1999, BIOSCIENCE, V49, P311, DOI 10.2307/1313614
   Wilhelm D, 2007, COMP BIOCHEM PHYS C, V146, P214, DOI 10.1016/
j.cbpc.2006.11.015
   Wilkinson GS, 2002, AGING CELL, V1, P124, DOI 10.1046/
j.1474-9728.2002.00020.x
   Yang DB, 2013, J EXP BIOL, V216, P4242, DOI 10.1242/jeb.092049
   Zahn A, 2004, J ZOOL, V262, P383, DOI 10.1017/S0952836903004722
NR 63
TC 20
Z9 22
U1 0
U2 54
PU SPRINGER
PI NEW YORK
PA ONE NEW YORK PLAZA, SUITE 4600, NEW YORK, NY, UNITED STATES
SN 0029-8549
EI 1432-1939
J9 OECOLOGIA
JI Oecologia
PD JUL
PY 2014
VL 175
IS 3
BP 811
EP 823
DI 10.1007/s00442-014-2959-x
PG 13
WC Ecology
WE Science Citation Index Expanded (SCI-EXPANDED)
SC Environmental Sciences & Ecology
GA AK1UO
UT WOS:000338202600007
PM 24839093
DA 2024-10-21
ER

PT J
AU Dimitrov, DT
   Hallam, TG
   Rupprecht, CE
   Turmelle, AS
   McCracken, GF
AF Dimitrov, Dobromir T.
   Hallam, Thomas G.
   Rupprecht, Charles E.
   Turmelle, Amy S.
   McCracken, Gary F.
TI Integrative models of bat rabies immunology, epizootiology and disease
   demography
SO JOURNAL OF THEORETICAL BIOLOGY
LA English
DT Article
DE immune system; epizootic model; viral infection; rabies

ID TADARIDA-BRASILIENSIS-MEXICANA; IMMUNE-RESPONSE; UNITED-STATES; VIRUS;
   ANTIBODY
AB Bats are natural reservoirs of rabies. We address the maintenance of the disease in bat colonies by developing individual and population models that generate indicators of risk of rabies to bats, that provide dynamic estimates of effects of rabies on population densities, and that suggest consequences of viral exposures and infections in bats relative to physiological and ecological characteristics of bats in different habitats. We present individual models (within host) for the immune responses to a rabies virus challenge, an immunotypic disease model that describes the evolution of the disease and a disease demographics model, which is structured by immunotypic response governed by immune system efficiency. Model simulations are consistent with available data, characterized by relatively low prevalence of the virus in colonies and much higher prevalence of rabies virus-neutralizing antibodies. Under model conditions, there is a robust non-clinical state that can be attained by the exposed individual that allows persistence of the disease in the population. (c) 2006 Elsevier Ltd. All rights reserved.
C1 Univ Tennessee, Dept Ecol & Evolut Biol, Knoxville, TN 37996 USA.
   Ctr Dis Control & Prevent, Poxvirus & Rabies Branch, Div Viral & Rickettsial Dis, Atlanta, GA 30333 USA.
C3 University of Tennessee System; University of Tennessee Knoxville;
   Centers for Disease Control & Prevention - USA
RP Dimitrov, DT (corresponding author), Univ Tennessee, Dept Ecol & Evolut Biol, 569 Dubney Hall,1416 Circle Dr, Knoxville, TN 37996 USA.
EM ddimitr1@utk.edu; thallam@utk.edu; cyr5@cdc.gov; aturmell@utk.edu;
   gmccrack@utk.edu
RI Rupprecht, Charles/AAI-1544-2020
OI Dimitrov, Dobromir/0000-0002-2842-5436; McCracken,
   Gary/0000-0002-2493-8103; Gilbert, Amy T./0000-0002-8256-0081
CR Aguilar-Setien A, 2005, EPIDEMIOL INFECT, V133, P517, DOI 10.1017/S0950268805003705
   Barclay RMR, 2003, BAT ECOLOGY, P209
   Bona CA., 1996, Textbook of Immunology, V2nd
   BURNS KENNETH F., 1956, JOUR AMER VET MED ASSOC, V128, P27
   BURNS KF, 1955, J INFECT DIS, V97, P211, DOI 10.1093/infdis/97.2.211
   Calisher CH, 2006, CLIN MICROBIOL REV, V19, P531, DOI 10.1128/CMR.00017-06
   CHOWDHURY D, 1993, J THEOR BIOL, V165, P135, DOI 10.1006/jtbi.1993.1180
   Clark Donald R. Jr, 2001, P159
   Cleveland CJ, 2006, FRONT ECOL ENVIRON, V4, P238, DOI 10.1890/1540-9295(2006)004[0238:EVOTPC]2.0.CO;2
   Constantine D.G., 1967, U NEW MEXICO PUBLICA, V7, P1
   CONSTANTINE DG, 1968, PUBLIC HEALTH REP, V83, P303, DOI 10.2307/4593275
   DEAN W D, 1960, Ariz Med, V17, P69

Echevarría JE, 2001, J CLIN MICROBIOL, V39, P3678, DOI 10.1128/JCM.39.10.3678-3683.2001
GIRARD KF, 1965, NEW ENGL J MED, V272, P75, DOI 10.1056/NEJM196501142720204
Hooper DC, 1998, J VIROL, V72, P3711, DOI 10.1128/JVI.72.5.3711-3719.1998
Iwasaki Y., 2002, Rabies, P283
Jackson A.C., 2002, RABIES, P219
Jackson A.C., 2002, Rabies, P246
KAUFMAN M, 1987, J THEOR BIOL, V129, P141, DOI 10.1016/S0022-5193(87)80009-7
KAUFMAN M, 1985, J THEOR BIOL, V114, P527, DOI 10.1016/S0022-5193(85)80042-4
Kot Mark, 2001, ELEMENTS MATH ECOLOG
Krebs JW, 2005, JAVMA-J AM VET MED A, V227, P1912, DOI 10.2460/javma.2005.227.1912
KUNZ TH, 1995, J MAMMAL, V76, P769, DOI 10.2307/1382746
LORD R D, 1975, Journal of Wildlife Diseases, V11, P210
Messenger SL, 2002, CLIN INFECT DIS, V35, P738, DOI 10.1086/342387
Messenger SL, 2003, BAT ECOLOGY, P622
Montgomery D. C., 2001, DESIGN ANAL EXPT
Niezgoda Michael, 2002, P163
PRICE JL, 1977, J WILDLIFE DIS, V13, P131, DOI 10.7589/0090-3558-13.2.131
PYBUS MJ, 1986, J WILDLIFE DIS, V22, P449, DOI 10.7589/0090-3558-22.3.449
STEECE R, 1989, J WILDLIFE DIS, V25, P490, DOI 10.7589/0090-3558-25.4.490
Sulkin S E, 1974, Monogr Virol, V8, P1
NR 32
TC 26
Z9 28
U1 2
U2 18
PU ACADEMIC PRESS LTD- ELSEVIER SCIENCE LTD
PI LONDON
PA 24-28 OVAL RD, LONDON NW1 7DX, ENGLAND
SN 0022-5193
EI 1095-8541
J9 J THEOR BIOL
JI J. Theor. Biol.
PD APR 7
PY 2007
VL 245
IS 3
BP 498
EP 509
DI 10.1016/j.jtbi.2006.11.001
PG 12
WC Biology; Mathematical & Computational Biology
WE Science Citation Index Expanded (SCI-EXPANDED)
SC Life Sciences & Biomedicine - Other Topics; Mathematical & Computational

Biology
GA 156TF
UT WOS:000245672100010
PM 17184793
DA 2024-10-21
ER

PT J
AU Meteyer, CU
Barber, D
Mandl, JN
AF Meteyer, Carol U.
Barber, Daniel
Mandl, Judith N.
TI Pathology in euthermic bats with white nose syndrome suggests a natural
manifestation of immune reconstitution inflammatory syndrome
SO VIRULENCE
LA English
DT Article
DE cave-hibernating bats; Geomyces destructans; hibernation-induced; immune
suppression; immune cell trafficking; immune reconstitution inflammatory
syndrome (IRIS); Myotis lucifugus; white nose syndrome
ID GEOMYCES-DESTRUCTANS; GROUND-SQUIRRELS; HIBERNATION; INFECTION; SYSTEM;
TEMPERATURE; RESPONSES; THERAPY
AB White nose syndrome, caused by Geomyces destructans, has killed more than 5 million cave hibernating bats in eastern North America. During hibernation, the lack of inflammatory cell recruitment at the site of fungal infection and erosion is consistent with a temperature-induced inhibition of immune cell trafficking. This immune suppression allows G. destructans to colonize and erode the skin of wings, ears and muzzle of bat hosts unchecked. Yet, paradoxically, within weeks of emergence from hibernation an intense neutrophilic inflammatory response to G. destructans is generated, causing severe pathology that can contribute to death. We hypothesize that the sudden reversal of immune suppression in bats upon the return to euthermia leads to a form of immune reconstitution inflammatory syndrome (IRIS). IRIS was first described in HIV-infected humans with low helper T lymphocyte counts and bacterial or fungal opportunistic infections. IRIS is a paradoxical and rapid worsening of symptoms in immune compromised humans upon restoration of immunity in the face of an ongoing infectious process. In humans with HIV, the restoration of adaptive immunity following suppression of HIV replication with anti-retroviral therapy (ART) can trigger severe immune-mediated tissue damage that can result in death. We propose that the sudden restoration of immune responses in bats infected with G. destructans results in an IRIS-like dysregulated immune response that causes the post-emergent pathology.
C1 [Meteyer, Carol U.] US Geol Survey, Natl Wildlife Hlth Ctr, Madison, WI USA.

[Barber, Daniel] NIAID, Lymphocyte Biol Unit T, Parasit Dis Lab, NIH, Bethesda, MD 20892 USA.
[Mandl, Judith N.] NIAID, Lymphocyte Biol Sect, Lab Syst Biol, NIH, Bethesda, MD 20892 USA.
C3 United States Department of the Interior; United States Geological Survey; National Institutes of Health (NIH) - USA; NIH National Institute of Allergy & Infectious Diseases (NIAID); National Institutes of Health (NIH) - USA; NIH National Institute of Allergy & Infectious Diseases (NIAID)
RP Mandl, JN (corresponding author), NIAID, Lymphocyte Biol Sect, Lab Syst Biol, NIH, 9000 Rockville Pike, Bethesda, MD 20892 USA.
EM mandlj@niaid.nih.gov
RI Barber, Daniel/V-1190-2019
OI Barber, Daniel/0000-0001-7008-7509
FU US. Geological Survey
FX Authors are grateful to Gregory Turner (Pennsylvania Game Commission), Mick Valent (New Jersey Division of Fish and Wild-life) and Jackie Kashmer (New Jersey Bat Sanctuary) for permission to use the photographs in Figure 1 and Ann Rivers (Acadia Wildlife Foundation) for submitting the Little Brown Bats from Maine. Diagnostic work was funded by the US. Geological Survey. Use of trade or product names does not imply endorsement by the US government.
CR [Anonymous], 2012, North American bat death toll exceeds 5.5 million from white-nose syndrome
Barber DL, 2012, NAT REV MICROBIOL, V10, P150, DOI 10.1038/nrmicro2712
Barber DL, 2010, BLOOD, V116, P3485, DOI 10.1182/blood-2010-05-286336
Blehert DS., 2011, Microbe, V6, P267
Bouma HR, 2010, VET IMMUNOL IMMUNOP, V136, P319, DOI 10.1016/j.vetimm.2010.03.016
Bouma HR, 2011, P NATL ACAD SCI USA, V108, P2052, DOI 10.1073/pnas.1008823108
Bouma HR, 2010, J LEUKOCYTE BIOL, V88, P619, DOI 10.1189/jlb.0310174
Carey HV, 2003, PHYSIOL REV, V83, P1153, DOI 10.1152/physrev.00008.2003
Chaturvedi V, 2010, PLOS ONE, V5, DOI 10.1371/journal.pone.0010783
Cryan PM, 2010, BMC BIOL, V8, DOI 10.1186/1741-7007-8-135
EMMONS RW, 1966, AM J TROP MED HYG, V15, P428, DOI 10.4269/ajtmh.1966.15.428
Fuchs TA, 2007, J CELL BIOL, V176, P231, DOI 10.1083/jcb.200606027
Gargas A, 2009, MYCOTAXON, V108, P147, DOI 10.5248/108.147
Germain RN, 2006, NAT REV IMMUNOL, V6, P497, DOI 10.1038/nri1884

Grossman Z, 2006, NAT MED, V12, P289, DOI 10.1038/nm1380
Gupta AO, 2011, CURR OPIN INFECT DIS, V24, P527, DOI 10.1097/QCO.0b013e32834ab20a
Johnson T, 2011, CURR OPIN NEUROL, V24, P284, DOI 10.1097/WCO.0b013e328346be57
Lochmiller RL, 2000, OIKOS, V88, P87, DOI 10.1034/j.1600-0706.2000.880110.x
Lorch JM, 2011, NATURE, V480, P376, DOI 10.1038/nature10590
Melvin RG, 2009, TRENDS ENDOCRIN MET, V20, P490, DOI 10.1016/j.tem.2009.09.005
Meteyer CU, 2011, J WILDLIFE DIS, V47, P618, DOI 10.7589/0090-3558-47.3.618
Meteyer CU, 2009, J VET DIAGN INVEST, V21, P411, DOI 10.1177/104063870902100401
Miceli MH, 2007, CANCER-AM CANCER SOC, V110, P112, DOI 10.1002/cncr.22738
Miravalle A, 2011, ARCH NEUROL-CHICAGO, V68, P186, DOI 10.1001/archneurol.2010.257
Moore MS, 2011, PLOS ONE, V6, DOI 10.1371/journal.pone.0027430
Neuweiler G, 2000, BIOL BATS, P68
Prendergast BJ, 2002, AM J PHYSIOL-REG I, V282, pR1054, DOI 10.1152/ajpregu.00562.2001
Puechmaille SJ, 2010, EMERG INFECT DIS, V16, P290, DOI 10.3201/eid1602.091391
Reeder DM, 2012, PLOS ONE, V7, DOI 10.1371/journal.pone.0038920
Rivoisy C, 2011, JOINT BONE SPINE, V78, P312, DOI 10.1016/j.jbspin.2011.01.003
Shelburne SA, 2006, J ANTIMICROB CHEMOTH, V57, P167, DOI 10.1093/jac/dki444
Shelburne SA, 2002, MEDICINE, V81, P213, DOI 10.1097/00005792-200205000-00005
SHIVATCHEVA TM, 1988, FOLIA BIOL-KRAKOW, V36, P213
Singh N, 2007, LANCET INFECT DIS, V7, P395, DOI 10.1016/S1473-3099(07)70085-3
Sun HY, 2009, CURR OPIN INFECT DIS, V22, P394, DOI 10.1097/QCO.0b013e32832d7aff
Warnecke L, 2012, P NATL ACAD SCI USA, V109, P6999, DOI 10.1073/pnas.1200374109
Wibbelt G, 2010, EMERG INFECT DIS, V16, P1237, DOI 10.3201/eid1608.100002
Zhang JF, 2006, NATURE, V439, P340, DOI 10.1038/nature04368
Zimmerman R, 2009, SCIENCE, V324, P1134, DOI 10.1126/science.324_1134
NR 39
TC 88
Z9 101
U1 0
U2 95
PU LANDES BIOSCIENCE
PI AUSTIN
PA 1806 RIO GRANDE ST, AUSTIN, TX 78702 USA
SN 2150-5594
J9 VIRULENCE
JI Virulence

PD NOV
PY 2012
VL 3
IS 7
BP 583
EP 588
DI 10.4161/viru.22330
PG 6
WC Immunology; Infectious Diseases; Microbiology
WE Science Citation Index Expanded (SCI-EXPANDED)
SC Immunology; Infectious Diseases; Microbiology
GA 067HU
UT WOS:000313285700006
PM 23154286
OA gold, Green Published
DA 2024-10-21
ER

PT J
AU Pilosof, S
   Korine, C
   Moore, MS
   Krasnov, BR
AF Pilosof, Shai
   Korine, Carmi
   Moore, Marianne S.
   Krasnov, Boris R.
TI Effects of sewage-water contamination on the immune response of a desert
   bat
SO MAMMALIAN BIOLOGY
LA English
DT Article
DE Chiroptera; Immune function; Leukocyte profiles; Sewage pollution;
   Desert
ID COMPLEMENT EVASION; HEAVY-METALS; IMMUNOCOMPETENCE; DIVERSITY;
   COMMUNITY; STRESS; FOREST
AB Environmental pollutants may negatively affect the immune system of animals. Yet, this phenomenon has not been studied thoroughly in terrestrial animals that use polluted water for drinking and/or foraging. We experimentally tested the hypothesis that exposure to sewage water would affect the activation of the immune response in the bat Pipistrellus kuhlii that drinks from bodies of open water. We selected two water sources where bats forage in the Negev desert, Israel: natural springs and a sewage-polluted man-made reservoir. We captured 13 non-reproductive female bats in the vicinity of the natural springs and offered seven of them water from the sewage-polluted source for 30 days (treatment) and the remaining six bats were offered water from the natural spring (control). Consumption of contaminated water did not alter the bactericidal ability of blood plasma or the proportions of monocytes circulating in the blood. However, our data provided evidence that the 30-day treatment can cause a decrease in the relative levels of neutrophils and an

increase in the levels of lymphocytes. Our study provides a first account for the effect of sewage pollution on bat immune response which may be important in desert environments. where water sources are scarce. We suggest hypotheses for future, more focused studies.
(C) 2013 Deutsche Gesellschaft fur Saugetierkunde. Published by Elsevier GmbH. All rights reserved.
C1 [Pilosof, Shai; Korine, Carmi; Krasnov, Boris R.] Ben Gurion Univ Negev, Jacob Blaustein Inst Desert Res, Albert Katz Int Sch Desert Studies, IL-8499000 Midreshet Ben Gurion, Israel.
[Pilosof, Shai; Korine, Carmi; Krasnov, Boris R.] Ben Gurion Univ Negev, Jacob Blaustein Inst Desert Res, Mitrani Dept Desert Ecol, IL-8499000 Midreshet Ben Gurion, Israel.
[Korine, Carmi] Tamar Reg Council, Dead Sea & Arava Sci Ctr, IL-8691000 Neveh Zohar, Israel.
[Moore, Marianne S.] Bucknell Univ, Dept Biol, Lewisburg, PA 17837 USA.
C3 Ben Gurion University; Ben Gurion University; Bucknell University
RP Pilosof, S (corresponding author), Ben Gurion Univ Negev, Jacob Blaustein Inst Desert Res, Sede Boqer Campus, IL-8499000 Midreshet Ben Gurion, Israel.
EM spilosof@post.bgu.ac.il
RI Korine, Carmi/F-4116-2012; Pilosof, Shai/AAX-8055-2020; Krasnov, Boris/F-2234-2012
OI Pilosof, Shai/0000-0003-0430-5568
FU Ministry of Environmental Protection of Israel [6-70-106]; Ministry of
Science and Technology of Israel [1.111]
FX We thank Julie Shapiro for her technical assistance, Dr. Amit Gross for
aid with the water analyses and Prof. Dina Zilberg and Tamar Sinai for
providing access to and help with materials for blood smear preparation.
We are grateful to Dr. Itzick Vatnick for helpful comments on a previous
draft of this manuscript. This study was supported by the Ministry of
Environmental Protection of Israel (6-70-106 to CK and BRK) and the
Ministry of Science and Technology of Israel (1.111 to CK). This is
publication no. 819 of the Mitrani Department of Desert Ecology.
CR Abbott IM, 2009, BIOL CONSERV, V142, P2904, DOI 10.1016/j.biocon.2009.07.012
Anderson EP, 2011, CONSERV BIOL, V25, P30, DOI 10.1111/j.1523-1739.2010.01568.x
[Anonymous], 2010, FUNDAMENTALS VET CLI
[Anonymous], COLOR ATLAS INSTRUCT
[Anonymous], P BAT CONS MIN TECHN
[Anonymous], LME LINEAR NONLINEAR
Barton K., 2015, PACKAGE MUMI MODEL S
Beldomenico PM, 2008, J ANIM ECOL, V77, P984, DOI 10.1111/j.1365-2656.2008.01413.x
Brown B A., 1993, Hematology: Principles and procedures, V6th

Burnham K.P., 1998, Model Selection and Multimodel Inference: A Practical Information-theoretic Approach
Christe P, 2000, ECOL LETT, V3, P207
Christin MS, 2003, ENVIRON TOXICOL CHEM, V22, P1127, DOI 10.1002/etc.5620220522
Conrad PA, 2005, INT J PARASITOL, V35, P1155, DOI 10.1016/j.ijpara.2005.07.002
Cotter SC, 2011, FUNCT ECOL, V25, P186, DOI 10.1111/j.1365-2435.2010.01766.x
Cottontail VM, 2009, PARASITOLOGY, V136, P1133, DOI 10.1017/S0031182009990485
Davis AK, 2008, FUNCT ECOL, V22, P760, DOI 10.1111/j.1365-2435.2008.01467.x
Jones Gareth, 2009, Endangered Species Research, V8, P93, DOI 10.3354/esr00182
Kalcounis-Rueppell MC, 2007, BIOL CONSERV, V138, P120, DOI 10.1016/j.biocon.2007.04.009
Korine C, 2004, J ZOOL, V262, P187, DOI 10.1017/S0952836903004539
Kozul CD, 2009, ENVIRON HEALTH PERSP, V117, P1108, DOI 10.1289/ehp.0800199
Lambris JD, 2008, NAT REV MICROBIOL, V6, P132, DOI 10.1038/nrmicro1824
Maceda-Veiga A, 2010, AQUAT TOXICOL, V100, P229, DOI 10.1016/j.aquatox.2010.07.014
Milla S, 2011, ECOTOXICOLOGY, V20, P305, DOI 10.1007/s10646-010-0588-7
Mitch AA, 2010, WATER RES, V44, P4776, DOI 10.1016/j.watres.2010.05.060
Moore MS, 2011, PLOS ONE, V6, DOI 10.1371/journal.pone.0027430
NAGELKERKE NJD, 1991, BIOMETRIKA, V78, P691, DOI 10.1093/biomet/78.3.691
Nollet L., 2007, Handbook of Water Analysis
Park KJ, 2006, ANIM CONSERV, V9, P259, DOI 10.1111/j.1469-1795.2006.00031.x
Pikula J, 2010, ENVIRON TOXICOL CHEM, V29, P501, DOI 10.1002/etc.80
Pilosof S, 2012, PLOS ONE, V7, DOI 10.1371/journal.pone.0041487
R Development Core Team, 2012, R: A language and environment for statistical computing
Raida MK, 2009, DEV COMP IMMUNOL, V33, P35, DOI 10.1016/j.dci.2008.07.001
Razgour O, 2010, ANIM CONSERV, V13, P505, DOI 10.1111/j.1469-1795.2010.00371.x
Reynolds D. Scott, 2009, P674
Rohr JR, 2010, ENVIRON HEALTH PERSP, V118, P20, DOI 10.1289/ehp.0901164
Schwarzenbach RP, 2006, SCIENCE, V313, P1072, DOI 10.1126/science.1127291
Song WC, 2000, IMMUNOPHARMACOLOGY, V49, P187, DOI 10.1016/S0162-3109(00)80303-3
Suorsa P, 2004, P ROY SOC B-BIOL SCI, V271, P435, DOI 10.1098/rspb.2003.2620
Tieleman BI, 2005, P ROY SOC B-BIOL SCI, V272, P1715, DOI 10.1098/rspb.2005.3155

   Voigt Christian C., 2009, P623
   Weiss DJ., 2010, SCHALMS VET HEMATOLO
   Zipfel PF, 2007, MOL IMMUNOL, V44, P3850, DOI 10.1016/
j.molimm.2007.06.149
   Zipfel PF, 2009, NAT REV IMMUNOL, V9, P729, DOI 10.1038/nri2620
   Zocche JJ, 2010, ENVIRON RES, V110, P684, DOI 10.1016/
j.envres.2010.06.003
   Zuur Alain F., 2009, P1
NR 45
TC 18
Z9 18
U1 2
U2 42
PU SPRINGER HEIDELBERG
PI HEIDELBERG
PA TIERGARTENSTRASSE 17, D-69121 HEIDELBERG, GERMANY
SN 1616-5047
EI 1618-1476
J9 MAMM BIOL
JI Mamm. Biol.
PY 2014
VL 79
IS 3
BP 183
EP 188
DI 10.1016/j.mambio.2013.10.005
PG 6
WC Zoology
WE Science Citation Index Expanded (SCI-EXPANDED)
SC Zoology
GA AI2US
UT WOS:000336714200004
DA 2024-10-21
ER

PT J
AU Rupprecht, CE
   Turmelle, A
   Kuzmin, IV
AF Rupprecht, Charles E.
   Turmelle, Amy
   Kuzmin, Ivan V.
TI A perspective on lyssavirus emergence and perpetuation
SO CURRENT OPINION IN VIROLOGY
LA English
DT Article
ID RABIES VIRUS; IMMUNE EVASION; BAT-LYSSAVIRUS; SANTA-ROSALIA;
   UNITED-STATES; EPIDEMIOLOGY; EVOLUTION; PATHOGENICITY; PHYLOGENY;
   ECOLOGY
AB Rabies is propagated globally by viruses in the Family
Rhabdoviridae, Genus Lyssavirus. These RNA viruses utilize the
mammalian central nervous system as their ultimate niche, and
exploit routine social mechanisms, as well as host behavioral
alterations, to facilitate transmission by neural transport and

innervations of the salivary glands, and ultimately excretion via the saliva, towards circulation thereafter in host populations. All mammals are susceptible to infection, but lyssavirus reservoirs are represented by several species of Carnivore, with viral global diversity and distribution in toto driven by a wide variety of the Chiroptera. Pathogen diversity is maintained by multiple faunas, and facilitated by pronounced host vagility, as exemplified by the ease of routine daily and seasonal movements by bats. Viral 'ensembles', or subpopulations associated with productive transmission events, emerge locally in vivo through a combination of naive host infections in some individuals versus acquired immunity by others, using complex metapopulation dynamics. Enhanced surveillance, improved diagnostics, increased pathogen detection, and an integrated One Health approach, targeting human, domestic animal and wildlife interfaces, provide modern insights to the ecology of bat lyssaviruses to augment future prevention and control.
C1 [Rupprecht, Charles E.; Turmelle, Amy; Kuzmin, Ivan V.] Ctr Dis Control & Prevent, Atlanta, GA 30333 USA.
C3 Centers for Disease Control & Prevention - USA
RP Rupprecht, CE (corresponding author), Ctr Dis Control & Prevent, Atlanta, GA 30333 USA.
EM cyr5@cdc.gov
RI Rupprecht, Charles/AAI-1544-2020
OI Gilbert, Amy T./0000-0002-8256-0081
CR [Anonymous], FIELDS VIROLOGY
[Anonymous], 2007, Rabies
Aréchiga-Ceballos N, 2010, EPIDEMIOL INFECT, V138, P1586, DOI 10.1017/S0950268810000762
BAER GM, 1990, LANCET, V335, P664, DOI 10.1016/0140-6736(90)90454-D
Banyard AC, 2011, ADV VIRUS RES, V79, P239, DOI 10.1016/B978-0-12-387040-7.00012-3
Blanton JD, 2010, JAVMA-J AM VET MED A, V237, P646, DOI 10.2460/javma.237.6.646
Brackney DE, 2010, VIROLOGY, V402, P366, DOI 10.1016/j.virol.2010.03.035
Chen ZG, 2008, HUM MOL GENET, V17, pR84, DOI 10.1093/hmg/ddn104
Chopy D, 2011, J VIROL
Diamond J, 2002, NATURE, V418, P700, DOI 10.1038/nature01019
Dietzgen R. G., 2011, VIRUS TAXONOMY
DIETZSCHOLD B, 1983, P NATL ACAD SCI-BIOL, V80, P70, DOI 10.1073/pnas.80.1.70
Dimitrov DT, 2007, J THEOR BIOL, V245, P498, DOI 10.1016/j.jtbi.2006.11.001
Donzé A, 2010, J COMPUT BIOL, V17, P325, DOI 10.1089/cmb.2009.0172
Freuling CM, 2011, EMERG INFECT DIS, V17, P1519, DOI 10.3201/eid1708.110201
Gaunt MW, 2001, J GEN VIROL, V82, P1867, DOI 10.1099/0022-1317-82-8-1867
George DB, 2011, P NAT ACAD SCI US
GOULD SJ, 1993, NATURE, V366, P223, DOI 10.1038/366223a0
Griffin DE, 2003, NAT REV IMMUNOL, V3, P493, DOI 10.1038/nri1105
Harkess G, 2011, METHODS MOL BIOL, V665, P279, DOI

10.1007/978-1-60761-817-1_16
Hogenhout SA, 2003, TRENDS MICROBIOL, V11, P264, DOI 10.1016/S0966-842X(03)00120-3
Holbrook MR, 2005, J INFECT DIS, V191, P100, DOI 10.1086/426397
Holmes EC, 2010, J MOL BIOL, V400, P271, DOI 10.1016/j.jmb.2010.05.032
Hughes GJ, 2005, J GEN VIROL, V86, P1467, DOI 10.1099/vir.0.80710-0
HUTCHINSON GE, 1959, AM NAT, V93, P145, DOI 10.1086/282070
Kaplan Henry J, 2007, Chem Immunol Allergy, V92, P11, DOI 10.1159/000099237
Kawecki TJ, 1998, AM NAT, V152, P635, DOI 10.1086/286195
Klug BJ, 2011, J WILDLIFE DIS, V47, P64, DOI 10.7589/0090-3558-47.1.64
Kumin IV, 2009, INFECT GENET EVOL, V9, P541, DOI 10.1016/j.meegid.2009.02.005
Lafon M, 2008, DEV BIOLOGICALS, V131, P413
Larson SR, 2010, J INSECT SCI, V10, DOI 10.1673/031.010.11001
Leslie MJ, 2006, EMERG INFECT DIS, V12, P1274, DOI 10.3201/eid1708.051526
Markotter W, 2008, DEV BIOLOGICALS, V131, P317
Masuoka P, 2010, GEOSPATIAL HEALTH, V5, P45, DOI 10.4081/gh.2010.186
Messenger SL, 2002, CLIN INFECT DIS, V35, P738, DOI 10.1086/342387
Moore PR, 2010, ANN TROP MED PARASIT, V104, P613, DOI 10.1179/136485910X12851868779948
Morimoto K, 1996, P NATL ACAD SCI USA, V93, P5653, DOI 10.1073/pnas.93.11.5653
Roy A, 2008, J NEUROVIROL, V14, P401, DOI 10.1080/13550280802235924
Smith GC, 2011, EPIDEMIOL INFECT, V139, P1463, DOI 10.1017/S0950268810002876
Steinhauer DA, 1987, VIROLOGY, V38, P651
Streicker DG, 2010, SCIENCE, V329, P676, DOI 10.1126/science.1188836
Tang K, 2011, INT J EVOL BIOL
Tordo N, 2009, Bull Mem Acad R Med Belg, V164, P7
Turmelle AS, 2010, J GEN VIROL, V91, P2360, DOI 10.1099/vir.0.020073-0
Turmelle AS, 2010, VECTOR-BORNE ZOONOT, V10, P165, DOI 10.1089/vbz.2008.0163
Vázquez-Morón S, 2011, EMERG INFECT DIS, V17, P520, DOI 10.3201/eid1703.100894
Wargo AR, 2011, J VIROL, V85, P3959, DOI 10.1128/JVI.01891-10
Wekerle H, 2006, CURR TOP MICROBIOL, V305, P25, DOI 10.1007/3-540-29714-6_2
Wilson DS, 2007, Q REV BIOL, V82, P327, DOI 10.1086/522809
Wright S, 2006, P NATL ACAD SCI USA, V103, P7718, DOI 10.1073/pnas.0510383103
NR 50
TC 64
Z9 70
U1 1

U2 28
PU ELSEVIER SCI LTD
PI OXFORD
PA THE BOULEVARD, LANGFORD LANE, KIDLINGTON, OXFORD OX5 1GB, OXON,
ENGLAND
SN 1879-6257
J9 CURR OPIN VIROL
JI Curr. Opin. Virol.
PD DEC
PY 2011
VL 1
IS 6
BP 662
EP 670
DI 10.1016/j.coviro.2011.10.014
PG 9
WC Virology
WE Science Citation Index Expanded (SCI-EXPANDED)
SC Virology
GA 051FV
UT WOS:000312112500028
PM 22440925
DA 2024-10-21
ER

PT J
AU Moore, MS
   Reichard, JD
   Murtha, TD
   Nabhan, ML
   Pian, RE
   Ferreira, JS
   Kunz, TH
AF Moore, Marianne S.
   Reichard, Jonathan D.
   Murtha, Timothy D.
   Nabhan, Morgan L.
   Pian, Rachel E.
   Ferreira, Jennifer S.
   Kunz, Thomas H.
TI Hibernating Little Brown Myotis (<i>Myotis lucifugus</i>) Show
Variable
   Immunological Responses to White-Nose Syndrome
SO PLOS ONE
LA English
DT Article
ID OXIDATIVE STRESS; IMMUNE-RESPONSE; EXPERIMENTAL-INFECTION;
   SUPEROXIDE-DISMUTASE; ANTIOXIDANT DEFENSES; BATS; DISEASE;
MECHANISMS;
   PREVENTION; CHIROPTERA
AB White-nose syndrome (WNS) is an emerging infectious disease
devastating hibernating North American bat populations that is
caused by the psychrophilic fungus Geomyces destructans. Previous
histopathological analysis demonstrated little evidence of

inflammatory responses in infected bats, however few studies have compared other aspects of immune function between WNS-affected and unaffected bats. We collected bats from confirmed WNS-affected and unaffected sites during the winter of 2008-2009 and compared estimates of their circulating levels of total leukocytes, total immunoglobulins, cytokines and total antioxidants. Bats from affected and unaffected sites did not differ in their total circulating immunoglobulin levels, but significantly higher leukocyte counts were observed in bats from affected sites and particularly in affected bats with elevated body temperatures (above 20 degrees C). Bats from WNS-affected sites exhibited significantly lower antioxidant activity and levels of interleukin-4 (IL-4), a cytokine that induces T cell differentiation. Within affected sites only, bats exhibiting visible fungal infections had significantly lower antioxidant activity and levels of IL-4 compared to bats without visible fungal infections. Overall, bats hibernating in WNS-affected sites showed immunological changes that may be evident of attempted defense against G. destructans. Observed changes, specifically elevated circulating leukocytes, may also be related to the documented changes in thermoregulatory behaviors of affected bats (i.e. increased frequencies in arousal from torpor). Alterations in immune function may reflect expensive energetic costs associated with these processes and intrinsic qualities of the immunocapability of hibernating bats to clear fungal infections. Additionally, lowered antioxidant activity indicates a possible imbalance in the pro-versus antioxidant system, may reflect oxidative tissue damage, and should be investigated as a contributor to WNS-associated morbidity and mortality.
C1 [Moore, Marianne S.; Reichard, Jonathan D.; Murtha, Timothy D.; Nabhan, Morgan L.; Pian, Rachel E.; Ferreira, Jennifer S.; Kunz, Thomas H.] Boston Univ, Dept Biol, Ctr Ecol & Conservat Biol, Boston, MA 02215 USA.
C3 Boston University
RP Moore, MS (corresponding author), Bucknell Univ, Dept Biol, Lewisburg, PA 17837 USA.
EM msm035@bucknell.edu
OI Murtha, Timothy/0000-0001-5427-9340
FU National Speleological Society; Bat Conservation International; New York
Department of Environmental Conservation; U.S. Fish and Wildlife Service
FX This study was made possible by funding through the National Speleological Society, Bat Conservation International, New York Department of Environmental Conservation and the U.S. Fish and Wildlife Service. The funders had no role in study design, data collection and analysis, decision to publish, or preparation of the manuscript.
CR Allen LC, 2009, J COMP PHYSIOL B, V179, P315, DOI 10.1007/s00360-008-0315-3
[Anonymous], 1969, BATS AM
Apak R, 2004, J AGR FOOD CHEM, V52, P7970, DOI 10.1021/jf048741x
ASHMAN RB, 1993, J INFECT DIS, V168, P511, DOI 10.1093/infdis/168.2.511-a

Bertrand S, 2006, FUNCT ECOL, V20, P1022, DOI 10.1111/j.1365-2435.2006.01191.x
Blehert DS, 2009, SCIENCE, V323, P227, DOI 10.1126/science.1163874
Bouma HR, 2010, VET IMMUNOL IMMUNOP, V136, P319, DOI 10.1016/j.vetimm.2010.03.016
Bouma HR, 2010, J LEUKOCYTE BIOL, V88, P619, DOI 10.1189/jlb.0310174
BRACK V, 1985, CAN J ZOOL, V63, P2952, DOI 10.1139/z85-442
Bratsch S, 2011, DEV COMP IMMUNOL, V35, P421, DOI 10.1016/j.dci.2010.06.004
Butler JE, 2011, DEV COMP IMMUNOL, V35, P273, DOI 10.1016/j.dci.2010.08.011
Carey HV, 2003, PHYSIOL REV, V83, P1153, DOI 10.1152/physrev.00008.2003
CASADEVALL A, 1995, INFECT IMMUN, V63, P4211, DOI 10.1128/IAI.63.11.4211-4218.1995
Courtin F, 2010, VET PATHOL, V47, P214, DOI 10.1177/0300985809358614
Dahlgren C, 1999, J IMMUNOL METHODS, V232, P3, DOI 10.1016/S0022-1759(99)00146-5
Frick WF, 2010, SCIENCE, V329, P679, DOI 10.1126/science.1188594
FRIDOVICH I, 1995, ANNU REV BIOCHEM, V64, P97, DOI 10.1146/annurev.bi.64.070195.000525
Fuller NW, 2011, ECOHEALTH, V8, P154, DOI 10.1007/s10393-011-0705-y
Gargas A, 2009, MYCOTAXON, V108, P147, DOI 10.5248/108.147
Geiser F., 2004, LIFE COLD EVOLUTION, P209
Gharib B, 1999, J HEPATOL, V30, P594, DOI 10.1016/S0168-8278(99)80189-5
GREER DL, 1981, AM J TROP MED HYG, V30, P653, DOI 10.4269/ajtmh.1981.30.653
HALLIWELL B, 1993, AM J CLIN NUTR, V57, P715
HATTEN BA, 1968, J IMMUNOL, V101, P141
HATTEN BA, 1970, J IMMUNOL, V105, P872
Ihara Y, 1999, J NEUROL SCI, V170, P90, DOI 10.1016/S0022-510X(99)00192-6
JAEGER R, 1963, EXPERIENTIA, V19, P596, DOI 10.1007/BF02151011
Jain NC, 1993, ESSENTIALS VET HEMAT
Kelly SA, 1998, ENVIRON HEALTH PERSP, V106, P375, DOI 10.2307/3434064
Kepler TB, 2010, BMC GENOMICS, V11, DOI 10.1186/1471-2164-11-444
Kindt T.J., 2007, Kuby immunology
KOTTS CE, 1987, J ANIM SCI, V64, P615, DOI 10.2527/jas1987.642615x
KRUTZSCH PHILIP H., 1959, JOUR MAMMAL, V40, P547, DOI 10.2307/1376274
Kupper TS, 2004, NAT REV IMMUNOL, V4, P211, DOI 10.1038/nri1310
Langwig KE, 2012, ECOL LETT, V15, P1050, DOI 10.1111/j.1461-0248.2012.01829.x
LOPEZTORRES M, 1991, MECH AGEING DEV, V59, P129, DOI 10.1016/0047-6374(91)90079-F
Lorch JM, 2011, NATURE, V480, P376, DOI 10.1038/nature10590
Lyman C. P., 1970, P301

Martínková N, 2010, PLOS ONE, V5, DOI 10.1371/journal.pone.0013853
MCMURRAY DN, 1979, AM J TROP MED HYG, V28, P1036, DOI 10.4269/ajtmh.1979.28.1036
Meteyer CU, 2009, J VET DIAGN INVEST, V21, P411, DOI 10.1177/104063870902100401
Metnitz PGH, 1999, INTENS CARE MED, V25, P180, DOI 10.1007/s001340050813
Monaghan P, 2009, ECOL LETT, V12, P75, DOI 10.1111/j.1461-0248.2008.01258.x
Moore MS, 2011, PLOS ONE, V6, DOI 10.1371/journal.pone.0027430
Ono J, 2008, CLIN CHIM ACTA, V390, P115, DOI 10.1016/j.cca.2008.01.008
Peterhans E, 1997, J NUTR, V127, pS962, DOI 10.1093/jn/127.5.962S
Pikula J, 2012, J WILDLIFE DIS, V48, P207, DOI 10.7589/0090-3558-48.1.207
Polonelli L, 2000, MED MYCOL, V38, P281, DOI 10.1080/mmy.38.s1.281.292
Postma NS, 1996, PHARM WORLD SCI, V18, P121, DOI 10.1007/BF00717727
Puechmaille SJ, 2011, PLOS ONE, V6, DOI 10.1371/journal.pone.0019167
Puechmaille SJ, 2010, EMERG INFECT DIS, V16, P290, DOI 10.3201/eid1602.091391
Reeder DM, 2012, PLOS ONE, V7, DOI 10.1371/journal.pone.0038920
Reichard JD, 2009, ACTA CHIROPTEROL, V11, P457, DOI 10.3161/150811009X485684
Romani L, 2011, NAT REV IMMUNOL, V11, P275, DOI 10.1038/nri2939
Shoham S, 2005, BRIT J HAEMATOL, V129, P569, DOI 10.1111/j.1365-2141.2005.05397.x
Sorci G, 2009, PHILOS T R SOC B, V364, P71, DOI 10.1098/rstb.2008.0151
Speth C, 2008, MYCOSES, V51, P477, DOI 10.1111/j.1439-0507.2008.01597.x
Sulkin S E, 1974, Monogr Virol, V8, P1
Takeda K, 2003, ANNU REV IMMUNOL, V21, P335, DOI 10.1146/annurev.immunol.21.120601.141126
TESH RB, 1966, AM J TROP MED HYG, V15, P544, DOI 10.4269/ajtmh.1966.15.544
Toien O, 2001, AM J PHYSIOL-REG I, V281, pR572
Turmelle AS, 2010, J GEN VIROL, V91, P2360, DOI 10.1099/vir.0.020073-0
Turmelle AS, 2010, J COMP PHYSIOL B, V180, P1155, DOI 10.1007/s00360-010-0486-6
Turner Gregory G., 2011, Bat Research News, V52, P13
Turner Gregory G., 2009, Bat Research News, V50, P47
Valentine JS, 2005, ANNU REV BIOCHEM, V74, P563, DOI 10.1146/annurev.biochem.72.121801.161647
Warnecke L, 2012, P NATL ACAD SCI USA, V109, P6999, DOI 10.1073/pnas.1200374109
Wibbelt G, 2010, EMERG INFECT DIS, V16, P1237, DOI 10.3201/eid1608.100002
NR 68
TC 49

Z9 57
U1 2
U2 116
PU PUBLIC LIBRARY SCIENCE
PI SAN FRANCISCO
PA 1160 BATTERY STREET, STE 100, SAN FRANCISCO, CA 94111 USA
SN 1932-6203
J9 PLOS ONE
JI PLoS One
PD MAR 20
PY 2013
VL 8
IS 3
AR e58976
DI 10.1371/journal.pone.0058976
PG 9
WC Multidisciplinary Sciences
WE Science Citation Index Expanded (SCI-EXPANDED)
SC Science & Technology - Other Topics
GA 125SW
UT WOS:000317562600047
PM 23527062
OA Green Published, Green Submitted, gold
DA 2024-10-21
ER

PT J
AU Lane, JK
Negash, Y
Randhawa, N
Kebede, N
Wells, H
Ayalew, G
Anthony, SJ
Smith, B
Goldstein, T
Kassa, T
Mazet, JAK
Smith, WA
AF Lane, Jennifer K.
Negash, Yohannes
Randhawa, Nistara
Kebede, Nigatu
Wells, Heather
Ayalew, Girma
Anthony, Simon J.
Smith, Brett
Goldstein, Tracey
Kassa, Tesfu
Mazet, Jonna A. K.
Smith, Woutrina A.
CA PREDICT Consortium
TI Coronavirus and Paramyxovirus Shedding by Bats in a Cave and Buildings

in Ethiopia
SO ECOHEALTH
LA English
DT Article
DE bat; coronavirus; paramyxovirus; Ethiopia; cave; viral shedding
ID RESPIRATORY SYNDROME CORONAVIRUS; VIRUS-INFECTION; SARS-LIKE; IDENTIFICATION; CHIROPTERA; RESERVOIR
AB Bats are important hosts of zoonotic viruses with pandemic potential, including filoviruses, MERS-Coronavirus (CoV), SARS-CoV-1, and likely SARS-CoV-2. Viral infection and transmission among wildlife are dependent on a combination of factors that include host ecology and immunology, life history traits, roosting habitats, biogeography, and external stressors. Between 2016 and 2018, four species of insectivorous bats from a readily accessed roadside cave and buildings in Ethiopia were sampled and tested for viruses using consensus PCR assays for five viral families/genera. Previously identified and novel coronaviruses and paramyxoviruses were identified in 99 of the 589 sampled bats. Bats sampled from the cave site were more likely to test positive for a CoV than bats sampled from buildings; viral shedding was more common in the wet season; and rectal swabs were the most common sample type to test positive. A previously undescribed alphacoronavirus was detected in two bat species from different taxonomic families, sampling interfaces, geographic locations, and years. These findings expand knowledge of the range and diversity of coronaviruses and paramyxoviruses in insectivorous bats in Ethiopia and reinforce that an improved understanding of viral diversity and species-specific shedding dynamics is important for designing informed zoonotic disease surveillance and spillover risk reduction efforts.
C1 [Lane, Jennifer K.; Randhawa, Nistara; Mazet, Jonna A. K.; Smith, Woutrina A.] Univ Calif Davis, Sch Vet Med, One Hlth Inst, 1089 Vet Med Dr,VM3B, Davis, CA 95616 USA.
[Lane, Jennifer K.; Randhawa, Nistara; Mazet, Jonna A. K.; Smith, Woutrina A.] Univ Calif Davis, Sch Vet Med, Karen C Drayer Wildlife Hlth Ctr, 1089 Vet Med Dr,VM3B, Davis, CA 95616 USA.
[Negash, Yohannes; Kebede, Nigatu; Kassa, Tesfu] Addis Ababa Univ, Aklilu Lemma Inst Pathobiol, POB 1176, Addis Ababa, Ethiopia.
[Wells, Heather] Columbia Univ, Dept Ecol Evolut & Environm Biol, New York, NY 10027 USA.
[Ayalew, Girma] Minist Environm Forestry & Climate Change, Ethiopian Wildlife Conservat Author, Addis Ababa, Ethiopia.
[Anthony, Simon J.] Univ Calif Davis, Sch Vet Med, Davis, CA 95616 USA.
[Smith, Brett] Univ Calif Davis, Sch Med, Genome Ctr, Davis, CA 95616 USA.
[Smith, Brett] Univ Calif Davis, Sch Med, Biomed Engn, Davis, CA 95616 USA.
[Goldstein, Tracey] Univ Illinois, Zool Pathol Program, Brookfield, IL 60513 USA.
C3 University of California System; University of California Davis; University of California System; University of California Davis; Addis Ababa University; Columbia University; University of California System;

University of California Davis; University of California System;
University of California Davis; University of California System;
University of California Davis; University of Illinois System
RP Lane, JK; Smith, WA (corresponding author), Univ Calif Davis, Sch Vet Med, One Hlth Inst, 1089 Vet Med Dr,VM3B, Davis, CA 95616 USA.; Lane, JK; Smith, WA (corresponding author), Univ Calif Davis, Sch Vet Med, Karen C Drayer Wildlife Hlth Ctr, 1089 Vet Med Dr,VM3B, Davis, CA 95616 USA.
EM jklane@ucdavis.edu; wasmith@ucdavis.edu
RI Mazet, Jonna/B-4811-2012; Wells, Heather/ABG-2823-2021
OI Mengistu, Girma Ayalew/0009-0006-1291-0388; Mazet, Jonna/0000-0002-8712-5951
FU United States Agency for International Development (USAID) Emerging Pandemic Threats PREDICT project [AID-OAA-A-14-00102]
FX This study was made possible by the generous support of the American people through the United States Agency for International Development (USAID) Emerging Pandemic Threats PREDICT project (cooperative agreement number AID-OAA-A-14-00102). We thank the government of Ethiopia, relevant ministry partners, and the Metehara as well as Bati City Health Bureaus for their permission to undertake this study. The authors also thank the Metehara and Bati residents and Awash National Park Staff for their collaboration during animal sampling and would like to thank Mr. Nega Nigussie, Dr. Yewbanesh Asnake, and Mr. Aklilu Belay for assisting during bat capturing and sampling in the field. The authors thank Mrs. Alexandra Gibson and the entire team in the UC Davis One Health Institute Laboratory for their assistance with viral testing and species barcoding. The contents are the responsibility of the authors and do not necessarily reflect the views of USAID or the United States Government. The sponsor did not play any role in the study design, data collection and analysis, decision to publish, or preparation of the manuscript.
CR Abatzoglou JT, 2018, SCI DATA, V5, DOI 10.1038/sdata.2017.191
Allen T, 2017, NAT COMMUN, V8, DOI 10.1038/s41467-017-00923-8
Amman BR, 2020, NAT COMMUN, V11, DOI 10.1038/s41467-020-14327-8
Amman BR, 2014, EMERG INFECT DIS, V20, P1761, DOI 10.3201/eid2010.140696
Amman BR, 2012, PLOS PATHOG, V8, DOI 10.1371/journal.ppat.1002877
Anthony SJ, 2017, MBIO, V8, DOI [10.1128/mBio.00373-17, 10.1128/mbio.00373-17]
Anthony SJ, 2013, J GEN VIROL, V94, P1028, DOI 10.1099/

vir.0.049759-0
Anthony SJ, 2012, MBIO, V3, DOI 10.1128/mBio.00166-12
Anthony SJ, 2017, VIRUS EVOL, V3, DOI 10.1093/ve/vex012
Anthony SJ, 2015, NAT COMMUN, V6, DOI 10.1038/ncomms9147
Azmy SN, 2012, SCI REP-UK, V2, DOI 10.1038/srep00524
Calisher CH, 2006, CLIN MICROBIOL REV, V19, P531, DOI 10.1128/CMR.00017-06
Changula K, 2018, J INFECT DIS, V218, pS312, DOI 10.1093/infdis/jiy266
Cheung WH, 2008, INT J CLIMATOL, V28, P1723, DOI 10.1002/joc.1623
Corman VM, 2020, EUROSURVEILLANCE, V25, P23, DOI 10.2807/1560-7917.ES.2020.25.3.2000045
Corman VM, 2014, J VIROL, V88, P11297, DOI 10.1128/JVI.01498-14
Corman VM, 2014, J VIROL, V88, P717, DOI 10.1128/JVI.01600-13
Crook JM, 2021, SCI REP-UK, V11, DOI 10.1038/s41598-021-94011-z
Fan Y, 2019, VIRUSES-BASEL, V11, DOI 10.3390/v11030210
Forbes KM, 2019, EMERG INFECT DIS, V25, P955, DOI 10.3201/eid2505.181666
Goldstein T, 2018, NAT MICROBIOL, V3, P1084, DOI 10.1038/s41564-018-0227-2
Han BA, 2016, PLOS NEGLECT TROP D, V10, DOI 10.1371/journal.pntd.0004815
Jang SS, 2020, MICROORGANISMS, V8, DOI 10.3390/microorganisms8060844
Joffrin L, 2022, ROY SOC OPEN SCI, V9, DOI 10.1098/rsos.211600
Johara MY, 2001, EMERG INFECT DIS, V7, P439
Johnson CK, 2020, P ROY SOC B-BIOL SCI, V287, DOI 10.1098/rspb.2019.2736
Johnson CK, 2015, SCI REP-UK, V5, DOI 10.1038/srep14830
Kajihara M, 2019, EMERG INFECT DIS, V25, P1577, DOI 10.3201/eid2508.190268
KARIM KB, 1989, REPROD FERT DEVELOP, V1, P255, DOI 10.1071/RD9890255
Kearse M, 2012, BIOINFORMATICS, V28, P1647, DOI 10.1093/bioinformatics/bts199
KINGDON J., 1974, E AFRICAN MAMMALS AT, VII
Kumakamba C, 2021, PLOS ONE, V16, DOI 10.1371/journal.pone.0236971
Kumar N, 2018, CLIN MICROBIOL REV, V31, DOI 10.1128/CMR.00111-17
Kunz TH, 2011, ANN NY ACAD SCI, V1223, P1, DOI 10.1111/j.1749-6632.2011.06004.x
Kuzmin IV, 2010, EMERG INFECT DIS, V16, P352, DOI 10.3201/eid1602.091269
Latinne Alice, 2020, Nat Commun, V11, P4235, DOI [10.1038/s41467-020-17687-3, 10.1101/2020.05.31.116061]
Lau SKP, 2005, P NATL ACAD SCI USA, V102, P14040, DOI 10.1073/pnas.0506735102
Lau SKP, 2010, J VIROL, V84, P2808, DOI 10.1128/JVI.02219-09
Letko M, 2020, NAT REV MICROBIOL, V18, P461, DOI 10.1038/s41579-020-0394-z
Li WD, 2005, SCIENCE, V310, P676, DOI 10.1126/science.1118391
Ludecke D., 2020, J OPEN SOURCE SOFTW, V5, P2445, DOI [DOI 10.21105/JOSS.02445, 10.21105/JOSS.02445, 10.21105/joss.02445]
Maganga GD, 2020, SCI REP-UK, V10, DOI 10.1038/s41598-020-64159-1

Markotter W, 2019, TROP MED INFECT DIS, V4, DOI 10.3390/tropicalmed4030099
Memish ZA, 2013, EMERG INFECT DIS, V19, P1819, DOI 10.3201/eid1911.131172
MENZEL M.A., 2002, Illustrated key to skins and skulls of bats in the southeastern and Mid-Atlantic states
Minh BQ, 2020, MOL BIOL EVOL, V37, P1530, DOI 10.1093/molbev/msaa015
Montecino-Latorre D, 2020, ONE HEALTH OUTLOOK, V2, DOI 10.1186/s42522-019-0008-8
Moureau G, 2007, VECTOR-BORNE ZOONOT, V7, P467, DOI 10.1089/vbz.2007.0206
[Newman S.H. Food and Agriculture Organisation of the United Nations Food and Agriculture Organisation of the United Nations], 2011, FAO Animal Production and Health Manual No. 12
Noh JY, 2018, SCI REP-UK, V8, DOI 10.1038/s41598-018-30319-7
Nziza J, 2020, ECOHEALTH, V17, P152, DOI 10.1007/s10393-019-01458-8
Paweska JT, 2018, EMERG INFECT DIS, V24, P1134, DOI 10.3201/eid2406.172165
Plowright RK, 2017, NAT REV MICROBIOL, V15, P502, DOI 10.1038/nrmicro.2017.45
Pourrut X, 2009, BMC INFECT DIS, V9, DOI 10.1186/1471-2334-9-159
PREDICT Consortium, 2016, PREDICT OP PROC 5 2
Quan PL, 2010, MBIO, V1, DOI 10.1128/mBio.00208-10
QUMSIYEH M B, 1986, Mammalian Species, P1, DOI 10.2307/3503821
*R COR TEAM, 2019, R LANG ENV STAT COMP
Smithers ReayH. N., 1983, MAMMALS SO AFRICAN S
The Nature and Biodiversity Conservation Union (NABU), 2017, NABUS BIODIVERSITY A
Tong S, 2008, J CLIN MICROBIOL, V46, P2652, DOI 10.1128/JCM.00192-08
Tong SX, 2009, EMERG INFECT DIS, V15, P482, DOI 10.3201/eid1503.081013
Towner JS, 2007, PLOS ONE, V2, DOI 10.1371/journal.pone.0000764
Towner JS, 2009, PLOS PATHOG, V5, DOI 10.1371/journal.ppat.1000536
Townzen JS, 2008, MED VET ENTOMOL, V22, P386, DOI 10.1111/j.1365-2915.2008.00760.x
Vijaykrishna D, 2007, J VIROL, V81, P4012, DOI 10.1128/JVI.02605-06
Voigt C.C., 2016, Bats in the Anthropocene: Conservation of Bats in a Changing World, P606
Wacharapluesadee S, 2021, NAT COMMUN, V12, DOI 10.1038/s41467-021-21240-1
Wacharapluesadee S, 2015, VIROL J, V12, DOI 10.1186/s12985-015-0289-1
Waruhiu C, 2017, VIROL SIN, V32, P101, DOI 10.1007/s12250-016-3930-2
Watanabe S, 2010, EMERG INFECT DIS, V16, P1217, DOI 10.3201/eid1608.100208
Wells HL, 2021, VIRUS EVOL, V7, DOI 10.1093/ve/veab007
Wickham H., 2016, ggplot2: Elegant Graphics for Data Analysis, DOI 10.1007/978-3-319-24277-4

   Wickham H., 2021, tidyr: Tidy Messy Data. R
   Woo PCY, 2006, VIROLOGY, V351, P180, DOI 10.1016/
j.virol.2006.02.041
   Zhai JH, 2007, J CLIN MICROBIOL, V45, P224, DOI 10.1128/
JCM.01893-06
   Zhou P, 2020, NATURE, V579, P270, DOI [10.1038/s41586-020-2012-7,
10.1038/s41586-020-2951-z]
NR 77
TC 5
Z9 5
U1 0
U2 8
PU SPRINGER
PI NEW YORK
PA ONE NEW YORK PLAZA, SUITE 4600, NEW YORK, NY, UNITED STATES
SN 1612-9202
EI 1612-9210
J9 ECOHEALTH
JI EcoHealth
PD JUN
PY 2022
VL 19
IS 2
BP 216
EP 232
DI 10.1007/s10393-022-01590-y
EA JUN 2022
PG 17
WC Biodiversity Conservation; Ecology; Environmental Sciences
WE Science Citation Index Expanded (SCI-EXPANDED)
SC Biodiversity & Conservation; Environmental Sciences & Ecology
GA 2X0KJ
UT WOS:000819273100001
PM 35771308
OA hybrid, Green Published
DA 2024-10-21
ER

PT J
AU Hawkins, JA
   Kaczmarek, ME
   Müller, MA
   Drosten, C
   Press, WH
   Sawyer, SL
AF Hawkins, John A.
   Kaczmarek, Maria E.
   Mueller, Marcel A.
   Drosten, Christian
   Press, William H.
   Sawyer, Sara L.
TI A metaanalysis of bat phylogenetics and positive selection based
on
   genomes and transcriptomes from 18 species

SO PROCEEDINGS OF THE NATIONAL ACADEMY OF SCIENCES OF THE UNITED STATES OF
   AMERICA
LA English
DT Article
DE Chiroptera; phylogenetics; transcriptome; gene alignment; orthologous
   genes
ID MOLECULAR PHYLOGENETICS; PHYLOGENOMIC ANALYSES; ADAPTIVE EVOLUTION;
   FRUIT BATS; FLIGHT; HOST; BIOGEOGRAPHY; CHIROPTERA; GENERATION;
   EXPRESSION
AB Historically, the evolution of bats has been analyzed using a small number of genetic loci for many species or many genetic loci for a few species. Here we present a phylogeny of 18 bat species, each of which is represented in 1,107 orthologous gene alignments used to build the tree. We generated a transcriptome sequence of Hypsignathus monstrosus, the African hammer-headed bat, and additional transcriptome sequence for Rousettus aegyptiacus, the Egyptian fruit bat. We then combined these data with existing genomic and transcriptomic data from 16 other bat species. In the analysis of such datasets, there is no clear consensus on the most reliable computational methods for the curation of quality multiple sequence alignments since these public datasets represent multiple investigators and methods, including different source materials (chromosomal DNA or expressed RNA). Here we lay out a systematic analysis of parameters and produce an advanced pipeline for curating orthologous gene alignments from combined transcriptomic and genomic data, including a software package: the Mismatching Isoform eXon Remover (MIXR). Using this method, we created alignments of 11,677 bat genes, 1,107 of which contain orthologs from all 18 species. Using the orthologous gene alignments created, we assessed bat phylogeny and also performed a holistic analysis of positive selection acting in bat genomes. We found that 181 genes have been subject to positive natural selection. This list is dominated by genes involved in immune responses and genes involved in the production of collagens.
C1 [Hawkins, John A.; Press, William H.] Univ Texas Austin, Inst Computat Engn & Sci, Austin, TX 78712 USA.
   [Hawkins, John A.; Press, William H.] Univ Texas Austin, Inst Cellular & Mol Biol, Austin, TX 78712 USA.
   [Kaczmarek, Maria E.; Press, William H.] Univ Texas Austin, Dept Integrat Biol, Austin, TX 78712 USA.
   [Kaczmarek, Maria E.; Sawyer, Sara L.] Univ Colorado, BioFrontiers Inst, Boulder, CO 80303 USA.
   [Mueller, Marcel A.; Drosten, Christian] Charite Univ Med Berlin, Inst Virol, D-10117 Berlin, Germany.
   [Mueller, Marcel A.; Drosten, Christian] Charite Univ Med Berlin, German Ctr Infect Res DZIF, D-10117 Berlin, Germany.
   [Mueller, Marcel A.] Sechenov Univ, Martsinovsky Inst Med Parasitol Trop & Vector Bor, Moscow 119991, Russia.
   [Sawyer, Sara L.] Univ Colorado, Dept Mol Cellular & Dev Biol, Boulder, CO 80303 USA.
C3 University of Texas System; University of Texas Austin;

University of
Texas System; University of Texas Austin; University of Texas System;
University of Texas Austin; University of Colorado System; University of
Colorado Boulder; Berlin Institute of Health; Berlin Institute of
Health; Free University of Berlin; Humboldt University of Berlin;
Charite Universitatsmedizin Berlin; Berlin Institute of Health; Berlin
Institute of Health; Free University of Berlin; Humboldt University of
Berlin; Charite Universitatsmedizin Berlin; German Center for Infection
Research; Sechenov First Moscow State Medical University; University of
Colorado System; University of Colorado Boulder
RP Press, WH (corresponding author), Univ Texas Austin, Inst
Computat Engn & Sci, Austin, TX 78712 USA.; Press, WH (corresponding
author), Univ Texas Austin, Inst Cellular & Mol Biol, Austin, TX
78712 USA.; Press, WH (corresponding author), Univ Texas Austin,
Dept Integrat Biol, Austin, TX 78712 USA.; Sawyer, SL (corresponding
author), Univ Colorado, BioFrontiers Inst, Boulder, CO 80303 USA.;
Sawyer, SL (corresponding author), Univ Colorado, Dept Mol Cellular
& Dev Biol, Boulder, CO 80303 USA.
EM wpress@cs.utexas.edu; ssawyer@colorado.edu
RI Müller, Marcel/O-2425-2019; Drosten, Christian/AFF-8529-2022
OI Press, William/0000-0003-0771-0841; Mueller, Marcel/0000-0003-2242-5117;
SAWYER, SARA/0000-0002-6965-1085
FU NIH [R01-AI-137011]; DFG grant SPP 1596 [DR 772/10-2]; RAPID consortium
of the Bundesministerium fur Bildung und Forschung [01KI1723A]; National
Science Foundation Graduate Research Fellowship
FX We thank the Texas Advanced Computing Center for hundreds of thousands
of hours of compute time. Thanks to Claire Hemingway, Kathleen Lyons,
Siavash Mirarab, Susan Tsang, and Qing Yang for valuable insights into
the project and manuscript. This work was supported by NIH grant
R01-AI-137011 (to S.L.S.), DFG grant SPP 1596 (DR 772/10-2; to C.D.),
and the RAPID consortium of the Bundesministerium fur Bildung und
Forschung (01KI1723A; to C.D.). M.E.K. is supported by a National
Science Foundation Graduate Research Fellowship, and S.L.S. is a
Burroughs Wellcome Investigator in the Pathogenesis of Infectious
Disease.
CR Agnarsson I, 2011, PLOS CURR-TREE LIFE, DOI 10.1371/currents.RRN1212
Altschul SF, 1997, NUCLEIC ACIDS RES, V25, P3389, DOI 10.1093/nar/25.17.3389
Amador LI, 2018, J MAMM EVOL, V25, P37, DOI 10.1007/s10914-016-9363-8

Arroyo-Cabrales J., 2008, MYOTIS LUCIFUGUS IUC
Baker ML, 2013, ZOONOSES PUBLIC HLTH, V60, P104, DOI 10.1111/j.1863-2378.2012.01528.x
Banerjee A, 2018, VIRUS RES, V248, P5, DOI 10.1016/j.virusres.2018.02.013
Bolger AM, 2014, BIOINFORMATICS, V30, P2114, DOI 10.1093/bioinformatics/btu170
Cagan A, 2016, MOL BIOL EVOL, V33, P3268, DOI 10.1093/molbev/msw215
Calisher CH, 2006, CLIN MICROBIOL REV, V19, P531, DOI 10.1128/CMR.00017-06
Ceballos-Vasquez A, 2015, BIOL OPEN, V4, P95, DOI 10.1242/bio.201410264
Chen Y, 2013, BIOCHEM GENET, V51, P7, DOI 10.1007/s10528-012-9540-2
Cheney JA, 2017, J ANAT, V230, P510, DOI 10.1111/joa.12580
Csorba G., 2008, MYOTIS PILOSUS IUCN
Dalquen DA, 2013, GENOME BIOL EVOL, V5, P1800, DOI 10.1093/gbe/evt132
Daugherty MD, 2012, ANNU REV GENET, V46, P677, DOI 10.1146/annurev-genet-110711-155522
Demogines A, 2012, J VIROL, V86, P6350, DOI 10.1128/JVI.00311-12
Dong D, 2013, BMC GENOMICS, V14, DOI 10.1186/1471-2164-14-916
Eddy S.R., 2003, HMMER USERS GUIDE
Eick GN, 2005, MOL BIOL EVOL, V22, P1869, DOI 10.1093/molbev/msi180
Escalera-Zamudio M, 2015, MOL ECOL, V24, P5899, DOI 10.1111/mec.13431
Fang L, 2014, BIOCHEM GENET, V52, P443, DOI 10.1007/s10528-014-9659-4
Francis WR, 2013, BMC GENOMICS, V14, DOI 10.1186/1471-2164-14-167
Francischetti IMB, 2013, J PROTEOMICS, V82, P288, DOI 10.1016/j.jprot.2013.01.009
Fuchs J, 2017, J VIROL, V91, DOI [10.1128/JVI.00361-17, 10.1128/jvi.00361-17]
Fushan AA, 2015, AGING CELL, V14, P352, DOI 10.1111/acel.12283
Grabherr MG, 2011, NAT BIOTECHNOL, V29, P644, DOI 10.1038/nbt.1883
Gracheva EO, 2011, NATURE, V476, P88, DOI 10.1038/nature10245
Harris RS., 2007, IMPROVED PAIRWISE AL
Hayward JA, 2018, MOL BIOL EVOL, V35, P1626, DOI 10.1093/molbev/msy048
He GM, 2010, BIOCHEM GENET, V48, P840, DOI 10.1007/s10528-010-9365-9
Hoofer SR, 2003, J MAMMAL, V84, P809, DOI 10.1644/BWG-034
Hoofer SR, 2003, ACTA CHIROPTEROL, V5, P1, DOI 10.3161/001.005.s101
Huelsenbeck JP, 2001, BIOINFORMATICS, V17, P754, DOI 10.1093/bioinformatics/17.8.754
Hutson A. M., 2008, IUCN RED LIST THREAT
Jarvis ED, 2014, SCIENCE, V346, P1320, DOI 10.1126/science.1253451
Jiang HY, 2017, IMMUNOGENETICS, V69, P271, DOI 10.1007/s00251-016-0966-2

Jones KE, 2002, BIOL REV, V77, P223, DOI 10.1017/S1464793101005899
Kammerer R, 2017, BMC GENOMICS, V18, DOI 10.1186/s12864-017-4106-7
Katoh Kazutaka, 2013, Mol Biol Evol, V30, P772, DOI 10.1093/molbev/mst010
KIMURA M, 1977, NATURE, V267, P275, DOI 10.1038/267275a0
Kühl A, 2011, J INFECT DIS, V204, pS840, DOI 10.1093/infdis/jir306
Larkin MA, 2007, BIOINFORMATICS, V23, P2947, DOI 10.1093/bioinformatics/btm404
Lee AK, 2015, BMC GENOMICS, V16, DOI 10.1186/s12864-015-2124-x
Lei M, 2016, SCI REP-UK, V6, DOI 10.1038/srep27726
Lei M, 2014, PLOS ONE, V9, DOI 10.1371/journal.pone.0107746
Leroy EM, 2005, NATURE, V438, P575, DOI 10.1038/438575a
Leroy EM, 2004, J INFECT DIS, V190, P1895, DOI 10.1086/425421
Li G, 2008, P NATL ACAD SCI USA, V105, P13959, DOI 10.1073/pnas.0802097105
Li G, 2007, PLOS ONE, V2, DOI 10.1371/journal.pone.0000900
Liang L, 2013, PLOS ONE, V8, DOI 10.1371/journal.pone.0065944
Low DHW, 2013, J PROTEOMICS, V89, P95, DOI 10.1016/j.jprot.2013.05.034
Mandl JN, 2018, FRONT IMMUNOL, V9, DOI 10.3389/fimmu.2018.02112
Martin JA, 2011, NAT REV GENET, V12, P671, DOI 10.1038/nrg3068
McBee RM, 2015, MOL BIOL EVOL, V32, P1091, DOI 10.1093/molbev/msu399
Meyerson NR, 2011, TRENDS MICROBIOL, V19, P286, DOI 10.1016/j.tim.2011.03.006
Mirarab S, 2014, BIOINFORMATICS, V30, pI541, DOI 10.1093/bioinformatics/btu462
O'Donoghue AJ, 2015, P NATL ACAD SCI USA, V112, P7478, DOI 10.1073/pnas.1507082112
Olival KJ, 2017, NATURE, V546, P646, DOI 10.1038/nature22975
Papenfuss AT, 2012, BMC GENOMICS, V13, DOI 10.1186/1471-2164-13-261
Pavlovich SS, 2018, CELL, V173, P1098, DOI 10.1016/j.cell.2018.03.070
Pearson William R, 2013, Curr Protoc Bioinformatics, V43, DOI 10.1002/0471250953.bi0305s43
Phillips CD, 2015, FRONT ECOL EVOL, V3, DOI 10.3389/fevo.2015.00122
Phillips CJ, 2014, PLOS ONE, V9, DOI 10.1371/journal.pone.0083512
Rambaut A., FIGTREE GRAPHICAL VI
Ricard-Blum S, 2011, CSH PERSPECT BIOL, V3, DOI 10.1101/cshperspect.a004978
Robertson G, 2010, NAT METHODS, V7, P909, DOI 10.1038/nmeth.1517
Rojas D, 2016, SYST BIOL, V65, P432, DOI 10.1093/sysbio/syw011
Roux J, 2014, MOL BIOL EVOL, V31, P1661, DOI 10.1093/molbev/msu141
Schad J, 2016, IMMUNOGENETICS, V68, P783, DOI 10.1007/s00251-016-0940-z
Schowalter T, 2017, FORESTS, V8, DOI 10.3390/f8040097
Shaw TI, 2012, PLOS ONE, V7, DOI 10.1371/journal.pone.0048472
Shen B, 2013, PLOS ONE, V8, DOI 10.1371/journal.pone.0062307

Shen B, 2012, PLOS ONE, V7, DOI 10.1371/journal.pone.0033197
Shen YY, 2010, P NATL ACAD SCI USA, V107, P8666, DOI 10.1073/pnas.0912613107
Shi JJ, 2015, EVOLUTION, V69, P1528, DOI 10.1111/evo.12681
Smith A. T., 2008, MYOTIS DAVIDII IUCN
Smith I, 2013, CURR OPIN VIROL, V3, P84, DOI 10.1016/j.coviro.2012.11.006
Springer MS, 2001, P NATL ACAD SCI USA, V98, P6241, DOI 10.1073/pnas.111551998
Stamatakis A, 2014, BIOINFORMATICS, V30, P1312, DOI 10.1093/bioinformatics/btu033
Stubbe M., 2016, IUCN RED LIST THREAT
Suyama M, 2006, NUCLEIC ACIDS RES, V34, pW609, DOI 10.1093/nar/gkl315
Teeling EC, 2005, SCIENCE, V307, P580, DOI 10.1126/science.1105113
Towner JS, 2007, PLOS ONE, V2, DOI 10.1371/journal.pone.0000764
Towner JS, 2009, PLOS PATHOG, V5, DOI 10.1371/journal.ppat.1000536
Townsley MI, 2012, COMPR PHYSIOL, V2, P675, DOI 10.1002/cphy.c100081
Tsagkogeorga G, 2013, CURR BIOL, V23, P2262, DOI 10.1016/j.cub.2013.09.014
van der Lee R, 2017, NUCLEIC ACIDS RES, V45, P10634, DOI 10.1093/nar/gkx704
von Mering C, 2005, NUCLEIC ACIDS RES, V33, pD433, DOI 10.1093/nar/gki005
Wang Z, 2014, P ROY SOC B-BIOL SCI, V281, DOI 10.1098/rspb.2013.3133
Wetterer AL, 2000, B AM MUS NAT HIST, P4, DOI 10.1206/0003-0090(2000)248<0001:POPBMC>2.0.CO;2
Wu LJ, 2013, J VIROL, V87, P503, DOI 10.1128/JVI.02332-12
Xiao J, 2015, FISH SHELLFISH IMMUN, V44, P60, DOI 10.1016/j.fsi.2015.01.022
Yang ZH, 2000, TRENDS ECOL EVOL, V15, P496, DOI 10.1016/S0169-5347(00)01994-7
Yang ZH, 2007, MOL BIOL EVOL, V24, P1586, DOI 10.1093/molbev/msm088
Yang ZH, 2012, NAT REV GENET, V13, P303, DOI 10.1038/nrg3186
Yin QY, 2016, PLOS ONE, V11, DOI 10.1371/journal.pone.0146274
Yuan LH, 2011, PLOS ONE, V6, DOI 10.1371/journal.pone.0027189
Zhang GJ, 2013, SCIENCE, V339, P456, DOI 10.1126/science.1230835
Zhou P, 2016, P NATL ACAD SCI USA, V113, P2696, DOI 10.1073/pnas.1518240113
Zhou YY, 2009, BIOCHEM GENET, V47, P207, DOI 10.1007/s10528-008-9218-y
NR 100
TC 48
Z9 50
U1 0
U2 29
PU NATL ACAD SCIENCES
PI WASHINGTON
PA 2101 CONSTITUTION AVE NW, WASHINGTON, DC 20418 USA

SN 0027-8424
EI 1091-6490
J9 P NATL ACAD SCI USA
JI Proc. Natl. Acad. Sci. U. S. A.
PD JUN 4
PY 2019
VL 116
IS 23
BP 11351
EP 11360
DI 10.1073/pnas.1814995116
PG 10
WC Multidisciplinary Sciences
WE Science Citation Index Expanded (SCI-EXPANDED)
SC Science & Technology - Other Topics
GA IB2ZA
UT WOS:000470136000045
PM 31113885
OA Green Published, hybrid
DA 2024-10-21
ER

PT J
AU Moreno, KR
Weinberg, M
Harten, L
Ramos, VSB
Herrera, MLG
Czirják, GA
Yovel, Y
AF Moreno, Kelsey R.
Weinberg, Maya
Harten, Lee
Salinas Ramos, Valeria B.
Herrera M., L. Gerardo
Czirjak, Gabor a.
Yovel, Yossi
TI Sick bats stay home alone: fruit bats practice social distancing when
faced with an immunological challenge
SO ANNALS OF THE NEW YORK ACADEMY OF SCIENCES
LA English
DT Article
DE sickness behavior; immune response; social behavior; foraging;
chiroptera
ID LAND-USE; BEHAVIOR; INFECTION; FEVER; BACTERIAL; ANOREXIA; STRESS; MICE;
LIPOPOLYSACCHARIDE; TRANSMISSION
AB Along with its many advantages, social roosting imposes a major risk of pathogen transmission. How social animals reduce this risk is poorly documented. We used lipopolysaccharide challenge to imitate bacterial infection in both a captive and a free-living colony of an extremely social, long-lived mammal-the Egyptian fruit bat. We monitored behavioral and physiological responses using an

arsenal of methods, including onboard GPS to track foraging, acceleration sensors to monitor movement, infrared video to record social behavior, and blood samples to measure immune markers. Sick-like (immune-challenged) bats exhibited an increased immune response, as well as classic illness symptoms, including fever, weight loss, anorexia, and lethargy. Notably, the bats also exhibited behaviors that would reduce pathogen transfer. They perched alone and appeared to voluntarily isolate themselves from the group by leaving the social cluster, which is extremely atypical for this species. The sick-like individuals in the open colony ceased foraging outdoors for at least two nights, thus reducing transmission to neighboring colonies. Together, these sickness behaviors demonstrate a strong, integrative immune response that promotes recovery of infected individuals while reducing pathogen transmission inside and outside the roost, including spillover events to other species, such as humans.
C1 [Moreno, Kelsey R.; Weinberg, Maya; Harten, Lee; Yovel, Yossi] Tel Aviv Univ, Dept Zool, IL-6997801 Tel Aviv, Israel.
[Salinas Ramos, Valeria B.] Univ Naples Federico II, Dept Agr, Naples, Italy.
[Salinas Ramos, Valeria B.; Herrera M., L. Gerardo] Univ Nacl Autonoma Mexico, Inst Biol, Estn Biol Chamela, Mexico City, DF, Mexico.
[Czirjak, Gabor a.] Leibniz Inst Zoo & Wildlife Res, Dept Wildlife Dis, Berlin, Germany.
[Yovel, Yossi] Tel Aviv Univ, Sagol Sch Neurosci, Tel Aviv, Israel.
C3 Tel Aviv University; University of Naples Federico II; Universidad
Nacional Autonoma de Mexico; Leibniz Institut fur Zoo und Wildtierforschung; Tel Aviv University
RP Moreno, KR (corresponding author), Tel Aviv Univ, Dept Zool, IL-6997801 Tel Aviv, Israel.
EM Kelsey.R.Moreno@gmail.com
RI Czirják, Gábor/F-5440-2011; Salinas-Ramos, Valeria/AAK-3597-2021
OI Salinas-Ramos, Valeria B./0000-0002-3854-6637; Yovel,
Yossi/0000-0001-5429-9245; Moreno, Kelsey/0000-0002-0890-0682; Weinberg,
Maya/0000-0003-1156-3223; Czirjak, Gabor Arpad/
0000-0001-9488-0069
FU Zuckerman STEM Leadership Program; Leibniz Institute for Zoo and
Wildlife Research, Berlin; Consejo Nacional de Ciencia y Tecnologia
[237774]; Israel Science Foundation [677/17]
FX We are grateful to Katja Pohle for her crucial help with the laboratory
analysis. We thank Linoy Alkalay, Eidan Loushi, Liad Pinchasi, and Nofar
Gergrood for their contributions to processing many hours of video,
SharonKrelenstein andNoamGolan for counting WBCs, and Reut Assa for bat
handling assistance. We appreciate the technical assistance of Aya

Goldstein with the Vespers and Ofri Eitan with the cameras. Thanks to
Zohar Tal for helping rear the pups used in the open colony portion of
the experiment. K.R.M. was supported by the Zuckerman STEM Leadership
Program. G.A.C. was supported by funds from the Leibniz Institute for
Zoo and Wildlife Research, Berlin. L.G.H.M. was funded by Consejo
Nacional de Ciencia y Tecnologia (#237774). This research was supported
by the Israel Science Foundation, Grant number 677/17.
CR Abdel-Rachied H.G., 2014, Journal of Entomology and Zoology Studies, V2, P153
Adelman JS, 2014, INTEGR COMP BIOL, V54, P377, DOI 10.1093/icb/icu088
Adelman JS, 2010, J EXP BIOL, V213, P4099, DOI 10.1242/jeb.049528
Anderson RM, 2020, LANCET, V395, P931, DOI 10.1016/S0140-6736(20)30567-5
Arakawa H, 2010, HORM BEHAV, V57, P330, DOI 10.1016/j.yhbeh.2010.01.002
Barclay RMR, 2017, J MAMMAL, V98, P572, DOI 10.1093/jmammal/gyw234
BAUMANN H, 1994, IMMUNOL TODAY, V15, P74, DOI 10.1016/0167-5699(94)90137-6
Boillat M, 2015, CURR BIOL, V25, P251, DOI 10.1016/j.cub.2014.11.061
Bonneaud C, 2003, AM NAT, V161, P367, DOI 10.1086/346134
Bos N, 2012, J EVOLUTION BIOL, V25, P342, DOI 10.1111/j.1420-9101.2011.02425.x
Cabrera-Martinez LV, 2019, COMP BIOCHEM PHYS A, V229, P93, DOI 10.1016/j.cbpa.2018.12.003
Callewaert L, 2010, J BIOSCIENCES, V35, P127, DOI 10.1007/s12038-010-0015-5
Costantini D, 2019, SCI TOTAL ENVIRON, V671, P1077, DOI 10.1016/j.scitotenv.2019.03.398
Cray C, 2009, COMPARATIVE MED, V59, P517
Dantzer R, 2006, NEUROL CLIN, V24, P441, DOI 10.1016/j.ncl.2006.03.003
Davis AK, 2008, FUNCT ECOL, V22, P760, DOI 10.1111/j.1365-2435.2008.01467.x
Demas GE, 2011, J ANIM ECOL, V80, P710, DOI 10.1111/j.1365-2656.2011.01813.x
Exton MS, 1997, APPETITE, V29, P369, DOI 10.1006/appe.1997.0116
Fishkin RJ, 1997, PSYCHOPHARMACOLOGY, V132, P335, DOI 10.1007/s002130050353
Fritze M, 2019, BIOL OPEN, V8, DOI 10.1242/bio.046078
Giffen PS, 2003, ARCH TOXICOL, V77, P392, DOI 10.1007/s00204-003-0458-7
Gilbert C, 2010, BIOL REV, V85, P545, DOI 10.1111/j.1469-185X.2009.00115.x
GILL J, 1995, COMP BIOCHEM PHYS B, V110, P235, DOI 10.1016/0305-0491(94)00127-G
Giraudeau M, 2010, PLOS ONE, V5, DOI 10.1371/journal.pone.0013555

Godbout JP, 2005, FASEB J, V19, P1329, DOI 10.1096/fj.05-3776fje
Gottdenker NL, 2014, ECOHEALTH, V11, P619, DOI 10.1007/s10393-014-0941-z
Guerrero-Chacón AL, 2018, MAMMAL RES, V63, P397, DOI 10.1007/s13364-018-0375-z
HART BL, 1988, NEUROSCI BIOBEHAV R, V12, P123, DOI 10.1016/S0149-7634(88)80004-6
Harten L, 2020, SCIENCE, V369, P194, DOI 10.1126/science.aay3354
Harten L, 2019, CURR BIOL, V29, P1895, DOI 10.1016/j.cub.2019.04.066
He CZ, 2019, J MOL EVOL, V87, P309, DOI 10.1007/s00239-019-09910-7
Heinze J, 2010, CURR BIOL, V20, P249, DOI 10.1016/j.cub.2009.12.031
Hetem RS, 2008, AM J PHYSIOL-REG I, V294, pR246, DOI 10.1152/ajpregu.00570.2007
Hopwood N, 2009, CAN J PHYSIOL PHARM, V87, P211, DOI [10.1139/Y09-003, 10.1139/y09-003]
JOHNSON RW, 1993, PHYSIOL BEHAV, V53, P343, DOI 10.1016/0031-9384(93)90215-2
Johnson RW, 2002, VET IMMUNOL IMMUNOP, V87, P443, DOI 10.1016/S0165-2427(02)00069-7
JOHNSON RW, 1994, J ANIM SCI, V72, P309, DOI 10.2527/1994.722309x
Kazlauskas N, 2016, FRONT PHYSIOL, V7, DOI 10.3389/fphys.2016.00261
Kiesecker JM, 1999, P NATL ACAD SCI USA, V96, P9165, DOI 10.1073/pnas.96.16.9165
Kolodny O, 2019, NAT ECOL EVOL, V3, P116, DOI 10.1038/s41559-018-0731-z
LOEHLE C, 1995, ECOLOGY, V76, P326, DOI 10.2307/1941192
Lopes PC, 2012, BEHAV ECOL SOCIOBIOL, V66, P1421, DOI 10.1007/s00265-012-1397-1
López P, 2016, SCI REP-UK, V6, DOI 10.1038/srep20651
Martin LB, 2008, FUNCT ECOL, V22, P68, DOI 10.1111/j.1365-2435.2007.01347.x
Melhado G, 2020, J EXP ZOOL PART A, V333, P536, DOI 10.1002/jez.2399
MOLLER AP, 1993, ADV STUD BEHAV, V22, P65, DOI 10.1016/S0065-3454(08)60405-2
Moore J, 2009, ENCY ANIMAL BEHAV, P205
Muise KA, 2018, PHYSIOL BEHAV, V194, P356, DOI 10.1016/j.physbeh.2018.06.003
MURRAY MJ, 1979, AM J CLIN NUTR, V32, P593, DOI 10.1093/ajcn/32.3.593
OREILLY B, 1988, PHYSIOL BEHAV, V42, P287, DOI 10.1016/0031-9384(88)90084-4
Otálora-Ardila A, 2016, PLOS ONE, V11, DOI 10.1371/journal.pone.0164938
Prat Y, 2016, SCI REP-UK, V6, DOI 10.1038/srep39419
Pull CD, 2018, ELIFE, V7, DOI [10.7554/eLife.32073, 10.7554/elife.32073]
Ragland SA, 2017, PLOS PATHOG, V13, DOI 10.1371/journal.ppat.1006512
Renault J, 2008, BRAIN BEHAV IMMUN, V22, P1201, DOI 10.1016/

j.bbi.2008.05.008
Ripperger SP, 2020, BEHAV ECOL, V31, P1296, DOI 10.1093/beheco/araa111
Rowe M, 2013, BIOL J LINN SOC, V109, P92, DOI 10.1111/bij.12044
Rueppell O, 2010, J EVOLUTION BIOL, V23, P1538, DOI 10.1111/j.1420-9101.2010.02022.x
Schneeberger K, 2013, J EXP BIOL, V216, P4514, DOI 10.1242/jeb.090837
Schneeberger K, 2013, PLOS ONE, V8, DOI 10.1371/journal.pone.0054023
Shakhar K, 2015, PLOS BIOL, V13, DOI 10.1371/journal.pbio.1002276
Stockmaier S, 2020, J ANIM ECOL, V89, P1387, DOI 10.1111/1365-2656.13193
Stockmaier S, 2018, ANIM BEHAV, V140, P141, DOI 10.1016/j.anbehav.2018.04.021
Stockmaier S, 2015, BIOL LETTERS, V11, DOI 10.1098/rsbl.2015.0576
Stroeymeyt N, 2018, SCIENCE, V362, P941, DOI 10.1126/science.aat4793
Ugelvig LV, 2007, CURR BIOL, V17, P1967, DOI 10.1016/j.cub.2007.10.029
Van Kerckhove K, 2013, AM J EPIDEMIOL, V178, P1655, DOI 10.1093/aje/kwt196
Voigt C.C., 2021, SCI REP-UK, V10, P1
Wang A, 2016, CELL, V166, P1512, DOI 10.1016/j.cell.2016.07.026
Weise P, 2017, PEERJ, V5, DOI 10.7717/peerj.3570
NR 70
TC 17
Z9 19
U1 1
U2 16
PU WILEY
PI HOBOKEN
PA 111 RIVER ST, HOBOKEN 07030-5774, NJ USA
SN 0077-8923
EI 1749-6632
J9 ANN NY ACAD SCI
JI Ann. N.Y. Acad. Sci.
PD DEC
PY 2021
VL 1505
IS 1
BP 178
EP 190
DI 10.1111/nyas.14600
EA APR 2021
PG 13
WC Multidisciplinary Sciences
WE Science Citation Index Expanded (SCI-EXPANDED)
SC Science & Technology - Other Topics
GA XP8MK
UT WOS:000641131500001
PM 33876431
OA Green Published
DA 2024-10-21

ER

PT J
AU Schuh, AJ
   Amman, BR
   Sealy, TK
   Spengler, JR
   Nichol, ST
   Towner, JS
AF Schuh, Amy J.
   Amman, Brian R.
   Sealy, Tara K.
   Spengler, Jessica R.
   Nichol, Stuart T.
   Towner, Jonathan S.
TI Egyptian rousette bats maintain long-term protective immunity against
   Marburg virus infection despite diminished antibody levels
SO SCIENTIFIC REPORTS
LA English
DT Article
ID BLACK FLYING-FOX; PTEROPID BATS; NIPAH VIRUS; HENDRA VIRUS; MEASLES
   EPIDEMICS; RESERVOIR HOSTS; RISK-FACTORS; FRUIT BATS; TRANSMISSION;
   DYNAMICS
AB Although bats are natural reservoir hosts for numerous zoonotic viruses, little is known about the long-term dynamics of the host immune response following infection and how these viruses are maintained in nature. The Egyptian rousette bat (ERB) is a known reservoir host for Marburg virus (MARV). Following infection of ERBs with MARV, virus-specific IgG antibodies are induced but rapidly wane and by 3 months post-infection the bats are seronegative. To determine whether reinfection of ERBs plays a role in MARV maintenance, we challenge groups of ERBs that were "naturally" or experimentally infected with MARV 17-24 months prior. No bats in either group exhibit evidence of MARV replication or shedding and all bats develop virus-specific secondary immune responses. This study demonstrates that infection of ERBs with MARV induces long-term protective immunity against reinfection and indicates that other factors, such as host population dynamics, drive MARV maintenance in nature.
C1 [Schuh, Amy J.; Amman, Brian R.; Sealy, Tara K.; Spengler, Jessica R.; Nichol, Stuart T.; Towner, Jonathan S.] Ctr Dis Control & Prevent, Viral Special Pathogens Branch, Div High Consequence Pathogens & Pathol, Atlanta, GA 30333 USA.
   [Towner, Jonathan S.] Univ Georgia, Coll Vet Med, Dept Pathol, Athens, GA 30602 USA.
C3 Centers for Disease Control & Prevention - USA; University System of
   Georgia; University of Georgia
RP Towner, JS (corresponding author), Ctr Dis Control & Prevent, Viral Special Pathogens Branch, Div High Consequence Pathogens & Pathol, Atlanta, GA 30333 USA.; Towner, JS (corresponding author),

Univ Georgia, Coll Vet Med, Dept Pathol, Athens, GA 30602 USA.
EM jit8@cdc.gov
RI Towner, Jonathan/ABC-3780-2020; Spengler, Jessica/AAN-9099-2020
FU DTRA [HDTRA-14-1-0016, S-1340-03]
FX We thank Peter Eworonsky, Lester Slough, Eddie Jackson and Sharon Dietz
from the Comparative Medicine Branch at the Centers for Disease Control
and Prevention for providing care to the bats during this study. This
study was partially funded by DTRA Grant HDTRA-14-1-0016, Subaward
S-1340-03. The findings and conclusions in this report are those of the
authors and do not necessarily represent the official position of the
Centers for Disease Control and Prevention.
CR Altizer S, 2006, ECOL LETT, V9, P467, DOI 10.1111/j.1461-0248.2005.00879.x
Amman BR, 2015, J WILDLIFE DIS, V51, P113, DOI 10.7589/2014-08-198
Amman BR, 2012, PLOS PATHOG, V8, DOI 10.1371/journal.ppat.1002877
Benda Petr, 2011, Vespertilio, V15, P3
Bjornstad ON, 2002, ECOL MONOGR, V72, P169, DOI 10.1890/0012-9615(2002)072[0169:DOMEES]2.0.CO;2
Blackwood JC, 2013, P NATL ACAD SCI USA, V110, P20837, DOI 10.1073/pnas.1308817110
Breed AC, 2011, PLOS ONE, V6, DOI 10.1371/journal.pone.0028816
Chua KB, 2002, MICROBES INFECT, V4, P145, DOI 10.1016/S1286-4579(01)01522-2
Ge XY, 2013, NATURE, V503, P535, DOI 10.1038/nature12711
Georgea DB, 2011, P NATL ACAD SCI USA, V108, P10208, DOI 10.1073/pnas.1010875108
Gloza-Rausch F, 2008, EMERG INFECT DIS, V14, P626, DOI 10.3201/eid1404.071439
Grenfell BT, 2002, ECOL MONOGR, V72, P185, DOI 10.1890/0012-9615(2002)072[0185:DOMESN]2.0.CO;2
Halpin K, 2000, J GEN VIROL, V81, P1927, DOI 10.1099/0022-1317-81-8-1927
Halpin K, 2011, AM J TROP MED HYG, V85, P946, DOI 10.4269/ajtmh.2011.10-0567
Hartman AL, 2010, CLIN LAB MED, V30, P161, DOI 10.1016/j.cll.2009.12.001
Hayman DTS, 2015, P ROY SOC B-BIOL SCI, V282, DOI 10.1098/rspb.2014.2591
Hayman DTS, 2012, J MAMMAL, V93, P1393, DOI 10.1644/11-MAMM-A-270.1
JACOBSEN NHG, 1976, S AFR J SCI, V72, P270
Kwiecinski Gary G., 1999, Mammalian Species, V611, P1
Leigh Jr E. G., 1991, DEMOGRAPHY NATURAL H, V511, P77
Lloyd-Smith JO, 2005, TRENDS ECOL EVOL, V20, P511, DOI 10.1016/j.tree.2005.07.004
Middleton DJ, 2007, J COMP PATHOL, V136, P266, DOI 10.1016/j.jcpa.2007.03.002

MUTERE F A, 1968, Acta Tropica, V25, P97
Pawan J. L., 1936, Annals of Tropical Medicine and Parasitology, V30, P101
Paweska JT, 2015, J INFECT DIS, V212, pS109, DOI 10.1093/infdis/jiv132
Paweska JT, 2012, PLOS ONE, V7, DOI 10.1371/journal.pone.0045479
Plowright RK, 2008, P ROY SOC B-BIOL SCI, V275, P861, DOI 10.1098/rspb.2007.1260
Plowright RK, 2011, P ROY SOC B-BIOL SCI, V278, P3703, DOI 10.1098/rspb.2011.0522
Rahman SA, 2013, EMERG INFECT DIS, V19, P51, DOI 10.3201/eid1901.120221
Schuh AJ, 2017, NAT COMMUN, V8, DOI 10.1038/ncomms14446
Sohayati A R, 2011, Epidemiol Infect, V139, P1570, DOI 10.1017/S0950268811000550
Swanepoel R, 2007, EMERG INFECT DIS, V13, P1847, DOI 10.3201/eid1312.071115
Towner JS, 2009, PLOS PATHOG, V5, DOI 10.1371/journal.ppat.1000536
Turmelle AS, 2010, J GEN VIROL, V91, P2360, DOI 10.1099/vir.0.020073-0
van den Hurk AF, 2009, AM J TROP MED HYG, V81, P457, DOI 10.4269/ajtmh.2009.81.457
Vardon MJ, 2000, AUST J ZOOL, V48, P91, DOI 10.1071/ZO99060
Xia YC, 2004, AM NAT, V164, P267, DOI 10.1086/422341
NR 37
TC 47
Z9 54
U1 0
U2 23
PU NATURE PORTFOLIO
PI BERLIN
PA HEIDELBERGER PLATZ 3, BERLIN, 14197, GERMANY
SN 2045-2322
J9 SCI REP-UK
JI Sci Rep
PD AUG 18
PY 2017
VL 7
AR 8763
DI 10.1038/s41598-017-07824-2
PG 7
WC Multidisciplinary Sciences
WE Science Citation Index Expanded (SCI-EXPANDED)
SC Science & Technology - Other Topics
GA FE1LP
UT WOS:000407980000017
PM 28821722
OA Green Published, gold
DA 2024-10-21
ER

PT J
AU Wang, J

Lin, ZY
Liu, QJ
Fu, FY
Wang, ZF
Ma, JJ
Wang, HA
Yan, YX
Cheng, YQ
Sun, JH
AF Wang, Jie
Lin, Zhenyu
Liu, Qiuju
Fu, Feiyu
Wang, Zhaofei
Ma, Jingjiao
Wang, Hengan
Yan, Yaxian
Cheng, Yuqiang
Sun, Jianhe
TI Bat Employs a Conserved MDA5 Gene to Trigger Antiviral Innate Immune
Responses
SO FRONTIERS IN IMMUNOLOGY
LA English
DT Article
DE BAT; MDA5; antiviral innate immunity; virus; IFNb
ID PATTERN-RECOGNITION RECEPTORS; BLACK FLYING FOX; RIG-I; MOLECULAR
CHARACTERIZATION; VIRUS; EXPRESSION; HELICASES; BINDING; SYSTEM;
LGP2
AB Bats are important hosts for various zoonotic viral diseases. However, they rarely show signs of disease infection with such viruses. As the first line for virus control, the innate immune system of bats attracted our full attention. In this study, the Tadarida brasiliensis MDA5 gene (batMDA5), a major sensor for anti-RNA viral infection, was first cloned, and its biological functions in antiviral innate immunity were identified. Bioinformatics analysis shows that the amino acid sequence of batMDA5 is poorly conserved among species, and it is evolutionarily closer to humans. The mRNA of batMDA5 was significantly upregulated in Newcastle disease virus (NDV), avian influenza virus (AIV), and vesicular stomatitis virus (VSV)-infected bat TB 1 Lu cells. Overexpression of batMDA5 could activate IFN beta and inhibit vesicular stomatitis virus (VSV-GFP) replication in TB 1 Lu cells, while knockdown of batMDA5 yielded the opposite result. In addition, we found that the CARD domain was essential for MDA5 to activate IFN beta by constructing MDA5 domain mutant plasmids. These results indicated that bat employs a conserved MDA5 gene to trigger anti-RNA virus innate immune response. This study helps understand the biological role of MDA5 in innate immunity during evolution.
C1 [Wang, Jie; Lin, Zhenyu; Liu, Qiuju; Fu, Feiyu; Wang, Zhaofei; Ma, Jingjiao; Wang, Hengan; Yan, Yaxian; Cheng, Yuqiang; Sun, Jianhe] Shanghai Jiao Tong Univ, Sch Agr & Biol, Shanghai Key Lab Vet Biotechnol, Key Lab Urban Agr South,Minist Agr, Shanghai, Peoples R China.

C3 Ministry of Agriculture & Rural Affairs; Shanghai Jiao Tong University
RP Cheng, YQ; Sun, JH (corresponding author), Shanghai Jiao Tong Univ, Sch Agr & Biol, Shanghai Key Lab Vet Biotechnol, Key Lab Urban Agr South,Minist Agr, Shanghai, Peoples R China.
EM wyycyq@sjtu.edu.cn; sunjhe@sjtu.edu.cn
RI Qi, Ling/KHE-3068-2024; , sunjhe/S-5843-2019; SUN, Ye/KBC-8159-2024
FU National Natural Science Foundation of China [32072864, 32072865];
Natural Science Foundation of Shanghai [20ZR1425100]; Science and Technology Commission of Shanghai Municipality [21N41900100]; Shanghai Agriculture Applied Technology Development Program, China [2022-02-08-00-12-F01191]
FX Funding This research was supported by the National Natural Science Foundation of China (32072864 and 32072865), the Natural Science Foundation of Shanghai (20ZR1425100), Science and Technology Commission of Shanghai Municipality (21N41900100), Shanghai Agriculture Applied Technology Development Program, China (2022-02-08-00-12-F01191).
CR Allen LC, 2009, J COMP PHYSIOL B, V179, P315, DOI 10.1007/s00360-008-0315-3
Anthony SJ, 2017, MBIO, V8, DOI [10.1128/mBio.00373-17, 10.1128/mbio.00373-17]
Barrat FJ, 2016, ANNU REV MED, V67, P323, DOI 10.1146/annurev-med-052814-023338
Berke IC, 2012, EMBO J, V31, P1714, DOI 10.1038/emboj.2012.19
Boyd A, 2007, BIOCHEM SOC T, V35, P1504, DOI 10.1042/BST0351504
Brisse M, 2019, FRONT IMMUNOL, V10, DOI 10.3389/fimmu.2019.01586
Broquet AH, 2011, J IMMUNOL, V186, P1618, DOI 10.4049/jimmunol.1002862
Campbell LK, 2020, FRONT CELL INFECT MI, V10, DOI 10.3389/fcimb.2020.00209
Cheng YQ, 2019, FRONT IMMUNOL, V10, DOI 10.3389/fimmu.2019.02224
Cheng YQ, 2019, J IMMUNOL, V203, P1930, DOI 10.4049/jimmunol.1900293
Cibulski SP, 2021, ARCH VIROL, V166, P207, DOI 10.1007/s00705-020-04825-x
Clayton E, 2020, FRONT CELL INFECT MI, V10, DOI 10.3389/fcimb.2020.527921
Cowled C, 2012, DEV COMP IMMUNOL, V36, P657, DOI 10.1016/j.dci.2011.11.008
Cowled C, 2011, DEV COMP IMMUNOL, V35, P7, DOI 10.1016/j.dci.2010.07.006
De La Cruz-Rivera PC, 2018, J IMMUNOL, V200, P209, DOI 10.4049/jimmunol.1701214
Dias AG, 2019, TRENDS MICROBIOL, V27, P75, DOI 10.1016/j.tim.2018.08.007
Fajgenbaum DC, 2020, NEW ENGL J MED, V383, P2255, DOI 10.1056/NEJMra2026131
Forbes KM, 2019, EMERG INFECT DIS, V25, P955, DOI 10.3201/

eid2505.181666
   Ge XY, 2013, NATURE, V503, P535, DOI 10.1038/nature12711
   Giotis ES, 2021, FRONT CELL INFECT MI, V11, DOI 10.3389/fcimb.2021.651289
   Huang YH, 2016, FISH SHELLFISH IMMUN, V54, P188, DOI 10.1016/j.fsi.2016.04.001
   Irving AT, 2020, CELL REP, V33, DOI 10.1016/j.celrep.2020.108345
   Iwasaki A, 2014, NAT REV IMMUNOL, V14, P315, DOI 10.1038/nri3665
   Jacobs JL, 2013, J MOL BIOL, V425, P5009, DOI 10.1016/j.jmb.2013.10.007
   Kawai T, 2010, NAT IMMUNOL, V11, P373, DOI 10.1038/ni.1863
   Lee SB, 2020, FRONT IMMUNOL, V11, DOI 10.3389/fimmu.2020.00678
   Letunic I, 2021, NUCLEIC ACIDS RES, V49, pD458, DOI 10.1093/nar/gkaa937
   Lin HH, 2022, MICROBIOL IMMUNOL, V66, P97, DOI 10.1111/1348-0421.12952
   Lin ZY, 2021, FRONT IMMUNOL, V12, DOI 10.3389/fimmu.2021.742074
   Loo YM, 2011, IMMUNITY, V34, P680, DOI 10.1016/j.immuni.2011.05.003
   Lu HL, 2013, J IMMUNOL, V191, P3264, DOI 10.4049/jimmunol.1300512
   Pierce JD, 2022, NURS RES, V71, P164, DOI 10.1097/NNR.0000000000000565
   Swanepoel R, 2007, EMERG INFECT DIS, V13, P1847, DOI 10.3201/eid1312.071115
   Tarigan R, 2021, VIRUS GENES, V57, P510, DOI 10.1007/s11262-021-01865-6
   Tarigan R, 2020, BIOCHEM BIOPH RES CO, V527, P1, DOI 10.1016/j.bbrc.2020.04.060
   Uchikawa E, 2016, MOL CELL, V62, P586, DOI 10.1016/j.molcel.2016.04.021
   Wang LF, 2019, CURR OPIN VIROL, V34, P79, DOI 10.1016/j.coviro.2018.12.007
   Waterhouse A, 2018, NUCLEIC ACIDS RES, V46, pW296, DOI 10.1093/nar/gky427
   Woo PCY, 2009, EXP BIOL MED, V234, P1117, DOI 10.3181/0903-MR-94
   Wu B, 2015, CURR OPIN VIROL, V12, P91, DOI 10.1016/j.coviro.2015.04.004
   Yoneyama M, 2005, J IMMUNOL, V175, P2851, DOI 10.4049/jimmunol.175.5.2851
   Zhang GJ, 2013, SCIENCE, V339, P456, DOI 10.1126/science.1230835
   Zhou P., 2020, NATURE, V579, P270, DOI DOI 10.1038/s41586-020-2012-7
   Zhou P, 2016, P NATL ACAD SCI USA, V113, P2696, DOI 10.1073/pnas.1518240113
   Zhou P, 2014, PLOS ONE, V9, DOI 10.1371/journal.pone.0103875
   Zou J, 2009, BMC EVOL BIOL, V9, DOI 10.1186/1471-2148-9-85
NR 46
TC 7
Z9 7
U1 2
U2 14
PU FRONTIERS MEDIA SA
PI LAUSANNE
PA AVENUE DU TRIBUNAL FEDERAL 34, LAUSANNE, CH-1015, SWITZERLAND

SN 1664-3224
J9 FRONT IMMUNOL
JI Front. Immunol.
PD MAY 23
PY 2022
VL 13
AR 904481
DI 10.3389/fimmu.2022.904481
PG 11
WC Immunology
WE Science Citation Index Expanded (SCI-EXPANDED)
SC Immunology
GA 1X0ON
UT WOS:000807163800001
PM 35677039
OA Green Published, gold
DA 2024-10-21
ER

PT J
AU Storm, N
   van Vuren, PJ
   Markotter, W
   Paweska, JT
AF Storm, Nadia
   van Vuren, Petrus Jansen
   Markotter, Wanda
   Paweska, Janusz T.
TI Antibody Responses to Marburg Virus in Egyptian Rousette Bats and Their
   Role in Protection against Infection
SO VIRUSES-BASEL
LA English
DT Article
DE Marburg virus; Egyptian rousette bat; antibody response; maternal
   immunity; immune duration; reinfection; viral shedding; South Africa
ID HEMORRHAGIC-FEVER; RESERVOIR HOSTS; PTEROPID BATS; FRUIT BATS;
   TRANSMISSION; DISEASE; UGANDA
AB Egyptian rousette bats (ERBs) are reservoir hosts for the Marburg
virus (MARV). The immune dynamics and responses to MARV infection in
ERBs are poorly understood, and limited information exists on the
role of antibodies in protection of ERBs against MARV infection.
Here, we determine the duration of maternal immunity to MARV in
juvenile ERBs, and evaluate the duration of the antibody response to
MARV in bats naturally or experimentally infected with the virus. We
further explore whether antibodies in previously naturally exposed
bats is fully protective against experimental reinfection with MARV.
Maternal immunity was lost in juvenile ERBs by 5 months of age.
Antibodies to MARV remained detectable in 67% of experimentally
infected bats approximately 4 months post inoculation (p.i.), while
antibodies to MARV remained present in 84% of naturally exposed bats
at least 11 months after capture. Reinfection of seropositive ERBs
with MARV produced an anamnestic response from day 5 p.i. Although

PCR-defined viremia was present in 73.3% of reinfected ERBs, replicating virus was recovered from the serum of only one bat on day 3 p.i. The negative PCR results in the salivary glands, intestines, bladders and reproductive tracts of reinfected bats, and the apparent absence of MARV in the majority of swabs collected from these bats suggest that reinfection may only play a minor role in the transmission and maintenance of MARV amongst ERBs in nature.
C1 [Storm, Nadia; van Vuren, Petrus Jansen; Paweska, Janusz T.] Natl Inst Communicable Dis, Natl Hlth Lab Serv, Ctr Emerging Zoonot & Parasit Dis, ZA-2131 Johannesburg, South Africa.
[Storm, Nadia] Univ Pretoria, Fac Nat & Agr Sci, Dept Microbiol & Plant Pathol, ZA-0002 Pretoria, South Africa.
[van Vuren, Petrus Jansen; Markotter, Wanda; Paweska, Janusz T.] Univ Pretoria, Ctr Viral Zoonoses, Dept Med Virol, Sch Med,Fac Hlth Sci, ZA-0001 Pretoria, South Africa.
[Paweska, Janusz T.] Univ Witwatersrand, Sch Pathol, Div Virol & Communicable Dis Surveillance, Johannesburg, South Africa.
C3 National Health Laboratory Service; National Institute for Communicable
Diseases (NICD); University of Pretoria; University of Pretoria; University of Witwatersrand; National Institute for Communicable Diseases (NICD)
RP Paweska, JT (corresponding author), Natl Inst Communicable Dis, Natl Hlth Lab Serv, Ctr Emerging Zoonot & Parasit Dis, ZA-2131 Johannesburg, South Africa.; Paweska, JT (corresponding author), Univ Pretoria, Ctr Viral Zoonoses, Dept Med Virol, Sch Med,Fac Hlth Sci, ZA-0001 Pretoria, South Africa.; Paweska, JT (corresponding author), Univ Witwatersrand, Sch Pathol, Div Virol & Communicable Dis Surveillance, Johannesburg, South Africa.
EM nadias@nicd.ac.za; petrusv@nicd.ac.za; wanda.markotter@up.ac.za; januszp@nicd.ac.za
RI Storm, Nadia/HJY-8259-2023; Jansen van Vuren, Petrus/
AAW-6963-2020;
Markotter, Wanda/A-2129-2010
OI Storm, Nadia/0000-0002-2596-0468; Markotter, Wanda/
0000-0002-7550-0080;
Jansen van Vuren, Petrus/0000-0003-2862-7983; Paweska, Janusz/0000-0001-8776-7519
FU National Institute for Communicable Diseases; National Research Foundation of South Africa [86228]; Poliomyelitis Research
Foundation
[13/54]; University of Pretoria
FX We thank Gunther Meier and Zibusiso Masuku of the CEZPD-NICD for their
assistance with the biosafety aspects of this project. We are also
grateful to Alan Kemp for commenting on and proofreading this work, and
to Dr Naazneen Moolla for assisting with the graphical abstract. This
study was supported by the National Institute for Communicable Diseases,
the National Research Foundation of South Africa (86228), the Poliomyelitis Research Foundation (13/54), and the University of

Pretoria (postgraduate bursary).
CR Adjemian J, 2011, J INFECT DIS, V204, pS796, DOI 10.1093/infdis/jir312
Amman BR, 2015, J WILDLIFE DIS, V51, P113, DOI 10.7589/2014-08-198
Amman BR, 2014, EMERG INFECT DIS, V20, P1761, DOI 10.3201/eid2010.140696
Amman BR, 2012, PLOS PATHOG, V8, DOI 10.1371/journal.ppat.1002877
Bausch DG, 2006, NEW ENGL J MED, V355, P909, DOI 10.1056/NEJMoa051465
Brunet-Rossinni Anja K., 2009, P315
CONRAD JL, 1978, AM J TROP MED HYG, V27, P1210, DOI 10.4269/ajtmh.1978.27.1210
Fujita N., 2009, Morbidity and Mortality Weekly Report, V58, P1377
Halpin K, 2011, AM J TROP MED HYG, V85, P946, DOI 10.4269/ajtmh.2011.10-0567
Hayman DTS, 2015, P ROY SOC B-BIOL SCI, V282, DOI 10.1098/rspb.2014.2591
Hierholzer J. C., 1996, P25, DOI 10.1016/B978-012465330-6/50003-8
Jansen van Vuren P., 2016, J CLIN MICROBIOL, V52, P35967, DOI [10.1128/JCM.02724-15, DOI 10.1128/JCM.02724-1526637383]
Johnson ED, 1996, ARCH VIROL, P101
Middleton DJ, 2007, J COMP PATHOL, V136, P266, DOI 10.1016/j.jcpa.2007.03.002
MUTERE F A, 1968, Acta Tropica, V25, P97
Nakayama E, 2011, J INFECT DIS, V204, pS978, DOI 10.1093/infdis/jir334
Paweska J.T., 2014, P 6 INT S FIL GALV T
Paweska JT, 2015, J INFECT DIS, V212, pS109, DOI 10.1093/infdis/jiv132
Paweska JT, 2012, PLOS ONE, V7, DOI 10.1371/journal.pone.0045479
Paweska JT, 2005, J VIROL METHODS, V124, P173, DOI 10.1016/j.jviromet.2004.11.020
Peel AJ, 2014, P ROY SOC B-BIOL SCI, V281, DOI 10.1098/rspb.2013.2962
Plowright RK, 2016, PLOS NEGLECT TROP D, V10, DOI 10.1371/journal.pntd.0004796
Sanchez A., 2007, FIELDS VIROLOGY, P1409
Schuh AJ, 2017, SCI REP-UK, V7, DOI 10.1038/s41598-017-07824-2
Schuh AJ, 2017, NAT COMMUN, V8, DOI 10.1038/ncomms14446
SMITH DH, 1982, LANCET, V1, P816
Swanepoel R, 2007, EMERG INFECT DIS, V13, P1847, DOI 10.3201/eid1312.071115
Timen A, 2009, EMERG INFECT DIS, V15, P1171, DOI [10.3201/eid1508.090051, 10.3201/eid1508.090015]
Towner JS, 2007, PLOS ONE, V2, DOI 10.1371/journal.pone.0000764
Towner JS, 2009, PLOS PATHOG, V5, DOI 10.1371/journal.ppat.1000536
NR 30
TC 24
Z9 25
U1 0
U2 13

PU MDPI
PI BASEL
PA ST ALBAN-ANLAGE 66, CH-4052 BASEL, SWITZERLAND
SN 1999-4915
J9 VIRUSES-BASEL
JI Viruses-Basel
PD FEB
PY 2018
VL 10
IS 2
AR 73
DI 10.3390/v10020073
PG 12
WC Virology
WE Science Citation Index Expanded (SCI-EXPANDED)
SC Virology
GA FZ4EN
UT WOS:000427544100023
PM 29439384
OA Green Published, Green Submitted, gold
DA 2024-10-21
ER

PT J
AU Zhou, P
Cowled, C
Todd, S
Crameri, G
Virtue, ER
Marsh, GA
Klein, R
Shi, ZL
Wang, LF
Baker, ML
AF Zhou, Peng
Cowled, Chris
Todd, Shawn
Crameri, Gary
Virtue, Elena R.
Marsh, Glenn A.
Klein, Reuben
Shi, Zhengli
Wang, Lin-Fa
Baker, Michelle L.
TI Type III IFNs in Pteropid Bats: Differential Expression Patterns Provide
Evidence for Distinct Roles in Antiviral Immunity
SO JOURNAL OF IMMUNOLOGY
LA English
DT Article
ID BLACK FLYING FOX; FRUIT BATS; INTERFERON GENES; HENDRA VIRUS; NIPAH
VIRUS; RECEPTOR; LAMBDA; INDUCTION; INFECTION; MORBILLIVIRUS
AB Bats are known to harbor a number of emerging and re-emerging

zoonotic viruses, many of which are highly pathogenic in other mammals but result in no clinical symptoms in bats. The ability of bats to coexist with viruses may be the result of rapid control of viral replication early in the immune response. IFNs provide the first line of defense against viral infection in vertebrates. Type III IFNs (IFN-lambda s) are a recently identified IFN family that share similar antiviral activities with type I IFNs. To our knowledge, we demonstrate the first functional analysis of type III IFNs from any species of bat, with the investigation of two IFN-lambda genes from the pteropid bat, Pteropus alecto. Our results demonstrate that bat type III IFN has similar antiviral activity to type I and III IFNs from other mammals. In addition, the two bat type III IFNs are differentially induced relative to each other and to type I IFNs after treatment or transfection with synthetic dsRNA. Infection with the bat paramyxovirus, Tioman virus, resulted in no upregulation of type I IFN production in bat splenocytes but was capable of inducing a type III IFN response in three of the four bats tested. To our knowledge, this is the first report to describe the simultaneous suppression of type I IFN and induction of type III IFN after virus infection. These results may have important implications for the role of type III IFNs in the ability of bats to coexist with viruses. The Journal of Immunology, 2011, 186: 3138-3147.
C1 [Zhou, Peng; Cowled, Chris; Todd, Shawn; Crameri, Gary; Virtue, Elena R.; Marsh, Glenn A.; Klein, Reuben; Wang, Lin-Fa; Baker, Michelle L.] Commonwealth Sci & Ind Res Org Livestock Ind, Australian Anim Hlth Lab, Geelong, Vic 3220, Australia.
[Zhou, Peng; Shi, Zhengli] Chinese Acad Sci, State Key Lab Virol, Wuhan Inst Virol, Wuhan 430071, Peoples R China.
[Virtue, Elena R.; Wang, Lin-Fa] Univ Melbourne, Dept Microbiol & Immunol, Parkville, Vic 3010, Australia.
[Baker, Michelle L.] Univ New Mexico, Dept Biol, Ctr Evolutionary & Theoret Immunol, Albuquerque, NM 87131 USA.
C3 Commonwealth Scientific & Industrial Research Organisation (CSIRO);
Chinese Academy of Sciences; Wuhan Institute of Virology, CAS;
University of Melbourne; University of New Mexico
RP Baker, ML (corresponding author), Commonwealth Sci & Ind Res Org Livestock Ind, Australian Anim Hlth Lab, PO Bag 24, Geelong, Vic 3220, Australia.
EM michelle.baker@csiro.au
RI Todd, Shawn/JJE-5658-2023; lu, yuan/JZD-0832-2024; Zhou, Peng/I-8578-2015; Crameri, Gary/H-8441-2013; Baker, Michelle/C-9694-2013; Cowled, Christopher/H-5308-2013; Marsh, Glenn Andrew/A-5418-2013; Shi, Zheng-Li/A-1013-2013
OI Todd, Shawn/0009-0009-2218-7842; Baker, Michelle/0000-0002-7993-9971;
Cowled, Christopher/0000-0002-1969-9371; Virtue, Elena/0000-0002-2499-1043; Marsh, Glenn Andrew/0000-0002-3469-1837;
Wang, Lin-Fa/0000-0003-2752-0535; Shi, Zheng-Li/0000-0001-8089-163X
FU Commonwealth Scientific and Industrial Research Organization;

National
Center for Research Resources [P20RR018754]; Chinese Academy of Sciences
FX This work was supported by a Commonwealth Scientific and Industrial
Research Organization Chief Executive Officer Science Leaders award (to
L.-F.W.), National Institutes of Health Grant P20RR018754 from the
Institutional Development Award Programme of the National Center for
Research Resources (to M.L.B.), and a scholarship from the Chinese
Academy of Sciences (to P.Z.).
CR ALTSCHUL SF, 1990, J MOL BIOL, V215, P403, DOI 10.1016/S0022-2836(05)80360-2
Ank N, 2008, J IMMUNOL, V180, P2474, DOI 10.4049/jimmunol.180.4.2474
Ank N, 2006, J INTERF CYTOK RES, V26, P373, DOI 10.1089/jir.2006.26.373
Ank N, 2009, BIOFACTORS, V35, P82, DOI 10.1002/biof.19
Baker ML, 2010, IMMUNOGENETICS, V62, P173, DOI 10.1007/s00251-010-0425-4
Bartlett NW, 2005, J GEN VIROL, V86, P1589, DOI 10.1099/vir.0.80904-0
Calisher CH, 2006, CLIN MICROBIOL REV, V19, P531, DOI 10.1128/CMR.00017-06
Chua KB, 2007, P NATL ACAD SCI USA, V104, P11424, DOI 10.1073/pnas.0701372104
Chua KB, 2000, SCIENCE, V288, P1432, DOI 10.1126/science.288.5470.1432
Chua KB, 2001, VIROLOGY, V283, P215, DOI 10.1006/viro.2000.0882
Coccia EM, 2004, EUR J IMMUNOL, V34, P796, DOI 10.1002/eji.200324610
Cowled C, 2011, DEV COMP IMMUNOL, V35, P7, DOI 10.1016/j.dci.2010.07.006
Crameri G, 2009, PLOS ONE, V4, DOI 10.1371/journal.pone.0008266
Fox BA, 2009, PLOS ONE, V4, DOI 10.1371/journal.pone.0004933
Gad HH, 2009, J BIOL CHEM, V284, P20869, DOI 10.1074/jbc.M109.002923
Halpin K, 2000, J GEN VIROL, V81, P1927, DOI 10.1099/0022-1317-81-8-1927
Iversen MB, 2010, J VIROL, V84, P4579, DOI 10.1128/JVI.02591-09
Karpala AJ, 2008, J INTERF CYTOK RES, V28, P341, DOI 10.1089/jir.2007.0117
Kepler TB, 2010, BMC GENOMICS, V11, DOI 10.1186/1471-2164-11-444
Kotenko SV, 2003, NAT IMMUNOL, V4, P69, DOI 10.1038/ni875
Lasfar A, 2006, CANCER RES, V66, P4468, DOI 10.1158/0008-5472.CAN-05-3653
Lau SKP, 2005, P NATL ACAD SCI USA, V102, P14040, DOI 10.1073/pnas.0506735102
Leroy EM, 2005, NATURE, V438, P575, DOI 10.1038/438575a
Levraud JP, 2007, J IMMUNOL, V178, P4385, DOI 10.4049/jimmunol.178.7.4385

Li WD, 2005, SCIENCE, V310, P676, DOI 10.1126/science.1118391
Middleton DJ, 2007, J COMP PATHOL, V136, P266, DOI 10.1016/j.jcpa.2007.03.002
MURRAY K, 1995, SCIENCE, V268, P94, DOI 10.1126/science.7701348
Omatsu T, 2008, VET IMMUNOL IMMUNOP, V124, P169, DOI 10.1016/j.vetimm.2008.03.004
Onoguchi K, 2007, J BIOL CHEM, V282, P7576, DOI 10.1074/jbc.M608618200
Pestka S, 2004, IMMUNOL REV, V202, P8, DOI 10.1111/j.0105-2896.2004.00204.x
Prescott J, 2010, PLOS ONE, V5, DOI 10.1371/journal.pone.0011159
Pritchard LI, 2006, ARCH VIROL, V151, P229, DOI 10.1007/s00705-005-0644-4
Qi ZT, 2010, J IMMUNOL, V184, P5038, DOI 10.4049/jimmunol.0903374
Randall RE, 2008, J GEN VIROL, V89, P1, DOI 10.1099/vir.0.83391-0
SAITOU N, 1987, MOL BIOL EVOL, V4, P406, DOI 10.1093/oxfordjournals.molbev.a040454
Schroder K, 2004, J LEUKOCYTE BIOL, V75, P163, DOI 10.1189/jlb.0603252
Sheppard P, 2003, NAT IMMUNOL, V4, P63, DOI 10.1038/ni873
Smith JDL, 2009, BIOL LETTERS, V5, P347, DOI 10.1098/rsbl.2009.0016
Sommereyns C, 2008, PLOS PATHOG, V4, DOI 10.1371/journal.ppat.1000017
Tamura K, 2007, MOL BIOL EVOL, V24, P1596, DOI 10.1093/molbev/msm092
Terenzi F, 2006, J BIOL CHEM, V281, P34064, DOI 10.1074/jbc.M605771200
THOMPSON JD, 1994, NUCLEIC ACIDS RES, V22, P4673, DOI 10.1093/nar/22.22.4673
Towner JS, 2009, PLOS PATHOG, V5, DOI 10.1371/journal.ppat.1000536
Uzé G, 2007, BIOCHIMIE, V89, P729, DOI 10.1016/j.biochi.2007.01.008
van den Hurk AF, 2009, AM J TROP MED HYG, V81, P457, DOI 10.4269/ajtmh.2009.81.457
van der Poel WHM, 2006, VECTOR-BORNE ZOONOT, V6, P315, DOI 10.1089/vbz.2006.6.315
Williamson MM, 1998, AUST VET J, V76, P813, DOI 10.1111/j.1751-0813.1998.tb12335.x
Witte K, 2010, CYTOKINE GROWTH F R, V21, P237, DOI 10.1016/j.cytogfr.2010.04.002
Yoneyama M, 2004, NAT IMMUNOL, V5, P730, DOI 10.1038/ni1087
Zhou ZL, 2007, J VIROL, V81, P7749, DOI 10.1128/JVI.02438-06
NR 50
TC 77
Z9 88
U1 0
U2 28
PU AMER ASSOC IMMUNOLOGISTS
PI BETHESDA
PA 9650 ROCKVILLE PIKE, BETHESDA, MD 20814 USA
SN 0022-1767
EI 1550-6606

J9 J IMMUNOL
JI J. Immunol.
PD MAR 1
PY 2011
VL 186
IS 5
BP 3138
EP 3147
DI 10.4049/jimmunol.1003115
PG 10
WC Immunology
WE Science Citation Index Expanded (SCI-EXPANDED)
SC Immunology
GA 721LT
UT WOS:000287356900052
PM 21278349
OA Green Accepted, Bronze
DA 2024-10-21
ER

PT J
AU Liu, S
   Xiao, YH
   Wang, XF
   Guo, DG
   Wang, YM
   Wang, Y
AF Liu, Sen
   Xiao, Yanhong
   Wang, Xufan
   Guo, Dongge
   Wang, Yanmei
   Wang, Ying
TI Effects of Microhabitat Temperature Variations on the Gut Microbiotas of
   Free-Living Hibernating Animals
SO MICROBIOLOGY SPECTRUM
LA English
DT Article
DE temperature; gut microbiota; 16S rRNA gene sequencing; hibernation;
   Rhinolophus pusillus
ID IMMUNE
AB Temperature variations affect gut microbiome diversity and structure in both ectothermic and endothermic animals. Here, we aimed to characterize temperature-induced changes in the gut microbiotas of adjacent natural populations of the least horseshoe bat (Rhinolophus pusillus) which hibernate at different ambient temperatures.
   Variations in ambient temperature (T-a) may significantly influence the gut microbiotas of ectothermic and endothermic animals, affecting fitness. It remains unclear, however, whether temperature fluctuations affect the gut microbial communities of hibernating animals during torpor. To investigate temperature-

induced changes in the gut microbiota during hibernation under entirely natural conditions, we took advantage of two adjacent but distinct populations of the least horseshoe bat (Rhinolophus pusillus), which inhabit sites with a similar summer T-a but a different winter T-a. Using 16S rRNA gene high-throughput sequencing, we estimated differences in gut microbial diversity and composition between the hibernating (winter) and active (summer) R. pusillus populations at both sites. During the active period, gut microbiotas did not differ significantly between the two populations, probably due to the similar T(a)s. However, during hibernation, a higher T-a was associated with decreased & alpha;-diversity in the gut microbiome. During hibernation, temperature variation did not significantly affect the relative abundance of Proteobacteria, the dominant phylum at both sites, but marked site-specific differences were detected in the relative abundances of Firmicutes, Actinobacteria, and Tenericutes. In total, 74 amplicon sequence variants (ASVs) were significantly differentially abundant between the hibernating and active bat guts across the two sites; most of these ASVs were associated with the cooler site, and many belonged to pathogenic genera, suggesting that lower ambient temperatures during hibernation may increase the risk of pathogen proliferation in the host gut. Our findings help to clarify the mechanisms underlying the gut microbiota-driven adaptation of hibernating mammals to temperature changes.IMPORTANCE Temperature variations affect gut microbiome diversity and structure in both ectothermic and endothermic animals. Here, we aimed to characterize temperature-induced changes in the gut microbiotas of adjacent natural populations of the least horseshoe bat (Rhinolophus pusillus) which hibernate at different ambient temperatures. We found that the ambient temperature significantly affected the & alpha;-diversity, but not the & beta;-diversity, of the gut microbiota. Bats hibernating at cooler temperatures experienced more drastic shifts in gut microbiome structure, with consequent effects on energy-related metabolic pathways. Our results provide novel insights into the effects of ambient temperature on the gut microbiotas of hibernating animals.
C1 [Liu, Sen; Wang, Xufan; Guo, Dongge; Wang, Yanmei; Wang, Ying] Henan Normal Univ, Coll Life Sci, Xinxiang, Peoples R China.
[Xiao, Yanhong] Northeast Normal Univ, Jilin Prov Key Lab Anim Resource Conservat & Utili, Changchun, Peoples R China.
C3 Henan Normal University; Northeast Normal University - China
RP Liu, S; Wang, Y (corresponding author), Henan Normal Univ, Coll Life Sci, Xinxiang, Peoples R China.
EM liusen2021@htu.edu.cn; 2021044@htu.edu.cn
RI zhang, meng/JMB-0951-2023; Chen, Chao/JHS-6563-2023
OI Liu, Sen/0000-0003-2258-3676
FU National Natural Science Foundation of China [31870354, 32171481];
Research Fund for Doctoral Talents of Henan Normal University [QD2021004]
FX ACKNOWLEDGMENTS This work was supported by the National Natural Science
Foundation of China (grant numbers 31870354 and 32171481) and the Research Fund for Doctoral Talents of Henan Normal University

(grant
number QD2021004). We thank Longru Jin from Northeast Normal University,
China, for donating the aluminum rings. We thank LetPub for linguistic
assistance and presubmission expert review. We declare that we have no
conflicts of interest.
CR Alberdi A, 2021, SCI REP-UK, V11, DOI 10.1038/s41598-021-02015-6
Alberdi A, 2016, TRENDS ECOL EVOL, V31, P689, DOI 10.1016/j.tree.2016.06.008
Arpigny JL, 1999, BIOCHEM J, V343, P177, DOI 10.1042/0264-6021:3430177
Bestion E, 2017, NAT ECOL EVOL, V1, DOI 10.1038/s41559-017-0161
Bolyen E, 2019, NAT BIOTECHNOL, V37, P852, DOI 10.1038/s41587-019-0209-9
Bosmans L, 2018, INSECTS, V9, DOI 10.3390/insects9040188
Cannon B, 2004, PHYSIOL REV, V84, P277, DOI 10.1152/physrev.00015.2003
Cao Y, 2022, MICROB BIOTECHNOL, V15, P276, DOI 10.1111/1751-7915.13772
Carey HV, 2017, ANNU REV NUTR, V37, P477, DOI 10.1146/annurev-nutr-071816-064740
Carey HV, 2013, AM J PHYSIOL-REG I, V304, pR33, DOI 10.1152/ajpregu.00387.2012
Carey Hannah V., 2012, P281
Carrillo-Araujo M, 2015, FRONT MICROBIOL, V6, DOI 10.3389/fmicb.2015.00447
Chevalier C, 2015, CELL, V163, P1360, DOI 10.1016/j.cell.2015.11.004
Chiang E, 2022, ANIM MICROBIOME, V4, DOI 10.1186/s42523-021-00154-9
Dill-McFarland KA, 2014, MOL ECOL, V23, P4658, DOI 10.1111/mec.12884
Douglas GM, 2018, METHODS MOL BIOL, V1849, P169, DOI 10.1007/978-1-4939-8728-3_11
Fontaine SS, 2018, J EXP BIOL, V221, DOI 10.1242/jeb.187559
Fontaine SS., 2021, BIORXIV, DOI [10.1101/2021.06.04.447101, DOI 10.1101/2021.06.04.447101]
Fung TC, 2017, NAT NEUROSCI, V20, P145, DOI 10.1038/nn.4476
Geiser F., 2021, Ecological Physiology of Daily Torpor and Hibernation, DOI DOI 10.1007/978-3-030-75525-6
Gould AL, 2018, P NATL ACAD SCI USA, V115, pE11951, DOI 10.1073/pnas.1809349115
Heijtza RD, 2011, P NATL ACAD SCI USA, V108, P3047, DOI 10.1073/pnas.1010529108
Hooper LV, 2002, ANNU REV NUTR, V22, P283, DOI 10.1146/annurev.nutr.22.011602.092259
Kanehisa M, 2004, NUCLEIC ACIDS RES, V32, pD277, DOI 10.1093/nar/gkh063
Kappelle M., 2020, 1248 WORLD MET ORG
Kemp MU, 2012, METHODS ECOL EVOL, V3, P65, DOI 10.1111/j.2041-210X.2011.00138.x
Kohl KD, 2016, ENVIRON MICROBIOL, V18, P1561, DOI

10.1111/1462-2920.13255
KUNZ TH, 1982, J MAMMAL, V63, P23, DOI 10.2307/1380667
Kurtz CC, 2007, DEV COMP IMMUNOL, V31, P415, DOI 10.1016/j.dci.2006.07.003
Levin E, 2015, P ROY SOC B-BIOL SCI, V282, DOI 10.1098/rspb.2014.2781
Lutz HL, 2019, MSYSTEMS, V4, DOI 10.1128/mSystems.00511-19
Moreno-Navarrete JM, 2019, REV ENDOCR METAB DIS, V20, P387, DOI 10.1007/s11154-019-09523-x
Quast Christian, 2013, Nucleic Acids Res, V41, pD590, DOI 10.1093/nar/gks1219
Regan MD., 2021, BIORXIV, DOI [10.1101/2021.02.24.432731, DOI 10.1101/2021.02.24.432731]
Shin NR, 2015, TRENDS BIOTECHNOL, V33, P496, DOI 10.1016/j.tibtech.2015.06.011
Song SJ, 2020, MBIO, V11, DOI 10.1128/mBio.02901-19
Team, 2022, R LANG ENV STAT COMP
Wiebler JM, 2018, P ROY SOC B-BIOL SCI, V285, DOI 10.1098/rspb.2018.0241
Xiao GH, 2019, FRONT MICROBIOL, V10, DOI 10.3389/fmicb.2019.02247
Yang YT, 2021, ANIM BIOSCI, V34, P93, DOI 10.5713/ajas.20.0314
Zhu LF, 2021, FRONT MICROBIOL, V12, DOI 10.3389/fmicb.2021.543767
Zhu LH, 2019, APPL MICROBIOL BIOT, V103, P461, DOI 10.1007/s00253-018-9465-8
NR 42
TC 3
Z9 3
U1 8
U2 27
PU AMER SOC MICROBIOLOGY
PI WASHINGTON
PA 1752 N ST NW, WASHINGTON, DC 20036-2904 USA
SN 2165-0497
J9 MICROBIOL SPECTR
JI Microbiol. Spectr.
PD AUG 17
PY 2023
VL 11
IS 4
DI 10.1128/spectrum.00433-23
EA JUN 2023
PG 10
WC Microbiology
WE Science Citation Index Expanded (SCI-EXPANDED)
SC Microbiology
GA W8TH0
UT WOS:001015914500001
PM 37378560
OA Green Published, gold
DA 2024-10-21
ER

PT J
AU Plowright, RK

Foley, P
Field, HE
Dobson, AP
Foley, JE
Eby, P
Daszak, P
AF Plowright, Raina K.
Foley, Patrick
Field, Hume E.
Dobson, Andy P.
Foley, Janet E.
Eby, Peggy
Daszak, Peter
TI Urban habituation, ecological connectivity and epidemic dampening: the emergence of Hendra virus from flying foxes (<i>Pteropus</i> spp.)
SO PROCEEDINGS OF THE ROYAL SOCIETY B-BIOLOGICAL SCIENCES
LA English
DT Article
DE Hendra virus; Pteropus; flying fox; bat virus; connectivity; metapopulation disease model
ID COLONY SITE; NORTHERN-TERRITORY; TRAVELING-WAVES; RISK-FACTORS; DYNAMICS; POLIOCEPHALUS; BATS; MOVEMENTS; ALECTO; PTEROPODIDAE
AB Anthropogenic environmental change is often implicated in the emergence of new zoonoses from wildlife; however, there is little mechanistic understanding of these causal links. Here, we examine the transmission dynamics of an emerging zoonotic paramyxovirus, Hendra virus (HeV), in its endemic host, Australian Pteropus bats (fruit bats or flying foxes). HeV is a biosecurity level 4 (BSL-4) pathogen, with a high case-fatality rate in humans and horses. With models parametrized from field and laboratory data, we explore a set of probable contributory mechanisms that explain the spatial and temporal pattern of HeV emergence; including urban habituation and decreased migration-two widely observed changes in flying fox ecology that result from anthropogenic transformation of bat habitat in Australia. Urban habituation increases the number of flying foxes in contact with human and domestic animal populations, and our models suggest that, in addition, decreased bat migratory behaviour could lead to a decline in population immunity, giving rise to more intense outbreaks after local viral reintroduction. Ten of the 14 known HeV outbreaks occurred near urbanized or sedentary flying fox populations, supporting these predictions. We also demonstrate that by incorporating waning maternal immunity into our models, the peak modelled prevalence coincides with the peak annual spill-over hazard for HeV. These results provide the first detailed mechanistic framework for understanding the sporadic temporal pattern of HeV emergence, and of the urban/peri-urban distribution of HeV outbreaks in horses and people.
C1 [Plowright, Raina K.] Penn State Univ, Ctr Infect Dis Dynam, University Pk, PA 16802 USA.
[Plowright, Raina K.; Foley, Janet E.] Univ Calif Davis, Sch Vet Med, Dept Med & Epidemiol, Davis, CA 95616 USA.
[Plowright, Raina K.; Daszak, Peter] EcoHlth Alliance, New York,

NY 10001 USA.
[Foley, Patrick] Calif State Univ Sacramento, Dept Biol Sci, Sacramento, CA 95819 USA.
[Field, Hume E.] Queensland Ctr Emerging Infect Dis, Dept Employment Econ Dev & Innovat, Archerfield Bc, Qld 4108, Australia.
[Dobson, Andy P.] Princeton Univ, Dept Ecol & Evolut Biol, Princeton, NJ 08544 USA.
[Eby, Peggy] Dept Environm & Climate Change NSW, Hurstville, NSW 2220, Australia.
C3 Pennsylvania Commonwealth System of Higher Education (PCSHE); Pennsylvania State University; Pennsylvania State University - University Park; University of California System; University of California Davis; EcoHealth Alliance; California State University System; California State University Sacramento; Princeton University
RP Plowright, RK (corresponding author), Penn State Univ, Ctr Infect Dis Dynam, University Pk, PA 16802 USA.
EM rplowright@gmail.com; daszak@ecohealthalliance.org
RI daszak, peter/U-4588-2017
FU NIH/NSF from the John E. Fogarty International Center [R01-TW05869];
Australian Biosecurity Cooperative Research Center for Emerging Infectious Diseases (AB-CRC); V. Kann Rasmussen Foundation; Australian-American Fulbright Commission; Foundation for Young Australians; David H. Smith Fellowship in Conservation Research
FX We thank Gary Tabor, Paul Cross, Rosie Woodroffe and Leslie Bienen for comments on an early draft; Kezia Manlove, Billie Roberts, Andra Toivala and Alan Swanson for technical help; Les Hall, Patrina Birt, Hamish McCallum and the Henipavirus Ecology Research Group (www.henipavirus.org) for conversations that helped inspire this paper.
This work was supported in part by an NIH/NSF 'Ecology of Infectious Diseases' award from the John E. Fogarty International Center R01-TW05869 by core funding to the Consortium for Conservation Medicine from the V. Kann Rasmussen Foundation and collaboration with the Australian Biosecurity Cooperative Research Center for Emerging Infectious Diseases (AB-CRC). R. K. P. was supported by the V. Kann Rasmussen Foundation, the Australian-American Fulbright Commission, the Foundation for Young Australians and a David H. Smith Fellowship in Conservation Research.
CR Altizer S, 2006, ECOL LETT, V9, P467, DOI 10.1111/j.1461-0248.2005.00879.x
ANDERSON R M, 1991
[Anonymous], 2000, P WORKSH ASS STAT GR
[Anonymous], CONSERVATION OUTSIDE
[Anonymous], MEASLES VIRUS ITS BI

Begon M, 1999, P ROY SOC B-BIOL SCI, V266, P1939, DOI 10.1098/rspb.1999.0870
Birt P., 2004, THESIS U QUEENSLAND
Birt Patrina, 1999, Australian Mammalogy, V21, P107
Black FL., 1991, The Paramyxoviruses ed, P509
Calisher CH, 2006, CLIN MICROBIOL REV, V19, P531, DOI 10.1128/CMR.00017-06
Coyne MJ, 2001, VET REC, V149, P509, DOI 10.1136/vr.149.17.509
Cummings DAT, 2004, NATURE, V427, P344, DOI 10.1038/nature02225
Dobson A, 2001, PHILOS T R SOC B, V356, P1001, DOI 10.1098/rstb.2001.0900
Dobson AP, 2005, SCIENCE, V310, P628, DOI 10.1126/science.1120872
Duncan A. M., 1999, ACTION PLAN AUSTR BA
EBY P, 1991, WILDLIFE RES, V18, P547, DOI 10.1071/WR9910547
Eby P., 2004, NATL COUNT GREY HEAD
Eby P., 2002, A report to: Environment Australia Queensland Parks and Wildlife Service NSW National Parks and Wildlife Service Victoria Dept Natural Resources and Environment
Eby P., 2003, A report to: A report to: Dept. Environment and Heritage Queensland Parks and Wildlife Service NSW Dept. Environment and Conservation Victoria Dept. Sustainability and Environment
Eby Peggy, 1999, Australian Zoologist, V31, P240
Field H, 2001, MICROBES INFECT, V3, P307, DOI 10.1016/S1286-4579(01)01384-3
Field H., 2004, ECOLOGY HENDRA VIRUS
Field HE, 2007, AUST VET J, V85, P268, DOI 10.1111/j.1751-0813.2007.00170.x
Grenfell B, 1997, TRENDS ECOL EVOL, V12, P395, DOI 10.1016/S0169-5347(97)01174-9
Grenfell BT, 2001, NATURE, V414, P716, DOI 10.1038/414716a
Hall L, 2000, AUSTR NATURAL HIST S
Halpin K, 2000, J GEN VIROL, V81, P1927, DOI 10.1099/0022-1317-81-8-1927
Halpin K., AM J TROP MED HYG
Halpin K., 2000, GENETIC STUDIES HEND
Halpin K, 2007, CLIN INFECT DIS, V44, P711, DOI 10.1086/511078
Henipavirus Ecology Research Group (HERG), 2006, P186
Hooper PT, 1996, AUST VET J, V74, P244, DOI 10.1111/j.1751-0813.1996.tb15414.x
Hosseini PR, 2004, P ROY SOC B-BIOL SCI, V271, P2569, DOI 10.1098/rspb.2004.2938
Hsu VP, 2004, EMERG INFECT DIS, V10, P2082, DOI 10.3201/eid1012.040701
Jones KE, 2008, NATURE, V451, P990, DOI 10.1038/nature06536
Keeling MJ, 2000, NATURE, V407, P903, DOI 10.1038/35038073
Kermack WO, 1927, P R SOC LOND A-CONTA, V115, P700, DOI 10.1098/rspa.1927.0118
Lloyd-Smith JO, 2009, SCIENCE, V326, P1362, DOI 10.1126/science.1177345
Mackenzie JS, 2001, EMERG INFECT DIS, V7, P497, DOI 10.3201/eid0707.017703
Markus N, 2004, WILDLIFE RES, V31, P345, DOI 10.1071/WR01117
Markus N, 2002, ACTA CHIROPTEROL, V4, P153, DOI 10.3161/001.004.0204

McDonald-Madden E, 2005, AUSTRAL ECOL, V30, P600, DOI 10.1111/j.1442-9993.2005.01492.x
McIlwee A. P., 2002, Australian Zoologist, V32, P76
Morse Stephen S., 1993, P10
MURRAY K, 1995, SCIENCE, V268, P94, DOI 10.1126/science.7701348
NELSON J. E., 1965, AUSTRALIAN J ZOOL, V13, P53, DOI 10.1071/ZO9650053
Noble I., 1996, AUSTR STATE ENV 1996
OSullivan JD, 1997, LANCET, V349, P93, DOI 10.1016/S0140-6736(96)06162-4
Palmer C, 1999, WILDLIFE RES, V26, P823, DOI 10.1071/WR97106
PALMER C., 1997, Ecology of the black flying fox, Pteropus alecto, in the seasonal tropics of the Northern Territory: resource tracking in a landscape mosaic and role in seed dispersal
Parry-Jones KA, 2001, AUSTRAL ECOL, V26, P47, DOI 10.1046/j.1442-9993.2001.01072.x
PARRYJONES K, 1991, WILDLIFE RES, V18, P111, DOI 10.1071/WR9910111
PARRYJONES KA, 1992, WILDLIFE RES, V19, P331, DOI 10.1071/WR9920331
Plowright RK, 2008, FRONT ECOL ENVIRON, V6, P420, DOI 10.1890/070086
Plowright RK, 2008, P ROY SOC B-BIOL SCI, V275, P861, DOI 10.1098/rspb.2007.1260
Pulliam JRC, 2008, ECOHEALTH, V5, P80, DOI 10.1007/s10393-007-0149-6
Rahman SA, 2010, EMERG INFECT DIS, V16, P1990, DOI 10.3201/eid1612.091790
Ratcliffe F, 1932, J ANIM ECOL, V1, P32, DOI 10.2307/993
Ratcliffe F. N., 1931, Bulletin Council for Scientific and Industrial Research Australia, Vno. 53, P1
Roberts BJ, 2005, THESIS GRIFFITH U NA
Shilton LA, 2008, AUSTRAL ECOL, V33, P549, DOI 10.1111/j.1442-9993.2008.01910.x
Swinton J, 1998, J ANIM ECOL, V67, P54, DOI 10.1046/j.1365-2656.1998.00176.x
Tidemann CR, 2004, J ZOOL, V263, P141, DOI 10.1017/S0952836904004960
van der Ree R, 2006, J ZOOL, V268, P177, DOI 10.1111/j.1469-7998.2005.00005.x
Vardon MJ, 1998, AUST J ZOOL, V46, P329, DOI 10.1071/ZO98023
Weiss RA, 2004, NAT MED, V10, pS70, DOI 10.1038/nm1150
WOINARSKI JCZ, 1992, GLOBAL ECOL BIOGEOGR, V2, P1, DOI 10.2307/2997325
Woolhouse MEJ, 2005, TRENDS ECOL EVOL, V20, P238, DOI 10.1016/j.tree.2005.02.009
Woolhouse MEJ, 2005, EMERG INFECT DIS, V11, P1842, DOI 10.3201/eid1112.050997
NR 69
TC 240
Z9 277
U1 1
U2 210
PU ROYAL SOC

PI LONDON
PA 6-9 CARLTON HOUSE TERRACE, LONDON SW1Y 5AG, ENGLAND
SN 0962-8452
EI 1471-2954
J9 P ROY SOC B-BIOL SCI
JI Proc. R. Soc. B-Biol. Sci.
PD DEC 22
PY 2011
VL 278
IS 1725
BP 3703
EP 3712
DI 10.1098/rspb.2011.0522
PG 10
WC Biology; Ecology; Evolutionary Biology
WE Science Citation Index Expanded (SCI-EXPANDED)
SC Life Sciences & Biomedicine - Other Topics; Environmental Sciences &
   Ecology; Evolutionary Biology
GA 846CK
UT WOS:000296874600011
PM 21561971
OA Green Published, Green Accepted
DA 2024-10-21
ER

PT J
AU Zhou, H
   Li, J
   Zhou, DW
   Wu, YJ
   Wang, XL
   Zhou, J
   Ma, QQ
   Yao, XS
   Ma, L
AF Zhou, Hao
   Li, Jun
   Zhou, Dewei
   Wu, Yingjie
   Wang, Xingliang
   Zhou, Jiang
   Ma, Qingqing
   Yao, Xinsheng
   Ma, Long
TI New insights into the germline genes and CDR3 repertoire of the TCRb
   chain in <i>Chiroptera</i>
SO FRONTIERS IN IMMUNOLOGY
LA English
DT Article
DE bat; TR loci annotation; high-throughput sequencing; TCR beta chain
   repertoire; germline genes

ID SCANNING-ELECTRON; BAT; IDENTIFICATION; EXPRESSION; REVEAL; CELLS
AB Introduction: Bats are recognized as natural reservoirs for many viruses, and their unique immune system enables them to coexist with these viruses without frequently exhibiting disease symptoms. However, the current understanding of the bat adaptive immune system is limited due to the lack of a database or tool capable of processing T-cell receptor (TCR) sequences for bats.

Methods: We performed germline gene annotation in three bat species using homologous genes and RSSs (Recombinational Signal Sequences) scanning method. Then we used the conserved C gene to construct the TCRb chain receptor library of the Intermediate Horseshoe Bat. Bats ' TCRb data will be analyzed using MiXCR and constructed reference library.

Results: Regarding the annotation results, we found that the Pale Spear-nosed Bat has 37 members in the TRBV12 family, which is more than the total number of TRBV genes in the Greater Horseshoe Bat. The average number of unique TCRb chain receptor sequences in each Intermediate Horseshoe Bat sample reached 24,904.

Discussion: The distinct variations in the distribution of TRBV genes among the three types of bats could have a direct impact on the diversity of the TCR repertoire, as evidenced by the presence of conserved amino acids that indicate the T-cell recognition of antigens in bats is MHC-restricted. The bats ' TCRb repertoire is formed through the rearrangement of the V-D-J-C genes, with D-J/V-D deletions and insertions resulting in high diversity.
C1 [Zhou, Hao; Li, Jun; Zhou, Dewei; Wu, Yingjie; Ma, Qingqing; Yao, Xinsheng; Ma, Long] Zunyi Med Univ, Ctr Immunomolecular Engn, Dept Immunol, Innovat & Practice Base Grad Students Educ, Zunyi, Peoples R China.

[Zhou, Hao] Osaka Univ, Res Inst Microbial Dis, Dept Genome Informat, Suita, Japan.

[Wang, Xingliang; Zhou, Jiang] Guizhou Normal Univ, Sch Life Sci, Guiyang, Peoples R China.
C3 Zunyi Medical University; Osaka University; Guizhou Normal University
RP Yao, XS; Ma, L (corresponding author), Zunyi Med Univ, Ctr Immunomolecular Engn, Dept Immunol, Innovat & Practice Base Grad Students Educ, Zunyi, Peoples R China.
EM immunology@126.com; 164849671@qq.com
RI Ma, Linan/GYR-2386-2022; ZHOU, Jiang/AAH-2558-2020; Wang, Xingliang/J-8757-2014
FU National Natural Science Foundation of China [31860257, 81860300];
Guizhou Provincial Hundred Talent Fund [(2018) 5637]
FX This study was supported by the National Natural Science Foundation of China (31860257 & 81860300) and the Guizhou Provincial Hundred Talent Fund [No. (2018) 5637].
CR Ahn M, 2019, NAT MICROBIOL, V4, P789, DOI 10.1038/s41564-019-0371-3
Ahn M, 2016, SCI REP-UK, V6, DOI 10.1038/srep21722
Alli R, 2011, PLOS ONE, V6, DOI 10.1371/journal.pone.0018027
Andersen KG, 2020, NAT MED, V26, P450, DOI 10.1038/

s41591-020-0820-9
Antonacci R, 2019, FRONT GENET, V10, DOI 10.3389/fgene.2019.00482
Bolotin DA, 2015, NAT METHODS, V12, P380, DOI 10.1038/nmeth.3364
Burgin CJ, 2018, J MAMMAL, V99, P1, DOI 10.1093/jmammal/gyx147
CIBOTTI R, 1994, J EXP MED, V180, P861, DOI 10.1084/jem.180.3.861
Connelley T, 2009, BMC GENOMICS, V10, DOI 10.1186/1471-2164-10-192
Connelley TK, 2014, BMC GENOMICS, V15, DOI 10.1186/1471-2164-15-994
Fu LF, 2017, PLOS ONE, V12, DOI 10.1371/journal.pone.0182733
Gerritsen B, 2020, IMMUNOGENETICS, V72, P101, DOI 10.1007/s00251-019-01142-9
Giannico F, 2020, BMC GENOMICS, V21, DOI 10.1186/s12864-020-07022-x
Giudicelli Veronique, 2012, Frontiers in Genetics, V3, P79, DOI 10.3389/fgene.2012.00079
Goh G, 2020, P NATL ACAD SCI USA, V117, P28939, DOI 10.1073/pnas.2003352117
Gómez JMM, 2016, SCI REP-UK, V6, DOI 10.1038/srep37796
Greenaway HY, 2009, IMMUNOL CELL BIOL, V87, P546, DOI 10.1038/icb.2009.38
Hansen TO, 2015, J IMMUNOL, V194, P973, DOI 10.4049/jimmunol.1401443
HAQQI TM, 1992, P NATL ACAD SCI USA, V89, P1253, DOI 10.1073/pnas.89.4.1253
Irving AT, 2021, NATURE, V589, P363, DOI 10.1038/s41586-020-03128-0
Jaiswal S, 2022, BMC GENOMICS, V23, DOI 10.1186/s12864-022-08867-0
Jebb D, 2020, NATURE, V583, P578, DOI 10.1038/s41586-020-2486-3
Koay HF, 2019, NAT COMMUN, V10, DOI 10.1038/s41467-019-10198-w
Lane J, 2010, BMC BIOINFORMATICS, V11, DOI 10.1186/1471-2105-11-223
Larson PA, 2021, CELL REP, V35, DOI 10.1016/j.celrep.2021.109140
Letko M, 2020, NAT REV MICROBIOL, V18, P461, DOI 10.1038/s41579-020-0394-z
Li S, 2013, NAT COMMUN, V4, DOI 10.1038/ncomms3333
Mage RG, 1998, IMMUNOL REV, V162, P49, DOI 10.1111/j.1600-065X.1998.tb01428.x
MAKANYA AN, 1994, AFR J ECOL, V32, P158, DOI 10.1111/j.1365-2028.1994.tb00566.x
Masuda K, 1997, J IMMUNOL, V158, P2076
Merelli I, 2010, NUCLEIC ACIDS RES, V38, pW262, DOI 10.1093/nar/gkq391
Mineccia M, 2012, DEV COMP IMMUNOL, V37, P279, DOI 10.1016/j.dci.2012.03.010
Ng JHJ, 2016, SCI REP-UK, V6, DOI 10.1038/srep21256
Park JE, 2020, SCIENCE, V367, P868, DOI 10.1126/science.aay3224
Pavlovich SS, 2018, CELL, V173, P1098, DOI 10.1016/j.cell.2018.03.070
Pégorier P, 2020, FRONT IMMUNOL, V11, DOI 10.3389/fimmu.2020.00821
Qu ZH, 2019, J IMMUNOL, V202, P3493, DOI 10.4049/jimmunol.1900001
Qurkhuli T, 2019, IMMUNOGENETICS, V71, P575, DOI 10.1007/

s00251-019-01128-7
Radtanakatikanon A, 2020, BMC GENOMICS, V21, DOI 10.1186/s12864-019-6431-5
RAMSDEN DA, 1994, NUCLEIC ACIDS RES, V22, P1785, DOI 10.1093/nar/22.10.1785
Randolph HE, 2018, IMMUNITY, V48, P1074, DOI 10.1016/j.immuni.2018.05.016
REYNAUD CA, 1987, CELL, V48, P379, DOI 10.1016/0092-8674(87)90189-9
Rhie A, 2021, NATURE, V592, P737, DOI 10.1038/s41586-021-03451-0
Ruiz-Aravena M, 2022, NAT REV MICROBIOL, V20, P299, DOI 10.1038/s41579-021-00652-2
SARKAR SK, 1991, DEV COMP IMMUNOL, V15, P423, DOI 10.1016/0145-305X(91)90034-V
Six A, 2013, FRONT IMMUNOL, V4, DOI 10.3389/fimmu.2013.00413
Song L, 2021, NAT METHODS, V18, P627, DOI 10.1038/s41592-021-01142-2
Sotero-Caio CG, 2017, GENES-BASEL, V8, DOI 10.3390/genes8100272
Strobel S, 2015, EUR J HISTOCHEM, V59, P107, DOI 10.4081/ejh.2015.2477
Turner SJ, 2006, NAT REV IMMUNOL, V6, P883, DOI 10.1038/nri1977
Xie JZ, 2018, CELL HOST MICROBE, V23, P297, DOI 10.1016/j.chom.2018.01.006
Zhou H, 2021, FRONT IMMUNOL, V12, DOI 10.3389/fimmu.2021.741408
Zhou P., 2020, NATURE, V579, P270, DOI DOI 10.1038/s41586-020-2012-7
NR 53
TC 4
Z9 4
U1 1
U2 4
PU FRONTIERS MEDIA SA
PI LAUSANNE
PA AVENUE DU TRIBUNAL FEDERAL 34, LAUSANNE, CH-1015, SWITZERLAND
SN 1664-3224
J9 FRONT IMMUNOL
JI Front. Immunol.
PD MAR 27
PY 2023
VL 14
AR 1147859
DI 10.3389/fimmu.2023.1147859
PG 11
WC Immunology
WE Science Citation Index Expanded (SCI-EXPANDED)
SC Immunology
GA D2UV2
UT WOS:000967333400001
PM 37051236
OA Green Published, gold
DA 2024-10-21
ER

PT J

AU Johnson, JS
Reeder, DM
Lilley, TM
Czirják, GA
Voigt, CC
McMichael, JW
Meierhofer, MB
Seery, CW
Lumadue, SS
Altmann, AJ
Toro, MO
Field, KA
AF Johnson, Joseph S.
Reeder, DeeAnn M.
Lilley, Thomas M.
Czirjak, Gabor A.
Voigt, Christian C.
McMichael, James W., III
Meierhofer, Melissa B.
Seery, Christopher W.
Lumadue, Shayne S.
Altmann, Alexander J.
Toro, Michael O.
Field, Kenneth A.
TI Antibodies to <i>Pseudogymnoascus destructans</i> are not sufficient for
protection against white-nose syndrome
SO ECOLOGY AND EVOLUTION
LA English
DT Article
DE Antibody-mediated immunity; antifungal immunity; Corynorhinus; disease
ecology; Eptesicus; hibernation; Myotis; Perimyotis; wildlife disease;
WNS
ID MYOTIS MYOTIS-LUCIFUGUS; GROUND-SQUIRREL; VESPERTILIONID BATS;
HOST-DEFENSE; TH17 CELLS; HIBERNATION; GEOMYCES; PATHOGEN; DURATION;
IMMUNITY
AB White-nose syndrome (WNS) is a fungal disease caused by Pseudogymnoascus destructans (Pd) that affects bats during hibernation. Although millions of bats have died from WNS in North America, mass mortality has not been observed among European bats infected by the fungus, leading to the suggestion that bats in Europe are immune. We tested the hypothesis that an antibody-mediated immune response can provide protection against WNS by quantifying antibodies reactive to Pd in blood samples from seven species of free-ranging bats in North America and two free-ranging species in Europe. We also quantified antibodies in blood samples from little brown myotis (Myotis lucifugus) that were part of a captive colony that we injected with live Pd spores mixed with adjuvant, as well as individuals surviving a captive Pd infection trial. Seroprevalence of antibodies against Pd, as well as antibody titers, was greater among little brown myotis than among four other

species of cave-hibernating bats in North America, including species with markedly lower WNS mortality rates. Among little brown myotis, the greatest titers occurred in populations occupying regions with longer histories of WNS, where bats lacked secondary symptoms of WNS. We detected antibodies cross-reactive with Pd among little brown myotis naive to the fungus. We observed high titers among captive little brown myotis injected with Pd. We did not detect antibodies against Pd in Pd-infected European bats during winter, and titers during the active season were lower than among little brown myotis. These results show that antibody-mediated immunity cannot explain survival of European bats infected with Pd and that little brown myotis respond differently to Pd than species with higher WNS survival rates. Although it appears that some species of bats in North America may be developing resistance to WNS, an antibody-mediated immune response does not provide an explanation for these remnant populations.
C1 [Johnson, Joseph S.; Reeder, DeeAnn M.; McMichael, James W., III; Meierhofer, Melissa B.; Seery, Christopher W.; Lumadue, Shayne S.; Altmann, Alexander J.; Toro, Michael O.; Field, Kenneth A.] Bucknell Univ, Dept Biol, Lewisburg, PA 17837 USA.
[Lilley, Thomas M.] Univ Turku, Dept Biol, SF-20500 Turku, Finland.
[Czirjak, Gabor A.; Voigt, Christian C.] Leibniz Inst Zoo & Wildlife Res, Berlin, Germany.
C3 Bucknell University; University of Turku; Leibniz Institut fur Zoo und
Wildtierforschung
RP Field, KA (corresponding author), Bucknell Univ, Dept Biol, Lewisburg, PA 17837 USA.
EM kfield@bucknell.edu
RI Meierhofer, Melissa/AAS-3473-2021; Czirják, Gábor/F-5440-2011; Lilley,
Thomas/F-2236-2015
OI Czirjak, Gabor Arpad/0000-0001-9488-0069; Lilley,
Thomas/0000-0001-5864-4958; Reeder, DeeAnn/0000-0001-8651-2012;
Meierhofer, Melissa B./0000-0003-2384-1999; Field,
Kenneth/0000-0001-7417-4386
FU United States Fish and Wildlife Service [F12AP01210]; Woodtiger
Foundation
FX Funding for this project was provided by the United States Fish and
Wildlife Service grant F12AP01210 (DMR and KAF) and the Woodtiger
Foundation (DMR).
CR BEAUDOIN RL, 1969, EXP PARASITOL, V24, P42, DOI 10.1016/0014-4894(69)90219-7
Blehert DS, 2012, PLOS PATHOG, V8, DOI 10.1371/journal.ppat.1002779
Blehert DS, 2009, SCIENCE, V323, P227, DOI 10.1126/science.1163874
Bouma HR, 2010, VET IMMUNOL IMMUNOP, V136, P319, DOI 10.1016/j.vetimm.2010.03.016
Bouma HR, 2013, DEV COMP IMMUNOL, V39, P154, DOI 10.1016/j.dci.2012.11.004
Bouma HR, 2011, P NATL ACAD SCI USA, V108, P2052, DOI 10.1073/

pnas.1008823108
Bouma HR, 2010, J LEUKOCYTE BIOL, V88, P619, DOI 10.1189/jlb.0310174
BRACK V, 1985, CAN J ZOOL, V63, P2952, DOI 10.1139/z85-442
Brook CE, 2015, TRENDS MICROBIOL, V23, P172, DOI 10.1016/j.tim.2014.12.004
Brownlee-Bouboulis SA, 2013, J WILDLIFE DIS, V49, P850, DOI 10.7589/2012-10-242
Burton RS, 1999, FUNCT ECOL, V13, P232, DOI 10.1046/j.1365-2435.1999.00302.x
Casadevall A, 2012, CELL HOST MICROBE, V11, P447, DOI 10.1016/j.chom.2012.04.004
Casadevall A, 2012, NAT IMMUNOL, V13, P21, DOI 10.1038/ni.2184
Cenci E, 1997, INFECT IMMUN, V65, P564, DOI 10.1128/IAI.65.2.564-570.1997
Coleman J., 2014, Outlooks on Pest Management, P374, DOI DOI 10.1564/V25_DEC_08
Conti HR, 2009, J EXP MED, V206, P299, DOI 10.1084/jem.20081463
Cryan PM, 2013, J WILDLIFE DIS, V49, P398, DOI 10.7589/2012-04-121
Cryan PM, 2010, BMC BIOL, V8, DOI 10.1186/1741-7007-8-135
Dobony CA, 2011, J FISH WILDL MANAG, V2, P190, DOI 10.3996/022011-JFWM-014
Frank CL, 2014, PLOS ONE, V9, DOI 10.1371/journal.pone.0113958
Frick WF, 2015, GLOBAL ECOL BIOGEOGR, V24, P741, DOI 10.1111/geb.12290
Frick WF, 2010, SCIENCE, V329, P679, DOI 10.1126/science.1188594
Gargas A, 2009, MYCOTAXON, V108, P147, DOI 10.5248/108.147
Haraguchi N, 2010, INFECT IMMUN, V78, P2302, DOI 10.1128/IAI.01398-09
Hernández-Santos N, 2012, CELL HOST MICROBE, V11, P425, DOI 10.1016/j.chom.2012.04.008
Humphries MM, 2002, NATURE, V418, P313, DOI 10.1038/nature00828
Jain AV, 2009, INFECT IMMUN, V77, P5389, DOI 10.1128/IAI.00809-09
Johnson J. S., 2014, PLOS ONE, V11
Johnson JS, 2012, PLOS ONE, V7, DOI 10.1371/journal.pone.0049754
Lorch JM, 2013, MYCOLOGIA, V105, P237, DOI 10.3852/12-207
Lorch JM, 2011, NATURE, V480, P376, DOI 10.1038/nature10590
Maniero GD, 2002, DEV COMP IMMUNOL, V26, P563, DOI 10.1016/S0145-305X(02)00006-X
Meteyer CU, 2012, VIRULENCE, V3, P583, DOI 10.4161/viru.22330
Meteyer CU, 2009, J VET DIAGN INVEST, V21, P411, DOI 10.1177/104063870902100401
Minnis AM, 2013, FUNGAL BIOL-UK, V117, P638, DOI 10.1016/j.funbio.2013.07.001
Moore MS, 2013, PLOS ONE, V8, DOI 10.1371/journal.pone.0058976
Pikula J, 2012, J WILDLIFE DIS, V48, P207, DOI 10.7589/0090-3558-48.1.207
Puechmaille SJ, 2011, PLOS ONE, V6, DOI 10.1371/journal.pone.0019167
Puechmaille SJ, 2010, EMERG INFECT DIS, V16, P290, DOI 10.3201/eid1602.091391
Rappleye CA, 2007, P NATL ACAD SCI USA, V104, P1366, DOI 10.1073/pnas.0609848104

Reeder DeeAnn M., 2013, P413
Reeder DM, 2012, PLOS ONE, V7, DOI 10.1371/journal.pone.0038920
Reeder Deeann M., 2009, P554
Reichard JD, 2014, NORTHEAST NAT, V21, pN56, DOI 10.1656/045.021.0410
Reichard JD, 2009, ACTA CHIROPTEROL, V11, P457, DOI 10.3161/150811009X485684
Spellberg B, 2008, J INFECT DIS, V197, P967, DOI 10.1086/529204
Torosantucci A, 2005, J EXP MED, V202, P597, DOI 10.1084/jem.20050749
Turner Gregory G., 2011, Bat Research News, V52, P13
TWENTE JW, 1985, CAN J ZOOL, V63, P2955, DOI 10.1139/z85-443
Verant Michelle L., 2014, BMC Physiology, V14, P10, DOI 10.1186/s12899-014-0010-4
Verant ML, 2012, PLOS ONE, V7, DOI 10.1371/journal.pone.0046280
Warnecke L, 2012, P NATL ACAD SCI USA, V109, P6999, DOI 10.1073/pnas.1200374109
Webb PI, 1996, CAN J ZOOL, V74, P761, DOI 10.1139/z96-087
Wibbelt G, 2013, PLOS ONE, V8, DOI 10.1371/journal.pone.0074105
Wilcox A, 2014, ANIM BEHAV, V88, P157, DOI 10.1016/j.anbehav.2013.11.026
Wüthrich M, 2012, ANNU REV IMMUNOL, V30, P115, DOI 10.1146/annurev-immunol-020711-074958
NR 56
TC 31
Z9 34
U1 1
U2 87
PU WILEY-BLACKWELL
PI HOBOKEN
PA 111 RIVER ST, HOBOKEN 07030-5774, NJ USA
SN 2045-7758
J9 ECOL EVOL
JI Ecol. Evol.
PD JUN
PY 2015
VL 5
IS 11
BP 2203
EP 2214
DI 10.1002/ece3.1502
PG 12
WC Ecology; Evolutionary Biology
WE Science Citation Index Expanded (SCI-EXPANDED)
SC Environmental Sciences & Ecology; Evolutionary Biology
GA CJ8DZ
UT WOS:000355731300008
PM 26078857
OA Green Published, gold
DA 2024-10-21
ER

PT J
AU Frank, R

Kuhn, T
Werblow, A
Liston, A
Kochmann, J
Klimpel, S
AF Frank, Raphael
Kuhn, Thomas
Werblow, Antje
Liston, Andrew
Kochmann, Judith
Klimpel, Sven
TI Parasite diversity of European <i>Myotis</i> species with special emphasis on <i>Myotis myotis</i> (Microchiroptera, Vespertilionidae) from a typical nursery roost
SO PARASITES & VECTORS
LA English
DT Article
DE Myotis myotis; Spinturnix myoti; Ectoparasites; Roosting place
ID MESOSTIGMATA SPINTURNICIDAE; BAT HOST; CHIROPTERA; ACARI; ECTOPARASITES; EVOLUTION; NEMATODA; ROMANIA; DIPTERA; SEARCH
AB Background: Bats belong to one of the most species-rich orders within the Mammalia. They show a worldwide distribution, a high degree of ecological diversification as well as a high diversity of associated parasites and pathogens. Despite their prominent and unique role, the knowledge of their parasite-host-relationships as well as the mechanisms of co-evolutionary processes are, partly due to strict conservation regulations, scarce.

Methods: Juvenile specimens of the greater mouse-eared bat (Myotis myotis) from a roosting colony in Gladenbach (Hesse, Germany) were examined for their metazoan endo-and ectoparasite infections and pathogens. Morphometric data were recorded and the individuals were checked for Lyssavirus-specific antigen using a direct immunofluorescence test. For unambiguous species identification, the bats were analysed by cyt-b sequence comparison.

Results: Myotis myotis were parasitized by the six insect and arachnid ectoparasite species, i.e. Ixodes ricinus, Ischnopsyllus octactenus, Ichoronyssus scutatus, Steatonyssus periblepharus, Spinturnix myoti and Cimex dissimilis. Additionally, the nematode Molinostrongylus alatus and the cestode Vampirolepis balsaci were recorded. Each bat was parasitized by at least four species. The parasites showed partially extreme rates of infection, never recorded before, with more than 1,440 parasites per single host. Ichoronyssus scutatus, Steatonyssus periblepharus, Vampirolepis balsaci and Molinostrongylus alatus are recorded for the first time in Germany. A checklist for Europe is presented containing records of 98 parasite species of 14 Myotis species.

Conclusions: The Myotis myotis from Gladenbach (Hesse, Germany) were parasitized by a diverse parasite fauna with high infestation rates. We assume that in juvenile Myotis the number of parasites is generally higher than in adults due to only later acquired immune competence and behavioural adaptations. Our results revealed new insights into parasite fauna of M. myotis and European bats in

general. The finding of endoparasitic cyclophyllidean cestodes that have a two-host lifecycle is, considering the stationary behaviour of the juvenile bats, rather unusual and suggests a non-predatory transmission mechanism (e.g. via autoinfection). A new insight gained from the collated literature was that the European wide composition of the Myotis parasite fauna is dominated by a few specific taxonomic groups in Europe.
C1 [Frank, Raphael; Kuhn, Thomas; Werblow, Antje; Kochmann, Judith; Klimpel, Sven] Goethe Univ Frankfurt, Inst Ecol Evolut & Divers, Senckenberg Biodivers & Climate Res Ctr SBiK F, SGN, D-60438 Frankfurt, Germany.
[Liston, Andrew] Senckenberg German Entomol Inst SDEI, D-15374 Muencheberg, Germany.
C3 Goethe University Frankfurt; Senckenberg Biodiversitat & Klima-Forschungszentrum (BiK-F); Senckenberg Gesellschaft fur Naturforschung
(SGN); Senckenberg Gesellschaft fur Naturforschung (SGN)
RP Klimpel, S (corresponding author), Goethe Univ Frankfurt, Inst Ecol Evolut & Divers, Senckenberg Biodivers & Climate Res Ctr SBiK F, SGN, Max von Laue Str 13, D-60438 Frankfurt, Germany.
EM klimpel@bio.uni-frankfurt.de
RI Kochmann, Judith/AAG-3574-2021; Liston, Andrew/AAD-2628-2020
OI Kochmann, Judith/0000-0001-6312-7859
CR ALTSCHUL SF, 1990, J MOL BIOL, V215, P403, DOI 10.1016/S0022-2836(05)80360-2
[Anonymous], PARASIT WIRT BEZIEHU
[Anonymous], 2014, PARASITE VECTOR
[Anonymous], 2007, FLEAS SIPHONAPTERA F
[Anonymous], 1998, The Mathematical Theory of Communication 1949
Baker Anne S., 2003, Systematic and Applied Acarology Special Publications, V14, P1
BARUS V, 1972, Folia Parasitologica (Ceske Budejovice), V19, P111
Berthier P, 2006, P R SOC B, V42, P143
Bogdanowicz W, 2009, ACTA CHIROPTEROL, V11, P289, DOI 10.3161/150811009X485530
BOTELLA P, 1995, FOLIA PARASIT, V42, P143
BRGLEZ J, 1987, Zbornik Biotehniske Fakultete Univerze Edvarda Kardelja v Ljubljani Veterinarstvo, V24, P167
Bruyndonckx N, 2010, MOL ECOL, V19, P2770, DOI 10.1111/j.1365-294X.2010.04699.x
Bruyndonckx N, 2009, MOL PHYLOGENET EVOL, V51, P227, DOI 10.1016/j.ympev.2009.02.005
Bush AO, 1997, J PARASITOL, V83, P575, DOI 10.2307/3284227
Castella V, 2000, MOL ECOL, V9, P1761, DOI 10.1046/j.1365-294x.2000.01069.x
Christe P, 2003, J ANIM ECOL, V72, P866, DOI 10.1046/j.1365-2656.2003.00759.x
Christe P, 2000, ECOL LETT, V3, P207
Christe P, 2007, J ANIM ECOL, V76, P703, DOI 10.1111/j.1365-2656.2007.01255.x
Danko Stefan, 2010, Vespertilio, V13-14, P77
Datzmann T, 2010, BMC EVOL BIOL, V10, DOI 10.1186/1471-2148-10-165
Dekeukeleire D., 2012, THESIS RES GROUP EVO

DENEGRI GM, 1993, EXP APPL ACAROL, V17, P567, DOI 10.1007/BF00053486
Deunff J, 2004, J MED ENTOMOL, V41, P826, DOI 10.1603/0022-2585-41.5.826
Dietz M, 2013, HESSEN FORST FENA NA
Drexler JF, 2012, EMERG INFECT DIS, V17, P449
Esteban J.G., 1999, Research and Reviews in Parasitology, V59, P57
ESTEBAN JG, 1991, FOLIA PARASIT, V38, P319
Frank R, 2014, PARASITOL RES MG, V5, P87, DOI 10.1007/978-3-642-39333-4_5
Friedrich-Loeffler-Institut Bundesforschungsinstitut fur Tiergesundheit, 2014, AMTL METH JAN 2014
Genov Todor, 1992, Parasitologia Hungarica, V25, P53
Giorgi MS, 2001, P R SOC B, V268, P2070
Gloza-Rausch F, 2008, EMERG INFECT DIS, V14, P626, DOI 10.3201/eid1404.071439
Gunnell Gregg F., 2005, Journal of Mammalian Evolution, V12, P209, DOI 10.1007/s10914-005-6945-2
Haitlinger Ryszard, 2008, Wiad Parazytol, V54, P123
Haitlinger Ryszard, 2012, Annals of Parasitology, V58, P15
Hennecke M, 1994, FLEDERMAUSE HESSENS
Hutson A, 2001, Microchiropteran bats: global status survey and conservation action plan, volume56
Ivanova NV, 2006, MOL ECOL NOTES, V6, P998, DOI 10.1111/j.1471-8286.2006.01428.x
Jaunbauere Gunita, 2008, Latvijas Entomologs, V45, P38
Kemenesi G, 2014, ACTA VIROL, V58, P95, DOI 10.4149/av_2014_01_95
King A. A., 2004, Historical perspective of rabies in Europe and the Mediterranean Basin: a testament to rabies by Dr Arthur A. King, P221
KOCHSEDER G, 1969, Z PARASITENK, V32, P43
Kristofik Jan, 2012, Vespertilio, V16, P167
Kristofik Jan, 2012, Polish Journal of Entomology, V81, P101, DOI 10.2478/v10200-011-0069-1
Kulzer E, 2005, HDB ZOOLOGIE CHIROPT, V3
Lanza B., 1999, I parassiti dei pipistrelli (Mammalia, Chiroptera) della fauna italiana
Lindecke Oliver, 2011, Hercynia, V44, P241
Lourenço SI, 2007, J ZOOL, V273, P161, DOI 10.1111/j.1469-7998.2007.00322.x
Lucan RK, 2006, FOLIA PARASIT, V53, P147, DOI 10.14411/fp.2006.019
MESZAROS F, 1971, Allattani Kozlemenyek, V58, P78
Mihalca AD, 2012, EXP APPL ACAROL, V58, P183, DOI 10.1007/s10493-012-9566-5
Müller T, 2007, ARCH VIROL, V152, P273, DOI 10.1007/s00705-006-0853-5
MURAI E, 1976, Parasitologia Hungarica, V9, P41
Orlova M. V., 2014, Russian Journal of Biological Invasions, V5, P29, DOI 10.1134/S207511171401007X
Orlova Maria V., 2012, Annals of Parasitology, V58, P211
Piksa K, 2011, FOLIA PARASIT, V58, P322, DOI 10.14411/fp.2011.033
Pocora I, 2013, INT J ACAROL, V39, P153, DOI

10.1080/01647954.2012.757251
Poinar G, 2012, SYST PARASITOL, V81, P79, DOI 10.1007/s11230-011-9339-2
Racey PA, 2013, ZOONOSES PUBLIC HLTH, V60, P58, DOI 10.1111/j.1863-2378.2012.01533.x
Radovsky F., 1967, The Macronyssidae and Laelapidae parasitic on bats
Ruedi M, 2001, MOL PHYLOGENET EVOL, V21, P436, DOI 10.1006/mpev.2001.1017
Rupp Doris, 2004, Spixiana, V27, P185
SCHMOLZER K, 1995, CATALOGUS FAUNAE AUS
Sevcík M, 2013, TURK J ZOOL, V37, P31, DOI 10.3906/zoo-1112-20
Sevcik Martin, 2010, Vespertilio, V13-14, P139
Shimalov VV, 2002, PARASITOL RES, V88, P1011, DOI 10.1007/s004360100415
Simmons NB, 2005, SCIENCE, V307, P527, DOI 10.1126/science.1108871
Siuda Krzysztof, 2009, Wiad Parazytol, V55, P39
Stadelmann B, 2004, ACTA CHIROPTEROL, V6, P177, DOI 10.3161/001.006.0201
Steinmann P, 2012, PARASITOL RES, V111, P749, DOI 10.1007/s00436-012-2895-9
Tian ZZ, 2012, INT J ACAROL, V38, P179, DOI 10.1080/01647954.2011.633559
Till W. M., 1964, Bulletin of the British Museum (Natural History) Zoology, V11, P511
Traversa D, 2013, PARASITE VECTOR, V6, DOI 10.1186/1756-3305-6-59
Uhrin M, 2010, ACTA THERIOL, V55, P153, DOI 10.4098/j.at.0001-7051.073.2009
Volleth M, 2012, CYTOGENET GENOME RES, V137, P165, DOI 10.1159/000338929
Walter Gottfried, 1994, Senckenbergiana Biologica, V74, P103
Zahn A, 2004, J ZOOL, V262, P383, DOI 10.1017/S0952836903004722
ZDZITOWIECKI K, 1970, Acta Parasitologica Polonica, V17, P175
NR 78
TC 24
Z9 27
U1 0
U2 27
PU BMC
PI LONDON
PA CAMPUS, 4 CRINAN ST, LONDON N1 9XW, ENGLAND
SN 1756-3305
J9 PARASITE VECTOR
JI Parasites Vectors
PD FEB 15
PY 2015
VL 8
AR 101
DI 10.1186/s13071-015-0707-7
PG 13
WC Parasitology; Tropical Medicine
WE Science Citation Index Expanded (SCI-EXPANDED)
SC Parasitology; Tropical Medicine

GA CC2LJ
UT WOS:000350176200001
PM 25880235
OA Green Published, gold
DA 2024-10-21
ER

PT J
AU Schad, J
Voigt, CC
Greiner, S
Dechmann, DKN
Sommer, S
AF Schad, Julia
Voigt, Christian C.
Greiner, Sabine
Dechmann, Dina K. N.
Sommer, Simone
TI Independent evolution of functional MHC class II <i>DRB</i> genes in New
World bat species
SO IMMUNOGENETICS
LA English
DT Article
DE Major histocompatibility complex; DRB evolution; Carollia perspicillata;
Noctilio albiventris; Noctilio leporinus; Saccopteryx bilineata
ID MAJOR HISTOCOMPATIBILITY COMPLEX; SEQUENCE DIVERSITY; RECOMBINATION
DRIVE; POSITIVE SELECTION; NATURAL-SELECTION; HIGH SIMILARITY;
POLYMORPHISM; ORIGIN; LOCI; CHIROPTERA
AB Genes of the major histocompatibility complex (MHC) play a pivotal role in the vertebrate immune system and are attractive markers for functional, fitness-related, genetic variation. Although bats (Chiroptera) represent the second largest mammalian order and are prone to various emerging infectious diseases, little is known about MHC evolution in bats. In the present study, we examined expressed MHC class II DRB sequences (exons 1 to 4) of New World bat species, Saccopteryx bilineata, Carollia perspicillata, Noctilio albiventris and Noctilio leporinus (only exon 2). We found a wide range of copy number variation of DRB loci with one locus detected in the genus Noctilio and up to ten functional loci observed in S. bilineata. Sequence variation between alleles of the same taxa was high with evidence for positive selection. We found statistical support for recombination or gene conversion events among sequences within the same but not between bat species. Phylogenetic relationships among DRB alleles provided strong evidence for independent evolution of the functional MHC class II DRB genes in the three investigated species, either by recent gene duplication, or homogenization of duplicated loci by frequent gene conversion events. Phylogenetic analysis of all available chiropteran DRB exon 2 sequences confirmed their monophyletic origin within families, but revealed a possible trans-species mode of evolution pattern in congeneric bat species, e.g. within the genera Noctilio and Myotis.

This is the first study investigating phylogenetic relationships of MHC genes within bats and therefore contributes to a better understanding of MHC evolution in one of the most dominant mammalian order.
C1 [Schad, Julia; Voigt, Christian C.; Greiner, Sabine; Dechmann, Dina K. N.; Sommer, Simone] Leibniz Inst Zoo & Wildlife Res, D-10315 Berlin, Germany.
[Dechmann, Dina K. N.] Vogelwarte Radolfzell, Max Planck Inst Ornithol, D-78315 Radolfzell am Bodensee, Germany.
C3 Leibniz Institut fur Zoo und Wildtierforschung; Max Planck Society
RP Sommer, S (corresponding author), Leibniz Inst Zoo & Wildlife Res, Alfred Kowalke Str 17, D-10315 Berlin, Germany.
EM Sommer@izw-berlin.de
RI Dechmann, Dina/A-1827-2010
OI Dechmann, Dina K. N./0000-0003-0043-8267
FU Leibniz Institute for Zoo and Wildlife Research (IZW), Germany
FX We would like to thank PD Dr. Karl-Heinz Esser of the Tierarztliche Hochschule Hannover, Germany for providing a specimen of C. perspicillata for our cDNA analyses. We thank Anke Schmidt for technical laboratory assistance and Aines Castro-Prieto and Pablo Santos for helpful comments on earlier versions of the manuscript. We further thank Charles Gunn for linguistic advice as well as an anonymous reviewer for the constructive scientific comments on the former version of the manuscript. All capture and handling of animals as well as collection and export of samples was done in concordance with national laws. Field work and animal handling was carried out according to the protocol approved by the Smithsonian Tropical Research Institute-Institutional Animal Care and Use Committee (STRI-IUCAC). Permits were issued from the Panamanian authority Autoridad National del Ambiente (ANAM, SE/A 98-08, SEX/A78-08, SEX/A-138-08) and from the Ministerio del Ambiente y Energia, Costa Rica (MINAE, Permit Number: No 183-2008-SINAC). This work was supported by the Leibniz Institute for Zoo and Wildlife Research (IZW), Germany.
CR Anisimova M, 2003, GENETICS, V164, P1229
Axtner J., 2011, Inf Gen Evol, P2063
Babik W, 2007, ACTA THERIOL, V52, P227, DOI 10.1007/BF03194219
Baker ML, 2010, IMMUNOGENETICS, V62, P173, DOI 10.1007/s00251-010-0425-4
Bollmer JL, 2010, J HERED, V101, P448, DOI 10.1093/jhered/esq018
Bowen L, 2004, IMMUNOGENETICS, V56, P12, DOI 10.1007/

s00251-004-0655-4
Bowen L, 2002, IMMUNOGENETICS, V54, P332, DOI 10.1007/s00251-002-0472-6
BROWN JH, 1993, NATURE, V364, P33, DOI 10.1038/364033a0
Bryja J, 2006, IMMUNOGENETICS, V58, P191, DOI 10.1007/s00251-006-0085-6
Burri R, 2008, MOL BIOL EVOL, V25, P1180, DOI 10.1093/molbev/msn065
Burri R, 2010, MOL BIOL EVOL, V27, P2360, DOI 10.1093/molbev/msq120
Calisher CH, 2006, CLIN MICROBIOL REV, V19, P531, DOI 10.1128/CMR.00017-06
de Bellocq JG, 2009, IMMUNOGENETICS, V61, P131, DOI 10.1007/s00251-008-0349-4
Dobson AP, 2005, SCIENCE, V310, P628, DOI 10.1126/science.1120872
Doxiadis GGM, 2010, IMMUNOGENETICS, V62, P137, DOI 10.1007/s00251-010-0422-7
Ellis SA, 2006, IMMUNOGENETICS, V57, P953, DOI 10.1007/s00251-005-0071-4
Field HE, 2009, ZOONOSES PUBLIC HLTH, V56, P278, DOI 10.1111/j.1863-2378.2008.01218.x
FIGUEROA F, 1988, NATURE, V335, P265, DOI 10.1038/335265a0
Gannon WL, 2007, J MAMMAL, V88, P809, DOI 10.1644/06-MAMM-F-185R1.1
Hess CM, 2002, BIOSCIENCE, V52, P423, DOI 10.1641/0006-3568(2002)052[0423:TEOTMH]2.0.CO;2
Huelsenbeck JP, 2001, BIOINFORMATICS, V17, P754, DOI 10.1093/bioinformatics/17.8.754
Hughes AL, 2007, HEREDITY, V99, P364, DOI 10.1038/sj.hdy.6801031
HUGHES AL, 1994, P ROY SOC B-BIOL SCI, V256, P119, DOI 10.1098/rspb.1994.0058
HUGHES AL, 1988, NATURE, V335, P167, DOI 10.1038/335167a0
Hughes AL, 1998, ANNU REV GENET, V32, P415, DOI 10.1146/annurev.genet.32.1.415
Hughes AL, 2000, IMMUNOGENETICS, V51, P473, DOI 10.1007/s002510050646
Jukes T.H., 1969, MAMMALIAN PROTEIN ME, P21, DOI DOI 10.1093/BIOINFORMATICS/BTM404
Kennedy LJ, 2002, IMMUNOGENETICS, V54, P348, DOI 10.1007/s00251-002-0465-5
Kikkawa EF, 2009, IMMUNOGENETICS, V61, P341, DOI 10.1007/s00251-009-0363-1
KLEIN J, 1987, HUM IMMUNOL, V19, P155, DOI 10.1016/0198-8859(87)90066-8
Klein J., 1986, NATURAL HIST MAJOR H
Klein J, 2007, ANNU REV GENET, V41, P281, DOI 10.1146/annurev.genet.41.110306.130137
Kriener K, 2000, IMMUNOGENETICS, V51, P169, DOI 10.1007/s002510050028
Kriener K, 2001, HUM IMMUNOL, V62, P1, DOI 10.1016/S0198-8859(00)00233-0
Kumánovics A, 2003, ANNU REV IMMUNOL, V21, P629, DOI 10.1146/annurev.immunol.21.090501.080116
Kunz TH, 2011, ANN NY ACAD SCI, V1223, P1, DOI 10.1111/

j.1749-6632.2011.06004.x
Kupfermann H, 1999, J MOL EVOL, V48, P663, DOI 10.1007/PL00006510
Lewis-Oritt N, 2001, J MAMMAL, V82, P748, DOI 10.1644/1545-1542(2001)082<0748:MEFEOP>2.0.CO;2
Mansai SP, 2010, GENETICS, V184, P517, DOI 10.1534/genetics.109.111161
Mayer F, 2007, HEREDITY, V99, P257, DOI 10.1038/sj.hdy.6800989
MAYER WE, 1988, EMBO J, V7, P2765, DOI 10.1002/j.1460-2075.1988.tb03131.x
Mikko S, 1999, IMMUNOL REV, V167, P169, DOI 10.1111/j.1600-065X.1999.tb01390.x
Musolf K, 2004, IMMUNOGENETICS, V56, P420, DOI 10.1007/s00251-004-0715-9
Nei M, 2005, ANNU REV GENET, V39, P121, DOI 10.1146/annurev.genet.39.073003.112240
NEI M, 1986, MOL BIOL EVOL, V3, P418
Nei M, 2000, MOL EVOLUTION PHYLOG
Nei M, 1992, 11 HIST WORKSH C
Nemeth E, 2009, SEMIN IMMUNOPATHOL, V31, P333, DOI 10.1007/s00281-009-0173-4
Ohta T, 1999, IMMUNOL REV, V167, P319, DOI 10.1111/j.1600-065X.1999.tb01401.x
OHUIGIN C, 1995, IMMUNOL REV, V143, P123, DOI 10.1111/j.1600-065X.1995.tb00673.x
Piertney SB, 2006, HEREDITY, V96, P7, DOI 10.1038/sj.hdy.6800724
Posada D, 2002, MOL BIOL EVOL, V19, P708, DOI 10.1093/oxfordjournals.molbev.a004129
Posada D, 2008, MOL BIOL EVOL, V25, P1253, DOI 10.1093/molbev/msn083
Reusch TBH, 2005, J MOL EVOL, V61, P531, DOI 10.1007/s00239-004-0340-0
Richman AD, 2010, INT J IMMUNOGENET, V37, P401, DOI 10.1111/j.1744-313X.2010.00941.x
Richman AD, 2003, GENET RES, V82, P89, DOI 10.1017/S0016672303006347
Sawyer S.A., 1999, GENECONV: a computer package for the statistical detection of gene conversion. Distributed by the Author. Department of Mathematics
Schad J, 2004, CONSERV GENET, V5, P299, DOI 10.1023/B:COGE.0000031137.50239.d3
Schad J, 2011, HEREDITY, V107, P115, DOI 10.1038/hdy.2010.173
Schaschl H, 2006, HEREDITY, V97, P427, DOI 10.1038/sj.hdy.6800892
Schaschl H, 2005, IMMUNOGENETICS, V57, P108, DOI 10.1007/s00251-005-0784-4
Srithayakumar V, 2012, IMMUNOGENETICS, DOI [10.1007/s00251.011-0588-7, DOI 10.1007/S00251.011-0588-7]
STERN LJ, 1994, NATURE, V368, P215, DOI 10.1038/368215a0
Suárez CF, 2006, IMMUNOGENETICS, V58, P542, DOI 10.1007/s00251-006-0127-0
Tamura K, 2007, MOL BIOL EVOL, V24, P1596, DOI 10.1093/molbev/msm092
TROWSDALE J, 1995, IMMUNOGENETICS, V41, P1, DOI 10.1007/BF00188427
Vilamiu Rossana de Paula, 2010, Mastozool. neotrop., V17, P219

   Virtue ER, 2011, PLOS ONE, V6, DOI 10.1371/journal.pone.0022488
   Wei K, 2010, IMMUNOGENETICS, V62, P307, DOI 10.1007/
s00251-010-0440-5
   Wibbelt G, 2010, BIOL LETTERS, V6, P438, DOI 10.1098/
rsbl.2010.0267
   Wilmer Jessica Worthington, 1996, Bat Research News, V37, P1
   Xu SX, 2009, J MOL EVOL, V69, P260, DOI 10.1007/s00239-009-9272-z
   Xu SX, 2008, MOL PHYLOGENET EVOL, V47, P36, DOI 10.1016/
j.ympev.2007.05.026
   Yang ZH, 2005, MOL BIOL EVOL, V22, P1107, DOI 10.1093/molbev/
msi097
   Yang ZH, 2000, TRENDS ECOL EVOL, V15, P496, DOI 10.1016/
S0169-5347(00)01994-7
   Yang ZH, 2007, MOL BIOL EVOL, V24, P1586, DOI 10.1093/molbev/
msm088
   Yeager M, 1999, IMMUNOL REV, V167, P45, DOI 10.1111/
j.1600-065X.1999.tb01381.x
   Yuhki N, 2007, J HERED, V98, P390, DOI 10.1093/jhered/esm056
NR 78
TC 20
Z9 21
U1 0
U2 38
PU SPRINGER
PI NEW YORK
PA ONE NEW YORK PLAZA, SUITE 4600, NEW YORK, NY, UNITED STATES
SN 0093-7711
EI 1432-1211
J9 IMMUNOGENETICS
JI Immunogenetics
PD JUL
PY 2012
VL 64
IS 7
BP 535
EP 547
DI 10.1007/s00251-012-0609-1
PG 13
WC Genetics & Heredity; Immunology
WE Science Citation Index Expanded (SCI-EXPANDED)
SC Genetics & Heredity; Immunology
GA 957AH
UT WOS:000305130300005
PM 22426641
DA 2024-10-21
ER

PT J
AU Seltmann, A
   Troxell, SA
   Schad, J
   Fritze, M
   Bailey, LD
   Voigt, CC

Czirják, GA
AF Seltmann, Anne
Troxell, Sara A.
Schad, Julia
Fritze, Marcus
Bailey, Liam D.
Voigt, Christian C.
Czirjak, Gabor A.
TI Differences in acute phase response to bacterial, fungal and viral
antigens in greater mouse-eared bats (<i>Myotis myotis</i>)
SO SCIENTIFIC REPORTS
LA English
DT Article
ID PRO-INFLAMMATORY CYTOKINES; IMMUNE-SYSTEM; ENERGY-EXPENDITURE;
BODY-TEMPERATURE; FOOD RESTRICTION; INNATE IMMUNITY; HOUSE SPARROWS;
LIFE-HISTORY; FEVER; LIPOPOLYSACCHARIDE
AB The acute phase response (APR) is an evolutionarily well-conserved part of the innate immune defense against pathogens. However, recent studies in bats yielded surprisingly diverse results compared to previous APR studies on both vertebrate and invertebrate species. This is especially interesting due to the known role of bats as reservoirs for viruses and other intracellular pathogens, while being susceptible to extracellular microorganisms such as some bacteria and fungi. To better understand these discrepancies and the reservoir-competence of bats, we mimicked bacterial, viral and fungal infections in greater mouse-eared bats (Myotis myotis) and quantified different aspects of the APR over a two-day period. Individuals reacted most strongly to a viral (PolyI:C) and a bacterial (LPS) antigen, reflected by an increase of haptoglobin levels (LPS) and an increase of the neutrophil-to-lymphocyte-ratio (PolyI:C and LPS). We did not detect fever, leukocytosis, body mass loss, or a change in the overall functioning of the innate immunity upon challenge with any antigen. We add evidence that bats respond selectively with APR to specific pathogens and that the activation of different parts of the immune system is species-specific.
C1 [Seltmann, Anne; Troxell, Sara A.; Schad, Julia; Fritze, Marcus; Voigt, Christian C.] Leibniz Inst Zoo & Wildlife Res, Dept Evolutionary Ecol, Alfred Kowalke Str 17, D-10315 Berlin, Germany.
[Seltmann, Anne; Troxell, Sara A.; Fritze, Marcus; Voigt, Christian C.] Free Univ Berlin, Inst Biol, Takustr 3, D-14195 Berlin, Germany.
[Bailey, Liam D.] Leibniz Inst Zoo & Wildlife Res, Dept Evolutionary Genet, Alfred Kowalke Str 17, D-10315 Berlin, Germany.
[Czirjak, Gabor A.] Leibniz Inst Zoo & Wildlife Res, Dept Wildlife Dis, Alfred Kowalke Str 17, D-10315 Berlin, Germany.
[Seltmann, Anne] Natl Pk Adm Saxon Switzerland, Dept Publ Relat & Environm Educ 3, Elbe 4, D-01814 Bad Schandau, Germany.
[Fritze, Marcus] Univ Greifswald, Appl Zool & Nat Conservat, Loitzer Str 26, D-17489 Greifswald, Germany.
C3 Leibniz Institut fur Zoo und Wildtierforschung; Free University of
Berlin; Leibniz Institut fur Zoo und Wildtierforschung; Leibniz

Institut
fur Zoo und Wildtierforschung; Universitat Greifswald
RP Czirják, GA (corresponding author), Leibniz Inst Zoo & Wildlife Res, Dept Wildlife Dis, Alfred Kowalke Str 17, D-10315 Berlin, Germany.
EM czirjak@izw-berlin.de
RI Fritze, Marcus/H-3597-2015; Czirják, Gábor/F-5440-2011
OI Czirjak, Gabor Arpad/0000-0001-9488-0069
FU Leibniz Institute for Zoo and Wildlife Research; German Research Foundation (DFG Priority Programme 1596); DFG [GO2091/2-1]; Deutsche Forschungsgemeinschaft (German Research Foundation) [491292795]; Projekt DEAL
FX We are very grateful to the Directorate of the Rusenski Lom Nature Park (Director Tsonka Hristova) for cooperation and support. We thank the responsible Bulgarian authorities (MOEW-Sofia and RIOSV-Ruse) for granting us permission to conduct this research. We would like to thank Stefan Greif for his support in capturing bats in Bulgaria and Katja Pohle for her help in the laboratory analyses. This work was supported by institutional funds of the Leibniz Institute for Zoo and Wildlife Research and the German Research Foundation (DFG Priority Programme 1596). Funding for the research station in Tabachka was provided by the DFG to Holger R. Goerlitz (Emmy Noether Program GO2091/2-1). Open Access funding enabled and organized by Projekt DEAL and was funded by the Deutsche Forschungsgemeinschaft (German Research Foundation-Projektnummer 491292795).
CR Ahn M, 2016, SCI REP-UK, V6, DOI 10.1038/srep21722
Allen LC, 2009, J COMP PHYSIOL B, V179, P315, DOI 10.1007/s00360-008-0315-3
[Anonymous], 2019, CAR AN R COMPANION A
[Anonymous], 2007, R PACKAGE VERSION
Audebert HJ, 2004, EUR NEUROL, V51, P125, DOI 10.1159/000077012
Baker ML, 2013, ZOONOSES PUBLIC HLTH, V60, P104, DOI 10.1111/j.1863-2378.2012.01528.x
Baker ML, 2015, BATS AND VIRUSES, P327
Barclay RMR, 1996, J MAMMAL, V77, P1102, DOI 10.2307/1382791
Barlow A, 2011, VET REC, V169, DOI 10.1136/vr.d7783
Barrientos RM, 2009, BRAIN BEHAV IMMUN, V23, P450, DOI 10.1016/j.bbi.2009.01.016
Becker DJ, 2020, TRENDS ECOL EVOL, V35, P1062, DOI 10.1016/j.tree.2020.08.014
Becker DJ, 2019, INTEGR COMP BIOL, V59, P1176, DOI 10.1093/icb/icz007

Becker DJ, 2018, PHILOS T R SOC B, V373, DOI 10.1098/rstb.2017.0089
Blehert DS, 2009, SCIENCE, V323, P227, DOI 10.1126/science.1163874
Bonneaud C, 2003, AM NAT, V161, P367, DOI 10.1086/346134
Bouma HR, 2010, J LEUKOCYTE BIOL, V88, P619, DOI 10.1189/jlb.0310174
Brinkmann V, 2012, J CELL BIOL, V198, P773, DOI 10.1083/jcb.201203170
Brook CE, 2015, TRENDS MICROBIOL, V23, P172, DOI 10.1016/j.tim.2014.12.004
Cabrera-Martinez LV, 2019, COMP BIOCHEM PHYS A, V229, P93, DOI 10.1016/j.cbpa.2018.12.003
Cabrera-Martínez LV, 2018, PEERJ, V6, DOI 10.7717/peerj.4627
Coon CAC, 2011, AM J PHYSIOL-REG I, V300, pR1418, DOI 10.1152/ajpregu.00187.2010
Copeland S, 2005, CLIN DIAGN LAB IMMUN, V12, P60, DOI 10.1128/CDLI.12.1.60-67.2005
Costantini D, 2022, CONSERV PHYSIOL, V10, DOI 10.1093/conphys/coac028
Crameri G, 2009, PLOS ONE, V4, DOI 10.1371/journal.pone.0008266
Cray C, 2009, COMPARATIVE MED, V59, P517
Davis AK, 2008, FUNCT ECOL, V22, P760, DOI 10.1111/j.1365-2435.2008.01467.x
Evans NJ, 2009, EMERG INFECT DIS, V15, P1331, DOI 10.3201/eid1508.090475
Evans SS, 2015, NAT REV IMMUNOL, V15, P335, DOI 10.1038/nri3843
Field KA, 2015, PLOS PATHOG, V11, DOI 10.1371/journal.ppat.1005168
Frick WF, 2010, SCIENCE, V329, P679, DOI 10.1126/science.1188594
Fritze M, 2021, DEV COMP IMMUNOL, V119, DOI 10.1016/j.dci.2021.104017
Fritze M, 2019, BIOL OPEN, V8, DOI 10.1242/bio.046078
Gomez CR, 2006, SHOCK, V25, P581, DOI 10.1097/01.shk.000029553.39081.ec
Gonzalez V, 2022, ISCIENCE, V25, DOI 10.1016/j.isci.2022.104779
Grimble RF, 1996, CLIN SCI, V91, P121, DOI 10.1042/cs0910121
Gruys E, 2005, J Zhejiang Univ Sci B, V6, P1045, DOI 10.1631/jzus.2005.B1045
Guerrero-Chacón AL, 2018, MAMMAL RES, V63, P397, DOI 10.1007/s13364-018-0375-z
HART BL, 1988, NEUROSCI BIOBEHAV R, V12, P123, DOI 10.1016/S0149-7634(88)80004-6
Hasselquist D, 2007, J ORNITHOL, V148, pS571, DOI 10.1007/s10336-007-0201-x
Hecht AM, 2015, SCI REP-UK, V5, DOI 10.1038/srep16604
Hecht-Höger AM, 2020, MOL ECOL, V29, P1745, DOI 10.1111/mec.15437
Heinrich SK, 2017, SCI REP-UK, V7, DOI 10.1038/srep44837
Heinrich SK, 2016, BIOL OPEN, V5, P550, DOI 10.1242/bio.014902
Hothorn T, 2008, BIOMETRICAL J, V50, P346, DOI 10.1002/bimj.200810425
Kacprzyk J, 2017, ACTA CHIROPTEROL, V19, P219, DOI [10.3161/15081109ACC2017.19.2.001, 10.3161/15081109acc2017.19.2.001]
Kimura Mayumi, 1994, American Journal of Physiology, V267, pR1596

Kosoy M, 2010, EMERG INFECT DIS, V16, P1875, DOI 10.3201/eid1612.100601
KOZAK W, 1994, AM J PHYSIOL, V266, pR125, DOI 10.1152/ajpregu.1994.266.1.R125
Langlois MR, 1996, CLIN CHEM, V42, P1589
Lee KA, 2005, OECOLOGIA, V145, P244, DOI 10.1007/s00442-005-0113-5
Lee KA, 2006, INTEGR COMP BIOL, V46, P1000, DOI 10.1093/icb/icl049
Lilley TM, 2017, P ROY SOC B-BIOL SCI, V284, DOI 10.1098/rspb.2016.2232
Martin LB, 2003, P ROY SOC B-BIOL SCI, V270, P153, DOI 10.1098/rspb.2002.2185
Mayberry HW, 2018, J COMP PHYSIOL B, V188, P333, DOI 10.1007/s00360-017-1119-0
Melhado G, 2020, J EXP ZOOL PART A, V333, P536, DOI 10.1002/jez.2399
MORELL V, 1993, SOUTHERN MED J, V86, P658, DOI 10.1097/00007611-199306000-00014
Moreno KR, 2021, ANN NY ACAD SCI, V1505, P178, DOI 10.1111/nyas.14600
Mühldorfer K, 2013, ZOONOSES PUBLIC HLTH, V60, P93, DOI 10.1111/j.1863-2378.2012.01536.x
Mühldorfer K, 2011, BMC VET RES, V7, DOI 10.1186/1746-6148-7-61
Mühldorfer K, 2010, EMERG INFECT DIS, V16, P578, DOI 10.3201/eid1603.091035
Murphy K, 2012, JANEWAYS IMMUNOBIOLO
Neely BA, 2021, J PROTEOME RES, V20, P2547, DOI 10.1021/acs.jproteome.0c00995
Ohmer MEB, 2021, CONSERV PHYSIOL, V9, DOI 10.1093/conphys/coab074
Otálora-Ardila A, 2017, MAMM BIOL, V82, P41, DOI 10.1016/j.mambio.2016.11.002
Otálora-Ardila A, 2016, PLOS ONE, V11, DOI 10.1371/journal.pone.0164938
Owen-Ashley NT, 2007, J ORNITHOL, V148, pS583, DOI 10.1007/s10336-007-0197-2
Owen-Ashley NT, 2006, HORM BEHAV, V49, P15, DOI 10.1016/j.yhbeh.2005.04.009
Pap PL, 2010, NATURWISSENSCHAFTEN, V97, P891, DOI 10.1007/s00114-010-0706-7
Raats M. M., 1991, Food Quality and Preference, V3, P89, DOI 10.1016/0950-3293(91)90028-D
Schaer J, 2013, P NATL ACAD SCI USA, V110, P17415, DOI 10.1073/pnas.1311016110
Scheiermann C, 2013, NAT REV IMMUNOL, V13, P190, DOI 10.1038/nri3386
Schneeberger K, 2013, J EXP BIOL, V216, P4514, DOI 10.1242/jeb.090837
Schneeberger K, 2013, PLOS ONE, V8, DOI 10.1371/journal.pone.0054023
Schultz EM, 2017, J EXP BIOL, V220, P722, DOI 10.1242/jeb.149898
Sheldon BC, 1996, TRENDS ECOL EVOL, V11, P317, DOI 10.1016/0169-5347(96)10039-2
Simpson VR, 2013, J VET DIAGN INVEST, V25, P551, DOI

10.1177/1040638713493780
Sköld-Chiriac S, 2015, J EXP BIOL, V218, P2961, DOI 10.1242/jeb.122150
Sköld-Chiriac S, 2014, PHYSIOL BIOCHEM ZOOL, V87, P288, DOI 10.1086/674789
Stockmaier S, 2018, ANIM BEHAV, V140, P141, DOI 10.1016/j.anbehav.2018.04.021
Stockmaier S, 2015, BIOL LETTERS, V11, DOI 10.1098/rsbl.2015.0576
Taylor ML, 2005, FEMS IMMUNOL MED MIC, V45, P451, DOI 10.1016/j.femsim.2005.05.019
Triana-Llanos C, 2019, BIOLOGIA, V74, P667, DOI 10.2478/s11756-019-00204-3
Vermeulen A, 2016, BEHAV ECOL SOCIOBIOL, V70, P585, DOI 10.1007/s00265-016-2077-3
Viljoen H, 2011, J ZOOL, V285, P222, DOI 10.1111/j.1469-7998.2011.00833.x
Voigt CC, 2020, SCI REP-UK, V10, DOI 10.1038/s41598-020-74473-3
Wang LF, 2011, CURR OPIN VIROL, V1, P649, DOI 10.1016/j.coviro.2011.10.013
Watkins LR, 1995, PAIN, V63, P289, DOI 10.1016/0304-3959(95)00186-7
Weise P, 2017, PEERJ, V5, DOI 10.7717/peerj.3570
Wibbelt G, 2010, BIOL LETTERS, V6, P438, DOI 10.1098/rsbl.2010.0267
NR 89
TC 8
Z9 8
U1 2
U2 7
PU NATURE PORTFOLIO
PI BERLIN
PA HEIDELBERGER PLATZ 3, BERLIN, 14197, GERMANY
SN 2045-2322
J9 SCI REP-UK
JI Sci Rep
PD SEP 10
PY 2022
VL 12
IS 1
AR 15259
DI 10.1038/s41598-022-18240-6
PG 13
WC Multidisciplinary Sciences
WE Science Citation Index Expanded (SCI-EXPANDED)
SC Science & Technology - Other Topics
GA 4L1WU
UT WOS:000852426200010
PM 36088405
OA Green Published, gold
DA 2024-10-21
ER

PT J
AU Yi, XL

Donner, DM
Marquardt, PE
Palmer, JM
Jusino, MA
Frair, J
Lindner, DL
Latch, EK
AF Yi, Xueling
Donner, Deahn M.
Marquardt, Paula E.
Palmer, Jonathan M.
Jusino, Michelle A.
Frair, Jacqueline
Lindner, Daniel L.
Latch, Emily K.
TI Major histocompatibility complex variation is similar in little brown
bats before and after white-nose syndrome outbreak
SO ECOLOGY AND EVOLUTION
LA English
DT Article
DE fungal disease; immunity; major histocompatibility complex; Myotis
lucifugus; North American bat; Pseudogymnoascus destructans
ID PSEUDOGYMNOASCUS-DESTRUCTANS; GENETIC DIVERSITY; SPATIAL-PATTERNS;
IMMUNE-RESPONSE; MHC; POPULATION; SELECTION; GEOMYCES; DISEASE; SUITE
AB White-nose syndrome (WNS), caused by the fungal pathogenPseudogymnoascus destructans(Pd), has driven alarming declines in North American hibernating bats, such as little brown bat (Myotis lucifugus). During hibernation, infected little brown bats are able to initiate anti-Pd immune responses, indicating pathogen-mediated selection on the major histocompatibility complex (MHC) genes. However, such immune responses may not be protective as they interrupt torpor, elevate energy costs, and potentially lead to higher mortality rates. To assess whether WNS drives selection on MHC genes, we compared the MHCDRBgene in little brown bats pre- (Wisconsin) and post- (Michigan, New York, Vermont, and Pennsylvania) WNS (detection spanning 2014-2015). We genotyped 131 individuals and found 45 nucleotide alleles (27 amino acid alleles) indicating a maximum of 3 loci (1-5 alleles per individual). We observed high allelic admixture and a lack of genetic differentiation both among sampling sites and between pre- and post-WNS populations, indicating no signal of selection on MHC genes. However, post-WNS populations exhibited decreased allelic richness, reflecting effects from bottleneck and drift following rapid population declines. We propose that mechanisms other than adaptive immunity are more likely driving current persistence of little brown bats in affected regions.
C1 [Yi, Xueling; Latch, Emily K.] Univ Wisconsin, Dept Biol Sci, 3209 N Maryland Ave,5181 Lapham Hall, Milwaukee, WI 53211 USA.
[Donner, Deahn M.; Marquardt, Paula E.] US Forest Serv, Northern Res Stn, USDA, Rhinelander, WI USA.

[Palmer, Jonathan M.; Jusino, Michelle A.; Lindner, Daniel L.] US Forest Serv, Northern Res Stn, USDA, Madison, WI USA.
[Jusino, Michelle A.] Univ Florida, Dept Plant Pathol, Gainesville, FL 32611 USA.
[Frair, Jacqueline] SUNY Coll Environm Sci & Forestry, Roosevelt Wild Life Stn, Syracuse, NY 13210 USA.
C3 University of Wisconsin System; University of Wisconsin Milwaukee;
United States Department of Agriculture (USDA); United States Forest
Service; United States Department of Agriculture (USDA); United States
Forest Service; State University System of Florida; University of
Florida; State University of New York (SUNY) System; State University of
New York (SUNY) College of Environmental Science & Forestry
RP Yi, XL (corresponding author), Univ Wisconsin, Dept Biol Sci, 3209 N Maryland Ave,5181 Lapham Hall, Milwaukee, WI 53211 USA.
EM xyi@uwm.edu
RI Marquardt, Paula/HPG-1103-2023; Jusino, Michelle/S-5828-2019
OI Latch, Emily/0000-0002-9892-1056; Marquardt, Paula
E./0000-0001-8854-0172; Jusino, Michelle/0000-0002-3284-4254; Yi,
Xueling/0000-0003-4860-7429
FU United States Department of Agriculture (USDA) Northeastern States
Research Cooperative award [69461]; USDA Forest Service, Northern
Research Station, Rhinelander, Wisconsin
FX This research was supported by the United States Department of
Agriculture (USDA) Northeastern States Research Cooperative award No.
69461, and by the USDA Forest Service, Northern Research Station,
Rhinelander, Wisconsin.
CR Ange-Stark M.A., 2019, White-nose syndrome restructures bat skin microbiomes, P614842, DOI DOI 10.1101/614842
[Anonymous], **DATA OBJECT**
Auteri GG, 2020, SCI REP-UK, V10, DOI 10.1038/s41598-020-59797-4
Bataille A, 2015, P ROY SOC B-BIOL SCI, V282, DOI 10.1098/rspb.2014.3127
Bateson ZW, 2015, MOL ECOL, V24, P6095, DOI 10.1111/mec.13459
Baucom RS, 2011, FUNCT ECOL, V25, P18, DOI 10.1111/j.1365-2435.2010.01742.x
Bernatchez L, 2003, J EVOLUTION BIOL, V16, P363, DOI 10.1046/j.1420-9101.2003.00531.x
Blanco JL, 2008, VET IMMUNOL IMMUNOP, V125, P47, DOI 10.1016/j.vetimm.2008.04.020
Blehert DS, 2009, SCIENCE, V323, P227, DOI 10.1126/science.1163874
Bouma HR, 2010, J LEUKOCYTE BIOL, V88, P619, DOI 10.1189/jlb.0310174
Chao A., 2016, SpadeR (Species-Richness Prediction and Diversity Estimation in R): An R package in CRAN
Cheng TNL, 2019, J ANIM ECOL, V88, P591, DOI 10.1111/1365-2656.12954
Davy CM, 2017, FACETS, V2, P690, DOI 10.1139/facets-2017-0022

Del Real-Monroy M, 2014, ACTA CHIROPTEROL, V16, P301, DOI 10.3161/150811014X687260
Dobony CA, 2011, J FISH WILDL MANAG, V2, P190, DOI 10.3996/022011-JFWM-014
Donaldson ME, 2017, EVOL APPL, V10, P1076, DOI 10.1111/eva.12514
Eimes JA, 2011, J EVOLUTION BIOL, V24, P1847, DOI 10.1111/j.1420-9101.2011.02311.x
Eizaguirre C, 2012, NAT COMMUN, V3, DOI 10.1038/ncomms1632
Ellison AR, 2014, G3-GENES GENOM GENET, V4, P1275, DOI 10.1534/g3.114.010744
Excoffier L, 2010, MOL ECOL RESOUR, V10, P564, DOI 10.1111/j.1755-0998.2010.02847.x
Field KA, 2018, MOL ECOL, V27, P3727, DOI 10.1111/mec.14827
Field KA, 2015, PLOS PATHOG, V11, DOI 10.1371/journal.ppat.1005168
Fisher MC, 2012, NATURE, V484, P186, DOI 10.1038/nature10947
Flory AR, 2012, J APPL ECOL, V49, P680, DOI 10.1111/j.1365-2664.2012.02129.x
Frick WF, 2015, GLOBAL ECOL BIOGEOGR, V24, P741, DOI 10.1111/geb.12290
Frick WF, 2010, SCIENCE, V329, P679, DOI 10.1126/science.1188594
Gannon WL, 2007, J MAMMAL, V88, P809, DOI 10.1644/06-MAMM-F-185R1.1
Gignoux-Wolfsohn S. A., 2018, GENOMIC SIGNATURES E, DOI [10.1101/470294, DOI 10.1101/470294]
Harazim M, 2018, BMC ZOOL, V3, DOI 10.1186/s40850-018-0035-4
Hoyt JR, 2019, SCI REP-UK, V9, DOI 10.1038/s41598-019-45453-z
Huang Y, 2010, BIOINFORMATICS, V26, P680, DOI 10.1093/bioinformatics/btq003
Johnson JS, 2015, ECOL EVOL, V5, P2203, DOI 10.1002/ece3.1502
Jombart T, 2008, BIOINFORMATICS, V24, P1403, DOI 10.1093/bioinformatics/btn129
Jombart T, 2010, BMC GENET, V11, DOI 10.1186/1471-2156-11-94
Jombart T, 2011, BIOINFORMATICS, V27, P3070, DOI 10.1093/bioinformatics/btr521
Jost L, 2018, EVOL APPL, V11, P1139, DOI 10.1111/eva.12590
Kanagawa T, 2003, J BIOSCI BIOENG, V96, P317, DOI 10.1263/jbb.96.317
Kosch TA, 2019, ANIM CONSERV, V22, P238, DOI 10.1111/acv.12459
Kryazhimskiy S, 2008, PLOS GENET, V4, DOI 10.1371/journal.pgen.1000304
Kumar S, 2016, MOL BIOL EVOL, V33, P1870, DOI [10.1093/molbev/msw054, 10.1093/molbev/msv279]
Kyle CJ, 2014, MOL ECOL, V23, P2287, DOI 10.1111/mec.12726
Langwig KE, 2017, PHILOS T R SOC B, V372, DOI 10.1098/rstb.2016.0044
Langwig KE, 2015, P ROY SOC B-BIOL SCI, V282, DOI 10.1098/rspb.2014.2335
Langwig KE, 2012, ECOL LETT, V15, P1050, DOI 10.1111/j.1461-0248.2012.01829.x
Leopardi S, 2015, CURR BIOL, V25, pR217, DOI 10.1016/j.cub.2015.01.047
Lighten J, 2014, MOL ECOL RESOUR, V14, P753, DOI 10.1111/1755-0998.12225

Lilley TM, 2017, P ROY SOC B-BIOL SCI, V284, DOI 10.1098/rspb.2016.2232
Lilley TM, 2020, G3-GENES GENOM GENET, V10, P2007, DOI 10.1534/g3.119.400966
Loiseau C, 2009, MOL ECOL, V18, P1331, DOI 10.1111/j.1365-294X.2009.04105.x
Lorch JM, 2011, NATURE, V480, P376, DOI 10.1038/nature10590
Mainguy J, 2007, CONSERV GENET, V8, P885, DOI 10.1007/s10592-006-9243-5
Mayer F, 2007, HEREDITY, V99, P257, DOI 10.1038/sj.hdy.6800989
Meurling S., 2019, LATITUDINAL MHC VARI, DOI [10.1101/597559, DOI 10.1101/597559]
Minnis AM, 2013, FUNGAL BIOL-UK, V117, P638, DOI 10.1016/j.funbio.2013.07.001
Moore MS, 2013, PLOS ONE, V8, DOI 10.1371/journal.pone.0058976
NEI M, 1986, MOL BIOL EVOL, V3, P418
Palmer JM, 2018, PEERJ, V6, DOI 10.7717/peerj.4925
Quinlan AR, 2010, BIOINFORMATICS, V26, P841, DOI 10.1093/bioinformatics/btq033
*R COR TEAM, 2019, R LANG ENV STAT COMP
Rapin N, 2014, PLOS ONE, V9, DOI 10.1371/journal.pone.0112285
Reeder DM, 2012, PLOS ONE, V7, DOI 10.1371/journal.pone.0038920
Richman AD, 2010, INT J IMMUNOGENET, V37, P401, DOI 10.1111/j.1744-313X.2010.00941.x
Rico Y, 2016, EVOL APPL, V9, P1271, DOI 10.1111/eva.12410
Rico Y, 2015, PLOS ONE, V10, DOI 10.1371/journal.pone.0140170
Rocke TE, 2019, SCI REP-UK, V9, DOI 10.1038/s41598-019-43210-w
Romani Luigina, 2004, Nat Rev Immunol, V4, P1, DOI 10.1038/nri2939
Salmier A, 2016, BMC EVOL BIOL, V16, DOI 10.1186/s12862-016-0802-1
Savage AE, 2016, P ROY SOC B-BIOL SCI, V283, DOI 10.1098/rspb.2015.3115
Savage AE, 2011, P NATL ACAD SCI USA, V108, P16705, DOI 10.1073/pnas.1106893108
Schad J, 2011, HEREDITY, V107, P115, DOI 10.1038/hdy.2010.173
Schad J, 2012, IMMUNOGENETICS, V64, P535, DOI 10.1007/s00251-012-0609-1
Sommer S, 2013, BMC GENOMICS, V14, DOI 10.1186/1471-2164-14-542
Spurgin LG, 2010, P ROY SOC B-BIOL SCI, V277, P979, DOI 10.1098/rspb.2009.2084
Strand TM, 2012, ECOL EVOL, V2, P341, DOI 10.1002/ece3.86
Stuglik MT, 2011, MOL ECOL RESOUR, V11, P739, DOI 10.1111/j.1755-0998.2011.02997.x
Sutton JT, 2015, MOL ECOL, V24, P362, DOI 10.1111/mec.13039
Vander Wal E, 2014, EVOL APPL, V7, P856, DOI 10.1111/eva.12168
Verant Michelle L., 2014, BMC Physiology, V14, P10, DOI 10.1186/s12899-014-0010-4
Verant ML, 2012, PLOS ONE, V7, DOI 10.1371/journal.pone.0046280
Verma A, 2015, CSH PERSPECT MED, V5, DOI 10.1101/cshperspect.a019612
Vonhof MJ, 2015, PLOS ONE, V10, DOI 10.1371/journal.pone.0128713
Warnecke L, 2012, P NATL ACAD SCI USA, V109, P6999, DOI 10.1073/pnas.1200374109

   Wegner KM, 2003, J EVOLUTION BIOL, V16, P224, DOI 10.1046/
j.1420-9101.2003.00519.x
   Wilmer Jessica Worthington, 1996, Bat Research News, V37, P1
   Zerbino DR, 2018, NUCLEIC ACIDS RES, V46, pD754, DOI 10.1093/nar/
gkx1098
   Zhang L, 2015, HEREDITY, V114, P85, DOI 10.1038/hdy.2014.73
   Zukal J, 2016, SCI REP-UK, V6, DOI 10.1038/srep19829
NR 87
TC 3
Z9 6
U1 1
U2 21
PU WILEY
PI HOBOKEN
PA 111 RIVER ST, HOBOKEN 07030-5774, NJ USA
SN 2045-7758
J9 ECOL EVOL
JI Ecol. Evol.
PD SEP
PY 2020
VL 10
IS 18
BP 10031
EP 10043
DI 10.1002/ece3.6662
EA AUG 2020
PG 13
WC Ecology; Evolutionary Biology
WE Science Citation Index Expanded (SCI-EXPANDED)
SC Environmental Sciences & Ecology; Evolutionary Biology
GA NT2YS
UT WOS:000564545500001
PM 33005361
OA gold, Green Published
DA 2024-10-21
ER

PT J
AU Karunarathna, SC
   Haelewaters, D
   Lionakis, MS
   Tibpromma, S
   Jianchu, X
   Hughes, AC
   Mortimer, PE
AF Karunarathna, Samantha C.
   Haelewaters, Danny
   Lionakis, Michail S.
   Tibpromma, Saowaluck
   Jianchu, Xu
   Hughes, Alice C.
   Mortimer, Peter E.
TI Assessing the threat of bat-associated fungal pathogens
SO ONE HEALTH

LA English
DT Article
DE Bat-associated fungi; Bat roosts; Chiroptera; Community ecology;
Emerging infectious diseases; Hibernacula; Landscape management;
Microbiome -pathogen interactions; White-nose syndrome
ID FUSARIUM; RESERVOIRS; SPREAD
AB Fungal pathogens have become an increasingly important topic in recent decades. Yet whilst various cankers and blights have gained attention in temperate woodlands and crops, the scope for fungal pathogens of animals and their potential threat has received far less attention. With a shifting climate, the threat from fungal pathogens is predicted to increase in the future, thus understanding the spread of fungi over landscapes as well as taxa that may be at risk is of particular importance. Cave ecosystems provide potential refugia for various fungi, and roosts for bats. With their well vascularized wings and wide-ranging distributions, bats present potential fungal vectors. Furthermore, whilst bat immune systems are generally robust to bacterial and viral pathogens, they can be susceptible to fungal pathogens, particularly during periods of stress such as hibernation. Here we explore why bats are important and interesting vectors for fungi across landscapes and discuss knowledge gaps that require further research.
C1 [Karunarathna, Samantha C.; Tibpromma, Saowaluck; Jianchu, Xu; Mortimer, Peter E.] Chinese Acad Sci, Kunming Inst Bot, Ctr Mt Futures, Kunming, Peoples R China.
[Haelewaters, Danny] Univ South Bohemia, Fac Sci, Ceske Budejovice 37005, Czech Republic.
[Haelewaters, Danny] Univ Ghent, Dept Biol, Res Grp Mycol, B-9000 Ghent, Belgium.
[Lionakis, Michail S.] Natl Inst Allergy & Infect Dis, Fungal Pathogenesis Sect, Lab Clin Immunol & Microbiol, NIH, Rockville, MD USA.
[Hughes, Alice C.] Univ Hong Kong, Sch Biol Sci, Hong Kong, Peoples R China.
C3 Chinese Academy of Sciences; Kunming Institute of Botany, CAS;
University of South Bohemia Ceske Budejovice; Ghent University; National
Institutes of Health (NIH) - USA; NIH National Institute of Allergy &
Infectious Diseases (NIAID); University of Hong Kong
RP Mortimer, PE (corresponding author), Chinese Acad Sci, Kunming Inst Bot, Ctr Mt Futures, Kunming, Peoples R China.; Haelewaters, D (corresponding author), Univ South Bohemia, Fac Sci, Ceske Budejovice 37005, Czech Republic.; Haelewaters, D (corresponding author), Univ Ghent, Dept Biol, Res Grp Mycol, B-9000 Ghent, Belgium.; Hughes, AC (corresponding author), Univ Hong Kong, Sch Biol Sci, Hong Kong, Peoples R China.
EM danny.haelewaters@gmail.com; ach_conservation2@hotmail.com;
peter@mail.kib.ac.cn
RI Karunarathna, Samantha/JCE-5110-2023; Tibpromma,
Saowaluck/AAY-9436-2021; hughes, Alice/HCH-3838-2022
OI hughes, Alice/0000-0002-4899-3158
FU Chinese Academy of Sciences President's International Fellowship
Initiative (PIFI) [2020FYC0002]; National Science Foundation of

China
[31851110759, Y71L481211]; Research Foundation - Flanders
[1206620N];
Division of Intramural Research of the National Institute of
Allergy and
Infectious Diseases, National Institutes of Health
FX This work was supported in part by the Chinese Academy of
Sciences
President's International Fellowship Initiative (PIFI) young
staff
(2020FYC0002 to S.C.K.) , National Science Foundation of China
(31851110759 to S.C.K., Y71L481211 to P.E.M.) , Research
Foundation -
Flanders (junior postdoctoral fellowship 1206620N to D.H.) , and
the
Division of Intramural Research of the National Institute of
Allergy and
Infectious Diseases, National Institutes of Health.
CR Alves V. C. S., 2022, Fungal Systematics and Evolution, V10, DOI
10.3114/fuse.2022.10.06
BALDAUF SL, 1993, P NATL ACAD SCI USA, V90, P11558, DOI 10.1073/
pnas.90.24.11558
Bausch DG, 2014, PLOS NEGLECT TROP D, V8, DOI 10.1371/
journal.pntd.0003056
Bergman A, 2010, MBIO, V1, DOI 10.1128/mBio.00212-10
Brook CE, 2015, TRENDS MICROBIOL, V23, P172, DOI 10.1016/
j.tim.2014.12.004
Carpouron JE, 2022, J FUNGI, V8, DOI 10.3390/jof8060611
Carvalho JLVR, 2022, BRAZ J MICROBIOL, V53, P2077, DOI 10.1007/
s42770-022-00841-y
Chaverri P, 2013, EVOLUTION, V67, P2823, DOI 10.1111/evo.12169
Colley D.E., 2022, THESIS E WASHINGTON
Crum NF, 2022, INFECT DIS THER, V11, P713, DOI 10.1007/
s40121-022-00606-y
Cunha AOB, 2020, PLOS ONE, V15, DOI 10.1371/journal.pone.0243494
Dimkic I, 2021, APPL MICROBIOL BIOT, V105, P1407, DOI 10.1007/
s00253-021-11143-y
Eby P, 2023, NATURE, V613, P340, DOI 10.1038/s41586-022-05506-2
Fisher MC, 2012, NATURE, V484, P186, DOI 10.1038/nature10947
Garcia-Solache MA, 2010, MBIO, V1, DOI 10.1128/mBio.00061-10
Haelewaters D, 2021, FASCINAT LIFE SCI, P349, DOI
10.1007/978-3-030-54727-1_21
Hawksworth DL, 2017, MICROBIOL SPECTR, V5, DOI 10.1128/
microbiolspec.FUNK-0052-2016
Hoyt JR, 2021, NAT REV MICROBIOL, V19, P196, DOI 10.1038/
s41579-020-00493-5
Huang ZX, 2019, NAT ECOL EVOL, V3, P1110, DOI 10.1038/
s41559-019-0913-3
Ingala MR, 2018, MSPHERE, V3, DOI 10.1128/mSphere.00397-18
Jackson RT, 2022, SCI REP-UK, V12, DOI 10.1038/s41598-022-09692-x
Jacobs A, 2018, ANTON LEEUW INT J G, V111, P1999, DOI 10.1007/
s10482-018-1093-x
Jones DN, 2022, TRENDS MICROBIOL, V30, P632, DOI 10.1016/
j.tim.2021.12.009

Karunarathna SC, 2020, EMERG MICROBES INFEC, V9, P1554, DOI 10.1080/22221751.2020.1785333
Bonilla-Aldana DK, 2021, INT J INFECT DIS, V102, P87, DOI 10.1016/j.ijid.2020.08.050
Kearns P.J., 2022, RESEARCHSQUARE, DOI [10.21203/rs.3.rs-2092895/v1, DOI 10.21203/RS.3.RS-2092895/V1]
Khoa LV, 2004, J FISH DIS, V27, P507, DOI 10.1111/j.1365-2761.2004.00562.x
Kirk P., 2023, CATALOGUE LIFE CHECK, DOI [10.48580/dfrt-4hj, DOI 10.48580/DFRT-4HJ]
Konopka J.B., 2019, One Health: Fungal Pathogens of Humans, Animals, and Plants: Report on an American Academy of Microbiology Colloquium held in Washington, DC, DOI DOI 10.1128/AAMCOL.18OCT.2017
Li AQ, 2022, ENVIRON MICROBIOL, V24, P1484, DOI 10.1111/1462-2920.15754
Li WD, 2005, SCIENCE, V310, P676, DOI 10.1126/science.1118391
Li ZL, 2022, MICROB BIOTECHNOL, V15, P469, DOI 10.1111/1751-7915.13765
Lockhart SR, 2019, SEMIN DIAGN PATHOL, V36, P177, DOI 10.1053/j.semdp.2019.04.010
Low Chian-Yong, 2011, F1000 Med Rep, V3, P14, DOI 10.3410/M3-14
Taylor ML, 2022, APPL ENVIRON MICROB, V88, DOI 10.1128/aem.02010-21
Lutz HL, 2019, MSYSTEMS, V4, DOI 10.1128/mSystems.00511-19
Mulec J., 2013, Open Journal of Veterinary Medicine, V3, P161, DOI 10.4236/ojvm.2013.32025
Nabi G, 2021, SCI TOTAL ENVIRON, V754, DOI 10.1016/j.scitotenv.2020.142372
Nucci M, 2007, CLIN MICROBIOL REV, V20, P695, DOI 10.1128/CMR.00014-07
Pandian G.N., 2022, HELIYON, DOI [10.2139/ssrn.4265433, DOI 10.2139/SSRN.4265433]
Pereira MLS, 2022, MYCOL PROG, V21, P345, DOI 10.1007/s11557-021-01760-2
Perrone G, 2007, STUD MYCOL, P53, DOI 10.3114/sim.2007.59.07
Péter A, 2022, FRONT ECOL EVOL, V10, DOI 10.3389/fevo.2022.795020
Prakash H, 2021, MICROORGANISMS, V9, DOI 10.3390/microorganisms9030523
Ramanantsalama RV, 2022, ACTA TROP, V231, DOI 10.1016/j.actatropica.2022.106462
Sandoval-Denis M, 2018, PERSOONIA, V40, P1, DOI 10.3767/persoonia.2018.40.01
Scheele B, 2019, SCIENCE, V363, P1459, DOI 10.1126/science.aav0379
Skerratt LF, 2007, ECOHEALTH, V4, P125, DOI 10.1007/s10393-007-0093-5
Villanueva-Lozano H, 2021, CLIN MICROBIOL INFEC, V27, P813, DOI 10.1016/j.cmi.2020.12.030
Xu JP, 2022, MLIFE, V1, P223, DOI 10.1002/mlf2.12036
Zhelyazkova V, 2020, INT J SPELEOL, V49, P149, DOI 10.5038/1827-806X.49.2.2326
Zhou H, 2021, CELL, V184, P4380, DOI 10.1016/j.cell.2021.06.008
NR 52
TC 4

Z9 4
U1 7
U2 21
PU ELSEVIER
PI AMSTERDAM
PA RADARWEG 29, 1043 NX AMSTERDAM, NETHERLANDS
EI 2352-7714
J9 ONE HEALTH-AMSTERDAM
JI One Health
PD JUN
PY 2023
VL 16
AR 100553
DI 10.1016/j.onehlt.2023.100553
EA MAY 2023
PG 5
WC Public, Environmental & Occupational Health; Infectious Diseases
WE Science Citation Index Expanded (SCI-EXPANDED)
SC Public, Environmental & Occupational Health; Infectious Diseases
GA O3NI9
UT WOS:001042914700001
PM 37363244
OA gold, Green Published
DA 2024-10-21
ER

PT J
AU Allen, LC
   Turmelle, AS
   Mendonça, MT
   Navara, KJ
   Kunz, TH
   McCracken, GF
AF Allen, Louise C.
   Turmelle, Amy S.
   Mendonca, Mary T.
   Navara, Kristen J.
   Kunz, Thomas H.
   McCracken, Gary F.
TI Roosting ecology and variation in adaptive and innate immune system
   function in the Brazilian free-tailed bat (<i>Tadarida brasiliensis</i>)
SO JOURNAL OF COMPARATIVE PHYSIOLOGY B-BIOCHEMICAL SYSTEMS AND
   ENVIRONMENTAL PHYSIOLOGY
LA English
DT Article
DE Artificial roosts; Bactericidal ability; Bats; Immune response;
   Phytohaemagglutinin
ID LOUIS ENCEPHALITIS-VIRUS; BIG BROWN BATS; HISTOPLASMA-CAPSULATUM; FRUIT
   BATS; TRADE-OFFS; INFECTION; IMMUNOCOMPETENCE; IMMUNOLOGY; EVOLUTION;
   RESERVOIR

AB Bats have recently been implicated as reservoirs of important emerging diseases. However, few studies have examined immune responses in bats, and even fewer have evaluated these responses in an ecological context. We examined aspects of both innate and adaptive immune response in adult female Brazilian free-tailed bats (Tadarida brasiliensis) at four maternity roosts (two natural caves and two human-made bridges) in south-central Texas. Immune measurements included in vitro bactericidal ability of whole blood and in vivo T cell mediated response to mitogenic challenge. Bactericidal activity in T. brasiliensis varied with roosting ecology, but appears to be sensitive to colony-level effects. Blood from females living at one cave had significantly lower bactericidal ability than blood from females at three other sites. T cell mediated response in this species was associated with variation in roost ecology, with females from two caves having greater responses than females from two bridges. T cell mediated response and bactericidal activity were negatively correlated with one another within individuals that were tested for both. Variation in immunological response of T. brasiliensis is important for understanding the influence of the environment on the frequency and distribution of immunologically competent individuals and for understanding disease-host dynamics in this and other colonial species.
C1 [Allen, Louise C.; Kunz, Thomas H.] Boston Univ, Ctr Ecol & Conservat Biol, Boston, MA 02215 USA.
[Turmelle, Amy S.; McCracken, Gary F.] Univ Tennessee, Dept Ecol & Evolutionary Biol, Knoxville, TN 37996 USA.
[Mendonca, Mary T.; Navara, Kristen J.] Auburn Univ, Dept Biol Sci, Auburn, AL 36849 USA.
[Navara, Kristen J.] Univ Georgia, Dept Poultry Sci, Athens, GA 30602 USA.
C3 Boston University; University of Tennessee System; University of Tennessee Knoxville; Auburn University System; Auburn University; University System of Georgia; University of Georgia
RP Allen, LC (corresponding author), Boston Univ, Ctr Ecol & Conservat Biol, Boston, MA 02215 USA.
EM allenlou@bu.edu
OI McCracken, Gary/0000-0002-2493-8103; Gilbert, Amy T./0000-0002-8256-0081
FU NSF/NIH [EID 0426082]
FX We thank Nickolay Hristov, Jonathan Reichard, Sarah Duncan, Cynthia Schmaeman, and Laura Borrelli for countless hours of assistance with fieldwork. We thank Irving Marbach, Dwayne Davis, and Bill Cofer for allowing access to their property. Special thanks to Pat Morton and Meg Goodman (Texas Parks and Wildlife), Mark Bloschock (Texas Department of Transportation), Bain Walker (Hill Country Adventure Tours), and Barbara French (Bat Conservation International) for support in the field, and to

Margaret and J. David Bamberger (Selah-Bamberger Ranch) for hospitality.
Additionally, we thank Christopher Richardson and James Sullivan for
help with statistical analysis. Funding was provided by grant EID
0426082 from NSF/NIH Program for the Ecology of Infectious Disease to G.
F. M. and T. H. K. All experimental procedures comply with the current
laws of the United States of America.
CR ALLEN R, 1970, AM J TROP MED HYG, V19, P851, DOI 10.4269/ajtmh.1970.19.851
[Anonymous], 1998, CAPTIVE CARE MED REF
[Anonymous], USGSBRDITR2003003
Anthony E.L.P., 1988, P47
Betke M, 2008, J MAMMAL, V89, P18, DOI 10.1644/07-MAMM-A-011.1
Blount JD, 2003, OIKOS, V102, P340, DOI 10.1034/j.1600-0706.2003.12413.x
Blue CE, 2004, VIROLOGY, V319, P176, DOI 10.1016/j.virol.2003.11.029
BONFORTE RJ, 1972, J PEDIATR-US, V81, P775, DOI 10.1016/S0022-3476(72)80101-X
Botvinkin AD, 2003, EMERG INFECT DIS, V9, P1623, DOI 10.3201/eid0912.030374
Cain JA, 1998, INFECT IMMUN, V66, P1473, DOI 10.1128/IAI.66.4.1473-1481.1998
Calisher CH, 2006, CLIN MICROBIOL REV, V19, P531, DOI 10.1128/CMR.00017-06
Christe P, 2000, ECOL LETT, V3, P207
Cockrum E.L., 1969, Misc Publ Univ Kans Mus Nat Hist, V51, P303
Constantine D.G., 1970, BIOL BATS, V2, P319
CONSTANTINE DG, 1967, PUBLIC HEALTH REP, V82, P867, DOI 10.2307/4593153
CONSTANTINE DG, 1964, PUBLIC HEALTH REP, V79, P1033, DOI 10.2307/4592318
Davis A, 2005, AM J TROP MED HYG, V73, P467, DOI 10.4269/ajtmh.2005.73.467
DAVIS RB, 1962, ECOL MONOGR, V32, P311, DOI 10.2307/1942378
DEEPE GS, 1994, J LAB CLIN MED, V123, P201
Dimitrov DT, 2007, J THEOR BIOL, V245, P498, DOI 10.1016/j.jtbi.2006.11.001
DIMITROV DT, 2006, ENV MODELING SIMULAT, P107
Dobson AP, 2005, SCIENCE, V310, P628, DOI 10.1126/science.1120872
Dominguez SR, 2007, EMERG INFECT DIS, V13, P1295, DOI 10.3201/eid1309.070491
Forsman AM, 2008, J EVOLUTION BIOL, V21, P873, DOI 10.1111/j.1420-9101.2008.01503.x
Fraser GC, 1996, EMERG INFECT DIS, V2, P327, DOI 10.3201/eid0204.960408
Gloza-Rausch F, 2008, EMERG INFECT DIS, V14, P626, DOI 10.3201/eid1404.071439
Goldsby R.A., 2003, KUBY IMMUNOLOGY, V5th
Halpin K, 2000, J GEN VIROL, V81, P1927, DOI 10.1099/0022-1317-81-8-1927

HERBOLD JR, 1983, AM J VET RES, V44, P1889
Hooper DC, 1998, J VIROL, V72, P3711, DOI 10.1128/JVI.72.5.3711-3719.1998
Horn JW, 2008, INTEGR COMP BIOL, V48, P24, DOI 10.1093/icb/icn051
Johara MY, 2001, EMERG INFECT DIS, V7, P439
Keeley ATH, 2004, J MAMMAL, V85, P113, DOI 10.1644/BME-004
Kennedy MW, 2006, TRENDS ECOL EVOL, V21, P653, DOI 10.1016/j.tree.2006.09.017
Klasing Kirk C., 2004, Acta Zoologica Sinica, V50, P961
KUNZ TH, 1988, ECOLOGICAL BEHAV MET, P283
KUNZ TH, 2003, MONITORING TRENDS BA, P91
Lausen CL, 2006, J MAMMAL, V87, P362, DOI 10.1644/05-MAMM-A-127R1.1
Leroy EM, 2005, NATURE, V438, P575, DOI 10.1038/438575a
Li WD, 2005, SCIENCE, V310, P676, DOI 10.1126/science.1118391
Lochmiller RL, 1996, OIKOS, V76, P594, DOI 10.2307/3546356
Martin LB, 2006, FUNCT ECOL, V20, P630, DOI 10.1111/j.1365-2435.2006.01138.x
Martinez MC, 2006, EDUC POLICY, V20, P299, DOI 10.1177/0895904805285017
Matson KD, 2006, PHYSIOL BIOCHEM ZOOL, V79, P556, DOI 10.1086/501057
McCracken G.F., 2003, MONITORING TRENDS BA, P21
MCMURRAY DN, 1982, AM J TROP MED HYG, V31, P527, DOI 10.4269/ajtmh.1982.31.527
Merchant ME, 2003, COMP BIOCHEM PHYS B, V136, P505, DOI 10.1016/S1096-4959(03)00256-2
Messenger SL, 2003, BAT ECOLOGY, P622
Moller AP, 2001, AM NAT, V158, P136, DOI 10.1086/321308
Moller AP, 1999, Q REV BIOL, V74, P3, DOI 10.1086/392949
Navara KJ, 2005, PHYSIOL BIOCHEM ZOOL, V78, P570, DOI 10.1086/430689
NELSON RJ, 2000, SEASONAL PATTERNS ST
Neubaum DJ, 2006, J MAMMAL, V87, P470, DOI 10.1644/05-MAMM-A-252R1.1
Pilipski JD, 2004, J WILDLIFE DIS, V40, P335, DOI 10.7589/0090-3558-40.2.335
Read AF, 2000, SCIENCE, V290, P1104, DOI 10.1126/science.290.5494.1104
Sgro MP, 2003, WEST N AM NATURALIST, V63, P366
Sheldon BC, 1996, TRENDS ECOL EVOL, V11, P317, DOI 10.1016/0169-5347(96)10039-2
Tella JL, 2002, P ROY SOC B-BIOL SCI, V269, P1059, DOI 10.1098/rspb.2001.1951
Tella JL, 2001, P ROY SOC B-BIOL SCI, V268, P1455, DOI 10.1098/rspb.2001.1688
Tieleman BI, 2005, P ROY SOC B-BIOL SCI, V272, P1715, DOI 10.1098/rspb.2005.3155
TURMELLE AS, 2005, BAT RES NEWS, V46, P83
Wibbelt G, 2007, J GEN VIROL, V88, P2651, DOI 10.1099/vir.0.83045-0
NR 62
TC 64
Z9 80

U1 0
U2 49
PU SPRINGER HEIDELBERG
PI HEIDELBERG
PA TIERGARTENSTRASSE 17, D-69121 HEIDELBERG, GERMANY
SN 0174-1578
EI 1432-136X
J9 J COMP PHYSIOL B
JI J. Comp. Physiol. B-Biochem. Syst. Environ. Physiol.
PD APR
PY 2009
VL 179
IS 3
BP 315
EP 323
DI 10.1007/s00360-008-0315-3
PG 9
WC Physiology; Zoology
WE Science Citation Index Expanded (SCI-EXPANDED)
SC Physiology; Zoology
GA 423GF
UT WOS:000264484100009
PM 19002470
OA Green Published, Bronze
DA 2024-10-21
ER

PT J
AU Herrera, LG
   Ortega-García, S
   Morales-Malacara, JB
   Flores-Martinez, JJ
   López-Ortega, G
   Richman, AD
AF Herrera M, L. Gerardo
   Ortega-Garcia, Stephanie
   Morales-Malacara, Juan B.
   Juan Flores-Martinez, Jose
   Lopez-Ortega, Gerardo
   Richman, Adam D.
TI Geographical and seasonal patterns of spleen mass and acarine load in
   tropical and subtropical leaf-nosed bats
SO ACTA CHIROPTEROLOGICA
LA English
DT Article
DE ectoparasites; immune response; insectivorous bats; subtropics; tropics
ID PARASITE LOAD; SPINTURNIX-MYOTI; HOUSE SPARROWS; POPULATION; CHIROPTERA;
   ABUNDANCE; CAVE; SIZE; MESOSTIGMATA; TEMPERATE
AB We investigated intra-annual variability in acarine parasite load (species richness, prevalence and abundance) and spleen mass in populations of leaf-nosed bats in highly seasonal tropical and

subtropical climates: one population of California leaf-nosed bat (Macrotus californicus) in a subtropical thorn forest, one population of Waterhouse's leaf-nosed bat (M. waterhousii) in a subtropical desert, and one population of Waterhouse's leaf-nosed bat in a tropical deciduous forest. We tested the hypothesis that hosts in highly seasonal tropical and subtropical climates exhibit significant seasonal changes in immune response and parasite load. Prevalence was 100% in most populations examined, except in the subtropical population of M. waterhousii in the rainy season. The tropical population had the highest parasite richness in both seasons and presented species belonging to the four acarine orders examined: Mesostigmata, Ixodidae, Trombidiformes, and Sarcoptiformes. Abundance values of Trombidiformes in M. californicus, and of Ixodida and Sarcoptiformes in the tropical M. waterhousii population were higher in the rainy than in the dry season. Spleen mass was larger in the tropical population in the rainy season and in the subtropical population in the dry season. Spleen mass was not related to abundance of any of the acarine orders (Mesostigmata, Ixodidae, Trombidiformes, and Sarcoptiformes) analyzed. Our findings suggest that bats in highly seasonal tropical and subtropical environments experience significant seasonal changes in parasite burden and in immune response.
C1 [Herrera M, L. Gerardo] Univ Nacl Autonoma Mexico, Inst Biol, Estn Biol Chamela, AP 21, San Patricio 48980, Jalisco, Mexico.
[Ortega-Garcia, Stephanie] Univ Nacl Autonoma Mexico, Inst Biol, Posgrad Ciencias Biol, AP 70-153, Mexico City 04510, DF, Mexico.
[Morales-Malacara, Juan B.] Univ Nacl Autonoma Mexico, Fac Ciencias, UMDI, Campus Juriquilla,Blvd Juriquilla 3001, Queretaro 76230, Queretaro, Mexico.
[Juan Flores-Martinez, Jose] Univ Nacl Autonoma Mexico, Inst Biol, Dept Zool, AP 70-153, Mexico City 04510, DF, Mexico.
[Lopez-Ortega, Gerardo] Univ Nacl Autonoma Mexico, Unidad Iztapalapa, Dept Biol, Div CBS, AP 55-535, Mexico City 09340, DF, Mexico.
[Richman, Adam D.] Montana State Univ, Plant Sci Dept, 119 ABS Bldg, Bozeman, MT 59717 USA.
C3 Universidad Nacional Autonoma de Mexico; Universidad Nacional Autonoma
de Mexico; Universidad Nacional Autonoma de Mexico; Universidad Nacional
Autonoma de Mexico; Universidad Autonoma Metropolitana - Mexico;
Universidad Nacional Autonoma de Mexico; Montana State University
System; Montana State University Bozeman
RP Herrera, LG (corresponding author), Univ Nacl Autonoma Mexico, Inst Biol, Estn Biol Chamela, AP 21, San Patricio 48980, Jalisco, Mexico.
EM gherrera@ib.unam.mx
RI Morales-Malacara, Juan/AAN-2929-2020; Ortega-García,
Stephanie/AAC-8538-2021
OI Herrera Montalvo, Luis Gerardo/0000-0001-9348-5040; Morales-Malacara,
Juan B./0000-0002-9828-5176
FU Direccion General de Asuntos del Personal Academico, Universidad
Nacional Autonoma de Mexico [IN221906, IN219113, IN207005-3]

FX We thank to Marisol E. Vega Orihuela for helping in the fieldwork. We
also thank to Laura Del Castillo Martinez for her helpful assistance in
mounting some of the material on microscopic slides. Financial support
was provided by Direccion General de Asuntos del Personal Academico,
Universidad Nacional Autonoma de Mexico, grants Nos. IN221906 and
IN219113 to JBMM, and IN207005-3 to LGHM.
CR [Anonymous], THESIS
Arneberg P, 1998, P ROY SOC B-BIOL SCI, V265, P1283, DOI 10.1098/rspb.1998.0431
Avila-Flores R, 2004, J MAMMAL, V85, P675, DOI 10.1644/BOS-127
BELL GP, 1986, J COMP PHYSIOL B, V156, P441, DOI 10.1007/BF01101107
Bize P, 2008, AM NAT, V171, P107, DOI 10.1086/523943
Bochkov AV, 2006, FOLIA PARASIT, V53, P302, DOI 10.14411/fp.2006.037
Bordes F, 2011, OIKOS, V120, P1889, DOI 10.1111/j.1600-0706.2011.19314.x
BRADSHAW G. V. R., 1961, THESIS
BRENNAN J. M., 1967, STUD FAUNA CURACAO, V24, P147
BRENNAN JM, 1977, J PARASITOL, V63, P554, DOI 10.2307/3280021
Brown CR, 2002, P ROY SOC B-BIOL SCI, V269, P1367, DOI 10.1098/rspb.2002.2036
Christe P, 2000, ECOL LETT, V3, P207
CONNELL J H, 1971, P298
Corbin E, 2008, MAMMAL REV, V38, P108, DOI 10.1111/j.1365-2907.2007.00112.x
de Bellocq JG, 2007, PARASITOL RES, V100, P321, DOI 10.1007/s00436-006-0273-1
Dick CW, 2006, MICROMAMMALS AND MACROPARASITES: FROM EVOLUTIONARY ECOLOGY TO MANAGEMENT, P179, DOI 10.1007/978-4-431-36025-4_11
Dittmar K, 2011, PLOS ONE, V6, DOI 10.1371/journal.pone.0019438
Dittmar K, 2009, J PARASITOL, V95, P308, DOI 10.1645/GE-1664.1
DUSBABEK F, 1975, Acarologia (Paris), V17, P306
Fain A., 1984, Malayan Nature Journal, V37, P175
FAIN A, 1973, Bulletin de l'Institut Royal des Sciences Naturelles de Belgique Entomologie, V49, P1
Fernández-Llario P, 2004, EUR J WILDLIFE RES, V50, P13, DOI 10.1007/s10344-003-0028-6
GUERRERO R, 1992, STUD NEOTROP FAUNA E, V27, P19, DOI 10.1080/01650529209360864
Hadidi S, 2008, J IMMUNOL, V180, P4156, DOI 10.4049/jimmunol.180.6.4156
Hoffmann A., 1990, TROMBICULIDOS MEXICO
Hornok S, 2012, VET MICROBIOL, V159, P541, DOI 10.1016/j.vetmic.2012.04.003
IUCN, 2001, IUCN red list of threatened species, version 3.1
IZDEBSKA J. N., 2012, ARTHROPODS MED EC IM, P123
JANZEN DH, 1970, AM NAT, V104, P501, DOI 10.1086/282687
JOHN JL, 1994, Q REV BIOL, V69, P327, DOI 10.1086/418649
Kohls G. M., 1965, Annals of the Entomological Society of

America, V58, P331, DOI 10.1093/aesa/58.3.331
KOHLS GM, 1969, ANN ENTOMOL SOC AM, V62, P1035, DOI 10.1093/aesa/62.5.1035
Lalli PN, 2004, J PARASITOL, V90, P711, DOI 10.1645/GE-214R
Leary S., 2013, AVMA guidelines for the euthanasia of animals: 2013 edition, V2013
Lefebvre F, 2004, J FISH BIOL, V64, P435, DOI 10.1111/j.0022-1112.2004.00309.x
Lerdthusnee K, 2002, J MED ENTOMOL, V39, P521, DOI 10.1603/0022-2585-39.3.521
LIDICKER WZ, 1955, P SOC EXP BIOL MED, V89, P640
López-González Celia, 2006, Acta Zool. Mex, V22, P109
Lourenço EC, 2016, INT J ACAROL, V42, P63, DOI 10.1080/01647954.2015.1117525
Martin LB, 2004, ECOLOGY, V85, P2323, DOI 10.1890/03-0365
Martin LB, 2007, IBIS, V149, P419, DOI 10.1111/j.1474-919X.2007.00668.x
Medellin R., 2008, Identificacion de los murcielagos de Mexico, clave de campo
Moller AP, 2002, OIKOS, V99, P95, DOI 10.1034/j.1600-0706.2002.990110.x
Moller AP, 1998, OIKOS, V82, P265, DOI 10.2307/3546966
Morales-Malacara JB, 2002, J MED ENTOMOL, V39, P298, DOI 10.1603/0022-2585-39.2.298
Morand S, 2000, CAN J ZOOL, V78, P1356, DOI 10.1139/cjz-78-8-1356
Moya-Larano Jordi, 2010, Open Ecology Journal, V3, P1, DOI 10.2174/1874213001003010001
Moyer BR, 2002, OIKOS, V97, P223, DOI 10.1034/j.1600-0706.2002.970208.x
MURPHY PG, 1986, ANNU REV ECOL SYST, V17, P67, DOI 10.1146/annurev.es.17.110186.000435
O'Hara RB, 2010, METHODS ECOL EVOL, V1, P118, DOI 10.1111/j.2041-210X.2010.00021.x
OFARRELL MJ, 1973, ECOLOGY, V54, P18, DOI 10.2307/1934371
Pearce RD, 2007, J PARASITOL, V93, P518, DOI 10.1645/GE-973R.1
Postawa T, 2014, FOLIA PARASIT, V61, P581, DOI 10.14411/fp.2014.060
RADOVSKY FRANK J., 1967, UNIV CALIF PUBL ENTOMOL, V46., P1
Reeves WK, 2006, EXP APPL ACAROL, V38, P181, DOI 10.1007/s10493-006-0007-1
Rozsa L, 1997, J AVIAN BIOL, V28, P249, DOI 10.2307/3676976
RUDNICK ALBERT, 1960, UNIV CALIFORNIA PUBL ENT, V17, P157
Rzedowski J., 1988, VEGETACION MEXICO
Salkeld DJ, 2008, ECOGRAPHY, V31, P538, DOI 10.1111/j.0906-7590.2008.05414.x
Santibanez P., 2015, An Overview of Tropical Diseases, DOI [10.5772/61978, DOI 10.5772/61978]
SAS INSTITUTE INC, 2008, JMP VERS 8
Scantlebury M, 2010, J ZOOL, V282, P246, DOI 10.1111/j.1469-7998.2010.00734.x
Schemske DW, 2009, ANNU REV ECOL EVOL S, V40, P245, DOI 10.1146/annurev.ecolsys.39.110707.173430
Schulte-Hostedde AI, 2011, J MAMMAL, V92, P221, DOI 10.1644/10-MAMM-A-020.1

Sebela S, 2011, THEOR APPL CLIMATOL, V105, P371, DOI 10.1007/s00704-011-0397-9
Sheeler-Gordon L. L., 1999, Acta Zoologica Mexicana, P85
Shutler D, 1999, CAN J ZOOL, V77, P351, DOI 10.1139/cjz-77-3-351
Smith KG, 2004, OECOLOGIA, V138, P28, DOI 10.1007/s00442-003-1409-y
SMITHSON PA, 1991, THEOR APPL CLIMATOL, V44, P65, DOI 10.1007/BF00865553
Socolovschi C, 2012, EMERG INFECT DIS, V18, P1966, DOI 10.3201/eid1812.111237
Sparagano OAE, 1999, EXP APPL ACAROL, V23, P929, DOI 10.1023/A:1006313803979
Stoner KE, 2005, BIOTROPICA, V37, P591, DOI 10.1111/j.1744-7429.2005.00076.x
Taylor ML, 2000, FUNGAL GENET BIOL, V30, P207, DOI 10.1006/fgbi.2000.1219
Tuttle M.D., 1977, National Cave Management Symposium Proceedings, P108
Vercammen-Grandjean P. H., 1967, Acarologia, V9, P848
VERCAMMEN-GRANDJEAN P. H., 1965, ACAROLOGIA, V7, P493
WENZEL R L, 1976, Brigham Young University Science Bulletin Biological Series, V20, P1
Wenzel R. L., 1966, P405
WIBBELT G., 2006, BIOL LETTERS, V6, P438
Wiens GD, 2013, PLOS ONE, V8, DOI 10.1371/journal.pone.0075749
Torres-Flores JW, 2012, REV BIOL TROP, V60, P1369
WOLDA H, 1978, J ANIM ECOL, V47, P369, DOI 10.2307/3789
Zhang LB, 2010, J MAMMAL, V91, P136, DOI [10.1644/08-MAMM-A-306R2.1, 10.1644/08-MAMM-A-306R2.1.]
NR 83
TC 7
Z9 7
U1 0
U2 21
PU MUSEUM & INST ZOOLOGY PAS-POLISH ACAD SCIENCES
PI WARSAW
PA WILCZA STREET 64, 00-679 WARSAW, POLAND
SN 1508-1109
EI 1733-5329
J9 ACTA CHIROPTEROL
JI Acta Chiropt.
PD DEC
PY 2016
VL 18
IS 2
BP 517
EP 526
DI 10.3161/15081109ACC2016.18.2.018
PG 10
WC Zoology
WE Science Citation Index Expanded (SCI-EXPANDED)
SC Zoology
GA EE1NY
UT WOS:000389350200018

DA 2024-10-21
ER

PT J
AU Kirejczyk, SGM
   Amman, BR
   Schuh, AJ
   Sealy, TK
   Albariño, CG
   Zhang, J
   Brown, CC
   Towner, JS
AF Kirejczyk, Shannon G. M.
   Amman, Brian R.
   Schuh, Amy J.
   Sealy, Tara K.
   Albarino, Cesar G.
   Zhang, Jian
   Brown, Corrie C.
   Towner, Jonathan S.
TI Histopathologic and Immunohistochemical Evaluation of Induced Lesions,
   Tissue Tropism and Host Responses following Experimental Infection of
   Egyptian Rousette Bats (<i>Rousettus aegyptiacus</i>) with the Zoonotic
   Paramyxovirus, Sosuga Virus
SO VIRUSES-BASEL
LA English
DT Article
DE Sosuga virus; zoonoses; paramyxovirus; Rousettus aegyptiacus; Egyptian
   rousette bat; natural reservoir; histopathology; immunohistochemistry
ID MARBURG VIRUS; DISEASE TOLERANCE; KASOKERO VIRUS; FRUIT BATS; AFRICA
AB Ecological and experimental infection studies have identified Egyptian rousette bats (ERBs; Rousettus aegyptiacus: family Pteropodidae) as a reservoir host for the zoonotic rubula-like paramyxovirus Sosuga virus (SOSV). A serial sacrifice study of colony-bred ERBs inoculated with wild-type, recombinant SOSV identified small intestines and salivary gland as major sites of viral replication. In the current study, archived formalin-fixed paraffin-embedded (FFPE) tissues from the serial sacrifice study were analyzed in depth-histologically and immunohistochemically, for SOSV, mononuclear phagocytes and T cells. Histopathologic lesion scores increased over time and viral antigen persisted in a subset of tissues, indicating ongoing host responses and underscoring the possibility of chronic infection. Despite the presence of SOSV NP antigen and villus ulcerations in the small intestines, there were only mild increases in mononuclear phagocytes and T cells, a host response aligned with disease tolerance. In contrast, there was a statistically significant, robust and targeted mononuclear phagocyte cell responses in the salivary glands at 21 DPI, where viral antigen

was sparse. These findings may have broader implications for chiropteran-paramyxovirus interactions, as bats are hypothesized to be the ancestral hosts of this diverse virus family and for ERB immunology in general, as this species is also the reservoir host for the marburgviruses Marburg virus (MARV) and Ravn virus (RAVV) (family Filoviridae).
C1 [Kirejczyk, Shannon G. M.; Zhang, Jian; Brown, Corrie C.; Towner, Jonathan S.] Univ Georgia, Coll Vet Med, Dept Pathol, Athens, GA 30602 USA.
[Kirejczyk, Shannon G. M.; Amman, Brian R.; Schuh, Amy J.; Sealy, Tara K.; Albarino, Cesar G.; Towner, Jonathan S.] Ctr Dis Control & Prevent, Viral Special Pathogens Branch, Atlanta, GA 30333 USA.
[Kirejczyk, Shannon G. M.] Emory Univ, Emory Natl Primate Res Ctr, Div Pathol, Atlanta, GA 30329 USA.
C3 University System of Georgia; University of Georgia; Centers for Disease
Control & Prevention - USA; Emory University
RP Towner, JS (corresponding author), Univ Georgia, Coll Vet Med, Dept Pathol, Athens, GA 30602 USA.; Towner, JS (corresponding author), Ctr Dis Control & Prevent, Viral Special Pathogens Branch, Atlanta, GA 30333 USA.
EM skirejc@gmail.com; cxx1@cdc.gov; wuc2@cdc.gov; tss3@cdc.gov;
bwu4@cdc.gov; juliazh@uga.edu; corbrown@uga.edu; jit8@cdc.gov
RI Towner, Jonathan/ABC-3780-2020
OI Schuh, Amy/0000-0001-8069-349X; Amman, Brian/0000-0002-4726-2361; Sealy,
Tara/0000-0002-2212-0551; Towner, Jonathan S./0000-0002-6473-3049
FU University of Georgia; Centers for Disease Control and Prevention; DTRA
[HDTRA1033037]
FX This research was funded by the University of Georgia, the Centers for
Disease Control and Prevention, and in part by DTRA grant HDTRA1033037.
CR Albariño CG, 2014, EMERG INFECT DIS, V20, P211, DOI 10.3201/eid2002.131620
Amman BR, 2020, PLOS NEGLECT TROP D, V14, DOI 10.1371/journal.pntd.0008092
Amman BR, 2015, J WILDLIFE DIS, V51, P774, DOI 10.7589/2015-02-044
Amman BR, 2015, J WILDLIFE DIS, V51, P113, DOI 10.7589/2014-08-198
Amman BR, 2014, EMERG INFECT DIS, V20, P1761, DOI 10.3201/eid2010.140696
Amman BR, 2012, PLOS PATHOG, V8, DOI 10.1371/journal.ppat.1002877
[Anonymous], Database of Bat-Associated Viruses
Bain CC, 2018, FRONT IMMUNOL, V9, DOI 10.3389/fimmu.2018.02733
Banerjee A, 2019, VIRUSES-BASEL, V11, DOI 10.3390/v11010041
Bankhead P, 2017, SCI REP-UK, V7, DOI 10.1038/s41598-017-17204-5
Cermák V, 2018, SCI DATA, V5, DOI 10.1038/sdata.2018.198
Constantine DG:., 2009, Bat Rabies and Other Lyssavirus Infections, P68
Cui J, 2019, NAT REV MICROBIOL, V17, P181, DOI 10.1038/s41579-018-0118-9

De Schepper S, 2018, CELL, V175, P400, DOI 10.1016/j.cell.2018.07.048
Desalegn G, 2019, NAT COMMUN, V10, DOI 10.1038/s41467-019-11148-2
Drexler JF, 2012, NAT COMMUN, V3, DOI 10.1038/ncomms1796
Fagre AC, 2019, VIRUSES-BASEL, V11, DOI 10.3390/v11030209
Gibson-Corley KN, 2013, VET PATHOL, V50, P1007, DOI 10.1177/0300985813485099
Guito JC, 2021, CURR BIOL, V31, P257, DOI 10.1016/j.cub.2020.10.015
Jelley-Gibbs DM, 2005, J EXP MED, V202, P697, DOI 10.1084/jem.20050227
Jones MEB, 2019, VIRUSES-BASEL, V11, DOI 10.3390/v11030214
Jones MEB, 2015, VIRUSES-BASEL, V7, P3420, DOI 10.3390/v7072779
KALUNDA M, 1986, AM J TROP MED HYG, V35, P387, DOI 10.4269/ajtmh.1986.35.387
Latinne A, 2020, NAT COMMUN, V11, DOI 10.1038/s41467-020-17687-3
Letko M, 2020, NAT REV MICROBIOL, V18, P461, DOI 10.1038/s41579-020-0394-z
Mahalingam S, 2012, LANCET INFECT DIS, V12, P799, DOI 10.1016/S1473-3099(12)70158-5
Mandl JN, 2018, FRONT IMMUNOL, V9, DOI 10.3389/fimmu.2018.02112
Martins R, 2019, ANNU REV IMMUNOL, V37, P405, DOI 10.1146/annurev-immunol-042718-041739
McCarville JL, 2018, CURR OPIN IMMUNOL, V50, P88, DOI 10.1016/j.coi.2017.12.003
McColl KA, 2002, AUST VET J, V80, P636, DOI 10.1111/j.1751-0813.2002.tb10973.x
Medzhitov R, 2012, SCIENCE, V335, P936, DOI 10.1126/science.1214935
Middleton DJ, 2007, J COMP PATHOL, V136, P266, DOI 10.1016/j.jcpa.2007.03.002
Mortlock M, 2019, VIRUSES-BASEL, V11, DOI 10.3390/v11010037
Mortlock M, 2015, EMERG INFECT DIS, V21, P1840, DOI 10.3201/eid2110.140368
Pavlovich SS, 2018, CELL, V173, P1098, DOI 10.1016/j.cell.2018.03.070
Paweska JT, 2020, EMERG INFECT DIS, V26, P3051, DOI 10.3201/eid2612.202108
Schuh AJ, 2020, EMERG INFECT DIS, V26, P2944, DOI 10.3201/eid2612.202411
Schuh AJ, 2019, J INFECT DIS, V219, P1716, DOI 10.1093/infdis/jiy733
Smith PD, 2011, MUCOSAL IMMUNOL, V4, P31, DOI 10.1038/mi.2010.66
Soares MP, 2014, TRENDS IMMUNOL, V35, P483, DOI 10.1016/j.it.2014.08.001
Sohayati AR, 2011, EPIDEMIOL INFECT, V139, P1570, DOI 10.1017/S0950268811000550
Towner JS, 2007, PLOS ONE, V2, DOI 10.1371/journal.pone.0000764
Towner JS, 2006, J VIROL, V80, P6497, DOI 10.1128/JVI.00069-06
Towner JS, 2009, PLOS PATHOG, V5, DOI 10.1371/journal.ppat.1000536
Young DF, 2019, PLOS PATHOG, V15, DOI 10.1371/journal.ppat.1007561
NR 45

TC 5
Z9 7
U1 0
U2 4
PU MDPI
PI BASEL
PA ST ALBAN-ANLAGE 66, CH-4052 BASEL, SWITZERLAND
EI 1999-4915
J9 VIRUSES-BASEL
JI Viruses-Basel
PD JUN
PY 2022
VL 14
IS 6
AR 1278
DI 10.3390/v14061278
PG 17
WC Virology
WE Science Citation Index Expanded (SCI-EXPANDED)
SC Virology
GA 2K2GK
UT WOS:000816160800001
PM 35746749
OA gold, Green Published
DA 2024-10-21
ER

PT J
AU Baker, ML
   Tachedjian, M
   Wang, LF
AF Baker, Michelle L.
   Tachedjian, Mary
   Wang, Lin-Fa
TI Immunoglobulin heavy chain diversity in Pteropid bats: evidence for a
   diverse and highly specific antigen binding repertoire
SO IMMUNOGENETICS
LA English
DT Article
DE Immunoglobulin; Evolution; Bats
ID VARIABLE-REGION; V-H; IMMUNE-RESPONSE; FRUIT BATS; ANTIBODY REPERTOIRE;
   GENE FAMILIES; DEATH PROCESS; EVOLUTIONARY; ORGANIZATION; CHIROPTERA
AB Bats are the natural host reservoir for range of emerging and re-emerging viruses, many of which cause significant morbidity and mortality in other mammals, yet appear to result in no clinical consequences for bats. The ability of bats to coexist with a variety of viruses presents an interesting immunological problem that has not been examined in any detail but which could provide significant insights into the evolution of antiviral mechanisms in mammals. Towards a better understanding of the bat immune system, we analysed the expressed heavy chain variable (VH) regions of antibodies from

the black flying fox, Pteropus alecto. The germline repertoire of the closely related Pteropid bat, Pteropus vampyrus, whose genome has been sequenced was also examined for comparative purposes. Representative VH genes were found in all three mammalian VH clans (I, II and III) in both the expressed P. alecto VH repertoire and the germline P. vampyrus VH repertoire. Evidence for the use of multiple heavy chain diversity (DH) and joining (JH) segments for the generation of diverse VDJ rearrangements was also present in the expressed antibody repertoire of P. alecto. The long period of co-evolutionary history of bats with viruses may have resulted in a variety of highly specific VH segments being hardwired into the genomes of bats and may have implications for their ability to successfully cope with a diversity of viral antigens.
C1 [Baker, Michelle L.; Tachedjian, Mary; Wang, Lin-Fa] CSIRO Livestock Ind, Australian Anim Hlth Lab, Geelong, Vic 3220, Australia.
[Baker, Michelle L.] Univ New Mexico, Dept Biol, Ctr Evolutionary & Theoret Immunol, Albuquerque, NM 87131 USA.
[Wang, Lin-Fa] Australian Biosecur Cooperat Res Ctr Emerging Inf, Geelong, Vic 3220, Australia.
C3 Commonwealth Scientific & Industrial Research Organisation (CSIRO);
University of New Mexico; Commonwealth Scientific & Industrial Research
Organisation (CSIRO)
RP Baker, ML (corresponding author), CSIRO Livestock Ind, Australian Anim Hlth Lab, PO Bag 24, Geelong, Vic 3220, Australia.
EM Michelle.Baker@csiro.au
RI lu, yuan/JZD-0832-2024; Baker, Michelle/C-9694-2013
OI Wang, Lin-Fa/0000-0003-2752-0535; Baker, Michelle/0000-0002-7993-9971
FU CSIRO
FX The authors wish to acknowledge support from a CSIRO CEO Science Leaders
award to L-F. W. We thank Craig Smith, Hume Field, Gary Crameri and
Deborah Middleton for provision of bat spleen material for this study.
CR ALTSCHUL SF, 1990, J MOL BIOL, V215, P403, DOI 10.1016/S0022-2836(05)80360-2
Baker ML, 2005, J IMMUNOL, V174, P5665, DOI 10.4049/jimmunol.174.9.5665
Berens SJ, 1997, INT IMMUNOL, V9, P189, DOI 10.1093/intimm/9.1.189
Birtalan S, 2008, J MOL BIOL, V377, P1518, DOI 10.1016/j.jmb.2008.01.093
Bossart KN, 2008, VIROLOGY, V372, P357, DOI 10.1016/j.virol.2007.11.011
BRODEUR PH, 1984, EUR J IMMUNOL, V14, P922, DOI 10.1002/eji.1830141012
Butler JE, 2006, DEV COMP IMMUNOL, V30, P1, DOI 10.1016/j.dci.2005.06.005
Butler JE, 1997, SCAND J IMMUNOL, V45, P455, DOI 10.1046/j.1365-3083.1997.d01-423.x

Calisher CH, 2006, CLIN MICROBIOL REV, V19, P531, DOI 10.1128/CMR.00017-06
CHAKRABORTY AK, 1984, DEV COMP IMMUNOL, V8, P415, DOI 10.1016/0145-305X(84)90048-X
Das S, 2008, IMMUNOGENETICS, V60, P47, DOI 10.1007/s00251-007-0270-2
Field H, 1999, EMERG INFECT DIS, V5, P438, DOI 10.3201/eid0503.990316
FRIEDMAN ML, 1994, J IMMUNOL, V152, P632
Golub R, 1997, IMMUNOGENETICS, V46, P402, DOI 10.1007/s002510050294
HAIRE RN, 1990, J EXP MED, V171, P1721, DOI 10.1084/jem.171.5.1721
HATTEN BA, 1968, J IMMUNOL, V101, P141
Hubbard TJP, 2009, NUCLEIC ACIDS RES, V37, pD690, DOI 10.1093/nar/gkn828
IVANOV II, 2002, ANTIBODIES, V7, P4
Johansson J, 2002, J IMMUNOL, V168, P5155, DOI 10.4049/jimmunol.168.10.5155
Jones KE, 2002, BIOL REV, V77, P223, DOI 10.1017/S1464793101005899
Kabat EA, 1991, SEQUENCES PROTEINS I
KIRKHAM PM, 1992, EMBO J, V11, P603, DOI 10.1002/j.1460-2075.1992.tb05092.x
KNIGHT KL, 1992, ANNU REV IMMUNOL, V10, P593, DOI 10.1146/annurev.iy.10.040192.003113
Kumar S, 2004, BRIEF BIOINFORM, V5, P150, DOI 10.1093/bib/5.2.150
LANGMAN RE, 1987, MOL IMMUNOL, V24, P675, DOI 10.1016/0161-5890(87)90050-2
Leroy EM, 2005, NATURE, V438, P575, DOI 10.1038/438575a
Leroy EM, 2009, VECTOR-BORNE ZOONOT, V9, P723, DOI 10.1089/vbz.2008.0167
Mainville CA, 1996, J IMMUNOL, V156, P1038
MANSIKKA A, 1991, SCAND J IMMUNOL, V33, P543, DOI 10.1111/j.1365-3083.1991.tb02524.x
McColl KA, 2002, AUST VET J, V80, P636, DOI 10.1111/j.1751-0813.2002.tb10973.x
MCCORMACK WT, 1991, ANNU REV IMMUNOL, V9, P219
MIAN IS, 1991, J MOL BIOL, V217, P133, DOI 10.1016/0022-2836(91)90617-F
Nei M, 1997, P NATL ACAD SCI USA, V94, P7799, DOI 10.1073/pnas.94.15.7799
Nowak RM, 1994, WALKERS BATS WORLD
OTA T, 1994, MOL BIOL EVOL, V11, P469
PADLAN EA, 1994, MOL IMMUNOL, V31, P169, DOI 10.1016/0161-5890(94)90001-9
RADIC MZ, 1993, J IMMUNOL, V150, P4966
RAJEWSKY K, 1987, SCIENCE, V238, P1088, DOI 10.1126/science.3317826
RAVETCH JV, 1981, CELL, V27, P583, DOI 10.1016/0092-8674(81)90400-1
REYNAUD CA, 1989, CELL, V59, P171, DOI 10.1016/0092-8674(89)90879-9
ROOST HP, 1995, P NATL ACAD SCI USA, V92, P1257, DOI 10.1073/

pnas.92.5.1257
SAITOU N, 1987, MOL BIOL EVOL, V4, P406, DOI 10.1093/oxfordjournals.molbev.a040454
Schelonka RL, 2007, EUR J IMMUNOL, V37, P1010, DOI 10.1002/eji.200636569
Simmons NB, 2005, MAMMAL SPECIES WORLD, V1, P312, DOI [DOI 10.1111/J.1365-2656.2010.01742.X, 10.1093/acprof]
Sitnikova T, 1998, MOL BIOL EVOL, V15, P617, DOI 10.1093/oxfordjournals.molbev.a025965
Stenvik J, 2000, IMMUNOGENETICS, V51, P670, DOI 10.1007/s002510000188
SULKIN SE, 1966, AM J TROP MED HYG, V15, P418, DOI 10.4269/ajtmh.1966.15.418
Swanepoel R, 1996, EMERG INFECT DIS, V2, P321, DOI 10.3201/eid0204.960407
Teeling EC, 2005, SCIENCE, V307, P580, DOI 10.1126/science.1105113
THOMPSON JD, 1994, NUCLEIC ACIDS RES, V22, P4673, DOI 10.1093/nar/22.22.4673
Tomlinson I.M., 1996, VBASE SEQUENCE DIREC
TUTTER A, 1989, P NATL ACAD SCI USA, V86, P7460, DOI 10.1073/pnas.86.19.7460
VANDIJK KW, 1993, EUR J IMMUNOL, V23, P832, DOI 10.1002/eji.1830230410
Wellehan JFX, 2009, COMP IMMUNOL MICROB, V32, P379, DOI 10.1016/j.cimid.2007.11.002
Williamson MM, 1998, AUST VET J, V76, P813, DOI 10.1111/j.1751-0813.1998.tb12335.x
Williamson MM, 2000, J COMP PATHOL, V122, P201, DOI 10.1053/jcpa.1999.0364
WILSON IA, 1993, CURR OPIN STRUC BIOL, V3, P113, DOI 10.1016/0959-440X(93)90210-C
Wong S, 2007, REV MED VIROL, V17, P67, DOI 10.1002/rmv.520
Xu JL, 2000, IMMUNITY, V13, P37, DOI 10.1016/S1074-7613(00)00006-6
Zemlin M, 2003, J MOL BIOL, V334, P733, DOI 10.1016/j.jmb.2003.10.007
NR 60
TC 52
Z9 59
U1 0
U2 15
PU SPRINGER
PI NEW YORK
PA ONE NEW YORK PLAZA, SUITE 4600, NEW YORK, NY, UNITED STATES
SN 0093-7711
EI 1432-1211
J9 IMMUNOGENETICS
JI Immunogenetics
PD MAR
PY 2010
VL 62
IS 3
BP 173

EP 184
DI 10.1007/s00251-010-0425-4
PG 12
WC Genetics & Heredity; Immunology
WE Science Citation Index Expanded (SCI-EXPANDED)
SC Genetics & Heredity; Immunology
GA 560LI
UT WOS:000274903800005
PM 20162414
OA Bronze, Green Accepted, Green Published
DA 2024-10-21
ER

PT J
AU Gómez, JMM
   Periasamy, P
   Dutertre, CA
   Irving, AT
   Ng, JHJ
   Crameri, G
   Baker, ML
   Ginhoux, F
   Wang, LF
   Alonso, S
AF Gomez, Julia Maria Martinez
   Periasamy, Pravin
   Dutertre, Charles-Antoine
   Irving, Aaron Trent
   Ng, Justin Han Jia
   Crameri, Gary
   Baker, Michelle L.
   Ginhoux, Florent
   Wang, Lin-Fa
   Alonso, Sylvie
TI Phenotypic and functional characterization of the major lymphocyte
   populations in the fruit-eating bat <i>Pteropus alecto</i>
SO SCIENTIFIC REPORTS
LA English
DT Article
ID T-CELL DIFFERENTIATION; NK CELLS; EFFECTOR; BET; EXPRESSION; INFECTION;
   VIRUS; EOMES; CD4; PROLIFERATION
AB The unique ability of bats to act as reservoir for viruses that are highly pathogenic to humans suggests unique properties and functional characteristics of their immune system. However, the lack of bat specific reagents, in particular antibodies, has limited our knowledge of bat's immunity. Using cross-reactive antibodies, we report the phenotypic and functional characterization of T cell subsets, B and NK cells in the fruit-eating bat Pteropus alecto. Our findings indicate the predominance of CD8(+) T cells in the spleen from wild-caught bats that may reflect either the presence of viruses in this organ or predominance of this cell subset at steady state. Instead majority of T cells in circulation, lymph nodes and

bone marrow (BM) were CD4(+) subsets. Interestingly, 40% of spleen T cells expressed constitutively IL-17, IL-22 and TGF-beta mRNA, which may indicate a strong bias towards the Th17 and regulatory T cell subsets. Furthermore, the unexpected high number of T cells in bats BM could suggest an important role in T cell development. Finally, mitogenic stimulation induced proliferation and production of effector molecules by bats immune cells. This work contributes to a better understanding of bat's immunity, opening up new perspectives of therapeutic interventions for humans.
C1 [Gomez, Julia Maria Martinez; Periasamy, Pravin; Alonso, Sylvie] Natl Univ Singapore, Yong Loo Lin Sch Med, Dept Microbiol & Immunol, Singapore, Singapore.
[Gomez, Julia Maria Martinez; Periasamy, Pravin; Alonso, Sylvie] Natl Univ Singapore, Yong Loo Lin Sch Med, Immunol Programme, Inst Life Sci, Singapore, Singapore.
[Dutertre, Charles-Antoine; Irving, Aaron Trent; Ng, Justin Han Jia; Wang, Lin-Fa] Duke NUS Med Sch, Programme Emerging Infect Dis, Singapore, Singapore.
[Ginhoux, Florent] ASTAR, Singapore Immunol Network SIgN, Singapore, Singapore.
[Crameri, Gary; Baker, Michelle L.] CSIRO, Hlth & Biosecur Business Unit, Australian Anim Hlth Lab, Geelong, Vic, Australia.
C3 National University of Singapore; National University of Singapore;
National University of Singapore; Agency for Science Technology & Research (A*STAR); A*STAR - Singapore Immunology Network (SIgN);
Commonwealth Scientific & Industrial Research Organisation (CSIRO)
RP Alonso, S (corresponding author), Natl Univ Singapore, Yong Loo Lin Sch Med, Dept Microbiol & Immunol, Singapore, Singapore.; Alonso, S (corresponding author), Natl Univ Singapore, Yong Loo Lin Sch Med, Immunol Programme, Inst Life Sci, Singapore, Singapore.
EM micas@nus.edu.sg
RI Baker, Michelle/AAX-9171-2020; Irving, Aaron/AAE-8874-2021; Ginhoux,
Florent/AFN-0385-2022; Dutertre, Charles Antoine/JRX-2224-2023; Irving,
Aaron/I-6754-2013; Baker, Michelle/C-9694-2013
OI Irving, Aaron/0000-0002-0196-1570; Baker, Michelle/0000-0002-7993-9971;
Wang, Lin-Fa/0000-0003-2752-0535; Dutertre,
Charles-Antoine/0000-0001-7950-3652
CR [Anonymous], PRINCIPLES IMMUNOPHA
[Anonymous], ECOHEALTH
BOFILL M, 1992, CLIN EXP IMMUNOL, V88, P243, DOI 10.1111/j.1365-2249.1992.tb03068.x
Bousso P, 2008, NAT REV IMMUNOL, V8, P675, DOI 10.1038/nri2379
Boyman O, 2012, NAT REV IMMUNOL, V12, P180, DOI 10.1038/nri3156
CHAKRAVARTY AK, 1987, DEV COMP IMMUNOL, V11, P649, DOI 10.1016/0145-305X(87)90053-X
CHAKRAVARTY AK, 1994, LYMPHOLOGY, V27, P97
Cho KA, 2012, INT IMMUNOL, V24, P147, DOI 10.1093/intimm/dxr110
Cruz-Guilloty F, 2009, J EXP MED, V206, P51, DOI 10.1084/jem.20081242

De Silva NS, 2015, NAT REV IMMUNOL, V15, P137, DOI 10.1038/nri3804
Gordon SM, 2012, IMMUNITY, V36, P55, DOI 10.1016/j.immuni.2011.11.016
Halpin K, 2000, J GEN VIROL, V81, P1927, DOI 10.1099/0022-1317-81-8-1927
Hamilton SE, 2007, IMMUNITY, V27, P180, DOI 10.1016/j.immuni.2007.08.003
Hernández-Hoyos G, 2003, IMMUNITY, V19, P83, DOI 10.1016/S1074-7613(03)00176-6
Holling TM, 2004, HUM IMMUNOL, V65, P282, DOI 10.1016/j.humimm.2004.01.005
Intlekofer AM, 2005, NAT IMMUNOL, V6, P1236, DOI 10.1038/ni1268
Jamieson AM, 2004, J IMMUNOL, V172, P864, DOI 10.4049/jimmunol.172.2.864
Janardhana V, 2012, DEV COMP IMMUNOL, V36, P610, DOI 10.1016/j.dci.2011.11.001
Johnson C. K., 2015, SCI REP-UK, P1
Kaech SM, 2012, NAT REV IMMUNOL, V12, P749, DOI 10.1038/nri3307
Kryczek I, 2009, BLOOD, V114, P357, DOI 10.1182/blood-2008-09-177360
Lee AK, 2015, BMC GENOMICS, V16, DOI 10.1186/s12864-015-2124-x
Leong JW, 2011, BLOOD, V117, P2297, DOI 10.1182/blood-2011-01-327247
Moore PR, 2010, ANN TROP MED PARASIT, V104, P613, DOI 10.1179/136485910X12851868779948
Muller GC, 2015, CELL IMMUNOL, V296, P149, DOI 10.1016/j.cellimm.2015.05.006
Munster VJ, 2016, SCI REP-UK, V6, DOI 10.1038/srep21878
Nutt SL, 2015, NAT REV IMMUNOL, V15, P160, DOI 10.1038/nri3795
Omatsu T, 2006, VET IMMUNOL IMMUNOP, V111, P309, DOI 10.1016/j.vetimm.2005.12.002
Papenfuss AT, 2012, BMC GENOMICS, V13, DOI 10.1186/1471-2164-13-261
Parkin J, 2001, LANCET, V357, P1777, DOI 10.1016/S0140-6736(00)04904-7
PAUL BN, 1986, DEV COMP IMMUNOL, V10, P55, DOI 10.1016/0145-305X(86)90044-3
Philbey AW, 2008, AUST VET J, V86, P449, DOI 10.1111/j.1751-0813.2008.00361.x
Popescu I, 2014, J IMMUNOL, V193, P5709, DOI 10.4049/jimmunol.1401436
Porichis F, 2014, NAT COMMUN, V5, DOI 10.1038/ncomms6641
SARKAR SK, 1991, DEV COMP IMMUNOL, V15, P423, DOI 10.1016/0145-305X(91)90034-V
Sathaliyawala T, 2013, IMMUNITY, V38, P187, DOI 10.1016/j.immuni.2012.09.020
Shaw TI, 2012, PLOS ONE, V7, DOI 10.1371/journal.pone.0048472
Smith CS, 2016, ECOHEALTH, V13, P72, DOI 10.1007/s10393-016-1116-x
Smith I, 2013, CURR OPIN VIROL, V3, P84, DOI 10.1016/j.coviro.2012.11.006
SWAIN SL, 1991, IMMUNOL REV, V123, P115, DOI 10.1111/j.1600-065X.1991.tb00608.x

Tayade C, 2005, J LEUKOCYTE BIOL, V78, P1347, DOI 10.1189/jlb.0305142
van Lier RAW, 2003, NAT REV IMMUNOL, V3, P931, DOI 10.1038/nri1228
Vidgen ME, 2015, J GEN VIROL, V96, P1619, DOI 10.1099/vir.0.000099
Voskoboinik I, 2015, NAT REV IMMUNOL, V15, P388, DOI 10.1038/nri3839
Weaver CT, 2006, IMMUNITY, V24, P677, DOI 10.1016/j.immuni.2006.06.002
Yokoyama WM, 2004, ANNU REV IMMUNOL, V22, P405, DOI 10.1146/annurev.immunol.22.012703.104711
Zhang GJ, 2013, SCIENCE, V339, P456, DOI 10.1126/science.1230835
Zhang N, 2011, IMMUNITY, V35, P161, DOI 10.1016/j.immuni.2011.07.010
Zhao ED, 2012, CELL MOL IMMUNOL, V9, P11, DOI 10.1038/cmi.2011.47
Zhou P, 2016, P NATL ACAD SCI USA, V113, P2696, DOI 10.1073/pnas.1518240113
Zhou P, 2014, PLOS ONE, V9, DOI 10.1371/journal.pone.0103875
Zhou P, 2011, J IMMUNOL, V186, P3138, DOI 10.4049/jimmunol.1003115
Zhu JF, 2008, BLOOD, V112, P1557, DOI 10.1182/blood-2008-05-078154
NR 53
TC 36
Z9 39
U1 0
U2 22
PU NATURE PORTFOLIO
PI BERLIN
PA HEIDELBERGER PLATZ 3, BERLIN, 14197, GERMANY
SN 2045-2322
J9 SCI REP-UK
JI Sci Rep
PD NOV 24
PY 2016
VL 6
AR 37796
DI 10.1038/srep37796
PG 13
WC Multidisciplinary Sciences
WE Science Citation Index Expanded (SCI-EXPANDED)
SC Science & Technology - Other Topics
GA EH9CF
UT WOS:000392068600001
PM 27883085
OA Green Published, gold
DA 2024-10-21
ER

PT J
AU Holz, PH
Lumsden, LF
Hufschmid, J

AF Holz, Peter H.
   Lumsden, Linda F.
   Hufschmid, Jasmin
TI Ectoparasites are unlikely to be a primary cause of population declines
   of bent-winged bats in south-eastern Australia
SO INTERNATIONAL JOURNAL FOR PARASITOLOGY-PARASITES AND WILDLIFE
LA English
DT Article
DE Bat fly; Miniopterus orianae bassanii; Miniopterus orianae oceanensis;
   Mite; Tick; Nematode; Riouxgolvania beveridgei
ID MITE SPINTURNIX-MYOTI; MINIOPTERUS-SCHREIBERSII; SARCOPTIC MANGE;
   GROOMING BEHAVIOR; HOST; MUSPICEIDAE; CHIROPTERA; NEMATODA; PARASITE;
   STREBLIDAE
AB While bats carry a diverse range of ectoparasites, little research has been conducted on the effects these organisms may have on bat populations. The southern bent-winged bat (Miniopterus orianae bassanii) is a critically endangered subspecies endemic to south-eastern Australia, whose numbers have declined over the past 50 years for unknown reasons. As part of a larger study to investigate the potential role of disease in these declines, southern bent-winged bats from four locations were captured and examined for the presence of bat flies, mites, ticks and the nematode Riouxgolvania beveridgei (previously associated with skin nodules in bent-winged bats). Results were compared with those obtained from the more common eastern bent-winged bat (Miniopterus orianae oceanensis), sampling animals from three different locations. All four types of parasite were found on both subspecies. There was no correlation between the presence of ectoparasites, body weight or any signs of disease. However, prevalence of tick and R. beveridgei infections were greater in Victorian southern bent-winged bats than South Australian southern bent-winged bats and eastern bent-winged bats, possibly indicative of some type of chronic stress impacting the immune system of this subspecies.
C1 [Holz, Peter H.; Hufschmid, Jasmin] Univ Melbourne, Dept Vet Biosci, Melbourne Vet Sch, Fac Vet & Agr Sci, 250 Princes Highway, Werribee, Vic 3030, Australia.
   [Lumsden, Linda F.] Arthur Rylah Inst Environm Res, Dept Environm Land Water & Planning, 123 Brown St, Heidelberg, Vic 3084, Australia.
C3 University of Melbourne; Arthur Rylah Institute for Environmental
   Research (ARI)
RP Holz, PH (corresponding author), Univ Melbourne, Dept Vet Biosci, Melbourne Vet Sch, Fac Vet & Agr Sci, 250 Princes Highway, Werribee, Vic 3030, Australia.
EM holzp@student.unimelb.edu.au
RI Hufschmid, Jasmin/Y-9079-2019
OI Lumsden, Lindy/0000-0002-4967-4626; Holz, Peter/0000-0002-5602-1973;
   Hufschmid, Jasmin/0000-0001-9427-7702
FU Holsworth Wildlife Research Endowment; Wildlife Disease Association

Australasia; Australian Government's Threatened Species Discretionary
Grants Program; Department of Environment, Land, Water and Planning
Victoria, Karst Conservation Fund; David Middleton; Australian
Postgraduate Award scholarship
FX The authors would like to thank the Holsworth Wildlife Research
Endowment, Wildlife Disease Association Australasia, Australian
Government's Threatened Species Discretionary Grants Program, Department
of Environment, Land, Water and Planning Victoria, Karst Conservation
Fund and David Middleton for providing generous financial support for
this project. The lead author is supported by an Australian Postgraduate
Award scholarship. The authors acknowledge the valuable assistance
provided by Melanie Archer (Victorian Institute of Forensic Medicine)
for bat fly species confirmation, Bruce Halliday (CSIRO Canberra) for
mite species confirmation and Stephen Barker (University of Queensland)
for tick identification. The assistance provided by Ian Beveridge,
Amanda Bush, David McLelland and the numerous volunteers involved with
the bat trapping and sampling trips is also gratefully acknowledged.
CR Abundes-Gallegos J, 2018, VECTOR-BORNE ZOONOT, V18, P70, DOI 10.1089/vbz.2017.2163
Anderson R. C, 2000, SUPERFAMILY MUSPICEO, P622
[Anonymous], 1970, AUSTR TICKS
ARTHUR DR, 1956, J PARASITOL, V42, P180, DOI 10.2307/3274734
BAIN O, 1979, ANN PARASIT HUM COMP, V54, P207, DOI 10.1051/parasite/1979542207
Barrett JL, 2002, AUST VET J, V80, P554, DOI 10.1111/j.1751-0813.2002.tb11039.x
Blehert DS, 2012, PLOS PATHOG, V8, DOI 10.1371/journal.ppat.1002779
Brunet-Rossinni Anja K., 2009, P315
Cardinal BR, 2000, AUST J ZOOL, V48, P1, DOI 10.1071/ZO99067
Christe P, 2000, ECOL LETT, V3, P207
Christe P, 2007, J ANIM ECOL, V76, P703, DOI 10.1111/j.1365-2656.2007.01255.x
Codd JR, 2003, AUST J ZOOL, V51, P307, DOI 10.1071/ZO01079
de Castro F, 2005, ECOL LETT, V8, P117, DOI 10.1111/j.1461-0248.2004.00693.x
DELWP, 2017, NAT REC PLAN SO BENT
Dick CW, 2006, MICROMAMMALS AND MACROPARASITES: FROM EVOLUTIONARY ECOLOGY TO MANAGEMENT, P179, DOI 10.1007/978-4-431-36025-4_11
DOMROW R, 1987, Invertebrate Taxonomy, V1, P817, DOI 10.1071/IT9870817

DOMROW R, 1991, Invertebrate Taxonomy, V4, P1283
Domrow R, 1971, ACAROLOGIA, V13, P552
Dwyer P. D., 1965, Helictite, V4, P3
Evans L. N, 2009, ROOSTING BEHAV URBAN, P226
Giorgi MS, 2001, P ROY SOC B-BIOL SCI, V268, P2071, DOI 10.1098/rspb.2001.1686
Glenelg Hopkins Catchment Management Authority, 2013, GLEN HOPK REG CATCHM, P84
Godinho LN, 2013, ACTA CHIROPTEROL, V15, P463, DOI 10.3161/150811013X679080
Goldberg TL, 2017, SCI REP-UK, V7, DOI 10.1038/s41598-017-05236-w
González-Candela M, 2004, J WILDLIFE DIS, V40, P456, DOI 10.7589/0090-3558-40.3.456
Hasegawa H, 2012, J PARASITOL, V98, P995, DOI 10.1645/GE-2710.1
Holz PH, 2018, PLOS ONE, V13, DOI 10.1371/journal.pone.0204282
Holz Peter H., 2018, PLoS ONE, V13, pe0197625, DOI 10.1371/journal.pone.0197625
Lear K, 2012, J AUST CAVE KARST MA, V86, P15
Linhares AX, 2000, J PARASITOL, V86, P167, DOI 10.1645/0022-3395(2000)086[0167:TJAFAM]2.0.CO;2
Lourenço S, 2008, PARASITOL RES, V104, P127, DOI 10.1007/s00436-008-1170-6
Lucan RK, 2006, FOLIA PARASIT, V53, P147, DOI 10.14411/fp.2006.019
Lucan RK, 2016, PARASITE VECTOR, V9, DOI 10.1186/s13071-016-1302-2
MAA T C, 1971, Pacific Insects Monograph, V(28, P1
Martin RW, 1998, AUST VET J, V76, P411, DOI 10.1111/j.1751-0813.1998.tb12391.x
McCallum H, 2012, PHILOS T R SOC B, V367, P2828, DOI 10.1098/rstb.2012.0224
McKelvie P, 2013, J CLIN NEUROSCI, V20, P1019, DOI 10.1016/j.jocn.2012.08.009
McLean JA, 1997, ANIM BEHAV, V54, P1193, DOI 10.1006/anbe.1997.0498
McLelland DJ, 2013, J WILDLIFE DIS, V49, P1009, DOI 10.7589/2012-11-288
Obame-Nkoghe J, 2016, PARASITE VECTOR, V9, DOI 10.1186/s13071-016-1625-z
Olsson Annabelle, 2008, P465
Ramanantsalama RV, 2018, PARASITE VECTOR, V11, DOI 10.1186/s13071-018-2918-1
Randolph SE, 1999, J MED ENTOMOL, V36, P741, DOI 10.1093/jmedent/36.6.741
RAUSCH RL, 1983, ANN PARASIT HUM COMP, V58, P361, DOI 10.1051/parasite/1983584361
Richards G. C., 2008, MAMMALS AUSTR, P503
Rosenkranz P, 2010, J INVERTEBR PATHOL, V103, pS96, DOI 10.1016/j.jip.2009.07.016
Seltmann A, 2017, CONSERV PHYSIOL, V5, DOI 10.1093/conphys/cox020
Soulsbury CD, 2007, MAMMAL REV, V37, P278, DOI 10.1111/j.1365-2907.2007.00101.x
South East Natural Resources Management Board, 2010, S E NAT RES MAN PLAN, P158

Spratt DM, 2002, PARASITE, V9, P199, DOI 10.1051/parasite/2002093199
ter Hofstede HM, 2005, J ZOOL, V266, P333, DOI 10.1017/S095283690500693X
TIDEMANN CR, 1978, AUST WILDLIFE RES, V5, P355, DOI 10.1071/WR9780355
Victorian Environmental Assessment Council, 2010, REMN NAT VEG INV DIS, P57
Wibbelt G, 2010, BIOL LETTERS, V6, P438, DOI 10.1098/rsbl.2010.0267
Wilkinson DA, 2016, APPL ENVIRON MICROB, V82, P1778, DOI 10.1128/AEM.03505-15
Zahn A, 2004, J ZOOL, V262, P383, DOI 10.1017/S0952836903004722
NR 56
TC 7
Z9 8
U1 0
U2 8
PU ELSEVIER
PI AMSTERDAM
PA RADARWEG 29, 1043 NX AMSTERDAM, NETHERLANDS
SN 2213-2244
J9 INT J PARASITOL-PAR
JI Int. J. Parasitol.-Parasit. Wildl.
PD DEC
PY 2018
VL 7
IS 3
BP 423
EP 428
DI 10.1016/j.ijppaw.2018.10.006
PG 6
WC Ecology; Parasitology
WE Science Citation Index Expanded (SCI-EXPANDED)
SC Environmental Sciences & Ecology; Parasitology
GA HC8PH
UT WOS:000452065700026
PM 30425921
OA Green Published, gold
DA 2024-10-21
ER

PT J
AU Zhou, P
Tachedjian, M
Wynne, JW
Boyd, V
Cui, J
Smith, I
Cowled, C
Ng, JHJ
Mok, L
Michalski, WP
Mendenhall, IH

Tachedjian, G
   Wang, LF
   Baker, ML
AF Zhou, Peng
   Tachedjian, Mary
   Wynne, James W.
   Boyd, Victoria
   Cui, Jie
   Smith, Ina
   Cowled, Christopher
   Ng, Justin H. J.
   Mok, Lawrence
   Michalski, Wojtek P.
   Mendenhall, Ian H.
   Tachedjian, Gilda
   Wang, Lin-Fa
   Baker, Michelle L.
TI Contraction of the type I IFN locus and unusual constitutive expression
   of <i>IFN</i>-α in bats
SO PROCEEDINGS OF THE NATIONAL ACADEMY OF SCIENCES OF THE UNITED STATES OF
   AMERICA
LA English
DT Article
DE interferon; innate immunity; bat immunology
ID INTERFERON-ALPHA; GENOMIC ANALYSIS; MESSENGER-RNA; GENE; RESPONSES;
   VIRUS; IDENTIFICATION; ACTIVATION; INDUCTION; EVOLUTION
AB Bats harbor many emerging and reemerging viruses, several of which are highly pathogenic in other mammals but cause no clinical signs of disease in bats. To determine the role of interferons (IFNs) in the ability of bats to coexist with viruses, we sequenced the type I IFN locus of the Australian black flying fox, Pteropus alecto, providing what is, to our knowledge, the first gene map of the IFN region of any bat species. Our results reveal a highly contracted type I IFN family consisting of only 10 IFNs, including three functional IFN-alpha loci. Furthermore, the three IFN-alpha genes are constitutively expressed in unstimulated bat tissues and cells and their expression is unaffected by viral infection. Constitutively expressed IFN-alpha results in the induction of a subset of IFN-stimulated genes associated with antiviral activity and resistance to DNA damage, providing evidence for a unique IFN system that may be linked to the ability of bats to coexist with viruses.
C1 [Zhou, Peng; Tachedjian, Mary; Wynne, James W.; Boyd, Victoria; Smith, Ina; Cowled, Christopher; Ng, Justin H. J.; Wang, Lin-Fa; Baker, Michelle L.] CSIRO, Hlth & Biosecur Business Unit, Australian Anim Hlth Lab, Geelong, Vic 3220, Australia.
   [Zhou, Peng; Cui, Jie; Ng, Justin H. J.; Mendenhall, Ian H.; Wang, Lin-Fa] Duke Natl Univ Singapore, Sch Med, Program Emerging Infect Dis, Singapore 169857, Singapore.
   [Mok, Lawrence; Michalski, Wojtek P.] CSIRO, Australian Anim Hlth Lab, Geelong, Vic 3220, Australia.

[Tachedjian, Gilda] Burnet Inst, Ctr Biomed Res, Melbourne, Vic 3004, Australia.
[Tachedjian, Gilda] Monash Univ, Dept Microbiol, Clayton, Vic 3168, Australia.
[Tachedjian, Gilda] Monash Univ, Dept Infect Dis, Melbourne, Vic 3004, Australia.
[Tachedjian, Gilda] Univ Melbourne, Peter Doherty Inst Infect & Immun, Dept Microbiol & Immunol, Parkville, Vic 3010, Australia.
C3 Commonwealth Scientific & Industrial Research Organisation (CSIRO);
National University of Singapore; Commonwealth Scientific & Industrial
Research Organisation (CSIRO); Burnet Institute; Monash University;
Monash University; University of Melbourne; Peter Doherty Institute
RP Zhou, P; Baker, ML (corresponding author), CSIRO, Hlth & Biosecur Business Unit, Australian Anim Hlth Lab, Geelong, Vic 3220, Australia.; Zhou, P (corresponding author), Duke Natl Univ Singapore, Sch Med, Program Emerging Infect Dis, Singapore 169857, Singapore.
EM peng.zhou@duke-nus.edu.sg; michelle.baker@csiro.au
RI Tachedjian, Gilda/AAF-1327-2020; Michalski, Wojtek/HME-2772-2023; Smith,
Ina/H-7225-2013; Baker, Michelle/AAX-9171-2020; Cowled,
Christopher/H-5308-2013; Wynne, James/H-7957-2013; Cui,
Jie/ABB-3831-2020; Baker, Michelle/C-9694-2013
OI Cowled, Christopher/0000-0002-1969-9371; Mok,
Lawrence/0000-0003-0760-1370; Wynne, James/0000-0001-6846-757X; Cui,
Jie/0000-0001-8176-9951; Tachedjian, Gilda/0000-0002-7733-7037; Baker,
Michelle/0000-0002-7993-9971; Wang, Lin-Fa/0000-0003-2752-0535;
Mendenhall, Ian/0000-0003-4250-6459
FU National Institutes of Health Institutional Development Award Programme
of the National Centre for Research Resources [P20RR018754]; Australian
Research Council Future Fellowship [FT110100234]; Commonwealth
Scientific and Industrial Research Organization Chief Executive Officer
Science Leaders Award; Singaporean National Research Foundation
Competitive Research Programme [NRF-CRP10-2012-05]; National
Collaborative Research Infrastructure Strategy
FX We thank Susanne Wilson for P. alecto tissue collection and Yok Teng
Chionh and Dolyce Low Hong Wen for C. brachyotis tissue collection and
RNA extraction. This work was supported in part by National Institutes
of Health Institutional Development Award Programme of the National
Centre for Research Resources Grant P20RR018754 (to M.L.B.), Australian

Research Council Future Fellowship FT110100234 (to M.L.B.), a
Commonwealth Scientific and Industrial Research Organization
Chief
Executive Officer Science Leaders Award (to L-.F.W.), and
Singaporean
National Research Foundation Competitive Research Programme Grant
NRF-CRP10-2012-05 (to L-.F.W.), and by the National Collaborative
Research Infrastructure Strategy.
CR Abt MC, 2012, IMMUNITY, V37, P158, DOI 10.1016/
j.immuni.2012.04.011
Baker ML, 2013, ZOONOSES PUBLIC HLTH, V60, P104, DOI 10.1111/
j.1863-2378.2012.01528.x
Borden EC, 2007, NAT REV DRUG DISCOV, V6, P975, DOI 10.1038/
nrd2422
Calisher CH, 2006, CLIN MICROBIOL REV, V19, P531, DOI 10.1128/
CMR.00017-06
Cheon H, 2013, EMBO J, V32, P2751, DOI 10.1038/emboj.2013.203
CIVAS A, 1991, NUCLEIC ACIDS RES, V19, P4497, DOI 10.1093/nar/
19.16.4497
Crameri G, 2009, PLOS ONE, V4, DOI 10.1371/journal.pone.0008266
Dacheux L, 2014, PLOS ONE, V9, DOI 10.1371/journal.pone.0087194
de Weerd NA, 2012, IMMUNOL CELL BIOL, V90, P483, DOI 10.1038/
icb.2012.9
Detournay O, 2013, J INTERF CYTOK RES, V33, P746, DOI 10.1089/
jir.2012.0130
Fung KY, 2013, SCIENCE, V339, P1088, DOI 10.1126/science.1233321
Génin P, 2009, CYTOKINE GROWTH F R, V20, P283, DOI 10.1016/
j.cytogfr.2009.07.005
Génin P, 2009, MOL CELL BIOL, V29, P3435, DOI 10.1128/
MCB.01805-08
Gough DJ, 2012, IMMUNITY, V36, P166, DOI 10.1016/
j.immuni.2012.01.011
Guindon S, 2010, SYST BIOL, V59, P307, DOI 10.1093/sysbio/syq010
Haller O, 2011, J INTERF CYTOK RES, V31, P79, DOI 10.1089/
jir.2010.0076
Hamilton JA, 1996, J IMMUNOL, V156, P2553
Hardy MP, 2004, GENOMICS, V84, P331, DOI 10.1016/
j.ygeno.2004.03.003
He GM, 2010, BIOCHEM GENET, V48, P840, DOI 10.1007/
s10528-010-9365-9
He X, 2014, J VIROL, V88, P5444, DOI 10.1128/JVI.03403-13
HUGHES AL, 1995, NATURE, V377, P391, DOI 10.1038/377391a0
ITO Y, 1983, INFECT IMMUN, V39, P1019, DOI 10.1128/
IAI.39.3.1019-1023.1983
Jaks E, 2007, J MOL BIOL, V366, P525, DOI 10.1016/
j.jmb.2006.11.053
Kaiser P, 2005, J INTERF CYTOK RES, V25, P467, DOI 10.1089/
jir.2005.25.467
Kepler TB, 2010, BMC GENOMICS, V11, DOI 10.1186/1471-2164-11-444
Kumar S, 2011, BIOINFORMATICS, V27, P2023, DOI 10.1093/
bioinformatics/btr315
Li H, 2009, BIOINFORMATICS, V25, P1754, DOI 10.1093/
bioinformatics/btp324
Lovell PV, 2014, GENOME BIOL, V15, DOI 10.1186/s13059-014-0565-1

   Martin D, 2000, BIOINFORMATICS, V16, P562, DOI 10.1093/bioinformatics/16.6.562
   Middleton DJ, 2007, J COMP PATHOL, V136, P266, DOI 10.1016/j.jcpa.2007.03.002
   Neil SJD, 2008, NATURE, V451, P425, DOI 10.1038/nature06553
   Olson MV, 1999, AM J HUM GENET, V64, P18, DOI 10.1086/302219
   Omatsu T, 2008, VET IMMUNOL IMMUNOP, V124, P169, DOI 10.1016/j.vetimm.2008.03.004
   Pestka S, 2004, IMMUNOL REV, V202, P8, DOI 10.1111/j.0105-2896.2004.00204.x
   RAGG H, 1983, NATURE, V303, P439, DOI 10.1038/303439a0
   Randall RE, 2008, J GEN VIROL, V89, P1, DOI 10.1099/vir.0.83391-0
   Roberts RM, 1998, J INTERF CYTOK RES, V18, P805, DOI 10.1089/jir.1998.18.805
   Sétien AA, 1998, VACCINE, V16, P1122, DOI 10.1016/S0264-410X(98)80108-4
   Smith JDL, 2009, BIOL LETTERS, V5, P347, DOI 10.1098/rsbl.2009.0016
   Taniguchi T, 2001, NAT REV MOL CELL BIO, V2, P378, DOI 10.1038/35073080
   Thomas C, 2011, CELL, V146, P621, DOI 10.1016/j.cell.2011.06.048
   TOVEY MG, 1987, P NATL ACAD SCI USA, V84, P5038, DOI 10.1073/pnas.84.14.5038
   van Pesch V, 2003, J BIOL CHEM, V278, P46321, DOI 10.1074/jbc.M302554200
   Virtue ER, 2011, PLOS ONE, V6, DOI 10.1371/journal.pone.0022488
   VOGEL SN, 1984, INFECT IMMUN, V45, P417, DOI 10.1128/IAI.45.2.417-423.1984
   Walker AM, 2009, BMC GENOMICS, V10, DOI 10.1186/1471-2164-10-187
   Wynne JW, 2014, GENOME BIOL, V15, DOI 10.1186/s13059-014-0532-x
   Yang ZH, 2007, MOL BIOL EVOL, V24, P1586, DOI 10.1093/molbev/msm088
   Zhang GJ, 2013, SCIENCE, V339, P456, DOI 10.1126/science.1230835
   Zhou P, 2014, PLOS ONE, V9, DOI 10.1371/journal.pone.0103875
   Zhou P, 2013, DEV COMP IMMUNOL, V40, P240, DOI 10.1016/j.dci.2013.03.006
   Zhou P, 2011, PLOS ONE, V6, DOI 10.1371/journal.pone.0025385
   Zhou P, 2011, J IMMUNOL, V186, P3138, DOI 10.4049/jimmunol.1003115
NR 53
TC 220
Z9 245
U1 0
U2 47
PU NATL ACAD SCIENCES
PI WASHINGTON
PA 2101 CONSTITUTION AVE NW, WASHINGTON, DC 20418 USA
SN 0027-8424
J9 P NATL ACAD SCI USA
JI Proc. Natl. Acad. Sci. U. S. A.
PD MAR 8
PY 2016
VL 113
IS 10

BP 2696
EP 2701
DI 10.1073/pnas.1518240113
PG 6
WC Multidisciplinary Sciences
WE Science Citation Index Expanded (SCI-EXPANDED)
SC Science & Technology - Other Topics
GA DG4AW
UT WOS:000372013300043
PM 26903655
OA Bronze, Green Published
DA 2024-10-21
ER

PT J
AU Campolo, M
   Lorusso, E
   Leone, A
   Teodori, L
   Fogola, T
   Ronchi, FG
   Cito, F
   Di Sabatino, D
   Lorusso, A
AF Campolo, Marco
   Lorusso, Eleonora
   Leone, Alessandra
   Teodori, Liana
   Fogola, Teresa
   Ronchi, Federico Gaetano
   Cito, Francesca
   Di Sabatino, Daria
   Lorusso, Alessio
TI Development of a serum neutralization assay to detect Pteropine
   Orthoreovirus Indonesia/2010 neutralizing antibodies
SO VETERINARIA ITALIANA
LA English
DT Article
DE Pteropine Orthoreovirus; Serum-neutralization; Sera; Domestic
animals
ID FRUIT BATS; VIRUS; IDENTIFICATION; SURVEILLANCE; BLUETONGUE;
MALAYSIA;
   MEMBER
AB Pteropine Orthoreoviruses (PRVs) are fusogenic bat-borne
orthoreoviruses that cause flu-like upper respiratory tract
infections in humans. The presence of this group of viruses in bats
and humans has been well documented in areas where their biological
reservoirs - fruit bats (family Pteropodidae) - live densely. In the
present study, a serum neutralization (SN) assay to detect
neutralizing antibodies against PRV Indonesia/2010 isolate was set
up and used to assess the seroprevalence of this virus in Italian
domestic animals. The new developed assay was able of detecting PRV
neutralizing antibodies in the hyper-immune polyclonal serum
produced in rabbits (titer of 1:160). The negative serum was

negative at all tested dilutions. No cross-reactions have been evidenced neither against reference MRVs nor against their respective hyper-immune sera. Eight hundred and fifty-three serum samples collected from 524 bovines, 271 small ruminants, and 58 horses (all used as sentinel animals in the Bluetongue and West Nile disease National surveillance program) were also tested with the new developed SN assay. According to the results of this survey, neither PRV nor PRV cross-reacting viruses antibodies have been demonstrated in Italian domestic animals. However, the new developed SN assay could be a very valuable diagnostic tool to detect infection in animals and humans.
C1 [Campolo, Marco] Giardino Zool Pistoia, Via Pieve Celle, Pistoia, Italy.
[Campolo, Marco; Lorusso, Eleonora] Dipartimento Med Vet, Valenzano Bari, Italy.
[Leone, Alessandra; Teodori, Liana; Fogola, Teresa; Ronchi, Federico Gaetano; Cito, Francesca; Di Sabatino, Daria; Lorusso, Alessio] Ist Zooprofilatt Sperimentale Abruzzo & Molise G, I-64100 Teramo, Italy.
C3 IZS Dell'abruzzo E Del Molise Giuseppe Caporale
RP Lorusso, A (corresponding author), Ist Zooprofilatt Sperimentale Abruzzo & Molise G, I-64100 Teramo, Italy.
EM a.lorusso@izs.it
RI Teodori, Liana/E-5623-2016; Leone, Alessandra/E-3955-2016; Ronchi,
Gaetano Federico/G-2543-2016; Lorusso, Alessio/A-7311-2016; Cito,
Francesca/J-6371-2016; Di Sabatino, Daria/I-2868-2016
OI Ronchi, Gaetano Federico/0000-0002-7152-3373; Lorusso,
Alessio/0000-0001-6156-8212; LORUSSO, ELEONORA/0000-0002-5016-878X;
Cito, Francesca/0000-0002-4982-476X; Di Sabatino,
Daria/0000-0003-0348-3709
FU Italian Ministry of Health
FX Funding was provided by the Italian Ministry of Health. The authors
acknowledge Dr Davide Lelli (Istituto Zooprofilattico della Lombardia e
dell'Emilia-Romagna) for testing Indonesia 2010-positive serum sample
with representative MRV isolates.
CR CANNON RM, 1982, FIELD MANUAL VET
Cheng P, 2009, J CLIN VIROL, V45, P79, DOI 10.1016/j.jcv.2009.03.001
Chua KB, 2007, P NATL ACAD SCI USA, V104, P11424, DOI 10.1073/pnas.0701372104
Chua KB, 2011, PLOS ONE, V6, DOI 10.1371/journal.pone.0025434
Chua KB, 2008, PLOS ONE, V3, DOI 10.1371/journal.pone.0003803
Di Gennaro A, 2014, CLIN VACCINE IMMUNOL, V21, P1460, DOI 10.1128/CVI.00426-14
Du LF, 2010, ARCH VIROL, V155, P1295, DOI 10.1007/s00705-010-0690-4
Gard G.P., 1973, ARCH GESAMTE VIRUSFO, V43
Giovannini A, 2004, VETER ITAL SER, V40, P188
Giovannini A, 2004, VETER ITAL SER, V40, P369

   Hu TS, 2014, BMC MICROBIOL, V14, DOI 10.1186/s12866-014-0293-4
   International Committee on Taxonomy of Viruses, 2014, VIR TAX
2014 REL
   Kohl C, 2012, PLOS ONE, V7, DOI 10.1371/journal.pone.0043106
   Lelli D, 2013, ZOONOSES PUBLIC HLTH, V60, P84, DOI 10.1111/
zph.12001
   Lelli D, 2013, VIRUSES-BASEL, V5, P2679, DOI 10.3390/v5112679
   Lorusso A, 2015, INFECT GENET EVOL, V30, P55, DOI 10.1016/
j.meegid.2014.12.006
   Pritchard LI, 2006, ARCH VIROL, V151, P229, DOI 10.1007/
s00705-005-0644-4
   Ronchi GF, 2012, VET ITAL, V48, P67
   Singh H, 2016, EMERG MICROBES INFEC, V5, DOI 10.1038/emi.2016.35
   Voon K, 2015, J MED VIROL, V87, P2149, DOI 10.1002/jmv.24304
   Wong AH, 2012, EMERG INFECT DIS, V18, P944, DOI 10.3201/
eid1806.111688
   Yamanaka A, 2014, PLOS ONE, V9, DOI 10.1371/journal.pone.0092777
NR 22
TC 1
Z9 3
U1 0
U2 2
PU IST ZOOPROFILATTICO SPERIMENTALE ABRUZZO & MOLISE G CAPORALE-IZS
A&M
PI TERAMO
PA CAMPO BOARIO, TERAMO, 64100, ITALY
SN 0505-401X
EI 1828-1427
J9 VET ITAL
JI Vet. Ital.
PD APR-JUN
PY 2018
VL 54
IS 2
BP 161
EP 164
DI 10.12834/VetIt.1313.7525.1
PG 4
WC Veterinary Sciences
WE Science Citation Index Expanded (SCI-EXPANDED)
SC Veterinary Sciences
GA GU5XM
UT WOS:000445370300008
PM 29633229
DA 2024-10-21
ER

PT J
AU Kacprzyk, J
   Hughes, GM
   Palsson-McDermott, EM
   Quinn, SR
   Puechmaille, SJ
   O'Neill, LAJ

Teeling, EC
AF Kacprzyk, Joanna
Hughes, Graham M.
Palsson-McDermott, Eva M.
Quinn, Susan R.
Puechmaille, Sebastien J.
O'Neill, Luke A. J.
Teeling, Emma C.
TI A potent anti-inflammatory response in bat macrophages may be linked to extended longevity and viral tolerance
SO ACTA CHIROPTEROLOGICA
LA English
DT Article
DE longevity; bat; viral response; phylogenetics; immune system evolution
ID OXIDATIVE STRESS; IMMUNE-RESPONSE; DENDRITIC CELLS; EVOLUTION; DISEASE;
INFLAMMATION; IMMUNOLOGY; EXPRESSION; IL-1-BETA; ALIGNMENT
AB Bats are unique among mammals given their ability to fly, apparent tolerance of deadly viruses and extraordinary longevity. We propose that these traits are linked and driven by adaptations of the innate immune system. To explore this hypothesis we challenged macrophages from the greater mouse-eared bat, Myotis myotis and the house mouse, Mus musculus with toll like receptors (TLRs) ligands, lipopolysaccharides, LPS and polyinosinic-polycytidylic acid, Poly(I: C). Macrophages from both species presented a high level of mRNA induction of inferon beta (INF-beta), tumor necrosis factor (TNF) and interleukin-1 beta (Il-1 beta). However, in bat macrophages, this antiviral, proinflammatory response was balanced by a sustained high-level transcription of the anti-inflammatory cytokine Il-10, which was not observed in mouse, potentially resulting from adaptive regulation in bats. Additionally, phylogenomic selection tests across the basal divergences in mammals (n = 39) uncovered bat-specific adaptations in six genes involved in antiviral and proinflammatory signalling. Based on this pilot study, we put forward a hypothesis that bats may have evolved unique antiinflammatory responses to neutralize proinflammatory stimuli resulting from flight. This in turn may drive their extraordinary longevity and viral tolerance by limiting inflammation driven ageing and infection-induced immunopathology. Further data from other individuals and bat species are required to advance this intriguing hypothesis.
C1 [Kacprzyk, Joanna; Hughes, Graham M.; Teeling, Emma C.] Univ Coll Dublin, Sch Biol & Environm Sci, Dublin 4, Ireland.
[Palsson-McDermott, Eva M.; Quinn, Susan R.; O'Neill, Luke A. J.] Trinity Coll Dublin, Trinity Biomed Sci Inst, Sch Biochem & Immunol, 152-160 Pearse St, Dublin 2, Ireland.
[Puechmaille, Sebastien J.] Greifswald Univ, Zool Inst & Museum, Soldmann Str 14, D-17489 Greifswald, Germany.
C3 University College Dublin; Trinity College Dublin
RP Teeling, EC (corresponding author), Univ Coll Dublin, Sch Biol & Environm Sci, Dublin 4, Ireland.
EM emma.teeling@ucd.ie

RI Kacprzyk, Janusz/M-9574-2014; Puechmaille, Sebastien/D-1612-2010
OI Hughes, Graham/0000-0003-3088-345X; Teeling, Emma/
0000-0002-3309-1346;
Puechmaille, Sebastien/0000-0001-9517-5775; Kacprzyk,
Joanna/0000-0003-1054-2367
FU Science Foundation Ireland; [ERC-2012-StG311000]
FX We would like to thank M. Haneklaus, F. Touzalin, N. Foley, D. Jebb and
S. Dool for their assistance. This study was funded by
ERC-2012-StG311000 grant awarded to Emma C. Teeling, UCD Seed Funding
SF1123 and Science Foundation Ireland funding awarded to Luke O'Neill.
We thank Bretagne Vivante, and numerous volunteers and students from UCD
for help in the field. We thank the Prefet du Morbihan for the permit
granted to Frederic Touzalin and Sebastien Puechmaille (Arrete
prefectoral [26/09/2016]) to collect, transport and use dead bats for
scientific purposes.
CR Ahn M, 2016, SCI REP-UK, V6, DOI 10.1038/srep21722
Akira S, 2004, NAT REV IMMUNOL, V4, P499, DOI 10.1038/nri1391
ALTSCHUL SF, 1990, J MOL BIOL, V215, P403, DOI 10.1016/S0022-2836(05)80360-2
[Anonymous], ELS J
[Anonymous], J INTERFERON CYTOKIN
[Anonymous], 2010, PLOS ONE, DOI DOI 10.1371/journal.pone.0011978
Arai Y, 2015, EBIOMEDICINE, V2, P1549, DOI 10.1016/j.ebiom.2015.07.029
Austad SN, 2010, J COMP PATHOL, V141, pS10, DOI 10.1016/j.jcpa.2009.10.024
BENJAMINI Y, 1995, J R STAT SOC B, V57, P289, DOI 10.1111/j.2517-6161.1995.tb02031.x
Benson DA, 2015, NUCLEIC ACIDS RES, V43, pD30, DOI 10.1093/nar/gku1216
Brook CE, 2015, TRENDS MICROBIOL, V23, P172, DOI 10.1016/j.tim.2014.12.004
Brooks DG, 2006, NAT MED, V12, P1301, DOI 10.1038/nm1492
Buffenstein R, 2008, AGE, V30, P99, DOI 10.1007/s11357-008-9058-z
Burdette DL, 2013, NAT IMMUNOL, V14, P19, DOI 10.1038/ni.2491
Dimitrijevic M, 2014, BIOGERONTOLOGY, V15, P475, DOI 10.1007/s10522-014-9513-4
Dinarello CA, 2013, FRONT IMMUNOL, V4, DOI 10.3389/fimmu.2013.00289
Dinarello CA, 2011, EUR J IMMUNOL, V41, P1203, DOI 10.1002/eji.201141550
Ding XB, 2017, SCI REP-UK, V7, DOI 10.1038/srep34278
Edgar RC, 2004, NUCLEIC ACIDS RES, V32, P1792, DOI 10.1093/nar/gkh340
Foley NM, 2016, PHILOS T R SOC B, V371, DOI 10.1098/rstb.2015.0140
Franceschi C, 2014, J GERONTOL A-BIOL, V69, pS4, DOI 10.1093/gerona/glu057

Gaisler J., 2003, VESPERTILIO, V7, P3
GREER DL, 1981, AM J TROP MED HYG, V30, P653, DOI 10.4269/ajtmh.1981.30.653
Guarda G, 2011, IMMUNITY, V34, P213, DOI 10.1016/j.immuni.2011.02.006
Guiyedi V, 2015, MALARIA J, V14, DOI 10.1186/s12936-015-0658-7
Guo HT, 2015, NAT MED, V21, P677, DOI 10.1038/nm.3893
Healy K, 2014, P ROY SOC B-BIOL SCI, V281, DOI 10.1098/rspb.2014.0298
Huang ZX, 2016, MOL ECOL RESOUR, V16, P150, DOI 10.1111/1755-0998.12447
Kelly B, 2015, CELL RES, V25, P771, DOI 10.1038/cr.2015.68
Kirwan JD, 2013, EVOL APPL, V6, P412, DOI 10.1111/eva.12047
Kosoy M, 2010, EMERG INFECT DIS, V16, P1875, DOI 10.3201/eid1612.100601
Ksiazek A, 2012, PHYSIOL BIOCHEM ZOOL, V85, P51, DOI 10.1086/663696
Kühl A, 2011, J INFECT DIS, V204, pS840, DOI 10.1093/infdis/jir306
Livak KJ, 2001, METHODS, V25, P402, DOI 10.1006/meth.2001.1262
Lood C, 2016, NAT MED, V22, P146, DOI 10.1038/nm.4027
Lu YC, 2008, CYTOKINE, V42, P145, DOI 10.1016/j.cyto.2008.01.006
Matsumoto M, 2008, ADV DRUG DELIVER REV, V60, P805, DOI 10.1016/j.addr.2007.11.005
Medzhitov R, 2000, NEW ENGL J MED, V343, P338, DOI 10.1056/NEJM200008033430506
Meredith RW, 2011, SCIENCE, V334, P521, DOI 10.1126/science.1211028
Miller MR, 2016, J INFECT DIS, V214, pS297, DOI 10.1093/infdis/jiw199
Munshi-South J, 2010, AGEING RES REV, V9, P12, DOI 10.1016/j.arr.2009.07.006
O'Shea TJ, 2014, EMERG INFECT DIS, V20, P741, DOI 10.3201/eid2005.130539
Okumura A, 2010, J VIROL, V84, P27, DOI 10.1128/JVI.01462-09
Otálora-Ardila A, 2016, PLOS ONE, V11, DOI 10.1371/journal.pone.0164938
R Core Team, 2016, LANG ENV STAT COMP
Rouse BT, 2010, NAT REV IMMUNOL, V10, P514, DOI 10.1038/nri2802
Ruedi M, 2013, MOL PHYLOGENET EVOL, V69, P437, DOI 10.1016/j.ympev.2013.08.011
Sabharwal SS, 2014, NAT REV CANCER, V14, P709, DOI 10.1038/nrc3803
Schaer J, 2013, P NATL ACAD SCI USA, V110, P17415, DOI 10.1073/pnas.1311016110
Schneeberger K, 2013, J EXP BIOL, V216, P4514, DOI 10.1242/jeb.090837
Schwarz F, 2015, ELIFE, V4, DOI 10.7554/eLife.06184
Speakman JR, 2003, BAT ECOLOGY, P430
Stockmaier S, 2015, BIOL LETTERS, V11, DOI 10.1098/rsbl.2015.0576
Suyama M, 2006, NUCLEIC ACIDS RES, V34, pW609, DOI 10.1093/nar/gkl315
Tannahill GM, 2013, NATURE, V496, P238, DOI 10.1038/nature11986
Tatematsu M, 2014, BIOCHEM J, V458, P195, DOI 10.1042/BJ20131492

   Tisoncik JR, 2012, MICROBIOL MOL BIOL R, V76, P16, DOI 10.1128/
MMBR.05015-11
   Untergasser A, 2012, NUCLEIC ACIDS RES, V40, DOI 10.1093/nar/
gks596
   Wang F, 2016, NAT IMMUNOL, V17, P514, DOI 10.1038/ni.3433
   Weyer J, 2015, CURR INFECT DIS REP, V17, DOI 10.1007/
s11908-015-0480-y
   Yang ZH, 2005, MOL BIOL EVOL, V22, P1107, DOI 10.1093/molbev/
msi097
   Yang ZH, 2007, MOL BIOL EVOL, V24, P1586, DOI 10.1093/molbev/
msm088
   Zhang GJ, 2013, SCIENCE, V339, P456, DOI 10.1126/science.1230835
   Zhou P, 2016, SCI REP-UK, V6, DOI 10.1038/srep38597
   Zhou P, 2016, P NATL ACAD SCI USA, V113, P2696, DOI 10.1073/
pnas.1518240113
NR 65
TC 46
Z9 51
U1 1
U2 44
PU MUSEUM & INST ZOOLOGY PAS-POLISH ACAD SCIENCES
PI WARSAW
PA WILCZA STREET 64, 00-679 WARSAW, POLAND
SN 1508-1109
EI 1733-5329
J9 ACTA CHIROPTEROL
JI Acta Chiropt.
PD DEC
PY 2017
VL 19
IS 2
BP 219
EP 228
DI 10.3161/15081109ACC2017.19.2.001
PG 10
WC Zoology
WE Science Citation Index Expanded (SCI-EXPANDED)
SC Zoology
GA FT3EN
UT WOS:000423030100001
OA Green Submitted
DA 2024-10-21
ER

PT J
AU Pasquesi, GIM
   Kelly, CJ
   Ordonez, AD
   Chuong, EB
AF Pasquesi, Giulia Irene Maria
   Kelly, Conor J.
   Ordonez, Andrea D.
   Chuong, Edward B.
TI Transcriptional dynamics of transposable elements in the type I

IFN
   response in <i>Myotis lucifugus</i> cells
SO MOBILE DNA
LA English
DT Article
DE Bat immunity; Transposable elements; Epigenomics
ID CO-OPTION; HI-C; BAT; EXPRESSION; ACTIVATION; EVOLUTION; SEARCH; CANCER;
   GENES
AB Background Bats are a major reservoir of zoonotic viruses, and there has been growing interest in characterizing bat-specific features of innate immunity and inflammation. Recent studies have revealed bat-specific adaptations affecting interferon (IFN) signaling and IFN-stimulated genes (ISGs), but we still have a limited understanding of the genetic mechanisms that have shaped the evolution of bat immunity. Here we investigated the transcriptional and epigenetic dynamics of transposable elements (TEs) during the type I IFN response in little brown bat (Myotis lucifugus) primary embryonic fibroblast cells, using RNA-seq and CUT&RUN. Results We found multiple bat-specific TEs that undergo both locus-specific and family-level transcriptional induction in response to IFN. Our transcriptome reassembly identified multiple ISGs that have acquired novel exons from bat-specific TEs, including NLRC5, SLNF5 and a previously unannotated isoform of the IFITM2 gene. We also identified examples of TE-derived regulatory elements, but did not find strong evidence supporting genome-wide epigenetic activation of TEs in response to IFN. Conclusion Collectively, our study uncovers numerous TE-derived transcripts, proteins, and alternative isoforms that are induced by IFN in Myotis lucifugus cells, highlighting candidate loci that may contribute to bat-specific immune function.
C1 [Chuong, Edward B.] Univ Colorado, BioFrontiers Inst, 596 UCB, Boulder, CO 80309 USA.
   Univ Colorado, Dept Mol Cellular & Dev Biol, 596 UCB, Boulder, CO 80309 USA.
C3 University of Colorado System; University of Colorado Boulder;
   University of Colorado System; University of Colorado Boulder
RP Chuong, EB (corresponding author), Univ Colorado, BioFrontiers Inst, 596 UCB, Boulder, CO 80309 USA.
EM edward.chuong@colorado.edu
OI Kelly, Conor/0000-0003-2410-7892; Pasquesi, Giulia Irene
   Maria/0000-0002-3400-8057; Chuong, Edward/0000-0002-5392-937X
FU NIH [1R35GM128822]; Alfred P. Sloan Foundation; David and Lucile Packard
   Foundation; Boettcher foundation; National Institute of General Medical
   Sciences [R35GM128822] Funding Source: NIH RePORTER
FX This study was supported by the NIH (1R35GM128822), the Alfred P. Sloan
   Foundation, the David and Lucile Packard Foundation, and the Boettcher
   foundation.
CR Ahn M, 2019, NAT MICROBIOL, V4, P789, DOI 10.1038/s41564-019-0371-3
   Ahn M, 2016, SCI REP-UK, V6, DOI 10.1038/srep21722

Badarinarayan SS, 2020, NUCLEIC ACIDS RES, V48, P10890, DOI 10.1093/nar/gkaa832
Bairoch A, 2000, NUCLEIC ACIDS RES, V28, P45, DOI 10.1093/nar/28.1.45
Bao WD, 2015, MOBILE DNA-UK, V6, DOI 10.1186/s13100-015-0041-9
BBMap, SourceForge
Belton JM, 2012, METHODS, V58, P268, DOI 10.1016/j.ymeth.2012.05.001
Benkö S, 2017, FRONT IMMUNOL, V8, DOI 10.3389/fimmu.2017.00150
bioinformatics, Babraham Bioinformatics-Trim Galore!
Blanco-Melo D, 2017, ELIFE, V6, DOI 10.7554/eLife.22519
Böhne A, 2012, MOL BIOL EVOL, V29, P631, DOI 10.1093/molbev/msr208
Bogdan L, 2020, PHILOS T R SOC B, V375, DOI 10.1098/rstb.2019.0332
Bondet V, 2021, FRONT IMMUNOL, V12, DOI 10.3389/fimmu.2021.735866
Camacho C, 2009, BMC BIOINFORMATICS, V10, DOI 10.1186/1471-2105-10-421
CCGB: Miller Lab LASTZ, US
Chiappinelli KB, 2015, CELL, V162, P974, DOI 10.1016/j.cell.2015.07.011
Chuong EB, 2016, SCIENCE, V351, P1083, DOI 10.1126/science.aad5497
Cosby RL, 2021, SCIENCE, V371, P797, DOI 10.1126/science.abc6405
De La Cruz-Rivera PC, 2018, J IMMUNOL, V200, P209, DOI 10.4049/jimmunol.1701214
Dobin A, 2013, BIOINFORMATICS, V29, P15, DOI 10.1093/bioinformatics/bts635
Dudchenko O., 2018, bioRxiv, DOI DOI 10.1101/254797
Dudchenko O, 2017, SCIENCE, V356, P92, DOI 10.1126/science.aal3327
Dunham I, 2012, NATURE, V489, P57, DOI 10.1038/nature11247
Edgar RC, 2010, BIOINFORMATICS, V26, P2460, DOI 10.1093/bioinformatics/btq461
Escalera-Zamudio M, 2015, MOL ECOL, V24, P5899, DOI 10.1111/mec.13431
Ewels P, 2016, BIOINFORMATICS, V32, P3047, DOI 10.1093/bioinformatics/btw354
Flynn JM, 2020, P NATL ACAD SCI USA, V117, P9451, DOI 10.1073/pnas.1921046117
Fornes O, 2020, NUCLEIC ACIDS RES, V48, pD87, DOI 10.1093/nar/gkz1001
Frank JA, 2017, CURR OPIN VIROL, V25, P81, DOI 10.1016/j.coviro.2017.07.021
Gorbunova V, 2020, CELL METAB, V32, P31, DOI 10.1016/j.cmet.2020.06.013
Grabundzija I, 2016, NAT COMMUN, V7, DOI 10.1038/ncomms10716
Grant CE., 2021, BIORXIV, DOI DOI 10.1101/2021.09.02.458722
Halpin K, 2011, AM J TROP MED HYG, V85, P946, DOI 10.4269/ajtmh.2011.10-0567
Horie M, 2016, SCI REP-UK, V6, DOI 10.1038/srep25873
Janssens D., 2019, CUT RUN TARGETED IN
Jin Y, 2015, BIOINFORMATICS, V31, P3593, DOI 10.1093/bioinformatics/btv422

Kelly CJ, 2021, bioRxiv, DOI [10.1101/2021.10.01.462810, DOI 10.1101/2021.10.01.462810, 10.1101/2021.10.01.462810]
Kepler TB, 2010, BMC GENOMICS, V11, DOI 10.1186/1471-2164-11-444
Kobayashi KS, 2012, NAT REV IMMUNOL, V12, P813, DOI 10.1038/nri3339
Kovaka S, 2019, GENOME BIOL, V20, DOI 10.1186/s13059-019-1910-1
Langmead B, 2012, NAT METHODS, V9, P357, DOI [10.1038/NMETH.1923, 10.1038/nmeth.1923]
Layer RM, 2018, NAT METHODS, V15, P123, DOI 10.1038/nmeth.4556
Li B, 2011, BMC BIOINFORMATICS, V12, DOI 10.1186/1471-2105-12-323
Li H, 2009, BIOINFORMATICS, V25, P2078, DOI 10.1093/bioinformatics/btp352
Liao YX, 2019, NUCLEIC ACIDS RES, V47, pW199, DOI 10.1093/nar/gkz401
Lindblad-Toh K, 2011, NATURE, V478, P476, DOI 10.1038/nature10530
Liu T, 2014, METHODS MOL BIOL, V1150, P81, DOI 10.1007/978-1-4939-0512-6_4
Love MI, 2014, GENOME BIOL, V15, DOI 10.1186/s13059-014-0550-8
Luis AD, 2013, P ROY SOC B-BIOL SCI, V280, DOI 10.1098/rspb.2012.2753
Meers MP, 2019, ELIFE, V8, DOI 10.7554/eLife.46314
Mi S, 2000, NATURE, V403, P785, DOI 10.1038/35001608
Mitra R, 2013, P NATL ACAD SCI USA, V110, P234, DOI 10.1073/pnas.1217548110
Munster VJ, 2016, SCI REP-UK, V6, DOI 10.1038/srep21878
Mura M, 2004, P NATL ACAD SCI USA, V101, P11117, DOI 10.1073/pnas.0402877101
Myotis_lucifugus, MYOT LUC
Nakagawa S, 2016, DATABASE-OXFORD, DOI 10.1093/database/baw087
Ostuni R, 2013, CELL, V152, P157, DOI 10.1016/j.cell.2012.12.018
Pasquesi GIM, 2020, GENOME BIOL EVOL, V12, P506, DOI 10.1093/gbe/evaa068
Pasquesi GIM, 2018, NAT COMMUN, V9, DOI 10.1038/s41467-018-05279-1
Pavlovich SS, 2018, CELL, V173, P1098, DOI 10.1016/j.cell.2018.03.070
Pertea Geo, 2020, F1000Res, V9, P304, DOI 10.12688/f1000research.23297.1
Pritham EJ, 2007, P NATL ACAD SCI USA, V104, P1895, DOI 10.1073/pnas.0609601104
Qiao Y, 2013, IMMUNITY, V39, P454, DOI 10.1016/j.immuni.2013.08.009
Quinlan AR, 2010, BIOINFORMATICS, V26, P841, DOI 10.1093/bioinformatics/btq033
Ramírez F, 2016, NUCLEIC ACIDS RES, V44, pW160, DOI 10.1093/nar/gkw257
Ramírez F, 2014, NUCLEIC ACIDS RES, V42, pW187, DOI 10.1093/nar/gku365
Ray DA, 2008, GENOME RES, V18, P717, DOI 10.1101/gr.071886.107
Ray DA, 2007, MOL BIOL EVOL, V24, P632, DOI 10.1093/molbev/msl192
repeatmasker, RepeatMasker Home Page
Roulois D, 2015, CELL, V162, P961, DOI 10.1016/j.cell.2015.07.056
Sayers EW, 2022, NUCLEIC ACIDS RES, V50, pD20, DOI 10.1093/nar/gkab1112

Schmidt N, 2019, P NATL ACAD SCI USA, V116, P17399, DOI 10.1073/pnas.1907031116
Schuh AJ, 2017, SCI REP-UK, V7, DOI 10.1038/s41598-017-07824-2
Shaw AE, 2017, PLOS BIOL, V15, DOI 10.1371/journal.pbio.2004086
Skirmuntt EC, 2020, ANNU REV VIROL, V7, P103, DOI 10.1146/annurev-virology-092818-015613
Skirmuntt EC, 2019, VIRUS RES, V270, DOI 10.1016/j.virusres.2019.197645
Swanepoel R, 1996, EMERG INFECT DIS, V2, P321, DOI 10.3201/eid0204.960407
Tempel Sebastien, 2012, Methods Mol Biol, V859, P29, DOI 10.1007/978-1-61779-603-6_2
van de Lagemaat LN, 2003, TRENDS GENET, V19, P530, DOI 10.1016/j.tig.2003.08.004
Wickenhagen A, 2021, SCIENCE, V374, DOI 10.1126/science.abj3624
Xie JZ, 2018, CELL HOST MICROBE, V23, P297, DOI 10.1016/j.chom.2018.01.006
Xiong WW, 2014, P NATL ACAD SCI USA, V111, P10263, DOI 10.1073/pnas.1410068111
Xue AT, 2018, GENOME BIOL EVOL, V10, P1265, DOI 10.1093/gbe/evy083
Young GR, 2018, P NATL ACAD SCI USA, V115, P10130, DOI 10.1073/pnas.1808516115
Zhang GJ, 2013, SCIENCE, V339, P456, DOI 10.1126/science.1230835
Zhou P, 2016, P NATL ACAD SCI USA, V113, P2696, DOI 10.1073/pnas.1518240113
Zhu AQ, 2019, BIOINFORMATICS, V35, P2084, DOI 10.1093/bioinformatics/bty895
NR 87
TC 3
Z9 3
U1 0
U2 6
PU BMC
PI LONDON
PA CAMPUS, 4 CRINAN ST, LONDON N1 9XW, ENGLAND
SN 1759-8753
J9 MOBILE DNA-UK
JI Mob. DNA
PD SEP 6
PY 2022
VL 13
IS 1
AR 22
DI 10.1186/s13100-022-00277-z
PG 17
WC Genetics & Heredity
WE Science Citation Index Expanded (SCI-EXPANDED)
SC Genetics & Heredity
GA 4I4WF
UT WOS:000850578100001
PM 36068622
OA gold, Green Published, Green Submitted
DA 2024-10-21

ER

PT J
AU Pons-Salort, M
   Serra-Cobo, J
   Jay, F
   López-Roig, M
   Lavenir, R
   Guillemot, D
   Letort, V
   Bourhy, H
   Opatowski, L
AF Pons-Salort, Margarita
   Serra-Cobo, Jordi
   Jay, Flora
   Lopez-Roig, Marc
   Lavenir, Rachel
   Guillemot, Didier
   Letort, Veronique
   Bourhy, Herve
   Opatowski, Lulla
TI Insights into Persistence Mechanisms of a Zoonotic Virus in Bat Colonies
   Using a Multispecies Metapopulation Model
SO PLOS ONE
LA English
DT Article
ID SENSITIVITY-ANALYSIS; NATURAL COLONIES; MYOTIS-MYOTIS; RABIES;
   LYSSAVIRUSES; INFECTION; DYNAMICS; TRANSMISSION; EVOLUTION; RECORD
AB Rabies is a worldwide zoonosis resulting from Lyssavirus infection. In Europe, Eptesicus serotinus is the most frequently reported bat species infected with Lyssavirus, and thus considered to be the reservoir of European bat Lyssavirus type 1 (EBLV-1). To date, the role of other bat species in EBLV-1 epidemiology and persistence remains unknown. Here, we built an EBLV-12transmission model based on local observations of a three-cave and four-bat species (Myotis capaccinii, Myotis myotis, Miniopterus schreibersii, Rhinolophus ferrumequinum) system in the Balearic Islands, for which a 1995-2011 serological dataset indicated the continuous presence of EBLV-1. Eptesicus serotinus was never observed in the system during the 16-year follow-up and therefore was not included in the model. We used the model to explore virus persistence mechanisms and to assess the importance of each bat species in the transmission dynamics. We found that EBLV-1 could not be sustained if transmission between M. schreibersii and other bat species was eliminated, suggesting that this species serves as a regional reservoir. Global sensitivity analysis using Sobol's method revealed that following the rate of autumn-winter infectious contacts, M. schreibersii's incubation-and immune-period durations, but not the infectious period length, were the most relevant factors driving virus persistence.
C1 [Pons-Salort, Margarita; Guillemot, Didier; Opatowski, Lulla] Inst Pasteur, Unite Pharmacoepidemiol & Malad Infect, Paris, France.

[Pons-Salort, Margarita; Guillemot, Didier; Opatowski, Lulla] INSERM, U657, Paris, France.
[Pons-Salort, Margarita; Guillemot, Didier; Opatowski, Lulla] Univ Versailles St Quentin En Yvelines, EA 4499, Fac Med Paris Ile de France Ouest, Garches, France.
[Serra-Cobo, Jordi; Lopez-Roig, Marc] Ctr Recerca Infecc Viriques Illes Balears, Palma De Mallorca, Spain.
[Serra-Cobo, Jordi] Univ Barcelona, Dept Biol Anim, Inst Recerca Biodiversitat, Barcelona, Spain.
[Jay, Flora] Univ Calif Berkeley, Dept Integrat Biol, Berkeley, CA 94720 USA.
[Lavenir, Rachel; Bourhy, Herve] WHO Collaborat Ctr Reference & Res Rabies, Natl Reference Ctr Rabies, Inst Pasteur, Unite Dynam Lyssavirus & Adaptat Hote, Paris, France.
[Guillemot, Didier] Hop Raymond Poincare, AP HP, Garches, France.
[Letort, Veronique] Ecole Cent Paris, Lab Math Appl Syst, Chatenay Malabry, France.
C3 Pasteur Network; Universite Paris Cite; Institut Pasteur Paris; Institut
National de la Sante et de la Recherche Medicale (Inserm); Universite
Paris Saclay; University of Barcelona; University of California System;
University of California Berkeley; Pasteur Network; Universite Paris
Cite; Institut Pasteur Paris; Assistance Publique Hopitaux Paris (APHP);
Hopital Universitaire Raymond-Poincare - APHP; Universite Paris Saclay
RP Pons-Salort, M (corresponding author), Inst Pasteur, Unite Pharmacoepidemiol & Malad Infect, Paris, France.
EM margapons@gmail.com
RI Guillemot, Didier/AAN-3051-2020; Serra-Cobo, Jordi/G-4673-2016;
Opatowski, Lulla/I-3143-2015
OI Letort, Veronique/0000-0002-0991-2351; jay, flora/0000-0001-5884-4730;
Opatowski, Lulla/0000-0001-8348-700X; Guillemot,
Didier/0000-0002-8497-1765; Pons-Salort, Margarita/0000-0001-5597-9285;
Lopez-Roig, Marc/0000-0002-8204-0895
FU PREDEMICS UE [278433]; French Government's Investissement d'Avenir
program, Laboratoire d'Excellence "Integrative Biology of Emerging
Infectious Diseases'' [ANR- 10-LABX-62-IBEID]
FX This work was funded by the PREDEMICS UE project No. 278433, and the
French Government's Investissement d'Avenir program, Laboratoire
d'Excellence "Integrative Biology of Emerging Infectious Diseases''
(grant n degrees ANR- 10-LABX-62-IBEID). The funders had no role in
study design, data collection and analysis, decision to publish, or

preparation of the manuscript.
CR Amengual B, 2008, DEV BIOLOGICALS, V131, P547
Amengual B, 1997, J GEN VIROL, V78, P2319, DOI 10.1099/0022-1317-78-9-2319
Amengual B, 2005, EVOLUCIO PODER PATOG
Amengual B, 2007, PLOS ONE, V2, DOI 10.1371/journal.pone.0000566
Amengual B, 2007, ACTA CHIROPTEROL, V9, P319, DOI 10.3161/1733-5329(2007)9[319:FROSOS]2.0.CO;2
AUBERT MFA, 1991, ANN RECH VET, V22, P77
Banyard AC, 2011, ADV VIRUS RES, V79, P239, DOI 10.1016/B978-0-12-387040-7.00012-3
Cariboni J, 2007, ECOL MODEL, V203, P167, DOI 10.1016/j.ecolmodel.2005.10.045
Dacheux L, 2009, EMERG INFECT DIS, V15, P280, DOI 10.3201/eid1502.080637
Davis PL, 2005, J VIROL, V79, P10487, DOI 10.1128/JVI.79.16.10487-10497.2005
Fooks AR, 2003, EPIDEMIOL INFECT, V131, P1029, DOI 10.1017/S0950268803001481
Georgea DB, 2011, P NATL ACAD SCI USA, V108, P10208, DOI 10.1073/pnas.1010875108
Hayman DTS, 2012, EPIDEMIOL INFECT, V140, P2163, DOI 10.1017/S0950268812000167
Hutterer R, 2005, BAT MIGRATIONS EUROP
Jackson FR, 2008, J WILDLIFE DIS, V44, P612, DOI 10.7589/0090-3558-44.3.612
Knobel DL, 2005, B WORLD HEALTH ORGAN, V83, P360
Lloyd-Smith JO, 2005, TRENDS ECOL EVOL, V20, P511, DOI 10.1016/j.tree.2005.07.004
PEREZJORDA JL, 1995, J WILDLIFE DIS, V31, P372, DOI 10.7589/0090-3558-31.3.372
Restif O, 2012, ECOL LETT, V15, P1083, DOI 10.1111/j.1461-0248.2012.01836.x
Saltelli A, 2000, STAT SCI, V15, P377, DOI 10.1214/ss/1009213004
Serra-Cobo J, 2002, EMERG INFECT DIS, V8, P413, DOI 10.3201/eid0804.010263
Serra-Cobo J, 2009, Ratpenats. Ciencia i mite
Serra-Cobo J, 2013, PLOS ONE, V8, DOI 10.1371/journal.pone.0064467
Streicker DG, 2012, P ROY SOC B-BIOL SCI, V279, P3384, DOI 10.1098/rspb.2012.0538
Teeling EC, 2005, SCIENCE, V307, P580, DOI 10.1126/science.1105113
Wu JY, 2013, J R SOC INTERFACE, V10, DOI 10.1098/rsif.2012.1018
NR 26
TC 13
Z9 14
U1 0
U2 38
PU PUBLIC LIBRARY SCIENCE
PI SAN FRANCISCO
PA 1160 BATTERY STREET, STE 100, SAN FRANCISCO, CA 94111 USA
SN 1932-6203
J9 PLOS ONE

JI PLoS One
PD APR 22
PY 2014
VL 9
IS 4
AR e95610
DI 10.1371/journal.pone.0095610
PG 11
WC Multidisciplinary Sciences
WE Science Citation Index Expanded (SCI-EXPANDED)
SC Science & Technology - Other Topics
GA AG2IS
UT WOS:000335240300061
PM 24755619
OA Green Published, Green Submitted, gold
DA 2024-10-21
ER

PT J
AU Schad, J
   Voigt, CC
AF Schad, Julia
   Voigt, Christian C.
TI Adaptive evolution of virus-sensing toll-like receptor 8 in bats
SO IMMUNOGENETICS
LA English
DT Article
DE Toll-like receptor; TLR8; Persistent positive selection; Episodic
   positive selection; ssRNA virus; Chiroptera
ID PHYLOGENETIC ANALYSIS; STRANDED-RNA; RECOGNITION; SIGNATURES;
SELECTION;
   ACTIVATION; LIGAND; GENES; SUSCEPTIBILITY; POLYMORPHISMS
AB Recently, bats have gained attention as potential reservoir hosts
for emerging zoonotic single-stranded (ssRNA) viruses that may prove
fatal for humans and other mammals. It has been hypothesized that
some features of their innate immune system may enable bats to
trigger an efficient early immune response. Toll-like receptors
(TLRs) represent a first line defense within the innate immune
system and lie directly at the host-pathogen interface in targeting
specific microbe-molecular patterns. However, the direction and
strength of selection acting on TLRs are largely unknown for bats.
Here, we studied the selection on viral ssRNA sensing TLR8 based on
sequence data of 21 bat species. The major part (63 %) of the TLR8
gene evolved under purifying selection, likely due to functional
constraints. We also found evidence for persistent positive
selection acting on specific amino acid sites (7 %), especially when
compared to viral TLR evolution of other mammals. All of these
putatively positively selected codons were located in the ligand-
binding ectodomain, some coincidenced or were in close proximity to
functional sites, as suggested by the crystallographic structure of
the human TLR8. This might contribute to the inter-species variation
in the ability to recognize molecular patterns of viruses. TLR8
evolution within bats revealed that branches leading to ancestral
and recent lineages evolved under episodic positive selection,

indicating selective selection pressures in restricted bat lineages. Altogether, we found that the TLR8 displays extensive sequence variation within bats and that unique features separate them from humans and other mammals.
C1 [Schad, Julia; Voigt, Christian C.] Leibniz Inst Zoo & Wildlife Res, Dept Evolutionary Ecol, Alfred Kowalke Str 17, D-10315 Berlin, Germany.
C3 Leibniz Institut fur Zoo und Wildtierforschung
RP Schad, J (corresponding author), Leibniz Inst Zoo & Wildlife Res, Dept Evolutionary Ecol, Alfred Kowalke Str 17, D-10315 Berlin, Germany.
EM schad@izw-berlin.de
FU Deutsche Forschungsgemeinschaft [SPP1596, Vo890/23]
FX We thank several co-workers for the help in capturing bats in the field.
This work was supported by the Deutsche Forschungsgemeinschaft (priority
program SPP1596 (Vo890/23) to CCV).
CR Alcaide M, 2011, MOL BIOL EVOL, V28, P1703, DOI 10.1093/molbev/msq351
Alexopoulou L, 2001, NATURE, V413, P732, DOI 10.1038/35099560
Andersen-Nissen E, 2007, J EXP MED, V204, P393, DOI 10.1084/jem.20061400
Areal H, 2011, BMC EVOL BIOL, V11, DOI 10.1186/1471-2148-11-368
Ashkenazy H, 2010, NUCLEIC ACIDS RES, V38, pW529, DOI 10.1093/nar/gkq399
Atchley WR, 2005, P NATL ACAD SCI USA, V102, P6395, DOI 10.1073/pnas.0408677102
Babik W, 2015, GENOME BIOL EVOL, V7, P81, DOI 10.1093/gbe/evu266
Baker ML, 2013, ZOONOSES PUBLIC HLTH, V60, P104, DOI 10.1111/j.1863-2378.2012.01528.x
Bean AGD, 2013, NAT REV IMMUNOL, V13, P851, DOI 10.1038/nri3551
Biasini Marco, 2014, Nucleic Acids Res, V42, pW252, DOI 10.1093/nar/gku340
Brook CE, 2015, TRENDS MICROBIOL, V23, P172, DOI 10.1016/j.tim.2014.12.004
Calisher CH., 2015, Bats and Viruses: A New Frontier of Emerging Infectious Diseases, V1st, P23
Castel G, 2014, VIRUSES-BASEL, V6, P1112, DOI 10.3390/v6031112
Cowled C, 2011, DEV COMP IMMUNOL, V35, P7, DOI 10.1016/j.dci.2010.07.006
Darfour-Oduro KA, 2015, PLOS ONE, V10, DOI 10.1371/journal.pone.0124069
Daugherty MD, 2012, ANNU REV GENET, V46, P677, DOI 10.1146/annurev-genet-110711-155522
de Matos AL, 2013, PLOS ONE, V8, DOI 10.1371/journal.pone.0081864
DeLano W.L., 2002, PYMOL MOL GRAPH SYST
Delport W, 2010, BIOINFORMATICS, V26, P2455, DOI 10.1093/bioinformatics/btq429
Ellegren H, 2008, MOL ECOL, V17, P4586, DOI 10.1111/j.1365-294X.2008.03954.x
Escalera-Zamudio M, 2015, MOL ECOL, V24, P5899, DOI 10.1111/mec.13431
Fornusková A, 2013, BMC EVOL BIOL, V13, DOI

10.1186/1471-2148-13-194
Gannon WL, 2007, J MAMMAL, V88, P809, DOI 10.1644/06-MAMM-F-185R1.1
Gautam JK, 2006, J BIOL CHEM, V281, P30132, DOI 10.1074/jbc.M602057200
Georgel P, 2009, PLOS ONE, V4, DOI 10.1371/journal.pone.0007803
Geyer M, 2015, NAT STRUCT MOL BIOL, V22, P99, DOI 10.1038/nsmb.2967
Gorden KKB, 2006, J IMMUNOL, V177, P6584, DOI 10.4049/jimmunol.177.10.6584
Govindaraj EG, 2011, PLOS ONE, V6
Grueber CE, 2012, PLOS ONE, V7, DOI 10.1371/journal.pone.0045011
Hancks DC, 2015, PLOS GENET, V11, DOI 10.1371/journal.pgen.1005203
Hofmann K., 1993, BIOL CHEM HOPPESEYLE, V374, P166, DOI [DOI 10.1515/BCHM3.1993.374.1-6.143, DOI 10.1016/J.JTBI.2009.11.002]
Jensen S, 2012, J VIROL, V86, P2900, DOI 10.1128/JVI.05738-11
Jukes T.H., 1969, MAMMALIAN PROTEIN ME, P21, DOI DOI 10.1093/BIOINFORMATICS/BTM404
Kawai T, 2006, CELL DEATH DIFFER, V13, P816, DOI 10.1038/sj.cdd.4401850
Kawai T, 2010, NAT IMMUNOL, V11, P373, DOI 10.1038/ni.1863
Lien E, 2000, J CLIN INVEST, V105, P497, DOI 10.1172/JCI8541
Liu J, 2010, MOL IMMUNOL, V47, P1083, DOI 10.1016/j.molimm.2009.11.003
Lizundia R, 2008, INNATE IMMUN-LONDON, V14, P223, DOI 10.1177/1753425908095957
Lund JM, 2004, P NATL ACAD SCI USA, V101, P5598, DOI 10.1073/pnas.0400937101
Maldonado E, 2014, PLOS ONE, V9, DOI 10.1371/journal.pone.0096243
Mikami T, 2012, GENE, V503, P235, DOI 10.1016/j.gene.2012.04.007
Morozumi T, 2009, BBA-GEN SUBJECTS, V1790, P267, DOI 10.1016/j.bbagen.2009.01.002
Mukherjee S, 2009, P NATL ACAD SCI USA, V106, P7073, DOI 10.1073/pnas.0811357106
Murrell B, 2013, MOL BIOL EVOL, V30, P1196, DOI 10.1093/molbev/mst030
Murrell B, 2012, PLOS GENET, V8, DOI 10.1371/journal.pgen.1002764
NEI M, 1986, MOL BIOL EVOL, V3, P418
Offord V, 2010, DEV COMP IMMUNOL, V34, P1035, DOI 10.1016/j.dci.2010.05.004
Patin E, 2009, PLOS GENET, V5, DOI 10.1371/journal.pgen.1000448
Plowright RK, 2015, P ROY SOC B-BIOL SCI, V282, DOI 10.1098/rspb.2014.2124
Pond SLK, 2011, MOL BIOL EVOL, V28, P3033, DOI 10.1093/molbev/msr125
Pond SLK, 2005, MOL BIOL EVOL, V22, P1208, DOI 10.1093/molbev/msi105
Pond SLK, 2005, BIOINFORMATICS, V21, P676, DOI 10.1093/bioinformatics/bti079
Roach JC, 2005, P NATL ACAD SCI USA, V102, P9577, DOI 10.1073/pnas.0502272102
Schröder NW, 2005, LANCET INFECT DIS, V5, P156, DOI 10.1016/S1473-3099(05)70023-2

Schultz J, 1998, P NATL ACAD SCI USA, V95, P5857, DOI 10.1073/pnas.95.11.5857
Smith NGC, 2003, J MOL EVOL, V57, P467, DOI 10.1007/s00239-003-2500-z
Smith SA, 2012, BMC EVOL BIOL, V12, DOI 10.1186/1471-2148-12-122
Tamura K, 2013, MOL BIOL EVOL, V30, P2725, DOI [10.1093/molbev/mst197, 10.1093/molbev/msr121]
Tanji H, 2015, NAT STRUCT MOL BIOL, V22, P109, DOI 10.1038/nsmb.2943
Tanji H, 2013, SCIENCE, V339, P1426, DOI 10.1126/science.1229159
Taylor BD, 2012, J INFECT DIS, V205, P603, DOI 10.1093/infdis/jir822
Technelysium Pty Ltd, 1998, CHROM LIT 2 1 1
Tschirren B, 2011, J EVOLUTION BIOL, V24, P1232, DOI 10.1111/j.1420-9101.2011.02254.x
Tschirren B, 2013, P ROY SOC B-BIOL SCI, V280, DOI 10.1098/rspb.2013.0364
Uciechowski P, 2011, J LEUKOCYTE BIOL, V90, P377, DOI 10.1189/jlb.0409233
Vinkler M, 2014, GENET SEL EVOL, V46, DOI 10.1186/s12711-014-0072-6
Wang CM, 2014, SCI REP-UK, V4, DOI 10.1038/srep03792
Wang LF, 2011, CURR OPIN VIROL, V1, P649, DOI 10.1016/j.coviro.2011.10.013
Werling D, 2009, TRENDS IMMUNOL, V30, P124, DOI 10.1016/j.it.2008.12.001
Wibbelt G, 2010, BIOL LETTERS, V6, P438, DOI 10.1098/rsbl.2010.0267
Wlasiuk G, 2010, MOL BIOL EVOL, V27, P2172, DOI 10.1093/molbev/msq104
Wong SH, 2010, PLOS PATHOG, V6, DOI 10.1371/journal.ppat.1000979
Wynne JW, 2013, PLOS PATHOG, V9, DOI 10.1371/journal.ppat.1003651
Xagorari Angeliki, 2008, Open Microbiol J, V2, P49, DOI 10.2174/1874285800802010049
Yang ZH, 2005, MOL BIOL EVOL, V22, P1107, DOI 10.1093/molbev/msi097
Yang ZH, 2007, MOL BIOL EVOL, V24, P1586, DOI 10.1093/molbev/msm088
Zhang GJ, 2013, SCIENCE, V339, P456, DOI 10.1126/science.1230835
Zhu JZ, 2008, MOL IMMUNOL, V45, P3238, DOI 10.1016/j.molimm.2008.02.028
Zhu JZ, 2009, MOL IMMUNOL, V46, P978, DOI 10.1016/j.molimm.2008.09.024
NR 79
TC 18
Z9 21
U1 0
U2 28
PU SPRINGER
PI NEW YORK
PA ONE NEW YORK PLAZA, SUITE 4600, NEW YORK, NY, UNITED STATES
SN 0093-7711
EI 1432-1211
J9 IMMUNOGENETICS

JI Immunogenetics
PD NOV
PY 2016
VL 68
IS 10
BP 783
EP 795
DI 10.1007/s00251-016-0940-z
PG 13
WC Genetics & Heredity; Immunology
WE Science Citation Index Expanded (SCI-EXPANDED)
SC Genetics & Heredity; Immunology
GA DY5KI
UT WOS:000385138100004
PM 27502317
OA Green Published
DA 2024-10-21
ER

PT J
AU Dimitrov, DT
   Hallam, TG
   Rupprecht, CE
   McCracken, GF
AF Dimitrov, Dobromir T.
   Hallam, Thomas G.
   Rupprecht, Charles E.
   McCracken, Gary F.
TI Adaptive modeling of viral diseases in bats with a focus on
rabies
SO JOURNAL OF THEORETICAL BIOLOGY
LA English
DT Article
DE Immune system; Viral infection; Rabies; Individual heterogeneity;
   Disease processes and demographics; Bats
ID IMMUNE-RESPONSE; VIRUS-INFECTION; NORTH-AMERICA; FLYING-FOXES;
NIPAH
   VIRUS; NEW-MEXICO; CORONAVIRUSES; IMMUNOLOGY; RESERVOIRS; FUSCUS
AB Many emerging and reemerging viruses, such as rabies, SARS,
Marburg, and Ebola have bat populations as disease reservoirs.
Understanding the spillover from bats to humans and other animals,
and the associated health risks requires an analysis of the disease
dynamics in bat populations. Traditional compartmental epizootic
models, which are relatively easy to implement and analyze, usually
impose unrealistic aggregation assumptions about disease-related
structure and depend on parameters that frequently are not
measurable in field conditions. We propose a novel combination of
computational and adaptive modeling approaches that address the
maintenance of emerging diseases in bat colonies through individual
(intra-host) models of the response of the host to a viral
challenge. The dynamics of the individual models are used to define
survival, susceptibility and transmission conditions relevant to
epizootics as well as to develop and parametrize models of the
disease evolution into uniform and diverse populations. Applications

of the proposed approach to modeling the effects of immunological heterogeneity on the dynamics of bat rabies are presented. (C) 2008 Elsevier Ltd. All rights reserved.
C1 [Dimitrov, Dobromir T.; Hallam, Thomas G.; McCracken, Gary F.] Univ Tennessee, Dept Ecol & Evolutionary Biol, Knoxville, TN 37996 USA.
[Rupprecht, Charles E.] Ctr Dis Control & Prevent, Div Viral & Rickettsial Dis, Poxvirus & Rabies Branch, Atlanta, GA 30333 USA.
C3 University of Tennessee System; University of Tennessee Knoxville;
Centers for Disease Control & Prevention - USA
RP Dimitrov, DT (corresponding author), Fred Hutchinson Canc Res Ctr, Stat Ctr HIVAIDS Res & Prevent, 1100 Fairview Ave N,LE-400,POB 19024, Seattle, WA 98109 USA.
EM dobromir@scharp.org; thallam@utk.edu; cyr5@cdc.gov; gmccrack@utk.edu
RI Rupprecht, Charles/AAI-1544-2020
OI Dimitrov, Dobromir/0000-0002-2842-5436; McCracken, Gary/0000-0002-2493-8103
FU NSF/NIH-EID [043041]
FX This project is supported by NSF/NIH-EID Grant 043041. The authors thank
two anonymous referees for many useful comments on an earlier draft. The
findings and conclusions in this article are those of the authors and do
not necessarily represent the views of their Institutions.
CR Aguilar-Setien A, 2005, EPIDEMIOL INFECT, V133, P517, DOI 10.1017/S0950268805003705
[Anonymous], 2007, Rabies
Antia R, 1997, PARASITOLOGY, V115, pS155, DOI 10.1017/S003118209700200X
ASACHENKOV AL, 1994, IEEE T BIO-MED ENG, V41, P943, DOI 10.1109/10.324526
Bona CA., 1996, Textbook of Immunology, V2nd
Brass D.A., 1994, RABIES BATS NATURAL
Breed AC, 2006, BIOL CONSERV, V131, P211, DOI 10.1016/j.biocon.2006.04.007
BURNS KF, 1955, J INFECT DIS, V97, P211, DOI 10.1093/infdis/97.2.211
CHOWDHURY D, 1993, J THEOR BIOL, V165, P135, DOI 10.1006/jtbi.1993.1180
Chua KB, 2002, MICROBES INFECT, V4, P145, DOI 10.1016/S1286-4579(01)01522-2
Clark Donald R. Jr, 2001, P159
Constantine D.G., 1988, P491
Constantine D.G., 1967, U NEW MEXICO PUBLICA, V7, P1
CONSTANTINE DG, 1968, PUBLIC HEALTH REP, V83, P303, DOI 10.2307/4593275
CONSTANTINE DG, 1979, J WILDLIFE DIS, V15, P347, DOI 10.7589/0090-3558-15.2.347
DEAN W D, 1960, Ariz Med, V17, P69
Dimitrov DT, 2007, J THEOR BIOL, V245, P498, DOI 10.1016/j.jtbi.2006.11.001

DIMITROV DT, 2008, MATH BIOSCI IN PRESS
Dominguez SR, 2007, EMERG INFECT DIS, V13, P1295, DOI 10.3201/eid1309.070491
Echevarría JE, 2001, J CLIN MICROBIOL, V39, P3678, DOI 10.1128/JCM.39.10.3678-3683.2001
GIRARD KF, 1965, NEW ENGL J MED, V272, P75, DOI 10.1056/NEJM196501142720204
Hooper DC, 1998, J VIROL, V72, P3711, DOI 10.1128/JVI.72.5.3711-3719.1998
Jackson FR, 2008, J WILDLIFE DIS, V44, P612, DOI 10.7589/0090-3558-44.3.612
Johara MY, 2001, EMERG INFECT DIS, V7, P439
KAUFMAN M, 1987, J THEOR BIOL, V129, P141, DOI 10.1016/S0022-5193(87)80009-7
KAUFMAN M, 1985, J THEOR BIOL, V114, P527, DOI 10.1016/S0022-5193(85)80042-4
Lau SKP, 2005, P NATL ACAD SCI USA, V102, P14040, DOI 10.1073/pnas.0506735102
Leroy EM, 2005, NATURE, V438, P575, DOI 10.1038/438575a
Li WD, 2005, SCIENCE, V310, P676, DOI 10.1126/science.1118391
Poon LLM, 2005, J VIROL, V79, P2001, DOI 10.1128/JVI.79.4.2001-2009.2005
PYBUS MJ, 1986, J WILDLIFE DIS, V22, P449, DOI 10.7589/0090-3558-22.3.449
Shankar V, 2004, J WILDLIFE DIS, V40, P403, DOI 10.7589/0090-3558-40.3.403
STEECE R, 1989, J WILDLIFE DIS, V25, P490, DOI 10.7589/0090-3558-25.4.490
Swanepoel R, 2007, EMERG INFECT DIS, V13, P1847, DOI 10.3201/eid1312.071115
NR 34
TC 10
Z9 13
U1 1
U2 21
PU ACADEMIC PRESS LTD- ELSEVIER SCIENCE LTD
PI LONDON
PA 24-28 OVAL RD, LONDON NW1 7DX, ENGLAND
SN 0022-5193
EI 1095-8541
J9 J THEOR BIOL
JI J. Theor. Biol.
PD NOV 7
PY 2008
VL 255
IS 1
BP 69
EP 80
DI 10.1016/j.jtbi.2008.08.007
PG 12
WC Biology; Mathematical & Computational Biology
WE Science Citation Index Expanded (SCI-EXPANDED)
SC Life Sciences & Biomedicine - Other Topics; Mathematical & Computational

Biology
GA 364TF
UT WOS:000260357500008
PM 18761020
OA Green Accepted, Green Published
DA 2024-10-21
ER

PT J
AU Xiao, YH
Wu, YH
Sun, KP
Wang, H
Zhang, B
Song, SH
Du, ZL
Jiang, TL
Shi, LM
Wang, L
Lin, AQ
Yue, XK
Li, CJ
Chen, TT
Feng, J
AF Xiao, Yanhong
Wu, Yonghua
Sun, Keping
Wang, Hui
Zhang, Bing
Song, Shuhui
Du, Zhenglin
Jiang, Tinglei
Shi, Limin
Wang, Lei
Lin, Aiqing
Yue, Xinke
Li, Chenji
Chen, Tingting
Feng, Jiang
TI Differential Expression of Hepatic Genes of the Greater Horseshoe Bat
(<i>Rhinolophus ferrumequinum</i>) between the Summer Active and Winter
Torpid States
SO PLOS ONE
LA English
DT Article
ID HIBERNATING GROUND-SQUIRRELS; RNA-SEQ DATA; BURROWS-WHEELER TRANSFORM;
BEARS URSUS-AMERICANUS; MAMMALIAN HIBERNATION; LIPID-METABOLISM;
SEASONAL-CHANGES; READ ALIGNMENT; IMMUNE-SYSTEM; LIVER
AB Hibernation is one type of torpor, a hypometabolic state in heterothermic mammals, which can be used as an energy-conservation strategy in response to harsh environments, e.g. limited food

resource. The liver, in particular, plays a crucial role in adaptive metabolic adjustment during hibernation. Studies on ground squirrels and bears reveal that many genes involved in metabolism are differentially expressed during hibernation. Especially, the genes involved in carbohydrate catabolism are down-regulated during hibernation, while genes responsible for lipid beta-oxidation are up-regulated. However, there is little transcriptional evidence to suggest physiological changes to the liver during hibernation in the greater horseshoe bat, a representative heterothermic bat. In this study, we explored the transcriptional changes in the livers of active and torpid greater horseshoe bats using the Illumina HiSeq 2000 platform. A total of 1358 genes were identified as differentially expressed during torpor. In the functional analyses, differentially expressed genes were mainly involved in metabolic depression, shifts in the fuel utilization, immune function and response to stresses. Our findings provide a comprehensive evidence of differential gene expression in the livers of greater horseshoe bats during active and torpid states and highlight potential evidence for physiological adaptations that occur in the liver during hibernation.
C1 [Xiao, Yanhong; Sun, Keping; Wang, Hui; Jiang, Tinglei; Wang, Lei; Lin, Aiqing; Yue, Xinke; Feng, Jiang] NE Normal Univ, Jilin Prov Key Lab Anim Resource Conservat & Util, Changchun, Peoples R China.
[Xiao, Yanhong; Sun, Keping; Wang, Hui; Jiang, Tinglei; Wang, Lei; Lin, Aiqing; Yue, Xinke; Feng, Jiang] NE Normal Univ, Key Lab Wetland Ecol & Vegetat Restorat Natl Envi, Changchun, Peoples R China.
[Wu, Yonghua] NE Normal Univ, Sch Life Sci, Changchun, Peoples R China.
[Zhang, Bing; Song, Shuhui; Du, Zhenglin; Li, Chenji; Chen, Tingting] Chinese Acad Sci, Beijing Inst Genom, Core Genom Facil, Beijing, Peoples R China.
[Shi, Limin] Yunnan Normal Univ, Sch Life Sci, Kunming, Peoples R China.
C3 Northeast Normal University - China; Northeast Normal University -
China; Northeast Normal University - China; Chinese Academy of Sciences;
Beijing Institute of Genomics, CAS; Yunnan Normal University
RP Wu, YH (corresponding author), NE Normal Univ, Sch Life Sci, Changchun, Peoples R China.
EM wuyh442@nenu.edu.cn; fengj@nenu.edu.cn
RI shi, li/HSF-9608-2023; song, shuhui/D-6015-2014; sun, ke/KMX-1265-2024;
Zhu, Li/GWV-0143-2022; Jiang, Tinglei/GQQ-9485-2022
FU National Natural Science Foundation of China [91131003, 31270414]
FX This work was funded by the National Natural Science Foundation of
China. The grant numbers were 91131003
(http://isisn.nsfc.gov.cn/egrantindex/funcindex/prjsearch-list) and
31270414
(http://isisn.nsfc.gov.cn/egrantindex/funcindex/prjsearch-list).

The
funders had no role in study design, data collection and analysis,
decision to publish, or preparation of the manuscript.
CR AHLQUIST DA, 1984, J COMP PHYSIOL B, V155, P75, DOI 10.1007/BF00688794
Andrews MT, 2004, BIOCHEM SOC T, V32, P1021, DOI 10.1042/BST0321021
[Anonymous], 2002, International Wildlife Encyclopedia
[Anonymous], ELS
[Anonymous], 2003, HORSESHOE BATS WORLD
Artavanis-Tsakonas S, 1999, SCIENCE, V284, P770, DOI 10.1126/science.284.5415.770
ATANASSOV CL, 1995, COMP BIOCHEM PHYS C, V112, P93, DOI 10.1016/0742-8413(95)00000-3
BENJAMINI Y, 1995, J R STAT SOC B, V57, P289, DOI 10.1111/j.2517-6161.1995.tb02031.x
Blehert DS, 2009, SCIENCE, V323, P227, DOI 10.1126/science.1163874
Boss O, 1997, FEBS LETT, V412, P111, DOI 10.1016/S0014-5793(97)00755-2
Bouma HR, 2010, J LEUKOCYTE BIOL, V88, P619, DOI 10.1189/jlb.0310174
Carey HV, 2003, PHYSIOL REV, V83, P1153, DOI 10.1152/physrev.00008.2003
Chang LF, 2001, NATURE, V410, P37, DOI 10.1038/35065000
Chen JP, 2008, COMP BIOCHEM PHYS B, V149, P388, DOI 10.1016/j.cbpb.2007.10.011
Dave KR, 2012, COMP BIOCHEM PHYS B, V162, P1, DOI 10.1016/j.cbpb.2012.01.008
Diano S, 2012, TRENDS MOL MED, V18, P52, DOI 10.1016/j.molmed.2011.08.003
Fedorov VB, 2011, BMC GENOMICS, V12, DOI 10.1186/1471-2164-12-171
Fedorov VB, 2009, PHYSIOL GENOMICS, V37, P108, DOI 10.1152/physiolgenomics.90398.2008
Feng JX, 2012, BIOINFORMATICS, V28, P2782, DOI 10.1093/bioinformatics/bts515
GALLETTI G, 1972, ACTA ANAT, V83, P593
Gao B, 2008, HEPATOLOGY, V47, P729, DOI 10.1002/hep.22034
Geiser F, 2013, CURR BIOL, V23, pR188, DOI 10.1016/j.cub.2013.01.062
Gobin S, 2003, J BIOL CHEM, V278, P50428, DOI 10.1074/jbc.M310130200
Hampton M, 2011, PLOS ONE, V6, DOI 10.1371/journal.pone.0027021
Harris MA, 2004, NUCLEIC ACIDS RES, V32, pD258, DOI 10.1093/nar/gkh036
Hegardt FG, 1999, BIOCHEM J, V338, P569, DOI 10.1042/0264-6021:3380569
HOFMANN GE, 1995, J EXP BIOL, V198, P1509
Huang Y, 2010, BIOINFORMATICS, V26, P680, DOI 10.1093/bioinformatics/btq003
Jolly C, 2000, JNCI-J NATL CANCER I, V92, P1564, DOI 10.1093/jnci/92.19.1564
Kandror O, 1997, P NATL ACAD SCI USA, V94, P4978, DOI 10.1073/

pnas.94.10.4978
Kanehisa M, 2000, NUCLEIC ACIDS RES, V28, P27, DOI 10.1093/nar/28.1.27
Kurtz CC, 2007, DEV COMP IMMUNOL, V31, P415, DOI 10.1016/j.dci.2006.07.003
Lei M, 2014, PLOS ONE, V9, DOI 10.1371/journal.pone.0107746
Li H, 2010, BIOINFORMATICS, V26, P589, DOI 10.1093/bioinformatics/btp698
Li H, 2009, BIOINFORMATICS, V25, P1754, DOI 10.1093/bioinformatics/btp324
Livak KJ, 2001, METHODS, V25, P402, DOI 10.1006/meth.2001.1262
Louet JF, 2001, BIOCHEM SOC T, V29, P310, DOI 10.1042/0300-5127:0290310
Moore MS, 2013, PLOS ONE, V8, DOI 10.1371/journal.pone.0058976
Mortazavi A, 2008, NAT METHODS, V5, P621, DOI 10.1038/nmeth.1226
Nguyen P, 2008, J ANIM PHYSIOL AN N, V92, P272, DOI 10.1111/j.1439-0396.2007.00752.x
Novoselova E, 2004, DOKLADY BIOL SCI
Nowak R. M., 1999, WALKERS MAMMALS WORL
Park KJ, 2000, FUNCT ECOL, V14, P580, DOI 10.1046/j.1365-2435.2000.t01-1-00460.x
Piechotta K, 2002, J BIOL CHEM, V277, P50812, DOI 10.1074/jbc.M208108200
Prendergast BJ, 2002, AM J PHYSIOL-REG I, V282, pR1054, DOI 10.1152/ajpregu.00562.2001
Racanelli V, 2006, HEPATOLOGY, V43, pS54, DOI 10.1002/hep.21060
Ransome RD., 1990, NATURAL HIST HIBERNA, p235 pp
Robertson G, 2010, NAT METHODS, V7, P909, DOI 10.1038/nmeth.1517
Roots C., 2006, Hibernation
Rowles J, 1996, J BIOL CHEM, V271, P22376, DOI 10.1074/jbc.271.37.22376
Ruf T, 2015, BIOL REV, V90, P891, DOI 10.1111/brv.12137
Seim I, 2013, NAT COMMUN, V4, DOI 10.1038/ncomms3212
Shimozuru M, 2012, COMP BIOCHEM PHYS B, V163, P254, DOI 10.1016/j.cbpb.2012.06.007
Shivatcheva T, 1987, FOLIA BIOL-PRAGUE, V36, P213
Smith-Unna R.D., 2015, BIORXIV
Sonna LA, 2002, J APPL PHYSIOL, V92, P1725, DOI 10.1152/japplphysiol.01143.2001
South F.E., 1967, Mammalian Hibernation III, P305
SRERE HK, 1992, P NATL ACAD SCI USA, V89, P7119, DOI 10.1073/pnas.89.15.7119
Storey KB, 2004, BIOL REV, V79, P207, DOI 10.1017/S1464793103006195
van Breukelen F, 2015, PHYSIOLOGY, V30, P273, DOI 10.1152/physiol.00010.2015
vanBreukelen F, 2002, J COMP PHYSIOL B, V172, P269, DOI 10.1007/s00360-002-0252-5
Wang L.C. H., 1996, Handbook of physiology: environmental physiology
Wang LK, 2010, BIOINFORMATICS, V26, P136, DOI 10.1093/bioinformatics/btp612
Williams DR, 2005, PHYSIOL GENOMICS, V24, P13, DOI 10.1152/physiolgenomics.00301.2004

   Yan J, 2008, PHYSIOL GENOMICS, V32, P170, DOI 10.1152/
physiolgenomics.00075.2007
   Yarden Y, 2001, NAT REV MOL CELL BIO, V2, P127, DOI
10.1038/35052073
   Zhao WM, 2011, BIOINFORMATICS, V27, P3076, DOI 10.1093/
bioinformatics/btr504
   Zhou HJ, 2004, ARCH BIOCHEM BIOPHYS, V422, P23, DOI 10.1016/
j.abb.2003.11.019
NR 68
TC 16
Z9 19
U1 1
U2 44
PU PUBLIC LIBRARY SCIENCE
PI SAN FRANCISCO
PA 1160 BATTERY STREET, STE 100, SAN FRANCISCO, CA 94111 USA
SN 1932-6203
J9 PLOS ONE
JI PLoS One
PD DEC 23
PY 2015
VL 10
IS 12
AR e0145702
DI 10.1371/journal.pone.0145702
PG 21
WC Multidisciplinary Sciences
WE Science Citation Index Expanded (SCI-EXPANDED)
SC Science & Technology - Other Topics
GA CZ4SI
UT WOS:000367092600121
PM 26698122
OA Green Submitted, gold, Green Published
DA 2024-10-21
ER

PT J
AU Hernández-Arciga, U
   Herrera, MLG
   Ibáñez-Contreras, A
   Miranda-Labra, RU
   Flores-Martínez, JJ
   Königsberg, M
AF Hernandez-Arciga, Ulalume
   Gerardo Herrera, L. M.
   Ibanez-Contreras, Alejandra
   Miranda-Labra, Roxana U.
   Juan Flores-Martinez, Jose
   Konigsberg, Mina
TI Baseline and post-stress seasonal changes in immunocompetence and
redox
   state maintenance in the fishing bat <i>Myotis vivesi</i>
SO PLOS ONE
LA English

DT Article
ID STRESS-INDUCED ENHANCEMENT; ANTIOXIDANT DEFENSE ENZYMES; SQUIRREL CITELLUS-CITELLUS; OXIDATIVE STRESS; IMMUNE-RESPONSE; ECOLOGICAL IMMUNOLOGY; FREE-RADICALS; COLD-STRESS; LEUKOCYTE TRAFFICKING; SUPEROXIDE-DISMUTASE
AB Little is known of how the stress response varies when animals confront seasonal life-history processes. Antioxidant defenses and damage caused by oxidative stress and their link with immunocompetence are powerful biomarkers to assess animal A s physiological stress response. The aim of this study was A) to determine redox state and variation in basal (preacute stress) immune function during summer, autumn and winter (spring was not assessed due to restrictions in collecting permit) in the fish-eating Myotis (Myotis vivesi; Chiroptera), and B) to determine the effect of acute stress on immunocompetence and redox state during each season. Acute stress was stimulated by restricting animal movement for 6 and 12 h. The magnitude of the cellular immune response was higher during winter whilst that of the humoral response was at its highest during summer. Humoral response increased after 6 h of movement restriction stress and returned to baseline levels after 12 h. Basal redox state was maintained throughout the year, with no significant changes in protein damage, and antioxidant activity was modulated mainly in relation to variation to environment cues, increasing during high temperatures and decreasing during windy nights. Antioxidant activity increased after the 6 h of stressful stimuli especially during summer and autumn, and to a lesser extent in early winter, but redox state did not vary. However, protein damage increased after 12 h of stress during summer. Prolonged stress when the bat is engaged in activities of high energy demand overcame its capacity to maintain homeostasis resulting in oxidative damage.
C1 [Hernandez-Arciga, Ulalume] Univ Nacl Autonoma Mexico, Inst Biol, Posgrad Ciencias Biol, Ciudad De Mexico, Mexico.
[Hernandez-Arciga, Ulalume; Konigsberg, Mina] Univ Autonoma Metropolitana, Unidad Iztapalapa, Dept Ciencias Salud, Lab Bioenerget & Envejecimiento Celular, Ciudad De Mexico, Mexico.
[Gerardo Herrera, L. M.] Univ Nacl Autonoma Mexico, Inst Biol, Estn Biol Chamela, San Patricio, Jalisco, Mexico.
[Ibanez-Contreras, Alejandra] Appl Res Expt Biomed SA CV APREXBIO, Lab Neurofisiol, Ciudad De Mexico, Mexico.
[Ibanez-Contreras, Alejandra] Biol Integral Vertebrados BIOINVERT, Unidad Experimentac Anim, Mexico City, Estado De Mexic, Mexico.
[Miranda-Labra, Roxana U.] Univ Autonoma Metropolitana, Unidad Iztapalapa, Dept Ciencias Salud, Lab Fisiol Celular, Ciudad De Mexico, Mexico.
[Juan Flores-Martinez, Jose] Univ Nacl Autonoma Mexico, Dept Zool, Inst Biol, Lab Sistemas Informac Geog, Ciudad De Mexico, Mexico.
C3 Universidad Nacional Autonoma de Mexico; Universidad Autonoma Metropolitana - Mexico; Universidad Nacional Autonoma de Mexico; Universidad Autonoma Metropolitana - Mexico; Universidad Nacional Autonoma de Mexico
RP Hernández-Arciga, U (corresponding author), Univ Nacl Autonoma

Mexico, Inst Biol, Posgrad Ciencias Biol, Ciudad De Mexico, Mexico.;
Hernández-Arciga, U; Königsberg, M (corresponding author), Univ
Autonoma Metropolitana, Unidad Iztapalapa, Dept Ciencias Salud, Lab
Bioenerget & Envejecimiento Celular, Ciudad De Mexico, Mexico.
EM herarc_ula9@hotmail.com; mkf@xanum.uam.mx
OI Herrera Montalvo, Luis Gerardo/0000-0001-9348-5040
FU Consejo Nacional de Ciencia y Tecnologoia (CONACyT) [23774];
CONACyT
Fronteras en la Ciencia grant [FON.INST/298/2016]; Biologia
Integral
para Vertebrados (BIOINVERT(R)); Posgrado en Ciencias Bioloagicas
UNAM
FX This work was supported by a grant (# 23774) given by Consejo
Nacional
de Ciencia y Tecnologoia (CONACyT) to LGHM, partially supported
by
CONACyT Fronteras en la Ciencia grant FON.INST/298/2016 to MKF.
UHA
acknowledges the scholarship provided by CONACyT. Alejandra
IbaAnez-Contreras is employed by Unidad de Experimentacioan
Animal,
Biologia Integral para Vertebrados (BIOINVERT (R)). Unidad de
Experimentacioan Animal, Biologia Integral para Vertebrados
(BIOINVERT
(R)) provided support in the form of salary for author AI-C, but
did not
have any additional role in the study design, data collection and
analysis, decision to publish, or preparation of the manuscript.
The
specific role of this author is articulated in the author
contributions'
section. UHA acknowledges the scholarship provided by CONACyT and
the
support of the Posgrado en Ciencias Bioloagicas UNAM. This paper
constitutes a partial fulfillment of the Graduate Program in
Biological
Science of the UNAM. Transport to Partida Norte Island was
generously
provided by the Secretaria de Marina Armada de Mexico. Kenneth C.
Welch,
Jr, provided constructive comments on previous drafts of the
manuscript.
The Prescott College Kino Bay Center provided invaluable logistic
support during fieldwork. Rodrigo Monjaraz Ruedas, Diego
Alejandro
Barrales Alcala A, Viridiana Gonzales Meneses, and Eric Moreno
for their
valuable assistance during fieldwork.
CR Acevedo-Whitehouse K, 2009, PHILOS T R SOC B, V364, P3429, DOI
10.1098/rstb.2009.0128
AEBI H, 1984, METHOD ENZYMOL, V105, P121
Ahmad S., 1989, ARCH INSECT BIOCH PH, V31-49, P49
Amutha C., 2012, American Journal of Animal and Veterinary
Sciences, V7, P96

Apanius V, 1998, ADV STUD BEHAV, V27, P133
Arriero E, 2009, OECOLOGIA, V159, P697, DOI 10.1007/s00442-008-1253-1
Beckman KB, 1998, PHYSIOL REV, V78, P547, DOI 10.1152/physrev.1998.78.2.547
Bijlsma R, 2005, J EVOLUTION BIOL, V18, P744, DOI 10.1111/j.1420-9101.2005.00962.x
Bilbo SD, 2002, P NATL ACAD SCI USA, V99, P4067, DOI 10.1073/pnas.062001899
Bílková B, 2015, J EXP ZOOL PART A, V323, P767, DOI 10.1002/jez.1990
Blagojevic Dusko, 1998, Journal of Environmental Pathology Toxicology and Oncology, V17, P241
Bohonak AJ, 2004, NO TITLESOFTWARE RED
Bourgeon S, 2009, GEN COMP ENDOCR, V163, P77, DOI 10.1016/j.ygcen.2008.11.015
Bowden TJ, 2008, FISH SHELLFISH IMMUN, V25, P373, DOI 10.1016/j.fsi.2008.03.017
BRADFORD MM, 1976, ANAL BIOCHEM, V72, P248, DOI 10.1016/0003-2697(76)90527-3
Brunet-Rossinni Anja K., 2009, P315
Buehler DM, 2008, AM NAT, V172, P783, DOI 10.1086/592865
Buzadzic B, 1997, COMP BIOCHEM PHYS C, V117, P141, DOI 10.1016/S0742-8413(97)00061-3
BUZADZIC B, 1992, COMP BIOCHEM PHYS B, V101, P547, DOI 10.1016/0305-0491(92)90336-P
Carey HV, 2003, PHYSIOL REV, V83, P1153, DOI 10.1152/physrev.00008.2003
CARPENTER RE, 1968, COMP BIOCHEM PHYSIOL, V24, P951, DOI 10.1016/0010-406X(68)90807-4
Carreno Ana Luisa, 2002, P14
Chen WH, 2002, GEN COMP ENDOCR, V126, P90, DOI 10.1006/gcen.2001.7772
Christe P, 2000, ECOL LETT, V3, P207
Cichon M, 2002, P ROY SOC B-BIOL SCI, V269, P1493, DOI 10.1098/rspb.2002.2054
Conde-Pérezprina JC, 2012, AGE, V34, P1473, DOI 10.1007/s11357-012-9399-5
Costantini D, 2009, COMP BIOCHEM PHYS A, V153, P339, DOI 10.1016/j.cbpa.2009.03.010
Dabbert CB, 1997, AUK, V114, P103, DOI 10.2307/4089069
DAVIES KJA, 1982, BIOCHEM BIOPH RES CO, V107, P1198, DOI 10.1016/S0006-291X(82)80124-1
de Neve L, 2007, IBIS, V149, P763, DOI 10.1111/j.1474-919X.2007.00708.x
Demas GE, 2011, J ANIM ECOL, V80, P710, DOI 10.1111/j.1365-2656.2011.01813.x
Dhabhar FS, 2013, CLIN DERMATOL, V31, P18, DOI 10.1016/j.clindermatol.2011.11.003
Dhabhar FS, 2005, AM J PHYSIOL-REG I, V289, pR738, DOI 10.1152/ajpregu.00145.2005
Dhabhar FS, 1999, P NATL ACAD SCI USA, V96, P1059, DOI 10.1073/pnas.96.3.1059
Dhabhar FS, 2000, P NATL ACAD SCI USA, V97, P2846, DOI 10.1073/

pnas.050569397
Dhabhar FS, 1996, J IMMUNOL, V156, P2608
Dröge W, 2002, PHYSIOL REV, V82, P47, DOI 10.1152/physrev.00018.2001
ESSER AF, 1994, TOXICOLOGY, V87, P229, DOI 10.1016/0300-483X(94)90253-4
Finkel T, 2000, NATURE, V408, P239, DOI 10.1038/35041687
Fletcher Quinn E, 2013, Evolution, V67, P1527, DOI 10.1111/evo.12014
FLOHE L, 1984, METHOD ENZYMOL, V105, P114
Flores J, 2005, CULT CRIT, P187
Fontella F, 2005, NEUROCHEM RES, V30, P105, DOI 10.1007/s11064-004-9691-6
GERSCHMAN R, 1954, SCIENCE, V119, P623, DOI 10.1126/science.119.3097.623
GITTLEMAN JL, 1988, AM ZOOL, V28, P863, DOI 10.1093/icb/28.3.863
GODIN DV, 1992, COMP BIOCHEM PHYS B, V103, P737, DOI 10.1016/0305-0491(92)90399-C
Hangalapura BN, 2005, POULTRY SCI, V84, P1520, DOI 10.1093/ps/84.10.1520
Hangalapura BN, 2003, POULTRY SCI, V82, P1692, DOI 10.1093/ps/82.11.1692
Harper JM, 2013, PHYSIOL BIOCHEM ZOOL, V73, P12
Hau M, 2001, HORM BEHAV, V40, P281, DOI 10.1006/hbeh.2001.1673
Hawlena H, 2008, COMP BIOCHEM PHYS A, V150, P66, DOI 10.1016/j.cbpa.2008.03.004
Hopkins WA, 2011, GEN COMP ENDOCR, V174, P107, DOI 10.1016/j.ygcen.2011.08.006
Huyghe K, 2010, SEASONAL CHANGES PAR, P867
Isaksson C, 2011, BIOSCIENCE, V61, P194, DOI 10.1525/bio.2011.61.3.5
Jakob EM, 1996, OIKOS, V77, P61, DOI 10.2307/3545585
Kamhawi S, 2000, SCIENCE, V290, P1351, DOI 10.1126/science.290.5495.1351
Kennedy MW, 2006, TRENDS ECOL EVOL, V21, P653, DOI 10.1016/j.tree.2006.09.017
Knight JA, 2000, ANN CLIN LAB SCI, V30, P145
Konisberg-Fainstein M., 2008, RADICALES LIBRES EST, P1
Koziorowska-Gilun M, 2011, REPROD BIOL, V11, P37, DOI 10.1016/S1642-431X(12)60062-X
Liebl AL, 2009, FUNCT ECOL, V23, P1091, DOI 10.1111/j.1365-2435.2009.01592.x
Lifjeld JT, 2002, OECOLOGIA, V130, P185, DOI 10.1007/s004420100798
LOCHMILLER RL, 1993, AUK, V110, P503, DOI 10.2307/4088414
LOCHMILLER RL, 1994, ECOLOGY, V75, P236, DOI 10.2307/1939397
Martin LB, 2006, FUNCT ECOL, V20, P290, DOI 10.1111/j.1365-2435.2006.01094.x
Martin LB, 2005, GEN COMP ENDOCR, V140, P126, DOI 10.1016/j.ygcen.2004.10.010
Martin LB, 2006, INTEGR COMP BIOL, V46, P1030, DOI 10.1093/icb/icl039
Matson KD, 2006, P ROY SOC B-BIOL SCI, V273, P815, DOI 10.1098/rspb.2005.3376

Matson KD, 2006, PHYSIOL BIOCHEM ZOOL, V79, P556, DOI 10.1086/501057
Maya J., 1968, The natural history of the fish-eating bat, Pizonyx vivesi
McGraw KJ, 2010, FUNCT ECOL, V24, P947, DOI 10.1111/j.1365-2435.2010.01772.x
McMichael LA, 2014, ECOHEALTH, V11, P400, DOI 10.1007/s10393-014-0954-7
Mecocci P, 1999, FREE RADICAL BIO MED, V26, P303, DOI 10.1016/S0891-5849(98)00208-1
Merchant ME, 2003, COMP BIOCHEM PHYS B, V136, P505, DOI 10.1016/S1096-4959(03)00256-2
Merlo JL, 2014, CAN J ZOOL, V92, P689, DOI 10.1139/cjz-2013-0306
Merlo JL, 2014, COMP BIOCHEM PHYS A, V175, P90, DOI 10.1016/j.cbpa.2014.05.021
Mesquita CS, 2014, ANAL BIOCHEM, V458, P69, DOI 10.1016/j.ab.2014.04.034
Milsom WK, 1999, COMP BIOCHEM PHYS A, V124, P383, DOI 10.1016/S1095-6433(99)00130-0
Montes I, 2011, J ZOOL, V284, P114, DOI 10.1111/j.1469-7998.2010.00787.x
NAKANISHI T, 1986, VET IMMUNOL IMMUNOP, V12, P213, DOI 10.1016/0165-2427(86)90125-X
Neeman E, 2012, BRAIN BEHAV IMMUN, V26, P267, DOI 10.1016/j.bbi.2011.09.007
Nelson RJ, 1996, Q REV BIOL, V71, P511, DOI 10.1086/419555
Nelson RJ, 2004, TRENDS IMMUNOL, V25, P187, DOI 10.1016/j.it.2004.02.001
Nordahl EA, 2004, P NATL ACAD SCI USA, V101, P16879, DOI 10.1073/pnas.0406678101
Ochsenbein AF, 1999, SCIENCE, V286, P2156, DOI 10.1126/science.286.5447.2156
Otálora-Ardila A, 2016, PLOS ONE, V11, DOI 10.1371/journal.pone.0164938
Otalora-Ardila A, 2013, J MAMMAL, V94, P1102, DOI 10.1644/12-MAMM-A-281.1
Packer L, 2001, J NUTR, V131, p369S, DOI 10.1093/jn/131.2.369S
PAOLETTI F, 1986, ANAL BIOCHEM, V154, P536, DOI 10.1016/0003-2697(86)90026-6
Pavlovic SZ, 2010, MAR DRUGS, V8, P413, DOI 10.3390/md8030413
Peig J, 2009, OIKOS, V118, P1883, DOI 10.1111/j.1600-0706.2009.17643.x
Pigeon G, 2013, ECOL EVOL, V3, P1091, DOI 10.1002/ece3.504
Radovanovic TB, 2010, ARCH BIOL SCI, V62, P97, DOI 10.2298/ABS1001097R
Raja-aho S, 2012, PHYSIOL BIOCHEM ZOOL, V85, P148, DOI 10.1086/664826
Raouf SA, 2006, ANIM BEHAV, V71, P39, DOI 10.1016/j.anbehav.2005.03.027
Reeder DM, 2004, GEN COMP ENDOCR, V136, P260, DOI 10.1016/j.ygcen.2003.12.020
Reth M, 2002, NAT IMMUNOL, V3, P1129, DOI 10.1038/ni1202-1129
Rhee SG., 2013, SCIENCE, V312, p[2, 103]
Romero LM, 2008, AM J PHYSIOL-REG I, V294, pR614, DOI 10.1152/

ajpregu.00752.2007
Romero ML., 2007, INT J COMP PSYCHOL, V20, P89, DOI [10.46867/IJCP.2007.20.02.15, DOI 10.46867/IJCP.2007.20.02.15]
Salinas VBR, 2014, ACTA CHIROPTEROL, V16, P327, DOI 10.3161/150811014X687288
Sapolsky RM, 2000, ENDOCR REV, V21, P55, DOI 10.1210/er.21.1.55
SELSTED ME, 1978, INFECT IMMUN, V20, P782, DOI 10.1128/IAI.20.3.782-791.1978
Sheldon BC, 1996, TRENDS ECOL EVOL, V11, P317, DOI 10.1016/0169-5347(96)10039-2
Sorci G, 2009, PHILOS T R SOC B, V364, P71, DOI 10.1098/rstb.2008.0151
Speakman JR, 2008, PHILOS T R SOC B, V363, P375, DOI 10.1098/rstb.2007.2145
Storey KB, 2010, GERONTOLOGY, V56, P220, DOI 10.1159/000228829
Strobel S, 2015, MAMM BIOL, V80, P312, DOI 10.1016/j.mambio.2015.02.005
Turmelle AS, 2010, J COMP PHYSIOL B, V180, P1155, DOI 10.1007/s00360-010-0486-6
TYLER DD, 1975, FEBS LETT, V51, P180, DOI 10.1016/0014-5793(75)80882-9
Vinkler M, 2012, ANIM BEHAV, V83, P17, DOI 10.1016/j.anbehav.2011.10.001
Welch KC Jr, 2015, J EXP BIOL, V218, P1180, DOI 10.1242/jeb.115964
Wikelski M, 2006, TRENDS ECOL EVOL, V21, P38, DOI 10.1016/j.tree.2005.10.018
Wikelski M, 2003, P ROY SOC B-BIOL SCI, V270, P2383, DOI 10.1098/rspb.2003.2500
Wingfield JC, 2003, ANIM BEHAV, V66, P807, DOI 10.1006/anbe.2003.2298
Yang DB, 2013, J EXP BIOL, V216, P4242, DOI 10.1242/jeb.092049
Zhang ZQ, 2015, INTEGR ZOOL, V10, P302, DOI 10.1111/1749-4877.12133
NR 117
TC 19
Z9 21
U1 0
U2 13
PU PUBLIC LIBRARY SCIENCE
PI SAN FRANCISCO
PA 1160 BATTERY STREET, STE 100, SAN FRANCISCO, CA 94111 USA
SN 1932-6203
J9 PLOS ONE
JI PLoS One
PD JAN 2
PY 2018
VL 13
IS 1
AR e0190047
DI 10.1371/journal.pone.0190047
PG 27
WC Multidisciplinary Sciences
WE Science Citation Index Expanded (SCI-EXPANDED)

SC Science & Technology - Other Topics
GA FR5JA
UT WOS:000419101600056
PM 29293551
OA Green Submitted, Green Published, gold
DA 2024-10-21
ER

PT J
AU Cockrell, C
   An, G
AF Cockrell, Chase
   An, Gary
TI Comparative Computational Modeling of the Bat and Human Immune Response
   to Viral Infection with the Comparative Biology Immune Agent Based Model
SO VIRUSES-BASEL
LA English
DT Article
DE comparative biology; computational biology; mathematical modeling;
   innate immunity; bats; viral tolerance; agent based model; inflammasome;
   zoonotic transfer; viral pandemic; COVID-19
ID MECHANISMS; GAMMA; INFLAMMASOMES; INFLAMMATION; RESERVOIRS; DISEASES;
   SCIENCE; VIRUS; IL-10; FOCUS
AB Given the impact of pandemics due to viruses of bat origin, there is increasing interest in comparative investigation into the differences between bat and human immune responses. The practice of comparative biology can be enhanced by computational methods used for dynamic knowledge representation to visualize and interrogate the putative differences between the two systems. We present an agent based model that encompasses and bridges differences between bat and human responses to viral infection: the comparative biology immune agent based model, or CBIABM. The CBIABM examines differences in innate immune mechanisms between bats and humans, specifically regarding inflammasome activity and type 1 interferon dynamics, in terms of tolerance to viral infection. Simulation experiments with the CBIABM demonstrate the efficacy of bat-related features in conferring viral tolerance and also suggest a crucial role for endothelial inflammasome activity as a mechanism for bat systemic viral tolerance and affecting the severity of disease in human viral infections. We hope that this initial study will inspire additional comparative modeling projects to link, compare, and contrast immunological functions shared across different species, and in so doing, provide insight and aid in preparation for future viral pandemics of zoonotic origin.
C1 [Cockrell, Chase; An, Gary] Univ Vermont, Dept Surg, Larner Coll Med, Burlington, VT 05405 USA.
C3 University of Vermont
RP An, G (corresponding author), Univ Vermont, Dept Surg, Larner Coll Med, Burlington, VT 05405 USA.

EM Robert.cockrell@med.uvm.edu; gan@med.uvm.edu
OI An, Gary/0000-0003-4549-9004
FU NATIONAL INSTITUTES OF HEALTH [UO1EB025825]; NIH Office of the Director;
National Institute of Biomedical Imaging and Bioengineering [U01EB025825] Funding Source: NIH RePORTER
FX This research was funded by the NATIONAL INSTITUTES OF HEALTH, grant
number UO1EB025825.
CR Abegglen LM, 2015, JAMA-J AM MED ASSOC, V314, P1850, DOI 10.1001/jama.2015.13134
An G, 2004, CRIT CARE MED, V32, P2050, DOI 10.1097/01.CCM.0000139707.13729.7D
An G, 2001, SHOCK, V16, P266, DOI 10.1097/00024382-200116040-00006
An G., 2021, COMPLEX SYSTEMS COMP, P71
An G, 2018, B MATH BIOL, V80, P3071, DOI 10.1007/s11538-018-0497-0
An Gary, 2009, V500, P445, DOI 10.1007/978-1-59745-525-1_15
An G, 2008, THEOR BIOL MED MODEL, V5, DOI 10.1186/1742-4682-5-11
Banerjee A, 2020, FRONT IMMUNOL, V11, DOI 10.3389/fimmu.2020.00026
Bertheloot D, 2021, CELL MOL IMMUNOL, V18, P1106, DOI 10.1038/s41423-020-00630-3
Bonabeau E, 2002, P NATL ACAD SCI USA, V99, P7280, DOI 10.1073/pnas.082080899
BOX GEP, 1976, J AM STAT ASSOC, V71, P791, DOI 10.2307/2286841
Brandstadter JD, 2011, J INNATE IMMUN, V3, P274, DOI 10.1159/000324176
Brook CE, 2020, ELIFE, V9, DOI 10.7554/eLife.48401
Chen K, 2017, J AUTOIMMUN, V83, P1, DOI 10.1016/j.jaut.2017.03.008
Cockrell C, 2021, FRONT PHYSIOL, V12, DOI 10.3389/fphys.2021.662845
Cockrell C, 2017, J THEOR BIOL, V430, P157, DOI 10.1016/j.jtbi.2017.07.016
Cockrell RC, 2018, PLOS COMPUT BIOL, V14, DOI 10.1371/journal.pcbi.1005876
Couper KN, 2008, J IMMUNOL, V180, P5771, DOI 10.4049/jimmunol.180.9.5771
Vaccari JCD, 2020, FRONT IMMUNOL, V11, DOI 10.3389/fimmu.2020.583373
Dimitrov DT, 2008, J THEOR BIOL, V255, P69, DOI 10.1016/j.jtbi.2008.08.007
Dimitrov DT, 2007, J THEOR BIOL, V245, P498, DOI 10.1016/j.jtbi.2006.11.001
Dimitrov DT, 2008, MATH BIOSCI ENG, V5, P729, DOI 10.3934/mbe.2008.5.729
Dinarello CA, 2013, FRONT IMMUNOL, V4, DOI 10.3389/fimmu.2013.00289
Falasca L, 2015, CELL DEATH DIFFER, V22, P1250, DOI 10.1038/cdd.2015.67
Gentles AD, 2020, PATHOG GLOB HEALTH, V114, P407, DOI 10.1080/20477724.2020.1833161

Goh G, 2020, P NATL ACAD SCI USA, V117, P28939, DOI 10.1073/pnas.2003352117
Goldenberg NM, 2011, SCI TRANSL MED, V3, DOI 10.1126/scitranslmed.3002011
Guo HT, 2015, NAT MED, V21, P677, DOI 10.1038/nm.3893
Han HJ, 2015, VIRUS RES, V205, P1, DOI 10.1016/j.virusres.2015.05.006
Hu BY, 2021, J MED VIROL, V93, P250, DOI 10.1002/jmv.26232
Iba T, 2020, CRIT CARE MED, V48, P1358, DOI 10.1097/CCM.0000000000004458
Irving AT, 2021, NATURE, V589, P363, DOI 10.1038/s41586-020-03128-0
Jafarzadeh A, 2020, LIFE SCI, V257, DOI 10.1016/j.lfs.2020.118102
Kesavardhana S, 2020, ANNU REV IMMUNOL, V38, P567, DOI 10.1146/annurev-immunol-073119-095439
Lee JJ, 2019, BIOMECH MODEL MECHAN, V18, P1297, DOI 10.1007/s10237-019-01145-1
Leroy EM, 2005, NATURE, V438, P575, DOI 10.1038/438575a
Liu Q, 2016, CELL MOL IMMUNOL, V13, P3, DOI 10.1038/cmi.2015.74
Martín-Sánchez F, 2016, CELL DEATH DIFFER, V23, P1219, DOI 10.1038/cdd.2015.176
Metzcar J, 2019, JCO CLIN CANCER INFO, V3, DOI 10.1200/CCI.18.00069
Mortaz E, 2018, FRONT IMMUNOL, V9, DOI 10.3389/fimmu.2018.02171
Murira A, 2016, FRONT IMMUNOL, V7, DOI 10.3389/fimmu.2016.00609
Nguyen KB, 2002, J IMMUNOL, V169, P4279, DOI 10.4049/jimmunol.169.8.4279
Petersen BK, 2019, J COMPUT BIOL, V26, P597, DOI 10.1089/cmb.2018.0168
Prescott SM, 2000, ANNU REV BIOCHEM, V69, P419, DOI 10.1146/annurev.biochem.69.1.419
Price GE, 2000, J VIROL, V74, P3996, DOI 10.1128/JVI.74.9.3996-4003.2000
Saraiva M, 2010, NAT REV IMMUNOL, V10, P170, DOI 10.1038/nri2711
Schmidt ME, 2018, FRONT IMMUNOL, V9, DOI 10.3389/fimmu.2018.00678
Schroder K, 2004, J LEUKOCYTE BIOL, V75, P163, DOI 10.1189/jlb.0603252
Segovia-Juarez JL, 2004, J THEOR BIOL, V231, P357, DOI 10.1016/j.jtbi.2004.06.031
Smallwood R, 2006, I S BIOMED IMAGING, P816
Srikiatkhachorn A, 2017, SEMIN IMMUNOPATHOL, V39, P563, DOI 10.1007/s00281-017-0625-1
Subudhi S, 2019, VIRUSES-BASEL, V11, DOI 10.3390/v11020192
Sulak M, 2016, ELIFE, V5, DOI [10.7554/eLife.11994, 10.7554/eLife.24307]
Tanaka T, 2012, INT J BIOL SCI, V8, P1227, DOI 10.7150/ijbs.4666
Tisue S., 2004, INT C COMPL SYST, V21, P16
Velazquez-Salinas L, 2019, FRONT MICROBIOL, V10, DOI 10.3389/fmicb.2019.01057
Walpole J, 2017, MICROCIRCULATION, V24, DOI 10.1111/micc.12393
Wu C, 2014, J IMMUNOL, V193, P3036, DOI 10.4049/jimmunol.1302379
Yap JKY, 2020, J IMMUNOL, V205, P307, DOI 10.4049/jimmunol.2000513
Zent CS, 2017, FEBS J, V284, P1021, DOI 10.1111/febs.13961

Zheng DP, 2020, CELL DISCOV, V6, DOI 10.1038/s41421-020-0167-x
NR 61
TC 8
Z9 10
U1 1
U2 6
PU MDPI
PI BASEL
PA ST ALBAN-ANLAGE 66, CH-4052 BASEL, SWITZERLAND
EI 1999-4915
J9 VIRUSES-BASEL
JI Viruses-Basel
PD AUG
PY 2021
VL 13
IS 8
AR 1620
DI 10.3390/v13081620
PG 24
WC Virology
WE Science Citation Index Expanded (SCI-EXPANDED)
SC Virology
GA UI0AQ
UT WOS:000690282000001
PM 34452484
OA Green Submitted, Green Published, gold
DA 2024-10-21
ER

PT J
AU Iha, K
Omatsu, T
Watanabe, S
Ueda, N
Taniguchi, S
Fujii, H
Ishii, Y
Kyuwa, S
Akashi, H
Yoshikawa, Y
AF Iha, Koichiro
Omatsu, Tsutomu
Watanabe, Shumpei
Ueda, Naoya
Taniguchi, Satoshi
Fujii, Hikaru
Ishii, Yoshiyuki
Kyuwa, Shigeru
Akashi, Hiroomi
Yoshikawa, Yasuhiro
TI Molecular Cloning and Sequencing of the cDNAs Encoding the Bat
Interleukin (IL)-2, IL-4, IL-6, IL40, IL-12p40, and Tumor
Necrosis
Factor-Alpha

SO JOURNAL OF VETERINARY MEDICAL SCIENCE
LA English
DT Article
DE chiroptera; cytokine; molecular cloning; natural reservoir; zoonoses
ID CELL STIMULATORY FACTOR; IMMUNE-RESPONSE; VIRUS-INFECTION; CYTOKINE;
EXPRESSION; RESERVOIR
AB This is the first report on the cDNA sequences of bat interleukin (IL)-2, IL-4, IL-6, IL-10, IL-12 p40, and tumor necrosis factor (TNF)-alpha. The cDNAs of bat IL-2 IL-4, IL-6, IL-10, IL-12 p40, and TNF-alpha comprise 459, 405, 624, 537, 990, and 699 base pairs respectively. Moreover, each of the cDNAs of bat IL-2, IL-4, IL-6, IL-10, IL-12 p40, and TNF-alpha contain a single open reading frames encoding 152, 134, 207, 178, 329, and 232 amino acids, respectively. The comparison of bat cytokines with Perrissodactyla (horse), Carnivora (dog and cat), and Cetartiodactyla (cattle and pig) orthologs revealed a high degree of homology. Although the N-terminal amino acids and cysteine residues are highly conserved in each mature cytokine, the deduced N-linked glycosylation sites vary across species.
C1 [Iha, Koichiro; Ueda, Naoya; Taniguchi, Satoshi; Ishii, Yoshiyuki; Kyuwa, Shigeru; Yoshikawa, Yasuhiro] Univ Tokyo, Dept Biomed Sci, Grad Sch Agr & Life Sci, Bunkyo Ku, Tokyo 1138657, Japan.
[Omatsu, Tsutomu] Univ Tokyo, Dept Vet Microbiol, Grad Sch Agr & Life Sci, Bunkyo Ku, Tokyo 1138657, Japan.
[Watanabe, Shumpei; Fujii, Hikaru; Akashi, Hiroomi] Natl Inst Infect Dis, Lab Vector Borne Viruses, Shinjuku Ku, Tokyo 1628650, Japan.
C3 University of Tokyo; University of Tokyo; National Institute of Infectious Diseases (NIID)
RP Iha, K (corresponding author), Univ Tokyo, Dept Biomed Sci, Grad Sch Agr & Life Sci, Bunkyo Ku, 1-1-1 Yayoi, Tokyo 1138657, Japan.
EM aa097127@mail.ecc.u-tokyo.ac.jp
RI Omatsu, Tsutomu/C-8344-2013
FU Ministry of Agriculture, Forestry and Fisheries, Japan; Ministry of
Health, Labour and Welfare, Japan
FX This study was supported in part by grants from the Ministry of Agriculture, Forestry and Fisheries, Japan, and the Ministry of Health,
Labour and Welfare, Japan. We thank Maiko Endo for her technical expertise in maintaining animals.
CR Calisher CH, 2006, CLIN MICROBIOL REV, V19, P531, DOI 10.1128/CMR.00017-06
CHAKRABORTY AK, 1984, DEV COMP IMMUNOL, V8, P415, DOI 10.1016/0145-305X(84)90048-X
CHAKRAVARTY AK, 1987, DEV COMP IMMUNOL, V11, P649, DOI 10.1016/0145-305X(87)90053-X
Davis A, 2005, AM J TROP MED HYG, V73, P467, DOI 10.4269/ajtmh.2005.73.467
HATTEN BA, 1968, J IMMUNOL, V101, P141
HIRANO T, 1986, NATURE, V324, P73, DOI 10.1038/324073a0

Hymowitz SG, 2000, BIOCHEMISTRY-US, V39, P633, DOI 10.1021/bi992242l
Kidd Parris, 2003, Altern Med Rev, V8, P223
Lam J, 2001, J CLIN INVEST, V108, P971, DOI 10.1172/JCI200113890
Middleton DJ, 2007, J COMP PATHOL, V136, P266, DOI 10.1016/j.jcpa.2007.03.002
Nishihara H, 2006, P NATL ACAD SCI USA, V103, P9929, DOI 10.1073/pnas.0603797103
Odbileg R, 2005, VET IMMUNOL IMMUNOP, V104, P145, DOI 10.1016/j.vetimm.2004.11.002
Odbileg R, 2004, VET IMMUNOL IMMUNOP, V102, P91, DOI 10.1016/j.vetimm.2004.06.002
Omatsu T, 2007, COMP IMMUNOL MICROB, V30, P357, DOI 10.1016/j.cimid.2007.05.006
PAUL BN, 1986, DEV COMP IMMUNOL, V10, P55, DOI 10.1016/0145-305X(86)90044-3
Swanepoel R, 1996, EMERG INFECT DIS, V2, P321, DOI 10.3201/eid0204.960407
TANIGUCHI T, 1983, NATURE, V302, P305, DOI 10.1038/302305a0
VIEIRA P, 1991, P NATL ACAD SCI USA, V88, P1172, DOI 10.1073/pnas.88.4.1172
Williamson MM, 2000, J COMP PATHOL, V122, P201, DOI 10.1053/jcpa.1999.0364
WOLF SF, 1991, J IMMUNOL, V146, P3074
Woolhouse MEJ, 2005, EMERG INFECT DIS, V11, P1842, DOI 10.3201/eid1112.050997
YOKOTA T, 1986, P NATL ACAD SCI USA, V83, P5894, DOI 10.1073/pnas.83.16.5894
NR 22
TC 8
Z9 12
U1 0
U2 3
PU JAPAN SOC VET SCI
PI TOKYO
PA UNIV TOKYO, 1-1-1 YAYOI, BUNKYO-KU, TOKYO, 103, JAPAN
SN 0916-7250
EI 1347-7439
J9 J VET MED SCI
JI J. Vet. Med. Sci.
PD DEC
PY 2009
VL 71
IS 12
BP 1691
EP 1695
DI 10.1292/jvms.001691
PG 5
WC Veterinary Sciences
WE Science Citation Index Expanded (SCI-EXPANDED)
SC Veterinary Sciences
GA 546SH
UT WOS:000273831100024
PM 20046044

OA hybrid
DA 2024-10-21
ER

PT J
AU Li, XL
   Liu, T
   Li, AQ
   Xiao, YH
   Sun, KP
   Feng, J
AF Li, Xiaolin
   Liu, Tong
   Li, Aoqiang
   Xiao, Yanhong
   Sun, Keping
   Feng, Jiang
TI Diversifying selection and climatic effects on major histocompatibility
   complex class II gene diversity in the greater horseshoe bat
SO EVOLUTIONARY APPLICATIONS
LA English
DT Article
DE bat; climatic effect; evolution; MHC; selection
ID MHC CLASS-I; COPY NUMBER VARIATION; RHINOLOPHUS-FERRUMEQUINUM; BALANCING
   SELECTION; TRANSSPECIES POLYMORPHISM; POPULATION-STRUCTURE; LOCAL
   ADAPTATION; PATTERNS; ADVANTAGE; DRIFT
AB Heterogeneous pathogenic stress can shape major histocompatibility complex (MHC) diversity by influencing the functional plasticity of the immune response. Therefore, MHC diversity could reflect environmental stress, demonstrating its importance in uncovering the mechanisms of adaptive genetic variation. In this study, we combined neutral microsatellite loci, an immune-related MHC II-DRB locus, and climatic factors to unravel the mechanisms affecting the diversity and genetic differentiation of MHC genes in the greater horseshoe bat (Rhinolophus ferrumequinum), a species with a wide geographical distribution that has three distinct genetic lineages in China. First, increased genetic differentiation at the MHC locus among populations compared using microsatellites indicated diversifying selection. Second, the genetic differentiation of MHC and microsatellites were significantly correlated, suggesting that demographic processes exist. However, MHC genetic differentiation was significantly correlated with geographical distance among populations, even after controlling for the neutral markers, suggesting a major effect of selection. Third, although the MHC genetic differentiation was larger than that for microsatellites, there was no significant difference in the genetic differentiation between the two markers among genetic lineages, indicating the effect of balancing selection. Fourth, combined with climatic factors, MHC diversity and supertypes showed significant correlations with temperature and precipitation, but not with the phylogeographic structure of R. ferrumequinum, suggesting an effect of local adaptation driven by

climate on MHC diversity. Moreover, the number of MHC supertypes varied between populations and lineages, suggesting regional characteristics and support for local adaptation. Taken together, the results of our study provide insights into the adaptive evolutionary driving forces at different geographic scales in R. ferrumequinum. In addition, climate factors may have played a vital role in driving adaptive evolution in this species.
C1 [Li, Xiaolin; Li, Aoqiang; Xiao, Yanhong; Sun, Keping; Feng, Jiang] Northeast Normal Univ, Jilin Prov Key Lab Anim Resource Conservat & Utili, Changchun, Peoples R China.
[Li, Xiaolin; Sun, Keping] Minist Educ, Key Lab Vegetat Ecol, Changchun, Peoples R China.
[Liu, Tong; Feng, Jiang] Jilin Agr Univ, Coll Life Sci, Changchun, Peoples R China.
[Sun, Keping; Feng, Jiang] Northeast Normal Univ, Jilin Prov Key Lab Anim Resource Conservat & Utili, Changchun 130117, Peoples R China.
C3 Northeast Normal University - China; Jilin Agricultural University;
Northeast Normal University - China
RP Sun, KP; Feng, J (corresponding author), Northeast Normal Univ, Jilin Prov Key Lab Anim Resource Conservat & Utili, Changchun 130117, Peoples R China.
EM sunkp129@nenu.edu.cn; fengj@nenu.edu.cn
RI sun, ke/KMX-1265-2024; Li, Xiaolin/O-5795-2015
FU National Natural Science Foundation of China [31570390, 31670390, 31770403, 31961123001]
FX National Natural Science Foundation of China, Grant/Award Number: 31570390, 31670390, 31770403 and 31961123001
CR Adams RI, 2013, EVOL ECOL, V27, P133, DOI 10.1007/s10682-012-9587-x
Agudo R, 2011, MOL ECOL, V20, P2329, DOI 10.1111/j.1365-294X.2011.05107.x
Alcaide M, 2014, ECOL EVOL, V4, P688, DOI 10.1002/ece3.974
[Anonymous], 2017, RASTER GEOGRAPHIC DA
[Anonymous], 2016, SPADER SPECIES PREDI
[Anonymous], 1991, Molecular Evolution of the Major Histocompatibility Complex
[Anonymous], 2015, ZOONOSES INFECT AFFE
[Anonymous], 2017, Geographic Distance Matrix Generator (version 1.2.3)
Awadi A, 2018, SCI REP-UK, V8, DOI 10.1038/s41598-018-29657-3
Baker CS, 2006, IMMUNOGENETICS, V58, P283, DOI 10.1007/s00251-006-0080-y
Björklund M, 2015, ECOL EVOL, V5, P1440, DOI 10.1002/ece3.1426
Brunner FS, 2016, ZOOLOGY, V119, P384, DOI 10.1016/j.zool.2016.04.001
Darriba D, 2012, NAT METHODS, V9, P772, DOI 10.1038/nmeth.2109
Del Real-Monroy M, 2014, ACTA CHIROPTEROL, V16, P301, DOI 10.3161/150811014X687260
Dionne M, 2007, EVOLUTION, V61, P2154, DOI 10.1111/j.1558-5646.2007.00178.x
Dixon P, 2003, J VEG SCI, V14, P927, DOI 10.1111/j.1654-1103.2003.tb02228.x

Doherty P C, 1975, Nature, V256, P50
Dubey J.P., 1988, P1
Eimes JA, 2011, J EVOLUTION BIOL, V24, P1847, DOI 10.1111/j.1420-9101.2011.02311.x
Ekblom R, 2007, MOL ECOL, V16, P1439, DOI 10.1111/j.1365-294X.2007.03281.x
Ellegren H, 2016, NAT REV GENET, V17, P422, DOI 10.1038/nrg.2016.58
Excoffier L, 2010, MOL ECOL RESOUR, V10, P564, DOI 10.1111/j.1755-0998.2010.02847.x
Flanders J, 2011, J BIOGEOGR, V38, P439, DOI 10.1111/j.1365-2699.2010.02411.x
Flanders J, 2009, MOL ECOL, V18, P306, DOI 10.1111/j.1365-294X.2008.04021.x
Froeschke G, 2014, EVOL ECOL, V28, P1169, DOI 10.1007/s10682-014-9731-x
Futuyma D. J., 2009, EVOLUTION, V49
Garrigan D, 2003, EVOLUTION, V57, P1707
Goslee SC, 2007, J STAT SOFTW, V22, P1, DOI 10.18637/jss.v022.i07
Goudet J, 2005, MOL ECOL NOTES, V5, P184, DOI 10.1111/j.1471-8286.2004.00828.x
Goudet J, 1995, J HERED, V86, P485, DOI 10.1093/oxfordjournals.jhered.a111627
Graser R, 1996, IMMUNOGENETICS, V44, P36, DOI 10.1007/BF02602655
Grunsky EC, 2002, COMPUT GEOSCI-UK, V28, P1219, DOI 10.1016/S0098-3004(02)00034-1
Hammer Oyvind, 2001, Palaeontologia Electronica, V4, pUnpaginated
Herdegen M, 2014, J EVOLUTION BIOL, V27, P2347, DOI 10.1111/jeb.12476
Hoyt JR, 2020, P NATL ACAD SCI USA, V117, P7255, DOI 10.1073/pnas.1914794117
Hoyt JR, 2016, P ROY SOC B-BIOL SCI, V283, DOI 10.1098/rspb.2015.2861
Huelsenbeck JP, 2001, BIOINFORMATICS, V17, P754, DOI 10.1093/bioinformatics/17.8.754
Hughes Austin L., 1998, Frontiers in Bioscience, V3, pD509
Jacobs DS, 2017, PLOS ONE, V12, DOI 10.1371/journal.pone.0187769
Jaratlerdsiri W, 2014, IMMUNOGENETICS, V66, P53, DOI 10.1007/s00251-013-0746-1
Jiang TL, 2015, INTEGR ZOOL, V10, P241, DOI 10.1111/1749-4877.12129
Johnson JA, 2016, ANIM CONSERV, V19, P97, DOI 10.1111/acv.12273
Jombart T, 2010, BMC GENET, V11, DOI 10.1186/1471-2156-11-94
Jost L, 2018, EVOL APPL, V11, P1139, DOI 10.1111/eva.12590
Jukes T.H., 1969, MAMMALIAN PROTEIN ME, P21, DOI DOI 10.1093/BIOINFORMATICS/BTM404
Jurasinski G, 2012, R Package Version 0.3-5
Karger DN, 2017, SCI DATA, V4, DOI 10.1038/sdata.2017.122
Kaufman J, 2018, ANNU REV IMMUNOL, V36, P383, DOI 10.1146/annurev-immunol-051116-052450
Kim YM, 2017, COMMUN STAT APPL MET, V24, P1, DOI 10.5351/CSAM.2017.24.1.001
KLEIN D, 1993, NATURE, V364, P330, DOI 10.1038/364330a0
Klein J, 1998, ANNU REV ECOL SYST, V29, P1, DOI 10.1146/

annurev.ecolsys.29.1.1
Kumar S, 2016, MOL BIOL EVOL, V33, P1870, DOI [10.1093/molbev/msw054, 10.1093/molbev/msv279]
Kyle CJ, 2014, MOL ECOL, V23, P2287, DOI 10.1111/mec.12726
Landry C, 2001, MOL ECOL, V10, P2525, DOI 10.1046/j.1365-294X.2001.01383.x
Leigh JW, 2015, METHODS ECOL EVOL, V6, P1110, DOI 10.1111/2041-210X.12410
Lenz TL, 2013, EVOLUTION, V67, P2400, DOI 10.1111/evo.12124
Li AQ, 2022, ENVIRON MICROBIOL, V24, P1484, DOI 10.1111/1462-2920.15754
Li J, 2016, J ZOOL, V300, P197, DOI 10.1111/jzo.12373
Li XL, 2021, J ZOOL SYST EVOL RES, V59, P887, DOI 10.1111/jzs.12462
Li YM, 2021, ECOGRAPHY, V44, P206, DOI 10.1111/ecog.05082
Lichstein JW, 2007, PLANT ECOL, V188, P117, DOI 10.1007/s11258-006-9126-3
Lighten J, 2014, MOL ECOL RESOUR, V14, P753, DOI 10.1111/1755-0998.12225
Loiseau C, 2009, MOL ECOL, V18, P1331, DOI 10.1111/j.1365-294X.2009.04105.x
Magoc T, 2011, BIOINFORMATICS, V27, P2957, DOI 10.1093/bioinformatics/btr507
Martin DP, 2017, METHODS MOL BIOL, V1525, P433, DOI 10.1007/978-1-4939-6622-6_17
Matthews B, 2010, PLOS ONE, V5, DOI 10.1371/journal.pone.0010948
Meyer-Lucht Y, 2016, CONSERV GENET, V17, P615, DOI 10.1007/s10592-016-0810-0
Meyers LA, 2002, TRENDS ECOL EVOL, V17, P551, DOI 10.1016/S0169-5347(02)02633-2
Miller HC, 2010, MOL ECOL, V19, P3894, DOI 10.1111/j.1365-294X.2010.04771.x
Miller KM, 2001, GENETICA, V111, P237, DOI 10.1023/A:1013716020351
Millstein J, 2022, BIOINFORM ADV, V2, DOI 10.1093/bioadv/vbac047
Moreno-Santillán DD, 2016, PLOS ONE, V11, DOI 10.1371/journal.pone.0141296
Murray GGR, 2013, P ROY SOC B-BIOL SCI, V280, DOI 10.1098/rspb.2013.0965
Murrell B, 2013, MOL BIOL EVOL, V30, P1196, DOI 10.1093/molbev/mst030
Murrell B, 2012, PLOS GENET, V8, DOI 10.1371/journal.pgen.1002764
Mutumi GL, 2016, PLOS ONE, V11, DOI 10.1371/journal.pone.0148053
NEI M, 1986, MOL BIOL EVOL, V3, P418
Niskanen AK, 2014, MOL ECOL, V23, P875, DOI 10.1111/mec.12647
Otting N, 2005, P NATL ACAD SCI USA, V102, P1626, DOI 10.1073/pnas.0409084102
Peakall R, 2012, BIOINFORMATICS, V28, P2537, DOI 10.1093/bioinformatics/bts460
Pearson SK, 2018, MOL ECOL, V27, P2204, DOI 10.1111/mec.14571
Phillips KP, 2018, P NATL ACAD SCI USA, V115, P1552, DOI 10.1073/pnas.1708597115
Piertney SB, 2006, HEREDITY, V96, P7, DOI 10.1038/sj.hdy.6800724
Pond SLK, 2006, BIOINFORMATICS, V22, P3096, DOI 10.1093/

bioinformatics/btl474
Pond SLK, 2005, MOL BIOL EVOL, V22, P1208, DOI 10.1093/molbev/msi105
Pritchard JK, 2000, GENETICS, V155, P945
Qin SY, 2014, PARASITE VECTOR, V7, DOI 10.1186/s13071-014-0558-7
Qurkhuli T, 2019, IMMUNOGENETICS, V71, P575, DOI 10.1007/s00251-019-01128-7
Rekdal SL, 2018, ECOL EVOL, V8, P1680, DOI 10.1002/ece3.3757
Richman AD, 2010, INT J IMMUNOGENET, V37, P401, DOI 10.1111/j.1744-313X.2010.00941.x
Rossiter SJ, 2002, BEHAV ECOL SOCIOBIOL, V51, P510, DOI 10.1007/s00265-002-0467-1
Rozas J, 2017, MOL BIOL EVOL, V34, P3299, DOI 10.1093/molbev/msx248
Sagonas K, 2019, HEREDITY, V122, P468, DOI 10.1038/s41437-018-0146-2
Salmier A, 2016, BMC EVOL BIOL, V16, DOI 10.1186/s12862-016-0802-1
Sandberg M, 1998, J MED CHEM, V41, P2481, DOI 10.1021/jm9700575
Savage AE, 2016, P ROY SOC B-BIOL SCI, V283, DOI 10.1098/rspb.2015.3115
Savage AE, 2011, P NATL ACAD SCI USA, V108, P16705, DOI 10.1073/pnas.1106893108
Schad J, 2012, PLOS ONE, V7, DOI 10.1371/journal.pone.0037101
Schad J, 2012, IMMUNOGENETICS, V64, P535, DOI 10.1007/s00251-012-0609-1
Sebastian A, 2016, MOL ECOL RESOUR, V16, P498, DOI 10.1111/1755-0998.12453
Sepil I, 2013, P ROY SOC B-BIOL SCI, V280, DOI 10.1098/rspb.2013.0134
Siddle HV, 2010, P ROY SOC B-BIOL SCI, V277, P2001, DOI 10.1098/rspb.2009.2362
SLADE RW, 1992, GENETICS, V132, P861
Smallbone W, 2021, MOL ECOL, V30, P736, DOI 10.1111/mec.15763
Sommer Simone, 2005, Frontiers in Zoology, V2, P1, DOI 10.1186/1742-9994-2-16
Spurgin LG, 2010, P ROY SOC B-BIOL SCI, V277, P979, DOI 10.1098/rspb.2009.2084
Stefanovic M, 2021, BMC ECOL EVOL, V21, DOI 10.1186/s12862-021-01856-z
Strand TM, 2012, ECOL EVOL, V2, P341, DOI 10.1002/ece3.86
Stutz WE, 2014, PLOS ONE, V9, DOI 10.1371/journal.pone.0100587
Sun KP, 2013, PLOS ONE, V8, DOI 10.1371/journal.pone.0070368
Symonds M. R. E., 2014, MODERN PHYLOGENETIC, P105, DOI [DOI 10.1007/978-3-662-43550-2_5, 10.1007/978-3-662-43550-2_5, DOI 10.1007/978-3-662-43550-25]
Talarico L, 2019, HEREDITY, V123, P593, DOI 10.1038/s41437-019-0221-3
Tesicky M, 2015, J IMMUNOL RES, V2015, DOI 10.1155/2015/838035
Trachtenberg E, 2003, NAT MED, V9, P928, DOI 10.1038/nm893
Trujillo AL, 2021, HEREDITY, V126, P640, DOI 10.1038/s41437-020-00402-9
van Tienderen PH, 2002, TRENDS ECOL EVOL, V17, P577, DOI 10.1016/S0169-5347(02)02624-1

   Vlcek J, 2016, MOL ECOL, V25, P4757, DOI 10.1111/mec.13807
   Wang SP, 2017, MOL ECOL, V26, P4671, DOI 10.1111/mec.14242
   Weaver S, 2018, MOL BIOL EVOL, V35, P773, DOI 10.1093/molbev/msx335
   Wegner KM, 2008, BMC EVOL BIOL, V8, DOI 10.1186/1471-2148-8-124
   Weir B.S., 1997, BIOMETRICS VOL, V53, P392, DOI DOI 10.2307/2533134
   Yi XL, 2020, ECOL EVOL, V10, P10031, DOI 10.1002/ece3.6662
   Zeisset I, 2014, PLOS ONE, V9, DOI 10.1371/journal.pone.0100176
   Zhang L, 2018, ZOOL SCR, V47, P655, DOI 10.1111/zsc.12308
   Zhao HB, 2019, FRONT ZOOL, V16, DOI 10.1186/s12983-019-0336-7
NR 125
TC 3
Z9 4
U1 4
U2 17
PU WILEY
PI HOBOKEN
PA 111 RIVER ST, HOBOKEN 07030-5774, NJ USA
SN 1752-4571
J9 EVOL APPL
JI Evol. Appl.
PD MAR
PY 2023
VL 16
IS 3
BP 688
EP 704
DI 10.1111/eva.13528
EA JAN 2023
PG 17
WC Evolutionary Biology
WE Science Citation Index Expanded (SCI-EXPANDED)
SC Evolutionary Biology
GA 9Z6IT
UT WOS:000910879700001
PM 36969140
OA Green Published, gold
DA 2024-10-21
ER

PT J
AU Bianco, C
   Baker, KS
   Pazzini, L
   Cafiso, A
   Suu-Ire, RD
   Cunningham, AA
   Wood, JLN
   Nuñez, A
AF Bianco, Carlo
   Baker, Kate S.
   Pazzini, Luca
   Cafiso, Alessandra

Suu-Ire, Richard D.
Cunningham, Andrew A.
Wood, James L. N.
Nunez, Alejandro
TI Demodicosis in a captive African straw-coloured fruit bat (Eidolon helvum)
SO EXPERIMENTAL AND APPLIED ACAROLOGY
LA English
DT Article
DE Bat; Demodex; Dermatology; Veterinary pathology; Wildlife diseases
ID DEMODICIDAE; ACARI; IDENTIFICATION
AB Demodicosis is most frequently observed in the domestic dog (Canis familiaris), but it has rarely been reported in bats (Chiroptera). The overpopulation of Demodex spp. that causes dermatological changes is generally associated with a compromised immune system. We describe the gross and histological features of generalized demodicosis in an adult female African straw-coloured fruit bat (Eidolon helvum) drawn from a captive research colony. The histology of the lesions revealed comedones and follicular infundubular cysts harbouring numerous Demodex spp. mites, eliciting a minimal inflammatory response in the adjacent dermis. The histological examination of a full set of tissues did not reveal clear evidence of immunosuppression, although a clinical history of recent abortion and possible stressors due to captivity could be considered risk factors for the demodicosis. Attempts to determine the Demodex species using PCR on DNA extracted from the formalin fixed paraffin embedded tissue failed. This is the first clinical and histological description of demodicosis in Eidolon helvum.
C1 [Bianco, Carlo; Nunez, Alejandro] Anim & Plant Hlth Agcy APHA Weybridge, Pathol Dept, Woodham Lane, Addlestone KT15 3NB, Surrey, England.
[Bianco, Carlo] Anim & Plant Hlth Agcy APHA Lasswade, Pathol Dept, Pentlands Sci Pk, Penicuik EH26 0PZ, Midlothian, Scotland.
[Baker, Kate S.] Univ Liverpool, Inst Integrat Biol, Biosci Bldg,Crown St, Liverpool L69 7ZB, Merseyside, England.
[Pazzini, Luca] LA Vallonea Vet Lab, Via Sirtori 9, I-20017 Milan, MI, Italy.
[Cafiso, Alessandra] Univ Milan, Dept Vet Med, Via Celoria 10, I-20133 Milan, Italy.
[Suu-Ire, Richard D.] Forestry Commiss, Wildlife Div, Box M239, Accra, Ghana.
[Suu-Ire, Richard D.] Minist Food & Agr, Vet Serv, Box M161, Accra, Ghana.
[Cunningham, Andrew A.] Zool Soc London, Inst Zool, Regents Pk, London NW1 4RY, England.
[Wood, James L. N.] Univ Cambridge, Dept Vet Med, Madingley Rd, Cambridge CB3 0ES, England.
C3 Animal & Plant Health Agency UK; University of Liverpool; University of Milan; Zoological Society of London; University of Cambridge
RP Bianco, C (corresponding author), Anim & Plant Hlth Agcy APHA Weybridge, Pathol Dept, Woodham Lane, Addlestone KT15 3NB, Surrey,

England.; Bianco, C (corresponding author), Anim & Plant Hlth Agcy APHA Lasswade, Pathol Dept, Pentlands Sci Pk, Penicuik EH26 0PZ, Midlothian, Scotland.
EM carlo.bianco@apha.gov.uk
RI Cunningham, Andrew/E-7536-2010; CAFISO, ALESSANDRA/AAF-8310-2021; Baker,
Kate/P-2087-2016; CAFISO, ALESSANDRA/K-7438-2017
OI Baker, Kate/0000-0001-5850-1949; CAFISO, ALESSANDRA/
0000-0001-8739-3422
FU Wellcome Trust Research Training Fellowship; Wellcome Trust Clinical
Career Development Fellowship [106690/Z/14/Z]; Wellcome Trust
[106690/Z/14/Z] Funding Source: Wellcome Trust
FX This work was part-funded by a Wellcome Trust Research Training
Fellowship (KSB) and KSB is supported by a Wellcome Trust Clinical
Career Development Fellowship (106690/Z/14/Z).
CR Baker KS, 2014, J ANIM ECOL, V83, P415, DOI 10.1111/1365-2656.12153
Baker KS, 2013, VIROLOGY, V441, P95, DOI 10.1016/j.virol.2013.03.014
Childs-Sanford SE, 2009, J ZOO WILDLIFE MED, V40, P8, DOI 10.1638/2007-0033.1
Dedhia P, 2007, ASIAN PAC J CANCER P, V8, P55
DESCH CE, 1989, NEW ZEAL J ZOOL, V16, P221, DOI 10.1080/03014223.1989.10422572
Fountain KI, 2017, VET DERMATOL, V28, P219, DOI 10.1111/vde.12410
Gross TL, 2005, SKIN DISEASES OF THE DOG AND CAT: CLINICAL AND HISTOPATHOLOGIC DIAGNOSIS, 2ND EDITION, P420
Kitano T, 2007, INT J LEGAL MED, V121, P423, DOI 10.1007/s00414-006-0113-y
Lankton JS, 2013, J ZOO WILDLIFE MED, V44, P124, DOI 10.1638/1042-7260-44.1.124
Mathison BA, 2014, CLIN MICROBIOL REV, V27, P48, DOI 10.1128/CMR.00008-13
Mauldine EA, 2015, JUBB KENNEDY PALMERS, V1, P509
Mühldorfer K, 2011, PLOS ONE, V6, DOI 10.1371/journal.pone.0029773
Otto Juergen C., 2001, P100
Rait VK, 2006, J HISTOCHEM CYTOCHEM, V54, P301, DOI 10.1369/jhc.5A6725.2005
Sastre N, 2016, PLOS ONE, V11, DOI 10.1371/journal.pone.0165765
Vargas Margarita, 1995, International Journal of Acarology, V21, P75
Zewe CM, 2017, VET DERMATOL, V28, P468, DOI 10.1111/vde.12453
Zhao YE, 2013, J ZHEJIANG UNIV-SC B, V14, P829, DOI 10.1631/jzus.B1200363
Zhao YE, 2012, PARASITOL RES, V111, P1113, DOI 10.1007/s00436-012-2941-7
NR 19
TC 3
Z9 4
U1 0
U2 4

PU SPRINGER
PI DORDRECHT
PA VAN GODEWIJCKSTRAAT 30, 3311 GZ DORDRECHT, NETHERLANDS
SN 0168-8162
EI 1572-9702
J9 EXP APPL ACAROL
JI Exp. Appl. Acarol.
PD AUG
PY 2019
VL 78
IS 4
BP 547
EP 554
DI 10.1007/s10493-019-00399-9
PG 8
WC Entomology
WE Science Citation Index Expanded (SCI-EXPANDED)
SC Entomology
GA IQ1CQ
UT WOS:000480487800006
PM 31289968
OA Green Accepted
DA 2024-10-21
ER

PT J
AU Rogers, EJ
   McGuire, L
   Longstaffe, FJ
   Clerc, J
   Kunkel, E
   Fraser, E
AF Rogers, Elizabeth J.
   McGuire, Liam
   Longstaffe, Fred J.
   Clerc, Jeff
   Kunkel, Emma
   Fraser, Erin
TI Relating wing morphology and immune function to patterns of partial and
   differential bat migration using stable isotopes
SO JOURNAL OF ANIMAL ECOLOGY
LA English
DT Article
DE bat migration; ecoimmunology; energetic trade-offs; stable isotopes;
   wing morphology
ID BODY-COMPOSITION; PHENOTYPIC FLEXIBILITY; ECOLOGICAL IMMUNOLOGY;
   FORAGING BEHAVIOR; LIFE-HISTORY; TRADE-OFFS; LASIONYCTERIS; FLIGHT;
   STOPOVER; STRESS
AB Migration is energetically expensive and is predicted to drive similar morphological adaptations and physiological trade-offs in migratory bats and birds. Previous studies suggest that fixed traits

like wing morphology vary among species and individuals according to selective pressures on flight, while immune defences can vary flexibly within individuals as energy is variably reallocated throughout the year. We assessed intraspecific variation in wing morphology and immune function in silver-haired bats Lasionycteris noctivagans, a species that follows both partial and differential migration patterns. We hypothesized that if bats experience energy constraints associated with migration, then wing morphology and immune function should vary based on migratory tendency (sedentary or migratory) and migration distance. We predicted that long-distance migrants would have reduced immune function and more migration-adapted wing shapes compared to resident or short-distance migrating bats. We estimated breeding latitude of spring migrants using stable hydrogen isotope techniques. Our sample consisted primarily of male bats, which we categorized as residents, long-distance northern migrants, short-distance northern migrants and southern migrants (apparent breeding location south of capture site). Controlling for individual condition and capture date, we related wing characteristics and immune indices among groups. Some, but not all, aspects of wing form and immune function varied between migrants and residents. Long-distance northern migrants had larger wings than short-distance northern migrants and lower wing loading than southern migrants. Compared with resident bats, short-distance northern migrants had reduced IgG while southern migrants had heightened neutrophils and neutrophil-to-lymphocyte ratios. Body fat, aspect ratio, wing tip shape and bacteria killing ability did not vary with migration status or distance. In general, male silver-haired bats do not appear to mediate migration costs by substantially downregulating immune defences or to be under stronger selection for wing forms adapted for fast, energy-efficient flight. Such phenotypic changes may be more adaptive for female silver-haired bats, which migrate farther and are more constrained by time in spring than males. Adaptations for aerial hawking and the use of heterothermy by migrating bats may also reduce the energetic cost of migration and the need for more substantial morphological and physiological trade-offs.
C1 [Rogers, Elizabeth J.; McGuire, Liam; Clerc, Jeff; Kunkel, Emma] Texas Tech Univ, Dept Biol Sci, Lubbock, TX 79409 USA.
[Rogers, Elizabeth J.] Univ Massachusetts, Organism & Evolutionary Biol Program, Amherst, MA 01003 USA.
[McGuire, Liam] Univ Waterloo, Dept Biol, Waterloo, ON, Canada.
[Longstaffe, Fred J.] Univ Western Ontario, Dept Earth Sci, London, ON, Canada.
[Clerc, Jeff] Normandeau Associates Inc, Gainesville, FL USA.
[Fraser, Erin] Mem Univ Newfoundland, Environm Sci Program, Grenfell Campus, Corner Brook, NL, Canada.
C3 Texas Tech University System; Texas Tech University; University of
Massachusetts System; University of Massachusetts Amherst; University of
Waterloo; Western University (University of Western Ontario); Memorial
University Newfoundland
RP Rogers, EJ (corresponding author), Texas Tech Univ, Dept Biol

Sci, Lubbock, TX 79409 USA.; Rogers, EJ (corresponding author), Univ Massachusetts, Organism & Evolutionary Biol Program, Amherst, MA 01003 USA.
EM bethrogs@gmail.com
RI McGuire, Liam/CAE-8434-2022; Longstaffe, Fred/A-6843-2008
OI Longstaffe, Fred/0000-0003-4103-4808; McGuire, Liam/0000-0002-5690-0804
FU Canada Research Chairs; Texas Tech University; Canada Foundation for
Innovation; Natural Sciences and Engineering Research Council of Canada
FX Canada Research Chairs; Texas Tech University; Canada Foundation for
Innovation; Natural Sciences and Engineering Research Council of Canada
CR Allen LC, 2009, J COMP PHYSIOL B, V179, P315, DOI 10.1007/s00360-008-0315-3
Ashton KG, 2002, GLOBAL ECOL BIOGEOGR, V11, P505, DOI 10.1046/j.1466-822X.2002.00313.x
Baloun DE, 2020, OECOLOGIA, V193, P67, DOI 10.1007/s00442-020-04650-8
Baloun DE, 2019, J EXP BIOL, V222, DOI 10.1242/jeb.196691
Bates D, 2015, J STAT SOFTW, V67, P1, DOI 10.18637/jss.v067.i01
Becker DJ, 2017, ROY SOC OPEN SCI, V4, DOI 10.1098/rsos.170073
Brooks ME, 2017, R J, V9, P378, DOI 10.32614/RJ-2017-066
Buehler DM, 2010, AUK, V127, P394, DOI 10.1525/auk.2010.09017
Campbell Caitlin J., 2020, Animal Migration, V7, P27, DOI 10.1515/ami-2020-0004
Chapman BB, 2011, OIKOS, V120, P1764, DOI 10.1111/j.1600-0706.2011.20131.x
CLERC J, 2021, FRONT ECOL EVOL, V9
Clerc J, 2021, OIKOS, V130, P1739, DOI 10.1111/oik.08178
Costantini D, 2019, CURR ZOOL, V65, P147, DOI 10.1093/cz/zoy039
Cryan PM, 2012, PLOS ONE, V7, DOI 10.1371/journal.pone.0047586
Cryan PM, 2003, J MAMMAL, V84, P579, DOI 10.1644/1545-1542(2003)084<0579:SDOMTB>2.0.CO;2
Davis AK, 2008, FUNCT ECOL, V22, P760, DOI 10.1111/j.1365-2435.2008.01467.x
Dechmann DKN, 2014, PLOS ONE, V9, DOI 10.1371/journal.pone.0114810
DHABHAR FS, 1995, J IMMUNOL, V154, P5511
Dingle Hugh, 1996, P1
Eikenaar C, 2020, CURR ZOOL, V66, P21, DOI 10.1093/cz/zoz009
Eikenaar C, 2016, BIOL LETTERS, V12, DOI 10.1098/rsbl.2016.0078
Evans SW, 2021, J ORNITHOL, V162, P765, DOI 10.1007/s10336-021-01870-4
Fenton MB, 2002, CAN J ZOOL, V80, P1004, DOI 10.1139/Z02-083
Fernández G, 2007, AUK, V124, P1037, DOI 10.1642/0004-8038(2007)124[1037:VITWMO]2.0.CO;2
Fiedler W, 2005, ANN NY ACAD SCI, V1046, P253, DOI 10.1196/annals.1343.022
Fleming T.H., 2019, ENCY ANIMAL BEHAV, V605, DOI DOI 10.1016/B978-0-12-809633-8.20764-4
Foo YZ, 2017, BIOL REV, V92, P551, DOI 10.1111/brv.12243

Fox J., 2011, An {R} companion to applied regression second
Fraser EE, 2017, J MAMMAL, V98, P1225, DOI 10.1093/jmammal/gyx085
Fraser EE, 2013, CAN J ZOOL, V91, P533, DOI 10.1139/cjz-2013-0072
French SS, 2012, BIOL OPEN, V1, P482, DOI 10.1242/bio.2012919
Fudickar AM, 2012, PLOS ONE, V7, DOI 10.1371/journal.pone.0051920
Gibson D, 2019, ECOGRAPHY, V42, P1482, DOI 10.1111/ecog.04325
Hasselquist D, 2012, ANIM BEHAV, V83, P1303, DOI 10.1016/j.anbehav.2012.03.025
Hedenström A, 2009, J MAMMAL, V90, P1298, DOI 10.1644/09-MAMM-S-075R2.1
Hegemann A, 2018, OECOLOGIA, V188, P1011, DOI 10.1007/s00442-018-4291-3
Hegemann A, 2015, AM NAT, V186, P531, DOI 10.1086/682667
Hothorn T, 2008, BIOMETRICAL J, V50, P346, DOI 10.1002/bimj.200810425
Huber GH, 2017, BIOL J LINN SOC, V120, P228
Jonasson KA, 2019, CAN J ZOOL, V97, P961, DOI 10.1139/cjz-2019-0036
Jonasson KA, 2016, J MAMMAL, V97, P1535, DOI 10.1093/jmammal/gyw119
Klasing Kirk C., 2004, Acta Zoologica Sinica, V50, P961
Lee KA, 2006, INTEGR COMP BIOL, V46, P1000, DOI 10.1093/icb/icl049
Lehnert LS, 2018, P ROY SOC B-BIOL SCI, V285, DOI 10.1098/rspb.2018.2174
Lochmiller RL, 2000, OIKOS, V88, P87, DOI 10.1034/j.1600-0706.2000.880110.x
Luo B, 2019, SCI REP-UK, V9, DOI 10.1038/s41598-019-41125-0
MARCHETTI K, 1995, J AVIAN BIOL, V26, P177, DOI 10.2307/3677316
Martin LB, 2008, PHILOS T R SOC B, V363, P321, DOI 10.1098/rstb.2007.2142
McGuire LP, 2014, PLOS ONE, V9, DOI 10.1371/journal.pone.0115724
McGuire LP, 2013, BIOL REV, V88, P767, DOI 10.1111/brv.12024
McGuire LP, 2013, COMP BIOCHEM PHYS B, V165, P138, DOI 10.1016/j.cbpb.2013.03.013
McGuire LP, 2013, J EXP BIOL, V216, P800, DOI 10.1242/jeb.072868
McGuire LP, 2012, J ANIM ECOL, V81, P377, DOI 10.1111/j.1365-2656.2011.01912.x
McGuire LP, 2010, J MAMMAL, V91, P1375, DOI 10.1644/10-MAMM-A-051.1
McGuire LP, 2009, J MAMMAL, V90, P1290, DOI 10.1644/09-MAMM-S-084R.1
Merriam C.H., 1887, T ROY SOC CAN, V4, P85
Moore MS, 2013, PLOS ONE, V8, DOI 10.1371/journal.pone.0058976
Neubaum DJ, 2006, J MAMMAL, V87, P470, DOI 10.1644/05-MAMM-A-252R1.1
NORBERG U M, 1987, Philosophical Transactions of the Royal Society of London B Biological Sciences, V316, P335, DOI 10.1098/rstb.1987.0030
Norris K, 2000, BEHAV ECOL, V11, P19, DOI 10.1093/beheco/11.1.19
O'Mara MT, 2016, PLOS ONE, V11, DOI 10.1371/journal.pone.0167027
O'SHEA T J, 1976, Southwestern Naturalist, V21, P321, DOI 10.2307/3669717
Owen JC, 2006, CONDOR, V108, P389, DOI

10.1650/0010-5422(2006)108[389:SDIICO]2.0.CO;2
Piersma T, 2003, TRENDS ECOL EVOL, V18, P228, DOI 10.1016/S0169-5347(03)00036-3
Pylant CL, 2014, PEERJ, V2, DOI 10.7717/peerj.629
R Core Team, 2021, R FDN STAT COMPUTING, DOI DOI 10.1016/J.DENDRO.2008.01.002
Racey P.A., 1982, P57
Rasband W., 1997, ImageJ, U. S. National Institutes of Health
Rayner J.M.V., 1988, Current Ornithology, V5, P1
Rogers EJ, 2019, PHYSIOL BIOCHEM ZOOL, V92, P386, DOI 10.1086/704107
Romero LM, 2005, COMP BIOCHEM PHYS A, V140, P73, DOI 10.1016/j.cbpb.2004.11.004
Ruhs EC, 2021, J EXP BIOL, V224, DOI 10.1242/jeb.241109
Ruoss S, 2019, MAMM BIOL, V94, P120, DOI 10.1016/j.mambio.2018.05.010
Schad J, 2012, PLOS ONE, V7, DOI 10.1371/journal.pone.0037101
Schmid-Hempel P, 2003, P ROY SOC B-BIOL SCI, V270, P357, DOI 10.1098/rspb.2002.2265
Schneeberger K, 2014, PLOS ONE, V9, DOI 10.1371/journal.pone.0108268
Schneeberger K, 2013, PLOS ONE, V8, DOI 10.1371/journal.pone.0054023
Senawi J, 2019, J EXP BIOL, V222, DOI 10.1242/jeb.203950
Smith RJ, 2009, AM J PHYS ANTHROPOL, V140, P476, DOI 10.1002/ajpa.21090
Soto DX, 2017, RAPID COMMUN MASS SP, V31, P1193, DOI 10.1002/rcm.7893
Stockmaier S, 2015, BIOL LETTERS, V11, DOI 10.1098/rsbl.2015.0576
Alcalde JT, 2021, MAMMALIA, V85, P161, DOI 10.1515/mammalia-2020-0069
van Dijk JGB, 2016, INTEGR COMP BIOL, V56, P290, DOI 10.1093/icb/icw045
Voigt CC, 2020, SCI REP-UK, V10, DOI 10.1038/s41598-020-74473-3
Warburton EM, 2016, PARASITOL RES, V115, P2155, DOI 10.1007/s00436-016-4957-x
Zuur A., 2009, Mixed effects models and extensions in ecology with R
NR 86
TC 7
Z9 9
U1 3
U2 37
PU WILEY
PI HOBOKEN
PA 111 RIVER ST, HOBOKEN 07030-5774, NJ USA
SN 0021-8790
EI 1365-2656
J9 J ANIM ECOL
JI J. Anim. Ecol.
PD APR
PY 2022
VL 91
IS 4

BP 858
EP 869
DI 10.1111/1365-2656.13681
EA MAR 2022
PG 12
WC Ecology; Zoology
WE Science Citation Index Expanded (SCI-EXPANDED)
SC Environmental Sciences & Ecology; Zoology
GA 0G4LR
UT WOS:000763748900001
PM 35218220
OA Green Submitted
DA 2024-10-21
ER

PT J
AU Gamage, AM
Chan, WOY
Zhu, F
Lim, YT
Long, S
Ahn, M
Tan, CW
Foo, RJH
Sia, WR
Lim, XF
He, HP
Zhai, WW
Anderson, DE
Sobota, RM
Dutertre, CA
Wang, LF
AF Gamage, Akshamal M.
Chan, Wharton O. Y.
Zhu, Feng
Lim, Yan Ting
Long, Sandy
Ahn, Matae
Tan, Chee Wah
Foo, Randy Jee Hiang
Sia, Wan Rong
Lim, Xiao Fang
He, Haopeng
Zhai, Weiwei
Anderson, Danielle E.
Sobota, Radoslaw Mikolaj
Dutertre, Charles-Antoine
Wang, Lin-Fa
TI Single-cell transcriptome analysis of the<i> in</i><i> vivo</i> response
to viral infection in the cave nectar bat<i> Eonycteris</i><i> spelaea</i>
SO IMMUNITY
LA English

DT Article
ID HUMAN INDOLEAMINE 2,3-DIOXYGENASE; RNA-SEQ DATA;
PTEROPINE-ORTHOREOVIRUS; FRUIT BATS; POSTTRANSLATIONAL
REGULATION;
SEROLOGICAL EVIDENCE; RESPIRATORY-DISEASE; DENDRITIC CELLS;
RESERVOIR
HOSTS; MARBURG VIRUS
AB Bats are reservoir hosts of many zoonotic viruses with pandemic potential. We utilized single-cell transcrip-tome sequencing (scRNA-seq) to analyze the immune response in bat lungs upon in vivo infection with a dou-ble-stranded RNA virus, Pteropine orthoreovirus PRV3M. Bat neutrophils were distinguished by high basal IDO1 expression. NK cells and T cells were the most abundant immune cells in lung tissue. Three distinct CD8+ effector T cell populations could be delineated by differential expression of KLRB1, GFRA2, and DPP4. Select NK and T clusters increased expression of genes involved in T cell activation and effector func-tion early after viral infection. Alveolar macrophages and classical monocytes drove antiviral interferon signaling. Infection expanded a CSF1R+ population expressing collagen-like genes, which became the pre-dominant myeloid cell type post-infection. This work uncovers features relevant to viral disease tolerance in bats, lays a foundation for future experimental work, and serves as a resource for comparative immunology studies.
C1 [Gamage, Akshamal M.; Chan, Wharton O. Y.; Zhu, Feng; Ahn, Matae; Tan, Chee Wah; Foo, Randy Jee Hiang; Sia, Wan Rong; Lim, Xiao Fang; Anderson, Danielle E.; Wang, Lin-Fa] Duke NUS Med Sch, Programme Emerging Infect Dis, Singapore, Singapore.
[Lim, Yan Ting; Long, Sandy; Sobota, Radoslaw Mikolaj] ASTAR, Funct Prote Lab, SingMass Natl Lab, Inst Mol & Cell Biol, Singapore, Singapore.
[He, Haopeng; Zhai, Weiwei] Chinese Acad Sci, Inst Zool, Key Lab Zool Systemat & Evolut, Beijing 100101, Peoples R China.
[Zhai, Weiwei] Chinese Acad Sci, Ctr Excellence Anim Evolut & Genet, Kunming 650223, Yunnan, Peoples R China.
[Zhai, Weiwei] Agcy Sci Technol & Res, Human Genet, Genome Inst Singapore, Singapore 138672, Singapore.
[Anderson, Danielle E.] Univ Melbourne, Dept Microbiol & Immunol, Peter Doherty Inst Infect & Immun, Melbourne, Vic 3000, Australia.
[Dutertre, Charles-Antoine] Equipe Labellisee Ligue Natl Canc, Inst Natl Sante & Rech Med INSERM, U1015, Villejuif, France.
[Wang, Lin-Fa] Singhlth Duke NUS Global Hlth Inst, Singapore, Singapore.
[Dutertre, Charles-Antoine] Gustave Roussy Canc Campus, Villejuif, France.
C3 National University of Singapore; Agency for Science Technology &
Research (A*STAR); A*STAR - Institute of Molecular & Cell Biology
(IMCB); Chinese Academy of Sciences; Institute of Zoology, CAS;
Chinese
Academy of Sciences; Agency for Science Technology & Research
(A*STAR);
A*STAR - Genome Institute of Singapore (GIS); University of
Melbourne;
Peter Doherty Institute; Universite Paris Saclay; Institut

National de
la Sante et de la Recherche Medicale (Inserm)
RP Wang, LF (corresponding author), Duke NUS Med Sch, Programme
Emerging Infect Dis, Singapore, Singapore.; Wang, LF (corresponding
author), Singhlth Duke NUS Global Hlth Inst, Singapore, Singapore.
EM linfa.wang@duke-nus.edu.sg
RI Wang, Linfa/ACW-3768-2022; Dutertre, Charles Antoine/
JRX-2224-2023;
Anderson, Danielle/HCH-2004-2022; Tan, Chee/K-7450-2012; Zhu,
Feng/GOH-1593-2022
OI Zhu, Feng/0000-0002-8131-1219; Ahn, Matae/0000-0003-2114-8250;
Sobota,
Radoslaw/0000-0002-2455-2526; Tan, Chee Wah/0000-0001-9837-1413;
Chan,
Wharton O Yinn/0000-0002-4526-470X; Anderson,
Danielle/0000-0003-4791-5024; Dutertre,
Charles-Antoine/0000-0001-7950-3652
FU Singapore National Research Foundation [NRF2012NRF-CRP001-056,
NRF2016NRF-NSFC002-013]; National Medical Research Council of
Singapore
[MOH-OFIRG19MAY-0011, MOH-OFIRG10-NOV-0050]; Ministry of
Education-Singapore [MOE2019-T2-2-130]; Singapore National
Research
Foundation grant NRF-SIS [NRF2017_SISFP08]; A*STAR
FX We thank Dr. Edgar M. Pena, Rommel E. Yroy. and the rest of the
veterinary team at SingHealth Experimental Medicine Centre (SEMC)
for
technical expertise and assistance in maintaining the E. spelaea
colony
and in vivo experiments. We also thank Jae Tan Peck Yean from
Research
Instruments, Singapore for technical expertise and assistance in
library
preparation for the 10X Genomics platform. This research was
funded by
Singapore National Research Foundation (NRF2012NRF-CRP001-056,
NRF2016NRF-NSFC002-013), the National Medical Research Council of
Singapore (MOH-OFIRG19MAY-0011, MOH-OFIRG10-NOV-0050) and the
Ministry
of Education -Singapore (MOE2019-T2-2-130) to L.-F.W. R.M.S. is
supported by A*STAR Core funding and Singapore National Research
Foundation grant NRF-SIS (NRF2017_SISFP08) "SingMass"share
infrastructure scheme.
NR 0
TC 12
Z9 12
U1 1
U2 16
PU CELL PRESS
PI CAMBRIDGE
PA 50 HAMPSHIRE ST, FLOOR 5, CAMBRIDGE, MA 02139 USA
SN 1074-7613
EI 1097-4180
J9 IMMUNITY

JI Immunity
PD NOV 8
PY 2022
VL 55
IS 11
BP 2187
EP +
DI 10.1016/j.immuni.2022.10.008
EA NOV 2022
PG 25
WC Immunology
WE Science Citation Index Expanded (SCI-EXPANDED)
SC Immunology
GA 7F5CL
UT WOS:000901865300001
PM 36351376
OA Bronze
DA 2024-10-21
ER

PT J
AU Edenborough, KM
   Bokelmann, M
   Lander, A
   Couacy-Hymann, E
   Lechner, J
   Drechsel, O
   Renard, BY
   Radonic, A
   Feldmann, H
   Kurth, A
   Prescott, J
AF Edenborough, Kathryn M.
   Bokelmann, Marcel
   Lander, Angelika
   Couacy-Hymann, Emmanuel
   Lechner, Johanna
   Drechsel, Oliver
   Renard, Bernhard Y.
   Radonic, Aleksandar
   Feldmann, Heinz
   Kurth, Andreas
   Prescott, Joseph
TI Dendritic Cells Generated From <i>Mops condylurus</i>, a Likely
   Filovirus Reservoir Host, Are Susceptible to and Activated by
Zaire
   Ebolavirus Infection
SO FRONTIERS IN IMMUNOLOGY
LA English
DT Article
DE Ebola virus (EBOV); reservoir hosts; dendritic cells;
transcriptome;
   Mops condylurus; filovirus
ID EXPERIMENTAL INOCULATION; HUMAN MACROPHAGES; VIRUS INFECTION;

FRUIT
   BATS; MARBURG; IMMUNOLOGY; IMMUNITY; RABBIT; MICE; CSF
AB Ebola virus infection of human dendritic cells (DCs) induces atypical adaptive immune responses and thereby exacerbates Ebola virus disease (EVD). Human DCs, infected with Ebola virus aberrantly express low levels of the DC activation markers CD80, CD86, and MHC class II. The T cell responses ensuing are commonly anergic rather than protective against EVD. We hypothesize that DCs derived from potential reservoir hosts such as bats, which do not develop disease signs in response to Ebola virus infection, would exhibit features associated with activation. In this study, we have examined Zaire ebolavirus (EBOV) infection of DCs derived from the Angolan free-tailed bat species, Mops condylurus. This species was previously identified as permissive to EBOV infection in vivo, in the absence of disease signs. M. condylurus has also been recently implicated as the reservoir host for Bombali ebolavirus, a virus species that is closely related to EBOV. Due to the absence of pre-existing M. condylurus species-specific reagents, we characterized its de novo assembled transcriptome and defined its phylogenetic similarity to other mammals, which enabled the identification of cross-reactive reagents for M. condylurus bone marrow-derived DC (bat-BMDC) differentiation and immune cell phenotyping. Our results reveal that bat-BMDCs are susceptible to EBOV infection as determined by detection of EBOV specific viral RNA (vRNA). vRNA increased significantly 72 h after EBOV-infection and was detected in both cells and in culture supernatants. Bat-BMDC infection was further confirmed by the observation of GFP expression in DC cultures infected with a recombinant GFP-EBOV. Bat-BMDCs upregulated CD80 and chemokine ligand 3 (CCL3) transcripts in response to EBOV infection, which positively correlated with the expression levels of EBOV vRNA. In contrast to the aberrant responses to EBOV infection that are typical for human-DC, our findings from bat-BMDCs provide evidence for an immunological basis of asymptomatic EBOV infection outcomes.
C1 [Edenborough, Kathryn M.; Bokelmann, Marcel; Lander, Angelika; Kurth, Andreas; Prescott, Joseph] Robert Koch Inst, Ctr Biol Threats & Special Pathogens, Berlin, Germany.
   [Couacy-Hymann, Emmanuel] Lab Natl Appui Dev Agr, LANADA, Bingerville, Cote Ivoire.
   [Lechner, Johanna; Drechsel, Oliver; Renard, Bernhard Y.; Radonic, Aleksandar] Robert Koch Inst, Methodol & Res Infrastruct, Berlin, Germany.
   [Feldmann, Heinz] NIAID, Lab Virol, Div Intramural Res, NIH, Hamilton, MT USA.
   [Edenborough, Kathryn M.] Monash Univ, Inst Vector Borne Dis, Clayton, Vic, Australia.
C3 Robert Koch Institute; Robert Koch Institute; National Institutes of
   Health (NIH) - USA; NIH National Institute of Allergy & Infectious
   Diseases (NIAID); Monash University
RP Edenborough, KM; Prescott, J (corresponding author), Robert Koch Inst, Ctr Biol Threats & Special Pathogens, Berlin, Germany.; Edenborough, KM (corresponding author), Monash Univ, Inst Vector Borne Dis, Clayton, Vic, Australia.

EM kat.edenborough@monash.edu; PrescottJ@rki.de
RI Edenborough, Kathryn/K-2626-2019; Radonić, Aleksandar/
AAP-2612-2020
OI Edenborough, Kathryn/0000-0002-9598-5740; Prescott,
   Joseph/0000-0001-5718-5155; Renard, Bernhard/0000-0003-4589-9809
FU Division of Intramural Research (DIR), National Institute of
Allergy and
   Infectious Diseases (NIAID), National Institutes of Health (NIH)
FX This work was partially funded by the Division of Intramural
Research
   (DIR), National Institute of Allergy and Infectious Diseases
(NIAID),
   National Institutes of Health (NIH).
CR Bachem A, 2012, FRONT IMMUNOL, V3, DOI 10.3389/fimmu.2012.00214
   BarrattBoyes SM, 1996, IMMUNOLOGY, V87, P528, DOI 10.1046/
j.1365-2567.1996.514588.x
   Bosio CM, 2003, J INFECT DIS, V188, P1630, DOI 10.1086/379199
   Chen N, 2016, SCI REP-UK, V6, DOI 10.1038/srep19420
   Cody V, 2005, VET IMMUNOL IMMUNOP, V103, P163, DOI 10.1016/
j.vetimm.2004.08.022
   Dutertre CA, 2014, CELL IMMUNOL, V291, P3, DOI 10.1016/
j.cellimm.2014.08.006
   Escudero-Pérez B, 2014, PLOS PATHOG, V10, DOI 10.1371/
journal.ppat.1004509
   Feldmann H, 2018, ANNU REV MICROBIOL, V72, P423, DOI 10.1146/
annurev-micro-090817-062414
   Feldmann H, 2011, LANCET, V377, P849, DOI 10.1016/
S0140-6736(10)60667-8
   Forbes KM, 2019, EMERG INFECT DIS, V25, P955, DOI 10.3201/
eid2505.181666
   Geisbert TW, 2003, AM J PATHOL, V163, P2347, DOI 10.1016/
S0002-9440(10)63591-2
   Goeijenbier M, 2014, NETH J MED, V72, P442
   Goldstein T, 2018, NAT MICROBIOL, V3, P1084, DOI 10.1038/
s41564-018-0227-2
   Gotz S, 2008, NUCLEIC ACIDS RES, V36, P3420, DOI 10.1093/nar/
gkn176
   Guilliams M, 2016, IMMUNITY, V45, P669, DOI 10.1016/
j.immuni.2016.08.015
   Gupta M, 2001, VIROLOGY, V284, P20, DOI 10.1006/viro.2001.0836
   Helft J, 2015, IMMUNITY, V42, P1197, DOI 10.1016/
j.immuni.2015.05.018
   Holling TM, 2004, HUM IMMUNOL, V65, P282, DOI 10.1016/
j.humimm.2004.01.005
   Jasper PJ, 2003, J IMMUNOL, V171, P6372, DOI 10.4049/
jimmunol.171.12.6372
   Jin HL, 2010, J GEN VIROL, V91, P352, DOI 10.1099/vir.0.017343-0
   Jones MEB, 2015, VIRUSES-BASEL, V7, P3420, DOI 10.3390/v7072779
   Kuhn J, 2008, ARCH VIROL, V20, P59, DOI
[10.1007/978-3-211-69495-4, DOI 10.1007/978-3-211-69495-4]
   Kuzmin IV, 2017, J VIROL, V91, DOI [10.1128/JVI.02471-16,
10.1128/jvi.02471-16]
   Lee AK, 2015, BMC GENOMICS, V16, DOI 10.1186/s12864-015-2124-x
   Leendertz SAJ, 2016, ECOHEALTH, V13, P18, DOI 10.1007/

s10393-015-1053-0
Leroy EM, 2005, NATURE, V438, P575, DOI 10.1038/438575a
Leroy EM, 2009, VECTOR-BORNE ZOONOT, V9, P723, DOI 10.1089/vbz.2008.0167
Lüdtke A, 2017, SCI REP-UK, V7, DOI 10.1038/srep43776
Mahanty S, 2003, J IMMUNOL, V170, P2797, DOI 10.4049/jimmunol.170.6.2797
MaManes MD, 2018, PEERJ, V6, DOI 10.7717/peerj.5428
Menicucci AR, 2017, FRONT IMMUNOL, V8, DOI 10.3389/fimmu.2017.01372
Mohamadzadeh M, 2007, NAT REV IMMUNOL, V7, P556, DOI 10.1038/nri2098
Monadjem A., 2010, BATS SO CENTRAL AFRI
Ng M, 2015, ELIFE, V4, DOI 10.7554/eLife.11785
Okumura A, 2010, J VIROL, V84, P27, DOI 10.1128/JVI.01462-09
Olejnik J, 2017, J VIROL, V91, DOI 10.1128/JVI.00179-17
Peixoto FP, 2018, BMC ECOL, V18, DOI 10.1186/s12898-018-0174-z
Pourrut X, 2009, BMC INFECT DIS, V9, DOI 10.1186/1471-2334-9-159
Saéz AM, 2015, EMBO MOL MED, V7, P17, DOI 10.15252/emmm.201404792
Schountz T, 2014, VIRUSES-BASEL, V6, P4880, DOI 10.3390/v6124880
Schumann J, 2015, PLOS ONE, V10, DOI 10.1371/journal.pone.0132479
Shaw TI, 2012, PLOS ONE, V7, DOI 10.1371/journal.pone.0048472
Singh-Jasuja H, 2013, IMMUNOBIOLOGY, V218, P28, DOI 10.1016/j.imbio.2012.01.021
Speranza E, 2017, VACCINES-BASEL, V5, DOI 10.3390/vaccines5030030
Ströher U, 2001, J VIROL, V75, P11025, DOI 10.1128/JVI.75.22.11025-11033.2001
Swanepoel R, 1996, EMERG INFECT DIS, V2, P321, DOI 10.3201/eid0204.960407
Teeling EC, 2018, ANNU REV ANIM BIOSCI, V6, P23, DOI 10.1146/annurev-animal-022516-022811
Manh TPV, 2015, FRONT IMMUNOL, V6, DOI 10.3389/fimmu.2015.00260
Thompson Julie D, 2002, Curr Protoc Bioinformatics, VChapter 2, DOI 10.1002/0471250953.bi0203s00
Towner JS, 2009, PLOS PATHOG, V5, DOI 10.1371/journal.ppat.1000536
Twenhafel NA, 2013, VET PATHOL, V50, P514, DOI 10.1177/0300985812469636
Wahl-Jensen V, 2011, PLOS NEGLECT TROP D, V5, DOI 10.1371/journal.pntd.0001359
Wauquier N, 2010, PLOS NEGLECT TROP D, V4, DOI 10.1371/journal.pntd.0000837
Younan P, 2017, MBIO, V8, DOI [10.1128/mBio.00845-17, 10.1128/mbio.00845-17]
Zhou P, 2016, SCI REP-UK, V6, DOI 10.1038/srep38597
NR 55
TC 5
Z9 6
U1 0
U2 8
PU FRONTIERS MEDIA SA
PI LAUSANNE
PA AVENUE DU TRIBUNAL FEDERAL 34, LAUSANNE, CH-1015, SWITZERLAND
SN 1664-3224

J9 FRONT IMMUNOL
JI Front. Immunol.
PD OCT 11
PY 2019
VL 10
AR 2414
DI 10.3389/fimmu.2019.02414
PG 13
WC Immunology
WE Science Citation Index Expanded (SCI-EXPANDED)
SC Immunology
GA JO1BB
UT WOS:000497319300001
PM 31681302
OA Green Published, gold
DA 2024-10-21
ER

PT J
AU Pavlovich, SS
   Lovett, SP
   Koroleva, G
   Guito, JC
   Arnold, CE
   Nagle, ER
   Kulcsar, K
   Lee, A
   Thibaud-Nissen, F
   Hume, AJ
   Mühlberger, E
   Uebelhoer, LS
   Towner, JS
   Rabadan, R
   Sanchez-Lockhart, M
   Kepler, TB
   Palacios, G
AF Pavlovich, Stephanie S.
   Lovett, Sean P.
   Koroleva, Galina
   Guito, Jonathan C.
   Arnold, Catherine E.
   Nagle, Elyse R.
   Kulcsar, Kirsten
   Lee, Albert
   Thibaud-Nissen, Francoise
   Hume, Adam J.
   Muhlberger, Elke
   Uebelhoer, Luke S.
   Towner, Jonathan S.
   Rabadan, Raul
   Sanchez-Lockhart, Mariano
   Kepler, Thomas B.
   Palacios, Gustavo
TI The Egyptian Rousette Genome Reveals Unexpected Features of Bat

Antiviral Immunity
SO CELL
LA English
DT Article
ID MARBURG VIRUS; MESSENGER-RNAS; ALIGNMENT; EVOLUTION; SEQUENCE; TOOL;
RECOGNITION; EXPRESSION; RECEPTORS; GENES
AB Bats harbor many viruses asymptomatically, including several notorious for causing extreme virulence in humans. To identify differences between antiviral mechanisms in humans and bats, we sequenced, assembled, and analyzed the genome of Rousettus aegyptiacus, a natural reservoir of Marburg virus and the only known reservoir for any filovirus. We found an expanded and diversified KLRC/KLRD family of natural killer cell receptors, MHC class I genes, and type I interferons, which dramatically differ from their functional counterparts in other mammals. Such concerted evolution of key components of bat immunity is strongly suggestive of novel modes of antiviral defense. An evaluation of the theoretical function of these genes suggests that an inhibitory immune state may exist in bats. Based on our findings, we hypothesize that tolerance of viral infection, rather than enhanced potency of antiviral defenses, may be a key mechanism by which bats asymptomatically host viruses that are pathogenic in humans.
C1 [Pavlovich, Stephanie S.; Hume, Adam J.; Muhlberger, Elke; Kepler, Thomas B.] Boston Univ, Sch Med, Dept Microbiol, Boston, MA 02118 USA.
[Lovett, Sean P.; Koroleva, Galina; Arnold, Catherine E.; Nagle, Elyse R.; Kulcsar, Kirsten; Sanchez-Lockhart, Mariano; Palacios, Gustavo] US Army, Ctr Genome Sci, Res Inst Infect Dis USAMRIID, Frederick, MD 21702 USA.
[Lee, Albert; Rabadan, Raul] Columbia Univ, Dept Syst Biol, New York, NY USA.
[Lee, Albert; Rabadan, Raul] Columbia Univ, Dept Biomed Informat, New York, NY USA.
[Thibaud-Nissen, Francoise] NIH, Natl Ctr Biotechnol Informat, Natl Lib Med, Bethesda, MD 20892 USA.
[Guito, Jonathan C.; Uebelhoer, Luke S.; Towner, Jonathan S.] Ctr Dis Control & Prevent, Viral Special Pathogens Branch, Atlanta, GA 30333 USA.
[Sanchez-Lockhart, Mariano] Univ Nebraska Med Ctr, Dept Pathol & Microbiol, Omaha, NE 68198 USA.
[Kepler, Thomas B.] Boston Univ, Dept Math & Stat, Boston, MA 02215 USA.
[Kepler, Thomas B.] Boston Univ, Natl Emerging Infect Dis Lab, Boston, MA 02118 USA.
C3 Boston University; Columbia University; Columbia University; National
Institutes of Health (NIH) - USA; NIH National Library of Medicine
(NLM); Centers for Disease Control & Prevention - USA; University of
Nebraska System; University of Nebraska Medical Center; Boston
University; Boston University
RP Kepler, TB (corresponding author), Boston Univ, Sch Med, Dept

Microbiol, Boston, MA 02118 USA.; Palacios, G (corresponding
author), US Army, Ctr Genome Sci, Res Inst Infect Dis USAMRIID,
Frederick, MD 21702 USA.; Kepler, TB (corresponding author), Boston
Univ, Dept Math & Stat, Boston, MA 02215 USA.; Kepler, TB
(corresponding author), Boston Univ, Natl Emerging Infect Dis Lab,
Boston, MA 02118 USA.
EM tbkepler@bu.edu; gustavo.f.palacios.ctr@mail.mil
RI Towner, Jonathan/ABC-3780-2020; Hume, Adam/ABA-7439-2020;
Rabadan,
Raul/AAZ-7367-2020; Muhlberger, Elke/AAD-6924-2019; Palacios,
Gustavo/I-7773-2015; Sanchez-Lockhart, Mariano/H-1321-2019
OI Arnold, Catherine/0000-0002-1635-4941; Kulcsar,
Kirsten/0000-0001-9385-8775; Hume, Adam/0000-0001-8454-3472;
Rabadan,
Raul/0000-0001-7946-9255; Muhlberger, Elke/0000-0003-3547-9376;
Palacios, Gustavo/0000-0001-5062-1938; Sanchez-Lockhart,
Mariano/0000-0001-8009-7540
FU Defense Threat Reduction Agency, Department of Defense
[HDTRA1-14-1-0016]; NIH NIAID [T32AI007309-26]; NIH NIGMS
[5R01GM117591,
1R01GM109018]; NIH, National Library of Medicine; EU ANTIGONE
[278976];
German Research Council [DR 772/10-2]
FX We thank John H. Connor for his generous contribution of VSV-
eGFP,
Kaitlin Sawatzki for her help with Figure 1 and members of the
Kepler
lab for their helpful discussion. We gratefully acknowledge Ayan
K.
Chakrabarti, who performed the RNA extraction for the interferon
induction experiment. Opinions, interpretations, conclusions, and
recommendations are those of the authors and are not necessarily
endorsed by the U.S. Army or the Centers for Disease Control and
Prevention. Research was conducted under an IACUC-approved
protocol in
compliance with the Animal Welfare Act, PHS Policy, and other
federal
statutes and regulations relating to animals and experiments
involving
animals. The facility where this research was conducted is
accredited by
the Association for Assessment and Accreditation of Laboratory
Animal
Care, International and adheres to principles stated in the Guide
for
the Care and Use of Laboratory Animals, National Research
Council, 2011.
This work has been funded by the Defense Threat Reduction Agency,
Department of Defense (HDTRA1-14-1-0016 to G.P. and J.S.T.) and
NIH
NIAID (training grant T32AI007309-26 to S.S.P., awarded to David
H.
Sherr). Part of the work was funded by NIH NIGMS (5R01GM117591
and

1R01GM109018 to R.R. and A.L.). Part of this project was also supported
by the Intramural Research Program of the NIH, National Library of
Medicine (F.T.-N.). The Rousettus aegyptiacus cell line RoNi/7.1 was
generated by M.A. Muller and C. Drosten, Charite-Universitatsmedizin
Berlin, Germany with funds from the EU-FP7 ANTIGONE (no. 278976)
framework and the German Research Council (DR 772/10-2).
CR ALTSCHUL SF, 1990, J MOL BIOL, V215, P403, DOI 10.1016/S0022-2836(05)80360-2
Amman BR, 2015, J WILDLIFE DIS, V51, P113, DOI 10.7589/2014-08-198
Averdam A, 2009, PLOS GENET, V5, DOI 10.1371/journal.pgen.1000688
Bailly-Bechet M, 2014, MOBILE DNA-UK, V5, DOI 10.1186/1759-8753-5-13
Baker ML, 2013, ZOONOSES PUBLIC HLTH, V60, P104, DOI 10.1111/j.1863-2378.2012.01528.x
Banerjee A, 2017, SCI REP-UK, V7, DOI 10.1038/s41598-017-01513-w
Bateman A, 2015, NUCLEIC ACIDS RES, V43, pD204, DOI 10.1093/nar/gku989
BENJAMINI Y, 1995, J R STAT SOC B, V57, P289, DOI 10.1111/j.2517-6161.1995.tb02031.x
Bielawski JP, 2005, STAT BIOL HEALTH, P103, DOI 10.1007/0-387-27733-1_5
Biesold SE, 2011, PLOS ONE, V6, DOI 10.1371/journal.pone.0028131
Boetzer M, 2011, BIOINFORMATICS, V27, P578, DOI 10.1093/bioinformatics/btq683
Bolger AM, 2014, BIOINFORMATICS, V30, P2114, DOI 10.1093/bioinformatics/btu170
Bray NL, 2016, NAT BIOTECHNOL, V34, P888, DOI 10.1038/nbt0816-888d
Calisher CH, 2006, CLIN MICROBIOL REV, V19, P531, DOI 10.1128/CMR.00017-06
Capella-Gutiérrez S, 2009, BIOINFORMATICS, V25, P1972, DOI 10.1093/bioinformatics/btp348
Connor JH, 2015, J VIROL, V89, P9865, DOI 10.1128/JVI.01142-15
De Bie T, 2006, BIOINFORMATICS, V22, P1269, DOI 10.1093/bioinformatics/btl097
De La Cruz-Rivera PC, 2018, J IMMUNOL, V200, P209, DOI 10.4049/jimmunol.1701214
Edgar RC, 2004, NUCLEIC ACIDS RES, V32, P1792, DOI 10.1093/nar/gkh340
Fischer Steve, 2011, Curr Protoc Bioinformatics, VChapter 6, DOI 10.1002/0471250953.bi0612s35
Gifford R. J., DIGS FOR EVES
Hall T., 1999, NUCL ACIDS S SER, P95, DOI [DOI 10.14344/IOC.ML.11.1, 10.1021/bk-1999-0734.ch008]
Hölzer M, 2016, SCI REP-UK, V6, DOI 10.1038/srep34589
Hoffmann HH, 2015, TRENDS IMMUNOL, V36, P124, DOI 10.1016/j.it.2015.01.004
Jones MEB, 2015, VIRUSES-BASEL, V7, P3420, DOI 10.3390/v7072779
Kaiser BK, 2008, P NATL ACAD SCI USA, V105, P6696, DOI 10.1073/

pnas.0802736105
Kapustin Y, 2008, BIOL DIRECT, V3, DOI 10.1186/1745-6150-3-20
Katoh Kazutaka, 2013, Mol Biol Evol, V30, P772, DOI 10.1093/molbev/mst010
Kepler TB, 2010, BMC GENOMICS, V11, DOI 10.1186/1471-2164-11-444
Kikuchi-Maki A, 2005, J IMMUNOL, V174, P3859, DOI 10.4049/jimmunol.174.7.3859
Koch J, 2013, TRENDS IMMUNOL, V34, P182, DOI 10.1016/j.it.2013.01.003
Kolde, 2012, R package version
Kulski JK, 2002, IMMUNOL REV, V190, P95, DOI 10.1034/j.1600-065X.2002.19008.x
Kwiecinski Gary G., 1999, Mammalian Species, V611, P1
Langmead B, 2012, NAT METHODS, V9, P357, DOI [10.1038/NMETH.1923, 10.1038/nmeth.1923]
Lanier LL, 2009, IMMUNOL REV, V227, P150, DOI 10.1111/j.1600-065X.2008.00720.x
Lee AK, 2015, BMC GENOMICS, V16, DOI 10.1186/s12864-015-2124-x
Li H., 2013, PREPRINT, DOI DOI 10.48550/ARXIV.1303.3997
Li H, 2009, BIOINFORMATICS, V25, P1094, DOI [10.1093/bioinformatics/btp100, 10.1093/bioinformatics/btp324]
Li RQ, 2010, NATURE, V463, P311, DOI 10.1038/nature08696
Liu X, 2017, GENOME BIOL, V18, DOI 10.1186/s13059-016-1137-3
Lowe TM, 1997, NUCLEIC ACIDS RES, V25, P955, DOI 10.1093/nar/25.5.955
Maldonado E, 2016, BMC BIOINFORMATICS, V17, DOI 10.1186/s12859-016-1204-5
Malireddi RKS, 2013, FRONT CELL INFECT MI, V3, DOI 10.3389/fcimb.2013.00077
Marcais G, 2011, BIOINFORMATICS, V27, P764, DOI 10.1093/bioinformatics/btr011
Mi HY, 2016, NUCLEIC ACIDS RES, V44, pD336, DOI 10.1093/nar/gkv1194
Molfetta R, 2016, TRENDS IMMUNOL, V37, P790, DOI 10.1016/j.it.2016.08.015
Morgulis A, 2006, BIOINFORMATICS, V22, P134, DOI 10.1093/bioinformatics/bti774
Mudunuri U, 2009, BIOINFORMATICS, V25, P555, DOI 10.1093/bioinformatics/btn654
National Center for Biotechnology Information, GNOM NCBI EUK GEN PR
NCBI Eukaryotic Annotation Group, 2016, NCBI ROUS AEG ANN RE
Ng JHJ, 2016, SCI REP-UK, V6, DOI 10.1038/srep21256
O'Callaghan CA, 2000, CLIN SCI, V99, P9, DOI 10.1042/CS19990334
Olival KJ, 2017, NATURE, V546, P646, DOI 10.1038/nature22975
Papenfuss AT, 2012, BMC GENOMICS, V13, DOI 10.1186/1471-2164-13-261
Parham P, 2013, NAT REV IMMUNOL, V13, P133, DOI 10.1038/nri3370
Parker J, 2013, NATURE, V502, P228, DOI 10.1038/nature12511
Parra G, 2007, BIOINFORMATICS, V23, P1061, DOI 10.1093/bioinformatics/btm071
Petrie EJ, 2008, J EXP MED, V205, P725, DOI 10.1084/jem.20072525
Rapaport AS, 2015, IMMUNITY, V43, P1112, DOI 10.1016/j.immuni.2015.11.005

Salmela L, 2014, BIOINFORMATICS, V30, P3506, DOI 10.1093/bioinformatics/btu538
Schuh AJ, 2017, NAT COMMUN, V8, DOI 10.1038/ncomms14446
Secombes CJ, 2017, FRONT IMMUNOL, V8, DOI 10.3389/fimmu.2017.00209
Seim I, 2013, NAT COMMUN, V4, DOI 10.1038/ncomms3212
Shaw TI, 2012, PLOS ONE, V7, DOI 10.1371/journal.pone.0048472
Smit AFA, Re peatMask er Open -4.0
Smith I, 2013, CURR OPIN VIROL, V3, P84, DOI 10.1016/j.coviro.2012.11.006
Stamatakis A, 2014, BIOINFORMATICS, V30, P1312, DOI 10.1093/bioinformatics/btu033
Tamura K, 2013, MOL BIOL EVOL, V30, P2725, DOI [10.1093/molbev/mst197, 10.1093/molbev/msr121]
Thibaud-Nissen F., 2013, EUKARYOTIC GENOME AN
Towner JS, 2009, PLOS PATHOG, V5, DOI 10.1371/journal.ppat.1000536
Tsagkogeorga G, 2017, SCI REP-UK, V7, DOI 10.1038/s41598-017-00132-9
Walker BJ, 2014, PLOS ONE, V9, DOI 10.1371/journal.pone.0112963
Warren RL, 2015, GIGASCIENCE, V4, DOI 10.1186/s13742-015-0076-3
Whitlow ZW, 2008, J VIROL, V82, P2286, DOI 10.1128/JVI.01761-07
Whitlow ZW, 2006, J VIROL, V80, P11733, DOI 10.1128/JVI.00971-06
Xue W, 2013, BMC GENOMICS, V14, DOI 10.1186/1471-2164-14-604
Yang ZH, 1998, MOL BIOL EVOL, V15, P568, DOI 10.1093/oxfordjournals.molbev.a025957
Yates A, 2016, NUCLEIC ACIDS RES, V44, pD710, DOI 10.1093/nar/gkv1157
Ye CX, 2016, SCI REP-UK, V6, DOI 10.1038/srep31900
Ye CX, 2012, BMC BIOINFORMATICS, V13, DOI 10.1186/1471-2105-13-S6-S1
Yokoyama WM, 2003, NAT REV IMMUNOL, V3, P304, DOI 10.1038/nri1055
Zhang GJ, 2013, SCIENCE, V339, P456, DOI 10.1126/science.1230835
Zhang Q, 2017, PLOS ONE, V12, DOI 10.1371/journal.pone.0182866
Zhou P, 2016, P NATL ACAD SCI USA, V113, P2696, DOI 10.1073/pnas.1518240113
NR 85
TC 158
Z9 176
U1 1
U2 44
PU CELL PRESS
PI CAMBRIDGE
PA 50 HAMPSHIRE ST, FLOOR 5, CAMBRIDGE, MA 02139 USA
SN 0092-8674
EI 1097-4172
J9 CELL
JI Cell
PD MAY 17
PY 2018
VL 173
IS 5
BP 1098
EP +

DI 10.1016/j.cell.2018.03.070
PG 31
WC Biochemistry & Molecular Biology; Cell Biology
WE Science Citation Index Expanded (SCI-EXPANDED)
SC Biochemistry & Molecular Biology; Cell Biology
GA GG1UA
UT WOS:000432471700008
PM 29706541
OA Green Published, Bronze
DA 2024-10-21
ER

PT J
AU Banerjee, A
   Misra, V
   Schountz, T
   Baker, ML
AF Banerjee, Arinjay
   Misra, Vikram
   Schountz, Tony
   Baker, Michelle L.
TI Tools to study pathogen-host interactions in bats
SO VIRUS RESEARCH
LA English
DT Article
DE Bats; Chiroptera; Viral reservoirs; Virus isolation; Cell lines;
   Host-pathogen interaction
ID CELL-LINE; RESERVOIR HOSTS; INFLUENZA-A; FRUIT BAT; INTERFERON-BETA;
   PTEROPUS-ALECTO; VIRUS; ESTABLISHMENT; REPLICATION; EVOLUTION
AB Bats are natural reservoirs for a variety of emerging viruses that cause significant disease in humans and domestic animals yet rarely cause clinical disease in bats. The co-evolutionary history of bats with viruses has been hypothesized to have shaped the bat-virus relationship, allowing both to exist in equilibrium. Progress in understanding bat-virus interactions and the isolation of bat-borne viruses has been accelerated in recent years by the development of susceptible bat cell lines. Viral sequences similar to severe acute respiratory syndrome corona virus (SARS-CoV) have been detected in bats, and filoviruses such as Marburg virus have been isolated from bats, providing definitive evidence for the role of bats as the natural host reservoir. Although viruses can be readily detected in bats using molecular approaches, virus isolation is far more challenging. One of the limitations in using traditional culture systems from non-reservoir species is that cell types and culture conditions may not be compatible for isolation of bat-borne viruses. There is, therefore, a need to develop additional bat cell lines that correspond to different cell types, including less represented cell types such as immune cells, and culture them under more physiologically relevant conditions to study virus host interactions and for virus isolation. In this review, we highlight the current progress in understanding bat-virus interactions in bat cell line systems and some of the challenges and limitations associated with cell lines. Future directions to address some of

these challenges to better understand host-pathogen interactions in these intriguing mammals are also discussed, not only in relation to viruses but also other pathogens carried by bats including bacteria and fungi.
C1 [Banerjee, Arinjay; Misra, Vikram] Univ Saskatchewan, Dept Vet Microbiol, Western Coll Vet Med, Saskatoon, SK, Canada.
[Schountz, Tony] Colorado State Univ, Arthropod Borne & Infect Dis Lab, Dept Microbiol Immunol & Pathol, Ft Collins, CO 80523 USA.
[Baker, Michelle L.] CSIRO, Australian Anim Hlth Lab, Hlth & Biosecur Business Unit, Geelong, Vic, Australia.
C3 University of Saskatchewan; Colorado State University; Commonwealth
Scientific & Industrial Research Organisation (CSIRO)
RP Baker, ML (corresponding author), CSIRO, Australian Anim Hlth Lab, Hlth & Biosecur Business Unit, Geelong, Vic, Australia.
EM michelle.baker@csiro.au
RI Baker, Michelle/AAX-9171-2020; Misra, Vikram/AAC-4266-2020; Banerjee,
Arinjay/L-7514-2019; Baker, Michelle/C-9694-2013
OI Banerjee, Arinjay/0000-0002-2821-8357; Baker,
Michelle/0000-0002-7993-9971; Misra, Vikram/0000-0001-6818-7156
FU Saskatchewan Innovation and Opportunity scholarship; Department of
Veterinary Microbiology at the University of Saskatchewan, Canada;
Natural Sciences and Engineering Research Council of Canada (NSERC)
FX AB is funded through a Saskatchewan Innovation and Opportunity
scholarship and the Department of Veterinary Microbiology at the
University of Saskatchewan, Canada. VM is funded through a Discovery
Grant awarded by the Natural Sciences and Engineering Research Council
of Canada (NSERC).
CR Ali SH, 2001, SEMIN CANCER BIOL, V11, P15, DOI 10.1006/scbi.2000.0342
Alves DMCC, 2014, PLOS ONE, V9, DOI 10.1371/journal.pone.0107395
Avena CV, 2016, FRONT MICROBIOL, V7, DOI 10.3389/fmicb.2016.01753
Baker ML, 2013, ZOONOSES PUBLIC HLTH, V60, P104, DOI 10.1111/j.1863-2378.2012.01528.x
BANERJEE A, 2017, SCI REP UK, V7
Banerjee A, 2016, J VIROL METHODS, V237, P166, DOI 10.1016/j.jviromet.2016.09.008
Banskar S, 2016, SCI REP-UK, V6, DOI 10.1038/srep36948
Barr JA, 2012, J GEN VIROL, V93, P2590, DOI 10.1099/vir.0.045385-0
Biesold SE, 2011, PLOS ONE, V6, DOI 10.1371/journal.pone.0028131
Boire N, 2016, PLOS ONE, V11, DOI 10.1371/journal.pone.0148473
Cai Yanhong, 2014, J INVESTMENT RES, V2014, P4
Calisher CH, 2006, CLIN MICROBIOL REV, V19, P531, DOI 10.1128/CMR.00017-06
Cogswell-Hawkinson A, 2012, J VIROL, V86, P5791, DOI 10.1128/JVI.00201-12
Combe M, 2014, PLOS PATHOG, V10, DOI 10.1371/journal.ppat.1003855

Crameri G, 2009, PLOS ONE, V4, DOI 10.1371/journal.pone.0008266
Davis AR, 2013, PLOS ONE, V8, DOI [10.1371/journal.pone.0055939, 10.1371/journal.pone.0064808]
Davis A, 2012, VECTOR-BORNE ZOONOT, V12, P55, DOI 10.1089/vbz.2011.0674
Dlugolenski D, 2013, INFLUENZA OTHER RESP, V7, P900, DOI 10.1111/irv.12128
DOWNS WG, 1963, AM J TROP MED HYG, V12, P640, DOI 10.4269/ajtmh.1963.12.640
Eckerle I, 2014, PLOS ONE, V9, DOI 10.1371/journal.pone.0084679
Frank C, 2014, PLOS ONE, V9, DOI 10.1371/journal.pone.0095175
Gómez JMM, 2016, SCI REP-UK, V6, DOI 10.1038/srep37796
Govorkova EA, 1996, J VIROL, V70, P5519, DOI 10.1128/JVI.70.8.5519-5524.1996
GRAVES DC, 1976, CANCER RES, V36, P4152
Halpin K, 2011, AM J TROP MED HYG, V85, P946, DOI 10.4269/ajtmh.2011.10-0567
Hamm PS, 2017, APPL ENVIRON MICROB, V83, DOI 10.1128/AEM.03057-16
Hare D, 2016, VIRUSES-BASEL, V8, DOI 10.3390/v8110297
Hartwig A., 2015, NEW HORIZ TRANSL MED, V2, P128
Haydon DT, 2002, EMERG INFECT DIS, V8, P1468
HE X, 2014, PLOS ONE, V9
He YL, 2009, THERIOGENOLOGY, V71, P1417, DOI 10.1016/j.theriogenology.2009.01.012
Hoffmann M, 2016, PLOS ONE, V11, DOI 10.1371/journal.pone.0152134
Hoffmann M, 2013, PLOS ONE, V8, DOI 10.1371/journal.pone.0072942
Host KM, 2016, MSPHERE, V1, DOI 10.1128/mSphere.00016-16
Jordan I, 2012, VIRUSES-BASEL, V4, P889, DOI 10.3390/v4050889
Jordan I, 2009, VIRUS RES, V145, P54, DOI 10.1016/j.virusres.2009.06.007
Knudsen GR, 2013, NORTHWEST SCI, V87, P292, DOI 10.3955/046.087.0401
Krähling V, 2010, PLOS NEGLECT TROP D, V4, DOI 10.1371/journal.pntd.0000802
Krüger N, 2013, J VIROL, V87, P13889, DOI 10.1128/JVI.02458-13
Lau SKP, 2005, P NATL ACAD SCI USA, V102, P14040, DOI 10.1073/pnas.0506735102
Lei M, 2016, SCI REP-UK, V6, DOI 10.1038/srep27726
LEMM JA, 1990, J VIROL, V64, P3001, DOI 10.1128/JVI.64.6.3001-3011.1990
Leroy EM, 2005, NATURE, V438, P575, DOI 10.1038/438575a
Li JJ, 2015, GENE, V561, P115, DOI 10.1016/j.gene.2015.02.013
Li WD, 2005, SCIENCE, V310, P676, DOI 10.1126/science.1118391
Liang XZ, 2011, PLOS PATHOG, V7, DOI 10.1371/journal.ppat.1002220
Liang YZ, 2015, VIROL SIN, V30, P425, DOI 10.1007/s12250-015-3668-2
Luis AD, 2013, P ROY SOC B-BIOL SCI, V280, DOI 10.1098/rspb.2012.2753
Malmlov A, 2017, PLOS ONE, V12, DOI 10.1371/journal.pone.0185308
Marsh GA, 2012, PLOS PATHOG, V8, DOI 10.1371/journal.ppat.1002836
McColl KA, 2002, AUST VET J, V80, P636, DOI 10.1111/j.1751-0813.2002.tb10973.x
Miller MR, 2016, J INFECT DIS, V214, pS297, DOI 10.1093/infdis/jiw199

Misra V, 2009, J GEN VIROL, V90, P2015, DOI 10.1099/vir.0.010694-0
Moratelli R, 2015, MEM I OSWALDO CRUZ, V110, P1
Moreira-Soto A, 2017, COMP IMMUNOL MICROB, V50, P101, DOI 10.1016/j.cimid.2016.12.004
Mourya DT, 2013, INDIAN J MED RES, V138, P224
Munster VJ, 2016, SCI REP-UK, V6, DOI 10.1038/srep21878
Negredo A, 2011, PLOS PATHOG, V7, DOI 10.1371/journal.ppat.1002304
Nepelska M, 2017, SCI REP-UK, V7, DOI 10.1038/srep43199
Ng M, 2015, ELIFE, V4, DOI 10.7554/eLife.11785
O'Leary MA, 2013, SCIENCE, V339, P662, DOI 10.1126/science.1229237
O'Shea TJ, 2014, EMERG INFECT DIS, V20, P741, DOI 10.3201/eid2005.130539
Olival KJ, 2017, NATURE, V546, P646, DOI 10.1038/nature22975
Olival KJ, 2014, VIRUSES-BASEL, V6, P1759, DOI 10.3390/v6041759
Omatsu T, 2008, VET IMMUNOL IMMUNOP, V124, P169, DOI 10.1016/j.vetimm.2008.03.004
OTSUKI K, 1979, ARCH VIROL, V60, P249, DOI 10.1007/BF01317496
Plowright RK, 2015, P ROY SOC B-BIOL SCI, V282, DOI 10.1098/rspb.2014.2124
Poole DS, 2014, J VIROL, V88, P12572, DOI 10.1128/JVI.01857-14
Prescott J, 2017, ANTIVIR RES, V137, P1, DOI 10.1016/j.antiviral.2016.11.002
Qiu XG, 2014, J VIROL, V88, P12703, DOI 10.1128/JVI.01643-14
Sandekian V, 2013, VIRUS RES, V173, P327, DOI 10.1016/j.virusres.2013.01.010
Sayler KA, 2014, PLOS ONE, V9, DOI 10.1371/journal.pone.0115769
Schountz T, 2017, FRONT IMMUNOL, V8, DOI 10.3389/fimmu.2017.01098
Schountz T, 2014, VIRUSES-BASEL, V6, P4880, DOI 10.3390/v6124880
Schuh AJ, 2017, NAT COMMUN, V8, DOI 10.1038/ncomms14446
Shabman RS, 2016, MSPHERE, V1, DOI 10.1128/mSphere.00070-15
Simmons NB, 2008, NATURE, V451, P818, DOI 10.1038/nature06549
Subudhi S, 2017, J GEN VIROL, V98, P2297, DOI 10.1099/jgv.0.000898
Swanepoel R, 2007, EMERG INFECT DIS, V13, P1847, DOI 10.3201/eid1312.071115
Tait J, 2014, PLOS ONE, V9, DOI 10.1371/journal.pone.0109810
Teeling EC, 2005, SCIENCE, V307, P580, DOI 10.1126/science.1105113
Teeling EC, 2016, SPRINGER HANDB AUDIT, V54, P25, DOI 10.1007/978-1-4939-3527-7_2
Virtue ER, 2011, PLOS ONE, V6, DOI 10.1371/journal.pone.0022488
Warnecke L, 2012, P NATL ACAD SCI USA, V109, P6999, DOI 10.1073/pnas.1200374109
Wei HY, 2017, SCI REP-UK, V7, DOI 10.1038/s41598-017-03318-3
Widagdo W, 2017, SCI REP-UK, V7, DOI 10.1038/s41598-017-01290-6
Wynne JW, 2016, J IMMUNOL, V196, P4468, DOI 10.4049/jimmunol.1502062
Xie X, 2015, APPL MICROBIOL BIOT, V99, P9135, DOI 10.1007/s00253-015-6794-8
Young PL, 1996, EMERG INFECT DIS, V2, P239, DOI 10.3201/eid0203.960315

Zhang HJ, 2012, J VIROL, V86, P8014, DOI 10.1128/JVI.00723-12
Zhang Q, 2017, PLOS ONE, V12, DOI 10.1371/journal.pone.0182866
Zhou P, 2016, SCI REP-UK, V6, DOI 10.1038/srep38597
Zhou P, 2016, P NATL ACAD SCI USA, V113, P2696, DOI 10.1073/pnas.1518240113
Zhou P, 2013, DEV COMP IMMUNOL, V40, P240, DOI 10.1016/j.dci.2013.03.006
Zhou P, 2011, J IMMUNOL, V186, P3138, DOI 10.4049/jimmunol.1003115
Zukal J, 2014, PLOS ONE, V9, DOI 10.1371/journal.pone.0097224
NR 96
TC 23
Z9 29
U1 0
U2 53
PU ELSEVIER
PI AMSTERDAM
PA RADARWEG 29, 1043 NX AMSTERDAM, NETHERLANDS
SN 0168-1702
EI 1872-7492
J9 VIRUS RES
JI Virus Res.
PD MAR 15
PY 2018
VL 248
BP 5
EP 12
DI 10.1016/j.virusres.2018.02.013
PG 8
WC Virology
WE Science Citation Index Expanded (SCI-EXPANDED)
SC Virology
GA GD8NT
UT WOS:000430769900002
PM 29454637
OA Green Published
DA 2024-10-21
ER

PT J
AU Zhou, P
Cowled, C
Marsh, GA
Shi, ZL
Wang, LF
Baker, ML
AF Zhou, Peng
Cowled, Chris
Marsh, Glenn A.
Shi, Zhengli
Wang, Lin-Fa
Baker, Michelle L.
TI Type III IFN Receptor Expression and Functional Characterisation in the

Pteropid Bat, <i>Pteropus alecto</i>
SO PLOS ONE
LA English
DT Article
ID IL-10 RECEPTOR-2; CYTOKINE; INTERLEUKIN-22; INTERFERONS; ALIGNMENT;
IMMUNITY; COMPLEX; FAMILY; CELLS; CHAIN
AB Bats are rich reservoir hosts for a variety of viruses, many of which are capable of spillover to other susceptible mammals with lethal consequences. The ability of bats to remain asymptomatic to viral infection may be due to the rapid control of viral replication very early in the immune response through innate antiviral mechanisms. Type I and III interferons (IFNs) represent the first line of defence against viral infection in mammals, with both families of IFNs present in pteropid bats. To obtain further insight into the type III IFN system in bats, we describe the characterization of the type III IFN receptor (IFN lambda R) in the black flying fox, P. alecto with the characterization of IFN lambda R1 and IL10R2 genes that make up the type III IFN receptor complex. The bat IFN lambda R complex has a wide tissue distribution and at the cellular level, both epithelial and immune cells are responsive to IFN-lambda treatment. Furthermore, we demonstrate that the bat IFN lambda R1 chain acts as a functional receptor. To our knowledge, this report represents the first description of an IFN receptor in any species of bat. The responsiveness of bat cells to IFN-lambda support a role for the type III IFN system by epithelial and immune cells in bats.
C1 [Zhou, Peng; Cowled, Chris; Marsh, Glenn A.; Wang, Lin-Fa; Baker, Michelle L.] CSIRO Livestock Ind, Australian Anim Hlth Lab, Geelong, Vic, Australia.
[Zhou, Peng; Shi, Zhengli] Chinese Acad Sci, Wuhan Inst Virol, State Key Lab Virol, Wuhan, Peoples R China.
[Wang, Lin-Fa] Univ Melbourne, Dept Microbiol & Immunol, Parkville, Vic 3052, Australia.
[Baker, Michelle L.] Univ New Mexico, Dept Biol, Ctr Evolutionary & Theoret Immunol, Albuquerque, NM 87131 USA.
C3 Commonwealth Scientific & Industrial Research Organisation (CSIRO);
Chinese Academy of Sciences; Wuhan Institute of Virology, CAS;
University of Melbourne; University of New Mexico
RP Zhou, P (corresponding author), CSIRO Livestock Ind, Australian Anim Hlth Lab, Geelong, Vic, Australia.
EM Michelle.Baker@csiro.au
RI lu, yuan/JZD-0832-2024; Zhou, Peng/I-8578-2015; Shi,
Zheng-Li/A-1013-2013; Marsh, Glenn Andrew/A-5418-2013; Cowled,
Christopher/H-5308-2013; Baker, Michelle/C-9694-2013
OI Shi, Zheng-Li/0000-0001-8089-163X; Marsh, Glenn
Andrew/0000-0002-3469-1837; Wang, Lin-Fa/0000-0003-2752-0535; Cowled,
Christopher/0000-0002-1969-9371; Baker, Michelle/
0000-0002-7993-9971
FU Commonwealth Scientific and Industrial Research Organisation; National
Institutes of Health of the National Center for Research

Resources
[P20RR018754]; Chinese Academy of Sciences
FX This work was supported by a Commonwealth Scientific and Industrial
Research Organisation Chief Executive Officer Science Leaders award to
L-F.W, a grant from the National Institutes of Health (grant no.
P20RR018754) from the Institutional Development Award (IDeA) programme
of the National Center for Research Resources to M. L. B., and a
Scholarship from the Chinese Academy of Sciences to P.Z. The funders had
no role in study design, data collection and analysis, decision to
publish, or preparation of the manuscript.
CR ALTSCHUL SF, 1990, J MOL BIOL, V215, P403, DOI 10.1016/S0022-2836(05)80360-2
Ank N, 2008, J IMMUNOL, V180, P2474, DOI 10.4049/jimmunol.180.4.2474
Ank N, 2009, BIOFACTORS, V35, P82, DOI 10.1002/biof.19
Baker ML, 2010, IMMUNOGENETICS, V62, P173, DOI 10.1007/s00251-010-0425-4
Calisher CH, 2006, CLIN MICROBIOL REV, V19, P531, DOI 10.1128/CMR.00017-06
Cowled C, 2011, DEV COMP IMMUNOL, V35, P7, DOI 10.1016/j.dci.2010.07.006
Crameri G, 2009, PLOS ONE, V4, DOI 10.1371/journal.pone.0008266
Donnelly RP, 2004, J LEUKOCYTE BIOL, V76, P314, DOI 10.1189/jlb.0204117
Dumoutier L, 2004, J BIOL CHEM, V279, P32269, DOI 10.1074/jbc.M404789200
Kotenko SV, 2003, NAT IMMUNOL, V4, P69, DOI 10.1038/ni875
Langer JA, 2004, CYTOKINE GROWTH F R, V15, P33, DOI 10.1016/j.cytogfr.2003.10.001
Lasfar A, 2006, CANCER RES, V66, P4468, DOI 10.1158/0008-5472.CAN-05-3653
Li J, 2004, INT IMMUNOPHARMACOL, V4, P693, DOI 10.1016/j.intimp.2004.01.010
Moore KW, 2001, ANNU REV IMMUNOL, V19, P683, DOI 10.1146/annurev.immunol.19.1.683
Pestka S, 2004, IMMUNOL REV, V202, P8, DOI 10.1111/j.0105-2896.2004.00204.x
Qi ZT, 2010, J IMMUNOL, V184, P5038, DOI 10.4049/jimmunol.0903374
Sheikh F, 2004, J IMMUNOL, V172, P2006, DOI 10.4049/jimmunol.172.4.2006
Sheppard P, 2003, NAT IMMUNOL, V4, P63, DOI 10.1038/ni873
Sommereyns C, 2008, PLOS PATHOG, V4, DOI 10.1371/journal.ppat.1000017
THOMPSON JD, 1994, NUCLEIC ACIDS RES, V22, P4673, DOI 10.1093/nar/22.22.4673
Witte K, 2009, GENES IMMUN, V10, P702, DOI 10.1038/gene.2009.72
Witte K, 2010, CYTOKINE GROWTH F R, V21, P237, DOI 10.1016/j.cytogfr.2010.04.002
Wolk K, 2004, IMMUNITY, V21, P241, DOI 10.1016/

j.immuni.2004.07.007
   Wolk K, 2006, CYTOKINE GROWTH F R, V17, P367, DOI 10.1016/
j.cytogfr.2006.09.001
   Zhou P, 2011, J IMMUNOL, V186, P3138, DOI 10.4049/
jimmunol.1003115
NR 25
TC 39
Z9 42
U1 0
U2 20
PU PUBLIC LIBRARY SCIENCE
PI SAN FRANCISCO
PA 185 BERRY ST, STE 1300, SAN FRANCISCO, CA 94107 USA
SN 1932-6203
J9 PLOS ONE
JI PLoS One
PD SEP 27
PY 2011
VL 6
IS 9
AR e25385
DI 10.1371/journal.pone.0025385
PG 8
WC Multidisciplinary Sciences
WE Science Citation Index Expanded (SCI-EXPANDED)
SC Science & Technology - Other Topics
GA 834BW
UT WOS:000295933700029
PM 21980438
OA Green Published, gold
DA 2024-10-21
ER

PT J
AU Bowen, RA
   O'Shea, TJ
   Shankar, V
   Neubaum, MA
   Neubaum, DJ
   Rupprecht, CE
AF Bowen, Richard A.
   O'Shea, Thomas J.
   Shankar, Vidya
   Neubaum, Melissa A.
   Neubaum, Daniel J.
   Rupprecht, Charles E.
TI PREVALENCE OF NEUTRALIZING ANTIBODIES TO RABIES VIRUS IN SERUM OF
SEVEN
   SPECIES OF INSECTIVOROUS BATS FROM COLORADO AND NEW MEXICO,
UNITED
   STATES
SO JOURNAL OF WILDLIFE DISEASES
LA English
DT Article

DE Bats; Chiroptera; Colorado; New Mexico; rabies; serology;
virus-neutralizing antibodies
ID BIG BROWN BATS; EPTESICUS-FUSCUS; NORTH-AMERICA; TADARIDA-BRASILIENSIS;
EXPOSURE; ECOLOGY; HISTORY; ANESTHESIA; INFECTION; SURVIVAL
AB We determined the presence of rabies-virus-neutralizing antibodies (RVNA) in serum of 721 insectivorous bats of seven species captured, sampled, and released in Colorado and New Mexico, United States in 2003-2005. A subsample of 160 bats was tested for rabies-virus RNA in saliva. We sampled little brown bats (Myotis lucifugus) at two maternity roosts in Larimer County, Colorado; big brown bats (Eptesicus fuscus) at three maternity roosts in Morgan County, Colorado; and big brown bats at five maternity roosts in Larimer County. We also sampled hoary bats (Lasiurus cinereus) and silver-haired bats (Lasionycteris noctivagans) captured while drinking or foraging over water in Bernalillo County, New Mexico and at various locations in Larimer County. Big brown bats, little brown bats, long-legged myotis (Myotis volans), long-eared nip:A:is (Myotis evotis), and fringed myotis (Myotis thysanodes) were also sampled over water in Larimer County. All species except long-eared myotis included individuals with RVNA, with prevalences ranging from 7% in adult female silver-haired bats to 32% in adult female hoary bats. None of the bats had detectable rabies-virus RNA in oropharyngeal swabs, including 51 bats of 5 species that had RVNA in serum. Antibody-positive bats were present in nine of the 10 maternity colonies sampled. These data suggest that wild bats are commonly exposed to rabies virus and develop a humoral immune response suggesting some degree of viral replication, but many infections fail to progress to clinical disease.
C1 [Bowen, Richard A.; Shankar, Vidya; Neubaum, Melissa A.; Neubaum, Daniel J.] Colorado State Univ, Dept Biomed Sci, Ft Collins, CO 80523 USA.
[O'Shea, Thomas J.] US Geol Survey, Ft Collins Sci Ctr, Ft Collins, CO 80526 USA.
[Shankar, Vidya; Rupprecht, Charles E.] Ctr Dis Control & Prevent, Atlanta, GA 30333 USA.
C3 Colorado State University; United States Department of the Interior;
United States Geological Survey; Centers for Disease Control &
Prevention - USA
RP Bowen, RA (corresponding author), Colorado State Univ, Dept Biomed Sci, Ft Collins, CO 80523 USA.
EM richard.bowen@colostate.edu
RI Rupprecht, Charles/AAI-1544-2020; O'Shea, Thomas/AAY-5848-2021
FU National Science Foundation EID grant [0094959]; US Geological Survey;
Research and Policy for Infectious Disease Dynamics (RAPIDD) program of
the Science and Technology Directorate (US Department of Homeland
Security); Fogarty International Center (National Institutes of Health);
Emerging Frontiers; Direct For Biological Sciences [0094959] Funding
Source: National Science Foundation

FX We thank the following for assistance in capturing and sampling bats
during 2003-2005: S. Almon, T. Barnes, J. Benson, J. Boland, S. Cooper,
T. Dawes, L. Ellison, D. Emptage, M. Hayes, B. Iannone, E. Kennedy, R.
Kerscher, J. LaPlante, G. Nance, S. Neils, C. Newby, V. Price, C.
Reynolds, L. Taraba, J. Tharp, and M. Vrabely. C. Andrews provided
technical support in rabies-virus serology. P. Cryan and E. Valdez
showed us productive capture sites in New Mexico and worked with us in
the field. Financial support was provided via National Science
Foundation EID grant 0094959 to Colorado State University, and by the US
Geological Survey. R.A.B. and T.J.O. were supported by the Research and
Policy for Infectious Disease Dynamics (RAPIDD) program of the Science
and Technology Directorate (US Department of Homeland Security) and the
Fogarty International Center (National Institutes of Health) during
preparation of the manuscript. Any use of trade, firm, or product names
is for descriptive purposes only and does not imply endorsement by the
US Government. The findings and conclusions in this report are those of
the authors and do not necessarily represent the views of the Centers
for Disease Control; they do represent the views of the US Geological
Survey.
CR [Anonymous], MAMMALIAN SPECIES
[Anonymous], 2007, Rabies
[Anonymous], MAMMALIAN SPECIES
Badrane H, 2001, J VIROL, V75, P8096, DOI 10.1128/JVI.75.17.8096-8104.2001
Black D, 1986, J Fla Med Assoc, V73, P517
Brass D.A., 1994, RABIES BATS NATURAL
CONSTANTINE DG, 1968, PUBLIC HEALTH REP, V83, P303, DOI 10.2307/4593275
CONSTANTINE DG, 1979, J WILDLIFE DIS, V15, P347, DOI 10.7589/0090-3558-15.2.347
Cryan PM, 2003, J MAMMAL, V84, P579, DOI 10.1644/1545-1542(2003)084<0579:SDOMTB>2.0.CO;2
Davis AD, 2007, J INFECT DIS, V195, P1144, DOI 10.1086/512616
Davis PL, 2006, INFECT GENET EVOL, V6, P464, DOI 10.1016/j.meegid.2006.02.007
Ellison LE, 2006, J WILDLIFE DIS, V42, P849, DOI 10.7589/0090-3558-42.4.849
FOLLMANN EH, 1994, EPIDEMIOL INFECT, V113, P137, DOI 10.1017/

S0950268800051554
Franka R, 2006, J GEN VIROL, V87, P2309, DOI 10.1099/vir.0.81822-0
Hanlon C.A., 2007, RABIES, V2nd, P201
Hughes GJ, 2005, J GEN VIROL, V86, P1467, DOI 10.1099/vir.0.80710-0
Jackson FR, 2008, J WILDLIFE DIS, V44, P612, DOI 10.7589/0090-3558-44.3.612
Kurta A., 1990, Mammalian Species, V356, P1
Messenger SL, 2002, CLIN INFECT DIS, V35, P738, DOI 10.1086/342387
Messenger SL, 2003, BAT ECOLOGY, P622
Morimoto K, 1996, P NATL ACAD SCI USA, V93, P5653, DOI 10.1073/pnas.93.11.5653
Neubaum MA, 2007, J MAMMAL, V88, P1230, DOI 10.1644/06-MAMM-A-228R1.1
O'Shea Thomas J., 2004, P297
O'Shea TJ, 2011, URBAN ECOSYST, V14, P665, DOI 10.1007/s11252-011-0182-7
O'Shea TJ, 2003, WILDLIFE SOC B, V31, P16
Pape WJ, 2003, WILDLIFE SOC B, V31, P16
Pape WJ, 2004, SAMPLING RARE ELUSIV, P297
Salas-Rojas M, 2004, T ROY SOC TROP MED H, V98, P577, DOI 10.1016/j.trstmh.2003.10.019
SCATTERDAY JE, 1954, J AM VET MED ASSOC, V124, P125
Shankar V, 2004, J WILDLIFE DIS, V40, P403, DOI 10.7589/0090-3558-40.3.403
Smith J. S., 1996, P181
STEECE R, 1989, J WILDLIFE DIS, V25, P490, DOI 10.7589/0090-3558-25.4.490
Streicker DG, 2012, P ROY SOC B-BIOL SCI, V279, P3384, DOI 10.1098/rspb.2012.0538
Streicker DG, 2010, SCIENCE, V329, P676, DOI 10.1126/science.1188836
SULKIN S E, 1954, Tex State J Med, V50, P620
TRIMARCHI CV, 1977, J WILDLIFE DIS, V13, P366, DOI 10.7589/0090-3558-13.4.366
Turmelle AS, 2010, J GEN VIROL, V91, P2360, DOI 10.1099/vir.0.020073-0
Turmelle AS, 2010, VECTOR-BORNE ZOONOT, V10, P165, DOI 10.1089/vbz.2008.0163
Venters H D., 1954, American Journal of Public Health, V44, P182
Wimsatt J, 2005, J WILDLIFE DIS, V41, P87, DOI 10.7589/0090-3558-41.1.87
Wright E, 2008, J GEN VIROL, V89, P2204, DOI 10.1099/vir.0.2008/000349-0
Yan X, 2002, J NEUROVIROL, V7, P518
NR 42
TC 7
Z9 10
U1 0
U2 33
PU WILDLIFE DISEASE ASSOC, INC
PI LAWRENCE

PA 810 EAST 10TH ST, LAWRENCE, KS 66044-8897 USA
SN 0090-3558
J9 J WILDLIFE DIS
JI J. Wildl. Dis.
PD APR
PY 2013
VL 49
IS 2
BP 367
EP 374
DI 10.7589/2012-05-124
PG 8
WC Veterinary Sciences
WE Science Citation Index Expanded (SCI-EXPANDED)
SC Veterinary Sciences
GA 128YP
UT WOS:000317805900017
PM 23568912
DA 2024-10-21
ER

PT J
AU Schountz, T
   Baker, ML
   Butler, J
   Munster, V
AF Schountz, Tony
   Baker, Michelle L.
   Butler, John
   Munster, Vincent
TI Immunological Control of Viral Infections in Bats and the Emergence of
   Viruses Highly Pathogenic to Humans
SO FRONTIERS IN IMMUNOLOGY
LA English
DT Article
DE bats; Chiroptera; zoonosis; antibody repertoire; emerging infectious
   disease; virus ecology
ID RESPIRATORY SYNDROME CORONAVIRUS; EGYPTIAN FRUIT BATS; SIN-NOMBRE-VIRUS;
   MOUSE PEROMYSCUS-MANICULATUS; INACTIVATED RABIES VACCINE; NIPAH VIRUS;
   MARBURG VIRUS; IMMUNE-RESPONSES; ROUSETTUS-AEGYPTIACUS; SOMATIC
   HYPERMUTATION
AB Bats are reservoir hosts of many important viruses that cause substantial disease in humans, including coronaviruses, filoviruses, lyssaviruses, and henipaviruses. Other than the lyssaviruses, they do not appear to cause disease in the reservoir bats, thus an explanation for the dichotomous outcomes of infections of humans and bat reservoirs remains to be determined. Bats appear to have a few unusual features that may account for these differences, including evidence of constitutive interferon (IFN) activation and greater combinatorial diversity in immunoglobulin genes that do not undergo

substantial affinity maturation. We propose these features may, in part, account for why bats can host these viruses without disease and how they may contribute to the highly pathogenic nature of bat-borne viruses after spillover into humans. Because of the constitutive IFN activity, bat-borne viruses may be shed at low levels from bat cells. With large naive antibody repertoires, bats may control the limited virus replication without the need for rapid affinity maturation, and this may explain why bats typically have low antibody titers to viruses. However, because bat viruses have evolved in high IFN environments, they have enhanced countermeasures against the IFN response. Thus, upon infection of human cells, where the IFN response is not constitutive, the viruses overwhelm the IFN response, leading to abundant virus replication and pathology.
C1 [Schountz, Tony] Colorado State Univ, Dept Microbiol Immunol & Pathol, Arthropod Borne & Infect Dis Lab, Ft Collins, CO 80523 USA.
[Baker, Michelle L.] CSIRO, Australian Anim Hlth Lab, Hlth & Biosecur Business Unit, Geelong, Vic, Australia.
[Butler, John] Univ Iowa, Dept Microbiol, Carver Coll Med, Iowa City, IA 52242 USA.
[Munster, Vincent] NIH, Virus Ecol Unit, Rocky Mt Labs, Hamilton, MT USA.
C3 Colorado State University; Commonwealth Scientific & Industrial Research
Organisation (CSIRO); University of Iowa; National Institutes of Health
(NIH) - USA
RP Schountz, T (corresponding author), Colorado State Univ, Dept Microbiol Immunol & Pathol, Arthropod Borne & Infect Dis Lab, Ft Collins, CO 80523 USA.
EM tony.schountz@colostate.edu
RI Baker, Michelle/AAX-9171-2020; Baker, Michelle/C-9694-2013; Munster,
Vincent/I-7607-2018
OI Baker, Michelle/0000-0002-7993-9971; Munster,
Vincent/0000-0002-2288-3196
FU National Institute of Allergy and Infectious Diseases, National
Institutes of Health
FX The authors thank Charles H. Calisher for review of the manuscript and
Jacalyn S. Strong for support. VM is supported by the Intramural
Research Program of the National Institute of Allergy and Infectious
Diseases, National Institutes of Health.
CR Ahn M, 2016, SCI REP-UK, V6, DOI 10.1038/srep21722
Amman BR, 2015, J WILDLIFE DIS, V51, P113, DOI 10.7589/2014-08-198
Anthony SJ, 2013, MBIO, V4, DOI 10.1128/mBio.00598-13
Baker ML, 2013, ZOONOSES PUBLIC HLTH, V60, P104, DOI 10.1111/j.1863-2378.2012.01528.x
Baker ML, 2010, IMMUNOGENETICS, V62, P173, DOI 10.1007/s00251-010-0425-4
Banadyga L, 2016, J MOL BIOL, V428, P3449, DOI 10.1016/j.jmb.2016.05.008
Basler CF, 2012, CURR TOP MICROBIOL, V359, P123, DOI

10.1007/82_2012_209
Bean AGD, 2013, NAT REV IMMUNOL, V13, P851, DOI 10.1038/nri3551
Benton MJ, 2003, SCIENCE, V300, P1698, DOI 10.1126/science.1077795
Blachly JS, 2015, P NATL ACAD SCI USA, V112, P4322, DOI 10.1073/pnas.1503587112
Botten J, 2000, P NATL ACAD SCI USA, V97, P10578, DOI 10.1073/pnas.180197197
Botten J, 2003, J VIROL, V77, P1540, DOI 10.1128/JVI.77.2.1540-1550.2002
Bratsch S, 2011, DEV COMP IMMUNOL, V35, P421, DOI 10.1016/j.dci.2010.06.004
Brook CE, 2015, TRENDS MICROBIOL, V23, P172, DOI 10.1016/j.tim.2014.12.004
Butler JE, 2012, FRONT IMMUNOL, V3, DOI 10.3389/fimmu.2012.00153
Butler JE, 2011, DEV COMP IMMUNOL, V35, P273, DOI 10.1016/j.dci.2010.08.011
Calisher CH, 2006, CLIN MICROBIOL REV, V19, P531, DOI 10.1128/CMR.00017-06
CHAKRABORTY AK, 1984, DEV COMP IMMUNOL, V8, P415, DOI 10.1016/0145-305X(84)90048-X
Ching S, 2007, J NEUROSCI, V27, P10476, DOI 10.1523/JNEUROSCI.3357-07.2007
Chua KB, 2000, SCIENCE, V288, P1432, DOI 10.1126/science.288.5470.1432
Cogswell-Hawkinson A, 2012, J VIROL, V86, P5791, DOI 10.1128/JVI.00201-12
de Wit E, 2011, PLOS NEGLECT TROP D, V5, DOI 10.1371/journal.pntd.0001432
Drexler JF, 2011, EMERG INFECT DIS, V17, P449, DOI 10.3201/eid1703.100526
Easterbrook JD, 2007, P NATL ACAD SCI USA, V104, P15502, DOI 10.1073/pnas.0707453104
Frieman M, 2007, J VIROL, V81, P9812, DOI 10.1128/JVI.01012-07
Gralinski LE, 2015, J PATHOL, V235, P185, DOI 10.1002/path.4454
Halpin K, 2011, AM J TROP MED HYG, V85, P946, DOI 10.4269/ajtmh.2011.10-0567
Jackson KJL, 2013, FRONT IMMUNOL, V4, DOI 10.3389/fimmu.2013.00263
Jung D, 2006, ANNU REV IMMUNOL, V24, P541, DOI 10.1146/annurev.immunol.23.021704.115830
Kopecky-Bromberg SA, 2007, J VIROL, V81, P548, DOI 10.1128/JVI.01782-06
Lau SKP, 2013, J VIROL, V87, P8638, DOI 10.1128/JVI.01055-13
Lee WT, 2016, DEV COMP IMMUNOL, V65, P114, DOI 10.1016/j.dci.2016.06.024
Lo MK, 2010, VIROLOGY, V404, P78, DOI 10.1016/j.virol.2010.05.005
Luis AD, 2013, P ROY SOC B-BIOL SCI, V280, DOI 10.1098/rspb.2012.2753
Mandl JN, 2015, CELL, V160, P20, DOI 10.1016/j.cell.2014.12.003
Mathieu C, 2012, J VIROL, V86, P10766, DOI 10.1128/JVI.01203-12
Max E., 2013, Fundamental immunology
McGuire A, 2016, VIRUSES-BASEL, V8, DOI 10.3390/v8100286
Miller MR, 2016, J INFECT DIS, V214, pS297, DOI 10.1093/infdis/

jiw199
Neave MJ, 2017, SCI REP-UK, V7, DOI 10.1038/srep41531
Negrete OA, 2005, NATURE, V436, P401, DOI 10.1038/nature03838
O'Shea TJ, 2014, EMERG INFECT DIS, V20, P741, DOI 10.3201/eid2005.130539
Olival KJ, 2017, NATURE, V546, P646, DOI 10.1038/nature22975
Papenfuss AT, 2012, BMC GENOMICS, V13, DOI 10.1186/1471-2164-13-261
Paweska JT, 2015, J INFECT DIS, V212, pS109, DOI 10.1093/infdis/jiv132
Paweska JT, 2012, PLOS ONE, V7, DOI 10.1371/journal.pone.0045479
Peled JU, 2008, ANNU REV IMMUNOL, V26, P481, DOI 10.1146/annurev.immunol.26.021607.090236
Peters C, 2004, J ZOO WILDLIFE MED, V35, P55, DOI 10.1638/03-027
Ramanan P, 2012, P NATL ACAD SCI USA, V109, P20661, DOI 10.1073/pnas.1213559109
Schountz T, 2007, P NATL ACAD SCI USA, V104, P15496, DOI 10.1073/pnas.0707454104
Schountz T, 2014, VIRUSES-BASEL, V6, P4880, DOI 10.3390/v6124880
Schountz T, 2014, VIRUSES-BASEL, V6, P1317, DOI 10.3390/v6031317
Schountz T, 2012, J VIROL, V86, P10015, DOI 10.1128/JVI.06875-11
Schuh AJ, 2017, NAT COMMUN, V8, DOI 10.1038/ncomms14446
Shaw ML, 2004, J VIROL, V78, P5633, DOI 10.1128/JVI.78.11.5633-5641.2004
Shaw TI, 2012, PLOS ONE, V7, DOI 10.1371/journal.pone.0048472
Stavnezer J, 2008, ANNU REV IMMUNOL, V26, P261, DOI 10.1146/annurev.immunol.26.021607.090248
Thornbrough JM, 2016, MBIO, V7, DOI 10.1128/mBio.00258-16
Turmelle AS, 2010, J GEN VIROL, V91, P2360, DOI 10.1099/vir.0.020073-0
Turmelle AS, 2010, J ZOO WILDLIFE MED, V41, P140, DOI 10.1638/2008-0161.1
Valmas C, 2010, PLOS PATHOG, V6, DOI 10.1371/journal.ppat.1000721
Virtue ER, 2011, PLOS ONE, V6, DOI 10.1371/journal.pone.0022488
Virtue ER, 2011, J VIROL, V85, P4031, DOI 10.1128/JVI.02412-10
Volchkova VA, 2015, J INFECT DIS, V212, pS226, DOI 10.1093/infdis/jiv309
Warren TK, 2015, MBIO, V6, DOI 10.1128/mBio.02344-14
Wellehan JFX, 2009, COMP IMMUNOL MICROB, V32, P379, DOI 10.1016/j.cimid.2007.11.002
Wilhelms DB, 2014, J NEUROSCI, V34, P11684, DOI 10.1523/JNEUROSCI.1838-14.2014
Williamson MM, 1998, AUST VET J, V76, P813, DOI 10.1111/j.1751-0813.1998.tb12335.x
Wong KT, 2003, AM J PATHOL, V163, P2127, DOI 10.1016/S0002-9440(10)63569-9
Wynne JW, 2013, PLOS ONE, V8, DOI 10.1371/journal.pone.0052930
YANAGIHARA R, 1985, J VIROL, V55, P34, DOI 10.1128/JVI.55.1.34-38.1985
Zhang APP, 2012, PLOS PATHOG, V8, DOI 10.1371/journal.ppat.1002550
Zhang GJ, 2013, SCIENCE, V339, P456, DOI 10.1126/science.1230835
Zhou P, 2016, P NATL ACAD SCI USA, V113, P2696, DOI 10.1073/pnas.1518240113

   Zhou P, 2011, PLOS ONE, V6, DOI 10.1371/journal.pone.0025385
   Zhou P, 2011, J IMMUNOL, V186, P3138, DOI 10.4049/
jimmunol.1003115
NR 76
TC 79
Z9 89
U1 2
U2 63
PU FRONTIERS MEDIA SA
PI LAUSANNE
PA AVENUE DU TRIBUNAL FEDERAL 34, LAUSANNE, CH-1015, SWITZERLAND
SN 1664-3224
J9 FRONT IMMUNOL
JI Front. Immunol.
PD SEP 11
PY 2017
VL 8
AR 1098
DI 10.3389/fimmu.2017.01098
PG 9
WC Immunology
WE Science Citation Index Expanded (SCI-EXPANDED)
SC Immunology
GA FG2WC
UT WOS:000409989900001
PM 28959255
OA gold, Green Published
DA 2024-10-21
ER

PT J
AU Hanadhita, D
   Rahma, A
   Prawira, AY
   Mayasari, NLPI
   Satyaningtijas, AS
   Hondo, E
   Agungpriyono, S
AF Hanadhita, Desrayni
   Rahma, Anisa
   Prawira, Andhika Yudha
   Mayasari, Ni Luh Putu Ika
   Satyaningtijas, Aryani Sismin
   Hondo, Eiichi
   Agungpriyono, Srihadi
TI The spleen morphophysiology of fruit bats
SO ANATOMIA HISTOLOGIA EMBRYOLOGIA
LA English
DT Article
DE flying foxes; histology; immunology; morphology; physiology;
viral
   reservoir
ID MARGINAL ZONE; IMMUNE-RESPONSES; OXYGEN-TRANSPORT; FLYING FOXES;
VIRUS;

TORPOR; SEAL; SPLENECTOMY; EXTINCTIONS; ADAPTATIONS
AB Spleen is one of the important lymphoid organs with wide variations of morphological and physiological functions according to species. Morphology and function of the spleen in bats, which are hosts to several viral strains without exhibiting clinical symptoms, remain to be fully elucidated. This study aims to examine the spleen morphology of fruit bats associated with their physiological functions. Spleen histological observations were performed in three fruit bats species: Cynopterus titthaecheilus (n = 9), Rousettus leschenaultii (n = 3) and Pteropus vampyrus (n = 3). The spleens of these fruit bats were surrounded by a thin capsule. Red pulp consisted of splenic cord and wide vascular space filled with blood. Ellipsoids in all three studied species were found numerously and adjacent to one another forming macrophages aggregates. White pulp consisted of periarteriolar lymphoid sheaths (PALS), lymphoid follicles and marginal zone. The lymphoid follicle contained a germinal centre and a tingible body macrophage that might reflect an active immune system. The marginal zone was prominent and well developed. This study reports some differences in spleen structure of fruit bats compared to other bat species previously reported and discusses possible physiological implications of the spleen based on its morphology.
C1 [Hanadhita, Desrayni; Rahma, Anisa; Prawira, Andhika Yudha; Satyaningtijas, Aryani Sismin; Agungpriyono, Srihadi] Bogor Agr Univ IPB, Dept Anat Physiol & Pharmacol, Fac Vet Med, Bogor, Indonesia.
[Mayasari, Ni Luh Putu Ika] Bogor Agr Univ IPB, Dept Anim Dis & Vet Publ Hlth, Fac Vet Med, Bogor, Indonesia.
[Hondo, Eiichi] Nagoya Univ, Grad Sch Bioagr Sci, Lab Anim Morphol, Nagoya, Aichi, Japan.
C3 Bogor Agricultural University; Bogor Agricultural University; Nagoya
University
RP Agungpriyono, S (corresponding author), Bogor Agr Univ IPB, Dept Anat Physiol & Pharmacol, Fac Vet Med, Bogor, Indonesia.
EM ysrihadi@apps.ipb.ac.id
RI Satyaningtijas, Aryani/GPK-4817-2022; Rahma, Anisa/GPX-9673-2022; Mayasari, Ni Luh Putu Ika/GPG-4038-2022; agungpriyono, srihadi/AFC-8640-2022
OI agungpriyono, srihadi/0000-0002-8348-5570; Supratikno, Supratikno/0000-0002-1756-1846; Rahma, Anisa/0000-0002-2714-6013; Hanadhita, Desrayni/0000-0002-1681-3749; Prawira, Andhika Yudha/0000-0002-9596-9003
FU Directorate of Research and Community Services, Directorate General of
Research and Development, Ministry of Research, Technology, and Higher
Education Republic of Indonesia, Penelitian Pendidikan Magister Menuju
Doktor untuk
FX Directorate of Research and Community Services, Directorate General of
Research and Development, Ministry of Research, Technology, and Higher
Education Republic of Indonesia, Grant/Award Number: Penelitian

Pendidikan Magister Menuju Doktor untuk
CR Anindita PD, 2015, ARCH VIROL, V160, P1113, DOI 10.1007/s00705-015-2342-1
[Anonymous], 2011, BATS EVOLUTION CONSE
Attia WY, 2007, EGYPT J EXP BIOL ZOO, V3, P151
Bacha W., 2000, Color Atlas of Veterinary Histology, V2nd
Bao HJ, 2009, ANAT REC, V292, P1079, DOI 10.1002/ar.20917
Barclay RMR, 2017, J MAMMAL, V98, P572, DOI 10.1093/jmammal/gyw234
Barthold S. W., 2016, Pathology of laboratory rodents and rabbits, P253
Basri C, 2017, J VET MED SCI, V79, P1615, DOI 10.1292/jvms.17-0222
Bergman R. A., 2000, COMPLETE SPLEEN STRU, P3
BLUE J, 1981, AM J ANAT, V161, P115, DOI 10.1002/aja.1001610202
Bowdler A. J., 2002, COMPLETE SPLEEN STRU, P139
Brendolan A, 2007, BIOESSAYS, V29, P166, DOI 10.1002/bies.20528
Briand S, 2014, NEW ENGL J MED, V371, P1180, DOI 10.1056/NEJMp1409858
Bronte V, 2013, IMMUNITY, V39, P806, DOI 10.1016/j.immuni.2013.10.010
Buffet PA, 2011, BLOOD, V117, P381, DOI 10.1182/blood-2010-04-202911
Calisher CH, 2006, CLIN MICROBIOL REV, V19, P531, DOI 10.1128/CMR.00017-06
Cesta MF, 2006, TOXICOL PATHOL, V34, P455, DOI 10.1080/01926230600867743
Chen LH, 2014, DATABASE-OXFORD, DOI 10.1093/database/bau021
Coburn DK, 1998, OECOLOGIA, V113, P467, DOI 10.1007/s004420050399
Cooper ST, 2012, AM J PHYSIOL-REG I, V302, pR1202, DOI 10.1152/ajpregu.00018.2012
Dane DM, 2006, J APPL PHYSIOL, V101, P289, DOI 10.1152/japplphysiol.01600.2005
de Back DZ, 2014, FRONT PHYSIOL, V5, DOI 10.3389/fphys.2014.00009
den Haan JMM, 2012, J INNATE IMMUN, V4, P437, DOI 10.1159/000335216
Dobson AP, 2005, SCIENCE, V310, P628, DOI 10.1126/science.1120872
Ekele I., 2014, J VET ADV, V4, P564
Field H, 2011, PLOS ONE, V6, DOI 10.1371/journal.pone.0028678
Fry M., 2017, Pathologic Basis of Veterinary Disease, P724
Galíndez EJ, 2000, ANAT REC, V258, P286
Gatto D, 2004, J IMMUNOL, V173, P4308, DOI 10.4049/jimmunol.173.7.4308
Geiser F, 2011, INTEGR COMP BIOL, V51, P337, DOI 10.1093/icb/icr042
Gray R, 2006, J ANAT, V209, P179, DOI 10.1111/j.1469-7580.2006.00591.x
Haley PJ, 2003, TOXICOLOGY, V188, P49, DOI 10.1016/S0300-483X(03)00043-X
Hanadhita D., 2018, JURNAL VET, V19, P62, DOI [10.19087/jveteriner.2018.19.1.62, DOI 10.19087/JVETERINER.2018.19.1.62]
Hayman DTS, 2012, EMERG INFECT DIS, V18, P1207, DOI 10.3201/eid1807.111654
Hengjan Y, 2018, J VET MED SCI, V80, P1146, DOI 10.1292/

jvms.17-0665
Hengjan Y, 2017, J VET MED SCI, V79, P1125, DOI 10.1292/jvms.16-0643
Ilardo MA, 2018, CELL, V173, P569, DOI 10.1016/j.cell.2018.03.054
JURGENS KD, 1981, RESP PHYSIOL, V45, P243, DOI 10.1016/0034-5687(81)90009-8
Kelm DH, 2007, J COMP PHYSIOL B, V177, P667, DOI 10.1007/s00360-007-0164-5
Kobayashi S, 2015, ARCH VIROL, V160, P1075, DOI 10.1007/s00705-015-2349-7
KRAAL G, 1992, INT REV CYTOL, V132, P31, DOI 10.1016/S0074-7696(08)62453-5
Kuzmin Ivan V, 2011, Emerg Health Threats J, V4, P7159, DOI 10.3402/ehtj.v4i0.7159
Lee F, 2011, VET MICROBIOL, V152, P96, DOI 10.1016/j.vetmic.2011.04.025
Lei M, 2016, SCI REP-UK, V6, DOI 10.1038/srep27726
LIDICKER WZ, 1955, P SOC EXP BIOL MED, V89, P640
Looi Lai-Meng, 2007, Malays J Pathol, V29, P63
Luis AD, 2013, P ROY SOC B-BIOL SCI, V280, DOI 10.1098/rspb.2012.2753
Maina JN, 2000, J EXP BIOL, V203, P3045
MAINA JN, 1984, J EXP BIOL, V111, P43
Mann FC, 1917, J EXP ZOOL, V23, P277, DOI 10.1002/jez.1400230204
McNab BK, 2001, J COMP PHYSIOL B, V171, P201, DOI 10.1007/s003600000163
Mebius RE, 2005, NAT REV IMMUNOL, V5, P606, DOI 10.1038/nri1669
Mohd HA, 2016, VIROL J, V13, DOI 10.1186/s12985-016-0544-0
Neuweiler G., 2000, BIOL BATS
O'Shea TJ, 2014, EMERG INFECT DIS, V20, P741, DOI 10.3201/eid2005.130539
Oehen S, 2002, J IMMUNOL, V169, P1453, DOI 10.4049/jimmunol.169.3.1453
Omatsu T, 2007, COMP IMMUNOL MICROB, V30, P357, DOI 10.1016/j.cimid.2007.05.006
Páez DJ, 2018, PHILOS T R SOC B, V373, DOI 10.1098/rstb.2017.0097
Pavlovich SS, 2018, CELL, V173, P1098, DOI 10.1016/j.cell.2018.03.070
Peel AJ, 2018, SCI REP-UK, V8, DOI 10.1038/s41598-018-22236-6
PERSSON SGB, 1973, ZBL VET MED A, V20, P521
Poole D C., 2015, Dukes' Physiology of Domestic Animals ed, P443
Press C. M., 2006, TXB VET HISTOLOGY, P117
Rothermel J. E., 1930, ANAT REC, V47, P49
Sasaki M, 2016, SCI REP-UK, V6, DOI 10.1038/srep24257
Sasaki M, 2014, J VIROL, V88, P9819, DOI 10.1128/JVI.01277-14
Sasaki M, 2012, VIROL J, V9, DOI 10.1186/1743-422X-9-240
Schowalter T, 2017, FORESTS, V8, DOI 10.3390/f8040097
Sendow I, 2010, ZOONOSES PUBLIC HLTH, V57, P499, DOI 10.1111/j.1863-2378.2009.01252.x
Sendow I, 2013, PLOS ONE, V8, DOI 10.1371/journal.pone.0069544
SNOOK T, 1950, AM J ANAT, V87, P31, DOI 10.1002/aja.1000870103
Sohayati AR, 2008, J ZOO WILDLIFE MED, V39, P674, DOI 10.1638/2006-0061.1
Stawski C, 2014, J ZOOL, V292, P86, DOI 10.1111/jzo.12105

Steiniger B, 2000, ADV ANAT EMBRYOL CEL, V151, p[III, 1]
Steiniger BS, 2015, IMMUNOLOGY, V145, P334, DOI 10.1111/imm.12469
Stewardson CL, 1999, J ANAT, V195, P235, DOI 10.1046/j.1469-7580.1999.19520235.x
Suyanto A., 2001, LIPI SERI PANDUAN LA
Tanaka Y., 1990, ACTA ANAT NIPPON, V63, P502
Teeling EC, 2005, SCIENCE, V307, P580, DOI 10.1126/science.1105113
THOMAS SP, 1975, J EXP BIOL, V63, P273
Thornton SJ, 2001, P NATL ACAD SCI USA, V98, P9413, DOI 10.1073/pnas.151192098
Udroiu I, 2017, Q REV BIOL, V92, P411, DOI 10.1086/695327
Udroiu Ion, 2006, Hystrix, V17, P99
Wagneft P., 1995, Equine Veterinary Journal, V27, P82
Wang LF, 2011, CURR OPIN VIROL, V1, P649, DOI 10.1016/j.coviro.2011.10.013
Wang Manli, 2013, Virologica Sinica, V28, P315, DOI 10.1007/s12250-013-3402-x
Ward J.M., 2018, Comparative Anatomy and Histology, VSecond, P365, DOI DOI 10.1016/B978-0-12-802900-8.00019-1
Webster S. H., 1977, DEV DYNAM, V97, P129
Zhang Q, 2015, VET J, V204, P110, DOI 10.1016/j.tvjl.2015.01.013
Zhao LT, 2015, FRONT MICROBIOL, V6, DOI 10.3389/fmicb.2015.00645
Zidan M, 2000, ANAT HISTOL EMBRYOL, V29, P221, DOI 10.1046/j.1439-0264.2000.00268.x
Zidan M, 2000, J ANAT, V196, P425, DOI 10.1046/j.1469-7580.2000.19630425.x
NR 92
TC 6
Z9 7
U1 2
U2 12
PU WILEY
PI HOBOKEN
PA 111 RIVER ST, HOBOKEN 07030-5774, NJ USA
SN 0340-2096
EI 1439-0264
J9 ANAT HISTOL EMBRYOL
JI Anat. Histol. Embryol.
PD JUL
PY 2019
VL 48
IS 4
BP 315
EP 324
DI 10.1111/ahe.12442
PG 10
WC Anatomy & Morphology; Veterinary Sciences
WE Science Citation Index Expanded (SCI-EXPANDED)
SC Anatomy & Morphology; Veterinary Sciences
GA II7WS
UT WOS:000475403000005
PM 30968443
OA Green Published, Bronze

DA 2024-10-21
ER

PT J
AU Weise, P
   Czirják, GA
   Lindecke, O
   Bumrungsri, S
   Voigt, CC
AF Weise, Philipp
   Czirjak, Gabor A.
   Lindecke, Oliver
   Bumrungsri, Sara
   Voigt, Christian C.
TI Simulated bacterial infection disrupts the circadian fluctuation of
   immune cells in wrinkle-lipped bats (<i>Chaerephon plicatus</i>)
SO PEERJ
LA English
DT Article
DE Acute phase response; Diurnal immune changes; Eosinophils; Neutrophils;
   Body mass loss; Leukocytosis
ID MITE SPINTURNIX-MYOTI; DISEASE RISK; TRADE-OFFS; STRESS; SYSTEM; RHYTHM;
   SKIN; SEX; HIBERNATION; CHIROPTERA
AB Background. Leukocyte concentrations follow a circadian pattern in mammals, with elevated values at times of potential contact with pathogens and parasites. We hypothesized that this pattern is disturbed after an immune challenge.
   Methods. In Thailand, we captured wrinkle-lipped bats (Chaerephon plicatus), when they returned to their colony at dawn. We challenged half of the animals (experimental group) with bacterial lipopolysaccharides and treated the others only with the carrier liquid (control group). We then compared body mass changes and differences in circulating immune cell counts at 8 h post-treatment.
   Results. In experimental animals, we observed an increase in total leukocyte and neutrophil numbers of 17% and 95%, respectively. In control animals, concentrations of leukocytes decreased by 44% and those of neutrophils remained constant. Experimental treatment had no effect on lymphocytes, yet changes in eosinophil numbers were explained by sex. Eosinophils decreased by 66% in females and by 62% in males. Basophils and monocytes were rarest among all observed cell types and analysis was either impossible because of low numbers or yielded no significant effects, respectively.
   Discussion. Our findings show that a simulated bacterial infection triggered a neutrophil-associated immune response in wrinkle-lipped bats, indicating a disruption of the diurnal fluctuation of immune cells. Our study suggests that bats exhibit circadian rhythms in immune cell counts. The magnitude of these fluctuations may vary across species according to specific-specific infection risks associated with colony sizes or specific roosting habits.
C1 [Weise, Philipp; Lindecke, Oliver; Voigt, Christian C.] Leibniz

Inst Zoo & Wildlife Res, Dept Evolutionary Ecol, Berlin, Germany.
[Weise, Philipp] Humboldt Univ, Dept Biol, Berlin, Germany.
[Czirjak, Gabor A.] Leibniz Inst Zoo & Wildlife Res, Dept Wildlife Dis, Berlin, Germany.
[Lindecke, Oliver; Voigt, Christian C.] Free Univ Berlin, Dept Anim Behav, Berlin, Germany.
[Bumrungsri, Sara] Prince Songkla Univ, Dept Biol, Hat Yai, Thailand.
C3 Leibniz Institut fur Zoo und Wildtierforschung; Humboldt University of
Berlin; Leibniz Institut fur Zoo und Wildtierforschung; Free University
of Berlin; Prince of Songkla University
RP Voigt, CC (corresponding author), Leibniz Inst Zoo & Wildlife Res, Dept Evolutionary Ecol, Berlin, Germany.; Voigt, CC (corresponding author), Free Univ Berlin, Dept Anim Behav, Berlin, Germany.
EM voigt@izw-berlin.de
RI Lindecke, Oliver/Z-4304-2019; Czirják, Gábor/F-5440-2011
OI Lindecke, Oliver/0000-0002-2545-9999; Czirjak, Gabor
Arpad/0000-0001-9488-0069
FU German Federal Ministry of Education and Research [01DP14004]
FX This work was financed by a grant (01DP14004) to CCV and SB by the dual
THAI-GERMAN funding scheme of the German Federal Ministry of Education
and Research. The funders had no role in study design, data collection
and analysis, decision to publish, or preparation of the manuscript.
CR Abolins SR, 2011, MOL ECOL, V20, P881, DOI 10.1111/j.1365-294X.2010.04910.x
Allen LC, 2009, J COMP PHYSIOL B, V179, P315, DOI 10.1007/s00360-008-0315-3
[Anonymous], 2019, CAR AN R COMPANION A
[Anonymous], 2013, A language and environment for statistical computing
Baker M L., 2015, Bats and viruses: a new frontier of emerging infectious diseases, P327
Born J, 1997, J IMMUNOL, V158, P4454
Bouma HR, 2010, J LEUKOCYTE BIOL, V88, P619, DOI 10.1189/jlb.0310174
Boysen P, 2011, MOL ECOL, V20, P5103, DOI 10.1111/j.1365-294X.2011.05269.x
Christe P, 2000, ECOL LETT, V3, P207
Christe P, 2007, J ANIM ECOL, V76, P703, DOI 10.1111/j.1365-2656.2007.01255.x
Copeland S, 2005, CLIN DIAGN LAB IMMUN, V12, P60, DOI 10.1128/CDLI.12.1.60-67.2005
Davis AK, 2008, FUNCT ECOL, V22, P760, DOI 10.1111/j.1365-2435.2008.01467.x
DHABHAR FS, 1994, BRAIN BEHAV IMMUN, V8, P66, DOI 10.1006/brbi.1994.1006
Greiner S, 2010, GEN COMP ENDOCR, V168, P466, DOI 10.1016/

j.ygcen.2010.06.008
GRIFFIN AC, 1991, J NEUROIMMUNOL, V35, P53, DOI 10.1016/0165-5728(91)90161-Y
HALBERG F, 1960, P SOC EXP BIOL MED, V103, P142
Halpin K, 2000, J GEN VIROL, V81, P1927, DOI 10.1099/0022-1317-81-8-1927
Hansen K, 1997, AM J PHYSIOL-REG I, V273, pR548, DOI 10.1152/ajpregu.1997.273.2.R548
Hillman A., 2006, J WILDL THAIL, V13, P31
Johara MY, 2001, EMERG INFECT DIS, V7, P439
KOZAK W, 1994, AM J PHYSIOL, V266, pR125, DOI 10.1152/ajpregu.1994.266.1.R125
Kuzmin Ivan V, 2011, Emerg Health Threats J, V4, P7159, DOI 10.3402/ehtj.v4i0.7159
Labrecque N, 2015, J BIOL RHYTHM, V30, P277, DOI 10.1177/0748730415577723
Leelapaibul W, 2005, ACTA CHIROPTEROL, V7, P111, DOI 10.3161/1733-5329(2005)7[111:DOWFBT]2.0.CO;2
Leroy EM, 2005, NATURE, V438, P575, DOI 10.1038/438575a
Lippi G, 2010, BLOOD TRANSFUS-ITALY, V8, P94, DOI 10.2450/2009.0142-09
Lochmiller RL, 2000, OIKOS, V88, P87, DOI 10.1034/j.1600-0706.2000.880110.x
Luis AD, 2013, P ROY SOC B-BIOL SCI, V280, DOI 10.1098/rspb.2012.2753
Murphy K, 2016, JANEWAYS IMMUNOBIOLO, DOI DOI 10.1201/9781315533247
Navarro C, 2003, OIKOS, V101, P291, DOI 10.1034/j.1600-0706.2003.11663.x
Nunn CL, 2002, EVOLUTION, V56, P177
Nunn CL, 2003, P ROY SOC B-BIOL SCI, V270, P347, DOI 10.1098/rspb.2002.2249
Olsson A, 2009, BATS CAPTIVITY, V1, P339
Otálora-Ardila A, 2016, PLOS ONE, V11, DOI 10.1371/journal.pone.0164938
Ots I, 1998, FUNCT ECOL, V12, P700, DOI 10.1046/j.1365-2435.1998.00219.x
Pap PL, 2010, NATURWISSENSCHAFTEN, V97, P891, DOI 10.1007/s00114-010-0706-7
Patterson BD, 2008, J TROP ECOL, V24, P387, DOI 10.1017/S0266467408005117
Postawa T, 2014, PARASITOL RES, V113, P1803, DOI 10.1007/s00436-014-3826-8
Prendergast BJ, 2002, AM J PHYSIOL-REG I, V282, pR1054, DOI 10.1152/ajpregu.00562.2001
RICHARDSON RP, 1989, ANN SURG, V210, P239, DOI 10.1097/00000658-198908000-00018
Scheiermann C, 2013, NAT REV IMMUNOL, V13, P190, DOI 10.1038/nri3386
Schinnerl M, 2011, J ZOO WILDLIFE MED, V42, P382, DOI 10.1638/2010-0060.1
Schneeberger K, 2014, PLOS ONE, V9, DOI 10.1371/journal.pone.0108268
Schneeberger K, 2013, J EXP BIOL, V216, P4514, DOI 10.1242/

jeb.090837
Schneeberger K, 2013, PLOS ONE, V8, DOI 10.1371/journal.pone.0054023
Sharifi M, 2008, J PARASITOL, V94, P622, DOI 10.1645/GE-1334.1
Sheldon BC, 1996, TRENDS ECOL EVOL, V11, P317, DOI 10.1016/0169-5347(96)10039-2
Shiraishi M, 1996, Environ Health Prev Med, V1, P65, DOI 10.1007/BF02931192
Stockmaier S, 2015, BIOL LETTERS, V11, DOI 10.1098/rsbl.2015.0576
Tian JD, 2015, OECOLOGIA, V179, P679, DOI 10.1007/s00442-015-3397-0
Tian JD, 2015, FUNCT ECOL, V29, P823, DOI 10.1111/1365-2435.12394
Tsang SM, 2016, ROLES TAXONOMY SYSTE, P503
Turmelle AS, 2010, J COMP PHYSIOL B, V180, P1155, DOI 10.1007/s00360-010-0486-6
Turner PV, 2011, J AM ASSOC LAB ANIM, V50, P600
Utthammachai Kessarin, 2008, Thai Journal of Forestry, V27, P21
VALDIVIESO D, 1971, CAN J ZOOLOG, V49, P31, DOI 10.1139/z71-007
Venables WN, 2002, MODERN
Wang L., 2015, Bats and Viruses: A New Frontier of Emerging Infectious Diseases
Wanger TC, 2014, BIOL CONSERV, V171, P220, DOI 10.1016/j.biocon.2014.01.030
WEG VB, 1995, IMMUNOLOGY, V84, P36
WIDMAIER EP, 1993, J EXP ZOOL, V265, P533, DOI 10.1002/jez.1402650509
Zahorec R, 2001, Bratisl Lek Listy, V102, P5
Zylberberg M, 2015, J EXP BIOL, V218, P757, DOI 10.1242/jeb.111716
NR 63
TC 19
Z9 22
U1 0
U2 10
PU PEERJ INC
PI LONDON
PA 341-345 OLD ST, THIRD FLR, LONDON, EC1V 9LL, ENGLAND
SN 2167-8359
J9 PEERJ
JI PeerJ
PD AUG 3
PY 2017
VL 5
AR e3570
DI 10.7717/peerj.3570
PG 18
WC Multidisciplinary Sciences
WE Science Citation Index Expanded (SCI-EXPANDED)
SC Science & Technology - Other Topics
GA FD0UJ
UT WOS:000407253900001
PM 28791196
OA Green Published, Green Submitted, gold
DA 2024-10-21

ER

PT J
AU Cabrera-Martinez, LV
   Herrera, LG
   Cruz-Neto, AP
AF Cabrera-Martinez, Lucia V.
   Herrera M, L. Gerardo
   Cruz-Neto, Ariovaldo P.
TI Food restriction, but not seasonality, modulates the acute phase
   response of a Neotropical bat
SO COMPARATIVE BIOCHEMISTRY AND PHYSIOLOGY A-MOLECULAR & INTEGRATIVE
   PHYSIOLOGY
LA English
DT Article
DE Energetic cost; Immune response; Acute phase response; Metabolic
rate;
   Seasonality; Food restriction
ID IMMUNE FUNCTION; SICKNESS BEHAVIOR; INNATE IMMUNITY; METABOLIC-
RATE;
   PHOTOPERIOD; TEMPERATURE; INFECTION; FEVER; IMMUNOCOMPETENCE;
   CONSEQUENCES
AB Season and food intake are known to affect immune response of
vertebrates yet their effects on metabolic rate have been rarely
explored. We tested the effect of season and acute food restriction
and their interaction on the energetic cost of immune response
activation of a tropical vertebrate, the Seba's short-tailed fruit
bat (Carollia perspicillata). We specifically stimulated the acute
phase response (APR) with bacterial lipopolysaccharide (LPS) to
measure metabolic changes along with changes in body temperature (T-
b), body mass (M-b), white blood cell counts and the Neutrophil/
Lymphocyte ratio (N/L). We found no effect of season on the
different factors associated to the activation of the APR. In
contrast to our expectations, unfed bats reached similar T-b
increments and RMR peak values and had higher RMR scope values and
higher caloric costs than fed bats after LPS injection. However,
food deprivation led to delayed metabolic response indicated by
longer time required to reach peak RMR values in unfed bats. Both
food-deprived and fed bats did not present leukocytosis after APR
activation and their WBC counts were similar, but unfed bats had a
significant increase of N/L. APR activation represented a small
fraction of the bat daily energy requirements which might explain
why unfed bats were not limited to mount a metabolic response. Our
study adds to recent evidence showing that activating the innate
immune system is not an energetically expensive process for plant-
eating bats.
C1 [Cabrera-Martinez, Lucia V.] Univ Estadual Paulista Julho de
Mesquita Filho, Inst Biociencias, Posgrad Programa Zool, Rio Claro,
SP, Brazil.
   [Herrera M, L. Gerardo] Univ Nacl Autonoma Mexico, Inst Biol,
Estn Biol Chamela, Apartado Postal 21, San Patricio 48980, Jalisco,
Mexico.
   [Cruz-Neto, Ariovaldo P.] Univ Estadual Paulista Julho de
Mesquita Filho, Inst Biociencias, Dept Zool, Rio Claro, SP, Brazil.

C3 Universidad Nacional Autonoma de Mexico
RP Herrera, LG (corresponding author), Univ Nacl Autonoma Mexico,
Inst Biol, Estn Biol Chamela, Apartado Postal 21, San Patricio
48980, Jalisco, Mexico.
EM gherrera@ib.unam.mx
RI cruz-neto, ariovaldo/Y-7225-2019; Cabrera-Martinez, Lucia/
JKI-1730-2023;
Cruz-Neto, Ariovaldo/B-2958-2013
OI Cruz-Neto, Ariovaldo/0000-0001-5270-7276; Cabrera-Martinez,
Lucia/0000-0002-5487-9837; Herrera Montalvo, Luis
Gerardo/0000-0001-9348-5040
FU Conselho Nacional de Pesquisa e Desenvolvimento Tecnologico
(CNPq)
[490586/2013-9]; Consejo Nacional de Ciencia y Tecnologia
[490586/2013-9]; Fundacao de Amparo a Pesquisa do Estado de Sao
Paolo
[2017-17607-6]; CNPq [161027/2014-8]; PASPA-DGAPA program of the
Universidad Nacional Autonoma de Mexico [814-2018]
FX This work was supported by grants to LGHM and APCN from the
Bilateral
Program of Conselho Nacional de Pesquisa e Desenvolvimento
Tecnologico
(CNPq) and the Consejo Nacional de Ciencia y Tecnologia
(#490586/2013-9)
and by the Visiting Research Program of the Fundacao de Amparo a
Pesquisa do Estado de Sao Paolo(#2017-17607-6). LVCM received a
master
grant from the CNPq (#161027/2014-8). LGHM was supported by the
PASPA-DGAPA program of the Universidad Nacional Autonoma de
Mexico(#814-2018).
CR ADER R, 1993, ANNU REV PSYCHOL, V44, P53, DOI 10.1146/
annurev.ps.44.020193.000413
Ahmad R, 2009, J NEUROENDOCRINOL, V21, P2, DOI 10.1111/
j.1365-2826.2008.01805.x
ALLISON DB, 1995, DIABETES CARE, V18, P245, DOI 10.2337/
diacare.18.2.245
Almeida MC, 2011, J THERM BIOL, V36, P225, DOI 10.1016/
j.jtherbio.2011.03.005
Alonso-Alvarez C, 2001, CAN J ZOOL, V79, P101, DOI 10.1139/
cjz-79-1-101
[Anonymous], COMP PHYSL
[Anonymous], J COMP PHYSL B
[Anonymous], J REPR FERT
[Anonymous], P NATL ACAD SCI US
[Anonymous], DIABETES CARE
[Anonymous], FUNCT ECOL
[Anonymous], ECOLOGICAL BEHAV MET
[Anonymous], 2002, REV BRAS ZOOL
BANET M, 1981, EXPERIENTIA, V37, P1302, DOI 10.1007/BF01948375
Berger J, 2013, J APPL BIOMED, V11, P1, DOI 10.2478/
v10136-012-0029-4
Bilbo SD, 2002, P ROY SOC B-BIOL SCI, V269, P447, DOI 10.1098/
rspb.2001.1915
Bonneaud C, 2003, AM NAT, V161, P367, DOI 10.1086/346134

Bozinovic F, 2007, J COMP PHYSIOL B, V177, P393, DOI 10.1007/s00360-006-0137-0
Cabrera-Martínez LV, 2018, PEERJ, V6, DOI 10.7717/peerj.4627
Calder PC, 2008, PROSTAG LEUKOTR ESS, V79, P101, DOI 10.1016/j.plefa.2008.09.016
Christe P, 2000, ECOL LETT, V3, P207
Cruz-Neto A.P., 2006, FUNCTIONAL EVOLUTION, P56
Cutrera AP, 2010, J EXP BIOL, V213, P715, DOI 10.1242/jeb.037887
Davis AK, 2008, FUNCT ECOL, V22, P760, DOI 10.1111/j.1365-2435.2008.01467.x
De Coster G, 2010, J EXP BIOL, V213, P3012, DOI 10.1242/jeb.042721
de Neve L, 2007, IBIS, V149, P763, DOI 10.1111/j.1474-919X.2007.00708.x
Demas GE, 1998, J BIOL RHYTHM, V13, P253, DOI 10.1177/074873098129000093
Geiser F, 2005, J THERM BIOL, V30, P574, DOI 10.1016/j.jtherbio.2005.08.002
Guerrero-Chacón AL, 2018, MAMMAL RES, V63, P397, DOI 10.1007/s13364-018-0375-z
Gutiérrez JS, 2011, J AVIAN BIOL, V42, P259, DOI 10.1111/j.1600-048X.2011.05323.x
Hawley DM, 2011, FUNCT ECOL, V25, P48, DOI 10.1111/j.1365-2435.2010.01753.x
Hegemann A, 2012, OECOLOGIA, V170, P605, DOI 10.1007/s00442-012-2339-3
Herrera LG, 2002, OECOLOGIA, V133, P280, DOI 10.1007/s00442-002-1036-z
Inoue W, 2008, AM J PHYSIOL-REG I, V295, pR133, DOI 10.1152/ajpregu.90335.2008
Killpack TL, 2015, PHYSIOL BIOCHEM ZOOL, V88, P195, DOI 10.1086/680168
Klein SL, 2016, NAT REV IMMUNOL, V16, P626, DOI 10.1038/nri.2016.90
Krams I, 2012, COMP BIOCHEM PHYS A, V161, P422, DOI 10.1016/j.cbpa.2011.12.018
Ksiazek A, 2012, PHYSIOL BIOCHEM ZOOL, V85, P51, DOI 10.1086/663696
Lee KA, 2006, OECOLOGIA, V150, P383, DOI 10.1007/s00442-006-0537-6
LEVEY DJ, 1988, ECOL MONOGR, V58, P251, DOI 10.2307/1942539
Lighton J.R.B., 2008, MEASURING METABOLIC, DOI DOI 10.1242/JEB.65.3.643
Lochmiller RL, 2000, OIKOS, V88, P87, DOI 10.1034/j.1600-0706.2000.880110.x
LOCHMILLER RL, 1993, AUK, V110, P503, DOI 10.2307/4088414
Mabuchi Y, 2016, ROY SOC OPEN SCI, V3, DOI 10.1098/rsos.150408
MacDonald L, 2014, BRAIN BEHAV IMMUN, V40, P18, DOI 10.1016/j.bbi.2014.01.005
MacDonald L, 2012, PHYSIOL BEHAV, V107, P762, DOI 10.1016/j.physbeh.2012.06.009
MacDonald L, 2011, AM J PHYSIOL-REG I, V301, pR172, DOI 10.1152/ajpregu.00057.2011
Martin LB, 2004, ECOLOGY, V85, P2323, DOI 10.1890/03-0365

Martin LB, 2008, PHILOS T R SOC B, V363, P321, DOI 10.1098/rstb.2007.2142
Mcnab BK, 2008, COMP BIOCHEM PHYS A, V151, P5, DOI 10.1016/j.cbpa.2008.05.008
Mello MAR, 2004, MAMMALIA, V68, P49, DOI 10.1515/mamm.2004.006
Merlo JL, 2016, J EXP ZOOL PART A, V325, P675, DOI 10.1002/jez.2060
Nelson RJ, 1996, Q REV BIOL, V71, P511, DOI 10.1086/419555
Nelson RJ, 2004, TRENDS IMMUNOL, V25, P187, DOI 10.1016/j.it.2004.02.001
Otálora-Ardila A, 2017, MAMM BIOL, V82, P41, DOI 10.1016/j.mambio.2016.11.002
Otálora-Ardila A, 2016, PLOS ONE, V11, DOI 10.1371/journal.pone.0164938
Owen-Ashley NT, 2006, J EXP BIOL, V209, P3062, DOI 10.1242/jeb.02371
R. Development Core Team, 2018, A language and environment for statistical corn-mating
Radler ME, 2014, BRAIN BEHAV IMMUN, V38, P13, DOI 10.1016/j.bbi.2013.11.014
Ramirez-Otarola N, 2018, J THERM BIOL, V71, P17, DOI 10.1016/j.jtherbio.2017.10.005
Schneeberger K, 2013, J EXP BIOL, V216, P4514, DOI 10.1242/jeb.090837
Schultz EM, 2017, J EXP BIOL, V220, P722, DOI 10.1242/jeb.149898
Singh SS, 2007, COMP BIOCHEM PHYS A, V146, P446, DOI 10.1016/j.cbpa.2006.12.024
Sköld-Chiriac S, 2015, J EXP BIOL, V218, P2961, DOI 10.1242/jeb.122150
Speakman JR, 2010, J ANIM ECOL, V79, P726, DOI 10.1111/j.1365-2656.2010.01689.x
VANSCHAIK CP, 1993, ANNU REV ECOL SYST, V24, P353, DOI 10.1146/annurev.es.24.110193.002033
Versteegh MA, 2014, J EXP BIOL, V217, P1510, DOI 10.1242/jeb.097105
Voigt CC, 2006, J COMP PHYSIOL B, V176, P213, DOI 10.1007/s00360-005-0042-y
Wilckens T, 1997, IMMUNOL TODAY, V18, P418, DOI 10.1016/S0167-5699(97)01111-0
Xu DL, 2017, J EXP BIOL, V220, P2187, DOI 10.1242/jeb.153601
Xu DL, 2010, COMP BIOCHEM PHYS A, V155, P25, DOI 10.1016/j.cbpa.2009.09.003
Zysling DA, 2009, FUNCT ECOL, V23, P979, DOI 10.1111/j.1365-2435.2009.01572.x
NR 72
TC 7
Z9 7
U1 0
U2 13
PU ELSEVIER SCIENCE INC
PI NEW YORK
PA STE 800, 230 PARK AVE, NEW YORK, NY 10169 USA
SN 1095-6433
EI 1531-4332

J9 COMP BIOCHEM PHYS A
JI Comp. Biochem. Physiol. A-Mol. Integr. Physiol.
PD MAR
PY 2019
VL 229
BP 93
EP 100
DI 10.1016/j.cbpa.2018.12.003
PG 8
WC Biochemistry & Molecular Biology; Physiology; Zoology
WE Science Citation Index Expanded (SCI-EXPANDED)
SC Biochemistry & Molecular Biology; Physiology; Zoology
GA IE7XJ
UT WOS:000472588000013
PM 30553882
DA 2024-10-21
ER

PT J
AU Fumagalli, MR
   Zapperi, S
   La Porta, CAM
AF Fumagalli, Maria Rita
   Zapperi, Stefano
   La Porta, Caterina A. M.
TI Role of body temperature variations in bat immune response to viral
   infections
SO JOURNAL OF THE ROYAL SOCIETY INTERFACE
LA English
DT Article
DE bats; interferon-alpha; immunity; virus
ID TORPOR; ENERGETICS; PATTERNS; KINETICS
AB The ability of bats to coexist with viruses without being harmed is an interesting issue that is still under investigation. Here we use a mathematical model to show that the pattern of body temperature variations observed in bats between day and night is responsible for their ability to keep viruses in check. From the dynamical systems point of view, our model displays an intriguing quasi-periodic behaviour that might be relevant in making the system robust by avoiding viral escape due to perturbations in the body temperature cycle.
C1 [Fumagalli, Maria Rita; La Porta, Caterina A. M.] Univ Milan, Dept Environm Sci & Policy, Ctr Complex & Biosyst, Via Celoria 26, I-20133 Milan, Italy.
   [Fumagalli, Maria Rita; La Porta, Caterina A. M.] CNR Consiglio Nazl Ric, Biophys Inst, Via Marini 6, I-16149 Genoa, Italy.
   [Zapperi, Stefano] Univ Milan, Dept Phys, Ctr Complex & Biosyst, Via Celoria 16, I-20133 Milan, Italy.
   [Zapperi, Stefano] ICMATE, CNR Consiglio Nazl Ric, Via R Cozzi 53, I-20125 Milan, Italy.
C3 University of Milan; Consiglio Nazionale delle Ricerche (CNR);
   University of Milan; Consiglio Nazionale delle Ricerche (CNR); Istituto

di Chimica della Materia Condensata e di Tecnologie per l Energia (ICMATE-CNR)
RP La Porta, CAM (corresponding author), Univ Milan, Dept Environm Sci & Policy, Ctr Complex & Biosyst, Via Celoria 26, I-20133 Milan, Italy.; La Porta, CAM (corresponding author), CNR Consiglio Nazl Ric, Biophys Inst, Via Marini 6, I-16149 Genoa, Italy.
EM caterina.laporta@unimi.it
RI Fumagalli, Maria Rita/HLV-7667-2023; Zapperi, Stefano/C-9473-2009; la porta, caterina/K-7345-2013
OI Fumagalli, Maria Rita/0000-0003-3404-5779; Zapperi, Stefano/0000-0001-5692-5465; la porta, caterina/0000-0002-3010-8966
CR Baccam P, 2006, J VIROL, V80, P7590, DOI 10.1128/JVI.01623-05
Bartels W, 1998, J COMP PHYSIOL B, V168, P233, DOI 10.1007/s003600050141
Chen LH, 2014, DATABASE-OXFORD, DOI 10.1093/database/bau021
Chen PQ, 2010, BIOPHYS J, V98, P1109, DOI 10.1016/j.bpj.2009.11.048
Coburn DK, 1998, OECOLOGIA, V113, P467, DOI 10.1007/s004420050399
De La Cruz-Rivera PC, 2018, J IMMUNOL, V200, P209, DOI 10.4049/jimmunol.1701214
Geiser F., 2010, ZOOLOGIST, V35, P2
Geiser F, 2011, INTEGR COMP BIOL, V51, P337, DOI 10.1093/icb/icr042
Hrvatin S, 2020, NATURE, V583, P115, DOI 10.1038/s41586-020-2387-5
Jones KE, 2005, EVOLUTION, V59, P2243, DOI 10.1111/j.0014-3820.2005.tb00932.x
Kelm DH, 2007, J COMP PHYSIOL B, V177, P667, DOI 10.1007/s00360-007-0164-5
Matos AD, 2019, EMERG MICROBES INFEC, V8, P1763, DOI 10.1080/22221751.2019.1698271
Miller MR, 2016, J INFECT DIS, V214, pS297, DOI 10.1093/infdis/jiw199
Munster VJ, 2016, SCI REP-UK, V6, DOI 10.1038/srep21878
O'Shea TJ, 2014, EMERG INFECT DIS, V20, P741, DOI 10.3201/eid2005.130539
Perelson AS, 2002, NAT REV IMMUNOL, V2, P28, DOI 10.1038/nri700
Rintoul JLP, 2014, J COMP PHYSIOL B, V184, P777, DOI 10.1007/s00360-014-0837-9
Ruf T, 2015, BIOL REV, V90, P891, DOI 10.1111/brv.12137
Sanjuán R, 2010, J VIROL, V84, P9733, DOI 10.1128/JVI.00694-10
Shechter Y, 2001, P NATL ACAD SCI USA, V98, P1212, DOI 10.1073/pnas.98.3.1212
Stawski C, 2014, J ZOOL, V292, P86, DOI 10.1111/jzo.12105
Takahashi TM, 2020, NATURE, V583, P109, DOI 10.1038/s41586-020-2163-6
THOMAS SP, 1972, J EXP BIOL, V57, P317
Wacharapluesadee S, 2021, NAT COMMUN, V12, DOI 10.1038/s41467-021-21240-1
Wodarz D, 2002, TRENDS IMMUNOL, V23, P194, DOI 10.1016/S1471-4906(02)02189-0
Wojciechowski MS, 2007, COMP BIOCHEM PHYS A, V147, P828, DOI

10.1016/j.cbpa.2006.06.039
NR 26
TC 6
Z9 7
U1 1
U2 11
PU ROYAL SOC
PI LONDON
PA 6-9 CARLTON HOUSE TERRACE, LONDON SW1Y 5AG, ENGLAND
SN 1742-5689
EI 1742-5662
J9 J R SOC INTERFACE
JI J. R. Soc. Interface
PD JUL 28
PY 2021
VL 18
IS 180
AR 20210211
DI 10.1098/rsif.2021.0211
PG 5
WC Multidisciplinary Sciences
WE Science Citation Index Expanded (SCI-EXPANDED)
SC Science & Technology - Other Topics
GA TR1AV
UT WOS:000678705300001
PM 34314652
OA Bronze, Green Published
DA 2024-10-21
ER

PT J
AU Chen, WL
   Mao, XG
AF Chen, Wenli
   Mao, Xiuguang
TI Extensive alternative splicing triggered by mitonuclear mismatch in
   naturally introgressed Rhinolophus bats
SO ECOLOGY AND EVOLUTION
LA English
DT Article
DE gene expression; horseshoe bats; introgressive hybridization;
   mitonuclear interaction; transcriptome
ID GENE-EXPRESSION; MITOCHONDRIAL GENOTYPE; LOCAL ADAPTATION; NUCLEAR;
   EVOLUTION; DRIVES; COEVOLUTION; METABOLISM; RESPONSES; ROBUST
AB Mitochondrial function needs strong interactions of mitochondrial and nuclear (mitonuclear) genomes, which can be disrupted by mitonuclear mismatch due to mitochondrial DNA (mtDNA) introgression between two formerly isolated populations or taxa. This mitonuclear disruption may cause severe cellular stress in mismatched individuals. Gene expression changes and alternative splicing (AS) are two important transcriptional regulations to respond to environmental or cellular stresses. We previously identified a

naturally introgressed population in the intermediate horseshoe bat (Rhinolophus affinis). Individuals from this population belong to R. a. himalayanus and share almost identical nuclear genetic background; however, some of them had mtDNA from another subspecies (R. a. macrurus). With this unique natural system, we examined gene expression changes in six tissues between five mitonuclear mismatched and five matched individuals. A small number of differentially expressed genes (DEGs) were identified, and functional enrichment analysis revealed that most DEGs were related to immune response although some may be involved in response to oxidative stress. In contrast, we identified extensive AS events and alternatively spliced genes (ASGs) between mismatched and matched individuals. Functional enrichment analysis revealed that multiple ASGs were directly or indirectly associated with energy production in mitochondria which is vital for survival of organism. To our knowledge, this is the first study to examine the role of AS in responding to cellular stress caused by mitonuclear mismatch in natural populations. Our results suggest that AS may play a more important role than gene expression regulation in responding to severe environmental or cellular stresses.
C1 [Chen, Wenli; Mao, Xiuguang] East China Normal Univ, Sch Ecol & Environm Sci, Shanghai, Peoples R China.
[Mao, Xiuguang] East China Normal Univ, Inst Ecochongming IEC, Shanghai 200062, Peoples R China.
C3 East China Normal University; East China Normal University
RP Mao, XG (corresponding author), East China Normal Univ, Inst Ecochongming IEC, Shanghai 200062, Peoples R China.
EM xgmao@sklec.ecnu.edu.cn
OI Chen, Wenli/0000-0003-3129-3662
FU National Natural Science Foundation of China [31570378, 31630008]
FX National Natural Science Foundation of China, Grant/Award Number: 31570378 and 31630008
CR Balaban RS, 2005, CELL, V120, P483, DOI 10.1016/j.cell.2005.02.001
Ballard JWO, 2020, CELL SIGNAL, V75, DOI 10.1016/j.cellsig.2020.109737
Bar-Yaacov D, 2012, BBA-GENE REGUL MECH, V1819, P1107, DOI 10.1016/j.bbagrm.2011.10.008
Barreto FS, 2018, NAT ECOL EVOL, V2, P1250, DOI 10.1038/s41559-018-0588-1
Barreto FS, 2013, P ROY SOC B-BIOL SCI, V280, DOI 10.1098/rspb.2013.1521
Barrier M, 2001, P NATL ACAD SCI USA, V98, P10208, DOI 10.1073/pnas.181257698
BENJAMINI Y, 1995, J R STAT SOC B, V57, P289, DOI 10.1111/j.2517-6161.1995.tb02031.x
Bolger AM, 2014, BIOINFORMATICS, V30, P2114, DOI 10.1093/bioinformatics/btu170
Burton RS, 2013, ANNU REV ECOL EVOL S, V44, P281, DOI 10.1146/annurev-ecolsys-110512-135758
Burton RS, 2012, MOL ECOL, V21, P4942, DOI 10.1111/mec.12006
Bush SJ, 2017, PHILOS T R SOC B, V372, DOI 10.1098/rstb.2015.0474
Croux C, 2007, CHEMOMETR INTELL LAB, V87, P218, DOI 10.1016/j.chemolab.2007.01.004

El Taher A, 2021, NAT ECOL EVOL, V5, P243, DOI 10.1038/s41559-020-01354-3
Ellison CK, 2006, EVOLUTION, V60, P1382, DOI 10.1111/j.0014-3820.2006.tb01217.x
Flight PA, 2011, MOL ECOL, V20, P4503, DOI 10.1111/j.1365-294X.2011.05290.x
Fraser HB, 2013, GENOME RES, V23, P1089, DOI 10.1101/gr.152710.112
Grantham ME, 2018, MOL BIOL EVOL, V35, P1934, DOI 10.1093/molbev/msy095
Gusdon AM, 2007, J BIOL CHEM, V282, P5171, DOI 10.1074/jbc.M609367200
Healy TM, 2017, MOL ECOL, V26, P814, DOI 10.1111/mec.13945
Hill GE, 2019, BIOL REV, V94, P1089, DOI 10.1111/brv.12493
Hill GE, 2015, MOL BIOL EVOL, V32, P1917, DOI 10.1093/molbev/msv104
Hodgins-Davis A, 2009, TRENDS ECOL EVOL, V24, P649, DOI 10.1016/j.tree.2009.06.011
Ibrahim HMM, 2021, MOL PLANT PATHOL, V22, P31, DOI 10.1111/mpp.13006
Jacobs A, 2021, MOL ECOL, V30, P4955, DOI 10.1111/mec.15817
Kenkel CD, 2017, NAT ECOL EVOL, V1, DOI 10.1038/s41559-016-0014
Kijewska A, 2018, SCI REP-UK, V8, DOI 10.1038/s41598-018-29723-w
Kim D, 2015, NAT METHODS, V12, P357, DOI [10.1038/NMETH.3317, 10.1038/nmeth.3317]
Kærn M, 2005, NAT REV GENET, V6, P451, DOI 10.1038/nrg1615
Lasky JR, 2014, MOL BIOL EVOL, V31, P2283, DOI 10.1093/molbev/msu170
Latorre-Pellicer A, 2016, NATURE, V535, P561, DOI 10.1038/nature18618
Lee JS, 2021, AGRONOMY-BASEL, V11, DOI 10.3390/agronomy11010092
Levin L, 2014, FRONT GENET, V5, DOI 10.3389/fgene.2014.00448
Liao Y, 2014, BIOINFORMATICS, V30, P923, DOI 10.1093/bioinformatics/btt656
López-Maury L, 2008, NAT REV GENET, V9, P583, DOI 10.1038/nrg2398
Love MI, 2014, GENOME BIOL, V15, DOI 10.1186/s13059-014-0550-8
Mank JE, 2017, NAT ECOL EVOL, V1, DOI 10.1038/s41559-016-0006
Mao X.G, 2021, MITONUCLEAR MISMATCH
Mao XG, 2010, MOL ECOL, V19, P2754, DOI 10.1111/j.1365-294X.2010.04704.x
Mao XG, 2020, MOL PHYLOGENET EVOL, V150, DOI 10.1016/j.ympev.2020.106886
Mao XG, 2014, BMC EVOL BIOL, V14, DOI 10.1186/1471-2148-14-154
Mao XG, 2013, MOL ECOL, V22, P1035, DOI 10.1111/mec.12154
Martín G, 2021, GENOME BIOL, V22, DOI 10.1186/s13059-020-02258-y
Mossman JA, 2019, G3-GENES GENOM GENET, V9, P1175, DOI 10.1534/g3.119.400067
Mossman JA, 2017, MOL BIOL EVOL, V34, P447, DOI 10.1093/molbev/msw246
Mossman JA, 2016, GENETICS, V204, P613, DOI 10.1534/genetics.116.192328
Rand DM, 2018, IUBMB LIFE, V70, P1275, DOI 10.1002/iub.1954
Rand DM, 2004, TRENDS ECOL EVOL, V19, P645, DOI 10.1016/j.tree.2004.10.003

Rank NE, 2020, EVOLUTION, V74, P1724, DOI 10.1111/evo.13962
Rivera HE, 2021, MOL ECOL, V30, P1381, DOI 10.1111/mec.15820
Rogers TF, 2021, MOL BIOL EVOL, V38, P519, DOI 10.1093/molbev/msaa242
Santiago JC, 2021, BMC GENOMICS, V22, DOI 10.1186/s12864-021-07516-2
Shen SH, 2014, P NATL ACAD SCI USA, V111, pE5593, DOI 10.1073/pnas.1419161111
Shen YY, 2010, P NATL ACAD SCI USA, V107, P8666, DOI 10.1073/pnas.0912613107
Singh P, 2017, GENOME BIOL EVOL, V9, P2764, DOI 10.1093/gbe/evx204
Sloan DB, 2018, NAT REV GENET, V19, P635, DOI 10.1038/s41576-018-0035-9
Sloan DB, 2017, MOL ECOL, V26, P2212, DOI 10.1111/mec.13959
Smith CCR, 2018, P NATL ACAD SCI USA, V115, P6768, DOI 10.1073/pnas.1803361115
Tigano M, 2021, NATURE, V591, P477, DOI 10.1038/s41586-021-03269-w
Vaught RC, 2020, J EVOLUTION BIOL, V33, P694, DOI 10.1111/jeb.13605
Vives-Bauza C, 2006, NEUROSCI LETT, V391, P136, DOI 10.1016/j.neulet.2005.08.049
Wallace DC, 2010, P NATL ACAD SCI USA, V107, P8947, DOI 10.1073/pnas.0914635107
Whittall JB, 2006, MOL ECOL, V15, P4645, DOI 10.1111/j.1365-294X.2006.03114.x
Zar J., 1999, Biostatistical analysis, V4nd, P523
Zhou YY, 2019, NAT COMMUN, V10, DOI 10.1038/s41467-019-09234-6
NR 64
TC 2
Z9 2
U1 0
U2 12
PU WILEY
PI HOBOKEN
PA 111 RIVER ST, HOBOKEN 07030-5774, NJ USA
SN 2045-7758
J9 ECOL EVOL
JI Ecol. Evol.
PD SEP
PY 2021
VL 11
IS 17
BP 12003
EP 12010
DI 10.1002/ece3.7966
EA JUL 2021
PG 8
WC Ecology; Evolutionary Biology
WE Science Citation Index Expanded (SCI-EXPANDED)
SC Environmental Sciences & Ecology; Evolutionary Biology
GA UN4RM
UT WOS:000678156600001

PM 34522356
OA Green Published
DA 2024-10-21
ER

PT J
AU Gerow, CM
Rapin, N
Voordouw, MJ
Elliot, M
Misra, V
Subudhi, S
AF Gerow, Caleigh M.
Rapin, Noreen
Voordouw, Maarten J.
Elliot, Melanie
Misra, Vikram
Subudhi, Sonu
TI Arousal from hibernation and reactivation of <i>Eptesicus fuscus</i>
gammaherpesvirus (<i>Ef</i>HV) in big brown bats
SO TRANSBOUNDARY AND EMERGING DISEASES
LA English
DT Article
DE Eptesicus fuscus; gammaherpesvirus; hibernation; reactivation; stress
ID B-CELLS; INFECTION; LATENCY; SPILLOVER; ANTIBODY; LESSONS; STRESS;
FLIGHT; HOST
AB Many viruses that cause serious and often fatal disease in humans have spilled over from bats. Recent evidence suggests that stress may enhance virus shedding by bats increasing the possibility of transmission to other species. To understand the reasons for spillover is therefore important to determine the molecular pathways that link stress to virus reactivation and shedding in bats. We recently isolated and characterized a gammaherpesvirus (Eptesicus fuscus herpesvirus, EfHV) autochthonous to North American big brown bats. Since herpesviruses are known to reactivate from latent infections in response to a wide variety of stressors, EfHV presents us with an opportunity to study how physiological, behavioural or environmental changes may influence the big brown bats' relationship with EfHV. To understand the biology of the virus and how the extended periods of torpor experienced by these bats during hibernation along with the stress of arousal might influence the virus-host relationship, we attempted to detect the virus in the blood of wild-caught non-hibernating bats as well as captive bats arising from hibernation. We compared the prevalence of EfHV in the blood (using PCR) and EfHV-specific antibodies (using ELISA) between captive hibernating bats and wild-caught non-hibernating bats. We detected EfHV only in the blood of captive hibernating bats (27.8% = 10/36) and not in wild-caught non-hibernating bats (0.0% = 0/43). In contrast, the EfHV-specific antibody titres were higher in the non-hibernating bats compared to the hibernating bats. Our study suggests that: (a) viral DNA in blood indicates reactivation from

latency, (b) long periods of hibernation lead to suppression of immunity, (c) stress of arousal from hibernation reactivates the virus in bats with lower levels of anti-viral immunity (indicated by humoral immune response), and (d) levels of anti-viral immunity increase in non-hibernating bats following reactivation.
C1 [Gerow, Caleigh M.; Rapin, Noreen; Voordouw, Maarten J.; Misra, Vikram; Subudhi, Sonu] Univ Saskatchewan, Dept Microbiol, Western Coll Vet Microbiol, Saskatoon, SK, Canada.
[Elliot, Melanie] Wildlife Rehab Soc Saskatchewan, Saskatoon, SK, Canada.
C3 University of Saskatchewan
RP Subudhi, S (corresponding author), Univ Saskatchewan, Dept Microbiol, Western Coll Vet Microbiol, Saskatoon, SK, Canada.
EM sonu.subudhi@usask.ca
RI Misra, Vikram/AAC-4266-2020; Voordouw, Maarten/G-3686-2013; Subudhi,
Sonu/L-8509-2019
OI Subudhi, Sonu/0000-0002-5937-1880
FU Research Office of the Western College of Veterinary Medicine (WCVM) in
Saskatoon, Saskatchewan; University of Saskatchewan Devolved
Scholarship; Natural Sciences and Engineering Research Council (NSERC)
of Canada
FX CG was a recipient of a 2018 Interprovincial Undergraduate Student
Research Award from the Research Office of the Western College of
Veterinary Medicine (WCVM) in Saskatoon, Saskatchewan. SS was supported
by a University of Saskatchewan Devolved Scholarship. The work was
funded by a Discovery Grant from the Natural Sciences and Engineering
Research Council (NSERC) of Canada to VM.
CR Baker ML, 2013, ZOONOSES PUBLIC HLTH, V60, P104, DOI 10.1111/j.1863-2378.2012.01528.x
Barton E, 2011, ANNU REV IMMUNOL, V29, P351, DOI 10.1146/annurev-immunol-072710-081639
Bouma HR, 2011, P NATL ACAD SCI USA, V108, P2052, DOI 10.1073/pnas.1008823108
BRIGHAM RM, 1991, CAN J ZOOL, V69, P117, DOI 10.1139/z91-017
Chow BA, 2013, PLOS ONE, V8, DOI 10.1371/journal.pone.0066119
Cogswell-Hawkinson A, 2012, J VIROL, V86, P5791, DOI 10.1128/JVI.00201-12
Davy CM, 2018, SCI REP-UK, V8, DOI 10.1038/s41598-018-33975-x
Development Core TeamR, 2011, R LANG ENV STAT COMP
Flaño E, 2003, J IMMUNOL, V170, P3828, DOI 10.4049/jimmunol.170.7.3828
Flaño E, 2002, J EXP MED, V196, P1363, DOI 10.1084/jem.20020890
Gangappa S, 2002, J VIROL, V76, P11460, DOI 10.1128/JVI.76.22.11460-11468.2002
Kessler MK, 2018, ANN NY ACAD SCI, V1429, P78, DOI 10.1111/nyas.13910
Killick R, 2012, J AM STAT ASSOC, V107, P1590, DOI

10.1080/01621459.2012.737745
Killick R, 2014, J STAT SOFTW, V58, P1
Kim IJ, 2002, J IMMUNOL, V168, P3958, DOI 10.4049/jimmunol.168.8.3958
Lardeux F, 2016, MEM I OSWALDO CRUZ, V111, P501, DOI 10.1590/0074-02760160119
Lee M, 2002, J NEUROCHEM, V82, P867, DOI 10.1046/j.1471-4159.2002.01022.x
Li XD, 2011, J VIROL, V85, P715, DOI 10.1128/JVI.01742-10
Lieberman PM, 2013, NAT REV MICROBIOL, V11, P863, DOI 10.1038/nrmicro3135
Logan SM, 2016, MOL CELL BIOCHEM, V414, P115, DOI 10.1007/s11010-016-2665-6
Luis AD, 2013, P ROY SOC B-BIOL SCI, V280, DOI 10.1098/rspb.2012.2753
Matar CG, 2014, J VIROL, V88, P11600, DOI 10.1128/JVI.01876-14
Nash AA, 2001, PHILOS T R SOC B, V356, P569, DOI 10.1098/rstb.2000.0779
O'Shea TJ, 2014, EMERG INFECT DIS, V20, P741, DOI 10.3201/eid2005.130539
Olival KJ, 2017, NATURE, V546, P646, DOI 10.1038/nature22975
Orr AL, 2009, COMP BIOCHEM PHYS A, V153, P213, DOI 10.1016/j.cbpa.2009.02.016
Plowright RK, 2015, P ROY SOC B-BIOL SCI, V282, DOI 10.1098/rspb.2014.2124
Schountz T, 2017, FRONT IMMUNOL, V8, DOI 10.3389/fimmu.2017.01098
Stevenson PG, 2009, J GEN VIROL, V90, P2317, DOI 10.1099/vir.0.013300-0
Subudhi S, 2018, VIROLOGY, V516, P227, DOI 10.1016/j.virol.2018.01.024
Weck KE, 1996, J VIROL, V70, P6775, DOI 10.1128/JVI.70.10.6775-6780.1996
Zhang GJ, 2013, SCIENCE, V339, P456, DOI 10.1126/science.1230835
NR 32
TC 12
Z9 12
U1 1
U2 22
PU WILEY
PI HOBOKEN
PA 111 RIVER ST, HOBOKEN 07030-5774, NJ USA
SN 1865-1674
EI 1865-1682
J9 TRANSBOUND EMERG DIS
JI Transbound. Emerg. Dis.
PD MAR
PY 2019
VL 66
IS 2
BP 1054
EP 1062
DI 10.1111/tbed.13102
PG 9
WC Infectious Diseases; Veterinary Sciences

WE Science Citation Index Expanded (SCI-EXPANDED)
SC Infectious Diseases; Veterinary Sciences
GA HP0YQ
UT WOS:000461392900049
PM 30554475
OA gold
DA 2024-10-21
ER

PT J
AU Epstein, JH
   Baker, ML
   Zambrana-Torrelio, C
   Middleton, D
   Barr, JA
   DuBovi, E
   Boyd, V
   Pope, B
   Todd, S
   Crameri, G
   Walsh, A
   Pelican, K
   Fielder, MD
   Davies, AJ
   Wang, LF
   Daszak, P
AF Epstein, Jonathan H.
   Baker, Michelle L.
   Zambrana-Torrelio, Carlos
   Middleton, Deborah
   Barr, Jennifer A.
   DuBovi, Edward
   Boyd, Victoria
   Pope, Brian
   Todd, Shawn
   Crameri, Gary
   Walsh, Allyson
   Pelican, Katey
   Fielder, Mark D.
   Davies, Angela J.
   Wang, Lin-Fa
   Daszak, Peter
TI Duration of Maternal Antibodies against Canine Distemper Virus and
   Hendra Virus in Pteropid Bats
SO PLOS ONE
LA English
DT Article
ID NIPAH VIRUS; EMERGING VIRUSES; RESERVOIR HOSTS; INFECTION; HENIPAVIRUS;
   VACCINATION; GIGANTEUS; PLACENTA; MEASLES
AB Old World frugivorous bats have been identified as natural hosts for emerging zoonotic viruses of significant public health concern, including henipaviruses (Nipah and Hendra virus), Ebola virus, and

Marburg virus. Epidemiological studies of these viruses in bats
often utilize serology to describe viral dynamics, with particular
attention paid to juveniles, whose birth increases the overall
susceptibility of the population to a viral outbreak once maternal
immunity wanes. However, little is understood about bat immunology,
including the duration of maternal antibodies in neonates.
Understanding duration of maternally derived immunity is critical
for characterizing viral dynamics in bat populations, which may help
assess the risk of spillover to humans. We conducted two separate
studies of pregnant Pteropus bat species and their offspring to
measure the half-life and duration of antibodies to 1) canine
distemper virus antigen in vaccinated captive Pteropus hypomelanus;
and 2) Hendra virus in wild-caught, naturally infected Pteropus
alecto. Both of these pteropid bat species are known reservoirs for
henipaviruses. We found that in both species, antibodies were
transferred from dam to pup. In P. hypomelanus pups, titers against
CDV waned over a mean period of 228.6 days (95% CI: 185.4-271.8) and
had a mean terminal phase half-life of 96.0 days (CI 95%:
30.7-299.7). In P. alecto pups, antibodies waned over 255.13 days
(95% CI: 221.0-289.3) and had a mean terminal phase half-life of
52.24 days (CI 95%: 33.76-80.83). Each species showed a duration of
transferred maternal immunity of between 7.5 and 8.5 months, which
was longer than has been previously estimated. These data will allow
for more accurate interpretation of age-related Henipavirus
serological data collected from wild pteropid bats.
C1 [Epstein, Jonathan H.; Zambrana-Torrelio, Carlos; Daszak, Peter]
EcoHlth Alliance, New York, NY 10001 USA.
[Baker, Michelle L.; Middleton, Deborah; Barr, Jennifer A.; Boyd,
Victoria; Todd, Shawn; Crameri, Gary; Wang, Lin-Fa] CSIRO,
Australian Anim Hlth Lab, Geelong, Vic, Australia.
[DuBovi, Edward] Cornell Univ, Anim Hlth Diagnost Ctr, Ithaca, NY
USA.
[Pope, Brian] Lubee Bat Conservancy, Gainesville, FL USA.
[Walsh, Allyson] San Diego Zoo Inst Conservat Res, Escondido, CA
USA.
[Pelican, Katey] Univ Minnesota, Dept Vet Populat Med, St Paul,
MN 55108 USA.
[Epstein, Jonathan H.; Fielder, Mark D.; Davies, Angela J.] Univ
Kingston, Fac Sci Engn & Comp, Kingston Upon Thames KT1 2EE, Surrey,
England.
[Wang, Lin-Fa] Duke NUS Grad Med Sch, Program Emerging Infect
Dis, Singapore, Singapore.
C3 EcoHealth Alliance; Commonwealth Scientific & Industrial Research
Organisation (CSIRO); Cornell University; Zoological Society of
San
Diego; University of Minnesota System; University of Minnesota
Twin
Cities; Kingston University; National University of Singapore
RP Epstein, JH (corresponding author), EcoHlth Alliance, New York,
NY 10001 USA.
EM epstein@ecohealthalliance.org
RI Zambrana-Torrelio, Carlos/AAH-3911-2020; Todd, Shawn/
JJE-5658-2023; lu,
yuan/JZD-0832-2024; Crameri, Gary/H-8441-2013; Barr,

Jennifer/H-9254-2013; daszak, peter/U-4588-2017; Baker,
Michelle/C-9694-2013
OI Baker, Michelle/0000-0002-7993-9971; Zambrana-Torrelio,
Carlos/0000-0002-5614-7496; Todd, Shawn/0009-0009-2218-7842;
Barr,
Jennifer/0000-0001-5266-9142; Wang, Lin-Fa/0000-0003-2752-0535
FU National Institutes of Health (NIH) National Institute for
Allergy and
Infectious Disease [K08AI067549]; NIH Fogarty International
Center
[R01TW005869]; Australian Research Council [FT110100234];
Commonwealth
Science and Industrial Research Organization OCE Science Leaders
award;
Australian Research Council [FT110100234] Funding Source:
Australian
Research Council
FX Funding provided by National Institutes of Health (NIH) National
Institute for Allergy and Infectious Disease award K08AI067549;
NIH
Fogarty International Center award R01TW005869; Australian
Research
Council Future Fellowship FT110100234; Commonwealth Science and
Industrial Research Organization OCE Science Leaders award. The
funders
had no role in study design, data collection and analysis,
decision to
publish, or preparation of the manuscript.
CR Amman BR, 2012, PLOS PATHOG, V8, DOI 10.1371/journal.ppat.1002877
Baker ML, 2013, ZOONOSES PUBLIC HLTH, V60, P104, DOI 10.1111/
j.1863-2378.2012.01528.x
Baker ML, 2010, IMMUNOGENETICS, V62, P173, DOI 10.1007/
s00251-010-0425-4
Bossart KN, 2007, J VIROL METHODS, V142, P29, DOI 10.1016/
j.jviromet.2007.01.003
Bratsch S, 2011, DEV COMP IMMUNOL, V35, P421, DOI 10.1016/
j.dci.2010.06.004
Broder CC, 2012, CURR OPIN VIROL, V2, P176, DOI 10.1016/
j.coviro.2012.02.016
Butler JE, 2011, DEV COMP IMMUNOL, V35, P273, DOI 10.1016/
j.dci.2010.08.011
Cáceres VM, 2000, CLIN INFECT DIS, V31, P110, DOI 10.1086/313926
Calisher CH, 2006, CLIN MICROBIOL REV, V19, P531, DOI 10.1128/
CMR.00017-06
Chua KB, 2002, MICROBES INFECT, V4, P145, DOI 10.1016/
S1286-4579(01)01522-2
Daniels P, 2001, MICROBES INFECT, V3, P289, DOI 10.1016/
S1286-4579(01)01382-X
Drexler JF, 2011, EMERG INFECT DIS, V17, P449, DOI 10.3201/
eid1703.100526
Enders AC, 2012, PLACENTA, V33, P319, DOI 10.1016/
j.placenta.2012.02.008
Enders AC, 2004, PLACENTA, V25, pS3, DOI 10.1016/
j.placenta.2004.01.011

Epstein JH, 2008, EMERG INFECT DIS, V14, P1309, DOI 10.3201/eid1408.071492
Epstein Jonathan H, 2006, Curr Infect Dis Rep, V8, P59, DOI 10.1007/s11908-006-0036-2
Field H., 2004, ECOLOGY HENDRA VIRUS
Hall L.S., 2000, Flying foxes: fruit and blossom bats of Australia
Halpin K, 2000, J GEN VIROL, V81, P1927, DOI 10.1099/0022-1317-81-8-1927
Halpin K, 2007, CLIN INFECT DIS, V44, P711, DOI 10.1086/511078
Halpin K, 2011, AM J TROP MED HYG, V85, P946, DOI 10.4269/ajtmh.2011.10-0567
Hasebe F, 2012, EMERG INFECT DIS, V18, P536, DOI 10.3201/eid1803.111121
Hayman DTS, 2008, PLOS ONE, V3, DOI 10.1371/journal.pone.0002739
Henipavirus Ecology Research Group (HERG), 2006, P186
Hsu VP, 2004, EMERG INFECT DIS, V10, P2082, DOI 10.3201/eid1012.040701
Iehlé C, 2007, EMERG INFECT DIS, V13, P159, DOI 10.3201/eid1301.060791
Johara MY, 2001, EMERG INFECT DIS, V7, P439
Jones DP., 2000, Mammalian Species, V639, P1, DOI DOI 10.1644/1545-1410(2000)639<0001:PH>2.0.CO;2
Karim KB, 1996, ANN ANAT, V178, P523, DOI 10.1016/S0940-9602(96)80110-5
Kunz TH., 2000, Mammalian Species, V642, P1, DOI DOI 10.1644/1545-1410(2000)642<0001:PV>2.0.CO;2
Larson LJ, 2006, VET THER, V7, P113
Leuridan E, 2007, VACCINE, V25, P6296, DOI 10.1016/j.vaccine.2007.06.020
Leuridan E, 2010, BMJ, V340, pc1626, DOI 10.1136/bmj.c1626
Mestecky J, 2005, MUCOSAL IMMUNOLOGY, 3RD EDITION, P1
Middleton DJ, 2007, J COMP PATHOL, V136, P266, DOI 10.1016/j.jcpa.2007.03.002
Mouzin DE, 2004, JAVMA-J AM VET MED A, V224, P55, DOI 10.2460/javma.2004.224.55
Müller T, 2005, J VET MED B, V52, P432, DOI 10.1111/j.1439-0450.2005.00896.x
Olson JG, 2002, EMERG INFECT DIS, V8, P987, DOI 10.3201/eid0809.010515
Pastoret PP, 2007, J COMP PATHOL, V137, pS2, DOI 10.1016/j.jcpa.2007.04.003
Pentsuk N, 2009, BIRTH DEFECTS RES B, V86, P328, DOI 10.1002/bdrb.20201
Philbey AW, 2008, AUST VET J, V86, P449, DOI 10.1111/j.1751-0813.2008.00361.x
Plowright RK, 2008, P ROY SOC B-BIOL SCI, V275, P861, DOI 10.1098/rspb.2007.1260
Plowright RK, 2011, P ROY SOC B-BIOL SCI, V278, P3703, DOI 10.1098/rspb.2011.0522
Rahman SA, 2010, EMERG INFECT DIS, V16, P1990, DOI 10.3201/eid1612.091790
Sétien AA, 1998, VACCINE, V16, P1122, DOI 10.1016/S0264-410X(98)80108-4

Sohayati AR, 2011, EPIDEMIOL INFECT, V139, P1570, DOI 10.1017/S0950268811000550
Sohayati AL, 2007, PTEROPUS HYPOMELANUS
TALIAFERRO WH, 1957, J CELL COMPAR PHYSL, V50, P1, DOI 10.1002/jcp.1030500403
Wacharapluesadee S, 2005, EMERG INFECT DIS, V11, P1949, DOI 10.3201/eid1112.050613
Williamson MM, 2000, J COMP PATHOL, V122, P201, DOI 10.1053/jcpa.1999.0364
Wynne JW, PLOS ONE
Zaki AM, 2012, NEW ENGL J MED, V367, P1814, DOI 10.1056/NEJMoa1211721
NR 52
TC 34
Z9 38
U1 0
U2 33
PU PUBLIC LIBRARY SCIENCE
PI SAN FRANCISCO
PA 1160 BATTERY STREET, STE 100, SAN FRANCISCO, CA 94111 USA
SN 1932-6203
J9 PLOS ONE
JI PLoS One
PD JUN 27
PY 2013
VL 8
IS 6
AR e67584
DI 10.1371/journal.pone.0067584
PG 8
WC Multidisciplinary Sciences
WE Science Citation Index Expanded (SCI-EXPANDED)
SC Science & Technology - Other Topics
GA 174IX
UT WOS:000321150000090
PM 23826322
OA Green Accepted, gold, Green Published, Green Submitted
DA 2024-10-21
ER

PT J
AU Power, ML
Foley, NM
Jones, G
Teeling, EC
AF Power, Megan L.
Foley, Nicole M.
Jones, Gareth
Teeling, Emma C.
TI Taking flight: An ecological, evolutionary and genomic perspective on
bat telomeres
SO MOLECULAR ECOLOGY
LA English

DT Article
DE bats; comparative genomics; life-history; telomerase; telomere dynamics
ID BODY-MASS; TEMPERATURE REGULATION; LIFE; LENGTH; TORPOR; LONGEVITY;
HIBERNATION; DYNAMICS; BIOLOGY; THERMOREGULATION
AB Over 20% of all living mammals are bats (order Chiroptera). Bats possess extraordinary adaptations including powered flight, laryngeal echolocation and a unique immune system that enables them to tolerate a diversity of viral infections without presenting clinical disease symptoms. They occupy multiple trophic niches and environments globally. Significant physiological and ecological diversity occurs across the order. Bats also exhibit extreme longevity given their body size with many species showing few signs of ageing. The molecular basis of this extended longevity has recently attracted attention. Telomere maintenance potentially underpins bats' extended healthspan, although functional studies are still required to validate the causative mechanisms. In this review, we detail the current knowledge on bat telomeres, telomerase expression, and how these relate to ecology, longevity and life-history strategies. Patterns of telomere shortening and telomerase expression vary across species, and comparative genomic analyses suggest that alternative telomere maintenance mechanisms evolved in the longest-lived bats. We discuss the unique challenges faced when working with populations of wild bats and highlight ways to advance the field including expanding long-term monitoring across species that display contrasting life-histories and occupy different environmental niches. We further review how new high quality, chromosome-level genome assemblies can enable us to uncover the molecular mechanisms governing telomere dynamics and how phylogenomic analyses can reveal the adaptive significance of telomere maintenance and variation in bats.
C1 [Power, Megan L.; Teeling, Emma C.] Univ Coll Dublin, Sci Ctr West, Sch Biol & Environm Sci, Belfield, Ireland.
[Foley, Nicole M.] Texas A&M Univ, Vet Integrat Biosci, College Stn, TX USA.
[Jones, Gareth] Univ Bristol, Sch Biol Sci, Bristol, Avon, England.
C3 University College Dublin; Texas A&M University System; Texas A&M University College Station; University of Bristol
RP Teeling, EC (corresponding author), Univ Coll Dublin, Sci Ctr West, Sch Biol & Environm Sci, Dublin 4, Ireland.
EM emma.teeling@ucd.ie
OI Teeling, Emma/0000-0002-3309-1346; Jones, Gareth/0000-0002-1904-3735;
Power, Megan/0000-0001-7402-3254; Foley, Nicole/0000-0002-8169-9436
FU Irish Research Council [IRCLA/2017/58]; Science Foundation Ireland
[19/FFP/6790]; IReL; WOA Institution: University College Dublin; Blended
DEAL: IReL; Irish Research Council (IRC) [IRCLA/2017/58] Funding Source:
Irish Research Council (IRC)

FX Irish Research Council, Grant/Award Number: IRCLA/2017/58; Science
Foundation Ireland, Grant/Award Number: 19/FFP/6790; Open access funding
provided by IReL. WOA Institution: University College Dublin; Blended
DEAL: IReL
CR Alkan C, 2011, NAT METHODS, V8, P61, DOI 10.1038/NMETH.1527
Altringham John, 2016, P35
Alviola P.A., 2015, MUSEUM PUBLICATIONS, V4, P1
Ammerman LK, 2019, J HERED, V110, P675, DOI 10.1093/jhered/esz044
Anthony SJ, 2017, VIRUS EVOL, V3, DOI 10.1093/ve/vex012
Asghar M, 2015, SCIENCE, V347, P436, DOI 10.1126/science.1261121
Asghar M, 2016, P ROY SOC B-BIOL SCI, V283, DOI 10.1098/rspb.2016.1184
Austad SN, 2010, J COMP PATHOL, V141, pS10, DOI 10.1016/j.jcpa.2009.10.024
Baird DM, 2003, NAT GENET, V33, P203, DOI 10.1038/ng1084
Banerjee A, 2020, FRONT IMMUNOL, V11, DOI 10.3389/fimmu.2020.00026
Banszerus VL, 2019, INT J MOL SCI, V20, DOI 10.3390/ijms20123032
Barclay RMR, 2004, CAN J ZOOL, V82, P688, DOI 10.1139/Z04-057
Barclay RMR, 2003, BAT ECOLOGY, P209
Beirne C, 2014, PLOS ONE, V9, DOI 10.1371/journal.pone.0108964
Bérénos C, 2014, MOL ECOL, V23, P3434, DOI 10.1111/mec.12827
Blackburn EH, 2005, FEBS LETT, V579, P859, DOI 10.1016/j.febslet.2004.11.036
BLACKBURN EH, 1991, NATURE, V350, P569, DOI 10.1038/350569a0
Boldogh S, 2007, ACTA CHIROPTEROL, V9, P527, DOI 10.3161/1733-5329(2007)9[527:TEOTIO]2.0.CO;2
BONACCORSO FJ, 1992, J MAMMAL, V73, P365, DOI 10.2307/1382071
Bondarenco A, 2013, J COMP PHYSIOL B, V183, P1113, DOI 10.1007/s00360-013-0779-7
Boonman A, 2014, CURR BIOL, V24, DOI 10.1016/j.cub.2014.10.077
Brown CR, 1999, S AFR J ZOOL, V34, P166
Brunet-Rossinni AK, 2004, MECH AGEING DEV, V125, P11, DOI 10.1016/j.mad.2003.09.003
Brunet-Rossinni AK, 2004, BIOGERONTOLOGY, V5, P211, DOI 10.1023/B:BGEN.0000038022.65024.d8
Brunet-Rossinni Anja K., 2009, P315
Bunkley JP, 2015, ETHOLOGY, V121, P1116, DOI 10.1111/eth.12428
Chatelain M, 2020, ECOL LETT, V23, P381, DOI 10.1111/ele.13426
Chattopadhyay B, 2020, GENOME BIOL EVOL, V12, P259, DOI 10.1093/gbe/evaa030
Coburn DK, 1998, OECOLOGIA, V113, P467, DOI 10.1007/s004420050399
Codd V, 2013, NAT GENET, V45, P422, DOI 10.1038/ng.2528
Cram DL, 2017, P ROY SOC B-BIOL SCI, V284, DOI 10.1098/rspb.2017.1383
Crichton Elizabeth G., 2000, P295, DOI 10.1016/B978-012195670-7/50008-4
Czenze ZJ, 2017, OECOLOGIA, V183, P1, DOI 10.1007/s00442-016-3707-1
Czenze ZJ, 2015, J COMP PHYSIOL B, V185, P575, DOI 10.1007/s00360-015-0900-1

Davey JL, 2010, BRIEF FUNCT GENOMICS, V9, P416, DOI 10.1093/bfgp/elq031
David KT, 2019, MOL BIOL EVOL, V36, P1624, DOI 10.1093/molbev/msz117
Ding ZH, 2014, NUCLEIC ACIDS RES, V42, DOI 10.1093/nar/gku181
Dlouha D, 2014, PHYSIOL RES, V63, pS343, DOI 10.33549/physiolres.932856
Drexler JF, 2012, NAT COMMUN, V3, DOI 10.1038/ncomms1796
Dugdale HL, 2018, PHILOS T R SOC B, V373, DOI 10.1098/rstb.2016.0450
Eastwood JR, 2019, MOL ECOL, V28, P1127, DOI 10.1111/mec.15002
Eshar D, 2010, LAB ANIMAL, V39, P175, DOI 10.1038/laban0610-175
Fairlie J, 2016, AGING CELL, V15, P140, DOI 10.1111/acel.12417
Farmery JHR, 2018, SCI REP-UK, V8, DOI 10.1038/s41598-017-14403-y
Faure PA, 2009, J MAMMAL, V90, P1148, DOI 10.1644/08-MAMM-A-332.1
Finch D, 2020, ENVIRON POLLUT, V263, DOI 10.1016/j.envpol.2020.114405
Fleischer T, 2017, SCI REP-UK, V7, DOI 10.1038/s41598-017-06392-9
Foley NM, 2020, MOL ECOL, V29, P2963, DOI 10.1111/mec.15395
Foley NM, 2018, SCI ADV, V4, DOI 10.1126/sciadv.aao0926
Fox S, 2008, ACTA CHIROPTEROL, V10, P271, DOI 10.3161/150811008X414845
Frick Winifred F., 2016, P245
Froy H, 2017, SCI REP-UK, V7, DOI 10.1038/s41598-017-09861-3
Gaisler J., 2003, VESPERTILIO, V7, P3
Geiser F, 2011, INTEGR COMP BIOL, V51, P337, DOI 10.1093/icb/icr042
GENOUD M, 1990, COMP BIOCHEM PHYS A, V97, P229, DOI 10.1016/0300-9629(90)90177-T
Giraudeau M, 2019, BIOL LETTERS, V15, DOI 10.1098/rsbl.2019.0190
Gomes NMV, 2011, AGING CELL, V10, P761, DOI 10.1111/j.1474-9726.2011.00718.x
Gorbunova V, 2009, MECH AGEING DEV, V130, P3, DOI 10.1016/j.mad.2008.02.008
Graham JL, 2019, MOL ECOL, V28, P114, DOI 10.1111/mec.14952
Griffin Donald R., 1944, SCIENCE, V100, P589, DOI 10.1126/science.100.2609.589
Grunst ML, 2019, SCI TOTAL ENVIRON, V662, P266, DOI 10.1016/j.scitotenv.2018.12.469
Gunnell GF, 2012, CAM STUD MORPHOL MOL, P1, DOI 10.1017/CBO9781139045599
Heidinger BJ, 2012, P NATL ACAD SCI USA, V109, P1743, DOI 10.1073/pnas.1113306109
Hoelzl F, 2016, J EXP BIOL, V219, P2469, DOI 10.1242/jeb.140871
Holland RA, 2004, J EXP BIOL, V207, P4361, DOI 10.1242/jeb.01288
Horvath S, 2013, GENOME BIOL, V14, DOI 10.1186/gb-2013-14-10-r115
Huang ZX, 2019, NAT ECOL EVOL, V3, P1110, DOI 10.1038/s41559-019-0913-3
Ilska-Warner JJ, 2019, FRONT GENET, V10, DOI 10.3389/fgene.2019.01048
Ineson KM, 2020, J MAMMAL, V101, P958, DOI 10.1093/jmammal/gyaa064
Injaiana AS, 2019, GEN COMP ENDOCR, V276, P14, DOI 10.1016/j.ygcen.2019.02.017

Jebb D, 2020, NATURE, V583, P578, DOI 10.1038/s41586-020-2486-3
Jones Gareth, 2009, Endangered Species Research, V8, P93, DOI 10.3354/esr00182
Jylhävä J, 2017, EBIOMEDICINE, V21, P29, DOI 10.1016/j.ebiom.2017.03.046
Kardos M, 2015, HEREDITY, V115, P63, DOI 10.1038/hdy.2015.17
Karell P, 2017, P ROY SOC B-BIOL SCI, V284, DOI 10.1098/rspb.2017.1127
Kelm DH, 2007, J COMP PHYSIOL B, V177, P667, DOI 10.1007/s00360-007-0164-5
Kesäniemi J, 2019, SCI REP-UK, V9, DOI 10.1038/s41598-018-37164-8
Kirby R, 2019, SCI REP-UK, V9, DOI 10.1038/s41598-019-38937-5
KULZER E, 1970, Z VERGL PHYSIOL, V69, P426, DOI 10.1007/BF00333769
Kunz T.H., 2000, REPROD BIOL BATS, P321
Lee M, 2017, METHODS, V114, P4, DOI 10.1016/j.ymeth.2016.08.008
Lee SS, 2015, CELL REP, V13, DOI 10.1016/j.celrep.2015.10.035
Lee Y, 2019, AGING-US, V11, P5876, DOI 10.18632/aging.102230
Levin E, 2015, P ROY SOC B-BIOL SCI, V282, DOI 10.1098/rspb.2014.2781
Liu JN, 2011, J COMP PHYSIOL B, V181, P125, DOI 10.1007/s00360-010-0509-3
Lorenzini A, 2005, MECH AGEING DEV, V126, P1130, DOI 10.1016/j.mad.2005.05.004
Lorenzini A, 2009, MECH AGEING DEV, V130, P784, DOI 10.1016/j.mad.2009.10.004
Maina JN, 2000, J EXP BIOL, V203, P3045
Matzenbacher CA, 2019, SCI REP-UK, V9, DOI 10.1038/s41598-019-42804-8
Meillère A, 2015, BIOL LETTERS, V11, DOI 10.1098/rsbl.2015.0559
Meniri M, 2020, BEHAV ECOL, V31, P475, DOI 10.1093/beheco/arz205
Monaghan P, 2018, PHILOS T R SOC B, V373, DOI 10.1098/rstb.2016.0435
Moratelli R, 2015, MEM I OSWALDO CRUZ, V110, P1
Morgan CC, 2013, BMC EVOL BIOL, V13, DOI 10.1186/1471-2148-13-251
Munshi-South J, 2010, AGEING RES REV, V9, P12, DOI 10.1016/j.arr.2009.07.006
Nersisyan L, 2015, PLOS ONE, V10, DOI 10.1371/journal.pone.0125201
Nussey DH, 2014, METHODS ECOL EVOL, V5, P299, DOI 10.1111/2041-210X.12161
O'Mara MT, 2017, ROY SOC OPEN SCI, V4, DOI 10.1098/rsos.171359
Oizumi Y, 2021, NAT COMMUN, V12, DOI 10.1038/s41467-020-20595-1
Olsson M, 2011, PLOS ONE, V6, DOI 10.1371/journal.pone.0017473
Olsson M, 2011, MOL ECOL, V20, P2085, DOI 10.1111/j.1365-294X.2011.05085.x
Orr TJ, 2014, BIOL REV, V89, P889, DOI 10.1111/brv.12085
Ouyang JQ, 2017, GLOBAL CHANGE BIOL, V23, P4987, DOI 10.1111/gcb.13756
Park KJ, 2000, FUNCT ECOL, V14, P580, DOI 10.1046/j.1365-2435.2000.t01-1-00460.x
Power ML, 2021, MOL ECOL RESOUR, V21, P421, DOI 10.1111/1755-0998.13276
Prescott J, 2011, PLOS ONE, V6, DOI 10.1371/journal.pone.0019635

Puechmaille SJ, 2011, PLOS ONE, V6, DOI 10.1371/journal.pone.0019167
Racey P.A., 1982, P57
Racey Paul A., 2000, P363, DOI 10.1016/B978-012195670-7/50010-2
Ransome R.D., 2000, CONV CONS EUR WILDL, V109
Ransome RD, 1995, PHILOS T ROY SOC B, V350, P153, DOI 10.1098/rstb.1995.0149
Ransome RD., 1990, NATURAL HIST HIBERNA, p235 pp
Reher S, 2018, J COMP PHYSIOL B, V188, P1015, DOI 10.1007/s00360-018-1171-4
Rhie A, 2021, NATURE, V592, P737, DOI 10.1038/s41586-021-03451-0
ROHME D, 1981, P NATL ACAD SCI-BIOL, V78, P5009, DOI 10.1073/pnas.78.8.5009
Schountz T, 2014, VIRUSES-BASEL, V6, P4880, DOI 10.3390/v6124880
Schuh AJ, 2017, SCI REP-UK, V7, DOI 10.1038/s41598-017-07824-2
Seluanov A, 2007, AGING CELL, V6, P45, DOI 10.1111/j.1474-9726.2006.00262.x
Shultz AJ, 2019, ELIFE, V8, DOI 10.7554/eLife.41815
Simmons N.B., 2020, Bat species of the world: A taxonomic and geographic database
Simmons NB, 2005, MAMMAL SPECIES WORLD, V1, P312, DOI [DOI 10.1111/J.1365-2656.2010.01742.X, 10.1093/acprof]
Stevens RD, 2001, MAMM BIOL, V66, P178
Stone EL, 2012, GLOBAL CHANGE BIOL, V18, P2458, DOI 10.1111/j.1365-2486.2012.02705.x
Stone EL, 2009, CURR BIOL, V19, P1123, DOI 10.1016/j.cub.2009.05.058
Sudyka J, 2019, BIOESSAYS, V41, DOI 10.1002/bies.201900095
Sudyka J, 2019, BIOL LETTERS, V15, DOI 10.1098/rsbl.2018.0637
Teeling EC, 2018, ANNU REV ANIM BIOSCI, V6, P23, DOI 10.1146/annurev-animal-022516-022811
Terrien J, 2018, INT J OBESITY, V42, P221, DOI 10.1038/ijo.2017.195
Tian X, 2019, PHILOS T R SOC B, V374, DOI [10.1098/rstb.2018.0319, 10.1098/rstb.2016.0443]
Toussaint DC, 2013, MAMM BIOL, V78, P365, DOI 10.1016/j.mambio.2012.10.001
Toussaint DC, 2010, MAMM BIOL, V75, P466, DOI 10.1016/j.mambio.2009.06.001
Turbill C, 2013, BIOL LETTERS, V9, DOI 10.1098/rsbl.2012.1095
Turbill C, 2011, P ROY SOC B-BIOL SCI, V278, P3355, DOI 10.1098/rspb.2011.0190
Tutton S, 2016, EMBO J, V35, P193, DOI 10.15252/embj.201490880
van Lieshout SHJ, 2022, MOL ECOL, V31, P5993, DOI 10.1111/mec.16014
van Lieshout SHJ, 2021, J EVOLUTION BIOL, V34, P296, DOI 10.1111/jeb.13728
van Lieshout SHJ, 2019, MOL ECOL, V28, P4152, DOI 10.1111/mec.15212
Vonhof MJ, 2006, MOL ECOL, V15, P241, DOI 10.1111/j.1365-294X.2005.02801.x
Wang L, 2011, BIOCHEMISTRY-MOSCOW+, V76, P1017, DOI 10.1134/S0006297911090057
Wang LF, 2011, CURR OPIN VIROL, V1, P649, DOI 10.1016/

j.coviro.2011.10.013
Whittemore K, 2019, P NATL ACAD SCI USA, V116, P15122, DOI 10.1073/pnas.1902452116
Wilkinson GS, 2021, NAT COMMUN, V12, DOI 10.1038/s41467-021-21900-2
Wilkinson GS, 2019, BIOL LETTERS, V15, DOI 10.1098/rsbl.2018.0860
Wilkinson GS, 2002, AGING CELL, V1, P124, DOI 10.1046/j.1474-9728.2002.00020.x
Woon AP, 2020, PLOS PATHOG, V16, DOI 10.1371/journal.ppat.1008412
Wright PGR, 2018, MOL ECOL RESOUR, V18, P1374, DOI 10.1111/1755-0998.12925
Yang ZH, 2007, MOL BIOL EVOL, V24, P1586, DOI 10.1093/molbev/msm088
Young AJ, 2018, PHILOS T R SOC B, V373, DOI 10.1098/rstb.2016.0452
Zhang GJ, 2013, SCIENCE, V339, P456, DOI 10.1126/science.1230835
NR 149
TC 8
Z9 8
U1 2
U2 27
PU WILEY
PI HOBOKEN
PA 111 RIVER ST, HOBOKEN 07030-5774, NJ USA
SN 0962-1083
EI 1365-294X
J9 MOL ECOL
JI Mol. Ecol.
PD DEC
PY 2022
VL 31
IS 23
SI SI
BP 6053
EP 6068
DI 10.1111/mec.16117
EA AUG 2021
PG 16
WC Biochemistry & Molecular Biology; Ecology; Evolutionary Biology
WE Science Citation Index Expanded (SCI-EXPANDED)
SC Biochemistry & Molecular Biology; Environmental Sciences & Ecology;
Evolutionary Biology
GA 6L5PB
UT WOS:000690867800001
PM 34387012
OA hybrid
DA 2024-10-21
ER

PT J
AU Prescott, J
Guito, JC
Spengler, JR

Arnold, CE
   Schuh, AJ
   Amman, BR
   Sealy, TK
   Guerrero, LW
   Palacios, GF
   Sanchez-Lockhart, M
   Albariño, CG
   Towner, JS
AF Prescott, Joseph
   Guito, Jonathan C.
   Spengler, Jessica R.
   Arnold, Catherine E.
   Schuh, Amy J.
   Amman, Brian R.
   Sealy, Tara K.
   Guerrero, Lisa W.
   Palacios, Gustavo F.
   Sanchez-Lockhart, Mariano
   Albarino, Cesar G.
   Towner, Jonathan S.
TI Rousette Bat Dendritic Cells Overcome Marburg Virus-Mediated Antiviral
   Responses by Upregulation of Interferon-Related Genes While
   Downregulating Proinflammatory Disease Mediators
SO MSPHERE
LA English
DT Article
DE Ebola virus; Marburg virus; immune response; rousette bat; viral
   hemorrhagic fever; virology
ID HEMORRHAGIC-FEVER; IMMUNE EVASION; EBOLA; IMMUNOBIOLOGY; IMMUNOLOGY;
   EXPRESSION; GENERATION; REVEALS; INNATE; CSF
AB Dysregulated and maladaptive immune responses are at the forefront of human diseases caused by infection with zoonotic viral hemorrhagic fever viruses. Elucidating mechanisms of how the natural animal reservoirs of these viruses coexist with these agents without overt disease, while permitting sufficient replication to allow for transmission and maintenance in a population, is important for understanding the viral ecology and spillover to humans. The Egyptian rousette bat (ERB) has been identified as a reservoir for Marburg virus (MARV), a filovirus and the etiological agent of the highly lethal Marburg virus disease. Little is known regarding how these bats immunologically respond to MARV infection. In humans, macrophages and dendritic cells (DCs) are primary targets of infection, and their dysregulation is thought to play a central role in filovirus diseases, by disturbing their normal functions as innate sensors and adaptive immune response facilitators while serving as amplification and dissemination agents for the virus. The infection status and responses to MARV in bat myeloid-lineage cells are uncharacterized and likely represent an important modulator of the bat's immune response to MARV infection. Here, we generate DCs from the bone marrow of rousette bats. Infection with a bat isolate of MARV resulted in a low level of transcription in these cells and

significantly downregulated DC maturation and adaptive immune-stimulatory pathways while simultaneously upregulating interferon-related pathogen-sensing pathways. This study provides a first insight into how the bat immune response is directed toward preventing aberrant inflammatory responses while mounting an antiviral response to defend against MARV infection.
IMPORTANCE Marburg viruses (MARVs) cause severe human disease resulting from aberrant immune responses. Dendritic cells (DCs) are primary targets of infection and are dysregulated by MARV. Dysregulation of DCs facilitates MARV replication and virus dissemination and influences downstream immune responses that result in immunopathology. Egyptian rousette bats (ERBs) are natural reservoirs of MARV, and infection results in virus replication and shedding, with asymptomatic control of the virus within weeks. The mechanisms that bats employ to appropriately respond to infection while avoiding disease are unknown. Because DC infection and modulation are important early events in human disease, we measured the transcriptional responses of ERB DCs to MARV. The significance of this work is in identifying cell type-specific coevolved responses between ERBs and MARV, which gives insight into how bat reservoirs are able to harbor MARV and permit viral replication, allowing transmission and maintenance in the population while simultaneously preventing immunopathogenesis.
C1 [Prescott, Joseph] Robert Koch Inst, Ctr Biol Threats & Special Pathogens, Berlin, Germany.
[Prescott, Joseph; Guito, Jonathan C.; Spengler, Jessica R.; Schuh, Amy J.; Amman, Brian R.; Sealy, Tara K.; Guerrero, Lisa W.; Albarino, Cesar G.; Towner, Jonathan S.] Ctr Dis Control & Prevent, Viral Special Pathogens Branch, Atlanta, GA 30333 USA.
[Arnold, Catherine E.] US Army Med Res Inst Infect Dis, Diagnost Syst Div, Ft Detrick, MD USA.
[Palacios, Gustavo F.; Sanchez-Lockhart, Mariano] US Army Med Res Inst Infect Dis, Ctr Genome Sci, Ft Detrick, MD USA.
[Sanchez-Lockhart, Mariano] Univ Nebraska Med Ctr, Dept Pathol & Microbiol, Omaha, NE USA.
C3 Robert Koch Institute; Centers for Disease Control & Prevention - USA;
United States Army Medical Research Institute of Infectious Diseases;
United States Department of Defense; United States Army; United States
Army Medical Research Institute of Infectious Diseases; United States
Department of Defense; United States Army; University of Nebraska
System; University of Nebraska Medical Center
RP Prescott, J (corresponding author), Robert Koch Inst, Ctr Biol Threats & Special Pathogens, Berlin, Germany.; Prescott, J; Towner, JS (corresponding author), Ctr Dis Control & Prevent, Viral Special Pathogens Branch, Atlanta, GA 30333 USA.
EM prescottj@rki.de; jit8@cdc.gov
RI Sanchez-Lockhart, Mariano/H-1321-2019; Spengler, Jessica/AAN-9099-2020;
Towner, Jonathan/ABC-3780-2020; Palacios, Gustavo/I-7773-2015
OI Prescott, Joseph/0000-0001-5718-5155; Arnold,

Catherine/0000-0002-1635-4941; Palacios, Gustavo/
0000-0001-5062-1938
FU DTRA grant [HDTRA-14-1-0016]
FX This study was funded in part by DTRA grant HDTRA-14-1-0016.
CR Albariño CG, 2018, PLOS ONE, V13, DOI 10.1371/
journal.pone.0201827
Alves DA, 2010, VET PATHOL, V47, P831, DOI
10.1177/0300985810378597
Amman BR, 2015, J WILDLIFE DIS, V51, P113, DOI
10.7589/2014-08-198
Arnold CE, 2018, VIRUSES-BASEL, V10, DOI 10.3390/v10110607
Banchereau J, 2000, ANNU REV IMMUNOL, V18, P767, DOI 10.1146/
annurev.immunol.18.1.767
Basler CF, 2015, VIROLOGY, V479, P122, DOI 10.1016/
j.virol.2015.03.030
Benjamini Y, 2001, ANN STAT, V29, P1165
Bosio CM, 2003, J INFECT DIS, V188, P1630, DOI 10.1086/379199
Cano LQ, 2019, BIORXIV, DOI [10.1101/739797, DOI 10.1101/739797]
Cilloniz C, 2011, J VIROL, V85, P9060, DOI 10.1128/JVI.00659-11
Cody V, 2005, VET IMMUNOL IMMUNOP, V103, P163, DOI 10.1016/
j.vetimm.2004.08.022
Coltart CEM, 2017, PHILOS T R SOC B, V372, DOI 10.1098/
rstb.2016.0297
Connor JH, 2015, J VIROL, V89, P9865, DOI 10.1128/JVI.01142-15
Davenport Bennett J., 2004, BMC Immunology, V5, DOI
10.1186/1471-2172-5-23
Easterbrook JD, 2007, P NATL ACAD SCI USA, V104, P15502, DOI
10.1073/pnas.0707453104
Feldmann H, 1996, J VIROL, V70, P2208, DOI 10.1128/
JVI.70.4.2208-2214.1996
Geisbert TW, 2003, AM J PATHOL, V163, P2347, DOI 10.1016/
S0002-9440(10)63591-2
Goldstein T, 2018, NAT MICROBIOL, V3, P1084, DOI 10.1038/
s41564-018-0227-2
Gustafson MP, 2015, PLOS ONE, V10, DOI 10.1371/
journal.pone.0121546
Ilinykh PA, 2015, J VIROL, V89, P7567, DOI 10.1128/JVI.00924-15
Jones MEB, 2019, VIRUSES-BASEL, V11, DOI 10.3390/v11030214
Liu YJ, 2001, CELL, V106, P259, DOI 10.1016/S0092-8674(01)00456-1
Lubaki NM, 2013, J VIROL, V87, P7471, DOI 10.1128/JVI.03316-12
Marques RE, 2018, IMMUNOLOGY, V155, P477, DOI 10.1111/imm.12988
Mayuzumi N, 2009, EUR J IMMUNOL, V39, P3331, DOI 10.1002/
eji.200939472
Negredo A, 2011, PLOS PATHOG, V7, DOI 10.1371/
journal.ppat.1002304
Pavlovich SS, 2018, CELL, V173, P1098, DOI 10.1016/
j.cell.2018.03.070
Pigott DM, 2015, T ROY SOC TROP MED H, V109, P366, DOI 10.1093/
trstmh/trv024
Prescott JB, 2017, NAT REV IMMUNOL, V17, P195, DOI 10.1038/
nri.2016.138
Ramanan P, 2011, VIRUSES-BASEL, V3, P1634, DOI 10.3390/v3091634
Rougeron V, 2015, J CLIN VIROL, V64, P111, DOI 10.1016/
j.jcv.2015.01.014

Schountz T, 2007, P NATL ACAD SCI USA, V104, P15496, DOI 10.1073/pnas.0707454104
Schountz T, 2014, VIRUSES-BASEL, V6, P4880, DOI 10.3390/v6124880
Schuh AJ, 2017, MICROBIOL AUST, V38, P12, DOI 10.1071/MA17005
Schuh AJ, 2017, NAT COMMUN, V8, DOI 10.1038/ncomms14446
Speranza E, 2017, VACCINES-BASEL, V5, DOI 10.3390/vaccines5030030
Ströher U, 2001, J VIROL, V75, P11025, DOI 10.1128/JVI.75.22.11025-11033.2001
Towner JS, 2009, PLOS PATHOG, V5, DOI 10.1371/journal.ppat.1000536
Wang J, 2012, MEDIAT INFLAMM, V2012, DOI 10.1155/2012/819636
Wu C, 2014, IMMUNITY, V41, P270, DOI 10.1016/j.immuni.2014.06.011
Wu ZG, 2010, IMMUNOLOGY, V129, P133, DOI 10.1111/j.1365-2567.2009.03129.x
Yang XL, 2019, NAT MICROBIOL, V4, P390, DOI 10.1038/s41564-018-0328-y
Zhou P, 2016, SCI REP-UK, V6, DOI 10.1038/srep38597
Zlotnik A, 2006, GENOME BIOL, V7, DOI 10.1186/gb-2006-7-12-243
NR 44
TC 14
Z9 15
U1 0
U2 11
PU AMER SOC MICROBIOLOGY
PI WASHINGTON
PA 1752 N ST NW, WASHINGTON, DC 20036-2904 USA
SN 2379-5042
J9 MSPHERE
JI mSphere
PD NOV-DEC
PY 2019
VL 4
IS 6
AR e00728-19
DI 10.1128/mSphere.00728-19
PG 14
WC Microbiology
WE Science Citation Index Expanded (SCI-EXPANDED)
SC Microbiology
GA KB0LY
UT WOS:000506191700027
PM 31801842
OA Green Published, gold
DA 2024-10-21
ER

PT J
AU Davis, A
Gordy, P
Rudd, R
Jarvis, JA
Bowen, RA
AF Davis, April
Gordy, Paul

Rudd, Robert
   Jarvis, Jodie A.
   Bowen, Richard A.
TI Naturally Acquired Rabies Virus Infections in Wild-Caught Bats
SO VECTOR-BORNE AND ZOONOTIC DISEASES
LA English
DT Article
DE Antibodies; Bats; Rabies; Viral isolation
ID NORTH-AMERICAN BATS; DYNAMICS; HISTORY
AB The study of a zoonotic disease requires an understanding of the disease incidence in animal reservoirs. Rabies incidence in bats submitted to diagnostic laboratories does not accurately reflect the true incidence in wild bat populations as a bias exists for testing bats that have been in contact with humans or pets. This article details the rabies incidence in two species of bats collected from natural settings without such bias. In this study, brain smears from 0.6% and 2.5% of wild-caught and apparently healthy Tadarida brasiliensis and Eptesicus fuscus, respectively, were positive for rabies virus (RV) antigen. Conversely, 92% of the grounded T. brasiliensis were positive for RV. Serology performed on captive colony and sick bats reveal an immune response to rabies. This work illustrates the complex interplay between immunity, disease state, and the conundrum of RV maintenance in bats.
C1 [Davis, April; Rudd, Robert; Jarvis, Jodie A.] Wadsworth Ctr, Dept Infect Dis, Slingerlands, NY 12159 USA.
   [Gordy, Paul; Bowen, Richard A.] Colorado State Univ, Dept Biomed Sci, Ft Collins, CO 80523 USA.
C3 Wadsworth Center; Colorado State University
RP Davis, A (corresponding author), Wadsworth Ctr, Dept Infect Dis, 5668 State Farm Rd, Slingerlands, NY 12159 USA.
EM add02@health.state.ny.us
FU National Science Foundation [0094959]; Emerging Frontiers; Direct For
   Biological Sciences [0094959] Funding Source: National Science
   Foundation
FX We are grateful to Thomas O'Shea, the bat crew, and Valerie Plane for
   their assistance in acquiring and maintaining the captive bat colonies.
   We thank Barbara French and BCI for their help and guidance in
   understanding bat health and husbandry. We are grateful for the valuable
   comments provided by two anonymous reviewers. Financial support:
   National Science Foundation (grant 0094959 to CSU, Fort Collins).
CR [Anonymous], NYSDOH 2009
   Badrane H, 2001, J VIROL, V75, P8096, DOI 10.1128/JVI.75.17.8096-8104.2001
   Betke M, 2008, J MAMMAL, V89, P18, DOI 10.1644/07-MAMM-A-011.1
   Bourhy H, 2008, J GEN VIROL, V89, P2673, DOI 10.1099/vir.0.2008/003913-0
   Brass D, 1994, RABIES BATS, P177
   Brass D, 1994, RABIES BATS, P157
   Childs J.E., 2002, Rabies, P114
   Constantine DG, 1988, ECOLOGICAL BEHAV MET

Davis A, 2005, AM J TROP MED HYG, V73, P467, DOI 10.4269/ajtmh.2005.73.467
Davis PL, 2006, INFECT GENET EVOL, V6, P464, DOI 10.1016/j.meegid.2006.02.007
Dean D.J., 1996, Laboratory Techniques in Rabies, V4th, P47
GIRARD KF, 1978, NEW ENGL J MED, V298, P915, DOI 10.1056/NEJM197804202981616
Hughes GJ, 2005, J GEN VIROL, V86, P1467, DOI 10.1099/vir.0.80710-0
Jackson FR, 2008, J WILDLIFE DIS, V44, P612, DOI 10.7589/0090-3558-44.3.612
Nadin-Davis SA, 2010, MOL ECOL, V19, P2120, DOI 10.1111/j.1365-294X.2010.04630.x
Pape WJ, 1999, EMERG INFECT DIS, V5, P433, DOI 10.3201/eid0503.990315
REED L. J., 1938, AMER JOUR HYG, V27, P493
Rohde RE, 2004, EMERG INFECT DIS, V10, P948, DOI 10.3201/eid1005.030719
RUDD RJ, 1989, J CLIN MICROBIOL, V27, P2522, DOI 10.1128/JCM.27.11.2522-2528.1989
RUDD RJ, 1987, J CLIN MICROBIOL, V25, P1456, DOI 10.1128/JCM.25.8.1456-1458.1987
Sarmento L, 2005, J NEUROVIROL, V11, P571, DOI 10.1080/13550280500385310
STEECE R, 1989, J WILDLIFE DIS, V25, P490, DOI 10.7589/0090-3558-25.4.490
Trimarchi CV, 1996, LAB TECHNIQUES RABIE
NR 23
TC 19
Z9 20
U1 1
U2 22
PU MARY ANN LIEBERT, INC
PI NEW ROCHELLE
PA 140 HUGUENOT STREET, 3RD FL, NEW ROCHELLE, NY 10801 USA
SN 1530-3667
EI 1557-7759
J9 VECTOR-BORNE ZOONOT
JI Vector-Borne Zoonotic Dis.
PD JAN
PY 2012
VL 12
IS 1
BP 55
EP 60
DI 10.1089/vbz.2011.0674
PG 6
WC Public, Environmental & Occupational Health; Infectious Diseases
WE Science Citation Index Expanded (SCI-EXPANDED)
SC Public, Environmental & Occupational Health; Infectious Diseases
GA 876MY
UT WOS:000299112800009
PM 21923271
OA Green Published

DA 2024-10-21
ER

PT J
AU Wilkinson, GS
   Adams, DM
   Haghani, A
   Lu, AT
   Zoller, J
   Breeze, CE
   Arnold, BD
   Ball, HC
   Carter, GG
   Cooper, LN
   Dechmann, DKN
   Devanna, P
   Fasel, NJ
   Galazyuk, AV
   Günther, L
   Hurme, E
   Jones, G
   Knörnschild, M
   Lattenkamp, EZ
   Li, CZ
   Mayer, F
   Reinhardt, JA
   Medellin, RA
   Nagy, M
   Pope, B
   Power, ML
   Ransome, RD
   Teeling, EC
   Vernes, SC
   Zamora-Mejías, D
   Zhang, J
   Faure, PA
   Greville, LJ
   Horvath, S
AF Wilkinson, Gerald S.
   Adams, Danielle M.
   Haghani, Amin
   Lu, Ake T.
   Zoller, Joseph
   Breeze, Charles E.
   Arnold, Bryan D.
   Ball, Hope C.
   Carter, Gerald G.
   Cooper, Lisa Noelle
   Dechmann, Dina K. N.
   Devanna, Paolo
   Fasel, Nicolas J.
   Galazyuk, Alexander, V
   Gunther, Linus
   Hurme, Edward

Jones, Gareth
Knornschild, Mirjam
Lattenkamp, Ella Z.
Li, Caesar Z.
Mayer, Frieder
Reinhardt, Josephine A.
Medellin, Rodrigo A.
Nagy, Martina
Pope, Brian
Power, Megan L.
Ransome, Roger D.
Teeling, Emma C.
Vernes, Sonja C.
Zamora-Mejias, Daniel
Zhang, Joshua
Faure, Paul A.
Greville, Lucas J.
Horvath, Steve
TI DNA methylation predicts age and provides insight into exceptional
longevity of bats
SO NATURE COMMUNICATIONS
LA English
DT Article
AB Exceptionally long-lived species, including many bats, rarely show overt signs of aging, making it difficult to determine why species differ in lifespan. Here, we use DNA methylation (DNAm) profiles from 712 known-age bats, representing 26 species, to identify epigenetic changes associated with age and longevity. We demonstrate that DNAm accurately predicts chronological age. Across species, longevity is negatively associated with the rate of DNAm change at age-associated sites. Furthermore, analysis of several bat genomes reveals that hypermethylated age- and longevity-associated sites are disproportionately located in promoter regions of key transcription factors (TF) and enriched for histone and chromatin features associated with transcriptional regulation. Predicted TF binding site motifs and enrichment analyses indicate that age-related methylation change is influenced by developmental processes, while longevity-related DNAm change is associated with innate immunity or tumorigenesis genes, suggesting that bat longevity results from augmented immune response and cancer suppression. DNA methylation profiles from 26 bat species accurately predicts chronological age, while longevity-related methylation patterns across the genome suggest that bat longevity results from augmented immune response and cancer suppression.
C1 [Wilkinson, Gerald S.; Adams, Danielle M.; Hurme, Edward] Univ Maryland, Dept Biol, College Pk, MD 20742 USA.
[Haghani, Amin; Lu, Ake T.; Zhang, Joshua; Horvath, Steve] Univ Calif Los Angeles, David Geffen Sch Med, Dept Human Genet, Los Angeles, CA 90095 USA.
[Zoller, Joseph; Li, Caesar Z.; Horvath, Steve] Univ Calif Los Angeles, Fielding Sch Publ Hlth, Dept Biostat, Los Angeles, CA 90095 USA.
[Breeze, Charles E.] Altius Inst Biomed Sci, Seattle, WA USA.

[Arnold, Bryan D.] Illinois Coll, Dept Biol, Jacksonville, IL USA.
[Ball, Hope C.; Cooper, Lisa Noelle; Galazyuk, Alexander, V] Northeast Ohio Med Univ, Dept Anat & Neurobiol, Rootstown, OH USA.
[Carter, Gerald G.] Ohio State Univ, Dept Evolut Ecol & Organismal Biol, Columbus, OH 43210 USA.
[Dechmann, Dina K. N.] Max Planck Inst Anim Behav, Dept Migrat, Radolfzell am Bodensee, Germany.
[Dechmann, Dina K. N.; Hurme, Edward] Univ Konstanz, Dept Biol, Constance, Germany.
[Dechmann, Dina K. N.; Knornschild, Mirjam] Smithsonian Trop Res Inst, Panama City, FL USA.
[Devanna, Paolo; Lattenkamp, Ella Z.; Vernes, Sonja C.] Max Planck Inst Psycholinguist, Neurogenet Vocal Commun Grp, Nijmegen, Netherlands.
[Fasel, Nicolas J.] Univ Lausanne, Dept Ecol & Evolut, Lausanne, Switzerland.
[Gunther, Linus; Knornschild, Mirjam; Mayer, Frieder; Nagy, Martina] Museum Nat Kunde, Leibniz Inst Evolut & Biodivers Sci, Berlin, Germany.
[Jones, Gareth; Ransome, Roger D.] Univ Bristol, Sch Biol Sci, Bristol, Avon, England.
[Lattenkamp, Ella Z.] Ludwig Maximilians Univ Munchen, Dept Biol 2, Martinsried, Germany.
[Reinhardt, Josephine A.] SUNY Coll Geneseo, Dept Biol, Geneseo, NY 14454 USA.
[Medellin, Rodrigo A.; Zamora-Mejias, Daniel] Univ Nacl Autonoma Mexico, Inst Ecol, Ciudad Univ, Mexico City, DF, Mexico.
[Pope, Brian] Lubee Bat Conservancy, Gainesville, FL USA.
[Power, Megan L.; Teeling, Emma C.] Univ Coll Dublin, Sch Biol & Environm Sci, Dublin 4, Ireland.
[Vernes, Sonja C.] Donders Inst Brain Cognit & Behav, Nijmegen, Netherlands.
[Vernes, Sonja C.] Univ St Andrews, Sch Biol, St Andrews, Fife, Scotland.
[Faure, Paul A.; Greville, Lucas J.] McMaster Univ, Dept Psychol Neurosci & Behav, Hamilton, ON, Canada.
C3 University System of Maryland; University of Maryland College Park;
University of California System; University of California Los Angeles;
University of California Los Angeles Medical Center; David Geffen School
of Medicine at UCLA; University of California System; University of
California Los Angeles; University System of Ohio; Northeast Ohio
Medical University (NEOMED); University System of Ohio; Ohio State
University; Max Planck Society; University of Konstanz; Smithsonian
Institution; Smithsonian Tropical Research Institute; Max Planck
Society; University of Lausanne; Leibniz Institut fur Evolutions und
Biodiversitatsforschung; University of Bristol; University of

Munich;
State University of New York (SUNY) System; SUNY Geneseo; Universidad
Nacional Autonoma de Mexico; University College Dublin; Radboud
University Nijmegen; University of St Andrews; McMaster University
RP Wilkinson, GS (corresponding author), Univ Maryland, Dept Biol,
College Pk, MD 20742 USA.; Horvath, S (corresponding author), Univ
Calif Los Angeles, David Geffen Sch Med, Dept Human Genet, Los
Angeles, CA 90095 USA.; Horvath, S (corresponding author), Univ
Calif Los Angeles, Fielding Sch Publ Hlth, Dept Biostat, Los
Angeles, CA 90095 USA.
EM wilkinso@umd.edu; shorvath@mednet.ucla
RI Nagy, Martina/D-2636-2012; Dechmann, Dina/A-1827-2010; Li,
Caesar/KJD-6542-2024; Knörnschild, Mirjam/C-9401-2011; Zamora-Mejias,
Daniel/GQR-0676-2022; Haghani, Amin/C-5543-2015; Vernes,
Sonja/E-8454-2012; Wilkinson, Gerald/E-4424-2011
OI Lu, Ake/0000-0002-2866-7961; Teeling, Emma/0000-0002-3309-1346; Carter,
Gerald/0000-0001-6933-5501; Haghani, Amin/0000-0002-6052-8793;
Zamora-Mejias, Daniel/0000-0002-8531-2147; Li,
Caesar/0000-0002-3907-7983; Power, Megan/0000-0001-7402-3254; ZHANG,
JOSHUA/0000-0002-4488-1175; Vernes, Sonja/0000-0003-0305-4584; Adams,
Danielle/0000-0002-8260-4064; Jones, Gareth/0000-0002-1904-3735;
Wilkinson, Gerald/0000-0001-7799-8444; Zoller,
Joseph/0000-0001-6309-0291; Arnold, Bryan/0000-0001-5589-8143; Horvath,
Steve/0000-0002-4110-3589
FU Paul G. Allen Frontiers Group grant; University of Maryland, College of
Computer, Mathematical and Natural Sciences; Irish Research Council
Consolidator Laureate Award; UKRI [MR/T021985/1]; Natural Sciences and
Engineering Research Council (NSERC) of Canada; Max Planck Research
Group by the Max Planck Gesellschaft; Human Frontiers Science Program
Grant [RGP0058/2016]; NSERC; FLF [MR/T021985/1] Funding Source: UKRI
FX This work was supported by a Paul G. Allen Frontiers Group grant to
S.H., the University of Maryland, College of Computer, Mathematical and
Natural Sciences to G.S.W., an Irish Research Council Consolidator
Laureate Award to E.C.T., a UKRI Future Leaders Fellowship
(MR/T021985/1) to S.C.V. and a Discovery Grant from the Natural Sciences
and Engineering Research Council (NSERC) of Canada to P.A.F. S.C.V. and

P.D. were supported by a Max Planck Research Group awarded to S.C.V. by
the Max Planck Gesellschaft, and S.C.V. and E.Z.L. were supported by a
Human Frontiers Science Program Grant (RGP0058/2016) awarded to S.C.V.
L.J.G. was supported by an NSERC PGS-D scholarship. We thank the
Neurogenomics Core at UCLA for laboratory assistance, A. Lollar for
providing Tadarida samples, M. Brooks for sharing a new maximum recorded
lifespan for Pteropus giganteus, K. Bennett for graphical assistance,
and to the Banbury Center, Cold Spring Harbor Labs for hosting the
workshop that inspired this collaboration.
CR Ahn M, 2019, NAT MICROBIOL, V4, P789, DOI 10.1038/s41564-019-0371-3
Amador LI, 2018, J MAMM EVOL, V25, P37, DOI 10.1007/s10914-016-9363-8
Arneson A, 2021, MAMMALIAN METHYLATIO, DOI [10.1101/2021.01.07.425637v1, DOI 10.1101/2021.01.07.425637V1]
Arthur RK, 2017, NUCLEIC ACIDS RES, V45, P6350, DOI 10.1093/nar/gkx218
Banerjee A, 2020, FRONT IMMUNOL, V11, DOI 10.3389/fimmu.2020.00026
Benjamini Y, 2001, ANN STAT, V29, P1165
Breeze CE, 2019, BIOINFORMATICS, V35, P4767, DOI 10.1093/bioinformatics/btz456
Chen BH, 2016, AGING-US, V8, P1844, DOI 10.18632/aging.101020
Chen YT, 2016, EPIGENET CHROMATIN, V9, DOI 10.1186/s13072-016-0058-4
Cheney JA, 2017, J ANAT, V230, P510, DOI 10.1111/joa.12580
Choi J, 2020, MOL CELL, V77, P310, DOI 10.1016/j.molcel.2019.10.011
Christensen BC, 2009, PLOS GENET, V5, DOI 10.1371/journal.pgen.1000602
Cole JJ, 2017, GENOME BIOL, V18, DOI 10.1186/s13059-017-1185-3
Day K, 2013, GENOME BIOL, V14, DOI 10.1186/gb-2013-14-9-r102
De Keersmaecker K, 2013, NAT GENET, V45, P186, DOI 10.1038/ng.2508
Dixon JR, 2012, NATURE, V485, P376, DOI 10.1038/nature11082
Engelmann D, 2012, CANCER RES, V72, P571, DOI 10.1158/0008-5472.CAN-11-2575
Faure PA, 2009, J MAMMAL, V90, P1148, DOI 10.1644/08-MAMM-A-332.1
Fornes O, 2020, NUCLEIC ACIDS RES, V48, pD87, DOI 10.1093/nar/gkz1001
Friedman J, 2010, J STAT SOFTW, V33, P1, DOI 10.18637/jss.v033.i01
Gaidatzis D, 2015, BIOINFORMATICS, V31, P1130, DOI 10.1093/bioinformatics/btu781
GARTLER SM, 1983, ANNU REV GENET, V17, P155, DOI 10.1146/annurev.ge.17.120183.001103
Gorbunova V, 2020, CELL METAB, V32, P31, DOI 10.1016/

j.cmet.2020.06.013
Hannum G, 2013, MOL CELL, V49, P359, DOI 10.1016/j.molcel.2012.10.016
Horvath S, 2018, NAT REV GENET, V19, P371, DOI 10.1038/s41576-018-0004-3
Horvath S, 2013, GENOME BIOL, V14, DOI 10.1186/gb-2013-14-10-r115
Huang ZX, 2016, BMC GENOMICS, V17, DOI 10.1186/s12864-016-3227-8
Hume MA, 2015, NUCLEIC ACIDS RES, V43, pD117, DOI 10.1093/nar/gku1045
Jebb D, 2020, NATURE, V583, P578, DOI 10.1038/s41586-020-2486-3
Kane AE, 2019, CRIT REV BIOCHEM MOL, V54, P61, DOI 10.1080/10409238.2019.1570075
Kim W, 2019, NAT COMMUN, V10, DOI 10.1038/s41467-019-13321-z
Klutstein M, 2016, CANCER RES, V76, P3446, DOI 10.1158/0008-5472.CAN-15-3278
Kumar S, 2014, MOL CELL BIOCHEM, V395, P281, DOI 10.1007/s11010-014-2137-9
Lambert SA, 2018, CELL, V172, P650, DOI 10.1016/j.cell.2018.01.029
Li LJ, 2019, CELL STEM CELL, V24, P271, DOI 10.1016/j.stem.2018.12.012
Lowe R, 2018, GENOME BIOL, V19, DOI 10.1186/s13059-018-1397-1
Lu T, 2014, NATURE, V507, P448, DOI 10.1038/nature13163
Maegawa S, 2017, NAT COMMUN, V8, DOI 10.1038/s41467-017-00607-3
Marioni RE, 2015, GENOME BIOL, V16, DOI 10.1186/s13059-015-0584-6
Marttila S, 2015, BMC GENOMICS, V16, DOI 10.1186/s12864-015-1381-z
Matys V, 2006, NUCLEIC ACIDS RES, V34, pD108, DOI 10.1093/nar/gkj143
Maurano MT, 2015, NAT GENET, V47, P1393, DOI 10.1038/ng.3432
Mayne B, 2019, SCI REP-UK, V9, DOI 10.1038/s41598-019-54447-w
McLain AT, 2018, AGING-US, V10, P561, DOI 10.18632/aging.101413
Mi HY, 2019, NAT PROTOC, V14, P703, DOI 10.1038/s41596-019-0128-8
Olds JE, 2015, J ZOO WILDLIFE MED, V46, P325, DOI 10.1638/2014-0069R2.1
Orso F, 2008, FASEB J, V22, P2702, DOI 10.1096/fj.08-106492
Petkovich DA, 2017, CELL METAB, V25, P954, DOI 10.1016/j.cmet.2017.03.016
Polanowski AM, 2014, MOL ECOL RESOUR, V14, P976, DOI 10.1111/1755-0998.12247
Poulin H, 2005, CANCER LETT, V227, P185, DOI 10.1016/j.canlet.2004.12.007
Rakyan VK, 2010, GENOME RES, V20, P434, DOI 10.1101/gr.103101.109
RAZIN A, 1980, SCIENCE, V210, P604, DOI 10.1126/science.6254144
Seim I, 2013, NAT COMMUN, V4, DOI 10.1038/ncomms3212
Sen P, 2016, CELL, V166, P822, DOI 10.1016/j.cell.2016.07.050
Shemer R, 1996, P NATL ACAD SCI USA, V93, P6371, DOI 10.1073/pnas.93.13.6371
Shirai YT, 2019, ONCOGENE, V38, P2580, DOI 10.1038/s41388-018-0603-7
Stouffer S.A., 1949, The American soldier, V1
Stubbs TM, 2017, GENOME BIOL, V18, DOI 10.1186/s13059-017-1203-5
Teschendorff AE, 2010, GENOME RES, V20, P440, DOI 10.1101/gr.103606.109

   Thompson MJ, 2017, AGING-US, V9, P1055, DOI 10.18632/aging.101211
   Unnikrishnan A, 2018, ANN NY ACAD SCI, V1418, P69, DOI 10.1111/nyas.13533
   Wang T, 2020, CELL SYST, V11, P176, DOI 10.1016/j.cels.2020.06.006
   Wang ZJ, 2018, BIOINFORMATICS, V34, P2867, DOI 10.1093/bioinformatics/bty194
   Wilkinson GS, 2019, BIOL LETTERS, V15, DOI 10.1098/rsbl.2018.0860
   Wilsker D, 2002, CELL GROWTH DIFFER, V13, P95
   Wright PGR, 2018, MOL ECOL RESOUR, V18, P1374, DOI 10.1111/1755-0998.12925
   Yin YM, 2017, SCIENCE, V356, DOI 10.1126/science.aaj2239
   Yu GC, 2015, BIOINFORMATICS, V31, P2382, DOI 10.1093/bioinformatics/btv145
   Zhou P, 2014, PLOS ONE, V9, DOI 10.1371/journal.pone.0103875
   Zhou WD, 2018, NUCLEIC ACIDS RES, V46, DOI 10.1093/nar/gky691
NR 70
TC 86
Z9 95
U1 2
U2 32
PU NATURE RESEARCH
PI BERLIN
PA HEIDELBERGER PLATZ 3, BERLIN, 14197, GERMANY
SN 2041-1723
J9 NAT COMMUN
JI Nat. Commun.
PD MAR 12
PY 2021
VL 12
IS 1
AR 1615
DI 10.1038/s41467-021-21900-2
PG 13
WC Multidisciplinary Sciences
WE Science Citation Index Expanded (SCI-EXPANDED)
SC Science & Technology - Other Topics
GA QZ0IT
UT WOS:000630419400006
PM 33712580
OA Green Submitted, gold, Green Published
DA 2024-10-21
ER

PT J
AU Cowled, C
   Stewart, CR
   Likic, VA
   Friedländer, MR
   Tachedjian, M
   Jenkins, KA
   Tizard, ML
   Cottee, P
   Marsh, GA

Zhou, P
Baker, ML
Bean, AG
Wang, LF
AF Cowled, Christopher
Stewart, Cameron R.
Likic, Vladimir A.
Friedlaender, Marc R.
Tachedjian, Mary
Jenkins, Kristie A.
Tizard, Mark L.
Cottee, Pauline
Marsh, Glenn A.
Zhou, Peng
Baker, Michelle L.
Bean, Andrew G.
Wang, Lin-fa
TI Characterisation of novel microRNAs in the Black flying fox (<i>Pteropus
alecto</i>) by deep sequencing
SO BMC GENOMICS
LA English
DT Article
DE Bats; Chiroptera; Pteropus alecto; MicroRNA; Non-coding RNA;
Transcriptome
ID OXIDATIVE-PHOSPHORYLATION; DIFFERENTIAL EXPRESSION; MIRNAS; BATS; GENES;
EVOLUTION; APOPTOSIS; CLUSTER; FLIGHT; MODEL
AB Background: Bats are a major source of new and emerging viral diseases. Despite the fact that bats carry and shed highly pathogenic viruses including Ebola, Nipah and SARS, they rarely display clinical symptoms of infection. Host factors influencing viral replication are poorly understood in bats and are likely to include both pre- and post-transcriptional regulatory mechanisms. MicroRNAs are a major mechanism of post-transcriptional gene regulation, however very little is known about them in bats.

Results: This study describes 399 microRNAs identified by deep sequencing of small RNA isolated from tissues of the Black flying fox, Pteropus alecto, a confirmed natural reservoir of the human pathogens Hendra virus and Australian bat lyssavirus. Of the microRNAs identified, more than 100 are unique amongst vertebrates, including a subset containing mutations in critical seed regions. Clusters of rapidly-evolving microRNAs were identified, as well as microRNAs predicted to target genes involved in antiviral immunity, the DNA damage response, apoptosis and autophagy. Closer inspection of the predicted targets for several highly supported novel miRNA candidates suggests putative roles in host-virus interaction.

Conclusions: MicroRNAs are likely to play major roles in regulating virus-host interaction in bats, via dampening of inflammatory responses (limiting the effects of immunopathology), and directly limiting the extent of viral replication, either through restricting the availability of essential factors or by controlling apoptosis. Characterisation of the bat microRNA repertoire is an essential step towards understanding

transcriptional regulation during viral infection, and will assist in the identification of mechanisms that enable bats to act as natural virus reservoirs. This in turn will facilitate the development of antiviral strategies for use in humans and other species.
C1 [Cowled, Christopher; Stewart, Cameron R.; Tachedjian, Mary; Jenkins, Kristie A.; Tizard, Mark L.; Cottee, Pauline; Marsh, Glenn A.; Zhou, Peng; Baker, Michelle L.; Bean, Andrew G.; Wang, Lin-fa] CSIRO, Australian Anim Hlth Lab, Geelong, Vic 3220, Australia.
   [Likic, Vladimir A.] Bio21 Mol Sci & Biotechnol Inst, Melbourne, Australia.
   [Friedlaender, Marc R.] Ctr Genom Regulat CRG, Barcelona, Spain.
   [Friedlaender, Marc R.] Univ Pompeu Fabra UPF, Barcelona, Spain.
   [Wang, Lin-fa] Duke NUS Grad Med Sch, Program Emerging Infect Dis, Singapore 169857, Singapore.
C3 Commonwealth Scientific & Industrial Research Organisation (CSIRO);
   Barcelona Institute of Science & Technology; Pompeu Fabra University;
   Centre de Regulacio Genomica (CRG); Pompeu Fabra University; National
   University of Singapore
RP Cowled, C (corresponding author), CSIRO, Australian Anim Hlth Lab, 5 Portarlington Rd, Geelong, Vic 3220, Australia.
EM chris.cowled@csiro.au
RI Jenkins, Kristie/H-2786-2013; Bean, Andrew/H-8664-2013; Stewart,
   Cameron/E-6823-2011; Zhou, Peng/I-8578-2015; Tizard, Mark/B-6610-2011;
   Marsh, Glenn Andrew/A-5418-2013; Baker, Michelle/C-9694-2013; Cowled,
   Christopher/H-5308-2013; Tachedjian, Mary/H-4572-2013
OI Tizard, Mark/0000-0001-8421-2497; Wang, Lin-Fa/0000-0003-2752-0535;
   Marsh, Glenn Andrew/0000-0002-3469-1837; Friedlander,
   Marc/0000-0001-6577-4363; Baker, Michelle/0000-0002-7993-9971; Cowled,
   Christopher/0000-0002-1969-9371; Stewart, Cameron/0000-0002-3395-7859;
   Tachedjian, Mary/0000-0002-8936-6564
FU EMBO [ALTF 225-2011]
FX M.R.F. is supported by EMBO Long-Term fellowship ALTF 225-2011. We wish
   to acknowledge Alysha Heimberg for helpful advice regarding the
   identification of novel miRNAs.
CR Altuvia Y, 2005, NUCLEIC ACIDS RES, V33, P2697, DOI 10.1093/nar/gki567
   Aschrafi A, 2012, CELL MOL LIFE SCI, V69, P4017, DOI 10.1007/s00018-012-1064-8
   Bartel DP, 2009, CELL, V136, P215, DOI 10.1016/j.cell.2009.01.002
   Benetatos L, 2013, CELL MOL LIFE SCI, V70, P795, DOI 10.1007/s00018-012-1080-8
   Biggar KK, 2014, GENE, V544, P67, DOI 10.1016/j.gene.2014.04.048
   Brunet-Rossinni AK, 2004, BIOGERONTOLOGY, V5, P211, DOI 10.1023/B:BGEN.0000038022.65024.d8

Calisher CH, 2006, CLIN MICROBIOL REV, V19, P531, DOI 10.1128/CMR.00017-06
Chang SJ, 2011, BMC MED GENOMICS, V4, DOI 10.1186/1755-8794-4-65
Enright Anton J, 2003, Genome Biol, V5, pR1, DOI 10.1186/gb-2003-5-1-r1
Friedländer MR, 2012, NUCLEIC ACIDS RES, V40, P37, DOI 10.1093/nar/gkr688
Fujimoto T, 2009, FEBS LETT, V583, P568, DOI 10.1016/j.febslet.2008.12.063
Griffiths-Jones S, 2004, NUCLEIC ACIDS RES, V32, pD109, DOI 10.1093/nar/gkh023
Griffiths-Jones S, 2006, NUCLEIC ACIDS RES, V34, pD140, DOI 10.1093/nar/gkj112
Gromak N, 2012, BIOCHEM SOC T, V40, P759, DOI 10.1042/BST20120023
Iwanowicz DD, 2013, US GEOLOGICAL SURVEY
Jopling CL, 2005, SCIENCE, V309, P1577, DOI 10.1126/science.1113329
Jovanovic M, 2006, ONCOGENE, V25, P6176, DOI 10.1038/sj.onc.1209912
Kornfeld Samantha F., 2012, Genomics Proteomics & Bioinformatics, V10, P295, DOI 10.1016/j.gpb.2012.09.001
Kunz TH, 2011, ANN NY ACAD SCI, V1223, P1, DOI 10.1111/j.1749-6632.2011.06004.x
Lewis BP, 2003, CELL, V115, P787, DOI 10.1016/S0092-8674(03)01018-3
Li HX, 2013, INT J MOL MED, V32, P219, DOI 10.3892/ijmm.2013.1381
Li N, 2013, NUCLEIC ACIDS RES, V41, P3619, DOI 10.1093/nar/gkt072
Liu YT, 2010, PHYSIOL GENOMICS, V42A, P39, DOI 10.1152/physiolgenomics.00054.2010
Lu T, 2013, CANCER GENE THER, V20, P33, DOI 10.1038/cgt.2012.81
Maistrovski Y, 2012, J COMP PHYSIOL B, V182, P849, DOI 10.1007/s00360-012-0662-y
Melamed Z, 2013, MOL CELL, V50, P869, DOI 10.1016/j.molcel.2013.05.007
Pichiorri F, 2010, CANCER CELL, V18, P367, DOI 10.1016/j.ccr.2010.09.005
Platt RN, 2014, MOL BIOL EVOL, V31, P1536, DOI 10.1093/molbev/msu112
Sharbati S, 2010, BMC GENOMICS, V11, DOI 10.1186/1471-2164-11-275
Shaw TI, 2012, PLOS ONE, V7, DOI 10.1371/journal.pone.0048472
Shen YY, 2010, P NATL ACAD SCI USA, V107, P8666, DOI 10.1073/pnas.0912613107
Streicher KL, 2012, ONCOGENE, V31, P1558, DOI 10.1038/onc.2011.345
Tamura K, 2011, MOL BIOL EVOL, V28, P2731, DOI 10.1093/molbev/msr121
Teeling EC, 2005, SCIENCE, V307, P580, DOI 10.1126/science.1105113
Thomas MP, 2013, IMMUNOL REV, V253, P237, DOI 10.1111/imr.12052
Triboulet R, 2009, RNA, V15, P1005, DOI 10.1261/rna.1591709
Vaquerizas JM, 2009, NAT REV GENET, V10, P252, DOI 10.1038/nrg2538
White NMA, 2011, BRIT J CANCER, V105, P1741, DOI 10.1038/bjc.2011.401

Yu J, 2006, BIOCHEM BIOPH RES CO, V349, P59, DOI 10.1016/
j.bbrc.2006.07.207
Zhang GJ, 2013, SCIENCE, V339, P456, DOI 10.1126/science.1230835
Zhang J, 2011, CELL BIOCHEM FUNCT, V29, P279, DOI 10.1002/
cbf.1747
Zhang R, 2007, GENOME RES, V17, P612, DOI 10.1101/gr.6146507
Zhou M, 2009, GENOMICS, V94, P125, DOI 10.1016/
j.ygeno.2009.04.006
NR 43
TC 22
Z9 24
U1 0
U2 25
PU BMC
PI LONDON
PA CAMPUS, 4 CRINAN ST, LONDON N1 9XW, ENGLAND
SN 1471-2164
J9 BMC GENOMICS
JI BMC Genomics
PD AUG 15
PY 2014
VL 15
AR 682
DI 10.1186/1471-2164-15-682
PG 13
WC Biotechnology & Applied Microbiology; Genetics & Heredity
WE Science Citation Index Expanded (SCI-EXPANDED)
SC Biotechnology & Applied Microbiology; Genetics & Heredity
GA AO0XP
UT WOS:000341036500001
PM 25128405
OA gold, Green Published
DA 2024-10-21
ER

PT J
AU Zhang, Q
Zeng, LP
Zhou, P
Irving, AT
Li, S
Shi, ZL
Wang, LF
AF Zhang, Qian
Zeng, Lei-Ping
Zhou, Peng
Irving, Aaron T.
Li, Shang
Shi, Zheng-Li
Wang, Lin-Fa
TI <i>IFNAR2</i>-dependent gene expression profile induced by IFN-α
in
<i>Pteropus alecto</i> bat cells and impact of <i>IFNAR2</i>
knockout on

virus infection
SO PLOS ONE
LA English
DT Article
ID HUMAN MACROPHAGES; INTERFERON; RESPONSES; RECEPTOR; RESERVOIRS; MUTANT;
ROLES
AB Bats are important reservoirs of many viruses, which are capable of infecting the host without inducing obvious clinical diseases. Interferon and the downstream interferon regulated genes (IRGs) are known to act as the first line of defense against viral infections. Little is known about the transcriptional profile of genes being induced by interferon in bats and their role in controlling virus infection. In this study, we constructed IFNAR2 knockout bat cell lines using CRISPR technology and further characterized gene expression profiles induced by the most abundant IFN-alpha (IFN-alpha 3). Firstly, we demonstrated that the CRISPR/Cas9 system is applicable for bat cells as this represents the first CRIPSR knockout cell line for bats. Our results showed the pleiotropic effect of IFN-a3 on the bat kidney cell line, PaKiT03. As expected, we confirmed that IFNAR2 is indispensable for IFN-a signaling pathway and plays an important role in antiviral immunity. Unexpectedly, we also identified novel IFNAR2-dependent IRGs which are enriched in pathways related to cancer. To our knowledge, this seems to be bat-specific as no such observation has been reported for other mammalian species. This study expands our knowledge about bat immunology and the cell line established can provide a powerful tool for future study into virus-bat interaction and cancer biology.
C1 [Zhang, Qian; Zeng, Lei-Ping; Zhou, Peng; Shi, Zheng-Li] Chinese Acad Sci, Wuhan Inst Virol, Key Lab Special Pathogens, Wuhan, Hubei, Peoples R China.
[Irving, Aaron T.; Wang, Lin-Fa] Duke Natl Univ Singapore Med Sch, Programme Emerging Infect Dis, Singapore, Singapore.
[Li, Shang] Duke Natl Univ Singapore Med Sch, Programme Canc & Stem Cell Biol, Singapore, Singapore.
C3 Chinese Academy of Sciences; Wuhan Institute of Virology, CAS; National
University of Singapore; National University of Singapore
RP Shi, ZL (corresponding author), Chinese Acad Sci, Wuhan Inst Virol, Key Lab Special Pathogens, Wuhan, Hubei, Peoples R China.
EM zlshi@wh.iov.cn
RI Zeng, Leiping/HMO-8157-2023; Irving, Aaron/AAE-8874-2021; Irving, Aaron/I-6754-2013; Shi, Zheng-Li/A-1013-2013
OI Wang, Lin-Fa/0000-0003-2752-0535; Irving, Aaron/0000-0002-0196-1570;
Zeng, Leiping/0000-0002-6421-0950; Shi, Zheng-Li/0000-0001-8089-163X
FU National Natural Science Foundation of China [31621061]; National Research Foundation-Competitive Research Program (NRF-CRP) from National
Research Foundation in Singapore [NRF2012NRF-CRP001-056)]
FX This study was supported in part by National Natural Science Foundation
of China (31621061) (to Z-LS) and the National Research

Foundation-Competitive Research Program (NRF-CRP) grant
(NRF2012NRF-CRP001-056) from the National Research Foundation in
Singapore (to L-FW). The funders had no role in study design, data
collection and analysis, decision to publish, or preparation of the
manuscript. We acknowledge and thank Muhammad Khairul RAMLEE and Jing
WANG for technical help with CRISPR work. This study was supported in
part by National Natural Science Foundation of China (31621061) (to
Z-LS) and the NRF-CRP grant (NRF2012NRF-CRP001-056) from the National
Research Foundation in Singapore (to L-FW).
CR Ahn M, 2016, SCI REP-UK, V6, DOI 10.1038/srep21722
Anderson AC, 2006, CURR OPIN IMMUNOL, V18, P665, DOI 10.1016/j.coi.2006.09.009
Brook CE, 2015, TRENDS MICROBIOL, V23, P172, DOI 10.1016/j.tim.2014.12.004
Calisher CH, 2006, CLIN MICROBIOL REV, V19, P531, DOI 10.1128/CMR.00017-06
Conlan LA, 2004, ONCOGENE, V23, P307, DOI 10.1038/sj.onc.1207119
Cowled C, 2012, DEV COMP IMMUNOL, V36, P657, DOI 10.1016/j.dci.2011.11.008
Crameri G, 2009, PLOS ONE, V4, DOI 10.1371/journal.pone.0008266
Curtiss ML, 2012, EUR J IMMUNOL, V42, P651, DOI 10.1002/eji.201141581
Dai SY, 2014, CELL IMMUNOL, V290, P72, DOI 10.1016/j.cellimm.2014.05.006
de Weerd NA, 2013, NAT IMMUNOL, V14, P901, DOI 10.1038/ni.2667
Dölken L, 2008, RNA, V14, P1959, DOI 10.1261/rna.1136108
François-Newton V, 2011, PLOS ONE, V6, DOI 10.1371/journal.pone.0022200
Huang W, 2011, BMC BIOINFORMATICS, V12, DOI 10.1186/1471-2105-12-1
Indraccolo S, 2007, J IMMUNOL, V178, P1122, DOI 10.4049/jimmunol.178.2.1122
Jaitin DA, 2006, MOL CELL BIOL, V26, P1888, DOI 10.1128/MCB.26.5.1888-1897.2006
Koyama S, 2007, J IMMUNOL, V179, P4711, DOI 10.4049/jimmunol.179.7.4711
Krebs DL, 2000, J CELL SCI, V113, P2813
Lamken P, 2004, J MOL BIOL, V341, P303, DOI 10.1016/j.jmb.2004.05.059
Leng N, 2013, BIOINFORMATICS, V29, P1035, DOI 10.1093/bioinformatics/btt087
Levin D, 2014, SCI SIGNAL, V7, DOI 10.1126/scisignal.2004998
Lieber MR, 2010, ANNU REV BIOCHEM, V79, P181, DOI 10.1146/annurev.biochem.052308.093131
Livak KJ, 2001, METHODS, V25, P402, DOI 10.1006/meth.2001.1262
Luheshi N, 2014, EUR J IMMUNOL, V44, P162, DOI 10.1002/eji.201343351
Minn AJ, 2015, TRENDS IMMUNOL, V36, P725, DOI 10.1016/

j.it.2015.09.007
Ni W, 2014, PLOS ONE, V9, DOI 10.1371/journal.pone.0106718
Oeckinghaus A, 2011, NAT IMMUNOL, V12, P695, DOI 10.1038/ni.2065
Österlund P, 2010, J VIROL, V84, P1414, DOI 10.1128/JVI.01619-09
Ramlee MK, 2015, SCI REP-UK, V5, DOI 10.1038/srep15587
Ran FA, 2013, NAT PROTOC, V8, P2281, DOI 10.1038/nprot.2013.143
Robinson MD, 2010, BIOINFORMATICS, V26, P139, DOI 10.1093/bioinformatics/btp616
Romagnani S, 1997, IMMUNOL TODAY, V18, P263, DOI 10.1016/S0167-5699(97)80019-9
Sakai R, 2014, INT J MOL SCI, V15, P17256, DOI 10.3390/ijms151017256
Samarajiwa SA, 2009, NUCLEIC ACIDS RES, V37, pD852, DOI 10.1093/nar/gkn732
Sanda C, 2006, J INTERF CYTOK RES, V26, P462, DOI 10.1089/jir.2006.26.462
Schoggins JW, 2014, CURR OPIN VIROL, V6, P40, DOI 10.1016/j.coviro.2014.03.006
Schreiber G, 2015, TRENDS IMMUNOL, V36, P139, DOI 10.1016/j.it.2015.01.002
Smith I, 2013, CURR OPIN VIROL, V3, P84, DOI 10.1016/j.coviro.2012.11.006
Vinay DS, 2011, CLIN EXP IMMUNOL, V164, P145, DOI 10.1111/j.1365-2249.2011.04375.x
Wang LF, 2011, CURR OPIN VIROL, V1, P649, DOI 10.1016/j.coviro.2011.10.013
Wang RH, 2008, CANCER CELL, V14, P312, DOI 10.1016/j.ccr.2008.09.001
Wang XL, 2016, SCI REP-UK, V6, DOI 10.1038/srep20620
Welsby I, 2012, BIOCHEM PHARMACOL, V84, P11, DOI 10.1016/j.bcp.2012.02.016
Yang XO, 2009, NAT IMMUNOL, V10, P1260, DOI 10.1038/ni.1821
Zhang GJ, 2013, SCIENCE, V339, P456, DOI 10.1126/science.1230835
Zhou P, 2016, P NATL ACAD SCI USA, V113, P2696, DOI 10.1073/pnas.1518240113
Zhou P, 2013, DEV COMP IMMUNOL, V40, P240, DOI 10.1016/j.dci.2013.03.006
NR 46
TC 26
Z9 29
U1 0
U2 13
PU PUBLIC LIBRARY SCIENCE
PI SAN FRANCISCO
PA 1160 BATTERY STREET, STE 100, SAN FRANCISCO, CA 94111 USA
SN 1932-6203
J9 PLOS ONE
JI PLoS One
PD AUG 9
PY 2017
VL 12
IS 8
AR e0182866
DI 10.1371/journal.pone.0182866

PG 13
WC Multidisciplinary Sciences
WE Science Citation Index Expanded (SCI-EXPANDED)
SC Science & Technology - Other Topics
GA FC9YJ
UT WOS:000407196700075
PM 28793350
OA Green Submitted, gold, Green Published
DA 2024-10-21
ER

PT J
AU Pulscher, LA
   Peel, AJ
   Rose, K
   Welbergen, JA
   Baker, ML
   Boyd, V
   Low-Choy, S
   Edson, D
   Todd, C
   Dorrestein, A
   Hall, J
   Todd, S
   Broder, CC
   Yan, LY
   Xu, K
   Peck, GR
   Phalen, DN
AF Pulscher, Laura A.
   Peel, Alison J.
   Rose, Karrie
   Welbergen, Justin A.
   Baker, Michelle L.
   Boyd, Victoria
   Low-Choy, Samantha
   Edson, Dan
   Todd, Christopher
   Dorrestein, Annabel
   Hall, Jane
   Todd, Shawn
   Broder, Christopher C.
   Yan, Lianying
   Xu, Kai
   Peck, Grantley R.
   Phalen, David N.
TI Serological evidence of a pararubulavirus and a betacoronavirus in the
   geographically isolated Christmas Island flying-fox (<i>Pteropus
   natalis</i>)
SO TRANSBOUNDARY AND EMERGING DISEASES
LA English
DT Article
DE bat; betacoronavirus; insular populations; pararubulavirus;

Pteropodidae; viral maintenance
ID HENDRA VIRUS; BATS; HENIPAVIRUS; CORONAVIRUS; ASSAYS; INFECTIONS; DIVERSITY; PATHOGENS; EXPOSURE
AB Due to their geographical isolation and small populations, insular bats may not be able to maintain acute immunizing viruses that rely on a large population for viral maintenance. Instead, endemic transmission may rely on viruses establishing persistent infections within hosts or inducing only short-lived neutralizing immunity. Therefore, studies on insular populations are valuable for developing broader understanding of viral maintenance in bats. The Christmas Island flying-fox (CIFF; Pteropus natalis) is endemic on Christmas Island, a remote Australian territory, and is an ideal model species to understand viral maintenance in small, geographically isolated bat populations. Serum or plasma (n = 190), oral swabs (n = 199), faeces (n = 31), urine (n = 32) and urine swabs (n = 25) were collected from 228 CIFFs. Samples were tested using multiplex serological and molecular assays, and attempts at virus isolation to determine the presence of paramyxoviruses, betacoronaviruses and Australian bat lyssavirus. Analysis of serological data provides evidence that the species is maintaining a pararubulavirus and a betacoronavirus. There was little serological evidence supporting the presence of active circulation of the other viruses assessed in the present study. No viral nucleic acid was detected and no viruses were isolated. Age-seropositivity results support the hypothesis that geographically isolated bat populations can maintain some paramyxoviruses and coronaviruses. Further studies are required to elucidate infection dynamics and characterize viruses in the CIFF. Lastly, apparent absence of some pathogens could have implications for the conservation of the CIFF if a novel disease were introduced into the population through human carriage or an invasive species. Adopting increased biosecurity protocols for ships porting on Christmas Island and for researchers and bat carers working with flying-foxes are recommended to decrease the risk of pathogen introduction and contribute to the health and conservation of the species.
C1 [Pulscher, Laura A.; Phalen, David N.] Univ Sydney, Fac Sci, Sydney Sch Vet Sci, Sydney, NSW, Australia.
[Peel, Alison J.; Low-Choy, Samantha] Griffith Univ, Ctr Planetary Hlth & Food Secur, Nathan, Qld, Australia.
[Rose, Karrie; Hall, Jane] Taronga Conservat Soc Australia, Australian Registry Wildlife Hlth, Mosman, NSW, Australia.
[Welbergen, Justin A.; Todd, Christopher; Dorrestein, Annabel] Univ Western Sydney, Hawkesbury Inst Environm, Richmond, NSW, Australia.
[Baker, Michelle L.; Boyd, Victoria; Todd, Shawn; Peck, Grantley R.] Commonwealth Sci & Ind Res Org, Australian Ctr Dis Preparedness, Hlth & Biosecur Business Unit, Geelong, Vic, Australia.
[Low-Choy, Samantha] Griffith Univ, Off Vice Chancellor, Arts Educ Law, Brisbane, Qld, Australia.
[Edson, Dan] Dept Agr Water & Environm, Canberra, ACT, Australia.
[Broder, Christopher C.; Yan, Lianying] Uniformed Serv Univ Hlth Sci, Dept Microbiol, Bethesda, MD USA.
[Yan, Lianying] Henry M Jackson Fdn Advancement Mil Med, Bethesda, MD USA.

[Xu, Kai] Ohio State Univ, Coll Vet Med, Dept Vet Biosci,
Columbus, OH USA.
C3 University of Sydney; Griffith University; Taronga Conservation
Society
Australia; Western Sydney University; Commonwealth Scientific &
Industrial Research Organisation (CSIRO); Australian Centre for
Disease
Preparedness; Griffith University; Uniformed Services University
of the
Health Sciences - USA; Henry M. Jackson Foundation for the
Advancement
of Military Medicine, Inc; University System of Ohio; Ohio State
University
RP Pulscher, LA (corresponding author), Univ Sydney, Fac Sci, Sydney
Sch Vet Sci, Sydney, NSW, Australia.
EM Laura.pulscher@sydney.edu.au
RI Broder, Christopher/E-1169-2013; Choy, Samantha/A-7161-2012;
Welbergen,
Justin/AGN-0091-2022; Peck, Grantley/H-2318-2013; Dorrestein,
Annabel/JFB-3285-2023; Todd, Shawn/JJE-5658-2023; Hall,
Jane/ABC-6552-2021; Baker, Michelle/AAX-9171-2020; Peel,
Alison/I-3202-2012
OI Rose, Karrie/0000-0001-9828-538X; Todd, Shawn/
0009-0009-2218-7842; Peel,
Alison/0000-0003-3538-3550; xu, kai/0000-0001-5412-6942;
Welbergen,
Justin/0000-0002-8085-5759; Low Choy, Samantha/
0000-0002-1722-4428
FU Department of Agriculture, Water and the Environment, Australian
Government; Taronga Conservation Society Australia's Taronga
Foundation;
Marie Bashir Institute, University of Sydney; Christmas Island
National
Park; Sydney School of Veterinary Science at the University of
Sydney;
National Institutes of Health [AI142764]; ARC DECRA fellowship
[DE190100710]; Australian Research Council [DE190100710] Funding
Source:
Australian Research Council
FX Department of Agriculture, Water and the Environment, Australian
Government; Taronga Conservation Society Australia's Taronga
Foundation;
Marie Bashir Institute, University of Sydney; Christmas Island
National
Park; Sydney School of Veterinary Science at the University of
Sydney;
National Institutes of Health, Grant/Award Number: AI142764; ARC
DECRA
fellowship, Grant/Award Number: DE190100710
CR [Anonymous], 2007, CHRISTMAS ISLAND FLY
Arguin PM, 2002, EMERG INFECT DIS, V8, P258, DOI 10.3201/
eid0803.010330
Barr JA, 2012, J GEN VIROL, V93, P2590, DOI 10.1099/
vir.0.045385-0

Boardman WSJ, 2021, TRANSBOUND EMERG DIS, V68, P2628, DOI 10.1111/tbed.13908
Boardman WSJ, 2020, PLOS ONE, V15, DOI 10.1371/journal.pone.0232339
Bossart KN, 2007, J VIROL METHODS, V142, P29, DOI 10.1016/j.jviromet.2007.01.003
Boyd V, 2015, J VIROL METHODS, V223, P5, DOI 10.1016/j.jviromet.2015.07.004
Breed AC, 2010, EMERG INFECT DIS, V16, P1997, DOI 10.3201/eid1612.100879
Brook CE, 2015, TRENDS MICROBIOL, V23, P172, DOI 10.1016/j.tim.2014.12.004
Burroughs AL, 2016, PLOS ONE, V11, DOI 10.1371/journal.pone.0155252
Calisher CH, 2006, CLIN MICROBIOL REV, V19, P531, DOI 10.1128/CMR.00017-06
Da Silva SC, 2021, VIRUSES-BASEL, V13, DOI 10.3390/v13030517
Chowdhury S, 2014, PLOS NEGLECT TROP D, V8, DOI 10.1371/journal.pntd.0003302
Crameri G, 2009, PLOS ONE, V4, DOI 10.1371/journal.pone.0008266
De Nys HM, 2018, EMERG INFECT DIS, V24, P2228, DOI 10.3201/eid2412.180740
Dominguez SR, 2007, EMERG INFECT DIS, V13, P1295, DOI 10.3201/eid1309.070491
Dovih P, 2019, PLOS NEGLECT TROP D, V13, DOI 10.1371/journal.pntd.0007733
Drexler JF, 2014, ANTIVIR RES, V101, P45, DOI 10.1016/j.antiviral.2013.10.013
Eddelbuettel D, 2018, AM STAT, V72, P28, DOI 10.1080/00031305.2017.1375990
Edson D, 2019, EPIDEMIOL INFECT, V147, P1, DOI [10.1017/S0950268819001237, 10.1017/s0950268819001237]
Epstein JH, 2013, PLOS ONE, V8, DOI 10.1371/journal.pone.0067584
Feldman KS, 2009, J VIROL METHODS, V161, P52, DOI 10.1016/j.jviromet.2009.05.014
Field HE, 2018, ZOONOSES PUBLIC HLTH, V65, P742, DOI 10.1111/zph.12480
Foord AJ, 2013, BIOMED RES INT, V2013, DOI 10.1155/2013/289295
Glennon EE, 2019, PHILOS T R SOC B, V374, DOI 10.1098/rstb.2019.0021
Hall J, 2014, J WILDLIFE DIS, V50, P447, DOI 10.7589/2013-09-245
IUCN Bat Specialist Group , 2020, IUCN SSC BAT SPEC GR
Jeong J, 2017, EPIDEMIOL INFECT, V145, P2053, DOI [10.1017/S0950268817000991, 10.1017/s0950268817000991]
Joffrin L, 2020, SCI REP-UK, V10, DOI 10.1038/s41598-020-63799-7
Jonsson NN, 2004, VET REC, V154, P664, DOI 10.1136/vr.154.21.664
Laing E.D., 2021, MEDRXIV
Laing ED, 2019, P NATL ACAD SCI USA, V116, P20707, DOI 10.1073/pnas.1911773116
Laing ED, 2018, EMERG INFECT DIS, V24, P122, DOI 10.3201/eid2401.170401
Low-Choy S., 2021, SHIFTED GOMPER UNPUB
Lumsden L., 2017, PIPISTRELLUS MURRAYI, DOI 10.2305/IUCN.UK.2017-2.RLTS.T136769A518894.en

Mélade J, 2016, PLOS ONE, V11, DOI 10.1371/journal.pone.0160553
Moro D, 2003, J WILDLIFE DIS, V39, P762, DOI 10.7589/0090-3558-39.4.762
O'Shea TJ, 2016, MAMMAL REV, V46, P175, DOI 10.1111/mam.12064
Peel AJ, 2019, AUST J ZOOL, V67, P346, DOI 10.1071/ZO20046
Peel AJ, 2013, J VIROL METHODS, V193, P295, DOI 10.1016/j.jviromet.2013.06.030
Peel AJ, 2012, PLOS ONE, V7, DOI 10.1371/journal.pone.0030346
Petraityte R, 2009, VIRUS RES, V146, P141, DOI 10.1016/j.virusres.2009.09.010
Phalen DN, 2017, J MAMMAL, V98, P428, DOI 10.1093/jmammal/gyx001
Plowright RK, 2016, PLOS NEGLECT TROP D, V10, DOI 10.1371/journal.pntd.0004796
Plowright RK, 2011, P ROY SOC B-BIOL SCI, V278, P3703, DOI 10.1098/rspb.2011.0522
Plummer M., 2006, R News, V6, P7
Poon Leo L. M., 2008, V454, P13, DOI 10.1007/978-1-59745-181-9_2
Prada D, 2019, VIRUSES-BASEL, V11, DOI 10.3390/v11121157
Prada D, 2019, TROP MED INFECT DIS, V4, DOI 10.3390/tropicalmed4010046
Rahmadane I, 2017, PLOS NEGLECT TROP D, V11, DOI 10.1371/journal.pntd.0006079
Schulz JE, 2020, J INFECT DIS, V221, pS375, DOI 10.1093/infdis/jiz648
Smith CS, 2016, ECOHEALTH, V13, P72, DOI 10.1007/s10393-016-1116-x
Sohayati A R, 2011, Epidemiol Infect, V139, P1570, DOI 10.1017/S0950268811000550
Tidemann C. R., 1985, A study of the status, habitat requirements and management of the two species of bats on Christmas Island (Indian Ocean)
Todd C.M., 2020, THESIS W SYDNEY U
Todd CM, 2018, J MAMMAL, V99, P1510, DOI 10.1093/jmammal/gyy110
Tsang SM, 2021, VIRUSES-BASEL, V13, DOI 10.3390/v13040563
Turmelle AS, 2009, ECOHEALTH, V6, P522, DOI 10.1007/s10393-009-0263-8
Vidgen ME, 2015, J GEN VIROL, V96, P1619, DOI 10.1099/vir.0.000099
Vijgen Leen, 2008, V454, P3, DOI 10.1007/978-1-59745-181-9_1
Wang HH, 2013, PLOS ONE, V8, DOI 10.1371/journal.pone.0080430
Woinarski J, 2018, BATS END CHRISTMAS I
Yan L., 2021, METHODS MOL BIOL
Yu M, 2008, J IMMUNOL METHODS, V331, P1, DOI 10.1016/j.jim.2007.11.009
NR 64
TC 1
Z9 1
U1 1
U2 5
PU WILEY-HINDAWI
PI LONDON
PA ADAM HOUSE, 3RD FL, 1 FITZROY SQ, LONDON, WIT 5HE, ENGLAND
SN 1865-1674
EI 1865-1682

J9 TRANSBOUND EMERG DIS
JI Transbound. Emerg. Dis.
PD SEP
PY 2022
VL 69
IS 5
BP E2366
EP E2377
DI 10.1111/tbed.14579
EA MAY 2022
PG 12
WC Infectious Diseases; Veterinary Sciences
WE Science Citation Index Expanded (SCI-EXPANDED)
SC Infectious Diseases; Veterinary Sciences
GA 4V0LT
UT WOS:000793405300001
PM 35491954
OA Green Published, gold
DA 2024-10-21
ER

PT J
AU Edwards, CE
   Yount, BL
   Graham, RL
   Leist, SR
   Hou, YXJ
   Dinnon, KH
   Sims, AC
   Swanstrom, J
   Gully, K
   Scobey, TD
   Cooley, MR
   Currie, CG
   Randell, SH
   Baric, RS
AF Edwards, Caitlin E.
   Yount, Boyd L.
   Graham, Rachel L.
   Leist, Sarah R.
   Hou, Yixuan J.
   Dinnon, Kenneth H., III
   Sims, Amy C.
   Swanstrom, Jesica
   Gully, Kendra
   Scobey, Trevor D.
   Cooley, Michelle R.
   Currie, Caroline G.
   Randell, Scott H.
   Baric, Ralph S.
TI Swine acute diarrhea syndrome coronavirus replication in primary human
   cells reveals potential susceptibility to infection
SO PROCEEDINGS OF THE NATIONAL ACADEMY OF SCIENCES OF THE UNITED

STATES OF
AMERICA
LA English
DT Article
DE SADS; coronavirus; One Health; emerging infectious disease
ID PORCINE EPIDEMIC DIARRHEA; TRANSMISSIBLE GASTROENTERITIS VIRUS; MOUSE
HEPATITIS-VIRUS; FUNCTIONAL RECEPTOR; KARYOTYPE VARIATION; REVERSE
GENETICS; BAT CORONAVIRUS; SPIKE PROTEIN; EVOLUTION; ENTRY
AB Zoonotic coronaviruses represent an ongoing threat, yet the myriads of circulating animal viruses complicate the identification of higherrisk isolates that threaten human health. Swine acute diarrhea syndrome coronavirus (SADS-CoV) is a newly discovered, highly pathogenic virus that likely evolved from closely related HKU2 bat coronaviruses, circulating in Rhinolophus spp. bats in China and elsewhere. As coronaviruses cause severe economic losses in the pork industry and swine are key intermediate hosts of human disease outbreaks, we synthetically resurrected a recombinant virus (rSADS-CoV) as well as a derivative encoding tomato red fluorescent protein (tRFP) in place of ORF3. rSADS-CoV replicated efficiently in a variety of continuous animal and primate cell lines, including human liver and rectal carcinoma cell lines. Of concern, rSADS-CoV also replicated efficiently in several different primary human lung cell types, as well as primary human intestinal cells. rSADS-CoV did not use human coronavirus ACE-2, DPP4, or CD13 receptors for docking and entry. Contemporary human donor sera neutralized the group I human coronavirus NL63, but not rSADS-CoV, suggesting limited human group I coronavirus cross protective herd immunity. Importantly, remdesivir, a broad-spectrum nucleoside analog that is effective against other group 1 and 2 coronaviruses, efficiently blocked rSADS-CoV replication in vitro. rSADS-CoV demonstrated little, if any, replicative capacity in either immune-competent or immunodeficient mice, indicating a critical need for improved animal models. Efficient growth in primary human lung and intestinal cells implicate SADS-CoV as a potential higher-risk emerging coronavirus pathogen that could negatively impact the global economy and human health.
C1 [Edwards, Caitlin E.; Yount, Boyd L.; Graham, Rachel L.; Leist, Sarah R.; Hou, Yixuan J.; Swanstrom, Jesica; Scobey, Trevor D.; Cooley, Michelle R.; Currie, Caroline G.; Baric, Ralph S.] Univ N Carolina, Dept Epidemiol, Chapel Hill, NC 27599 USA.
[Dinnon, Kenneth H., III; Baric, Ralph S.] Univ N Carolina, Dept Microbiol & Immunol, Chapel Hill, NC 27599 USA.
[Sims, Amy C.] Pacific Northwest Natl Lab, Chem & Biol Signatures Div, Richland, WA 99354 USA.
[Gully, Kendra] Univ N Carolina, Dept Comparat Med, Chapel Hill, NC 27599 USA.
[Randell, Scott H.] Univ N Carolina, Marsico Lung Inst, Chapel Hill, NC 27599 USA.
[Baric, Ralph S.] Univ N Carolina, Rapidly Emerging Antiviral Drug Discovery Initiat, Chapel Hill, NC 27599 USA.
C3 University of North Carolina; University of North Carolina Chapel Hill;

University of North Carolina; University of North Carolina Chapel Hill;
United States Department of Energy (DOE); Pacific Northwest National
Laboratory; University of North Carolina; University of North Carolina
Chapel Hill; University of North Carolina; University of North Carolina
Chapel Hill; University of North Carolina; University of North Carolina
Chapel Hill
RP Baric, RS (corresponding author), Univ N Carolina, Dept Epidemiol, Chapel Hill, NC 27599 USA.; Baric, RS (corresponding author), Univ N Carolina, Dept Microbiol & Immunol, Chapel Hill, NC 27599 USA.; Baric, RS (corresponding author), Univ N Carolina, Rapidly Emerging Antiviral Drug Discovery Initiat, Chapel Hill, NC 27599 USA.
EM rbaric@email.unc.edu
RI Sims, Amy/HGD-6501-2022; Edwards, Caitlin/KGL-7135-2024; Dinnon, Kenneth/JPK-3608-2023; Graham, Rachel/AAM-9048-2020; Baric, Ralph/AFE-6797-2022; Hou, Yixuan/P-6443-2019; Rebecca, Leist/AGR-2435-2022
OI Edwards, Caitlin/0000-0003-4228-2192; Hou, Yixuan/0000-0002-8323-7243;
Leist, Sarah Rebecca/0000-0002-4989-5381; Graham, Rachel/0000-0002-3143-6515; Gully, Kendra/0000-0001-5742-2341
FU Cystic Fibrosis Foundation [BOUCHE19R0]; NIH [DK065988, AI089728, AI142759, AI132178, AI151797]; North Carolina Policy Collaboratory at the University of North Carolina at Chapel Hill; North Carolina Coronavirus Relief Fund
FX Provision of primary human lung cells by Dr. Randell was supported by grants from the Cystic Fibrosis Foundation (BOUCHE19R0) and NIH (DK065988). We thank Dr. Camille Ehre for providing the primary human intestinal cells for this study. This project was supported by the North Carolina Policy Collaboratory at the University of North Carolina at Chapel Hill with funding from the North Carolina Coronavirus Relief Fund established and appropriated by the North Carolina General Assembly. This work was also supported by NIH Grants AI089728, AI142759, AI132178, and AI151797 (to R.S.B.). The content is solely the responsibility of the authors and does not necessarily represent the official views of the NIH.
CR Agnihothram S, 2018, J VIROL, V92, DOI [10.1128/JVI.00027-18, 10.1128/jvi.00027-18]
Agnihothram S, 2014, MBIO, V5, DOI 10.1128/mBio.00047-14

Agostini ML, 2018, MBIO, V9, DOI 10.1128/mBio.00221-18
Aman J, 2016, AM J PHYSIOL-LUNG C, V311, pL453, DOI 10.1152/ajplung.00393.2015
Amuguni H, 2019, ONE HEALTH-AMSTERDAM, V7, DOI 10.1016/j.onehlt.2018.08.002
Baik JY, 2017, BIOTECHNOL BIOENG, V114, P1045, DOI 10.1002/bit.26231
Baudon E, 2018, EMERG MICROBES INFEC, V7, DOI 10.1038/s41426-018-0109-y
Baum SE, 2017, ONE HEALTH-AMSTERDAM, V3, P5, DOI 10.1016/j.onehlt.2016.10.004
Beall A, 2016, MBIO, V7, DOI 10.1128/mBio.01451-15
Bonavia A, 2003, J VIROL, V77, P2530, DOI 10.1128/JVI.77.4.2530-2538.2003
Brown AJ, 2019, ANTIVIR RES, V169, DOI 10.1016/j.antiviral.2019.104541
Carrasco-Hernandez R, 2017, ILAR J, V58, P343, DOI 10.1093/ilar/ilx026
Corman VM, 2018, ADV VIRUS RES, V100, P163, DOI 10.1016/bs.aivir.2018.01.001
Corman VM, 2016, P NATL ACAD SCI USA, V113, P9864, DOI 10.1073/pnas.1604472113
Corman VM, 2015, J VIROL, V89, P11858, DOI 10.1128/JVI.01755-15
Cotten M, 2014, MBIO, V5, DOI 10.1128/mBio.01062-13
Debbink Kari, 2013, Curr Protoc Microbiol, V31, DOI 10.1002/9780471729259.mc15k01s31
Donaldson EF, 2008, J VIROL, V82, P11948, DOI 10.1128/JVI.01804-08
Drexler JF, 2010, J VIROL, V84, P11336, DOI 10.1128/JVI.00650-10
Du LY, 2009, NAT REV MICROBIOL, V7, P226, DOI 10.1038/nrmicro2090
Edwards C. E., 2020, MUTANT SWINE ACUTE D
Forni D, 2017, TRENDS MICROBIOL, V25, P35, DOI 10.1016/j.tim.2016.09.001
Frieman MB, 2010, PLOS PATHOG, V6, DOI 10.1371/journal.ppat.1000849
Fulcher M Leslie, 2013, Methods Mol Biol, V945, P109, DOI 10.1007/978-1-62703-125-7_8
Ge XY, 2013, NATURE, V503, P535, DOI 10.1038/nature12711
Gilchuk I, 2016, CELL, V167, P684, DOI 10.1016/j.cell.2016.09.049
Gong L, 2017, EMERG INFECT DIS, V23, P1607, DOI 10.3201/eid2309.170915
Graham RL, 2010, J VIROL, V84, P3134, DOI 10.1128/JVI.01394-09
Grein J, 2020, NEW ENGL J MED, V382, P2327, DOI 10.1056/NEJMoa2007016
Guy JS, 2000, J CLIN MICROBIOL, V38, P4523, DOI 10.1128/JCM.38.12.4523-4526.2000
Hatch GJ, 2013, J VIROL, V87, P7805, DOI 10.1128/JVI.03481-12
Hoffmann M, 2020, CELL, V181, P271, DOI 10.1016/j.cell.2020.02.052
Hou YXJ, 2020, CELL, V182, P429, DOI 10.1016/j.cell.2020.05.042
Hu B, 2015, VIROL J, V12, DOI 10.1186/s12985-015-0422-1
Huang XC, 2015, J VIROL, V89, P7202, DOI 10.1128/JVI.00854-15
Huang YW, 2013, MBIO, V4, DOI 10.1128/mBio.00737-13
Huynh J, 2012, J VIROL, V86, P12816, DOI 10.1128/JVI.00906-12

Jiang GL, 2016, BMC GENOMICS, V17, DOI 10.1186/s12864-016-2911-z
Jung K, 2016, VIRUS RES, V226, P50, DOI 10.1016/j.virusres.2016.04.009
Kim H, 2016, CLIN EXP VACCINE RES, V5, P12, DOI 10.7774/cevr.2016.5.1.12
Latinne Alice, 2020, Nat Commun, V11, P4235, DOI [10.1038/s41467-020-17687-3, 10.1101/2020.05.31.116061]
Li F, 2016, ANNU REV VIROL, V3, P237, DOI 10.1146/annurev-virology-110615-042301
Li WT, 2018, P NATL ACAD SCI USA, V115, pE5135, DOI 10.1073/pnas.1802879115
Li WH, 2003, NATURE, V426, P450, DOI 10.1038/nature02145
Lin CM, 2016, VIRUS RES, V226, P20, DOI 10.1016/j.virusres.2016.05.023
Liu C, 2016, J BIOL CHEM, V291, P24779, DOI 10.1074/jbc.M116.740746
Mackenzie JS, 2019, TROP MED INFECT DIS, V4, DOI 10.3390/tropicalmed4020088
Menachery VD, 2020, J VIROL, V94, DOI 10.1128/JVI.01774-19
Menachery VD, 2016, P NATL ACAD SCI USA, V113, P3048, DOI 10.1073/pnas.1517719113
Menachery VD, 2015, NAT MED, V21, P1508, DOI 10.1038/nm.3985
Mora-Díaz JC, 2019, FRONT VET SCI, V6, DOI 10.3389/fvets.2019.00053
Mulangu S, 2019, NEW ENGL J MED, V381, P2293, DOI 10.1056/NEJMoa1910993
Niederwerder MC, 2018, TRANSBOUND EMERG DIS, V65, P660, DOI 10.1111/tbed.12823
Paarlberg P. L., 2008, ERR57 USDA
Pruijssers Andrea J, 2020, bioRxiv, DOI 10.1101/2020.04.27.064279
Raj VS, 2013, NATURE, V495, P251, DOI 10.1038/nature12005
Roth-Cross JK, 2008, J VIROL, V82, P9829, DOI 10.1128/JVI.01199-08
SAIF LJ, 1994, VET IMMUNOL IMMUNOP, V43, P89, DOI 10.1016/0165-2427(94)90124-4
Scobey T, 2013, P NATL ACAD SCI USA, V110, P16157, DOI 10.1073/pnas.1311542110
Sharma V, 2019, REV MED VIROL, V29, DOI 10.1002/rmv.2010
Sheahan T, 2008, J VIROL, V82, P8721, DOI 10.1128/JVI.00818-08
Sheahan TP, 2020, SCI TRANSL MED, V12, DOI 10.1126/scitranslmed.abb5883
Sheahan TP, 2020, NAT COMMUN, V11, DOI 10.1038/s41467-019-13940-6
Sheahan TP, 2017, SCI TRANSL MED, V9, DOI 10.1126/scitranslmed.aal3653
Sims AC, 2013, J VIROL, V87, P3885, DOI 10.1128/JVI.02520-12
Tang JW, 2017, LANCET INFECT DIS, V17, pE320, DOI 10.1016/S1473-3099(17)30238-4
Tao Y, 2017, J VIROL, V91, DOI 10.1128/JVI.01953-16
Vcelar S, 2018, BIOTECHNOL BIOENG, V115, P165, DOI 10.1002/bit.26453
Vijgen L, 2005, J VIROL, V79, P1595, DOI 10.1128/JVI.79.3.1595-1604.2005
Vojtek I, 2019, VACCINE, V37, P539, DOI 10.1016/j.vaccine.2018.12.005

Wang D, 2016, VIRUS RES, V226, P7, DOI 10.1016/j.virusres.2016.05.026
Wang QH, 2014, CELL HOST MICROBE, V16, P328, DOI 10.1016/j.chom.2014.08.009
Wang QH, 2019, CURR OPIN VIROL, V34, P39, DOI 10.1016/j.coviro.2018.12.001
Wang YL, 2017, CELL MOL GASTROENTER, V4, P165, DOI 10.1016/j.jcmgh.2017.02.011
White Eric S, 2015, Ann Am Thorac Soc, V12 Suppl 1, pS30, DOI 10.1513/AnnalsATS.201406-240MG
Woo PCY, 2017, EMERG MICROBES INFEC, V6, DOI 10.1038/emi.2017.37
World Health Organization, MIDDL E RESP SYNDR C
World Health Organization, NOV COR CHIN
World Health Organization, WHO Summary of Probable SARS Cases with Onset of Illness from 1 November 2002 to 31 July 2003
Yang Yang, 2014, Proc Natl Acad Sci U S A, V111, P12516, DOI 10.1073/pnas.1405889111
Yang YL, 2020, VIRUS RES, V285, DOI 10.1016/j.virusres.2020.198024
Yang YL, 2019, J VIROL, V93, DOI 10.1128/JVI.01448-19
Yang YL, 2019, VIROLOGY, V536, P110, DOI 10.1016/j.virol.2019.08.006
Yount B, 2000, J VIROL, V74, P10600, DOI 10.1128/JVI.74.22.10600-10611.2000
Yount B, 2003, P NATL ACAD SCI USA, V100, P12995, DOI 10.1073/pnas.1735582100
Yount B, 2002, J VIROL, V76, P11065, DOI 10.1128/JVI.76.21.11065-11078.2002
Zhao JC, 2016, IMMUNITY, V44, P1379, DOI 10.1016/j.immuni.2016.05.006
Zheng Y, 2018, J VIROL, V92, DOI 10.1128/JVI.01504-18
Zhou L, 2019, TRANSBOUND EMERG DIS, V66, P2180, DOI 10.1111/tbed.13270
Zhou P, 2018, NATURE, V556, P255, DOI 10.1038/s41586-018-0010-9
NR 90
TC 100
Z9 115
U1 0
U2 22
PU NATL ACAD SCIENCES
PI WASHINGTON
PA 2101 CONSTITUTION AVE NW, WASHINGTON, DC 20418 USA
SN 0027-8424
EI 1091-6490
J9 P NATL ACAD SCI USA
JI Proc. Natl. Acad. Sci. U. S. A.
PD OCT 27
PY 2020
VL 117
IS 43
BP 26915
EP 26925
DI 10.1073/pnas.2001046117
PG 11

WC Multidisciplinary Sciences
WE Science Citation Index Expanded (SCI-EXPANDED)
SC Science & Technology - Other Topics
GA OH6ZD
UT WOS:000582743300050
PM 33046644
OA Green Published, Green Submitted, hybrid
DA 2024-10-21
ER

PT J
AU Morrison, JH
   Miller, C
   Bankers, L
   Crameri, G
   Wang, LF
   Poeschla, EM
AF Morrison, James H.
   Miller, Caitlin
   Bankers, Laura
   Crameri, Gary
   Wang, Lin-Fa
   Poeschla, Eric M.
TI A Potent Postentry Restriction to Primate Lentiviruses in a
   Yinpterochiropteran Bat
SO MBIO
LA English
DT Article
DE retrovirus; HIV-1; lentivirus; bats; restriction factors;
cyclophilin A;
   MX2; Megachiroptera; Yinpterchiroptera; Pteropus alecto;
restriction
   factor
ID AMINO-TERMINAL DOMAIN; IMMUNODEFICIENCY-VIRUS; HIV-1 INFECTION;
   CYCLOPHILIN-A; RETROVIRAL RESTRICTION; REVERSE TRANSCRIPTION;
VIRAL
   DETERMINANTS; B30.2(SPRY) DOMAIN; CAPSID-BINDING; MXB PROTEIN
AB Bats are primary reservoirs for multiple lethal human viruses,
such as Ebola, Nipah, Hendra, rabies, severe acute respiratory
syndrome coronavirus (SARSCoV), Middle East respiratory syndrome-
related coronavirus (MERS-CoV), and, most recently, SARS-CoV-2. The
innate immune systems of these immensely abundant, anciently
diverged mammals remain insufficiently characterized. While bat
genomes contain many endogenous retroviral elements indicative of
past exogenous infections, little is known about restrictions to
extant retroviruses. Here, we describe a major postentry restriction
in cells of the yinpterochiropteran bat Pteropus alecto. Primate
lentiviruses (HIV-1, SIVmac) were potently blocked at early life
cycle steps, with up to 1,000-fold decreases in infectivity. The
block was specific, because nonprimate lentiviruses such as equine
infectious anemia virus and feline immunodeficiency virus were
unimpaired, as were foamy retroviruses. Interspecies heterokaryons
demonstrated a dominant block consistent with restriction of
incoming viruses. Several features suggested potential TRIM5

(tripartite motif 5) or myxovirus resistance protein 2 (MX2) protein restriction, including postentry action, cyclosporine sensitivity, and reversal by capsid cyclophilin A (CypA) binding loop mutations. Viral nuclear import was significantly reduced, and this deficit was substantially rescued by cyclosporine treatment. However, saturation with HIV-1 virus-like particles did not relieve the restriction at all. P. alecto TRIM5 was inactive against HIV-1 although it blocked the gammaretrovirus N-tropic murine leukemia virus. Despite major divergence in a critical N-terminal motif required for human MX2 activity, P. alecto MX2 had anti-HIV activity. However, this did not quantitatively account for the restriction and was independent of and synergistic with an additional CypA-dependent restriction. These results reveal a novel, specific restriction to primate lentiviruses in the Pteropodidae and advance understanding of bat innate immunity.

IMPORTANCE The COVID-19 pandemic suggests that bat innate immune systems are insufficiently characterized relative to the medical importance of these animals. Retroviruses, e.g., HIV-1, can be severe pathogens when they cross species barriers, and bat restrictions corresponding to retroviruses are comparatively unstudied. Here, we compared the abilities of retroviruses from three genera (Lentivirus, Gammaretrovirus, and Spumavirus) to infect cells of the large fruit-eating bat P. alecto and other mammals. We identified a major, specific postentry restriction to primate lentiviruses. HIV-1 and SIVmac are potently blocked at early life cycle steps, but nonprimate lentiviruses and foamy retroviruses are entirely unrestricted. Despite acting postentry and in a CypA-dependent manner with features reminiscent of antiretroviral factors from other mammals, this restriction was not saturable with virus-like particles and was independent of P. alecto TRIM5, TRIM21, TRIM22, TRIM34, and MX2. These results identify a novel restriction and highlight cyclophilin-capsid interactions as ancient species-specific determinants of retroviral infection.
C1 [Morrison, James H.; Miller, Caitlin; Bankers, Laura; Poeschla, Eric M.] Univ Colorado, Div Infect Dis, Aurora, CO 80045 USA.
[Crameri, Gary] CSIRO, Australian Anim Hlth Lab, Geelong, Vic, Australia.
[Wang, Lin-Fa] Duke NUS Med Sch, Programme Emerging Infect Dis, Singapore, Singapore.
C3 University of Colorado System; University of Colorado Anschutz Medical
Campus; Commonwealth Scientific & Industrial Research Organisation
(CSIRO); National University of Singapore
RP Poeschla, EM (corresponding author), Univ Colorado, Div Infect Dis, Aurora, CO 80045 USA.
EM eric.poeschla@cuanschutz.edu
FU NIH [AI077344, DA043915]; Tietze Foundation; NRF [NRF2012NRF-CRP001-056,
NRF2016NRF-NSFC002-013]
FX This work was supported by NIH grants AI077344 and DA043915 and a Tietze
Foundation grant to E.M.P. L.-F.W. is supported NRF grants
NRF2012NRF-CRP001-056 and NRF2016NRF-NSFC002-013.

CR Ahn M, 2019, NAT MICROBIOL, V4, P789, DOI 10.1038/s41564-019-0371-3
Almeida FC, 2014, MOL PHYLOGENET EVOL, V77, P83, DOI 10.1016/j.ympev.2014.03.009
Andersen KG, 2020, NAT MED, V26, P450, DOI 10.1038/s41591-020-0820-9
Baker ML, 2013, ZOONOSES PUBLIC HLTH, V60, P104, DOI 10.1111/j.1863-2378.2012.01528.x
Buffone C, 2015, J VIROL, V89, P3285, DOI 10.1128/JVI.03730-14
Busnadiego I, 2014, J VIROL, V88, P7738, DOI 10.1128/JVI.00214-14
Crameri G, 2009, PLOS ONE, V4, DOI 10.1371/journal.pone.0008266
Cui J, 2012, J GEN VIROL, V93, P2037, DOI 10.1099/vir.0.043760-0
De La Cruz-Rivera PC, 2018, J IMMUNOL, V200, P209, DOI 10.4049/jimmunol.1701214
deCamp A, 2014, J VIROL, V88, P2489, DOI 10.1128/JVI.02853-13
Deyle DR, 2013, GENE THER, V20, P868, DOI 10.1038/gt.2013.6
Fadel HJ, 2012, J VIROL, V86, P2312, DOI 10.1128/JVI.06035-11
FRANKE EK, 1994, NATURE, V372, P359, DOI 10.1038/372359a0
Fricke T, 2014, RETROVIROLOGY, V11, DOI 10.1186/s12977-014-0068-x
Giotis ES, 2019, NAT MICROBIOL, V4, P2035, DOI 10.1038/s41564-019-0517-3
Goujon C, 2015, J VIROL, V89, P4676, DOI 10.1128/JVI.00169-15
Goujon C, 2014, J VIROL, V88, P9017, DOI 10.1128/JVI.01269-14
Goujon C, 2013, NATURE, V502, P559, DOI 10.1038/nature12542
Halpin K, 2011, AM J TROP MED HYG, V85, P946, DOI 10.4269/ajtmh.2011.10-0567
Hayward JA, 2020, P NATL ACAD SCI USA, V117, P9529, DOI 10.1073/pnas.1915400117
Hayward JA, 2018, MOL BIOL EVOL, V35, P1626, DOI 10.1093/molbev/msy048
Hayward JA, 2013, RETROVIROLOGY, V10, DOI 10.1186/1742-4690-10-35
Hrecka K, 2011, NATURE, V474, P658, DOI 10.1038/nature10195
Hron T, 2018, VIRUSES-BASEL, V10, DOI 10.3390/v10040185
Jáuregui P, 2012, J VIROL, V86, P9504, DOI 10.1128/JVI.00440-12
JONES DT, 1992, COMPUT APPL BIOSCI, V8, P275, DOI 10.1093/bioinformatics/8.3.275
Kane M, 2013, NATURE, V502, P563, DOI 10.1038/nature12653
Karakus U, 2019, NATURE, V567, P109, DOI 10.1038/s41586-019-0955-3
Kim K, 2019, NAT MICROBIOL, V4, P2044, DOI 10.1038/s41564-019-0592-5
Koh J, 2019, NAT COMMUN, V10, DOI 10.1038/s41467-019-10495-4
Kong J, 2014, PROTEIN CELL, V5, P954, DOI 10.1007/s13238-014-0113-5
Kozak CA, 1996, VIROLOGY, V225, P300, DOI 10.1006/viro.1996.0604
Kumar S, 2016, MOL BIOL EVOL, DOI [DOI 10.1093/MOLBEV/MSW054, 10.1093/molbev/msw054, doi: 10.1093/molbev/msw054]
Laguette N, 2011, NATURE, V474, P654, DOI 10.1038/nature10117
Lee K, 2010, CELL HOST MICROBE, V7, P221, DOI 10.1016/j.chom.2010.02.007
Li Y, 2009, J VIROL, V83, P10951, DOI 10.1128/JVI.00682-09
Liu ZL, 2013, CELL HOST MICROBE, V14, P398, DOI 10.1016/j.chom.2013.08.015
Llano M, 2006, SCIENCE, V314, P461, DOI 10.1126/science.1132319

Luis AD, 2013, P ROY SOC B-BIOL SCI, V280, DOI 10.1098/rspb.2012.2753
Mahalingam S, 2012, LANCET INFECT DIS, V12, P799, DOI 10.1016/S1473-3099(12)70158-5
Malim MH, 2012, CSH PERSPECT MED, V2, DOI 10.1101/cshperspect.a006940
Matreyek KA, 2014, RETROVIROLOGY, V11, DOI 10.1186/s12977-014-0090-z
McEwan WA, 2009, J VIROL, V83, P8270, DOI 10.1128/JVI.00670-09
McMichael L, 2019, VIRUS GENES, V55, P421, DOI 10.1007/s11262-019-01653-3
Meehan AM, 2014, PLOS PATHOG, V10, DOI 10.1371/journal.ppat.1003969
Meehan AM, 2009, PLOS PATHOG, V5, DOI 10.1371/journal.ppat.1000522
Melen K, 1996, J BIOL CHEM, V271, P23478, DOI 10.1074/jbc.271.38.23478
Mollentze N, 2020, P NATL ACAD SCI USA, V117, P9423, DOI 10.1073/pnas.1919176117
Münk C, 2002, P NATL ACAD SCI USA, V99, P13843, DOI 10.1073/pnas.212400099
O'Rourke JP, 2002, J VIROL, V76, P1510, DOI 10.1128/JVI.76.3.1510-1515.2002
Ohainle M, 2020, PLOS PATHOG, V16, DOI 10.1371/journal.ppat.1008507
Ohkura S, 2006, J VIROL, V80, P8554, DOI 10.1128/JVI.00688-06
Olival KJ, 2017, NATURE, V546, P646, DOI 10.1038/nature22975
Ozato K, 2008, NAT REV IMMUNOL, V8, P849, DOI 10.1038/nri2413
Papenfuss AT, 2012, BMC GENOMICS, V13, DOI 10.1186/1471-2164-13-261
Pertel T, 2011, NATURE, V472, P361, DOI 10.1038/nature09976
Rasaiyaah J, 2013, NATURE, V503, P402, DOI 10.1038/nature12769
Saenz DT, 2005, J VIROL, V79, P15175, DOI 10.1128/JVI.79.24.15175-15188.2005
Saenz Dyana T, 2012, Cold Spring Harb Protoc, V2012, P124, DOI 10.1101/pdb.prot067553
Sayah DM, 2004, NATURE, V430, P569, DOI 10.1038/nature02777
Schulte B, 2015, J VIROL, V89, P8599, DOI 10.1128/JVI.00753-15
Shah VB, 2013, J VIROL, V87, P422, DOI 10.1128/JVI.07177-11
Sheehy AM, 2002, NATURE, V418, P646, DOI 10.1038/nature00939
Song B, 2005, VIROLOGY, V343, P201, DOI 10.1016/j.virol.2005.08.019
Song BW, 2005, J VIROL, V79, P6111, DOI 10.1128/JVI.79.10.6111-6121.2005
Springer MS, 2003, P NATL ACAD SCI USA, V100, P1056, DOI 10.1073/pnas.0334222100
Stremlau M, 2005, J VIROL, V79, P3139, DOI 10.1128/JVI.79.5.3139-3145.2005
Stremlau M, 2004, NATURE, V427, P848, DOI 10.1038/nature02343
Teeling EC, 2005, SCIENCE, V307, P580, DOI 10.1126/science.1105113
THALI M, 1994, NATURE, V372, P363, DOI 10.1038/372363a0
Wang LF, 2019, CURR OPIN VIROL, V34, P79, DOI 10.1016/j.coviro.2018.12.007

   Wang LF, 2011, CURR OPIN VIROL, V1, P649, DOI 10.1016/
j.coviro.2011.10.013
   Wu F, 2020, NATURE, V579, P265, DOI 10.1038/s41586-020-2008-3
   Wynne JW, 2013, PLOS PATHOG, V9, DOI 10.1371/journal.ppat.1003651
   Xie JZ, 2018, CELL HOST MICROBE, V23, P297, DOI 10.1016/
j.chom.2018.01.006
   Yang YL, 2007, VIROLOGY, V365, P92, DOI 10.1016/
j.virol.2007.03.022
   Young GR, 2018, P NATL ACAD SCI USA, V115, P10130, DOI 10.1073/
pnas.1808516115
   Zhang GJ, 2013, SCIENCE, V339, P456, DOI 10.1126/science.1230835
   Zhou P, 2016, P NATL ACAD SCI USA, V113, P2696, DOI 10.1073/
pnas.1518240113
NR 79
TC 12
Z9 13
U1 0
U2 7
PU AMER SOC MICROBIOLOGY
PI WASHINGTON
PA 1752 N ST NW, WASHINGTON, DC 20036-2904 USA
SN 2150-7511
J9 MBIO
JI mBio
PD SEP-OCT
PY 2020
VL 11
IS 5
AR e01854-20
DI 10.1128/mBio.01854-20
PG 20
WC Microbiology
WE Science Citation Index Expanded (SCI-EXPANDED)
SC Microbiology
GA OC9VF
UT WOS:000579503600032
PM 32934084
OA gold, Green Published
DA 2024-10-21
ER

PT J
AU Thakur, A
   Rathore, R
   Kondadasula, SV
   Uberti, JP
   Ratanatharathorn, V
   Lum, LG
AF Thakur, Archana
   Rathore, Ritesh
   Kondadasula, Sri Vidya
   Uberti, Joseph P.
   Ratanatharathorn, Voravit
   Lum, Lawrence G.

TI Immune T cells can transfer and boost anti-breast cancer immunity
SO ONCOIMMUNOLOGY
LA English
DT Article
DE Vaccination; transfer of tumor specific immunity; specific cytotoxic T
   lymphocytes; in vitro tumor specific antibody synthesis; breast cancer;
   bispecific antibody; HER2/neu; immunotherapy
ID HUMAN MARROW TRANSPLANTATION; BISPECIFIC ANTIBODY; CLINICAL-TRIAL;
   IN-VITRO; B-CELLS; RESPONSES; THERAPY; RECONSTITUTION; INTERLEUKIN-2;
   VACCINATION
AB This proof-of-concept study investigates the immune effects in metastatic breast cancer (MBC) patients after "vaccination" with activated T cells (ATC) armed with anti-CD3 x anti-HER2 bispecific antibody (HER2 BATs) followed by immune consolidation with immune ATC "boost" after high dose chemotherapy (HDC) and autologous stem cell transplant (SCT). Approximately 2 weeks after completion of vaccination portion of the study, immune T cells were obtained by leukopheresis, activated and expanded ex vivo and re-infused after HDC and SCT to test the hypothesis that transfer of immune unarmed ATC would accelerate reconstitution of anti-tumor activity after SCT. Eight metastatic breast cancer (MBC) patients received 8 infusions of HER2 BATs, low dose IL-2, and GM-CSF in the first part of the protocol to induce adaptive cellular and humoral responses. In the "boost" portion of the protocol, 6 of 8 patients received multiple infusions of unarmed ATC post SCT. There were no dose-limiting toxicities or delays in engraftment. Four of 6 patients tested for the immune correlative studies exhibited increases in anti-breast cancer (BrCa) cytotoxicity, antigen specific IFN-gamma Elispots, anti-BrCa antibodies and increased IL-12 and Th-1 serum cytokine levels after HER2 BATs infusions. Anti-BrCa tumor responses were seen as early as 2 weeks after SCT and persisted up to 2 years post-SCT. One out of 6 patients' rapidly progressed and showed poor immune responses and high Th-2 cytokine levels. There was a significant correlation ($p < 0.002$) between time to progression (TTP) and anti-BrCa cytotoxicity by immune T cells. This is the first study to show that adoptive transfer of immune T cells after SCT accelerates reconstitution of anti-BrCa specific immunity and correlates with delay TTP.
C1 [Thakur, Archana; Lum, Lawrence G.] Univ Virginia, Canc Ctr, Dept Med, Div Hematol Oncol, 1335 Lee St,West Complex 7191, Charlottesville, VA 22908 USA.
   [Rathore, Ritesh] Roger Williams Hosp, Dept Hematol & Oncol, Providence, RI USA.
   [Kondadasula, Sri Vidya; Uberti, Joseph P.; Ratanatharathorn, Voravit] Wayne State Univ, Sch Med, Dept Oncol, Detroit, MI USA.
   [Kondadasula, Sri Vidya; Uberti, Joseph P.; Ratanatharathorn, Voravit] Karmanos Canc Inst, Detroit, MI USA.
C3 University of Virginia; Roger Williams Medical Center; Wayne State
   University; Barbara Ann Karmanos Cancer Institute

RP Thakur, A (corresponding author), Univ Virginia, Canc Ctr, Dept Med, Div Hematol Oncol, 1335 Lee St,West Complex 7191, Charlottesville, VA 22908 USA.
EM at2fx@virginia.edu
FU Susan G. Komen for the cure [BCTR0707125]; DHHS [R01 CA 092344, R01 CA
140314, P30CA022453]; University of Virginia Cancer Center
FX This study was primarily supported by funding from the Susan G. Komen
for the cure (Grant #BCTR0707125) and in part by DHHS R01 CA 092344, R01
CA 140314, and P30CA022453 (Microscopy, Imaging, and Cytometry Resources
Core), a startup funds from the University of Virginia Cancer Center.
CR Berry DA, 2011, J CLIN ONCOL, V29, P3214, DOI 10.1200/JCO.2010.32.5910
Curti BD, 1998, J CLIN ONCOL, V16, P2752, DOI 10.1200/JCO.1998.16.8.2752
CURTI BD, 1993, J CLIN ONCOL, V11, P652, DOI 10.1200/JCO.1993.11.4.652
Gall JM, 2005, EXP HEMATOL, V33, P452, DOI 10.1016/j.exphem.2005.01.007
Grabert RC, 2006, CLIN CANCER RES, V12, P569, DOI 10.1158/1078-0432.CCR-05-2005
Lum LG, 2015, CLIN CANCER RES, V21, P2305, DOI 10.1158/1078-0432.CCR-14-2280
LUM LG, 1986, J CLIN IMMUNOL, V6, P389, DOI 10.1007/BF00915378
LUM LG, 1985, J IMMUNOL, V135, P185
LUM LG, 1987, BLOOD, V69, P369
Lum LG, 2000, P 10 INT S AUT BLOOD, P95
MATSUE K, 1987, BLOOD, V69, P308
Rapoport AP, 2011, BLOOD, V117, P788, DOI 10.1182/blood-2010-08-299396
Rapoport AP, 2005, NAT MED, V11, P1230, DOI 10.1038/nm1310
SAXON A, 1986, J CLIN INVEST, V78, P959, DOI 10.1172/JCI112686
Sen M, 2001, J HEMATOTH STEM CELL, V10, P247, DOI 10.1089/152581601511349944
Simpson-Abelson MR, 2009, CLIN IMMUNOL, V132, P71, DOI 10.1016/j.clim.2009.03.516
Stadtmauer EA, 2011, BLOOD, V117, P63, DOI 10.1182/blood-2010-07-296822
STEVENS RH, 1978, J CLIN INVEST, V62, P1154, DOI 10.1172/JCI109234
Thakur A, 2011, CANCER IMMUNOL IMMUN, V60, P1707, DOI 10.1007/s00262-011-1056-9
Thakur A, 2011, J IMMUNOTHER, V34, P457, DOI 10.1097/CJI.0b013e31821dcba5
WAHREN B, 1984, J INFECT DIS, V150, P358, DOI 10.1093/infdis/150.3.358
WITHERSPOON RP, 1982, BLOOD, V59, P844
NR 22
TC 18
Z9 19

U1 0
U2 8
PU TAYLOR & FRANCIS INC
PI PHILADELPHIA
PA 530 WALNUT STREET, STE 850, PHILADELPHIA, PA 19106 USA
SN 2162-402X
J9 ONCOIMMUNOLOGY
JI OncoImmunology
PD DEC 2
PY 2018
VL 7
IS 12
AR e1500672
DI 10.1080/2162402X.2018.1500672
PG 11
WC Oncology; Immunology
WE Science Citation Index Expanded (SCI-EXPANDED)
SC Oncology; Immunology
GA HA7LE
UT WOS:000450462000002
PM 30524893
OA gold, Green Published
DA 2024-10-21
ER

PT J
AU Nemcova, M
   Seidlova, V
   Zukal, J
   Dundarova, H
   Zukalova, K
   Pikula, J
AF Nemcova, Monika
   Seidlova, Veronika
   Zukal, Jan
   Dundarova, Heliana
   Zukalova, Katerina
   Pikula, Jiri
TI Performance of bat-derived macrophages at different temperatures
SO FRONTIERS IN VETERINARY SCIENCE
LA English
DT Article
DE Chiroptera (bats); in vitro model; hibernation; phagocytic activity;
   temperature-dependent proliferation; daily torpor; macrophage biology
ID WHITE-NOSE SYNDROME; PSEUDOGYMNOASCUS-DESTRUCTANS; MAMMALIAN
   HIBERNATION; IMMUNE-RESPONSES; SEASONAL-CHANGES; CELLS; AROUSAL; COLD;
   PHAGOCYTOSIS; EXPRESSION
AB Heterothermy, as a temperature-dependent physiological continuum, may affect host-pathogen interactions through modulation of immune responses. Here, we evaluated proliferation and functional performance of a macrophage cell line established from the greater

mouse-eared (Myotis myotis) bat at 8, 17.5, and 37 degrees C to simulate body temperatures during hibernation, daily torpor and euthermia. Macrophages were also frozen to -20 degrees C and then examined for their ability to proliferate in the immediate post-thaw period. We show that bat macrophages can proliferate at lower temperatures, though their growth rate is significantly slower than at 37 degrees C. The cells differed in their shape, size and ability to attach to the plate surface at both lower temperatures, being spheroidal and free in suspension at 8 degrees C and epithelial-like, spindle-shaped and/or spheroidal at 17.5 degrees C. While phagocytosis at temperatures of 8 and 17.5 degrees C amounted to 85.8 and 83.1% of the activity observed at 37 degrees C, respectively, full phagocytic activity was restored within minutes of translocation into a higher temperature. Bat-derived macrophages were also able to withstand temperatures of -20 degrees C in a cryoprotectant-free cultivation medium and, in the immediate post-thaw period, became viable and were able to proliferate. Our in vitro data enhance understanding of macrophage biology.
C1 [Nemcova, Monika; Seidlova, Veronika; Zukalova, Katerina; Pikula, Jiri] Univ Vet Sci Brno, Dept Ecol & Dis Zoo Anim Game Fish & Bees, Brno, Czech Republic.
[Seidlova, Veronika; Zukal, Jan] Czech Acad Sci, Inst Vertebrate Biol, Brno, Czech Republic.
[Zukal, Jan] Masaryk Univ, Dept Bot & Zool, Brno, Czech Republic.
[Dundarova, Heliana] Bulgarian Acad Sci, Inst Biodivers & Ecosyst Res, Sofia, Bulgaria.
[Pikula, Jiri] Univ Vet Sci Brno, Cent European Inst Technol, CEITEC, Brno, Czech Republic.
C3 University of Veterinary Sciences Brno; Czech Academy of Sciences;
Institute of Vertebrate Biology of the Czech Academy of Sciences;
Masaryk University Brno; Bulgarian Academy of Sciences; University of
Veterinary Sciences Brno
RP Pikula, J (corresponding author), Univ Vet Sci Brno, Dept Ecol & Dis Zoo Anim Game Fish & Bees, Brno, Czech Republic.; Pikula, J (corresponding author), Univ Vet Sci Brno, Cent European Inst Technol, CEITEC, Brno, Czech Republic.
EM pikulaj@vfu.cz
RI Dundarova, Heliana/HNI-7801-2023; Seidlova, Veronika/HNQ-0598-2023;
Pikula, Jiri/V-8620-2018; Zukal, Jan/G-1180-2014
OI Pikula, Jiri/0000-0001-8747-9365; Nemcova, Monika/0000-0001-8835-2956;
Zukal, Jan/0000-0003-4967-6880; Dundarova, Heliana/0000-0002-3136-6457
CR Abdelsalam EEE, 2020, ACTA VET BRNO, V89, P151, DOI 10.2754/avb202089020151
Andrews MT, 2007, BIOESSAYS, V29, P431, DOI 10.1002/bies.20560
Bachorec E, 2021, J THERM BIOL, V101, DOI 10.1016/j.jtherbio.2021.103107
Bandouchova H, 2018, SCI REP-UK, V8, DOI 10.1038/s41598-018-24461-5
Bartonicka T, 2017, J THERM BIOL, V63, P119, DOI 10.1016/

j.jtherbio.2016.12.005
Blazek J, 2019, J THERM BIOL, V82, P150, DOI 10.1016/j.jtherbio.2019.04.002
Blehert DS, 2009, SCIENCE, V323, P227, DOI 10.1126/science.1163874
Bouma Hjalmar R., 2012, P259
Bouma HR, 2010, J LEUKOCYTE BIOL, V88, P619, DOI 10.1189/jlb.0310174
Boyer BB, 1999, BIOSCIENCE, V49, P713, DOI 10.2307/1313595
Brown EJ., 2003, FUNDAMENTAL IMMUNOLO, V5th
Burdon R H, 1987, Symp Soc Exp Biol, V41, P113
Calisher CH, 2006, CLIN MICROBIOL REV, V19, P531, DOI 10.1128/CMR.00017-06
Carey HV, 2003, PHYSIOL REV, V83, P1153, DOI 10.1152/physrev.00008.2003
ChangLiu CM, 1997, CANCER LETT, V113, P77, DOI 10.1016/S0304-3835(97)04599-0
Davy CM, 2020, VIRULENCE, V11, P781, DOI 10.1080/21505594.2020.1768018
Dietz M, 2006, J COMP PHYSIOL B, V176, P223, DOI 10.1007/s00360-005-0043-x
Douglas S.D., 2015, Williams Hematology
Flieger M, 2016, SCI REP-UK, V6, DOI 10.1038/srep33200
Frick WF, 2010, SCIENCE, V329, P679, DOI 10.1126/science.1188594
Fujita Jun, 1999, Journal of Molecular Microbiology and Biotechnology, V1, P243
Fuller B., 2004, Problems Cryobiol, V3, P58
Gordon S, 2017, BMC BIOL, V15, DOI 10.1186/s12915-017-0392-4
Gordon S, 2007, EUR J IMMUNOL, V37, pS9, DOI 10.1002/eji.200737638
Harding RL, 2016, PHYSIOL REP, V4, DOI 10.14814/phy2.12770
He XC, 2014, PLOS ONE, V9, DOI 10.1371/journal.pone.0109795
Heger T, 2020, ACTA VET BRNO, V89, P79, DOI 10.2754/avb202089010079
Horsthemke M, 2017, J BIOL CHEM, V292, P7258, DOI 10.1074/jbc.M116.766923
Kacprzyk J, 2017, ACTA CHIROPTEROL, V19, P219, DOI [10.3161/15081109ACC2017.19.2.001, 10.3161/15081109acc2017.19.2.001]
Kuznetsova EV, 2016, BIOL BULL+, V43, P344, DOI 10.1134/S1062359016040087
Lake NC, 1917, LANCET, V2, P557
Lausen CL, 2003, J ZOOL, V260, P235, DOI 10.1017/S0952836903003686
LEIBO S P, 1970, Cryobiology, V6, P315, DOI 10.1016/S0011-2240(70)80086-4
Lin HH, 2005, J EXP MED, V201, P1615, DOI 10.1084/jem.20042307
Lorch JM, 2011, NATURE, V480, P376, DOI 10.1038/nature10590
Luis AD, 2006, FUNCT ECOL, V20, P471, DOI 10.1111/j.1365-2435.2006.01119.x
Mandl JN, 2018, FRONT IMMUNOL, V9, DOI 10.3389/fimmu.2018.02112
Mandl JN, 2015, CELL, V160, P20, DOI 10.1016/j.cell.2014.12.003
Maniero GD, 2005, COMP IMMUNOL MICROB, V28, P297, DOI 10.1016/j.cimid.2005.08.001
Martínková N, 2018, VIRULENCE, V9, P1734, DOI

10.1080/21505594.2018.1548685
MAZUR P, 1969, CRYOBIOLOGY, V6, P1, DOI 10.1016/S0011-2240(69)80002-7
MAZUR P, 1963, J GEN PHYSIOL, V47, P347, DOI 10.1085/jgp.47.2.347
MCNAB BK, 1974, ECOLOGY, V55, P943, DOI 10.2307/1940347
Medalia O, 2007, CURR BIOL, V17, P79, DOI 10.1016/j.cub.2006.11.022
Meteyer CU, 2012, VIRULENCE, V3, P583, DOI 10.4161/viru.22330
Murray PJ, 2011, NAT REV IMMUNOL, V11, P723, DOI 10.1038/nri3073
Mylvaganam S, 2021, CURR BIOL, V31, pR619, DOI 10.1016/j.cub.2021.01.036
O'Neill LAJ, 2016, J EXP MED, V213, P15, DOI 10.1084/jem.20151570
Pikula J, 2020, BMC VET RES, V16, DOI 10.1186/s12917-020-02450-z
Pikula J, 2017, PLOS ONE, V12, DOI 10.1371/journal.pone.0180435
Pohanka M, 2011, CURR MED CHEM, V18, P539, DOI 10.2174/092986711794480140
Prendergast BJ, 2002, AM J PHYSIOL-REG I, V282, pR1054, DOI 10.1152/ajpregu.00562.2001
Ruf T, 2015, BIOL REV, V90, P891, DOI 10.1111/brv.12137
Schmidt EP, 2016, COMPR PHYSIOL, V6, P945, DOI 10.1002/cphy.c150020
Seidlova V, 2021, TRANSBOUND EMERG DIS, V68, P3089, DOI 10.1111/tbed.14011
Shima A, 2008, ZOOL SCI, V25, P1066, DOI 10.2108/zsj.25.1066
STEPHENSON NG, 1966, J EMBRYOL EXP MORPH, V16, P455
Tamura Y, 2006, BRAIN RES, V1108, P107, DOI 10.1016/j.brainres.2006.06.020
Ungai-Salánki R, 2019, ADV COLLOID INTERFAC, V269, P309, DOI 10.1016/j.cis.2019.05.005
Uribe-Querol E, 2020, FRONT IMMUNOL, V11, DOI 10.3389/fimmu.2020.01066
WARHEIT DB, 1984, EXP LUNG RES, V6, P71, DOI 10.3109/01902148409087896
Webb PI, 1996, CAN J ZOOL, V74, P761, DOI 10.1139/z96-087
WINOKUR R, 1995, BLOOD, V85, P1796, DOI 10.1182/blood.V85.7.1796.bloodjournal8571796
Wojciechowski MS, 2007, COMP BIOCHEM PHYS A, V147, P828, DOI 10.1016/j.cbpa.2006.06.039
Xu YC, 2013, BMC GENOMICS, V14, DOI 10.1186/1471-2164-14-567
YANCEY PH, 1978, J COMP PHYSIOL, V125, P129, DOI 10.1007/BF00686748
Zhou P., 2020, NATURE, V579, P270, DOI DOI 10.1038/s41586-020-2012-7
Zukal J, 2016, SCI REP-UK, V6, DOI 10.1038/srep19829
NR 68
TC 3
Z9 3
U1 0
U2 7
PU FRONTIERS MEDIA SA
PI LAUSANNE
PA AVENUE DU TRIBUNAL FEDERAL 34, LAUSANNE, CH-1015, SWITZERLAND
EI 2297-1769
J9 FRONT VET SCI

JI Front. Vet. Sci.
PD SEP 9
PY 2022
VL 9
AR 978756
DI 10.3389/fvets.2022.978756
PG 12
WC Veterinary Sciences
WE Science Citation Index Expanded (SCI-EXPANDED)
SC Veterinary Sciences
GA 4U1FI
UT WOS:000858548200001
PM 36157196
OA gold, Green Published
DA 2024-10-21
ER

PT J
AU Guerrero-Chacón, AL
   Rivera-Ruíz, D
   Rojas-Díaz, V
   Triana-Llanos, C
   Niño-Castro, A
AF Guerrero-Chacon, Adriana L.
   Rivera-Ruiz, David
   Rojas-Diaz, Vladimir
   Triana-Llanos, Camila
   Nino-Castro, Andrea
TI Metabolic cost of acute phase response in the frugivorous bat,
   <i>Artibeus lituratus</i>
SO MAMMAL RESEARCH
LA English
DT Article
DE Artibeus lituratus; Immune challenge; Metabolic cost; Bacterial
   lipopolysaccharide; Acute phase response
ID FRUIT BATS; RESERVOIR HOSTS; PTEROPID BATS; NIPAH VIRUS;
TRANSMISSION;
   LEPTOSPIRA; MAMMALS; LPS
AB Bats play a key role as host for multiple microorganism and virus
without showing clinical manifestations of disease. After
recognition of a potential threat, innate immunity triggers acute
phase response, a systemic reaction that contributes to restrain
microbial and viral growth. APR is characterized by fever,
leukocytosis, and production of acute phase proteins, but also by
behavioral changes, including somnolence, lethargy, and anorexia.
Deploying immune responses, such as acute phase response, represents
an energetic cost for vertebrates. In bats, it has been suggested
that higher metabolic rates reached during flight might subsidize
any inherent cost of raising metabolism to activate an immune
response. Therefore, a central question is whether immune response
represents a significant cost to bats and, if so, how much is the
metabolic cost of these responses. Here, we assess the resting
metabolic rate of Artibeus lituratus in response to challenge with
LPS. In addition, we assessed parameters of acute phase response

including fever, body mass loss, and leukocytosis in this specie. We found that challenge with LPS leads to an increase of 40% in resting metabolic rate of A. lituratus, concomitant with body mass loss and an increase in body temperature of 1.5 A degrees C.
C1 [Guerrero-Chacon, Adriana L.; Rivera-Ruiz, David; Triana-Llanos, Camila; Nino-Castro, Andrea] Univ Valle, Dept Biol, Lab Fisiol Anim, Calle 13,100-00 Bldg,320 Off, Cali 3115, Colombia.
[Rojas-Diaz, Vladimir] Wildlife Conservat Soc, Cali, Colombia.
C3 Universidad del Valle
RP Guerrero-Chacón, AL (corresponding author), Univ Valle, Dept Biol, Lab Fisiol Anim, Calle 13,100-00 Bldg,320 Off, Cali 3115, Colombia.
EM andrea.nino@correounivalle.edu.co
OI ROJAS DIAZ, VLADIMIR/0000-0002-3864-2055
FU Vicerrectoria de Investigacion Universidad del Valle CI [71027]; Posgrado en Ciencias Biologia Universidad del Valle
FX This research was supported by Vicerrectoria de Investigacion Universidad del Valle CI 71027 and Posgrado en Ciencias Biologia Universidad del Valle. We thank Dr. Luis Gerardo Herrera Montalvo for his critical reading of the manuscript and Alexander Torres MSc for his valuable advice on statistical analysis of data.
CR Akira S, 2004, NAT REV IMMUNOL, V4, P499, DOI 10.1038/nri1391
[Anonymous], MAXIMAL HEAT DISSIPA
[Anonymous], 2008, MAMMALS S AM
[Anonymous], 2019, CAR AN R COMPANION A
[Anonymous], ACUTE PHASE RESPONSE
[Anonymous], PEERJ PREPR
[Anonymous], 2017, LINEAR NONLINEAR MIX
[Anonymous], 2015, PLOS PATHOG, DOI DOI 10.1371/JOURNAL.PPAT.1005176
[Anonymous], MAMMAL SPECIES WORLD
[Anonymous], MEASURES CONSTITUTIV
Baker ML, 2013, ZOONOSES PUBLIC HLTH, V60, P104, DOI 10.1111/j.1863-2378.2012.01528.x
BAUMANN H, 1994, IMMUNOL TODAY, V15, P74, DOI 10.1016/0167-5699(94)90137-6
Berzunza-Cruz M, 2015, PLOS NEGLECT TROP D, V9, DOI 10.1371/journal.pntd.0003438
Beutler B, 2000, CURR OPIN IMMUNOL, V12, P20, DOI 10.1016/S0952-7915(99)00046-1
Blander JM, 2012, NAT REV IMMUNOL, V12, P215, DOI 10.1038/nri3167
BROGDEN KA, 1984, EXP LUNG RES, V7, P123, DOI 10.3109/01902148409069673
Brook CE, 2015, TRENDS MICROBIOL, V23, P172, DOI 10.1016/j.tim.2014.12.004
Bunnell JE, 2000, AM J TROP MED HYG, V63, P255, DOI 10.4269/ajtmh.2000.63.255
Burness G, 2010, J EXP BIOL, V213, P1386, DOI 10.1242/jeb.027011
Calisher CH, 2006, CLIN MICROBIOL REV, V19, P531, DOI 10.1128/CMR.00017-06
Chua KB, 2002, MICROBES INFECT, V4, P145, DOI 10.1016/S1286-4579(01)01522-2

Cray C, 2009, COMPARATIVE MED, V59, P517
EMANUEL ML, 1964, J HYG-CAMBRIDGE, V62, P451, DOI 10.1017/S0022172400040195
Evans SS, 2015, NAT REV IMMUNOL, V15, P335, DOI 10.1038/nri3843
Gabay C, 1999, NEW ENGL J MED, V340, P448, DOI 10.1056/NEJM199902113400607
Gannon WL, 2007, J MAMMAL, V88, P809, DOI 10.1644/06-MAMM-F-185R1.1
Gomard Y, 2016, FEMS MICROBIOL ECOL, V92, DOI 10.1093/femsec/fiw037
Halpin K, 2011, AM J TROP MED HYG, V85, P946, DOI 10.4269/ajtmh.2011.10-0567
Hodo CL, 2016, ACTA TROP, V164, P259, DOI 10.1016/j.actatropica.2016.09.013
Hothorn T, 2008, BIOMETRICAL J, V50, P346, DOI 10.1002/bimj.200810425
King MO, 2013, J EXP BIOL, V216, P2097, DOI 10.1242/jeb.079574
KOVTUN MF, 1994, FOLIA ZOOL, V43, P377
Kuzmin IV, 2017, J VIROL, V91, DOI [10.1128/JVI.02471-16, 10.1128/jvi.02471-16]
Lauvau G, 2001, SCIENCE, V294, P1735, DOI 10.1126/science.1064571
Leigh E.G., 1982, ECOLOGY TROPICAL FOR, P111
Leroy EM, 2005, NATURE, V438, P575, DOI 10.1038/438575a
Lighton J.R.B., 2008, MEASURING METABOLIC, DOI DOI 10.1242/JEB.65.3.643
Lochmiller RL, 2000, OIKOS, V88, P87, DOI 10.1034/j.1600-0706.2000.880110.x
MacDonald L, 2012, PHYSIOL BEHAV, V107, P762, DOI 10.1016/j.physbeh.2012.06.009
Marais M, 2011, J THERM BIOL, V36, P116, DOI 10.1016/j.jtherbio.2010.12.004
Matthias MA, 2005, AM J TROP MED HYG, V73, P964, DOI 10.4269/ajtmh.2005.73.964
Middleton DJ, 2007, J COMP PATHOL, V136, P266, DOI 10.1016/j.jcpa.2007.03.002
Munster VJ, 2016, SCI REP-UK, V6, DOI 10.1038/srep21878
O'Mara MT, 2017, ROY SOC OPEN SCI, V4, DOI 10.1098/rsos.171359
O'Mara MT, 2017, ELIFE, V6, DOI 10.7554/eLife.26686.001
Otálora-Ardila A, 2016, PLOS ONE, V11, DOI 10.1371/journal.pone.0164938
Paweska JT, 2016, VIRUSES-BASEL, V8, DOI 10.3390/v8020029
R Core Team, 2017, R LANG ENV STAT COMP
Pereira MJR, 2010, BIOTROPICA, V42, P680, DOI 10.1111/j.1744-7429.2010.00635.x
Schneeberger K, 2013, J EXP BIOL, V216, P4514, DOI 10.1242/jeb.090837
Stockmaier S, 2015, BIOL LETTERS, V11, DOI 10.1098/rsbl.2015.0576
Tulsiani SM, 2011, ANN TROP MED PARASIT, V105, P71, DOI 10.1179/136485911X12899838413501
Vance RE, 2009, CELL HOST MICROBE, V6, P10, DOI 10.1016/j.chom.2009.06.007
Viney M.E., 2014, Eco-immunology: Evolutive Aspects and Future Perspectives, P1
VONKOENIG CHW, 1982, NATURE, V297, P233, DOI 10.1038/297233a0

   Weise P, 2017, PEERJ, V5, DOI 10.7717/peerj.3570
   Williamson MM, 1998, AUST VET J, V76, P813, DOI 10.1111/j.1751-0813.1998.tb12335.x
   ZORTEA M, 1993, J TROP ECOL, V9, P117, DOI 10.1017/S0266467400007057
NR 58
TC 10
Z9 12
U1 1
U2 33
PU SPRINGER HEIDELBERG
PI HEIDELBERG
PA TIERGARTENSTRASSE 17, D-69121 HEIDELBERG, GERMANY
SN 2199-2401
EI 2199-241X
J9 MAMMAL RES
JI Mammal Res.
PD OCT
PY 2018
VL 63
IS 4
BP 397
EP 404
DI 10.1007/s13364-018-0375-z
PG 8
WC Zoology
WE Science Citation Index Expanded (SCI-EXPANDED)
SC Zoology
GA GT7XK
UT WOS:000444745100001
DA 2024-10-21
ER

PT J
AU Burke, B
   Rocha, SM
   Zhan, SJ
   Eckley, M
   Reasoner, C
   Addetia, A
   Lewis, J
   Fagre, A
   Charley, PA
   Richt, JA
   Weiss, SR
   Tjalkens, RB
   Veesler, D
   Aboellail, T
   Schountz, T
AF Burke, Bradly
   Rocha, Savannah M.
   Zhan, Shijun
   Eckley, Miles
   Reasoner, Clara

Addetia, Amin
Lewis, Juliette
Fagre, Anna
Charley, Phillida A.
Richt, Juergen A.
Weiss, Susan R.
Tjalkens, Ronald B.
Veesler, David
Aboellail, Tawfik
Schountz, Tony
TI Regulatory T cell-like response to SARS-CoV-2 in Jamaican fruit bats
(Artibeus jamaicensis) transduced with human ACE2
SO PLOS PATHOGENS
LA English
DT Article
ID RESPIRATORY SYNDROME CORONAVIRUS; MARBURG VIRUS; B-CELLS; MICE; INFECTION
AB Insectivorous Old World horseshoe bats (Rhinolophus spp.) are the likely source of the ancestral SARS-CoV-2 prior to its spillover into humans and causing the COVID-19 pandemic. Natural coronavirus infections of bats appear to be principally confined to the intestines, suggesting fecal-oral transmission; however, little is known about the biology of SARS-related coronaviruses in bats. Previous experimental challenges of Egyptian fruit bats (Rousettus aegyptiacus) resulted in limited infection restricted to the respiratory tract, whereas insectivorous North American big brown bats (Eptesicus fuscus) showed no evidence of infection. In the present study, we challenged Jamaican fruit bats (Artibeus jamaicensis) with SARS-CoV-2 to determine their susceptibility. Infection was confined to the intestine for only a few days with prominent viral nucleocapsid antigen in epithelial cells, and mononuclear cells of the lamina propria and Peyer's patches, but with no evidence of infection of other tissues; none of the bats showed visible signs of disease or seroconverted. Expression levels of ACE2 were low in the lungs, which may account for the lack of pulmonary infection. Bats were then intranasally inoculated with a replication-defective adenovirus encoding human ACE2 and 5 days later challenged with SARS-CoV-2. Viral antigen was prominent in lungs for up to 14 days, with loss of pulmonary cellularity during this time; however, the bats did not exhibit weight loss or visible signs of disease. From day 7, bats had low to moderate IgG antibody titers to spike protein by ELISA, and one bat on day 10 had low-titer neutralizing antibodies. CD4(+) helper T cells became activated upon ex vivo recall stimulation with SARS-CoV-2 nucleocapsid peptide library and exhibited elevated mRNA expression of the regulatory T cell cytokines interleukin-10 and transforming growth factor-beta, which may have limited inflammatory pathology. Collectively, these data show that Jamaican fruit bats are poorly susceptible to SARS-CoV-2 but that expression of human ACE2 in their lungs leads to robust infection and an adaptive immune response with low-titer antibodies and a regulatory T cell-like response that may explain the lack of prominent inflammation in the lungs. This model will allow for insight of how SARS-CoV-2 infects bats and how bat

innate and adaptive immune responses engage the virus without overt clinical disease.
C1 [Burke, Bradly; Zhan, Shijun; Eckley, Miles; Reasoner, Clara; Lewis, Juliette; Fagre, Anna; Charley, Phillida A.; Aboellail, Tawfik; Schountz, Tony] Colorado State Univ, Coll Vet Med, Dept Microbiol Immunol & Pathol, Ft Collins, CO 80523 USA.
[Rocha, Savannah M.; Tjalkens, Ronald B.] Colorado State Univ, Coll Vet Med, Dept Environm & Radiol Hlth Sci, Ft Collins, CO USA.
[Addetia, Amin; Veesler, David] Univ Washington, Dept Biochem, Seattle, WA USA.
[Richt, Juergen A.] Kansas State Univ, Coll Vet Med, Ctr Excellence Emerging & Zoonot Anim Dis, Diagnost Med Pathobiol, Manhattan, KS USA.
[Weiss, Susan R.] Univ Penn, Perelman Sch Med, Dept Microbiol, Philadelphia, PA USA.
C3 Colorado State University; Colorado State University; University of
Washington; University of Washington Seattle; Kansas State University;
University of Pennsylvania
RP Schountz, T (corresponding author), Colorado State Univ, Coll Vet Med, Dept Microbiol Immunol & Pathol, Ft Collins, CO 80523 USA.
EM tony.schountz@colostate.edu
RI Weiss, Susan/J-8263-2015; Aboellail, Tawfik/AAB-8990-2019; Veesler,
David/HCH-3986-2022
FU We thank Corey Campbell for assistance with statistical analysis.
FX We thank Corey Campbell for assistance with statistical analysis.
CR Aicher SM, 2022, J VIROL, V96, DOI 10.1128/jvi.00608-22
Amman BR, 2015, J WILDLIFE DIS, V51, P113, DOI 10.7589/2014-08-198
Annan A, 2013, EMERG INFECT DIS, V19, P456, DOI 10.3201/eid1903.121503
Avery DT, 2008, J IMMUNOL, V181, P1767, DOI 10.4049/jimmunol.181.3.1767
Bosco-Lauth AM, 2022, VIRUSES-BASEL, V14, DOI 10.3390/v14081809
Chan JFW, 2020, CLIN INFECT DIS, V71, P2428, DOI 10.1093/cid/ciaa325
Chattopadhyay PK, 2005, NAT MED, V11, P1113, DOI 10.1038/nm1293
Ciminski K, 2019, NAT MICROBIOL, V4, P2298, DOI 10.1038/s41564-019-0556-9
Cogswell-Hawkinson A, 2012, J VIROL, V86, P5791, DOI 10.1128/JVI.00201-12
Corman VM, 2020, EUROSURVEILLANCE, V25, P23, DOI 10.2807/1560-7917.ES.2020.25.3.2000045
Delaune D, 2021, NAT COMMUN, V12, DOI 10.1038/s41467-021-26809-4
Dikiy S, 2023, IMMUNITY, V56, P240, DOI 10.1016/j.immuni.2023.01.004
Dinnon KH III, 2022, SCI TRANSL MED, V14, DOI 10.1126/scitranslmed.abo5070
Easterbrook JD, 2007, P NATL ACAD SCI USA, V104, P15502, DOI 10.1073/pnas.0707453104
Fagre A, 2021, PLOS PATHOG, V17, DOI 10.1371/journal.ppat.1009585
Ferragut F, 2023, IMMUNOLOGY, V169, P185, DOI 10.1111/imm.13622

Foo R, 2022, J AM ASSOC LAB ANIM, V61, P344, DOI 10.30802/AALAS-JAALAS-21-000090
Frentsch M, 2005, NAT MED, V11, P1118, DOI 10.1038/nm1292
Ge XY, 2013, NATURE, V503, P535, DOI 10.1038/nature12711
Gerrard Diana L, 2017, mSphere, V2, DOI 10.1128/mSphere.00245-17
Griffin BD, 2021, NAT COMMUN, V12, DOI 10.1038/s41467-021-23848-9
Guito JC, 2021, CURR BIOL, V31, P257, DOI 10.1016/j.cub.2020.10.015
Hale VL., 2021, bioRxiv, DOI DOI 10.1101/2021.11.04.467308
Hall J, 2022, bioRxiv, DOI [10.1101/2022.07.18.500430, 10.1101/2022.07.18.500430, DOI 10.1101/2022.07.18.500430]
Hall JS, 2021, TRANSBOUND EMERG DIS, V68, P3443, DOI 10.1111/tbed.13949
Halpin K, 2011, AM J TROP MED HYG, V85, P946, DOI 10.4269/ajtmh.2011.10-0567
Hassan AO, 2020, CELL, V182, P744, DOI 10.1016/j.cell.2020.06.011
Hayward JA, 2022, J VIROL, V96, DOI 10.1128/jvi.01152-22
HENIKOFF S, 1992, P NATL ACAD SCI USA, V89, P10915, DOI 10.1073/pnas.89.22.10915
Hsieh CL, 2020, SCIENCE, V369, P1501, DOI 10.1126/science.abd0826
Hu B, 2017, PLOS PATHOG, V13, DOI 10.1371/journal.ppat.1006698
Ithete NL, 2013, EMERG INFECT DIS, V19, P1697, DOI 10.3201/eid1910.130946
Kelley LA, 2015, NAT PROTOC, V10, P845, DOI 10.1038/nprot.2015.053
Kirejczyk SGM, 2022, VIRUSES-BASEL, V14, DOI 10.3390/v14061278
Lau SKP, 2005, P NATL ACAD SCI USA, V102, P14040, DOI 10.1073/pnas.0506735102
Leng L, 2020, SIGNAL TRANSDUCT TAR, V5, DOI 10.1038/s41392-020-00355-9
Letko M, 2020, NAT MICROBIOL, V5, P562, DOI [10.1038/s41564-020-0688-y, 10.1101/2020.01.22.915660]
Li WD, 2005, SCIENCE, V310, P676, DOI 10.1126/science.1118391
Linterman MA, 2010, J EXP MED, V207, P353, DOI 10.1084/jem.20091738
Luan JW, 2020, J MED VIROL, V92, P1649, DOI 10.1002/jmv.25817
Malmlov A, 2019, PLOS NEGLECT TROP D, V13, DOI 10.1371/journal.pntd.0007071
McCallum M, 2021, SCIENCE, V374, P1621, DOI [10.1126/science.abl8506, 10.1101/2021.08.11.455956]
McCallum M, 2021, SCIENCE, V373, P648, DOI 10.1126/science.abi7994
Molenaar RJ, 2020, VET PATHOL, V57, P653, DOI 10.1177/0300985820943535
Munster VJ, 2016, SCI REP-UK, V6, DOI 10.1038/srep21878
Neidleman J, 2021, CELL REP, V36, DOI 10.1016/j.celrep.2021.109414
Oko L, 2006, BMC IMMUNOL, V7, DOI 10.1186/1471-2172-7-18
Oreshkova N, 2020, EUROSURVEILLANCE, V25, P2, DOI 10.2807/1560-7917.ES.2020.25.23.2001005
Paweska JT, 2012, PLOS ONE, V7, DOI 10.1371/journal.pone.0045479
Poon LLM, 2005, J VIROL, V79, P2001, DOI 10.1128/JVI.79.4.2001-2009.2005
Pusnik J, 2021, CELL REP, V35, DOI 10.1016/j.celrep.2021.109320

Rathnasinghe R, 2020, EMERG MICROBES INFEC, V9, P2433, DOI 10.1080/22221751.2020.1838955
Reusken CBEM, 2013, LANCET INFECT DIS, V13, P859, DOI 10.1016/S1473-3099(13)70164-6
Rocha SM, 2022, FRONT IMMUNOL, V13, DOI 10.3389/fimmu.2022.811430
Ruiz-Aravena M, 2022, NAT REV MICROBIOL, V20, P299, DOI 10.1038/s41579-021-00652-2
Schlottau K, 2020, LANCET MICROBE, V1, pE218, DOI 10.1016/S2666-5247(20)30089-6
Schountz T, 2007, P NATL ACAD SCI USA, V104, P15496, DOI 10.1073/pnas.0707454104
Schountz T, 2014, VIRUSES-BASEL, V6, P4880, DOI 10.3390/v6124880
Schountz T, 2012, J VIROL, V86, P10015, DOI 10.1128/JVI.06875-11
Schuh AJ, 2019, J INFECT DIS, V219, P1716, DOI 10.1093/infdis/jiy733
Schuh AJ, 2017, SCI REP-UK, V7, DOI 10.1038/s41598-017-07824-2
Sia SF, 2020, NATURE, V583, P834, DOI 10.1038/s41586-020-2342-5
Simmons G, 2005, P NATL ACAD SCI USA, V102, P11876, DOI 10.1073/pnas.0505577102
Sun J, 2020, CELL, V182, P734, DOI 10.1016/j.cell.2020.06.010
Temmam S, 2022, NATURE, V604, P330, DOI 10.1038/s41586-022-04532-4
van Aart AE, 2022, TRANSBOUND EMERG DIS, V69, P3001, DOI 10.1111/tbed.14173
van Boheemen S, 2012, MBIO, V3, DOI 10.1128/mBio.00473-12
Wacharapluesadee S, 2021, NAT COMMUN, V12, DOI 10.1038/s41467-021-21240-1
Walls AC, 2020, CELL, V181, P281, DOI [10.1016/j.cell.2020.02.058, 10.1016/j.cell.2020.11.032]
Wan YS, 2020, J VIROL, V94, DOI 10.1128/JVI.00127-20
Weiskopf D, 2020, SCI IMMUNOL, V5, DOI 10.1126/sciimmunol.abd2071
Wong LYR, 2020, STAR PROTOC, V1, DOI 10.1016/j.xpro.2020.100169
Worobey M, 2022, SCIENCE, V377, P951, DOI 10.1126/science.abp8715
Yan H, 2021, NAT ECOL EVOL, V5, P600, DOI 10.1038/s41559-021-01407-1
Zhang Y, 2001, MOL THER, V3, P697, DOI 10.1006/mthe.2001.0329
Zhou H, 2021, CELL, V184, P4380, DOI 10.1016/j.cell.2021.06.008
Zhou P., 2020, NATURE, V579, P270, DOI DOI 10.1038/s41586-020-2012-7
Zotos D, 2010, J EXP MED, V207, P365, DOI 10.1084/jem.20091777
NR 78
TC 2
Z9 2
U1 1
U2 2
PU PUBLIC LIBRARY SCIENCE
PI SAN FRANCISCO
PA 1160 BATTERY STREET, STE 100, SAN FRANCISCO, CA 94111 USA
SN 1553-7366
EI 1553-7374
J9 PLOS PATHOG
JI PLoS Pathog.
PD OCT
PY 2023

VL 19
IS 10
AR e1011728
DI 10.1371/journal.ppat.1011728
PG 26
WC Microbiology; Parasitology; Virology
WE Science Citation Index Expanded (SCI-EXPANDED)
SC Microbiology; Parasitology; Virology
GA W8LZ2
UT WOS:001094101200005
PM 37856551
OA gold, Green Published
DA 2024-10-21
ER

PT J
AU Fu, FY
   Shao, Q
   Wang, J
   Zhang, JJ
   Wang, ZF
   Ma, JJ
   Yan, YX
   Cheng, YQ
   Sun, JH
AF Fu, Feiyu
   Shao, Qi
   Wang, Jie
   Zhang, Jianjian
   Wang, Zhaofei
   Ma, Jingjiao
   Yan, Yaxian
   Cheng, Yuqiang
   Sun, Jianhe
TI Bat MAVS involved in antiviral innate immunity via regulating
IFN-beta
   production
SO DEVELOPMENTAL AND COMPARATIVE IMMUNOLOGY
LA English
DT Article
DE Bat; MAVS; IFN; Innate immunity; RNA virus
ID I-LIKE RECEPTORS; PATTERN-RECOGNITION RECEPTORS; INTERFERON;
ACTIVATION;
   RESPONSES; VIRUSES; PATHWAY
AB Mitochondrial antiviral signaling protein (MAVS) is an essential
articulatory protein in immune responses against most RNA viruses.
Whether bats, the natural hosts of numerous zoonotic RNA viruses,
utilize conserved signaling pathways involving MAVS-mediated
interferon (IFN) responses remains elusive. In this study, we
performed the cloning and functional analysis of bat MAVS (BatMAVS).
Amino acid sequence analysis revealed that BatMAVS was poorly
conserved among species and evolutionarily closer to other mammals.
Overexpression of BatMAVS significantly inhibited the replication of
green fluorescent protein (GFP)-tagged VSV (VSV-GFP) and GFP-tagged

Newcastle disease virus (NDV) (NDV-GFP) by activating the type I IFN pathway, and its expression at the transcriptional level was upregulated at the late stage of VSV-GFP infection. We further demonstrated that the CARD_2 and TM domains occupy a large proportion in the ability of BatMAVS to activate IFN-& beta;. These results suggest that BatMAVS acts as an important regulatory molecule in IFN-induction and anti-RNA viruses in bats.
C1 [Fu, Feiyu; Shao, Qi; Wang, Jie; Zhang, Jianjian; Wang, Zhaofei; Ma, Jingjiao; Yan, Yaxian; Cheng, Yuqiang; Sun, Jianhe] Shanghai Jiao Tong Univ, Sch Agr & Biol, Shanghai Key Lab Vet Biotechnol, Agr Minist,Key Lab Urban Agr South, Shanghai 200240, Peoples R China.
C3 Shanghai Jiao Tong University
RP Cheng, YQ; Sun, JH (corresponding author), Shanghai Jiao Tong Univ, Sch Agr & Biol, Shanghai Key Lab Vet Biotechnol, Agr Minist,Key Lab Urban Agr South, Shanghai 200240, Peoples R China.
EM wyycyq@sjtu.edu.cn; sunjhe@sjtu.edu.cn
RI li, liu/JXN-7328-2024; zhang, jianjian/AAI-1432-2019; xie, jing/KDO-9486-2024; SUN, Ye/KBC-8159-2024
FU National Natural Science Foundation of China [32072865, 32072864];
Shanghai Natural Science foundation [20ZR1425100]; State Key Laboratory of Veterinary Biotechnology Foundation [SKLVBF202107]
FX This work was supported by the National Natural Science Foundation of China (32072865 , 32072864) , the Shanghai Natural Science foundation (20ZR1425100) , and State Key Laboratory of Veterinary Biotechnology Foundation (SKLVBF202107) .
CR Aicher SM, 2022, J VIROL, V96, DOI 10.1128/jvi.00608-22
Allen LC, 2009, J COMP PHYSIOL B, V179, P315, DOI 10.1007/s00360-008-0315-3
Baker ML, 2013, ZOONOSES PUBLIC HLTH, V60, P104, DOI 10.1111/j.1863-2378.2012.01528.x
Brisse M, 2019, FRONT IMMUNOL, V10, DOI 10.3389/fimmu.2019.01586
Brzózka K, 2006, J VIROL, V80, P2675, DOI 10.1128/JVI.80.6.2675-2683.2006
Chen YQ, 2021, FRONT MICROBIOL, V12, DOI 10.3389/fmicb.2021.744348
Cheng YQ, 2015, J IMMUNOL, V195, P3922, DOI 10.4049/jimmunol.1500638
Chothe SK, 2017, SCI REP-UK, V7, DOI 10.1038/s41598-017-00793-6
Cibulski SP, 2021, ARCH VIROL, V166, P207, DOI 10.1007/s00705-020-04825-x
Clayton E, 2020, FRONT CELL INFECT MI, V10, DOI 10.3389/fcimb.2020.527921
Cowled C, 2012, DEV COMP IMMUNOL, V36, P657, DOI 10.1016/j.dci.2011.11.008
Han HJ, 2015, VIRUS RES, V205, P1, DOI 10.1016/j.virusres.2015.05.006
Hou FJ, 2011, CELL, V146, P448, DOI 10.1016/j.cell.2011.06.041
Irving AT, 2021, NATURE, V589, P363, DOI 10.1038/s41586-020-03128-0

Jablonska A, 2014, POSTEP HIG MED DOSW, V68, P541, DOI 10.5604/17322693.1102281
Kato H, 2011, IMMUNOL REV, V243, P91, DOI 10.1111/j.1600-065X.2011.01052.x
Kawai T, 2010, NAT IMMUNOL, V11, P373, DOI 10.1038/ni.1863
Kumagai Y, 2010, J ALLERGY CLIN IMMUN, V125, P985, DOI 10.1016/j.jaci.2010.01.058
Lee JH, 2019, J INTERF CYTOK RES, V39, P450, DOI 10.1089/jir.2019.0015
Leung DW, 2019, J MOL BIOL, V431, P4281, DOI 10.1016/j.jmb.2019.06.002
Li YZ, 2019, MBIO, V10, DOI 10.1128/mBio.02414-19
Lin HH, 2022, MICROBIOL IMMUNOL, V66, P97, DOI 10.1111/1348-0421.12952
Niu QN, 2019, FRONT IMMUNOL, V10, DOI 10.3389/fimmu.2019.00822
Onomoto K, 2021, CELL MOL IMMUNOL, V18, P539, DOI 10.1038/s41423-020-00602-7
Randall RE, 2008, J GEN VIROL, V89, P1, DOI 10.1099/vir.0.83391-0
Ren ZH, 2020, FRONT IMMUNOL, V11, DOI 10.3389/fimmu.2020.01030
Rodhain E., 2015, Bulletin de la Societe de Pathologie Exotique, V108, P272, DOI 10.1007/s13149-015-0448-z
Sarkis S, 2018, DEV COMP IMMUNOL, V81, P1, DOI 10.1016/j.dci.2017.10.023
Seth RB, 2005, CELL, V122, P669, DOI 10.1016/j.cell.2005.08.012
Slater T, 2018, VIROL J, V15, DOI 10.1186/s12985-018-0979-6
Smith I, 2013, CURR OPIN VIROL, V3, P84, DOI 10.1016/j.coviro.2012.11.006
Sun YJ, 2019, DEV COMP IMMUNOL, V93, P58, DOI 10.1016/j.dci.2018.12.006
Thompson MR, 2011, VIRUSES-BASEL, V3, P920, DOI 10.3390/v3060920
Wu HX, 2016, IMMUNOL RES, V64, P82, DOI 10.1007/s12026-015-8682-9
Xie JZ, 2018, CELL HOST MICROBE, V23, P297, DOI 10.1016/j.chom.2018.01.006
Xu Q, 2021, FRONT MED-PRC, V15, P333, DOI 10.1007/s11684-020-0776-7
Zhang GJ, 2013, SCIENCE, V339, P456, DOI 10.1126/science.1230835
Zhang J, 2014, FISH SHELLFISH IMMUN, V41, P222, DOI 10.1016/j.fsi.2014.09.002
Zhao Y, 2019, J VIROL, V93, DOI 10.1128/JVI.01085-19
Zhou P, 2014, PLOS ONE, V9, DOI 10.1371/journal.pone.0103875
Zhou P, 2013, DEV COMP IMMUNOL, V40, P240, DOI 10.1016/j.dci.2013.03.006
NR 41
TC 0
Z9 0
U1 2
U2 6
PU ELSEVIER SCI LTD
PI OXFORD
PA THE BOULEVARD, LANGFORD LANE, KIDLINGTON, OXFORD OX5 1GB, OXON, ENGLAND
SN 0145-305X
EI 1879-0089
J9 DEV COMP IMMUNOL

JI Dev. Comp. Immunol.
PD SEP
PY 2023
VL 146
AR 104724
DI 10.1016/j.dci.2023.104724
EA MAY 2023
PG 9
WC Fisheries; Immunology; Veterinary Sciences; Zoology
WE Science Citation Index Expanded (SCI-EXPANDED)
SC Fisheries; Immunology; Veterinary Sciences; Zoology
GA J9VG4
UT WOS:001013024700001
PM 37178925
DA 2024-10-21
ER

PT J
AU Toshkova, N
Zhelyazkova, V
Justesen, S
Dimitrov, JD
AF Toshkova, Nia
Zhelyazkova, Violeta
Justesen, Sune
Dimitrov, Jordan D.
TI Conservative pattern of interaction of bat and human IgG antibodies with
FcRn
SO DEVELOPMENTAL AND COMPARATIVE IMMUNOLOGY
LA English
DT Article
DE Immunoglobulins; IgG; Neonatal Fc receptor; Bat immunity; Surface plasmon resonance
ID RECEPTOR FCRN; BINDING
AB Recently, numerous studies report bats as reservoirs of emerging pathogens with little to no signs of infections. This is thought to be connected to the unique immune system of bats, which remains poorly characterized. Despite the physiological importance of the Neonatal Fc receptor (FcRn) in the homeostasis of IgG antibodies, it is unclear how its functional activity is evolutionary conservative among mammals, and so is the case for bats. Using surface plasmon resonance-based technology, we tested the interactions of IgG antibodies isolated from three bat species with recombinant human and mouse FcRn. Our data show that IgG from the studied bat species binds to both human and mouse FcRn, albeit with distinct affinities. Importantly, the binding pattern of bat IgG is similar to human IgG. This confirms the conservative nature of IgG-FcRn interaction and highlights the importance of FcRn IgG salvaging system in bats.
C1 [Toshkova, Nia; Zhelyazkova, Violeta] Bulgarian Acad Sci, Natl Museum Nat Hist, 1 Tsar Osvoboditel Blvd, Sofia 1000, Bulgaria.
[Toshkova, Nia] Bulgarian Acad Sci, Inst Biodivers & Ecosyst Res, 1 Tsar Osvoboditel Blvd, Sofia 1000, Bulgaria.
[Zhelyazkova, Violeta] Univ Paris, Sorbonne Univ, Ctr Rech

Cordeliers, INSERM, F-75006 Paris, France.
[Justesen, Sune] Immunitrack Aps, Lersoe Pk 42, DK-2100 Copenhagen East, Denmark.
[Dimitrov, Jordan D.] Ctr Rech Cordeliers, INSERM, UMRS 1138, F-75006 Paris, France.
[Toshkova, Nia] Bulgarian Acad Sci, Natl Museum Nat Hist, Sofia 1000, Bulgaria.
C3 Bulgarian Academy of Sciences; Bulgarian Academy of Sciences; Institut
National de la Sante et de la Recherche Medicale (Inserm); Sorbonne
Universite; Universite Paris Cite; Sorbonne Universite; Universite Paris
Cite; Institut National de la Sante et de la Recherche Medicale
(Inserm); Bulgarian Academy of Sciences
RP Dimitrov, JD (corresponding author), Ctr Rech Cordeliers, INSERM, UMRS 1138, F-75006 Paris, France.; Toshkova, N (corresponding author), Bulgarian Acad Sci, Natl Museum Nat Hist, Sofia 1000, Bulgaria.
EM niyatoshkova@gmail.com; jordan.dimitrov@sorbonne-universite.fr
RI Dimitrov, Jordan/N-2141-2014; Zhelyazkova, Violeta/AET-7798-2022
OI Dimitrov, Jordan/0000-0001-8536-8995; Zhelyazkova,
Violeta/0000-0001-9381-2208
CR Abdiche YN, 2015, MABS-AUSTIN, V7, P331, DOI 10.1080/19420862.2015.1008353
Ahn M, 2019, NAT MICROBIOL, V4, P789, DOI 10.1038/s41564-019-0371-3
Ahn M, 2016, SCI REP-UK, V6, DOI 10.1038/srep21722
Andersen JT, 2008, FEBS J, V275, P4097, DOI 10.1111/j.1742-4658.2008.06551.x
Andersen JT, 2008, J IMMUNOL METHODS, V331, P39, DOI 10.1016/j.jim.2007.11.003
Avery LB, 2016, MABS-AUSTIN, V8, P1064, DOI 10.1080/19420862.2016.1193660
Baker K, 2013, IMMUNITY, V39, P1095, DOI 10.1016/j.immuni.2013.11.003
Baker ML, 2010, IMMUNOGENETICS, V62, P173, DOI 10.1007/s00251-010-0425-4
Banerjee A, 2020, FRONT IMMUNOL, V11, DOI 10.3389/fimmu.2020.00026
Bateman A, 2021, NUCLEIC ACIDS RES, V49, pD480, DOI 10.1093/nar/gkaa1100
Bratsch S, 2011, DEV COMP IMMUNOL, V35, P421, DOI 10.1016/j.dci.2010.06.004
Butler JE, 2011, DEV COMP IMMUNOL, V35, P273, DOI 10.1016/j.dci.2010.08.011
CHAKRAVARTY AK, 1994, LYMPHOLOGY, V27, P97
Gamage AM, 2020, SCI REP-UK, V10, DOI 10.1038/s41598-019-57212-1
Goh G, 2020, P NATL ACAD SCI USA, V117, P28939, DOI 10.1073/pnas.2003352117
Gómez JMM, 2016, SCI REP-UK, V6, DOI 10.1038/srep37796
Grevys A, 2018, NAT COMMUN, V9, DOI 10.1038/s41467-018-03061-x
HATTEN BA, 1968, J IMMUNOL, V101, P141
Irving AT, 2021, NATURE, V589, P363, DOI 10.1038/

s41586-020-03128-0
Lemos APC, 2012, PLOS ONE, V7, DOI 10.1371/journal.pone.0028869
Letko M, 2020, NAT REV MICROBIOL, V18, P461, DOI 10.1038/s41579-020-0394-z
Lu LL, 2018, NAT REV IMMUNOL, V18, P46, DOI 10.1038/nri.2017.106
Pavlovich SS, 2018, CELL, V173, P1098, DOI 10.1016/j.cell.2018.03.070
Periasamy P, 2019, FRONT IMMUNOL, V10, DOI 10.3389/fimmu.2019.00489
Piche-Nicholas NM, 2018, MABS-AUSTIN, V10, P81, DOI 10.1080/19420862.2017.1389355
Roopenian DC, 2007, NAT REV IMMUNOL, V7, P715, DOI 10.1038/nri2155
Rossini S, 2020, J IMMUNOL, V205, P2850, DOI 10.4049/jimmunol.2000732
Schoch A, 2015, P NATL ACAD SCI USA, V112, P5997, DOI 10.1073/pnas.1408766112
Schuh AJ, 2019, J INFECT DIS, V219, P1716, DOI 10.1093/infdis/jiy733
Schuh AJ, 2017, SCI REP-UK, V7, DOI 10.1038/s41598-017-07824-2
Stapleton NM, 2015, IMMUNOL REV, V268, P253, DOI 10.1111/imr.12331
Sun Y, 2020, MABS-AUSTIN, V12, DOI 10.1080/19420862.2020.1802135
Vidarsson G, 2006, BLOOD, V108, P3573, DOI 10.1182/blood-2006-05-024539
Wang WR, 2011, DRUG METAB DISPOS, V39, P1469, DOI 10.1124/dmd.111.039453
Ward ES, 2009, ADV IMMUNOL, V103, P77, DOI 10.1016/S0065-2776(09)03004-1
Wimsatt J, 2005, J WILDLIFE DIS, V41, P87, DOI 10.7589/0090-3558-41.1.87
Xie JZ, 2018, CELL HOST MICROBE, V23, P297, DOI 10.1016/j.chom.2018.01.006
NR 37
TC 3
Z9 3
U1 1
U2 12
PU ELSEVIER SCI LTD
PI OXFORD
PA THE BOULEVARD, LANGFORD LANE, KIDLINGTON, OXFORD OX5 1GB, OXON, ENGLAND
SN 0145-305X
EI 1879-0089
J9 DEV COMP IMMUNOL
JI Dev. Comp. Immunol.
PD FEB
PY 2023
VL 139
AR 104579
DI 10.1016/j.dci.2022.104579
EA OCT 2022
PG 6
WC Fisheries; Immunology; Veterinary Sciences; Zoology

WE Science Citation Index Expanded (SCI-EXPANDED)
SC Fisheries; Immunology; Veterinary Sciences; Zoology
GA 5V6TK
UT WOS:000877360300002
PM 36272453
OA Green Submitted, Bronze
DA 2024-10-21
ER

PT J
AU Otálora-Ardila, A
Herrera, LG
Flores-Martínez, JJ
Welch, KC
AF Otalora-Ardila, Aida
Gerardo Herrera M, L.
Juan Flores-Martinez, Jose
Welch, Kenneth C., Jr.
TI The effect of short-term food restriction on the metabolic cost of the
acute phase response in the fish-eating <i>Myotis</i> (<i>Myotis vivesi</i>)
SO MAMMALIAN BIOLOGY
LA English
DT Article
DE Caloric cost; Food restriction; Innate immune response; Lipopolysaccharidae; Resting metabolic
ID CALORIE RESTRICTION; IMMUNE-RESPONSE; ECOLOGICAL IMMUNOLOGY; SICKNESS
BEHAVIOR; ENERGETIC COSTS; TRADE-OFFS; FEVER; PERFORMANCE; ACTIVATION;
SYSTEM
AB Food restriction affects the activation of the immune system although the metabolic cost associated with mounting such a response has rarely been examined except in model animals. Wild animals are constantly exposed to variations in the availability of food resources and they need to balance their energy budget to fight against pathogens. We examined the effect of food restriction in the fish eating Myotis (Myotis vivesi), a species of bat that experiences periods in which foraging is limited due to ambient conditions. We tested the hypothesis that acute food restriction (similar to 65% restriction for 1 night) would reduce the caloric response to lipopolysaccharidae (LPS) injection compared to bats fed ad libitum. We also measured a proxy for body temperature (T-skin) and expected reduced fever development when food intake was limited. Bats on the restricted diet had similar resting metabolic rate, total caloric cost and Tskin after the LPS challenge than when fed ad libitum. However, there was a delay in the metabolic and pyrogenic responses when bats were on the restricted diet. The effect of acute food restriction in delaying the hyperthermia development in fish eating Myotis might be of importance for its capacity to fight pathogens. Similar to other bats, the fish eating Myotis can fast for several consecutive days by entering torpor and future work is warranted to understand the effect of long periods of

food restriction on bat immune response. (C) 2016 Deutsche Gesellschaft fur Saugetierkunde. Published by Elsevier GmbH. All rights reserved.
C1 [Otalora-Ardila, Aida] Univ Nacl Autonoma Mexico, Inst Biol, Posgrad Ciencias Biol, Circuito Exterior S-N,Ciudad Univ, Mexico City 04510, DF, Mexico.
[Gerardo Herrera M, L.] Univ Nacl Autonoma Mexico, Inst Biol, Estac Biol Chamela, Apartado Postal 21, San Patricio 48980, Jalisco, Mexico.
[Juan Flores-Martinez, Jose] Univ Nacl Autonoma Mexico, Inst Biol, Dept Zool, Lab Sistemas Informac Geog, Circuito Exterior S-N,Ciudad Univ, Mexico City 04510, DF, Mexico.
[Welch, Kenneth C., Jr.] Univ Toronto Scarborough, Dept Biol Sci, 1265 Mil Trail, Toronto, ON M1C 1A4, Canada.
C3 Universidad Nacional Autonoma de Mexico; Universidad Nacional Autonoma
de Mexico; Universidad Nacional Autonoma de Mexico; University of Toronto; University Toronto Scarborough
RP Herrera, LG (corresponding author), Univ Nacl Autonoma Mexico, Inst Biol, Estac Biol Chamela, Apartado Postal 21, San Patricio 48980, Jalisco, Mexico.
EM aotalora@gmail.com; gherrera@ib.unam.mx; josef@ib.unam.mx; kwelch@utsc.utoronto.ca
RI Otálora, Aída/AAE-7280-2019; Welch, Kenneth/F-1276-2011
OI Welch, Kenneth/0000-0002-3283-6510; Herrera Montalvo, Luis Gerardo/0000-0001-9348-5040
FU Direction General de Asuntos del Personal Academic [PAPIIT] [IN202113];
Natural Sciences and Engineering Research Council of Canada Discovery
Grant [386466]; Consejo Nacional de Ciencia y Tecnologia (CONACYT)
FX This work was supported by Direction General de Asuntos del Personal
Academic [PAPIIT # IN202113] to LGHM, and by a Natural Sciences and
Engineering Research Council of Canada Discovery Grant [# 386466] to
KCW. AOA was supported by a scholarship from Consejo Nacional de Ciencia
y Tecnologia (CONACYT).
CR Berger J, 2013, J APPL BIOMED, V11, P1, DOI 10.2478/v10136-012-0029-4
Blatteis CM, 2003, J THERM BIOL, V28, P1, DOI 10.1016/S0306-4565(02)00034-7
Bonneaud C, 2003, AM NAT, V161, P367, DOI 10.1086/346134
Bouma HR, 2010, J LEUKOCYTE BIOL, V88, P619, DOI 10.1189/jlb.0310174
Buchanan JB, 2003, AM J PHYSIOL-REG I, V285, pR1165, DOI 10.1152/ajpregu.00238.2003
Burness G, 2010, J EXP BIOL, V213, P1386, DOI 10.1242/jeb.027011
Canale CI, 2011, FUNCT ECOL, V25, P557, DOI 10.1111/j.1365-2435.2010.01815.x
Carreno Ana Luisa, 2002, P14

Cutrera AP, 2010, J EXP BIOL, V213, P715, DOI 10.1242/jeb.037887
Demas GE, 2011, J ANIM ECOL, V80, P710, DOI 10.1111/j.1365-2656.2011.01813.x
French SS, 2009, INTEGR COMP BIOL, V49, P246, DOI 10.1093/icb/icp032
Geiser F, 1999, J COMP PHYSIOL B, V169, P133, DOI 10.1007/s003600050203
Gutiérrez JS, 2011, J AVIAN BIOL, V42, P259, DOI 10.1111/j.1600-048X.2011.05323.x
Inoue W, 2008, AM J PHYSIOL-REG I, V295, pR133, DOI 10.1152/ajpregu.90335.2008
Killpack TL, 2015, PHYSIOL BIOCHEM ZOOL, V88, P195, DOI 10.1086/680168
King MO, 2013, J EXP BIOL, V216, P2097, DOI 10.1242/jeb.079574
Kristan DM, 2008, AGE, V30, P147, DOI 10.1007/s11357-008-9056-1
Ksiazek A, 2012, PHYSIOL BIOCHEM ZOOL, V85, P51, DOI 10.1086/663696
Lee KA, 2005, OECOLOGIA, V145, P244, DOI 10.1007/s00442-005-0113-5
Lighton JRB, 2019, MEASURING METABOLIC RATES: A MANUAL FOR SCIENTISTS, 2ND EDITION, DOI 10.1093/oso/9780198830399.001.0001
Lilley TM, 2016, FRONT ZOOL, V13, DOI 10.1186/s12983-016-0143-3
MacDonald L, 2014, BRAIN BEHAV IMMUN, V40, P18, DOI 10.1016/j.bbi.2014.01.005
MacDonald L, 2012, PHYSIOL BEHAV, V107, P762, DOI 10.1016/j.physbeh.2012.06.009
MacDonald L, 2011, AM J PHYSIOL-REG I, V301, pR172, DOI 10.1152/ajpregu.00057.2011
Marais M, 2011, J THERM BIOL, V36, P116, DOI 10.1016/j.jtherbio.2010.12.004
Martin II LB, 2006, OECOLOGIA, V147, P565, DOI 10.1007/s00442-005-0314-y
Martin LB, 2006, FUNCT ECOL, V20, P630, DOI 10.1111/j.1365-2435.2006.01138.x
Moore MS, 2013, PLOS ONE, V8, DOI 10.1371/journal.pone.0058976
Moore MS, 2011, PLOS ONE, V6, DOI 10.1371/journal.pone.0027430
Munshi-South J, 2010, AGEING RES REV, V9, P12, DOI 10.1016/j.arr.2009.07.006
Nelson RJ, 1996, Q REV BIOL, V71, P511, DOI 10.1086/419555
Norris K, 2000, BEHAV ECOL, V11, P19, DOI 10.1093/beheco/11.1.19
Otalora-Ardila A, 2013, J MAMMAL, V94, P1102, DOI 10.1644/12-MAMM-A-281.1
Radler ME, 2014, BRAIN BEHAV IMMUN, V38, P13, DOI 10.1016/j.bbi.2013.11.014
Ricklefs RE, 2002, TRENDS ECOL EVOL, V17, P462, DOI 10.1016/S0169-5347(02)02578-8
Robertson LT, 2013, EXP GERONTOL, V48, P1043, DOI 10.1016/j.exger.2013.01.009
Salinas VBR, 2014, ACTA CHIROPTEROL, V16, P327, DOI 10.3161/150811014X687288
Speakman JR, 2010, J ANIM ECOL, V79, P726, DOI 10.1111/j.1365-2656.2010.01689.x
Twining CW, 2016, P NATL ACAD SCI USA, V113, P10920, DOI 10.1073/pnas.1603998113

Welch KC Jr, 2015, J EXP BIOL, V218, P1180, DOI 10.1242/
jeb.115964
Wilhelm D, 2007, COMP BIOCHEM PHYS C, V146, P214, DOI 10.1016/
j.cbpc.2006.11.015
Xu DL, 2010, COMP BIOCHEM PHYS A, V155, P25, DOI 10.1016/
j.cbpa.2009.09.003
Zeileis A., 2005, J. Stat. Softw, V6, P1, DOI [DOI 10.18637/
JSS.V014.I06, 10.18637/jss.v014.i06, 10.48550/arXiv.math/0505527,
DOI 10.48550/ARXIV.MATH/0505527]
Zysling DA, 2009, FUNCT ECOL, V23, P979, DOI 10.1111/
j.1365-2435.2009.01572.x
NR 44
TC 9
Z9 12
U1 2
U2 20
PU ELSEVIER GMBH
PI MUNICH
PA HACKERBRUCKE 6, 80335 MUNICH, GERMANY
SN 1616-5047
EI 1618-1476
J9 MAMM BIOL
JI Mamm. Biol.
PD JAN
PY 2017
VL 82
BP 41
EP 47
DI 10.1016/j.mambio.2016.11.002
PG 7
WC Zoology
WE Science Citation Index Expanded (SCI-EXPANDED)
SC Zoology
GA EK8TM
UT WOS:000394197500006
DA 2024-10-21
ER

PT J
AU Raiter, A
Rodionov, G
Novogrodsky, A
Hardy, B
AF Raiter, A
Rodionov, G
Novogrodsky, A
Hardy, B
TI CD4⁺ T lymphocytes as a primary cellular target for
BAT mAb
stimulation
SO INTERNATIONAL IMMUNOLOGY
LA English
DT Article
DE agonistic antibody; BAT receptors; IFN-gamma; immune stimulation;

T cell
   activation
ID MONOCLONAL-ANTIBODY; ANTITUMOR IMMUNITY; TUMOR-CELLS; REGRESSION;
   ACTIVATION; RESPONSES; MICE
AB BAT is a monoclonal antibody (mAb) produced against membranes of a human Burkitt lymphoma cell line (Daudi) that was selected for its ability to stimulate lymphocyte proliferation. BAT manifests anti-tumor properties in mice bearing a variety of murine tumors. BAT also induced regression of human tumors inoculated into SCID mice that had been engrafted with human lymphocytes, The anti-tumor activity of BAT was related to its immune stimulatory properties. Previous data indicated that T lymphocytes and NK cells mediate in vivo the anti-tumor activity. In order to define the primary target cell for BAT stimulatory activity, the in vitro stimulatory effect of BAT on purified lymphocyte subpopulations was investigated. Human CD4(+), CD8(+) T cells and CD56(+) NK cells were purified and their in vitro response to BAT was investigated, Results indicate that BAT selectively stimulated CD4(+) cells as assessed by proliferation and secretion of IFN-gamma, FAGS analysis has also revealed a selective increase in BAT antigen on CD4+ T cells that were cultured with BAT antibody. The effector cells that mediate BAT-induced tumor eradication may, however, be distinct from those that serve as the primary cellular target of the antibody, Cytokines such as IFN-gamma that are produced by CD4(+) cells may be involved in activation of additional cell types that may be involved in tumor destruction.
C1 Tel Aviv Univ, Sackler Sch Med, Rabin Med Ctr, Felsenstein Med Res Ctr, IL-49100 Petah Tiqwa, Israel.
C3 Rabin Medical Center; Tel Aviv University
RP Hardy, B (corresponding author), Tel Aviv Univ, Sackler Sch Med, Rabin Med Ctr, Felsenstein Med Res Ctr, Beilinson Campus, IL-49100 Petah Tiqwa, Israel.
CR HARDY B, 1994, CANCER RES, V54, P5793
   Hardy B, 1997, P NATL ACAD SCI USA, V94, P5756, DOI 10.1073/pnas.94.11.5756
   HARDY B, 1995, CANCER IMMUNOL IMMUN, V40, P376, DOI 10.1007/s002620050187
   Hardy Britta, 1997, Human Antibodies, V8, P95
   Kos FJ, 1996, IMMUNOL TODAY, V17, P174, DOI 10.1016/0167-5699(96)80616-5
   La Motte RN, 1998, J IMMUNOL, V161, P6552
   Li Q, 1999, CLIN CANCER RES, V5, P461
   Pardoll DM, 1998, CURR OPIN IMMUNOL, V10, P588, DOI 10.1016/S0952-7915(98)80228-8
   Sadanaga N, 1999, J IMMUNOTHER, V22, P315, DOI 10.1097/00002371-199907000-00005
   Schlom J, 1999, IMMUNOL REV, V170, P73, DOI 10.1111/j.1600-065X.1999.tb01330.x
NR 10
TC 9
Z9 13
U1 0
U2 1
PU OXFORD UNIV PRESS
PI OXFORD

PA GREAT CLARENDON ST, OXFORD OX2 6DP, ENGLAND
SN 0953-8178
J9 INT IMMUNOL
JI Int. Immunol.
PD NOV
PY 2000
VL 12
IS 11
BP 1623
EP 1628
DI 10.1093/intimm/12.11.1623
PG 6
WC Immunology
WE Science Citation Index Expanded (SCI-EXPANDED)
SC Immunology
GA 372ZP
UT WOS:000165264200015
PM 11058582
OA Bronze
DA 2024-10-21
ER

PT J
AU Vicente-Santos, A
   Lock, LR
   Allira, M
   Dyer, KE
   Dunsmore, A
   Tu, WH
   Volokhov, DV
   Herrera, C
   Lei, GS
   Relich, RF
   Janech, MG
   Bland, AM
   Simmons, NB
   Becker, DJ
AF Vicente-Santos, Amanda
   Lock, Lauren R.
   Allira, Meagan
   Dyer, Kristin E.
   Dunsmore, Annalise
   Tu, Weihong
   Volokhov, Dmitriy V.
   Herrera, Claudia
   Lei, Guang-Sheng
   Relich, Ryan F.
   Janech, Michael G.
   Bland, Alison M.
   Simmons, Nancy B.
   Becker, Daniel J.
TI Serum proteomics reveals a tolerant immune phenotype across multiple
   pathogen taxa in wild vampire bats

SO FRONTIERS IN IMMUNOLOGY
LA English
DT Article
DE Desmodus rotundus; bacteria; virus; protozoa; biomarker; shotgun proteomics
ID ACUTE CHAGAS-DISEASE; TRYPANOSOMA-CRUZI; DESMODUS-ROTUNDUS; ANTIVIRAL
RESPONSE; PREY PREFERENCE; VIRUS; INFECTION; MECHANISMS; THROMBOCYTOPENIA; AMPLIFICATION
AB Bats carry many zoonotic pathogens without showing pronounced pathology, with a few exceptions. The underlying immune tolerance mechanisms in bats remain poorly understood, although information-rich omics tools hold promise for identifying a wide range of immune markers and their relationship with infection. To evaluate the generality of immune responses to infection, we assessed the differences and similarities in serum proteomes of wild vampire bats (Desmodus rotundus) across infection status with five taxonomically distinct pathogens: bacteria (Bartonella spp., hemoplasmas), protozoa (Trypanosoma cruzi), and DNA (herpesviruses) and RNA (alphacoronaviruses) viruses. From 19 bats sampled in 2019 in Belize, we evaluated the up- and downregulated immune responses of infected versus uninfected individuals for each pathogen. Using a high-quality genome annotation for vampire bats, we identified 586 serum proteins but found no evidence for differential abundance nor differences in composition between infected and uninfected bats. However, using receiver operating characteristic curves, we identified four to 48 candidate biomarkers of infection depending on the pathogen, including seven overlapping biomarkers (DSG2, PCBP1, MGAM, APOA4, DPEP1, GOT1, and IGFALS). Enrichment analysis of these proteins revealed that our viral pathogens, but not the bacteria or protozoa studied, were associated with upregulation of extracellular and cytoplasmatic secretory vesicles (indicative of viral replication) and downregulation of complement activation and coagulation cascades. Additionally, herpesvirus infection elicited a downregulation of leukocyte-mediated immunity and defense response but an upregulation of an inflammatory and humoral immune response. In contrast to our two viral infections, we found downregulation of lipid and cholesterol homeostasis and metabolism with Bartonella spp. infection, of platelet-dense and secretory granules with hemoplasma infection, and of blood coagulation pathways with T. cruzi infection. Despite the small sample size, our results suggest that vampire bats have a similar suite of immune mechanisms for viruses distinct from responses to the other pathogen taxa, and we identify potential biomarkers that can expand our understanding of pathogenesis of these infections in bats. By applying a proteomic approach to a multi-pathogen system in wild animals, our study provides a distinct framework that could be expanded across bat species to increase our understanding of how bats tolerate pathogens.
C1 [Vicente-Santos, Amanda; Lock, Lauren R.; Allira, Meagan; Dyer, Kristin E.; Becker, Daniel J.] Univ Oklahoma, Sch Biol Sci, Norman, OK 73019 USA.
[Dunsmore, Annalise; Tu, Weihong; Herrera, Claudia] Tulane Univ, Sch Publ Hlth & Trop Med, Dept Trop Med, New Orleans, LA USA.

[Dunsmore, Annalise; Tu, Weihong; Herrera, Claudia] Tulane Univ, Vector Borne & Infect Dis Res Ctr, New Orleans, LA USA.
[Volokhov, Dmitriy V.] US FDA, Ctr Biol Evaluat & Res, Silver Spring, MD USA.
[Lei, Guang-Sheng; Relich, Ryan F.] Indiana Univ Sch Med, Dept Pathol & Lab Med, Indianapolis, IN USA.
[Janech, Michael G.; Bland, Alison M.] Hollings Marine Lab, Charleston, SC USA.
[Janech, Michael G.; Bland, Alison M.] Coll Charleston, Dept Biol, Charleston, SC USA.
[Simmons, Nancy B.] Amer Museum Nat Hist, Dept Mammal, Div Vertebrate Zool, New York, NY USA.
C3 University of Oklahoma System; University of Oklahoma - Norman; Tulane
University; Tulane University; US Food & Drug Administration (FDA);
Indiana University System; Indiana University Bloomington; College of
Charleston; American Museum of Natural History (AMNH)
RP Vicente-Santos, A (corresponding author), Univ Oklahoma, Sch Biol Sci, Norman, OK 73019 USA.
EM amanda.vicente@ou.edu
RI janech, michael/AAA-1011-2019; Lock, Lauren/LHA-6905-2024;
Vicente-Santos, Amanda/GQI-4061-2022
FU National Geographic Society10.13039/100006363
FX We thank Mark Howells and the staff of the Lamanai Field Research Center
for assistance with field logistics and permits in Belize. We are
grateful to Benjamin Neely for his support in the proteomic analysis and
feedback on the manuscript. Finally, we thank Konstantin Chumakov for
laboratory support and three reviewers for their feedback on this
manuscript.
CR Ahn M, 2019, NAT MICROBIOL, V4, P789, DOI 10.1038/s41564-019-0371-3
Ahn M, 2016, SCI REP-UK, V6, DOI 10.1038/srep21722
Albert-Bayo M, 2019, CELLS-BASEL, V8, DOI 10.3390/cells8020135
Alenquer M, 2015, VIRUSES-BASEL, V7, P5066, DOI 10.3390/v7092862
Almeida PE, 2012, PPAR RES, V2012, DOI 10.1155/2012/383829
Amman BR, 2015, J WILDLIFE DIS, V51, P113, DOI 10.7589/2014-08-198
Anderson NL, 2002, MOL CELL PROTEOMICS, V1, P845, DOI 10.1074/mcp.R200007-MCP200
Ansa-Addo EA, 2020, SCI ADV, V6, DOI 10.1126/sciadv.aaz3865
Ansa-Addo EA, 2019, J IMMUNOL, V202
Anthony SJ, 2015, NAT COMMUN, V6, DOI 10.1038/ncomms9147
Bai Y, 2016, J WILDLIFE DIS, V52, P844, DOI 10.7589/2016-01-015
Baker ML, 2013, ZOONOSES PUBLIC HLTH, V60, P104, DOI 10.1111/j.1863-2378.2012.01528.x
Banerjee A, 2020, FRONT IMMUNOL, V11, DOI 10.3389/fimmu.2020.00026
Battcock SA, 2006, J VIROL, V80, P4422, DOI 10.1128/JVI.80.9.4422-4430.2006

Becker DJ, 2022, FRONT VIROL-LAUSANNE, V2, DOI 10.3389/fviro.2022.862961
Becker DJ, 2021, TRANSBOUND EMERG DIS, V68, P870, DOI 10.1111/tbed.13754
Becker DJ, 2020, MOL ECOL, V29, P1534, DOI 10.1111/mec.15422
Becker DJ, 2018, PLOS NEGLECT TROP D, V12, DOI 10.1371/journal.pntd.0006786
Becker DJ, 2018, PHILOS T R SOC B, V373, DOI 10.1098/rstb.2017.0089
BENJAMINI Y, 1995, J R STAT SOC B, V57, P289, DOI 10.1111/j.2517-6161.1995.tb02031.x
Bergner LM, 2021, ZOONOSES PUBLIC HLTH, V68, P271, DOI 10.1111/zph.12808
Bergner LM, 2020, MOL ECOL, V29, P26, DOI 10.1111/mec.15250
Blanc M, 2011, PLOS BIOL, V9, DOI 10.1371/journal.pbio.1000598
Bobrowiec PED, 2015, J MAMMAL, V96, P54, DOI 10.1093/jmammal/gyu002
Bonnet J, 2019, J PROTEOMICS, V196, P150, DOI 10.1016/j.jprot.2018.11.005
Brook CE, 2015, TRENDS MICROBIOL, V23, P172, DOI 10.1016/j.tim.2014.12.004
CALLOW KA, 1990, EPIDEMIOL INFECT, V105, P435, DOI 10.1017/S0950268800048019
Camacho C, 2009, BMC BIOINFORMATICS, V10, DOI 10.1186/1471-2105-10-421
Canani Roberto Berni, 2011, Curr Gastroenterol Rep, V13, P257, DOI 10.1007/s11894-011-0188-6
Cano-Monreal GL, 2009, VIROLOGY, V392, P137, DOI 10.1016/j.virol.2009.06.051
Cardoso J.E., 1980, Memorias do Instituto Oswaldo Cruz, V75, P97, DOI 10.1590/S0074-02761980000200009
Chagas C., 1909, Memorias do Instituto Oswaldo Cruz, V1
Choudhury SR, 2019, CELL, V178, P1205, DOI 10.1016/j.cell.2019.07.017
Colunga-Salas P, 2021, FASCINAT LIFE SCI, P331, DOI 10.1007/978-3-030-54727-1_20
Costantini D, 2023, J EXP BIOL, V226, DOI 10.1242/jeb.246332
Cox D, 2011, J THROMB HAEMOST, V9, P1097, DOI 10.1111/j.1538-7836.2011.04264.x
Crump CA, 2007, J VIROL, V81, P7380, DOI 10.1128/JVI.00222-07
Dahlbäck B, 2016, INT J LAB HEMATOL, V38, P4, DOI 10.1111/ijlh.12508
DeAnglis IK., 2023, bioRxiv, V2023, P0522541709, DOI [10.1101/2023.05.22.541709, DOI 10.1101/2023.05.22.541709]
DeDiego ML, 2014, J VIROL, V88, P913, DOI 10.1128/JVI.02576-13
Desaire H, 2022, J PROTEOME RES, V21, P2071, DOI 10.1021/acs.jproteome.2c00117
Descloux E, 2021, CLIN INFECT DIS, V73, pE1445, DOI 10.1093/cid/ciaa1648
Ding XM, 2016, PLOS ONE, V11, DOI 10.1371/journal.pone.0156017
Dixon P, 2003, J VEG SCI, V14, P927, DOI 10.1111/j.1654-1103.2003.tb02228.x
Dunn C, 2009, GASTROENTEROLOGY, V137, P1289, DOI 10.1053/j.gastro.2009.06.054

DuShane JK, 2019, INT J MOL SCI, V20, DOI 10.3390/ijms20143427
Ericson JA, 2014, PLOS ONE, V9, DOI 10.1371/journal.pone.0108553
Filippakis H, 2010, BBA-MOL CELL RES, V1803, P777, DOI 10.1016/j.bbamcr.2010.03.007
Flores M, 2015, J IMMUNOL, V194, P4240, DOI 10.4049/jimmunol.1401296
Green R, 2017, NAT REV DIS PRIMERS, V3, DOI 10.1038/nrdp.2017.40
Griffiths ME, 2020, NAT COMMUN, V11, DOI 10.1038/s41467-020-19832-4
Gu J, 2007, AM J PATHOL, V170, P1136, DOI 10.2353/ajpath.2007.061088
Ha M, 2019, ANIM CELLS SYST, V23, P112, DOI 10.1080/19768354.2019.1583126
Halpin K, 2011, AM J TROP MED HYG, V85, P946, DOI 10.4269/ajtmh.2011.10-0567
He QY, 2003, PROTEOMICS, V3, P666, DOI 10.1002/pmic.200300394
Hecht-Höger AM, 2020, MOL ECOL, V29, P1745, DOI 10.1111/mec.15437
Holzmuller P, 2010, EXPERT REV PROTEOMIC, V7, P113, DOI 10.1586/EPR.09.92
Irving AT, 2021, NATURE, V589, P363, DOI 10.1038/s41586-020-03128-0
Johansen MD, 2019, MICROB PATHOGENESIS, V130, P44, DOI 10.1016/j.micpath.2019.02.031
Kobayashi T, 2019, SCI REP-UK, V9, DOI 10.1038/s41598-018-37224-z
Koczera P, 2016, INT J MOL SCI, V17, DOI 10.3390/ijms17081278
Kolberg Liis, 2020, F1000Res, V9, DOI 10.12688/f1000research.24956.1
Kolde R., 2015, R Package, V1, P790, DOI DOI 10.32614/CRAN.PACKAGE.PHEATMAP
Krishnamachary B, 2021, J EXTRACELL VESICLES, V10, DOI 10.1002/jev2.12117
Lau SKP, 2013, J GEN VIROL, V94, P2679, DOI 10.1099/vir.0.055533-0
Lazar C, 2016, J PROTEOME RES, V15, P1116, DOI 10.1021/acs.jproteome.5b00981
Li M, 2012, PROTEOMICS, V12, P2203, DOI 10.1002/pmic.201100376
Mallick P, 2007, NAT BIOTECHNOL, V25, P125, DOI 10.1038/nbt1275
Mandl JN, 2018, FRONT IMMUNOL, V9, DOI 10.3389/fimmu.2018.02112
Mandrekar JN, 2010, J THORAC ONCOL, V5, P1315, DOI 10.1097/JTO.0b013e3181ec173d
Marcondes MCG, 2000, MICROBES INFECT, V2, P347, DOI 10.1016/S1286-4579(00)00333-6
de los Reyes-García AM, 2019, BIOMED REP, V10, P195, DOI 10.3892/br.2019.1187
Minguela A, 2021, BRIT J HAEMATOL, V195, P743, DOI 10.1111/bjh.17828
Santillán DDM, 2021, MOL ECOL, V30, P6449, DOI 10.1111/mec.16027
Morrison J, 2014, J VIROL, V88, P10556, DOI 10.1128/JVI.00570-14
MOSER DR, 1989, J CLIN MICROBIOL, V27, P1477, DOI 10.1128/JCM.27.7.1477-1482.1989
Mou F, 2008, J VIROL, V82, P8094, DOI 10.1128/JVI.00874-08
Murtagh F, 2014, J CLASSIF, V31, P274, DOI 10.1007/s00357-014-9161-z
Neely BA, 2021, J PROTEOME RES, V20, P2547, DOI 10.1021/

acs.jproteome.0c00995
Ni GX, 2018, PLOS PATHOG, V14, DOI 10.1371/journal.ppat.1007148
Noser JA, 2007, MOL THER, V15, P1531, DOI 10.1038/sj.mt.6300193
NOSSAL GJV, 1983, ANNU REV IMMUNOL, V1, P33, DOI 10.1146/annurev.iy.01.040183.000341
Park J, 2021, SCI REP-UK, V11, DOI 10.1038/s41598-021-90250-2
Råberg L, 2009, PHILOS T R SOC B, V364, P37, DOI 10.1098/rstb.2008.0184
Raudvere U, 2019, NUCLEIC ACIDS RES, V47, pW191, DOI 10.1093/nar/gkz369
Romo MR, 2016, IMMUNOLOGY, V148, P125, DOI 10.1111/imm.12597
Robin X, 2011, BMC BIOINFORMATICS, V12, DOI 10.1186/1471-2105-12-77
Sager G, 2018, VIRUSES-BASEL, V10, DOI 10.3390/v10100559
Sampson DL, 2017, SCI REP-UK, V7, DOI 10.1038/s41598-017-02325-8
Schwartz L, 2018, VACCINES-BASEL, V6, DOI 10.3390/vaccines6040076
Shi TT, 2022, HELIYON, V8, DOI 10.1016/j.heliyon.2022.e10807
SHIKANAIYASUDA MA, 1990, REV INST MED TROP SP, V32, P16, DOI 10.1590/S0036-46651990000100004
Streicker DG, 2016, J APPL ECOL, V53, P1280, DOI 10.1111/1365-2664.12690
STURM NR, 1989, MOL BIOCHEM PARASIT, V33, P205, DOI 10.1016/0166-6851(89)90082-0
Subudhi S, 2019, VIRUSES-BASEL, V11, DOI 10.3390/v11020192
Subudhi S, 2018, VIROLOGY, V516, P227, DOI 10.1016/j.virol.2018.01.024
Szentivanyi T, 2023, TRANSBOUND EMERG DIS, V2023, DOI 10.1155/2023/9285855
Tanigawa K, 2021, INT J MOL SCI, V22, DOI 10.3390/ijms22147597
Thirunavukkarasu S, 2014, VET IMMUNOL IMMUNOP, V160, P147, DOI 10.1016/j.vetimm.2014.04.002
Tian WM, 2020, NAT COMMUN, V11, DOI 10.1038/s41467-020-19706-9
Tribulatti MV, 2005, INFECT IMMUN, V73, P201, DOI 10.1128/IAI.73.1.201-207.2005
Uhlén M, 2019, SCI SIGNAL, V12, DOI 10.1126/scisignal.aaz0274
Vandamme TF, 2014, J PHARM BIOALLIED SC, V6, P2, DOI 10.4103/0975-7406.124301
Veikkolainen V, 2014, EMERG INFECT DIS, V20, P960, DOI 10.3201/eid2006.130956
Volokhov DV, 2017, EPIDEMIOL INFECT, V145, P3154, DOI 10.1017/S095026881700231X
Wang HJ, 2011, NAT MED, V17, P96, DOI 10.1038/nm.2270
Wang LF, 2021, NAT REV IMMUNOL, V21, P269, DOI 10.1038/s41577-021-00523-0
Wang N, 2018, J INFLAMM-LOND, V15, DOI 10.1186/s12950-017-0178-z
Weisshaar N., 2018, Proc Cancer-Immuno-Oncol, V1, P5859, DOI [10.18416/CIO.2018.1810040, DOI 10.18416/CIO.2018.1810040]
Wenk MR, 2006, FEBS LETT, V580, P5541, DOI 10.1016/j.febslet.2006.07.007
White DW, 2012, IMMUNOL REV, V245, P189, DOI 10.1111/j.1600-065X.2011.01074.x
Wilkinson GS, 2021, NAT COMMUN, V12, DOI 10.1038/s41467-021-21900-2
Williamson MM, 2000, J COMP PATHOL, V122, P201, DOI 10.1053/

jcpa.1999.0364
   Woon AP, 2020, PLOS PATHOG, V16, DOI 10.1371/journal.ppat.1008412
   Wray AK, 2016, ECOHEALTH, V13, P761, DOI 10.1007/s10393-016-1183-z
   Xie JZ, 2018, CELL HOST MICROBE, V23, P297, DOI 10.1016/j.chom.2018.01.006
   Xu G, 2021, CELL MOL IMMUNOL, V18, P1587, DOI 10.1038/s41423-021-00649-0
   Xu W, 2023, CELL REP, V42, DOI 10.1016/j.celrep.2022.111987
   Young IS, 2001, J CLIN PATHOL, V54, P176, DOI 10.1136/jcp.54.3.176
   Yulis M, 2018, CELL DEATH DIS, V9, DOI 10.1038/s41419-018-0380-9
   Mendoza MLZ, 2018, NAT ECOL EVOL, V2, P659, DOI 10.1038/s41559-018-0476-8
   Zhang GJ, 2013, SCIENCE, V339, P456, DOI 10.1126/science.1230835
   Zhang N, 2019, J EXP MED, V216, P1291, DOI 10.1084/jem.20182024
   Zhang TP, 2022, FRONT IMMUNOL, V13, DOI 10.3389/fimmu.2022.947897
   Zhou JF, 2013, NATURE, V499, P500, DOI 10.1038/nature12379
   Zhou P, 2016, P NATL ACAD SCI USA, V113, P2696, DOI 10.1073/pnas.1518240113
   Zhou P, 2014, PLOS ONE, V9, DOI 10.1371/journal.pone.0103875
   Zhou X, 2012, CELL RES, V22, P717, DOI 10.1038/cr.2011.184
   Zou FG, 2018, FRONT IMMUNOL, V9, DOI 10.3389/fimmu.2018.01606
NR 129
TC 2
Z9 2
U1 5
U2 9
PU FRONTIERS MEDIA SA
PI LAUSANNE
PA AVENUE DU TRIBUNAL FEDERAL 34, LAUSANNE, CH-1015, SWITZERLAND
SN 1664-3224
J9 FRONT IMMUNOL
JI Front. Immunol.
PD DEC 12
PY 2023
VL 14
AR 1281732
DI 10.3389/fimmu.2023.1281732
PG 14
WC Immunology
WE Science Citation Index Expanded (SCI-EXPANDED)
SC Immunology
GA DE9M0
UT WOS:001130471100001
PM 38193073
OA gold
DA 2024-10-21
ER

PT J
AU Korine, C
   Pilosof, S
   Gross, A

Morales-Malacara, JB
Krasnov, BR
AF Korine, Carmi
Pilosof, Shai
Gross, Amit
Morales-Malacara, Juan B.
Krasnov, Boris R.
TI The effect of water contamination and host-related factors on ectoparasite load in an insectivorous bat
SO PARASITOLOGY RESEARCH
LA English
DT Article
DE Desert; Bats; Ectoparasites; Mites; Cimicid; Water quality
ID PIPISTRELLE BATS; IMMUNE-RESPONSE; URBAN ADAPTER; FLIES DIPTERA; PARASITE; SEX; CHIROPTERA; INFECTION; COMMUNITY; IMMUNOCOMPETENCE
AB We examined the effects of sex, age, and reproductive state of the insectivorous bat Pipistrellus kuhlii on the abundance and prevalence of arthropod ectoparasites (Macronyssidae and Cimicidae) in habitats with either sewage-polluted or natural bodies of water, in the Negev Desert, Israel. We chose water pollution as an environmental factor because of the importance of water availability in desert environments, particularly for P. kuhlii, which needs to drink on a daily basis. We predicted that parasite infestation rates would be affected by both environment and demographic cohort of the host. We found that female bats in the polluted site harbored significantly more mites than female bats in the natural site and that juveniles in the polluted site harbored significantly more cimicid individuals than juveniles in the natural site. We further found that age and sex (host-related factors) affected ectoparasite prevalence and intensity (i.e., the abundance of parasites) in the polluted site. Our results may suggest that the interaction between host-related and environment-related factors affected parasite infestations, with females and young bats being more susceptible to ectoparasites when foraging over polluted water. This effect may be particularly important for bats that must drink or forage above water for other wildlife that depend on drinking water for survival.
C1 [Korine, Carmi; Pilosof, Shai; Krasnov, Boris R.] Ben Gurion Univ Negev, Jacob Blaustein Inst Desert Res, Swiss Inst Dryland Environm & Energy Res, Mitrani Dept Desert Ecol, Sede Boqer Campus, IL-8499000 Midreshet Ben Gurion, Beersheba, Israel.
[Gross, Amit] Ben Gurion Univ Negev, Jacob Blaustein Inst Desert Res, Zuckerberg Inst Water Res, Dept Environm Hydrol & Microbiol, Sede Boqer Campus, IL-8499000 Midreshet Ben Gurion, Beersheba, Israel.
[Morales-Malacara, Juan B.] Univ Nacl Autonoma Mexico, Fac Ciencias, Unidad Multidisciplinaria Docencia & Invest, Lab Espeleobiol & Acarol, Campus Juriquilla,Blvd Juriquilla 3001, Queretaro 76230, Mexico.
C3 Ben Gurion University; Ben Gurion University; Universidad Nacional
Autonoma de Mexico
RP Korine, C (corresponding author), Ben Gurion Univ Negev, Jacob Blaustein Inst Desert Res, Swiss Inst Dryland Environm & Energy Res, Mitrani Dept Desert Ecol, Sede Boqer Campus, IL-8499000 Midreshet

Ben Gurion, Beersheba, Israel.
EM ckorine@bgu.ac.il
RI Krasnov, Boris/F-2234-2012; Korine, Carmi/F-4116-2012; Pilosof, Shai/AAX-8055-2020; GROSS, AMIT/F-1464-2012; Morales-Malacara, Juan/AAN-2929-2020
OI Morales-Malacara, Juan B./0000-0002-9828-5176; Gross, Amit/0000-0002-6223-7084
FU Israeli Ministry of Science and Technology
FX We thank Julie Shapiro, Marcela Gallero, Dr. Munoz-Garcia, and Laura Del Castillo for their technical assistance in the field and the laboratory. We also thank the reviewers for their helpful comments. This study was supported by Israeli Ministry of Science and Technology (to CK and AG). This is publication no. 936 of the Mitrani Department of Desert Ecology.
CR AKAIKE H, 1974, IEEE T AUTOMAT CONTR, VAC19, P716, DOI 10.1109/TAC.1974.1100705
Altizer S, 2003, ANNU REV ECOL EVOL S, V34, P517, DOI 10.1146/annurev.ecolsys.34.030102.151725
Ancillotto L, 2012, ETHOLOGY, V118, P331, DOI 10.1111/j.1439-0310.2011.02016.x
[Anonymous], 1988, REPROD ASSESSMENT BA
[Anonymous], ACARINA FAMILY CHEYL
[Anonymous], THESIS
[Anonymous], 1994, AMBIO
[Anonymous], [No title captured]
[Anonymous], J PARASITOL RES
[Anonymous], BATS ANTHROPOCENE CO
Apha, 2005, Standard Methods for the Examinations of Water and Wastewater
BARAK Y, 1991, MAMMALIA, V55, P285, DOI 10.1515/mamm.1991.55.2.285
Bartonicka T, 2008, PARASITOL RES, V104, P163, DOI 10.1007/s00436-008-1175-1
Bayat S, 2014, ENVIRON INT, V63, P40, DOI 10.1016/j.envint.2013.10.009
Beldomenico Pablo M, 2010, Trends Ecol Evol, V25, P21, DOI 10.1016/j.tree.2009.06.015
Benejam L, 2010, WATER AIR SOIL POLL, V210, P231, DOI 10.1007/s11270-009-0245-z
Bennett BS, 2007, B ENVIRON CONTAM TOX, V78, P191, DOI 10.1007/s00128-007-9089-7
Blanar CA, 2016, PARASITOL RES, V115, P3853, DOI 10.1007/s00436-016-5151-x
Boag B, 2001, INT J PARASITOL, V31, P1421, DOI 10.1016/S0020-7519(01)00270-3
Brodkin MA, 2007, ENVIRON TOXICOL CHEM, V26, P80, DOI 10.1897/05-469.1
Carravieri Alice, 2013, Rhinolophe, V19, P1
Christe P, 2000, ECOL LETT, V3, P207
Christe P, 2007, J ANIM ECOL, V76, P703, DOI 10.1111/

j.1365-2656.2007.01255.x
Clark Donald R. Jr, 2001, P159
Cotter SC, 2011, FUNCT ECOL, V25, P186, DOI 10.1111/j.1365-2435.2010.01766.x
Cottontail VM, 2009, PARASITOLOGY, V136, P1133, DOI 10.1017/S0031182009990485
Dick CW, 2006, MICROMAMMALS AND MACROPARASITES: FROM EVOLUTIONARY ECOLOGY TO MANAGEMENT, P179, DOI 10.1007/978-4-431-36025-4_11
Eeva T, 2013, PARASITOLOGY, V140, P1384, DOI 10.1017/S0031182013000796
Fenton M, 2015, BATS WORLD SCI MYSTE, V1st
FOLSTAD I, 1992, AM NAT, V139, P603, DOI 10.1086/285346
Frank HK, 2016, ECOL EVOL, V6, P4898, DOI 10.1002/ece3.2245
GERELL R, 1993, BIOL CONSERV, V65, P153, DOI 10.1016/0006-3207(93)90444-6
Gillespie TR, 2005, J APPL ECOL, V42, P699, DOI 10.1111/j.1365-2664.2005.01049.x
Grasman KA, 2001, ECOTOXICOLOGY, V10, P101, DOI 10.1023/A:1008950025622
Hörak P, 2006, PARASITOL RES, V98, P396, DOI 10.1007/s00436-006-0142-y
Kanuch P, 2005, ACTA CHIROPTEROL, V7, P249, DOI 10.3161/1733-5329(2005)7[249:PDAEOL]2.0.CO;2
Khan R.A., 1991, Advances in Parasitology, V30, P201
Korine C, 2004, J ZOOL, V262, P187, DOI 10.1017/S0952836903004539
Korine C, 2012, J EXP BIOL, V215, P1435, DOI 10.1242/jeb.061796
Krasnov BR, 2005, OECOLOGIA, V146, P209, DOI 10.1007/s00442-005-0189-y
Kunz TH, 2003, BAT ECOLOGY, P3
KUNZ TH, 1982, J MAMMAL, V63, P23, DOI 10.2307/1380667
Lafferty KD, 1999, LIMNOL OCEANOGR, V44, P925, DOI 10.4319/lo.1999.44.3_part_2.0925
Lafferty Kevin D., 2005, P113
Lewis SE, 1996, BEHAV ECOL SOCIOBIOL, V39, P335, DOI 10.1007/s002650050298
Lourenço SI, 2007, J ZOOL, V273, P161, DOI 10.1111/j.1469-7998.2007.00322.x
Lucan RK, 2006, FOLIA PARASIT, V53, P147, DOI 10.14411/fp.2006.019
Lüftl S, 2005, FRESEN ENVIRON BULL, V14, P167
Marshall A.G., 1982, P369
McCracken Gary F., 2000, P321, DOI 10.1016/B978-012195670-7/50009-6
McLean JA, 1997, ANIM BEHAV, V54, P1193, DOI 10.1006/anbe.1997.0498
Moore MS, 2011, PLOS ONE, V6, DOI 10.1371/journal.pone.0027430
Morales-Malacara JB, 2002, J MED ENTOMOL, V39, P298, DOI 10.1603/0022-2585-39.2.298
Morley NJ, 2010, ARCH ENVIRON CON TOX, V58, P783, DOI 10.1007/s00244-009-9386-8
Mos L, 2006, ENVIRON TOXICOL CHEM, V25, P3110, DOI 10.1897/06-027R.1
Naidoo S, 2016, ENVIRON POLLUT, V208, P830, DOI 10.1016/j.envpol.2015.09.056

Naidoo S, 2015, ECOTOX ENVIRON SAFE, V114, P304, DOI 10.1016/j.ecoenv.2014.04.043
O'Shea TJ, 2001, ARCH ENVIRON CON TOX, V40, P112, DOI 10.1007/s002440010153
Patterson BD, 2008, J TROP ECOL, V24, P387, DOI 10.1017/S0266467408005117
Patterson BD, 2007, J TROP ECOL, V23, P177, DOI 10.1017/S0266467406003816
Pedersen SC, 2012, J MAMMAL, V93, P1380, DOI 10.1644/12-MAMM-A-033.1
Pikula J, 2010, ENVIRON TOXICOL CHEM, V29, P501, DOI 10.1002/etc.80
Pilosof S, 2014, MAMM BIOL, V79, P183, DOI 10.1016/j.mambio.2013.10.005
Pilosof S, 2012, PLOS ONE, V7, DOI 10.1371/journal.pone.0041487
Poulin R., 2007, EVOL ECOL
Presley SJ, 2008, J TROP ECOL, V24, P75, DOI 10.1017/S0266467407004683
RACEY PA, 1974, J ZOOL, V173, P264, DOI 10.1111/j.1469-7998.1974.tb03136.x
RADOVSKY FRANK J., 1967, UNIV CALIF PUBL ENTOMOL, V46., P1
Razgour O, 2010, ANIM CONSERV, V13, P505, DOI 10.1111/j.1469-1795.2010.00371.x
Reckardt K, 2009, OIKOS, V118, P183, DOI 10.1111/j.1600-0706.2008.16950.x
Reiczigel J., 2005, QUANTITATIVE PARASIT
Reinhardt K, 2007, ANNU REV ENTOMOL, V52, P351, DOI 10.1146/annurev.ento.52.040306.133913
Rózsa L, 2000, J PARASITOL, V86, P228, DOI 10.1645/0022-3395(2000)086[0228:QPISOH]2.0.CO;2
Safi K, 2007, AM NAT, V170, P465, DOI 10.1086/520116
SAVILLE DJ, 1990, AM STAT, V44, P174, DOI 10.2307/2684163
Serangeli MT, 2012, ANIM WELFARE, V21, P9, DOI 10.7120/096272812799129510
Shore R.F., 2001, Ecotoxicology of Wild Mammals
SONENSHINE DANIEL E., 1962, ACAROLOGIA, V4, P193
Sures B, 2017, PARASITE VECTOR, V10, DOI 10.1186/s13071-017-2001-3
Swanepoel RE, 1999, ENVIRON POLLUT, V104, P169, DOI 10.1016/S0269-7491(98)00196-1
ter Hofstede HM, 2005, J ZOOL, V266, P333, DOI 10.1017/S095283690500693X
UNESCO/FAO, 1963, ARID ZONE RES, V21, P1
Veiga JP, 1998, OIKOS, V82, P313, DOI 10.2307/3546971
Vidal-Martínez VM, 2010, TRENDS PARASITOL, V26, P44, DOI 10.1016/j.pt.2009.11.001
Wada H, 2010, ECOTOXICOLOGY, V19, P1277, DOI 10.1007/s10646-010-0513-0
Walker LA, 2007, ENVIRON POLLUT, V148, P483, DOI 10.1016/j.envpol.2006.12.006
Warburton EM, 2016, PARASITOL RES, V115, P2155, DOI 10.1007/s00436-016-4957-x
Wolinska J, 2009, TRENDS PARASITOL, V25, P236, DOI 10.1016/j.pt.2009.02.004

   Zahn A, 2004, J ZOOL, V262, P383, DOI 10.1017/S0952836903004722
NR 90
TC 6
Z9 7
U1 2
U2 32
PU SPRINGER
PI NEW YORK
PA ONE NEW YORK PLAZA, SUITE 4600, NEW YORK, NY, UNITED STATES
SN 0932-0113
EI 1432-1955
J9 PARASITOL RES
JI Parasitol. Res.
PD SEP
PY 2017
VL 116
IS 9
BP 2517
EP 2526
DI 10.1007/s00436-017-5561-4
PG 10
WC Parasitology
WE Science Citation Index Expanded (SCI-EXPANDED)
SC Parasitology
GA FD2WU
UT WOS:000407396300017
PM 28735468
DA 2024-10-21
ER

PT J
AU Li, P
   Hu, JX
   Liu, Y
   Ou, XY
   Mu, ZX
   Lu, X
   Zan, FW
   Cao, MM
   Tan, L
   Dong, SW
   Zhou, Y
   Lu, J
   Jin, Q
   Wang, JW
   Wu, ZQ
   Zhang, YT
   Qian, ZH
AF Li, Pei
   Hu, Jiaxin
   Liu, Yan
   Ou, Xiuyuan
   Mu, Zhixia
   Lu, Xing

Zan, Fuwen
Cao, Mengmeng
Tan, Lin
Dong, Siwen
Zhou, Yao
Lu, Jian
Jin, Qi
Wang, Jianwei
Wu, Zhiqiang
Zhang, Yingtao
Qian, Zhaohui
TI Effect of polymorphism in <i>Rhinolophus affinis</i> ACE2 on entry of
SARS-CoV-2 related bat coronaviruses
SO PLOS PATHOGENS
LA English
DT Article
ID GENE
AB Author summaryBat CoV RaTG13 has zoonotic transmission potential, and interactions between S protein and ACE2 play essential roles. RaTG13 uses RaACE2 as an entry receptor, and there are at least seven polymorphic RaACE2 variants. The effect of RaACE2 polymorphism on entry of RaTG13 and other SARS-CoV-2 related bat CoV (SC2r-CoV) has not been determined. In this study, we find that RaTG13 S pseudovirions only highly efficiently transduce cells expressing RaACE2 among available 18 bat species commonly habituating in Southwestern China and Southeastern Asia, suggesting that there might be limited bat species including R. affinis susceptible to infection of RaTG13 virus. The RaACE2 polymorphism has a marked effect on the entry of RaTG13, whereas it shows a minimal effect on the entry of SARS-CoV-2 and SC2r-CoVs like BANAL-20-52, BANAL-20-236, and pangolin-CoV GD. Further sequence and mutagenesis analyses identify residues 34, 38, and 83 in RaACE2 and residues 501 and 505 in the S proteins critical for S protein and receptor interactions, indicating that RaTG13 might have not been well adapted to R. affinis bats. We also find that T372A substitution in RaTG13 S significantly enhances entry on all RaACE2 variants and several bat ACE2s, and immune evasion might contribute to the natural selection of T372 over A372 in bat S proteins. Altogether, our work provides detailed analyses of the effect of RaACE2 polymorphism on the entry of RaTG13 and other SC2r-CoVs and a better understanding of the entry and evolution of CoVs.
Bat coronavirus RaTG13 shares about 96.2% nucleotide sequence identity with that of SARS-CoV-2 and uses human and Rhinolophus affinis (Ra) angiotensin-converting enzyme 2 (ACE2) as entry receptors. Whether there are bat species other than R. affinis susceptible to RaTG13 infection remains elusive. Here, we show that, among 18 different bat ACE2s tested, only RaACE2 is highly susceptible to transduction by RaTG13 S pseudovirions, indicating that the bat species harboring RaTG13 might be very limited. RaACE2 has seven polymorphic variants, RA-01 to RA-07, and they show different susceptibilities to RaTG13 S transduction. Sequence and mutagenesis analyses reveal that residues 34, 38, and 83 in RaACE2 might play critical roles in interaction with the RaTG13 S protein.

Of note, RaACE2 polymorphisms have minimal effect on S proteins of SARS-CoV-2 and several SARS-CoV-2 related CoVs (SC2r-CoV) including BANAL-20-52 and BANAL-20-236 in terms of binding, membrane fusion, and pseudovirus entry. Further mutagenesis analyses identify residues 501 and 505 in S proteins critical for the recognition of different RaACE2 variants and pangolin ACE2 (pACE2), indicating that RaTG13 might have not been well adapted to R. affinis bats. While single D501N and H505Y changes in RaTG13 S protein significantly enhance the infectivity and minimize the difference in susceptibility among different RaACE2 variants, an N501D substitution in SARS-CoV-2 S protein displays marked disparity in transduction efficiencies among RaACE2 variants with a significant reduction in infectivity on several RaACE2 variants. Finally, a T372A substitution in RaTG13 S protein not only significantly increases infectivity on all RaACE2 variants, but it also markedly enhances entry on several bat ACE2s including R. sinicus YN, R. pearsonii, and R. ferrumeiqunum. However, the T372A mutant is about 4-fold more sensitive to neutralizing sera from mice immunized with BANAL-20-52 S, suggesting that the better immune evasion ability of T372 over A372 might contribute to the natural selective advantage of T372 over A372 among bat CoVs. Together, our study aids a better understanding of coronavirus entry, vaccine design, and evolution.
C1 [Li, Pei; Hu, Jiaxin; Liu, Yan; Ou, Xiuyuan; Mu, Zhixia; Lu, Xing; Zan, Fuwen; Cao, Mengmeng; Tan, Lin; Dong, Siwen; Zhou, Yao; Jin, Qi; Wang, Jianwei; Wu, Zhiqiang; Qian, Zhaohui] Chinese Acad Med Sci & Peking Union Med Coll, Inst Pathogen Biol, NHC Key Lab Syst Biol Pathogens, Beijing, Peoples R China.
[Lu, Jian] Peking Univ, Coll Life Sci, Beijing, Peoples R China.
[Zhang, Yingtao] Peking Univ, Sch Pharmaceut Sci, Beijing, Peoples R China.
C3 Chinese Academy of Medical Sciences - Peking Union Medical College;
Peking Union Medical College; Institute of Pathogen Biology - CAMS;
Peking University; Peking University
RP Wu, ZQ; Qian, ZH (corresponding author), Chinese Acad Med Sci & Peking Union Med Coll, Inst Pathogen Biol, NHC Key Lab Syst Biol Pathogens, Beijing, Peoples R China.; Zhang, YT (corresponding author), Peking Univ, Sch Pharmaceut Sci, Beijing, Peoples R China.
EM wuzq2009@ipbcams.ac.cn; zytao@bjmu.edu.cn; zqian2013@sina.com
RI zhang, Yingtao/HKE-5506-2023; Wang, Jianwei/W-5916-2019; Cao, Mengmeng/JFA-9946-2023; LU, JIAN/C-6044-2013; wu, zhiqiang/HTP-9144-2023; Qian, Zhaohui/AFR-9488-2022
OI Qian, Zhaohui/0000-0003-4116-4450
FU National Key R&D Program of China [2020YFA0707600, 2021YFC0866100,
2022YFE0210300]; National Natural Science Foundation of China [31970171,
32270174]; CAMS Innovation Fund for Medical Sciences [2021-12M-1-038];
Beijing Natural Science Foundation [5222032]; Fundamental Research Funds
for the Central Universities [3332021054]; Nonprofit Central Research

Institute Fund of Chinese Academy of Medical Sciences [2020-PT310-004,
2021-PT310-004]; National Science and Technology Infrastructure of China
[NPRC-32]
FX This work was supported by the National Key R&D Program of China
(2020YFA0707600 (ZQ), 2021YFC0866100 (ZQ), 2022YFE0210300 (ZW)), the
National Natural Science Foundation of China (31970171 and 32270174)
(ZQ), the CAMS Innovation Fund for Medical Sciences (2021-12M-1-038)
(JW), Beijing Natural Science Foundation (5222032) (XO), the Fundamental
Research Funds for the Central Universities (3332021054), the Nonprofit
Central Research Institute Fund of Chinese Academy of Medical Sciences
(2020-PT310-004, 2021-PT310-004); the National Science and Technology
Infrastructure of China (Project No. National Pathogen Resource
Center-NPRC-32). The funders had no role in study design, data
collection and analysis, decision to publish, or preparation of the
manuscript.
CR Chiu RWK, 2004, CLIN CHEM, V50, P1683, DOI 10.1373/clinchem.2004.035436
Crackower MA, 2002, NATURE, V417, P822, DOI 10.1038/nature00786
Devaux CA, 2020, J MICROBIOL IMMUNOL, V53, P425, DOI 10.1016/j.jmii.2020.04.015
Drosten C, 2003, NEW ENGL J MED, V348, P1967, DOI 10.1056/NEJMoa030747
Fröjdö S, 2005, DIABETOLOGIA, V48, P2278, DOI 10.1007/s00125-005-1955-4
Gorbalenya AE, 2020, NAT MICROBIOL, V5, P536, DOI 10.1038/s41564-020-0695-z
Gu HJ, 2020, SCIENCE, V369, P1603, DOI 10.1126/science.abc4730
Guo H, 2020, J VIROL, V94, DOI 10.1128/JVI.00902-20
Hattori T, 2022, MICROBIOL SPECTR, V10, DOI 10.1128/spectrum.00870-22
Hoffmann M, 2020, CELL, V181, P271, DOI 10.1016/j.cell.2020.02.052
Hou YX, 2010, ARCH VIROL, V155, P1563, DOI 10.1007/s00705-010-0729-6
IUCN, 2021, IUCN RED LIST THER S
Kang L, 2021, CELL, V184, P4392, DOI 10.1016/j.cell.2021.07.007
Ksiazek TG, 2003, NEW ENGL J MED, V348, P1953, DOI 10.1056/NEJMoa030781
Lan J, 2020, NATURE, V581, P215, DOI 10.1038/s41586-020-2180-5
Letko M, 2020, NAT MICROBIOL, V5, P562, DOI [10.1038/s41564-020-0688-y, 10.1101/2020.01.22.915660]
Li P, 2021, SCI BULL, V66, P1215, DOI 10.1016/j.scib.2021.01.011
Li YJ, 2020, J VIROL, V94, DOI 10.1128/JVI.01283-20
Liu KF, 2021, CELL, V184, P3438, DOI 10.1016/j.cell.2021.05.031

Liu KF, 2021, P NATL ACAD SCI USA, V118, DOI 10.1073/pnas.2020216118
Liu Y, 2022, NATURE, V602, P294, DOI 10.1038/s41586-021-04245-0
Lu GW, 2015, TRENDS MICROBIOL, V23, P468, DOI 10.1016/j.tim.2015.06.003
Muruato A, 2021, PLOS BIOL, V19, DOI 10.1371/journal.pbio.3001284
Ou XY, 2020, NAT COMMUN, V11, DOI 10.1038/s41467-020-15562-9
Palmer BR, 2008, AM HEART J, V156, P752, DOI 10.1016/j.ahj.2008.06.013
Patel SK, 2012, AM J HYPERTENS, V25, P216, DOI 10.1038/ajh.2011.188
Peiris JSM, 2003, LANCET, V361, P1319, DOI 10.1016/S0140-6736(03)13077-2
Ren LL, 2020, CHINESE MED J-PEKING, V133, P1015, DOI 10.1097/CM9.0000000000000722
Ren WL, 2022, J VIROL, V96, DOI 10.1128/JVI.01492-21
Shang J, 2020, NATURE, V581, P221, DOI 10.1038/s41586-020-2179-y
Shukla N, 2021, PLOS PATHOG, V17, DOI 10.1371/journal.ppat.1009715
Starr TN, 2022, NATURE, V603, P913, DOI 10.1038/s41586-022-04464-z
Suryamohan K, 2021, COMMUN BIOL, V4, DOI 10.1038/s42003-021-02030-3
Temmam S, 2022, NATURE, V604, P330, DOI 10.1038/s41586-022-04532-4
Tian F, 2021, ELIFE, V10, DOI 10.7554/eLife.69091
Wang B, 2018, J VIROL, V92, DOI [10.1128/JVI.00318-18, 10.1128/jvi.00318-18]
Wang QH, 2019, CURR OPIN VIROL, V34, P39, DOI 10.1016/j.coviro.2018.12.001
Wrobel AG, 2020, NAT STRUCT MOL BIOL, V27, P763, DOI 10.1038/s41594-020-0468-7
Zaki AM, 2012, NEW ENGL J MED, V367, P1814, DOI 10.1056/NEJMoa1211721
Zhang SY, 2022, CELL RES, V32, P315, DOI 10.1038/s41422-021-00600-y
Zhang SY, 2021, NAT COMMUN, V12, DOI 10.1038/s41467-021-21767-3
Zhang W, 2022, P NATL ACAD SCI USA, V119, DOI 10.1073/pnas.2206509119
Zhou P, 2020, NATURE, V579, P270, DOI [10.1038/s41586-020-2012-7, 10.1038/s41586-020-2951-z]
Zhu N, 2020, NEW ENGL J MED, V382, P727, DOI [10.1056/NEJMoa2001017, 10.1172/JCI89857]
NR 44
TC 6
Z9 6
U1 2
U2 6
PU PUBLIC LIBRARY SCIENCE
PI SAN FRANCISCO
PA 1160 BATTERY STREET, STE 100, SAN FRANCISCO, CA 94111 USA
SN 1553-7366
EI 1553-7374
J9 PLOS PATHOG

JI PLoS Pathog.
PD JAN
PY 2023
VL 19
IS 1
AR e1011116
DI 10.1371/journal.ppat.1011116
PG 24
WC Microbiology; Parasitology; Virology
WE Science Citation Index Expanded (SCI-EXPANDED)
SC Microbiology; Parasitology; Virology
GA 9C3MM
UT WOS:000935325700001
PM 36689489
OA gold, Green Published
DA 2024-10-21
ER

PT J
AU Schad, J
   Dechmann, DKN
   Voigt, CC
   Sommer, S
AF Schad, J.
   Dechmann, D. K. N.
   Voigt, C. C.
   Sommer, S.
TI MHC class II <i>DRB</i> diversity, selection pattern and population
   structure in a neotropical bat species, <i>Noctilio albiventris</i>
SO HEREDITY
LA English
DT Article
DE major histocompatibility complex polymorphism; selection; population
   structure; neotropical bat; Noctilio albiventris; Panama
ID MAJOR HISTOCOMPATIBILITY COMPLEX; SACCOPTERYX-BILINEATA CHIROPTERA;
   FREQUENCY-DEPENDENT SELECTION; NUCLEOTIDE SUBSTITUTION; REGION
   CONFIGURATIONS; STATISTICAL-METHODS; GENETIC DIVERSITY; SEX-RATIO; LOCI;
   OVERDOMINANT
AB Genes of the major histocompatibility complex (MHC) have a crucial role in the immune response of vertebrates, alter the individual odour and are involved in shaping mating preferences. Pathogen-mediated selection, sexual selection and maternal-fetal interactions have been proposed as the main drivers of frequently observed high levels of polymorphism in functionally important parts of the MHC. Bats constitute the second largest mammalian order and have recently emerged as important vectors of infectious diseases. In addition, Chiroptera are interesting study subjects in evolutionary ecology in the context of olfactory communication, mate choice and associated fitness benefits. Thus, it is surprising that

they belong to the least studied mammalian taxa in terms of their MHC diversity. In this study, we investigated the variability in the functionally important MHC class II gene DRB, evidence for selection and population structure in the group-living lesser bulldog bat, Noctilio albiventris, in Panama. We found a single expressed, polymorphic Noal-DRB gene. The substitution pattern of the nucleotide sequences of the 18 detected alleles provided evidence for positive selection acting above the evolutionary history of the species in shaping MHC diversity. Roosting colonies were not genetically differentiated but females showed lower levels of heterozygosity than males, which might be a sign that the sexes differ in the selection pressures acting on the MHC. This study provides the prerequisites for further investigations of the role of the individual MHC constitution in parasite resistance, olfactory communication and mate choice in N. albiventris and other bats.
Heredity (2011) 107, 115-126; doi:10.1038/hdy.2010.173; published online 19 January 2011
C1 [Schad, J.; Dechmann, D. K. N.; Voigt, C. C.; Sommer, S.] Leibniz Inst Zoo & Wildlife Res, D-10315 Berlin, Germany.
[Dechmann, D. K. N.] Max Planck Inst Ornithol, Vogelwarte Radolfzell, Radolfzell am Bodensee, Germany.
[Dechmann, D. K. N.] Smithsonian Trop Res Inst, Balboa, Ancon, Panama.
C3 Leibniz Institut fur Zoo und Wildtierforschung; Max Planck Society;
Smithsonian Institution; Smithsonian Tropical Research Institute
RP Sommer, S (corresponding author), Leibniz Inst Zoo & Wildlife Res, Alfred Kowalke St 17, D-10315 Berlin, Germany.
EM sommer@izw-berlin.de
RI Dechmann, Dina/A-1827-2010
OI Dechmann, Dina K. N./0000-0003-0043-8267
FU German Science Foundation [VO 890/11-1]
FX We thank Silke L Voigt-Heucke, Antje Kretzschmar, Megan Lupek, Martin
Wikelski and Apanie Wood for help with catching bats and sample
collection. We thank Wieslaw Babik for initial advice on the vectorette
PCR approach. We are grateful to Charles Gunn for linguistic advice. We
thank three anonymous reviewers for helpful comments on former versions
of the manuscript. The German Science Foundation funded C. Voigt, S.
Speck and D. Dechmann (VO 890/11-1). All capture and handling of
animals, as well as collection and export of samples were done in
concordance with Panamanian laws under permits from the Panamanian
authorities (ANAM) and under an IUCAC protocol. We are indebted to the
Smithsonian Tropical Research Institute and their staff for help with
logistics and interaction with the Panamanian authorities.
CR Altringham JD, 2003, BAT ECOLOGY, P90
[Anonymous], 1991, Molecular Evolution of the Major

Histocompatibility Complex
ASTOLFI P, 1990, TISSUE ANTIGENS, V35, P172, DOI 10.1111/j.1399-0039.1990.tb01775.x
Astolfi P, 1996, HUM HERED, V46, P155, DOI 10.1159/000154345
Babik W, 2008, MOL ECOL, V17, P2339, DOI 10.1111/j.1365-294X.2008.03757.x
Babik W, 2005, MOL ECOL, V14, P4249, DOI 10.1111/j.1365-294X.2005.02751.x
Baker CS, 2006, IMMUNOGENETICS, V58, P283, DOI 10.1007/s00251-006-0080-y
Bernatchez L, 2003, J EVOLUTION BIOL, V16, P363, DOI 10.1046/j.1420-9101.2003.00531.x
Bouchard S, 2001, J ZOOL, V254, P109, DOI 10.1017/S0952836901000607
Bowen L, 2004, IMMUNOGENETICS, V56, P12, DOI 10.1007/s00251-004-0655-4
Brooke AP, 1996, J CHEM ECOL, V22, P1411, DOI 10.1007/BF02027721
BROWN JH, 1993, NATURE, V364, P33, DOI 10.1038/364033a0
Calisher CH, 2006, CLIN MICROBIOL REV, V19, P531, DOI 10.1128/CMR.00017-06
Caspers B, 2009, ZOOMORPHOLOGY, V128, P37, DOI 10.1007/s00435-008-0072-6
Chaix R, 2008, PLOS GENET, V4, DOI 10.1371/journal.pgen.1000184
CHESSER RK, 1991, GENETICS, V127, P437
Coulson T, 1999, EVOLUTION, V53, P1951, DOI [10.2307/2640453, 10.1111/j.1558-5646.1999.tb04575.x]
Cummings SM, 2010, CONSERV GENET, V11, P1095, DOI 10.1007/s10592-009-9864-6
Cutrera AP, 2006, J MAMMAL, V87, P706, DOI 10.1644/05-MAMM-A-383R1.1
Dakin EE, 2004, HEREDITY, V93, P504, DOI 10.1038/sj.hdy.6800545
DAPSON RW, 1977, J MAMMAL, V58, P531, DOI 10.2307/1380001
Dechmann DKN, 2009, P ROY SOC B-BIOL SCI, V276, P2721, DOI 10.1098/rspb.2009.0473
Doherty P C, 1975, Nature, V256, P50
Dorak MT, 2002, GENES IMMUN, V3, P263, DOI 10.1038/sj.gene.6363862
Doxiadis GGM, 2000, J IMMUNOL, V164, P3193, DOI 10.4049/jimmunol.164.6.3193
Edwards Scott V., 1996, P214
EISENACH M, 2006, MOL CELL BIOL, V7, P276
Ellis SA, 2006, IMMUNOGENETICS, V57, P953, DOI 10.1007/s00251-005-0071-4
Excoffier L, 2005, EVOL BIOINFORM, V1, P47, DOI 10.1177/117693430500100003
Fernandez N, 1999, HUM REPROD UPDATE, V5, P234, DOI 10.1093/humupd/5.3.234
Field HE, 2009, ZOONOSES PUBLIC HLTH, V56, P278, DOI 10.1111/j.1863-2378.2008.01218.x
GILL TJ, 1992, AM J HUM GENET, V50, P1
GOUDET, 2001, FSTAT VERSION PROGRA
GUSTIN MK, 1987, ANIM BEHAV, V35, P13, DOI 10.1016/S0003-3472(87)80205-1
Hambuch TM, 2002, EVOLUTION, V56, P841

HAMILTON MS, 1978, BIOL REPROD, V19, P267, DOI 10.1095/biolreprod19.2.267
Hedrick PW, 1999, EVOLUTION, V53, P313, DOI [10.2307/2640768, 10.1111/j.1558-5646.1999.tb03767.x]
Hedrick PW, 1997, AM J HUM GENET, V61, P505, DOI 10.1086/515519
Hedrick PW, 2002, EVOLUTION, V56, P1902, DOI 10.1111/j.0014-3820.2002.tb00116.x
HO HN, 1990, AM J OBSTET GYNECOL, V163, P178, DOI 10.1016/S0002-9378(11)90696-6
Hughes AL, 2007, HEREDITY, V99, P364, DOI 10.1038/sj.hdy.6801031
HUGHES AL, 1988, NATURE, V335, P167, DOI 10.1038/335167a0
HUGHES AL, 1989, P NATL ACAD SCI USA, V86, P958, DOI 10.1073/pnas.86.3.958
Ilmonen P, 2009, BMC EVOL BIOL, V9, DOI 10.1186/1471-2148-9-104
JANEWAY CA, 2002, IMMUNOLOGY
Jukes T.H., 1969, MAMMALIAN PROTEIN ME, P21, DOI DOI 10.1093/BIOINFORMATICS/BTM404
KIMURA M, 1977, NATURE, V267, P275, DOI 10.1038/267275a0
KLEIN J, 1990, IMMUNOGENETICS, V31, P217, DOI 10.1007/BF00204890
Klein J., 1986, NATURAL HIST MAJOR H
Ko WY, 2003, J MOL EVOL, V57, P562, DOI 10.1007/s00239-003-2510-x
Kumánovics A, 2003, ANNU REV IMMUNOL, V21, P629, DOI 10.1146/annurev.immunol.21.090501.080116
Kundu S, 2004, P ROY SOC B-BIOL SCI, V271, P273, DOI 10.1098/rspb.2003.2584
Kwak J, 2009, PHYSIOL BEHAV, V96, P184, DOI 10.1016/j.physbeh.2008.10.003
Matocq MD, 2004, BEHAV ECOL, V15, P647, DOI 10.1093/beheco/arh056
Mayer F, 2007, HEREDITY, V99, P257, DOI 10.1038/sj.hdy.6800989
McCracken Gary F., 2000, P321, DOI 10.1016/B978-012195670-7/50009-6
Meagher S, 2000, P NATL ACAD SCI USA, V97, P3324, DOI 10.1073/pnas.060284797
Meyer-Lucht Y, 2008, BMC GENET, V9, DOI 10.1186/1471-2156-9-39
Milinski M, 2006, ANNU REV ECOL EVOL S, V37, P159, DOI 10.1146/annurev.ecolsys.37.091305.110242
NEI M, 1986, MOL BIOL EVOL, V3, P418
Nei M, 2000, MOL EVOLUTION PHYLOG
Nielsen R, 2001, HEREDITY, V86, P641, DOI 10.1046/j.1365-2540.2001.00895.x
OBER C, 1987, AM J REPROD IM MIC, V15, P141
Ober C, 1997, CURR TOP MICROBIOL, V222, P1
PALM J, 1974, CANCER RES, V34, P2061
PALM J, 1969, TRANSPLANT P, V1, P82
PALM J, 1970, TRANSPLANT P, V2, P162
Parham P, 1996, SCIENCE, V272, P67, DOI 10.1126/science.272.5258.67
PATTERSON S, 1997, P ROY SOC LOND B BIO, V264, P1813
Penn D, 1998, ADV IMMUNOL, V69, P411, DOI 10.1016/S0065-2776(08)60612-4
Penn DJ, 1999, AM NAT, V153, P145, DOI 10.1086/303166
Piertney SB, 2006, HEREDITY, V96, P7, DOI 10.1038/sj.hdy.6800724
Quay W. B., 1970, P1
RICE WR, 1989, EVOLUTION, V43, P223, DOI 10.1111/

j.1558-5646.1989.tb04220.x
Rossiter SJ, 2001, P ROY SOC B-BIOL SCI, V268, P1055, DOI 10.1098/rspb.2001.1612
Rülicke T, 1998, P ROY SOC B-BIOL SCI, V265, P711, DOI 10.1098/rspb.1998.0351
Safi K, 2003, ANIM BEHAV, V65, P363, DOI 10.1006/anbe.2003.2067
Schad J, 2004, CONSERV GENET, V5, P299, DOI 10.1023/B:COGE.0000031137.50239.d3
Schwensow N, 2007, HEREDITY, V99, P265, DOI 10.1038/sj.hdy.6800993
Schwensow N, 2008, EVOL ECOL, V22, P617, DOI 10.1007/s10682-007-9186-4
Scully WMR, 2000, CAN J ZOOL, V78, P613, DOI 10.1139/cjz-78-4-613
Seddon JM, 2004, P ROY SOC B-BIOL SCI, V271, P2283, DOI 10.1098/rspb.2004.2869
SLADE RW, 1992, GENETICS, V132, P861
Solomon NG, 2003, J MAMMAL, V84, P1182, DOI 10.1644/BLe-013
Sommer S, 2002, BEHAV ECOL SOCIOBIOL, V51, P214, DOI 10.1007/S00265-001-0432-4
Sommer Simone, 2005, Frontiers in Zoology, V2, P1, DOI 10.1186/1742-9994-2-16
Spurgin LG, 2010, P ROY SOC B-BIOL SCI, V277, P979, DOI 10.1098/rspb.2009.2084
STERN LJ, 1994, NATURE, V368, P215, DOI 10.1038/368215a0
STUDIER EH, 1984, J MAMMAL, V65, P711, DOI 10.2307/1380864
TAKAHATA N, 1990, GENETICS, V124, P967
Tamura K, 2007, MOL BIOL EVOL, V24, P1596, DOI 10.1093/molbev/msm092
TRAVIS SE, 1995, ECOLOGY, V76, P1794, DOI 10.2307/1940711
Voigt CC, 1999, BEHAV ECOL SOCIOBIOL, V47, P29, DOI 10.1007/s002650050646
Wedekind C, 1996, HEREDITY, V77, P400, DOI 10.1038/hdy.1996.160
WEDEKIND C, 1995, P ROY SOC B-BIOL SCI, V260, P245, DOI 10.1098/rspb.1995.0087
Wegner KM, 2003, SCIENCE, V301, P1343, DOI 10.1126/science.1088293
Wibbelt G, 2010, BIOL LETTERS, V6, P438, DOI 10.1098/rsbl.2010.0267
Wilmer Jessica Worthington, 1996, Bat Research News, V37, P1
Woelfing B, 2009, PHILOS T R SOC B, V364, P117, DOI 10.1098/rstb.2008.0174
Wong S, 2007, REV MED VIROL, V17, P67, DOI 10.1002/rmv.520
Wong WSW, 2004, GENETICS, V168, P1041, DOI 10.1534/genetics.104.031153
WRIGHT S, 1965, EVOLUTION, V19, P395, DOI 10.2307/2406450
WRIGHT S, 1951, ANN EUGENIC, V15, P323
Yang ZH, 2005, MOL BIOL EVOL, V22, P1107, DOI 10.1093/molbev/msi097
Yang ZH, 1997, COMPUT APPL BIOSCI, V13, P555
Yang ZH, 2000, TRENDS ECOL EVOL, V15, P496, DOI 10.1016/S0169-5347(00)01994-7
Yang ZH, 2007, MOL BIOL EVOL, V24, P1586, DOI 10.1093/molbev/msm088
Ziegler A, 2005, TRENDS IMMUNOL, V26, P496, DOI 10.1016/

j.it.2005.07.003
   Ziegler A, 2002, AM J REPROD IMMUNOL, V48, P34, DOI 10.1034/
j.1600-0897.2002.01097.x
NR 109
TC 22
Z9 23
U1 0
U2 45
PU SPRINGERNATURE
PI LONDON
PA CAMPUS, 4 CRINAN ST, LONDON, N1 9XW, ENGLAND
SN 0018-067X
EI 1365-2540
J9 HEREDITY
JI Heredity
PD AUG
PY 2011
VL 107
IS 2
BP 115
EP 126
DI 10.1038/hdy.2010.173
PG 12
WC Ecology; Evolutionary Biology; Genetics & Heredity
WE Science Citation Index Expanded (SCI-EXPANDED)
SC Environmental Sciences & Ecology; Evolutionary Biology; Genetics
&
   Heredity
GA 794PD
UT WOS:000292911500004
PM 21245894
OA Green Published, Bronze
DA 2024-10-21
ER

PT J
AU Schneeberger, K
   Courtiol, A
   Czirják, GA
   Voigt, CC
AF Schneeberger, Karin
   Courtiol, Alexandre
   Czirjak, Gabor A.
   Voigt, Christian C.
TI Immune Profile Predicts Survival and Reflects Senescence in a
Small,
   Long-Lived Mammal, the Greater Sac-Winged Bat (<i>Saccopteryx
   bilineata</i>)
SO PLOS ONE
LA English
DT Article
ID BODY ELECTRICAL-CONDUCTIVITY; INNATE IMMUNITY; LIFE-HISTORY;
   ENVIRONMENTAL-CONDITIONS; PARASITE RESISTANCE; TROPICAL BAT; SOAY
SHEEP;

IMMUNOSENESCENCE; ECOLOGY; SEX
AB The immune system imposes costs that may have to be traded against investment of resources in other costly life-history traits. Yet, it is unknown if a trade-off between immunity and longevity occurs in free-ranging mammals. Here, we tested if age and survival, two aspects associated with longevity, are linked to immune parameters in an 8 g bat species. Using a combination of cross-sectional and longitudinal data, we assessed whether total white blood cell (WBC) counts, bacterial killing ability of the plasma (BKA) and immunoglobulin G (IgG) concentration change with age. Furthermore, we asked if these immune parameters impose costs resulting in decreased survival probabilities. We found that WBC counts decreased with age both within and among individuals. IgG concentrations were higher in older individuals, but did not change with age within individuals. Furthermore, individuals with above average WBC counts or IgG concentration had lower probabilities to survive the next six months. High WBC counts and IgG concentrations may reflect infections with parasites and pathogens, however, individuals that were infected with trypanosomes or nematodes showed neither higher WBC counts or IgG concentrations, nor was infection connected with survival rates. BKA was higher in infected compared with uninfected bats, but not related to age or survival. In conclusion, cellular (WBC) and humoral (IgG) parts of the immune system were both connected to age and survival, but not to parasite infections, which supports the hypothesis that energetically costly immunological defences are traded against other costly life-history traits, leading to a reduced lifespan in this free-ranging mammal.
C1 [Schneeberger, Karin; Courtiol, Alexandre; Czirjak, Gabor A.; Voigt, Christian C.] Leibniz Inst Zoo & Wildlife Res, Berlin, Germany.
[Schneeberger, Karin; Voigt, Christian C.] Free Univ Berlin, Dept Anim Behav, Berlin, Germany.
C3 Leibniz Institut fur Zoo und Wildtierforschung; Free University of
Berlin
RP Schneeberger, K (corresponding author), Leibniz Inst Zoo & Wildlife Res, Berlin, Germany.
EM schneeberger@izw-berlin.de
RI Czirják, Gábor/F-5440-2011; Schneeberger, Karin/C-9836-2013; Courtiol,
Alexandre/O-3040-2017
OI Schneeberger, Karin/0000-0003-2098-2542; Courtiol,
Alexandre/0000-0003-0637-2959; Czirjak, Gabor Arpad/
0000-0001-9488-0069
FU Deutsche Forschungsgemeinschaft (DFG) [Vo 890/25]; Leibniz Institute for
Zoo and Wildlife Research
FX Funding was provided by the Deutsche Forschungsgemeinschaft (DFG Vo
890/25) and the Leibniz Institute for Zoo and Wildlife Research. The
funders had no role in study design, data collection and analysis,
decision to publish, or preparation of the manuscript.

CR Allen LC, 2009, J COMP PHYSIOL B, V179, P315, DOI 10.1007/s00360-008-0315-3
[Anonymous], THESIS BOSTON U
Balomenos D, 2000, IMMUNOL TODAY, V21, P551, DOI 10.1016/S0167-5699(00)01748-5
Belloni V, 2010, PLOS ONE, V5, DOI 10.1371/journal.pone.0012940
Bize P, 2014, OECOLOGIA, V174, P1097, DOI 10.1007/s00442-013-2840-3
Blount DG, 2005, VET IMMUNOL IMMUNOP, V108, P399, DOI 10.1016/j.vetimm.2005.06.015
BOX GEP, 1964, J ROY STAT SOC B, V26, P211, DOI 10.1111/j.2517-6161.1964.tb00553.x
Bradbury J.W., 1974, Z TIERPSYCHOL, V36, P137, DOI [DOI 10.1111/J.1439-0310.1974.TB02130.X, 10.1111/j.1439-0310.1974.tb02130.x]
Christe P, 2001, OECOLOGIA, V126, P333, DOI 10.1007/s004420000527
Christe P, 2000, ECOL LETT, V3, P207
Christe P, 2007, J ANIM ECOL, V76, P703, DOI 10.1111/j.1365-2656.2007.01255.x
Coltman DW, 1999, EVOLUTION, V53, P1259, DOI 10.2307/2640828
Devevey G, 2009, BIOL J LINN SOC, V97, P328, DOI 10.1111/j.1095-8312.2009.01216.x
Dröge W, 2002, PHYSIOL REV, V82, P47, DOI 10.1152/physrev.00018.2001
Durbin C, 2009, VET CLIN PATH, V38, P157, DOI 10.1111/j.1939-165X.2009.00110.x
Eraud C, 2009, EVOLUTION, V63, P1036, DOI 10.1111/j.1558-5646.2008.00540.x
Fox J., 2018, R COMPANION APPL REG
Franceschi C, 2000, VACCINE, V18, P1717, DOI 10.1016/S0264-410X(99)00513-7
Frasca D, 2005, SEMIN IMMUNOL, V17, P378, DOI 10.1016/j.smim.2005.05.005
Geiger H, 2002, NAT IMMUNOL, V3, P329, DOI 10.1038/ni0402-329
Ginaldi L, 2001, MICROBES INFECT, V3, P851, DOI 10.1016/S1286-4579(01)01443-5
Graham AL, 2010, SCIENCE, V330, P662, DOI 10.1126/science.1194878
Greiner S, 2014, ETHOLOGY, V120, P197, DOI 10.1111/eth.12193
Greiner S, 2011, CAN J ZOOL, V89, P1157, DOI 10.1139/Z11-092
GULLAND FMD, 1992, PARASITOLOGY, V105, P493, DOI 10.1017/S0031182000074679
Hakim FT, 2007, TISSUE ANTIGENS, V70, P179, DOI 10.1111/j.1399-0039.2007.00891.x
Hanssen SA, 2004, P ROY SOC B-BIOL SCI, V271, P925, DOI 10.1098/rspb.2004.2678
Harman D., 1955, Aging: a theory based on free radical and radiation chemistry
Hayward AD, 2011, J EVOLUTION BIOL, V24, P1664, DOI 10.1111/j.1420-9101.2011.02300.x
Hayward AD, 2009, P ROY SOC B-BIOL SCI, V276, P3477, DOI 10.1098/rspb.2009.0906
Kraus C, 2008, P ROY SOC B-BIOL SCI, V275, P1635, DOI 10.1098/rspb.2008.0200
Lee KA, 2006, INTEGR COMP BIOL, V46, P1000, DOI 10.1093/icb/icl049

LEHMANN T, 1993, PARASITOL TODAY, V9, P8, DOI 10.1016/0169-4758(93)90153-7
Linton P.J., 2006, Clinical and Applied Immunology Reviews, V6, P73
Listì F, 2006, ANN NY ACAD SCI, V1089, P487, DOI 10.1196/annals.1386.013
Lochmiller RL, 2000, OIKOS, V88, P87, DOI 10.1034/j.1600-0706.2000.880110.x
Lorch JM, 2011, NATURE, V480, P376, DOI 10.1038/nature10590
Meyer-Lucht Y, 2005, MOL ECOL, V14, P2233, DOI 10.1111/j.1365-294X.2005.02557.x
Monaghan P, 2008, FUNCT ECOL, V22, P371, DOI 10.1111/j.1365-2435.2008.01418.x
Moore MS, 2013, PLOS ONE, V8, DOI 10.1371/journal.pone.0058976
Moore MS, 2011, PLOS ONE, V6, DOI 10.1371/journal.pone.0027430
Nagy M, 2007, P ROY SOC B-BIOL SCI, V274, P3019, DOI 10.1098/rspb.2007.1008
Noreen E, 2011, J COMP PHYSIOL B, V181, P649, DOI 10.1007/s00360-011-0553-7
Nussey DH, 2008, FUNCT ECOL, V22, P393, DOI 10.1111/j.1365-2435.2008.01408.x
Nussey DH, 2012, AGING CELL, V11, P178, DOI 10.1111/j.1474-9726.2011.00771.x
Panda A, 2009, TRENDS IMMUNOL, V30, P325, DOI 10.1016/j.it.2009.05.004
Pearce RD, 2008, ACTA CHIROPTEROL, V10, P153, DOI 10.3161/150811008X331171
Reth M, 2002, NAT IMMUNOL, V3, P1129, DOI 10.1038/ni1202-1129
Reynolds DS, 2009, J WILDLIFE MANAGE, V73, P1197, DOI 10.2193/2008-151
Ricklefs RE, 2002, TRENDS ECOL EVOL, V17, P462, DOI 10.1016/S0169-5347(02)02578-8
ROSS PS, 1993, J WILDLIFE DIS, V29, P21, DOI 10.7589/0090-3558-29.1.21
Saino N, 1997, P NATL ACAD SCI USA, V94, P549, DOI 10.1073/pnas.94.2.549
Schinnerl M, 2011, J ZOO WILDLIFE MED, V42, P382, DOI 10.1638/2010-0060.1
Schneeberger K, 2013, J EXP BIOL, V216, P4514, DOI 10.1242/jeb.090837
Schneeberger K, 2013, PLOS ONE, V8, DOI 10.1371/journal.pone.0054023
Selman C, 2012, TRENDS ECOL EVOL, V27, P570, DOI 10.1016/j.tree.2012.06.006
Speakman JR, 2011, BIOESSAYS, V33, P255, DOI 10.1002/bies.201000132
Stöbel K, 2002, INT J MED MICROBIOL, V291, P88
Team R.C., 2019, LANG ENV STAT COMP
Tieleman BI, 2005, P ROY SOC B-BIOL SCI, V272, P1715, DOI 10.1098/rspb.2005.3155
UTSUYAMA M, 1992, MECH AGEING DEV, V63, P57, DOI 10.1016/0047-6374(92)90016-7
van de Pol M, 2006, AM NAT, V167, P766, DOI 10.1086/503331
VAZIRI H, 1994, P NATL ACAD SCI USA, V91, P9857, DOI 10.1073/

pnas.91.21.9857
   Venables W.N., 1994, MODERN APPL STAT S P
   Viau M, 2005, CLIN IMMUNOL, V114, P17, DOI 10.1016/
j.clim.2004.08.019
   Voigt CC, 2001, BEHAV ECOL SOCIOBIOL, V50, P31, DOI 10.1007/
s002650100337
   Voigt CC, 2008, J MAMMAL, V89, P50, DOI 10.1644/06-MAMM-A-432.1
   Voigt CC, 2007, ACTA CHIROPTEROL, V9, P463, DOI
10.3161/1733-5329(2007)9[463:AOFTMA]2.0.CO;2
   Wellehan JFX, 2009, COMP IMMUNOL MICROB, V32, P379, DOI 10.1016/
j.cimid.2007.11.002
   WHITLOCK H. V., 1950, JOUR HELMINTHOL, V24, P47, DOI 10.1017/
S0022149X00019106
   Wilkinson GS, 2002, AGING CELL, V1, P124, DOI 10.1046/
j.1474-9728.2002.00020.x
NR 71
TC 33
Z9 38
U1 0
U2 30
PU PUBLIC LIBRARY SCIENCE
PI SAN FRANCISCO
PA 1160 BATTERY STREET, STE 100, SAN FRANCISCO, CA 94111 USA
SN 1932-6203
J9 PLOS ONE
JI PLoS One
PD SEP 25
PY 2014
VL 9
IS 9
AR e108268
DI 10.1371/journal.pone.0108268
PG 8
WC Multidisciplinary Sciences
WE Science Citation Index Expanded (SCI-EXPANDED)
SC Science & Technology - Other Topics
GA AT3UZ
UT WOS:000344862300067
PM 25254988
OA Green Published, Green Submitted, gold
DA 2024-10-21
ER

PT J
AU Aicher, SM
   Streicher, F
   Chazal, M
   Planas, D
   Luo, DS
   Buchrieser, J
   Nemcova, M
   Seidlova, V
   Zukal, J
   Serra-Cobo, J

Pontier, D
Schwartz, O
Pikula, J
Dacheux, L
Jouvenet, N
Pain, B
Zimmer, G
Roingeard, P
AF Aicher, Sophie-Marie
Streicher, Felix
Chazal, Maxime
Planas, Delphine
Luo, Dongsheng
Buchrieser, Julian
Nemcova, Monika
Seidlova, Veronika
Zukal, Jan
Serra-Cobo, Jordi
Pontier, Dominique
Schwartz, Olivier
Pikula, Jiri
Dacheux, Laurent
Jouvenet, Nolwenn
Pain, Bertrand
Zimmer, Gert
Roingeard, Philippe
TI Species-Specific Molecular Barriers to SARS-CoV-2 Replication in Bat
Cells
SO JOURNAL OF VIROLOGY
LA English
DT Article
DE SARS-CoV-2; bat cells; coronavirus; innate immunity
ID ALPHA; CORONAVIRUSES; VIRUS; ACE2; EXPRESSION; TROPISM
AB Bats are natural reservoirs of numerous coronaviruses, including the potential ancestor of SARS-CoV-2. Knowledge concerning the interaction between coronaviruses and bat cells is sparse. We investigated the ability of primary cells from Rhinolophus and Myotis species, as well as of established and novel cell lines from Myotis myotis, Eptesicus serotinus, Tadarida brasiliensis, and Nyctalus noctula, to support SARS-CoV-2 replication. None of these cells were permissive to infection, not even the ones expressing detectable levels of angiotensin-converting enzyme 2 (ACE2), which serves as the viral receptor in many mammalian species. The resistance to infection was overcome by expression of human ACE2 (hACE2) in three cell lines, suggesting that the restriction to viral replication was due to a low expression of bat ACE2 (bACE2) or the absence of bACE2 binding in these cells. Infectious virions were produced but not released from hACE2-transduced M. myotis brain cells. E. serotinus brain cells and M. myotis nasal epithelial cells expressing hACE2 efficiently controlled viral replication, which correlated with a potent interferon response. Our data highlight the existence of species-specific and cell-specific molecular barriers to viral replication in bat cells. These novel chiropteran cellular

models are valuable tools to investigate the evolutionary relationships between bats and coronaviruses. IMPORTANCE Bats are host ancestors of several viruses that cause serious disease in humans, as illustrated by the ongoing SARS-CoV-2 pandemic. Progress in investigating bat-virus interactions has been hampered by a limited number of available bat cellular models. We have generated primary cells and cell lines from several bat species that are relevant for coronavirus research. The various permissivities of the cells to SARS-CoV-2 infection offered the opportunity to uncover some species-specific molecular restrictions to viral replication. All bat cells exhibited a potent entry-dependent restriction. Once this block was overcome by overexpression of human ACE2, which serves at the viral receptor, two bat cell lines controlled well viral replication, which correlated with the inability of the virus to counteract antiviral responses. Other cells potently inhibited viral release. Our novel bat cellular models contribute to a better understanding of the molecular interplays between bat cells and viruses.

Bats are host ancestors of several viruses that cause serious disease in humans, as illustrated by the ongoing SARS-CoV-2 pandemic. Progress in investigating bat-virus interactions has been hampered by a limited number of available bat cellular models.
C1 [Aicher, Sophie-Marie; Streicher, Felix; Chazal, Maxime; Jouvenet, Nolwenn] Univ Paris Cite, Inst Pasteur, Virus Sensing & Signaling Unit, CNRS,UMR 3569, Paris, France.

[Planas, Delphine; Buchrieser, Julian; Schwartz, Olivier] Univ Paris Cite, Inst Pasteur, Virus & Immun Unit, CNRS,UMR 3569, Paris, France.

[Planas, Delphine; Schwartz, Olivier] Vaccine Res Inst, Crete, France.

[Luo, Dongsheng; Dacheux, Laurent] Univ Paris Cite, Inst Pasteur, Lyssavirus Epidemiol & Neuropathol Unit, Paris, France.

[Nemcova, Monika; Seidlova, Veronika; Pikula, Jiri] Univ Vet Sci Brno, Dept Ecol & Dis Zoo Anim Game Fish & Bees, Brno, Czech Republic.

[Zukal, Jan] Czech Acad Sci Brno, Inst Vertebrate Biol, Brno, Czech Republic.

[Serra-Cobo, Jordi] Univ Barcelona, Fac Biol, Inst Recerca Biodiversitat IRBio, Barcelona, Spain.

[Serra-Cobo, Jordi] Univ Barcelona, Fac Biol, Dept Biol Evolut Ecol & Ciencies Ambientals, Barcelona, Spain.

[Pontier, Dominique] Univ Lyon, LabEx Ecofect, Lyon, France.

[Pontier, Dominique] Univ Lyon 1, CNRS, Lab Biometrie & Biol Evolut, UMR5558, Villeurbanne, France.

[Pain, Bertrand] Univ Lyon 1, Univ Lyon, INSERM, INRAE,Stem Cell & Brain Res Inst, Bron, France.

[Zimmer, Gert] Inst Virol & Immunol, Bern, Switzerland.

[Zimmer, Gert] Inst Virol & Immunol, Mittelhausern, Switzerland.

[Zimmer, Gert] Univ Bern, Vetsuisse Fac, Dept Infect Dis & Pathobiol, Bern, Switzerland.

[Roingeard, Philippe] Univ Tours, Fac Med, INSERM U1259 MAVIVH, Tours, France.

[Roingeard, Philippe] Univ Tours, Fac Med, Plateforme IBiSA Microscopie Elect, Tours, France.

C3 Centre National de la Recherche Scientifique (CNRS); CNRS - National
Institute for Biology (INSB); Pasteur Network; Universite Paris Cite;
Institut Pasteur Paris; Pasteur Network; Universite Paris Cite; Institut
Pasteur Paris; Centre National de la Recherche Scientifique (CNRS); CNRS
- National Institute for Biology (INSB); Pasteur Network; Universite
Paris Cite; Institut Pasteur Paris; University of Veterinary Sciences
Brno; Czech Academy of Sciences; University of Barcelona; University of
Barcelona; VetAgro Sup; Centre National de la Recherche Scientifique
(CNRS); CNRS - Institute of Ecology & Environment (INEE); Universite
Claude Bernard Lyon 1; Universite Claude Bernard Lyon 1; INRAE; Institut
National de la Sante et de la Recherche Medicale (Inserm); University of
Bern; Universite de Tours; Universite de Tours
RP Jouvenet, N (corresponding author), Univ Paris Cite, Inst
Pasteur, Virus Sensing & Signaling Unit, CNRS,UMR 3569, Paris,
France.; Dacheux, L (corresponding author), Univ Paris Cite, Inst
Pasteur, Lyssavirus Epidemiol & Neuropathol Unit, Paris, France.
EM laurent.dacheux@pasteur.fr; nolwenn.jouvenet@pasteur.fr
RI Seidlova, Veronika/HNQ-0598-2023; Dacheux, Laurent/AAJ-5372-2020;
Schwartz, Olivier/AAZ-3765-2021; jouvenet, nolwenn/K-2067-2019; Pikula,
Jiri/V-8620-2018; Pain, Bertrand/A-7686-2015; Zukal, Jan/G-1180-2014
OI Buchrieser, Julian/0000-0003-4790-7577; Pikula,
Jiri/0000-0001-8747-9365; Pain, Bertrand/0000-0002-1210-8444; Aicher,
Sophie-Marie/0000-0001-6114-2588; Zimmer, Gert/0000-0002-2708-2507;
Zukal, Jan/0000-0003-4967-6880; Pontier, Dominique/0000-0003-4700-3543;
jouvenet, nolwenn/0000-0001-6103-6048
FU French National Research Agency [ANR-10-INBS-04]; CNRS; "Urgence
COVID-19" fundraising campaign of Institut Pasteur; University of
Veterinary Sciences Brno [FVHE/Pikula/ITA2021]; FINOVI Fondation [AO13];
Covid IDEX Universite Lyon 1; LabEx Ecofect [ANR-11-LABX-0048]; Labex
IBEID [ANR-10-LABX-62-IBEID]; ANR/FRM Flash Covid PROTEO-SARS-CoV-2;
IDISCOVR; Pasteur-Paris University International Ph.D. Program; Vaccine
Research Institute [ANR-10-LABX-77]; Chinese Scholarship Council;
Institut Pasteur
FX We thank Noemie Aurine (SBRI, U1208 INSERM, France) for help

designing
RT-qPCR primers for bat samples; Ondine Filippi-Codaccioni and Marc
Lopez-Roig (Universite Lyon 1, France) for help in bat sampling; the
French National Reference Centre for Respiratory Viruses hosted by
Institut Pasteur (France) and headed by S. van der Werf for providing
the historical viral strains; Hugo Mouquet and Cyril Planchais (Institut
Pasteur) for providing anti-S antibodies; Francoise Porrot (Institut
Pasteur) for lentiviral production; Florence Guivel-Benhassine (Institut
Pasteur) for help in titration assays; and Matthias Lenk
(Friedrich-Loeffler-Institut, Germany) for providing the E. serotinus
and Tadarida brasiliensis cell lines. We are grateful to the members of
our laboratories for helpful discussions. We acknowledge the UTechS
Photonic BioImaging (Imagopole), C2RT, Institut Pasteur, supported by
the French National Research Agency (France BioImaging; ANR-10-INBS-04;
Investments for the Future) for the use of the confocal microscope. This
study was funded by the CNRS (N.J. and O.S.), Institut Pasteur (N.J.,
L.D., and O.S.), the "Urgence COVID-19" fundraising campaign of Institut
Pasteur (N.J., L.D., and O.S.), the University of Veterinary Sciences
Brno (FVHE/Pikula/ITA2021) (J.P.), the FINOVI Fondation (AO13) and Covid
IDEX Universite Lyon 1 (B.P.), LabEx Ecofect (ANR-11-LABX-0048) (Do.P.),
Labex IBEID (ANR-10-LABX-62-IBEID) (O.S.), ANR/FRM Flash Covid
PROTEO-SARS-CoV-2 (O.S.), and IDISCOVR (O.S.). S.-M.A. and De.P. are
supported by the Pasteur-Paris University International Ph.D. Program
and the Vaccine Research Institute (ANR-10-LABX-77), respectively. D.L.
was funded by the Chinese Scholarship Council and Institut Pasteur. The
funders had no role in study design, data collection and analysis,
decision to publish, or preparation of the manuscript. We declare that
no competing interests exist.
CR Allen JD, 2021, J MOL BIOL, V433, DOI 10.1016/j.jmb.2020.166762
Aurine N., 2019, BIORXIV, DOI [10.1101/846410v1, DOI

10.1101/846410V1]
Banerjee A, 2020, ISCIENCE, V23, DOI 10.1016/j.isci.2020.100958
Banerjee A, 2020, FRONT IMMUNOL, V11, DOI 10.3389/fimmu.2020.00026
Banerjee A, 2019, VIRUSES-BASEL, V11, DOI 10.3390/v11020152
Banerjee A, 2019, VIRUSES-BASEL, V11, DOI 10.3390/v11010041
Banerjee A, 2018, VIRUS RES, V248, P5, DOI 10.1016/j.virusres.2018.02.013
Blanco-Melo D, 2020, CELL, V181, P1036, DOI 10.1016/j.cell.2020.04.026
Bondet V, 2021, FRONT IMMUNOL, V12, DOI 10.3389/fimmu.2021.735866
Brook CE, 2020, ELIFE, V9, DOI 10.7554/eLife.48401
Buchrieser J, 2021, EMBO J, V40, DOI 10.15252/embj.2020107405
Bussani R, 2020, EBIOMEDICINE, V61, DOI 10.1016/j.ebiom.2020.103104
Cao YY, 2021, FRONT IMMUNOL, V12, DOI 10.3389/fimmu.2021.656433
Chu H, 2020, LANCET MICROBE, V1, pE14, DOI 10.1016/S2666-5247(20)30004-5
Cifuentes-Muñoz N, 2018, PLOS PATHOG, V14, DOI 10.1371/journal.ppat.1007015
Clausen TM, 2020, CELL, V183, P1043, DOI [10.1016/j.cell.2020.09.033, 10.1101/2020.07.14.201616]
Conceicao C, 2020, PLOS BIOL, V18, DOI 10.1371/journal.pbio.3001016
Cool K, 2022, EMERG MICROBES INFEC, V11, P95, DOI 10.1080/22221751.2021.2012528
Cortese M, 2020, CELL HOST MICROBE, V28, P853, DOI 10.1016/j.chom.2020.11.003
Cramer J, 2021, J AM CHEM SOC, V143, P17465, DOI 10.1021/jacs.1c06778
Damas Joana, 2020, bioRxiv, DOI [10.1073/pnas.2010146117, 10.1101/2020.04.16.045302]
De Benedictis P, 2014, VIRUS GENES, V48, P366, DOI 10.1007/s11262-013-1008-x
De La Cruz-Rivera PC, 2018, J IMMUNOL, V200, P209, DOI 10.4049/jimmunol.1701214
Delaune D, 2021, NAT COMMUN, V12, DOI 10.1038/s41467-021-26809-4
Dominguez SR, 2007, EMERG INFECT DIS, V13, P1295, DOI 10.3201/eid1309.070491
Eymieux S, 2021, CELL MOL LIFE SCI, V78, P3565, DOI 10.1007/s00018-020-03745-y
Falcón A, 2011, ARCH VIROL, V156, P1883, DOI 10.1007/s00705-011-1057-1
Frutos R, 2021, FRONT MICROBIOL, V12, DOI 10.3389/fmicb.2021.591535
Ge XY, 2013, NATURE, V503, P535, DOI 10.1038/nature12711
Glennon NB, 2015, J VIROL, V89, P7550, DOI 10.1128/JVI.00302-15
Hall JS, 2021, TRANSBOUND EMERG DIS, V68, P3443, DOI 10.1111/tbed.13949
Harcourt J, 2020, EMERG INFECT DIS, V26, P1266, DOI 10.3201/eid2606.200516
Hayn M, 2021, CELL REP, V35, DOI 10.1016/j.celrep.2021.109126
Hayward JA., 2020, BIORXIV, DOI [10.1101/2020.04.08.031203v3, DOI 10.1101/2020.04.08.031203V3]

He XC, 2014, PLOS ONE, V9, DOI 10.1371/journal.pone.0105373
Hikmet F, 2020, MOL SYST BIOL, V16, DOI 10.15252/msb.20209610
Hoffmann Markus, 2021, EBioMedicine, V65, P103255, DOI [10.1101/2020.08.05.237651, 10.1016/j.ebiom.2021.103255]
Hoffmann M, 2010, J GEN VIROL, V91, P2782, DOI 10.1099/vir.0.023978-0
Irving AT, 2021, NATURE, V589, P363, DOI 10.1038/s41586-020-03128-0
Koch J, 2021, EMBO J, V40, DOI 10.15252/embj.2021107821
Koopmans M, 2021, LANCET INFECT DIS, V21, P18, DOI [10.1016/1473-3099(20)30912-9, 10.1016/S1473-3099(20)30912-9]
Kurth A, 2012, PLOS ONE, V7, DOI 10.1371/journal.pone.0038688
Lau SKP, 2020, EMERG INFECT DIS, V26, P2961, DOI 10.3201/eid2612.202308
Lecis R, 2019, VIRUS GENES, V55, P60, DOI 10.1007/s11262-018-1614-8
Lee S, 2018, MICROB ECOL, V75, P174, DOI 10.1007/s00248-017-1033-8
Lelli D, 2013, VIRUSES-BASEL, V5, P2679, DOI 10.3390/v5112679
Lu RJ, 2020, LANCET, V395, P565, DOI 10.1016/S0140-6736(20)30251-8
Martin-Sancho L, 2021, MOL CELL, V81, P2656, DOI 10.1016/j.molcel.2021.04.008
Mendenhall IH, 2019, VIRUSES-BASEL, V11, DOI 10.3390/v11040356
Murakami S, 2020, EMERG INFECT DIS, V26, P3025, DOI 10.3201/eid2612.203386
Murgolo N, 2021, PLOS PATHOG, V17, DOI 10.1371/journal.ppat.1009225
Ogando NS, 2020, J GEN VIROL, V101, P925, DOI 10.1099/jgv.0.001453
Olival KJ, 2020, PLOS PATHOG, V16, DOI 10.1371/journal.ppat.1008758
Onabajo OO, 2020, NAT GENET, V52, P1283, DOI 10.1038/s41588-020-00731-9
Patterson E I, 2020, Nat Commun, V11, P6231, DOI [10.1038/s41467-020-20097-0, 10.1101/2020.07.21.214346]
Pikula Jiri, 2017, Veterinary Clinics of North America Exotic Animal Practice, V20, P665, DOI 10.1016/j.cvex.2016.11.013
Puelles VG, 2020, NEW ENGL J MED, V383, P590, DOI [10.1056/NEJMc2011400, 10.1056/NEJMc2013400]
Puray-Chavez M, 2021, CELL REP, V36, DOI [10.1016/j.celrep.2021.109364, 10.1101/2021.03.01.433431]
Qian ZH, 2013, PLOS ONE, V8, DOI 10.1371/journal.pone.0076469
Ramakrishnan Muthannan Andavar, 2016, World J Virol, V5, P85, DOI 10.5501/wjv.v5.i2.85
Rebendenne A, 2021, J VIROL, V95, DOI 10.1128/JVI.02415-20
Ren L., 2020, BIORXIV, DOI DOI 10.1101/2020.06.30.175778V1
Richter M, 2014, J GEN VIROL, V95, P2216, DOI 10.1099/vir.0.065995-0
Ruiz-Aravena M, 2022, NAT REV MICROBIOL, V20, P299, DOI 10.1038/s41579-021-00652-2
Schlottau K, 2020, LANCET MICROBE, V1, pE218, DOI 10.1016/S2666-5247(20)30089-6
Shaw AE, 2017, PLOS BIOL, V15, DOI 10.1371/journal.pbio.2004086

Straková P, 2017, INFECT GENET EVOL, V48, P127, DOI 10.1016/j.meegid.2016.12.025
Stukalov A, 2021, NATURE, V594, P246, DOI 10.1038/s41586-021-03493-4
Temmam S., 2021, Res. Sq, DOI DOI 10.21203/RS.3.RS-871965/V1
Temmam S, 2022, NATURE, V604, P330, DOI 10.1038/s41586-022-04532-4
Wacharapluesadee S, 2021, NAT COMMUN, V12, DOI 10.1038/s41467-021-21240-1
Yan H, 2021, NAT ECOL EVOL, V5, P600, DOI 10.1038/s41559-021-01407-1
Yin X, 2021, CELL REP, V34, DOI 10.1016/j.celrep.2020.108628
Yohe LR, 2019, JOVE-J VIS EXP, DOI 10.3791/59505
Zhang Qi, 2020, bioRxiv, DOI [10.1038/s41421-020-00222-5, 10.1101/2020.07.14.202549]
Zhou Hong, 2020, Curr Biol, V30, P2196, DOI 10.1016/j.cub.2020.05.023
Zhou J, 2020, NAT MED, V26, P1077, DOI 10.1038/s41591-020-0912-6
Zhou P, 2020, NATURE, V579, P270, DOI [10.1038/s41586-020-2012-7, 10.1038/s41586-020-2951-z]
Zhou P, 2016, P NATL ACAD SCI USA, V113, P2696, DOI 10.1073/pnas.1518240113
Zhou P, 2014, PLOS ONE, V9, DOI 10.1371/journal.pone.0103875
Zhou SR, 2021, NAT MED, V27, DOI 10.1038/s41591-021-01281-1
NR 81
TC 9
Z9 10
U1 1
U2 20
PU AMER SOC MICROBIOLOGY
PI WASHINGTON
PA 1752 N ST NW, WASHINGTON, DC 20036-2904 USA
SN 0022-538X
EI 1098-5514
J9 J VIROL
JI J. Virol.
PD JUL 27
PY 2022
VL 96
IS 14
DI 10.1128/jvi.00608-22
EA JUL 2022
PG 22
WC Virology
WE Science Citation Index Expanded (SCI-EXPANDED)
SC Virology
GA 3G8SA
UT WOS:000823133000002
PM 35862713
OA Green Published, Green Submitted
DA 2024-10-21
ER

PT J

AU Lilley, TM
   Ruokolainen, L
   Meierjohann, A
   Kanerva, M
   Stauffer, J
   Laine, VN
   Atosuo, J
   Lilius, EM
   Nikinmaa, M
AF Lilley, T. M.
   Ruokolainen, L.
   Meierjohann, A.
   Kanerva, M.
   Stauffer, J.
   Laine, V. N.
   Atosuo, J.
   Lilius, E. -M.
   Nikinmaa, M.
TI Resistance to oxidative damage but not immunosuppression by organic tin
   compounds in natural populations of Daubenton's bats (<i>Myotis
   daubentonii</i>)
SO COMPARATIVE BIOCHEMISTRY AND PHYSIOLOGY C-TOXICOLOGY & PHARMACOLOGY
LA English
DT Article
DE Chiroptera; TBT; Contaminants; Trophic transfer; Complement;
   Anti-oxidant enzymes
ID CHIRONOMUS-RIPARIUS; ARCHIPELAGO SEA; TRIBUTYLTIN CHLORIDE;
   ENVIRONMENTAL LEVELS; ANTIOXIDANT ENZYMES; ORGANOTIN COMPOUNDS;
   EXPOSURE; PROTEIN; STRESS; CELL
AB The acute toxicity of organic tin compounds (OTCs) has been studied in detail. However, due to their complex nature, very little is known about species-specific methods of accumulation and consequences for food-webs. Chironomids, on which e.g. Daubenton's bats feed, may act as vectors for the transport of organic tin compounds from aquatic to terrestrial ecosystems. Bats are prone to environmental toxins because of their longevity and their ecological role as top predators. Organic tin compounds are associated with increased formation of reactive oxygen species and associated oxidative damage as well as suppression of immune function. The present paper investigates whether the OTC, tributyltin (TBT) and its metabolite, dibutyltin (DBT), accumulate in natural populations of Daubenton's bats and whether TBT-associated effects are seen in general body condition, redox balance, redox enzyme activities, associated oxidative damage of red blood cells and complement function. We discovered the concentration of bat fur DBT correlated with local marine sediment TBT concentrations. However, we did not find a correlation between the explanatory factors, bat fur DBT and marine sediment TBT concentrations, and several physiological and physical response variables apart from complement activity. Higher DBT concentrations resulted in weaker complement activity and thus a weaker immune response. Although the observed physiological effects in the present study were not strongly correlated to,butyltin

concentrations in fur or sediment, the result is unique for natural populations so far and raises interesting questions for future ecotoxicological studies. (C) 2013 Elsevier Inc. All rights reserved.
C1 [Lilley, T. M.; Kanerva, M.; Stauffer, J.; Laine, V. N.; Nikinmaa, M.] Univ Turku, Biol Sect, SF-20500 Turku, Finland.
[Ruokolainen, L.] Univ Helsinki, Dept Biosci, Helsinki, Finland.
[Meierjohann, A.] Abo Akdemi, Organisk Kemi, Turku, Finland.
[Atosuo, J.; Lilius, E. -M.] Univ Turku, Dept Biochem & Food Chem, SF-20500 Turku, Finland.
C3 University of Turku; University of Helsinki; University of Turku
RP Lilley, TM (corresponding author), Univ Turku, Biol Sect, SF-20500 Turku, Finland.
EM tmlill@utu.fi
RI Nikinmaa, Mikko/AAZ-2803-2021; Lilley, Thomas/F-2236-2015; Laine, Veronika/B-7869-2014
OI Lilley, Thomas/0000-0001-5864-4958; Kanerva, Mirella/0000-0002-2463-2497; Laine, Veronika/0000-0002-4516-7002; Ruokolainen, Lasse/0000-0003-0951-9100; Nikinmaa, Mikko/0000-0003-0593-1018; Atosuo, Timo Janne/0000-0002-1100-4838
FU Maj and Tor Nessling Foundation; Academy of Finland
FX We thank Anna Kald and Mia Klaver for the help in the laboratory. The study was funded by Maj and Tor Nessling Foundation. LR was funded by the Academy of Finland.
CR Adema-Hannes R, 2008, ENVIRON TOXICOL CHEM, V27, P2131, DOI 10.1897/07-369.1
Alamdari DH, 2005, FREE RADICAL BIO MED, V39, P1362, DOI 10.1016/j.freeradbiomed.2005.06.023
ALZIEU C, 1986, MAR POLLUT BULL, V17, P494, DOI 10.1016/0025-326X(86)90636-3
[Anonymous], 2009, HDB BATS EUROPE NW A
[Anonymous], 2005, BAT MIGRATION EUROPE
Antizar-Ladislao B, 2008, ENVIRON INT, V34, P292, DOI 10.1016/j.envint.2007.09.005
Appel KE, 2004, DRUG METAB REV, V36, P763, DOI 10.1081/DMR-200033490
Atosuo J, 2011, EUR J CLIN INVEST, V41, P37
Bao VWW, 2011, MAR POLLUT BULL, V62, P1147, DOI 10.1016/j.marpolbul.2011.02.041
BOARD PG, 1981, FEBS LETT, V124, P163, DOI 10.1016/0014-5793(81)80127-5
BRADFORD MM, 1976, ANAL BIOCHEM, V72, P248, DOI 10.1016/0003-2697(76)90527-3
Brunet-Rossinni AK, 2004, MECH AGEING DEV, V125, P11, DOI 10.1016/j.mad.2003.09.003
Chen XJ, 2008, J THERM ANAL CALORIM, V94, P779, DOI 10.1007/s10973-007-8851-8
COHEN G, 1963, BIOCHEMISTRY-US, V2, P1420, DOI 10.1021/bi00906a038
Cooke GM, 2004, FOOD CHEM TOXICOL, V42, P211, DOI 10.1016/j.fct.2003.09.003
Corsini E, 1996, J INVEST DERMATOL, V107, P720, DOI

10.1111/1523-1747.ep12365608
Fent K, 1996, CRIT REV TOXICOL, V26, P3, DOI 10.3109/10408449609089891
FISH RH, 1976, J ORGANOMET CHEM, V118, P41, DOI 10.1016/S0022-328X(00)80816-5
Flavin DA, 2001, ACTA THERIOL, V46, P43, DOI 10.1007/BF03192415
Frouin H, 2008, AQUAT TOXICOL, V90, P243, DOI 10.1016/j.aquatox.2008.09.005
Gennari A, 2000, TOXICOL APPL PHARM, V169, P185, DOI 10.1006/taap.2000.9076
GIBBS PE, 1986, J MAR BIOL ASSOC UK, V66, P767, DOI 10.1017/S0025315400048414
Granlund K, 2005, J HYDROL, V304, P251, DOI 10.1016/j.jhydrol.2004.07.033
Gregory LA, 2003, J BIOL CHEM, V278, P32157, DOI 10.1074/jbc.M305175200
Halliwell B., 2000, FREE RADICAL BIO MED, V3rd
Helminen H, 1998, ENVIRON MODELL SOFTW, V13, P511, DOI 10.1016/S1364-8152(98)00058-9
Hill DA, 2005, MAMMAL REV, V35, P116, DOI 10.1111/j.1365-2907.2005.00058.x
Hoch M, 2001, APPL GEOCHEM, V16, P719, DOI 10.1016/S0883-2927(00)00067-6
Iwata H, 1997, APPL ORGANOMET CHEM, V11, P257
Jumppanen K, 1994, DEV STATE ARCHIPELAG
Kannan K, 1997, MAR POLLUT BULL, V34, P203, DOI 10.1016/S0025-326X(96)00146-4
Kilpi MK, 2009, DEV COMP IMMUNOL, V33, P1102, DOI 10.1016/j.dci.2009.06.007
Koivula MJ, 2010, ENVIRON POLLUT, V158, P2359, DOI 10.1016/j.envpol.2010.03.013
Lilley T, 2012, ECOTOXICOLOGY, V21, P1333, DOI 10.1007/s10646-012-0887-2
Lilley TM, 2012, ENVIRON TOXICOL CHEM, V31, P1781, DOI 10.1002/etc.1878
Lilley TM, 2012, ENVIRON SCI TECHNOL, V46, P7382, DOI 10.1021/es300536t
Looser PW, 2000, ENVIRON SCI TECHNOL, V34, P5165, DOI 10.1021/es001253v
McAllister BG, 2003, AQUAT TOXICOL, V65, P309, DOI 10.1016/S0166-445X(03)00154-1
Ministry of Environment, 2002, CONS PLAN FINN BALT
Misumi I, 2009, ARCH ENVIRON CON TOX, V56, P229, DOI 10.1007/s00244-008-9187-5
Nesci S, 2011, BIOCHIMIE, V93, P1157, DOI 10.1016/j.biochi.2011.04.008
Nikinmaa M., 1990, Vertebrate Red Blood Cells
NOLTMANN E, 1961, J BIOL CHEM, V236, P1225
NYHOLM ERJK S., 1965, ANN ZOOL FENN, V2, P77
Parsons KN, 2003, ANIM CONSERV, V6, P283, DOI 10.1017/S1367943003003342
Pavlikova N, 2011, J TOXICOL ENV HEAL A, V74, P478, DOI 10.1080/15287394.2011.550558
RAFFRAY M, 1991, ARCH TOXICOL, V65, P135, DOI 10.1007/BF02034940

Reinke NB, 2006, J COMP PHYSIOL B, V176, P729, DOI 10.1007/s00360-006-0094-7
RIVAS G, 1992, BIOCHEMISTRY-US, V31, P11707, DOI 10.1021/bi00162a006
Salmon AB, 2009, FASEB J, V23, P2317, DOI 10.1096/fj.08-122523
Schmidt K, 2005, AQUAT TOXICOL, V71, P49, DOI 10.1016/j.aquatox.2004.10.012
Senior P, 2005, P ROY SOC B-BIOL SCI, V272, P2467, DOI 10.1098/rspb.2005.3237
Senthilkumar K, 2001, ENVIRON SCI POLLUT R, V8, P35, DOI 10.1007/BF02987293
Sinha P, 2006, ENVIRON TOXICOL CHEM, V25, P2533, DOI 10.1897/05-548R.1
Socci DJ, 1999, EXP NEUROL, V155, P109, DOI 10.1006/exnr.1998.6969
Song L, 2005, BIOPHYS J, V88, P2350, DOI 10.1529/biophysj.104.053579
Stenalt E, 1998, ECOTOX ENVIRON SAFE, V40, P212, DOI 10.1006/eesa.1998.1687
Strand J, 2005, SCI TOTAL ENVIRON, V350, P72, DOI 10.1016/j.scitotenv.2005.02.039
Sun HW, 2001, WATER AIR SOIL POLL, V125, P55, DOI 10.1023/A:1005255713030
Suzuki N, 2006, LIFE SCI, V78, P2533, DOI 10.1016/j.lfs.2005.10.004
SUZUKI T, 1985, EXP ANIM TOKYO, V34, P353, DOI 10.1538/expanim1978.34.4_353
Taenzler V, 2007, ECOTOXICOLOGY, V16, P221, DOI 10.1007/s10646-006-0117-x
Tiunov MP, 2007, ACTA CHIROPTEROL, V9, P538, DOI 10.3161/1733-5329(2007)9[538:SMIMPC]2.0.CO;2
Vaughan N, 1997, MAMMAL REV, V27, P77, DOI 10.1111/j.1365-2907.1997.tb00373.x
Vogt C, 2007, CHEMOSPHERE, V67, P2192, DOI 10.1016/j.chemosphere.2006.12.025
Voigt CC, 2010, ECOLOGY, V91, P2908, DOI 10.1890/09-2232.1
Wada H, 2010, ECOTOXICOLOGY, V19, P1277, DOI 10.1007/s10646-010-0513-0
Walters DM, 2008, ECOL APPL, V18, P1835, DOI 10.1890/08-0354.1
Wilhelm D, 2007, COMP BIOCHEM PHYS C, V146, P214, DOI 10.1016/j.cbpc.2006.11.015
Ymparistoministerio, 2007, ORG TIN SUOM VES
NR 70
TC 30
Z9 31
U1 0
U2 54
PU ELSEVIER SCIENCE INC
PI NEW YORK
PA STE 800, 230 PARK AVE, NEW YORK, NY 10169 USA
SN 1532-0456
EI 1878-1659
J9 COMP BIOCHEM PHYS C
JI Comp. Biochem. Physiol. C-Toxicol. Pharmacol.

PD APR
PY 2013
VL 157
IS 3
BP 298
EP 305
DI 10.1016/j.cbpc.2013.01.003
PG 8
WC Biochemistry & Molecular Biology; Endocrinology & Metabolism;
Toxicology; Zoology
WE Science Citation Index Expanded (SCI-EXPANDED)
SC Biochemistry & Molecular Biology; Endocrinology & Metabolism;
Toxicology; Zoology
GA 124FS
UT WOS:000317449800006
PM 23369694
DA 2024-10-21
ER

PT J
AU Agueda-Pinto, A
Castro, LFC
Esteves, PJ
AF Agueda-Pinto, Ana
Castro, L. Filipe C.
Esteves, Pedro J.
TI The evolution of S100A7: an unusual gene expansion in Myotis bats
SO BMC EVOLUTIONARY BIOLOGY
LA English
DT Article
DE S100A7 (psoriasin); Chiroptera; Gene duplication; Model of concerted and
birth-and-death evolution
ID CALCIUM-BINDING PROTEINS; EPIDERMAL DIFFERENTIATION; MOLECULAR
PHYLOGENETICS; ATOPIC-DERMATITIS; PSORIASIN S100A7; HUMAN SKIN;
EXPRESSION; FAMILY; COMPLEX; DUPLICATIONS
AB BackgroundThe S100A7 gene, also called psoriasin, was first described as an upregulated protein in psoriatic skin. For the past years, the importance of this protein as a key effector of innate immunity has been clearly established, not only due to its importance protecting against bacteria skin insult in humans, but also because of its important role in amplifying inflammatory processes. Given the importance of S100A7 in host defense, S100A7 genes have been mostly studied in humans. Here we provide a detailed analysis of the evolution of the gene family encoding for the S100A7 protein in mammals.ResultsExamination of several mammalian genomes revealed an unexpected variation in the copy number of S100A7. Among the most representative mammalian groups, we report that multiple events of duplication, gene loss and high mutation rates are shaping the evolution of this gene family. An unexpected result comes from Myotis species (order Chiroptera), where we found an outstanding S100A7 gene radiation, resulting in more than 10 copies in M. lucifugus and 5 copies in M. brandtii. These findings suggest a unique adaptive road in these species and are suggestive of special

role of this protein in their immune system.ConclusionsWe found
different evolutionary histories among different mammalian groups.
Overall, our results suggest that this gene family is evolving under
the birth-and-death model of evolution. To our knowledge, this work
represents the first detailed analysis of phylogenetic relationships
of S100A7 within mammals and therefore will pave the way to further
clarify their unique function in the immune system.
C1 [Agueda-Pinto, Ana; Esteves, Pedro J.] Univ Porto, CIBIO InBio,
Ctr Invest Biodiversidade & Recursos Genet, Rua Padre Armando
Quintas, P-4485661 Vairao, Portugal.
[Agueda-Pinto, Ana; Castro, L. Filipe C.; Esteves, Pedro J.] Univ
Porto, Dept Biol, Fac Ciencias, Rua Campo Alegre S-N, P-4169007
Porto, Portugal.
[Castro, L. Filipe C.] Univ Porto, CIIMAR CIMAR, Ctr
Interdisciplinar Invest Marinha & Ambiental, Ave Gen Norton Matos S-
N, P-4450208 Matosinhos, Portugal.
[Esteves, Pedro J.] CESPU, Inst Invest & Formacao Avancada
Ciencias & Tecnol, Rua Cent Gandra 1317, P-4585116 Gandra, Portugal.
C3 Universidade do Porto; Universidade do Porto; Universidade do
Porto
RP Esteves, PJ (corresponding author), Univ Porto, CIBIO InBio, Ctr
Invest Biodiversidade & Recursos Genet, Rua Padre Armando Quintas,
P-4485661 Vairao, Portugal.; Esteves, PJ (corresponding author),
Univ Porto, Dept Biol, Fac Ciencias, Rua Campo Alegre S-N, P-4169007
Porto, Portugal.
EM pjesteves@cibio.up.pt
RI Castro, Filipe/AAA-2197-2022; Esteves, Pedro/A-7537-2010
OI Esteves, Pedro/0000-0002-6055-8298; Castro, Luis
Filipe/0000-0001-7697-386X
FU FCT - Foundation for Science and Technology [SFRH/BD/128752/2017,
IF/00376/2015]; Norte Portugal Regional Operational Programme
(NORTE2020), under the PORTUGAL 2020 Partnership Agreement,
through the
European Regional Development Fund (ERDF) [NORTE-01-0145-
FEDER-000007];
Fundação para a Ciência e a Tecnologia [SFRH/BD/128752/2017]
Funding
Source: FCT
FX FCT - Foundation for Science and Technology supported the
doctoral
fellowship of AAP (ref. SFRH/BD/128752/2017) and the investigator
grant
of PJE (IF/00376/2015). This article is a result of the project
NORTE-01-0145-FEDER-000007, supported by Norte Portugal Regional
Operational Programme (NORTE2020), under the PORTUGAL 2020
Partnership
Agreement, through the European Regional Development Fund (ERDF).
The
funders had no role in the design of the study, collection,
analysis and
interpretation of data, and in writing the manuscript.
CR Algermissen B, 1996, ARCH DERMATOL RES, V288, P426, DOI 10.1007/
BF02505229
[Anonymous], 1999, NUCL ACIDS S SER

[Anonymous], 1991, WORLD LIST MAMMALIAN
Boniface K, 2005, J IMMUNOL, V174, P3695, DOI 10.4049/jimmunol.174.6.3695
Donato R, 2001, INT J BIOCHEM CELL B, V33, P637, DOI 10.1016/S1357-2725(01)00046-2
Eckert RL, 2004, J INVEST DERMATOL, V123, P23, DOI 10.1111/j.0022-202X.2004.22719.x
Foote M, 1999, SCIENCE, V283, P1310, DOI 10.1126/science.283.5406.1310
Fritz G., 2011, ENCY INORGANIC BIOIN, DOI DOI 10.1002/9781119951438.EIBC0511
Gläser R, 2009, J INVEST DERMATOL, V129, P641, DOI 10.1038/jid.2008.268
Gläser R, 2005, NAT IMMUNOL, V6, P57, DOI 10.1038/ni1142
Guttman-Yassky E, 2008, J IMMUNOL, V181, P7420, DOI 10.4049/jimmunol.181.10.7420
Hahn Y, 2007, MOL BIOL EVOL, V24, P2203, DOI 10.1093/molbev/msm146
Handunnetthi L, 2010, GENES IMMUN, V11, P99, DOI 10.1038/gene.2009.83
HOFFMANN HJ, 1994, J INVEST DERMATOL, V103, P370, DOI 10.1111/1523-1747.ep12395202
Hoofer SR, 2003, ACTA CHIROPTEROL, V5, P1, DOI 10.3161/001.005.s101
Huang Q, 2015, INT J MOL MED, V35, P587, DOI 10.3892/ijmm.2014.2047
Innan H, 2010, NAT REV GENET, V11, P97, DOI 10.1038/nrg2689
Jia GL, 2003, CHINESE SCI BULL, V48, P1179, DOI 10.1007/BF03183931
Jiao H, 2018, MOL ECOL
Karev GP, 2006, MOL B INT U, P65
Kemp T.S., 2005, The Origin and Evolution of Mammals
Kulski JK, 2003, J MOL EVOL, V56, P397, DOI 10.1007/s00239-002-2410-5
Kumar S, 2016, MOL BIOL EVOL, V33, P1870, DOI [10.1093/molbev/msw054, 10.1093/molbev/msv279]
Kwek JHL, 2013, MOL PHYLOGENET EVOL, V69, P4, DOI 10.1016/j.ympev.2013.05.005
Lack JB, 2010, J MAMMAL, V91, P976, DOI 10.1644/09-MAMM-A-192.1
Lan X, 2016, SCIENCE, V352, P1009, DOI 10.1126/science.aad8411
Leclerc E, 2009, BBA-MOL CELL RES, V1793, P993, DOI 10.1016/j.bbamcr.2008.11.016
Lee KC, 2007, J INVEST DERMATOL, V127, P945, DOI 10.1038/sj.jid.5700663
Luis AD, 2013, P ROY SOC B-BIOL SCI, V280, DOI 10.1098/rspb.2012.2753
MADSEN P, 1991, J INVEST DERMATOL, V97, P701, DOI 10.1111/1523-1747.ep12484041
Magadum S, 2013, J GENET, V92, P155, DOI 10.1007/s12041-013-0212-8
Marenholz I, 2004, BIOCHEM BIOPH RES CO, V322, P1111, DOI 10.1016/j.bbrc.2004.07.096
Martin DP, 2010, BIOINFORMATICS, V26, P2462, DOI 10.1093/bioinformatics/btq467

Mikkelsen TS, 2007, NATURE, V447, P167, DOI 10.1038/nature05805
Mischke D, 1996, J INVEST DERMATOL, V106, P989, DOI 10.1111/1523-1747.ep12338501
Mühldorfer K, 2013, ZOONOSES PUBLIC HLTH, V60, P93, DOI 10.1111/j.1863-2378.2012.01536.x
Nei M, 2005, ANNU REV GENET, V39, P121, DOI 10.1146/annurev.genet.39.073003.112240
NEI M, 2015, P NATL ACAD SCI USA, V94, P7799
Neves F, 2019, IMMUNOGENETICS
Olival KJ, 2017, NATURE, V546, P646, DOI 10.1038/nature22975
Pavlovich SS, 2018, CELL, V173, P1098, DOI 10.1016/j.cell.2018.03.070
Platt RN, 2016, MOBILE DNA-UK, V7, DOI 10.1186/s13100-016-0071-y
Pond SLK, 2005, BIOINFORMATICS, V21, P2531, DOI 10.1093/bioinformatics/bti320
Rastogi S, 2005, BMC EVOL BIOL, V5, DOI 10.1186/1471-2148-5-28
Rogozin IB, 2014, GENETICS RES INT, V2014, P8
Ruedi M, 2001, MOL PHYLOGENET EVOL, V21, P436, DOI 10.1006/mpev.2001.1017
Salama I, 2008, EJSO-EUR J SURG ONC, V34, P357, DOI 10.1016/j.ejso.2007.04.009
Schad J, 2016, IMMUNOGENETICS, V68, P783, DOI 10.1007/s00251-016-0940-z
Schad J, 2012, IMMUNOGENETICS, V64, P535, DOI 10.1007/s00251-012-0609-1
Schaer J, 2013, P NATL ACAD SCI USA, V110, P17415, DOI 10.1073/pnas.1311016110
Schafer BW, 1996, TRENDS BIOCHEM SCI, V21, P134, DOI 10.1016/0968-0004(96)10020-7
Schrago CG, 2003, MOL BIOL EVOL, V20, P1620, DOI 10.1093/molbev/msg172
Schröder JM, 2006, CELL MOL LIFE SCI, V63, P469, DOI 10.1007/s00018-005-5364-0
Schwensow N, 2007, HEREDITY, V99, P265, DOI 10.1038/sj.hdy.6800993
Shang X, 2008, GENET SEL EVOL, V40, P449, DOI 10.1051/gse:2008013
SIMMONS E, 2005, ORDER CHIROPTERA MAM, P1
TAMURA K, 1993, MOL BIOL EVOL, V10, P512, DOI 10.1093/oxfordjournals.molbev.a040023
Tan JQ, 1996, J INVEST DERMATOL, V107, P5, DOI 10.1111/1523-1747.ep12294284
THOMPSON JD, 1994, NUCLEIC ACIDS RES, V22, P4673, DOI 10.1093/nar/22.22.4673
Wertheim JO, 2015, MOL BIOL EVOL, V32, P820, DOI 10.1093/molbev/msu400
Wolf R, 2006, J INVEST DERMATOL, V126, P1600, DOI 10.1038/sj.jid.5700210
Wolk K, 2004, IMMUNITY, V21, P241, DOI 10.1016/j.immuni.2004.07.007
Wong S, 2007, REV MED VIROL, V17, P67, DOI 10.1002/rmv.520
Yang ZH, 2007, MOL BIOL EVOL, V24, P1586, DOI 10.1093/molbev/msm088
Zackular JP, 2015, J BIOL CHEM, V290, P18991, DOI 10.1074/jbc.R115.645085

   Zahn LM, 2016, SCIENCE, V352, P949
   Zimmer DB, 2013, CELL CALCIUM, V53, P170, DOI 10.1016/
j.ceca.2012.11.006
NR 67
TC 7
Z9 8
U1 0
U2 13
PU BMC
PI LONDON
PA CAMPUS, 4 CRINAN ST, LONDON N1 9XW, ENGLAND
SN 1471-2148
J9 BMC EVOL BIOL
JI BMC Evol. Biol.
PD MAY 14
PY 2019
VL 19
AR 102
DI 10.1186/s12862-019-1433-0
PG 11
WC Evolutionary Biology; Genetics & Heredity
WE Science Citation Index Expanded (SCI-EXPANDED)
SC Evolutionary Biology; Genetics & Heredity
GA HY3SZ
UT WOS:000468048500001
PM 31088346
OA Green Published, gold
DA 2024-10-21
ER

PT J
AU CHAKRABORTY, AK
   CHAKRAVARTY, AK
AF CHAKRABORTY, AK
   CHAKRAVARTY, AK
TI PLAQUE-FORMING CELL ASSAY FOR ANTIBODY SECRETING CELLS IN THE BAT
   PTEROPUS-GIGANTEUS
SO INDIAN JOURNAL OF EXPERIMENTAL BIOLOGY
LA English
DT Article
AB Humoral immune response of the bat, P. giganteus, immunized with
sheep erythrocytes was studied by plaque forming cell assay.
Homologous serum is a better source of complement for the formation
of plaques than the guinea pig or rabbit complement. The degree of
immunity depends on the amount of antigen injected as in other
species. Higher doses of antigen induce early response. Specificity
of antibody for lysing a particular type of erythrocyte antigen was
also observed.
C1 UNIV N BENGAL, CTR LIFE SCI, IMMUNOL & CELL BIOL LAB, DARJEELING
734430, INDIA.
CR CHAKRAVARTY AK, 1982, UNPUB INDIAN J EXP B
   CUNNINGHAM AJ, 1968, IMMUNOLOGY, V14, P599
   FRIEDMAN H, 1965, P SOC EXP BIOL MED, V118, P1176
   FRIEDMAN H, 1969, IMMUNOLOGICAL TOLERA, P104

   HATTEN BA, 1968, J IMMUNOL, V101, P141
   Ivanyi J, 1969, Curr Top Microbiol Immunol, V49, P114
   KANAKAMBIKA P, 1972, J IMMUNOL, V109, P415
   KERCKHAERT JA, 1974, ANN INST PASTEUR IMM, VC125, P559
   MACKANESS GB, 1974, J EXP MED, V139, P543, DOI 10.1084/
jem.139.3.543
   SHALABY M R, 1973, Differentiation, V1, P167, DOI 10.1111/
j.1432-0436.1973.tb00110.x
   SULKIN SE, 1966, AM J TROP MED HYG, V15, P418, DOI 10.4269/
ajtmh.1966.15.418
NR 11
TC 6
Z9 6
U1 0
U2 1
PU NATL INST SCIENCE COMMUNICATION-NISCAIR
PI NEW DELHI
PA DR K S KRISHNAN MARG, PUSA CAMPUS, NEW DELHI 110 012, INDIA
SN 0019-5189
EI 0975-1009
J9 INDIAN J EXP BIOL
JI Indian J. Exp. Biol.
PY 1983
VL 21
IS 1
BP 5
EP 7
PG 3
WC Biology
WE Science Citation Index Expanded (SCI-EXPANDED)
SC Life Sciences & Biomedicine - Other Topics
GA QK557
UT WOS:A1983QK55700002
PM 6354927
DA 2024-10-21
ER

PT J
AU Woon, AP
   Boyd, V
   Todd, S
   Smith, I
   Klein, R
   Woodhouse, IB
   Riddell, S
   Crameri, G
   Bingham, J
   Wang, LF
   Purcell, AW
   Middleton, D
   Baker, ML
AF Woon, Amanda P.
   Boyd, Victoria
   Todd, Shawn

Smith, Ina
Klein, Reuben
Woodhouse, Isaac B.
Riddell, Sarah
Crameri, Gary
Bingham, John
Wang, Lin-Fa
Purcell, Anthony W.
Middleton, Deborah
Baker, Michelle L.
TI Acute experimental infection of bats and ferrets with Hendra virus:
Insights into the early host response of the reservoir host and susceptible model species
SO PLOS PATHOGENS
LA English
DT Article
ID NIPAH-VIRUS; PTEROPID BATS; GENE ONTOLOGY; INNATE; HENIPAVIRUSES; TRANSMISSION; PATHOGENESIS; CYTOSCAPE; SOFTWARE; PLATFORM
AB Author summary
Bats are natural reservoirs for a number of viruses, including HeV that cause severe disease in humans and other susceptible hosts. We examined acute HeV infection in pteropid bats, compared to ferrets, a species that develops fulminating disease following exposure to HeV, similar to humans. Analysis of HeV replication and transcription of innate immune genes was performed at 12, 36 and 60 hpe and global proteomics was performed on tissues at 60 hpe to obtain insight into the mechanisms responsible for innocuous (bats) compared to fatal (ferrets) HeV infection. We confirmed that both animal species had become infected on the basis of detection of viral RNA in bat lung (60 hpe) and ferret lung, lymph node, spleen, heart and intestine (36 and/or 60 hpe). Analysis of the transcription of IFNs and CXCL10, combined with global proteomics analysis revealed differences in the activation of the immune response between bats and ferrets, consistent with the difference in the control of viral replication and the development of pathology associated with disease between the two species. This study represents the first in vivo comparison between bats and a susceptible host and contributes important information on the kinetics and control of HeV in these two model species.
Bats are the natural reservoir host for a number of zoonotic viruses, including Hendra virus (HeV) which causes severe clinical disease in humans and other susceptible hosts. Our understanding of the ability of bats to avoid clinical disease following infection with viruses such as HeV has come predominantly from in vitro studies focusing on innate immunity. Information on the early host response to infection in vivo is lacking and there is no comparative data on responses in bats compared with animals that succumb to disease. In this study, we examined the sites of HeV replication and the immune response of infected Australian black flying foxes and ferrets at 12, 36 and 60 hours post exposure (hpe). Viral antigen was detected at 60 hpe in bats and was confined to the lungs whereas in ferrets there was evidence of widespread viral RNA and antigen by 60 hpe. The mRNA expression of IFNs revealed antagonism of type I

and III IFNs and a significant increase in the chemokine, CXCL10, in bat lung and spleen following infection. In ferrets, there was an increase in the transcription of IFN in the spleen following infection. Liquid chromatography tandem mass spectrometry (LC-MS/MS) on lung tissue from bats and ferrets was performed at 0 and 60 hpe to obtain a global overview of viral and host protein expression. Gene Ontology (GO) enrichment analysis of immune pathways revealed that six pathways, including a number involved in cell mediated immunity were more likely to be upregulated in bat lung compared to ferrets. GO analysis also revealed enrichment of the type I IFN signaling pathway in bats and ferrets. This study contributes important comparative data on differences in the dissemination of HeV and the first to provide comparative data on the activation of immune pathways in bats and ferrets in vivo following infection.
C1 [Woon, Amanda P.; Purcell, Anthony W.] Monash Univ, Dept Biochem & Mol Biol, Clayton, Vic, Australia.
[Woon, Amanda P.; Purcell, Anthony W.] Monash Univ, Biomed Discovery Inst, Clayton, Vic, Australia.
[Woon, Amanda P.] Immunocore Ltd, Oxford, England.
[Boyd, Victoria; Todd, Shawn; Smith, Ina; Klein, Reuben; Crameri, Gary; Baker, Michelle L.] CSIRO, Australian Anim Hlth Lab, Hlth & Biosecur Business Unit, Geelong, Vic, Australia.
[Woodhouse, Isaac B.] Univ Oxford, John Radcliffe Hosp, WIMM, Human Immunol Unit,MRC, Oxford, England.
[Woodhouse, Isaac B.] Hudson Inst Med Search, Ctr Innate Immun & Infect Dis, Clayton, Vic, Australia.
[Riddell, Sarah; Bingham, John; Middleton, Deborah] CSIRO, Australian Anim Hlth Lab, Geelong, Vic, Australia.
[Wang, Lin-Fa] Duke Natl Univ, Singapore Med Sch, Programme Emerging Infect Dis, Singapore, Singapore.
C3 Monash University; Monash University; Commonwealth Scientific & Industrial Research Organisation (CSIRO); University of Oxford; Commonwealth Scientific & Industrial Research Organisation (CSIRO);
National University of Singapore
RP Purcell, AW (corresponding author), Monash Univ, Dept Biochem & Mol Biol, Clayton, Vic, Australia.; Purcell, AW (corresponding author), Monash Univ, Biomed Discovery Inst, Clayton, Vic, Australia.; Baker, ML (corresponding author), CSIRO, Australian Anim Hlth Lab, Hlth & Biosecur Business Unit, Geelong, Vic, Australia.
EM Anthony.Purcell@monash.edu; Michelle.Baker@csiro.au
RI Smith, Ina/H-7225-2013; Todd, Shawn/JJE-5658-2023; Baker, Michelle/AAX-9171-2020; Purcell, Anthony/AAH-1942-2019; Bingham, John/H-8591-2013; Baker, Michelle/C-9694-2013
OI Bingham, John/0000-0003-2360-3827; Todd, Shawn/0009-0009-2218-7842;
Baker, Michelle/0000-0002-7993-9971; Woodhouse, Isaac/0000-0002-8221-7697; Woon, Amanda/0000-0002-4781-0356
FU Australian Research Council Future Fellowship [FT110100234]; Commonwealth Scientific and Industrial Research Organization Chief Executive Officer Science Leaders Award; National Health and Medical Research Council (NHMRC) of Australia [APP1042602, APP1084283];

NHMRC
   [APP1137739]; Australian postgraduate award; Australian Research
Council
   [FT110100234] Funding Source: Australian Research Council
FX This work was supported in part by an Australian Research Council
Future
   Fellowship FT110100234 (to MLB), a Commonwealth Scientific and
   Industrial Research Organization Chief Executive Officer Science
Leaders
   Award (to L-FW) and project grants from the National Health and
Medical
   Research Council (NHMRC) of Australia (APP1042602 to AWP, L-FW
and MLB
   and APP1084283 to AWP). AWP is supported by a NHMRC Principal
research
   fellowship (APP1137739). APW was supported by an Australian
postgraduate
   award. The funders had no role in study design, data collection
and
   analysis, decision to publish, or preparation of the manuscript.
CR Almeida MF, 2005, EPIDEMIOL INFECT, V133, P523, DOI 10.1017/
S0950268804003656
   [Anonymous], 2017, EUR GERIATR MED, V8, pS98
   Bindea G, 2009, BIOINFORMATICS, V25, P1091, DOI 10.1093/
bioinformatics/btp101
   Bossart KN, 2009, PLOS PATHOG, V5, DOI 10.1371/
journal.ppat.1000642
   Boyle EI, 2004, BIOINFORMATICS, V20, P3710, DOI 10.1093/
bioinformatics/bth456
   Cameron CM, 2008, J VIROL, V82, P11308, DOI 10.1128/JVI.00691-08
   Carolan LA, 2014, J VIROL METHODS, V205, P38, DOI 10.1016/
j.jviromet.2014.04.014
   Chambers MC, 2012, NAT BIOTECHNOL, V30, P918, DOI 10.1038/
nbt.2377
   Chua KB, 2002, MICROBES INFECT, V4, P145, DOI 10.1016/
S1286-4579(01)01522-2
   Clayton BA, 2013, ZOONOSES PUBLIC HLTH, V60, P69, DOI 10.1111/
j.1863-2378.2012.01501.x
   Cogswell-Hawkinson A, 2012, J VIROLOGY
   Cox J, 2008, NAT BIOTECHNOL, V26, P1367, DOI 10.1038/nbt.1511
   Dups J, 2014, VIROL J, V11, DOI 10.1186/1743-422X-11-102
   Epstein JH, 2013, PLOS ONE, V8, DOI 10.1371/journal.pone.0067584
   Field HE, 2016, CURR OPIN VIROL, V16, P120, DOI 10.1016/
j.coviro.2016.02.004
   Government Q, 2019, SUMM HENDR VIR INC H
   Halpin K, 2000, J GEN VIROL, V81, P1927, DOI
10.1099/0022-1317-81-8-1927
   Halpin K, 2011, AM J TROP MED HYG, V85, P946, DOI 10.4269/
ajtmh.2011.10-0567
   Hooper P, 2001, MICROBES INFECT, V3, P315, DOI 10.1016/
S1286-4579(01)01385-5
   Johara MY, 2001, EMERG INFECT DIS, V7, P439
   Keiser NW, 2015, AM J RESP CELL MOL, V52, P683, DOI 10.1165/
rcmb.2014-0250OC

Leon AJ, 2018, PLOS NEGLECT TROP D, V12, DOI 10.1371/journal.pntd.0006343
Liu ML, 2011, CYTOKINE GROWTH F R, V22, P121, DOI 10.1016/j.cytogfr.2011.06.001
Lo MK, 2010, VIROLOGY, V404, P78, DOI 10.1016/j.virol.2010.05.005
Lo MK, 2009, J GEN VIROL, V90, P398, DOI 10.1099/vir.0.007294-0
Maines TR, 2012, J INFECT DIS, V205, P474, DOI 10.1093/infdis/jir768
Marsh GA, 2012, CURR OPIN VIROL, V2, P242, DOI 10.1016/j.coviro.2012.03.006
Mathieu C, 2012, J VIROL, V86, P10766, DOI 10.1128/JVI.01203-12
Mathieu C, 2012, PLOS ONE, V7, DOI 10.1371/journal.pone.0032157
McColl KA, 2002, AUST VET J, V80, P636, DOI 10.1111/j.1751-0813.2002.tb10973.x
Middleton DJ, 2002, J COMP PATHOL, V126, P124, DOI 10.1053/jcpa.2001.0532
Pallister J, 2011, VACCINE, V29, P5623, DOI 10.1016/j.vaccine.2011.06.015
Perez-Riverol Y, 2019, NUCLEIC ACIDS RES, V47, pD442, DOI 10.1093/nar/gky1106
Rosen BH, 2018, AM J RESP CRIT CARE, V197, P1308, DOI 10.1164/rccm.201708-1616OC
Roxas BAP, 2008, BMC BIOINFORMATICS, V9, DOI 10.1186/1471-2105-9-187
Shannon P, 2003, GENOME RES, V13, P2498, DOI 10.1101/gr.1239303
Smith IL, 2001, J VIROL METHODS, V98, P33, DOI 10.1016/S0166-0934(01)00354-8
Tisoncik-Go J, 2016, CELL HOST MICROBE, V19, P254, DOI 10.1016/j.chom.2016.01.002
Tyanova S, 2016, NAT METHODS, V13, P731, DOI [10.1038/nmeth.3901, 10.1038/NMETH.3901]
Virtue ER, 2011, PLOS ONE, V6, DOI 10.1371/journal.pone.0022488
Wibawa H, 2013, COMP IMMUNOL MICROB, V36, P175, DOI 10.1016/j.cimid.2012.12.001
Williamson MM, 1998, AUST VET J, V76, P813, DOI 10.1111/j.1751-0813.1998.tb12335.x
Williamson MM, 2000, J COMP PATHOL, V122, P201, DOI 10.1053/jcpa.1999.0364
Wisniewski JR, 2009, NAT METHODS, V6, P359, DOI [10.1038/NMETH.1322, 10.1038/nmeth.1322]
Wynne JW, 2014, GENOME BIOL, V15, DOI 10.1186/s13059-014-0532-x
Wynne JW, 2016, J IMMUNOL, V196, P4468, DOI 10.4049/jimmunol.1502062
Young PL, 1996, EMERG INFECT DIS, V2, P239, DOI 10.3201/eid0203.960315
Zhou P, 2016, P NATL ACAD SCI USA, V113, P2696, DOI 10.1073/pnas.1518240113
Zhou P, 2011, J IMMUNOL, V186, P3138, DOI 10.4049/jimmunol.1003115
NR 49
TC 26
Z9 28
U1 0
U2 6

PU PUBLIC LIBRARY SCIENCE
PI SAN FRANCISCO
PA 1160 BATTERY STREET, STE 100, SAN FRANCISCO, CA 94111 USA
SN 1553-7366
EI 1553-7374
J9 PLOS PATHOG
JI PLoS Pathog.
PD MAR
PY 2020
VL 16
IS 3
AR e1008412
DI 10.1371/journal.ppat.1008412
PG 22
WC Microbiology; Parasitology; Virology
WE Science Citation Index Expanded (SCI-EXPANDED)
SC Microbiology; Parasitology; Virology
GA LA1HP
UT WOS:000523706200015
PM 32226041
OA gold, Green Published
DA 2024-10-21
ER

PT J
AU Fleischer, R
   Schmid, DW
   Wasimuddin
   Brändel, SD
   Rasche, A
   Corman, VM
   Drosten, C
   Tschapka, M
   Sommer, S
AF Fleischer, Ramona
   Schmid, Dominik W.
   Wasimuddin
   Braendel, Stefan D.
   Rasche, Andrea
   Corman, Victor M.
   Drosten, Christian
   Tschapka, Marco
   Sommer, Simone
TI Interaction between MHC diversity and constitution, gut microbiota and
   Astrovirus infections in a neotropical bat
SO MOLECULAR ECOLOGY
LA English
DT Article
DE Artibeus jamaicensis; astrovirus infection; gut microbiome; immunity;
   Major Histocompatibility Complex (MHC); zoonosis
ID CLASS-I; PHYLOGENETIC ANALYSIS; HOST; SELECTION; BACTERIA; ASSOCIATION;

VIRUSES; CELLS; FRAGMENTATION; MYCOPLASMAS
AB Astroviruses (AstVs) infect numerous mammalian species including reservoirs such as bats. Peptides encoded by the genes of the highly polymorphic Major Histocompatibility Complex (MHC) form the first line of host defence against pathogens. Aside from direct involvement in mounting adaptive immune responses, MHC class II genes are hypothesized to regulate gut commensal diversity and shape the production of immune-modulatory substances by microbes, indirectly affecting host susceptibility. Despite initial empirical evidence for the link between host MHC and the microbiota, associations among these factors remain largely unknown. To fill this gap, we examined MHC allelic diversity and constitution, the gut bacterial community and abundance pattern of a wild population of a neotropical bat (Artibeus jamaicensis) challenged by AstV infections. First, we show an age-dependent relationship between the host MHC class II diversity and constitution and the gut microbiota in AstV-uninfected bats. Crucially, these associations changed in AstV-infected bats. Additionally, we identify changes in the abundance of specific bacterial taxa linked to the presence of certain MHC supertypes and AstV infection. We suggest changes in the microbiota to be either a result of AstV infection or the MHC-mediated modulation of microbial communities. The latter could subsequently affect microbe-mediated immunity and resistance against AstV infection. Our results emphasize that the reciprocal nature of host immune genetics, gut microbial diversity and pathogen infection require attention, which are particularly important given their repercussions for disease susceptibility and severity in wild animal populations with a history of zoonotic spillover and frequent human contact.
C1 [Fleischer, Ramona; Schmid, Dominik W.; Braendel, Stefan D.; Tschapka, Marco; Sommer, Simone] Univ Ulm, Inst Evolutionary Ecol & Conservat Genom, Ulm, Germany.
[Wasimuddin] CSIR Ctr Cellular & Mol Biol, Hyderabad, India.
[Braendel, Stefan D.; Rasche, Andrea; Tschapka, Marco] Smithsonian Trop Res Inst, Ancon, Panama.
[Rasche, Andrea; Corman, Victor M.; Drosten, Christian] Charite Univ Med Berlin, Inst Virol, Berlin, Germany.
[Rasche, Andrea; Corman, Victor M.; Drosten, Christian] Free Univ, Berlin, Germany.
[Rasche, Andrea; Corman, Victor M.; Drosten, Christian] Humboldt Univ, Berlin, Germany.
[Rasche, Andrea; Corman, Victor M.; Drosten, Christian] Berlin Inst Hlth, Berlin, Germany.
[Corman, Victor M.; Drosten, Christian] Associated Partner Charite, German Ctr Infect Res DZIF, Berlin, Germany.
C3 Ulm University; Council of Scientific & Industrial Research (CSIR) -
India; CSIR - Centre for Cellular & Molecular Biology (CCMB);
Smithsonian Institution; Smithsonian Tropical Research Institute; Berlin
Institute of Health; Berlin Institute of Health; Free University of
Berlin; Humboldt University of Berlin; Charite Universitatsmedizin

Berlin; Free University of Berlin; Humboldt University of Berlin; Berlin
Institute of Health; Berlin Institute of Health; German Center for
Infection Research
RP Fleischer, R; Sommer, S (corresponding author), Univ Ulm, Inst
Evolutionary Ecol & Conservat Genom, Ulm, Germany.
EM ramona.fleischer@uni-ulm.de; simone.sommer@uni-ulm.de
RI , Wasimuddin/X-3899-2019; Drosten, Christian/AFF-8529-2022; Schmid,
Dominik/U-2475-2019; Corman, Victor Max/K-1319-2019; ,
Wasimuddin/N-9486-2014
OI Corman, Victor Max/0000-0002-3605-0136; Fleischer,
Ramona/0000-0003-1657-9347; , Wasimuddin/0000-0002-8314-8160; Melville,
Dominik Werner/0000-0001-8908-3882
FU German Science Foundation (DFG) [SO 428/9-1, SO 428/9-2, TS 81/7-1, TS
81/7-2, DR 772/8-1]
FX German Science Foundation (DFG), Grant/Award Number: SO 428/9-1, SO
428/9-2, TS 81/7-1, TS 81/7-2 and DR 772/8-1
CR Aleman FDD, 2019, PLOS PATHOG, V15, DOI 10.1371/journal.ppat.1007727
Apanius V, 2017, CRIT REV IMMUNOL, V37, P75, DOI 10.1615/CritRevImmunol.v37.i2-6.10
Atarashi K, 2011, SCIENCE, V331, P337, DOI 10.1126/science.1198469
Barko PC, 2018, J VET INTERN MED, V32, P9, DOI 10.1111/jvim.14875
Bauer MA, 2018, MICROB CELL, V5, P215, DOI 10.15698/mic2018.05.628
Bäumler AJ, 2016, NATURE, V535, P85, DOI 10.1038/nature18849
Belasen AM, 2019, FRONT ECOL EVOL, V6, DOI 10.3389/fevo.2018.00236
Benedetti F, 2020, MICROORGANISMS, V8, DOI 10.3390/microorganisms8091351
Bernardo-Cravo AP, 2020, TRENDS PARASITOL, V36, P616, DOI 10.1016/j.pt.2020.04.010
Bogdanoff WA, 2017, J VIROL, V91, DOI 10.1128/JVI.01859-16
Bolnick DI, 2017, NATURE, V546, P285, DOI 10.1038/nature22351
Bolnick DI, 2014, MOL ECOL, V23, P4831, DOI 10.1111/mec.12846
Bonneaud C, 2006, EVOLUTION, V60, P383, DOI 10.1111/j.0014-3820.2006.tb01114.x
Bosch A, 2014, CLIN MICROBIOL REV, V27, P1048, DOI 10.1128/CMR.00013-14
Brändel SD, 2020, BIOL CONSERV, V252, DOI 10.1016/j.biocon.2020.108792
Burbelo PD, 2011, PLOS ONE, V6, DOI 10.1371/journal.pone.0022576
Calisher CH, 2006, CLIN MICROBIOL REV, V19, P531, DOI 10.1128/CMR.00017-06
Canuti M, 2011, PLOS ONE, V6, DOI 10.1371/journal.pone.0029140
Cheng TL, 2017, J APPL ECOL, V54, P701, DOI 10.1111/1365-2664.12757
Cheng YY, 2012, IMMUNOGENETICS, V64, P525, DOI 10.1007/

s00251-012-0614-4
Chu DKW, 2008, J VIROL, V82, P9107, DOI 10.1128/JVI.00857-08
Cohen S, 2002, MOL BIOL EVOL, V19, P1870, DOI 10.1093/oxfordjournals.molbev.a004011
Cordey S, 2016, EMERG INFECT DIS, V22, P846, DOI [10.3201/eid2205.150807, 10.3201/eid2205.151807]
Cortez V, 2020, NAT COMMUN, V11, DOI 10.1038/s41467-020-15999-y
Cortez V, 2019, CURR OPIN VIROL, V37, P10, DOI 10.1016/j.coviro.2019.05.002
Cortez V, 2017, ANNU REV VIROL, V4, P327, DOI 10.1146/annurev-virology-101416-041742
D'Souza MP, 2019, PLOS PATHOG, V15, DOI 10.1371/journal.ppat.1007567
Del Real-Monroy M, 2017, MAMM BIOL, V84, P1, DOI 10.1016/j.mambio.2016.12.005
Del Real-Monroy M, 2014, ACTA CHIROPTEROL, V16, P301, DOI 10.3161/150811014X687260
Delport W, 2010, BIOINFORMATICS, V26, P2455, DOI 10.1093/bioinformatics/btq429
Dixon P, 2003, J VEG SCI, V14, P927, DOI 10.1111/j.1654-1103.2003.tb02228.x
Dominguez-Díaz C, 2019, FRONT CELL INFECT MI, V9, DOI 10.3389/fcimb.2019.00256
Donaldson GP, 2018, SCIENCE, V360, P795, DOI 10.1126/science.aaq0926
Donato C, 2017, VIRUSES-BASEL, V9, DOI 10.3390/v9050102
Drexler JF, 2011, EMERG INFECT DIS, V17, P449, DOI 10.3201/eid1703.100526
Duvallet C, 2017, NAT COMMUN, V8, DOI 10.1038/s41467-017-01973-8
Edgar RC, 2011, BIOINFORMATICS, V27, P2194, DOI 10.1093/bioinformatics/btr381
Eizaguirre C, 2012, NAT COMMUN, V3, DOI 10.1038/ncomms1632
Fackelmann G, 2021, COMMUN BIOL, V4, DOI 10.1038/s42003-021-02315-7
Finoketti F, 2019, ARCH VIROL, V164, P1015, DOI 10.1007/s00705-018-04129-1
Froeschke G, 2012, PLOS ONE, V7, DOI 10.1371/journal.pone.0031820
Gaboriau-Routhiau V, 2009, IMMUNITY, V31, P677, DOI 10.1016/j.immuni.2009.08.020
Ghosh TS, 2020, ELIFE, V9, DOI 10.7554/eLife.50240
Gibb R, 2020, NATURE, V584, P398, DOI 10.1038/s41586-020-2562-8
Gillingham MAF, 2021, MOL ECOL RESOUR, V21, P982, DOI 10.1111/1755-0998.13290
Griffith DM, 2016, J STAT SOFTW, V69, P1
Gruen JR, 1997, BLOOD, V90, P4252
Guzman-Bautista ER, 2020, CURR OPIN IMMUNOL, V66, P82, DOI 10.1016/j.coi.2020.05.010
Hair PS, 2010, MOL IMMUNOL, V47, P792, DOI 10.1016/j.molimm.2009.10.006
Halfvarson J, 2017, NAT MICROBIOL, V2, DOI 10.1038/nmicrobiol.2017.4
Hall JA, 2008, IMMUNITY, V29, P637, DOI 10.1016/j.immuni.2008.08.009
Handley C.O., 1991, SMITHSON CONTRIB ZOO, V511, P19

Hong J, 2022, BIOINFORMATICS, V38, P2389, DOI 10.1093/bioinformatics/btac127
Hooks KB, 2017, MBIO, V8, DOI 10.1128/mBio.01492-17
Hoyt JR, 2015, PLOS ONE, V10, DOI 10.1371/journal.pone.0121329
Ichinohe T, 2011, P NATL ACAD SCI USA, V108, P5354, DOI 10.1073/pnas.1019378108
Jacobson A, 2018, CELL HOST MICROBE, V24, P296, DOI 10.1016/j.chom.2018.07.002
Jombart T., 2015, A tutorial for discriminant analysis of principal components (DAPC) using adegenet 2.0. 0
Jombart T, 2010, BMC GENET, V11, DOI 10.1186/1471-2156-11-94
Karakus U, 2019, NATURE, V567, P109, DOI 10.1038/s41586-019-0955-3
Kaufman J, 2020, FRONT IMMUNOL, V11, DOI 10.3389/fimmu.2020.601089
Kaul A, 2017, FRONT MICROBIOL, V8, DOI 10.3389/fmicb.2017.02114
Kelley J, 2005, IMMUNOGENETICS, V56, P683, DOI 10.1007/s00251-004-0717-7
Khan AA, 2019, CELL REP, V29, P541, DOI 10.1016/j.celrep.2019.09.010
Khan MAW, 2019, PLOS ONE, V14, DOI 10.1371/journal.pone.0215946
Kohl C., 2020, SCI REP-UK, V10, P1
Kong G, 2020, NEUROBIOL DIS, V135, DOI 10.1016/j.nbd.2018.09.001
Kubinak JL, 2015, NAT COMMUN, V6, DOI 10.1038/ncomms9642
Kumánovics A, 2003, ANNU REV IMMUNOL, V21, P629, DOI 10.1146/annurev.immunol.21.090501.080116
Kumar S, 2018, MOL BIOL EVOL, V35, P1547, DOI 10.1093/molbev/msy096
Kurilshikov A, 2021, NAT GENET, V53, P156, DOI 10.1038/s41588-020-00763-1
Lathrop SK, 2011, NATURE, V478, P250, DOI 10.1038/nature10434
Lenz TL, 2013, P ROY SOC B-BIOL SCI, V280, DOI 10.1098/rspb.2013.0714
Lenz TL, 2011, EVOLUTION, V65, P2380, DOI 10.1111/j.1558-5646.2011.01288.x
Levy M, 2017, CURR OPIN MICROBIOL, V35, P8, DOI 10.1016/j.mib.2016.10.003
Lighten J, 2017, NAT COMMUN, V8, DOI 10.1038/s41467-017-01183-2
Lin L, 2017, BMC IMMUNOL, V18, DOI 10.1186/s12865-016-0187-3
Luckey D, 2019, SCI REP-UK, V9, DOI 10.1038/s41598-019-55503-1
Ma CF, 2011, CURR MICROBIOL, V63, P259, DOI 10.1007/s00284-011-9972-7
Magoc T, 2011, BIOINFORMATICS, V27, P2957, DOI 10.1093/bioinformatics/btr507
Mandal Siddhartha, 2015, Microbial Ecology in Health and Disease, V26, P27663, DOI 10.3402/mehd.v26.27663
Mao KR, 2018, NATURE, V554, P255, DOI 10.1038/nature25437
Mazmanian SK, 2005, CELL, V122, P107, DOI 10.1016/j.cell.2005.05.007
McKiernan SM, 2004, HEPATOLOGY, V40, P108, DOI 10.1002/hep.20261
McKnight DT, 2019, METHODS ECOL EVOL, V10, P389, DOI 10.1111/2041-210X.13115
McMurdie PJ, 2014, PLOS COMPUT BIOL, V10, DOI 10.1371/journal.pcbi.1003531

Meliopoulos VA, 2016, MBIO, V7, DOI 10.1128/mBio.01494-16
Meyer-Lucht Y, 2005, MOL ECOL, V14, P2233, DOI 10.1111/j.1365-294X.2005.02557.x
Montero BK, 2021, PLOS PATHOG, V17, DOI 10.1371/journal.ppat.1009675
Moser LA, 2007, J VIROL, V81, P11937, DOI 10.1128/JVI.00942-07
Munster VJ, 2016, SCI REP-UK, V6, DOI 10.1038/srep21878
Naccache SN, 2015, CLIN INFECT DIS, V60, P919, DOI 10.1093/cid/ciu912
Neefjes J, 2011, NAT REV IMMUNOL, V11, P823, DOI 10.1038/nri3084
Noguera JC, 2018, ROY SOC OPEN SCI, V5, DOI 10.1098/rsos.171743
Odamaki T, 2016, BMC MICROBIOL, V16, DOI 10.1186/s12866-016-0708-5
Olival KJ, 2017, NATURE, V546, P646, DOI 10.1038/nature22975
Pelaseyed T, 2014, IMMUNOL REV, V260, P8, DOI 10.1111/imr.12182
Pfeiffer JK, 2016, SCIENCE, V351, DOI 10.1126/science.aad5872
Pierini F, 2018, MOL BIOL EVOL, V35, P2145, DOI 10.1093/molbev/msy116
Piertney SB, 2006, HEREDITY, V96, P7, DOI 10.1038/sj.hdy.6800724
Pineaux M, 2020, MOL ECOL, V29, P3316, DOI 10.1111/mec.15551
Pond SLK, 2005, BIOINFORMATICS, V21, P2531, DOI 10.1093/bioinformatics/bti320
Pond SLK, 2005, BIOINFORMATICS, V21, P676, DOI 10.1093/bioinformatics/bti079
R Core Team, R: A language and environment for statistical computing
Radwan J, 2020, TRENDS GENET, V36, P298, DOI 10.1016/j.tig.2020.01.008
Reese AT, 2021, CURR BIOL, V31, DOI 10.1016/j.cub.2020.10.075
Risely A, 2021, NAT COMMUN, V12, DOI 10.1038/s41467-021-26298-5
Rivero-de Aguilar J, 2016, J AVIAN BIOL, V47, P669, DOI 10.1111/jav.00830
Roach SN, 2021, VIRUSES-BASEL, V13, DOI 10.3390/v13061127
Rooks MG, 2016, NAT REV IMMUNOL, V16, P341, DOI 10.1038/nri.2016.42
Rottem S, 2003, PHYSIOL REV, V83, P417, DOI 10.1152/physrev.00030.2002
Round JL, 2009, NAT REV IMMUNOL, V9, P313, DOI 10.1038/nri2515
Sandberg M, 1998, J MED CHEM, V41, P2481, DOI 10.1021/jm9700575
Santos PSC, 2017, MOL ECOL, V26, P2392, DOI 10.1111/mec.14035
Santos PSC, 2016, SCI REP-UK, V6, DOI 10.1038/srep38490
Schad J, 2011, HEREDITY, V107, P115, DOI 10.1038/hdy.2010.173
Schad J, 2012, IMMUNOGENETICS, V64, P535, DOI 10.1007/s00251-012-0609-1
Schmeller DS, 2020, BIODIVERS CONSERV, V29, P3095, DOI 10.1007/s10531-020-02021-6
Schwensow N, 2019, CONSERV GENET, V20, P65, DOI 10.1007/s10592-019-01143-x
Schwensow N, 2017, BIOL INVASIONS, V19, P1255, DOI 10.1007/s10530-016-1329-5
Sepil I, 2013, P ROY SOC B-BIOL SCI, V280, DOI 10.1098/rspb.2013.0134
Shipley R, 2019, TROP MED INFECT DIS, V4, DOI 10.3390/tropicalmed4010031

Sidney J, 1996, IMMUNOL TODAY, V17, P261, DOI 10.1016/0167-5699(96)80542-1
Silverman M, 2017, P NATL ACAD SCI USA, V114, P9671, DOI 10.1073/pnas.1712280114
Sommer Simone, 2005, Frontiers in Zoology, V2, P1, DOI 10.1186/1742-9994-2-16
Veech JA, 2013, GLOBAL ECOL BIOGEOGR, V22, P252, DOI 10.1111/j.1466-8238.2012.00789.x
Visconti A, 2019, NAT COMMUN, V10, DOI 10.1038/s41467-019-12476-z
Vu DL, 2017, VIRUSES-BASEL, V9, DOI 10.3390/v9020033
WAKELAND EK, 1990, IMMUNOL RES, V9, P115, DOI 10.1007/BF02918202
Wang C, 2016, BIOMATERIALS, V79, P88, DOI 10.1016/j.biomaterials.2015.11.040
Wang LF, 2019, CURR OPIN VIROL, V34, P79, DOI 10.1016/j.coviro.2018.12.007
Wasimuddin, 2019, SCI REP-UK, V9, DOI 10.1038/s41598-019-49829-z
Wasimuddin, 2018, ISME J, V12, P2883, DOI 10.1038/s41396-018-0239-1
Wegner KM, 2003, SCIENCE, V301, P1343, DOI 10.1126/science.1088293
Weiss S, 2017, MICROBIOME, V5, DOI 10.1186/s40168-017-0237-y
Westerdahl H, 2005, P ROY SOC B-BIOL SCI, V272, P1511, DOI 10.1098/rspb.2005.3113
Wohlgemuth N, 2019, INFECT GENET EVOL, V69, P30, DOI 10.1016/j.meegid.2019.01.009
Xu B, 2013, MOL BIOL EVOL, V30, P2723, DOI 10.1093/molbev/mst179
Yang ZH, 2007, MOL BIOL EVOL, V24, P1586, DOI 10.1093/molbev/msm088
Zhao Q, 2018, IMMUNOLOGY, V154, P28, DOI 10.1111/imm.12896
NR 140
TC 19
Z9 19
U1 3
U2 23
PU WILEY
PI HOBOKEN
PA 111 RIVER ST, HOBOKEN 07030-5774, NJ USA
SN 0962-1083
EI 1365-294X
J9 MOL ECOL
JI Mol. Ecol.
PD JUN
PY 2022
VL 31
IS 12
BP 3342
EP 3359
DI 10.1111/mec.16491
EA MAY 2022
PG 18
WC Biochemistry & Molecular Biology; Ecology; Evolutionary Biology
WE Science Citation Index Expanded (SCI-EXPANDED)
SC Biochemistry & Molecular Biology; Environmental Sciences & Ecology;

Evolutionary Biology
GA 1V8LT
UT WOS:000796267300001
PM 35510794
OA hybrid
DA 2024-10-21
ER

PT J
AU Janardhana, V
Tachedjian, M
Crameri, G
Cowled, C
Wang, LF
Baker, ML
AF Janardhana, Vijaya
Tachedjian, Mary
Crameri, Gary
Cowled, Chris
Wang, Lin-Fa
Baker, Michelle L.
TI Cloning, expression and antiviral activity of IFNγ from the Australian
fruit bat, <i>Pteropus alecto</i>
SO DEVELOPMENTAL AND COMPARATIVE IMMUNOLOGY
LA English
DT Article
DE Bats; Interferon gamma; Antiviral
ID HENDRA VIRUS-INFECTION; INTERFERON-GAMMA; IMMUNE-RESPONSE; SEQUENCE
ALIGNMENT; HUMAN-LEUKOCYTES; FLYING FOXES; EBOLA-VIRUS; GIGANTEUS;
HORSES; MORBILLIVIRUS
AB Bats are natural reservoir hosts to a variety of viruses, many of which cause morbidity and mortality in other mammals. Currently there is a paucity of information regarding the nature of the immune response to viral infections in bats, partly due to a lack of appropriate bat specific reagents. IFN gamma plays a key role in controlling viral replication and coordinating a response for long term control of viral infection. Here we describe the cloning and expression of IFN gamma from the Australian flying fox, Pteropus alecto and the generation of mouse monoclonal and chicken egg yolk antibodies specific to bat IFN gamma. Our results demonstrate that P. alecto IFN gamma is conserved with IFN gamma from other species and is induced in bat splenocytes following stimulation with T cell mitogens. P. alecto IFN gamma has antiviral activity on Semliki forest virus in cell lines from P. alecto and the microbat, Tadarida brasiliensis. Additionally recombinant bat IFN gamma was able to mitigate Hendra virus infection in P. alecto cells. These results provide the first evidence for an antiviral role for bat IFN gamma in vitro in addition to the application of important immunological reagents for further studies of bat antiviral immunity. Crown Copyright (c) 2011 Published by Elsevier Ltd. All rights reserved.
C1 [Janardhana, Vijaya; Tachedjian, Mary; Crameri, Gary; Cowled,

Chris; Wang, Lin-Fa; Baker, Michelle L.] CSIRO Livestock Ind,
Australian Anim Hlth Lab, Geelong, Vic 3220, Australia.
[Baker, Michelle L.] Univ New Mexico, Dept Biol, Ctr Evolutionary
& Theoret Immunol, Albuquerque, NM 87131 USA.
[Wang, Lin-Fa] Univ Melbourne, Dept Microbiol & Immunol,
Parkville, Vic 3010, Australia.
C3 Commonwealth Scientific & Industrial Research Organisation
(CSIRO);
University of New Mexico; University of Melbourne
RP Baker, ML (corresponding author), CSIRO Livestock Ind, Australian
Anim Hlth Lab, PO Bag 24, Geelong, Vic 3220, Australia.
EM Michelle.Baker@csiro.au
RI Crameri, Gary/H-8441-2013; lu, yuan/JZD-0832-2024; Cowled,
Christopher/H-5308-2013; Baker, Michelle/C-9694-2013
OI Cowled, Christopher/0000-0002-1969-9371; Wang,
Lin-Fa/0000-0003-2752-0535; Baker, Michelle/0000-0002-7993-9971
FU CSIRO CEO
FX The authors wish to acknowledge support from a CSIRO CEO Science
Leaders
award to L.-F.W. We thank Craig Smith, Hume Field, Jenn Barr,
Deborah
Middleton and Susanne Wilson for provision of bat tissues for
this
study, Matt Bruce for statistical analyses and John Lowenthal and
Peng
Zhou for critical reading of the manuscript.
CR Aljofan M, 2009, VIRUS RES, V142, P92, DOI 10.1016/
j.virusres.2009.01.014
Aljofan M, 2008, J VIROL METHODS, V149, P12, DOI 10.1016/
j.jviromet.2008.01.016
ALTSCHUL SF, 1990, J MOL BIOL, V215, P403, DOI 10.1016/
S0022-2836(05)80360-2
Andrew M, 2007, CYTOKINE, V40, P193, DOI 10.1016/
j.cyto.2007.09.010
Arnold K, 2006, BIOINFORMATICS, V22, P195, DOI 10.1093/
bioinformatics/bti770
Baker ML, 2010, IMMUNOGENETICS, V62, P173, DOI 10.1007/
s00251-010-0425-4
Boehm U, 1997, ANNU REV IMMUNOL, V15, P749, DOI 10.1146/
annurev.immunol.15.1.749
Calisher CH, 2006, CLIN MICROBIOL REV, V19, P531, DOI 10.1128/
CMR.00017-06
CHAKRABORTY AK, 1984, DEV COMP IMMUNOL, V8, P415, DOI
10.1016/0145-305X(84)90048-X
CHAKRAVARTY AK, 1987, DEV COMP IMMUNOL, V11, P649, DOI
10.1016/0145-305X(87)90053-X
Cowled C, 2012, DEV COMP IMMUNOL, V36, P657, DOI 10.1016/
j.dci.2011.11.008
Cowled C, 2011, DEV COMP IMMUNOL, V35, P7, DOI 10.1016/
j.dci.2010.07.006
Crameri G, 2009, PLOS ONE, V4, DOI 10.1371/journal.pone.0008266
DELEY M, 1980, EUR J IMMUNOL, V10, P877, DOI 10.1002/
eji.1830101113
FARRAR MA, 1993, ANNU REV IMMUNOL, V11, P571, DOI 10.1146/

annurev.iy.11.040193.003035
Field HE, 2007, AUST VET J, V85, P268, DOI 10.1111/j.1751-0813.2007.00170.x
FINTER NB, 1969, J GEN VIROL, V5, P419, DOI 10.1099/0022-1317-5-3-419
Gotoh B, 2002, REV MED VIROL, V12, P337, DOI 10.1002/rmv.357
GRIGGS ND, 1992, J IMMUNOL, V149, P517
HATTEN BA, 1968, J IMMUNOL, V101, P141
Janardhana V, 2007, J INTERF CYTOK RES, V27, P937, DOI 10.1089/jir.2007.0020
Kepler TB, 2010, BMC GENOMICS, V11, DOI 10.1186/1471-2164-11-444
Kumar S, 2004, BRIEF BIOINFORM, V5, P150, DOI 10.1093/bib/5.2.150
LAEMMLI UK, 1970, NATURE, V227, P680, DOI 10.1038/227680a0
Leroy EM, 2005, NATURE, V438, P575, DOI 10.1038/438575a
Leroy EM, 2009, VECTOR-BORNE ZOONOT, V9, P723, DOI 10.1089/vbz.2008.0167
MCMURRAY DN, 1979, J MAMMAL, V60, P576, DOI 10.2307/1380099
MURRAY K, 1995, SCIENCE, V268, P94, DOI 10.1126/science.7701348
PAUL BN, 1986, DEV COMP IMMUNOL, V10, P55, DOI 10.1016/0145-305X(86)90044-3
Rodriguez JJ, 2004, VIRAL IMMUNOL, V17, P210, DOI 10.1089/0882824041310568
ROTHEL JS, 1990, AUST VET J, V67, P134, DOI 10.1111/j.1751-0813.1990.tb07730.x
SAITOU N, 1987, MOL BIOL EVOL, V4, P406, DOI 10.1093/oxfordjournals.molbev.a040454
Savan R, 2009, CYTOKINE GROWTH F R, V20, P115, DOI 10.1016/j.cytogfr.2009.02.006
Schroder K, 2004, J LEUKOCYTE BIOL, V75, P163, DOI 10.1189/jlb.0603252
SLODOWSKI O, 1991, EUR J BIOCHEM, V202, P1133, DOI 10.1111/j.1432-1033.1991.tb16481.x
Subramaniam PS, 1999, J BIOL CHEM, V274, P403, DOI 10.1074/jbc.274.1.403
Suradhat S, 2001, VET IMMUNOL IMMUNOP, V83, P177, DOI 10.1016/S0165-2427(01)00389-0
Swanepoel R, 1996, EMERG INFECT DIS, V2, P321, DOI 10.3201/eid0204.960407
Than K.A., 1998, SAPONIN ADJUVANT COM
THOMPSON JD, 1994, NUCLEIC ACIDS RES, V22, P4673, DOI 10.1093/nar/22.22.4673
VANDENBROEK MF, 1995, J VIROL, V69, P4792, DOI 10.1128/JVI.69.8.4792-4796.1995
Virtue ER, 2011, PLOS ONE, V6, DOI 10.1371/journal.pone.0022488
Virtue ER, 2011, J VIROL, V85, P4031, DOI 10.1128/JVI.02412-10
Wellehan JFX, 2009, COMP IMMUNOL MICROB, V32, P379, DOI 10.1016/j.cimid.2007.11.002
WHEELOCK EF, 1965, SCIENCE, V149, P310, DOI 10.1126/science.149.3681.310
Williamson MM, 1998, AUST VET J, V76, P813, DOI 10.1111/j.1751-0813.1998.tb12335.x
Williamson MM, 2000, J COMP PATHOL, V122, P201, DOI 10.1053/jcpa.1999.0364
Wong S, 2007, REV MED VIROL, V17, P67, DOI 10.1002/rmv.520

   Wood PR, 2001, TUBERCULOSIS, V81, P147, DOI 10.1054/
tube.2000.0272
   Zhou P, 2011, PLOS ONE, V6, DOI 10.1371/journal.pone.0025385
   Zhou P, 2011, J IMMUNOL, V186, P3138, DOI 10.4049/
jimmunol.1003115
NR 51
TC 20
Z9 25
U1 0
U2 15
PU ELSEVIER SCI LTD
PI OXFORD
PA THE BOULEVARD, LANGFORD LANE, KIDLINGTON, OXFORD OX5 1GB, OXON,
ENGLAND
SN 0145-305X
EI 1879-0089
J9 DEV COMP IMMUNOL
JI Dev. Comp. Immunol.
PD MAR
PY 2012
VL 36
IS 3
BP 610
EP 618
DI 10.1016/j.dci.2011.11.001
PG 9
WC Fisheries; Immunology; Veterinary Sciences; Zoology
WE Science Citation Index Expanded (SCI-EXPANDED)
SC Fisheries; Immunology; Veterinary Sciences; Zoology
GA 904CI
UT WOS:000301170800014
PM 22093696
OA Green Published
DA 2024-10-21
ER

PT J
AU Chattopadhyay, B
   Garg, KM
   Ray, R
   Mendenhall, IH
   Rheindt, FE
AF Chattopadhyay, Balaji
   Garg, Kritika M.
   Ray, Rajasri
   Mendenhall, Ian H.
   Rheindt, Frank E.
TI Novel de Novo Genome of <i>Cynopterus brachyotis</i> Reveals
   Evolutionarily Abrupt Shifts in Gene Family Composition across
Fruit
   Bats
SO GENOME BIOLOGY AND EVOLUTION
LA English
DT Article

DE lesser short-nosed fruit bat; gene family evolution; immunity;
olfactory; histones
ID PHYLOGENOMIC ANALYSES; MARBURG VIRUS; ANNOTATION; ALIGNMENT;
IDENTIFICATION; MITOCHONDRIAL; ADAPTATION; PREDICTION; SOFTWARE;
PATTERNS
AB Major novel physiological or phenotypic adaptations often require accompanying modifications at the genic level. Conversely, the detection of considerable contractions and/or expansions of gene families can be an indicator of fundamental but unrecognized physiological change. We sequenced a novel fruit bat genome (Cynopterus brachyotis) and adopted a comparative approach to reconstruct the evolutionof fruitbats, mappingcontractions andexpansionsofgene families along their evolutionary history. Despite a radical change in life history as compared with other bats (e.g., loss of echolocation, large size, and frugivory), fruit bats have undergone surprisingly limited change in their genic composition, perhaps apart from a potentially novel gene family expansion relating to telomere protection and longevity. In sharp contrast, within fruit bats, the new Cynopterus genome bears the signal of unusual gene loss and gene family contraction, despite its similarmorphology and lifestyle to two othermajor fruit bat lineages. Most missing genes are regulatory, immune-related, and olfactory in nature, illustrating the diversity of genomic strategies employed by bats to contend with responses to viral infection and olfactory requirements. Our results underscore that significant fluctuations in gene family composition are not always associated with obvious examples of novel physiological and phenotypic adaptations but may often relate to less-obvious shifts in immune strategies.
C1 [Chattopadhyay, Balaji; Garg, Kritika M.; Rheindt, Frank E.] Natl Univ Singapore, Dept Biol Sci, Singapore, Singapore.
[Ray, Rajasri] Indian Inst Sci, Ctr Ecol Sci, Bangalore, Karnataka, India.
[Ray, Rajasri] Ctr Studies Ethnobiol Biodivers & Sustainabil CEi, Malda, W Bengal, India.
[Mendenhall, Ian H.] Duke NUS Med Sch, Programme Emerging Infect Dis, Singapore, Singapore.
C3 National University of Singapore; Indian Institute of Science (IISC) -
Bangalore; National University of Singapore
RP Chattopadhyay, B; Rheindt, FE (corresponding author), Natl Univ Singapore, Dept Biol Sci, Singapore, Singapore.
EM balaji@nus.edu.sg; dbsrfe@nus.edu.sg
RI Ray, Rajasri/IWD-5043-2023; Rheindt, Frank/D-7826-2014
OI Chattopadhyay, Balaji/0000-0002-4423-3127; Garg, Kritika
M./0000-0003-3510-3408
FU South East Asian Biodiversity Genomics (SEABIG) [WBS R-154-000-648646,
WBS R-154-000-648-733]
FX The authors thank Ben Fulton for help with CAFE analyses. This work was
supported by South East Asian Biodiversity Genomics (SEABIG) (Grant
Numbers WBS R-154-000-648646 and WBS R-154-000-648-733).

CR Ahn M, 2016, SCI REP-UK, V6, DOI 10.1038/srep21722
   Alberto FJ, 2018, NAT COMMUN, V9, DOI 10.1038/s41467-018-03206-y
   Almeida FC, 2011, BMC EVOL BIOL, V11, DOI
10.1186/1471-2148-11-281
   Altringham J.D., 1999, Bats: biology and behaviour
   ALTSCHUL SF, 1990, J MOL BIOL, V215, P403, DOI 10.1016/
S0022-2836(05)80360-2
   Amman BR, 2015, J WILDLIFE DIS, V51, P113, DOI
10.7589/2014-08-198
   [Anonymous], 2009, ECOLOGICAL BEHAV MET, DOI DOI
10.1016/0165-0270(79)90017-7
   Axelsson E, 2013, NATURE, V495, P360, DOI 10.1038/nature11837
   Banerjee A, 2017, SCI REP-UK, V7, DOI 10.1038/s41598-017-01513-w
   Bao ZR, 2002, GENOME RES, V12, P1269, DOI 10.1101/gr.88502
   Beltz L.A., 2017, Bats and Human Health: Ebola, SARS, Rabies and
Beyond
   Bernt M, 2013, MOL PHYLOGENET EVOL, V69, P313, DOI 10.1016/
j.ympev.2012.08.023
   Bhak Y, 2017, PLOS ONE, V12, DOI 10.1371/journal.pone.0180418
   Boyle EI, 2004, BIOINFORMATICS, V20, P3710, DOI 10.1093/
bioinformatics/bth456
   Campbell P, 2004, MOL PHYLOGENET EVOL, V33, P764, DOI 10.1016/
j.ympev.2004.06.019
   Campbell P, 2008, J MAMMAL, V89, P1351, DOI 10.1644/08-MAMM-
S-059.1
   Castoe TA, 2013, P NATL ACAD SCI USA, V110, P20645, DOI 10.1073/
pnas.1314475110
   Chattopadhyay B, 2016, BMC EVOL BIOL, V16, DOI 10.1186/
s12862-016-0599-y
   Chattopadhyay B, 2011, MAMM BIOL, V76, P290, DOI 10.1016/
j.mambio.2010.08.003
   Chong HT, 2009, NEUROL ASIA, V14, P73
   Covey E, 2005, ANAT REC PART A, V287A, P1103, DOI 10.1002/
ar.a.20254
   Dierckxsens N, 2017, NUCLEIC ACIDS RES, V45, DOI 10.1093/nar/
gkw955
   Dong D, 2017, MOL BIOL EVOL, V34, P20, DOI 10.1093/molbev/msw231
   Dong D, 2013, BMC GENOMICS, V14, DOI 10.1186/1471-2164-14-916
   Emms DM, 2015, GENOME BIOL, V16, DOI 10.1186/s13059-015-0721-2
   Enright AJ, 2002, NUCLEIC ACIDS RES, V30, P1575, DOI 10.1093/nar/
30.7.1575
   Foley NM, 2018, SCI ADV, V4, DOI 10.1126/sciadv.aao0926
   Foley NM, 2016, PHILOS T R SOC B, V371, DOI 10.1098/
rstb.2015.0140
   Garg KM, 2012, MOL ECOL, V21, P4093, DOI 10.1111/
j.1365-294X.2012.05665.x
   Gotz S, 2008, NUCLEIC ACIDS RES, V36, P3420, DOI 10.1093/nar/
gkn176
   Gurevich A, 2013, BIOINFORMATICS, V29, P1072, DOI 10.1093/
bioinformatics/btt086
   Han MV, 2013, MOL BIOL EVOL, V30, P1987, DOI 10.1093/molbev/
mst100
   Hayden S, 2014, MOL BIOL EVOL, V31, P917, DOI 10.1093/molbev/
msu043

Huerta-Cepas J, 2007, GENOME BIOL, V8, DOI 10.1186/gb-2007-8-6-r109
Huerta-Cepas J, 2016, MOL BIOL EVOL, V33, P1635, DOI 10.1093/molbev/msw046
Jarvis ED, 2014, SCIENCE, V346, P1320, DOI 10.1126/science.1253451
Jones MEB, 2015, VIRUSES-BASEL, V7, P3420, DOI 10.3390/v7072779
Kajitani R, 2014, GENOME RES, V24, P1384, DOI 10.1101/gr.170720.113
Kapusta A, 2017, P NATL ACAD SCI USA, V114, pE1460, DOI 10.1073/pnas.1616702114
Katoh K, 2013, MOL BIOL EVOL, V30, P772, DOI 10.1093/molbev/mst010
Kim EB, 2011, NATURE, V479, P223, DOI 10.1038/nature10533
Laing ED, 2018, EMERG INFECT DIS, V24, P122, DOI 10.3201/eid2401.170401
Lei M, 2016, SCI REP-UK, V6, DOI 10.1038/srep27726
Li H, 2011, NATURE, V475, P493, DOI 10.1038/nature10231
Li H, 2009, BIOINFORMATICS, V25, P1754, DOI 10.1093/bioinformatics/btp324
Lindblad-Toh K, 2011, NATURE, V478, P476, DOI 10.1038/nature10530
Liu L, 2017, P NATL ACAD SCI USA, V114, pE7282, DOI 10.1073/pnas.1616744114
Loayza D, 2003, NATURE, V423, P1013, DOI 10.1038/nature01688
Luo RB, 2012, GIGASCIENCE, V1, DOI 10.1186/2047-217X-1-18
Mani RS, 2017, EPIDEMIOL INFECT, V145, P1635, DOI 10.1017/S0950268817000310
Marcais G, 2011, BIOINFORMATICS, V27, P764, DOI 10.1093/bioinformatics/btr011
McCracken Gary F., 2000, P321, DOI 10.1016/B978-012195670-7/50009-6
Morgan CC, 2013, BMC EVOL BIOL, V13, DOI 10.1186/1471-2148-13-251
Ng JHJ, 2016, SCI REP-UK, V6, DOI 10.1038/srep21256
Page RDM, 1997, MOL PHYLOGENET EVOL, V7, P231, DOI 10.1006/mpev.1996.0390
Pavlovich SS, 2018, CELL, V173, P1098, DOI 10.1016/j.cell.2018.03.070
Platt RN, 2018, SYST BIOL, V67, P236, DOI 10.1093/sysbio/syx070
Price AL, 2005, BIOINFORMATICS, V21, pI351, DOI 10.1093/bioinformatics/bti1018
Qu YH, 2013, NAT COMMUN, V4, DOI 10.1038/ncomms3071
Sanderson MJ, 2003, BIOINFORMATICS, V19, P301, DOI 10.1093/bioinformatics/19.2.301
Schountz T, 2014, VIRUSES-BASEL, V6, P4880, DOI 10.3390/v6124880
Schuh AJ, 2017, NAT COMMUN, V8, DOI 10.1038/ncomms14446
Sharma V, 2018, NAT COMMUN, V9, DOI 10.1038/s41467-018-03667-1
Shaw TI, 2012, PLOS ONE, V7, DOI 10.1371/journal.pone.0048472
Simao FA, 2015, BIOINFORMATICS, V31, P3210, DOI 10.1093/bioinformatics/btv351
Smit A.F.A., 2015, REPEATMODELER OPEN 1
Smit A. F. A., 2015, REPEATMASKER OPEN 4, P2013
Smith JDL, 2013, GENOME, V56, P457, DOI 10.1139/gen-2013-0046
Stamatakis A, 2014, BIOINFORMATICS, V30, P1312, DOI 10.1093/bioinformatics/btu033

Stanke M, 2006, NUCLEIC ACIDS RES, V34, pW435, DOI 10.1093/nar/gkl200
Supek F, 2011, PLOS ONE, V6, DOI 10.1371/journal.pone.0021800
Teeling EC, 2005, SCIENCE, V307, P580, DOI 10.1126/science.1105113
Teeling EC, 2018, ANNU REV ANIM BIOSCI, V6, P23, DOI 10.1146/annurev-animal-022516-022811
Tsagkogeorga G, 2017, SCI REP-UK, V7, DOI 10.1038/s41598-017-00132-9
Tsagkogeorga G, 2013, CURR BIOL, V23, P2262, DOI 10.1016/j.cub.2013.09.014
Wang Y, 2015, NUCLEIC ACIDS RES, V43, pW78, DOI 10.1093/nar/gkv487
Wang ZB, 2008, NAT GENET, V40, P897, DOI 10.1038/ng.154
Waterhouse RM, 2018, MOL BIOL EVOL, V35, P543, DOI 10.1093/molbev/msx319
Wen M, 2018, GIGASCIENCE, V7, DOI 10.1093/gigascience/giy116
Xu HF, 2012, PLOS ONE, V7, DOI 10.1371/journal.pone.0052209
Yoon KB, 2016, MITOCHONDRIAL DNA A, V27, P2091, DOI 10.3109/19401736.2014.982571
Zdobnov EM, 2017, NUCLEIC ACIDS RES, V45, pD744, DOI 10.1093/nar/gkw1119
Zhang GJ, 2013, SCIENCE, V339, P456, DOI 10.1126/science.1230835
Zhou P, 2016, P NATL ACAD SCI USA, V113, P2696, DOI 10.1073/pnas.1518240113
Zubaid A., 2006, FUNCTIONAL EVOLUTION
NR 85
TC 13
Z9 14
U1 1
U2 7
PU OXFORD UNIV PRESS
PI OXFORD
PA GREAT CLARENDON ST, OXFORD OX2 6DP, ENGLAND
SN 1759-6653
J9 GENOME BIOL EVOL
JI Genome Biol. Evol.
PD APR
PY 2020
VL 12
IS 4
BP 259
EP 272
DI 10.1093/gbe/evaa030
PG 14
WC Evolutionary Biology; Genetics & Heredity
WE Science Citation Index Expanded (SCI-EXPANDED)
SC Evolutionary Biology; Genetics & Heredity
GA LV8QG
UT WOS:000538703000002
PM 32068833
OA Green Published, gold
DA 2024-10-21
ER

PT J
AU Guito, JC
Prescott, JB
Arnold, CE
Amman, BR
Schuh, AJ
Spengler, JR
Sealy, TK
Harmon, JR
Coleman-McCray, JD
Kulcsar, KA
Nagle, ER
Kumar, R
Palacios, GF
Sanchez-Lockhart, M
Towner, JS
AF Guito, Jonathan C.
Prescott, Joseph B.
Arnold, Catherine E.
Amman, Brian R.
Schuh, Amy J.
Spengler, Jessica R.
Sealy, Tara K.
Harmon, Jessica R.
Coleman-McCray, Joann D.
Kulcsar, Kirsten A.
Nagle, Elyse R.
Kumar, Raina
Palacios, Gustavo F.
Sanchez-Lockhart, Mariano
Towner, Jonathan S.
TI Asymptomatic Infection of Marburg Virus Reservoir Bats Is Explained by a
Strategy of Immunoprotective Disease Tolerance
SO CURRENT BIOLOGY
LA English
DT Article
ID REVERSE GENETICS SYSTEM; EGYPTIAN ROUSETTE BATS; INNATE IMMUNE-RESPONSE;
EBOLA-VIRUS; NONHUMAN-PRIMATES; ANGOLA INFECTION; EXPRESSION;
PATHOGENESIS; CELLS; GENERATE
AB Marburg virus (MARV) is among the most virulent pathogens of primates, including humans. Contributors to severe MARV disease include immune response suppression and inflammatory gene dysregulation ("cytokine storm''), leading to systemic damage and often death. Conversely, MARV causes little to no clinical disease in its reservoir host, the Egyptian rousette bat (ERB). Previous genomic and in vitro data suggest that a tolerant ERB immune response may underlie MARV avirulence, but no significant examination of this response in vivo yet exists. Here, using colony-bred ERBs inoculated with a bat isolate of MARV, we use species-specific antibodies and an immune gene probe array (NanoString) to temporally characterize the transcriptional host response at sites

of MARV replication relevant to primate pathogenesis and immunity, including CD14(+) monocytes/macrophages, critical immune response mediators, primary MARV targets, and skin at the inoculation site, where highest viral loads and initial engagement of antiviral defenses are expected. Our analysis shows that ERBs upregulate canonical antiviral genes typical of mammalian systems, such as ISG15, IFIT1, and OAS3, yet demonstrate a remarkable lack of significant induction of proinflammatory genes classically implicated in primate filoviral pathogenesis, including CCL8, FAS, and IL6. Together, these findings offer the first in vivo functional evidence for disease tolerance as an immunological mechanism by which the bat reservoir asymptomatically hosts MARV. More broadly, these data highlight factors determining disparate outcomes between reservoir and spillover hosts and defensive strategies likely utilized by bat hosts of other emerging pathogens, knowledge that may guide development of effective antiviral therapies.
C1 [Guito, Jonathan C.; Amman, Brian R.; Schuh, Amy J.; Spengler, Jessica R.; Sealy, Tara K.; Harmon, Jessica R.; Coleman-McCray, Joann D.; Towner, Jonathan S.] Ctr Dis Control & Prevent, Viral Special Pathogens Branch, Atlanta, GA 30329 USA.
[Prescott, Joseph B.] Robert Koch Inst, Ctr Biol Threats & Special Pathogens, Berlin, Germany.
[Arnold, Catherine E.] US Army Med Res Inst Infect Dis USAMRIID, Diagnost Syst Div, Ft Detrick, MD 21702 USA.
[Kulcsar, Kirsten A.] Univ Maryland, Sch Med, Dept Microbiol & Immunol, Baltimore, MD 21201 USA.
[Nagle, Elyse R.; Kumar, Raina; Palacios, Gustavo F.; Sanchez-Lockhart, Mariano] USAMRIID, Ctr Genome Sci, Ft Detrick, MD 21702 USA.
[Sanchez-Lockhart, Mariano] Univ Nebraska Med Ctr, Dept Pathol & Microbiol, Omaha, NE 68198 USA.
[Towner, Jonathan S.] Univ Georgia, Coll Vet Med, Dept Pathol, Athens, GA 30602 USA.
C3 Centers for Disease Control & Prevention - USA; Robert Koch Institute;
University System of Maryland; University of Maryland Baltimore; United
States Army Medical Research Institute of Infectious Diseases;
University of Nebraska System; University of Nebraska Medical Center;
University System of Georgia; University of Georgia
RP Towner, JS (corresponding author), Ctr Dis Control & Prevent, Viral Special Pathogens Branch, Atlanta, GA 30329 USA.; Sanchez-Lockhart, M (corresponding author), USAMRIID, Ctr Genome Sci, Ft Detrick, MD 21702 USA.; Sanchez-Lockhart, M (corresponding author), Univ Nebraska Med Ctr, Dept Pathol & Microbiol, Omaha, NE 68198 USA.; Towner, JS (corresponding author), Univ Georgia, Coll Vet Med, Dept Pathol, Athens, GA 30602 USA.
EM mariano.sanchez-lockhart.ctr@mail.mil; jit8@cdc.gov
RI Towner, Jonathan/ABC-3780-2020; Palacios, Gustavo/I-7773-2015
OI Prescott, Joseph/0000-0001-5718-5155; Palacios, Gustavo/0000-0001-5062-1938
FU Defense Threat Reduction Agency, Department of Defense [HDTRA1-14-1-0016]

FX The authors wish to thank Tatyana Klimova for her assistance with
critical reading of the manuscript. Opinions, interpretations,
conclusions, and recommendations are those of the authors and are not
necessarily endorsed by the U.S. Army or the Centers for Disease Control
and Prevention. This work was partially funded by the Defense Threat
Reduction Agency, Department of Defense (HDTRA1-14-1-0016).
CR Albariño CG, 2018, PLOS ONE, V13, DOI 10.1371/journal.pone.0201827
Albariño CG, 2015, VIROLOGY, V484, P259, DOI 10.1016/j.virol.2015.06.013
Albariño CG, 2013, VIROLOGY, V446, P230, DOI 10.1016/j.virol.2013.07.038
Amman BR, 2017, CURR TOP MICROBIOL, V411, P23, DOI 10.1007/82_2017_10
Amman BR, 2015, J WILDLIFE DIS, V51, P113, DOI 10.7589/2014-08-198
Arnold CE, 2018, VIRUSES-BASEL, V10, DOI 10.3390/v10110607
Ashley CL, 2019, FRONT IMMUNOL, V10, DOI 10.3389/fimmu.2019.02751
Ashley CL, 2019, VIRUSES-BASEL, V11, DOI 10.3390/v11030246
Baker ML, 2013, ZOONOSES PUBLIC HLTH, V60, P104, DOI 10.1111/j.1863-2378.2012.01528.x
Banerjee A, 2017, SCI REP-UK, V7, DOI 10.1038/s41598-017-01513-w
Brook CE, 2015, TRENDS MICROBIOL, V23, P172, DOI 10.1016/j.tim.2014.12.004
Caballero IS, 2016, BMC GENOMICS, V17, DOI 10.1186/s12864-016-3060-0
Connor JH, 2015, J VIROL, V89, P9865, DOI 10.1128/JVI.01142-15
Cross RW, 2018, J INFECT DIS, V218, pS486, DOI 10.1093/infdis/jiy455
De La Cruz-Rivera PC, 2018, J IMMUNOL, V200, P209, DOI 10.4049/jimmunol.1701214
Dubois A, 2017, VIRUS RES, V235, P67, DOI 10.1016/j.virusres.2017.04.004
Easterbrook JD, 2008, J MED VIROL, V80, P1308, DOI 10.1002/jmv.21213
Fernando L, 2015, J INFECT DIS, V212, pS234, DOI 10.1093/infdis/jiv095
Fritz EA, 2008, VIRAL IMMUNOL, V21, P355, DOI 10.1089/vim.2008.0023
Glaze ER, 2015, COMPARATIVE MED, V65, P241
Glennon NB, 2015, J VIROL, V89, P7550, DOI 10.1128/JVI.00302-15
Gómez JMM, 2016, SCI REP-UK, V6, DOI 10.1038/srep37796
Guivier E, 2010, INFECT GENET EVOL, V10, P1208, DOI 10.1016/j.meegid.2010.07.022
Hayman DTS, 2019, NAT MICROBIOL, V4, P728, DOI 10.1038/s41564-019-0430-9
Heberle H, 2015, BMC BIOINFORMATICS, V16, DOI 10.1186/s12859-015-0611-3
Helin AS, 2018, MOL IMMUNOL, V95, P64, DOI 10.1016/j.molimm.2018.01.012
Hensley LE, 2011, J INFECT DIS, V204, pS1021, DOI 10.1093/infdis/

jir339
Huang DW, 2009, NAT PROTOC, V4, P44, DOI 10.1038/nprot.2008.211
Huang DW, 2009, NUCLEIC ACIDS RES, V37, P1, DOI 10.1093/nar/gkn923
Hume A, 2018, J INFECT DIS, V218, pS403, DOI 10.1093/infdis/jiy473
Jones MEB, 2019, VIRUSES-BASEL, V11, DOI 10.3390/v11030214
Jones MEB, 2015, VIRUSES-BASEL, V7, P3420, DOI 10.3390/v7072779
Lee AK, 2015, BMC GENOMICS, V16, DOI 10.1186/s12864-015-2124-x
Lin KL, 2015, J VIROL, V89, P9875, DOI 10.1128/JVI.01147-15
Luo WJ, 2017, NUCLEIC ACIDS RES, V45, pW501, DOI 10.1093/nar/gkx372
Mandl JN, 2018, FRONT IMMUNOL, V9, DOI 10.3389/fimmu.2018.02112
McCarville JL, 2018, CURR OPIN IMMUNOL, V50, P88, DOI 10.1016/j.coi.2017.12.003
Menicucci AR, 2017, FRONT IMMUNOL, V8, DOI 10.3389/fimmu.2017.01372
Messaoudi I, 2015, NAT REV MICROBIOL, V13, P663, DOI 10.1038/nrmicro3524
Mi HY, 2019, NUCLEIC ACIDS RES, V47, pD419, DOI 10.1093/nar/gky1038
Mire CE, 2017, SCI TRANSL MED, V9, DOI 10.1126/scitranslmed.aai8711
Munster VJ, 2016, SCI REP-UK, V6, DOI 10.1038/srep21878
Neumann B, 2015, J LEUKOCYTE BIOL, V97, P19, DOI 10.1189/jlb.1HI0514-243R
Pavlovich SS, 2018, CELL, V173, P1098, DOI 10.1016/j.cell.2018.03.070
Periasamy P, 2019, FRONT IMMUNOL, V10, DOI 10.3389/fimmu.2019.00489
Prescott J, 2019, MSPHERE, V4, DOI 10.1128/mSphere.00728-19
Schnell MA, 2001, MOL THER, V3, P708, DOI 10.1006/mthe.2001.0330
Schountz T, 2017, FRONT IMMUNOL, V8, DOI 10.3389/fimmu.2017.01098
Schountz T, 2012, J VIROL, V86, P10015, DOI 10.1128/JVI.06875-11
Schuh AJ, 2019, J INFECT DIS, V219, P1716, DOI 10.1093/infdis/jiy733
Schuh AJ, 2017, NAT COMMUN, V8, DOI 10.1038/ncomms14446
Shaw AE, 2017, PLOS BIOL, V15, DOI 10.1371/journal.pbio.2004086
Speranza E, 2017, SCI REP-UK, V7, DOI 10.1038/s41598-017-15145-7
Speranza E, 2017, VACCINES-BASEL, V5, DOI 10.3390/vaccines5030030
Srithayakumar V, 2014, J GEN VIROL, V95, P16, DOI 10.1099/vir.0.053942-0
Storm N, 2018, VIRUSES-BASEL, V10, DOI 10.3390/v10020073
Towner JS, 2007, PLOS ONE, V2, DOI 10.1371/journal.pone.0000764
Towner JS, 2009, PLOS PATHOG, V5, DOI 10.1371/journal.ppat.1000536
Woon AP, 2020, PLOS PATHOG, V16, DOI 10.1371/journal.ppat.1008412
Yen BC, 2016, J VIROL, V90, P5108, DOI 10.1128/JVI.00191-16
Zhou P, 2016, P NATL ACAD SCI USA, V113, P2696, DOI 10.1073/pnas.1518240113
NR 61
TC 47
Z9 51
U1 0

U2 11
PU CELL PRESS
PI CAMBRIDGE
PA 50 HAMPSHIRE ST, FLOOR 5, CAMBRIDGE, MA 02139 USA
SN 0960-9822
EI 1879-0445
J9 CURR BIOL
JI Curr. Biol.
PD JAN 25
PY 2021
VL 31
IS 2
BP 257
EP +
DI 10.1016/j.cub.2020.10.015
EA JAN 2021
PG 19
WC Biochemistry & Molecular Biology; Biology; Cell Biology
WE Science Citation Index Expanded (SCI-EXPANDED)
SC Biochemistry & Molecular Biology; Life Sciences & Biomedicine - Other
Topics; Cell Biology
GA RB2SS
UT WOS:000631965600015
PM 33157026
OA hybrid
DA 2024-10-21
ER

PT J
AU Ng, JHJ
Tachedjian, M
Deakin, J
Wynne, JW
Cui, J
Haring, V
Broz, I
Chen, HL
Belov, K
Wang, LF
Baker, ML
AF Ng, Justin H. J.
Tachedjian, Mary
Deakin, Janine
Wynne, James W.
Cui, Jie
Haring, Volker
Broz, Ivano
Chen, Honglei
Belov, Katherine
Wang, Lin-Fa
Baker, Michelle L.
TI Evolution and comparative analysis of the bat MHC-I region
SO SCIENTIFIC REPORTS

LA English
DT Article
ID MAJOR HISTOCOMPATIBILITY COMPLEX; PEPTIDE LENGTH; FRUIT BATS;
MAXIMUM-LIKELIHOOD; RESERVOIR HOSTS; VIRUS-INFECTION; PTEROPID BATS;
ORGANIZATION; SEQUENCE; PSEUDOGENES
AB Bats are natural hosts to numerous viruses and have ancient origins, having diverged from other eutherian mammals early in evolution. These characteristics place them in an important position to provide insights into the evolution of the mammalian immune system and antiviral immunity. We describe the first detailed partial map of a bat (Pteropus alecto) MHC-I region with comparative analysis of the MHC-I region and genes. The bat MHC-I region is highly condensed, yet relatively conserved in organisation, and is unusual in that MHC-I genes are present within only one of the three highly conserved class I duplication blocks. We hypothesise that MHC-I genes first originated in the beta duplication block, and subsequently duplicated in a step-wise manner across the MHC-I region during mammalian evolution. Furthermore, bat MHC-I genes contain unique insertions within their peptide-binding grooves potentially affecting the peptide repertoire presented to T cells, which may have implications for the ability of bats to control infection without overt disease.
C1 [Ng, Justin H. J.; Tachedjian, Mary; Wynne, James W.; Baker, Michelle L.] CSIRO, Hlth & Biosecur Business Unit, Australian Anim Hlth Lab, Geelong, Vic 3220, Australia.
[Ng, Justin H. J.; Belov, Katherine] Univ Sydney, Fac Vet Sci, Sydney, NSW 2006, Australia.
[Ng, Justin H. J.; Cui, Jie; Wang, Lin-Fa] Duke Natl Univ Singapore, Sch Med, Program Emerging Infect Dis, Singapore 169857, Singapore.
[Deakin, Janine] Univ Canberra, Inst Appl Ecol, Canberra, ACT 2617, Australia.
[Haring, Volker; Broz, Ivano; Chen, Honglei] CSIRO, Australian Anim Hlth Lab, Geelong, Vic 3220, Australia.
C3 Commonwealth Scientific & Industrial Research Organisation (CSIRO);
University of Sydney; National University of Singapore; University of
Canberra; Commonwealth Scientific & Industrial Research Organisation
(CSIRO)
RP Ng, JHJ; Baker, ML (corresponding author), CSIRO, Hlth & Biosecur Business Unit, Australian Anim Hlth Lab, Geelong, Vic 3220, Australia.; Ng, JHJ (corresponding author), Univ Sydney, Fac Vet Sci, Sydney, NSW 2006, Australia.; Ng, JHJ (corresponding author), Duke Natl Univ Singapore, Sch Med, Program Emerging Infect Dis, Singapore 169857, Singapore.
EM justin.ng@duke-nus.edu.sg; michelle.baker@csiro.au
RI Chen, Honglei/GYA-4252-2022; Cui, Jie/ABB-3831-2020; Deakin, Janine/A-1941-2008; Tachedjian, Mary/H-4572-2013; Wynne, James/H-7957-2013; Baker, Michelle/C-9694-2013
OI Belov, Katherine/0000-0002-9762-5554; Cui, Jie/
0000-0001-8176-9951;

Deakin, Janine/0000-0002-1259-3531; Wang, Lin-Fa/
0000-0003-2752-0535;
Tachedjian, Mary/0000-0002-8936-6564; Wynne, James/
0000-0001-6846-757X;
Baker, Michelle/0000-0002-7993-9971
FU NIH Institutional Development Award Programme of the National Centre for
Research Resources [P20RR018754]; Australian Research Council Future
Fellowship [FT110100234]; Commonwealth Scientific and Industrial
Research Organisation Chief Executive Officer Science Leaders award;
Singaporean NRF Competitive Research Programme Grant
[NRF-CRP10-2012-05]; Australian Biosecurity Cooperative Research Centre
for Emerging Infectious Diseases (AB-CRC) Postgraduate Scholarship
FX We thank Drs Chionh Yok Teng, Chris Cowled and Peng Zhou for their
constructive advice. This work was supported in part by grants from the
NIH Institutional Development Award Programme of the National Centre for
Research Resources (P20RR018754, MLB), the Australian Research Council
Future Fellowship (FT110100234, MLB), the Commonwealth Scientific and
Industrial Research Organisation Chief Executive Officer Science Leaders
award (L-FW), and the Singaporean NRF Competitive Research Programme
Grant (NRF-CRP10-2012-05, L-FW). JHJN was supported by the Australian
Biosecurity Cooperative Research Centre for Emerging Infectious Diseases
(AB-CRC) Postgraduate Scholarship.
CR Adams EJ, 2013, ANNU REV IMMUNOL, V31, P529, DOI 10.1146/annurev-immunol-032712-095912
Alsop AE, 2005, CHROMOSOME RES, V13, P627, DOI 10.1007/s10577-005-0989-2
ALTSCHUL SF, 1990, J MOL BIOL, V215, P403, DOI 10.1016/S0022-2836(05)80360-2
Amadou C, 1999, IMMUNOGENETICS, V49, P362, DOI 10.1007/s002510050507
[Anonymous], 2008, Microbe
Beck S, 1999, NATURE, V401, P921, DOI 10.1038/44853
Bell MJ, 2009, MOL IMMUNOL, V46, P1911, DOI 10.1016/j.molimm.2008.12.003
Belov K, 2006, PLOS BIOL, V4, P317, DOI 10.1371/journal.pbio.0040046
BJORKMAN PJ, 1990, ANNU REV BIOCHEM, V59, P253, DOI 10.1146/annurev.biochem.59.1.253
BJORKMAN PJ, 1987, NATURE, V329, P512, DOI 10.1038/329512a0
Burge C, 1997, J MOL BIOL, V268, P78, DOI 10.1006/jmbi.1997.0951

Burrows JM, 2008, MOL IMMUNOL, V45, P1818, DOI 10.1016/j.molimm.2007.09.026
Calisher CH, 2006, CLIN MICROBIOL REV, V19, P531, DOI 10.1128/CMR.00017-06
Crameri G, 2009, PLOS ONE, V4, DOI 10.1371/journal.pone.0008266
Dirscherl H, 2014, DEV COMP IMMUNOL, V46, P11, DOI 10.1016/j.dci.2014.02.018
Dohm JC, 2007, GENOME BIOL, V8, DOI 10.1186/gb-2007-8-8-r175
Ekeruche-Makinde J, 2013, BLOOD, V121, P1112, DOI 10.1182/blood-2012-06-437202
Ellis SA, 2006, IMMUNOGENETICS, V57, P953, DOI 10.1007/s00251-005-0071-4
FELSENSTEIN J, 1981, J MOL EVOL, V17, P368, DOI 10.1007/BF01734359
FELSENSTEIN J, 1985, EVOLUTION, V39, P783, DOI 10.1111/j.1558-5646.1985.tb00420.x
Grimholt U, 2015, BMC EVOL BIOL, V15, DOI 10.1186/s12862-015-0309-1
Gustafson AL, 2003, CYTOGENET GENOME RES, V102, P189, DOI 10.1159/000075747
Halpin K, 1999, VET MICROBIOL, V68, P83, DOI 10.1016/S0378-1135(99)00063-2
Halpin K, 2011, AM J TROP MED HYG, V85, P946, DOI 10.4269/ajtmh.2011.10-0567
Hillary William, 2011, Microb Inform Exp, V1, P2, DOI 10.1186/2042-5783-1-2
Horton R, 2004, NAT REV GENET, V5, P889, DOI 10.1038/nrg1489
Jia GL, 2003, CHINESE SCI BULL, V48, P1179, DOI 10.1007/BF03183931
KASAHARA S, 1983, ANN GENET-PARIS, V26, P197
Kaufman J, 1999, IMMUNOL REV, V167, P101, DOI 10.1111/j.1600-065X.1999.tb01385.x
Kelley J, 2005, IMMUNOGENETICS, V56, P683, DOI 10.1007/s00251-004-0717-7
Kulski JK, 2002, IMMUNOL REV, V190, P95, DOI 10.1034/j.1600-065X.2002.19008.x
Kumánovics A, 2003, ANNU REV IMMUNOL, V21, P629, DOI 10.1146/annurev.immunol.21.090501.080116
Leroy EM, 2005, NATURE, V438, P575, DOI 10.1038/438575a
Leroy EM, 2009, VECTOR-BORNE ZOONOT, V9, P723, DOI 10.1089/vbz.2008.0167
Luis AD, 2013, P ROY SOC B-BIOL SCI, V280, DOI 10.1098/rspb.2012.2753
Lunney JK, 2009, DEV COMP IMMUNOL, V33, P362, DOI 10.1016/j.dci.2008.07.002
Luo ZX, 2011, NATURE, V476, P442, DOI 10.1038/nature10291
MADDEN DR, 1995, ANNU REV IMMUNOL, V13, P587, DOI 10.1146/annurev.iy.13.040195.003103
MAIOROV VN, 1994, J MOL BIOL, V235, P625, DOI 10.1006/jmbi.1994.1017
Marsh StevenG. E., 1999, The HLA factsbook
Mayer F, 2007, HEREDITY, V99, P257, DOI 10.1038/sj.hdy.6800989
Middleton DJ, 2007, J COMP PATHOL, V136, P266, DOI 10.1016/j.jcpa.2007.03.002

MOMBURG F, 1994, J EXP MED, V179, P1613, DOI 10.1084/jem.179.5.1613
Nielsen M, 2010, NUCLEIC ACIDS RES, V38, pW576, DOI 10.1093/nar/gkq535
Ohta Y, 2006, J IMMUNOL, V176, P3674, DOI 10.4049/jimmunol.176.6.3674
Papenfuss AT, 2012, BMC GENOMICS, V13, DOI 10.1186/1471-2164-13-261
Pinto RD, 2013, DEV COMP IMMUNOL, V39, P234, DOI 10.1016/j.dci.2012.10.002
Renard C, 2006, GENOMICS, V88, P96, DOI 10.1016/j.ygeno.2006.01.004
Rist MJ, 2013, J IMMUNOL, V191, P561, DOI 10.4049/jimmunol.1300292
ROELSE J, 1994, J EXP MED, V180, P1591, DOI 10.1084/jem.180.5.1591
RZHETSKY A, 1992, MOL BIOL EVOL, V9, P945
SAITOU N, 1987, MOL BIOL EVOL, V4, P406, DOI 10.1093/oxfordjournals.molbev.a040454
SAPER MA, 1991, J MOL BIOL, V219, P277, DOI 10.1016/0022-2836(91)90567-P
Schad J, 2011, HEREDITY, V107, P115, DOI 10.1038/hdy.2010.173
Schad J, 2012, PLOS ONE, V7, DOI 10.1371/journal.pone.0037101
Schad J, 2012, IMMUNOGENETICS, V64, P535, DOI 10.1007/s00251-012-0609-1
SCHNEIDER TD, 1990, NUCLEIC ACIDS RES, V18, P6097, DOI 10.1093/nar/18.20.6097
SCHUMACHER TNM, 1994, J EXP MED, V179, P533, DOI 10.1084/jem.179.2.533
Siddle HV, 2011, BMC GENOMICS, V12, DOI 10.1186/1471-2164-12-421
Simmons NB, 2005, MAMMAL SPECIES WORLD, V1, P312, DOI [DOI 10.1111/J.1365-2656.2010.01742.X, 10.1093/acprof]
Smith JDL, 2009, BIOL LETTERS, V5, P347, DOI 10.1098/rsbl.2009.0016
Sullivan MJ, 2011, BIOINFORMATICS, V27, P1009, DOI 10.1093/bioinformatics/btr039
Tamura K, 2011, MOL BIOL EVOL, V28, P2731, DOI 10.1093/molbev/msr121
Teeling EC, 2005, SCIENCE, V307, P580, DOI 10.1126/science.1105113
TROWSDALE J, 1995, IMMUNOGENETICS, V41, P1, DOI 10.1007/BF00188427
Trowsdale J, 2001, IMMUNITY, V15, P363, DOI 10.1016/S1074-7613(01)00197-2
VANIN EF, 1985, ANNU REV GENET, V19, P253, DOI 10.1146/annurev.ge.19.120185.001345
Velten FW, 1999, IMMUNOGENETICS, V49, P919, DOI 10.1007/s002510050575
VITIELLO A, 1990, SCIENCE, V250, P1423, DOI 10.1126/science.2124002
Waddell PJ, 1997, MOL PHYLOGENET EVOL, V8, P398, DOI 10.1006/mpev.1997.0452
WEINER AM, 1986, ANNU REV BIOCHEM, V55, P631, DOI 10.1146/annurev.bi.55.070186.003215

   Wild J, 2002, GENOME RES, V12, P1434, DOI 10.1101/gr.130502
   Williamson MM, 1998, AUST VET J, V76, P813, DOI 10.1111/j.1751-0813.1998.tb12335.x
   Williamson MM, 2000, J COMP PATHOL, V122, P201, DOI 10.1053/jcpa.1999.0364
   Zhang GJ, 2013, SCIENCE, V339, P456, DOI 10.1126/science.1230835
   Zhang Y, 2008, BMC BIOINFORMATICS, V9, DOI 10.1186/1471-2105-9-40
   Zhang ZDD, 2008, MOL BIOL EVOL, V25, P131, DOI 10.1093/molbev/msm251
NR 77
TC 37
Z9 40
U1 0
U2 33
PU NATURE PORTFOLIO
PI BERLIN
PA HEIDELBERGER PLATZ 3, BERLIN, 14197, GERMANY
SN 2045-2322
J9 SCI REP-UK
JI Sci Rep
PD FEB 15
PY 2016
VL 6
AR 21256
DI 10.1038/srep21256
PG 18
WC Multidisciplinary Sciences
WE Science Citation Index Expanded (SCI-EXPANDED)
SC Science & Technology - Other Topics
GA DD6MS
UT WOS:000370038900001
PM 26876644
OA gold, Green Published
DA 2024-10-21
ER

PT J
AU Heger, T
   Zukal, J
   Seidlová, V
   Nemcova, M
   Necas, D
   Papezikova, I
   Piacek, V
   Zajickova, R
   Bandouchova, H
   Pikula, J
AF Heger, Tomas
   Zukal, Jan
   Seidlova, Veronika
   Nemcova, Monika
   Necas, David
   Papezikova, Ivana
   Piacek, Vladimir

   Zajickova, Renata
   Bandouchova, Hana
   Pikula, Jiri
TI Measurement of phagocyte activity in heterotherms
SO ACTA VETERINARIA BRNO
LA English
DT Article
DE Innate immunity; respiratory burst; torpor; hibernation; bat; laboratory
   mouse
ID WHOLE-BLOOD CHEMILUMINESCENCE; MAMMALIAN HIBERNATION; SEASONAL-CHANGES;
   IMMUNE-SYSTEM; TEMPERATURE; BATS
AB The heterotherm immune system undergoes significant variation in response to life cycle periodicity and torpor. As heterothermic bats are important reservoirs of zoonotic agents and modulation of immune activity can affect host-pathogen interactions, this work aimed at developing a suitable method for assessing heterotherm phagocyte activity. Chemiluminescence measurements were evaluated by mathematical and mechanistic approaches, both of which yielded comparable results in time-related parameters of phagocyte activity. Using a mathematical method, however, we developed a model that can be applied to particular specimens. The proposed equation offers a simple and reliable tool for comparing phagocyte activity, the values of which can be used for further analysis. While time-related parameters of bat phagocyte activity varied with measurement temperature, with the onset of respiratory burst at 38 degrees C being quicker than at 25 degrees C, quantitative values of phagocyte activity were not influenced by measurement temperature. Further, homeotherm phagocyte activity parameters were more variable at 25 degrees C. Considering there was no influence of measurement temperature on the total volume of heterotherm phagocyte activity, we suggest that parameters measured at 25 degrees C are more representative of the immune status adapted to physiological extremes at low body temperatures.
C1 [Heger, Tomas; Seidlova, Veronika; Nemcova, Monika; Papezikova, Ivana; Piacek, Vladimir; Bandouchova, Hana; Pikula, Jiri] Univ Vet & Pharmaceut Sci Brno, Fac Vet Hyg & Ecol, Dept Ecol & Dis Zoo Anim Game Fish & Bees, Brno, Czech Republic.
   [Zukal, Jan; Zajickova, Renata] Czech Acad Sci, Vvi, Inst Vertebrate Biol, Brno, Czech Republic.
   [Zukal, Jan] Masaryk Univ, Fac Sci, Dept Bot & Zool, Brno, Czech Republic.
   [Necas, David] Masaryk Univ, Plasma Technol, CEITEC, Brno, Czech Republic.
C3 University of Veterinary Sciences Brno; Czech Academy of Sciences;
   Institute of Vertebrate Biology of the Czech Academy of Sciences;
   Masaryk University Brno; Masaryk University Brno
RP Heger, T; Pikula, J (corresponding author), Univ Vet & Pharmaceut Sci Brno, Dept Ecol & Dis Game Fish & Bees, Palackeho Tr 1946-1, Brno 61242, Czech Republic.
EM hegert4@gmail.com; pikulaj@vfu.cz
RI Nečas, David/D-7166-2012; Piaček, Vladimír/AAI-1231-2020;

Kovacova,
Veronika/V-1815-2019; Bandouchova, Hana/AAI-1213-2020; Divinova,
Renata/KBA-8297-2024; Zukal, Jan/G-1180-2014; Pikula, Jiri/
V-8620-2018
OI Divinova, Renata/0000-0001-8541-5536; Bandouchova,
Hana/0000-0003-0373-7940; Zukal, Jan/0000-0003-4967-6880; Pikula,
Jiri/0000-0001-8747-9365
FU Czech Science Foundation [17-20286S]
FX This study was supported by the Czech Science Foundation (Grant
No.
17-20286S). We are grateful to Dr. Kevin Roche for correction and
improvement of the English text.
CR AINSWORTH AJ, 1991, COMP BIOCHEM PHYS A, V100, P907, DOI
10.1016/0300-9629(91)90313-2
[Anonymous], 1964, ANN ACAD SCI FENN BI
Bandouchova H, 2018, SCI REP-UK, V8, DOI 10.1038/
s41598-018-24461-5
Bartonicka T, 2017, J THERM BIOL, V63, P119, DOI 10.1016/
j.jtherbio.2016.12.005
Bouma HR, 2010, VET IMMUNOL IMMUNOP, V136, P319, DOI 10.1016/
j.vetimm.2010.03.016
Bouma HR, 2013, J LEUKOCYTE BIOL, V94, P431, DOI 10.1189/
jlb.0611298
Bouma HR, 2010, J LEUKOCYTE BIOL, V88, P619, DOI 10.1189/
jlb.0310174
Boyer BB, 1999, BIOSCIENCE, V49, P713, DOI 10.2307/1313595
Buchtíková S, 2011, AQUACULTURE, V318, P169, DOI 10.1016/
j.aquaculture.2011.05.013
Carey HV, 2003, PHYSIOL REV, V83, P1153, DOI 10.1152/
physrev.00008.2003
Chou Y.L., 1989, STAT ANAL BUSINESS E
COLLAZOS ME, 1994, COMP BIOCHEM PHYS A, V109, P643, DOI
10.1016/0300-9629(94)90204-6
Havenstein N, 2016, BRAIN BEHAV IMMUN, V52, P71, DOI 10.1016/
j.bbi.2015.09.018
Heldmaier G, 2004, RESP PHYSIOL NEUROBI, V141, P317, DOI 10.1016/
j.resp.2004.03.014
Inkovaara P., 1973, Suomalaisen Tiedeakat Toim (Ser A Biol), VNo.
200, P1
KAEVER V, 1992, EUR J CLIN CHEM CLIN, V30, P209
Maniero GD, 2002, DEV COMP IMMUNOL, V26, P563, DOI 10.1016/
S0145-305X(02)00006-X
MARNILA P, 1995, COMP BIOCHEM PHYS A, V111, P609, DOI
10.1016/0300-9629(95)00054-B
Mondal S, 2001, COMP BIOCHEM PHYS A, V129, P391, DOI 10.1016/
S1095-6433(00)00356-1
Murphy K, 2012, JANEWAYS IMMUNOBIOLO
Papezíková I, 2016, FISH SHELLFISH IMMUN, V57, P400, DOI 10.1016/
j.fsi.2016.08.048
Parasuraman S, 2010, J PHARMACOL PHARMACO, V1, P87, DOI
10.4103/0976-500X.72350
Pikula Jiri, 2017, Veterinary Clinics of North America Exotic
Animal Practice, V20, P665, DOI 10.1016/j.cvex.2016.11.013
Sutton P., 1997, Computers, Environment and Urban Systems, V21,

P227, DOI 10.1016/S0198-9715(97)01005-3
   WILLSON JT, 1958, SURGERY, V43, P979
NR 25
TC 10
Z9 10
U1 0
U2 8
PU VETERINARNI A FARMACEUTICKA UNIVERZITA BRNO
PI BRNO
PA PALACKEHO 1-3, BRNO CS-612 42, CZECH REPUBLIC
SN 0001-7213
EI 1801-7576
J9 ACTA VET BRNO
JI Acta Vet. BRNO
PY 2020
VL 89
IS 1
BP 79
EP 87
DI 10.2754/avb202089010079
PG 9
WC Veterinary Sciences
WE Science Citation Index Expanded (SCI-EXPANDED)
SC Veterinary Sciences
GA LH8VZ
UT WOS:000529064300010
OA gold
DA 2024-10-21
ER

PT J
AU Malavé, CM
   Lopera-Madrid, J
   Medina-Magües, LG
   Rocke, TE
   Osorio, JE
AF Malave, Carly M.
   Lopera-Madrid, Jaime
   Medina-Magues, Lex G.
   Rocke, Tonie E.
   Osorio, Jorge E.
TI Impact of Molecular Modifications on the Immunogenicity and Efficacy of
   Recombinant Raccoon Poxvirus-Vectored Rabies Vaccine Candidates in Mice
SO VACCINES
LA English
DT Article
DE rabies; rabies virus; RABV; raccoon poxvirus; recombinant; vaccine; A; J
   mice
ID VAMPIRE BATS; VIRUS; INFECTION; LYSSAVIRUSES; IMMUNIZATION; EXPRESSION;
   EMERGENCE; HUMANS

AB Rabies is an ancient disease that is responsible for approximately 59,000 human deaths annually. Bats (Order Chiroptera) are thought to be the original hosts of rabies virus (RABV) and currently account for most rabies cases in wildlife in the Americas. Vaccination is being used to manage rabies in other wildlife reservoirs like fox and raccoon, but no rabies vaccine is available for bats. We previously developed a recombinant raccoonpox virus (RCN) vaccine candidate expressing a mosaic glycoprotein (MoG) gene that protected mice and big brown bats when challenged with RABV. In this study, we developed two new recombinant RCN candidates expressing MoG (RCN-tPA-MoG and RCN-SS-TD-MoG) with the aim of improving RCN-MoG. We assessed and compared in vitro expression, in vivo immunogenicity, and protective efficacy in vaccinated mice challenged intracerebrally with RABV. All three candidates induced significant humoral immune responses, and inoculation with RCN-tPA-MoG or RCN-MoG significantly increased survival after RABV challenge. These results demonstrate the importance of considering molecular elements in the design of vaccines, and that vaccination with either RCN-tPA-MoG or RCN-MoG confers adequate protection from rabies infection, and either may be a sufficient vaccine candidate for bats in future work.
C1 [Malave, Carly M.; Rocke, Tonie E.] US Geol Survey, Natl Wildlife Hlth Ctr, Madison, WI 53711 USA.
[Malave, Carly M.; Lopera-Madrid, Jaime; Medina-Magues, Lex G.; Osorio, Jorge E.] Univ Wisconsin Madison, Sch Vet Med, Dept Pathobiol Sci, Madison, WI 53706 USA.
C3 United States Department of the Interior; United States Geological
Survey; University of Wisconsin System; University of Wisconsin Madison
RP Rocke, TE (corresponding author), US Geol Survey, Natl Wildlife Hlth Ctr, Madison, WI 53711 USA.; Osorio, JE (corresponding author), Univ Wisconsin Madison, Sch Vet Med, Dept Pathobiol Sci, Madison, WI 53706 USA.
EM malave@usgs.gov; loperamadrid@wisc.edu; medinamagues@wisc.edu;
rocke@usgs.gov; jorge.osorio@wisc.edu
OI Medina Magues, Lex Guillermo/0000-0002-4242-6945; OSORIO,
JORGE/0000-0002-7474-6150; Lopera Madrid, Jaime/
0000-0003-2215-8426
FU U.S. Geological Survey and the University of Wisconsin-Madison
FX FundingThis research was funded by the U.S. Geological Survey and the
University of Wisconsin-Madison (NSF award no. 2011069).
CR Banyard AC, 2014, VIRUSES-BASEL, V6, P2974, DOI 10.3390/v6082974
Barber RD, 2005, PHYSIOL GENOMICS, V21, P389, DOI 10.1152/physiolgenomics.00025.2005
Blackwood JC, 2013, P NATL ACAD SCI USA, V110, P20837, DOI 10.1073/pnas.1308817110
Calisher CH, 2012, TRAVEL MED INFECT DI, V10, P69, DOI 10.1016/j.tmaid.2012.01.003
Cárdenas-Canales EM, 2020, TROP MED INFECT DIS, V5, DOI 10.3390/tropicalmed5010034
Chakrabarti S, 1997, BIOTECHNIQUES, V23, P1094, DOI 10.2144/97236st07

Dato V., 2009, Morbidity and Mortality Weekly Report, V58, P1204
Dean DJ., 1996, LAB TECHNIQUES RABIE, P83
Earl P L, 2001, Curr Protoc Mol Biol, VChapter 16, DOI [10.1002/0471142727.mb1616s43, 10.1002/0471142727.mb1617s43]
Fisher CR, 2018, NAT REV MICROBIOL, V16, P241, DOI 10.1038/nrmicro.2018.11
Haddad D, 1997, FEMS IMMUNOL MED MIC, V18, P193, DOI 10.1016/S0928-8244(97)00039-4
Hwa SH, 2010, AVIAN DIS, V54, P1157, DOI 10.1637/9315-032410-Reg.1
Johnson N, 2010, VET MICROBIOL, V142, P151, DOI 10.1016/j.vetmic.2010.02.001
Kou YM, 2017, IMMUNOL LETT, V190, P51, DOI 10.1016/j.imlet.2017.07.007
Lui YLE, 2013, BIOCHEM BIOPH RES CO, V433, P607, DOI 10.1016/j.bbrc.2013.03.044
Ma XY, 2018, JAVMA-J AM VET MED A, V253, P1555, DOI 10.2460/javma.253.12.1555
Malave C.M., 2021, IN VITRO EXPRESSION
Megid J, 2008, AM J TROP MED HYG, V79, P647, DOI 10.4269/ajtmh.2008.79.647
Meza D. K, 2020, BIORXIV, DOI [10.1101/2020.04.24.060095, DOI 10.1101/2020.04.24.060095]
Moore SM, 2010, PLOS NEGLECT TROP D, V4, DOI 10.1371/journal.pntd.0000595
Osorio JE, 2003, VACCINE, V21, P1232, DOI 10.1016/S0264-410X(02)00557-1
Rasband W. S., IMAGEJ
REED L. J., 1938, AMER JOUR HYG, V27, P493
RUPPRECHT CE, 1986, P NATL ACAD SCI USA, V83, P7947, DOI 10.1073/pnas.83.20.7947
Schindelin J, 2012, NAT METHODS, V9, P676, DOI [10.1038/NMETH.2019, 10.1038/nmeth.2019]
Schneider MC, 2009, REV PANAM SALUD PUBL, V25, P260, DOI 10.1590/S1020-49892009000300010
Sétien AA, 1998, VACCINE, V16, P1122, DOI 10.1016/S0264-410X(98)80108-4
Sidwa TJ, 2005, JAVMA-J AM VET MED A, V227, P785, DOI 10.2460/javma.2005.227.785
Slate D, 2005, VIRUS RES, V111, P68, DOI 10.1016/j.virusres.2005.03.012
Smith JYP, 1996, RAPID FLUORESCENT FO
Smith TG, 2017, TROP MED INFECT DIS, V2, DOI 10.3390/tropicalmed2030024
Stading B, 2017, PLOS NEGLECT TROP D, V11, DOI 10.1371/journal.pntd.0005958
Streicker DG, 2012, P ROY SOC B-BIOL SCI, V279, P3384, DOI 10.1098/rspb.2012.0538
Thomas Philip, 2005, Journal of Pharmacological and Toxicological Methods, V51, P187, DOI 10.1016/j.vascn.2004.08.014
Wallace RM, 2014, PLOS ONE, V9, DOI 10.1371/journal.pone.0107539
Wang JY, 2011, APPL MICROBIOL BIOT, V91, P731, DOI 10.1007/s00253-011-3297-0
WHO, 2005, WHO TECH REP SER, V931, P1

   Wyatt LS, 1996, VACCINE, V14, P1451, DOI 10.1016/
S0264-410X(96)00072-2
   Zhao RQ, 2020, PROTEIN EXPRES PURIF, V168, DOI 10.1016/
j.pep.2019.105567
   Zhu JW, 2012, BIOTECHNOL ADV, V30, P1158, DOI 10.1016/
j.biotechadv.2011.08.022
NR 40
TC 1
Z9 1
U1 0
U2 5
PU MDPI
PI BASEL
PA ST ALBAN-ANLAGE 66, CH-4052 BASEL, SWITZERLAND
EI 2076-393X
J9 VACCINES-BASEL
JI Vaccines
PD DEC
PY 2021
VL 9
IS 12
AR 1436
DI 10.3390/vaccines9121436
PG 12
WC Immunology; Medicine, Research & Experimental
WE Science Citation Index Expanded (SCI-EXPANDED)
SC Immunology; Research & Experimental Medicine
GA XZ0WE
UT WOS:000737382100001
PM 34960182
OA Green Published, gold
DA 2024-10-21
ER

PT J
AU Cornman, RS
   Cryan, PM
AF Cornman, Robert S.
   Cryan, Paul M.
TI Positively selected genes in the hoary bat (<i>Lasiurus
cinereus</i>)
   lineage: prominence of thymus expression, immune and metabolic
function,
   and regions of ancient synteny
SO PEERJ
LA English
DT Article
DE Hoary bat; Adaptation; Positive selection; Immunogenetics;
Thymus;
   Conserved synteny; Cat-eye critical region; Tbx gene family;
Chrna9
ID ADIPOSE-TISSUE; EVOLUTIONARY FATE; PROVIDES INSIGHT; CANDIDATE
GENES;
   MICE; MOUSE; DNA; GENOMES; IDENTIFICATION; FLIGHT

AB Background: Bats of the genus Lasiurus occur throughout the Americas and have diversified into at least 20 species among three subgenera. The hoary bat (Lasiurus cinereus) is highly migratory and ranges farther across North America than any other wild mammal. Despite the ecological importance of this species as a major insect predator, and the particular susceptibility of lasiurine bats to wind turbine strikes, our understanding of hoary bat ecology, physiology, and behavior remains poor.

Methods: To better understand adaptive evolution in this lineage, we used whole-genome sequencing to identify protein-coding sequence and explore signatures of positive selection. Gene models were predicted with Maker and compared to seven well-annotated and phylogenetically representative species. Evolutionary rate analysis was performed with PAML.

Results: Of 9,447 single-copy orthologous groups that met evaluation criteria, 150 genes had a significant excess of nonsynonymous substitutions along the L. cinereus branch ($P < 0.001$ after manual review of alignments). Selected genes as a group had biased expression, most strongly in thymus tissue. We identified 23 selected genes with reported immune functions as well as a divergent paralog of Steepl within suborder Yangochiroptera. Seventeen genes had roles in lipid and glucose metabolic pathways, partially overlapping with 15 mitochondrion-associated genes; these adaptations may reflect the metabolic challenges of hibernation, long-distance migration, and seasonal variation in prey abundance. The genomic distribution of positively selected genes differed significantly from background expectation by discrete Kolmogorov-Smirnov test ($P < 0.001$). Remarkably, the top three physical clusters all coincided with islands of conserved synteny predating Mammalia, the largest of which shares synteny with the human cat-eye critical region (CECR) on 22q11. This observation coupled with the expansion of a novel Tbx1-like gene family may indicate evolutionary innovation during pharyngeal arch development: both the CECR and Tbx1 cause dosage-dependent congenital abnormalities in thymus, heart, and head, and craniodysmorphy is associated with human orthologs of other positively selected genes as well.
C1 [Cornman, Robert S.; Cryan, Paul M.] US Geol Survey, Ft Collins Sci Ctr, Ft Collins, CO 80526 USA.
C3 United States Department of the Interior; United States Geological
Survey
RP Cornman, RS (corresponding author), US Geol Survey, Ft Collins Sci Ctr, Ft Collins, CO 80526 USA.
EM rcornman@usgs.gov
OI Cryan, Paul/0000-0002-2915-8894
FU National Science Foundation [1744877]
FX Selection test methods applied in this study were facilitated using best
practices and scripts provided as part of a Next Generation
Sequencing-based workshop sponsored by the National Science Foundation
(Award # 1744877 to S. Santagata, Long Island University). We are
grateful to reviewers who provided their time and expertise to improve

the manuscript. Any use of trade, firm, or product names is for
descriptive purposes only and does not imply endorsement by the
U.S.
Government.
CR Adams RA., 2013, Bat evolution, ecology, and conservation
Alfano D, 2019, HUM MOL GENET, V28, P2295, DOI 10.1093/hmg/ddz058
ARNAOUT MA, 1990, BLOOD, V75, P1037
Ballinger MA, 2016, AM J PHYSIOL-REG I, V311, pR325, DOI 10.1152/
ajpregu.00463.2015
Bar-Lev Y, 2016, PLOS ONE, V11, DOI 10.1371/journal.pone.0157850
Bass C, 2013, P NATL ACAD SCI USA, V110, P19460, DOI 10.1073/
pnas.1314122110
Beichman AC, 2019, MOL BIOL EVOL, V36, P2631, DOI 10.1093/molbev/
msz101
Berthelot JM, 2020, EBIOMEDICINE, V56, DOI 10.1016/
j.ebiom.2020.102801
Bitter MC, 2019, NAT COMMUN, V10, DOI 10.1038/s41467-019-13767-1
Bockman DE, 1997, MICROSC RES TECHNIQ, V38, P209, DOI 10.1002/
(SICI)1097-0029(19970801)38:3<209::AID-JEMT2>3.0.CO;2-H
Boero LE, 2020, P NATL ACAD SCI USA, V117, P11811, DOI 10.1073/
pnas.2000760117
Brindley DN, 1998, J BIOL CHEM, V273, P24281, DOI 10.1074/
jbc.273.38.24281
Brown GR, 2015, NUCLEIC ACIDS RES, V43, pD36, DOI 10.1093/nar/
gku1055
Buchfink B, 2015, NAT METHODS, V12, P59, DOI 10.1038/nmeth.3176
Cantarel BL, 2008, GENOME RES, V18, P188, DOI 10.1101/gr.6743907
Casola C, 2010, GENETICS, V185, P95, DOI 10.1534/
genetics.110.115444
Chang JM, 2014, MOL BIOL EVOL, V31, P1625, DOI 10.1093/molbev/
msu117
Chazarin B, 2019, FRONT ZOOL, V16, DOI 10.1186/s12983-019-0312-2
Chen Y, 2019, BIOCHEMISTRY-MOSCOW+, V84, P1529, DOI 10.1134/
S0006297919120125
Chen YF, 2017, J EXP MED, V214, P991, DOI 10.1084/jem.20161387
Chiarella E, 2018, STEM CELL REV REP, V14, P901, DOI 10.1007/
s12015-018-9830-0
Clayton E, 2020, FRONT CELL INFECT MI, V10, DOI 10.3389/
fcimb.2020.527921
Corcoran AJ, 2018, P ROY SOC B-BIOL SCI, V285, DOI 10.1098/
rspb.2018.0441
Corcoran AJ, 2017, J EXP BIOL, V220, P4554, DOI 10.1242/
jeb.163063
Cornman RS, 2021, PEERJ, V9, DOI 10.7717/peerj.11285
Cornman RS., GENE ANNOTATIONS HOA
Cornman RS., 2020, GENETIC VARIATION HO
Corradi N, 2010, NAT COMMUN, V1, DOI 10.1038/ncomms1082
Cryan PM, 2014, P NATL ACAD SCI USA, V111, P15126, DOI 10.1073/
pnas.1406672111
Cryan PM, 2014, ECOL APPL, V24, P602, DOI 10.1890/13-0752.1
Cryan PM, 2003, J EXP BIOL, V206, P3381, DOI 10.1242/jeb.00574
D'Amico D, 2019, MOL CELL, V73, P775, DOI 10.1016/
j.molcel.2018.11.034
Dantas AG, 2019, HUM GENET, V138, P93, DOI 10.1007/

s00439-018-01967-6
Davies KTJ, 2020, MOL ECOL, V29, P1839, DOI 10.1111/mec.15445
Davis W. H., 1970, P265
Di Franco A, 2019, BMC EVOL BIOL, V19, DOI 10.1186/s12862-019-1350-2
Dimitrova DS, 2020, J STAT SOFTW, V95, P1, DOI 10.18637/jss.v095.i10
DOBZHANSKY T, 1958, P NATL ACAD SCI USA, V44, P622, DOI 10.1073/pnas.44.6.622
Dong D, 2017, MOL BIOL EVOL, V34, P20, DOI 10.1093/molbev/msw231
Du QM, 2020, FRONT GENET, V10, DOI 10.3389/fgene.2019.01365
Ehrhardt N, 2014, NUTR METAB, V11, DOI 10.1186/1743-7075-11-37
Emms DM, 2019, GENOME BIOL, V20, DOI 10.1186/s13059-019-1832-y
Footz TK, 2001, GENOME RES, V11, P1053, DOI 10.1101/gr.154901
Frick WF, 2017, BIOL CONSERV, V209, P172, DOI 10.1016/j.biocon.2017.02.023
Friederich MW, 2017, HUM MOL GENET, V26, P702, DOI 10.1093/hmg/ddw431
Gässler A, 2020, MOL METAB, V41, DOI 10.1016/j.molmet.2020.101042
Gayral P, 2007, J EVOLUTION BIOL, V20, P617, DOI 10.1111/j.1420-9101.2006.01245.x
Geiser F, 2013, CURR BIOL, V23, pR188, DOI 10.1016/j.cub.2013.01.062
Härtlova A, 2015, IMMUNITY, V42, P332, DOI 10.1016/j.immuni.2015.01.012
Hawkins JA, 2019, P NATL ACAD SCI USA, V116, P11351, DOI 10.1073/pnas.1814995116
Hein C., 2021, TECHNICAL REPORT ACO
Henquin JC, 2014, ENDOCRINOLOGY, V155, P3274, DOI 10.1210/en.2014-1247
Henriques BJ, 2021, GENE, V776, DOI 10.1016/j.gene.2021.145407
Hittinger CT, 2010, NATURE, V464, P54, DOI 10.1038/nature08791
Hogan PG, 2010, ANNU REV IMMUNOL, V28, P491, DOI 10.1146/annurev.immunol.021908.132550
Holder JL, 2004, AM J PHYSIOL-ENDOC M, V287, pE105, DOI 10.1152/ajpendo.00446.2003
Holm CK, 2012, NAT IMMUNOL, V13, P737, DOI 10.1038/ni.2350
Howard MJ, 2012, P NATL ACAD SCI USA, V109, P16149, DOI 10.1073/pnas.1209062109
Hoyt JR, 2021, NAT REV MICROBIOL, V19, P196, DOI 10.1038/s41579-020-00493-5
Huang DW, 2007, NUCLEIC ACIDS RES, V35, pW169, DOI 10.1093/nar/gkm415
Huber C, 2005, NAT GENET, V37, P1119, DOI 10.1038/ng1628
Hughes GM, 2018, MOL BIOL EVOL, V35, P1390, DOI 10.1093/molbev/msy028
Ishii M, 2003, P NATL ACAD SCI USA, V100, P10540, DOI 10.1073/pnas.1334189100
Ishikawa H, 2009, NATURE, V461, P788, DOI 10.1038/nature08476
Perez SI, 2013, PLOS ONE, V8, DOI 10.1371/journal.pone.0068029
Jebb D, 2020, NATURE, V583, P578, DOI 10.1038/s41586-020-2486-3
Jiang L, 2020, NAT COMMUN, V11, DOI 10.1038/s41467-020-15328-3
Jiang Q, 2019, FASEB J, V33, P2971, DOI 10.1096/fj.201801393RRR
Jorgenson E, 2018, P NATL ACAD SCI USA, V115, P11018, DOI

10.1073/pnas.1809872115
Jurka J, 2005, CYTOGENET GENOME RES, V110, P462, DOI 10.1159/000084979
Kataoka S, 2021, J ALLERGY CLIN IMMUN, V148, P639, DOI 10.1016/j.jaci.2021.03.010
Katoh K, 2002, NUCLEIC ACIDS RES, V30, P3059, DOI 10.1093/nar/gkf436
Koop D, 2014, EVODEVO, V5, DOI 10.1186/2041-9139-5-36
Korstian JM, 2015, J MAMMAL, V96, P972, DOI 10.1093/jmammal/gyv101
Kosiol C, 2008, PLOS GENET, V4, DOI 10.1371/journal.pgen.1000144
Koyasu S, 2003, NAT IMMUNOL, V4, P313, DOI 10.1038/ni0403-313
Kramerova I, 2009, HUM MOL GENET, V18, P3194, DOI 10.1093/hmg/ddp257
Kriventseva EV, 2019, NUCLEIC ACIDS RES, V47, pD807, DOI 10.1093/nar/gky1053
Krogh A, 2001, J MOL BIOL, V305, P567, DOI 10.1006/jmbi.2000.4315
Kulyté A, 2011, J CLIN ENDOCR METAB, V96, pE1661, DOI 10.1210/jc.2010-3050
Kumar S, 2018, MOL BIOL EVOL, V35, P1547, DOI 10.1093/molbev/msy096
Kunath A, 2016, BIOCHEM BIOPH RES CO, V478, P398, DOI 10.1016/j.bbrc.2016.07.038
Kurien P, 2019, P NATL ACAD SCI USA, V116, P12045, DOI 10.1073/pnas.1819110116
Lee PL, 2009, GLYCOBIOLOGY, V19, P655, DOI 10.1093/glycob/cwp035
Li X, 2001, J LEUKOCYTE BIOL, V69, P565
Liao J, 2004, HUM MOL GENET, V13, P1577, DOI 10.1093/hmg/ddh176
Lindblad-Toh K, 2011, NATURE, V478, P476, DOI 10.1038/nature10530
Lindsay EA, 2001, NATURE, V410, P97, DOI 10.1038/35065105
Lipovsek M, 2014, MOL BIOL EVOL, V31, P3250, DOI 10.1093/molbev/msu258
Liu D, 2019, CELL DEATH DIFFER, V26, P1735, DOI 10.1038/s41418-018-0251-z
Liu GY, 2019, P NATL ACAD SCI USA, V116, P3347, DOI 10.1073/pnas.1821550116
Liu HZ, 2018, FRONT MOL NEUROSCI, V11, DOI 10.3389/fnmol.2018.00356
Luo YW, 2019, J IMMUNOL, V203, P269, DOI 10.4049/jimmunol.1900125
Ma C, 2020, J PROTEOMICS, V213, DOI 10.1016/j.jprot.2019.103630
Malecki J, 2019, J BIOL CHEM, V294, P11654, DOI 10.1074/jbc.RA119.009045
Manceau M, 2010, PHILOS T R SOC B, V365, P2439, DOI 10.1098/rstb.2010.0104
Mao HZ, 2014, J BIOL CHEM, V289, P24417, DOI 10.1074/jbc.M114.588764
Marçais G, 2018, PLOS COMPUT BIOL, V14, DOI 10.1371/journal.pcbi.1005944
Marcovich I, 2020, MOL BIOL EVOL, V37, P1070, DOI 10.1093/molbev/msz290
McGuire LP, 2014, PLOS ONE, V9, DOI 10.1371/journal.pone.0115724
McGuire LP, 2013, COMP BIOCHEM PHYS B, V165, P138, DOI 10.1016/j.cbpb.2013.03.013
Melroy-Greif WE, 2017, TWIN RES HUM GENET, V20, P499, DOI

10.1017/thg.2017.62
Merscher S, 2001, CELL, V104, P619, DOI 10.1016/S0092-8674(01)00247-1
Meyer B, 2016, NUCLEIC ACIDS RES, V44, P4304, DOI 10.1093/nar/gkw244
Michalak P, 2008, GENOMICS, V91, P243, DOI 10.1016/j.ygeno.2007.11.002
Michaud JL, 1998, GENE DEV, V12, P3264, DOI 10.1101/gad.12.20.3264
Millar NS, 2003, BIOCHEM SOC T, V31, P869, DOI 10.1042/bst0310869
Mohammadi SA, 2017, BEHAV BRAIN RES, V328, P105, DOI 10.1016/j.bbr.2017.04.005
Nagata-Kuroiwa R, 2011, PLOS ONE, V6, DOI 10.1371/journal.pone.0016972
Nawrocki AR, 2014, DIABETES, V63, P300, DOI 10.2337/db13-0247
Neafsey DE, 2015, SCIENCE, V347, DOI 10.1126/science.1258522
Notredame C, 2000, J MOL BIOL, V302, P205, DOI 10.1006/jmbi.2000.4042
O'Shea TJ, 2014, EMERG INFECT DIS, V20, P741, DOI 10.3201/eid2005.130539
Oliver TA, 2010, GENOME BIOL EVOL, V2, P800, DOI 10.1093/gbe/evq063
Onyilagha C, 2015, PLOS NEGLECT TROP D, V9, DOI 10.1371/journal.pntd.0003716
Palmer DB, 2013, FRONT IMMUNOL, V4, DOI 10.3389/fimmu.2013.00316
Paradis E, 2004, BIOINFORMATICS, V20, P289, DOI [10.1093/bioinformatics/btg412, 10.1093/bioinformatics/bty633]
Piccart E, 2011, NEUROBIOL LEARN MEM, V95, P260, DOI 10.1016/j.nlm.2010.11.018
Pinzari CA, 2020, GENOME BIOL EVOL, V12, P1504, DOI 10.1093/gbe/evaa137
Poppi LA, 2018, J NEUROPHYSIOL, V119, P312, DOI 10.1152/jn.00030.2017
Puech A, 1997, P NATL ACAD SCI USA, V94, P14608, DOI 10.1073/pnas.94.26.14608
Pylant CL, 2016, ECOL APPL, V26, P1381, DOI 10.1890/15-0541
*R COR TEAM, 2019, R LANG ENV STAT COMP
Raft S, 2004, DEVELOPMENT, V131, P1801, DOI 10.1242/dev.01067
Ramachandrappa S, 2013, J CLIN INVEST, V123, P3042, DOI 10.1172/JCI68016
Ramos JSL, 2017, J COMP PHYSIOL A, V203, P383, DOI 10.1007/s00359-017-1184-6
Reimegård J, 2017, NUCLEIC ACIDS RES, V45, P3253, DOI 10.1093/nar/gkx087
Rezzani R, 2014, AGE, V36, P313, DOI 10.1007/s11357-013-9564-5
Rhein VF, 2014, J BIOL CHEM, V289, P24640, DOI 10.1074/jbc.M114.580464
Rodhouse TJ, 2019, ECOL EVOL, V9, P11078, DOI 10.1002/ece3.5612
Rokas A, 2018, NAT REV MICROBIOL, V16, P731, DOI 10.1038/s41579-018-0075-3
Roux J, 2014, MOL BIOL EVOL, V31, P1661, DOI 10.1093/molbev/msu141
Sandve SR, 2018, NAT GENET, V50, P908, DOI 10.1038/s41588-018-0162-4

Santagata S.;, 2021, SELECT TEST
Sarzi E, 2007, ANN NEUROL, V62, P579, DOI 10.1002/ana.21207
Scheffer DI, 2015, J NEUROSCI, V35, P6366, DOI 10.1523/JNEUROSCI.5126-14.2015
Seeger TF, 2003, BRAIN RES, V985, P113, DOI 10.1016/S0006-8993(03)02754-9
Semple E, 2018, ENDOCRINOLOGY, V159, P439, DOI 10.1210/en.2017-00488
Seppey M, 2019, METHODS MOL BIOL, V1962, P227, DOI 10.1007/978-1-4939-9173-0_14
Shabalina SA, 2010, MOL BIOL EVOL, V27, P1745, DOI 10.1093/molbev/msq086
Shen YY, 2010, P NATL ACAD SCI USA, V107, P8666, DOI 10.1073/pnas.0912613107
Shump K.A. Jr, 1982, Mammalian Species, P1
Simakov O, 2020, NAT ECOL EVOL, V4, P820, DOI 10.1038/s41559-020-1156-z
SINGAL DP, 1995, J BIOL CHEM, V270, P1966, DOI 10.1074/jbc.270.4.1966
Slater GS, 2005, BMC BIOINFORMATICS, V6, DOI 10.1186/1471-2105-6-31
Smith MD, 2015, MOL BIOL EVOL, V32, P1342, DOI 10.1093/molbev/msv022
Snyder HL, 1997, J EXP MED, V186, P1087, DOI 10.1084/jem.186.7.1087
Song WJ, 2013, DIABETES, V62, P2796, DOI 10.2337/db12-1394
Sonnhammer E L, 1998, Proc Int Conf Intell Syst Mol Biol, V6, P175
Sovic MG, 2016, PEERJ, V4, DOI 10.7717/peerj.1647
Srivastava DP, 2012, J NEUROSCI, V32, P11864, DOI 10.1523/JNEUROSCI.1349-12.2012
Stum M, 2006, HUM MUTAT, V27, P1082, DOI 10.1002/humu.20388
Suyama M, 2006, NUCLEIC ACIDS RES, V34, pW609, DOI 10.1093/nar/gkl315
Suzuki K, 2013, J BIOL CHEM, V288, P1929, DOI 10.1074/jbc.M112.423137
Taly A, 2009, NAT REV DRUG DISCOV, V8, P733, DOI 10.1038/nrd2927
Tanaka H, 2003, P NATL ACAD SCI USA, V100, P6251, DOI 10.1073/pnas.0837789100
Tarailo-Graovac M, 2009, Curr Protoc Bioinformatics, V25, P4, DOI 10.1002/0471250953.bi0410s25
Taranda J, 2009, PLOS BIOL, V7, P71, DOI 10.1371/journal.pbio.1000018
Teeling EC, 2018, ANNU REV ANIM BIOSCI, V6, P23, DOI 10.1146/annurev-animal-022516-022811
Thalen F, 2018, PHYLOPYPRUNER TREEBA
Thapa P, 2019, THORAC SURG CLIN, V29, P123, DOI 10.1016/j.thorsurg.2018.12.001
Tolson KP, 2014, ENDOCRINOLOGY, V155, P2436, DOI 10.1210/en.2013-2125
Tomanek I, 2020, NAT ECOL EVOL, V4, P612, DOI 10.1038/s41559-020-1132-7
Ujita M, 1999, J BIOL CHEM, V274, P16717, DOI 10.1074/jbc.274.24.16717

van Haelst MM, 2008, AM J MED GENET A, V146A, P2252, DOI 10.1002/ajmg.a.32440
Vinckenbosch N, 2006, P NATL ACAD SCI USA, V103, P3220, DOI 10.1073/pnas.0511307103
Vincler M, 2006, P NATL ACAD SCI USA, V103, P17880, DOI 10.1073/pnas.0608715103
Wanamaker CP, 2005, J BIOL CHEM, V280, P33800, DOI 10.1074/jbc.M501813200
Wang W, 2009, DEV CELL, V16, P764, DOI 10.1016/j.devcel.2009.04.006
Wang XF, 2017, VIRAL IMMUNOL, V30, P342, DOI 10.1089/vim.2017.0015
Watanabe N, 2012, PLOS ONE, V7, DOI 10.1371/journal.pone.0035390
Weaver SP, 2020, GLOB ECOL CONSERV, V24, DOI 10.1016/j.gecco.2020.e01099
Wedemeyer C, 2018, J NEUROSCI, V38, P3939, DOI 10.1523/JNEUROSCI.2528-17.2018
Weisenfeld NI, 2017, GENOME RES, V27, P757, DOI 10.1101/gr.214874.116
Weller TJ, 2016, SCI REP-UK, V6, DOI 10.1038/srep34585
Willour VL, 2012, MOL PSYCHIATR, V17, P433, DOI 10.1038/mp.2011.4
Xi D, 2012, PLOS ONE, V7, DOI 10.1371/journal.pone.0036453
Xie JZ, 2018, CELL HOST MICROBE, V23, P297, DOI 10.1016/j.chom.2018.01.006
Yalvac ME, 2017, SKELET MUSCLE, V7, DOI 10.1186/s13395-017-0146-6
Yamashita Y, 2018, SCI REP-UK, V8, DOI 10.1038/s41598-018-25635-x
Yang ZH, 2007, MOL BIOL EVOL, V24, P1586, DOI 10.1093/molbev/msm088
Yates AD, 2020, NUCLEIC ACIDS RES, V48, pD682, DOI 10.1093/nar/gkz966
Yue F, 2014, NATURE, V515, P355, DOI 10.1038/nature13992
Zhang GJ, 2013, SCIENCE, V339, P456, DOI 10.1126/science.1230835
Zhang H, 2012, ENDOCRINOLOGY, V153, P2665, DOI 10.1210/en.2011-2008
Zhang XY, 2019, EXP EYE RES, V181, P302, DOI 10.1016/j.exer.2019.02.013
Zhang ZP, 2019, FRONT IMMUNOL, V10, DOI 10.3389/fimmu.2019.00871
ZHENG XM, 1990, NATURE, V344, P556, DOI 10.1038/344556a0
NR 188
TC 1
Z9 1
U1 0
U2 14
PU PEERJ INC
PI LONDON
PA 341-345 OLD ST, THIRD FLR, LONDON, EC1V 9LL, ENGLAND
SN 2167-8359
J9 PEERJ
JI PeerJ
PD MAR 17
PY 2022
VL 10
AR e13130
DI 10.7717/peerj.13130

PG 39
WC Multidisciplinary Sciences
WE Science Citation Index Expanded (SCI-EXPANDED)
SC Science & Technology - Other Topics
GA 0C0HA
UT WOS:000775003400001
PM 35317076
OA Green Published, gold
DA 2024-10-21
ER

PT J
AU MCMURRAY, DN
THOMAS, ME
GREER, DL
TOLENTINO, NL
AF MCMURRAY, DN
THOMAS, ME
GREER, DL
TOLENTINO, NL
TI HUMORAL AND CELL-MEDIATED-IMMUNITY TO HISTOPLASMA-CAPSULATUM DURING
EXPERIMENTAL-INFECTION IN NEOTROPICAL BATS (ARTIBEUS-LITURATUS)
SO AMERICAN JOURNAL OF TROPICAL MEDICINE AND HYGIENE
LA English
DT Article
AB The humoral and cell-mediated immune responses of the large, frugivorus bat, A. lituratus were studied following i.p. injection of 10 or 104 viable mycelial particles of H. capsulatum. Most bats had cultural and histologic evidence of disease at autopsy. Utilizing a technique designed to measure the volume of footpad swelling in hypersensitive bats, a significant degree of delayed hypersensitivity [DH] to histoplasmin was evident in both groups 4-6 wk after infection. Precipitating antibodies first appeared in the serum 3 wk after infection, but only in the animals infected with 104 viable organisms. Double diffusion and counterimmunnoelectrophoresis techniques for antibody gave identical results. A. lituratus seems very susceptible to infection with H. capsulatum. A mild infection may stimulate DH to the organism but only low levels of antibody. The footpad test utilized in this study may be a sensitive and practical method for detecting natural infection in bats.
C1 TULANE UNIV INT CTR MED RES, CALI, COLOMBIA.
CR BAUMAN DS, 1975, J CLIN MICROBIOL, V2, P77
BAUMAN DS, 1969, AM REV RESPIR DIS, V100, P79
BIGGAR WD, 1971, ARCH INTERN MED, V128, P585, DOI 10.1001/archinte.128.4.585
DOMER JE, 1976, INFECT IMMUN, V13, P790, DOI 10.1128/IAI.13.3.790-799.1976
Doy R W, 1974, Cent Afr J Med, V20, P119
DYSON JE, 1955, J LAB CLIN MED, V45, P449
EMMONS CW, 1966, AM J EPIDEMIOL, V84, P103, DOI 10.1093/oxfordjournals.aje.a120615
HARRELL WK, 1970, PROCEDURAL MANUAL PR

HASENCLE.HF, 1967, AM J EPIDEMIOL, V86, P238, DOI 10.1093/oxfordjournals.aje.a120728
HASENCLEVER HF, 1972, HEALTH LAB SCI, V9, P125
HASENCLEVER HF, 1969, AM J EPIDEMIOL, V90, P77, DOI 10.1093/oxfordjournals.aje.a121052
HATTEN BA, 1970, J IMMUNOL, V105, P872
KAPLAN W, 1972, INFECT IMMUN, V5, P847, DOI 10.1128/IAI.5.6.847-853.1972
KAPLAN W, 1973, J AM VET MED ASSOC, V163, P1043
KLEGER B, 1973, APPL MICROBIOL, V26, P231, DOI 10.1128/AEM.26.3.231-238.1973
KLITE PD, 1965, AM J TROP MED HYG, V14, P433, DOI 10.4269/ajtmh.1965.14.433
KLITE PD, 1965, ANN INTERN MED, V62, P1263, DOI 10.7326/0003-4819-62-6-1263
Leonard L. L., 1968, Dissertation Abstracts, V28B, P5131
MACKENZIE DWR, 1975, MYCOPATHOLOGIA, V57, P1, DOI 10.1007/BF00431169
MCMURRAY DN, 1976, LAB ANIM SCI, V26, P960
ODDS FC, 1975, J IMMUNOL METHODS, V7, P211, DOI 10.1016/0022-1759(75)90018-6
OELS HC, 1969, AM REV RESPIR DIS, V99, P443
RASWEILER JJ, 1972, LAB ANIM SCI, V22, P658
SCHWARZ J, 1969, MYCOPATH MYCOL APPL, V37, P77, DOI 10.1007/BF02051334
SNYDER SB, 1973, LAB ANIM SCI, V23, P515
STANDARD PG, 1976, J CLIN MICROBIOL, V3, P191
SULKIN SE, 1966, AM J TROP MED HYG, V15, P418, DOI 10.4269/ajtmh.1966.15.418
SWEET GH, 1975, CAN J MICROBIOL, V21, P130, DOI 10.1139/m75-020
TAYLOR RL, 1962, AM J TROP MED HYG, V11, P790, DOI 10.4269/ajtmh.1962.11.790
TESH RB, 1967, AM J EPIDEMIOL, V86, P545, DOI 10.1093/oxfordjournals.aje.a120764
TESH RB, 1966, AM J TROP MED HYG, V15, P544, DOI 10.4269/ajtmh.1966.15.544
TESH RB, 1968, AM J TROP MED HYG, V17, P102, DOI 10.4269/ajtmh.1968.17.102
NR 32
TC 14
Z9 14
U1 0
U2 3
PU AMER SOC TROP MED & HYGIENE
PI MCLEAN
PA 8000 WESTPARK DR, STE 130, MCLEAN, VA 22101 USA
SN 0002-9637
EI 1476-1645
J9 AM J TROP MED HYG
JI Am. J. Trop. Med. Hyg.
PY 1978
VL 27
IS 4
BP 815

EP 821
DI 10.4269/ajtmh.1978.27.815
PG 7
WC Public, Environmental & Occupational Health; Tropical Medicine
WE Science Citation Index Expanded (SCI-EXPANDED)
SC Public, Environmental & Occupational Health; Tropical Medicine
GA FL638
UT WOS:A1978FL63800022
PM 686249
DA 2024-10-21
ER

PT J
AU He, X
   Korytar, T
   Schatz, J
   Freuling, CM
   Müller, T
   Köllner, B
AF He, Xiaocui
   Korytar, Tomas
   Schatz, Juliane
   Freuling, Conrad M.
   Mueller, Thomas
   Koellner, Bernd
TI Anti-Lyssaviral Activity of Interferons κ and ω from the Serotine Bat,
   <i>Eptesicus serotinus</i>
SO JOURNAL OF VIROLOGY
LA English
DT Article
ID PHYLOGENETIC-RELATIONSHIPS; IMMUNE-RESPONSE; FRUIT BAT; RABIES; VIRUS;
   EVOLUTION; SURVEILLANCE; CHIROPTERA; EXPRESSION; MICROBATS
AB Interferons (IFNs) are cytokines produced by host cells in response to the infection with pathogens. By binding to the corresponding receptors, IFNs trigger different pathways to block intracellular replication and growth of pathogens and to impede the infection of surrounding cells. Due to their key role in host defense against viral infections, as well as for clinical therapies, the IFN responses and regulation mechanisms are well studied. However, studies of type I IFNs have mainly focused on alpha interferon (IFN-alpha) and IFN-beta subtypes. Knowledge of IFN-kappa and IFN-omega is limited. Moreover, most studies are performed in humans or mouse models but not in the original host of zoonotic pathogens. Bats are important reservoirs and transmitters of zoonotic viruses such as lyssaviruses. A few studies have shown an antiviral activity of IFNs in fruit bats. However, the function of type I IFNs against lyssaviruses in bats has not been studied yet. Here, IFN-kappa and IFN-omega nes from the European serotine bat, Eptesicus serotinus, were cloned and functionally characterized. E. serotinus IFN-kappa and IFN-omega genes are intronless and well conserved between microchiropteran species. The promoter regions of both genes contain essential regulatory elements for transcription

factors. In vitro studies indicated a strong activation of IFN signaling by recombinant IFN-omega, whereas IFN-kappa displayed weaker activation. Noticeably, both IFNs inhibit to different extents the replication of different lyssaviruses in susceptible bat cell lines. The present study provides functional data on the innate host defense against lyssaviruses in endangered European bats.
C1 [He, Xiaocui; Korytar, Tomas; Koellner, Bernd] Fed Res Inst Anim Hlth, Friedrich Loeffler Inst, Inst Immunol, Greifswald Insel Riems, Germany.
[Schatz, Juliane; Freuling, Conrad M.; Mueller, Thomas] Fed Res Inst Anim Hlth, Friedrich Loeffler Inst, Inst Mol Biol, Greifswald Insel Riems, Germany.
C3 Friedrich Loeffler Institute; Friedrich Loeffler Institute
RP Köllner, B (corresponding author), Fed Res Inst Anim Hlth, Friedrich Loeffler Inst, Inst Immunol, Greifswald Insel Riems, Germany.
EM bernd.koellner@fli.bund.de
RI Korytář, Tomáš/AAY-7683-2020
FU Bundesministerium fur Bildung und Forschung [01KI1016A]
FX This study was financially supported by the Bundesministerium fur Bildung und Forschung (grant 01KI1016A).
CR Altringham JD, 2011, BATS: FROM EVOLUTION TO CONSERVATION, 2ND EDITION, P1
Amengual B, 1997, J GEN VIROL, V78, P2319, DOI 10.1099/0022-1317-78-9-2319
Ceballos NA, 2013, EMERG INFECT DIS, V19, P793, DOI 10.3201/eid1905.121071
Baker ML, 2013, ZOONOSES PUBLIC HLTH, V60, P104, DOI 10.1111/j.1863-2378.2012.01528.x
Biesold SE, 2011, PLOS ONE, V6, DOI 10.1371/journal.pone.0028131
Calisher CH, 2006, CLIN MICROBIOL REV, V19, P531, DOI 10.1128/CMR.00017-06
CHAKRAVARTY AK, 1994, LYMPHOLOGY, V27, P97
Chopy D, 2011, J NEUROVIROL, V17, P353, DOI 10.1007/s13365-011-0041-6
Chopy D, 2011, J VIROL, V85, P6657, DOI 10.1128/JVI.00302-11
De Angioletti M, 2004, BRIT J HAEMATOL, V124, P224, DOI 10.1046/j.1365-2141.2003.04754.x
DIAZ MO, 1994, GENOMICS, V22, P540, DOI 10.1006/geno.1994.1427
Dobson AP, 2005, SCIENCE, V310, P628, DOI 10.1126/science.1120872
Freuling C, 2009, BERL MUNCH TIERARZTL, V122, P425, DOI 10.2376/0005-9366-122-425
Freuling CM, 2011, EMERG INFECT DIS, V17, P1519, DOI 10.3201/eid1708.110201
Halpin K, 2000, J GEN VIROL, V81, P1927, DOI 10.1099/0022-1317-81-8-1927
Hardy MP, 2004, GENOMICS, V84, P331, DOI 10.1016/j.ygeno.2004.03.003
Harris SL, 2006, VET REC, V159, P439, DOI 10.1136/vr.159.14.439
He XC, 2011, FISH SHELLFISH IMMUN, V30, P870, DOI 10.1016/j.fsi.2011.01.009
HUGHES AL, 1995, J MOL EVOL, V41, P539, DOI 10.1007/BF00175811
Janardhana V, 2012, DEV COMP IMMUNOL, V36, P610, DOI 10.1016/j.dci.2011.11.001

Kuzmin IV, 2005, VIRUS RES, V111, P28, DOI 10.1016/j.virusres.2005.03.008
LaFleur DW, 2001, J BIOL CHEM, V276, P39765, DOI 10.1074/jbc.M102502200
Li WD, 2005, SCIENCE, V310, P676, DOI 10.1126/science.1118391
Lieu KG, 2013, J VIROL, V87, P8261, DOI 10.1128/JVI.00989-13
Livak KJ, 2001, METHODS, V25, P402, DOI 10.1006/meth.2001.1262
MINDELL DP, 1991, P NATL ACAD SCI USA, V88, P10322, DOI 10.1073/pnas.88.22.10322
Müller T, 2004, J VET MED B, V51, P49, DOI 10.1111/j.1439-0450.2003.00725.x
Omatsu T, 2008, VET IMMUNOL IMMUNOP, V124, P169, DOI 10.1016/j.vetimm.2008.03.004
PAUL BN, 1986, DEV COMP IMMUNOL, V10, P55, DOI 10.1016/0145-305X(86)90044-3
PETTIGREW JD, 1989, PHILOS T R SOC B, V325, P489, DOI 10.1098/rstb.1989.0102
Rieder M, 2012, BERL MUNCH TIERARZTL, V125, P209, DOI 10.2376/0005-9366-125-209
Rieder M, 2011, ADV VIRUS RES, V79, P91, DOI 10.1016/B978-0-12-387040-7.00006-8
Ruedi M, 2013, MOL PHYLOGENET EVOL, V69, P437, DOI 10.1016/j.ympev.2013.08.011
Schatz J, 2013, ZOONOSES PUBLIC HLTH, V60, P22, DOI 10.1111/zph.12002
Virtue ER, 2011, PLOS ONE, V6, DOI 10.1371/journal.pone.0022488
Walker AM, 2009, BMC GENOMICS, V10, DOI 10.1186/1471-2164-10-187
Wibbelt G, 2010, BIOL LETTERS, V6, P438, DOI 10.1098/rsbl.2010.0267
Wiltzer L, 2012, J VIROL, V86, P10194, DOI 10.1128/JVI.01249-12
Wynne JW, 2013, PLOS PATHOG, V9, DOI 10.1371/journal.ppat.1003651
Zhou P, 2013, DEV COMP IMMUNOL, V40, P240, DOI 10.1016/j.dci.2013.03.006
Zhou P, 2011, J IMMUNOL, V186, P3138, DOI 10.4049/jimmunol.1003115
NR 41
TC 31
Z9 34
U1 0
U2 14
PU AMER SOC MICROBIOLOGY
PI WASHINGTON
PA 1752 N ST NW, WASHINGTON, DC 20036-2904 USA
SN 0022-538X
EI 1098-5514
J9 J VIROL
JI J. Virol.
PD MAY
PY 2014
VL 88
IS 10
BP 5444
EP 5454
DI 10.1128/JVI.03403-13

PG 11
WC Virology
WE Science Citation Index Expanded (SCI-EXPANDED)
SC Virology
GA AG5GB
UT WOS:000335446400023
PM 24574413
OA Bronze, Green Published
DA 2024-10-21
ER

PT J
AU Kadam, A
   Sasidharan, S
   Saudagar, P
AF Kadam, Ajinkya
   Sasidharan, Santanu
   Saudagar, Prakash
TI Computational design of a potential multi-epitope subunit vaccine using
   immunoinformatics to fight Ebola virus
SO INFECTION GENETICS AND EVOLUTION
LA English
DT Article
DE Immunoinformatics; MD simulation; Ebola virus; Multi-epitope vaccine;
   in-silico clone
ID PROTEINS
AB Ebola virus (EBOV) is a rare but fatal disease that has been a burden to mankind for over 40 years. EBOV exhibits several symptoms including severe bleeding, organ failure and if left untreated causes death. It is assumed that fruit bats of the Pteropodidae family are natural hosts for the virus. Over the years, there has been no effective vaccine that can confer immunity to this virus. Considering the necessity of a vaccine against EBOV, this study to develop a multi-epitope subunit vaccine for the EBOV using the immunoinformatics approach was conducted. The construct was designed using structural and non-structural proteins of EBOV. Class I and Class II MHC epitopes were predicted and linked along with beta defensin and compatible linkers. B-cell linear epitopes were also assessed and the physiological parameters of the vaccine were determined. The vaccine was capable of administration to humans and also is capable of an immune response. The vaccine was modeled further and affinity towards the TLR4 receptor was studied by docking and simulation for 20 ns. The trajectory analysis high affinity between the vaccine and the construct with an average hydrogen bond of 18. For ease of purification, the vaccine construct was ligated into pET28a(+) vector with His-tag. Concluding from the results, the vaccine construct has the potentiality to help develop immunity against the Ebola virus. Furthermore, experimental and immunological investigations will be required to verify the feasibility of the multi-epitope subunit construct as a commercial vaccine.
C1 [Kadam, Ajinkya; Sasidharan, Santanu; Saudagar, Prakash] Natl

Inst Technol, Dept Biotechnol, Warangal 506004, Telangana, India.
C3 National Institute of Technology (NIT System); National Institute of
Technology Warangal
RP Saudagar, P (corresponding author), Natl Inst Technol, Dept Biotechnol, Warangal 506004, Telangana, India.
EM ps@nitw.ac.in
RI Saudagar, Prakash/ABE-7270-2020; Saudagar, Prakash/AAV-8341-2021;
Sasidharan, Santanu/M-3429-2016
OI Saudagar, Prakash/0000-0001-7211-0581; Sasidharan,
Santanu/0000-0002-0204-0263
FU NIT Warangal
FX The authors thank Centre for Automation and Instrumentation (CAI), NIT
Warangal for extending the computational power and facility required to
carry out this work. The author SS acknowledges research fellowship from
NIT Warangal.
CR Armanini D, 2005, STEROIDS, V70, P538, DOI 10.1016/j.steroids.2005.01.007
Bausch DG, 2007, J INFECT DIS, V196, pS142, DOI 10.1086/520545
Bohra N, 2020, MOL SIMULAT, V46, P419, DOI 10.1080/08927022.2020.1720916
Carbone A, 2003, BIOINFORMATICS, V19, P2005, DOI 10.1093/bioinformatics/btg272
Doytchinova IA, 2007, BMC BIOINFORMATICS, V8, DOI 10.1186/1471-2105-8-4
Feldmann H., 2013, FIELDS VIROLOGY
Genton C, 2012, PLOS ONE, V7, DOI 10.1371/journal.pone.0037106
GURUPRASAD K, 1990, PROTEIN ENG, V4, P155, DOI 10.1093/protein/4.2.155
Hajighahramani N, 2017, INFECT GENET EVOL, V48, P83, DOI 10.1016/j.meegid.2016.12.010
Henao-Restrepo AM, 2017, LANCET, V389, P505, DOI 10.1016/S0140-6736(16)32621-6
Ikram A, 2018, SCI REP-UK, V8, DOI 10.1038/s41598-018-34254-5
Kalita J, 2020, INFECT GENET EVOL, V83, DOI 10.1016/j.meegid.2020.104352
Kalita P, 2019, INT J BIOL MACROMOL, V138, P224, DOI 10.1016/j.ijbiomac.2019.07.024
KLAVINSKIS LS, 1989, J VIROL, V63, P4311, DOI 10.1128/JVI.63.10.4311-4316.1989
Larsen MV, 2007, BMC BIOINFORMATICS, V8, DOI 10.1186/1471-2105-8-424
신웅희, 2014, Biodesign, V2, P1
Lin HH, 2008, BMC IMMUNOL, V9, DOI 10.1186/1471-2172-9-8
Lund O, 2004, IMMUNOGENETICS, V55, P797, DOI 10.1007/s00251-004-0647-4
Mittal A, 2020, INT J PEPT RES THER, V26, P2231, DOI 10.1007/s10989-020-10020-y
Patronov A, 2013, OPEN BIOL, V3, DOI 10.1098/rsob.120139
Raj S, 2019, PLOS ONE, V14, DOI 10.1371/journal.pone.0221331
Saha S, 2006, NUCLEIC ACIDS RES, V34, pW202, DOI 10.1093/nar/

gkl343
   Sasidharan S, 2019, FEBS OPEN BIO, V9, P1769, DOI
10.1002/2211-5463.12715
   Schiller JT, 2015, J INFECT DIS, V211, P1373, DOI 10.1093/infdis/
jiu648
   Sievers F, 2011, MOL SYST BIOL, V7, DOI 10.1038/msb.2011.75
   Towner JS, 2008, PLOS PATHOG, V4, DOI 10.1371/
journal.ppat.1000212
   Vita R, 2015, NUCLEIC ACIDS RES, V43, pD405, DOI 10.1093/nar/
gku938
   Wang P, 2008, PLOS COMPUT BIOL, V4, DOI 10.1371/
journal.pcbi.1000048
   Yang JY, 2015, NAT METHODS, V12, P7, DOI 10.1038/nmeth.3213
NR 29
TC 20
Z9 21
U1 0
U2 0
PU ELSEVIER
PI AMSTERDAM
PA RADARWEG 29, 1043 NX AMSTERDAM, NETHERLANDS
SN 1567-1348
EI 1567-7257
J9 INFECT GENET EVOL
JI Infect. Genet. Evol.
PD NOV
PY 2020
VL 85
AR 104464
DI 10.1016/j.meegid.2020.104464
PG 8
WC Infectious Diseases
WE Science Citation Index Expanded (SCI-EXPANDED)
SC Infectious Diseases
GA OX9ZU
UT WOS:000593915100020
PM 32681997
DA 2024-10-21
ER

PT J
AU Hayward, JA
   Tachedjian, M
   Cui, J
   Cheng, AZ
   Johnson, A
   Baker, ML
   Harris, RS
   Wang, LF
   Tachedjian, G
AF Hayward, Joshua A.
   Tachedjian, Mary
   Cui, Jie
   Cheng, Adam Z.

Johnson, Adam
Baker, Michelle L.
Harris, Reuben S.
Wang, Lin-Fa
Tachedjian, Gilda
TI Differential Evolution of Antiretroviral Restriction Factors in Pteropid
Bats as Revealed by <i>APOBEC3</i> Gene Complexity
SO MOLECULAR BIOLOGY AND EVOLUTION
LA English
DT Article
DE restriction factors; bats; APOBEC3; evolution; antiviral immunity
ID IMMUNODEFICIENCY-VIRUS TYPE-1; EXPERIMENTAL INOCULATION;
ROUSETTUS-AEGYPTIACUS; CYTIDINE DEAMINASES; CRYSTAL-STRUCTURE; ENZYME
APOBEC3G; HENDRA VIRUS; NIPAH VIRUS; FLYING FOX; FRUIT BATS
AB Bats have attracted attention in recent years as important reservoirs of viruses deadly to humans and other mammals. These infections are typically nonpathogenic in bats raising questions about innate immune differences that might exist between bats and other mammals. The APOBEC3 gene family encodes antiviral DNA cytosine deaminases with important roles in the suppression of diverse viruses and genomic parasites. Here, we characterize pteropid APOBEC3 genes and show that species within the genus Pteropus possess the largest and most diverse array of APOBEC3 genes identified in any mammal reported to date. Several bat APOBEC3 proteins are antiviral as demonstrated by restriction of retroviral infectivity using HIV-1 as a model, and recombinant A3Z1 subtypes possess strong DNA deaminase activity. These genes represent the first group of antiviral restriction factors identified in bats with extensive diversification relative to homologues in other mammals.
C1 [Hayward, Joshua A.; Johnson, Adam; Tachedjian, Gilda] Burnet Inst, Hlth Secur Program, Life Sci Discipline, Melbourne, Vic, Australia.
[Hayward, Joshua A.; Tachedjian, Gilda] Monash Univ, Dept Microbiol, Clayton, Vic, Australia.
[Tachedjian, Mary; Baker, Michelle L.] CSIRO, Australian Anim Hlth Lab, Hlth & Biosecur Business Unit, Geelong, Vic, Australia.
[Cui, Jie] Chinese Acad Sci, Wuhan Inst Virol, Ctr Emerging Infect Dis, Key Lab Special Pathogens & Biosafety, Wuhan, Hubei, Peoples R China.
[Cheng, Adam Z.; Harris, Reuben S.] Univ Minnesota, Dept Biochem Mol Biol & Biophys, Inst Mol Virol, Minneapolis, MN USA.
[Harris, Reuben S.] Univ Minnesota, Howard Hughes Med Inst, Minneapolis, MN USA.
[Wang, Lin-Fa] Duke NUS Med Sch, Programme Emerging Infect Dis, Singapore, Singapore.
[Tachedjian, Gilda] RMIT Univ, Sch Sci, Coll Sci Engn & Hlth, Melbourne, Vic, Australia.
[Tachedjian, Gilda] Univ Melbourne, Doherty Inst Infect & Immun, Dept Microbiol & Immunol, Melbourne, Vic, Australia.
C3 Burnet Institute; Monash University; Commonwealth Scientific &
Industrial Research Organisation (CSIRO); Chinese Academy of Sciences;

Wuhan Institute of Virology, CAS; University of Minnesota System;
University of Minnesota Twin Cities; University of Minnesota System;
University of Minnesota Twin Cities; Howard Hughes Medical Institute;
National University of Singapore; Royal Melbourne Institute of
Technology (RMIT); University of Melbourne; Peter Doherty Institute
RP Tachedjian, G (corresponding author), Burnet Inst, Hlth Secur
Program, Life Sci Discipline, Melbourne, Vic, Australia.;
Tachedjian, G (corresponding author), Monash Univ, Dept Microbiol,
Clayton, Vic, Australia.; Tachedjian, G (corresponding author), RMIT
Univ, Sch Sci, Coll Sci Engn & Hlth, Melbourne, Vic, Australia.;
Tachedjian, G (corresponding author), Univ Melbourne, Doherty Inst
Infect & Immun, Dept Microbiol & Immunol, Melbourne, Vic, Australia.
EM gildat@burnet.edu.au
RI Baker, Michelle/AAX-9171-2020; Tachedjian, Gilda/AAF-1327-2020; Baker,
Michelle/C-9694-2013; Cui, Jie/ABB-3831-2020
OI Wang, Lin-Fa/0000-0003-2752-0535; Cheng, Adam/0000-0001-6277-9433;
Tachedjian, Gilda/0000-0002-7733-7037; Hayward,
Joshua/0000-0003-3904-3051; Baker, Michelle/0000-0002-7993-9971; Cui,
Jie/0000-0001-8176-9951
FU Perpetual Trustees [AR/00255]; Australian Postgraduate Award; Monash
University Vice Chancellor's Honours-PhD scholarship; National Health
and Medical Research Council [APP1117748]; National Research
Foundation-Competitive Research Programme [NRF2012NRF-CRP001-056];
National Institute of Health [R37AI064046]; National Institute of
Allergy and Infectious Diseases [R37AI064046]; National Natural Science
Foundation of China [31671324]; CAS Pioneer Hundred Talents Program
FX This study was supported by Perpetual Trustees (AR/00255 to G.T. and
L.-F.W.), the Australian Postgraduate Award and the Monash University
Vice Chancellor's Honours-PhD scholarship (to J.A.H.), the National
Health and Medical Research Council Senior Research Fellowship
(APP1117748 to G.T.), the National Research Foundation-Competitive
Research Programme (NRF2012NRF-CRP001-056 to L.-F.W.), and the National
Institute of Health and National Institute of Allergy and Infectious
Diseases (R37AI064046 to R.S.H.). R.S.H. is an Investigator of the
Howard Hughes Medical Institute and J.C. is supported by National
Natural Science Foundation of China (31671324) and CAS Pioneer

Hundred
Talents Program. The authors gratefully acknowledge the contribution to
this work of the Victorian Operational Infrastructure Support Program by
the Burnet Institute. The pteropid A3 loci and cDNA sequences have been
deposited in GenBank and are listed in supplementary table S2,
Supplementary Material online.
CR Almeida FC, 2014, MOL PHYLOGENET EVOL, V77, P83, DOI 10.1016/j.ympev.2014.03.009
Amman BR, 2015, J WILDLIFE DIS, V51, P774, DOI 10.7589/2015-02-044
Baker ML, 2013, ZOONOSES PUBLIC HLTH, V60, P104, DOI 10.1111/j.1863-2378.2012.01528.x
Berger G, 2011, PLOS PATHOG, V7, DOI 10.1371/journal.ppat.1002221
Burns MB, 2015, BIOMED J, V38, P102, DOI 10.4103/2319-4170.148904
Calisher CH, 2006, CLIN MICROBIOL REV, V19, P531, DOI 10.1128/CMR.00017-06
Cantrell MA, 2008, GENETICS, V178, P393, DOI 10.1534/genetics.107.080275
Chen H, 2006, CURR BIOL, V16, P480, DOI 10.1016/j.cub.2006.01.031
Chiu YL, 2008, ANNU REV IMMUNOL, V26, P317, DOI 10.1146/annurev.immunol.26.021607.090350
Conticello SG, 2005, MOL BIOL EVOL, V22, P367, DOI 10.1093/molbev/msi026
Cooke MS, 2003, FASEB J, V17, P1195, DOI 10.1096/fj.02-0752rev
Cowled C, 2011, DEV COMP IMMUNOL, V35, P7, DOI 10.1016/j.dci.2010.07.006
Cui J, 2015, SCI REP-UK, V5, DOI 10.1038/srep16561
Cui J, 2012, J GEN VIROL, V93, P2037, DOI 10.1099/vir.0.043760-0
Drummond AJ, 2005, MOL BIOL EVOL, V22, P1185, DOI [10.1093/molbev/msi103, 10.1093/molbev/mss075]
Edgar RC, 2004, NUCLEIC ACIDS RES, V32, P1792, DOI 10.1093/nar/gkh340
Fang JF, 2015, PLOS ONE, V10, DOI 10.1371/journal.pone.0131296
Farré D, 2003, NUCLEIC ACIDS RES, V31, P3651, DOI 10.1093/nar/gkg605
Green AM, 2016, CELL CYCLE, V15, P998, DOI 10.1080/15384101.2016.1152426
Halpin K, 2000, J GEN VIROL, V81, P1927, DOI 10.1099/0022-1317-81-8-1927
Harris RS, 2002, MOL CELL, V10, P1247, DOI 10.1016/S1097-2765(02)00742-6
Hayman DTS, 2016, ANNU REV VIROL, V3, P77, DOI 10.1146/annurev-virology-110615-042203
Hayward JA, 2013, RETROVIROLOGY, V10, DOI 10.1186/1742-4690-10-35
Holden LG, 2008, NATURE, V456, P121, DOI 10.1038/nature07357
Jones MEB, 2015, VIRUSES-BASEL, V7, P3420, DOI 10.3390/v7072779
Kane JR, 2015, CELL REP, V11, P1236, DOI 10.1016/j.celrep.2015.04.038
Kinomoto M, 2007, NUCLEIC ACIDS RES, V35, P2955, DOI 10.1093/nar/gkm181
Koito A, 2013, FRONT MICROBIOL, V4, DOI 10.3389/fmicb.2013.00028

Kumar S, 2017, MOL BIOL EVOL, V34, P1812, DOI 10.1093/molbev/msx116
Lackey L, 2013, CELL CYCLE, V12, P762, DOI 10.4161/cc.23713
Land AM, 2013, J BIOL CHEM, V288, P17253, DOI 10.1074/jbc.M113.458661
Larue RS, 2008, BMC MOL BIOL, V9, DOI 10.1186/1471-2199-9-104
Larue RS, 2009, J VIROL, V83, P494, DOI 10.1128/JVI.01976-08
Lecossier D, 2003, SCIENCE, V300, P1112, DOI 10.1126/science.1083338
Lee YN, 2008, J VIROL, V82, P8762, DOI 10.1128/JVI.00751-08
Mariani R, 2003, CELL, V114, P21, DOI 10.1016/S0092-8674(03)00515-4
Marin M, 2003, NAT MED, V9, P1398, DOI 10.1038/nm946
Martin DP, 2010, BIOINFORMATICS, V26, P2462, DOI 10.1093/bioinformatics/btq467
METZENBERG AB, 1991, GENETICS, V128, P143
Middleton DJ, 2007, J COMP PATHOL, V136, P266, DOI 10.1016/j.jcpa.2007.03.002
Muckenfuss H, 2006, J BIOL CHEM, V281, P22161, DOI 10.1074/jbc.M601716200
Nakano Y, 2017, RETROVIROLOGY, V14, DOI 10.1186/s12977-017-0355-4
O'Shea TJ, 2014, EMERG INFECT DIS, V20, P741, DOI 10.3201/eid2005.130539
Papenfuss AT, 2012, BMC GENOMICS, V13, DOI 10.1186/1471-2164-13-261
Perez-Caballero D, 2008, PLOS PATHOG, V4, DOI 10.1371/journal.ppat.1000181
Rahman SA, 2010, EMERG INFECT DIS, V16, P1990, DOI 10.3201/eid1612.091790
Reese MG, 2001, COMPUT CHEM, V26, P51, DOI 10.1016/S0097-8485(01)00099-7
Refsland EW, 2013, CURR TOP MICROBIOL, V371, P1, DOI 10.1007/978-3-642-37765-5_1
Refsland EW, 2010, NUCLEIC ACIDS RES, V38, P4274, DOI 10.1093/nar/gkq174
Renard M, 2010, J MOL BIOL, V400, P323, DOI 10.1016/j.jmb.2010.05.029
Roberts SA, 2013, NAT GENET, V45, P970, DOI 10.1038/ng.2702
Rose PP, 2000, BIOINFORMATICS, V16, P400, DOI 10.1093/bioinformatics/16.4.400
Sheehy AM, 2003, NAT MED, V9, P1404, DOI 10.1038/nm945
Sheehy AM, 2002, NATURE, V418, P646, DOI 10.1038/nature00939
Shen YY, 2010, P NATL ACAD SCI USA, V107, P8666, DOI 10.1073/pnas.0912613107
Shi K, 2015, J BIOL CHEM, V290, P28120, DOI 10.1074/jbc.M115.679951
SIMON JHM, 1995, J VIROL, V69, P4166, DOI 10.1128/JVI.69.7.4166-4172.1995
Smith I, 2013, CURR OPIN VIROL, V3, P84, DOI 10.1016/j.coviro.2012.11.006
Stenglein MD, 2006, J BIOL CHEM, V281, P16837, DOI 10.1074/jbc.M602367200
Swanepoel R, 1996, EMERG INFECT DIS, V2, P321, DOI 10.3201/eid0204.960407

Talavera G, 2007, SYST BIOL, V56, P564, DOI 10.1080/10635150701472164
Tamura K, 2011, MOL BIOL EVOL, V28, P2731, DOI 10.1093/molbev/msr121
Taylor BJM, 2013, ELIFE, V2, DOI 10.7554/eLife.00534
Tyssen D, 2010, PLOS ONE, V5, DOI 10.1371/journal.pone.0012309
Wapling J, 2005, J VIROL, V79, P10247, DOI 10.1128/JVI.79.16.10247-10257.2005
Warren CJ, 2015, J VIROL, V89, P688, DOI 10.1128/JVI.02383-14
Watanabe S, 2010, EMERG INFECT DIS, V16, P1217, DOI 10.3201/eid1608.100208
Wei XP, 2002, ANTIMICROB AGENTS CH, V46, P1896, DOI 10.1128/AAC.46.6.1896-1905.2002
Williamson MM, 1998, AUST VET J, V76, P813, DOI 10.1111/j.1751-0813.1998.tb12335.x
Wissing S, 2011, J BIOL CHEM, V286, P36427, DOI 10.1074/jbc.M111.251058
Yu Q, 2004, J BIOL CHEM, V279, P53379, DOI 10.1074/jbc.M408802200
Zhang GJ, 2013, SCIENCE, V339, P456, DOI 10.1126/science.1230835
Zheng YH, 2004, J VIROL, V78, P6073, DOI 10.1128/JVI.78.11.6073-6076.2004
Zhou P, 2016, P NATL ACAD SCI USA
NR 74
TC 39
Z9 44
U1 0
U2 10
PU OXFORD UNIV PRESS
PI OXFORD
PA GREAT CLARENDON ST, OXFORD OX2 6DP, ENGLAND
SN 0737-4038
EI 1537-1719
J9 MOL BIOL EVOL
JI Mol. Biol. Evol.
PD JUL
PY 2018
VL 35
IS 7
BP 1626
EP 1637
DI 10.1093/molbev/msy048
PG 12
WC Biochemistry & Molecular Biology; Evolutionary Biology; Genetics &
Heredity
WE Science Citation Index Expanded (SCI-EXPANDED)
SC Biochemistry & Molecular Biology; Evolutionary Biology; Genetics &
Heredity
GA GP8DY
UT WOS:000441141400006
PM 29617834
OA Green Published, hybrid
DA 2024-10-21

ER

PT J
AU Liu, XJ
   Li, C
   Wan, ZX
   Chiu, MC
   Huang, JJ
   Yu, YF
   Zhu, L
   Cai, JP
   Rong, L
   Song, YQ
   Chu, H
   Cai, ZW
   Jiang, SB
   Yuen, KY
   Zhou, J
AF Liu, Xiaojuan
   Li, Cun
   Wan, Zhixin
   Chiu, Man Chun
   Huang, Jingjing
   Yu, Yifei
   Zhu, Lin
   Cai, Jian-Piao
   Rong, Lei
   Song, You-qiang
   Chu, Hin
   Cai, Zongwei
   Jiang, Shibo
   Yuen, Kwok-yung
   Zhou, Jie
TI Analogous comparison unravels heightened antiviral defense and boosted
   viral infection upon immunosuppression in bat organoids
SO SIGNAL TRANSDUCTION AND TARGETED THERAPY
LA English
DT Article
ID RESPIRATORY SYNDROME CORONAVIRUS; INFLAMMATORY CYTOKINES; CELL-LINES;
   REPLICATION; INTERFERON; MECHANISMS; EVOLUTION; REVEALS
AB Horseshoe bats host numerous SARS-related coronaviruses without overt disease signs. Bat intestinal organoids, a unique model of bat intestinal epithelium, allow direct comparison with human intestinal organoids. We sought to unravel the cellular mechanism(s) underlying bat tolerance of coronaviruses by comparing the innate immunity in bat and human organoids. We optimized the culture medium, which enabled a consecutive passage of bat intestinal organoids for over one year. Basal expression levels of IFNs and IFN-stimulated genes were higher in bat organoids than in their human counterparts. Notably, bat organoids mounted a more rapid, robust and prolonged antiviral defense than human organoids upon Poly(I:C) stimulation. TLR3 and RLR might be the conserved pathways mediating antiviral

response in bat and human intestinal organoids. The susceptibility of bat organoids to a bat coronavirus CoV-HKU4, but resistance to EV-71, an enterovirus of exclusive human origin, indicated that bat organoids adequately recapitulated the authentic susceptibility of bats to certain viruses. Importantly, TLR3/RLR inhibition in bat organoids significantly boosted viral growth in the early phase after SARS-CoV-2 or CoV-HKU4 infection. Collectively, the higher basal expression of antiviral genes, especially more rapid and robust induction of innate immune response, empowered bat cells to curtail virus propagation in the early phase of infection.
C1 [Liu, Xiaojuan; Li, Cun; Wan, Zhixin; Chiu, Man Chun; Huang, Jingjing; Yu, Yifei; Cai, Jian-Piao; Chu, Hin; Yuen, Kwok-yung; Zhou, Jie] Univ Hong Kong, Li Ka Shing Fac Med, Sch Clin Med, Dept Microbiol, Hong Kong, Peoples R China.
[Zhu, Lin; Cai, Zongwei] Hong Kong Baptist Univ, Dept Chem, State Key Lab Environm & Biol Anal, Hong Kong, Peoples R China.
[Rong, Lei; Song, You-qiang] Univ Hong Kong, Shenzhen Hosp, Dept Med, Shenzhen, Guangdong, Peoples R China.
[Song, You-qiang] Univ Hongkong, Sch Biomed Sci, State Key Lab Brain & Cognit Sci, Hong Kong, Peoples R China.
[Chu, Hin; Yuen, Kwok-yung; Zhou, Jie] Univ Hong Kong, Carol Yu Ctr Infect, State Key Lab Emerging Infect Dis, Hong Kong, Peoples R China.
[Chu, Hin; Yuen, Kwok-yung; Zhou, Jie] Hong Kong Sci & Technol Pk, Ctr Virol Vaccinol & Therapeut, Hong Kong, Peoples R China.
[Jiang, Shibo] Fudan Univ, Inst Infect Dis & Biosecur, Sch Basic Med Sci, Key Lab Med Mol Virol MOE NHC CAMS, Shanghai, Peoples R China.
C3 University of Hong Kong; Hong Kong Baptist University; University of
Hong Kong; University of Hong Kong; University of Hong Kong; Fudan
University
RP Yuen, KY; Zhou, J (corresponding author), Univ Hong Kong, Li Ka Shing Fac Med, Sch Clin Med, Dept Microbiol, Hong Kong, Peoples R China.; Yuen, KY; Zhou, J (corresponding author), Univ Hong Kong, Carol Yu Ctr Infect, State Key Lab Emerging Infect Dis, Hong Kong, Peoples R China.; Yuen, KY; Zhou, J (corresponding author), Hong Kong Sci & Technol Pk, Ctr Virol Vaccinol & Therapeut, Hong Kong, Peoples R China.; Jiang, SB (corresponding author), Fudan Univ, Inst Infect Dis & Biosecur, Sch Basic Med Sci, Key Lab Med Mol Virol MOE NHC CAMS, Shanghai, Peoples R China.
EM shibojiang@fudan.edu.cn; kyyuen@hku.hk; jiezhou@hku.hk
RI Chu, Hin/HKF-0538-2023; Song, You-Qiang/E-5207-2011; Cai,
Jian-piao/GYD-5763-2022; Zhu, Lin/ADP-1657-2022; Jiang,
Shijin/E-5096-2013; zhong, jing/KBP-7800-2024; huang,
jing/JDC-2548-2023; Yu, Yifei/KFB-4358-2024; Yuen, Kwok/
C-4465-2009;
Chiu, Man Chun/JBS-3531-2023
OI Cai, Jian-Piao/0000-0003-4077-3852; Zhu, Lin/0000-0002-2801-3626; Song,
You-Qiang/0000-0001-9407-2256; Chiu, Man Chun/0000-0001-9503-6396
FU Health and Medical Research Fund (HMRF) of the Food and Health Bureau of

the HKSAR government [17161272, 19180392]; Research Grants Council of
HKSAR government [17105420, C7042-21G, T11-709/21-N]; Health@InnoHK,
Innovation and Technology Commission; HKSAR Government
FX We thank Professors Christopher Garcia (Standford University) and Hans
Clevers (Hubrecht Institute) for sharing the Wnt surrogate plasmid. We
thank the Center of PanorOmic Sciences and Electron Microscope Unit, Li
Ka Shing Faculty of Medicine, University of Hong Kong, for assistance in
confocal imaging flow cytometry, and electron microscopy. This work was
partly supported by funding from the Health and Medical Research Fund
(HMRF, 17161272 and 19180392) of the Food and Health Bureau of the HKSAR
government to J.Z.; General Research Fund (GRF, 17105420), Collaborative
Research Fund (CRF, C7042-21G) and Theme-based Research Scheme (TbRS,
T11-709/21-N) of the Research Grants Council of HKSAR government to
J.Z.; Health@InnoHK, Innovation and Technology Commission, HKSAR
Government to K.Y.Y.
NR 0
TC 5
Z9 5
U1 1
U2 12
PU SPRINGERNATURE
PI LONDON
PA CAMPUS, 4 CRINAN ST, LONDON, N1 9XW, ENGLAND
SN 2095-9907
EI 2059-3635
J9 SIGNAL TRANSDUCT TAR
JI Signal Transduct. Target. Ther.
PD DEC 19
PY 2022
VL 7
IS 1
AR 392
DI 10.1038/s41392-022-01247-w
PG 12
WC Biochemistry & Molecular Biology; Cell Biology
WE Science Citation Index Expanded (SCI-EXPANDED)
SC Biochemistry & Molecular Biology; Cell Biology
GA 7C9LI
UT WOS:000900125100001
PM 36529763
OA gold, Green Published
DA 2024-10-21

ER

PT J
AU Seltmann, A
   Czirják, GA
   Courtiol, A
   Bernard, H
   Struebig, MJ
   Voigt, CC
AF Seltmann, Anne
   Czirjak, Gabor A.
   Courtiol, Alexandre
   Bernard, Henry
   Struebig, Matthew J.
   Voigt, Christian C.
TI Habitat disturbance results in chronic stress and impaired health status
   in forest-dwelling paleotropical bats
SO CONSERVATION PHYSIOLOGY
LA English
DT Article
DE Anthropogenic disturbance; bats; body mass; chronic stress;
   fragmentation; white blood cell count
ID PHYSIOLOGICAL STRESS; DISEASE RISK; FRAGMENTATION; RESERVOIRS;
   MITOCHONDRIAL; RESPONSES; ECOLOGY; VIRUS; BIODIVERSITY; PHYLOGENIES
AB Anthropogenic habitat disturbance is a major threat to biodiversity worldwide. Yet, before population declines are detectable, individuals may suffer from chronic stress and impaired immunity in disturbed habitats, making them more susceptible to pathogens and adverse weather conditions. Here, we tested in a paleotropical forest with ongoing logging and fragmentation, whether habitat disturbance influences the body mass and immunity of bats. We measured and compared body mass, chronic stress (indicated by neutrophil to lymphocyte ratios) and the number of circulating immune cells between several bat species with different roost types living in recovering areas, actively logged forests, and fragmented forests in Sabah, Malaysia. In a cave-roosting species, chronic stress levels were higher in individuals from fragmented habitats compared with conspecifics from actively logged areas. Foliage-roosting species showed a reduced body mass and decrease in total white blood cell counts in actively logged areas and fragmented forests compared with conspecifics living in recovering habitats. Our study highlights that habitat disturbance may have species-specific effects on chronic stress and immunity in bats that are potentially related to the roost type. We identified foliage-roosting species as particularly sensitive to forest habitat deterioration. These species may face a heightened extinction risk in the near future if anthropogenic habitat alterations continue.
C1 [Seltmann, Anne; Voigt, Christian C.] Leibniz Inst Zoo & Wildlife Res, Dept Evolutionary Ecol, Alfred Kowalke Str 17, D-10315 Berlin, Germany.
   [Seltmann, Anne; Voigt, Christian C.] Free Univ Berlin, Inst Biol, Takustr 3, D-14195 Berlin, Germany.

[Czirjak, Gabor A.] Leibniz Inst Zoo & Wildlife Res, Dept
Wildlife Dis, Alfred Kowalke Str 17, D-10315 Berlin, Germany.
[Courtiol, Alexandre] Leibniz Inst Zoo & Wildlife Res, Dept
Evolutionary Genet, Alfred Kowalke Str 17, D-10315 Berlin, Germany.
[Bernard, Henry] Univ Malaysia Sabah, Inst Trop Biol & Conservat,
Jalan UMS, Kota Kinabalu 88400, Sabah, Malaysia.
[Struebig, Matthew J.] Univ Kent, DICE, Sch Anthropol &
Conservat, Canterbury CT2 7NR, Kent, England.
C3 Leibniz Institut fur Zoo und Wildtierforschung; Free University
of
Berlin; Leibniz Institut fur Zoo und Wildtierforschung; Leibniz
Institut
fur Zoo und Wildtierforschung; Universiti Malaysia Sabah;
University of
Kent
RP Seltmann, A (corresponding author), Leibniz Inst Zoo & Wildlife
Res, Dept Evolutionary Ecol, Alfred Kowalke Str 17, D-10315 Berlin,
Germany.
EM seltmann@izw-berlin.de
RI Czirják, Gábor/F-5440-2011; Courtiol, Alexandre/O-3040-2017
OI Courtiol, Alexandre/0000-0003-0637-2959; Czirjak, Gabor
Arpad/0000-0001-9488-0069; struebig, matthew/0000-0003-2058-8502
FU German Research Foundation (DFG Priority Programme 1596); UK
Natural
Environment Research Council (Human Modified Tropical Forests
programme
under the Land-use Options for Maintaining Biodiversity and
Ecosystem
Function consortium); Open Access Fund of the Leibniz Association
FX This work was supported by the German Research Foundation (DFG
Priority
Programme 1596) and the UK Natural Environment Research Council
(Human
Modified Tropical Forests programme under the Land-use Options
for
Maintaining Biodiversity and Ecosystem Function consortium). The
publication of this article was funded by the Open Access Fund of
the
Leibniz Association.
CR Allen LC, 2009, J COMP PHYSIOL B, V179, P315, DOI 10.1007/
s00360-008-0315-3
[Anonymous], DISCOVERING STAT USI
[Anonymous], 2019, CAR AN R COMPANION A
[Anonymous], BATS VIRUSES NEW FRO
[Anonymous], 2006, FOREST BAT SURVEYS U
Baker ML, 2013, ZOONOSES PUBLIC HLTH, V60, P104, DOI 10.1111/
j.1863-2378.2012.01528.x
Baker ML, 2015, BATS AND VIRUSES, P327
Barnosky AD, 2011, NATURE, V471, P51, DOI 10.1038/nature09678
Boonstra R, 2013, FUNCT ECOL, V27, P11, DOI
10.1111/1365-2435.12008
Brook CE, 2015, TRENDS MICROBIOL, V23, P172, DOI 10.1016/
j.tim.2014.12.004
Buchanan KL, 2000, TRENDS ECOL EVOL, V15, P156, DOI 10.1016/

S0169-5347(99)01812-1
Chua KB, 2000, SCIENCE, V288, P1432, DOI 10.1126/science.288.5470.1432
Davis AK, 2008, FUNCT ECOL, V22, P760, DOI 10.1111/j.1365-2435.2008.01467.x
Davis AK, 2005, J FIELD ORNITHOL, V76, P334, DOI 10.1648/0273-8570-76.4.334
Demas GE, 2011, J ANIM ECOL, V80, P710, DOI 10.1111/j.1365-2656.2011.01813.x
Dhabhar FS, 1996, J IMMUNOL, V157, P1638
Díaz M, 1999, ACTA OECOL, V20, P39, DOI 10.1016/S1146-609X(99)80014-9
Dickens MJ, 2013, GEN COMP ENDOCR, V191, P177, DOI 10.1016/j.ygcen.2013.06.014
Dinno A, 2015, STATA J, V15, P292, DOI 10.1177/1536867X1501500117
Ellis RD, 2012, LANDSCAPE ECOL, V27, P1, DOI 10.1007/s10980-011-9671-6
Estrada-Villegas S, 2010, BIOL CONSERV, V143, P597, DOI 10.1016/j.biocon.2009.11.009
Ewers RM, 2015, NAT COMMUN, V6, DOI 10.1038/ncomms7836
FELSENSTEIN J, 1985, AM NAT, V125, P1, DOI 10.1086/284325
Fitzherbert EB, 2008, TRENDS ECOL EVOL, V23, P538, DOI 10.1016/j.tree.2008.06.012
FOX J, 1992, J AM STAT ASSOC, V87, P178, DOI 10.2307/2290467
Gaveau DLA, 2014, PLOS ONE, V9, DOI 10.1371/journal.pone.0101654
Gibson L, 2011, NATURE, V478, P378, DOI 10.1038/nature10425
GRAFEN A, 1989, PHILOS T R SOC B, V326, P119, DOI 10.1098/rstb.1989.0106
Habel JC, 2014, HEREDITY, V113, P205, DOI 10.1038/hdy.2014.15
Haddad NM, 2015, SCI ADV, V1, DOI 10.1126/sciadv.1500052
Harmon LJ, 2008, BIOINFORMATICS, V24, P129, DOI 10.1093/bioinformatics/btm538
Harrel FE., 2001, REGRESSION MODELING
Hinam HL, 2008, BIOL CONSERV, V141, P524, DOI 10.1016/j.biocon.2007.11.011
Hinsley SA, 2000, LANDSCAPE ECOL, V15, P765, DOI 10.1023/A:1008149403852
Hothorn T, 2008, BIOMETRICAL J, V50, P346, DOI 10.1002/bimj.200810425
Irwin MT, 2010, AM J PRIMATOL, V72, P1013, DOI 10.1002/ajp.20847
Johnstone CP, 2014, BIOL CONSERV, V176, P80, DOI 10.1016/j.biocon.2014.04.025
Johnstone CP, 2012, J COMP PHYSIOL B, V182, P861, DOI 10.1007/s00360-012-0656-9
Johnstone CP, 2012, J COMP PHYSIOL B, V182, P139, DOI 10.1007/s00360-011-0598-7
Johnstone CP, 2011, PLOS ONE, V6, DOI 10.1371/journal.pone.0027158
Johnstone CP, 2010, J MAMMAL, V91, P1332, DOI 10.1644/10-MAMM-A-034.1
Jones Gareth, 2009, Endangered Species Research, V8, P93, DOI 10.3354/esr00182
Jones KE, 2003, AM NAT, V161, P601, DOI 10.1086/368289
Khan FAA, 2010, J MAMMAL, V91, P1058, DOI 10.1644/09-MAMM-A-361.1

Kingston T, 2003, J TROP ECOL, V19, P67, DOI 10.1017/S0266467403003080
Kingston T., 2006, Bats of Krau Wildlife Reserve
KUNZ TH, 1982, J MAMMAL, V63, P23, DOI 10.2307/1380667
Kunz Thomas H., 2009, P36
Lau SKP, 2010, J VIROL, V84, P2808, DOI 10.1128/JVI.02219-09
Laurance WF, 2014, TRENDS ECOL EVOL, V29, P107, DOI 10.1016/j.tree.2013.12.001
Lens L, 2002, CONSERV BIOL, V16, P479, DOI 10.1046/j.1523-1739.2002.00516.x
Leroy EM, 2005, NATURE, V438, P575, DOI 10.1038/438575a
Li WD, 2005, SCIENCE, V310, P676, DOI 10.1126/science.1118391
Luis AD, 2013, P ROY SOC B-BIOL SCI, V280, DOI 10.1098/rspb.2012.2753
Marlier ME, 2015, ENVIRON RES LETT, V10, DOI 10.1088/1748-9326/10/8/085005
Martins EP, 1997, AM NAT, V149, P646, DOI 10.1086/286013
Mazerolle DF, 2002, OECOLOGIA, V130, P356, DOI 10.1007/s00442-001-0818-z
Meyer CFJ, 2008, J APPL ECOL, V45, P381, DOI 10.1111/j.1365-2664.2007.01389.x
Meyer Christoph F.J., 2016, P63
Mildenstein Tammy, 2016, P325
Newbold T, 2014, P ROY SOC B-BIOL SCI, V281, DOI 10.1098/rspb.2014.1371
NORBERG UM, 1981, PHILOS T R SOC B, V292, P359, DOI 10.1098/rstb.1981.0034
Nunn CL, 2002, EVOLUTION, V56, P177
O'Brien RM, 2007, QUAL QUANT, V41, P673, DOI 10.1007/s11135-006-9018-6
Paradis E, 2004, BIOINFORMATICS, V20, P289, DOI [10.1093/bioinformatics/btg412, 10.1093/bioinformatics/bty633]
Payne J., 1985, FIELD GUIDE MAMMALS
Plowright RK, 2008, P ROY SOC B-BIOL SCI, V275, P861, DOI 10.1098/rspb.2007.1260
R Core Team, 2016, LANG ENV STAT COMP
Revell LJ, 2012, METHODS ECOL EVOL, V3, P217, DOI 10.1111/j.2041-210X.2011.00169.x
Romero LM, 2010, P ROY SOC B-BIOL SCI, V277, P3157, DOI 10.1098/rspb.2010.0678
Romero LM, 2004, TRENDS ECOL EVOL, V19, P249, DOI 10.1016/j.tree.2004.03.008
Romero LM, 2002, GEN COMP ENDOCR, V128, P1
Salvante KG, 2006, AUK, V123, P575, DOI 10.1642/0004-8038(2006)123[575:TFSIIF]2.0.CO;2
Sapolsky RM, 2000, ENDOCR REV, V21, P55, DOI 10.1210/er.21.1.55
Sazali S. N., 2011, Pertanika Journal of Tropical Agricultural Science, V34, P281
Schneeberger K, 2013, J EXP BIOL, V216, P4514, DOI 10.1242/jeb.090837
Schneeberger K, 2013, PLOS ONE, V8, DOI 10.1371/journal.pone.0054023
Stockmaier S, 2015, BIOL LETTERS, V11, DOI 10.1098/rsbl.2015.0576
Struebig MJ, 2013, ADV ECOL RES, V48, P183, DOI 10.1016/

B978-0-12-417199-2.00003-3
   Struebig MJ, 2011, ECOL LETT, V14, P582, DOI 10.1111/
j.1461-0248.2011.01623.x
   Suorsa P, 2004, P ROY SOC B-BIOL SCI, V271, P435, DOI 10.1098/
rspb.2003.2620
   Suorsa P, 2003, P ROY SOC B-BIOL SCI, V270, P963, DOI 10.1098/
rspb.2002.2326
   Tian JD, 2015, FUNCT ECOL, V29, P823, DOI 10.1111/1365-2435.12394
   Towner JS, 2009, PLOS PATHOG, V5, DOI 10.1371/
journal.ppat.1000536
   Turmelle AS, 2010, VECTOR-BORNE ZOONOT, V10, P165, DOI 10.1089/
vbz.2008.0163
   Villafuerte R, 1997, CAN J ZOOL, V75, P148, DOI 10.1139/z97-019
   Voigt Christian C., 2009, P623
   Wang LF, 2011, CURR OPIN VIROL, V1, P649, DOI 10.1016/
j.coviro.2011.10.013
   Wibbelt G, 2010, BIOL LETTERS, V6, P438, DOI 10.1098/
rsbl.2010.0267
   Wikelski M, 2006, TRENDS ECOL EVOL, V21, P38, DOI 10.1016/
j.tree.2005.10.018
   Wingfield JC, 1997, ANN NY ACAD SCI, V807, P22, DOI 10.1111/
j.1749-6632.1997.tb51911.x
   Wingfield JC, 2005, J MAMMAL, V86, P248, DOI 10.1644/BHE-004.1
   Young H, 2013, ECOL LETT, V16, P656, DOI 10.1111/ele.12094
NR 93
TC 44
Z9 54
U1 2
U2 48
PU OXFORD UNIV PRESS
PI OXFORD
PA GREAT CLARENDON ST, OXFORD OX2 6DP, ENGLAND
SN 2051-1434
J9 CONSERV PHYSIOL
JI Conserv. Physiol.
PD APR 5
PY 2017
VL 5
AR cox020
DI 10.1093/conphys/cox020
PG 14
WC Biodiversity Conservation; Ecology; Environmental Sciences;
Physiology
WE Science Citation Index Expanded (SCI-EXPANDED)
SC Biodiversity & Conservation; Environmental Sciences & Ecology;
   Physiology
GA EW6EL
UT WOS:000402601400001
PM 28421138
OA Green Published, Green Accepted, gold
DA 2024-10-21
ER

PT J

AU Lu, D
Liu, KF
Zhang, D
Yue, C
Lu, Q
Cheng, H
Wang, L
Chai, Y
Qi, JX
Wang, LF
Gao, GF
Liu, WJ
AF Lu, Dan
Liu, Kefang
Zhang, Di
Yue, Can
Lu, Qiong
Cheng, Hao
Wang, Liang
Chai, Yan
Qi, Jianxun
Wang, Lin-Fa
Gao, George F.
Liu, William J.
TI Peptide presentation by bat MHC class I provides new insight into the
antiviral immunity of bats
SO PLOS BIOLOGY
LA English
DT Article
ID T-CELL; CRYSTAL-STRUCTURE; FEATURES; VIRUS; HOST; POPULATION;
RESERVOIRS; EVOLUTION; EPITOPES; SEQUENCE
AB Bats harbor many zoonotic viruses, including highly pathogenic viruses of humans and other mammals, but they are typically asymptomatic in bats. To further understand the antiviral immunity of bats, we screened and identified a series of bat major histocompatibility complex (MHC) I Ptal-N*01:01-binding peptides derived from four different bat-borne viruses, i.e., Hendra virus (HeV), Ebola virus (EBOV), Middle East respiratory syndrome coronavirus (MERS-CoV), and H17N10 influenza-like virus. The structures of Ptal-N*01:01 display unusual peptide presentation features in that the bat-specific 3-amino acid (aa) insertion enables the tight "surface anchoring" of the P1-Asp in pocket A of bat MHC I. As the classical primary anchoring positions, the B and F pockets of Ptal-N*01:01 also show unconventional conformations, which contribute to unusual peptide motifs and distinct peptide presentation. Notably, the features of bat MHC I may be shared by MHC I from various marsupials. Our study sheds light on bat adaptive immunity and may benefit future vaccine development against bat-borne viruses of high impact on humans.
C1 [Lu, Dan; Liu, Kefang; Zhang, Di; Yue, Can; Gao, George F.; Liu, William J.] Chinese Ctr Dis Control & Prevent, Natl Inst Viral Dis Control & Prevent, NHC Key Lab Med Virol & Viral Dis, Beijing, Peoples R China.

[Liu, Kefang; Gao, George F.] Univ Macau, Fac Hlth Sci, Macau, Peoples R China.
[Yue, Can] Chinese Acad Sci, Inst Biophys, Beijing, Peoples R China.
[Lu, Qiong] Natl Inst Food & Drug Control, Div HIV AIDS & Sex Transmitted Virus Vaccines, Beijing, Peoples R China.
[Cheng, Hao; Gao, George F.] Univ Chinese Acad Sci, Beijing Inst Life Sci, Beijing, Peoples R China.
[Wang, Liang; Chai, Yan; Qi, Jianxun; Gao, George F.] Chinese Acad Sci, Inst Microbiol, CAS Key Lab Pathogen Microbiol & Immunol, Beijing, Peoples R China.
[Wang, Lin-Fa] Duke NUS Med Sch, Programme Emerging Infect Dis, Singapore, Singapore.
C3 Chinese Center for Disease Control & Prevention; National Institute for
Viral Disease Control & Prevention, Chinese Center for Disease Control &
Prevention; University of Macau; Chinese Academy of Sciences; Institute
of Biophysics, CAS; National Institute of Food & Drug Control - China;
Chinese Academy of Sciences; University of Chinese Academy of Sciences,
CAS; Chinese Academy of Sciences; Institute of Microbiology, CAS;
National University of Singapore
RP Gao, GF; Liu, WJ (corresponding author), Chinese Ctr Dis Control & Prevent, Natl Inst Viral Dis Control & Prevent, NHC Key Lab Med Virol & Viral Dis, Beijing, Peoples R China.; Gao, GF (corresponding author), Univ Macau, Fac Hlth Sci, Macau, Peoples R China.; Gao, GF (corresponding author), Univ Chinese Acad Sci, Beijing Inst Life Sci, Beijing, Peoples R China.; Gao, GF (corresponding author), Chinese Acad Sci, Inst Microbiol, CAS Key Lab Pathogen Microbiol & Immunol, Beijing, Peoples R China.
EM gaofu@chinacdc.cn; liujun@ivdc.chinacdc.cn
RI Gao, George/ABD-5229-2021; Liu, Kefang/GHK-4834-2022; Qi,
Jianxun/ABF-1646-2021; Li, Shiyue/KFA-3709-2024
OI Wang, Lin-Fa/0000-0003-2752-0535
FU Natural Science Foundation of China (NSFC) [81971501]; National Key
Research and Development Program of China [2017YFC1200202]; State's Key
Project of Research and Development Plan, International Cooperation in
Science and Technology Innovation [2016YFE0205800]; Major Special
Projects for Infectious Disease Research of China [2016ZX10004222-003];
Excellent Young Scientist Program of the NSFC [81822040]; NSFC
[81621091]; Singapore National Research Foundation Grants
[NRF2012NRF-CRP001-056, NRF2016NRF-NSFC002-013]
FX This work was supported by grants from Natural Science Foundation of
China (NSFC), http://www.nsfc.gov.cn/(grant number 81971501), the
National Key Research and Development Program of China,
http://program.most.gov.cn/#(grant number 2017YFC1200202),

State's Key
Project of Research and Development Plan, International
Cooperation in
Science and Technology Innovation, http://program.most.gov.cn/
#(grant
number 2016YFE0205800), and the Major Special Projects for
Infectious
Disease Research of China, http://www.nmp.gov.cn/(grant number
2016ZX10004222-003). WJL is supported by the Excellent Young
Scientist
Program of the NSFC, http://www.nsfc.gov.cn/(grant number
81822040). GFG
is a leading principal investigator of the NSFC Innovative
Research
Group, http://www.nsfc.gov.cn/(grant number 81621091). L-FW is
funded in
part by the Singapore National Research Foundation Grants,
https://www.nrf.gov.sg/(grant numbers NRF2012NRF-CRP001-056 and
NRF2016NRF-NSFC002-013). The funders had no role in the study
design,
data collection and analysis, decision to publish, or preparation
of the
manuscript.
CR Achour A, 1998, IMMUNITY, V9, P199, DOI 10.1016/
S1074-7613(00)80602-0
Balendiran GK, 1997, P NATL ACAD SCI USA, V94, P6880, DOI
10.1073/pnas.94.13.6880
Bean AGD, 2013, NAT REV IMMUNOL, V13, P851, DOI 10.1038/nri3551
Brunger AT, 1998, ACTA CRYSTALLOGR D, V54, P905, DOI 10.1107/
S0907444998003254
Chu FL, 2007, J IMMUNOL, V178, P944, DOI 10.4049/
jimmunol.178.2.944
Emsley P, 2010, ACTA CRYSTALLOGR D, V66, P486, DOI 10.1107/
S0907444910007493
Gao GF, 2018, CELL, V172, P1157, DOI 10.1016/j.cell.2018.02.025
GARRETT TPJ, 1989, NATURE, V342, P692, DOI 10.1038/342692a0
Gouet P, 2003, NUCLEIC ACIDS RES, V31, P3320, DOI 10.1093/nar/
gkg556
Hoof I, 2009, IMMUNOGENETICS, V61, P1, DOI 10.1007/
s00251-008-0341-z
Jiang JS, 2017, SCIENCE, V358, P1064, DOI 10.1126/science.aao5154
Lau SKP, 2005, P NATL ACAD SCI USA, V102, P14040, DOI 10.1073/
pnas.0506735102
Leroy EM, 2005, NATURE, V438, P575, DOI 10.1038/438575a
Li HT, 2005, J IMMUNOL, V174, P195, DOI 10.4049/
jimmunol.174.1.195
Li WD, 2005, SCIENCE, V310, P676, DOI 10.1126/science.1118391
Liang RY, 2018, J VIROL, V92, DOI 10.1128/JVI.01631-17
Liu J, 2010, J VIROL, V84, P11849, DOI 10.1128/JVI.01464-10
Liu J, 2010, J INFECT DIS, V202, P1171, DOI 10.1086/656315
Liu WJ, 2019, BIOSAF HEALTH, V1, P14, DOI 10.1016/
j.bsheal.2019.02.001
Liu WJ, 2017, SCI CHINA LIFE SCI, V60, P1295, DOI 10.1007/
s11427-017-9252-y

Liu WJ, 2017, J IMMUNOL, V198, P873, DOI 10.4049/jimmunol.1601542
Liu WJ, 2016, J INFECT DIS, V214, P1937, DOI 10.1093/infdis/jiw471
Mage MG, 2012, J IMMUNOL, V189, P1391, DOI 10.4049/jimmunol.1200831
Mandl JN, 2018, FRONT IMMUNOL, V9, DOI 10.3389/fimmu.2018.02112
McShan AC, 2018, NAT CHEM BIOL, V14, P811, DOI 10.1038/s41589-018-0096-2
Mohammed F, 2008, NAT IMMUNOL, V9, P1236, DOI 10.1038/ni.1660
Ng JHJ, 2016, SCI REP-UK, V6, DOI 10.1038/srep21256
Obameso JO, 2017, SCI CHINA LIFE SCI, V60, P1357, DOI 10.1007/s11427-017-9263-6
Olival KJ, 2017, NATURE, V546, P646, DOI 10.1038/nature22975
Papenfuss AT, 2012, BMC GENOMICS, V13, DOI 10.1186/1471-2164-13-261
Papenfuss AT, 2015, BMC GENOMICS, V16, DOI 10.1186/s12864-015-1745-4
Pavlovich SS, 2018, CELL, V173, P1098, DOI 10.1016/j.cell.2018.03.070
Raveh B, 2011, PLOS ONE, V6, DOI 10.1371/journal.pone.0018934
Salloum S, 2008, J VIROL, V82, P11803, DOI 10.1128/JVI.00997-08
SAPER MA, 1991, J MOL BIOL, V219, P277, DOI 10.1016/0022-2836(91)90567-P
Sarkis S, 2018, DEV COMP IMMUNOL, V85, P95, DOI 10.1016/j.dci.2018.04.006
SELVEY LA, 1995, MED J AUSTRALIA, V162, P642, DOI 10.5694/j.1326-5377.1995.tb126050.x
Shimbara N, 1998, J BIOL CHEM, V273, P23062, DOI 10.1074/jbc.273.36.23062
Smith I, 2013, CURR OPIN VIROL, V3, P84, DOI 10.1016/j.coviro.2012.11.006
Smith KJ, 1996, IMMUNITY, V4, P215, DOI 10.1016/S1074-7613(00)80430-6
Smith KJ, 1996, IMMUNITY, V4, P203, DOI 10.1016/S1074-7613(00)80429-X
Thompson JD, 1997, NUCLEIC ACIDS RES, V25, P4876, DOI 10.1093/nar/25.24.4876
Tobita T, 2003, IMMUNOL LETT, V85, P47, DOI 10.1016/S0165-2478(02)00206-7
TOKUNAGA F, 1994, J BIOL CHEM, V269, P17382
Tong SX, 2012, P NATL ACAD SCI USA, V109, P4269, DOI 10.1073/pnas.1116200109
Toor JS, 2018, FRONT IMMUNOL, V9, DOI 10.3389/fimmu.2018.00099
Turner SJ, 2005, NAT IMMUNOL, V6, P382, DOI 10.1038/ni1175
Wu Y, 2014, TRENDS MICROBIOL, V22, P183, DOI 10.1016/j.tim.2014.01.010
Wynne JW, 2016, J IMMUNOL, V196, P4468, DOI 10.4049/jimmunol.1502062
Xiao J, 2018, J IMMUNOL, V201, P3084, DOI 10.4049/jimmunol.1800611
Xiao J, 2016, J IMMUNOL, V197, P2306, DOI 10.4049/jimmunol.1600887
Xie JZ, 2018, CELL HOST MICROBE, V23, P297, DOI 10.1016/j.chom.2018.01.006

Zhang GJ, 2013, SCIENCE, V339, P456, DOI 10.1126/science.1230835
Zhang SH, 2011, MOL IMMUNOL, V49, P395, DOI 10.1016/j.molimm.2011.08.015
Zhao M, 2017, SCI CHINA LIFE SCI, V60, P1307, DOI 10.1007/s11427-017-9241-3
Zhou P, 2018, NATURE, V556, P255, DOI 10.1038/s41586-018-0010-9
Zhou P, 2016, P NATL ACAD SCI USA, V113, P2696, DOI 10.1073/pnas.1518240113
Zhou P, 2011, J IMMUNOL, V186, P3138, DOI 10.4049/jimmunol.1003115
NR 58
TC 23
Z9 25
U1 0
U2 28
PU PUBLIC LIBRARY SCIENCE
PI SAN FRANCISCO
PA 1160 BATTERY STREET, STE 100, SAN FRANCISCO, CA 94111 USA
SN 1544-9173
EI 1545-7885
J9 PLOS BIOL
JI PLoS. Biol.
PD SEP
PY 2019
VL 17
IS 9
AR e3000436
DI 10.1371/journal.pbio.3000436
PG 24
WC Biochemistry & Molecular Biology; Biology
WE Science Citation Index Expanded (SCI-EXPANDED)
SC Biochemistry & Molecular Biology; Life Sciences & Biomedicine - Other
Topics
GA JM8PM
UT WOS:000496470000005
PM 31498797
OA Green Published, Green Submitted, gold
DA 2024-10-21
ER

PT J
AU Lee, WT
Jones, DD
Yates, JL
Winslow, GM
Davis, AD
Rudd, RJ
Barron, CT
Cowan, C
AF Lee, William T.
Jones, Derek D.
Yates, Jennifer L.
Winslow, Gary M.

Davis, April D.
Rudd, Robert J.
Barron, Christopher T.
Cowan, Cailyn
TI Identification of secreted and membrane-bound bat immunoglobulin using a
Microchiropteran-specific mouse monoclonal antibody
SO DEVELOPMENTAL AND COMPARATIVE IMMUNOLOGY
LA English
DT Article
DE Bats; Microchiroptera; Monoclonal antibody; Immunoglobulins; B lymphocytes
ID WHITE-NOSE SYNDROME; LYMPH-NODE CELLS; PTEROPUS-GIGANTEUS; GENE REPERTOIRE; LIGHT-CHAINS; PROTEIN-L; VIRUSES; EVOLUTION; RESPONSES; RESERVOIR
AB Bat immunity has received increasing attention because some bat species are being decimated by the fungal disease, White Nose Syndrome, while other species are potential reservoirs of zoonotic viruses. Identifying specific immune processes requires new specific tools and reagents. In this study, we describe a new mouse monoclonal antibody (mAb) reactive with Eptesicus fuscus immunoglobulins. The epitope recognized by mAb BT1-4F10 was localized to immunoglobulin light (lambda) chains; hence, the mAb recognized serum immunoglobulins and B lymphocytes. The BT1-4F10 epitope appeared to be restricted to Microchiropteran immunoglobulins and absent from Megachiropteran immunoglobulins. Analyses of sera and other E. fuscus fluids showed that most, if not all, secreted immunoglobulins utilized lambda light chains. Finally, mAb BT1-4F10 permitted the identification of B cell follicles in splenic white pulp. This Microchiropteran-specific mAb has potential utility in seroassays; hence, this reagent may have both basic and practical applications for studying immune process. (C) 2016 Elsevier Ltd. All rights reserved.
C1 [Lee, William T.; Jones, Derek D.; Yates, Jennifer L.; Winslow, Gary M.; Cowan, Cailyn] SUNY Albany, Sch Publ Hlth, Dept Biomed Sci, Albany, NY 12201 USA.
[Lee, William T.; Winslow, Gary M.; Davis, April D.; Rudd, Robert J.; Barron, Christopher T.] New York State Dept Hlth, Wadsworth Ctr, Immunol Lab, Albany, NY 12201 USA.
[Winslow, Gary M.] Upstate Med Univ, Dept Microbiol & Immunol, Syracuse, NY 13210 USA.
C3 State University of New York (SUNY) System; University at Albany, SUNY;
Wadsworth Center; State University of New York (SUNY) System; State
University of New York (SUNY) System; State University of New York
(SUNY) Upstate Medical Center
RP Lee, WT (corresponding author), Wadsworth Ctr, David Axelrod Inst, POB 22002, Albany, NY 12201 USA.
EM William.lee@health.ny.gov
RI Lee, William/KHX-0414-2024
OI Jones, Derek/0000-0001-9982-0065; Lee, William/

0000-0003-2883-0391;
Yates, Jennifer/0000-0001-8841-9401
FU Wadsworth Center-Health Research, Inc intramural award
FX The authors would like to acknowledge the Wadsworth Center core facilities that contributed to this work. In particular, we thank Dr. Jane Jolley (Immunology Core) for her assistance in BIACore analyses, Dr. Abigail Snyder-Keller (Histopathology Core) for her technical expertise and advice in immunohistochemistry analyses, Ms. Tiffany Stifter (Veterinary Sciences Core) for assistance in mouse maintenance, and Mr. Richard Cole (Advanced Light Microscopy Core) for making the confocal instrumentation available to us. We thank Dr. Qishan Lin (Proteomics Core, Center for Functional Genomics, University at Albany) for his expert assistance in mass spectrometry analysis of proteins. We would like to acknowledge the Lubee Bat Conservancy for making their bat tissue bank available to us. We also would like to thank Dr. Elizabeth Leadbetter for her helpful discussions and advice on spleen morphology during the course of this work. This work was supported in part by a Wadsworth Center-Health Research, Inc intramural award.
CR Arun SS, 1996, J VET MED A, V43, P573, DOI 10.1111/j.1439-0442.1996.tb00489.x
Baker ML, 2013, ZOONOSES PUBLIC HLTH, V60, P104, DOI 10.1111/j.1863-2378.2012.01528.x
Baker ML, 2010, IMMUNOGENETICS, V62, P173, DOI 10.1007/s00251-010-0425-4
Blatteis CM, 2003, J THERM BIOL, V28, P1, DOI 10.1016/S0306-4565(02)00034-7
Blehert DS, 2009, SCIENCE, V323, P227, DOI 10.1126/science.1163874
Bratsch S, 2011, DEV COMP IMMUNOL, V35, P421, DOI 10.1016/j.dci.2010.06.004
Butler JE, 2006, DEV COMP IMMUNOL, V30, P199, DOI 10.1016/j.dci.2005.06.025
Butler JE, 2011, DEV COMP IMMUNOL, V35, P273, DOI 10.1016/j.dci.2010.08.011
Calisher CH, 2006, CLIN MICROBIOL REV, V19, P531, DOI 10.1128/CMR.00017-06
CHAKRAVARTY AK, 1994, LYMPHOLOGY, V27, P97
Chaturvedi V, 2010, PLOS ONE, V5, DOI 10.1371/journal.pone.0010783
COICO RF, 1983, J IMMUNOL, V131, P2254
Das S, 2008, P NATL ACAD SCI USA, V105, P16647, DOI 10.1073/pnas.0808800105
DECHATEAU M, 1993, SCAND J IMMUNOL, V37, P399

Dul JL, 1996, J IMMUNOL, V157, P2969
EDELMAN GM, 1962, J EXP MED, V116, P207
Field KA, 2015, PLOS PATHOG, V11, DOI 10.1371/journal.ppat.1005168
HOOD L, 1967, COLD SPRING HARB SYM, V32, P133, DOI 10.1101/SQB.1967.032.01.021
Lorch JM, 2011, NATURE, V480, P376, DOI 10.1038/nature10590
NILSON BHK, 1992, J BIOL CHEM, V267, P2234
O'Shea TJ, 2014, EMERG INFECT DIS, V20, P741, DOI 10.3201/eid2005.130539
Omatsu T, 2003, EXP ANIM TOKYO, V52, P425, DOI 10.1538/expanim.52.425
Papenfuss AT, 2012, BMC GENOMICS, V13, DOI 10.1186/1471-2164-13-261
Pellegrini A, 2007, INT IMMUNOL, V19, P1395, DOI 10.1093/intimm/dxm107
PENG Z, 1991, J IMMUNOL METHODS, V145, P255, DOI 10.1016/0022-1759(91)90335-D
SARKAR SK, 1991, DEV COMP IMMUNOL, V15, P423, DOI 10.1016/0145-305X(91)90034-V
Schountz T, 2014, VIRUSES-BASEL, V6, P4880, DOI 10.3390/v6124880
SHAPIRO AL, 1966, NATURE, V211, P243, DOI 10.1038/211243a0
SHULMAN M, 1978, NATURE, V276, P269, DOI 10.1038/276269a0
Tsagkogeorga G, 2013, CURR BIOL, V23, P2262, DOI 10.1016/j.cub.2013.09.014
VANBRUGGEN I, 1991, EXP MOL PATHOL, V55, P119, DOI 10.1016/0014-4800(91)90047-2
Wellehan JFX, 2009, COMP IMMUNOL MICROB, V32, P379, DOI 10.1016/j.cimid.2007.11.002
Woof JM, 2011, MUCOSAL IMMUNOL, V4, P590, DOI 10.1038/mi.2011.39
YELTON DE, 1981, HYBRIDOMA, V1, P5, DOI 10.1089/hyb.1.1981.1.5
Zhou XM, 2012, SYST BIOL, V61, P150, DOI 10.1093/sysbio/syr089
NR 35
TC 5
Z9 6
U1 0
U2 28
PU ELSEVIER SCI LTD
PI OXFORD
PA THE BOULEVARD, LANGFORD LANE, KIDLINGTON, OXFORD OX5 1GB, OXON, ENGLAND
SN 0145-305X
EI 1879-0089
J9 DEV COMP IMMUNOL
JI Dev. Comp. Immunol.
PD DEC
PY 2016
VL 65
BP 114
EP 123
DI 10.1016/j.dci.2016.06.024
PG 10
WC Fisheries; Immunology; Veterinary Sciences; Zoology
WE Science Citation Index Expanded (SCI-EXPANDED)

SC Fisheries; Immunology; Veterinary Sciences; Zoology
GA DX4ZA
UT WOS:000384388400013
PM 27377583
OA Green Accepted, Green Published
DA 2024-10-21
ER

PT J
AU Pikula, J
   Brichta, J
   Seidlova, V
   Piacek, V
   Zukal, J
AF Pikula, Jiri
   Brichta, Jiri
   Seidlova, Veronika
   Piacek, Vladimir
   Zukal, Jan
TI Higher antibody titres against <i>Pseudogymnoascus destructans</
i> are
   associated with less white-nose syndrome skin lesions in
Palearctic bats
SO FRONTIERS IN IMMUNOLOGY
LA English
DT Article
DE emerging wildlife infection; adaptive antifungal immunity;
disease
   severity; indirect ELISA; antibody prevalence; Myotis bat species
ID BODY-TEMPERATURE GOVERNS; INFECTIOUS-DISEASES; MAMMALIAN
HIBERNATION;
   CAUSATIVE AGENT; RESPONSES; BIODIVERSITY; EXPRESSION; EMERGENCE;
ECOLOGY
AB Introduction: Serological tests can be used to test whether an
animal has been exposed to an infectious agent, and whether its
immune system has recognized and produced antibodies against it.
Paired samples taken several weeks apart then document an ongoing
infection and/or seroconversion.Methods: In the absence of a
commercial kit, we developed an indirect enzyme-linked immunosorbent
assay (ELISA) to detect the fungus-specific antibodies for
Pseudogymnoascus destructans, the agent of white-nose syndrome in
bats.Results and Discussion: Samples collected from European Myotis
myotis (n=35) and Asian Myotis dasycneme (n=11) in their hibernacula
at the end of the hibernation period displayed 100% seroprevalence
of antibodies against P. destructans, demonstrating a high rate of
exposure. Our results showed that the higher the titre of antibodies
against P. destructans, the lower the infection intensity,
suggesting that a degree of protection is provided by this arm of
adaptive immunity in Palearctic bats. Moreover, P. destructans
infection appears to be a seasonally self-limiting disease of
Palearctic bats showing seroconversion as the WNS skin lesions heal
in the early post-hibernation period.
C1 [Pikula, Jiri; Brichta, Jiri; Seidlova, Veronika; Piacek,
Vladimir] Univ Vet Sci Brno, Dept Ecol & Dis Zoo Anim Game Fish &

Bees, Brno, Czech Republic.
[Pikula, Jiri] Univ Vet & Pharmaceut Sci Brno, CEITEC Cent European Inst Technol, Brno, Czech Republic.
[Zukal, Jan] Czech Acad Sci, Inst Vertebrate Biol, Brno, Czech Republic.
C3 University of Veterinary Sciences Brno; University of Veterinary Sciences Brno; Czech Academy of Sciences; Institute of Vertebrate Biology of the Czech Academy of Sciences
RP Pikula, J (corresponding author), Univ Vet Sci Brno, Dept Ecol & Dis Zoo Anim Game Fish & Bees, Brno, Czech Republic.; Pikula, J (corresponding author), Univ Vet & Pharmaceut Sci Brno, CEITEC Cent European Inst Technol, Brno, Czech Republic.
EM pikulaj@vfu.cz
RI Brichta, Jiri/Q-8681-2017; Piaček, Vladimír/AAI-1231-2020; Zukal, Jan/G-1180-2014
OI Piacek, Vladimir/0000-0003-0068-3801; Zukal, Jan/0000-0003-4967-6880
FU Internal Grant Agency of the University of Veterinary Sciences [219/2022/FVHE]
FX The author(s) declare financial support was received for the research,
authorship, and/or publication of this article. This study was supported
by the Internal Grant Agency of the University of Veterinary Sciences
(No. 219/2022/FVHE).
CR Aicher SM, 2022, J VIROL, V96, DOI 10.1128/jvi.00608-22
Bandouchova H, 2015, TRANSBOUND EMERG DIS, V62, P1, DOI 10.1111/tbed.12282
Bandouchova H, 2018, SCI REP-UK, V8, DOI 10.1038/s41598-018-24461-5
Banerjee A, 2020, FRONT IMMUNOL, V11, DOI 10.3389/fimmu.2020.00026
Blanco JL, 2008, VET IMMUNOL IMMUNOP, V125, P47, DOI 10.1016/j.vetimm.2008.04.020
Blehert DS, 2012, PLOS PATHOG, V8, DOI 10.1371/journal.ppat.1002779
Blehert DS, 2009, SCIENCE, V323, P227, DOI 10.1126/science.1163874
Bouma HR, 2013, J LEUKOCYTE BIOL, V94, P431, DOI 10.1189/jlb.0611298
Bouma Hjalmar R., 2012, P259
Bouma HR, 2011, P NATL ACAD SCI USA, V108, P2052, DOI 10.1073/pnas.1008823108
Bouma HR, 2010, J LEUKOCYTE BIOL, V88, P619, DOI 10.1189/jlb.0310174
Boyer BB, 1999, BIOSCIENCE, V49, P713, DOI 10.2307/1313595
Brennan RE, 2015, SOUTHWEST NAT, V60, P145, DOI 10.1894/SWNAT-D-14-00030.1
Brook CE, 2015, TRENDS MICROBIOL, V23, P172, DOI 10.1016/j.tim.2014.12.004
Brunet-Rossinni Anja K., 2009, P315
Calisher CH, 2006, CLIN MICROBIOL REV, V19, P531, DOI 10.1128/CMR.00017-06

Carey HV, 2003, PHYSIOL REV, V83, P1153, DOI 10.1152/physrev.00008.2003
Carvalho A, 2012, FRONT MICROBIOL, V3, DOI 10.3389/fmicb.2012.00176
Casadevall A, 2012, CELL HOST MICROBE, V11, P447, DOI 10.1016/j.chom.2012.04.004
Cryan PM, 2013, J WILDLIFE DIS, V49, P398, DOI 10.7589/2012-04-121
Cryan PM, 2010, BMC BIOL, V8, DOI 10.1186/1741-7007-8-135
Daszak P, 2000, SCIENCE, V287, P443, DOI 10.1126/science.287.5452.443
Daszak P, 2001, ACTA TROP, V78, P103, DOI 10.1016/S0001-706X(00)00179-0
Davy CM, 2020, VIRULENCE, V11, P781, DOI 10.1080/21505594.2020.1768018
Davy CM, 2017, CONSERV BIOL, V31, P615, DOI 10.1111/cobi.12841
Donaldson ME, 2017, EVOL APPL, V10, P1076, DOI 10.1111/eva.12514
Field KA, 2015, PLOS PATHOG, V11, DOI 10.1371/journal.ppat.1005168
Flieger M, 2016, SCI REP-UK, V6, DOI 10.1038/srep33200
Francl KE, 2012, J FISH WILDL MANAG, V3, P33, DOI 10.3996/062011-JFWM-039
Fritze M, 2019, BIOL OPEN, V8, DOI 10.1242/bio.046078
Guivier E, 2010, INFECT GENET EVOL, V10, P1208, DOI 10.1016/j.meegid.2010.07.022
Haase CG, 2021, ECOL EVOL, V11, P506, DOI 10.1002/ece3.7070
Harazim M, 2023, BMC IMMUNOL, V24, DOI 10.1186/s12865-023-00542-7
Harrison XA, 2011, J ANIM ECOL, V80, P4, DOI 10.1111/j.1365-2656.2010.01740.x
Hecht-Höger AM, 2020, MOL ECOL, V29, P1745, DOI 10.1111/mec.15437
Heger T, 2020, ACTA VET BRNO, V89, P79, DOI 10.2754/avb202089010079
Johnson JS, 2015, ECOL EVOL, V5, P2203, DOI 10.1002/ece3.1502
Jonasson KA, 2011, PLOS ONE, V6, DOI 10.1371/journal.pone.0021061
Kacprzyk J, 2017, ACTA CHIROPTEROL, V19, P219, DOI [10.3161/15081109ACC2017.19.2.001, 10.3161/15081109acc2017.19.2.001]
Keesing F, 2010, NATURE, V468, P647, DOI 10.1038/nature09575
Kunz TH, 1998, ECOSCIENCE, V5, P8, DOI 10.1080/11956860.1998.11682443
Lockhart SR, 2019, SEMIN DIAGN PATHOL, V36, P177, DOI 10.1053/j.semdp.2019.04.010
Lorch J. M., 2016, mSphere, V1, P00148
Lorch JM, 2011, NATURE, V480, P376, DOI 10.1038/nature10590
Mandl JN, 2018, FRONT IMMUNOL, V9, DOI 10.3389/fimmu.2018.02112
Mandl JN, 2015, CELL, V160, P20, DOI 10.1016/j.cell.2014.12.003
Martínková N, 2018, VIRULENCE, V9, P1734, DOI 10.1080/21505594.2018.1548685
Meteyer CU, 2012, VIRULENCE, V3, P583, DOI 10.4161/viru.22330
Meteyer CU, 2009, J VET DIAGN INVEST, V21, P411, DOI 10.1177/104063870902100401
Nemcova M, 2022, FRONT VET SCI, V9, DOI 10.3389/fvets.2022.978756
O'Connor CM, 2015, AMBIO, V44, P582, DOI 10.1007/s13280-015-0630-3
O'Donoghue AJ, 2015, P NATL ACAD SCI USA, V112, P7478, DOI

10.1073/pnas.1507082112
Perry RW, 2013, ENVIRON REV, V21, P28, DOI 10.1139/er-2012-0042
Pikula J, 2020, BMC VET RES, V16, DOI 10.1186/s12917-020-02450-z
Pikula J, 2017, PLOS ONE, V12, DOI 10.1371/journal.pone.0180435
Pikula J, 2012, J WILDLIFE DIS, V48, P207, DOI 10.7589/0090-3558-48.1.207
Puechmaille SJ, 2011, PLOS ONE, V6, DOI 10.1371/journal.pone.0019167
Reeder SM, 2017, VIRULENCE, V8, P1695, DOI 10.1080/21505594.2017.1342910
Rocke TE, 2019, SCI REP-UK, V9, DOI 10.1038/s41598-019-43210-w
Rosales C, 2016, J IMMUNOL RES, V2016, DOI 10.1155/2016/1469780
Santamaría R, 2011, IMMUNOBIOLOGY, V216, P1212, DOI 10.1016/j.imbio.2011.08.004
Schountz T, 2017, FRONT IMMUNOL, V8, DOI 10.3389/fimmu.2017.01098
Shuey MM, 2014, APPL ENVIRON MICROB, V80, P1726, DOI 10.1128/AEM.02897-13
Speakman JR, 2008, PHILOS T R SOC B, V363, P375, DOI 10.1098/rstb.2007.2145
Tada T, 1974, Allerg Immunol (Leipz), V20-21, P427
Turner GG, 2014, J WILDLIFE DIS, V50, P566, DOI 10.7589/2014-03-058
Verant Michelle L., 2014, BMC Physiology, V14, P10, DOI 10.1186/s12899-014-0010-4
Veselská T, 2020, SCI REP-UK, V10, DOI 10.1038/s41598-020-73619-7
Voigt CC, 2020, SCI REP-UK, V10, DOI 10.1038/s41598-020-74473-3
Warnecke L, 2013, BIOL LETTERS, V9, DOI 10.1098/rsbl.2013.0177
Warnecke L, 2012, P NATL ACAD SCI USA, V109, P6999, DOI 10.1073/pnas.1200374109
Webb PI, 1996, CAN J ZOOL, V74, P761, DOI 10.1139/z96-087
Willis CKR, 2017, INTEGR COMP BIOL, V57, P1214, DOI 10.1093/icb/icx087
Wüthrich M, 2012, ANNU REV IMMUNOL, V30, P115, DOI 10.1146/annurev-immunol-020711-074958
Xu YC, 2013, BMC GENOMICS, V14, DOI 10.1186/1471-2164-14-567
Zukal J., 2017, Cave investigation, P51
Zukal J, 2016, SCI REP-UK, V6, DOI 10.1038/srep19829
Zukal J, 2014, PLOS ONE, V9, DOI 10.1371/journal.pone.0097224
NR 78
TC 1
Z9 1
U1 3
U2 10
PU FRONTIERS MEDIA SA
PI LAUSANNE
PA AVENUE DU TRIBUNAL FEDERAL 34, LAUSANNE, CH-1015, SWITZERLAND
SN 1664-3224
J9 FRONT IMMUNOL
JI Front. Immunol.
PD DEC 8
PY 2023
VL 14
AR 1269526
DI 10.3389/fimmu.2023.1269526

PG 10
WC Immunology
WE Science Citation Index Expanded (SCI-EXPANDED)
SC Immunology
GA CY4B3
UT WOS:001128768400001
PM 38143741
OA Green Published, gold
DA 2024-10-21
ER

PT J
AU Nikaido, M
   Kondo, S
   Zhang, ZC
   Wu, JQ
   Nishihara, H
   Niimura, Y
   Suzuki, S
   Touhara, K
   Suzuki, Y
   Noguchi, H
   Minakuchi, Y
   Toyoda, A
   Fujiyama, A
   Sugano, S
   Yoneda, M
   Kai, CK
AF Nikaido, Masato
   Kondo, Shinji
   Zhang, Zicong
   Wu, Jiaqi
   Nishihara, Hidenori
   Niimura, Yoshihito
   Suzuki, Shunta
   Touhara, Kazushige
   Suzuki, Yutaka
   Noguchi, Hideki
   Minakuchi, Yohei
   Toyoda, Atsushi
   Fujiyama, Asao
   Sugano, Sumio
   Yoneda, Misako
   Kai, Chieko
TI Comparative genomic analyses illuminate the distinct evolution of
   megabats within Chiroptera
SO DNA RESEARCH
LA English
DT Article
DE megabat; whole genome; adaptive evolution; SINEs; chemosensory
receptor
   genes
ID PUTATIVE PHEROMONE RECEPTORS; PHYLOGENETIC ANALYSIS;
MITOCHONDRIAL

GENOME; MAXIMUM-LIKELIHOOD; GENE REPERTOIRES; RNASE-L;
VOMERONASAL;
ALIGNMENT; BATS; SEQUENCE
AB The revision of the sub-order Microchiroptera is one of the most intriguing outcomes in recent mammalian molecular phylogeny. The unexpected sister-taxon relationship between rhinolophoid microbats and megabats, with the exclusion of other microbats, suggests that megabats arose in a relatively short period of time from a microbat-like ancestor. In order to understand the genetic mechanism underlying adaptive evolution in megabats, we determined the whole-genome sequences of two rousette megabats, Leschenault's rousette (Rousettus leschenaultia) and the Egyptian fruit bat (R. aegyptiacus). The sequences were compared with those of 22 other mammals, including nine bats, available in the database. We identified that megabat genomes are distinct in that they have extremely low activity of SINE retrotranspositions, expansion of two chemosensory gene families, including the trace amine receptor (TAAR) and olfactory receptor (OR), and elevation of the dN/dS ratio in genes for immunity and protein catabolism. The adaptive signatures discovered in the genomes of megabats may provide crucial insight into their distinct evolution, including key processes such as virus resistance, loss of echolocation, and frugivorous feeding.
C1 [Nikaido, Masato; Wu, Jiaqi] Tokyo Inst Technol, Sch Life Sci & Technol, Meguro Ku, Tokyo 1528550, Japan.
[Kondo, Shinji; Noguchi, Hideki; Toyoda, Atsushi; Fujiyama, Asao] Natl Inst Genet, Adv Genom Ctr, Mishima, Shizuoka 4118540, Japan.
[Kondo, Shinji; Noguchi, Hideki] Res Org Informat & Syst, Joint Support Ctr Data Sci Res, Mishima, Shizuoka 4118540, Japan.
[Zhang, Zicong] Tokyo Inst Technol, Dept Computat Intelligence & Syst Sci, Yokohama, Kanagawa 2268502, Japan.
[Nishihara, Hidenori] Sch Life Sci & Technol, Tokyo Inst Technol, Midori Ku, 4259 Nagatsuta Cho, Yokohama, Kanagawa 2268501, Japan.
[Niimura, Yoshihito; Suzuki, Shunta; Touhara, Kazushige] Univ Tokyo, Grad Sch Agr & Life Sci, Dept Appl Biol Chem, Tokyo 1138657, Japan.
[Suzuki, Yutaka; Sugano, Sumio] Univ Tokyo, Grad Sch Frontier Sci, Dept Med Genome Sci, Kashiwa City, Chiba 2770882, Japan.
[Minakuchi, Yohei; Toyoda, Atsushi] Natl Inst Genet, Comparat Genom Lab, Mishima, Shizuoka 4118540, Japan.
[Yoneda, Misako] Univ Tokyo, Inst Med Sci, Minato Ku, Tokyo 1088639, Japan.
[Yoneda, Misako; Kai, Chieko] Univ Tokyo, Inst Ind Sci, Meguro Ku, Tokyo 1538505, Japan.
C3 Institute of Science Tokyo; Tokyo Institute of Technology;
Research
Organization of Information & Systems (ROIS); National Institute
of
Genetics (NIG) - Japan; Research Organization of Information &
Systems
(ROIS); Institute of Science Tokyo; Tokyo Institute of
Technology;
Institute of Science Tokyo; Tokyo Institute of Technology;
University of
Tokyo; University of Tokyo; Research Organization of Information

&
   Systems (ROIS); National Institute of Genetics (NIG) - Japan; University
   of Tokyo; University of Tokyo
RP Kai, CK (corresponding author), Univ Tokyo, Inst Ind Sci, Meguro Ku, Tokyo 1538505, Japan.
EM ckai@iis.u-tokyo.ac.jp
RI ....., .../HJI-0218-2023; Nikaido, Masato/B-1698-2015; Jiaqi,
   Wu/G-2209-2019; Nishihara, Hidenori/B-9324-2015
OI Nishihara, Hidenori/0000-0002-5843-9994; Zhang,
   Zicong/0000-0003-1444-5054
FU JSPS KAKENHI [22228005, 16H02587, 221S0002, 16H06279, 15H04591,
   20H03307]; JST ERATO Touhara Chemosensory Signal Project [JPMJER1202];
   Asahi Glass Foundation; Grants-in-Aid for Scientific Research [16H02587,
   15H04591, 20H03307, 22228005] Funding Source: KAKEN
FX This research was supported by JSPS KAKENHI to CK (22228005, 16H02587,
   221S0002, 16H06279), to MY (15H04591), to MN (20H03307), and JST ERATO
   Touhara Chemosensory Signal Project (JPMJER1202) to KT, and the Asahi
   Glass Foundation to MN.
CR Altringham J., 1996, Bats: Biology and Behaviour
   ALTSCHUL SF, 1990, J MOL BIOL, V215, P403, DOI 10.1016/S0022-2836(05)80360-2
   Aronesty E., 2011, Ea-utils: command-line tools for processing biological sequencing data'
   Bao WD, 2015, MOBILE DNA-UK, V6, DOI 10.1186/s13100-015-0041-9
   Bhatnagar KP, 1998, MICROSC RES TECHNIQ, V43, P465, DOI 10.1002/(SICI)1097-0029(19981215)43:6<465::AID-JEMT1>3.0.CO;2-1
   Burge C, 1997, J MOL BIOL, V268, P78, DOI 10.1006/jmbi.1997.0951
   Cantrell MA, 2008, GENETICS, V178, P393, DOI 10.1534/genetics.107.080275
   Chamero P, 2012, TRENDS NEUROSCI, V35, P597, DOI 10.1016/j.tins.2012.04.011
   Choo SW, 2016, GENOME RES, V26, P1312, DOI 10.1101/gr.203521.115
   Degnan JH, 2009, TRENDS ECOL EVOL, V24, P332, DOI 10.1016/j.tree.2009.01.009
   DeLano W.L., 2002, The PyMOL Molecular Graphics System
   Dewannieux M, 2003, NAT GENET, V35, P41, DOI 10.1038/ng1223
   Eyun SI, 2016, PLOS ONE, V11, DOI 10.1371/journal.pone.0151023
   Gogolevsky KP, 2009, GENOMICS, V93, P494, DOI 10.1016/j.ygeno.2009.02.001
   Goodier JL, 2016, MOBILE DNA-UK, V7, DOI 10.1186/s13100-016-0070-z
   Grus WE, 2005, P NATL ACAD SCI USA, V102, P5767, DOI 10.1073/pnas.0501589102
   Haga S, 2010, NATURE, V466, P118, DOI 10.1038/nature09142
   Hallström BM, 2011, PLOS ONE, V6, DOI 10.1371/journal.pone.0028199
   Hayakawa T, 2014, MOL BIOL EVOL, V31, P2018, DOI 10.1093/molbev/msu144

Hayden S, 2014, MOL BIOL EVOL, V31, P917, DOI 10.1093/molbev/msu043
Herrada G, 1997, CELL, V90, P763, DOI 10.1016/S0092-8674(00)80536-X
Hohenbrink P, 2013, BIOL LETTERS, V9, DOI 10.1098/rsbl.2012.1006
Isogai Y, 2011, NATURE, V478, P241, DOI 10.1038/nature10437
Kajitani R, 2014, GENOME RES, V24, P1384, DOI 10.1101/gr.170720.113
Kapitonov VV, 2009, GENE, V448, P207, DOI 10.1016/j.gene.2009.07.019
Kapusta A, 2017, P NATL ACAD SCI USA, V114, pE1460, DOI 10.1073/pnas.1616702114
Katoh Kazutaka, 2013, Mol Biol Evol, V30, P772, DOI 10.1093/molbev/mst010
Kent WJ, 2002, GENOME RES, V12, P656, DOI [10.1101/gr.229202. Article published online before March 2002, 10.1101/gr.229202]
Kohany O, 2006, BMC BIOINFORMATICS, V7, DOI 10.1186/1471-2105-7-474
Korf I, 2004, BMC BIOINFORMATICS, V5, DOI 10.1186/1471-2105-5-59
Lam TTY, 2020, NATURE, V583, P282, DOI 10.1038/s41586-020-2169-0
Li H, 2009, BIOINFORMATICS, V25, P1094, DOI [10.1093/bioinformatics/btp100, 10.1093/bioinformatics/btp324]
Li WZ, 2006, BIOINFORMATICS, V22, P1658, DOI 10.1093/bioinformatics/btl158
Liberles SD, 2006, NATURE, V442, P645, DOI 10.1038/nature05066
Liberles SD, 2015, CURR OPIN NEUROBIOL, V34, P1, DOI 10.1016/j.conb.2015.01.001
Lindemann L, 2005, TRENDS PHARMACOL SCI, V26, P274, DOI 10.1016/j.tips.2005.03.007
Löytynoja A, 2014, METHODS MOL BIOL, V1079, P155, DOI 10.1007/978-1-62703-646-7_10
Luo RB, 2012, GIGASCIENCE, V1, DOI 10.1186/2047-217X-1-18
Madsen O, 2001, NATURE, V409, P610, DOI 10.1038/35054544
Matsunami H, 1997, CELL, V90, P775, DOI 10.1016/S0092-8674(00)80537-1
Moriya-Ito K, 2018, GENE, V642, P343, DOI 10.1016/j.gene.2017.11.048
Muto Y, 2015, J MOL EVOL, V80, P130, DOI 10.1007/s00239-015-9666-z
Nei M, 2008, NAT REV GENET, V9, P951, DOI 10.1038/nrg2480
Niimura Y, 2018, MOL BIOL EVOL, V35, P1437, DOI 10.1093/molbev/msy042
Niimura Yoshihito, 2013, Methods Mol Biol, V1003, P39, DOI 10.1007/978-1-62703-377-0_3
Nikaido M, 2001, J MOL EVOL, V53, P508, DOI 10.1007/s002390010241
Nikaido M, 2000, J MOL EVOL, V51, P318, DOI 10.1007/s002390010094
Nikaido M, 2019, GENES GENET SYST, V94, P141, DOI 10.1266/ggs.19-00009
Nishihara H, 2006, P NATL ACAD SCI USA, V103, P9929, DOI 10.1073/pnas.0603797103
Nishihara H, 2019, GENES GENET SYST, V94, P269, DOI 10.1266/ggs.19-00029
NOVACEK MJ, 1992, NATURE, V356, P121, DOI 10.1038/356121a0
Organ CL, 2007, NATURE, V446, P180, DOI 10.1038/nature05621

Organ CL, 2009, BIOL LETTERS, V5, P47, DOI 10.1098/rsbl.2008.0491
Osakada T, 2018, NAT COMMUN, V9, DOI 10.1038/s41467-018-07003-5
Parker J, 2013, NATURE, V502, P228, DOI 10.1038/nature12511
Pavlovich SS, 2018, CELL, V173, P1098, DOI 10.1016/j.cell.2018.03.070
Pettigrew JD, 2008, NEUROSCIENCE, V153, P226, DOI 10.1016/j.neuroscience.2008.02.019
Platt RN, 2016, MOBILE DNA-UK, V7, DOI 10.1186/s13100-016-0071-y
Pumo DE, 1998, J MOL EVOL, V47, P709, DOI 10.1007/PL00006430
Rivière S, 2009, NATURE, V459, P574, DOI 10.1038/nature08029
Santos PSC, 2016, SCI REP-UK, V6, DOI 10.1038/srep38490
Seim I, 2013, NAT COMMUN, V4, DOI 10.1038/ncomms3212
Shen B, 2012, PLOS ONE, V7, DOI 10.1371/journal.pone.0033197
Shi P, 2007, GENOME RES, V17, P166, DOI 10.1101/gr.6040007
Silverman RH, 2007, J VIROL, V81, P12720, DOI 10.1128/JVI.01471-07
Simao FA, 2015, BIOINFORMATICS, V31, P3210, DOI 10.1093/bioinformatics/btv351
Simmons Nancy B., 1998, Bulletin of the American Museum of Natural History, V235, P1
Slater GS, 2005, BMC BIOINFORMATICS, V6, DOI 10.1186/1471-2105-6-31
Springer MS, 2001, P NATL ACAD SCI USA, V98, P6241, DOI 10.1073/pnas.111551998
Stamatakis A, 2014, BIOINFORMATICS, V30, P1312, DOI 10.1093/bioinformatics/btu033
Stanke M, 2008, BIOINFORMATICS, V24, P637, DOI 10.1093/bioinformatics/btn013
Suzuki H, 2018, MOL BIOL EVOL, V35, P2928, DOI 10.1093/molbev/msy186
Suzuki S, 2015, BIOINFORMATICS, V31, P1183, DOI 10.1093/bioinformatics/btu780
Szczesniak M, 2014, MITOCHONDR DNA, V25, P443, DOI 10.3109/19401736.2013.809451
Tamura K, 2011, MOL BIOL EVOL, V28, P2731, DOI 10.1093/molbev/msr121
Teeling EC, 2000, NATURE, V403, P188, DOI 10.1038/35003188
Teeling EC, 2005, SCIENCE, V307, P580, DOI 10.1126/science.1105113
Trapnell C, 2010, NAT BIOTECHNOL, V28, P511, DOI 10.1038/nbt.1621
Trapnell C, 2009, BIOINFORMATICS, V25, P1105, DOI 10.1093/bioinformatics/btp120
Wang J, 2017, NUCLEIC ACIDS RES, V45, pW130, DOI 10.1093/nar/gkx356
Wang Z., 2018, NAT ECOL EVOL, V1, pe21
Wu JQ, 2017, CURR BIOL, V27, P3025, DOI 10.1016/j.cub.2017.08.043
Xiao KP, 2020, NATURE, V583, P286, DOI 10.1038/s41586-020-2313-x
Yang L, 2014, PLOS GENET, V10, DOI 10.1371/journal.pgen.1004395
Yang ZH, 2007, MOL BIOL EVOL, V24, P1586, DOI 10.1093/molbev/msm088
Yohe LR, 2019, GENOME BIOL EVOL, V11, P2741, DOI 10.1093/gbe/evz179
Yohe LR, 2017, EVOLUTION, V71, P923, DOI 10.1111/evo.13187
Young JM, 2010, GENOME RES, V20, P10, DOI 10.1101/gr.098913.109

   Zhang A, 2014, NUCLEIC ACIDS RES, V42, P3803, DOI 10.1093/nar/
gkt1308
   Zhang C, 2018, BMC BIOINFORMATICS, V19, DOI 10.1186/
s12859-018-2129-y
   Zhang GJ, 2013, SCIENCE, V339, P456, DOI 10.1126/science.1230835
   Zhang ZC, 2020, GENOME BIOL EVOL, V12, P766, DOI 10.1093/gbe/
evaa082
   Zhao HB, 2012, GENOME BIOL EVOL, V4, P73, DOI 10.1093/gbe/evr126
   Zhao HB, 2011, MOL BIOL EVOL, V28, P7, DOI 10.1093/molbev/msq207
   Zhao HB, 2010, MOL BIOL EVOL, V27, P2642, DOI 10.1093/molbev/
msq152
   Zhou P., 2020, NATURE, V579, P270, DOI DOI 10.1038/
s41586-020-2012-7
   Zhou XM, 2012, SYST BIOL, V61, P150, DOI 10.1093/sysbio/syr089
NR 97
TC 11
Z9 12
U1 0
U2 2
PU OXFORD UNIV PRESS
PI OXFORD
PA GREAT CLARENDON ST, OXFORD OX2 6DP, ENGLAND
SN 1340-2838
EI 1756-1663
J9 DNA RES
JI DNA Res.
PD AUG
PY 2020
VL 27
IS 4
AR dsaa021
DI 10.1093/dnares/dsaa021
PG 12
WC Genetics & Heredity
WE Science Citation Index Expanded (SCI-EXPANDED)
SC Genetics & Heredity
GA PS2TI
UT WOS:000607779900003
PM 32966557
OA Green Published, gold
DA 2024-10-21
ER

PT J
AU Viola, MF
   Herrera, MLG
   da Cruz-Neto, AP
AF Viola, Matheus F.
   Herrera, L. Gerardo M.
   da Cruz-Neto, Ariovaldo P.
TI The acute phase response in bats (<i>Carollia perspicillata</i>) varies
   with time and dose of the immune challenge
SO JOURNAL OF EXPERIMENTAL BIOLOGY

LA English
DT Article
DE Chiroptera; Lipopolysaccharide; Innate immune response; Activity period;
   Resting period
ID TOLL-LIKE RECEPTORS; SICKNESS BEHAVIOR; BODY-TEMPERATURE;
   ENDOTOXIN-LIPOPOLYSACCHARIDE; THERMOREGULATORY RESPONSES; INFLAMMATORY
   RESPONSE; DIURNAL-VARIATIONS; HOUSE SPARROWS; NITRIC-OXIDE; TRADE-OFFS
AB The acute phase response (APR) is a core component of the innate immune response and represents the first line of immune defense used in response to infections. Although several studies with vertebrates reported fever, a decrease in food intake and body mass, and an increase in neutrophil/lymphocyte ratio and total white blood cell count after lipopolysaccharide (LPS) inoculation, there was great variability in the magnitude of these responses. Some of these differences might reflect, to some extent, differences in the time of endotoxin inoculation (during active or rest periods) and dose. Therefore, our study tested the interplay between LPS dose and time of injection on selected physiological (fever and increase in total white blood cell count and neutrophil/lymphocyte ratio) and behavioral (food intake) components of the APR using a Neotropical fruit-eating bat (Carollia perspicillata) as a model organism. We predicted that LPS would trigger a dose- and time-dependent response in APR components. APR components were assessed in rest and active periods after injection of three doses of LPS (5, 10 and 15 mg kg(-1) LPS). The results indicate a more robust decrease in food intake at higher doses during the active period, while increased neutrophil/lymphocyte ratio was more robust during the active period regardless of dose. Furthermore, the skin temperature increase lasted longer at higher doses regardless of the timing of injections. Our study offers important insights into the dependence of time as well as the LPS dosage effect in the APR of bats, and how they deal with the magnitude of infections at different times of day.
C1 [Viola, Matheus F.; da Cruz-Neto, Ariovaldo P.] Univ Estadual Paulista, Inst Biociencias, Dept Biodiversidade, Lab Fisiol Anim LaFA, BR-13506900 Rio Claro, SP, Brazil.
   [Herrera, L. Gerardo M.] Univ Nacl Autonoma Mexico, Inst Biol, Estn Biol Chamela, San Patricio 48980, Jalisco, Mexico.
C3 Universidade Estadual Paulista; Universidad Nacional Autonoma de Mexico
RP Viola, MF (corresponding author), Univ Estadual Paulista, Inst Biociencias, Dept Biodiversidade, Lab Fisiol Anim LaFA, BR-13506900 Rio Claro, SP, Brazil.
EM matheus.viola@unesp.br
OI Fernandes Viola, Matheus/0000-0003-1889-7584
FU Fundacao de Amparo a Pesquisa do Estado de Sao Paulo (FAPESP)
   [2017-17607-6, 2014/16320-7]; PASPA-DGAPA program of the Universidad
   Nacional Autonoma de Mexico [814-2018]; Coordenacao de Aperfeicoamento
   de Pessoal de Nivel Superior Tecnologico (CAPES)

[88882.434214/2019-01]
FX This study was supported by a grant to A.P.C.N. and L.G.H.M. from
Fundacao de Amparo a Pesquisa do Estado de Sao Paulo (FAPESP Visiting
Research Program 2017-17607-6). A.P.C.N. was also supported by a grant
from Fundaca~o de Amparo a Pesquisa do Estado de Sao Paulo (FAPESP,
2014/16320-7) and L.G.H.M. was supported by a grant from the PASPA-DGAPA
program of the Universidad Nacional Autonoma de Mexico (814-2018).
M.F.V. was supported by a grant from the Coordenacao de Aperfeicoamento
de Pessoal de Nivel Superior Tecnologico (CAPES, 88882.434214/2019-01).
CR Acevedo-Whitehouse K, 2009, PHILOS T R SOC B, V364, P3429, DOI 10.1098/rstb.2009.0128
Adelman JS, 2010, J EXP BIOL, V213, P4099, DOI 10.1242/jeb.049528
Akira S., 2016, JANEWAYS IMMUNOBIOLO, P77
[Anonymous], MAMM BIOL, V82, P41, DOI [10.1016/j.mambio.2016.11.002, DOI 10.1016/J.MAMBIO.2016.11.002]
Armour EM, 2020, J EXP BIOL, V223, DOI 10.1242/jeb.220095
Banerjee A, 2017, SCI REP-UK, V7, DOI 10.1038/s41598-017-01513-w
Bicego KC, 2002, J APPL PHYSIOL, V93, P512, DOI 10.1152/japplphysiol.00121.2002
Bornstein SR, 2006, ANN NY ACAD SCI, V1088, P307, DOI 10.1196/annals.1366.027
Bowers EK, 2017, J EXP ZOOL PART A, V327, P302, DOI 10.1002/jez.2090
Burness G, 2010, J EXP BIOL, V213, P1386, DOI 10.1242/jeb.027011
Butler MW, 2021, J EXP BIOL, V224, DOI 10.1242/jeb.243116
Cabrera-Martinez LV, 2019, COMP BIOCHEM PHYS A, V229, P93, DOI 10.1016/j.cbpa.2018.12.003
Cabrera-Martínez LV, 2018, PEERJ, V6, DOI 10.7717/peerj.4627
Cain DW, 2017, NAT REV IMMUNOL, V17, P233, DOI 10.1038/nri.2017.1
Coon CAC, 2011, AM J PHYSIOL-REG I, V300, pR1418, DOI 10.1152/ajpregu.00187.2010
Copeland S, 2005, CLIN DIAGN LAB IMMUN, V12, P60, DOI 10.1128/CDLI.12.1.60-67.2005
Cray C, 2012, PROG MOL BIOL TRANSL, V105, P113, DOI 10.1016/B978-0-12-394596-9.00005-6
Cray C, 2009, COMPARATIVE MED, V59, P517
Cruz-Neto A.P., 2006, FUNCTIONAL EVOLUTION, P56
Cyrino JC, 2022, INTEGR COMP BIOL, V62, P1606, DOI 10.1093/icb/icac028
Dantonio V, 2016, AM J PHYSIOL-REG I, V310, pR896, DOI 10.1152/ajpregu.00453.2015
Davis AK, 2008, FUNCT ECOL, V22, P760, DOI 10.1111/j.1365-2435.2008.01467.x
De Boever S, 2009, AVIAN PATHOL, V38, P403, DOI 10.1080/03079450903190871
Demas GE, 2011, J ANIM ECOL, V80, P710, DOI 10.1111/j.1365-2656.2011.01813.x

Dhabhar FS, 2002, BRAIN BEHAV IMMUN, V16, P785, DOI 10.1016/S0889-1591(02)00036-3
do Amaral JPS, 2002, PHYSIOL BIOCHEM ZOOL, V75, P273, DOI 10.1086/341816
do Amaral-Silva L, 2021, J PHYSIOL-LONDON, V599, P2969, DOI 10.1113/JP281385
Escalera-Zamudio M, 2015, MOL ECOL, V24, P5899, DOI 10.1111/mec.13431
Fedele MLM, 2020, FRONT CELL INFECT MI, V10, DOI 10.3389/fcimb.2020.00100
Fornusková A, 2013, BMC EVOL BIOL, V13, DOI 10.1186/1471-2148-13-194
Ganeshan K, 2019, CELL, V177, P399, DOI 10.1016/j.cell.2019.01.050
Gayle D, 1998, BRAIN RES, V795, P77, DOI 10.1016/S0006-8993(98)00280-7
Gong S, 2015, PLOS ONE, V10, DOI 10.1371/journal.pone.0117503
Grion N, 2007, J ENDOCRINOL, V194, P11, DOI 10.1677/JOE-06-0199
Guerrero-Chacón AL, 2018, MAMMAL RES, V63, P397, DOI 10.1007/s13364-018-0375-z
Haba R, 2012, BEHAV BRAIN RES, V228, P423, DOI 10.1016/j.bbr.2011.12.027
Hasselquist D, 2012, ANIM BEHAV, V83, P1303, DOI 10.1016/j.anbehav.2012.03.025
Jacobsen S, 2005, VET RES, V36, P167, DOI 10.1051/vetres:2004062
Jiang HY, 2017, IMMUNOGENETICS, V69, P271, DOI 10.1007/s00251-016-0966-2
JOHNSON RW, 1993, PHYSIOL BEHAV, V53, P343, DOI 10.1016/0031-9384(93)90215-2
Johnson RW, 2002, VET IMMUNOL IMMUNOP, V87, P443, DOI 10.1016/S0165-2427(02)00069-7
Kacprzyk J, 2017, ACTA CHIROPTEROL, V19, P219, DOI [10.3161/15081109ACC2017.19.2.001, 10.3161/15081109acc2017.19.2.001]
Kiernan D., 2014, Open SUNY Textbooks.
Kimura Mayumi, 1994, American Journal of Physiology, V267, pR1596
King MO, 2013, J EXP BIOL, V216, P2097, DOI 10.1242/jeb.079574
Koutsos EA, 2001, COMP BIOCHEM PHYS C, V128, P255, DOI 10.1016/S1532-0456(00)00199-X
KOZAK W, 1994, AM J PHYSIOL, V266, pR125, DOI 10.1152/ajpregu.1994.266.1.R125
Kuzmin Ivan V, 2011, Emerg Health Threats J, V4, P7159, DOI 10.3402/ehtj.v4i0.7159
Lind CM, 2020, J EXP BIOL, V223, DOI 10.1242/jeb.225185
Llewellyn D, 2011, PHYSIOL BIOCHEM ZOOL, V84, P77, DOI 10.1086/657609
Lochmiller RL, 2000, OIKOS, V88, P87, DOI 10.1034/j.1600-0706.2000.880110.x
Lopes PC, 2021, J EXP BIOL, V224, DOI 10.1242/jeb.225847
MacDonald L, 2012, PHYSIOL BEHAV, V107, P762, DOI 10.1016/j.physbeh.2012.06.009
MacDonald L, 2011, AM J PHYSIOL-REG I, V301, pR172, DOI 10.1152/ajpregu.00057.2011
Maloney SK, 1998, J COMP PHYSIOL B, V168, P177, DOI 10.1007/s003600050134

Markowska M, 2017, DEV COMP IMMUNOL, V66, P130, DOI 10.1016/j.dci.2016.05.017
Martin L. B., 2008, ECOLOGY, V22, P68
Mathias S, 2000, NEUROENDOCRINOLOGY, V71, P375, DOI 10.1159/000054558
Maxwell S.E., 2004, Designing experiments and analyzing data: A model comparison perspective, V2nd
Meitern R, 2013, PLOS ONE, V8, DOI 10.1371/journal.pone.0067545
Melhado G, 2020, J EXP ZOOL PART A, V333, P536, DOI 10.1002/jez.2399
Merlo JL, 2016, J EXP ZOOL PART A, V325, P675, DOI 10.1002/jez.2060
Moratelli R, 2015, MEM I OSWALDO CRUZ, V110, P1
Moreno KR, 2021, ANN NY ACAD SCI, V1505, P178, DOI 10.1111/nyas.14600
Morrow JD, 2005, BRAIN BEHAV IMMUN, V19, P40, DOI 10.1016/j.bbi.2004.04.001
Mühldorfer K, 2013, ZOONOSES PUBLIC HLTH, V60, P93, DOI 10.1111/j.1863-2378.2012.01536.x
Nathan C, 2006, NAT REV IMMUNOL, V6, P173, DOI 10.1038/nri1785
Nomoto S, 1996, PFLUG ARCH EUR J PHY, V431, P987, DOI 10.1007/BF02332188
O'Shea TJ, 2014, EMERG INFECT DIS, V20, P741, DOI 10.3201/eid2005.130539
Otalora-Ardila A., 2017, MAMM BIOL
Otálora-Ardila A, 2016, PLOS ONE, V11, DOI 10.1371/journal.pone.0164938
Owen-Ashley NT, 2008, BRAIN BEHAV IMMUN, V22, P614, DOI 10.1016/j.bbi.2007.12.005
Owen-Ashley NT, 2007, J ORNITHOL, V148, pS583, DOI 10.1007/s10336-007-0197-2
Paksuz S., 2009, TRAKYA U J SCI, V10, P55
Patz JA, 2008, MED CLIN N AM, V92, P1473, DOI 10.1016/j.mcna.2008.07.007
Pérez-Nievas BG, 2010, BRAIN RES, V1315, P159, DOI 10.1016/j.brainres.2009.12.014
Ramirez-Otarola N, 2019, J COMP PHYSIOL B, V189, P143, DOI 10.1007/s00360-018-1195-9
Romanovsky AA, 1998, MED HYPOTHESES, V50, P219, DOI 10.1016/S0306-9877(98)90022-6
Romanovsky AA, 1996, AM J PHYSIOL-REG I, V271, pR244, DOI 10.1152/ajpregu.1996.271.1.R244
Romanovsky AA, 1996, AM J PHYSIOL-REG I, V270, pR693, DOI 10.1152/ajpregu.1996.270.4.R693
Roy S, 2017, PROTEIN PEPTIDE LETT, V24, P78, DOI 10.2174/0929866524666161121142221
Rudaya AY, 2005, AM J PHYSIOL-REG I, V289, pR1244, DOI 10.1152/ajpregu.00370.2005
Sampath VishnuPriya, 2018, Agriculture and Natural Resources, V52, P115, DOI 10.1016/j.anres.2018.08.002
Scheiermann C, 2013, NAT REV IMMUNOL, V13, P190, DOI 10.1038/nri3386
Scheiermann C, 2012, IMMUNITY, V37, P290, DOI 10.1016/j.immuni.2012.05.021

Schneeberger K, 2013, J EXP BIOL, V216, P4514, DOI 10.1242/jeb.090837
Schoenle L. A., 2018, ADV COMP IMMUNOLOGY, P901
Seltmann A, 2022, SCI REP-UK, V12, DOI 10.1038/s41598-022-18240-6
Shiraishi M, 1996, Environ Health Prev Med, V1, P65, DOI 10.1007/BF02931192
Sköld-Chiriac S, 2015, J EXP BIOL, V218, P2961, DOI 10.1242/jeb.122150
Soto L, 1998, J ENDOCRINOL, V159, P239, DOI 10.1677/joe.0.1590239
Steiner AA, 2019, TRENDS ENDOCRIN MET, V30, P875, DOI 10.1016/j.tem.2019.08.012
Steiner AA, 2009, AM J PHYSIOL-REG I, V297, pR485, DOI 10.1152/ajpregu.91026.2008
Stockmaier S, 2018, ANIM BEHAV, V140, P141, DOI 10.1016/j.anbehav.2018.04.021
Stockmaier S, 2015, BIOL LETTERS, V11, DOI 10.1098/rsbl.2015.0576
SUZUKI S, 1986, BIOCHEM PHARMACOL, V35, P3039
Taves MD, 2021, NAT REV IMMUNOL, V21, P233, DOI 10.1038/s41577-020-00464-0
Titon SCM, 2022, COMP BIOCHEM PHYS A, V267, DOI 10.1016/j.cbpa.2022.111184
Turmelle AS, 2010, J COMP PHYSIOL B, V180, P1155, DOI 10.1007/s00360-010-0486-6
Vinkler M, 2014, GENET SEL EVOL, V46, DOI 10.1186/s12711-014-0072-6
Voigt CC, 2020, SCI REP-UK, V10, DOI 10.1038/s41598-020-74473-3
Voigt Christian C., 2016, P1
Volkoff H, 2004, BRAIN RES, V998, P139, DOI 10.1016/j.brainres.2003.11.011
Wang LM, 2018, AM J NUCL MED MOLEC, V8, P86
Wang W, 2003, POULTRY SCI, V82, P1886, DOI 10.1093/ps/82.12.1886
Webel DM, 1998, POULTRY SCI, V77, P1893, DOI 10.1093/ps/77.12.1893
Weber TE, 2005, DOMEST ANIM ENDOCRIN, V28, P64, DOI 10.1016/j.domaniend.2004.05.005
Weise P, 2017, PEERJ, V5, DOI 10.7717/peerj.3570
White RJ, 2020, MAMMAL REV, V50, P336, DOI 10.1111/mam.12201
Williams JB, 2009, ARDEA, V97, P120, DOI 10.5253/078.097.0115
Wong S, 2007, REV MED VIROL, V17, P67, DOI 10.1002/rmv.520
Yamashita H, 2017, SHOCK, V47, P455, DOI 10.1097/SHK.0000000000000747
NR 112
TC 2
Z9 2
U1 0
U2 0
PU COMPANY BIOLOGISTS LTD
PI CAMBRIDGE
PA BIDDER BUILDING, STATION RD, HISTON, CAMBRIDGE CB24 9LF, ENGLAND
SN 0022-0949
EI 1477-9145
J9 J EXP BIOL
JI J. Exp. Biol.
PD DEC

PY 2022
VL 225
IS 24
AR jeb244583
DI 10.1242/jeb.244583
PG 12
WC Biology; Zoology
WE Science Citation Index Expanded (SCI-EXPANDED)
SC Life Sciences & Biomedicine - Other Topics; Zoology
GA 8V4HI
UT WOS:000930592700006
PM 36448935
OA Bronze
DA 2024-10-21
ER

PT J
AU Lum, LG
   Thakur, A
   Kondadasula, V
   Al-Kadhimi, Z
   Deol, A
   Tomaszewski, EN
   Yano, H
   Schalk, DL
   Ayash, L
   Zonder, JA
   Uberti, JP
   Abidi, MH
   Ratanatharathorn, V
AF Lum, Lawrence G.
   Thakur, Archana
   Kondadasula, Vidya
   Al-Kadhimi, Zaid
   Deol, Abhinav
   Tomaszewski, Elyse N.
   Yano, Hiroshi
   Schalk, Dana L.
   Ayash, Lois
   Zonder, Jeffrey A.
   Uberti, Joseph P.
   Abidi, Muneer H.
   Ratanatharathorn, Voravit
TI Targeting CD138-/CD20+Clonogenic Myeloma Precursor Cells
Decreases These
   Cells and Induces Transferable Antimyeloma Immunity
SO BIOLOGY OF BLOOD AND MARROW TRANSPLANTATION
LA English
DT Article
DE Activated T cells; Bispecific antibodies; Multiple myeloma
ID DOSE INTRAVENOUS MELPHALAN; RECEPTOR T-CELLS; MULTIPLE-MYELOMA;
   BISPECIFIC ANTIBODY; MG/M(2) MELPHALAN; BREAST-CANCER; HIGH-RISK;
   B-CELLS; TRANSPLANTATION; THERAPY
AB This phase lb clinical trial evaluated whether pretargeting of

CD20(+) clonogenic myeloma precursor cells (CMPCs) with anti-CD3 x anti-CD20 bispecific antibody-armed T cells (BATs) before autologous stem cell transplantation (SO') in patients with standard-risk and high-risk multiple myeloma would induce anti-myeloma immunity that could be detected and boosted after SO'. All 12 patients enrolled in this study received 2 BATs infusions before SO', and 4 patients received a booster infusion of BATs after SCT. Pretargeting CD138(-)/CD20(+) CMPCs with BATs before SO' was safe and reduced levels of CMPCs by up to 58% in the postinfusion bone marrow in patients who remained in remission. Four of 5 patients who remained in remission had a >5-fold increase in IFN-gamma enzyme-linked immunospot responses. SOX2 antibody increased after BATs infusions and persisted after SCT. The median anti-SOX2 level at 3 months after SCT was 28.1 ng/mL (range, 4.6 to 256 ng/mL) in patients who relapsed and 46 ng/mL (range, 28.3 to 73.3 ng/mL) in patients who remained in remission. The immune correlates suggest that infusions of targeted T cells given before SCT were able to reduce CMPC levels and induced cellular and humoral anti-myeloma immunity that could be transferred and boosted after SCT. (C) 2016 American Society for Blood and Marrow Transplantation.
C1 [Lum, Lawrence G.; Thakur, Archana; Kondadasula, Vidya; Al-Kadhimi, Zaid; Deol, Abhinav; Tomaszewski, Elyse N.; Yano, Hiroshi; Schalk, Dana L.; Ayash, Lois; Zonder, Jeffrey A.; Uberti, Joseph P.; Abidi, Muneer H.; Ratanatharathorn, Voravit] Wayne State Univ, Dept Oncol, Detroit, MI USA.
[Lum, Lawrence G.; Al-Kadhimi, Zaid; Ayash, Lois; Uberti, Joseph P.; Abidi, Muneer H.; Ratanatharathorn, Voravit] Wayne State Univ, Dept Med, Detroit, MI 48202 USA.
[Lum, Lawrence G.] Wayne State Univ, Dept Immunol & Microbiol, Detroit, MI USA.
C3 Wayne State University; Wayne State University; Wayne State University
RP Lum, LG; Thakur, A (corresponding author), Wayne State Univ, Dept Oncol, Detroit, MI USA.; Lum, LG (corresponding author), Wayne State Univ, Dept Med, Detroit, MI 48202 USA.; Lum, LG (corresponding author), Wayne State Univ, Dept Immunol & Microbiol, Detroit, MI USA.
EM LGL4F@virginia.edu; AT2FX@virginia.edu
OI Yano, Hiroshi/0000-0002-3098-2629; Abidi, Muneer/0000-0002-9936-6031
FU Leukemia and Lymphoma Society [TRP 6066-06, TRP 6092-09]; National
Cancer Institute [R01 CA 092344, R01 CA 140314]; Ray and Lynn Wood Neag
Foundation; Young Family Foundation; Karmanos Cancer Institute
FX L.G.L was supported in part by grants from the Leukemia and Lymphoma
Society (TRP 6066-06 and TRP 6092-09) and the National Cancer Institute
(R01 CA 092344 and R01 CA 140314). We acknowledge support from the Ray
and Lynn Wood Neag Foundation and the Young Family Foundation. J.A.Z
received startup funding from the Karmanos Cancer Institute.

CR Attal M, 1996, NEW ENGL J MED, V335, P91, DOI 10.1056/NEJM199607113350204
Barlogie B, 1999, BLOOD, V93, P55, DOI 10.1182/blood.V93.1.55.401k04_55_65
Bladé J, 2010, BLOOD, V115, P3655, DOI 10.1182/blood-2009-08-238196
Boucher K, 2012, CLIN CANCER RES, V18, P6155, DOI 10.1158/1078-0432.CCR-12-0531
Chan JK, 2006, CLIN CANCER RES, V12, P1859, DOI 10.1158/1078-0432.CCR-05-2019
Child JA, 2003, NEW ENGL J MED, V348, P1875, DOI 10.1056/NEJMoa022340
Fermand J, 2005, J CLIN ONCOL, V23, P9227, DOI 10.1200/JCO.2005.03.0551
Gall JM, 2005, EXP HEMATOL, V33, P452, DOI 10.1016/j.exphem.2005.01.007
Garfall AL, 2015, NEW ENGL J MED, V373, P1040, DOI 10.1056/NEJMoa1504542
Hosen N, 2012, LEUKEMIA, V26, P2135, DOI 10.1038/leu.2012.80
Ikegame A, 2012, LEUKEMIA, V26, P2124, DOI 10.1038/leu.2012.78
Jakubikova J, 2011, BLOOD, V117, P4409, DOI 10.1182/blood-2010-02-267344
Kobold S, 2011, CLIN DEV IMMUNOL, DOI 10.1155/2011/302145
Li XF, 2013, CANCER LETT, V336, P379, DOI 10.1016/j.canlet.2013.03.027
Lum HE, 2005, ANTICANCER RES, V25, P43
Lum L G, 1987, Prog Clin Biol Res, V244, P449
Lum L.G., 2015, J. ImmunoTherapy Cancer, V3, pP55, DOI [10.1186/2051-1426-3-S2-P55, DOI 10.1186/2051-1426-3-S2-P55]
Lum LG, 2015, CLIN CANCER RES, V21, P2305, DOI 10.1158/1078-0432.CCR-14-2280
Lum LG, 2013, BIOL BLOOD MARROW TR, V19, P925, DOI 10.1016/j.bbmt.2013.03.010
LUM LG, 1986, BLOOD, V67, P582
LUM LG, 1988, BONE MARROW TRANSPL, V3, P399
Matsui W, 2008, CANCER RES, V68, P190, DOI 10.1158/0008-5472.CAN-07-3096
Maude SL, 2014, NEW ENGL J MED, V371, P1507, DOI 10.1056/NEJMoa1407222
MCELWAIN TJ, 1983, LANCET, V2, P822
Moreau P, 2006, BLOOD, V107, P397, DOI 10.1182/blood-2005-06-2573
Moreau P, 2002, BLOOD, V99, P731, DOI 10.1182/blood.V99.3.731
Moreau P, 1996, BRIT J HAEMATOL, V95, P527, DOI 10.1046/j.1365-2141.1996.d01-1932.x
Porter DL, 2011, J CANCER, V2, P331, DOI 10.7150/jca.2.331
Raab MS, 2009, LANCET, V374, P324, DOI 10.1016/S0140-6736(09)60221-X
SAMUELS BL, 1995, J CLIN ONCOL, V13, P1786, DOI 10.1200/JCO.1995.13.7.1786
Sen M, 2001, J HEMATOTH STEM CELL, V10, P247, DOI 10.1089/15258160151134944
Spisek R, 2007, J EXP MED, V204, P831, DOI 10.1084/jem.20062387
Thakur A, 2015, ONCOIMMUNOL IN PRESS
Thakur A, 2015, J CLIN ONCOL, V33, DOI 10.1200/

jco.2015.33.15_suppl.3076
   Tian T, 2012, J BIOMED RES, V26, P336, DOI 10.7555/
JBR.26.20120045
   Vaishampayan U, 2015, PROSTATE CANCER, V2015, DOI
10.1155/2015/285193
NR 36
TC 18
Z9 21
U1 0
U2 5
PU ELSEVIER SCIENCE INC
PI NEW YORK
PA STE 800, 230 PARK AVE, NEW YORK, NY 10169 USA
SN 1083-8791
EI 1523-6536
J9 BIOL BLOOD MARROW TR
JI Biol. Blood Marrow Transplant.
PD MAY
PY 2016
VL 22
IS 5
BP 869
EP 878
DI 10.1016/j.bbmt.2015.12.030
PG 10
WC Hematology; Immunology; Transplantation
WE Science Citation Index Expanded (SCI-EXPANDED)
SC Hematology; Immunology; Transplantation
GA DJ5VF
UT WOS:000374277500013
PM 26827660
OA hybrid, Green Accepted
DA 2024-10-21
ER

PT J
AU Moore, MS
   Reichard, JD
   Murtha, TD
   Zahedi, B
   Fallier, RM
   Kunz, TH
AF Moore, Marianne S.
   Reichard, Jonathan D.
   Murtha, Timothy D.
   Zahedi, Bita
   Fallier, Renee M.
   Kunz, Thomas H.
TI Specific Alterations in Complement Protein Activity of Little
Brown
   Myotis (<i>Myotis lucifugus</i>) Hibernating in White-Nose
Syndrome
   Affected Sites
SO PLOS ONE

LA English
DT Article
ID IMMUNE-RESPONSE; FUNGAL-INFECTIONS; GROUND-SQUIRRELS; CITELLUS TRIDECEMLINEATUS; HUMORAL IMMUNITY; ADIPOSE-TISSUE; TRADE-OFFS; BODY-FAT; BATS; COST

AB White-nose syndrome (WNS) is the most devastating condition ever reported for hibernating bats, causing widespread mortality in the northeastern United States. The syndrome is characterized by cutaneous lesions caused by a recently identified psychrophilic and keratinophylic fungus (Geomyces destructans), depleted fat reserves, atypical behavior, and damage to wings; however, the proximate cause of mortality is still uncertain. To assess relative levels of immunocompetence in bats hibernating in WNS-affected sites compared with levels in unaffected bats, we describe blood plasma complement protein activity in hibernating little brown myotis (Myotis lucifugus) based on microbicidal competence assays using Escherichia coli, Staphylococcus aureus and Candida albicans. Blood plasma from bats collected during mid-hibernation at WNS-affected sites had higher bactericidal ability against E. coli and S. aureus, but lower fungicidal ability against C. albicans when compared with blood plasma from bats collected at unaffected sites. Within affected sites during mid-hibernation, we observed no difference in microbicidal ability between bats displaying obvious fungal infections compared to those without. Bactericidal ability against E. coli decreased significantly as hibernation progressed in bats collected from an affected site. Bactericidal ability against E. coli and fungicidal ability against C. albicans were positively correlated with body mass index (BMI) during late hibernation. We also compared complement activity against the three microbes within individuals and found that the ability of blood plasma from hibernating M. lucifugus to lyse microbial cells differed as follows: E. coli > S. aureus > C. albicans. Overall, bats affected by WNS experience both relatively elevated and reduced innate immune responses depending on the microbe tested, although the cause of observed immunological changes remains unknown. Additionally, considerable trade-offs may exist between energy conservation and immunological responses. Relationships between immune activity and torpor, including associated energy expenditure, are likely critical components in the development of WNS.

C1 [Moore, Marianne S.; Reichard, Jonathan D.; Murtha, Timothy D.; Zahedi, Bita; Fallier, Renee M.; Kunz, Thomas H.] Boston Univ, Dept Biol, Ctr Ecol & Conservat Biol, Boston, MA 02215 USA.
C3 Boston University
RP Moore, MS (corresponding author), Bucknell Univ, Dept Biol, Lewisburg, PA 17837 USA.
EM msm035@bucknell.edu
OI Murtha, Timothy/0000-0001-5427-9340

FU Bat Conservation International; National Speleological Society; New York
Department of Environmental Conservation; United States Fish and Wildlife Service
FX Funding for this study was provided by Bat Conservation International
(http://batcon.org/), the National Speleological Society

(http://www.caves.org/index.shtml), New York Department of Environmental
Conservation (http://www.dec.ny.gov/) and the United States Fish and
Wildlife Service (http://www.fws.gov/). The funders had no role in study
design, data collection and analysis, decision to publish, or
preparation of the manuscript.
CR Ahima RS, 2000, TRENDS ENDOCRIN MET, V11, P327, DOI 10.1016/S1043-2760(00)00301-5
ARDAWI MSM, 1985, ESSAYS BIOCHEM, V21, P1
Barr DP, 1922, ARCH INTERN MED, V29, P608, DOI 10.1001/archinte.1922.00110050055004
Blanco JL, 2008, VET IMMUNOL IMMUNOP, V125, P47, DOI 10.1016/j.vetimm.2008.04.020
Blehert DS, 2009, SCIENCE, V323, P227, DOI 10.1126/science.1163874
Bonneaud C, 2003, AM NAT, V161, P367, DOI 10.1086/346134
BROWN MB, 1974, J AM STAT ASSOC, V69, P364, DOI 10.2307/2285659
Burton RS, 1999, FUNCT ECOL, V13, P232, DOI 10.1046/j.1365-2435.1999.00302.x
CAHILL JE, 1967, J PARASITOL, V53, P110, DOI 10.2307/3276633
CALDER PC, 1994, BIOCHEM J, V300, P509, DOI 10.1042/bj3000509
Carey HV, 2003, PHYSIOL REV, V83, P1153, DOI 10.1152/physrev.00008.2003
Chaturvedi V, 2010, PLOS ONE, V5, DOI 10.1371/journal.pone.0010783
CHUTE RM, 1960, J PARASITOL, V46, P855, DOI 10.2307/3275550
Chute RM, 1960, J PARASITOLOGY S, V46, P21
Courtin F, 2010, VET PATHOL, V47, P214, DOI 10.1177/0300985809358614
Cryan PM, 2010, BMC BIOL, V8, DOI 10.1186/1741-7007-8-135
Demas GE, 2005, P ROY SOC B-BIOL SCI, V272, P1845, DOI 10.1098/rspb.2005.3126
Demas GE, 2003, P ROY SOC B-BIOL SCI, V270, P905, DOI 10.1098/rspb.2003.2341
Demas GE, 2004, HORM BEHAV, V45, P173, DOI 10.1016/j.yhbeh.2003.11.002
Demas GE, 1997, AM J PHYSIOL-REG I, V273, pR1631, DOI 10.1152/ajpregu.1997.273.5.R1631
EMMONS RW, 1966, AM J TROP MED HYG, V15, P428, DOI 10.4269/ajtmh.1966.15.428
Eraud C, 2005, FUNCT ECOL, V19, P110, DOI 10.1111/j.0269-8463.2005.00934.x
Frick WF, 2010, SCIENCE, V329, P679, DOI 10.1126/science.1188594
Galletti G, 1982, ACTA LEPROL, V88, P23
Gargas A, 2009, MYCOTAXON, V108, P147, DOI 10.5248/108.147
JAROSLOW BN, 1972, J EXP ZOOL, V181, P111, DOI 10.1002/jez.1401810112
JAROSLOW BN, 1968, P NATL ACAD SCI USA, V61, P69, DOI 10.1073/pnas.61.1.69
Kalabukhov N. I., 1958, Journal of Microbiology, Epidemiology and Immunobiology, V29, P1453
Kurtz CC, 2007, DEV COMP IMMUNOL, V31, P415, DOI 10.1016/

j.dci.2006.07.003
LARSEN B, 1971, COMP BIOCHEM PHYSIOL, V38, P571, DOI 10.1016/0300-9629(71)90124-1
LEHMANN PF, 1985, VET IMMUNOL IMMUNOP, V10, P33, DOI 10.1016/0165-2427(85)90038-8
Lochmiller RL, 2000, OIKOS, V88, P87, DOI 10.1034/j.1600-0706.2000.880110.x
Lorch JM, 2011, NATURE, V480, P376, DOI 10.1038/nature10590
MANASEK FJ, 1965, J CELL COMPAR PHYSL, V65, P319, DOI 10.1002/jcp.1030650305
Maniero GD, 2002, DEV COMP IMMUNOL, V26, P563, DOI 10.1016/S0145-305X(02)00006-X
Maniero GD, 2000, LIFE IN THE COLD, P493
Martin LB, 2003, P ROY SOC B-BIOL SCI, V270, P153, DOI 10.1098/rspb.2002.2185
Martin LB, 2006, INTEGR COMP BIOL, V46, P1030, DOI 10.1093/icb/icl039
Martínková N, 2010, PLOS ONE, V5, DOI 10.1371/journal.pone.0013853
Meteyer CU, 2009, J VET DIAGN INVEST, V21, P411, DOI 10.1177/104063870902100401
Moro K, 2010, NATURE, V463, P540, DOI 10.1038/nature08636
Nicola AM, 2008, CURR OPIN MICROBIOL, V11, P313, DOI 10.1016/j.mib.2008.05.011
Ots I, 2001, P ROY SOC B-BIOL SCI, V268, P1175, DOI 10.1098/rspb.2001.1636
Prendergast BJ, 2002, AM J PHYSIOL-REG I, V282, pR1054, DOI 10.1152/ajpregu.00562.2001
Puechmaille SJ, 2010, EMERG INFECT DIS, V16, P290, DOI 10.3201/eid1602.091391
Reichard JD, 2009, ACTA CHIROPTEROL, V11, P457, DOI 10.3161/150811009X485684
Romani L, 2008, MED MYCOL, V46, P515, DOI 10.1080/13693780801971450
Sadd BM, 2009, EVOL APPL, V2, P113, DOI 10.1111/j.1752-4571.2008.00057.x
Shoman S, 2005, BRIT J HAEMATOL, V129, P569
Speth C, 2004, MYCOSES, V47, P93, DOI 10.1111/j.1439-0507.2004.00979.x
Speth C, 2008, MYCOSES, V51, P477, DOI 10.1111/j.1439-0507.2008.01597.x
Sulkin S E, 1974, Monogr Virol, V8, P1
THOMAS DW, 1990, J MAMMAL, V71, P475, DOI 10.2307/1381967
Tieleman BI, 2005, P ROY SOC B-BIOL SCI, V272, P1715, DOI 10.1098/rspb.2005.3155
Turner Gregory G., 2009, Bat Research News, V50, P47
Wibbelt G, 2010, EMERG INFECT DIS, V16, P1237, DOI 10.3201/eid1608.100002
Xu DL, 2010, COMP BIOCHEM PHYS A, V155, P25, DOI 10.1016/j.cbpa.2009.09.003
Zysling DA, 2007, J COMP PHYSIOL B, V177, P339, DOI 10.1007/s00360-006-0133-4
NR 58
TC 46

Z9 56
U1 0
U2 44
PU PUBLIC LIBRARY SCIENCE
PI SAN FRANCISCO
PA 1160 BATTERY STREET, STE 100, SAN FRANCISCO, CA 94111 USA
SN 1932-6203
J9 PLOS ONE
JI PLoS One
PD NOV 30
PY 2011
VL 6
IS 11
AR e27430
DI 10.1371/journal.pone.0027430
PG 11
WC Multidisciplinary Sciences
WE Science Citation Index Expanded (SCI-EXPANDED)
SC Science & Technology - Other Topics
GA 863MI
UT WOS:000298168100009
PM 22140440
OA Green Published, gold
DA 2024-10-21
ER

PT J
AU CORRUCCINI, RS
AF CORRUCCINI, RS
TI BOOTSTRAP APPROACHES TO ESTIMATING CONFIDENCE-INTERVALS FOR MOLECULAR
   DISSIMILARITIES AND RESULTANT TREES
SO JOURNAL OF HUMAN EVOLUTION
LA English
DT Article
DE PRIMATES; DNA; HYBRIDIZATION; TRANSFERRIN; IMMUNOLOGY; MOLECULAR;
   DISTANCE
ID MITOCHONDRIAL-DNA SEQUENCES; PRIMATES; HYBRIDIZATION; PHYLOGENIES;
   LIMITS; SYSTEMATICS; HOMINOIDEA; BATS
RP CORRUCCINI, RS (corresponding author), SO ILLINOIS UNIV,DEPT ANTHROPOL,CARBONDALE,IL 62901, USA.
CR BABA ML, 1979, FOLIA PRIMATOL, V32, P207, DOI 10.1159/000155914
   BAKER RJ, 1991, SYST ZOOL, V40, P216, DOI 10.2307/2992259
   Barry D., 1987, STAT SCI, V2, P191
   CORRUCCINI R, 1990, HUMAN EVOLUTION, V5, P579
   CORRUCCINI RS, 1980, EVOLUTION, V34, P1216, DOI 10.1111/j.1558-5646.1980.tb04069.x
   CORRUCCINI RS, 1991, AM J PHYS ANTHR S, V12, P61
   CRONIN J, 1977, KROEBER ANTHR SOC PA, V50, P75
   Cronin J.E., 1983, P115
   Efron B., 1986, Statistical Science, V1, P54, DOI [10.1214/ss/1177013815, DOI 10.1214/SS/1177013815]
   FELSENSTEIN J, 1985, EVOLUTION, V39, P783, DOI 10.1111/

j.1558-5646.1985.tb00420.x
Goodman M., 1983, P67
Gould StephenJ., 1989, WONDERFUL LIFE
GROVES CP, 1988, J HUM EVOL, V17, P513, DOI 10.1016/0047-2484(88)90040-1
HASEGAWA M, 1989, J HUM EVOL, V18, P461, DOI 10.1016/0047-2484(89)90075-4
HASEGAWA M, 1989, EVOLUTION, V43, P672, DOI 10.1111/j.1558-5646.1989.tb04264.x
HASEGAWA M, 1984, P JPN ACAD B-PHYS, V60, P389, DOI 10.2183/pjab.60.389
KRAJEWSKI C, 1990, SYST ZOOL, V39, P383, DOI 10.2307/2992358
KRAUS F, 1991, SYST ZOOL, V40, P117, DOI 10.2307/2992252
MACPHEE RDE, 1991, AM J PHYS ANTHR S, V12, P121
MARKS J, 1991, AM J PHYS ANTHROPOL, V85, P207, DOI 10.1002/ajpa.1330850209
PETTIGREW JD, 1991, SYST ZOOL, V40, P199, DOI 10.2307/2992258
SAITOU N, 1991, AM J PHYS ANTHROPOL, V84, P75, DOI 10.1002/ajpa.1330840107
SANDERSON MJ, 1989, CLADISTICS, V5, P113, DOI 10.1111/j.1096-0031.1989.tb00559.x
Sarich V.M., 1983, P137
Sarich V.M., 1980, P399
SARICH V.M., 1971, BACKGROUND FORMAN, P60, DOI DOI 10.1111/j.1365-294X.2008.03982.x
SARICH VM, 1989, CLADISTICS, V5, P3, DOI 10.1111/j.1096-0031.1989.tb00480.x
SIBLEY CG, 1984, J MOL EVOL, V20, P2, DOI 10.1007/BF02101980
NR 28
TC 3
Z9 4
U1 0
U2 2
PU ACADEMIC PRESS LTD
PI LONDON
PA 24-28 OVAL RD, LONDON, ENGLAND NW1 7DX
SN 0047-2484
J9 J HUM EVOL
JI J. Hum. Evol.
PD DEC
PY 1992
VL 23
IS 6
BP 481
EP 493
DI 10.1016/0047-2484(92)90048-E
PG 13
WC Anthropology; Evolutionary Biology
WE Social Science Citation Index (SSCI); Science Citation Index Expanded (SCI-EXPANDED)
SC Anthropology; Evolutionary Biology
GA KD030
UT WOS:A1992KD03000003
DA 2024-10-21

ER

PT J
AU Brook, CE
   Boots, M
   Chandran, K
   Dobson, AP
   Drosten, C
   Graham, AL
   Grenfell, BT
   Müller, MA
   Ng, M
   Wang, LF
   van Leeuwen, A
AF Brook, Cara E.
   Boots, Mike
   Chandran, Kartik
   Dobson, Andrew P.
   Drosten, Christian
   Graham, Andrea L.
   Grenfell, Bryan T.
   Mueller, Marcel A.
   Ng, Melinda
   Wang, Lin-Fa
   van Leeuwen, Anieke
TI Accelerated viral dynamics in bat cell lines, with implications for
   zoonotic emergence
SO ELIFE
LA English
DT Article
ID IN-VIVO; ANTIVIRAL STATE; VIRUS-INFECTION; INTERFERON; ESTABLISHMENT;
   PATHOGENESIS; REPLICATION; EXPRESSION; RESERVOIRS; PATHOLOGY
AB Bats host virulent zoonotic viruses without experiencing disease. A mechanistic understanding of the impact of bats' virus hosting capacities, including uniquely constitutive immune pathways, on cellular-scale viral dynamics is needed to elucidate zoonotic emergence. We carried out virus infectivity assays on bat cell lines expressing induced and constitutive immune phenotypes, then developed a theoretical model of our in vitro system, which we fit to empirical data. Best fit models recapitulated expected immune phenotypes for representative cell lines, supporting robust antiviral defenses in bat cells that correlated with higher estimates for within-host viral propagation rates. In general, heightened immune responses limit pathogen-induced cellular morbidity, which can facilitate the establishment of rapidly-propagating persistent infections within-host. Rapidly-transmitting viruses that have evolved with bat immune systems will likely cause enhanced virulence following emergence into secondary hosts with immune systems that diverge from those unique to bats.
C1 [Brook, Cara E.; Boots, Mike] Univ Calif Berkeley, Dept Integrat Biol, Berkeley, CA 94720 USA.
   [Brook, Cara E.; Dobson, Andrew P.; Graham, Andrea L.; Grenfell,

Bryan T.; van Leeuwen, Anieke] Princeton Univ, Dept Ecol & Evolutionary Biol, Princeton, NJ 08544 USA.
[Chandran, Kartik; Ng, Melinda] Albert Einstein Coll Med, Dept Microbiol & Immunol, Bronx, NY 10467 USA.
[Drosten, Christian; Mueller, Marcel A.] Charite Univ Med Berlin, Inst Virol, Berlin, Germany.
[Drosten, Christian; Mueller, Marcel A.] Free Univ Berlin, Berlin, Germany.
[Drosten, Christian; Mueller, Marcel A.] Humboldt Univ, Berlin, Germany.
[Drosten, Christian; Mueller, Marcel A.] Berlin Inst Hlth, Berlin, Germany.
[Grenfell, Bryan T.] NIH, Fogarty Int Ctr, Bldg 10, Bethesda, MD 20892 USA.
[Mueller, Marcel A.] Sechenov Univ, Martsinovsky Inst Med Parasitol Trop & Vector Bor, Moscow, Russia.
[Wang, Lin-Fa] Duke Natl Univ Singapore, Emerging Infect Dis Program, Med Sch, Singapore, Singapore.
[van Leeuwen, Anieke] Univ Utrecht, Royal Netherlands Inst Sea Res, Dept Coastal Syst, Den Burg, Netherlands.
C3 University of California System; University of California Berkeley;
Princeton University; Montefiore Medical Center; Albert Einstein College
of Medicine; Yeshiva University; Berlin Institute of Health; Berlin
Institute of Health; Free University of Berlin; Humboldt University of
Berlin; Charite Universitatsmedizin Berlin; Free University of Berlin;
Humboldt University of Berlin; Berlin Institute of Health; Berlin
Institute of Health; National Institutes of Health (NIH) - USA; NIH
Fogarty International Center (FIC); Sechenov First Moscow State Medical
University; National University of Singapore; Utrecht University; Royal
Netherlands Institute for Sea Research (NIOZ)
RP Brook, CE (corresponding author), Univ Calif Berkeley, Dept Integrat Biol, Berkeley, CA 94720 USA.; Brook, CE (corresponding author), Princeton Univ, Dept Ecol & Evolutionary Biol, Princeton, NJ 08544 USA.
EM cbrook@berkeley.edu
RI Müller, Marcel/O-2425-2019; Drosten, Christian/AFF-8529-2022; Graham,
Andrea L./A-8808-2010
OI Wang, Lin-Fa/0000-0003-2752-0535; Graham, Andrea L./0000-0002-6580-2755;
Grenfell, Bryan/0000-0003-3227-5909; Mueller, Marcel/0000-0003-2242-5117
FU National Science Foundation Graduate Research Fellowship; Adolph C. and
Mary Sprague Miller Institute for Basic Research in Science, University

of California Berkeley Postdoctoral Fellowship; National Institutes of
Health [R01-AI134824, 1R01AI129822-01]; Singapore National Research
Foundation [NRF2012NRF-CRP001-056, NRF2016NRF-NSFC002-013]; Deutsche
Forschungsgemeinschaft [DR 772/10-2]; Bundesministerium fur Bildung und
Forschung RAPID [01KI1723A]; Horizon 2020 [653316]; DARPA PREEMPT
Program [D18AC00031]
FX National Science Foundation Graduate Research Fellowship Cara E Brookr
Adolph C. and Mary Sprague Miller Institute for Basic Research in
Science, University of California Berkeley Postdoctoral Fellowship Cara
E Brookr National Institutes of Health R01-AI134824 Kartik Chandranr
Singapore National Research Foundation NRF2012NRF-CRP001-056 Lin-Fa
Wangr Singapore National Research Foundation NRF2016NRF-NSFC002-013
Lin-Fa Wangr Deutsche Forschungsgemeinschaft DR 772/10-2 Christian
Drostenr Bundesministerium fur Bildung und Forschung RAPID #01KI1723A
Christian Drostenr Horizon 2020 #653316 Christian Drostenr DARPA PREEMPT
Program Cooperative Agreement no. D18AC00031 Cara E Brookr National
Institutes of Health 1R01AI129822-01 Cara E Brookr The funders had no
role in study design, data collection and interpretation, or the
decision to submit the work for publication.
CR Ahn M, 2019, NAT MICROBIOL, V4, P789, DOI 10.1038/s41564-019-0371-3
[Anonymous], COMPUTATIONAL STAT D
Arnold CE, 2018, VIRUSES-BASEL, V10, DOI 10.3390/v10110607
Baccam P, 2006, J VIROL, V80, P7590, DOI 10.1128/JVI.01623-05
Biesold SE, 2011, PLOS ONE, V6, DOI 10.1371/journal.pone.0028131
Bonhoeffer S, 1997, P NATL ACAD SCI USA, V94, P6971, DOI 10.1073/pnas.94.13.6971
Brook CE, 2019, J ANIM ECOL, V88, P1001, DOI 10.1111/1365-2656.12985
Brook CE, 2015, TRENDS MICROBIOL, V23, P172, DOI 10.1016/j.tim.2014.12.004
Calisher CH, 2006, CLIN MICROBIOL REV, V19, P531, DOI 10.1128/CMR.00017-06
COFFIN JM, 1995, SCIENCE, V267, P483, DOI 10.1126/science.7824947
Crameri G, 2009, PLOS ONE, V4, DOI 10.1371/journal.pone.0008266
Dembowski JA, 2015, PLOS PATHOG, V11, DOI 10.1371/journal.ppat.1004939
DESMYTER J, 1968, J VIROL, V2, P955, DOI 10.1128/JVI.2.10.955-961.1968
Dhooge A, 2008, MATH COMP MODEL DYN, V14, P147, DOI

10.1080/13873950701742754
DIEKMANN O, 1990, J MATH BIOL, V28, P365
Eddelbuettel D, 2011, J STAT SOFTW, V40, P1
EMENY JM, 1979, J GEN VIROL, V43, P247, DOI 10.1099/0022-1317-43-1-247
Emery VC, 1999, J EXP MED, V190, P177, DOI 10.1084/jem.190.2.177
Haydon DT, 2002, EMERG INFECT DIS, V8, P1468
Heffernan JM, 2005, J R SOC INTERFACE, V2, P281, DOI 10.1098/rsif.2005.0042
HO DD, 1995, NATURE, V373, P123, DOI 10.1038/373123a0
Hooper P, 2001, MICROBES INFECT, V3, P315, DOI 10.1016/S1286-4579(01)01385-5
Howat TJ, 2006, J R SOC INTERFACE, V3, P699, DOI 10.1098/rsif.2006.0136
Kacprzyk J, 2017, ACTA CHIROPTEROL, V19, P219, DOI [10.3161/15081109ACC2017.19.2.001, 10.3161/15081109acc2017.19.2.001]
Keeling M. J., 2008, MODELING INFECT DIS
Kühl A, 2011, J INFECT DIS, V204, pS840, DOI 10.1093/infdis/jir306
Kuzmin IV, 2017, J VIROL, V91, DOI [10.1128/JVI.02471-16, 10.1128/jvi.02471-16]
Livak KJ, 2001, METHODS, V25, P402, DOI 10.1006/meth.2001.1262
Mahanty S, 2004, LANCET INFECT DIS, V4, P487, DOI 10.1016/S1473-3099(04)01103-X
Miller EH, 2012, EMBO J, V31, P1947, DOI 10.1038/emboj.2012.53
Morris SE, 2018, PLOS PATHOG, V14, DOI 10.1371/journal.ppat.1007493
Nagai T, 2001, PHILOS MAG B, V81, P699, DOI 10.1080/13642810110055328
Neumann AU, 1998, SCIENCE, V282, P103, DOI 10.1126/science.282.5386.103
Ng M, 2015, ELIFE, V4, DOI 10.7554/eLife.11785
Nicholls JM, 2003, LANCET, V361, P1773, DOI 10.1016/S0140-6736(03)13413-7
Nowak M., 2000, VIRUS DYNAMICS MATH
NOWAK MA, 1995, NATURE, V375, P606, DOI 10.1038/375606a0
Nowak MA, 1996, P NATL ACAD SCI USA, V93, P4398, DOI 10.1073/pnas.93.9.4398
Pau G, 2010, BIOINFORMATICS, V26, P979, DOI 10.1093/bioinformatics/btq046
Pavlovich SS, 2018, CELL, V173, P1098, DOI 10.1016/j.cell.2018.03.070
Pawelek KA, 2012, PLOS COMPUT BIOL, V8, DOI 10.1371/journal.pcbi.1002588
Peel AJ, 2018, SCI REP-UK, V8, DOI 10.1038/s41598-018-22236-6
Peel AJ, 2012, PLOS ONE, V7, DOI 10.1371/journal.pone.0030346
Perelson AS, 1996, SCIENCE, V271, P1582, DOI 10.1126/science.271.5255.1582
Perelson AS, 2002, NAT REV IMMUNOL, V2, P28, DOI 10.1038/nri700
Plowright RK, 2016, PLOS NEGLECT TROP D, V10, DOI 10.1371/journal.pntd.0004796
RADKE KL, 1974, J VIROL, V13, P623, DOI 10.1128/JVI.13.3.623-630.1974
RASMUSSEN L, 1975, INFECT IMMUN, V12, P104, DOI 10.1128/

IAI.12.1.104-108.1975
   RHIM JS, 1969, P SOC EXP BIOL MED, V132, P670, DOI
10.3181/00379727-132-34285
   Saenz RA, 2010, J VIROL, V84, P3974, DOI 10.1128/JVI.02078-09
   SAMUEL CE, 1982, J BIOL CHEM, V257, P1791
   Schountz T, 2017, FRONT IMMUNOL, V8, DOI 10.3389/fimmu.2017.01098
   Soetaert K, 2010, J STAT SOFTW, V33, P1, DOI 10.18637/
jss.v033.i09
   Stetson DB, 2006, IMMUNITY, V25, P373, DOI 10.1016/
j.immuni.2006.08.007
   Takadate Y, 2020, CELL REP, V30, P308, DOI 10.1016/
j.celrep.2019.12.042
   Team R. C., 2019, R: a language and environment for statistical
computing
   Wang LF, 2019, CURR OPIN VIROL, V34, P79, DOI 10.1016/
j.coviro.2018.12.007
   Webb SD, 2007, J THEOR BIOL, V249, P140, DOI 10.1016/
j.jtbi.2007.06.013
   Wong AC, 2010, J VIROL, V84, P163, DOI 10.1128/JVI.01832-09
   Xie JZ, 2018, CELL HOST MICROBE, V23, P297, DOI 10.1016/
j.chom.2018.01.006
   Zhang GJ, 2013, SCIENCE, V339, P456, DOI 10.1126/science.1230835
   Zhou P, 2016, P NATL ACAD SCI USA, V113, P2696, DOI 10.1073/
pnas.1518240113
NR 62
TC 85
Z9 90
U1 1
U2 28
PU eLIFE SCIENCES PUBL LTD
PI CAMBRIDGE
PA SHERATON HOUSE, CASTLE PARK, CAMBRIDGE, CB3 0AX, ENGLAND
SN 2050-084X
J9 ELIFE
JI eLife
PD FEB 3
PY 2020
VL 9
AR e48401
DI 10.7554/eLife.48401
PG 24
WC Biology
WE Science Citation Index Expanded (SCI-EXPANDED)
SC Life Sciences & Biomedicine - Other Topics
GA KU5EH
UT WOS:000519732300001
PM 32011232
OA gold, Green Submitted, Green Published
DA 2024-10-21
ER

PT J
AU Jacquet, S
   Pontier, D

Etienne, L
AF Jacquet, Stephanie
Pontier, Dominique
Etienne, Lucie
TI Rapid Evolution of HERC6 and Duplication of a Chimeric HERC5/6 Gene in
Rodents and Bats Suggest an Overlooked Role of HERCs in Mammalian
Immunity
SO FRONTIERS IN IMMUNOLOGY
LA English
DT Article
DE HERC; restriction factor; antiviral immunity; gene duplication; genetic
conflicts; positive selection; HERC5; HERC6
ID RESTRICTION FACTOR SAMHD1; ANTIVIRAL SPECIFICITY; MOLECULAR PHYLOGENY;
POSITIVE SELECTION; E3 PROTEIN; HOST; ISG15; VIRUS; ISGYLATION; MODEL
AB Studying the evolutionary diversification of mammalian antiviral defenses is of main importance to better understand our innate immune repertoire. The small HERC proteins are part of a multigene family, including HERC5 and HERC6, which have probably diversified through complex evolutionary history in mammals. Here, we performed mammalian-wide phylogenetic and genomic analyses of HERC5 and HERC6, using 83 orthologous sequences from bats, rodents, primates, artiodactyls, and carnivores-the top five representative groups of mammalian evolution. We found that HERC5 has been under weak and differential positive selection in mammals, with only primate HERC5 showing evidences of pathogen-driven selection. In contrast, HERC6 has been under strong and recurrent adaptive evolution in mammals, suggesting past and widespread genetic arms-races with viral pathogens. Importantly, the rapid evolution of mammalian HERC6 spacer domain suggests that it might be a host-pathogen interface, targeting viral proteins and/or being the target of virus antagonists. Finally, we identified a HERC5/6 chimeric gene that arose from independent duplication in rodent and bat lineages and encodes for a conserved HERC5 N-terminal domain and divergent HERC6 spacer and HECT domains. This duplicated chimeric gene highlights adaptations that potentially contribute to rodent and bat immunity. Our findings open new research avenues on the functions of HERC6 and HERC5/6 in mammals, and on their implication in antiviral innate immunity.
C1 [Jacquet, Stephanie; Pontier, Dominique] Univ Lyon 1, Univ Lyon, CNRS, UMR 5558,Lab Biometrie & Biol Evolut, Villeurbanne, France.
[Jacquet, Stephanie; Etienne, Lucie] Univ Claude Bernard Lyon 1, Univ Lyon, CIRI Ctr Int Rech Infectiol, CNRS UMR5308,ENS Lyon,Inserm U111, Lyon, France.
[Jacquet, Stephanie; Pontier, Dominique; Etienne, Lucie] Univ Lyon, LabEx Ecofect, Lyon, France.
C3 Centre National de la Recherche Scientifique (CNRS); CNRS - Institute of
Ecology & Environment (INEE); VetAgro Sup; Universite Claude Bernard
Lyon 1; Centre National de la Recherche Scientifique (CNRS); CNRS

-
National Institute for Biology (INSB); Institut National de la Sante et
de la Recherche Medicale (Inserm); Universite Claude Bernard Lyon 1;
Ecole Normale Superieure de Lyon (ENS de LYON)
RP Jacquet, S; Pontier, D (corresponding author), Univ Lyon 1, Univ Lyon, CNRS, UMR 5558,Lab Biometrie & Biol Evolut, Villeurbanne, France.; Jacquet, S; Etienne, L (corresponding author), Univ Claude Bernard Lyon 1, Univ Lyon, CIRI Ctr Int Rech Infectiol, CNRS UMR5308,ENS Lyon,Inserm U111, Lyon, France.; Jacquet, S; Pontier, D; Etienne, L (corresponding author), Univ Lyon, LabEx Ecofect, Lyon, France.
EM stephanie.jacquet@univ-lyon1.fr; dominique.pontier@univ-lyon1.fr;
lucie.etienne@ens-lyon.fr
OI Etienne, Lucie/0000-0002-8585-7534
FU ANR LABEX ECOFECT (Universite de Lyon) [ANR-11-LABX-0048,
ANR-11-IDEX-0007]; CNRS; amfAR (Mathilde Krim Phase II Fellowship)
[109140-58-RKHF]; Fondation pour la Recherche Medicale (FRM Projet
Innovant) [ING20160435028]; FINOVI; ANRS [ECTZ19143, ECTZ118944];
European Regional Development Fund (ERDF) [2018-4512410/convention
P-2020-BAFE-9, 2018-4729510/convention P-2020-BAFE-23]; JORISS; ANR
EBOFAC
FX This work is funded by the ANR LABEX ECOFECT (ANR-11-LABX-0048 of the
Universite de Lyon, within the program Investissements d'Avenir
[ANR-11-IDEX-0007] operated by the French National Research Agency). LE
is further supported by the CNRS and by grants from amfAR (Mathilde Krim
Phase II Fellowship no. 109140-58-RKHF), the Fondation pour la Recherche
Medicale (FRM Projet Innovant no. ING20160435028), the FINOVI ("recently
settled scientist" grant), the ANRS (no. ECTZ19143 and ECTZ118944), and
a JORISS incubating grant. DP is supported by the CNRS, the European
Regional Development Fund (ERDF) 2018-4512410/convention P-2020-BAFE-9
and 2018-4729510/convention P-2020-BAFE-23, and the ANR EBOFAC.
CR Agarwala R, 2018, NUCLEIC ACIDS RES, V46, pD8, DOI [10.1093/nar/gkx1095, 10.1093/nar/gkq1172, 10.1093/nar/gks1189]
Arimoto K, 2015, J INTERF CYTOK RES, V35, P351, DOI 10.1089/jir.2014.0113
Blanco-Melo D, 2016, CELL HOST MICROBE, V20, P189, DOI 10.1016/j.chom.2016.06.007
Boso G, 2019, SCI REP-UK, V9, DOI 10.1038/s41598-019-47720-5
Compton AA, 2012, CELL HOST MICROBE, V11, P91, DOI 10.1016/j.chom.2011.11.010

Conticello SG, 2005, MOL BIOL EVOL, V22, P367, DOI 10.1093/molbev/msi026
Dastur A, 2006, J BIOL CHEM, V281, P4334, DOI 10.1074/jbc.M512830200
Daugherty MD, 2019, CURR OPIN GENET DEV, V58-59, P49, DOI 10.1016/j.gde.2019.07.011
Daugherty MD, 2016, ELIFE, V5, DOI 10.7554/eLife.14228
Daugherty MD, 2012, ANNU REV GENET, V46, P677, DOI 10.1146/annurev-genet-110711-155522
Duggal NK, 2012, NAT REV IMMUNOL, V12, P687, DOI 10.1038/nri3295
Durfee LA, 2010, MOL CELL, V38, P722, DOI 10.1016/j.molcel.2010.05.002
Eduardo-Correia B, 2014, J VIROL, V88, P2312, DOI 10.1128/JVI.03293-13
Elde NC, 2009, NATURE, V457, P485, DOI 10.1038/nature07529
Etienne L, 2015, PLOS PATHOG, V11, DOI 10.1371/journal.ppat.1005149
Fabre PH, 2012, BMC EVOL BIOL, V12, DOI 10.1186/1471-2148-12-88
Garcia-Gonzalo FR, 2005, CELL MOL LIFE SCI, V62, P1826, DOI 10.1007/s00018-005-5119-y
Guindon S, 2010, SYST BIOL, V59, P307, DOI 10.1093/sysbio/syq010
Hayward JA, 2018, MOL BIOL EVOL, V35, P1626, DOI 10.1093/molbev/msy048
Heusinger E, 2015, J VIROL, V89, P12154, DOI 10.1128/JVI.02149-15
Hickford D, 2012, BMC GENOMICS, V13, DOI 10.1186/1471-2164-13-155
Hochrainer K, 2005, GENOMICS, V85, P153, DOI 10.1016/j.ygeno.2004.10.006
Hölzer M, 2019, ISCIENCE, V19, P647, DOI 10.1016/j.isci.2019.08.016
Huang L, 1999, SCIENCE, V286, P1321, DOI 10.1126/science.286.5443.1321
Hubel P, 2019, NAT IMMUNOL, V20, P493, DOI 10.1038/s41590-019-0323-3
Hurles M, 2004, PLOS BIOL, V2, P900, DOI 10.1371/journal.pbio.0020206
Ito J, 2020, P NATL ACAD SCI USA, V117, P610, DOI 10.1073/pnas.1914183116
Ketscher L, 2012, BIOCHEM BIOPH RES CO, V417, P135, DOI 10.1016/j.bbrc.2011.11.071
Kluge SF, 2015, CELL, V163, DOI 10.1016/j.cell.2015.10.019
Kondrashov FA, 2012, P ROY SOC B-BIOL SCI, V279, P5048, DOI 10.1098/rspb.2012.1108
Laguette N, 2012, CELL HOST MICROBE, V11, P205, DOI 10.1016/j.chom.2012.01.007
Larue RS, 2008, BMC MOL BIOL, V9, DOI 10.1186/1471-2199-9-104
Lefort V, 2017, MOL BIOL EVOL, V34, P2422, DOI 10.1093/molbev/msx149
Lim ES, 2012, CELL HOST MICROBE, V11, P194, DOI 10.1016/j.chom.2012.01.004
Lim ES, 2010, J VIROL, V84, P7124, DOI 10.1128/JVI.00468-10
Liu Y, 2013, PLOS ONE, V8, DOI 10.1371/journal.pone.0066859
Malfavon-Borja R, 2013, GENOME BIOL EVOL, V5, P2141, DOI 10.1093/gbe/evt163
Marín I, 2010, BMC EVOL BIOL, V10, DOI 10.1186/1471-2148-10-56

McBee RM, 2015, MOL BIOL EVOL, V32, P1091, DOI 10.1093/molbev/msu399
McCarthy KR, 2015, PLOS PATHOG, V11, DOI 10.1371/journal.ppat.1005085
McLaughlin RN, 2017, J EXP BIOL, V220, P6, DOI 10.1242/jeb.148148
Meyerson NR, 2011, TRENDS MICROBIOL, V19, P286, DOI 10.1016/j.tim.2011.03.006
Mitchell PS, 2013, CURR OPIN MICROBIOL, V16, P493, DOI 10.1016/j.mib.2013.04.005
Mitchell PS, 2012, CELL HOST MICROBE, V12, P598, DOI 10.1016/j.chom.2012.09.005
Mollentze N, 2020, P NATL ACAD SCI USA, V117, P9423, DOI 10.1073/pnas.1919176117
Münk C, 2012, BMC EVOL BIOL, V12, DOI 10.1186/1471-2148-12-71
Murrell B, 2013, MOL BIOL EVOL, V30, P1196, DOI 10.1093/molbev/mst030
Murrell B, 2012, PLOS GENET, V8, DOI 10.1371/journal.pgen.1002764
Olival KJ, 2017, NATURE, V546, P646, DOI 10.1038/nature22975
Opazo JC, 2005, MOL PHYLOGENET EVOL, V37, P932, DOI 10.1016/j.ympev.2005.05.002
Oudshoorn D, 2012, PLOS ONE, V7, DOI 10.1371/journal.pone.0029870
Paparisto E, 2018, J VIROL, V92, DOI 10.1128/JVI.00528-18
Picard L, 2020, NUCLEIC ACIDS RES, V48, DOI 10.1093/nar/gkaa680
Pond SLK, 2020, MOL BIOL EVOL, V37, P295, DOI 10.1093/molbev/msz197
Pond SLK, 2006, BIOINFORMATICS, V22, P3096, DOI 10.1093/bioinformatics/btl474
Pond SLK, 2011, MOL BIOL EVOL, V28, P3033, DOI 10.1093/molbev/msr125
Pond SLK, 2005, BIOINFORMATICS, V21, P676, DOI 10.1093/bioinformatics/bti079
Ranwez V, 2018, MOL BIOL EVOL, V35, P2582, DOI 10.1093/molbev/msy159
Renault L, 1998, NATURE, V392, P97, DOI 10.1038/32204
Rothenburg S, 2009, NAT STRUCT MOL BIOL, V16, P63, DOI 10.1038/nsmb.1529
Sauter D, 2019, CELL HOST MICROBE, V25, P27, DOI 10.1016/j.chom.2018.12.002
Sawyer SL, 2007, PLOS PATHOG, V3, P1918, DOI 10.1371/journal.ppat.0030197
Sawyer SL, 2005, P NATL ACAD SCI USA, V102, P2832, DOI 10.1073/pnas.0409853102
Sawyer SL, 2004, PLOS BIOL, V2, P1278, DOI 10.1371/journal.pbio.0020275
Sayers EW, 2020, NUCLEIC ACIDS RES, V48, pD84, DOI [10.1093/nar/gkz956, 10.1093/nar/gky989, 10.1093/nar/gkaa1023]
Sironi M, 2015, NAT REV GENET, V16, P224, DOI 10.1038/nrg3905
Smith MD, 2015, MOL BIOL EVOL, V32, P1342, DOI 10.1093/molbev/msv022
Stadelmann B, 2007, MOL PHYLOGENET EVOL, V43, P32, DOI 10.1016/j.ympev.2006.06.019
Sun Z, 2012, J VIROL, V86, P3839, DOI 10.1128/JVI.06466-11
Swatek KN, 2018, P NATL ACAD SCI USA, V115, P2371, DOI 10.1073/pnas.1710617115

   Tareen SU, 2009, VIROLOGY, V385, P473, DOI 10.1016/
j.virol.2008.12.018
   Teeling EC, 2005, SCIENCE, V307, P580, DOI 10.1126/
science.1105113
   Versteeg GA, 2010, J VIROL, V84, P5423, DOI 10.1128/JVI.02395-09
   Villarroya-Beltri C, 2017, J CELL SCI, V130, P2961, DOI 10.1242/
jcs.205468
   Voloch Carolina M., 2013, BMC Research Notes, V6, P160, DOI
10.1186/1756-0500-6-160
   Wong JJY, 2006, P NATL ACAD SCI USA, V103, P10735, DOI 10.1073/
pnas.0600397103
   Woods MW, 2011, RETROVIROLOGY, V8, DOI 10.1186/1742-4690-8-95
   Woods MW, 2014, RETROVIROLOGY, V11, DOI 10.1186/1742-4690-11-27
   Wu F, 2013, PLOS PATHOG, V9, DOI 10.1371/journal.ppat.1003577
   Xue F, 2015, J TRANSL MED, V13, DOI 10.1186/s12967-015-0743-2
   Yang L, 2020, ELIFE, V9, DOI 10.7554/eLife.58436
   Yang ZH, 2007, MOL BIOL EVOL, V24, P1586, DOI 10.1093/molbev/
msm088
   Zhang JZ, 2003, TRENDS ECOL EVOL, V18, P292, DOI 10.1016/
S0169-5347(03)00033-8
   Zhang ZL, 2017, PLOS PATHOG, V13, DOI 10.1371/
journal.ppat.1006746
   Zhao C, 2010, P NATL ACAD SCI USA, V107, P2253, DOI 10.1073/
pnas.0909144107
   Zhou X, 2013, INT J BIOL SCI, V9, P200, DOI 10.7150/ijbs.5613
NR 86
TC 16
Z9 18
U1 1
U2 10
PU FRONTIERS MEDIA SA
PI LAUSANNE
PA AVENUE DU TRIBUNAL FEDERAL 34, LAUSANNE, CH-1015, SWITZERLAND
SN 1664-3224
J9 FRONT IMMUNOL
JI Front. Immunol.
PD DEC 18
PY 2020
VL 11
AR 605270
DI 10.3389/fimmu.2020.605270
PG 15
WC Immunology
WE Science Citation Index Expanded (SCI-EXPANDED)
SC Immunology
GA PM7OU
UT WOS:000603984300001
PM 33391270
OA Green Published, gold, Green Submitted
DA 2024-10-21
ER

PT J
AU Stockmaier, S

Dechmann, DKN
Page, RA
O'Mara, MT
AF Stockmaier, Sebastian
Dechmann, Dina K. N.
Page, Rachel A.
Teague O'Mara, M.
TI No fever and leucocytosis in response to a lipopolysaccharide challenge
in an insectivorous bat
SO BIOLOGY LETTERS
LA English
DT Article
DE lipopolysaccharide; white blood cell count; Molossus; immune response;
toll-like receptor 4; mass loss
ID IMMUNE-SYSTEM; HIBERNATION; FLIGHT; LPS
AB Bat immune systems may allow them to respond to zoonotic agents more efficiently than other mammals. As the first line of defence, the taxonomically conserved acute phase immune reaction of leucocytosis and fever is crucial for coping with infections, but it is unknown if this response is a key constituent to bat immunological success. We investigated the acute phase reaction to a standard lipopolysaccharide (LPS) challenge in Pallas's mastiff bats (Molossus molossus). Challenged bats lost mass, but in contrast to other mammals showed no leucocytosis or fever. There also was no influence on body temperature reduction during torpor. When compared to recent genome-wide assays for constituent immune genes, this lack of a conserved fever response to LPS contributes to a clearer understanding of the innate immune system in bat species and of the coevolution of bats with a wide diversity of pathogens.
C1 [Stockmaier, Sebastian; Dechmann, Dina K. N.; Teague O'Mara, M.] Max Planck Inst Ornithol, Dept Migrat & Immunoecol, D-78315 Radolfzell am Bodensee, Germany.
[Stockmaier, Sebastian; Dechmann, Dina K. N.; Teague O'Mara, M.] Univ Konstanz, Dept Biol, D-78464 Constance, Germany.
[Teague O'Mara, M.] Univ Konstanz, Zukunftskolleg, D-78464 Constance, Germany.
[Stockmaier, Sebastian; Dechmann, Dina K. N.; Page, Rachel A.; Teague O'Mara, M.] Smithsonian Trop Res Inst, Balboa, Ancon, Panama.
C3 Max Planck Society; University of Konstanz; University of Konstanz;
Smithsonian Institution; Smithsonian Tropical Research Institute
RP O'Mara, MT (corresponding author), Max Planck Inst Ornithol, Dept Migrat & Immunoecol, Obstberg 1, D-78315 Radolfzell am Bodensee, Germany.
EM tomara@orn.mpg.de
RI Page, Rachel/AAE-4816-2021; Dechmann, Dina/A-1827-2010; Page, Rachel/GQQ-2818-2022; O'Mara, M. Teague/J-6090-2012
OI Page, Rachel/0000-0001-7072-0669; Stockmaier, Sebastian/0000-0001-8280-8086; O'Mara, M. Teague/ 0000-0002-6951-1648;
Dechmann, Dina K. N./0000-0003-0043-8267
FU Max Planck Institute for Ornithology; German Research Foundation

(DFG)
[DE 1807/3-1]; Smithsonian Tropical Research Institute
FX This work was funded by the Max Planck Institute for Ornithology,
Smithsonian Tropical Research Institute, and German Research
Foundation
(DFG) award DE 1807/3-1 to D.K.N.D.
CR Altenburg SP, 2002, PROG NEURO-PSYCHOPH, V26, P891, DOI 10.1016/S0278-5846(01)00335-9
[Anonymous], BMC EVOL BIOL, DOI DOI 10.1186/1471-2148-13-194)
Auguie B, 2017, gridExtra: miscellaneous functions for grid graphics. R package version 2.3
Bouma HR, 2010, J LEUKOCYTE BIOL, V88, P619, DOI 10.1189/jlb.0310174
Brook CE, 2015, TRENDS MICROBIOL, V23, P172, DOI 10.1016/j.tim.2014.12.004
Canale CI, 2011, FUNCT ECOL, V25, P557, DOI 10.1111/j.1365-2435.2010.01815.x
Copeland S, 2005, CLIN DIAGN LAB IMMUN, V12, P60, DOI 10.1128/CDLI.12.1.60-67.2005
Cowled C, 2011, DEV COMP IMMUNOL, V35, P7, DOI 10.1016/j.dci.2010.07.006
Dechmann DKN, 2011, J EXP BIOL, V214, P3605, DOI 10.1242/jeb.056010
Evans SS, 2015, NAT REV IMMUNOL, V15, P335, DOI 10.1038/nri3843
Gray DA, 2013, J COMP PHYSIOL B, V183, P297, DOI 10.1007/s00360-012-0718-z
KOZAK W, 1994, AM J PHYSIOL, V266, pR125, DOI 10.1152/ajpregu.1994.266.1.R125
Luis AD, 2013, P ROY SOC B-BIOL SCI, V280, DOI 10.1098/rspb.2012.2753
O'Shea TJ, 2014, EMERG INFECT DIS, V20, P741, DOI 10.3201/eid2005.130539
Prendergast BJ, 2002, AM J PHYSIOL-REG I, V282, pR1054, DOI 10.1152/ajpregu.00562.2001
Schneeberger K, 2013, J EXP BIOL, V216, P4514, DOI 10.1242/jeb.090837
Schneeberger K, 2013, PLOS ONE, V8, DOI 10.1371/journal.pone.0054023
Triantafilou M, 2002, TRENDS IMMUNOL, V23, P301, DOI 10.1016/S1471-4906(02)02233-0
Tschirren B, 2013, P ROY SOC B-BIOL SCI, V280, DOI 10.1098/rspb.2013.0364
Zhang GJ, 2013, SCIENCE, V339, P456, DOI 10.1126/science.1230835
NR 20
TC 36
Z9 38
U1 0
U2 20
PU ROYAL SOC
PI LONDON
PA 6-9 CARLTON HOUSE TERRACE, LONDON SW1Y 5AG, ENGLAND
SN 1744-9561
EI 1744-957X
J9 BIOL LETTERS

JI Biol. Lett.
PD SEP 1
PY 2015
VL 11
IS 9
AR 20150576
DI 10.1098/rsbl.2015.0576
PG 4
WC Biology; Ecology; Evolutionary Biology
WE Science Citation Index Expanded (SCI-EXPANDED)
SC Life Sciences & Biomedicine - Other Topics; Environmental Sciences &
   Ecology; Evolutionary Biology
GA CW1SS
UT WOS:000364772300016
PM 26333664
OA Bronze, Green Published
DA 2024-10-21
ER

PT J
AU Huang, ZX
   Jebb, D
   Teeling, EC
AF Huang, Zixia
   Jebb, David
   Teeling, Emma C.
TI Blood miRNomes and transcriptomes reveal novel longevity mechanisms in
   the long-lived bat, <i>Myotis myotis</i>
SO BMC GENOMICS
LA English
DT Article
DE Bats; Illumina miRNA-Seq; Blood miRNA; Comparative miRNome; Myotis
   myotis
ID DIFFERENTIAL EXPRESSION ANALYSIS; GENE-EXPRESSION; DOWN-REGULATION;
   MICRORNAS; EVOLUTION; ALIGNMENT; CANCER; PHYSIOLOGY; APOPTOSIS;
INSIGHTS
AB Background: Chiroptera, the bats, are the only order of mammals capable of true self-powered flight. Bats exhibit a number of other exceptional traits such as echolocation, viral tolerance and, perhaps most puzzlingly, extreme longevity given their body size. Little is known about the molecular mechanisms driving their extended longevity particularly at the levels of gene expression and post-transcriptional regulation. To elucidate the molecular mechanisms that may underlie their unusual longevity, we have deep sequenced 246.5 million small RNA reads from whole blood of the long-lived greater mouse-eared bats, Myotis myotis, and conducted a series of genome-wide comparative analyses between bat and non-bat mammals (human, pig and cow) in both blood miRNomes and transcriptomes, for the first time.
   Results: We identified 539 miRNA gene candidates from bats, of

which 468 unique mature miRNA were obtained. More than half of these miRNA (65.1 %) were regarded as bat-specific, regulating genes involved in the immune, ageing and tumorigenesis pathways. We have also developed a stringent pipeline for genome-wide miRNome comparisons across species, and identified 37 orthologous miRNA groups shared with bat, human, pig and cow, 6 of which were differentially expressed. For bats, 3 out of 4 up-regulated miRNA (miR-101-3p, miR-16-5p, miR-143-3p) likely function as tumor suppressors against various kinds of cancers, while one down-regulated miRNA (miR-221-5p) acts as a tumorigenesis promoter in human breast and pancreatic cancers. Additionally, a genome-wide comparison of mRNA transcriptomes across species also revealed specific gene expression patterns in bats. 127 up-regulated genes were enriched mainly in mitotic cell cycle and DNA repair mechanisms, while 364 down-regulated genes were involved primarily in mitochondrial activity.

Conclusions: Our comprehensive and integrative analyses revealed bat-specific and differentially expressed miRNA and mRNA that function in key longevity pathways, producing a distinct bat gene expression pattern. For the first time, we show that bats may possess unique regulatory mechanisms for resisting tumorigenesis, repairing cellular damage and preventing oxidative stresses, all of which likely contribute to the extraordinary lifespan of Myotis myotis.
C1 [Huang, Zixia; Jebb, David; Teeling, Emma C.] Univ Coll Dublin, UCD Sch Biol & Environm Sci, Dublin 4, Ireland.
C3 University College Dublin
RP Teeling, EC (corresponding author), Univ Coll Dublin, UCD Sch Biol & Environm Sci, Dublin 4, Ireland.
EM emma.teeling@ucd.ie
RI Huang, Zixia/AAO-6936-2021; Juan, David/AAA-2614-2020
OI Teeling, Emma/0000-0002-3309-1346; Huang, Zixia/0000-0002-1298-0486
FU European Research Council [ERC-2012-StG311000]; China Scholarship Council studentship (UCD-CSC funding programme); Contrat Nature 'Etude de la dynamique des populations de grand murin (Myotis myotis) en Bretagne et Pays de Loire'
FX This study was funded by a European Research Council Research Grant ERC-2012-StG311000 awarded to Emma C. Teeling and a China Scholarship Council studentship (under UCD-CSC funding programme) to Zixia Huang, and supported by the Contrat Nature 'Etude de la dynamique des populations de grand murin (Myotis myotis) en Bretagne et Pays de Loire' awarded to Bretagne Vivante (BV).
CR ALTSCHUL SF, 1990, J MOL BIOL, V215, P403, DOI 10.1016/S0022-2836(05)80360-2
Anders S, 2010, GENOME BIOL, V11, DOI 10.1186/gb-2010-11-10-r106
Austad SN, 2010, J COMP PATHOL, V141, pS10, DOI 10.1016/j.jcpa.2009.10.024
Bao H, 2013, BMC GENOMICS, V14, DOI 10.1186/1471-2164-14-609

Baskerville S, 2005, RNA, V11, P241, DOI 10.1261/rna.7240905
Berezikov E, 2011, NAT REV GENET, V12, P846, DOI 10.1038/nrg3079
Bos JL, 2007, CELL, V129, P865, DOI 10.1016/j.cell.2007.05.018
Brooks R, 2010, J IMMUNOL, V184, P3582, DOI 10.4049/jimmunol.0902844
Brunet-Rossinni AK, 2004, MECH AGEING DEV, V125, P11, DOI 10.1016/j.mad.2003.09.003
Conesa Ana, 2008, Int J Plant Genomics, V2008, P619832, DOI 10.1155/2008/619832
Cowled C, 2014, BMC GENOMICS, V15, DOI 10.1186/1471-2164-15-682
Crawford DR, 1997, FREE RADICAL BIO MED, V22, P551, DOI 10.1016/S0891-5849(96)00380-2
Deng BA, 2015, GENE, V571, P292, DOI 10.1016/j.gene.2015.06.083
Dhakshinamoorthy S, 2005, J BIOL CHEM, V280, P16891, DOI 10.1074/jbc.M500166200
Dong D, 2013, BMC GENOMICS, V14, DOI 10.1186/1471-2164-14-916
Enright Anton J, 2003, Genome Biol, V5, pR1, DOI 10.1186/gb-2003-5-1-r1
Etebari K, 2014, PLOS ONE, V9, DOI 10.1371/journal.pone.0084747
Faraoni I, 2009, BBA-MOL BASIS DIS, V1792, P497, DOI 10.1016/j.bbadis.2009.02.013
Friedländer MR, 2008, NAT BIOTECHNOL, V26, P407, DOI 10.1038/nbt1394
Glennon NB, 2015, J VIROL, V89, P7550, DOI 10.1128/JVI.00302-15
Gudmundsdottir K, 2006, ONCOGENE, V25, P5864, DOI 10.1038/sj.onc.1209874
Huang ZX, 2016, MOL ECOL RESOUR, V16, P150, DOI 10.1111/1755-0998.12447
Kim EB, 2011, NATURE, V479, P223, DOI 10.1038/nature10533
Kinsella RJ, 2011, DATABASE-OXFORD, DOI 10.1093/database/bar030
Kozomara A, 2014, NUCLEIC ACIDS RES, V42, pD68, DOI 10.1093/nar/gkt1181
Krzywinski M, 2009, GENOME RES, V19, P1639, DOI 10.1101/gr.092759.109
Ksionda Olga, 2013, Front Biol (Beijing), V8, P508
Langmead B, 2009, GENOME BIOL, V10, DOI 10.1186/gb-2009-10-3-r25
LEE RC, 1993, CELL, V75, P843, DOI 10.1016/0092-8674(93)90529-Y
Lei M, 2014, PLOS ONE, V9, DOI 10.1371/journal.pone.0107746
Leidinger P, 2013, GENOME BIOL, V14, DOI 10.1186/gb-2013-14-7-r78
Lenaz G, 2001, IUBMB LIFE, V52, P159, DOI 10.1080/15216540152845957
Lewis BP, 2003, CELL, V115, P787, DOI 10.1016/S0092-8674(03)01018-3
Li N, 2011, MECH AGEING DEV, V132, P75, DOI 10.1016/j.mad.2010.12.004
Li WZ, 2006, BIOINFORMATICS, V22, P1658, DOI 10.1093/bioinformatics/btl158
Liew CC, 2006, J LAB CLIN MED, V147, P126, DOI 10.1016/j.lab.2005.10.005
Liu LP, 2012, MOL MED REP, V5, P753, DOI 10.3892/mmr.2011.696
Maes OC, 2008, MECH AGEING DEV, V129, P534, DOI 10.1016/j.mad.2008.05.004
Martin M., 2011, EMBNET J, V17, P10, DOI [10.14806/ej.17.1.200, DOI 10.14806/EJ.17.1.200, 10.14806/EJ.17.1.200]

Memczak S, 2015, PLOS ONE, V10, DOI 10.1371/journal.pone.0141214
Mortazavi A, 2008, NAT METHODS, V5, P621, DOI 10.1038/nmeth.1226
Munshi-South J, 2010, AGEING RES REV, V9, P12, DOI 10.1016/j.arr.2009.07.006
Paczynska P, 2015, BMC GENET, V15, DOI 10.1186/s12863-015-0166-3
Parker J, 2013, NATURE, V502, P228, DOI 10.1038/nature12511
Parra G, 2007, BIOINFORMATICS, V23, P1061, DOI 10.1093/bioinformatics/btm071
Patel RK, 2012, PLOS ONE, V7, DOI 10.1371/journal.pone.0030619
Patro R, 2014, NAT BIOTECHNOL, V32, P462, DOI 10.1038/nbt.2862
Persengiev S, 2011, NEUROBIOL AGING, V32, DOI 10.1016/j.neurobiolaging.2010.03.014
Platt RN, 2014, MOL BIOL EVOL, V31, P1536, DOI 10.1093/molbev/msu112
Podlutsky AJ, 2005, J GERONTOL A-BIOL, V60, P1366, DOI 10.1093/gerona/60.11.1366
Quinlan AR, 2010, BIOINFORMATICS, V26, P841, DOI 10.1093/bioinformatics/btq033
Robinson MD, 2010, GENOME BIOL, V11, DOI 10.1186/gb-2010-11-3-r25
Rodriguez A, 2004, GENOME RES, V14, P1902, DOI 10.1101/gr.2722704
Seim I, 2013, NAT COMMUN, V4, DOI 10.1038/ncomms3212
Selman C, 2012, TRENDS ECOL EVOL, V27, P570, DOI 10.1016/j.tree.2012.06.006
Sharma A, 2014, CARCINOGENESIS, V35, P1084, DOI 10.1093/carcin/bgu016
Shen YY, 2010, P NATL ACAD SCI USA, V107, P8666, DOI 10.1073/pnas.0912613107
Simmons NB, 2005, MAMMAL SPECIES WORLD, V1, P312, DOI [DOI 10.1111/J.1365-2656.2010.01742.X, 10.1093/acprof]
Smith-Vikos T, 2012, J CELL SCI, V125, P7, DOI 10.1242/jcs.099200
Tari AM, 1999, ONCOGENE, V18, P1325, DOI 10.1038/sj.onc.1202422
Wang L, 2014, CELL PHYSIOL BIOCHEM, V34, P413, DOI 10.1159/000363010
Zhang GJ, 2013, SCIENCE, V339, P456, DOI 10.1126/science.1230835
Zhao Z, 2012, DEV CELL, V22, P1001, DOI 10.1016/j.devcel.2011.12.027
NR 63
TC 45
Z9 48
U1 2
U2 33
PU BMC
PI LONDON
PA CAMPUS, 4 CRINAN ST, LONDON N1 9XW, ENGLAND
SN 1471-2164
J9 BMC GENOMICS
JI BMC Genomics
PD NOV 10
PY 2016
VL 17
AR 906
DI 10.1186/s12864-016-3227-8
PG 15
WC Biotechnology & Applied Microbiology; Genetics & Heredity

WE Science Citation Index Expanded (SCI-EXPANDED)
SC Biotechnology & Applied Microbiology; Genetics & Heredity
GA EB5PI
UT WOS:000387428100002
PM 27832764
OA Green Published, gold
DA 2024-10-21
ER

PT J
AU Gamage, AM
Zhu, F
Ahn, M
Foo, RJH
Hey, YY
Low, DHW
Mendenhall, IH
Dutertre, CA
Wang, LF
AF Gamage, Akshamal M.
Zhu, Feng
Ahn, Matae
Foo, Randy Jee Hiang
Hey, Ying Ying
Low, Dolyce H. W.
Mendenhall, Ian H.
Dutertre, Charles-Antoine
Wang, Lin-Fa
TI Immunophenotyping monocytes, macrophages and granulocytes in the Pteropodid bat <i>Eonycteris spelaea</i>
SO SCIENTIFIC REPORTS
LA English
DT Article
ID CLASS-II MOLECULES; BLOOD MONOCYTES; DENDRITIC CELLS; IN-VIVO; LUNG;
ESTABLISHMENT; INFORMATION; PERFORMANCE; POPULATION; PHENOTYPE
AB Bats are asymptomatic reservoir hosts for several highly pathogenic viruses. Understanding this enigmatic relationship between bats and emerging zoonotic viruses requires tools and approaches which enable the comparative study of bat immune cell populations and their functions. We show that bat genomes have a conservation of immune marker genes which delineate phagocyte populations in humans, while lacking key mouse surface markers such as Ly6C and Ly6G. Cross-reactive antibodies against CD44, CD11b, CD14, MHC II, and CD206 were multiplexed to characterize circulating monocytes, granulocytes, bone-marrow derived macrophages (BMDMs) and lung alveolar macrophages (AMs) in the cave nectar bat Eonycteris spelaea. Transcriptional profiling of bat monocytes and BMDMs identified additional markers - including MARCO, CD68, CD163, CD172 alpha, and CD88 - which can be used to further characterize bat myeloid populations. Bat cells often resembled their human counterparts when comparing immune parameters that are divergent between humans and mice, such as the expression patterns of certain immune cell markers. A genome-wide comparison of immune-related

genes also revealed a much closer phylogenetic relationship between bats and humans compared to rodents. Taken together, this study provides a set of tools and a comparative framework which will be important for unravelling viral disease tolerance mechanisms in bats.
C1 [Gamage, Akshamal M.; Zhu, Feng; Ahn, Matae; Foo, Randy Jee Hiang; Hey, Ying Ying; Low, Dolyce H. W.; Mendenhall, Ian H.; Dutertre, Charles-Antoine; Wang, Lin-Fa] Duke NUS Med Sch, Programme Emerging Infect Dis, Singapore, Singapore.
[Dutertre, Charles-Antoine] ASTAR, Singapore Immunol Network SIgN, Singapore, Singapore.
C3 National University of Singapore; Agency for Science Technology & Research (A*STAR); A*STAR - Singapore Immunology Network (SIgN)
RP Wang, LF (corresponding author), Duke NUS Med Sch, Programme Emerging Infect Dis, Singapore, Singapore.
EM linfa.wang@duke-nus.edu.sg
RI Dutertre, Charles Antoine/JRX-2224-2023; Zhu, Feng/GOH-1593-2022
OI Mendenhall, Ian/0000-0003-4250-6459; FOO, RANDY/0000-0001-9661-8662;
Wang, Lin-Fa/0000-0003-2752-0535; Dutertre, Charles-Antoine/0000-0001-7950-3652; Zhu, Feng/0000-0002-8131-1219; Ahn, Matae/0000-0003-2114-8250
FU National Research Foundation [NRF-CRP10-2012-05]
FX We thank Dr Edgar M. Pena, Ryan Cabo Maniquiz, Rommel E Yroy, Marvin Mataquel Taquiam and the rest of the veterinary team at SingHealth Experimental Medicine Centre (SEMC) for their invaluable expertise and assistance in establishing and maintaining the E. spelaea bat colony.
The project was funded by a National Research Foundation grant NRF-CRP10-2012-05 (L.-F.W.).
CR Ahn M, 2019, NAT MICROBIOL, V4, P789, DOI 10.1038/s41564-019-0371-3
Ahn M, 2016, SCI REP-UK, V6, DOI 10.1038/srep21722
Becht E, 2019, NAT BIOTECHNOL, V37, P38, DOI 10.1038/nbt.4314
BECKER D, 1992, J INVEST DERMATOL, V99, P545, DOI 10.1111/1523-1747.ep12667308
Bruhns P, 2012, BLOOD, V119, P5640, DOI 10.1182/blood-2012-01-380121
Cai YH, 2014, J IMMUNOL, V192, P2821, DOI 10.4049/jimmunol.1302269
Calisher CH, 2006, CLIN MICROBIOL REV, V19, P531, DOI 10.1128/CMR.00017-06
Chamorro S, 2005, IMMUNOLOGY, V114, P63, DOI 10.1111/j.1365-2567.2004.01994.x
Cowland JB, 2016, IMMUNOL REV, V273, P11, DOI 10.1111/imr.12440
Crameri G, 2009, PLOS ONE, V4, DOI 10.1371/journal.pone.0008266
Crinier A, 2018, IMMUNITY, V49, P971, DOI 10.1016/j.immuni.2018.09.009
Desch AN, 2016, AM J RESP CRIT CARE, V193, P614, DOI 10.1164/rccm.201507-1376OC

Eshar D, 2010, LAB ANIMAL, V39, P175, DOI 10.1038/laban0610-175
Fairbairn L, 2013, J IMMUNOL, V190, P6389, DOI 10.4049/jimmunol.1300365
Fairbairn L, 2011, J LEUKOCYTE BIOL, V89, P855, DOI 10.1189/jlb.1110607
Fortunati E, 2009, CLIN EXP IMMUNOL, V155, P559, DOI 10.1111/j.1365-2249.2008.03791.x
Francis C., 2008, EONYCTERIS SPELAEA
Gómez JMM, 2016, SCI REP-UK, V6, DOI 10.1038/srep37796
Guilliams M, 2015, FRONT IMMUNOL, V6, DOI 10.3389/fimmu.2015.00406
Guilliams M, 2014, NAT REV IMMUNOL, V14, P571, DOI 10.1038/nri3712
Guindon S, 2010, SYST BIOL, V59, P307, DOI 10.1093/sysbio/syq010
Helft J, 2015, IMMUNITY, V42, P1197, DOI 10.1016/j.immuni.2015.05.018
Hol J, 2010, J LEUKOCYTE BIOL, V87, P501, DOI 10.1189/jlb.0809532
Holling TM, 2004, HUM IMMUNOL, V65, P282, DOI 10.1016/j.humimm.2004.01.005
Hume DA, 2019, TRENDS IMMUNOL, V40, P98, DOI 10.1016/j.it.2018.11.007
Hussen J, 2017, FRONT IMMUNOL, V8, DOI 10.3389/fimmu.2017.01875
Joshi N, 2018, CELL IMMUNOL, V330, P86, DOI 10.1016/j.cellimm.2018.01.005
Kapetanovic R, 2012, J IMMUNOL, V188, P3382, DOI 10.4049/jimmunol.1102649
Katoh K, 2013, MOL BIOL EVOL, V30, P772, DOI 10.1093/molbev/mst010
Kunz TH, 2011, ANN NY ACAD SCI, V1223, P1, DOI 10.1111/j.1749-6632.2011.06004.x
Lee PY, 2013, J LEUKOCYTE BIOL, V94, P585, DOI 10.1189/jlb.0113014
Lim JJ, 2017, FRONT CELL INFECT MI, V7, DOI 10.3389/fcimb.2017.00191
Loughner CL, 2016, HUM GENOMICS, V10, DOI 10.1186/s40246-016-0074-2
Mayadas TN, 2014, ANNU REV PATHOL-MECH, V9, P181, DOI 10.1146/annurev-pathol-020712-164023
McInnes L., 2018, J OPEN SOURCE SOFTW
Melzer S, 2015, CYTOM PART B-CLIN CY, V88, P270, DOI 10.1002/cyto.b.21234
Meurens F, 2012, TRENDS MICROBIOL, V20, P50, DOI 10.1016/j.tim.2011.11.002
Misharin AV, 2017, J EXP MED, V214, P2387, DOI 10.1084/jem.20162152
OHTA T, 1993, P NATL ACAD SCI USA, V90, P10676, DOI 10.1073/pnas.90.22.10676
Parekh C, 2013, J CLIN IMMUNOL, V33, P711, DOI 10.1007/s10875-012-9844-3
Park SH, 2014, CYTOM PART B-CLIN CY, V86, P244, DOI [10.1002/cyto.b.21123, 10.1002/cytob.21123]
Pavlovich SS, 2018, CELL, V173, P1098, DOI 10.1016/j.cell.2018.03.070
Petkova SB, 2008, PHYSIOL GENOMICS, V34, P304, DOI 10.1152/

physiolgenomics.00185.2007
Reynolds G, 2015, FRONT IMMUNOL, V6, DOI 10.3389/fimmu.2015.00330
Serbina NV, 2008, ANNU REV IMMUNOL, V26, P421, DOI 10.1146/annurev.immunol.26.021607.090326
Surnmerfield A, 2015, MOL IMMUNOL, V66, P14, DOI 10.1016/j.molimm.2014.10.023
Teeling EC, 2002, P NATL ACAD SCI USA, V99, P1431, DOI 10.1073/pnas.022477199
Thoma-Uszynski S, 2001, SCIENCE, V291, P1544, DOI 10.1126/science.291.5508.1544
Thybert D, 2018, GENOME RES, V28, P448, DOI 10.1101/gr.234096.117
Tsagkogeorga G, 2013, CURR BIOL, V23, P2262, DOI 10.1016/j.cub.2013.09.014
Underhill DM, 2012, NAT REV IMMUNOL, V12, P492, DOI 10.1038/nri3244
Villani AC, 2017, SCIENCE, V356, DOI 10.1126/science.aah4573
Wang LF, 2019, CURR OPIN VIROL, V34, P79, DOI 10.1016/j.coviro.2018.12.007
WEINBERG JB, 1995, BLOOD, V86, P1184
Wen M, 2018, GIGASCIENCE, V7, DOI 10.1093/gigascience/giy116
Wernersson R, 2005, BMC GENOMICS, V6, DOI 10.1186/1471-2164-6-70
Xie JZ, 2018, CELL HOST MICROBE, V23, P297, DOI 10.1016/j.chom.2018.01.006
Zhou P, 2016, SCI REP-UK, V6, DOI 10.1038/srep38597
NR 58
TC 19
Z9 21
U1 1
U2 13
PU NATURE PORTFOLIO
PI BERLIN
PA HEIDELBERGER PLATZ 3, BERLIN, 14197, GERMANY
SN 2045-2322
J9 SCI REP-UK
JI Sci Rep
PD JAN 15
PY 2020
VL 10
IS 1
AR 309
DI 10.1038/s41598-019-57212-1
PG 16
WC Multidisciplinary Sciences
WE Science Citation Index Expanded (SCI-EXPANDED)
SC Science & Technology - Other Topics
GA MO3PI
UT WOS:000551441800018
PM 31941952
OA gold, Green Published
DA 2024-10-21
ER

PT J
AU Neely, BA

Janech, MG
Fenton, MB
Simmons, NB
Bland, AM
Becker, DJ
AF Neely, Benjamin A.
Janech, Michael G.
Fenton, M. Brock
Simmons, Nancy B.
Bland, Alison M.
Becker, Daniel J.
TI Surveying the Vampire Bat (<i>Desmodus rotundus</i>) Serum Proteome: A
Resource for Identifying Immunological Proteins and Detecting Pathogens
SO JOURNAL OF PROTEOME RESEARCH
LA English
DT Article
DE vampire bat; serum; data-independent acquisition; shotgun proteomics
ID GUANYLATE-BINDING PROTEINS; SALIVARY AGGLUTININ; PREY PREFERENCE; VIRAL
DIVERSITY; VIRUS; COMPLEMENT; EVOLUTION; RESERVOIR; RECEPTOR; ECOLOGY
AB Bats are increasingly studied as model systems for longevity and as natural hosts for some virulent viruses. Yet the ability to characterize immune mechanisms of viral tolerance and to quantify infection dynamics in wild bats is often limited by small sample volumes and few species-specific reagents. Here, we demonstrate how proteomics can overcome these limitations by using data-independent acquisition-based shotgun proteomics to survey the serum proteome of 17 vampire bats (Desmodus rotundus) from Belize. Using just 2 mu L of sample and relatively short separations of undepleted serum digests, we identified 361 proteins across 5 orders of magnitude. Levels of immunological proteins in vampire bat serum were then compared to human plasma via published databases. Of particular interest were antiviral and antibacterial components, circulating 20S proteasome complex and proteins involved in redox activity. Lastly, we used known virus proteomes to putatively identify Rh186 from Macacine herpesvirus 3 and ORF1a from Middle East respiratory syndrome-related coronavirus, indicating that mass spectrometry-based techniques show promise for pathogen detection. Overall, these results can be used to design targeted mass-spectrometry assays to quantify immunological markers and detect pathogens. More broadly, our findings also highlight the application of proteomics in advancing wildlife immunology and pathogen surveillance.
C1 [Janech, Michael G.; Bland, Alison M.] Hollings Marine Lab, Charleston, SC 29412 USA.
[Janech, Michael G.; Bland, Alison M.] Coll Charleston, Dept Biol, Charleston, SC 29424 USA.
[Fenton, M. Brock] Western Univ, Dept Biol, London, ON N6A 3K7, Canada.
[Simmons, Nancy B.] Amer Museum Nat Hist, Div Vertebrate Zool, Dept Mammal, New York, NY 10024 USA.

[Neely, Benjamin A.] NIST, Chem Sci Div, Charleston, SC 29412 USA.
[Becker, Daniel J.] Univ Oklahoma, Dept Biol, Norman, OK 73019 USA.
C3 College of Charleston; Western University (University of Western Ontario); American Museum of Natural History (AMNH); National Institute of Standards & Technology (NIST) - USA; University of Oklahoma System; University of Oklahoma - Norman
RP Neely, BA (corresponding author), NIST, Chem Sci Div, Charleston, SC 29412 USA.; Becker, DJ (corresponding author), Univ Oklahoma, Dept Biol, Norman, OK 73019 USA.
EM benjamin.neely@nist.gov; danbeck@ou.edu
RI Becker, Daniel/A-6466-2014; Neely, Benjamin/AGR-8303-2022
OI Neely, Benjamin/0000-0001-6120-7695; Janech, Michael/0000-0002-3202-4811
CR Allen LC, 2009, J COMP PHYSIOL B, V179, P315, DOI 10.1007/s00360-008-0315-3
Alspach E, 2019, CSH PERSPECT BIOL, V11, DOI 10.1101/cshperspect.a028480
Amman BR, 2015, J WILDLIFE DIS, V51, P113, DOI 10.7589/2014-08-198
Anderson NL, 2002, MOL CELL PROTEOMICS, V1, P845, DOI 10.1074/mcp.R200007-MCP200
[Anonymous], 1993, MAMMAL SPECIES WORLD
Anthony SJ, 2013, J GEN VIROL, V94, P1028, DOI 10.1099/vir.0.049759-0
Asano KM, 2016, VIROL J, V13, DOI 10.1186/s12985-016-0581-8
Baker ML, 2013, ZOONOSES PUBLIC HLTH, V60, P104, DOI 10.1111/j.1863-2378.2012.01528.x
Banerjee A, 2020, FRONT IMMUNOL, V11, DOI 10.3389/fimmu.2020.00026
Banyard AC, 2011, ADV VIRUS RES, V79, P239, DOI 10.1016/B978-0-12-387040-7.00012-3
Bartucci R, 2019, INT J MOL SCI, V20, DOI 10.3390/ijms20163891
Bayram HL, 2016, J PROTEOMICS, V135, P38, DOI 10.1016/j.jprot.2015.12.027
Becker DJ, 2021, TRANSBOUND EMERG DIS, V68, P870, DOI 10.1111/tbed.13754
Becker DJ, 2019, BIOL LETTERS, V15, DOI 10.1098/rsbl.2019.0423
Becker DJ, 2019, PHYSIOL BIOCHEM ZOOL, V92, P37, DOI 10.1086/701069
Becker DJ, 2018, PHILOS T R SOC B, V373, DOI 10.1098/rstb.2017.0089
Bergner LM, 2021, P NATL ACAD SCI USA, V118, DOI 10.1073/pnas.2019907118
Bergner LM, 2020, MOL ECOL, V29, P26, DOI 10.1111/mec.15250
Bergner LM, 2019, MOL ECOL RESOUR, V19, P128, DOI 10.1111/1755-0998.12946
Bezstarosti K, 2021, PLOS ONE, V16, DOI 10.1371/journal.pone.0259165
Bikker FJ, 2002, J BIOL CHEM, V277, P32109, DOI 10.1074/jbc.M203788200

Bobrowiec PED, 2015, J MAMMAL, V96, P54, DOI 10.1093/jmammal/gyu002
Brandao PE, 2008, BRAZ J INFECT DIS, V12, P466, DOI 10.1590/S1413-86702008000600003
Braun E, 2019, CELL REP, V27, P2092, DOI 10.1016/j.celrep.2019.04.063
Brook CE, 2020, ELIFE, V9, DOI 10.7554/eLife.48401
Brook CE, 2015, TRENDS MICROBIOL, V23, P172, DOI 10.1016/j.tim.2014.12.004
Bruderer R, 2019, MOL CELL PROTEOMICS, V18, P1242, DOI 10.1074/mcp.RA118.001288
Brunet-Rossinni AK, 2004, MECH AGEING DEV, V125, P11, DOI 10.1016/j.mad.2003.09.003
Camacho C, 2009, BMC BIOINFORMATICS, V10, DOI 10.1186/1471-2105-10-421
Chen LH, 2014, DATABASE-OXFORD, DOI 10.1093/database/bau021
CORREAGIRON P, 1972, SCIENCE, V175, P546, DOI 10.1126/science.175.4021.546
de Araujo J, 2017, VECTOR-BORNE ZOONOT, V17, P271, DOI 10.1089/vbz.2016.2051
Delpietro VA, 2002, MAMM BIOL, V67, P65, DOI 10.1078/1616-5047-00011
Deshmukh FK, 2019, BIOMOLECULES, V9, DOI 10.3390/biom9050190
Donaldson EF, 2010, J VIROL, V84, P13004, DOI 10.1128/JVI.01255-10
Drexler JF, 2012, NAT COMMUN, V3, DOI 10.1038/ncomms1796
Dunkelberger JR, 2010, CELL RES, V20, P34, DOI 10.1038/cr.2009.139
Condori-Condori RE, 2013, EMERG INFECT DIS, V19, P1463, DOI 10.3201/eid1909.130083
Escalera-Zamudio M, 2018, ECOHEALTH, V15, P132, DOI 10.1007/s10393-017-1297-y
Field KA, 2015, PLOS PATHOG, V11, DOI 10.1371/journal.ppat.1005168
Francischetti IMB, 2013, J PROTEOMICS, V82, P288, DOI 10.1016/j.jprot.2013.01.009
Gannon WL, 2007, J MAMMAL, V88, P809, DOI 10.1644/06-MAMM-F-185R1.1
Gerrard DL, 2017, MSPHERE, V2, DOI [10.1128/msphere.00245-17, 10.1128/mSphere.00245-17]
Geyer PE, 2019, EMBO MOL MED, V11, DOI 10.15252/emmm.201910427
Geyer PE, 2017, MOL SYST BIOL, V13, DOI 10.15252/msb.20156297
Gilbert AT, 2013, ECOHEALTH, V10, P298, DOI 10.1007/s10393-013-0856-0
Gouveia D, 2020, J PROTEOME RES, V19, P4407, DOI 10.1021/acs.jproteome.0c00535
Greenhall A.M., 2018, NATURAL HIST VAMPIRE
Guth S, 2019, PHILOS T R SOC B, V374, DOI 10.1098/rstb.2019.0296
Halpin K, 2011, AM J TROP MED HYG, V85, P946, DOI 10.4269/ajtmh.2011.10-0567
Hartshorn KL, 2006, BIOCHEM J, V393, P545, DOI 10.1042/BJ20050695
Hecht AM, 2015, SCI REP-UK, V5, DOI 10.1038/srep16604
Hecht-Höger AM, 2020, MOL ECOL, V29, P1745, DOI 10.1111/mec.15437
Heck M, 2020, J PROTEOME RES, V19, P3595, DOI 10.1021/

acs.jproteome.0c00448
Ihling C, 2020, J PROTEOME RES, V19, P4389, DOI 10.1021/acs.jproteome.0c00280
Ingala MR, 2018, MSPHERE, V3, DOI 10.1128/mSphere.00397-18
James S, 2020, INFECT GENET EVOL, V84, DOI 10.1016/j.meegid.2020.104367
Jebb D, 2020, NATURE, V583, P578, DOI 10.1038/s41586-020-2486-3
Jones Kate E., 2009, Ecology (Washington D C), V90, P2648, DOI 10.1890/08-1494.1
Kessler MK, 2018, ANN NY ACAD SCI, V1429, P78, DOI 10.1111/nyas.13910
Krapp C, 2016, CELL HOST MICROBE, V19, P504, DOI 10.1016/j.chom.2016.02.019
Kunz TH, 2011, ANN NY ACAD SCI, V1223, P1, DOI 10.1111/j.1749-6632.2011.06004.x
Lai Ruenn Chai, 2012, Int J Proteomics, V2012, P971907, DOI 10.1155/2012/971907
Lee AK, 2015, BMC GENOMICS, V16, DOI 10.1186/s12864-015-2124-x
Letko M, 2018, CELL REP, V24, P1730, DOI 10.1016/j.celrep.2018.07.045
Li WD, 2005, SCIENCE, V310, P676, DOI 10.1126/science.1118391
Ligtenberg AJM, 2010, INT J MOL SCI, V11, P5213, DOI 10.3390/ijms1112521
Lilley TM, 2019, OECOLOGIA, V191, P295, DOI 10.1007/s00442-019-04499-6
Lin CY, 2019, EBIOMEDICINE, V48, P425, DOI 10.1016/j.ebiom.2019.09.014
Mandl JN, 2018, FRONT IMMUNOL, V9, DOI 10.3389/fimmu.2018.02112
McCarthy MK, 2015, FRONT MICROBIOL, V6, DOI 10.3389/fmicb.2015.00021
Megger DA, 2017, FRONT IMMUNOL, V8, DOI 10.3389/fimmu.2017.01139
Meyer JG, 2017, EXPERT REV PROTEOMIC, V14, P419, DOI 10.1080/14789450.2017.1322904
Min-Oo G, 2010, EXP PARASITOL, V125, P315, DOI 10.1016/j.exppara.2010.02.009
Mollentze N, 2020, P NATL ACAD SCI USA, V117, P9423, DOI 10.1073/pnas.1919176117
Moore MS, 2011, PLOS ONE, V6, DOI 10.1371/journal.pone.0027430
Neely BA, 2015, PLOS ONE, V10, DOI 10.1371/journal.pone.0123295
Neely Benjamin A, 2013, Front Endocrinol (Lausanne), V4, P132, DOI 10.3389/fendo.2013.00132
Nordahl EA, 2004, P NATL ACAD SCI USA, V101, P16879, DOI 10.1073/pnas.0406678101
Olival KJ, 2017, NATURE, V546, P646, DOI 10.1038/nature22975
Orsburn BC, 2020, BIORXIV, DOI [10.1101/2020.03.08.980383, DOI 10.1101/2020.03.08.980383]
Parker SJ, 2015, MOL CELL PROTEOMICS, V14, P2800, DOI 10.1074/mcp.O114.042267
Patterson C., 2016, DEFORESTATION AGR IN
Perez-Riverol Y, 2019, NUCLEIC ACIDS RES, V47, pD442, DOI 10.1093/nar/gky1106
Pilosof S, 2014, MAMM BIOL, V79, P183, DOI 10.1016/j.mambio.2013.10.005
Pitari G, 2000, FEBS LETT, V483, P149, DOI 10.1016/

S0014-5793(00)02110-4
Plowright RK, 2016, PLOS NEGLECT TROP D, V10, DOI 10.1371/journal.pntd.0004796
Plowright RK, 2015, P ROY SOC B-BIOL SCI, V282, DOI 10.1098/rspb.2014.2124
Quan PL, 2013, P NATL ACAD SCI USA, V110, P8194, DOI 10.1073/pnas.1303037110
Ricklin D, 2010, NAT IMMUNOL, V11, P785, DOI 10.1038/ni.1923
Ripperger SP, 2019, CURR BIOL, V29, P4139, DOI 10.1016/j.cub.2019.10.024
Ruoss S, 2019, MAMM BIOL, V94, P120, DOI 10.1016/j.mambio.2018.05.010
Schneider MC, 2009, REV PANAM SALUD PUBL, V25, P260, DOI 10.1590/S1020-49892009000300010
Schountz T, 2017, FRONT IMMUNOL, V8, DOI 10.3389/fimmu.2017.01098
Schountz T, 2014, VIRUSES-BASEL, V6, P4880, DOI 10.3390/v6124880
Shi TJ, 2016, PROTEOMICS, V16, P2160, DOI 10.1002/pmic.201500449
Stepanian PM, 2018, GLOBAL CHANGE BIOL, V24, P3266, DOI 10.1111/gcb.14051
Streicker DG, 2016, J APPL ECOL, V53, P1280, DOI 10.1111/1365-2664.12690
Tian X, 2013, NATURE, V499, P346, DOI 10.1038/nature12234
Ting YS, 2015, MOL CELL PROTEOMICS, V14, P2301, DOI 10.1074/mcp.O114.047035
Tretina K, 2019, J EXP MED, V216, P482, DOI 10.1084/jem.20182031
Uhlén M, 2019, SCI SIGNAL, V12, DOI 10.1126/scisignal.aaz0274
Wilkinson GS, 2002, AGING CELL, V1, P124, DOI 10.1046/j.1474-9728.2002.00020.x
Williamson MM, 2000, J COMP PATHOL, V122, P201, DOI 10.1053/jcpa.1999.0364
Woon AP, 2020, PLOS PATHOG, V16, DOI 10.1371/journal.ppat.1008412
Wray AK, 2016, ECOHEALTH, V13, P761, DOI 10.1007/s10393-016-1183-z
Yao ZL, 2019, J PROTEOME RES, V18, P855, DOI 10.1021/acs.jproteome.8b00514
Yin QY, 2017, BBA-PROTEINS PROTEOM, V1865, P1004, DOI 10.1016/j.bbapap.2017.04.006
Young CCW, 2016, PLOS ONE, V11, DOI 10.1371/journal.pone.0149237
Zhang GJ, 2013, SCIENCE, V339, P456, DOI 10.1126/science.1230835
Zhou P, 2016, P NATL ACAD SCI USA, V113, P2696, DOI 10.1073/pnas.1518240113
NR 111
TC 14
Z9 14
U1 3
U2 10
PU AMER CHEMICAL SOC
PI WASHINGTON
PA 1155 16TH ST, NW, WASHINGTON, DC 20036 USA
SN 1535-3893
EI 1535-3907
J9 J PROTEOME RES
JI J. Proteome Res.
PD MAY 7

PY 2021
VL 20
IS 5
BP 2547
EP 2559
DI 10.1021/acs.jproteome.0c00995
EA APR 2021
PG 13
WC Biochemical Research Methods
WE Science Citation Index Expanded (SCI-EXPANDED)
SC Biochemistry & Molecular Biology
GA SA4KR
UT WOS:000649269600034
PM 33840197
OA Green Submitted, Green Accepted
DA 2024-10-21
ER

PT J
AU Heiker, LM
   Adams, RA
   Ramos, CV
AF Heiker, Laura M.
   Adams, Rick A.
   Ramos, Claire V.
TI Mercury Bioaccumulation in Two Species of Insectivorous Bats from Urban
   China: Influence of Species, Age, and Land Use Type
SO ARCHIVES OF ENVIRONMENTAL CONTAMINATION AND TOXICOLOGY
LA English
DT Article
ID BROWN BATS; MYOTIS-LUCIFUGUS; METAL CONCENTRATIONS; HEAVY-METALS;
   EXPOSURE; CHIROPTERA; FUR; METHYLMERCURY; EMISSIONS; ECOLOGY
AB Mercury (Hg) is a widespread, toxic pollutant, and China is the world's largest emitter. We investigated Hg concentrations of fur in Japanese pipistrelles (Pipistrellus abramus) and Chinese noctules (Nyctalus plancyi) from Chengdu, Sichuan Province, in relation to degree of urbanization. Bats were mist-netted in June and July 2013, and the fur was analyzed via atomic absorption. Statistical comparisons were made between ages, species, and site types with unpaired t tests and between Hg concentration and body condition with Spearman's rank correlations. Across sites, adult pipistrelles (n = 10) had significantly greater concentrations than adult noctules (n = 16). Adult N. plancyi (n = 16) had significantly greater concentrations than juvenile N. plancyi (n = 14). Contrary to our predictions, there was no significant difference in Hg values between urban (n = 3) and peri-urban (n = 6) locations for P. abramus. While small sample sizes precluded additional comparisons, the highest value (33 mg/kg) came from an adult female P. abramus in the agricultural area. The relationship between body condition and Hg concentration was insignificant. However, most pipistrelles (7/13) and no noctules (0/31) had concentrations > 10 mg/kg, a threshold associated with disruption of homeostatic control and mobility. All bats had concentrations > 0.2 mg/kg, which is

associated with compromised immunity. These are the first published records of contaminant concentrations from bats in China. For future studies, we recommend P. abramus as a regional bioindicator, longer term assessments of pre- and post-exposure effects, and simultaneous assessment of blood and fur Hg concentrations.
C1 [Heiker, Laura M.; Adams, Rick A.] Univ Northern Colorado, 501 20th St, Greeley, CO 80639 USA.
[Ramos, Claire V.] Univ Colorado, 2200 Bonforte Blvd, Pueblo, CO 81001 USA.
C3 University of Northern Colorado
RP Heiker, LM (corresponding author), Univ Northern Colorado, 501 20th St, Greeley, CO 80639 USA.
EM Lmheiker@gmail.com
OI Heiker, Laura/0000-0002-5744-6041
FU Bat Conservation International; Colorado Mountain Club; Explorers Club
Exploration Fund; University of Northern Colorado
FX This study was made possible by grants provided to L. M. Heiker from Bat
Conservation International, Colorado Mountain Club, The Explorers Club
Exploration Fund, Luo Lan and Wang Ji Shan, and the University of
Northern Colorado. We thank Tang Ya, Sarah Bexell, Sichuan University,
Chengdu Panda Base, and the Gao family for help with site access and
housing. Deng Rui, Du Li, Feng Ruixi, Kelsey Gonzales, and Hongyan Shi
provided field assistance. Thomas O'Shea and three anonymous reviewers
contributed helpful manuscript comments.
CR Adams RA, 2008, J ANIM ECOL, V77, P1115, DOI 10.1111/j.1365-2656.2008.01447.x
Åkerblom S, 2017, B ENVIRON CONTAM TOX, V99, P561, DOI 10.1007/s00128-017-2206-3
[Anonymous], 2011, BATS EVOLUTION CONSE
[Anonymous], EPA452R97009 OFF AIR
[Anonymous], BIOGEOGRAPHY MERCURY
[Anonymous], 2014, STESASERA352 UN POP
[Anonymous], 235 USFWS
[Anonymous], IUCN RED LIST THREAT
[Anonymous], CHENGD UN HAB REV TH
[Anonymous], 201118 BRI
[Anonymous], CHENGDOO CITY LIFE 2
[Anonymous], 2017, THESIS U NO COLORADO
[Anonymous], PIPISTRELLUS ABRAMUS
[Anonymous], SUN MOON RIS SET TAB
Barclay RMR, 2003, BAT ECOLOGY, P209
BATES P. J. J., 1997, BATS INDIAN SUBCONTI
Bayat S, 2014, ENVIRON INT, V63, P40, DOI 10.1016/j.envint.2013.10.009
Becker DJ, 2018, ENVIRON POLLUT, V233, P1076, DOI 10.1016/j.envpol.2017.10.010
Becker DJ, 2017, ROY SOC OPEN SCI, V4, DOI 10.1098/rsos.170073

Becker DJ, 2015, ECOL LETT, V18, P483, DOI 10.1111/ele.12428
Beernaert J, 2007, ENVIRON POLLUT, V145, P443, DOI 10.1016/j.envpol.2006.04.025
Nevado JJB, 2010, ENVIRON INT, V36, P593, DOI 10.1016/j.envint.2010.03.011
Boyles JG, 2011, SCIENCE, V332, P41, DOI 10.1126/science.1201366
BURTON GV, 1977, ENVIRON RES, V14, P30, DOI 10.1016/0013-9351(77)90063-9
CLARK DR, 1991, CALIF FISH GAME, V77, P61
Clark DR, 2001, ARCH ENVIRON CON TOX, V40, P537, DOI 10.1007/s002440010207
Custer CM, 2009, ENVIRON MONIT ASSESS, V153, P161, DOI 10.1007/s10661-008-0346-y
De Francisco N, 2003, AVIAN PATHOL, V32, P3, DOI 10.1080/0307945021000070660
Driscoll CT, 2007, BIOSCIENCE, V57, P17, DOI 10.1641/B570106
Driscoll CT, 2013, ENVIRON SCI TECHNOL, V47, P4967, DOI 10.1021/es305071v
Elbert RA, 1998, ENVIRON TOXICOL CHEM, V17, P210, DOI 10.1002/etc.5620170212
Evers DC, 2007, BIOSCIENCE, V57, P29, DOI 10.1641/B570107
Evers DC, 2005, ECOTOXICOLOGY, V14, P193, DOI 10.1007/s10646-004-6269-7
Fallacara DM, 2011, ENVIRON TOXICOL CHEM, V30, P1320, DOI 10.1002/etc.494
Flache L, 2016, SCI TOTAL ENVIRON, V547, P182, DOI 10.1016/j.scitotenv.2015.12.131
Flache L, 2015, J ENVIRON SCI, V31, P184, DOI 10.1016/j.jes.2014.12.010
Fraser EE, 2013, CAN J ZOOL, V91, P533, DOI 10.1139/cjz-2013-0072
FUNAKOSHI K, 1978, J FAC AGR KYUSHU U, V23, P95
Gannon WL, 2007, J MAMMAL, V88, P809, DOI 10.1644/06-MAMM-F-185R1.1
GELUSO KN, 1976, SCIENCE, V194, P184, DOI 10.1126/science.959845
Hawley DM, 2011, FUNCT ECOL, V25, P48, DOI 10.1111/j.1365-2435.2010.01753.x
Hayman DTS, 2013, ZOONOSES PUBLIC HLTH, V60, P2, DOI 10.1111/zph.12000
Hernout BV, 2016, CHEMOSPHERE, V147, P376, DOI 10.1016/j.chemosphere.2015.12.104
Hernout BV, 2015, ENVIRON TOXICOL CHEM, V34, P898, DOI 10.1002/etc.2871
Hickey MBC, 2001, B ENVIRON CONTAM TOX, V66, P699
Jones Gareth, 2009, Endangered Species Research, V8, P93, DOI 10.3354/esr00182
Kales SN, 2005, ISS TOXICOL, P125
Kannan K, 2010, CHEMOSPHERE, V80, P613, DOI 10.1016/j.chemosphere.2010.04.060
Karouna-Renier NK, 2014, ECOTOXICOLOGY, V23, P1419, DOI 10.1007/s10646-014-1284-9
KLEIBER MAX, 1932, HILGARDIA, V6, P315
Kunz TH, 2011, ANN NY ACAD SCI, V1223, P1, DOI 10.1111/j.1749-6632.2011.06004.x
KURTA A, 1989, PHYSIOL ZOOL, V62, P804, DOI 10.1086/

physzool.62.3.30157928
Lee YF, 2005, ZOOL STUD, V44, P95
Lee YF, 2005, J MAMMAL, V86, P67, DOI 10.1644/1545-1542(2005)086<0067:DVOBFB>2.0.CO;2
Lisón F, 2017, ENVIRON SCI POLLUT R, V24, P5497, DOI 10.1007/s11356-016-8271-z
Little ME, 2015, ENVIRON SCI TECHNOL, V49, P2059, DOI 10.1021/es5050375
McKinney ML, 2002, BIOSCIENCE, V52, P883, DOI 10.1641/0006-3568(2002)052[0883:UBAC]2.0.CO;2
MIURA T, 1978, B ENVIRON CONTAM TOX, V20, P696, DOI 10.1007/BF01683587
Moreno-Brush M, 2018, ECOTOXICOLOGY, V27, P45, DOI 10.1007/s10646-017-1869-1
Nam DH, 2012, ECOTOXICOLOGY, V21, P1094, DOI 10.1007/s10646-012-0864-9
NORBERG UM, 1987, PHILOS T R SOC B, V316, P337
Osborne C.E., 2011, Mercury contamination within terrestrial ecosystems in New England and Mid-Atlantic states: profiles of soil, invertebrates, songbirds, and bats
Pacyna EG, 2010, ATMOS ENVIRON, V44, P2487, DOI 10.1016/j.atmosenv.2009.06.009
Pearce RD, 2008, ACTA CHIROPTEROL, V10, P153, DOI 10.3161/150811008X331171
Pikula J, 2010, ENVIRON TOXICOL CHEM, V29, P501, DOI 10.1002/etc.80
Raats M. M., 1992, Food Quality and Preference, V3, P89, DOI 10.1016/0950-3293(91)90028-D
Reinhold J. O., 1999, Aquatic Ecology, V33, P363, DOI 10.1023/A:1009958028204
Russo D, 2015, MAMM BIOL, V80, P205, DOI 10.1016/j.mambio.2014.10.003
Scheulhammer AM, 2007, AMBIO, V36, P12, DOI 10.1579/0044-7447(2007)36[12:EOEMOT]2.0.CO;2
Schneider A, 2005, ENVIRON PLANN B, V32, P323, DOI 10.1068/b31142
Shi Hongyan, 2001, Acta Theriologica Sinica, V21, P210
Streets DG, 2009, MERCURY FATE AND TRANSPORT IN THE GLOBAL ATMOSPHERE, P51, DOI 10.1007/978-0-387-93958-2_2
Streets DG, 2005, ATMOS ENVIRON, V39, P7789, DOI 10.1016/j.atmosenv.2005.08.029
Sutton R.H., 1987, Australian Mammalogy, V10, P125
Syaripuddin K, 2014, ECOTOXICOLOGY, V23, P1164, DOI 10.1007/s10646-014-1258-y
Tian HZ, 2015, ATMOS CHEM PHYS, V15, P10127, DOI 10.5194/acp-15-10127-2015
Tian LX, 2015, PLOS ONE, V10, DOI 10.1371/journal.pone.0123205
TIMMERMANS KR, 1989, ENVIRON POLLUT, V62, P73, DOI 10.1016/0269-7491(89)90097-3
Wada H, 2010, ECOTOXICOLOGY, V19, P1277, DOI 10.1007/s10646-010-0513-0
Walker LA, 2007, ENVIRON POLLUT, V148, P483, DOI 10.1016/j.envpol.2006.12.006
Wang GY, 2017, ENVIRON SCI POLLUT R, V24, P16618, DOI 10.1007/s11356-017-9288-7

Webb PI, 1995, SYM ZOOL S, P203
Wilkinson GS, 2002, AGING CELL, V1, P124, DOI 10.1046/j.1474-9728.2002.00020.x
Wu QR, 2017, ATMOS CHEM PHYS, V17, P10423, DOI 10.5194/acp-17-10423-2017
Yates DE, 2014, ECOTOXICOLOGY, V23, P45, DOI 10.1007/s10646-013-1150-1
Zhang H, 2010, ENVIRON HEALTH PERSP, V118, P1183, DOI 10.1289/ehp.1001915
Zukal J, 2015, MAMM BIOL, V80, P220, DOI 10.1016/j.mambio.2015.01.001
NR 87
TC 10
Z9 11
U1 1
U2 32
PU SPRINGER
PI NEW YORK
PA ONE NEW YORK PLAZA, SUITE 4600, NEW YORK, NY, UNITED STATES
SN 0090-4341
EI 1432-0703
J9 ARCH ENVIRON CON TOX
JI Arch. Environ. Contam. Toxicol.
PD NOV
PY 2018
VL 75
IS 4
BP 585
EP 593
DI 10.1007/s00244-018-0547-5
PG 9
WC Environmental Sciences; Toxicology
WE Science Citation Index Expanded (SCI-EXPANDED)
SC Environmental Sciences & Ecology; Toxicology
GA GV8TU
UT WOS:000446421100009
PM 30027306
DA 2024-10-21
ER

PT J
AU MONTANOHIROSE, JA
LAFAGE, M
WEBER, P
BADRANE, H
TORDO, N
LAFON, M
AF MONTANOHIROSE, JA
LAFAGE, M
WEBER, P
BADRANE, H
TORDO, N
LAFON, M
TI PROTECTIVE ACTIVITY OF A MURINE MONOCLONAL-ANTIBODY AGAINST

EUROPEAN BAT
   LYSSAVIRUS-1 (EBL1) INFECTION IN MICE
SO VACCINE
LA English
DT Article
DE EUROPEAN BAT LYSSAVIRUS; MONOCLONAL ANTIBODIES; PASSIVE IMMUNIZATION;
   RABIES VIRUS; NEUTRALIZATION
ID RABIES VIRUS GLYCOPROTEIN; ANTIGENIC VARIANTS; POSTEXPOSURE TREATMENT;
   IMMUNE GLOBULIN; SITES; EPIDEMIOLOGY; DUVENHAGE; VACCINES; PROTEIN; CELL
AB A mouse model was designed to test in vivo the efficacy of rabies immune globulins and specific neutralizing monoclonal antibodies to prevent European bat lyssavirus 1 infection. Human or equine rabies immune globulins previously found to contain variable amounts of neutralizing bat lyssavirus crossreactive antibodies were passively transferred to mice receiving intramuscularly a lethal dose of bat lyssavirus type 1. Immune globulins did not protect mice well against bat lyssavirus 1 whereas they reduced the mortality caused by rabies virus. In contrast, mice inoculated with bat lyssavirus 1 or rabies virus survived when passively immunized with bat lyssavirus 1 specific monoclonal antibody (mAb 8-2). This monoclonal antibody, an IgG2a, recognized an epitope located in the antigenic site IIa of rabies glycoprotein. A mutation replacing the lysine 198 by glutamate in a rabies variant abrogated sensitivity to this neutralizing antibody. Because of its broad neutralizing spectrum against wild virus isolates, including European bat lyssaviruses, this monoclonal antibody should be a good candidate for rabies immune globulin replacement. It could improve efficacy of rabies vaccination, used either alone or in conjunction with human rabies immune globulins or monoclonal antibody cocktail to supplement their lack of crossreactivity to European bat lyssavirus 1.
C1 INST PASTEUR,UNITE RAGE,25 RUE DR ROUX,F-75724 PARIS 15,FRANCE.
C3 Pasteur Network; Universite Paris Cite; Institut Pasteur Paris
RI TORDO, Noël/GQQ-9089-2022
OI Lafon, Monique/0000-0002-4423-7996
CR BALTAZARD M, 1955, Bull World Health Organ, V13, P747
   BENMANSOUR A, 1991, J VIROL, V65, P4198, DOI 10.1128/JVI.65.8.4198-4203.1991
   BLANCOU J, 1991, VACCINE, V9, P432, DOI 10.1016/0264-410X(91)90131-O
   BOURHY H, 1992, J CLIN MICROBIOL, V30, P2419, DOI 10.1128/JCM.30.9.2419-2426.1992
   BUNSCHOTEN H, 1989, J GEN VIROL, V70, P291, DOI 10.1099/0022-1317-70-2-291
   CELIS E, 1988, J VIROL, V62, P3128, DOI 10.1128/JVI.62.9.3128-3134.1988
   DIETZSCHOLD B, 1987, J INFECT DIS, V156, P815, DOI 10.1093/infdis/156.5.815
   DIETZSCHOLD B, 1988, REV INFECT DIS, V10, pS785
   DIETZSCHOLD B, 1990, J VIROL, V64, P3087, DOI 10.1128/JVI.64.6.3087-3090.1990
   ENSSLE K, 1991, HYBRIDOMA, V10, P547, DOI 10.1089/hyb.1991.10.547

HERZOG M, 1991, CLIN EXP IMMUNOL, V85, P224, DOI 10.1111/j.1365-2249.1991.tb05709.x
KAPPELER A, 1989, WHO RABIES B, V13, P12
KING A, 1990, VET MICROBIOL, V23, P165, DOI 10.1016/0378-1135(90)90146-M
KOPROWSKI H, 1954, J IMMUNOL, V72, P85
LAFON M, 1986, LANCET, V2, P515
LAFON M, 1985, J GEN VIROL, V66, P2125, DOI 10.1099/0022-1317-66-10-2125
LAFON M, 1990, J GEN VIROL, V71, P1689, DOI 10.1099/0022-1317-71-8-1689
LAFON M, 1983, J GEN VIROL, V64, P843, DOI 10.1099/0022-1317-64-4-843
LAFON M, 1988, VACCINE, V6, P362, DOI 10.1016/0264-410X(88)90184-3
LIBEAU G, 1984, DEV BIOLOGICALS, V57, P213
LIBEAU G, 1984, REV ELEV MED VET PAY, V374, P383
LORENZ RJ, WHO MONOGR SER, V23, P321
LUMIO J, 1986, LANCET, V1, P378
PETROVIC M, 1987, RABIES B EUROPE, V4, P16
PREHAUD C, 1988, J VIROL, V62, P1
SACRAMENTO D, 1991, MOL CELL PROBE, V5, P229, DOI 10.1016/0890-8508(91)90045-L
SATO M, 1977, ARCH VIROL, V53, P269, DOI 10.1007/BF01314672
SCHNEIDER LG, 1982, COMP IMMUNOL MICROB, V5, P101, DOI 10.1016/0147-9571(82)90021-2
SCHNEIDER LG, 1986, RABIES B EUROPE, V2, P9
SCHUMACHER CL, 1989, J CLIN INVEST, V84, P971, DOI 10.1172/JCI114260
SEARS HF, 1984, J BIOL RESP MODIF, V3, P138
SELIMOV MA, 1989, ACTA VIROL, V33, P542
SMITH JS, WHO MONOGR SER, V23, P354
TORDO N, 1986, P NATL ACAD SCI USA, V83, P3914, DOI 10.1073/pnas.83.11.3914
WIKTOR TJ, 1980, J EXP MED, V152, P99, DOI 10.1084/jem.152.1.99
WIKTOR TJ, 1978, P NATL ACAD SCI USA, V75, P3938, DOI 10.1073/pnas.75.8.3938
WILDE H, 1989, VACCINE, V7, P10, DOI 10.1016/0264-410X(89)90003-0
WUNNER WH, 1985, VIROLOGY, V140, P1, DOI 10.1016/0042-6822(85)90440-4
ZALEN E, 1979, J BIOL STAND, V7, P213
NR 39
TC 22
Z9 26
U1 0
U2 2
PU BUTTERWORTH-HEINEMANN LTD
PI OXFORD
PA THE BOULEVARD, LANGFORD LANE, KIDLINGTON, OXFORD, OXON, ENGLAND OX5 1GB
SN 0264-410X
J9 VACCINE
JI Vaccine
PD SEP

PY 1993
VL 11
IS 12
BP 1259
EP 1266
DI 10.1016/0264-410X(93)90052-Y
PG 8
WC Immunology; Medicine, Research & Experimental
WE Science Citation Index Expanded (SCI-EXPANDED)
SC Immunology; Research & Experimental Medicine
GA LZ908
UT WOS:A1993LZ90800014
PM 8256507
DA 2024-10-21
ER

PT J
AU Costantini, D
   Czirják, GA
   Bustamante, P
   Bumrungsri, S
   Voigt, CC
AF Costantini, David
   Czirjak, Gabor A.
   Bustamante, Paco
   Bumrungsri, Sara
   Voigt, Christian C.
TI Impacts of land use on an insectivorous tropical bat: The importance of
   mercury, physio-immunology and trophic position
SO SCIENCE OF THE TOTAL ENVIRONMENT
LA English
DT Article
DE Agriculture; Antioxidants; Deforestation; Landscape planning; Oxidative
   stress; Rice; Stable isotopes
ID ORYZA-SATIVA-L.; OXIDATIVE STRESS; METHYLMERCURY; BIOACCUMULATION; DIET;
   RISK; BUCHANNAN; ISOTOPES; TISSUES; FOREST
AB Deforestation, agricultural intensification, and habitat homogenization are critical threats to biodiversity in Southeast Asia. Limited information is available on the trophic and physiological responses of tropical animals to these environmental changes. The wrinkle-lipped free-tailed bat Chaerephon plicatus is a cave roosting species that is experiencing population declines across Southeast Asia, where landscapes have been drastically modified. In our study site in central Thailand, we tested the hypothesis that wrinkle-lipped free-tailed bats living in landscapes that contrast in heterogeneity and land-use differed in mercury contamination, trophic position and physio-immunological status. Bats from less heterogeneous landscapes (dominated by rice crops, absence of large forest patches) occupied a lower trophic position than conspecifics from more heterogeneous landscapes (including large forest patches). Additionally, bats from these habitats had

lower concentrations of mercury in erythrocytes, lower body mass,
higher antioxidant superoxide dismutase (SOD), lower antioxidant
glutathione peroxidase (GPx) and lower values of the GPx/SOD ratio
than bats from more heterogeneous landscapes. Individual bat mercury
concentrations were positively correlated with body mass and two
immune markers (lysozyme and immunoglobulin) but were negatively
correlated with plasma non-enzymatic antioxidant capacity. Our
results suggest various links among landscape heterogeneity, mercury
exposure/accumulation, and health status of wildlife in Southeast
Asian countries. (C) 2019 Elsevier B.V. All rights reserved.
C1 [Costantini, David] Sorbonne Univ, Museum Natl Hist Nat, UMR
7221, CNRS, 7 Rue Cuvier, F-75005 Paris, France.
[Costantini, David] Univ Antwerp, Dept Biol, Univ Pl 1, B-2610
Antwerp, Belgium.
[Costantini, David; Voigt, Christian C.] Leibniz Inst Zoo &
Wildlife Res, Dept Evolutionary Ecol, Alfred Kowalke Str 17, D-10315
Berlin, Germany.
[Czirjak, Gabor A.] Leibniz Inst Zoo & Wildlife Res, Dept
Wildlife Dis, Alfred Kowalke Str 17, D-10315 Berlin, Germany.
[Bustamante, Paco] Univ La Rochelle, UMR 7266, CNRS, Littoral
Environm & Soc, 2 Rue Olympe de Gouges, F-17000 La Rochelle, France.
[Bumrungsri, Sara] Prince Songkla Univ, Fac Sci, Dept Biol, Hat
Yai, Songkhla, Thailand.
[Voigt, Christian C.] Free Univ Berlin, Inst Biol, Takustr 6,
D-14195 Berlin, Germany.
C3 Sorbonne Universite; Centre National de la Recherche Scientifique
(CNRS); CNRS - National Institute for Biology (INSB); Museum
National
d'Histoire Naturelle (MNHN); University of Antwerp; Leibniz
Institut fur
Zoo und Wildtierforschung; Leibniz Institut fur Zoo und
Wildtierforschung; La Rochelle Universite; Centre National de la
Recherche Scientifique (CNRS); CNRS - Institute of Ecology &
Environment
(INEE); Prince of Songkla University; Free University of Berlin
RP Costantini, D (corresponding author), Sorbonne Univ, Museum Natl
Hist Nat, UMR 7221, CNRS, 7 Rue Cuvier, F-75005 Paris, France.
EM david.costantini@mnhn.fr
RI Czirják, Gábor/F-5440-2011; Costantini, David/C-6006-2013;
Bustamante,
Paco/G-5833-2011
OI Czirjak, Gabor Arpad/0000-0001-9488-0069; Bustamante,
Paco/0000-0003-3877-9390; Costantini, David/0000-0002-8140-8790
FU Alexander von Humboldt Foundation; Leibniz Institute for Zoo and
Wildlife Research; CPER (Contrat de Projet Etat-Region); FEDER
(European
Regional Development Fund); IUF (Institut Universitaire de
France)
FX This work was supported by the Alexander von Humboldt Foundation
and
funds from Leibniz Institute for Zoo and Wildlife Research. We
are
grateful to the platform "Analyses Elementaires" of LIENSs for
the

access to their analytical facilities. We are also grateful to the CPER
(Contrat de Projet Etat-Region) and the FEDER (European Regional
Development Fund) for funding theAMA. The IUF (Institut Universitaire de
France) is also acknowledged for its support to PB as a Senior Member.
The authors wish to thank Katja Pohle and Yvonne Klaar for technical
assistance in the lab, students for assistance in fieldwork, Daniel J.
Becker for kindly correcting our English, the editor and two reviewers
for providing valuable and constructive comments on our manuscript.
CR [Anonymous], 2009, FORAGING HABITAT USE
[Anonymous], INTEGR COMP BIOL
[Anonymous], 2018, Epinephelus fuscoguttatus, DOI DOI 10.2305/IUCN.UK.2018-2.RLTS.T44673A100468078.EN
[Anonymous], 2014, Global forest resources assessment 2015-country report: Turkey
Barbosa AC, 2003, ARCH ENVIRON CON TOX, V45, P235, DOI 10.1007/s00244-003-0207-1
Beaulieu M, 2014, CONSERV PHYSIOL, V2, DOI 10.1093/conphys/cou014
Becker DJ, 2018, PHILOS T R SOC B, V373, DOI 10.1098/rstb.2017.0089
Becker DJ, 2018, ENVIRON POLLUT, V233, P1076, DOI 10.1016/j.envpol.2017.10.010
Becker DJ, 2017, ROY SOC OPEN SCI, V4, DOI 10.1098/rsos.170073
Berglund Marika, 2005, Environ Health, V4, P20, DOI 10.1186/1476-069X-4-20
Böhm SM, 2011, PLOS ONE, V6, DOI 10.1371/journal.pone.0017857
Boyles JG, 2011, SCIENCE, V332, P41, DOI 10.1126/science.1201366
Bustamante P, 2008, MAR ENVIRON RES, V66, P278, DOI 10.1016/j.marenvres.2008.04.003
Chouvelon T, 2009, ENVIRON POLLUT, V157, P331, DOI 10.1016/j.envpol.2008.06.027
Cohen J, 2013, Statistical power analysis for the behavioral sciences
COOK RD, 1977, TECHNOMETRICS, V19, P15, DOI 10.2307/1268249
Costantini D, 2017, CONSERV PHYSIOL, V5, DOI 10.1093/conphys/cox069
Costantini D, 2012, J EXP BIOL, V215, P374, DOI 10.1242/jeb.062034
Demas GE, 2011, J ANIM ECOL, V80, P710, DOI 10.1111/j.1365-2656.2011.01813.x
DENIRO MJ, 1978, GEOCHIM COSMOCHIM AC, V42, P495, DOI 10.1016/0016-7037(78)90199-0
DENIRO MJ, 1981, GEOCHIM COSMOCHIM AC, V45, P341, DOI 10.1016/0016-7037(81)90244-1
Driscoll CT, 2007, BIOSCIENCE, V57, P17, DOI 10.1641/B570106
Eagles-Smith CA, 2008, ENVIRON TOXICOL CHEM, V27, P2136, DOI 10.1897/08-038.1
Eagles-Smith CA, 2018, AMBIO, V47, P170, DOI 10.1007/

s13280-017-1011-x
Feng C, 2016, ELEMENTA-SCI ANTHROP, V4, P1, DOI 10.12952/journal.elementa.000087
Ghanem SJ, 2012, ADV STUD BEHAV, V44, P279, DOI 10.1016/B978-0-12-394288-3.00007-1
Heinrich SK, 2017, SCI REP-UK, V7, DOI 10.1038/srep44837
Horvat M, 2003, SCI TOTAL ENVIRON, V304, P231, DOI 10.1016/S0048-9697(02)00572-7
Jayawardena UA, 2017, ENVIRON TOXICOL CHEM, V36, P2855, DOI 10.1002/etc.3848
Kalka MB, 2008, SCIENCE, V320, P71, DOI 10.1126/science.1153352
Karouna-Renier NK, 2014, ECOTOXICOLOGY, V23, P1419, DOI 10.1007/s10646-014-1284-9
Karp DS, 2014, ECOLOGY, V95, P1065, DOI 10.1890/13-1012.1
KERSHAW TG, 1980, ARCH ENVIRON HEALTH, V35, P28
Krisnayanti BD, 2012, J ENVIRON MONITOR, V14, P2598, DOI 10.1039/c2em30515a
Kumar A, 2018, ENVIRON POLLUT, V236, P862, DOI 10.1016/j.envpol.2018.01.109
Kunz TH, 2011, ANN NY ACAD SCI, V1223, P1, DOI 10.1111/j.1749-6632.2011.06004.x
Leelapaibul W, 2005, ACTA CHIROPTEROL, V7, P111, DOI 10.3161/1733-5329(2005)7[111:DOWFBT]2.0.CO;2
Maas B, 2013, ECOL LETT, V16, P1480, DOI 10.1111/ele.12194
Madliger CL, 2018, CONSERV PHYSIOL, V6, DOI 10.1093/conphys/coy029
Magos L., 1987, The Toxicity of Methyl Mercury, P24
Martin LB, 2011, FUNCT ECOL, V25, P1, DOI 10.1111/j.1365-2435.2010.01820.x
Meng B, 2011, ENVIRON SCI TECHNOL, V45, P2711, DOI 10.1021/es103384v
Meng B, 2010, J AGR FOOD CHEM, V58, P4951, DOI 10.1021/jf904557x
Park EM, 2007, FREE RADICAL BIO MED, V42, P280, DOI 10.1016/j.freeradbiomed.2006.10.044
Raats M. M., 1992, Food Quality and Preference, V3, P89, DOI 10.1016/0950-3293(91)90028-D
Ruadreo N, 2018, ACTA CHIROPTEROL, V20, P329, DOI 10.3161/15081109ACC2018.20.2.005
Ruoss S, 2019, MAMM BIOL, V94, P120, DOI 10.1016/j.mambio.2018.05.010
Sakamoto M, 2002, ENVIRON RES, V90, P185, DOI 10.1016/S0013-9351(02)00011-7
Schneeberger K, 2014, PLOS ONE, V9, DOI 10.1371/journal.pone.0108268
Schneeberger K, 2014, NATURWISSENSCHAFTEN, V101, P285, DOI 10.1007/s00114-014-1155-5
Schneeberger K, 2013, J EXP BIOL, V216, P4514, DOI 10.1242/jeb.090837
Schneeberger K, 2013, PLOS ONE, V8, DOI 10.1371/journal.pone.0054023
Seltmann A, 2017, CONSERV PHYSIOL, V5, DOI 10.1093/conphys/cox020
SKERFVING S, 1988, B ENVIRON CONTAM TOX, V41, P475, DOI 10.1007/BF02020989
Srilopan S, 2018, ACTA CHIROPTEROL, V20, P207, DOI

10.3161/15081109ACC2018.20.1.016
Struebig MJ, 2008, BIOL CONSERV, V141, P2112, DOI 10.1016/j.biocon.2008.06.009
Struebig MJ, 2009, BIOL CONSERV, V142, P2089, DOI 10.1016/j.biocon.2009.04.005
Stubner S, 1998, SYST APPL MICROBIOL, V21, P569, DOI 10.1016/S0723-2020(98)80069-6
Syaripuddin K, 2014, ECOTOXICOLOGY, V23, P1164, DOI 10.1007/s10646-014-1258-y
Tan SW, 2009, CRIT REV TOXICOL, V39, P228, DOI 10.1080/10408440802233259
Tang ZY, 2018, ECOTOX ENVIRON SAFE, V150, P116, DOI 10.1016/j.ecoenv.2017.12.021
Treitler JT, 2016, ECOL EVOL, V6, P4289, DOI 10.1002/ece3.2160
Viechtbauer W, 2010, J STAT SOFTW, V36, P1, DOI 10.18637/jss.v036.i03
Voigt CC, 2006, J MAMMAL, V87, P1, DOI 10.1644/05-MAMM-F-276R1.1
Voigt CC., 2016, BATS ANTHROPOCENE CO
Wada H, 2010, ECOTOXICOLOGY, V19, P1277, DOI 10.1007/s10646-010-0513-0
Waiyasusri K, 2016, ENVIRON EARTH SCI, V75, DOI 10.1007/s12665-016-5322-1
Wanger TC, 2014, BIOL CONSERV, V171, P220, DOI 10.1016/j.biocon.2014.01.030
Weise P, 2017, PEERJ, V5, DOI 10.7717/peerj.3570
Whitney MC, 2018, REV ENVIRON CONTAM T, V244, P113, DOI 10.1007/398_2017_4
Williams-Guillén K, 2008, SCIENCE, V320, P70, DOI 10.1126/science.1152944
Yates DE, 2014, ECOTOXICOLOGY, V23, P45, DOI 10.1007/s10646-013-1150-1
NR 72
TC 15
Z9 17
U1 6
U2 34
PU ELSEVIER
PI AMSTERDAM
PA RADARWEG 29, 1043 NX AMSTERDAM, NETHERLANDS
SN 0048-9697
EI 1879-1026
J9 SCI TOTAL ENVIRON
JI Sci. Total Environ.
PD JUN 25
PY 2019
VL 671
BP 1077
EP 1085
DI 10.1016/j.scitotenv.2019.03.398
PG 9
WC Environmental Sciences
WE Science Citation Index Expanded (SCI-EXPANDED)
SC Environmental Sciences & Ecology
GA HV6JU

UT WOS:000466090500109
OA Bronze, Green Submitted
DA 2024-10-21
ER

PT J
AU Wu, H
Zhu, JQ
Xu, XF
Xing, H
Wang, MD
Liang, L
Li, C
Jia, HD
Shen, F
Huang, DS
Yang, T
AF Wu, Han
Zhu, Jia-Qi
Xu, Xin-Fei
Xing, Hao
Wang, Ming-Da
Liang, Lei
Li, Chao
Jia, Hang-Dong
Shen, Feng
Huang, Dong-Sheng
Yang, Tian
TI Biointerfacing Antagonizing T-Cell Inhibitory Nanoparticles Potentiate
Hepatocellular Carcinoma Checkpoint Blockade Therapy
SO SMALL
LA English
DT Article
DE biointerfacing nanoparticles; checkpoint blockade; hepatocellular carcinoma; starvation therapy; tumor targeting
ID CANCER; DURVALUMAB; IMMUNITY; RECEPTOR
AB Hepatocellular carcinoma (HCC) is one of the most fatal malignancies with few effective treatment options all around the world. The efficacy of the arisen immune checkpoint therapy is still uncertain due to local immunosuppression. In order to further overcome T cell suppression in the tumor immune microenvironment while promoting the immune response of antigen-presenting cells, a biointerfacing antagonizing T-cell inhibitory nanoparticles (BAT NPs) has been developed by cloaking platelet membrane on the PLGA microsphere surface to load T-cell immunoglobulin domain and mucin domain-3 antibodies (anti-TIM-3) as well as PD-L1. Notably, in addition to activating the proliferation and migration of T cells, the contained anti-TIM-3 can cooperate with PD-L1 checkpoint blockade to exert therapeutic effects. Furthermore, the components of BAT NPs like anti-TIM-3 and platelet can act together for collagen deposition in tumor starvation treatment. Thus, a novel targeting therapeutic strategy that can effectively reverse the immune-inhibiting microenvironment is effectively applied to PD-L1

checkpoint combination therapy. Such therapeutic effect can
subsequently activate the effector T lymphocytes and antigen
presentation of dendritic cells as well as the polarization of M1-
type macrophages. Last, the study presented the synergistic effect
of immune therapeutic adjuvants and BAT NPs components in achieving
tumor inhibition and prolonging tumor-burden survival.
C1 [Wu, Han; Zhu, Jia-Qi; Liang, Lei; Jia, Hang-Dong; Huang, Dong-
Sheng; Yang, Tian] Hangzhou Med Coll, Key Lab Tumor Mol Diag &
Individualized Med Zheji, Zhejiang Prov Peoples Hosp, Peoples Hosp,
Hangzhou 310014, Peoples R China.
[Wu, Han; Zhu, Jia-Qi; Liang, Lei; Jia, Hang-Dong; Huang, Dong-
Sheng; Yang, Tian] Hangzhou Med Coll, Sch Clin Med, Hangzhou 310014,
Zhejiang, Peoples R China.
[Wu, Han; Xu, Xin-Fei; Xing, Hao; Wang, Ming-Da; Li, Chao; Shen,
Feng; Yang, Tian] Second Mil Med Univ, Eastern Hepatobiliary Surg
Hosp, Dept Hepatobiliary Surg, Naval Med Univ, Shanghai 200438,
Peoples R China.
[Zhu, Jia-Qi; Liang, Lei; Yang, Tian] Hangzhou Med Coll, Dept
Hepatobiliary Pancreat & Minimal Invas Surg, Peoples Hosp, Zhejiang
Prov Peoples Hosp, Hangzhou 310014, Zhejiang, Peoples R China.
C3 Hangzhou Medical College; Zhejiang Provincial People's Hospital;
Hangzhou Medical College; Naval Medical University; Hangzhou
Medical
College; Zhejiang Provincial People's Hospital
RP Huang, DS; Yang, T (corresponding author), Hangzhou Med Coll, Key
Lab Tumor Mol Diag & Individualized Med Zheji, Zhejiang Prov Peoples
Hosp, Peoples Hosp, Hangzhou 310014, Peoples R China.; Huang, DS;
Yang, T (corresponding author), Hangzhou Med Coll, Sch Clin Med,
Hangzhou 310014, Zhejiang, Peoples R China.; Yang, T (corresponding
author), Second Mil Med Univ, Eastern Hepatobiliary Surg Hosp, Dept
Hepatobiliary Surg, Naval Med Univ, Shanghai 200438, Peoples R
China.; Yang, T (corresponding author), Hangzhou Med Coll, Dept
Hepatobiliary Pancreat & Minimal Invas Surg, Peoples Hosp, Zhejiang
Prov Peoples Hosp, Hangzhou 310014, Zhejiang, Peoples R China.
EM huangdongshengzj@hotmail.com; yangtianehbh@smmu.edu.cn
RI Yang, Tian/CAH-2753-2022; Wang, Mingda/GRS-9652-2022; Wu,
Han/GPF-9325-2022
OI jiaqi, Zhu/0000-0001-8603-2254
CR Ally A, 2017, CELL, V169, P1327, DOI 10.1016/j.cell.2017.05.046
Blackman LD, 2021, CHEM SOC REV, V50, P1587, DOI 10.1039/
d0cs00986e
Brose MS, 2021, LANCET ONCOL, V22, P1126, DOI 10.1016/
S1470-2045(21)00332-6
Bruix J, 2021, J HEPATOL, V75, P960, DOI 10.1016/
j.jhep.2021.07.004
Chen DP, 2019, J HEPATOL, V71, P333, DOI 10.1016/
j.jhep.2019.04.007
Chen LF, 2012, IMMUNITY, V37, P930, DOI 10.1016/
j.immuni.2012.07.016
Chen L, 2019, ADV MATER, V31, DOI 10.1002/adma.201904997
Chen MS, 2021, NANO LETT, V21, P6031, DOI 10.1021/
acs.nanolett.1c01210
Cheng AL, 2020, J HEPATOL, V72, P307, DOI 10.1016/
j.jhep.2019.09.025

Cristescu R, 2018, SCIENCE, V362, P197, DOI 10.1126/science.aar3593
Di Pilato M, 2021, CELL, V184, P4512, DOI 10.1016/j.cell.2021.07.015
Dixon KO, 2021, NATURE, V595, P101, DOI 10.1038/s41586-021-03626-9
Fang RH, 2018, ADV MATER, V30, DOI 10.1002/adma.201706759
Ferrer-Fàbrega J, 2021, J HEPATOL, V75, P1154, DOI 10.1016/j.jhep.2021.06.015
Gong H, 2021, NANO LETT, V21, P2603, DOI 10.1021/acs.nanolett.1c00238
Guo JF, 2021, MOL CANCER, V20, DOI 10.1186/s12943-020-01297-0
He XY, 2020, ADV MATER, V32, DOI 10.1002/adma.202000208
Hu CMJ, 2015, NATURE, V526, P118, DOI 10.1038/nature15373
Hu QY, 2016, ADV MATER, V28, P9573, DOI 10.1002/adma.201603463
Hu QY, 2015, ADV MATER, V27, P7043, DOI 10.1002/adma.201503323
Huang YH, 2015, NATURE, V517, P386, DOI 10.1038/nature13848
Jhunjhunwala S, 2021, NAT REV CANCER, V21, P298, DOI 10.1038/s41568-021-00339-z
Jin HJ, 2021, NATURE, V595, P730, DOI 10.1038/s41586-021-03741-7
Kaczanowska S, 2021, CELL, V184, P2033, DOI 10.1016/j.cell.2021.02.048
Kelley RK, 2021, J CLIN ONCOL, V39, P2991, DOI 10.1200/JCO.20.03555
Kroll AV, 2017, BIOCONJUGATE CHEM, V28, P23, DOI 10.1021/acs.bioconjchem.6b00569
Llovet JM, 2021, NAT REV GASTRO HEPAT, V18, P293, DOI 10.1038/s41575-020-00395-0
Llovet JM, 2021, NAT REV DIS PRIMERS, V7, DOI 10.1038/s41572-020-00240-3
Ma BX, 2021, ACS APPL MATER INTER, V13, P35410, DOI 10.1021/acsami.1c08552
Mai HQ, 2021, NAT MED, V27, P1536, DOI 10.1038/s41591-021-01444-0
Melaiu O., 2020, SEMIN CANCER BIOL, V7, P10
Motzer RJ, 2020, CANCER CELL, V38, P803, DOI 10.1016/j.ccell.2020.10.011
Pan H, 2010, IMMUNITY, V33, P620, DOI 10.1016/j.immuni.2010.10.009
Pulido AD, 2021, IMMUNITY, V54, P1154, DOI 10.1016/j.immuni.2021.04.019
Pusztai L, 2021, CANCER CELL, V39, P989, DOI 10.1016/j.ccell.2021.05.009
Qin SK, 2021, J CLIN ONCOL, V39, P3002, DOI 10.1200/JCO.21.00163
Qiu NS, 2021, ADV MATER, V33, DOI 10.1002/adma.202006189
Raghav K., 2021, CANCER DISCOV, V10
Schnell A, 2020, CELL RES, V30, P285, DOI 10.1038/s41422-020-0277-x
Shang RZ, 2021, GUT, V70, P1746, DOI 10.1136/gutjnl-2020-320716
Sharma A, 2020, CELL, V183, P377, DOI 10.1016/j.cell.2020.08.040
Shields CED, 2021, CANCER DISCOV, V11, P1792, DOI 10.1158/2159-8290.CD-20-0770
Slevogt H, 2008, NAT IMMUNOL, V9, P1270, DOI 10.1038/ni.1661
Sun YF, 2021, CELL, V184, P404, DOI 10.1016/j.cell.2020.11.041
Torrens L, 2021, HEPATOLOGY, V74, P2652, DOI 10.1002/hep.32023

Wang HJ, 2019, J NANOBIOTECHNOL, V17, DOI 10.1186/
s12951-019-0494-y
Wei XL, 2018, ACS NANO, V12, P109, DOI 10.1021/acsnano.7b07720
Wu M, 2021, NAT COMMUN, V12, DOI 10.1038/s41467-021-23864-9
Xiao X, 2016, CANCER DISCOV, V6, P546, DOI
10.1158/2159-8290.CD-15-1408
Yang RY, 2021, NAT COMMUN, V12, DOI 10.1038/s41467-021-21099-2
Yang WQ, 2021, SCI TRANSL MED, V13, DOI 10.1126/
scitranslmed.aaz6804
Zhang LJ, 2021, NAT COMMUN, V12, DOI 10.1038/s41467-021-24610-x
Zhang X, 2019, J HEPATOL, V71, P163, DOI 10.1016/
j.jhep.2019.03.014
Zhuang J, 2020, SCI ADV, V6, DOI 10.1126/sciadv.aaz6108
NR 54
TC 5
Z9 5
U1 0
U2 48
PU WILEY-V C H VERLAG GMBH
PI WEINHEIM
PA POSTFACH 101161, 69451 WEINHEIM, GERMANY
SN 1613-6810
EI 1613-6829
J9 SMALL
JI Small
PD DEC
PY 2021
VL 17
IS 51
AR 2105237
DI 10.1002/smll.202105237
EA NOV 2021
PG 13
WC Chemistry, Multidisciplinary; Chemistry, Physical; Nanoscience &
Nanotechnology; Materials Science, Multidisciplinary; Physics,
Applied;
Physics, Condensed Matter
WE Science Citation Index Expanded (SCI-EXPANDED)
SC Chemistry; Science & Technology - Other Topics; Materials
Science;
Physics
GA XT1MI
UT WOS:000719629200001
PM 34791793
DA 2024-10-21
ER

PT J
AU Feng, X
Wang, LW
Zhou, RY
Zhou, R
Chen, LY
Peng, H

Huang, Y
Guo, Q
Luo, XH
Zhou, HY
AF Feng, Xu
Wang, Liwen
Zhou, Ruoyu
Zhou, Rui
Chen, Linyun
Peng, Hui
Huang, Yan
Guo, Qi
Luo, Xianghang
Zhou, Haiyan
TI Senescent immune cells accumulation promotes brown adipose tissue dysfunction during aging
SO NATURE COMMUNICATIONS
LA English
DT Article
ID DELTA T-CELLS; MESSENGER-RNA; RBM3; INNERVATION; HOMEOSTASIS; ACTIVATION; S100A8/A9; LANDSCAPE; FAT
AB Brown adipose tissue (BAT)-mediated thermogenesis declines with age. However, the underlying mechanism remains unclear. Here we reveal that bonemarrow-derived pro-inflammatory and senescent S100A8(+) immune cells, mainly T cells and neutrophils, invade the BAT of male rats and mice during aging. These S100A8+ immune cells, coupled with adipocytes and sympathetic nerves, compromise axonal networks. Mechanistically, these senescent immune cells secrete abundant S100A8 to inhibit adipose RNA-binding motif protein 3 expression. This downregulation results in the dysregulation of axon guidance-related genes, leading to impaired sympathetic innervation and thermogenic function. Xenotransplantation experiments show that human S100A8+ immune cells infiltrate mice BAT and are sufficient to induce aging-like BAT dysfunction. Notably, treatment with S100A8 inhibitor paquinimod rejuvenates BAT axon networks and thermogenic function in aged male mice. Our study suggests that targeting the bone marrow-derived senescent immune cells presents an avenue to improve BAT aging and related metabolic disorders.
C1 [Feng, Xu; Wang, Liwen; Zhou, Ruoyu; Zhou, Rui; Chen, Linyun; Peng, Hui; Huang, Yan; Guo, Qi; Luo, Xianghang; Zhou, Haiyan] Cent South Univ, Endocrinol Res Ctr, Dept Endocrinol, Xiangya Hosp, Changsha 410008, Hunan, Peoples R China.
[Luo, Xianghang; Zhou, Haiyan] Xiangya Hosp, Natl Clin Res Ctr Geriatr Disorders, Changsha 410008, Hunan, Peoples R China.
[Luo, Xianghang] Key Lab Organ Injury Aging & Regenerat Med Hunan P, Changsha 410008, Hunan, Peoples R China.
C3 Central South University; Central South University
RP Zhou, HY (corresponding author), Cent South Univ, Endocrinol Res Ctr, Dept Endocrinol, Xiangya Hosp, Changsha 410008, Hunan, Peoples R China.; Zhou, HY (corresponding author), Xiangya Hosp, Natl Clin Res Ctr Geriatr Disorders, Changsha 410008, Hunan, Peoples R China.
EM hyzhou02@csu.edu.cn
RI zhou, rui/KZT-9353-2024; Peng, Hui/KLZ-1175-2024; Zhou, Haiyan/HPF-7703-2023

OI Luo, xianghang/0000-0001-6413-572X; Zhou, Haiyan/
0000-0002-0868-3606
FU National Natural Science Foundation of China [92149306,
82120108009,
   81930022, 82170866]; Natural Science Foundation of Hunan
Province, China
   [2022JJ30990, 2023JJ30890]; China Postdoctoral Science Foundation
   [2022M713514]
FX This work was supported by the National Natural Science
Foundation of
   China (Grant Nos. 92149306, 82120108009, 81930022 to X.L.,
82170866 to
   H.Z.), Natural Science Foundation of Hunan Province, China (Grant
No.
   2022JJ30990 to H.Z., 2023JJ30890 to Y.H.), China Postdoctoral
Science
   Foundation (Grant No. 2022M713514 to H.Z.).
CR Adrover JM, 2019, IMMUNITY, V50, P390, DOI 10.1016/
j.immuni.2019.01.002
   Bahler L, 2016, J NUCL MED, V57, P372, DOI 10.2967/
jnumed.115.165829
   Bengtsson AA, 2012, ARTHRITIS RHEUM-US, V64, P1579, DOI 10.1002/
art.33493
   Brestoff JR, 2015, CELL, V161, P146, DOI 10.1016/
j.cell.2015.02.022
   Cannon B, 2004, PHYSIOL REV, V84, P277, DOI 10.1152/
physrev.00015.2003
   Cardoso F, 2021, NATURE, V597, P410, DOI 10.1038/
s41586-021-03830-7
   Casanova-Acebes M, 2013, CELL, V153, P1025, DOI 10.1016/
j.cell.2013.04.040
   Chappell SA, 2001, J BIOL CHEM, V276, P36917, DOI 10.1074/
jbc.M106008200
   Childs BG, 2017, NAT REV DRUG DISCOV, V16, P718, DOI 10.1038/
nrd.2017.116
   Collins N, 2019, CELL, V178, P1088, DOI 10.1016/
j.cell.2019.07.049
   Cui X, 2021, NAT COMMUN, V12, DOI 10.1038/s41467-021-25766-2
   De Filippo K, 2018, EUR J CLIN INVEST, V48, DOI 10.1111/eci.12949
   Desdín-Micó G, 2020, SCIENCE, V368, P1371, DOI 10.1126/
science.aax0860
   Ding XF, 2016, J ENDOCRINOL, V231, P35, DOI 10.1530/JOE-16-0229
   Dresios J, 2005, P NATL ACAD SCI USA, V102, P1865, DOI 10.1073/
pnas.0409764102
   Eash KJ, 2010, J CLIN INVEST, V120, P2423, DOI 10.1172/JCI41649
   Elmansi AM, 2020, BONE REP, V12, DOI 10.1016/j.bonr.2020.100270
   Fuentes-Fayos AC, 2020, BRAIN, V143, P3273, DOI 10.1093/brain/
awaa273
   Guo QR, 2021, CELL HOST MICROBE, V29, P222, DOI 10.1016/
j.chom.2020.12.016
   Hettinger ZR, 2021, EXP GERONTOL, V146, DOI 10.1016/
j.exger.2020.111214
   Hu B, 2020, NATURE, V578, P610, DOI 10.1038/s41586-020-2028-z
   Ibáñez-Costa A, 2022, ANN RHEUM DIS, V81, P56, DOI 10.1136/

annrheumdis-2021-220308
Kohlgruber AC, 2018, NAT IMMUNOL, V19, P464, DOI 10.1038/s41590-018-0094-2
Laustriat D, 2015, MOL THER-NUCL ACIDS, V4, DOI 10.1038/mtna.2015.35
Li CJ, 2021, CELL METAB, V33, P1957, DOI 10.1016/j.cmet.2021.08.009
Li ZS, 2020, J IMMUNOL, V204, P2589, DOI 10.4049/jimmunol.1900753
Luo OJ, 2022, NATURE AGING, V2, P348, DOI 10.1038/s43587-022-00198-9
Ma S, 2020, CELL, V180, P984, DOI 10.1016/j.cell.2020.02.008
Mantelmacher FD, 2019, NAT METAB, V1, P58, DOI 10.1038/s42255-018-0001-z
Mohanta SK, 2022, NATURE, V605, P152, DOI 10.1038/s41586-022-04673-6
Muñoz-Espín D, 2014, NAT REV MOL CELL BIO, V15, P482, DOI 10.1038/nrm3823
Okla M, 2018, INFLAMMATION, V41, P626, DOI 10.1007/s10753-017-0718-y
Palmer AK, 2019, AGING CELL, V18, DOI 10.1111/acel.12950
Pan XX, 2021, FRONT CELL DEV BIOL, V9, DOI 10.3389/fcell.2021.637424
Peretti D, 2015, NATURE, V518, P236, DOI 10.1038/nature14142
Pruenster M, 2016, PHARMACOL THERAPEUT, V167, P120, DOI 10.1016/j.pharmthera.2016.07.015
Scott-Solomon E, 2020, DEV CELL, V53, P691, DOI 10.1016/j.devcel.2020.05.020
Smart F, 2007, J NEUROCHEM, V101, P1367, DOI 10.1111/j.1471-4159.2007.04521.x
Tahvili S, 2018, PLOS ONE, V13, DOI 10.1371/journal.pone.0196598
Tao C, 2017, ENDOCRINOLOGY, V158, P1260, DOI 10.1210/en.2017-00024
Vogl T, 2007, NAT MED, V13, P1042, DOI 10.1038/nm1638
Wang LC, 2022, CELL METAB, V34, P75, DOI 10.1016/j.cmet.2021.11.002
Wang SW, 2018, FRONT IMMUNOL, V9, DOI 10.3389/fimmu.2018.01298
Wang YN, 2021, NAT METAB, V3, P1536, DOI 10.1038/s42255-021-00482-9
Wolf Y, 2017, NAT IMMUNOL, V18, P665, DOI 10.1038/ni.3746
Yang HJ, 2019, J CELL MOL MED, V23, P7010, DOI 10.1111/jcmm.14588
Yousefzadeh MJ, 2021, NATURE, V594, P100, DOI 10.1038/s41586-021-03547-7
Zeng Y, 2013, CANCER RES, V73, P4123, DOI 10.1158/0008-5472.CAN-12-1343
Zhou HY, 2021, NAT COMMUN, V12, DOI 10.1038/s41467-020-20665-4
Zhu XZ, 2019, NAT COMMUN, V10, DOI 10.1038/s41467-019-11870-x
Zhu XZ, 2016, CELL MOL LIFE SCI, V73, P3839, DOI 10.1007/s00018-016-2253-7
Zoico E, 2019, FRONT ENDOCRINOL, V10, DOI 10.3389/fendo.2019.00368
NR 52
TC 20
Z9 21
U1 10

U2 25
PU NATURE PORTFOLIO
PI BERLIN
PA HEIDELBERGER PLATZ 3, BERLIN, 14197, GERMANY
EI 2041-1723
J9 NAT COMMUN
JI Nat. Commun.
PD JUN 2
PY 2023
VL 14
IS 1
AR 3208
DI 10.1038/s41467-023-38842-6
PG 17
WC Multidisciplinary Sciences
WE Science Citation Index Expanded (SCI-EXPANDED)
SC Science & Technology - Other Topics
GA I6PY6
UT WOS:001003996200008
PM 37268694
OA Green Published, gold
DA 2024-10-21
ER

PT J
AU Wang, LF
   Anderson, DE
AF Wang, Lin-Fa
   Anderson, Danielle E.
TI Viruses in bats and potential spillover to animals and humans
SO CURRENT OPINION IN VIROLOGY
LA English
DT Article
ID RESPIRATORY SYNDROME CORONAVIRUS; FRUIT BATS; NIPAH VIRUS;
   PTEROPINE-ORTHOREOVIRUS; DROMEDARY CAMELS; TIOMAN VIRUS; HUMAN
   INFECTION; MENANGLE VIRUS; PARAMYXOVIRUS; ANTIBODIES
AB In the last two decades, several high impact zoonotic disease outbreaks have been linked to bat-borne viruses. These include SARS coronavirus, Hendra virus and Nipah virus. In addition, it has been suspected that ebolaviruses and MERS coronavirus are also linked to bats. It is being increasingly accepted that bats are potential reservoirs of a large number of known and unknown viruses, many of which could spillover into animal and human populations. However, our knowledge into basic bat biology and immunology is very limited and we have little understanding of major factors contributing to the risk of bat virus spillover events. Here we provide a brief review of the latest findings in bat viruses and their potential risk of cross-species transmission.
C1 [Wang, Lin-Fa; Anderson, Danielle E.] Duke NUS Med Sch, Programme Emerging Infect Dis, Singapore 169857, Singapore.
C3 National University of Singapore
RP Wang, LF (corresponding author), Duke NUS Med Sch, Programme Emerging Infect Dis, Singapore 169857, Singapore.
EM linfa.wang@duke-nus.edu.sg

RI Anderson, Danielle/HCH-2004-2022
OI Wang, Lin-Fa/0000-0003-2752-0535; Anderson, Danielle/
0000-0003-4791-5024
FU National Research Foundation [NRF2012NRF-CRP001-056,
NRF2016NRF-NSFC002-013]; Ministry of Health [CDPHRG/0006/2014];
Ministry
of Defence in Singapore [DIRP2015-9016102060]; Duke-NUS Medical
School;
Estate of Tan Sri Khoo Teck Puat [NUS-KBrFA/2018/0016]
FX We thank Xiao Fang Lim, Xinglou Yang and Anna Uehara for help
with
Figure 1, Figure 3 and Table 1, respectively. We are grateful to
Andrew
Hickey for critical review of the manuscript. The research in L-
FW's
group is funded by the National Research Foundation
[NRF2012NRF-CRP001-056 and NRF2016NRF-NSFC002-013], Ministry of
Health
[CDPHRG/0006/2014] and Ministry of Defence [DIRP2015-9016102060]
in
Singapore. Research in DEA's group is funded by Duke-NUS Medical
School
and the Estate of Tan Sri Khoo Teck Puat [NUS-KBrFA/2018/0016].
CR Ahn M, 2016, SCI REP-UK, V6, DOI 10.1038/srep21722
Allen T, 2017, NAT COMMUN, V8, DOI 10.1038/s41467-017-00923-8
Amman BR, 2015, J WILDLIFE DIS, V51, P774, DOI
10.7589/2015-02-044
Anderson DE, 2019, EMERG INFECT DIS, V25, P166, DOI 10.3201/
eid2501.180267
Anthony SJ, 2017, MBIO, V8, DOI [10.1128/mBio.00373-17, 10.1128/
mbio.00373-17]
Azhar EI, 2014, NEW ENGL J MED, V370, P2499, DOI 10.1056/
NEJMoa1401505
Barr JA, 2012, J GEN VIROL, V93, P2590, DOI 10.1099/
vir.0.045385-0
Bean AGD, 2013, NAT REV IMMUNOL, V13, P851, DOI 10.1038/nri3551
Beaty SM, 2017, J INFECT DIS, V215, P209, DOI 10.1093/infdis/
jiw534
Calisher CH, 2006, CLIN MICROBIOL REV, V19, P531, DOI 10.1128/
CMR.00017-06
Chant K, 1998, EMERG INFECT DIS, V4, P273, DOI 10.3201/
eid0402.980215
Cheng P, 2009, J CLIN VIROL, V45, P79, DOI 10.1016/
j.jcv.2009.03.001
Chu DKW, 2014, EMERG INFECT DIS, V20, P1049, DOI 10.3201/
eid2006.140299
Chua KB, 2011, PLOS ONE, V6, DOI 10.1371/journal.pone.0025434
Chua KB, 2008, PLOS ONE, V3, DOI 10.1371/journal.pone.0003803
Chua KB, 2000, SCIENCE, V288, P1432, DOI 10.1126/
science.288.5470.1432
Chua KB, 2002, MICROBES INFECT, V4, P145, DOI 10.1016/
S1286-4579(01)01522-2
Chua KB, 2001, VIROLOGY, V283, P215, DOI 10.1006/viro.2000.0882
Chua KB, 1999, LANCET, V354, P1257, DOI 10.1016/

S0140-6736(99)04299-3
Ciminski K, 2017, J GEN VIROL, V98, P2393, DOI 10.1099/jgv.0.000927
de Araujo Jansen, 2012, BMC Research Notes, V5, P690, DOI 10.1186/1756-0500-5-690
Denison MR, 2011, RNA BIOL, V8, P270, DOI 10.4161/rna.8.2.15013
Drexler JF, 2012, NAT COMMUN, V3, DOI 10.1038/ncomms1796
Epstein JH, 2008, EMERG INFECT DIS, V14, P1309, DOI 10.3201/eid1408.071492
Field H, 2011, PLOS ONE, V6, DOI 10.1371/journal.pone.0028678
Field HE, 2016, CURR OPIN VIROL, V16, P120, DOI 10.1016/j.coviro.2016.02.004
Francis JR, 2014, MED J AUSTRALIA, V201, P647, DOI 10.5694/mja13.00261
GARD GP, 1973, ARCH GES VIRUSFORSCH, V43, P34, DOI 10.1007/BF01249346
Ge XY, 2013, NATURE, V503, P535, DOI 10.1038/nature12711
Geldenhuys M, 2018, PLOS ONE, V13, DOI 10.1371/journal.pone.0194527
Goldstein T, 2018, NAT MICROBIOL, V3, P1084, DOI 10.1038/s41564-018-0227-2
Guo WP, 2013, PLOS PATHOG, V9, DOI 10.1371/journal.ppat.1003159
Haagmans BL, 2014, LANCET INFECT DIS, V14, P140, DOI 10.1016/S1473-3099(13)70690-X
Hayman DTS, 2008, PLOS ONE, V3, DOI 10.1371/journal.pone.0002739
He B, 2017, J VIROL, V91, DOI [10.1128/JVI.02493-16, 10.1128/jvi.02493-16]
He B, 2013, J VIROL, V87, P12357, DOI 10.1128/JVI.02013-13
Hsu VP, 2004, EMERG INFECT DIS, V10, P2082, DOI 10.3201/eid1012.040701
Hu B, 2017, PLOS PATHOG, V13, DOI 10.1371/journal.ppat.1006698
Hu D, 2018, EMERG MICROBES INFEC, V7, DOI 10.1038/s41426-018-0155-5
Hu TS, 2014, BMC MICROBIOL, V14, DOI 10.1186/s12866-014-0293-4
Huang CP, 2016, PLOS PATHOG, V12, DOI 10.1371/journal.ppat.1005883
Iehlé C, 2007, EMERG INFECT DIS, V13, P159, DOI 10.3201/eid1301.060791
Ithete NL, 2013, EMERG INFECT DIS, V19, P1697, DOI 10.3201/eid1910.130946
Johara MY, 2001, EMERG INFECT DIS, V7, P439
Johnson ED, 1996, ARCH VIROL, P101
Jones KE, 2008, NATURE, V451, P990, DOI 10.1038/nature06536
Kemenesi G, 2018, EMERG MICROBES INFEC, V7, DOI 10.1038/s41426-018-0067-4
Kemenesi G, 2014, VECTOR-BORNE ZOONOT, V14, P846, DOI 10.1089/vbz.2014.1637
KIM GR, 1994, ARCH VIROL, V134, P85, DOI 10.1007/BF01379109
Kocher JF, 2018, MBIO, V9, DOI 10.1128/mBio.00869-18
Krüger N, 2018, CELL REP, V25, P312, DOI 10.1016/j.celrep.2018.09.018
Lau SKP, 2005, P NATL ACAD SCI USA, V102, P14040, DOI 10.1073/pnas.0506735102
Lau SKP, 2015, VIROL J, V12, DOI 10.1186/s12985-015-0432-z

Lau SKP, 2010, VIROLOGY, V404, P106, DOI 10.1016/j.virol.2010.03.049
Leendertz SAJ, 2016, VIRUSES-BASEL, V8, DOI 10.3390/v8020030
Li WD, 2005, SCIENCE, V310, P676, DOI 10.1126/science.1118391
Lorusso A, 2015, INFECT GENET EVOL, V30, P55, DOI 10.1016/j.meegid.2014.12.006
Luby SP, 2009, EMERG INFECT DIS, V15, P1229, DOI 10.3201/eid1508.081237
Luis AD, 2013, P ROY SOC B-BIOL SCI, V280, DOI 10.1098/rspb.2012.2753
Luo CM, 2018, J VIROL, V92, DOI 10.1128/JVI.00116-18
Marsh GA, 2012, PLOS PATHOG, V8, DOI 10.1371/journal.ppat.1002836
McMullan LK, 2012, NEW ENGL J MED, V367, P834, DOI 10.1056/NEJMoa1203378
Menachery VD, 2016, P NATL ACAD SCI USA, V113, P3048, DOI 10.1073/pnas.1517719113
Moreira EA, 2016, P NATL ACAD SCI USA, V113, P12797, DOI 10.1073/pnas.1608821113
Mourya DT, 2014, J VIROL, V88, P3605, DOI 10.1128/JVI.02617-13
Müller MA, 2015, LANCET INFECT DIS, V15, P559, DOI 10.1016/S1473-3099(15)70090-3
Nakkazi E, 2018, LANCET, V392, P623, DOI 10.1016/S0140-6736(18)31981-0
Negredo A, 2011, PLOS PATHOG, V7, DOI 10.1371/journal.ppat.1002304
Obameso JO, 2017, SCI CHINA LIFE SCI, V60, P1357, DOI 10.1007/s11427-017-9263-6
Olival KJ, 2017, NATURE, V546, P646, DOI 10.1038/nature22975
Olson JG, 2002, EMERG INFECT DIS, V8, P987, DOI 10.3201/eid0809.010515
Pavlovich SS, 2018, CELL, V173, P1098, DOI 10.1016/j.cell.2018.03.070
PAWAN J L, 1959, Caribb Med J, V21, P137
Peel AJ, 2012, PLOS ONE, V7, DOI 10.1371/journal.pone.0030346
Peiris JSM, 2003, NEW ENGL J MED, V349, P2431, DOI 10.1056/NEJMra032498
Pernet O, 2014, NAT COMMUN, V5, DOI 10.1038/ncomms6342
Philbey AW, 2008, AUST VET J, V86, P449, DOI 10.1111/j.1751-0813.2008.00361.x
Piot P, 2018, T ROY SOC TROP MED H, V112, P527, DOI 10.1093/trstmh/try088
Pritchard LI, 2006, ARCH VIROL, V151, P229, DOI 10.1007/s00705-005-0644-4
Reusken CBEM, 2013, LANCET INFECT DIS, V13, P859, DOI 10.1016/S1473-3099(13)70164-6
Sabino-Santos G Jr, 2018, SCI REP-UK, V8, DOI 10.1038/s41598-018-27442-w
Sabino-Santos G, 2015, AM J TROP MED HYG, V93, P404, DOI 10.4269/ajtmh.15-0032
Sabir JSM, 2016, SCIENCE, V351, P81, DOI 10.1126/science.aac8608
Saif LJ, 2004, REV SCI TECH OIE, V23, P643, DOI 10.20506/rst.23.2.1513
Salas-Rojas M, 2004, T ROY SOC TROP MED H, V98, P577, DOI 10.1016/j.trstmh.2003.10.019

Schiff L.A., 2007, FIELDS VIROLOGY, V2, P1853
Schuh AJ, 2017, MICROBIOL AUST, V38, P12, DOI 10.1071/MA17005
Sendow I, 2013, PLOS ONE, V8, DOI 10.1371/journal.pone.0069544
Singh H, 2015, SCI REP-UK, V5, DOI 10.1038/srep16517
Singh H, 2015, J MED VIROL, V87, P2145, DOI 10.1002/jmv.24274
Smith EC, 2013, PLOS PATHOG, V10
Smith I, 2013, CURR OPIN VIROL, V3, P84, DOI 10.1016/j.coviro.2012.11.006
STEPHAN HA, 1988, VET REC, V122, P6, DOI 10.1136/vr.122.1.6
Takemae H, 2018, VIRUS GENES, V54, P823, DOI 10.1007/s11262-018-1603-y
Taniguchi S, 2017, ARCH VIROL, V162, P1529, DOI 10.1007/s00705-017-3251-2
Tao Y, 2017, J VIROL, V91, DOI 10.1128/JVI.01953-16
Taylor LH, 2001, PHILOS T R SOC B, V356, P983, DOI 10.1098/rstb.2001.0888
Teeling EC, 2005, SCIENCE, V307, P580, DOI 10.1126/science.1105113
Teeling EC, 2018, ANNU REV ANIM BIOSCI, V6, P23, DOI 10.1146/annurev-animal-022516-022811
Tong SX, 2013, PLOS PATHOG, V9, DOI 10.1371/journal.ppat.1003657
Tong SX, 2012, P NATL ACAD SCI USA, V109, P4269, DOI 10.1073/pnas.1116200109
Towner JS, 2006, J VIROL, V80, P6497, DOI 10.1128/JVI.00069-06
Towner JS, 2009, PLOS PATHOG, V5, DOI 10.1371/journal.ppat.1000536
Tse H, 2012, PLOS ONE, V7, DOI 10.1371/journal.pone.0034987
Voon K, 2015, J MED VIROL, V87, P2149, DOI 10.1002/jmv.24304
Wang J, 2015, J GEN VIROL, V96, P2442, DOI 10.1099/vir.0.000158
Wang LF, 2007, CURR TOP MICROBIOL, V315, P325
Wang LF, 2007, ARCH VIROL, V152, P1259, DOI 10.1007/s00705-007-0959-4
Wang LF, 2011, CURR OPIN VIROL, V1, P649, DOI 10.1016/j.coviro.2011.10.013
Waruhiu C, 2017, VIROL SIN, V32, P101, DOI 10.1007/s12250-016-3930-2
WATERMAN J A, 1959, Caribb Med J, V21, P1
Weiss S, 2012, EMERG INFECT DIS, V18, P159, DOI 10.3201/eid1801.111026
Wong AH, 2012, EMERG INFECT DIS, V18, P944, DOI 10.3201/eid1806.111688
Wong S, 2007, REV MED VIROL, V17, P67, DOI 10.1002/rmv.520
Wu ZQ, 2016, ISME J, V10, P609, DOI 10.1038/ismej.2015.138
Wynne JW, 2014, GENOME BIOL, V15, DOI 10.1186/s13059-014-0532-x
Xie JZ, 2018, CELL HOST MICROBE, V23, P297, DOI 10.1016/j.chom.2018.01.006
Xu BL, 2011, PLOS PATHOG, V7, DOI 10.1371/journal.ppat.1002369
Yadav PD, 2012, AM J TROP MED HYG, V87, P576, DOI 10.4269/ajtmh.2012.11-0416
Yaiw KC, 2008, J VIROL, V82, P565, DOI 10.1128/JVI.01660-07
Yang XL, 2019, NAT MICROBIOL, V4, P390, DOI 10.1038/s41564-018-0328-y
Yinda CK, 2018, VIRUS EVOL, V4, DOI 10.1093/ve/vey008
Yinda CK, 2016, SCI REP-UK, V6, DOI 10.1038/srep34209

   Zaki AM, 2012, NEW ENGL J MED, V367, P1814, DOI 10.1056/
NEJMoa1211721
   Zaki AM, PAPER OPENED WHOLE N
   ZELLER HG, 1989, ARCH VIROL, V108, P211, DOI 10.1007/BF01310935
   Zhou B, 2014, PLOS PATHOG, V10, DOI 10.1371/journal.ppat.1004420
   Zhou P, 2016, P NATL ACAD SCI USA, V113, P2696, DOI 10.1073/
pnas.1518240113
   1995, SCIENCE, V268, P94
   2010, ARCH VIROL, V155, P1295, DOI DOI 10.1007/S00705-010-0690-4
   2014, EMERG INFECT DIS, V20, P211, DOI DOI 10.3201/EID2002.131620
   2018, NATURE, V556, P255, DOI DOI 10.1038/S41586-018-0010-9
   2007, P NATL ACAD SCI USA, V104, P1142, DOI DOI 10.1073/
PNAS.0701372104
   2016, EMERG INFECT DIS, V22, P703
   2018, VIROL SIN, V33, P100, DOI DOI 10.1007/S12250-018-0010-9
   2015, J VIROL, V89, P4539, DOI DOI 10.1128/JVI.03693-14
NR 136
TC 174
Z9 187
U1 0
U2 83
PU ELSEVIER SCI LTD
PI OXFORD
PA THE BOULEVARD, LANGFORD LANE, KIDLINGTON, OXFORD OX5 1GB, OXON,
ENGLAND
SN 1879-6257
EI 1879-6265
J9 CURR OPIN VIROL
JI Curr. Opin. Virol.
PD FEB
PY 2019
VL 34
BP 79
EP 89
DI 10.1016/j.coviro.2018.12.007
PG 11
WC Virology
WE Science Citation Index Expanded (SCI-EXPANDED)
SC Virology
GA HX6JK
UT WOS:000467509700012
PM 30665189
OA Green Published
DA 2024-10-21
ER

PT J
AU Delpietro, HA
   Russo, RG
AF Delpietro, Horacio A.
   Russo, Roberto G.
TI ACQUIRED RESISTANCE TO SALIVA ANTICOAGULANTS BY PREY PREVIOUSLY
FED UPON
   BY VAMPIRE BATS (<i>DESMODUS ROTUNDUS</i>): EVIDENCE FOR IMMUNE

RESPONSE
SO JOURNAL OF MAMMALOGY
LA English
DT Article
DE acquired resistance; anticoagulants; common vampire bat; Desmodus rotundus; vampire saliva
ID PLASMINOGEN-ACTIVATOR; IMMUNOLOGICAL CONTROL; DRACULIN; ECTOPARASITES;
IMMUNIZATION; ATTACHMENT; PREDATION; VACCINES; CLONING; NORTH
AB Ranchers have observed that residual hemorrhage decreases after livestock suffer repeated bites from common vampire bats (Desmodus rotundus). We hypothesized that repeated exposure to the anticoagulants in the bats' saliva generated an immune response. We compared clotting, time of blood mixed with Desmodus saliva between individuals of several species of livestock that were regularly exposed to vampire bats ("batattacked") and individuals living where bat predation was not observed ("bat-naive"). We also compared the bleeding times of domestic sheep following a single defensive bite from a bat, before and after subjecting the animals to a series of feeding bites by Desmodus. Clotting time was significantly shorter in bat-attacked than in bat-naive livestock. Bleeding time after a provoked defensive bat bite was significantly shorter after sheep were exposed to a series of feeding bites from Desmodus. Bat predation induced increased resistance by the prey against the anticoagulants, suggesting an immune response.
C1 [Delpietro, Horacio A.; Russo, Roberto G.] Serv Nacl Sanidad & Calidad Agroalimentaria, SENASA, RA-3300 Posadas, Argentina.
RP Delpietro, HA (corresponding author), Serv Nacl Sanidad & Calidad Agroalimentaria, SENASA, Padre Serrano 1116, RA-3300 Posadas, Argentina.
EM hadelpietro@arnet.com.ar
CR [Anonymous], 1984, WHO TECH REP SER, P1
[Anonymous], 1992, WHO TECH REP SER, P1
[Anonymous], 1961, REV MUSEO ARGENTINO
APITZCASTRO R, 1995, THROMB HAEMOSTASIS, V73, P94
Arellano C., 1971, TEC PECU, V17, P23
Boag PR, 2003, VET IMMUNOL IMMUNOP, V92, P87, DOI 10.1016/S0165-2427(03)00006-0
Brass D.A., 1994, RABIES BATS NATURAL
*CHIR SPEC GROUP, 1996, IUCN 2006 2006 IUCN
da Rosa EST, 2006, EMERG INFECT DIS, V12, P1197, DOI 10.3201/eid1708.050929
DALQUEST WALTER W., 1955, AMER MIDLAND NAT, V53, P79, DOI 10.2307/2422300
Dalton JP, 2001, VET PARASITOL, V98, P149, DOI 10.1016/S0304-4017(01)00430-7
De Anda D.L., 1976, TEC PECU MEX, V30, P67
De Verteuil E, 1936, CARIBBEAN MED J, V21, P85
DELPIETRO H, 1994, APPL ANIM BEHAV SCI, V39, P141, DOI 10.1016/0168-1591(94)90134-1
Delpietro H. A., 1999, Revista de Medicina Veterinaria (Buenos Aires), V80, P460
DELPIETRO HA, 1992, PREV VET MED, V14, P13, DOI 10.1016/0167-5877(92)90080-Y

Delpietro HA, 1997, J VET MED B, V44, P477, DOI 10.1111/j.1439-0450.1997.tb00998.x
Di SANTO PAULA E., 1960, JOUR MORPHOL, V106, P301
DIZIJ A, 1995, PARASITE IMMUNOL, V17, P177, DOI 10.1111/j.1365-3024.1995.tb00887.x
Fernandez AZ, 1998, BBA-GEN SUBJECTS, V1425, P291, DOI 10.1016/S0304-4165(98)00082-8
Fernandez AZ, 1999, BBA-PROTEIN STRUCT M, V1434, P135, DOI 10.1016/S0167-4838(99)00160-0
Gannon WL, 2007, J MAMMAL, V88, P809, DOI 10.1644/06-MAMM-F-185R1.1
GARDELL SJ, 1989, J BIOL CHEM, V264, P17947
Gebbia JA, 1995, EXP APPL ACAROL, V19, P593, DOI 10.1007/BF00048814
GOODWIN GEORGE G., 1961, BULL AMER MUS NAT HIST, V122, P187
GREENHALL A M, 1983, Mammalian Species, P1, DOI 10.2307/3503895
GREENHALL AM, 1972, J ZOOL, V168, P451
GREENHALL AM, 1993, BATS RABIES
GREENHALL AM, 1969, AN I BIOL MEX, V40, P245
HAWKEY C, 1966, NATURE, V211, P434, DOI 10.1038/211434c0
HAWKEY C, 1967, BRIT J HAEMATOL, V13, P1014, DOI 10.1111/j.1365-2141.1967.tb08870.x
KRATZSCHMAR J, 1992, GENE, V116, P281, DOI 10.1016/0378-1119(92)90526-U
KRATZSCHMAR J, 1991, GENE, V105, P229, DOI 10.1016/0378-1119(91)90155-5
KVERNO NB, 1976, REV MUNDIAL ZOOTECNI, V17, P1
LINHART SB, 1972, B OFIC SANIT PANAM, V73, P100
Lord RD, 1981, ANALES I BIOL U NACL, V51, P591
Mayen F, 2003, J VET MED B, V50, P469, DOI 10.1046/j.1439-0450.2003.00713.x
MCFARLAND WN, 1969, COMP BIOCH PHYSL, V98, P985
MITCHELL GC, 1970, J MAMMAL, V51, P814, DOI 10.2307/1378317
MOMIN RR, 1991, TROP ANIM HEALTH PRO, V23, P227, DOI 10.1007/BF02357105
NEED JT, 1991, J MED ENTOMOL, V28, P37, DOI 10.1093/jmedent/28.1.37
Pihlgren M, 2004, VACCINE, V23, P329, DOI 10.1016/j.vaccine.2004.06.010
Pilorz V, 2005, PHYSIOL BEHAV, V85, P205, DOI 10.1016/j.physbeh.2005.04.008
Pruett JH, 1999, INT J PARASITOL, V29, P25, DOI 10.1016/S0020-7519(98)00172-6
ROMANA C, 1939, B SOC PATHOL EXOT, V32, P885
ROUK CS, 1970, J MAMMAL, V51, P455, DOI 10.2307/1378387
SCHMIDT U, 1978, Z SAUGETIERKD, V43, P70
THOMPSON RD, 1977, J WILDLIFE MANAGE, V41, P736, DOI 10.2307/3799998
Trimnell AR, 2002, VACCINE, V20, P3560, DOI 10.1016/S0264-410X(02)00334-1
van Alken WG, 2004, WHO TECH REP SER, V926, P1
Viney M, 2002, TRENDS PARASITOL, V18, P63, DOI 10.1016/S1471-4922(01)02171-7
Walker TM, 2001, J REPROD INFANT PSYC, V19, P7, DOI

10.1080/02646830020032365
   Wikel SK, 2001, VET PARASITOL, V101, P275, DOI 10.1016/
S0304-4017(01)00556-8
   Willadsen P, 1999, GENET ANAL-BIOMOL E, V15, P131, DOI 10.1016/
S1050-3862(99)00016-9
   Willadsen P, 2001, VET PARASITOL, V101, P353, DOI 10.1016/
S0304-4017(01)00560-X
   Wimsatt W. A., 1962, Journal of Mammalogy, V43, P17, DOI
10.2307/1376876
   WIMSATT WA, 1969, J MAMMAL, V50, P233, DOI 10.2307/1378339
NR 57
TC 12
Z9 13
U1 0
U2 15
PU OXFORD UNIV PRESS INC
PI CARY
PA JOURNALS DEPT, 2001 EVANS RD, CARY, NC 27513 USA
SN 0022-2372
EI 1545-1542
J9 J MAMMAL
JI J. Mammal.
PD OCT
PY 2009
VL 90
IS 5
BP 1132
EP 1138
DI 10.1644/07-MAMM-A-374.1
PG 7
WC Zoology
WE Science Citation Index Expanded (SCI-EXPANDED)
SC Zoology
GA 509GG
UT WOS:000271002400009
OA Bronze
DA 2024-10-21
ER

PT J
AU Kepple, JD
   Barra, JM
   Young, ME
   Hunter, CS
   Tse, HM
AF Kepple, Jessica D.
   Barra, Jessie M.
   Young, Martin E.
   Hunter, Chad S.
   Tse, Hubert M.
TI Islet transplantation into brown adipose tissue can delay immune
   rejection
SO JCI INSIGHT
LA English

DT Article
ID MESENCHYMAL STEM-CELLS; PORCINE ISLETS; MACROPHAGES; ENCAPSULATION;
EXPRESSION; ALLOGRAFT; RESPONSES; HUMANS; SITE
AB Type 1 diabetes is an autoimmune disease characterized by insulin-producing beta cell destruction. Although islet transplantation restores euglycemia and improves patient outcomes, an ideal transplant site remains elusive. Brown adipose tissue (BAT) has a highly vascularized and antiinflammatory microenvironment. Because these tissue features can promote islet graft survival, we hypothesized that islets transplanted into BAT will maintain islet graft and BAT function while delaying immune-mediated rejection. We transplanted syngeneic and allogeneic islets into BAT or under the kidney capsule of streptozotocin-induced diabetic NOD.Rag and NOD mice to investigate islet graft function, BAT function, metabolism, and immune-mediated rejection. Islet grafts within BAT restored euglycemia similarly to kidney capsule controls. Islets transplanted in BAT maintained expression of islet hormones and transcription factors and were vascularized. Compared with those in kidney capsule and euglycemic mock-surgery controls, no differences in glucose or insulin tolerance, thermogenic regulation, or energy expenditure were observed with islet grafts in BAT. Immune profiling of BAT revealed enriched antiinflammatory macrophages and T cells. Compared with the kidney capsule control, there were significant delays in autoimmune and allograft rejection of islets transplanted in BAT, possibly due to increased antiinflammatory immune populations. Our data support BAT as an alternative islet transplant site that may improve graft survival.
C1 [Kepple, Jessica D.; Barra, Jessie M.; Young, Martin E.; Hunter, Chad S.; Tse, Hubert M.] Univ Alabama Birmingham, Comprehens Diabet Ctr, Birmingham, AL 35294 USA.
[Kepple, Jessica D.; Hunter, Chad S.] Univ Alabama Birmingham, Div Endocrinol Diabet & Metab, Dept Med, Birmingham, AL 35294 USA.
[Barra, Jessie M.; Tse, Hubert M.] Univ Alabama Birmingham, Dept Microbiol, Birmingham, AL 35294 USA.
[Young, Martin E.] Univ Alabama Birmingham, Div Cardiovasc Dis, Birmingham, AL 35294 USA.
C3 University of Alabama System; University of Alabama Birmingham;
University of Alabama System; University of Alabama Birmingham;
University of Alabama System; University of Alabama Birmingham;
University of Alabama System; University of Alabama Birmingham
RP Tse, HM (corresponding author), Univ Alabama Birmingham, 1825 Univ Blvd,SHEL 1202, Birmingham, AL 35294 USA.; Hunter, CS (corresponding author), Univ Alabama Birmingham, 1825 Univ Blvd,SHEL 1211, Birmingham, AL 35294 USA.
EM huntercs@uab.edu; htse@uab.edu
OI Barra, Jessie/0000-0002-6456-9293; Kepple, Dr.
Jessica/0000-0002-0600-2045; Tse, Hubert/0000-0001-5861-7597; Hunter,
Chad/0000-0002-1644-447X
FU NIH/National Institute of Diabetes and Digestive and Kidney Diseases
(NIDDK) [DK099550, DK127497, DK111483, DK128132]; NIH/NIDDK [DK126456];

JDRF [SRA-2016-270-S-B, 2-SRA-2019-692-S-B]; NIH/National Institute of
General Medical Sciences (NIGMS) [T32GM109780]; NIH/NIGMS [T32GM008111];
2019 UCDC Graduate Student Innovator Award; National Institute of
Diabetes and Digestive and Kidney Diseases [F31DK121414, R01DK126456,
R01DK127497, R01DK128132, R01DK111483] Funding Source: NIH RePORTER;
National Institute of General Medical Sciences [T32GM008111] Funding
Source: NIH RePORTER
FX We acknowledge the UAB Small Animal Phenotyping Core for assistance with
body composition measure-ments using quantitative magnetic resonance. We
would especially like to thank Anath Shalev, Director of UAB
Comprehensive Diabetes Center (UCDC) , for her support and establishment
of the Graduate Innova-tor Award training grant, which helped make this
work possible. We thank Katie Heath, Eliana Toren, Tan-ya Pierre, KaLia
Burnette, Miranda Chavez, Samuel Blum, and Jared Taylor for critical
review of the manu-script. This work was supported by NIH/ National
Institute of Diabetes and Digestive and Kidney Diseases (NIDDK) R01
awards (DK099550 and DK127497 to HMT; DK111483 and DK128132 to CSH) , an
NIH/NIDDK R01 award (DK126456 to HMT and CSH) , and F31DK121414
Fellowship (JDK) ; JDRF awards (SRA-2016-270-S-B and 2-SRA-2019-692-S-B
to HMT) ; an NIH/National Institute of General Medical Sciences (NIGMS)
Translational and Molecular Sciences T32 training grant (T32GM109780 to
JDK and JMB) ; NIH/NIGMS Cell, Molecular, and Developmental Biology T32
training grant (T32GM008111 to JMB) ; and the 2019 UCDC Graduate Student
Innovator Award (to JMB and JDK) .
CR Anazawa T, 2019, ANN GASTROENT SURG, V3, P34, DOI 10.1002/ags3.12214
Ascon DB, 2008, J LEUKOCYTE BIOL, V84, P1400, DOI 10.1189/jlb.0907651
Ayenehdeh JM, 2017, IMMUNOL LETT, V188, P21, DOI 10.1016/j.imlet.2017.05.006
Barra JM, 2021, XENOTRANSPLANTATION, DOI 10.1111/xen.12706
Barra JM, 2020, DIABETES, V69, P1948, DOI 10.2337/db20-0248
Bartelt A, 2011, NAT MED, V17, P200, DOI 10.1038/nm.2297
Berman DM, 2010, DIABETES, V59, P2558, DOI 10.2337/db10-0136
Bethea M, 2019, AM J PHYSIOL-ENDOC M, V316, pE397, DOI 10.1152/

ajpendo.00235.2018
Bottino Rita, 2015, World J Transplant, V5, P243, DOI 10.5500/wjt.v5.i4.243
Cantarelli E, 2011, CURR DIABETES REP, V11, P364, DOI 10.1007/s11892-011-0216-9
Carpentier AC, 2018, FRONT ENDOCRINOL, V9, DOI 10.3389/fendo.2018.00447
Castro-Gutierrez R, 2021, FRONT ENDOCRINOL, V12, DOI 10.3389/fendo.2021.707881
Cerf ME, 2005, BIOCHEM BIOPH RES CO, V326, P699, DOI 10.1016/j.bbrc.2004.10.217
Chou HS, 2012, TRANSPLANTATION, V93, P272, DOI 10.1097/TP.0b013e31823ffd39
Cypess AM, 2015, CELL METAB, V21, P33, DOI 10.1016/j.cmet.2014.12.009
Cypess AM, 2009, NEW ENGL J MED, V360, P1509, DOI 10.1056/NEJMoa0810780
Pham-Hua D, 2017, BIOMATERIALS, V128, P19, DOI 10.1016/j.biomaterials.2017.03.002
Delaune V, 2017, TRANSPL INT, V30, P227, DOI 10.1111/tri.12919
Ediger BN, 2014, DIABETES, V63, P4206, DOI 10.2337/db14-0096
Eriksson O, 2019, INT J MOL SCI, V20, DOI 10.3390/ijms20235827
Ferrante AW, 2013, DIABETES OBES METAB, V15, P34, DOI 10.1111/dom.12154
Fitzgibbons TP, 2011, AM J PHYSIOL-HEART C, V301, pH1425, DOI 10.1152/ajpheart.00376.2011
Habegger KM, 2013, DIABETES, V62, P1453, DOI 10.2337/db12-1116
Hamada M, 2020, EXP ANIM TOKYO, V69, P1, DOI 10.1538/expanim.19-0076
Hayward JA, 2017, DIABETES, V66, P1312, DOI 10.2337/db16-1068
Herrero L, 2010, P NATL ACAD SCI USA, V107, P240, DOI 10.1073/pnas.0905310107
Hu HCH, 2014, MAGN RESON IMAGING, V32, P107, DOI 10.1016/j.mri.2013.10.003
Jensen DM, 2020, METABOLITES, V10, DOI 10.3390/metabo10120485
Jin HT, 2011, CURR TOP MICROBIOL, V350, P17, DOI 10.1007/82_2010_116
Johansson U, 2008, DIABETES, V57, P2393, DOI 10.2337/db07-0981
Krzystyniak A, 2014, CURR OPIN ORGAN TRAN, V19, P610, DOI 10.1097/MOT.0000000000000130
Kuppan P, 2020, AM J TRANSPLANT, V20, P714, DOI 10.1111/ajt.15662
Kuppan P, 2020, XENOTRANSPLANTATION, V27, DOI 10.1111/xen.12581
Lee P, 2013, ENDOCR REV, V34, P413, DOI 10.1210/er.2012-1081
Lidell ME, 2013, NAT MED, V19, P631, DOI 10.1038/nm.3017
Liu ZZ, 2017, CELL TRANSPLANT, V26, P925, DOI 10.3727/096368917X694859
Loyd C, 2017, ENDOCRINOLOGY, V158, P1289, DOI 10.1210/en.2016-1791
Ma XQ, 2012, CELL TRANSPLANT, V21, P61, DOI 10.3727/096368911X580563
Medrikova D, 2015, PLOS ONE, V10, DOI 10.1371/journal.pone.0118534
Mondanelli G, 2017, IMMUNITY, V46, P233, DOI 10.1016/j.immuni.2017.01.005

NAJARIAN JS, 1980, ANN SURG, V192, P526, DOI
10.1097/00000658-198010000-00011
Nedergaard J, 2011, CELL METAB, V13, P238, DOI 10.1016/
j.cmet.2011.02.009
Nguyen KD, 2011, NATURE, V480, P104, DOI 10.1038/nature10653
Odegaard JI, 2015, CURR OPIN IMMUNOL, V36, P67, DOI 10.1016/
j.coi.2015.07.003
Pepper AR, 2015, NAT BIOTECHNOL, V33, P518, DOI 10.1038/nbt.3211
Rickels MR, 2019, ENDOCR REV, V40, P631, DOI 10.1210/
er.2018-00154
Staels W, 2016, DIABETES OBES METAB, V18, P115, DOI 10.1111/
dom.12556
Stanford KI, 2013, J CLIN INVEST, V123, P215, DOI 10.1172/
JCI62308
Steffes MW, 2003, DIABETES CARE, V26, P832, DOI 10.2337/
diacare.26.3.832
Tahbaz M, 2021, FRONT ENDOCRINOL, V12, DOI 10.3389/
fendo.2021.716625
Takahashi H, 2019, J CLIN MED, V8, DOI 10.3390/jcm8020249
Thayer TC, 2011, DIABETES, V60, P2144, DOI 10.2337/db10-1222
Tran R, 2020, SCI REP-UK, V10, DOI 10.1038/s41598-020-57787-0
Tschöp MH, 2012, NAT METHODS, V9, P57, DOI [10.1038/NMETH.1806,
10.1038/nmeth.1806]
Tsuchiya M, 2009, INT J MOL MED, V23, P725, DOI 10.3892/
ijmm_00000186
Vaithilingam V, 2017, SCI REP-UK, V7, DOI 10.1038/
s41598-017-10359-1
Velazco-Cruz L, 2019, STEM CELL REP, V12, P351, DOI 10.1016/
j.stemcr.2018.12.012
Villarroya F, 2018, CELL METAB, V27, P954, DOI 10.1016/
j.cmet.2018.04.006
Virtanen KA, 2009, NEW ENGL J MED, V360, P1518, DOI 10.1056/
NEJMoa0808949
Wang WS, 2016, NAT REV MOL CELL BIO, V17, P691, DOI 10.1038/
nrm.2016.96
Witkowski Piotr, 2014, Adv Surg, V48, P223
Xiao XY, 2009, J AUTOIMMUN, V32, P85, DOI 10.1016/
j.jaut.2008.12.003
Xu K, 2020, TRANSPLANTATION, V104, P2059, DOI 10.1097/
TP.0000000000003322
Yasunami Y, 2005, J EXP MED, V202, P913, DOI 10.1084/jem.20050448
Yin D, 2006, AM J TRANSPLANT, V6, P60, DOI 10.1111/
j.1600-6143.2005.01157.x
Zeyland J, 2014, ARCH IMMUNOL THER EX, V62, P411, DOI 10.1007/
s00005-014-0280-3
NR 66
TC 10
Z9 10
U1 3
U2 21
PU AMER SOC CLINICAL INVESTIGATION INC
PI ANN ARBOR
PA 2015 MANCHESTER RD, ANN ARBOR, MI 48104 USA
EI 2379-3708

J9 JCI INSIGHT
JI JCI Insight
PD FEB 22
PY 2022
VL 7
IS 4
AR e152800
DI 10.1172/jci.insight.152800
PG 18
WC Medicine, Research & Experimental
WE Science Citation Index Expanded (SCI-EXPANDED)
SC Research & Experimental Medicine
GA ZG7JZ
UT WOS:000760432900001
PM 35015736
OA Green Published, gold
DA 2024-10-21
ER

PT J
AU Hayward, JA
   Tachedjian, M
   Johnson, A
   Irving, AT
   Gordon, TB
   Cui, J
   Nicolas, A
   Smith, I
   Boyd, V
   Marsh, GA
   Baker, ML
   Wang, LF
   Tachedjian, G
AF Hayward, Joshua A.
   Tachedjian, Mary
   Johnson, Adam
   Irving, Aaron T.
   Gordon, Tamsin B.
   Cui, Jie
   Nicolas, Alexis
   Smith, Ina
   Boyd, Victoria
   Marsh, Glenn A.
   Baker, Michelle L.
   Wang, Lin-Fa
   Tachedjian, Gilda
TI Unique Evolution of Antiviral Tetherin in Bats
SO JOURNAL OF VIROLOGY
LA English
DT Article
DE antiviral; bats; BST-2; evolution; filoviruses; innate immunity;
   restriction factors; retroviruses; tetherin; viruses
ID NF-KAPPA-B; FELINE IMMUNODEFICIENCY VIRUS; EXPERIMENTAL
INOCULATION;

ROUSETTUS-AEGYPTIACUS; RESTRICTION FACTOR; FRUIT BATS; RELEASE; PROTEIN;
GENOMES; EXPRESSION

AB Bats are an important host of various viruses which are deadly to humans and other mammals but do not cause outward signs of illness in bats. Furthering our understanding of the unique features of the immune system of bats will shed light on how they tolerate viral infections, potentially informing novel antiviral strategies in humans and other animals.

Bats are recognized as important reservoirs of viruses deadly to other mammals, including humans. These infections are typically nonpathogenic in bats, raising questions about host response differences that might exist between bats and other mammals. Tetherin is a restriction factor which inhibits the release of a diverse range of viruses from host cells, including retroviruses, coronaviruses, filoviruses, and paramyxoviruses, some of which are deadly to humans and transmitted by bats. Here, we characterize the tetherin genes from 27 bat species, revealing that they have evolved under strong selective pressure, and that fruit bats and vesper bats express unique structural variants of the tetherin protein. Tetherin was widely and variably expressed across fruit bat tissue types and upregulated in spleen tissue when stimulated with Toll-like receptor agonists. The expression of two computationally predicted splice isoforms of fruit bat tetherin was verified. We identified an additional third unique splice isoform which includes a C-terminal region that is not homologous to known mammalian tetherin variants but was functionally capable of restricting the release of filoviral virus-like particles. We also report that vesper bats possess and express at least five tetherin genes, including structural variants, more than any other mammal reported to date. These findings support the hypothesis of differential antiviral gene evolution in bats relative to other mammals. IMPORTANCE Bats are an important host of various viruses which are deadly to humans and other mammals but do not cause outward signs of illness in bats. Furthering our understanding of the unique features of the immune system of bats will shed light on how they tolerate viral infections, potentially informing novel antiviral strategies in humans and other animals. This study examines the antiviral protein tetherin, which prevents viral particles from escaping their host cell. Analysis of tetherin from 27 bat species reveals that it is under strong evolutionary pressure, and we show that multiple bat species have evolved to possess more tetherin genes than other mammals, some of which encode structurally unique tetherins capable of activity against different viral particles. These data suggest that bat tetherin plays a potentially broad and important role in the management of viral infections in bats.

C1 [Hayward, Joshua A.; Johnson, Adam; Gordon, Tamsin B.; Tachedjian, Gilda] Burnet Inst, Hlth Secur Program, Life Sci Discipline, Melbourne, Vic, Australia.

[Hayward, Joshua A.; Gordon, Tamsin B.; Tachedjian, Gilda] Monash Univ, Dept Microbiol, Clayton, Vic, Australia.

[Tachedjian, Mary; Nicolas, Alexis; Boyd, Victoria; Marsh, Glenn A.; Baker, Michelle L.] CSIRO, Hlth & Biosecur Business Unit, Australian Ctr Dis Preparedness, Geelong, Vic, Australia.

[Irving, Aaron T.] Zhejiang Univ, Zhejiang Univ Univ Edinburgh Inst, Sch Med, Int Campus, Haining, Zhejiang, Peoples R China.
[Irving, Aaron T.] Zhejiang Univ, Affiliated Hosp 2, Sch Med, Hangzhou, Zhejiang, Peoples R China.
[Irving, Aaron T.; Wang, Lin-Fa] Duke NUS Med Sch, Programme Emerging Infect Dis, Singapore, Singapore.
[Cui, Jie] Chinese Acad Sci, Inst Pasteur Shanghai, CAS Key Lab Mol Virol & Immunol, Beijing, Peoples R China.
[Smith, Ina] CSIRO, Hlth & Biosecur Business Unit, Acton, ACT, Australia.
[Wang, Lin-Fa] Singhlth Duke NUS Global Hlth Inst, Singapore, Singapore.
[Wang, Lin-Fa; Tachedjian, Gilda] Univ Melbourne, Peter Doherty Inst Infect & Immun, Dept Microbiol & Immunol, Melbourne, Vic, Australia.
C3 Burnet Institute; Monash University; Commonwealth Scientific & Industrial Research Organisation (CSIRO); Australian Centre for Disease Preparedness; Zhejiang University; Zhejiang University; National University of Singapore; Chinese Academy of Sciences; Commonwealth Scientific & Industrial Research Organisation (CSIRO); University of Melbourne; Peter Doherty Institute
RP Tachedjian, G (corresponding author), Burnet Inst, Hlth Secur Program, Life Sci Discipline, Melbourne, Vic, Australia.; Tachedjian, G (corresponding author), Monash Univ, Dept Microbiol, Clayton, Vic, Australia.; Tachedjian, G (corresponding author), Univ Melbourne, Peter Doherty Inst Infect & Immun, Dept Microbiol & Immunol, Melbourne, Vic, Australia.
EM gilda.tachedjian@burnet.edu.au
RI Tachedjian, Gilda/AAF-1327-2020; Marsh, Glenn/A-5418-2013; Wang, Xiaojun/JUU-9683-2023; Baker, Michelle/AAX-9171-2020; Cui, Jie/ABB-3831-2020; Irving, Aaron/AAE-8874-2021; Irving, Aaron/I-6754-2013; Tachedjian, Mary/H-4572-2013
OI Irving, Aaron/0000-0002-0196-1570; Hayward, Joshua/ 0000-0003-3904-3051; Tachedjian, Mary/0000-0002-8936-6564
FU National Health and Medical Research Council (NHMRC) [GNT1121077]; NHMRC Senior Research Fellowship [GNT1117748]; NHMRC [GNT1121077]; Monash University Vice Chancellor's Honours-PhD scholarship; Singapore National Research Foundation [NRF2012NRF-CRP001-056, NRF2016NRF-NSFC002-013]; National Natural Science Foundation of China [31671324]; CAS Pioneer Hundred Talents Program
FX We thank Paul Bieniasz for providing the pCRV1-NlgagpolDVpu and pTethHA463 plasmid constructs. We thank Richard Axel for providing HEK293T cells. This work was supported by the National Health and Medical Research Council (NHMRC) grant no. GNT1121077 awarded to

G.T.,
M.L.B., and G.A.M. and by NHMRC Senior Research Fellowship grant no.
GNT1117748 to G.T. J.A.H. was funded by NHMRC grant no. GNT1121077, a
Monash University Vice Chancellor's Honours-PhD scholarship, and through
the kind support of the Upotipotpon Foundation, the Estate of GWA
Griffiths, Miss Gwen Hotton, Mrs Rosemary Jacoby, and the Jim and
Margaret Beever Fellowship. L.-F.W. is funded by the Singapore National
Research Foundation grants NRF2012NRF-CRP001-056 and
NRF2016NRF-NSFC002-013. J.C. is funded by the National Natural Science
Foundation of China (31671324) and CAS Pioneer Hundred Talents Program.
We gratefully acknowledge the contribution of the Victorian Operational
Infrastructure Support Program, received by the Burnet Institute.
J.A.H., M.T., L.-F.W., and G.T. designed the study. J.A.H., J.C.,
A.T.I., A.N., I.S., T.B.G., and A.J. performed research and analyzed
data. J.A.H., M.T., A.J., A.N., V.B., G.A.M., M.L.B., L.-F.W., and G.T.
discussed and interpreted the results. J.A.H. wrote the first draft of
the paper. J.A.H. and G.T. critically edited the manuscript.
CR Amman BR, 2015, J WILDLIFE DIS, V51, P774, DOI 10.7589/2015-02-044
Andrew AJ, 2011, J BIOL CHEM, V286, P39967, DOI 10.1074/jbc.M111.287011
Andrew AJ, 2009, RETROVIROLOGY, V6, DOI 10.1186/1742-4690-6-80
Andrews S., 2010, FastQC: A Quality Control Tool for High Throughput Sequence Data
Arnaud F, 2010, J VIROL, V84, P4415, DOI 10.1128/JVI.00029-10
Baker ML, 2013, ZOONOSES PUBLIC HLTH, V60, P104, DOI 10.1111/j.1863-2378.2012.01528.x
Benton MJ, 2007, MOL BIOL EVOL, V24, P26, DOI 10.1093/molbev/msl150
Blanco-Melo D, 2016, CELL HOST MICROBE, V20, P189, DOI 10.1016/j.chom.2016.06.007
Brown T.A., 2002, GENOMES, V2nd
Calisher CH, 2006, CLIN MICROBIOL REV, V19, P531, DOI 10.1128/CMR.00017-06
Celestino M, 2012, J VIROL, V86, P6688, DOI 10.1128/JVI.07037-11
Cocka LJ, 2012, PLOS PATHOG, V8, DOI 10.1371/journal.ppat.1002931
Cowled C, 2011, DEV COMP IMMUNOL, V35, P7, DOI 10.1016/j.dci.2010.07.006
Dietrich I, 2011, J VIROL, V85, P5840, DOI 10.1128/JVI.00071-11
Doering T L, 2001, Curr Protoc Protein Sci, VChapter 12, DOI 10.1002/0471140864.ps1205s02
Dubé M, 2009, J VIROL, V83, P4574, DOI 10.1128/JVI.01800-08
Edgar RC, 2004, NUCLEIC ACIDS RES, V32, P1792, DOI 10.1093/nar/gkh340

Erikson E, 2011, P NATL ACAD SCI USA, V108, P13688, DOI 10.1073/pnas.1101684108
Fukuma A, 2011, PLOS ONE, V6, DOI 10.1371/journal.pone.0018247
Galao RP, 2012, CELL HOST MICROBE, V12, P633, DOI 10.1016/j.chom.2012.10.007
Gasteiger E., 2005, The Proteomics Protocols Handbook, P571, DOI [10.1385/1-59259-890-0:571, DOI 10.1385/1-59259-890-0:571]
Hawkins JA, 2019, P NATL ACAD SCI USA, V116, P11351, DOI 10.1073/pnas.1814995116
Hayward JA, 2020, P NATL ACAD SCI USA, V117, P9529, DOI 10.1073/pnas.1915400117
Hayward JA, 2018, MOL BIOL EVOL, V35, P1626, DOI 10.1093/molbev/msy048
Hinz A, 2010, CELL HOST MICROBE, V7, P314, DOI 10.1016/j.chom.2010.03.005
Hölzer M, 2019, ISCIENCE, V19, P647, DOI 10.1016/j.isci.2019.08.016
Hoffmann M, 2019, J VIROL, V93, DOI 10.1128/JVI.01821-18
Irving AT, 2020, METHODS ECOL EVOL, V11, P150, DOI 10.1111/2041-210X.13325
ISHIKAWA J, 1995, GENOMICS, V26, P527, DOI 10.1016/0888-7543(95)80171-H
Iwabu Y, 2009, J BIOL CHEM, V284, P35060, DOI 10.1074/jbc.M109.058305
Jebb D, 2020, NATURE, V583, P578, DOI 10.1038/s41586-020-2486-3
Jones MEB, 2015, VIRUSES-BASEL, V7, P3420, DOI 10.3390/v7072779
Jouvenet N, 2009, J VIROL, V83, P1837, DOI 10.1128/JVI.02211-08
Kacprzyk J, 2017, ACTA CHIROPTEROL, V19, P219, DOI [10.3161/15081109ACC2017.19.2.001, 10.3161/15081109acc2017.19.2.001]
Kent WJ, 2002, GENOME RES, V12, P656, DOI [10.1101/gr.229202. Article published online before March 2002, 10.1101/gr.229202]
Krogh A, 2001, J MOL BIOL, V305, P567, DOI 10.1006/jmbi.2000.4315
Kumar S, 2017, MOL BIOL EVOL, V34, P1812, DOI 10.1093/molbev/msx116
Kupzig S, 2003, TRAFFIC, V4, P694, DOI 10.1034/j.1600-0854.2003.00129.x
Letko M, 2020, NAT REV MICROBIOL, V18, P461, DOI 10.1038/s41579-020-0394-z
Li FY, 2015, BIOINFORMATICS, V31, P1411, DOI 10.1093/bioinformatics/btu852
Liberatore RA, 2011, P NATL ACAD SCI USA, V108, P18097, DOI 10.1073/pnas.1113694108
Lim ES, 2010, J VIROL, V84, P7124, DOI 10.1128/JVI.00468-10
Liu J, 2010, PLOS ONE, V5, DOI 10.1371/journal.pone.0011904
Livak KJ, 2001, METHODS, V25, P402, DOI 10.1006/meth.2001.1262
Mandl JN, 2018, FRONT IMMUNOL, V9, DOI 10.3389/fimmu.2018.02112
Matsuda A, 2003, ONCOGENE, V22, P3307, DOI 10.1038/sj.onc.1206406
McNatt MW, 2009, PLOS PATHOG, V5, DOI 10.1371/journal.ppat.1000300
Meri S, 1996, BIOCHEM J, V316, P923, DOI 10.1042/bj3160923
Middleton DJ, 2007, J COMP PATHOL, V136, P266, DOI 10.1016/j.jcpa.2007.03.002
Mok L, 2015, PROTEOME SCI, V13, DOI 10.1186/s12953-015-0081-6
Morrison JH, 2020, MBIO, V11, DOI 10.1128/mBio.01854-20

Murphy L, 2015, J VIROL, V89, P535, DOI 10.1128/JVI.02751-14
Nehls J, 2019, CELL REP, V26, P1841, DOI 10.1016/j.celrep.2019.01.065
Neil SJD, 2008, NATURE, V451, P425, DOI 10.1038/nature06553
O'Shea TJ, 2014, EMERG INFECT DIS, V20, P741, DOI 10.3201/eid2005.130539
Pavlovich SS, 2018, CELL, V173, P1098, DOI 10.1016/j.cell.2018.03.070
Perez-Caballero D, 2009, CELL, V139, P499, DOI 10.1016/j.cell.2009.08.039
Periasamy P, 2019, FRONT IMMUNOL, V10, DOI 10.3389/fimmu.2019.00489
Pierleoni A, 2008, BMC BIOINFORMATICS, V9, DOI 10.1186/1471-2105-9-392
Rollason R, 2007, J CELL SCI, V120, P3850, DOI 10.1242/jcs.003343
Ruedi M, 2013, MOL PHYLOGENET EVOL, V69, P437, DOI 10.1016/j.ympev.2013.08.011
Sakuma T, 2009, PLOS ONE, V4, DOI 10.1371/journal.pone.0006934
Sakuma T, 2009, J VIROL, V83, P2382, DOI 10.1128/JVI.01607-08
Sambrook J, 1989, MOL CLONING
Schubert HL, 2010, P NATL ACAD SCI USA, V107, P17951, DOI 10.1073/pnas.1008206107
Smith I, 2013, CURR OPIN VIROL, V3, P84, DOI 10.1016/j.coviro.2012.11.006
Swanepoel R, 1996, EMERG INFECT DIS, V2, P321, DOI 10.3201/eid0204.960407
Swiecki M, 2011, J BIOL CHEM, V286, P2987, DOI 10.1074/jbc.M110.190538
Takeda E, 2012, PLOS ONE, V7, DOI 10.1371/journal.pone.0041483
Teeling EC, 2018, ANNU REV ANIM BIOSCI, V6, P23, DOI 10.1146/annurev-animal-022516-022811
Tokarev A, 2013, J VIROL, V87, P2046, DOI 10.1128/JVI.02272-12
Venkatesh S, 2013, PLOS PATHOG, V9, DOI 10.1371/journal.ppat.1003483
Wang LF, 2019, CURR OPIN VIROL, V34, P79, DOI 10.1016/j.coviro.2018.12.007
Wang SM, 2019, J MED VIROL, V91, P1743, DOI 10.1002/jmv.25518
Watanabe S, 2010, EMERG INFECT DIS, V16, P1217, DOI 10.3201/eid1608.100208
Williamson MM, 1998, AUST VET J, V76, P813, DOI 10.1111/j.1751-0813.1998.tb12335.x
Wynne JW, 2013, PLOS PATHOG, V9, DOI 10.1371/journal.ppat.1003651
Yang HT, 2010, P NATL ACAD SCI USA, V107, P18428, DOI 10.1073/pnas.1011485107
Yang ZH, 2007, MOL BIOL EVOL, V24, P1586, DOI 10.1093/molbev/msm088
Zhang GJ, 2013, SCIENCE, V339, P456, DOI 10.1126/science.1230835
Zhou P, 2020, NATURE, V579, P270, DOI [10.1038/s41586-020-2012-7, 10.1038/s41586-020-2951-z]
Zhou P, 2016, P NATL ACAD SCI USA, V113, P2696, DOI 10.1073/pnas.1518240113
NR 82
TC 13
Z9 14

U1 3
U2 8
PU AMER SOC MICROBIOLOGY
PI WASHINGTON
PA 1752 N ST NW, WASHINGTON, DC 20036-2904 USA
SN 0022-538X
EI 1098-5514
J9 J VIROL
JI J. Virol.
PD OCT 26
PY 2022
VL 96
IS 20
DI 10.1128/jvi.01152-22
EA SEP 2022
PG 28
WC Virology
WE Science Citation Index Expanded (SCI-EXPANDED)
SC Virology
GA 5Q2AI
UT WOS:000863559400002
PM 36173189
OA Green Published, hybrid, Green Submitted
DA 2024-10-21
ER

PT J
AU Bennett, AJ
   Bushmaker, T
   Cameron, K
   Ondzie, A
   Niama, FR
   Parra, HJ
   Mombouli, JV
   Olson, SH
   Munster, VJ
   Goldberg, TL
AF Bennett, Andrew J.
   Bushmaker, Trenton
   Cameron, Kenneth
   Ondzie, Alain
   Niama, Fabien R.
   Parra, Henri-Joseph
   Mombouli, Jean-Vivien
   Olson, Sarah H.
   Munster, Vincent J.
   Goldberg, Tony L.
TI Diverse RNA viruses of arthropod origin in the blood of fruit bats
   suggest a link between bat and arthropod viromes
SO VIROLOGY
LA English
DT Article
DE Bats; Arthropods; Virus; Ecology; Evolution; Dicistrovirus;

Nodavirus;
   Tombusvirus; Hepadnavirus; Next-generation sequencing
ID NODAMURA-VIRUS; VIRAL-RNA; DELIBERATE INSECTIVORY;
HEPADNAVIRUSES;
   DINUCLEOTIDE; TRANSMISSION; INFECTION; ALIGNMENT; PROTEIN;
METAGENOMICS
AB Bats host diverse viruses due to their unique ecology, behavior, and immunology. However, the role of other organisms with which bats interact in nature is understudied as a contributor to bat viral diversity. We discovered five viruses in the blood of fruit bats (Hypsigrtathus monstrosus) from the Republic of Congo. Of these five viruses, four have phylogenetic and genomic features suggesting an arthropod origin (a dicistrovirus, a nodavirus, and two tombus-like viruses), while the fifth (a hepadnavirus) is clearly of mammalian origin. We also report the parallel discovery of related tombus-like viruses in fig wasps and primitive crane flies from bat habitats, as well as high infection rates of bats with haemosporidian parasites (Hepatocystis sp.). These findings suggest transmission between arthropods and bats, perhaps through ingestion or hyperparasitism (viral infection of bat parasites). Some "bat-associated" viruses may be epidemiologically linked to bats through their ecological associations with invertebrates.
C1 [Bennett, Andrew J.; Goldberg, Tony L.] Univ Wisconsin, Sch Vet Med, Dept Pathobiol Sci, Madison, WI 53706 USA.
   [Bushmaker, Trenton; Munster, Vincent J.] NIAID, Lab Virol, Virus Ecol Unit, Div Intramural Res,Rocky Mt Labs,NIH, Hamilton, MT USA.
   [Cameron, Kenneth; Ondzie, Alain; Olson, Sarah H.] Wildlife Conservat Soc, Wildlife Hlth Program, 2300 Southern Blvd, Bronx, NY USA.
   [Niama, Fabien R.; Parra, Henri-Joseph; Mombouli, Jean-Vivien] Lab Natl Sante Publ, Brazzaville, Rep Congo.
[truncated: 405,963 more chars]
